# Comparative analysis of miRNA expression profiles in transgenic and non-transgenic rice using miRNA-Seq

**DOI:** 10.1038/s41598-017-18723-x

**Published:** 2018-01-10

**Authors:** Cheng Peng, Xiaoyun Chen, Xiaofu Wang, Xiaoli Xu, Wei Wei, Congmao Wang, Junfeng Xu

**Affiliations:** 1 Institute of Quality and Standard for Agro-products, Zhejiang Academy of Agricultural Sciences, Hangzhou, 310021 China; 2 State Key Laboratory Breeding Base for Zhejiang Sustainable Pest and Disease Control, Hangzhou, 310021 China; 3 QIAGEN (Suzhou) Translational Medicine Co., Ltd, Suzhou, 215123 China

## Abstract

Safety assessment for genetically modified organisms (GMOs) is required before their release. To date, miRNAs that play important roles in eukaryotic gene regulation have not been considered in the current assessment system. In this study, we identified 6 independent Bt and EPSPS GM rice lines using PCR and immune strip. We analyzed the expression levels of Cry1Ac and EPSPS using quantitative real-time PCR and western blot. Further, miRNAs from the developing seeds of the 6 GM rice lines and the wild-type line were investigated using deep sequencing and bioinformatic approaches. Although these GM lines have different types of integration sites, copy numbers, and levels of gene expression, 21 differentially expressed miRNAs have been found compared to wild type. There is no correlation between transgenic protein expression level and the quantity of differentially expressed miRNAs. This study provides useful data about the miRNA composition of GM plants, and it might be helpful for future risk assessments of miRNA-based GM plants.

## Introduction

Genetic engineering of agricultural crops has played an important role in crop improvement. It has been used to increase resistance to diseases and other stresses and tolerance to herbicides as well as to improve the nutritive value of crops. Given the complexity of plant cells and the current limitations on genetic engineering, unintended effects may result from genetic engineering in addition to the transformation of the novel trait. Prior to the release of transgenic crops into the environment, they need to be examined for health and environmental safety. However, unintended, unexpected effects might occur, which are difficult to test.

Recently, advancements in molecular analysis techniques such as high-throughput sequencing and global profiling technologies have given us an unprecedented understanding of the unintended effects and impact of genetic changes in plants. Omics profiling technologies such as transcriptomics^1–4^, proteomics^5,6^ and metabolomics^7–9^ have been suggested to broaden the spectrum of detectable compounds and to supplement the current targeted analytical approaches. However, some molecular regulators have not been included in previous assessments, such as microRNA (miRNA).

miRNAs are endogenous, non-coding small RNAs that are usually 20–24 nt in length in plants^10^. miRNAs can regulate gene expression at the post-transcriptional level by degrading target mRNAs or inhibiting translation through complementary matching between miRNAs and specific sites in target genes. A large fraction of protein-coding genes can be miRNA targets, while a single miRNA can target hundreds to thousands mRNAs as well^11^. Recently, Zhang *et al*. found that miRNA168a is abundant in rice. It is also one of the most highly enriched exogenous plant miRNAs in the sera of Chinese that could bind to the mammalian/animal low-density lipoprotein receptor adapter protein 1 (LDLRAP1) mRNA, inhibit LDLRAP1 expression in liver, and decrease LDL removal from mouse plasma^12^. Zhou *et al*. found that miRNA2911 from honeysuckle (HS) could directly target influenza A viruses (IAVs) and suppress H1N1 viral replication in mice^13^. Jing Li and colleagues further revealed that exogenous/endogenous small non-coding RNAs in the maternal system can transfer through the placenta to the fetal side and influence fetal development and health^14^. Further studies have revealed that a variety of exogenous/endogenous plant RNAs can be found in the plasma and serum of mammals after ingestion^15–20^. These finding suggest that exogenous/endogenous miRNAs may influence mammalian/animal health. However, no one has addressed whether the miRNA expression profile changes in transgenic rice. In particular, some miRNAs such as miRNA2911 and miRNA168a, which have the potential to affect human health^12,13^, have not been studied.

In this study, we aimed to find the miRNA related with the insertion of the two transgenes *cry1Ac* (gene for insecticidal Bt protein) and *EPSPS* (herbicide-tolerance gene). We used 6 independent Bt and EPSPS GM rice lines as a transgenic group and compared it to the wild type. Using high-throughput sequencing and RT-PCR verification, we identified the common differentially expressed miRNAs in the 6 Bt and EPSPS transgenic rice lines. Our study provides useful information about the miRNA composition of GMOs. This will be helpful for the safety assessment of GMOs based on miRNAs.

## Results

### Detection of transgenic Bt and EPSPS rice lines

Cry1Ac and EPSPS are the most widely used exogenous proteins in transgenic crops around the world. To study and assess the microRNA-based differences between GMOs and non-GMOs, Cry1Ac and EPSPS, combined on one vector, have been transferred to rice through *Agrobacterium*-mediated transformation. A summary of the binary vector used to create these transgenic lines is shown in Supplementary Fig. S1a. From limited analyses of several independent primary transformation lines, six lines (L1 to L6), were randomly selected for a complete characterization of the homozygous T_5_ progeny. Molecular analyses were performed to verify both the presence of each transgene in the six lines and the expression of the corresponding recombinant protein. Two primers were designed based on the sequences of *cry1Ac* and *EPSPS* to detect the exogenous gene in these lines, and rice *sucrose phosphate synthase (SPS)* was used as an internal control gene. The transgenic plasmid and wild-type rice were used as positive and negative controls, respectively (Supplementary Fig. S1b). Commercial test strips have also been used to detect the GMOs, and we further used Cry1Ab/Ac and EPSPS quick strips to detect the expression of Cry1Ac and EPSPS (Supplementary Fig. S1c).

Furthermore, the copy number and insert location of the exogenous genes have been identified using quantitative real-time PCR^21^ and TAIL-PCR^21,22^. All integration events are summarized in Supplementary Table 1, which shows the different types of integration events in the six lines.

### Different expression levels of the transgenes in transgenic rice L1 to L6

The mRNA transcripts of the transgenes in these lines were examined using quantitative real-time PCR analysis. Supplementary Fig. S2a shows that the transcript level of *cry1Ac* in L1 was higher than that in the other lines. Consistent with the transcript level of *cry1Ac*, immunoblot analysis confirmed the higher expression of Cry1Ac protein in L1 using anti-Cry1Ac antibodies (Supplementary Fig. S2c). The anti-α-tubulin antibodies were used as a loading control. The transcript level of *EPSPS* was higher in L5 (Supplementary Fig. S2b), and the amount of EPSPS protein in L5 was also more abundant (Supplementary Fig. S2c). These results show that the transgenes have different expression levels in lines L1 through L6.

### Summary of small RNA sequencing data

**Figure 1:**
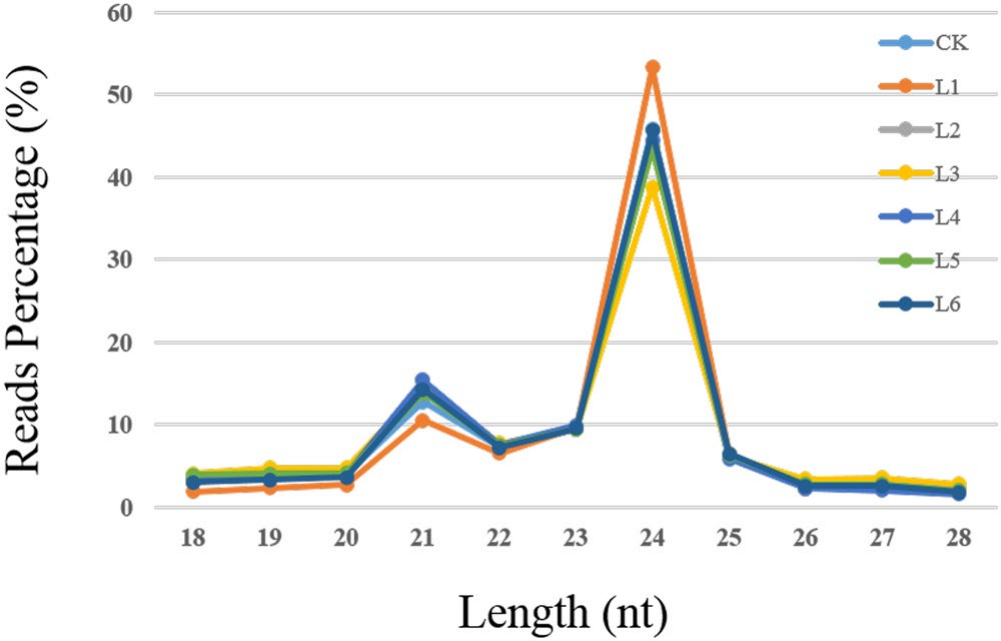
Small RNA reads percentage of different length distribution in CK and L1 to L6.

Total RNA was extracted from each group, and seven libraries were constructed from CK and L1 to L6 (CK, 13,580,216 reads; L1, 15,420,462 reads; L2, 10,373,946 reads; L3, 11,339,502 reads; L4, 12,268,197 reads; L5, 10,809,352 reads; L6, 10,263,170 reads). Effective reads (more than 95% of total reads) were obtained by removing reads containing poly-N, 5′ adapter contaminants; reads without 3′ adapters or the insert tags; reads containing poly A, T, G or C; low-quality reads from raw data; and reads shorter than 18 nucleotides (Supplementary Table 4). The size distribution of the clean reads is presented in Fig. 1. The results showed that most of the reads were 24 nt or 21 nt in size. This result is consistent with previous studies in rice seeds^23,24^. These high-quality reads were mapped to the rice genome in miRBase (release 21.0) to identify conserved miRNAs for further analysis. In total, there are 568 conserved miRNAs in the rice seeds based on the deep sequencing.

MIR2911, a miRNA in honeysuckle, targeting IAVs and suppressing H1N1 viral replication in mice^13^, was not found when we mapped our seven sequence libraries to the miRBase database.

### Differential expression analysis of conserved miRNAs between non-transgenic CK and transgenic lines L1 to L6

**Figure 2:**
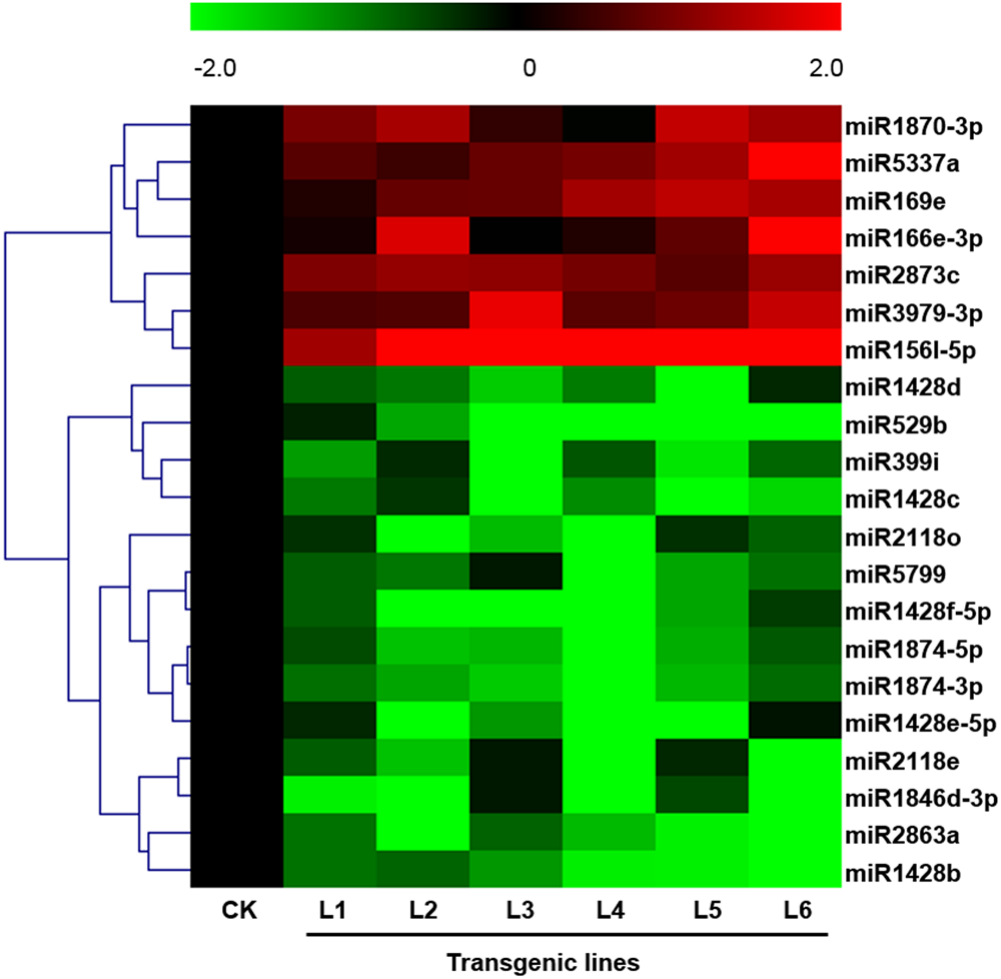
Hierarchical clustering analysis for the identified differentially expressed miRNAs. The horizontal axis represents the transgenic lines L1 to L6 and the non-transgenic control (CK). The miRNA names are shown on the right vertical axis. Red and green represent the up-regulated and down-regulated miRNAs, respectively. For each differentially expressed miRNA, relative expression was calculated by setting the CK as 0 (black), and the GM lines in color (red or green) compared to CK. The color value = log_2_ (TPM in each GM line/TPM in CK).

To identify differentially expressed miRNAs, we compared the expression of conserved miRNAs between non-transgenic CK and the transgenic group lines L1 to L6 (Supplementary Fig. S3). Based on the high-throughput sequencing results, we used a hierarchical clustering algorithm to analyze differentially expressed miRNAs in every sample (Fig. 2). We discovered a set of differentially expressed miRNAs from each line compared to the non-transgenic CK, and then we compared the tags per million (TPM) values of every sample from all the sets of differential expression miRNAs to make a hierarchical cluster. The results indicated that 7 miRNAs, i.e. miRNA5337a, miR3979-3p, miR2873c, miR1870-3p, miR169e, miR166e-3p, and miR156l-5p, were significantly up-regulated compared with the non-transgenic CK. Conversely, 14 miRNAs, i.e.miR5799, miR529b, miR399i, miR2863a, miR2118o, miR2118e, miR1874-5p, miR1874-3p, miR1846d-3p, miR1428f-5p, miR1428e-5p, miR1428d, miR1428c and miR1428b, were significantly down-regulated compared with the non-transgenic CK. The most abundant up-regulated miRNAs were miR156l-5p, miR166e-3p and miR5337a, and the most abundant down-regulated miRNAs were miR529b, miR1428f-5p and miR1428c (Table 1). In addition, there are 547 miRNAs (96% of the total) that showed no difference between non-transgenic CK and the transgenic group.

**Table 1:** Differentially expressed miRNAs between non-transgenic CK and the transgenic group.

Gene_id	Transgenic group (lines L1- L6)	Non-transgenic (CK)	Fold_change-Transgenic/CK	Corrected P-value	FDR	UP/DOWN regulate
miR156l-5p	119.39	11.00	10.85	8.83E-25	1.88E-23	UP
miR166e-3p	1131.35	313.46	3.61	6.62E-111	3.06E-109	UP
miR5337a	37.83	16.50	2.29	4.36E-03	2.37E-02	UP
miR3979-3p	48.07	22.00	2.19	1.23E-03	7.89E-03	UP
miR169e	34.36	16.50	2.08	1.22E-02	5.64E-02	UP
miR2873c	67.80	33.00	2.05	4.43E-04	3.32E-03	UP
miR1870-3p	55.07	27.50	2.00	2.09E-03	1.27E-02	UP
miR2118e	16.25	33.00	0.49	3.30E-02	1.23E-01	DOWN
miR5799	16.21	33.00	0.49	3.30E-02	1.23E-01	DOWN
miR399i	45.78	93.49	0.49	6.36E-05	5.78E-04	DOWN
miR1428d	21.45	43.99	0.49	9.29E-03	4.52E-02	DOWN
miR2118o	28.28	60.49	0.47	9.91E-04	6.61E-03	DOWN
miR1428e-5p	14.60	33.00	0.44	1.34E-02	6.02E-02	DOWN
miR1874-5p	2851.69	6615.63	0.43	0.00E + 00	0.00E + 00	DOWN
miR1874-3p	6306.25	15485.97	0.41	0.00E + 00	0.00E + 00	DOWN
miR1846d-3p	19.41	49.49	0.39	4.47E-04	3.30E-03	DOWN
miR2863a	18.36	49.49	0.37	2.49E-04	2.00E-03	DOWN
miR1428b	18.29	49.49	0.37	2.49E-04	2.00E-03	DOWN
miR1428c	14.19	38.49	0.37	1.51E-03	9.52E-03	DOWN
miR1428f-5p	11.66	33.00	0.35	2.48E-03	1.49E-02	DOWN
miR529b	22.40	71.49	0.31	4.96E-07	6.54E-06	DOWN

**Figure 3:**
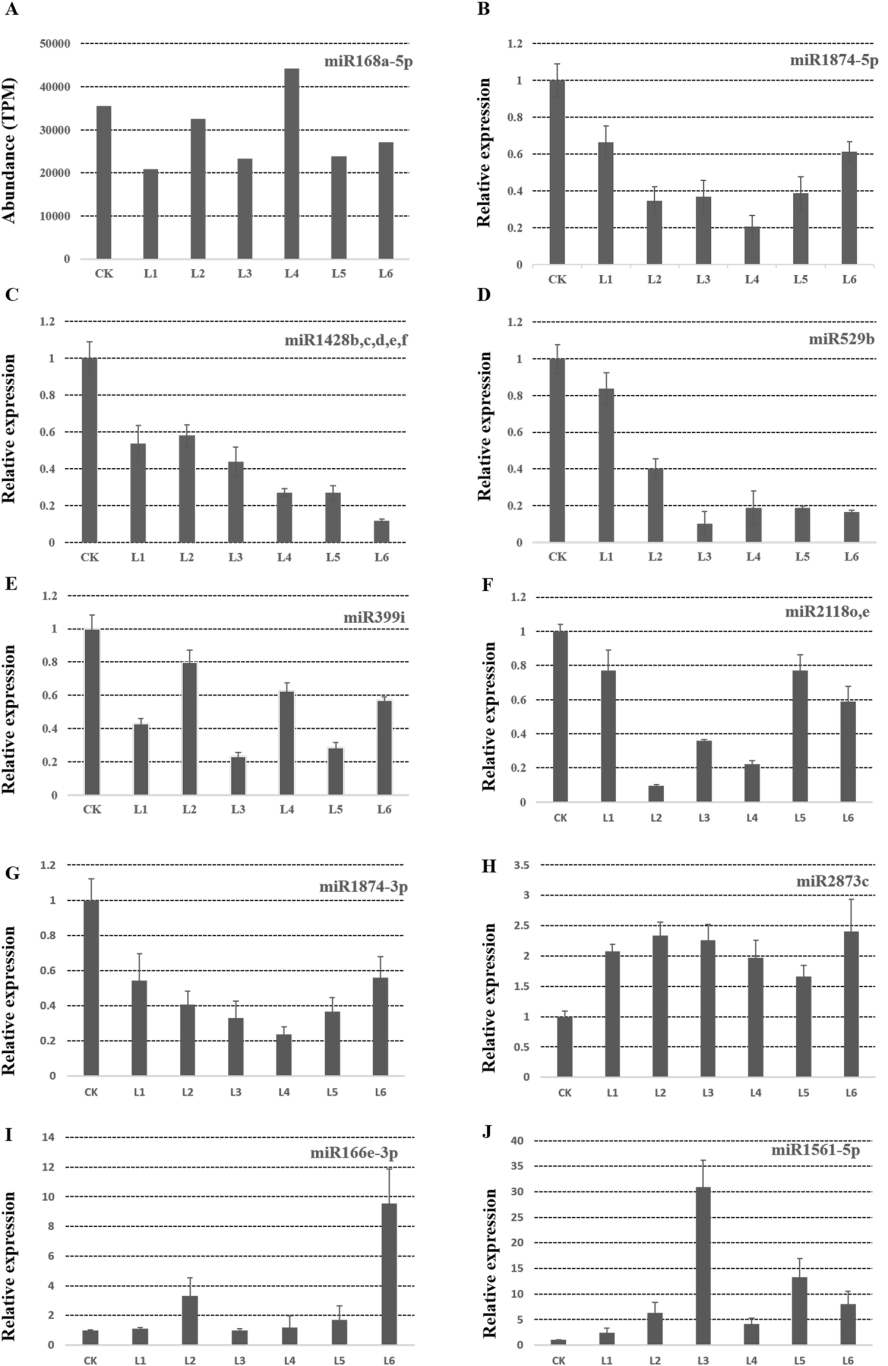
Expression of several miRNAs. (**A**) Normalized sequence reads of miR168a-5p. (**B**–**J**) Validation of the differentially expressed miRNAs by Quantitative real-time PCR. The 2^−ΔΔCt^ method was used for the experiments and the U6 snRNA was selected as the endogenous reference gene for normalization.

Of the down-regulated miRNA groups, we noticed that miR1428b, miR1428c, miR1428d, miR1428e-5p and miR1428f-5p were all from the miR1428 family. This indicated that the miR1428 family may respond to Cry1Ac and EPSPS insertion or expression independent from the site of insertion. The results also indicated that the expression level of miR168a was not significantly different between non-transgenic CK and the transgenic group (Fig. 3A). In addition, we compared the numbers of differentially expressed miRNAs between each line and wild type (Supplementary Fig. S4). The L4 line, which showed moderate protein expression, had the most divergent composition of microRNAs. The L1 and L5 lines, which had the highest expression level of Cry1Ac and EPSPS, showed moderate differences compared to CK (Fig. 2, Supplementary Fig. S4), indicating that there was no correlation between the transgenic protein expression level and the quantity of differentially expressed miRNAs. The differentially expressed miRNAs were selected for validation by RT-PCR. The results confirmed these miRNAs in transgenic lines (Fig. 3B–D) in a manner consistent with the alteration shown using miRNA sequencing. The differentially expressed miRNAs which were difficult to detect by RT-PCR because of low abundance were not listed here.

### miRNA target prediction in rice

**Figure 4:**
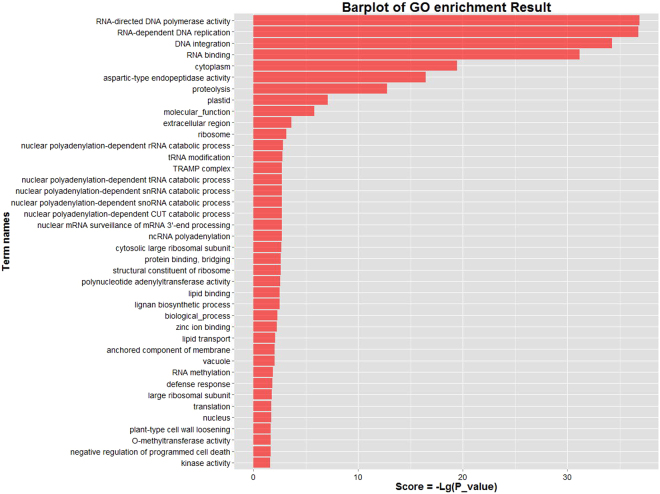
GO enrichment analysis of the target genes of differentially expressed miRNAs. Forty significantly enriched GO pathways were achieved using the target genes of differentially expressed miRNAs.

To obtain further insight into the biological functions of differentially expressed miRNAs between transgenic lines and non-transgenic CK, miRanda (http://www.microrna.org/) was used to predict target mRNAs. All target genes for the differentially expressed miRNAs were predicted, and the target ID data are listed in Supplementary Table 5. Gene Ontology (GO), the *de facto* standard for gene functionality descriptions, is widely used in functional annotation and enrichment analysis. The large-scale predicted target genes were subjected to GO enrichment analysis, and the top 40 enriched GO terms based on the false discovery rate (FDR) for gene targets are presented (Fig. 4, Supplementary Table 6). Among these, the first two enriched GO terms, “RNA-directed DNA polymerase activity” and “RNA-dependent DNA replication”, are related to the molecular function of RNA. The third most enriched GO term, “DNA integration,” is a biological process in which a segment of DNA is incorporated into another, usually larger, DNA molecule similar to a chromosome^25^. It is believed that the pathway of DNA integration in transgenic lines may be changed through the process of *Agrobacterium*-mediated transformation.

## Discussion

miRNAs are endogenous, non-coding small RNAs that act on many different molecular and biochemical processes in eukaryotes. Many studies have shown that miRNAs can transmit signals across species and that they can affect different species^15–20^. Until now, miRNAs have not been considered in the safety assessment of GMOs. The aim of this study was to identify the differentially expressed miRNAs between GM lines and the non-transgenic parental line.

Cry1Ac and EPSPS are the most widely used transgenic proteins in GM crops^26^. They are used to improve resistance to certain lepidopteran pests and tolerance to herbicide. Rice is a model plant with relatively clear genomic functional annotation^27,28^, and it is the most important cereal crop in the world. The rice seed is the edible part, which makes it practical for future research on cross-kingdom regulation of plant miRNAs. It is worth noting that the number and type of microRNAs in developing seeds may be more abundant than in mature seeds, and some key miRNAs in the developing seed influence seed development, starch biosynthesis, nutritional factors or systemic effects. In a previous study, we found that the rice grain grows in weight quickly during the first 12–18 days after flowering (DAF)^23,29^. This finding indicates that this is a critical period of starch accumulation, and some small-molecule regulators may be involved. To make each sample consistent, we chose developing seeds 15DAF from Bt and EPSPS transgenic rice for miRNA-sequencing in this study. Using these samples, we constructed and sequenced seven high-quality microRNA libraries, and all had more than 10,084,897 effective reads (Supplementary Table 4). Most of the reads were 24 nt and 21 nt in size, which is consistent with previous studies^23,24^ and suggests that the miRNA sequencing data are suitable for our analysis.

Many studies have been reported about the safety assessment of Bt and EPSPS GM crops based on transcriptomics, proteomics and metabolomics. Eugenia *et al*. compared the difference between a GM-Bt maize line and control line using three profiles^30^. Coll *et al*. used microarrays to compare the transcriptome profiles of the widely used commercial GM-Bt event MON810 compared to near-isogenic varieties, and they reported differential expression of a small set of sequences in the leaves of AristisBt/Aristis and PR33P67/PR33P66 for *in vitro* cultured plants^31^. Recently, Wang *et al*. found several differentially expressed miRNAs between GM-EPSPS event MON89788 and non-GM soybean A3244 using deep sequencing technology and bioinformatics approaches^32^. Jiang *et al*. focused their research on differentially expressed miRNAs in GM wheat seeds, and 23 differentially expressed miRNAs in wheat seeds were identified and confirmed between GM wheat and a non-GM acceptor^33^. These data on the profiling of GM plant lines reveal that some differences exist compared to control lines. This could be because GM lines have been selected by a process based on phenotypic and compositional equivalence with a close comparator followed by a number of crosses to introgress the new trait into elite lines instead of the suitable expression of a new trait^31^. Several environmental factors and insertional effects of transgenic genes have also been shown to exert a greater influence than transgenic gene expression^34,35^. To determine the real miRNA markers after transgenic Cry1Ac and EPSPS expression in plants and to exclude other effects as much as possible, we used 6 independent Bt and EPSPS GM rice lines as a transgenic group compared to wild type. The transgenic lines L1 to L6 had similar genetic background compared to wild type, and they differed in the type of integration site, copy number (Table 1), and gene expression level (Supplementary Fig. S2). Contrary to our expectation that the transgenic line with the highest transgene or protein expression level would show the most drastic changes compared to non-GM wild type, we found that the L4 line with moderate protein expression had the most divergent composition of miRNAs. The L1 and L5 lines, which had the highest expression levels of Cry1Ac and EPSPS, showed moderate changes (Supplementary Figs S2 and S4). There was no correlation between transgenic protein expression levels and the quantity of differentially expressed miRNAs detected, indicating that miRNAs might be differentially expressed in different strains of GM rice.

In this study, we identified the integration sites, copy number (Table 1), and gene expression levels (Supplementary Fig. S2) of the transgenic lines L1 to L6. Deep sequencing was used for comparative profiling of miRNA expression in transgenic lines L1 to L6 and their wild-type acceptor line. These GM lines have different types of integration sites, copy numbers (Table 1), and gene expression levels (Supplementary Fig. S2), and the results show that 21 common differentially expressed miRNAs have been found compared with wild type. Among these miRNAs, some have been shown to play roles in plant stress resistance and seed development. The miR166 target ATHB14-LIKE transcript was experimentally validated by RACE PCR^36^. The expression patterns of the miR166s and 12 target genes were examined during seed development and in response to abiotic stresses. miR169 targets the NF-YA family members, which play important roles in plant stress-induced response. It was reported that miR169 negatively regulates rice immunity against the fungal pathogen of rice blast (*Magnaporthe oryzae*) by differentially repressing its target genes^37^. In addition, GO enrichment analysis of all the target genes of differentially expressed miRNAs in the GM and non-GM rice lines showed that many targets are associated with “DNA integration” (Fig. 4, Supplementary Table 6), suggesting that the corresponding miRNAs may be involved in the process induced by T-DNA insertion. On the other hand, the differentially expressed miRNAs do not necessarily correlate with risk. The percentage of the differentially expressed miRNAs in transgenic plants compared to the total number of miRNAs was 4%. There are some putative plant miRNAs, including miRNA2911 and miRNA168a, that have been detected in the serum and plasma of human and animals^12,13^. We aligned these miRNAs, and all of the differentially expressed miRNAs in our study have not been mapped back to these targets. To our knowledge, there is no report showing that the differentially expressed miRNAs from our study can be detected in human or other animals *in vivo*. In addition, although we tried to exclude environmental factors as much as possible, considering some other factors, such as framework plasmid and natural variation, it is difficult to say that the 21 differentially expressed miRNAs are related with the insertion of transgenes. These differentially expressed miRNAs may be specific to the particular transgenic plants examined here. They might also be a result of *Agrobacterium*-mediated transformation and unrelated to the expression of Cry1Ac and EPSPS. However, our results show that the improvement of a plant variety through the acquisition of a new desired trait (by genetic engineering) might cause stress and thus may have an impact on miRNA expression. Finally, we believe that safety assessments of GM plants might benefit from miRNA-sequencing. This, however, should be considered on a case-by-case basis rather than suggesting it as a routine method.

## Materials and Methods

### Plant materials

Six independent transgenic lines L1 to L6 were developed using *Agrobacterium*-mediated transformation. The binary vector used to create these transgenic lines is shown in Supplementary Fig. S1a, and it was provided by Professor Shen Zhicheng, Zhejiang University. The six transgenic lines (homozygous T_5_ progeny) and the wild type (*Oryza sativa* spp. *japonica* cv. Xiushui11) were grown and placed randomly side by side under natural conditions without any treatments in an experimental site of the Zhejiang Academy of Agricultural Sciences. The developing rice seeds (15DAF) were gained after self-pollination. Each sample was pooled from the seeds collected from 3 different plants for each line. The resulting seven samples were ground into fine powder with liquid nitrogen and stored at −80 °C. 50 mg powder from the same sample was taken for DNA, mRNA, microRNA and protein extraction, respectively.

### DNA extraction, PCR analysis and immune strip test

DNA was prepared using a DNA Extraction Kit for GMO Detection, ver. 3.0 (Takara, Shiga, Japan). The DNA was quantified using PicoGreen reagent according to the manufacturer instructions (Qubit dsDNA BR Assay Kit, Invitrogen, Shanghai, China). Purity of the extracted DNA was determined using the ratio of the absorbance at 260 and 280 nm using a spectrophotometer (Ultrospec 1100 pro, GE Healthcare, USA), and its integrity was characterized using agarose gel electrophoresis. PCR amplification was performed in a 30-μL reaction volume with 10X PCR buffer, 200 μM dNTP, 0.4 μM of each primer, 1.25 U Taq DNA polymerase (TaKaRa Biotechnology Co.), and 50–100 ng DNA template. The amplification program for each primer set is listed in Supplementary Table 2. The samples were also detected by Cry1Ab/Ac and EPSPS quick strips (YouLong Biotech, China) according to the manufacturer instructions.

### Real-time PCR and western-blot analysis

Total RNA was extracted using an RNAprep pure Plant kit (TIANGEN Biotech, Beijing, China). mRNA was reverse transcribed to first-strand cDNA using the PrimeScript Reverse Transcriptase kit (Takara, Otsu, Japan). The amount of *actin I* mRNA (accession number AK100267) was used as an internal control for the expression of *cry1Ac* and *EPSPS*. miRNA was reverse transcribed to cDNA using a miRcute miRNA first-strand cDNA synthesis kit (TIANGEN Biotech, Beijing, China). The U6 snRNA was selected as the endogenous reference gene for normalization. All reactions were performed using one biological sample with three technical replicates, and the relative expression levels were calculated using 2^−ΔΔCt^ method as described. The primers used in this analysis are listed in Supplementary Table 3.

Total protein extraction was performed as described by Peng *et al*.^29^, and proteins were analyzed using SDS-PAGE. The immunoblot assay followed Wang *et al*.^38^. The anti-Cry1Ac toxin monoclonal antibody (ab113679) was purchased from Abcam Company. Synthetic peptide fragments of EPSPS were chemically synthesized and used as antigens in rabbits to elicit antiserum. The antiserum of EPSPS was provided by Professor Shen Zhicheng, Zhejiang University.

### MiRNA isolation, library construction and sequencing

Total RNA was extracted from rice seeds using TRIzol reagent (Invitrogen, Carlsbad, USA) and purified using the mirVanaTM miRNA Isolation Kit (Ambion, Austin, TX, USA) according to manufacturer’s instructions. The quality of the total RNA was detected using a Qubit® 2.0 Fluorometer (Invitrogen, USA). Total RNA integrity was checked using an Agilent 2100 Bioanalyzer (Agilent Technologies, Santa Clara, USA) with an RNA Integrity Number (RIN) value greater than 8. Library preparation and Illumina sequencing was performed by Shanghai Biotechnology Corporation according to the Illumina small RNA sample preparation protocol^39^. The purified small RNA molecules were ligated to a 5′-adaptor and a 3′-adaptor using T4 RNA ligase. Next, the adapter-ligated small RNAs were reverse transcribed into cDNA using a SuperScript II Reverse Transcription Kit (Invitrogen, USA), and the cDNA samples were amplified using PCR. PCR products were gel purified, and their quality and concentrations were confirmed using an Agilent 2100 Bioanalyzer (Agilent Technologies, Santa Clara, USA). Finally, the purified cDNA libraries were quantified using a Qubit Fluorometer (Invitrogen, USA), and used for cluster generation and 36 nt single-end sequencing analysis using the Illumina Genome Analyzer IIx. The raw data from the small RNA libraries were deposited in the NCBI Sequence Read Archive (SRA) under the accession no. SRP108191.

### Bioinformatics identification of conserved miRNAs

After sequencing, the low-quality and contaminant reads were removed from the raw reads using the following steps: (1) eliminating low quality reads; (2) eliminating reads without a 3′-primer; (3) eliminating reads with 5′-primer contaminants; (4) eliminating reads without the insert tag; (5) eliminating reads with poly A; and (6) eliminating reads shorter than 18 nt. The final clean reads of the small RNA libraries were obtained and mapped to the rice genome using SOAP^40^. Conserved miRNAs were identified using a BLASTn search against the miRBase database (Release 21.0, http://www.mirbase.org/) with up to two mismatches to identify “conserved” mature miRNA orthologs^41^.

### Analysis of differentially expressed miRNAs

Gene expression quantification was performed using the normalized number of fragment tags per million (TPM) values (TPM = (readCount * 1,000,000)/libsize)^11^. The TPM value of transgenic group is the average of L1 to L6. The ratio value of each miRNA was calculated by comparing its normalized expression in non-transgenic (CK) to that of the transgenic group. The P-value significance threshold in multiple tests was set by the false discovery rate (FDR). The fold changes (log_2_ ratio) were also estimated according to the normalized miRNA expression level for each sample. The differentially expressed miRNAs (DEMs) between the two pooled samples were selected using the following filter criteria: TPM >30, FDR <0.05 and log_2_ (fold change ratio) >2 or <0.5. The normalized read count of some miRNAs was set to be 0.01 for further calculation if there were no reads in the library.

### Target gene predictions and GO Analysis

Target genes were predicted using miRanda (http://www.microrna.org)^42^. To further understand the function and classification of the predicted miRNA target, Gene Ontology (GO) classification of the target genes was conducted with WEGO web service (http://www.geneontology.org/), GO terms assigned to the query sequences and catalogued groups were produced based on their biological process, molecular functions, and cellular components^43^.

## Electronic supplementary material

Supplementary information
