# Supplementary material for: Comparative analysis of miRNA expression profiles in transgenic and non-transgenic rice using miRNA-Seq
Source: Sci Rep. 2018 Jan 10;8:338. doi: 10.1038/s41598-017-18723-x (PMC5762784; doi:10.1038/s41598-017-18723-x)
Supplement: Supplementary file 1 — Supplementary information [file 41598_2017_18723_MOESM1_ESM.pdf]

Comparative analysis of miRNA expression profiles in transgenic and non-transgenic rice using miRNA-Seq

Cheng Peng[1,2]; Xiaoyun Chen[1,2]; Xiaofu Wang[1,2]; Xiaoli Xu[1,2]; Wei Wei[1,2]; Congmao Wang[3]; Junfeng Xu[1,2,*]

[1]Institute of Quality and Standard for Agro-products, Zhejiang Academy of Agricultural Sciences; Hangzhou 310021, China

[2]State Key Laboratory Breeding Base for Zhejiang Sustainable Pest and Disease Control；Hangzhou 310021, China

[3]QIAGEN (Suzhou) Translational Medicine Co., Ltd, Suzhou 215123, China

* For correspondence. Telephone: +86-0571-86415202; Fax: +86-0571-86401834; E-mail:njjfxu@163.com

Supplementary Figure S1 Verification of the presence of each respective transgene in transgenic rice L1 to L6

(A) The binary vectors used for Agrobacterium-mediated transformation are shown. The red triangle indicates the location of the primer. (B) Three percent agarose gel electrophoresis of PCR products amplified with the *Cry1Ac*, *G10evo*-EPSPS event-specific primers and rice sucrose phosphate synthase (*SPS*) gene primer. M, the 1,000bp DNA ladder; +, Transgenic plasmid as positive control; CK, non-transgenic rice DNA as negative control; L1 to L6, six transgenic rice lines. (C) Detection of Bt and EPSPS transgenic rice plants using immune strip.

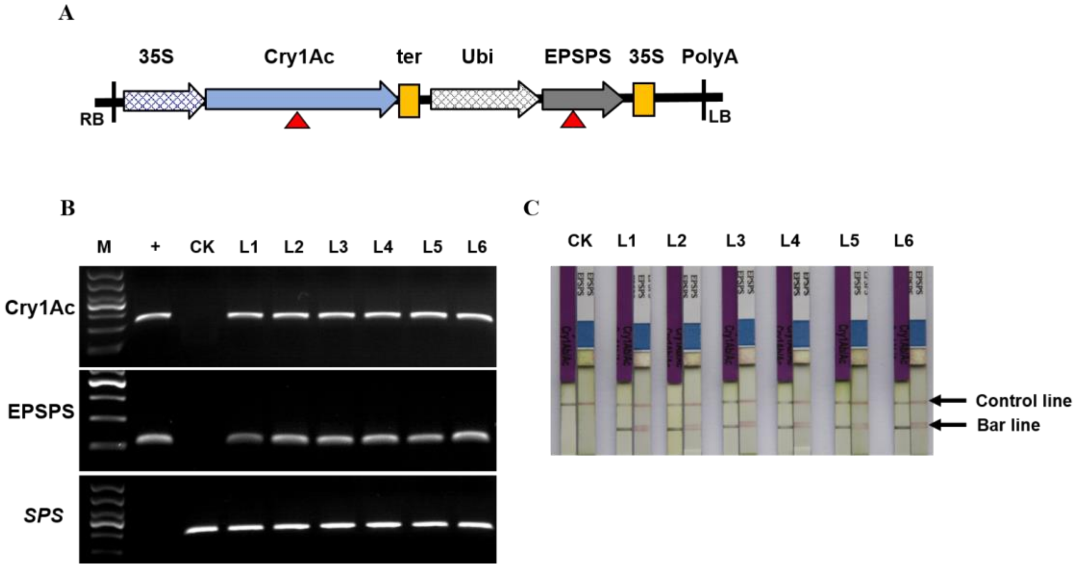

Supplementary Figure S2 Expression level of the transgenes in L1 to L6

(A) Expression levels of the *Cry1Ac* in non-transgenic rice and transgenic rice L1 to L6. (B) Expression levels of the *G10evo*-EPSPS in non-transgenic rice and transgenic rice L1 to L6. Quantitative real-time PCR was analyzed via the $2^{-\triangle\triangle CT}$ method using the *OsActin1* gene as an internal control. Each data point represents the mean (±SD) of three separate experiments. (C) Western blot analysis of expression of the Cry1Ac and EPSPS protein in non-transgenic rice and transgenic rice. One gel was stained with coomassie blue, the other three gels were used to perform western blot using anti-Cry1Ac antibody, anti-EPSPS antibody, and anti-α-Tubulin antibody respectively. Anti-α-Tubulin antibody was used as a loading control.

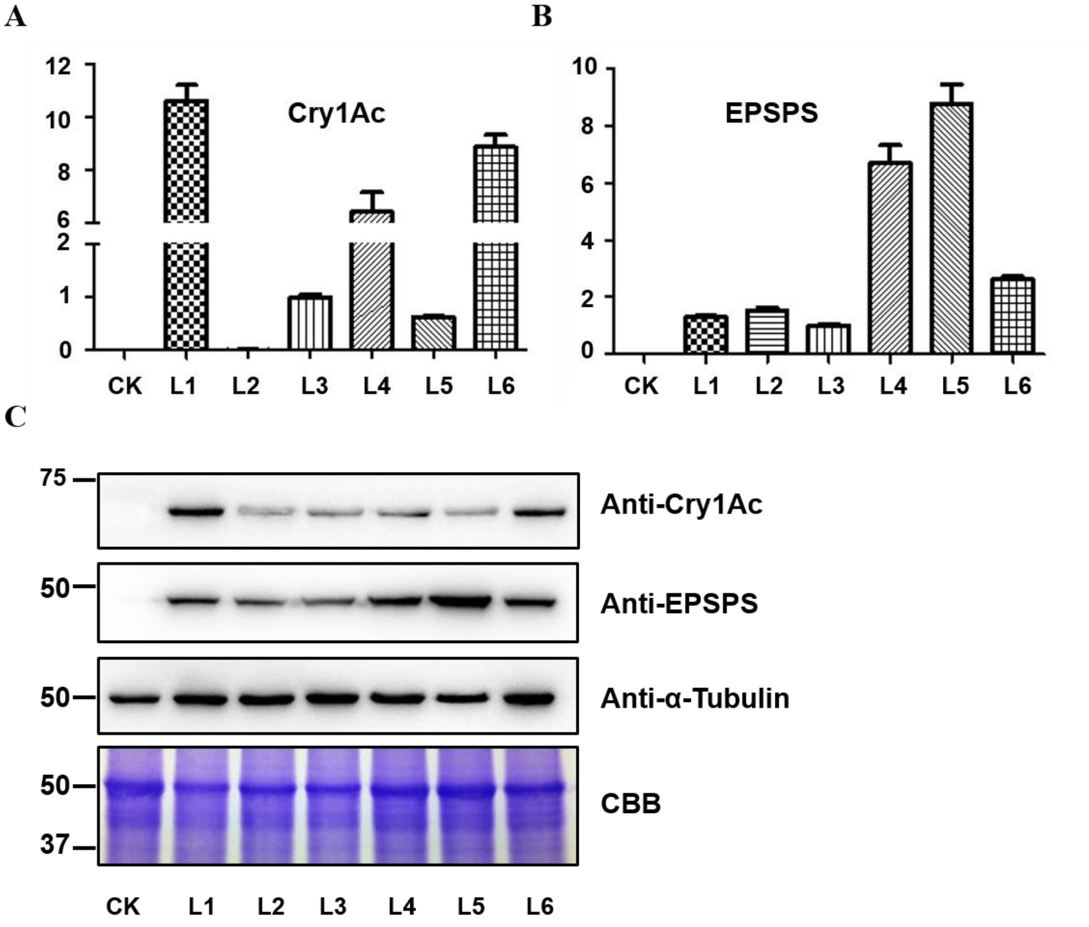

Supplementary Figure S3 Different miRNA expression in volcano diagram.

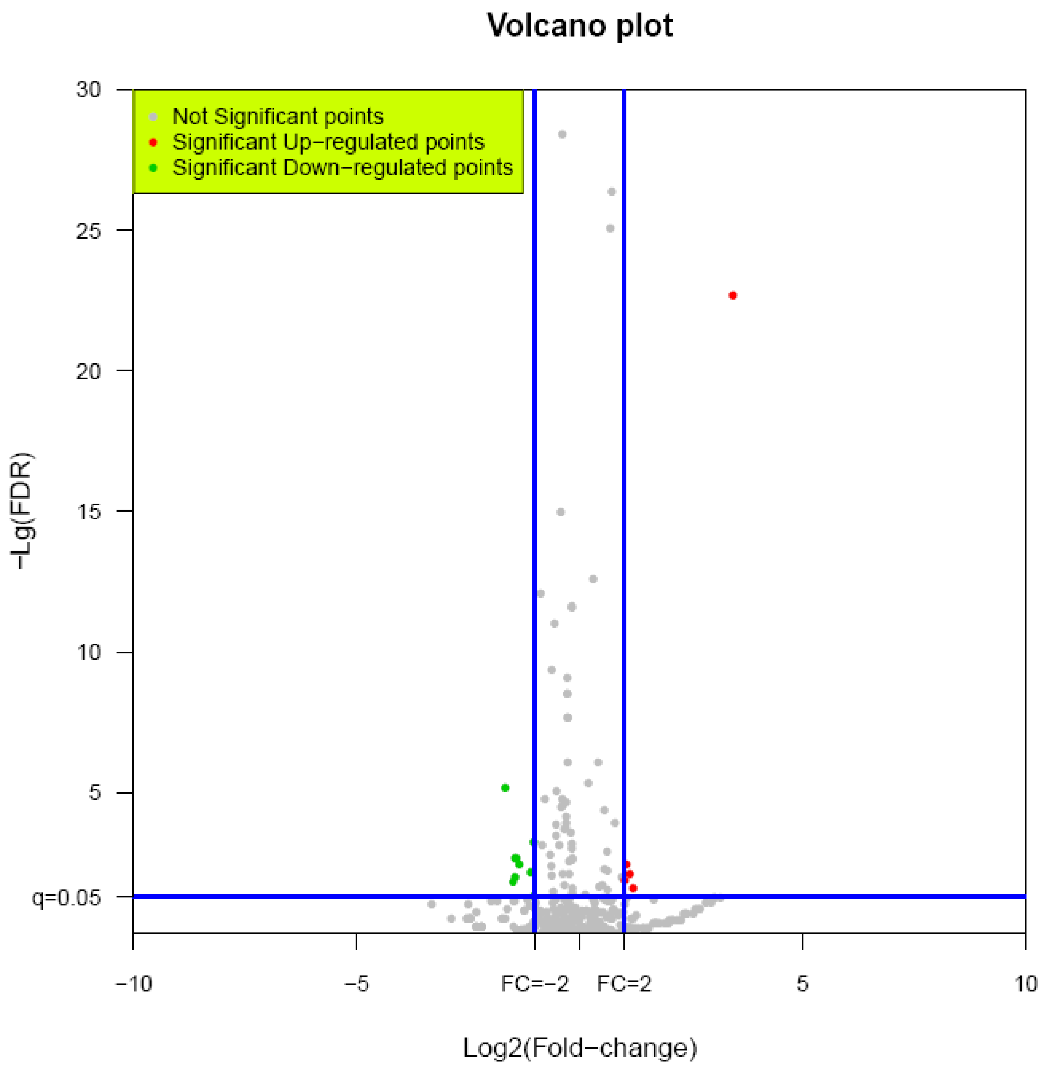

The X axis stands for the fold change of different miRNAs. The Y axis stands for significant difference of miRNA expression changes. Every miRNA are represented with the dots. The colorless dots indicate no significant difference miRNAs; the red dots indicate up-regulated miRNAs; the green dots mean down-regulated miRNAs.

Supplementary Figure S4 Numbers of differentially expressed miRNAs shared between each line are illustrated

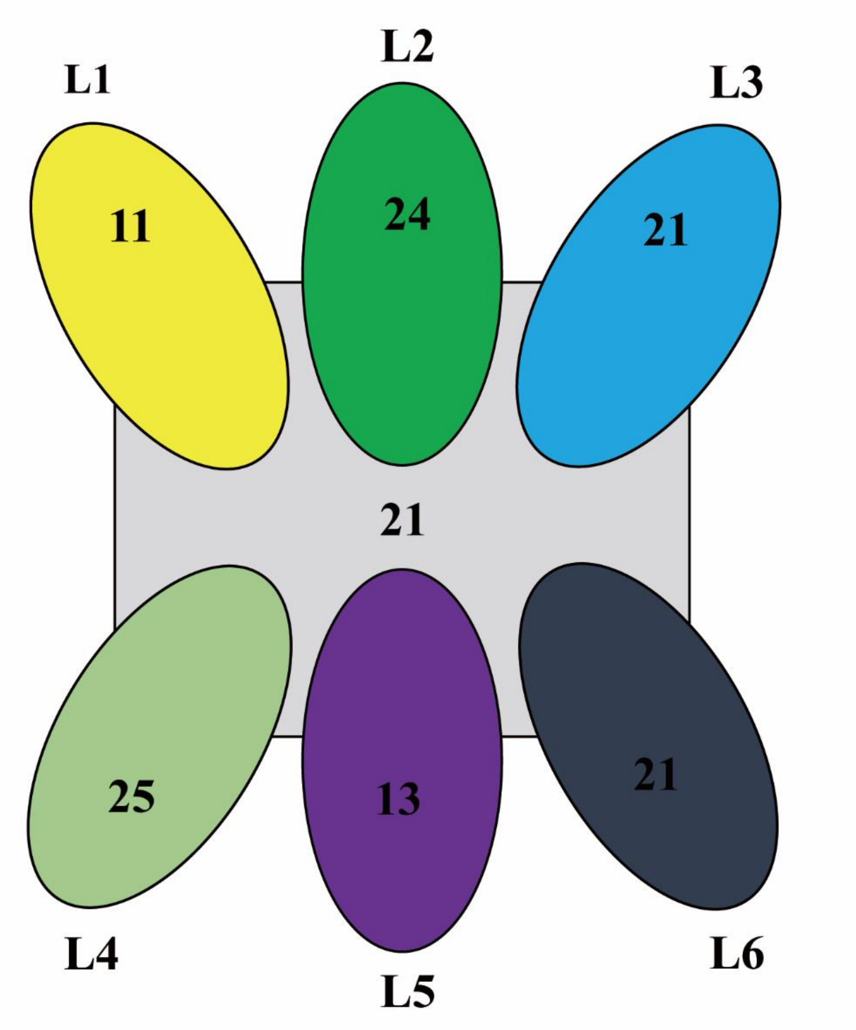

Compared the numbers of differentially expressed miRNAs between each line and wild type. The square indicates the common numbers of differentially expressed miRNAs in each line, the oval indicates the numbers of differentially expressed miRNAs in each line except the common ones.

Supplementary Table 1 Summary of integration events in the six GM lines estimated by various PCR techniques.

**Line**	**Chromosomal positions of T-DNA insertions**		**Copy no.**
	**Chromosome**	**BAC/PAC**	
L1	3	OSJNBb0016P23	1
L2	nd	nd	2
L3	9	OSJNBa0095O23	1
L4	nd	nd	3
L5	3	OSJNBa0004G17	1
L6	1	B1157F09	1

Insert location analysis was carried out only for those transgenic plants in which a single copy T-DNA insertion was confirmed. nd: not determined.

Supplementary Table 2 The primers used for PCR detection

Gene name	Primer name	Primer sequence(5’ to 3’)
Cry1Ac-BT	BT_RT-F	CCAGATCCCGGCTGTGAA
	BT_RT-R	TGGAGAAGATGGAGGAGTTGC
G10evo-EPSPS	EPSPS_RT-F	CACCTCTGGCACCACTTTCG
	EPSPS_RT-R	ACCGTCGTTGGAGGACACCC

Supplementary Table 3 The primers used for quantitative real-time PCR

	Gene name	Primer name	Primer sequence(5’ to 3’)
For mRNA	*Cry1Ac*-Bt	BT_RT-F	CCAGATCCCGGCTGTGAA
		BT_RT-R	TGGAGAAGATGGAGGAGTTGC
	G10evo-EPSPS	EPSPS_RT-F	CACCTCTGGCACCACTTTCG
		EPSPS_RT-R	ACCGTCGTTGGAGGACACCC
	OsActin1	Actin-F	CCCTCCTGAAAGGAAGTACAGTGT
		Actin-R	GTCCGAAGAATTAGAAGCATTTCC
For miRNA	miR1874-5p	miR1874-5p	TGGGTAGGGCTACTACACCATCC
	miR1428b,c,d,f,e	miR1428b,c,d,f,e	GGGCTAAGATAATGCCATGAATTC
	miR399i	miR399i	UGCCAAAGGAGAGCUGCCCUG
	miR2873c	miR2873c	CAAAUGAAGUUGAGUUUGGAC
	miR2118o,e	miR2118o,e	CUCCUGAUGCCUCCCAAGCCUA
	miR1874-3p	miR1874-3p	UAUGGAUGGAGGUGUAACCCGAUG
	miR166e-3p	miR166e-3p	UCGAACCAGGCUUCAUUCCCC
	miR1561-5p	miR1561-5p	CGACAGAAGAGAGUGAGCAUA
	miR529b	miR529b	CGGGAGAAGAGAGAGAGTACAGCTT
	miR168a-5p	miR168a-5p	AGGGTCGCTTGGTGCAGAT
	U6	U6	ACGCACAAATCGAGAAATGGTC

Supplementary Table 4 Summary of miRNA sequencing data

Sample name	Raw Reads	Effective reads	Effective ratio
CK	13580216	13408967	98.74%
L1	15420462	15295655	99.19%
L2	10373946	10243345	98.74%
L3	11339502	11135892	98.20%
L4	12268197	12003639	97.84%
L5	10809352	10676310	98.77%
L6	10263170	10084897	98.26%

Supplementary Table 5 Target prediction results using miRanda

Seq1	Seq2	Tot Score	Tot Energy	Max Score	Max Energy	Strand	Len1	Len2	Positions
miR5799	LOC_Os01g01730.1	142	-20.19	142	-20.19	66441	24	573	347
miR5799	LOC_Os01g02440.1	143	-20.06	143	-20.06	66526	24	1686	943
miR5799	LOC_Os01g03570.1	143	-23.08	143	-23.08	66647	24	2278	323
miR5799	LOC_Os01g03740.1	156	-22.04	156	-22.04	66663	24	3175	142
miR5799	LOC_Os01g04490.1	150	-20.78	150	-20.78	66745	24	1734	420
miR5799	LOC_Os01g04710.1	165	-23.15	165	-23.15	66772	24	1986	1860
miR5799	LOC_Os01g05289.1	151	-21.66	151	-21.66	66839	24	955	228
miR5799	LOC_Os01g09770.1	147	-21.08	147	-21.08	67362	24	2078	1114
miR5799	LOC_Os01g12340.1	171	-21.97	171	-21.97	67633	24	6243	4509
miR5799	LOC_Os01g13730.1	161	-21.99	161	-21.99	67810	24	1948	435
miR5799	LOC_Os01g15039.1	159	-25.48	159	-25.48	67953	24	2183	1060
miR5799	LOC_Os01g16310.1	145	-20.25	145	-20.25	68104	24	3111	339
miR5799	LOC_Os01g16380.1	148	-21.11	148	-21.11	68114	24	975	542
miR5799	LOC_Os01g16860.1	156	-20.87	156	-20.87	68161	24	2673	498
miR5799	LOC_Os01g16960.1	147	-22.55	147	-22.55	68177	24	2445	2149
miR5799	LOC_Os01g18110.1	160	-20.52	160	-20.52	68248	24	1063	422
miR5799	LOC_Os01g18120.1	160	-22.13	160	-22.13	68249	24	1467	434
miR5799	LOC_Os01g23760.1	144	-20.08	144	-20.08	68785	24	1224	114
miR5799	LOC_Os01g27100.1	156	-21.02	156	-21.02	69084	24	540	62
miR5799	LOC_Os01g38720.1	156	-22.6	156	-22.6	70196	24	1080	918
miR5799	LOC_Os01g40430.1	146	-21.26	146	-21.26	70382	24	933	759
miR5799	LOC_Os01g41930.1	145	-20.39	145	-20.39	70543	24	3887	997
miR5799	LOC_Os01g42700.1	149	-21.45	149	-21.45	70627	24	2576	2478
miR5799	LOC_Os01g42700.2	149	-21.45	149	-21.45	70628	24	1875	1781
miR5799	LOC_Os01g42700.3	149	-21.45	149	-21.45	70629	24	1748	1656
miR5799	LOC_Os01g42700.4	149	-21.45	149	-21.45	70630	24	1755	1661
miR5799	LOC_Os01g42700.5	149	-21.45	149	-21.45	70631	24	1837	1743
miR5799	LOC_Os01g43200.1	162	-20.92	162	-20.92	70693	24	342	267
miR5799	LOC_Os01g43430.1	146	-21.12	146	-21.12	70721	24	858	686
miR5799	LOC_Os01g46240.1	155	-24.06	155	-24.06	70977	24	1077	635

miR5799	LOC_Os01g46270.1	157	-21.14	157	-21.14	70980	24	1047	119
miR5799	LOC_Os01g48446.1	140	-21.18	140	-21.18	71248	24	1319	385
miR5799	LOC_Os01g50230.1	142	-20.52	142	-20.52	71447	24	2271	979
miR5799	LOC_Os01g52000.1	174	-24.26	174	-24.26	71653	24	4302	2497
miR5799	LOC_Os01g56020.1	157	-22.6	157	-22.6	72135	24	2595	2157
miR5799	LOC_Os01g56750.1	311	-43.38	158	-21.78	72234	24	1466	1307 282
miR5799	LOC_Os01g57740.1	158	-22.27	158	-22.27	72356	24	1443	4
miR5799	LOC_Os01g59060.1	151	-22.99	151	-22.99	72507	24	756	234
miR5799	LOC_Os01g61044.1	162	-21.54	162	-21.54	72731	24	1917	524
miR5799	LOC_Os01g61044.2	162	-21.54	162	-21.54	72732	24	1812	524
miR5799	LOC_Os01g62200.1	143	-20.52	143	-20.52	72886	24	1804	630
miR5799	LOC_Os01g62200.2	143	-20.52	143	-20.52	72887	24	1574	400
miR5799	LOC_Os01g62520.1	162	-24.47	162	-24.47	72933	24	1026	132
miR5799	LOC_Os01g62945.1	158	-20.03	158	-20.03	72985	24	993	175
miR5799	LOC_Os01g63270.1	158	-20.9	158	-20.9	73022	24	3019	342
miR5799	LOC_Os01g63270.2	158	-20.9	158	-20.9	73023	24	3287	243
miR5799	LOC_Os01g64520.1	177	-23.77	177	-23.77	73177	24	1570	95
miR5799	LOC_Os01g64520.2	177	-23.77	177	-23.77	73178	24	1585	95
miR5799	LOC_Os01g65060.1	141	-20.39	141	-20.39	73251	24	1743	96
miR5799	LOC_Os01g65330.1	150	-20.36	150	-20.36	73284	24	2336	210
miR5799	LOC_Os01g65330.2	150	-20.36	150	-20.36	73285	24	2422	210
miR5799	LOC_Os01g65790.1	141	-21.07	141	-21.07	73345	24	2023	1014
miR5799	LOC_Os01g66140.1	158	-21.48	158	-21.48	73387	24	3255	1391
miR5799	LOC_Os01g66530.1	149	-20.01	149	-20.01	73427	24	904	449
miR5799	LOC_Os01g66590.1	169	-24.84	169	-24.84	73432	24	1555	1147
miR5799	LOC_Os01g66590.2	169	-24.84	169	-24.84	73433	24	1702	1294
miR5799	LOC_Os01g66590.3	169	-24.84	169	-24.84	73434	24	1433	1147
miR5799	LOC_Os01g70190.1	175	-22.48	175	-22.48	73821	24	2200	272
miR5799	LOC_Os01g71810.1	178	-21.89	178	-21.89	74023	24	1211	88
miR5799	LOC_Os01g71820.1	174	-20.05	174	-20.05	74024	24	1230	111
miR5799	LOC_Os01g72930.1	158	-25.59	158	-25.59	74151	24	2817	424
miR5799	LOC_Os01g73250.1	171	-22.89	171	-22.89	74189	24	1340	957

miR5799	LOC_Os02g10470.1	176	-21.3	176	-21.3	75512	24	1227	634
miR5799	LOC_Os02g11070.1	171	-29.02	171	-29.02	75594	24	2035	832
miR5799	LOC_Os02g11070.2	171	-29.02	171	-29.02	75595	24	2054	851
miR5799	LOC_Os02g12310.1	144	-21.84	144	-21.84	75678	24	1130	392
miR5799	LOC_Os02g13210.1	172	-23.19	172	-23.19	75805	24	1650	1248
miR5799	LOC_Os02g13900.1	173	-23.73	173	-23.73	75882	24	1456	269
miR5799	LOC_Os02g14760.1	321	-45.66	163	-24.15	75974	24	1658	1394 456
miR5799	LOC_Os02g14760.2	321	-45.66	163	-24.15	75975	24	1785	1521 456
miR5799	LOC_Os02g17304.1	176	-26.04	176	-26.04	76266	24	3188	232
miR5799	LOC_Os02g18000.1	173	-21.65	173	-21.65	76347	24	4212	1126
miR5799	LOC_Os02g18650.1	141	-23.13	141	-23.13	76419	24	2015	1092
miR5799	LOC_Os02g20660.1	165	-23.72	165	-23.72	76626	24	4584	1894
miR5799	LOC_Os02g25680.1	147	-23.19	147	-23.19	77080	24	995	352
miR5799	LOC_Os02g25680.2	147	-23.19	147	-23.19	77081	24	1096	339
miR5799	LOC_Os02g26060.1	152	-20.13	152	-20.13	77118	24	3768	2073
miR5799	LOC_Os02g26190.1	157	-20.35	157	-20.35	77131	24	6728	4956
miR5799	LOC_Os02g26190.2	157	-20.35	157	-20.35	77132	24	6844	4956
miR5799	LOC_Os02g26290.1	154	-21.51	154	-21.51	77142	24	1066	839
miR5799	LOC_Os02g27110.1	160	-20.08	160	-20.08	77251	24	9893	4734
miR5799	LOC_Os02g32460.1	169	-21.68	169	-21.68	77780	24	1743	542
miR5799	LOC_Os02g38790.1	155	-22.39	155	-22.39	78514	24	1416	732
miR5799	LOC_Os02g40310.1	163	-21.47	163	-21.47	78720	24	2601	1740
miR5799	LOC_Os02g41460.1	158	-20.96	158	-20.96	78798	24	1212	480
miR5799	LOC_Os02g42310.1	154	-22.48	154	-22.48	78898	24	1740	336
miR5799	LOC_Os02g42406.1	148	-21.01	148	-21.01	78923	24	2204	2179
miR5799	LOC_Os02g42780.1	150	-23.42	150	-23.42	78965	24	2128	1354
miR5799	LOC_Os02g43370.1	171	-22.08	171	-22.08	79036	24	3207	782
miR5799	LOC_Os02g44680.1	145	-20.74	145	-20.74	79217	24	1741	1286
miR5799	LOC_Os02g44930.1	165	-20.04	165	-20.04	79250	24	1101	234
miR5799	LOC_Os02g45000.1	158	-23.08	158	-23.08	79257	24	1626	678
miR5799	LOC_Os02g45920.1	149	-24.94	149	-24.94	79367	24	2043	67
miR5799	LOC_Os02g47860.1	161	-23.06	161	-23.06	79613	24	2753	1275

miR5799	LOC_Os02g47860.2	161	-23.06	161	-23.06	79614	24	2645	1167
miR5799	LOC_Os02g50330.1	146	-21.11	146	-21.11	79923	24	3602	1106
miR5799	LOC_Os02g51600.1	158	-22.36	158	-22.36	80083	24	1899	1107
miR5799	LOC_Os02g51770.1	150	-20.82	150	-20.82	80101	24	1695	1307
miR5799	LOC_Os02g51770.2	150	-20.82	150	-20.82	80102	24	1513	1125
miR5799	LOC_Os02g51770.3	150	-20.82	150	-20.82	80103	24	1803	1415
miR5799	LOC_Os02g51770.4	150	-20.82	150	-20.82	80104	24	2338	1950
miR5799	LOC_Os02g51770.5	150	-20.82	150	-20.82	80105	24	1669	1281
miR5799	LOC_Os02g56610.1	162	-24.32	162	-24.32	80709	24	1587	689
miR5799	LOC_Os02g57810.1	149	-20.61	149	-20.61	80886	24	1596	257
miR5799	LOC_Os02g58590.1	166	-25.94	166	-25.94	80987	24	1347	81
miR5799	LOC_Os03g01360.1	172	-21.66	172	-21.66	81069	24	2430	131
miR5799	LOC_Os03g01360.2	172	-21.66	172	-21.66	81070	24	2753	131
miR5799	LOC_Os03g01360.3	172	-21.66	172	-21.66	81071	24	2856	131
miR5799	LOC_Os03g01360.4	172	-21.66	172	-21.66	81072	24	2852	131
miR5799	LOC_Os03g02920.1	148	-20.06	148	-20.06	81291	24	1584	837
miR5799	LOC_Os03g05850.1	174	-24.26	174	-24.26	81658	24	4230	2497
miR5799	LOC_Os03g08710.1	153	-23.41	153	-23.41	82022	24	727	254
miR5799	LOC_Os03g08710.2	153	-23.41	153	-23.41	82023	24	858	385
miR5799	LOC_Os03g09230.2	168	-20.31	168	-20.31	82083	24	1083	55
miR5799	LOC_Os03g10230.1	159	-22.02	159	-22.02	82152	24	2116	1047
miR5799	LOC_Os03g10950.1	146	-20.02	146	-20.02	82238	24	1780	295
miR5799	LOC_Os03g11650.1	147	-20.12	147	-20.12	82325	24	2283	1864
miR5799	LOC_Os03g12000.1	153	-22.49	153	-22.49	82367	24	2130	2097
miR5799	LOC_Os03g14050.2	186	-23.8	186	-23.8	82649	24	3093	1836
miR5799	LOC_Os03g16320.1	142	-20.52	142	-20.52	82944	24	2442	982
miR5799	LOC_Os03g16334.1	146	-20.41	146	-20.41	82945	24	2245	1380
miR5799	LOC_Os03g19670.1	159	-20.09	159	-20.09	83349	24	1711	366
miR5799	LOC_Os03g19670.2	159	-20.09	159	-20.09	83350	24	1801	366
miR5799	LOC_Os03g20680.1	146	-21.61	146	-21.61	83474	24	1325	817
miR5799	LOC_Os03g21890.1	153	-20.13	153	-20.13	83616	24	2577	1666
miR5799	LOC_Os03g22050.1	186	-32.28	186	-32.28	83636	24	3760	692

miR5799	LOC_Os03g22050.2	186	-32.28	186	-32.28	83637	24	3754	692
miR5799	LOC_Os03g22050.3	186	-32.28	186	-32.28	83638	24	3723	692
miR5799	LOC_Os03g22050.4	186	-32.28	186	-32.28	83639	24	2768	692
miR5799	LOC_Os03g24820.1	165	-23.37	165	-23.37	83888	24	2553	654
miR5799	LOC_Os03g25750.1	162	-20.39	162	-20.39	83996	24	1812	124
miR5799	LOC_Os03g28110.1	178	-26.01	178	-26.01	84244	24	3912	2134
miR5799	LOC_Os03g28980.1	151	-20.81	151	-20.81	84286	24	2622	362
miR5799	LOC_Os03g29180.1	156	-20.44	156	-20.44	84307	24	1403	1348
miR5799	LOC_Os03g29540.2	172	-23.19	172	-23.19	84348	24	4133	3093
miR5799	LOC_Os03g33230.1	149	-20.03	149	-20.03	84714	24	4896	2
miR5799	LOC_Os03g35630.1	163	-22.63	163	-22.63	84851	24	252	12
miR5799	LOC_Os03g36320.1	144	-21.72	144	-21.72	84908	24	414	112
miR5799	LOC_Os03g37720.1	158	-20.62	158	-20.62	85031	24	3801	2571
miR5799	LOC_Os03g38010.1	176	-21.37	176	-21.37	85069	24	1813	577
miR5799	LOC_Os03g41060.1	160	-20.84	160	-20.84	85353	24	773	631
miR5799	LOC_Os03g44360.1	155	-22.62	155	-22.62	85693	24	3900	1128
miR5799	LOC_Os03g45050.1	152	-20.12	152	-20.12	85779	24	3867	2190
miR5799	LOC_Os03g45290.1	149	-22.07	149	-22.07	85798	24	1671	1459
miR5799	LOC_Os03g45690.1	162	-22.99	162	-22.99	85832	24	831	624
miR5799	LOC_Os03g45990.1	185	-27.22	185	-27.22	85864	24	4865	2013
miR5799	LOC_Os03g46250.1	179	-23.02	179	-23.02	85893	24	991	941
miR5799	LOC_Os03g46400.1	165	-22.47	165	-22.47	85910	24	2278	2207
miR5799	LOC_Os03g47034.1	148	-23.17	148	-23.17	85977	24	574	337
miR5799	LOC_Os03g51080.1	148	-21.33	148	-21.33	86455	24	2184	239
miR5799	LOC_Os03g51330.1	154	-20.57	154	-20.57	86482	24	2547	827
miR5799	LOC_Os03g56270.1	149	-20.95	149	-20.95	87018	24	3741	1323
miR5799	LOC_Os03g56270.2	149	-20.95	149	-20.95	87019	24	3306	1317
miR5799	LOC_Os03g56270.3	149	-20.95	149	-20.95	87020	24	3553	1317
miR5799	LOC_Os03g56910.1	165	-21.44	165	-21.44	87109	24	3408	2958
miR5799	LOC_Os03g60570.1	143	-21.25	143	-21.25	87537	24	915	244
miR5799	LOC_Os03g61319.1	160	-21.1	160	-21.1	87632	24	951	88
miR5799	LOC_Os03g63910.1	141	-20.08	141	-20.08	87974	24	2793	924

miR5799	LOC_Os04g01790.1	155	-20.03	155	-20.03	88130	24	1710	12
miR5799	LOC_Os04g05280.1	142	-21.25	142	-21.25	88480	24	441	328
miR5799	LOC_Os04g05310.1	142	-21.25	142	-21.25	88483	24	2100	328
miR5799	LOC_Os04g06030.1	154	-20.04	154	-20.04	88549	24	2171	1564
miR5799	LOC_Os04g06290.1	162	-24.47	162	-24.47	88571	24	2298	621
miR5799	LOC_Os04g09580.1	161	-21.03	161	-21.03	88863	24	6520	914
miR5799	LOC_Os04g09570.1	161	-21.03	161	-21.03	88864	24	1151	913
miR5799	LOC_Os04g10270.1	142	-23.72	142	-23.72	88931	24	2652	807
miR5799	LOC_Os04g16590.1	153	-20.46	153	-20.46	89489	24	1536	181
miR5799	LOC_Os04g17540.1	171	-20.32	171	-20.32	89618	24	1263	602
miR5799	LOC_Os04g19760.1	165	-20.97	165	-20.97	89835	24	2643	1883
miR5799	LOC_Os04g20474.1	318	-50.89	178	-30.28	89905	24	4313	2416 167
miR5799	LOC_Os04g20474.2	318	-50.89	178	-30.28	89906	24	4181	2285 167
miR5799	LOC_Os04g20474.3	178	-30.28	178	-30.28	89907	24	3843	1947
miR5799	LOC_Os04g20474.4	318	-50.89	178	-30.28	89908	24	4085	2189 167
miR5799	LOC_Os04g20630.1	158	-22.63	158	-22.63	89922	24	615	27
miR5799	LOC_Os04g25800.1	145	-21.15	145	-21.15	90425	24	1661	288
miR5799	LOC_Os04g29650.1	160	-21	160	-21	90788	24	3198	524
miR5799	LOC_Os04g30420.1	157	-23.88	157	-23.88	90861	24	1426	91
miR5799	LOC_Os04g30470.1	156	-20.31	156	-20.31	90866	24	1887	177
miR5799	LOC_Os04g30790.1	158	-23.64	158	-23.64	90899	24	1530	1402
miR5799	LOC_Os04g30790.2	158	-23.64	158	-23.64	90900	24	1716	1588
miR5799	LOC_Os04g33720.1	182	-28.11	182	-28.11	91230	24	2288	1952
miR5799	LOC_Os04g33780.1	194	-30.19	194	-30.19	91234	24	2679	2628
miR5799	LOC_Os04g33780.2	194	-30.19	194	-30.19	91235	24	1565	1514
miR5799	LOC_Os04g35690.1	152	-20.56	152	-20.56	91441	24	1443	862
miR5799	LOC_Os04g35700.1	153	-23.24	153	-23.24	91442	24	2743	457
miR5799	LOC_Os04g37820.1	177	-21.28	177	-21.28	91573	24	1715	1636
miR5799	LOC_Os04g38230.1	151	-21.82	151	-21.82	91613	24	1473	301
miR5799	LOC_Os04g40920.1	166	-21.91	166	-21.91	91951	24	1643	108
miR5799	LOC_Os04g40920.2	166	-21.91	166	-21.91	91952	24	1664	108
miR5799	LOC_Os04g44354.3	171	-21.85	171	-21.85	92365	24	1819	1701

miR5799	LOC_Os04g44370.1	165	-21.45	165	-21.45	92367	24	4962	2997
miR5799	LOC_Os04g45700.1	157	-22.27	157	-22.27	92529	24	1119	10
miR5799	LOC_Os04g48380.1	176	-23.5	176	-23.5	92861	24	900	653
miR5799	LOC_Os04g49194.1	145	-20.06	145	-20.06	92954	24	1366	49
miR5799	LOC_Os04g50840.1	150	-23.33	150	-23.33	93096	24	2331	871
miR5799	LOC_Os04g51120.1	155	-21.58	155	-21.58	93129	24	2391	2232
miR5799	LOC_Os04g51120.3	155	-21.58	155	-21.58	93130	24	2623	2464
miR5799	LOC_Os04g51740.1	154	-22.69	154	-22.69	93210	24	5280	2697
miR5799	LOC_Os04g55920.1	330	-42.34	165	-21.17	93730	24	1205	1035 1153
miR5799	LOC_Os04g55920.2	330	-42.34	165	-21.17	93731	24	1202	1032 1150
miR5799	LOC_Os04g55920.5	330	-42.34	165	-21.17	93732	24	1036	866 984
miR5799	LOC_Os04g57810.1	157	-20.44	157	-20.44	93987	24	1654	131
miR5799	LOC_Os04g57810.3	157	-20.44	157	-20.44	93989	24	1852	128
miR5799	LOC_Os04g58220.1	146	-20.43	146	-20.43	94037	24	1617	1458
miR5799	LOC_Os04g58730.1	186	-27.77	186	-27.77	94102	24	3379	3020
miR5799	LOC_Os04g58770.1	166	-24.95	166	-24.95	94110	24	651	10
miR5799	LOC_Os04g59460.1	159	-20.83	159	-20.83	94187	24	5636	623
miR5799	LOC_Os05g02620.1	175	-21.23	175	-21.23	94450	24	782	492
miR5799	LOC_Os05g02790.1	169	-20.16	169	-20.16	94472	24	3105	1712
miR5799	LOC_Os05g03550.1	170	-21.81	170	-21.81	94562	24	5167	1234
miR5799	LOC_Os05g03620.1	149	-21.75	149	-21.75	94570	24	2731	195
miR5799	LOC_Os05g04860.1	156	-20.42	156	-20.42	94706	24	1464	1084
miR5799	LOC_Os05g04860.2	156	-20.42	156	-20.42	94707	24	2168	1085
miR5799	LOC_Os05g05080.1	159	-21.12	159	-21.12	94728	24	515	367
miR5799	LOC_Os05g06290.1	151	-21.95	151	-21.95	94872	24	929	246
miR5799	LOC_Os05g07560.1	177	-23.77	177	-23.77	95021	24	4291	3608
miR5799	LOC_Os05g11400.1	158	-21.72	158	-21.72	95426	24	1329	125
miR5799	LOC_Os05g16970.1	144	-20.07	144	-20.07	95959	24	2189	1885
miR5799	LOC_Os05g24960.1	162	-20.28	162	-20.28	96706	24	1902	933
miR5799	LOC_Os05g26820.1	165	-20.37	165	-20.37	96905	24	2111	1245
miR5799	LOC_Os05g34480.1	156	-24.46	156	-24.46	97705	24	1281	159
miR5799	LOC_Os05g34480.2	156	-24.46	156	-24.46	97706	24	848	166

miR5799	LOC_Os05g35860.1	156	-20.27	156	-20.27	97868	24	3795	1246
miR5799	LOC_Os05g39040.1	166	-21.21	166	-21.21	98210	24	3537	2377
miR5799	LOC_Os05g39540.1	182	-28.57	182	-28.57	98273	24	1640	1308
miR5799	LOC_Os05g46550.1	178	-28.81	178	-28.81	99056	24	1949	295
miR5799	LOC_Os05g46840.1	146	-21.8	146	-21.8	99091	24	932	53
miR5799	LOC_Os05g47446.2	165	-21.5	165	-21.5	99106	24	1063	603
miR5799	LOC_Os05g49120.1	175	-22.48	175	-22.48	99322	24	2351	1734
miR5799	LOC_Os05g49120.2	175	-22.48	175	-22.48	99323	24	2236	1619
miR5799	LOC_Os06g01210.1	173	-20.88	173	-20.88	99724	24	863	97
miR5799	LOC_Os06g02460.1	171	-22.17	171	-22.17	99869	24	1392	610
miR5799	LOC_Os06g02810.1	175	-20.46	175	-20.46	99913	24	1042	929
miR5799	LOC_Os06g02830.1	159	-22.01	159	-22.01	99915	24	2741	1369
miR5799	LOC_Os06g03676.1	161	-20.72	161	-20.72	100004	24	2015	1322
miR5799	LOC_Os06g03820.1	173	-26.66	173	-26.66	100023	24	1577	1232
miR5799	LOC_Os06g04840.1	185	-27.22	185	-27.22	100146	24	3516	78
miR5799	LOC_Os06g04840.2	185	-27.22	185	-27.22	100147	24	3505	78
miR5799	LOC_Os06g05300.1	152	-20.02	152	-20.02	100205	24	1470	117
miR5799	LOC_Os06g06850.1	155	-22.61	155	-22.61	100409	24	3344	2757
miR5799	LOC_Os06g07896.1	161	-25.44	161	-25.44	100534	24	1401	552
miR5799	LOC_Os06g08960.1	141	-21.45	141	-21.45	100649	24	1263	718
miR5799	LOC_Os06g09220.1	141	-21.45	141	-21.45	100678	24	1725	892
miR5799	LOC_Os06g09230.2	150	-20.73	150	-20.73	100679	24	3082	1133
miR5799	LOC_Os06g09717.1	145	-20.65	145	-20.65	100738	24	624	143
miR5799	LOC_Os06g13190.1	168	-21.86	168	-21.86	101137	24	789	454
miR5799	LOC_Os06g14460.1	149	-22.51	149	-22.51	101279	24	1520	1236
miR5799	LOC_Os06g14780.1	172	-23.19	172	-23.19	101314	24	2375	1176
miR5799	LOC_Os06g17090.1	141	-20.3	141	-20.3	101549	24	1107	943
miR5799	LOC_Os06g17120.1	141	-20.3	141	-20.3	101552	24	1452	939
miR5799	LOC_Os06g17880.1	181	-22.59	181	-22.59	101626	24	3228	2608
miR5799	LOC_Os06g19660.2	147	-21.32	147	-21.32	101757	24	3989	1840
miR5799	LOC_Os06g20090.1	153	-22.41	153	-22.41	101801	24	5669	5025
miR5799	LOC_Os06g24390.1	158	-25.51	158	-25.51	102260	24	243	48

miR5799	LOC_Os06g25670.1	142	-20.77	142	-20.77	102387	24	2176	629
miR5799	LOC_Os06g29880.1	150	-21.85	150	-21.85	102726	24	555	355
miR5799	LOC_Os06g30790.1	141	-20.74	141	-20.74	102817	24	1428	1008
miR5799	LOC_Os06g31780.1	161	-21.79	161	-21.79	102917	24	555	66
miR5799	LOC_Os06g40784.1	176	-26.31	176	-26.31	103856	24	488	331
miR5799	LOC_Os06g45940.1	146	-20.45	146	-20.45	104454	24	2954	1853
miR5799	LOC_Os06g45940.2	146	-20.45	146	-20.45	104455	24	2885	1784
miR5799	LOC_Os07g02120.1	179	-24.31	179	-24.31	105303	24	1609	1496
miR5799	LOC_Os07g02960.1	168	-21.08	168	-21.08	105392	24	3538	3150
miR5799	LOC_Os07g02960.2	168	-21.08	168	-21.08	105393	24	2741	2353
miR5799	LOC_Os07g02960.3	168	-21.08	168	-21.08	105394	24	1913	1525
miR5799	LOC_Os07g05430.1	174	-24.26	174	-24.26	105650	24	4302	2497
miR5799	LOC_Os07g06190.1	147	-21.59	147	-21.59	105733	24	4374	378
miR5799	LOC_Os07g06300.1	147	-21.59	147	-21.59	105741	24	4539	378
miR5799	LOC_Os07g07010.1	151	-22.46	151	-22.46	105827	24	1974	747
miR5799	LOC_Os07g07490.1	146	-20.52	146	-20.52	105882	24	2523	1894
miR5799	LOC_Os07g07490.2	146	-20.52	146	-20.52	105883	24	2451	1822
miR5799	LOC_Os07g13690.1	148	-20.43	148	-20.43	106546	24	1812	508
miR5799	LOC_Os07g26595.1	190	-27.66	190	-27.66	107730	24	4143	3853
miR5799	LOC_Os07g27970.1	170	-22.23	170	-22.23	107878	24	1424	118
miR5799	LOC_Os07g28570.1	176	-23.51	176	-23.51	107940	24	2752	2
miR5799	LOC_Os07g33550.1	176	-23.51	176	-23.51	108474	24	2846	1659
miR5799	LOC_Os07g35130.1	163	-21.07	163	-21.07	108640	24	1812	862
miR5799	LOC_Os07g35650.1	155	-22.5	155	-22.5	108703	24	2049	1936
miR5799	LOC_Os07g36960.1	161	-20.33	161	-20.33	108852	24	1897	727
miR5799	LOC_Os07g36960.2	161	-20.33	161	-20.33	108853	24	1712	727
miR5799	LOC_Os07g39290.1	175	-22.97	175	-22.97	109116	24	1130	998
miR5799	LOC_Os07g39830.1	179	-24.18	179	-24.18	109182	24	1535	1506
miR5799	LOC_Os07g40180.1	140	-20.13	140	-20.13	109220	24	450	369
miR5799	LOC_Os07g42410.1	170	-20.63	170	-20.63	109469	24	4850	737
miR5799	LOC_Os07g47100.2	160	-20.03	160	-20.03	110025	24	2394	128
miR5799	LOC_Os07g47230.1	145	-21.1	145	-21.1	110044	24	2022	402

miR5799	LOC_Os07g47760.1	180	-26.04	180	-26.04	110124	24	644	488
miR5799	LOC_Os07g47920.1	152	-20.67	152	-20.67	110144	24	2601	570
miR5799	LOC_Os07g49530.1	170	-21.76	170	-21.76	110354	24	1623	464
miR5799	LOC_Os08g02996.1	157	-21.39	157	-21.39	110593	24	2578	2318
miR5799	LOC_Os08g02996.2	157	-21.39	157	-21.39	110594	24	2434	2174
miR5799	LOC_Os08g04170.1	159	-22.08	159	-22.08	110735	24	3695	2639
miR5799	LOC_Os08g06260.1	163	-23.7	163	-23.7	110968	24	237	174
miR5799	LOC_Os08g06600.1	176	-21.37	176	-21.37	111019	24	1750	567
miR5799	LOC_Os08g12740.1	159	-23.24	159	-23.24	111670	24	3264	3232
miR5799	LOC_Os08g12740.2	159	-23.24	159	-23.24	111671	24	3063	3017
miR5799	LOC_Os08g13740.1	161	-26.07	161	-26.07	111783	24	828	369
miR5799	LOC_Os08g17090.1	161	-21.45	161	-21.45	112155	24	1212	468
miR5799	LOC_Os08g17540.1	157	-20.27	157	-20.27	112196	24	597	434
miR5799	LOC_Os08g17890.1	158	-20.13	158	-20.13	112232	24	4483	1164
miR5799	LOC_Os08g20660.1	172	-22.83	172	-22.83	112470	24	3879	3777
miR5799	LOC_Os08g20660.2	172	-22.83	172	-22.83	112471	24	3869	3767
miR5799	LOC_Os08g22760.1	152	-20.13	152	-20.13	112657	24	5106	2898
miR5799	LOC_Os08g28710.1	171	-22.17	171	-22.17	113274	24	2042	119
miR5799	LOC_Os08g30300.1	162	-24.47	162	-24.47	113441	24	1440	543
miR5799	LOC_Os08g30900.1	152	-20.83	152	-20.83	113500	24	4506	1059
miR5799	LOC_Os08g30900.2	152	-20.83	152	-20.83	113501	24	3928	570
miR5799	LOC_Os08g31250.1	157	-22.82	157	-22.82	113535	24	1068	371
miR5799	LOC_Os08g32630.1	161	-25	161	-25	113691	24	2221	103
miR5799	LOC_Os08g32630.2	161	-25	161	-25	113692	24	2234	103
miR5799	LOC_Os08g32630.3	161	-25	161	-25	113693	24	2336	103
miR5799	LOC_Os08g32630.4	161	-25	161	-25	113694	24	2312	103
miR5799	LOC_Os08g32630.5	161	-25	161	-25	113695	24	2309	103
miR5799	LOC_Os08g37210.1	165	-23.76	165	-23.76	114206	24	1722	1681
miR5799	LOC_Os08g39980.1	142	-20.55	142	-20.55	114561	24	2545	1889
miR5799	LOC_Os08g40200.1	158	-21.36	158	-21.36	114587	24	1667	448
miR5799	LOC_Os08g40490.1	152	-24.72	152	-24.72	114614	24	1509	882
miR5799	LOC_Os08g43050.1	177	-23.01	177	-23.01	114937	24	3966	2529

miR5799	LOC_Os08g43740.1	153	-20.21	153	-20.21	115018	24	1383	503
miR5799	LOC_Os08g44280.1	171	-22.54	171	-22.54	115077	24	2316	1136
miR5799	LOC_Os08g44280.2	171	-22.54	171	-22.54	115078	24	1759	579
miR5799	LOC_Os08g44450.1	171	-22.17	171	-22.17	115103	24	1203	983
miR5799	LOC_Os08g44450.2	171	-22.17	171	-22.17	115104	24	1203	983
miR5799	LOC_Os08g44450.3	171	-22.17	171	-22.17	115105	24	1276	1056
miR5799	LOC_Os09g07320.2	177	-23.39	177	-23.39	115781	24	3042	1135
miR5799	LOC_Os09g07870.1	170	-20.4	170	-20.4	115849	24	2412	2323
miR5799	LOC_Os09g09810.1	148	-20.24	148	-20.24	116048	24	2490	2332
miR5799	LOC_Os09g09820.1	148	-20.24	148	-20.24	116049	24	2964	237
miR5799	LOC_Os09g12400.1	147	-21.53	147	-21.53	116311	24	765	616
miR5799	LOC_Os09g13180.1	159	-22.4	159	-22.4	116395	24	4359	3568
miR5799	LOC_Os09g16380.1	150	-22.72	150	-22.72	116740	24	4161	1248
miR5799	LOC_Os09g17180.1	160	-23.99	160	-23.99	116825	24	414	88
miR5799	LOC_Os09g18020.1	158	-20.13	158	-20.13	116912	24	3707	1623
miR5799	LOC_Os09g20240.1	161	-20.22	161	-20.22	117081	24	1286	1152
miR5799	LOC_Os09g20240.2	161	-20.22	161	-20.22	117082	24	903	769
miR5799	LOC_Os09g20970.1	150	-23.69	150	-23.69	117167	24	2064	846
miR5799	LOC_Os09g23350.1	151	-22.89	151	-22.89	117363	24	4231	1103
miR5799	LOC_Os09g23620.1	175	-21.53	175	-21.53	117392	24	1404	73
miR5799	LOC_Os09g24720.1	157	-23.47	157	-23.47	117512	24	633	282
miR5799	LOC_Os09g27100.1	163	-20.35	163	-20.35	117780	24	1167	1101
miR5799	LOC_Os09g27220.1	164	-23.26	164	-23.26	117794	24	1219	1042
miR5799	LOC_Os09g28600.1	149	-20.17	149	-20.17	117967	24	1174	409
miR5799	LOC_Os09g29284.1	150	-20.69	150	-20.69	118043	24	1711	440
miR5799	LOC_Os09g37540.1	186	-30.55	186	-30.55	118848	24	1232	1172
miR5799	LOC_Os10g01470.1	148	-20.8	148	-20.8	119194	24	1450	1211
miR5799	LOC_Os10g01984.1	147	-23.86	147	-23.86	119247	24	5857	1732
miR5799	LOC_Os10g04600.1	153	-20.62	153	-20.62	119510	24	1747	788
miR5799	LOC_Os10g04600.2	153	-20.62	153	-20.62	119511	24	1802	788
miR5799	LOC_Os10g04600.3	153	-20.62	153	-20.62	119512	24	1858	788
miR5799	LOC_Os10g04674.1	155	-20.67	155	-20.67	119521	24	3397	1932

miR5799	LOC_Os10g04674.2	155	-20.67	155	-20.67	119522	24	3381	1932
miR5799	LOC_Os10g04674.3	155	-20.67	155	-20.67	119523	24	3441	1976
miR5799	LOC_Os10g05730.1	146	-21.8	146	-21.8	119629	24	2799	1464
miR5799	LOC_Os10g05730.2	146	-21.8	146	-21.8	119630	24	2429	1090
miR5799	LOC_Os10g06210.1	159	-20.67	159	-20.67	119679	24	927	256
miR5799	LOC_Os10g07010.1	159	-23.66	159	-23.66	119755	24	1896	675
miR5799	LOC_Os10g11910.1	159	-20.68	159	-20.68	120229	24	1389	12
miR5799	LOC_Os10g12740.1	145	-24.2	145	-24.2	120297	24	4428	138
miR5799	LOC_Os10g17140.1	155	-20.06	155	-20.06	120550	24	963	716
miR5799	LOC_Os10g19160.1	157	-20.6	157	-20.6	120733	24	3299	2055
miR5799	LOC_Os10g20820.1	157	-21.63	157	-21.63	120869	24	366	286
miR5799	LOC_Os10g21170.1	157	-21.98	157	-21.98	120900	24	393	14
miR5799	LOC_Os10g25040.1	177	-24.82	177	-24.82	121289	24	3692	781
miR5799	LOC_Os10g25040.2	177	-24.82	177	-24.82	121290	24	2238	781
miR5799	LOC_Os10g26960.1	164	-24.47	164	-24.47	121486	24	1629	750
miR5799	LOC_Os10g32160.1	149	-20.32	149	-20.32	121975	24	1976	1186
miR5799	LOC_Os10g34340.1	162	-22.24	162	-22.24	122206	24	2103	1407
miR5799	LOC_Os10g34500.1	141	-20.45	141	-20.45	122222	24	891	783
miR5799	LOC_Os10g35370.1	161	-23.62	161	-23.62	122325	24	1765	161
miR5799	LOC_Os10g35370.2	161	-23.62	161	-23.62	122326	24	2951	149
miR5799	LOC_Os10g39936.1	159	-22.4	159	-22.4	122830	24	687	114
miR5799	LOC_Os11g01470.1	149	-20.71	149	-20.71	123272	24	5073	304
miR5799	LOC_Os11g02670.1	152	-20.49	152	-20.49	123420	24	3358	2110
miR5799	LOC_Os11g04560.1	147	-23.55	147	-23.55	123640	24	1100	550
miR5799	LOC_Os11g05320.1	155	-20.61	155	-20.61	123731	24	1781	986
miR5799	LOC_Os11g05850.2	160	-20.05	160	-20.05	123790	24	5438	3858
miR5799	LOC_Os11g06580.1	151	-20.1	151	-20.1	123881	24	1572	895
miR5799	LOC_Os11g06960.1	166	-22.52	166	-22.52	123928	24	4715	4346
miR5799	LOC_Os11g08314.1	158	-21.28	158	-21.28	124092	24	3577	1878
miR5799	LOC_Os11g09260.1	190	-30.49	190	-30.49	124188	24	1887	834
miR5799	LOC_Os11g14610.1	158	-20.13	158	-20.13	124704	24	5544	2096
miR5799	LOC_Os11g17970.1	150	-20.64	150	-20.64	125005	24	2197	1491

miR5799	LOC_Os11g22000.1	155	-20.03	155	-20.03	125305	24	1923	12
miR5799	LOC_Os11g25510.1	183	-21.93	183	-21.93	125612	24	1526	1472
miR5799	LOC_Os11g32280.1	171	-21.85	171	-21.85	126286	24	1209	143
miR5799	LOC_Os11g34020.1	147	-21.42	147	-21.42	126425	24	1452	562
miR5799	LOC_Os11g34460.2	176	-23.14	176	-23.14	126482	24	3837	2251
miR5799	LOC_Os11g37540.1	182	-27.45	182	-27.45	126803	24	4071	3837
miR5799	LOC_Os11g42370.1	145	-20.21	145	-20.21	127328	24	1584	677
miR5799	LOC_Os11g43390.1	143	-20.71	143	-20.71	127432	24	2800	412
miR5799	LOC_Os11g45510.1	158	-20.26	158	-20.26	127658	24	1971	464
miR5799	LOC_Os11g48000.1	169	-23.23	169	-23.23	127896	24	1647	1062
miR5799	LOC_Os11g48070.1	151	-21.11	151	-21.11	127904	24	1440	1075
miR5799	LOC_Os12g01680.1	180	-23.45	180	-23.45	127994	24	834	520
miR5799	LOC_Os12g03050.1	163	-21.48	163	-21.48	128169	24	1632	1512
miR5799	LOC_Os12g03680.1	163	-24.37	163	-24.37	128239	24	2499	2156
miR5799	LOC_Os12g04360.1	147	-23.55	147	-23.55	128328	24	799	549
miR5799	LOC_Os12g04990.4	169	-22.36	169	-22.36	128404	24	2208	484
miR5799	LOC_Os12g09770.1	170	-24.5	170	-24.5	128929	24	1359	1010
miR5799	LOC_Os12g09990.1	152	-21.38	152	-21.38	128948	24	3864	2359
miR5799	LOC_Os12g12400.1	162	-24.47	162	-24.47	129199	24	1581	672
miR5799	LOC_Os12g16350.13	184	-25.53	184	-25.53	129603	24	4262	3101
miR5799	LOC_Os12g22110.1	141	-21	141	-21	130104	24	1719	1589
miR5799	LOC_Os12g23374.1	162	-21.79	162	-21.79	130228	24	2457	1774
miR5799	LOC_Os12g25980.1	166	-21.48	166	-21.48	130476	24	4626	1439
miR5799	LOC_Os12g28750.1	149	-20.35	149	-20.35	130750	24	1050	463
miR5799	LOC_Os12g31040.1	143	-21.36	143	-21.36	130980	24	3768	264
miR5799	LOC_Os12g33560.1	159	-20.16	159	-20.16	131235	24	1776	178
miR5799	LOC_Os12g40360.1	155	-26.2	155	-26.2	132009	24	1941	1328
miR5799	LOC_Os12g41620.1	162	-20.87	162	-20.87	132151	24	2992	444
miR5799	LOC_Os12g42230.1	156	-21.69	156	-21.69	132240	24	1697	1369
miR5799	ChrUn.fgenesh.mRNA.90	156	-21.02	156	-21.02	132581	24	1038	560
miR5337a	LOC_Os01g63820.1	176	-20.47	176	-20.47	139422	21	3646	1634
miR5337a	LOC_Os01g63820.2	176	-20.47	176	-20.47	139423	21	3470	1639

miR5337a	LOC_Os02g16140.1	161	-20.26	161	-20.26	142482	21	1092	717
miR5337a	LOC_Os02g53860.1	191	-29.1	191	-29.1	146714	21	2841	105
miR5337a	LOC_Os04g36058.1	161	-21.82	161	-21.82	157821	21	1833	768
miR5337a	LOC_Os04g36058.2	161	-21.82	161	-21.82	157822	21	1828	763
miR5337a	LOC_Os04g40910.1	175	-23.09	175	-23.09	158287	21	2810	1872
miR5337a	LOC_Os04g40910.2	175	-23.09	175	-23.09	158288	21	2711	1773
miR5337a	LOC_Os04g41000.1	166	-20.89	166	-20.89	158302	21	3679	966
miR5337a	LOC_Os04g57310.1	160	-20.63	160	-20.63	160253	21	1494	790
miR5337a	LOC_Os04g57310.2	160	-20.63	160	-20.63	160254	21	1490	786
miR5337a	LOC_Os04g57320.1	160	-20.63	160	-20.63	160255	21	1685	76
miR5337a	LOC_Os04g57320.2	160	-20.63	160	-20.63	160256	21	1871	76
miR5337a	LOC_Os05g46740.1	174	-20.2	174	-20.2	165416	21	3798	427
miR5337a	LOC_Os05g46740.2	174	-20.2	174	-20.2	165417	21	3801	424
miR5337a	LOC_Os05g50980.1	179	-23.16	179	-23.16	165897	21	1836	1765
miR5337a	LOC_Os06g03610.1	170	-20.55	170	-20.55	166334	21	3048	373
miR5337a	LOC_Os06g20770.1	161	-20.91	161	-20.91	168212	21	1273	566
miR5337a	LOC_Os06g28080.1	167	-21.02	167	-21.02	168875	21	1728	1084
miR5337a	LOC_Os07g33480.2	166	-20.84	166	-20.84	174804	21	2307	1173
miR5337a	LOC_Os09g01780.1	161	-20.26	161	-20.26	181651	21	1947	666
miR5337a	LOC_Os10g05750.1	169	-20.27	169	-20.27	185972	21	1289	143
miR5337a	LOC_Os10g39620.1	173	-23.05	173	-23.05	189120	21	2453	867
miR5337a	LOC_Os10g39620.2	173	-23.05	173	-23.05	189121	21	2536	950
miR5337a	LOC_Os10g39620.3	173	-23.05	173	-23.05	189122	21	2640	1054
miR5337a	LOC_Os10g39620.4	173	-23.05	173	-23.05	189123	21	2608	950
miR5337a	LOC_Os10g39620.5	173	-23.05	173	-23.05	189124	21	2682	1024
miR5337a	LOC_Os10g39620.6	173	-23.05	173	-23.05	189125	21	2712	1054
miR5337a	LOC_Os10g39620.7	173	-23.05	173	-23.05	189126	21	2311	653
miR5337a	LOC_Os11g07060.1	173	-21.2	173	-21.2	190281	21	3785	564
miR5337a	LOC_Os11g07060.2	173	-21.2	173	-21.2	190282	21	3699	478
miR5337a	LOC_Os11g07060.3	173	-21.2	173	-21.2	190283	21	3815	594
miR5337a	LOC_Os11g07060.4	173	-21.2	173	-21.2	190284	21	3789	568
miR5337a	LOC_Os11g07060.5	173	-21.2	173	-21.2	190285	21	3703	564

miR5337a	LOC_Os11g36830.1	169	-23.29	169	-23.29	193072	21	2121	598
miR5337a	LOC_Os11g46250.1	165	-22.05	165	-22.05	194077	21	4950	864
miR529b	LOC_Os01g01710.1	165	-20.92	165	-20.92	199113	21	1902	1877
miR529b	LOC_Os01g01710.2	165	-20.92	165	-20.92	199114	21	1801	1776
miR529b	LOC_Os01g02110.1	156	-20.4	156	-20.4	199164	21	1475	597
miR529b	LOC_Os01g02250.1	151	-23.97	151	-23.97	199180	21	4035	3541
miR529b	LOC_Os01g02280.1	151	-23.97	151	-23.97	199183	21	5367	4873
miR529b	LOC_Os01g02560.1	161	-24.07	161	-24.07	199215	21	2059	36
miR529b	LOC_Os01g02629.1	154	-20.29	154	-20.29	199221	21	1743	14
miR529b	LOC_Os01g02710.1	158	-23.54	158	-23.54	199228	21	603	17
miR529b	LOC_Os01g02920.1	339	-48.32	173	-25.65	199251	21	2288	1724 1826
miR529b	LOC_Os01g03420.1	171	-23.09	171	-23.09	199310	21	1254	590
miR529b	LOC_Os01g03520.1	168	-24.98	168	-24.98	199319	21	3960	6
miR529b	LOC_Os01g03914.1	161	-20.73	161	-20.73	199357	21	2241	1488
miR529b	LOC_Os01g03914.2	161	-20.73	161	-20.73	199358	21	2233	1488
miR529b	LOC_Os01g03914.3	161	-20.73	161	-20.73	199359	21	2314	1561
miR529b	LOC_Os01g03950.1	171	-27.51	171	-27.51	199362	21	3485	245
miR529b	LOC_Os01g04510.1	167	-22.32	167	-22.32	199423	21	567	479
miR529b	LOC_Os01g05130.1	165	-22.74	165	-22.74	199502	21	1027	1
miR529b	LOC_Os01g06300.1	156	-21.71	156	-21.71	199628	21	4458	2754
miR529b	LOC_Os01g06950.1	163	-20.91	163	-20.91	199699	21	465	54
miR529b	LOC_Os01g07180.1	164	-20.48	164	-20.48	199728	21	975	94
miR529b	LOC_Os01g07370.1	156	-23.48	156	-23.48	199755	21	2267	935
miR529b	LOC_Os01g07370.2	156	-23.48	156	-23.48	199756	21	2288	935
miR529b	LOC_Os01g08500.1	151	-20.15	151	-20.15	199884	21	1287	948
miR529b	LOC_Os01g08560.1	160	-21.48	160	-21.48	199890	21	3188	2488
miR529b	LOC_Os01g08560.2	160	-21.48	160	-21.48	199891	21	3184	2484
miR529b	LOC_Os01g09190.1	156	-23.21	156	-23.21	199967	21	946	644
miR529b	LOC_Os01g09640.1	159	-22.5	159	-22.5	200022	21	1769	894
miR529b	LOC_Os01g09670.1	165	-20.86	165	-20.86	200026	21	1520	56
miR529b	LOC_Os01g09760.1	152	-20.46	152	-20.46	200037	21	3314	3094
miR529b	LOC_Os01g10150.1	150	-23.22	150	-23.22	200079	21	2466	1122

miR529b	LOC_Os01g10150.2	150	-23.22	150	-23.22	200080	21	2462	1118
miR529b	LOC_Os01g10195.1	168	-21.32	168	-21.32	200084	21	1238	304
miR529b	LOC_Os01g10450.1	166	-26	166	-26	200115	21	2591	2549
miR529b	LOC_Os01g10450.2	166	-26	166	-26	200116	21	2579	2531
miR529b	LOC_Os01g10504.1	158	-21.89	158	-21.89	200121	21	1187	1053
miR529b	LOC_Os01g10504.2	158	-21.89	158	-21.89	200122	21	1434	1300
miR529b	LOC_Os01g10504.3	158	-21.89	158	-21.89	200123	21	1188	1054
miR529b	LOC_Os01g11820.1	157	-21.55	157	-21.55	200252	21	1023	1
miR529b	LOC_Os01g12150.1	156	-20.23	156	-20.23	200287	21	432	1
miR529b	LOC_Os01g12740.1	166	-21.66	166	-21.66	200363	21	2005	28
miR529b	LOC_Os01g14010.1	162	-23.48	162	-23.48	200515	21	2115	1039
miR529b	LOC_Os01g15200.1	161	-20.68	161	-20.68	200642	21	381	205
miR529b	LOC_Os01g15290.1	159	-21.97	159	-21.97	200651	21	754	273
miR529b	LOC_Os01g15290.2	159	-21.97	159	-21.97	200652	21	770	273
miR529b	LOC_Os01g15330.1	164	-20.48	164	-20.48	200657	21	369	30
miR529b	LOC_Os01g15790.4	162	-20.06	162	-20.06	200713	21	1658	384
miR529b	LOC_Os01g15900.1	164	-22.31	164	-22.31	200722	21	2228	1593
miR529b	LOC_Os01g16160.1	149	-21.86	149	-21.86	200755	21	4917	4229
miR529b	LOC_Os01g16190.1	175	-26.21	175	-26.21	200765	21	1339	1259
miR529b	LOC_Os01g16414.1	156	-22.08	156	-22.08	200793	21	2283	1964
miR529b	LOC_Os01g16414.2	156	-22.08	156	-22.08	200794	21	2044	1685
miR529b	LOC_Os01g16590.1	161	-20.91	161	-20.91	200813	21	408	261
miR529b	LOC_Os01g16650.1	168	-24.98	168	-24.98	200820	21	888	14
miR529b	LOC_Os01g16740.1	165	-22.78	165	-22.78	200827	21	3639	123
miR529b	LOC_Os01g17070.1	177	-28.92	177	-28.92	200869	21	1873	1762
miR529b	LOC_Os01g17070.2	177	-28.92	177	-28.92	200870	21	2164	2067
miR529b	LOC_Os01g17120.1	157	-21.69	157	-21.69	200875	21	3366	387
miR529b	LOC_Os01g17320.1	168	-23.18	168	-23.18	200891	21	2265	101
miR529b	LOC_Os01g18000.1	153	-20.93	153	-20.93	200913	21	4224	4139
miR529b	LOC_Os01g18230.1	161	-20.17	161	-20.17	200936	21	669	23
miR529b	LOC_Os01g18280.1	157	-22.95	157	-22.95	200941	21	1811	5
miR529b	LOC_Os01g18710.1	161	-23.47	161	-23.47	200982	21	570	336

miR529b	LOC_Os01g19410.1	168	-24.98	168	-24.98	201063	21	1650	1262
miR529b	LOC_Os01g19410.2	168	-24.98	168	-24.98	201064	21	1488	1104
miR529b	LOC_Os01g19410.3	168	-24.98	168	-24.98	201065	21	1468	1080
miR529b	LOC_Os01g20770.1	160	-20.69	160	-20.69	201157	21	678	129
miR529b	LOC_Os01g21100.1	156	-21.45	156	-21.45	201197	21	615	449
miR529b	LOC_Os01g21190.1	156	-21.71	156	-21.71	201210	21	3489	2502
miR529b	LOC_Os01g22954.1	167	-20.72	167	-20.72	201379	21	1984	296
miR529b	LOC_Os01g23100.1	156	-20.3	156	-20.3	201393	21	981	443
miR529b	LOC_Os01g23560.1	169	-23.03	169	-23.03	201434	21	1233	1
miR529b	LOC_Os01g23730.1	165	-21.46	165	-21.46	201457	21	804	57
miR529b	LOC_Os01g24130.1	169	-23.84	169	-23.84	201499	21	1377	950
miR529b	LOC_Os01g24370.1	154	-20.29	154	-20.29	201521	21	441	14
miR529b	LOC_Os01g24470.1	173	-27.1	173	-27.1	201531	21	3416	476
miR529b	LOC_Os01g24470.2	173	-27.1	173	-27.1	201532	21	3378	375
miR529b	LOC_Os01g25210.1	156	-22.58	156	-22.58	201617	21	585	535
miR529b	LOC_Os01g26130.3	152	-20.69	152	-20.69	201707	21	1511	379
miR529b	LOC_Os01g26130.5	152	-20.69	152	-20.69	201709	21	1663	531
miR529b	LOC_Os01g26390.1	160	-23.91	160	-23.91	201730	21	2602	24
miR529b	LOC_Os01g27040.1	167	-22.66	167	-22.66	201754	21	3750	1035
miR529b	LOC_Os01g27280.1	166	-23.94	166	-23.94	201778	21	2799	2650
miR529b	LOC_Os01g29020.1	149	-20.23	149	-20.23	201957	21	1164	157
miR529b	LOC_Os01g29270.1	161	-22	161	-22	201984	21	4491	3138
miR529b	LOC_Os01g29409.1	173	-22.24	173	-22.24	201997	21	2567	2436
miR529b	LOC_Os01g29530.1	161	-20.3	161	-20.3	202004	21	3387	2833
miR529b	LOC_Os01g29660.1	157	-21.69	157	-21.69	202016	21	1230	519
miR529b	LOC_Os01g31450.1	170	-23.4	170	-23.4	202100	21	2831	18
miR529b	LOC_Os01g32220.1	157	-21.69	157	-21.69	202184	21	4662	531
miR529b	LOC_Os01g32660.1	157	-20.52	157	-20.52	202224	21	1761	19
miR529b	LOC_Os01g32660.2	157	-20.52	157	-20.52	202225	21	1758	19
miR529b	LOC_Os01g32660.3	157	-20.52	157	-20.52	202226	21	1364	19
miR529b	LOC_Os01g32660.4	157	-20.52	157	-20.52	202227	21	1825	19
miR529b	LOC_Os01g32660.5	157	-20.52	157	-20.52	202228	21	2272	19

miR529b	LOC_Os01g32830.1	162	-23.43	162	-23.43	202245	21	2718	2628
miR529b	LOC_Os01g32830.2	162	-23.43	162	-23.43	202246	21	947	857
miR529b	LOC_Os01g32864.1	149	-20.68	149	-20.68	202250	21	833	617
miR529b	LOC_Os01g33040.1	154	-20.61	154	-20.61	202268	21	3824	2236
miR529b	LOC_Os01g33960.1	162	-20.32	162	-20.32	202355	21	2142	1946
miR529b	LOC_Os01g34420.1	168	-23.18	168	-23.18	202395	21	803	220
miR529b	LOC_Os01g34830.1	156	-20.31	156	-20.31	202449	21	1125	10
miR529b	LOC_Os01g35520.1	154	-20.29	154	-20.29	202518	21	375	14
miR529b	LOC_Os01g35760.1	156	-20.23	156	-20.23	202547	21	477	369
miR529b	LOC_Os01g36710.1	151	-20.15	151	-20.15	202642	21	1665	948
miR529b	LOC_Os01g38030.1	159	-20.37	159	-20.37	202800	21	876	1
miR529b	LOC_Os01g38530.1	158	-22.33	158	-22.33	202850	21	2832	1238
miR529b	LOC_Os01g38610.1	175	-24.5	175	-24.5	202860	21	2420	2240
miR529b	LOC_Os01g39050.1	165	-20.26	165	-20.26	202907	21	5391	570
miR529b	LOC_Os01g39110.1	171	-27.01	171	-27.01	202913	21	2783	305
miR529b	LOC_Os01g39990.1	163	-20.17	163	-20.17	203009	21	1658	1124
miR529b	LOC_Os01g40200.1	159	-20.26	159	-20.26	203029	21	2897	687
miR529b	LOC_Os01g40200.2	159	-20.26	159	-20.26	203030	21	2988	778
miR529b	LOC_Os01g40310.1	158	-20.41	158	-20.41	203041	21	885	769
miR529b	LOC_Os01g41950.1	163	-21.76	163	-21.76	203220	21	3201	304
miR529b	LOC_Os01g41960.1	156	-21.54	156	-21.54	203221	21	6519	583
miR529b	LOC_Os01g43330.1	166	-23.2	166	-23.2	203384	21	3403	2515
miR529b	LOC_Os01g43520.1	160	-22.48	160	-22.48	203409	21	3065	2083
miR529b	LOC_Os01g43520.2	160	-22.48	160	-22.48	203410	21	3214	2083
miR529b	LOC_Os01g44940.1	169	-25.54	169	-25.54	203515	21	672	408
miR529b	LOC_Os01g45220.1	161	-25	161	-25	203545	21	2436	2411
miR529b	LOC_Os01g45439.2	165	-22.56	165	-22.56	203567	21	1446	1194
miR529b	LOC_Os01g46100.1	169	-23.84	169	-23.84	203636	21	3162	1973
miR529b	LOC_Os01g46810.1	323	-41	162	-20.95	203718	21	4832	1842 1721
miR529b	LOC_Os01g47050.1	162	-22.17	162	-22.17	203745	21	1982	718
miR529b	LOC_Os01g48440.1	170	-24.1	170	-24.1	203920	21	2322	859
miR529b	LOC_Os01g48440.2	170	-24.1	170	-24.1	203921	21	2469	1006

miR529b	LOC_Os01g48440.3	170	-24.1	170	-24.1	203922	21	2750	1287
miR529b	LOC_Os01g48530.1	333	-49.25	168	-26.07	203931	21	971	25 651
miR529b	LOC_Os01g50100.1	162	-20.82	162	-20.82	204112	21	4079	2630
miR529b	LOC_Os01g50500.1	157	-21.69	157	-21.69	204149	21	4575	531
miR529b	LOC_Os01g51280.1	335	-44.99	168	-23.18	204243	21	2002	24 57
miR529b	LOC_Os01g51280.2	335	-44.99	168	-23.18	204244	21	2086	24 57
miR529b	LOC_Os01g52230.1	165	-24.89	165	-24.89	204359	21	1217	1150
miR529b	LOC_Os01g52640.3	165	-24.25	165	-24.25	204406	21	4050	1461
miR529b	LOC_Os01g52710.1	160	-20.59	160	-20.59	204413	21	2494	1450
miR529b	LOC_Os01g53960.1	157	-21.69	157	-21.69	204554	21	4704	531
miR529b	LOC_Os01g54100.1	166	-21.91	166	-21.91	204572	21	2389	1099
miR529b	LOC_Os01g54100.2	166	-21.91	166	-21.91	204573	21	2772	1100
miR529b	LOC_Os01g54480.1	154	-20.07	154	-20.07	204612	21	2284	1107
miR529b	LOC_Os01g54480.2	154	-20.07	154	-20.07	204613	21	2331	1234
miR529b	LOC_Os01g54480.3	154	-20.07	154	-20.07	204614	21	2361	1264
miR529b	LOC_Os01g55470.1	169	-23.84	169	-23.84	204737	21	2799	1808
miR529b	LOC_Os01g55490.1	163	-20.14	163	-20.14	204739	21	1716	127
miR529b	LOC_Os01g55490.2	163	-20.14	163	-20.14	204740	21	1713	127
miR529b	LOC_Os01g55490.3	163	-20.14	163	-20.14	204741	21	1667	127
miR529b	LOC_Os01g55490.4	163	-20.14	163	-20.14	204742	21	1648	127
miR529b	LOC_Os01g55530.1	160	-20.64	160	-20.64	204749	21	3675	2277
miR529b	LOC_Os01g55549.1	155	-20.81	155	-20.81	204752	21	2451	374
miR529b	LOC_Os01g56300.1	171	-28.36	171	-28.36	204849	21	3646	395
miR529b	LOC_Os01g57050.1	162	-20.68	162	-20.68	204947	21	1011	550
miR529b	LOC_Os01g57050.2	162	-20.68	162	-20.68	204948	21	898	550
miR529b	LOC_Os01g57120.1	156	-21.71	156	-21.71	204955	21	2661	2235
miR529b	LOC_Os01g57550.1	168	-23.18	168	-23.18	205005	21	1804	147
miR529b	LOC_Os01g57550.2	168	-23.18	168	-23.18	205006	21	1940	185
miR529b	LOC_Os01g57735.1	160	-20.17	160	-20.17	205031	21	1601	213
miR529b	LOC_Os01g57854.1	156	-21.65	156	-21.65	205042	21	1869	71
miR529b	LOC_Os01g58950.1	161	-21.52	161	-21.52	205172	21	1494	15
miR529b	LOC_Os01g59265.1	169	-24.35	169	-24.35	205207	21	625	470

miR529b	LOC_Os01g60040.1	153	-20.04	153	-20.04	205298	21	6605	2163
miR529b	LOC_Os01g60040.2	153	-20.04	153	-20.04	205299	21	6623	2163
miR529b	LOC_Os01g61590.1	169	-23.87	169	-23.87	205470	21	2139	57
miR529b	LOC_Os01g61680.1	165	-21.43	165	-21.43	205483	21	1203	673
miR529b	LOC_Os01g61860.1	165	-24.97	165	-24.97	205514	21	3308	419
miR529b	LOC_Os01g61860.2	165	-24.97	165	-24.97	205515	21	3177	419
miR529b	LOC_Os01g61880.1	159	-21.65	159	-21.65	205517	21	2900	1773
miR529b	LOC_Os01g61880.2	159	-21.65	159	-21.65	205518	21	2525	1398
miR529b	LOC_Os01g61890.2	159	-21.65	159	-21.65	205520	21	4288	1819
miR529b	LOC_Os01g61890.3	159	-21.65	159	-21.65	205521	21	5479	1819
miR529b	LOC_Os01g61910.1	164	-23.85	164	-23.85	205524	21	3151	2329
miR529b	LOC_Os01g62350.1	159	-20.61	159	-20.61	205580	21	620	265
miR529b	LOC_Os01g64490.1	166	-21.72	166	-21.72	205850	21	3544	1897
miR529b	LOC_Os01g64650.1	160	-20.11	160	-20.11	205870	21	1272	471
miR529b	LOC_Os01g64650.2	160	-20.11	160	-20.11	205871	21	1400	471
miR529b	LOC_Os01g64810.1	162	-21.73	162	-21.73	205901	21	2294	52
miR529b	LOC_Os01g65094.1	165	-21.62	165	-21.62	205936	21	1056	893
miR529b	LOC_Os01g65550.1	161	-20.09	161	-20.09	205988	21	2219	60
miR529b	LOC_Os01g65550.2	161	-20.09	161	-20.09	205989	21	2239	60
miR529b	LOC_Os01g65640.1	156	-20.67	156	-20.67	206000	21	1485	959
miR529b	LOC_Os01g65780.2	168	-23.7	168	-23.7	206018	21	2665	66
miR529b	LOC_Os01g66490.1	161	-20.91	161	-20.91	206099	21	2044	233
miR529b	LOC_Os01g66580.1	150	-20.58	150	-20.58	206107	21	861	681
miR529b	LOC_Os01g67370.1	167	-20.02	167	-20.02	206206	21	2199	2038
miR529b	LOC_Os01g67370.2	167	-20.02	167	-20.02	206207	21	2102	1941
miR529b	LOC_Os01g67370.3	167	-20.02	167	-20.02	206208	21	2318	2157
miR529b	LOC_Os01g67370.4	167	-20.02	167	-20.02	206209	21	1075	914
miR529b	LOC_Os01g67370.5	167	-20.02	167	-20.02	206210	21	1339	1178
miR529b	LOC_Os01g67370.6	167	-20.02	167	-20.02	206211	21	1576	1415
miR529b	LOC_Os01g67810.1	162	-20.02	162	-20.02	206262	21	2022	42
miR529b	LOC_Os01g67990.1	156	-22.71	156	-22.71	206281	21	2634	1026
miR529b	LOC_Os01g68010.1	157	-22.07	157	-22.07	206283	21	1311	1027

miR529b	LOC_Os01g68010.2	157	-22.07	157	-22.07	206284	21	1318	1034
miR529b	LOC_Os01g68010.3	157	-22.07	157	-22.07	206285	21	1317	1033
miR529b	LOC_Os01g68010.4	157	-22.07	157	-22.07	206286	21	855	571
miR529b	LOC_Os01g68010.5	157	-22.07	157	-22.07	206287	21	1329	571
miR529b	LOC_Os01g68020.1	162	-20.16	162	-20.16	206288	21	1482	37
miR529b	LOC_Os01g68120.1	166	-22.14	166	-22.14	206298	21	5199	5100
miR529b	LOC_Os01g68324.3	161	-25.27	161	-25.27	206323	21	2732	2254
miR529b	LOC_Os01g68340.1	166	-21.94	166	-21.94	206325	21	1623	123
miR529b	LOC_Os01g68524.1	171	-21.58	171	-21.58	206348	21	3327	1087
miR529b	LOC_Os01g68700.1	165	-22.49	165	-22.49	206367	21	2432	961
miR529b	LOC_Os01g68700.2	165	-22.49	165	-22.49	206368	21	2428	957
miR529b	LOC_Os01g69100.1	168	-21.29	168	-21.29	206424	21	1272	86
miR529b	LOC_Os01g69130.1	170	-23.83	170	-23.83	206430	21	3206	1336
miR529b	LOC_Os01g69130.2	170	-23.83	170	-23.83	206431	21	3098	1228
miR529b	LOC_Os01g69830.1	343	-56.66	186	-35.22	206454	21	1983	1148 1
miR529b	LOC_Os01g69950.1	165	-21.89	165	-21.89	206470	21	1968	32
miR529b	LOC_Os01g70360.1	165	-27.88	165	-27.88	206523	21	1925	1818
miR529b	LOC_Os01g70710.1	157	-21.22	157	-21.22	206566	21	1669	1552
miR529b	LOC_Os01g70710.2	157	-21.22	157	-21.22	206567	21	1917	1800
miR529b	LOC_Os01g70890.1	168	-22.71	168	-22.71	206589	21	1425	1334
miR529b	LOC_Os01g70960.1	154	-21.84	154	-21.84	206601	21	1497	270
miR529b	LOC_Os01g70960.2	154	-21.84	154	-21.84	206602	21	2661	277
miR529b	LOC_Os01g70980.1	168	-22.71	168	-22.71	206604	21	1041	1001
miR529b	LOC_Os01g71000.1	157	-20.36	157	-20.36	206606	21	2574	25
miR529b	LOC_Os01g71050.1	161	-21.58	161	-21.58	206611	21	2111	801
miR529b	LOC_Os01g71310.1	162	-23.75	162	-23.75	206641	21	2388	869
miR529b	LOC_Os01g71390.1	168	-23.44	168	-23.44	206649	21	327	165
miR529b	LOC_Os01g71624.1	156	-21.58	156	-21.58	206674	21	828	346
miR529b	LOC_Os01g71624.2	156	-21.58	156	-21.58	206675	21	1046	350
miR529b	LOC_Os01g71624.3	156	-21.58	156	-21.58	206676	21	756	350
miR529b	LOC_Os01g71624.4	156	-21.58	156	-21.58	206677	21	782	350
miR529b	LOC_Os01g71624.5	156	-21.58	156	-21.58	206678	21	1829	350

miR529b	LOC_Os01g71970.1	160	-23.29	160	-23.29	206714	21	1544	6
miR529b	LOC_Os01g72210.1	162	-25.65	162	-25.65	206740	21	2907	2734
miR529b	LOC_Os01g72210.2	162	-25.65	162	-25.65	206741	21	3068	2741
miR529b	LOC_Os01g72220.1	162	-25.65	162	-25.65	206742	21	2596	281
miR529b	LOC_Os01g72370.1	158	-20.41	158	-20.41	206758	21	4096	2055
miR529b	LOC_Os01g72860.1	156	-20.7	156	-20.7	206817	21	2808	1937
miR529b	LOC_Os01g73710.1	155	-20.75	155	-20.75	206917	21	951	802
miR529b	LOC_Os01g73790.1	162	-22.58	162	-22.58	206925	21	1920	1896
miR529b	LOC_Os01g74510.1	157	-22.38	157	-22.38	207013	21	3404	2894
miR529b	LOC_Os02g01220.1	157	-23.38	157	-23.38	207070	21	1369	21
miR529b	LOC_Os02g01580.1	157	-22.3	157	-22.3	207113	21	2502	2477
miR529b	LOC_Os02g01590.1	160	-22.56	160	-22.56	207114	21	2763	2080
miR529b	LOC_Os02g01590.2	160	-22.56	160	-22.56	207115	21	2252	1569
miR529b	LOC_Os02g01870.1	157	-20.08	157	-20.08	207148	21	1537	639
miR529b	LOC_Os02g01900.1	157	-20.08	157	-20.08	207151	21	1308	486
miR529b	LOC_Os02g01960.1	152	-21.03	152	-21.03	207157	21	2190	513
miR529b	LOC_Os02g02290.1	162	-21.81	162	-21.81	207194	21	7021	2146
miR529b	LOC_Os02g02410.1	153	-22.03	153	-22.03	207209	21	2476	2382
miR529b	LOC_Os02g02500.1	165	-22.78	165	-22.78	207220	21	1873	35
miR529b	LOC_Os02g02610.1	156	-21.71	156	-21.71	207234	21	3378	1674
miR529b	LOC_Os02g03470.1	156	-20.37	156	-20.37	207353	21	1425	42
miR529b	LOC_Os02g03670.1	157	-22.43	157	-22.43	207379	21	2051	579
miR529b	LOC_Os02g03670.3	157	-22.43	157	-22.43	207380	21	2831	579
miR529b	LOC_Os02g03670.5	157	-22.43	157	-22.43	207381	21	2358	579
miR529b	LOC_Os02g03670.6	157	-22.43	157	-22.43	207382	21	2259	579
miR529b	LOC_Os02g03670.7	157	-22.43	157	-22.43	207383	21	2190	579
miR529b	LOC_Os02g04020.1	165	-24.26	165	-24.26	207428	21	2478	193
miR529b	LOC_Os02g04480.1	161	-20.78	161	-20.78	207495	21	3120	1127
miR529b	LOC_Os02g04640.1	156	-22.09	156	-22.09	207513	21	2002	1889
miR529b	LOC_Os02g04680.1	327	-49.96	171	-29.1	207518	21	2517	1960 984
miR529b	LOC_Os02g04680.2	327	-49.96	171	-29.1	207519	21	2514	1957 984
miR529b	LOC_Os02g04910.1	156	-21.71	156	-21.71	207551	21	5157	3033

miR529b	LOC_Os02g04970.1	163	-20.67	163	-20.67	207559	21	4893	1666
miR529b	LOC_Os02g05570.1	169	-23.84	169	-23.84	207637	21	3066	1877
miR529b	LOC_Os02g05880.1	156	-20.55	156	-20.55	207677	21	6900	2699
miR529b	LOC_Os02g06000.1	156	-21.71	156	-21.71	207689	21	2493	921
miR529b	LOC_Os02g06830.1	168	-22.71	168	-22.71	207788	21	3642	3602
miR529b	LOC_Os02g06830.2	168	-22.71	168	-22.71	207789	21	3528	3488
miR529b	LOC_Os02g07170.1	154	-21.04	154	-21.04	207835	21	1022	865
miR529b	LOC_Os02g07780.1	177	-33.41	177	-33.41	207919	21	1668	983
miR529b	LOC_Os02g07780.2	177	-33.41	177	-33.41	207920	21	2902	2217
miR529b	LOC_Os02g07820.1	168	-24.98	168	-24.98	207924	21	1556	207
miR529b	LOC_Os02g08130.1	160	-20.18	160	-20.18	207965	21	2798	2503
miR529b	LOC_Os02g08130.2	160	-20.18	160	-20.18	207966	21	4810	2877
miR529b	LOC_Os02g08130.3	160	-20.18	160	-20.18	207967	21	4638	2860
miR529b	LOC_Os02g08140.1	160	-20.18	160	-20.18	207968	21	2131	853
miR529b	LOC_Os02g08440.1	158	-20.15	158	-20.15	208001	21	1876	155
miR529b	LOC_Os02g08440.2	158	-20.15	158	-20.15	208002	21	1833	3
miR529b	LOC_Os02g08440.3	158	-20.15	158	-20.15	208003	21	1987	155
miR529b	LOC_Os02g09060.1	162	-20.77	162	-20.77	208019	21	1778	1694
miR529b	LOC_Os02g09060.2	162	-20.77	162	-20.77	208020	21	2162	2078
miR529b	LOC_Os02g10200.1	173	-23.5	173	-23.5	208154	21	1122	1092
miR529b	LOC_Os02g10200.2	173	-23.5	173	-23.5	208155	21	1128	1098
miR529b	LOC_Os02g10640.1	168	-22.91	168	-22.91	208210	21	1732	1638
miR529b	LOC_Os02g10750.1	164	-21.12	164	-21.12	208223	21	726	63
miR529b	LOC_Os02g10770.2	153	-20.12	153	-20.12	208225	21	2132	1934
miR529b	LOC_Os02g11990.1	159	-21.3	159	-21.3	208324	21	897	693
miR529b	LOC_Os02g12660.1	165	-23.03	165	-23.03	208404	21	2993	1454
miR529b	LOC_Os02g12670.1	174	-25.75	174	-25.75	208405	21	2975	2870
miR529b	LOC_Os02g13020.1	167	-25.85	167	-25.85	208459	21	1897	304
miR529b	LOC_Os02g13100.1	155	-21.01	155	-21.01	208466	21	1919	33
miR529b	LOC_Os02g13520.1	153	-20.09	153	-20.09	208514	21	1533	660
miR529b	LOC_Os02g13560.2	166	-22.59	166	-22.59	208520	21	3159	273
miR529b	LOC_Os02g13560.3	166	-22.59	166	-22.59	208521	21	3025	139

miR529b	LOC_Os02g14130.1	160	-20.17	160	-20.17	208586	21	2112	2030
miR529b	LOC_Os02g15230.1	161	-21.97	161	-21.97	208709	21	1863	345
miR529b	LOC_Os02g15230.2	161	-21.97	161	-21.97	208710	21	3297	345
miR529b	LOC_Os02g15230.3	161	-21.97	161	-21.97	208711	21	3301	345
miR529b	LOC_Os02g15310.1	161	-20.12	161	-20.12	208719	21	1269	761
miR529b	LOC_Os02g15310.2	161	-20.12	161	-20.12	208720	21	2241	761
miR529b	LOC_Os02g15310.3	161	-20.12	161	-20.12	208721	21	1822	761
miR529b	LOC_Os02g15310.4	161	-20.12	161	-20.12	208722	21	2071	761
miR529b	LOC_Os02g15670.1	161	-25.21	161	-25.21	208760	21	387	53
miR529b	LOC_Os02g15820.1	163	-24.09	163	-24.09	208776	21	3137	2496
miR529b	LOC_Os02g15820.2	163	-24.09	163	-24.09	208777	21	3161	2614
miR529b	LOC_Os02g17040.1	174	-26.46	174	-26.46	208915	21	882	802
miR529b	LOC_Os02g17440.1	161	-20.76	161	-20.76	208957	21	1422	408
miR529b	LOC_Os02g17810.1	166	-21.94	166	-21.94	208996	21	1992	123
miR529b	LOC_Os02g18290.1	161	-22	161	-22	209056	21	4581	3405
miR529b	LOC_Os02g18730.1	162	-24.91	162	-24.91	209102	21	3120	3029
miR529b	LOC_Os02g18850.1	169	-24.94	169	-24.94	209117	21	2251	187
miR529b	LOC_Os02g19620.1	149	-21.86	149	-21.86	209196	21	4827	4034
miR529b	LOC_Os02g21430.1	167	-22.49	167	-22.49	209387	21	3347	61
miR529b	LOC_Os02g22140.1	157	-23.59	157	-23.59	209472	21	1516	376
miR529b	LOC_Os02g22190.1	149	-20.5	149	-20.5	209476	21	2052	378
miR529b	LOC_Os02g22520.1	161	-20.28	161	-20.28	209514	21	4488	528
miR529b	LOC_Os02g22650.1	165	-20.68	165	-20.68	209526	21	2754	35
miR529b	LOC_Os02g22650.2	165	-20.68	165	-20.68	209527	21	2751	35
miR529b	LOC_Os02g24090.1	170	-23.3	170	-23.3	209591	21	4725	4238
miR529b	LOC_Os02g24490.1	153	-20.34	153	-20.34	209630	21	1281	1251
miR529b	LOC_Os02g24586.1	153	-21.5	153	-21.5	209640	21	405	141
miR529b	LOC_Os02g25170.1	153	-20.34	153	-20.34	209705	21	6420	1581
miR529b	LOC_Os02g25230.1	177	-30.99	177	-30.99	209711	21	3840	594
miR529b	LOC_Os02g25760.1	164	-23.28	164	-23.28	209766	21	723	141
miR529b	LOC_Os02g26700.1	154	-20.3	154	-20.3	209872	21	1454	731
miR529b	LOC_Os02g26700.2	154	-20.3	154	-20.3	209873	21	1558	956

miR529b	LOC_Os02g26700.3	154	-20.3	154	-20.3	209874	21	1353	731
miR529b	LOC_Os02g26700.4	154	-20.3	154	-20.3	209875	21	1467	731
miR529b	LOC_Os02g26700.6	154	-20.3	154	-20.3	209876	21	1419	817
miR529b	LOC_Os02g26700.7	154	-20.3	154	-20.3	209877	21	1329	727
miR529b	LOC_Os02g27090.1	151	-22.25	151	-22.25	209925	21	960	753
miR529b	LOC_Os02g28010.1	157	-21.69	157	-21.69	210028	21	4554	324
miR529b	LOC_Os02g29464.1	169	-22.81	169	-22.81	210171	21	4549	221
miR529b	LOC_Os02g29550.1	160	-25.46	160	-25.46	210180	21	2702	392
miR529b	LOC_Os02g30530.1	167	-26.05	167	-26.05	210290	21	3497	786
miR529b	LOC_Os02g30580.1	157	-21.69	157	-21.69	210295	21	4542	531
miR529b	LOC_Os02g30590.1	162	-20.95	162	-20.95	210296	21	504	162
miR529b	LOC_Os02g30955.1	165	-24.08	165	-24.08	210340	21	762	96
miR529b	LOC_Os02g31150.1	157	-21.15	157	-21.15	210363	21	2673	1142
miR529b	LOC_Os02g31890.1	151	-20.95	151	-20.95	210391	21	1479	1349
miR529b	LOC_Os02g33220.1	164	-20.48	164	-20.48	210546	21	450	115
miR529b	LOC_Os02g33250.1	486	-68.25	162	-22.75	210549	21	411	11 34 65
miR529b	LOC_Os02g33335.1	168	-26.81	168	-26.81	210561	21	1078	192
miR529b	LOC_Os02g33450.1	159	-21.56	159	-21.56	210573	21	1707	392
miR529b	LOC_Os02g33450.2	159	-21.56	159	-21.56	210574	21	1707	392
miR529b	LOC_Os02g33670.1	165	-25.12	165	-25.12	210597	21	1442	328
miR529b	LOC_Os02g34650.1	164	-21.32	164	-21.32	210693	21	1820	1660
miR529b	LOC_Os02g34860.1	165	-23.03	165	-23.03	210714	21	1757	1
miR529b	LOC_Os02g35140.1	165	-21.81	165	-21.81	210749	21	3399	34
miR529b	LOC_Os02g36414.1	156	-20.27	156	-20.27	210898	21	2308	585
miR529b	LOC_Os02g38180.1	161	-24.01	161	-24.01	211119	21	1515	87
miR529b	LOC_Os02g38280.1	161	-25	161	-25	211129	21	2415	2390
miR529b	LOC_Os02g38420.1	152	-22.6	152	-22.6	211146	21	348	205
miR529b	LOC_Os02g38470.1	654	-87.13	174	-24.9	211151	21	3832	3389 3182 3224 3329
miR529b	LOC_Os02g38508.1	166	-26.23	166	-26.23	211159	21	1017	676
miR529b	LOC_Os02g38598.1	166	-26.23	166	-26.23	211169	21	1148	676
miR529b	LOC_Os02g39550.1	175	-28.99	175	-28.99	211290	21	3098	2021
miR529b	LOC_Os02g39550.2	175	-28.99	175	-28.99	211291	21	2982	2021

miR529b	LOC_Os02g39550.3	175	-28.99	175	-28.99	211292	21	3196	2119
miR529b	LOC_Os02g39630.1	170	-23.61	170	-23.61	211304	21	1041	4
miR529b	LOC_Os02g39630.2	170	-23.61	170	-23.61	211305	21	932	4
miR529b	LOC_Os02g39630.3	170	-23.61	170	-23.61	211306	21	1046	4
miR529b	LOC_Os02g39900.1	156	-21.71	156	-21.71	211342	21	3081	1527
miR529b	LOC_Os02g40040.1	159	-20.17	159	-20.17	211366	21	788	667
miR529b	LOC_Os02g40180.1	163	-20.12	163	-20.12	211383	21	2994	72
miR529b	LOC_Os02g40530.1	165	-24.77	165	-24.77	211426	21	1457	1370
miR529b	LOC_Os02g41720.1	156	-21.71	156	-21.71	211506	21	4029	3399
miR529b	LOC_Os02g42170.1	159	-20.22	159	-20.22	211559	21	1034	888
miR529b	LOC_Os02g42320.1	162	-23.41	162	-23.41	211580	21	1304	1160
miR529b	LOC_Os02g42320.2	162	-23.41	162	-23.41	211581	21	1195	1051
miR529b	LOC_Os02g42320.3	162	-23.41	162	-23.41	211582	21	1294	1150
miR529b	LOC_Os02g44102.1	171	-25.94	171	-25.94	211804	21	1689	575
miR529b	LOC_Os02g44111.1	158	-20.88	158	-20.88	211810	21	1658	697
miR529b	LOC_Os02g44111.2	158	-20.88	158	-20.88	211811	21	1294	669
miR529b	LOC_Os02g44550.1	166	-21.35	166	-21.35	211875	21	3882	1734
miR529b	LOC_Os02g45090.1	165	-22.01	165	-22.01	211945	21	1648	1601
miR529b	LOC_Os02g45090.2	165	-22.01	165	-22.01	211946	21	1538	1491
miR529b	LOC_Os02g45170.1	161	-20.3	161	-20.3	211958	21	2899	27
miR529b	LOC_Os02g45200.1	169	-24.99	169	-24.99	211960	21	2355	2239
miR529b	LOC_Os02g45410.1	168	-22.24	168	-22.24	211983	21	273	47
miR529b	LOC_Os02g45480.1	157	-21.55	157	-21.55	211989	21	2001	74
miR529b	LOC_Os02g45480.2	157	-21.55	157	-21.55	211990	21	3510	74
miR529b	LOC_Os02g45480.3	157	-21.55	157	-21.55	211991	21	3695	74
miR529b	LOC_Os02g45520.1	152	-22.44	152	-22.44	211999	21	2966	1380
miR529b	LOC_Os02g45520.2	152	-22.44	152	-22.44	212000	21	2487	1030
miR529b	LOC_Os02g46390.1	165	-21.55	165	-21.55	212099	21	690	46
miR529b	LOC_Os02g46640.1	153	-23.65	153	-23.65	212127	21	1075	833
miR529b	LOC_Os02g46940.1	163	-23.11	163	-23.11	212164	21	1259	144
miR529b	LOC_Os02g47010.1	174	-25.91	174	-25.91	212177	21	1739	589
miR529b	LOC_Os02g47020.1	149	-20.89	149	-20.89	212178	21	1824	1598

miR529b	LOC_Os02g47040.1	161	-20.16	161	-20.16	212180	21	567	35
miR529b	LOC_Os02g47180.1	158	-20.36	158	-20.36	212196	21	2655	198
miR529b	LOC_Os02g47180.2	158	-20.36	158	-20.36	212197	21	2597	198
miR529b	LOC_Os02g47430.1	165	-24.58	165	-24.58	212232	21	435	83
miR529b	LOC_Os02g47880.1	316	-43.09	158	-22.35	212292	21	2777	373 511
miR529b	LOC_Os02g47880.2	316	-43.09	158	-22.35	212293	21	2707	373 511
miR529b	LOC_Os02g48010.1	166	-23.45	166	-23.45	212308	21	4018	2371
miR529b	LOC_Os02g48150.1	157	-22.28	157	-22.28	212325	21	1159	1110
miR529b	LOC_Os02g48800.1	168	-24.08	168	-24.08	212412	21	805	1
miR529b	LOC_Os02g49480.1	153	-22.29	153	-22.29	212494	21	1678	762
miR529b	LOC_Os02g50840.1	165	-21.81	165	-21.81	212662	21	6170	33
miR529b	LOC_Os02g50840.2	165	-21.81	165	-21.81	212663	21	6076	33
miR529b	LOC_Os02g51390.1	168	-22.91	168	-22.91	212727	21	3752	452
miR529b	LOC_Os02g51390.2	168	-22.91	168	-22.91	212728	21	3374	452
miR529b	LOC_Os02g52510.1	152	-21.26	152	-21.26	212878	21	3465	1977
miR529b	LOC_Os02g53720.1	156	-20.18	156	-20.18	213032	21	1708	558
miR529b	LOC_Os02g53720.2	156	-20.18	156	-20.18	213033	21	1608	458
miR529b	LOC_Os02g53750.2	174	-31.89	174	-31.89	213038	21	3121	1324
miR529b	LOC_Os02g53770.1	161	-20.43	161	-20.43	213039	21	3611	28
miR529b	LOC_Os02g54110.1	154	-20.29	154	-20.29	213080	21	4192	1943
miR529b	LOC_Os02g54160.1	153	-20.11	153	-20.11	213091	21	1703	1613
miR529b	LOC_Os02g54160.2	153	-20.11	153	-20.11	213092	21	1778	1688
miR529b	LOC_Os02g54820.1	154	-20.77	154	-20.77	213161	21	3664	2863
miR529b	LOC_Os02g55000.1	146	-21.69	146	-21.69	213182	21	2017	680
miR529b	LOC_Os02g55130.1	162	-20.03	162	-20.03	213197	21	2807	250
miR529b	LOC_Os02g55130.2	162	-20.03	162	-20.03	213198	21	2663	106
miR529b	LOC_Os02g55130.3	162	-20.03	162	-20.03	213199	21	2908	250
miR529b	LOC_Os02g55130.5	162	-20.03	162	-20.03	213201	21	2912	250
miR529b	LOC_Os02g55770.4	168	-22.71	168	-22.71	213277	21	5708	5677
miR529b	LOC_Os02g55770.5	168	-22.71	168	-22.71	213278	21	2977	2946
miR529b	LOC_Os02g56100.1	165	-23.61	165	-23.61	213323	21	3008	32
miR529b	LOC_Os02g56530.1	163	-24.54	163	-24.54	213375	21	2308	1250

miR529b	LOC_Os02g56850.2	167	-24.5	167	-24.5	213411	21	2176	91
miR529b	LOC_Os02g56850.3	167	-24.5	167	-24.5	213412	21	2160	172
miR529b	LOC_Os02g57610.1	168	-22.53	168	-22.53	213533	21	2399	1
miR529b	LOC_Os02g57750.1	161	-20.56	161	-20.56	213554	21	2985	175
miR529b	LOC_Os02g57750.2	161	-20.56	161	-20.56	213555	21	3292	125
miR529b	LOC_Os03g01070.1	159	-21.03	159	-21.03	213704	21	2748	2554
miR529b	LOC_Os03g01230.1	165	-20.37	165	-20.37	213728	21	797	490
miR529b	LOC_Os03g01230.2	165	-20.37	165	-20.37	213729	21	733	426
miR529b	LOC_Os03g01350.1	165	-20.29	165	-20.29	213744	21	3086	90
miR529b	LOC_Os03g01580.1	155	-21.96	155	-21.96	213770	21	3882	3493
miR529b	LOC_Os03g01580.2	155	-21.96	155	-21.96	213771	21	3870	3481
miR529b	LOC_Os03g01750.1	170	-21.42	170	-21.42	213794	21	1651	513
miR529b	LOC_Os03g01750.2	170	-21.42	170	-21.42	213795	21	1605	467
miR529b	LOC_Os03g01750.4	170	-21.42	170	-21.42	213796	21	1858	513
miR529b	LOC_Os03g01750.5	170	-21.42	170	-21.42	213797	21	1752	513
miR529b	LOC_Os03g01750.6	170	-21.42	170	-21.42	213798	21	2024	513
miR529b	LOC_Os03g01750.7	170	-21.42	170	-21.42	213799	21	2128	617
miR529b	LOC_Os03g02200.1	160	-20.32	160	-20.32	213866	21	1400	8
miR529b	LOC_Os03g02200.2	160	-20.32	160	-20.32	213867	21	1397	8
miR529b	LOC_Os03g02200.3	160	-20.32	160	-20.32	213868	21	1380	8
miR529b	LOC_Os03g02320.1	166	-25.14	166	-25.14	213880	21	2930	1
miR529b	LOC_Os03g02320.2	166	-25.14	166	-25.14	213881	21	3004	1
miR529b	LOC_Os03g02440.1	157	-20.02	157	-20.02	213896	21	4464	3175
miR529b	LOC_Os03g02440.2	157	-20.02	157	-20.02	213897	21	4051	2762
miR529b	LOC_Os03g02900.1	156	-23.66	156	-23.66	213965	21	1959	1771
miR529b	LOC_Os03g03510.1	158	-21.31	158	-21.31	214038	21	2284	968
miR529b	LOC_Os03g03510.2	158	-21.31	158	-21.31	214039	21	2395	968
miR529b	LOC_Os03g04020.1	160	-20.83	160	-20.83	214104	21	1591	1
miR529b	LOC_Os03g04020.2	160	-20.83	160	-20.83	214105	21	1700	1
miR529b	LOC_Os03g04970.1	162	-24.78	162	-24.78	214218	21	2269	981
miR529b	LOC_Os03g05730.1	315	-41.35	159	-21.33	214317	21	2915	2861 2571
miR529b	LOC_Os03g06420.1	156	-20.42	156	-20.42	214399	21	2345	1906

miR529b	LOC_Os03g06420.2	156	-20.42	156	-20.42	214400	21	2487	1906
miR529b	LOC_Os03g06420.3	156	-20.42	156	-20.42	214401	21	2442	2003
miR529b	LOC_Os03g06420.4	156	-20.42	156	-20.42	214402	21	2601	2162
miR529b	LOC_Os03g06570.1	154	-21.45	154	-21.45	214417	21	1594	23
miR529b	LOC_Os03g06720.1	155	-21.4	155	-21.4	214437	21	2983	1284
miR529b	LOC_Os03g07200.1	164	-20.71	164	-20.71	214502	21	1589	1532
miR529b	LOC_Os03g07200.2	164	-20.71	164	-20.71	214503	21	1491	1433
miR529b	LOC_Os03g07580.1	161	-21.31	161	-21.31	214552	21	4354	3829
miR529b	LOC_Os03g07720.1	165	-23.83	165	-23.83	214566	21	2193	388
miR529b	LOC_Os03g07970.1	156	-21.33	156	-21.33	214592	21	2148	284
miR529b	LOC_Os03g07980.1	173	-22.16	173	-22.16	214593	21	748	163
miR529b	LOC_Os03g08280.1	162	-20.37	162	-20.37	214634	21	1209	523
miR529b	LOC_Os03g08280.2	162	-20.37	162	-20.37	214635	21	1107	523
miR529b	LOC_Os03g08280.3	162	-20.37	162	-20.37	214636	21	1194	610
miR529b	LOC_Os03g08530.1	167	-20.26	167	-20.26	214673	21	2214	991
miR529b	LOC_Os03g08700.1	166	-23.9	166	-23.9	214697	21	3972	2361
miR529b	LOC_Os03g10800.1	168	-22.71	168	-22.71	214892	21	3189	342
miR529b	LOC_Os03g10800.2	168	-22.71	168	-22.71	214893	21	3298	461
miR529b	LOC_Os03g10800.3	168	-22.71	168	-22.71	214894	21	3518	681
miR529b	LOC_Os03g10800.4	168	-22.71	168	-22.71	214895	21	3417	342
miR529b	LOC_Os03g11240.1	159	-20.96	159	-20.96	214946	21	4577	168
miR529b	LOC_Os03g11300.1	150	-20.42	150	-20.42	214952	21	1400	998
miR529b	LOC_Os03g11300.2	150	-20.42	150	-20.42	214953	21	1476	998
miR529b	LOC_Os03g11310.1	162	-22.71	162	-22.71	214954	21	2113	1
miR529b	LOC_Os03g11440.1	313	-44.87	157	-23.81	214974	21	1982	8 1256
miR529b	LOC_Os03g11490.1	165	-21.34	165	-21.34	214979	21	3681	28
miR529b	LOC_Os03g12250.1	166	-24.54	166	-24.54	215082	21	2657	81
miR529b	LOC_Os03g12840.1	154	-24.62	154	-24.62	215157	21	2070	1116
miR529b	LOC_Os03g12840.2	154	-24.62	154	-24.62	215158	21	1780	1116
miR529b	LOC_Os03g12840.3	154	-24.62	154	-24.62	215159	21	1877	1116
miR529b	LOC_Os03g12879.1	316	-42.59	165	-22.01	215163	21	1193	185 228
miR529b	LOC_Os03g12990.1	154	-22.37	154	-22.37	215187	21	838	86

miR529b	LOC_Os03g13460.1	168	-22.46	168	-22.46	215241	21	3291	3018
miR529b	LOC_Os03g13460.2	168	-22.46	168	-22.46	215242	21	3715	3442
miR529b	LOC_Os03g13680.1	145	-20.29	145	-20.29	215265	21	2109	1577
miR529b	LOC_Os03g13810.1	159	-21.02	159	-21.02	215282	21	2361	4
miR529b	LOC_Os03g13810.2	159	-21.02	159	-21.02	215283	21	2353	2
miR529b	LOC_Os03g13810.3	159	-21.02	159	-21.02	215284	21	3344	4
miR529b	LOC_Os03g14205.1	161	-20.08	161	-20.08	215343	21	1266	1
miR529b	LOC_Os03g14860.1	165	-24.72	165	-24.72	215417	21	1287	506
miR529b	LOC_Os03g14950.1	159	-21.25	159	-21.25	215427	21	2103	43
miR529b	LOC_Os03g15040.1	162	-23.61	162	-23.61	215438	21	3441	176
miR529b	LOC_Os03g15040.2	162	-23.61	162	-23.61	215439	21	3449	176
miR529b	LOC_Os03g15710.1	166	-22.18	166	-22.18	215514	21	1806	1707
miR529b	LOC_Os03g15890.1	157	-21.94	157	-21.94	215543	21	1464	602
miR529b	LOC_Os03g15890.2	157	-21.94	157	-21.94	215544	21	1449	602
miR529b	LOC_Os03g15890.3	157	-21.94	157	-21.94	215545	21	1827	602
miR529b	LOC_Os03g16390.1	160	-20.74	160	-20.74	215625	21	1937	656
miR529b	LOC_Os03g16530.1	153	-20.34	153	-20.34	215640	21	6336	1392
miR529b	LOC_Os03g17270.1	160	-21.27	160	-21.27	215737	21	2288	370
miR529b	LOC_Os03g17270.2	160	-21.27	160	-21.27	215738	21	2442	370
miR529b	LOC_Os03g17570.1	163	-20.01	163	-20.01	215770	21	3610	1582
miR529b	LOC_Os03g17570.2	163	-20.01	163	-20.01	215771	21	3596	1582
miR529b	LOC_Os03g17570.3	163	-20.01	163	-20.01	215772	21	3591	1582
miR529b	LOC_Os03g17570.4	163	-20.01	163	-20.01	215773	21	4061	2033
miR529b	LOC_Os03g17570.5	163	-20.01	163	-20.01	215774	21	3497	1469
miR529b	LOC_Os03g17570.6	163	-20.01	163	-20.01	215775	21	3691	1663
miR529b	LOC_Os03g18430.1	308	-42.02	158	-21.22	215878	21	2821	2024 1358
miR529b	LOC_Os03g18500.1	161	-21.49	161	-21.49	215885	21	1226	1139
miR529b	LOC_Os03g18500.2	161	-21.49	161	-21.49	215886	21	1136	1053
miR529b	LOC_Os03g18900.1	168	-24.98	168	-24.98	215931	21	909	251
miR529b	LOC_Os03g19010.1	161	-21.08	161	-21.08	215941	21	1211	78
miR529b	LOC_Os03g19150.1	174	-26.46	174	-26.46	215957	21	2208	631
miR529b	LOC_Os03g19270.1	156	-20.62	156	-20.62	215970	21	1058	995

miR529b	LOC_Os03g19270.2	156	-20.62	156	-20.62	215971	21	1018	955
miR529b	LOC_Os03g19270.3	156	-20.62	156	-20.62	215972	21	1009	946
miR529b	LOC_Os03g19270.4	156	-20.62	156	-20.62	215973	21	1152	1089
miR529b	LOC_Os03g20470.1	160	-24.14	160	-24.14	216125	21	1148	196
miR529b	LOC_Os03g20470.2	160	-24.14	160	-24.14	216126	21	1165	192
miR529b	LOC_Os03g20500.1	157	-20.14	157	-20.14	216129	21	1679	1291
miR529b	LOC_Os03g21360.1	166	-20.05	166	-20.05	216225	21	1113	1082
miR529b	LOC_Os03g21680.1	160	-20.16	160	-20.16	216266	21	4395	74
miR529b	LOC_Os03g21680.2	160	-20.16	160	-20.16	216267	21	4293	74
miR529b	LOC_Os03g21680.3	160	-20.16	160	-20.16	216268	21	4460	74
miR529b	LOC_Os03g21680.4	160	-20.16	160	-20.16	216269	21	4560	74
miR529b	LOC_Os03g21850.1	163	-22.6	163	-22.6	216288	21	3700	2898
miR529b	LOC_Os03g21850.2	163	-22.6	163	-22.6	216289	21	3424	2804
miR529b	LOC_Os03g23110.1	167	-21.93	167	-21.93	216451	21	4929	3693
miR529b	LOC_Os03g24040.1	171	-29.44	171	-29.44	216489	21	1493	1463
miR529b	LOC_Os03g25050.1	161	-24.34	161	-24.34	216592	21	671	593
miR529b	LOC_Os03g25170.1	174	-26.46	174	-26.46	216604	21	684	631
miR529b	LOC_Os03g25330.1	163	-22.35	163	-22.35	216624	21	1473	16
miR529b	LOC_Os03g25580.1	166	-21.94	166	-21.94	216657	21	1989	204
miR529b	LOC_Os03g25720.1	157	-21.94	157	-21.94	216671	21	1848	1128
miR529b	LOC_Os03g26570.1	163	-22.51	163	-22.51	216750	21	3285	2591
miR529b	LOC_Os03g27390.1	166	-21.43	166	-21.43	216842	21	2558	949
miR529b	LOC_Os03g27390.2	166	-21.43	166	-21.43	216843	21	2581	949
miR529b	LOC_Os03g27590.1	162	-20.54	162	-20.54	216867	21	1999	112
miR529b	LOC_Os03g27590.2	162	-20.54	162	-20.54	216868	21	2766	112
miR529b	LOC_Os03g27590.3	162	-20.54	162	-20.54	216869	21	2807	112
miR529b	LOC_Os03g27780.1	167	-23.04	167	-23.04	216884	21	2522	10
miR529b	LOC_Os03g27780.2	167	-23.04	167	-23.04	216885	21	2606	10
miR529b	LOC_Os03g29400.1	165	-21.38	165	-21.38	217012	21	675	214
miR529b	LOC_Os03g29410.1	155	-21.63	155	-21.63	217013	21	1879	1847
miR529b	LOC_Os03g29790.1	157	-21.69	157	-21.69	217046	21	4647	531
miR529b	LOC_Os03g29920.1	334	-46.86	169	-23.61	217055	21	1228	75 33

miR529b	LOC_Os03g30190.1	161	-20.5	161	-20.5	217087	21	1608	1583
miR529b	LOC_Os03g30810.1	157	-21.06	157	-21.06	217150	21	3411	1082
miR529b	LOC_Os03g31520.1	153	-20.34	153	-20.34	217229	21	1536	1431
miR529b	LOC_Os03g32170.1	154	-21	154	-21	217289	21	1864	727
miR529b	LOC_Os03g32650.1	156	-21.71	156	-21.71	217334	21	4593	2895
miR529b	LOC_Os03g33530.1	145	-20.29	145	-20.29	217417	21	6084	5291
miR529b	LOC_Os03g33590.1	161	-21.66	161	-21.66	217423	21	1839	332
miR529b	LOC_Os03g33590.2	161	-21.66	161	-21.66	217424	21	1815	308
miR529b	LOC_Os03g33810.1	153	-20.34	153	-20.34	217445	21	1641	1611
miR529b	LOC_Os03g35710.1	174	-21.84	174	-21.84	217533	21	1521	1401
miR529b	LOC_Os03g35720.1	161	-24.99	161	-24.99	217534	21	981	956
miR529b	LOC_Os03g35750.1	159	-20.89	159	-20.89	217537	21	357	257
miR529b	LOC_Os03g35788.1	149	-21.86	149	-21.86	217538	21	5724	4931
miR529b	LOC_Os03g35990.1	160	-20.8	160	-20.8	217557	21	1455	674
miR529b	LOC_Os03g36000.1	161	-21.9	161	-21.9	217558	21	3555	3040
miR529b	LOC_Os03g36460.1	164	-22.28	164	-22.28	217595	21	519	8
miR529b	LOC_Os03g36760.1	164	-20.86	164	-20.86	217625	21	4181	949
miR529b	LOC_Os03g36760.2	164	-20.86	164	-20.86	217626	21	4090	949
miR529b	LOC_Os03g37270.1	154	-21.03	154	-21.03	217671	21	1896	1506
miR529b	LOC_Os03g37520.1	154	-20.41	154	-20.41	217688	21	492	56
miR529b	LOC_Os03g37940.1	154	-23.15	154	-23.15	217731	21	294	126
miR529b	LOC_Os03g38010.1	169	-23.26	169	-23.26	217745	21	1813	906
miR529b	LOC_Os03g38270.1	157	-21.1	157	-21.1	217769	21	2217	153
miR529b	LOC_Os03g39250.1	157	-21.69	157	-21.69	217871	21	3717	492
miR529b	LOC_Os03g39330.1	161	-21.34	161	-21.34	217876	21	3603	387
miR529b	LOC_Os03g39362.1	165	-20.64	165	-20.64	217879	21	315	114
miR529b	LOC_Os03g40160.1	327	-47.72	164	-24.44	217956	21	3226	1830 3138
miR529b	LOC_Os03g41339.1	156	-20.15	156	-20.15	218057	21	468	20
miR529b	LOC_Os03g41840.1	145	-20.29	145	-20.29	218105	21	1305	548
miR529b	LOC_Os03g42070.1	161	-23.63	161	-23.63	218127	21	2173	404
miR529b	LOC_Os03g42100.1	160	-21.25	160	-21.25	218130	21	933	200
miR529b	LOC_Os03g42370.1	166	-20.28	166	-20.28	218158	21	3090	329

miR529b	LOC_Os03g42660.1	156	-20.55	156	-20.55	218184	21	1926	626
miR529b	LOC_Os03g42770.1	164	-20.92	164	-20.92	218195	21	2162	459
miR529b	LOC_Os03g43684.1	160	-22.52	160	-22.52	218291	21	5210	3089
miR529b	LOC_Os03g43720.1	159	-21.25	159	-21.25	218294	21	2380	1993
miR529b	LOC_Os03g43720.2	159	-21.25	159	-21.25	218295	21	2405	2018
miR529b	LOC_Os03g43720.3	159	-21.25	159	-21.25	218296	21	2428	2041
miR529b	LOC_Os03g43720.4	159	-21.25	159	-21.25	218297	21	2273	1886
miR529b	LOC_Os03g43720.5	159	-21.25	159	-21.25	218298	21	2337	1960
miR529b	LOC_Os03g43720.6	159	-21.25	159	-21.25	218299	21	2961	2574
miR529b	LOC_Os03g43990.1	168	-24.98	168	-24.98	218330	21	2694	2654
miR529b	LOC_Os03g44020.1	152	-20.28	152	-20.28	218335	21	1663	1462
miR529b	LOC_Os03g44890.1	161	-23.03	161	-23.03	218436	21	3543	2706
miR529b	LOC_Os03g45300.1	161	-25.45	161	-25.45	218475	21	4183	3997
miR529b	LOC_Os03g45390.1	166	-23.01	166	-23.01	218484	21	1815	22
miR529b	LOC_Os03g45750.1	160	-21.44	160	-21.44	218516	21	1263	10
miR529b	LOC_Os03g46330.1	151	-20.17	151	-20.17	218580	21	6123	6071
miR529b	LOC_Os03g46340.1	151	-20.17	151	-20.17	218581	21	3131	1769
miR529b	LOC_Os03g46440.3	151	-20.15	151	-20.15	218593	21	3770	1082
miR529b	LOC_Os03g46540.1	165	-20.32	165	-20.32	218604	21	525	19
miR529b	LOC_Os03g47740.1	158	-25.52	158	-25.52	218729	21	2894	2647
miR529b	LOC_Os03g47740.2	158	-25.52	158	-25.52	218730	21	2989	2742
miR529b	LOC_Os03g48140.1	151	-21.23	151	-21.23	218780	21	5166	2406
miR529b	LOC_Os03g48810.1	169	-23.19	169	-23.19	218857	21	1951	1286
miR529b	LOC_Os03g49710.1	164	-22.83	164	-22.83	218980	21	882	824
miR529b	LOC_Os03g49800.1	158	-20.42	158	-20.42	218991	21	2990	2878
miR529b	LOC_Os03g49940.1	161	-24.06	161	-24.06	219005	21	2040	1144
miR529b	LOC_Os03g50100.1	161	-20.2	161	-20.2	219024	21	3451	1414
miR529b	LOC_Os03g50970.1	151	-22.27	151	-22.27	219117	21	2446	630
miR529b	LOC_Os03g51580.1	161	-23.14	161	-23.14	219187	21	2011	1834
miR529b	LOC_Os03g52390.1	157	-20.26	157	-20.26	219280	21	503	343
miR529b	LOC_Os03g52794.1	162	-20.53	162	-20.53	219328	21	11361	7855
miR529b	LOC_Os03g52900.1	165	-21.77	165	-21.77	219340	21	2772	231

miR529b	LOC_Os03g52950.1	164	-22.71	164	-22.71	219345	21	3183	982
miR529b	LOC_Os03g53250.1	160	-20.1	160	-20.1	219379	21	3176	1834
miR529b	LOC_Os03g53980.1	159	-20.13	159	-20.13	219477	21	693	410
miR529b	LOC_Os03g53980.2	159	-20.13	159	-20.13	219478	21	613	329
miR529b	LOC_Os03g54114.1	157	-22.31	157	-22.31	219496	21	492	172
miR529b	LOC_Os03g54840.1	160	-21.55	160	-21.55	219520	21	474	39
miR529b	LOC_Os03g54850.1	161	-22.53	161	-22.53	219521	21	1885	60
miR529b	LOC_Os03g55850.1	157	-21.05	157	-21.05	219646	21	1375	631
miR529b	LOC_Os03g55950.3	159	-22.49	159	-22.49	219658	21	2437	249
miR529b	LOC_Os03g56310.1	163	-20.66	163	-20.66	219700	21	2422	1525
miR529b	LOC_Os03g56310.2	163	-20.66	163	-20.66	219701	21	2415	1525
miR529b	LOC_Os03g56310.3	163	-20.66	163	-20.66	219702	21	2556	1659
miR529b	LOC_Os03g56310.4	163	-20.66	163	-20.66	219703	21	1923	1026
miR529b	LOC_Os03g56590.1	161	-26.84	161	-26.84	219737	21	1853	734
miR529b	LOC_Os03g56730.1	328	-42.38	164	-21.29	219757	21	1656	1185 1225
miR529b	LOC_Os03g56730.2	328	-42.38	164	-21.29	219758	21	1701	1185 1225
miR529b	LOC_Os03g57569.1	168	-23.18	168	-23.18	219875	21	2196	1029
miR529b	LOC_Os03g57569.2	168	-23.18	168	-23.18	219876	21	1898	731
miR529b	LOC_Os03g57854.1	161	-23.62	161	-23.62	219908	21	1844	101
miR529b	LOC_Os03g57854.2	161	-23.62	161	-23.62	219909	21	1864	101
miR529b	LOC_Os03g57940.1	150	-21.96	150	-21.96	219921	21	2723	1753
miR529b	LOC_Os03g57940.2	150	-21.96	150	-21.96	219922	21	2715	1745
miR529b	LOC_Os03g57940.3	150	-21.96	150	-21.96	219923	21	2764	1794
miR529b	LOC_Os03g57940.4	150	-21.96	150	-21.96	219924	21	2791	1821
miR529b	LOC_Os03g58220.1	161	-20.84	161	-20.84	219960	21	1242	863
miR529b	LOC_Os03g58372.1	162	-21.54	162	-21.54	219975	21	2901	497
miR529b	LOC_Os03g58470.1	166	-26.62	166	-26.62	219984	21	1414	1043
miR529b	LOC_Os03g58870.1	163	-20.45	163	-20.45	220029	21	987	835
miR529b	LOC_Os03g59170.1	159	-23.66	159	-23.66	220063	21	1531	314
miR529b	LOC_Os03g59250.1	166	-22.95	166	-22.95	220072	21	1455	316
miR529b	LOC_Os03g59680.1	159	-20.11	159	-20.11	220116	21	2266	944
miR529b	LOC_Os03g60150.2	159	-20.38	159	-20.38	220168	21	2345	790

miR529b	LOC_Os03g60180.1	173	-28.2	173	-28.2	220170	21	2162	1827
miR529b	LOC_Os03g60200.1	163	-23.62	163	-23.62	220173	21	3085	2754
miR529b	LOC_Os03g60920.1	168	-24.99	168	-24.99	220257	21	1431	1
miR529b	LOC_Os03g61010.1	153	-20.56	153	-20.56	220265	21	2061	1741
miR529b	LOC_Os03g61620.1	168	-24.98	168	-24.98	220341	21	3188	85
miR529b	LOC_Os03g61620.2	168	-24.98	168	-24.98	220342	21	3452	85
miR529b	LOC_Os03g61800.1	160	-21.18	160	-21.18	220364	21	2278	300
miR529b	LOC_Os03g61800.2	160	-21.18	160	-21.18	220365	21	2290	300
miR529b	LOC_Os03g62090.1	161	-20.17	161	-20.17	220409	21	4365	222
miR529b	LOC_Os03g62224.1	171	-24.28	171	-24.28	220428	21	354	84
miR529b	LOC_Os03g62340.1	165	-25.76	165	-25.76	220440	21	3627	3514
miR529b	LOC_Os03g62379.1	164	-21.78	164	-21.78	220444	21	657	53
miR529b	LOC_Os03g62650.1	165	-23.73	165	-23.73	220484	21	3232	478
miR529b	LOC_Os03g62650.2	165	-23.73	165	-23.73	220485	21	3392	478
miR529b	LOC_Os03g62650.3	165	-23.73	165	-23.73	220486	21	3209	478
miR529b	LOC_Os03g62730.1	161	-20.56	161	-20.56	220500	21	3611	83
miR529b	LOC_Os03g62730.2	161	-20.56	161	-20.56	220501	21	3627	83
miR529b	LOC_Os03g62730.3	161	-20.56	161	-20.56	220502	21	3695	83
miR529b	LOC_Os03g62830.1	173	-24.33	173	-24.33	220517	21	2034	1087
miR529b	LOC_Os03g63710.1	161	-20.64	161	-20.64	220622	21	2249	708
miR529b	LOC_Os03g63710.2	161	-20.64	161	-20.64	220623	21	2200	914
miR529b	LOC_Os03g64080.1	162	-20.53	162	-20.53	220670	21	6225	791
miR529b	LOC_Os03g64210.1	156	-20.31	156	-20.31	220683	21	2439	1054
miR529b	LOC_Os03g64210.2	156	-20.31	156	-20.31	220684	21	2599	1054
miR529b	LOC_Os03g64210.3	156	-20.31	156	-20.31	220685	21	2401	1054
miR529b	LOC_Os03g64250.5	168	-23.5	168	-23.5	220691	21	2249	670
miR529b	LOC_Os04g01030.1	158	-22.08	158	-22.08	220720	21	4293	2284
miR529b	LOC_Os04g01380.1	162	-26.86	162	-26.86	220762	21	1614	612
miR529b	LOC_Os04g02420.1	149	-21.35	149	-21.35	220874	21	2778	460
miR529b	LOC_Os04g02920.1	166	-20.27	166	-20.27	220935	21	2552	358
miR529b	LOC_Os04g03490.1	157	-21.69	157	-21.69	220987	21	4005	594
miR529b	LOC_Os04g04170.1	169	-23.84	169	-23.84	221050	21	1689	548

miR529b	LOC_Os04g04490.1	157	-21.69	157	-21.69	221082	21	4401	531	
miR529b	LOC_Os04g05030.1	161	-20.09	161	-20.09	221131	21	1622	20	
miR529b	LOC_Os04g05140.1	154	-20.29	154	-20.29	221142	21	1608	14	
miR529b	LOC_Os04g05270.1	154	-20.29	154	-20.29	221155	21	1650	14	
miR529b	LOC_Os04g05680.1	157	-22.29	157	-22.29	221191	21	2418	2393	
miR529b	LOC_Os04g05770.1	167	-26.67	167	-26.67	221199	21	2019	1454	
miR529b	LOC_Os04g06130.1	167	-21.93	167	-21.93	221233	21	4980	3915	
miR529b	LOC_Os04g06420.1	154	-20.29	154	-20.29	221258	21	1686	14	
miR529b	LOC_Os04g07690.1	164	-21.57	164	-21.57	221379	21	513	10	
miR529b	LOC_Os04g08034.1	156	-21.26	156	-21.26	221412	21	2904	712	
miR529b	LOC_Os04g08034.2	156	-21.26	156	-21.26	221413	21	2847	655	
miR529b	LOC_Os04g08034.3	156	-21.26	156	-21.26	221414	21	2708	516	
miR529b	LOC_Os04g08034.6	156	-21.26	156	-21.26	221417	21	1655	264	
miR529b	LOC_Os04g08784.1	145	-20.29	145	-20.29	221494	21	1044	251	
miR529b	LOC_Os04g08804.1	169	-23.84	169	-23.84	221499	21	2901	1973	
miR529b	LOC_Os04g09710.1	162	-22.24	162	-22.24	221550	21	1752	1725	
miR529b	LOC_Os04g10380.1	165	-24.08	165	-24.08	221618	21	5131	1	
miR529b	LOC_Os04g10620.1	157	-21.69	157	-21.69	221636	21	4422	531	
miR529b	LOC_Os04g12120.1	168	-24.98	168	-24.98	221789	21	978	49	
miR529b	LOC_Os04g13830.1	154	-20.29	154	-20.29	221943	21	375	14	
miR529b	LOC_Os04g13970.1	161	-20.3	161	-20.3	221957	21	1686	1198	
miR529b	LOC_Os04g14340.1	163	-20.51	163	-20.51	221995	21	3398	1649	
miR529b	LOC_Os04g14890.1	167	-23.13	167	-23.13	222051	21	4494	3486	
miR529b	LOC_Os04g15820.1	172	-25.16	172	-25.16	222094	21	3199	2435	
miR529b	LOC_Os04g16280.1	171	-21.78	171	-21.78	222135	21	1845	1453	
miR529b	LOC_Os04g16450.1	328	-47.34	168	-23.78	222151	21	1629	1509	1495
miR529b	LOC_Os04g16722.1	153	-21.5	153	-21.5	222178	21	4203	2186	
miR529b	LOC_Os04g16896.1	153	-21.5	153	-21.5	222179	21	402	138	
miR529b	LOC_Os04g18340.1	171	-20.71	171	-20.71	222372	21	2592	549	
miR529b	LOC_Os04g18660.1	310	-42.61	156	-21.59	222403	21	2622	2379	2139
miR529b	LOC_Os04g18700.1	164	-20.26	164	-20.26	222407	21	5565	3436	
miR529b	LOC_Os04g19820.1	161	-20.17	161	-20.17	222517	21	3303	24	

miR529b	LOC_Os04g20090.1	178	-28.3	178	-28.3	222541	21	2415	1319
miR529b	LOC_Os04g20180.1	310	-40.4	155	-20.2	222550	21	2890	2246 2393
miR529b	LOC_Os04g20490.1	154	-21.25	154	-21.25	222585	21	306	53
miR529b	LOC_Os04g20960.1	154	-22.12	154	-22.12	222629	21	3648	140
miR529b	LOC_Os04g21100.1	173	-25.41	173	-25.41	222640	21	2229	1253
miR529b	LOC_Os04g21560.1	157	-21.69	157	-21.69	222684	21	4578	531
miR529b	LOC_Os04g21890.1	159	-21.04	159	-21.04	222718	21	2799	1805
miR529b	LOC_Os04g23130.1	165	-22.28	165	-22.28	222836	21	492	45
miR529b	LOC_Os04g23620.1	160	-20.53	160	-20.53	222880	21	2130	807
miR529b	LOC_Os04g23890.2	168	-22.71	168	-22.71	222900	21	3248	3153
miR529b	LOC_Os04g24130.1	174	-24.07	174	-24.07	222923	21	1302	464
miR529b	LOC_Os04g24670.1	174	-22.74	174	-22.74	222985	21	417	62
miR529b	LOC_Os04g24820.1	154	-20.28	154	-20.28	222999	21	1725	1694
miR529b	LOC_Os04g26320.1	162	-21.86	162	-21.86	223154	21	996	901
miR529b	LOC_Os04g26850.2	165	-22.22	165	-22.22	223201	21	1493	1305
miR529b	LOC_Os04g27270.1	165	-20.59	165	-20.59	223243	21	1486	889
miR529b	LOC_Os04g27400.1	168	-21.58	168	-21.58	223254	21	1524	1379
miR529b	LOC_Os04g28300.1	161	-22.41	161	-22.41	223341	21	2535	2432
miR529b	LOC_Os04g28620.1	161	-24.19	161	-24.19	223371	21	1866	1730
miR529b	LOC_Os04g29600.1	156	-21.71	156	-21.71	223459	21	2436	879
miR529b	LOC_Os04g30300.1	161	-25.68	161	-25.68	223527	21	2082	1505
miR529b	LOC_Os04g30870.1	321	-44.64	164	-23.28	223585	21	3486	1806 3397
miR529b	LOC_Os04g30920.1	165	-22.28	165	-22.28	223592	21	855	728
miR529b	LOC_Os04g32330.1	162	-21.06	162	-21.06	223738	21	1894	22
miR529b	LOC_Os04g32330.2	162	-21.06	162	-21.06	223739	21	1866	1
miR529b	LOC_Os04g32550.1	161	-20.33	161	-20.33	223759	21	854	2
miR529b	LOC_Os04g32670.1	164	-20.85	164	-20.85	223775	21	2826	1
miR529b	LOC_Os04g32900.1	157	-20.2	157	-20.2	223804	21	468	426
miR529b	LOC_Os04g33080.1	162	-20.44	162	-20.44	223828	21	2573	2333
miR529b	LOC_Os04g33080.2	162	-20.44	162	-20.44	223829	21	2558	2450
miR529b	LOC_Os04g33080.3	162	-20.44	162	-20.44	223830	21	2619	2466
miR529b	LOC_Os04g33600.1	161	-23.11	161	-23.11	223892	21	1584	14

miR529b	LOC_Os04g33610.1	159	-21.1	159	-21.1	223893	21	1252	125
miR529b	LOC_Os04g33810.1	170	-24.03	170	-24.03	223914	21	1005	17
miR529b	LOC_Os04g35430.1	159	-20.01	159	-20.01	224083	21	4486	1698
miR529b	LOC_Os04g35510.1	320	-40.46	164	-20.4	224091	21	1606	915 1225
miR529b	LOC_Os04g35510.2	320	-40.46	164	-20.4	224092	21	1555	915 1225
miR529b	LOC_Os04g37590.1	165	-23.33	165	-23.33	224223	21	1242	888
miR529b	LOC_Os04g37840.1	155	-20.37	155	-20.37	224251	21	4551	4014
miR529b	LOC_Os04g37904.1	158	-20.59	158	-20.59	224257	21	1768	1197
miR529b	LOC_Os04g37904.2	158	-20.59	158	-20.59	224258	21	1765	1194
miR529b	LOC_Os04g37980.1	152	-22.16	152	-22.16	224268	21	1834	626
miR529b	LOC_Os04g38290.1	162	-20.02	162	-20.02	224296	21	3620	1662
miR529b	LOC_Os04g38380.1	157	-26.74	157	-26.74	224307	21	762	214
miR529b	LOC_Os04g38410.1	155	-20.01	155	-20.01	224310	21	1589	1109
miR529b	LOC_Os04g38960.1	158	-21.75	158	-21.75	224386	21	1839	446
miR529b	LOC_Os04g39260.1	156	-22.48	156	-22.48	224421	21	2453	1455
miR529b	LOC_Os04g39260.2	156	-22.48	156	-22.48	224422	21	2570	1453
miR529b	LOC_Os04g39260.3	156	-22.48	156	-22.48	224423	21	2470	1453
miR529b	LOC_Os04g39410.1	156	-20.84	156	-20.84	224441	21	3371	313
miR529b	LOC_Os04g40360.1	156	-21.71	156	-21.71	224550	21	4110	2895
miR529b	LOC_Os04g40850.1	312	-41.4	156	-21	224615	21	1583	32 1505
miR529b	LOC_Os04g40850.2	156	-21	156	-21	224616	21	1538	1460
miR529b	LOC_Os04g40970.1	164	-20.09	164	-20.09	224636	21	3574	3337
miR529b	LOC_Os04g41950.1	171	-30.08	171	-30.08	224748	21	2796	1815
miR529b	LOC_Os04g42470.1	145	-20.76	145	-20.76	224810	21	2507	2148
miR529b	LOC_Os04g42470.2	145	-20.76	145	-20.76	224811	21	2429	2070
miR529b	LOC_Os04g42470.3	145	-20.76	145	-20.76	224812	21	2426	2067
miR529b	LOC_Os04g42470.4	145	-20.76	145	-20.76	224813	21	2315	2067
miR529b	LOC_Os04g42470.5	145	-20.76	145	-20.76	224814	21	2355	1996
miR529b	LOC_Os04g42470.6	145	-20.76	145	-20.76	224815	21	2499	2140
miR529b	LOC_Os04g42470.7	145	-20.76	145	-20.76	224816	21	2394	2140
miR529b	LOC_Os04g42470.8	145	-20.76	145	-20.76	224817	21	1934	1575
miR529b	LOC_Os04g42480.1	173	-29.13	173	-29.13	224818	21	3297	369

miR529b	LOC_Os04g42620.1	174	-27.24	174	-27.24	224834	21	1883	205
miR529b	LOC_Os04g42740.1	149	-25.81	149	-25.81	224844	21	2382	709
miR529b	LOC_Os04g43070.1	146	-22.71	146	-22.71	224891	21	2476	1703
miR529b	LOC_Os04g43480.1	161	-24.99	161	-24.99	224946	21	2241	2216
miR529b	LOC_Os04g43500.1	156	-21.71	156	-21.71	224949	21	4251	3213
miR529b	LOC_Os04g43850.1	156	-21.71	156	-21.71	224985	21	3315	1968
miR529b	LOC_Os04g44170.1	164	-21.79	164	-21.79	225018	21	621	1
miR529b	LOC_Os04g44850.1	157	-20.61	157	-20.61	225105	21	1918	150
miR529b	LOC_Os04g44950.1	159	-20.37	159	-20.37	225121	21	1318	1262
miR529b	LOC_Os04g45490.1	159	-22.76	159	-22.76	225185	21	2306	28
miR529b	LOC_Os04g46310.1	161	-20.31	161	-20.31	225275	21	6429	3539
miR529b	LOC_Os04g46580.1	182	-33.78	182	-33.78	225313	21	1518	812
miR529b	LOC_Os04g46740.1	169	-20.67	169	-20.67	225333	21	1542	1499
miR529b	LOC_Os04g46740.2	169	-20.67	169	-20.67	225334	21	1985	1945
miR529b	LOC_Os04g46990.1	159	-20.21	159	-20.21	225369	21	1663	999
miR529b	LOC_Os04g48760.1	158	-21.46	158	-21.46	225579	21	4296	57
miR529b	LOC_Os04g49500.1	159	-20.8	159	-20.8	225660	21	3369	3229
miR529b	LOC_Os04g50216.1	161	-21.54	161	-21.54	225753	21	1329	1278
miR529b	LOC_Os04g50920.1	165	-23.61	165	-23.61	225781	21	1815	24
miR529b	LOC_Os04g51250.1	155	-23.18	155	-23.18	225824	21	1160	1046
miR529b	LOC_Os04g51809.1	169	-20.93	169	-20.93	225900	21	2132	942
miR529b	LOC_Os04g51809.2	169	-20.93	169	-20.93	225901	21	1785	942
miR529b	LOC_Os04g51809.3	169	-20.93	169	-20.93	225902	21	1658	942
miR529b	LOC_Os04g52060.1	170	-21.78	170	-21.78	225939	21	2800	1812
miR529b	LOC_Os04g52190.1	162	-25.2	162	-25.2	225953	21	2824	2495
miR529b	LOC_Os04g52200.1	162	-25.2	162	-25.2	225954	21	3138	467
miR529b	LOC_Os04g53496.1	322	-43.67	163	-22.2	226119	21	5424	4314 5082
miR529b	LOC_Os04g53720.1	161	-20.65	161	-20.65	226150	21	3864	459
miR529b	LOC_Os04g55100.1	156	-22.62	156	-22.62	226313	21	547	122
miR529b	LOC_Os04g55510.1	162	-22.57	162	-22.57	226359	21	2891	89
miR529b	LOC_Os04g55560.2	161	-20.7	161	-20.7	226365	21	2490	207
miR529b	LOC_Os04g55560.3	161	-20.7	161	-20.7	226366	21	2501	221

miR529b	LOC_Os04g55640.1	158	-21.96	158	-21.96	226375	21	1419	119
miR529b	LOC_Os04g56440.1	153	-20.35	153	-20.35	226471	21	2566	1983
miR529b	LOC_Os04g56440.2	153	-20.35	153	-20.35	226472	21	2643	2060
miR529b	LOC_Os04g56450.1	153	-20.35	153	-20.35	226473	21	2162	621
miR529b	LOC_Os04g56450.2	153	-20.35	153	-20.35	226474	21	2015	621
miR529b	LOC_Os04g56630.1	163	-22.43	163	-22.43	226496	21	3323	752
miR529b	LOC_Os04g56630.2	163	-22.43	163	-22.43	226497	21	2922	351
miR529b	LOC_Os04g57610.1	171	-21.24	171	-21.24	226633	21	3361	3095
miR529b	LOC_Os04g57610.3	171	-21.24	171	-21.24	226634	21	3349	3083
miR529b	LOC_Os04g57830.1	160	-20.56	160	-20.56	226668	21	2052	13
miR529b	LOC_Os04g58140.1	150	-20.01	150	-20.01	226699	21	2491	2443
miR529b	LOC_Os04g58140.2	150	-20.01	150	-20.01	226700	21	3022	2974
miR529b	LOC_Os04g58250.1	152	-20.82	152	-20.82	226716	21	2608	1447
miR529b	LOC_Os04g58430.1	162	-21.03	162	-21.03	226738	21	906	189
miR529b	LOC_Os04g58580.1	158	-21.77	158	-21.77	226757	21	1314	1019
miR529b	LOC_Os04g58580.2	158	-21.77	158	-21.77	226758	21	1520	1228
miR529b	LOC_Os04g58720.1	157	-21.2	157	-21.2	226777	21	3860	39
miR529b	LOC_Os04g58820.1	162	-23.68	162	-23.68	226793	21	1293	277
miR529b	LOC_Os05g01060.1	152	-25.35	152	-25.35	226916	21	2774	1937
miR529b	LOC_Os05g01060.2	152	-25.35	152	-25.35	226917	21	2399	1562
miR529b	LOC_Os05g01240.1	160	-21.25	160	-21.25	226938	21	3944	3902
miR529b	LOC_Os05g01240.5	160	-21.25	160	-21.25	226942	21	3676	3422
miR529b	LOC_Os05g01256.1	161	-21.64	161	-21.64	226944	21	1663	1351
miR529b	LOC_Os05g01256.2	161	-21.64	161	-21.64	226945	21	1684	1388
miR529b	LOC_Os05g01290.1	160	-21.9	160	-21.9	226951	21	984	64
miR529b	LOC_Os05g01320.1	160	-21.1	160	-21.1	226956	21	1693	289
miR529b	LOC_Os05g01450.1	158	-21.44	158	-21.44	226969	21	1273	1192
miR529b	LOC_Os05g01450.2	158	-21.44	158	-21.44	226970	21	1193	1112
miR529b	LOC_Os05g01520.1	168	-24.98	168	-24.98	226984	21	2496	2469
miR529b	LOC_Os05g01790.1	162	-21.27	162	-21.27	227022	21	1314	53
miR529b	LOC_Os05g02790.1	330	-45.92	170	-25.62	227148	21	3105	1092 1344
miR529b	LOC_Os05g03150.1	161	-23.59	161	-23.59	227189	21	654	600

miR529b	LOC_Os05g03550.1	164	-22.75	164	-22.75	227238	21	5167	1082
miR529b	LOC_Os05g04460.1	164	-20.29	164	-20.29	227338	21	2181	1144
miR529b	LOC_Os05g04520.1	157	-23.99	157	-23.99	227345	21	1632	53
miR529b	LOC_Os05g04630.1	157	-21.15	157	-21.15	227354	21	1966	100
miR529b	LOC_Os05g04630.2	157	-21.15	157	-21.15	227355	21	1961	92
miR529b	LOC_Os05g04630.3	157	-21.15	157	-21.15	227356	21	1981	50
miR529b	LOC_Os05g04630.4	157	-21.15	157	-21.15	227357	21	2023	50
miR529b	LOC_Os05g04630.5	157	-21.15	157	-21.15	227358	21	2020	50
miR529b	LOC_Os05g04630.6	157	-21.15	157	-21.15	227359	21	2533	72
miR529b	LOC_Os05g05170.1	162	-21.74	162	-21.74	227414	21	873	565
miR529b	LOC_Os05g05220.1	161	-22.51	161	-22.51	227421	21	1683	1313
miR529b	LOC_Os05g05300.1	170	-26.33	170	-26.33	227430	21	3778	3612
miR529b	LOC_Os05g05310.1	170	-26.33	170	-26.33	227431	21	3863	2237
miR529b	LOC_Os05g05310.2	170	-26.33	170	-26.33	227432	21	3859	2237
miR529b	LOC_Os05g05420.1	157	-21.69	157	-21.69	227445	21	4935	531
miR529b	LOC_Os05g05820.1	168	-24.98	168	-24.98	227496	21	1454	8
miR529b	LOC_Os05g06110.1	168	-24.98	168	-24.98	227527	21	3260	239
miR529b	LOC_Os05g06710.1	161	-25.59	161	-25.59	227601	21	1841	1800
miR529b	LOC_Os05g07220.1	169	-22.02	169	-22.02	227663	21	3840	3723
miR529b	LOC_Os05g07220.2	169	-22.02	169	-22.02	227664	21	3683	3566
miR529b	LOC_Os05g07280.1	157	-23.38	157	-23.38	227673	21	2680	2452
miR529b	LOC_Os05g07550.1	161	-25	161	-25	227696	21	2481	2456
miR529b	LOC_Os05g08110.1	165	-24.08	165	-24.08	227766	21	940	243
miR529b	LOC_Os05g08370.1	161	-25.23	161	-25.23	227793	21	4069	1723
miR529b	LOC_Os05g08400.1	154	-20.31	154	-20.31	227796	21	1788	336
miR529b	LOC_Os05g08970.1	168	-24.98	168	-24.98	227869	21	2485	43
miR529b	LOC_Os05g09170.1	169	-23.84	169	-23.84	227893	21	3162	1973
miR529b	LOC_Os05g10840.1	164	-22.08	164	-22.08	228040	21	2153	21
miR529b	LOC_Os05g10840.2	164	-22.08	164	-22.08	228041	21	2148	21
miR529b	LOC_Os05g12080.1	162	-22.75	162	-22.75	228169	21	351	27
miR529b	LOC_Os05g12280.1	170	-23.99	170	-23.99	228195	21	986	11
miR529b	LOC_Os05g14320.1	161	-21.45	161	-21.45	228364	21	2950	2067

miR529b	LOC_Os05g14440.1	145	-20.68	145	-20.68	228376	21	2256	2172
miR529b	LOC_Os05g14930.1	155	-21.04	155	-21.04	228421	21	2304	2221
miR529b	LOC_Os05g14970.1	155	-21.04	155	-21.04	228425	21	1784	1441
miR529b	LOC_Os05g16850.1	155	-20.37	155	-20.37	228625	21	4551	4014
miR529b	LOC_Os05g17130.1	154	-20.29	154	-20.29	228651	21	1830	14
miR529b	LOC_Os05g17360.1	167	-21.93	167	-21.93	228674	21	4251	3768
miR529b	LOC_Os05g17420.1	167	-21.93	167	-21.93	228680	21	3591	3108
miR529b	LOC_Os05g17870.1	161	-24.99	161	-24.99	228726	21	1248	1223
miR529b	LOC_Os05g18480.1	167	-21.93	167	-21.93	228787	21	3888	3582
miR529b	LOC_Os05g18560.1	178	-28.3	178	-28.3	228796	21	2391	1241
miR529b	LOC_Os05g19440.1	153	-20.34	153	-20.34	228888	21	711	261
miR529b	LOC_Os05g20370.1	161	-20.5	161	-20.5	228990	21	2280	2255
miR529b	LOC_Os05g22260.1	166	-21.47	166	-21.47	229086	21	2744	623
miR529b	LOC_Os05g22260.2	166	-21.47	166	-21.47	229087	21	2848	623
miR529b	LOC_Os05g22730.1	164	-21.23	164	-21.23	229138	21	731	657
miR529b	LOC_Os05g23840.1	168	-23.18	168	-23.18	229262	21	2474	52
miR529b	LOC_Os05g23880.1	158	-20.94	158	-20.94	229266	21	3815	703
miR529b	LOC_Os05g24340.1	162	-23.37	162	-23.37	229315	21	1236	806
miR529b	LOC_Os05g24680.1	171	-22.79	171	-22.79	229355	21	5664	4248
miR529b	LOC_Os05g25100.1	167	-23.13	167	-23.13	229400	21	3372	2571
miR529b	LOC_Os05g26210.1	150	-24.35	150	-24.35	229520	21	714	616
miR529b	LOC_Os05g27090.1	162	-20.9	162	-20.9	229605	21	663	558
miR529b	LOC_Os05g28120.1	156	-21.71	156	-21.71	229714	21	5565	3861
miR529b	LOC_Os05g28980.1	162	-20.93	162	-20.93	229806	21	1419	243
miR529b	LOC_Os05g28980.2	162	-20.93	162	-20.93	229807	21	1981	243
miR529b	LOC_Os05g28980.6	162	-20.93	162	-20.93	229809	21	2119	243
miR529b	LOC_Os05g29090.1	164	-23.33	164	-23.33	229823	21	1993	647
miR529b	LOC_Os05g29160.1	155	-20.72	155	-20.72	229830	21	552	398
miR529b	LOC_Os05g31530.1	165	-24.01	165	-24.01	230051	21	5545	1439
miR529b	LOC_Os05g32110.1	149	-20.22	149	-20.22	230108	21	2167	329
miR529b	LOC_Os05g32110.2	149	-20.22	149	-20.22	230109	21	2089	329
miR529b	LOC_Os05g32110.3	149	-20.22	149	-20.22	230110	21	1846	329

miR529b	LOC_Os05g32110.4	149	-20.22	149	-20.22	230111	21	2287	329
miR529b	LOC_Os05g32200.1	157	-20.09	157	-20.09	230120	21	846	26
miR529b	LOC_Os05g32700.1	156	-21.36	156	-21.36	230178	21	2751	245
miR529b	LOC_Os05g33370.1	156	-21.71	156	-21.71	230252	21	4437	3078
miR529b	LOC_Os05g33820.1	166	-23.46	166	-23.46	230308	21	2059	1651
miR529b	LOC_Os05g34610.1	173	-24.66	173	-24.66	230397	21	930	718
miR529b	LOC_Os05g34830.1	159	-20.81	159	-20.81	230424	21	1523	24
miR529b	LOC_Os05g34830.2	159	-20.81	159	-20.81	230425	21	1491	15
miR529b	LOC_Os05g34830.3	159	-20.81	159	-20.81	230426	21	1592	15
miR529b	LOC_Os05g35200.2	168	-24.98	168	-24.98	230469	21	2628	2257
miR529b	LOC_Os05g36290.1	161	-20.92	161	-20.92	230599	21	1883	353
miR529b	LOC_Os05g37230.1	155	-20.37	155	-20.37	230661	21	4110	3573
miR529b	LOC_Os05g37380.1	154	-20.23	154	-20.23	230675	21	5979	4558
miR529b	LOC_Os05g37460.1	158	-20.47	158	-20.47	230684	21	1040	340
miR529b	LOC_Os05g37830.1	160	-21.41	160	-21.41	230725	21	969	285
miR529b	LOC_Os05g37830.2	160	-21.41	160	-21.41	230726	21	985	285
miR529b	LOC_Os05g38260.1	148	-21.19	148	-21.19	230783	21	3978	2006
miR529b	LOC_Os05g38660.1	173	-26.24	173	-26.24	230842	21	669	494
miR529b	LOC_Os05g38730.1	171	-21.2	171	-21.2	230851	21	3489	1918
miR529b	LOC_Os05g38730.2	171	-21.2	171	-21.2	230852	21	3529	2000
miR529b	LOC_Os05g38870.1	163	-21.42	163	-21.42	230868	21	312	78
miR529b	LOC_Os05g40280.1	160	-22.3	160	-22.3	231038	21	1852	1797
miR529b	LOC_Os05g40285.1	333	-49.56	168	-24.98	231039	21	944	345 193
miR529b	LOC_Os05g40410.1	165	-22.78	165	-22.78	231054	21	1937	51
miR529b	LOC_Os05g40680.1	166	-20.05	166	-20.05	231083	21	1365	1334
miR529b	LOC_Os05g40720.1	154	-20.21	154	-20.21	231086	21	2004	1321
miR529b	LOC_Os05g40820.1	149	-20.66	149	-20.66	231101	21	990	549
miR529b	LOC_Os05g40820.2	149	-20.66	149	-20.66	231102	21	783	345
miR529b	LOC_Os05g40820.3	149	-20.66	149	-20.66	231103	21	982	549
miR529b	LOC_Os05g41160.1	158	-21.17	158	-21.17	231140	21	969	688
miR529b	LOC_Os05g41560.1	156	-21.71	156	-21.71	231189	21	4062	2358
miR529b	LOC_Os05g41770.1	174	-26.46	174	-26.46	231211	21	2208	631

miR529b	LOC_Os05g41910.1	162	-22.76	162	-22.76	231230	21	2034	940
miR529b	LOC_Os05g42300.1	149	-22.81	149	-22.81	231272	21	3027	1393
miR529b	LOC_Os05g43020.1	161	-25	161	-25	231300	21	2481	2456
miR529b	LOC_Os05g43040.1	168	-22.91	168	-22.91	231302	21	1313	38
miR529b	LOC_Os05g43210.1	157	-22.05	157	-22.05	231317	21	489	455
miR529b	LOC_Os05g43610.1	170	-23.43	170	-23.43	231366	21	3212	2120
miR529b	LOC_Os05g43910.1	158	-20.82	158	-20.82	231401	21	2396	83
miR529b	LOC_Os05g44340.1	159	-22.02	159	-22.02	231459	21	3264	3190
miR529b	LOC_Os05g44810.1	157	-22.95	157	-22.95	231517	21	1396	1016
miR529b	LOC_Os05g44810.2	157	-22.95	157	-22.95	231518	21	1353	1016
miR529b	LOC_Os05g44810.3	157	-22.95	157	-22.95	231519	21	1333	1016
miR529b	LOC_Os05g45550.1	163	-22.18	163	-22.18	231603	21	3390	2613
miR529b	LOC_Os05g45630.1	157	-21.69	157	-21.69	231611	21	4575	531
miR529b	LOC_Os05g45850.1	156	-20.44	156	-20.44	231640	21	5614	233
miR529b	LOC_Os05g46220.1	149	-20.32	149	-20.32	231684	21	2175	437
miR529b	LOC_Os05g46230.1	149	-20.32	149	-20.32	231685	21	2753	40
miR529b	LOC_Os05g46510.1	145	-20.34	145	-20.34	231728	21	2075	442
miR529b	LOC_Os05g46950.1	162	-21.2	162	-21.2	231777	21	1132	373
miR529b	LOC_Os05g48030.1	156	-20.74	156	-20.74	231847	21	3047	239
miR529b	LOC_Os05g48170.1	150	-21.79	150	-21.79	231866	21	3300	3234
miR529b	LOC_Os05g49520.1	170	-26.69	170	-26.69	232053	21	1824	680
miR529b	LOC_Os05g50310.1	168	-22.71	168	-22.71	232152	21	1096	998
miR529b	LOC_Os05g50390.1	156	-20.83	156	-20.83	232161	21	880	20
miR529b	LOC_Os05g51710.1	161	-21.04	161	-21.04	232341	21	1295	782
miR529b	LOC_Os05g51770.1	157	-21.69	157	-21.69	232349	21	3762	531
miR529b	LOC_Os05g52070.1	155	-20.37	155	-20.37	232376	21	5016	3702
miR529b	LOC_Os06g02110.1	151	-21.71	151	-21.71	232501	21	1705	1317
miR529b	LOC_Os06g02130.1	164	-21.42	164	-21.42	232505	21	3611	2459
miR529b	LOC_Os06g02130.2	164	-21.42	164	-21.42	232506	21	3929	2777
miR529b	LOC_Os06g02450.1	164	-20.48	164	-20.48	232544	21	608	214
miR529b	LOC_Os06g02560.1	160	-20.72	160	-20.72	232562	21	1703	189
miR529b	LOC_Os06g02560.2	160	-20.72	160	-20.72	232563	21	1540	101

miR529b	LOC_Os06g02560.3	160	-20.72	160	-20.72	232564	21	1703	259
miR529b	LOC_Os06g02630.1	156	-21.58	156	-21.58	232571	21	1877	1631
miR529b	LOC_Os06g02830.1	156	-20.16	156	-20.16	232591	21	2741	2567
miR529b	LOC_Os06g03000.1	155	-20.44	155	-20.44	232606	21	1386	542
miR529b	LOC_Os06g03690.1	168	-24.51	168	-24.51	232683	21	1226	76
miR529b	LOC_Os06g03690.2	168	-24.51	168	-24.51	232684	21	1431	61
miR529b	LOC_Os06g03710.1	161	-20.2	161	-20.2	232686	21	3102	419
miR529b	LOC_Os06g03850.1	170	-22.75	170	-22.75	232703	21	1360	1324
miR529b	LOC_Os06g03970.1	165	-27.36	165	-27.36	232717	21	3796	16
miR529b	LOC_Os06g04510.1	165	-24.08	165	-24.08	232783	21	1689	1619
miR529b	LOC_Os06g04510.2	165	-24.08	165	-24.08	232784	21	2415	2353
miR529b	LOC_Os06g04690.1	159	-21.82	159	-21.82	232809	21	2006	547
miR529b	LOC_Os06g04710.1	159	-21.82	159	-21.82	232811	21	2049	635
miR529b	LOC_Os06g05550.1	162	-20.34	162	-20.34	232912	21	1571	95
miR529b	LOC_Os06g05570.1	157	-22.05	157	-22.05	232914	21	444	416
miR529b	LOC_Os06g05660.1	166	-21.46	166	-21.46	232926	21	1590	38
miR529b	LOC_Os06g05660.2	166	-21.46	166	-21.46	232927	21	1576	36
miR529b	LOC_Os06g05660.3	166	-21.46	166	-21.46	232928	21	1575	38
miR529b	LOC_Os06g05660.4	166	-21.46	166	-21.46	232929	21	1549	36
miR529b	LOC_Os06g05660.5	166	-21.46	166	-21.46	232930	21	1654	36
miR529b	LOC_Os06g05860.1	166	-22.02	166	-22.02	232957	21	2043	437
miR529b	LOC_Os06g06160.1	164	-23.12	164	-23.12	233002	21	1612	26
miR529b	LOC_Os06g06160.4	164	-23.12	164	-23.12	233005	21	1551	13
miR529b	LOC_Os06g07070.1	168	-24.05	168	-24.05	233110	21	1651	19
miR529b	LOC_Os06g07520.1	157	-21.69	157	-21.69	233159	21	4575	531
miR529b	LOC_Os06g07760.1	167	-20.33	167	-20.33	233184	21	749	12
miR529b	LOC_Os06g08060.1	158	-26.3	158	-26.3	233216	21	1302	864
miR529b	LOC_Os06g08550.1	164	-20.48	164	-20.48	233268	21	2365	2246
miR529b	LOC_Os06g08660.1	169	-23.06	169	-23.06	233280	21	3557	1379
miR529b	LOC_Os06g09320.1	158	-20.21	158	-20.21	233365	21	1869	961
miR529b	LOC_Os06g09320.2	158	-20.21	158	-20.21	233366	21	2040	1132
miR529b	LOC_Os06g09380.1	165	-23.53	165	-23.53	233374	21	480	323

miR529b	LOC_Os06g10355.1	178	-27.77	178	-27.77	233488	21	1534	628
miR529b	LOC_Os06g10880.1	156	-20.23	156	-20.23	233542	21	1718	131
miR529b	LOC_Os06g10880.2	156	-20.23	156	-20.23	233543	21	1803	131
miR529b	LOC_Os06g10880.3	156	-20.23	156	-20.23	233544	21	1400	131
miR529b	LOC_Os06g10990.1	173	-23.61	173	-23.61	233556	21	1723	1159
miR529b	LOC_Os06g11010.1	161	-21.26	161	-21.26	233557	21	1419	897
miR529b	LOC_Os06g11320.1	169	-21.69	169	-21.69	233592	21	1833	705
miR529b	LOC_Os06g11320.2	169	-21.69	169	-21.69	233593	21	1776	788
miR529b	LOC_Os06g11440.1	156	-20.23	156	-20.23	233606	21	4515	2152
miR529b	LOC_Os06g11440.2	322	-41.3	166	-21.07	233607	21	3962	922 1599
miR529b	LOC_Os06g11440.3	156	-20.23	156	-20.23	233608	21	2702	927
miR529b	LOC_Os06g11830.1	167	-21.97	167	-21.97	233650	21	2640	2318
miR529b	LOC_Os06g12310.1	157	-23.82	157	-23.82	233712	21	1544	243
miR529b	LOC_Os06g13540.1	152	-20.96	152	-20.96	233847	21	4353	2261
miR529b	LOC_Os06g13610.1	487	-70.21	168	-24.51	233858	21	732	55 27 5
miR529b	LOC_Os06g13630.1	154	-21.82	154	-21.82	233860	21	2841	231
miR529b	LOC_Os06g13820.1	165	-24.08	165	-24.08	233882	21	3391	21
miR529b	LOC_Os06g13820.2	165	-24.08	165	-24.08	233883	21	3313	21
miR529b	LOC_Os06g13940.1	156	-24.16	156	-24.16	233895	21	807	719
miR529b	LOC_Os06g14600.1	157	-22.3	157	-22.3	233973	21	2502	2477
miR529b	LOC_Os06g14690.1	157	-22.05	157	-22.05	233982	21	570	352
miR529b	LOC_Os06g16080.1	169	-23.84	169	-23.84	234119	21	2706	1715
miR529b	LOC_Os06g16850.1	154	-20.29	154	-20.29	234199	21	1863	14
miR529b	LOC_Os06g17170.1	154	-20.29	154	-20.29	234233	21	450	14
miR529b	LOC_Os06g17180.1	154	-20.29	154	-20.29	234234	21	375	14
miR529b	LOC_Os06g17280.1	164	-20.21	164	-20.21	234244	21	2602	2519
miR529b	LOC_Os06g17510.1	152	-20.23	152	-20.23	234267	21	546	71
miR529b	LOC_Os06g17770.1	154	-20.29	154	-20.29	234292	21	375	14
miR529b	LOC_Os06g20860.1	160	-21.14	160	-21.14	234558	21	2746	425
miR529b	LOC_Os06g21610.1	156	-21.71	156	-21.71	234640	21	4341	2829
miR529b	LOC_Os06g23080.1	161	-20.5	161	-20.5	234804	21	1998	1973
miR529b	LOC_Os06g23160.1	163	-20.13	163	-20.13	234811	21	1872	1091

miR529b	LOC_Os06g23730.1	149	-22.94	149	-22.94	234870	21	1995	704
miR529b	LOC_Os06g23890.1	153	-22.37	153	-22.37	234889	21	2592	1949
miR529b	LOC_Os06g25690.1	153	-20.34	153	-20.34	235065	21	891	852
miR529b	LOC_Os06g26030.1	175	-23.41	175	-23.41	235099	21	1557	426
miR529b	LOC_Os06g26320.1	152	-20.75	152	-20.75	235126	21	718	678
miR529b	LOC_Os06g27430.1	167	-25.04	167	-25.04	235139	21	456	134
miR529b	LOC_Os06g28850.1	157	-20.97	157	-20.97	235288	21	1896	379
miR529b	LOC_Os06g28990.1	156	-20.23	156	-20.23	235304	21	4106	891
miR529b	LOC_Os06g30520.1	156	-24.57	156	-24.57	235463	21	3678	870
miR529b	LOC_Os06g30800.1	164	-20.21	164	-20.21	235494	21	441	114
miR529b	LOC_Os06g30894.1	148	-20.3	148	-20.3	235506	21	4124	936
miR529b	LOC_Os06g30894.2	148	-20.3	148	-20.3	235507	21	4051	936
miR529b	LOC_Os06g30930.1	158	-24.5	158	-24.5	235511	21	1374	280
miR529b	LOC_Os06g31650.1	157	-22.29	157	-22.29	235580	21	648	623
miR529b	LOC_Os06g31660.1	165	-22.01	165	-22.01	235581	21	843	779
miR529b	LOC_Os06g32270.1	178	-28.3	178	-28.3	235639	21	2661	1517
miR529b	LOC_Os06g32860.1	162	-23.99	162	-23.99	235694	21	2534	7
miR529b	LOC_Os06g32880.1	161	-25	161	-25	235697	21	2436	2411
miR529b	LOC_Os06g33900.1	161	-25	161	-25	235798	21	2436	2411
miR529b	LOC_Os06g35050.1	154	-20.09	154	-20.09	235912	21	1695	1242
miR529b	LOC_Os06g35140.1	161	-22.38	161	-22.38	235921	21	2094	1936
miR529b	LOC_Os06g35920.1	163	-20.9	163	-20.9	236000	21	693	5
miR529b	LOC_Os06g36500.1	156	-20.87	156	-20.87	236064	21	3756	78
miR529b	LOC_Os06g36660.1	156	-20.76	156	-20.76	236080	21	1083	571
miR529b	LOC_Os06g36760.1	158	-20.12	158	-20.12	236090	21	483	29
miR529b	LOC_Os06g37560.1	170	-24.41	170	-24.41	236175	21	3071	40
miR529b	LOC_Os06g37810.1	158	-22.84	158	-22.84	236201	21	657	49
miR529b	LOC_Os06g37890.1	156	-21.71	156	-21.71	236209	21	3024	2562
miR529b	LOC_Os06g39515.1	168	-23.18	168	-23.18	236373	21	656	4
miR529b	LOC_Os06g39900.1	166	-22.72	166	-22.72	236426	21	1683	1642
miR529b	LOC_Os06g39900.2	166	-22.72	166	-22.72	236427	21	2808	2767
miR529b	LOC_Os06g39900.3	166	-22.72	166	-22.72	236428	21	2709	2668

miR529b	LOC_Os06g40680.1	152	-21.64	152	-21.64	236519	21	3486	1571
miR529b	LOC_Os06g41540.1	168	-22.91	168	-22.91	236613	21	685	34
miR529b	LOC_Os06g41570.1	158	-20.42	158	-20.42	236616	21	483	154
miR529b	LOC_Os06g42030.1	319	-44.72	170	-23.94	236674	21	5864	2996 2797
miR529b	LOC_Os06g42400.1	156	-21.71	156	-21.71	236714	21	4395	3096
miR529b	LOC_Os06g42950.1	154	-20.29	154	-20.29	236778	21	375	14
miR529b	LOC_Os06g43330.1	166	-21.94	166	-21.94	236816	21	2106	123
miR529b	LOC_Os06g43560.1	161	-23.39	161	-23.39	236840	21	2963	2269
miR529b	LOC_Os06g43600.2	155	-21.55	155	-21.55	236847	21	1657	922
miR529b	LOC_Os06g43620.1	165	-24.08	165	-24.08	236849	21	1689	49
miR529b	LOC_Os06g43690.1	169	-24.83	169	-24.83	236860	21	3134	2834
miR529b	LOC_Os06g43770.1	160	-22.13	160	-22.13	236868	21	963	887
miR529b	LOC_Os06g44030.1	159	-20.31	159	-20.31	236899	21	2509	1769
miR529b	LOC_Os06g44030.2	159	-20.31	159	-20.31	236900	21	2473	1769
miR529b	LOC_Os06g44034.1	175	-26.46	175	-26.46	236901	21	2020	639
miR529b	LOC_Os06g44230.1	162	-20.46	162	-20.46	236926	21	3746	37
miR529b	LOC_Os06g44880.1	167	-20.8	167	-20.8	236993	21	2646	1163
miR529b	LOC_Os06g45030.1	156	-23.02	156	-23.02	237011	21	3229	3162
miR529b	LOC_Os06g45090.1	153	-25.04	153	-25.04	237019	21	1148	254
miR529b	LOC_Os06g45090.2	153	-25.04	153	-25.04	237020	21	2200	185
miR529b	LOC_Os06g45100.1	157	-21.6	157	-21.6	237021	21	1923	193
miR529b	LOC_Os06g45350.1	165	-25.29	165	-25.29	237057	21	2140	67
miR529b	LOC_Os06g45530.1	157	-22.29	157	-22.29	237081	21	2412	2387
miR529b	LOC_Os06g45580.1	154	-20.79	154	-20.79	237086	21	975	93
miR529b	LOC_Os06g45630.1	144	-21.28	144	-21.28	237091	21	3811	1542
miR529b	LOC_Os06g45860.1	156	-20.19	156	-20.19	237122	21	3194	2172
miR529b	LOC_Os06g45900.1	165	-20.15	165	-20.15	237126	21	1647	233
miR529b	LOC_Os06g46140.1	170	-26.96	170	-26.96	237154	21	1534	507
miR529b	LOC_Os06g46270.1	333	-44.62	168	-23.79	237164	21	1470	114 25
miR529b	LOC_Os06g46550.1	164	-20.48	164	-20.48	237200	21	627	46
miR529b	LOC_Os06g46560.1	154	-20.5	154	-20.5	237201	21	1934	51
miR529b	LOC_Os06g47294.1	155	-21.12	155	-21.12	237297	21	2283	1030

miR529b	LOC_Os06g47590.1	158	-20.93	158	-20.93	237336	21	1019	802
miR529b	LOC_Os06g47840.1	165	-20.36	165	-20.36	237361	21	1684	25
miR529b	LOC_Os06g47900.1	163	-22.72	163	-22.72	237369	21	1456	987
miR529b	LOC_Os06g47900.3	163	-22.72	163	-22.72	237371	21	1649	934
miR529b	LOC_Os06g47900.4	163	-22.72	163	-22.72	237372	21	1539	934
miR529b	LOC_Os06g48030.1	156	-21.57	156	-21.57	237385	21	2170	1467
miR529b	LOC_Os06g48030.3	156	-21.57	156	-21.57	237386	21	2644	1941
miR529b	LOC_Os06g48040.1	168	-24.51	168	-24.51	237387	21	2664	2618
miR529b	LOC_Os06g48590.1	168	-22.71	168	-22.71	237451	21	1875	152
miR529b	LOC_Os06g49060.1	161	-21.33	161	-21.33	237514	21	2143	89
miR529b	LOC_Os06g49150.1	156	-20.46	156	-20.46	237528	21	1543	73
miR529b	LOC_Os06g49310.1	152	-21.12	152	-21.12	237549	21	3960	2279
miR529b	LOC_Os06g49320.1	152	-21.12	152	-21.12	237551	21	3711	420
miR529b	LOC_Os06g49510.1	157	-22.52	157	-22.52	237581	21	2263	1872
miR529b	LOC_Os06g49620.1	169	-26.47	169	-26.47	237593	21	390	159
miR529b	LOC_Os06g50030.1	168	-23.03	168	-23.03	237650	21	2327	2093
miR529b	LOC_Os06g50170.1	168	-24.98	168	-24.98	237665	21	1798	1745
miR529b	LOC_Os06g50210.1	169	-23.27	169	-23.27	237669	21	1749	389
miR529b	LOC_Os06g50400.1	174	-24.21	174	-24.21	237692	21	1106	921
miR529b	LOC_Os06g50880.1	169	-23.27	169	-23.27	237743	21	1800	267
miR529b	LOC_Os06g50930.1	161	-20.91	161	-20.91	237750	21	1287	914
miR529b	LOC_Os06g50930.2	161	-20.91	161	-20.91	237751	21	1447	1074
miR529b	LOC_Os06g51310.1	159	-24.07	159	-24.07	237804	21	3759	70
miR529b	LOC_Os06g51480.2	168	-22.71	168	-22.71	237832	21	1697	400
miR529b	LOC_Os06g51510.1	164	-23.27	164	-23.27	237835	21	1372	680
miR529b	LOC_Os06g51510.2	164	-23.27	164	-23.27	237836	21	1478	680
miR529b	LOC_Os07g01870.1	155	-20.29	155	-20.29	237950	21	1298	673
miR529b	LOC_Os07g01870.2	155	-20.29	155	-20.29	237951	21	1282	673
miR529b	LOC_Os07g02470.1	148	-21.14	148	-21.14	238014	21	1410	592
miR529b	LOC_Os07g03230.2	165	-21.81	165	-21.81	238105	21	3672	137
miR529b	LOC_Os07g03820.1	156	-20.85	156	-20.85	238158	21	2255	306
miR529b	LOC_Os07g04090.1	161	-23.5	161	-23.5	238184	21	1398	649

miR529b	LOC_Os07g04480.1	153	-20.31	153	-20.31	238225	21	3346	2118
miR529b	LOC_Os07g04530.1	158	-22.24	158	-22.24	238230	21	5617	4107
miR529b	LOC_Os07g04820.1	169	-25.08	169	-25.08	238260	21	1611	199
miR529b	LOC_Os07g05140.1	156	-21.71	156	-21.71	238296	21	3708	2004
miR529b	LOC_Os07g05540.1	145	-20.29	145	-20.29	238340	21	5325	4532
miR529b	LOC_Os07g05560.1	169	-23.98	169	-23.98	238342	21	1779	219
miR529b	LOC_Os07g05720.1	156	-20.16	156	-20.16	238363	21	2109	31
miR529b	LOC_Os07g06130.1	166	-21.51	166	-21.51	238404	21	4826	4536
miR529b	LOC_Os07g06130.2	166	-21.51	166	-21.51	238405	21	4831	4541
miR529b	LOC_Os07g06130.3	166	-21.51	166	-21.51	238406	21	4791	4501
miR529b	LOC_Os07g06166.1	162	-20.27	162	-20.27	238407	21	522	65
miR529b	LOC_Os07g06250.1	162	-20.27	162	-20.27	238415	21	522	65
miR529b	LOC_Os07g06300.1	166	-21.51	166	-21.51	238417	21	4539	4474
miR529b	LOC_Os07g06345.1	162	-20.27	162	-20.27	238421	21	522	65
miR529b	LOC_Os07g06620.1	165	-24.22	165	-24.22	238454	21	1380	938
miR529b	LOC_Os07g06620.2	165	-24.22	165	-24.22	238455	21	1345	903
miR529b	LOC_Os07g06620.4	165	-24.22	165	-24.22	238457	21	1374	932
miR529b	LOC_Os07g07010.1	167	-22.74	167	-22.74	238503	21	1974	1560
miR529b	LOC_Os07g07350.1	173	-24.45	173	-24.45	238541	21	908	825
miR529b	LOC_Os07g07350.3	173	-24.45	173	-24.45	238543	21	914	829
miR529b	LOC_Os07g07950.1	165	-24.08	165	-24.08	238609	21	2916	2768
miR529b	LOC_Os07g07950.2	165	-24.08	165	-24.08	238610	21	2968	2820
miR529b	LOC_Os07g09340.1	170	-28.27	170	-28.27	238759	21	3434	114
miR529b	LOC_Os07g10030.1	160	-20.09	160	-20.09	238831	21	1738	1688
miR529b	LOC_Os07g10560.1	157	-21.21	157	-21.21	238891	21	1110	27
miR529b	LOC_Os07g11010.2	157	-23.14	157	-23.14	238951	21	2383	7
miR529b	LOC_Os07g11110.1	143	-20.53	143	-20.53	238962	21	1562	1285
miR529b	LOC_Os07g11190.1	157	-22.72	157	-22.72	238971	21	2193	715
miR529b	LOC_Os07g11640.1	167	-21.93	167	-21.93	239018	21	2400	1917
miR529b	LOC_Os07g11680.1	160	-20.98	160	-20.98	239021	21	5976	3984
miR529b	LOC_Os07g11790.1	165	-23.44	165	-23.44	239029	21	2016	1909
miR529b	LOC_Os07g12490.1	156	-21.23	156	-21.23	239099	21	1480	46

miR529b	LOC_Os07g12610.1	156	-20.66	156	-20.66	239115	21	1996	96
miR529b	LOC_Os07g13700.1	166	-21.94	166	-21.94	239223	21	2241	204
miR529b	LOC_Os07g14150.5	159	-22.12	159	-22.12	239271	21	2180	120
miR529b	LOC_Os07g14580.1	168	-23.18	168	-23.18	239327	21	1671	34
miR529b	LOC_Os07g14920.1	166	-21.13	166	-21.13	239361	21	2602	1848
miR529b	LOC_Os07g15340.1	167	-21.97	167	-21.97	239403	21	2694	2318
miR529b	LOC_Os07g16420.1	155	-20.37	155	-20.37	239505	21	2346	142
miR529b	LOC_Os07g16490.1	157	-22.29	157	-22.29	239512	21	2331	2306
miR529b	LOC_Os07g16800.1	155	-20.37	155	-20.37	239543	21	2265	142
miR529b	LOC_Os07g17120.1	169	-21.83	169	-21.83	239578	21	954	644
miR529b	LOC_Os07g17810.1	167	-23.13	167	-23.13	239651	21	2151	1170
miR529b	LOC_Os07g18120.1	159	-20.5	159	-20.5	239683	21	4560	1754
miR529b	LOC_Os07g18154.1	159	-20.5	159	-20.5	239686	21	2953	1770
miR529b	LOC_Os07g18154.2	159	-20.5	159	-20.5	239687	21	3082	1914
miR529b	LOC_Os07g19520.1	168	-24.26	168	-24.26	239812	21	495	30
miR529b	LOC_Os07g20340.1	155	-20.98	155	-20.98	239846	21	1386	339
miR529b	LOC_Os07g20420.1	169	-24.02	169	-24.02	239853	21	2401	508
miR529b	LOC_Os07g20430.1	156	-21.71	156	-21.71	239854	21	5045	3213
miR529b	LOC_Os07g20700.1	163	-21.02	163	-21.02	239882	21	231	146
miR529b	LOC_Os07g21980.1	149	-20.36	149	-20.36	239910	21	1605	178
miR529b	LOC_Os07g22430.1	145	-20.29	145	-20.29	239955	21	5103	4388
miR529b	LOC_Os07g22600.1	158	-20.03	158	-20.03	239979	21	1558	1499
miR529b	LOC_Os07g22600.2	158	-20.03	158	-20.03	239980	21	1546	1487
miR529b	LOC_Os07g22860.1	158	-20.32	158	-20.32	240013	21	2865	1788
miR529b	LOC_Os07g22950.1	163	-24.82	163	-24.82	240024	21	978	601
miR529b	LOC_Os07g22950.2	163	-24.82	163	-24.82	240025	21	918	541
miR529b	LOC_Os07g22950.3	163	-24.82	163	-24.82	240026	21	944	567
miR529b	LOC_Os07g23130.1	157	-20.24	157	-20.24	240046	21	366	215
miR529b	LOC_Os07g23550.1	168	-23.18	168	-23.18	240085	21	1220	100
miR529b	LOC_Os07g23550.2	168	-23.18	168	-23.18	240086	21	1111	100
miR529b	LOC_Os07g23570.1	155	-20.34	155	-20.34	240088	21	2009	1693
miR529b	LOC_Os07g23770.1	168	-22.71	168	-22.71	240108	21	630	193

miR529b	LOC_Os07g26210.1	167	-20.52	167	-20.52	240363	21	4649	4474
miR529b	LOC_Os07g26410.1	162	-24.3	162	-24.3	240384	21	495	151
miR529b	LOC_Os07g27680.1	162	-20.9	162	-20.9	240522	21	225	69
miR529b	LOC_Os07g29300.1	156	-20.37	156	-20.37	240688	21	747	41
miR529b	LOC_Os07g29410.1	160	-21.95	160	-21.95	240705	21	1449	101
miR529b	LOC_Os07g29410.2	160	-21.95	160	-21.95	240706	21	1357	101
miR529b	LOC_Os07g29600.2	159	-20.97	159	-20.97	240729	21	1432	544
miR529b	LOC_Os07g29630.1	166	-24.88	166	-24.88	240732	21	1006	215
miR529b	LOC_Os07g32430.1	161	-24.76	161	-24.76	241030	21	3954	67
miR529b	LOC_Os07g32510.1	165	-20.96	165	-20.96	241036	21	2583	2464
miR529b	LOC_Os07g35310.1	154	-20.51	154	-20.51	241336	21	2562	1280
miR529b	LOC_Os07g35310.2	154	-20.51	154	-20.51	241337	21	2663	1381
miR529b	LOC_Os07g35550.1	157	-22.05	157	-22.05	241367	21	417	338
miR529b	LOC_Os07g35740.1	164	-23.32	164	-23.32	241393	21	2189	818
miR529b	LOC_Os07g36410.1	168	-23.18	168	-23.18	241470	21	1623	1007
miR529b	LOC_Os07g36465.1	170	-23.87	170	-23.87	241475	21	616	18
miR529b	LOC_Os07g37250.1	159	-21.19	159	-21.19	241562	21	1347	412
miR529b	LOC_Os07g37900.1	161	-21.18	161	-21.18	241634	21	6156	5660
miR529b	LOC_Os07g38610.1	174	-25.04	174	-25.04	241715	21	1828	1620
miR529b	LOC_Os07g38760.1	330	-47.7	165	-27.35	241731	21	3693	966 1599
miR529b	LOC_Os07g39090.1	165	-25.78	165	-25.78	241770	21	3036	235
miR529b	LOC_Os07g40520.1	152	-21.42	152	-21.42	241931	21	1914	580
miR529b	LOC_Os07g40680.1	166	-21.94	166	-21.94	241949	21	936	204
miR529b	LOC_Os07g40940.1	156	-22.53	156	-22.53	241980	21	1563	135
miR529b	LOC_Os07g40974.1	168	-24.98	168	-24.98	241982	21	1237	1176
miR529b	LOC_Os07g40974.2	168	-24.98	168	-24.98	241983	21	1478	1417
miR529b	LOC_Os07g41070.1	158	-21.04	158	-21.04	241991	21	1676	1595
miR529b	LOC_Os07g42730.1	158	-20.18	158	-20.18	242189	21	1207	1105
miR529b	LOC_Os07g43070.1	163	-20.13	163	-20.13	242238	21	1432	1360
miR529b	LOC_Os07g43510.1	171	-22.8	171	-22.8	242287	21	993	379
miR529b	LOC_Os07g43910.1	174	-25.06	174	-25.06	242327	21	363	53
miR529b	LOC_Os07g44180.1	165	-21.65	165	-21.65	242359	21	941	18

miR529b	LOC_Os07g45064.1	157	-20.21	157	-20.21	242457	21	3757	3726
miR529b	LOC_Os07g45090.1	165	-20.83	165	-20.83	242461	21	2424	72
miR529b	LOC_Os07g45660.1	157	-22.3	157	-22.3	242516	21	2481	2456
miR529b	LOC_Os07g45710.1	157	-22.29	157	-22.29	242521	21	2346	2321
miR529b	LOC_Os07g46920.1	156	-20.37	156	-20.37	242673	21	1246	1194
miR529b	LOC_Os07g47100.1	158	-21	158	-21	242700	21	2265	136
miR529b	LOC_Os07g47100.2	158	-21	158	-21	242701	21	2394	265
miR529b	LOC_Os07g47100.3	158	-21	158	-21	242702	21	1820	126
miR529b	LOC_Os07g47230.1	160	-20.26	160	-20.26	242720	21	2022	888
miR529b	LOC_Os07g47800.1	159	-21.27	159	-21.27	242806	21	2085	681
miR529b	LOC_Os07g48080.1	165	-22.06	165	-22.06	242837	21	1966	18
miR529b	LOC_Os07g48410.1	162	-23.19	162	-23.19	242877	21	3468	678
miR529b	LOC_Os07g48410.2	162	-23.19	162	-23.19	242878	21	3623	678
miR529b	LOC_Os07g49500.1	161	-22	161	-22	243027	21	4545	3471
miR529b	LOC_Os08g01740.1	165	-26.33	165	-26.33	243127	21	2297	16
miR529b	LOC_Os08g01740.2	165	-26.33	165	-26.33	243128	21	2225	16
miR529b	LOC_Os08g01750.3	161	-22.01	161	-22.01	243131	21	2026	1960
miR529b	LOC_Os08g01910.1	152	-23	152	-23	243149	21	998	406
miR529b	LOC_Os08g02570.1	144	-21.45	144	-21.45	243220	21	1179	39
miR529b	LOC_Os08g03360.1	159	-21.76	159	-21.76	243313	21	1949	885
miR529b	LOC_Os08g04440.1	162	-21.25	162	-21.25	243440	21	1080	1056
miR529b	LOC_Os08g04920.1	178	-27.88	178	-27.88	243495	21	2389	2084
miR529b	LOC_Os08g04920.2	178	-27.88	178	-27.88	243496	21	2265	1960
miR529b	LOC_Os08g04920.3	178	-27.88	178	-27.88	243497	21	1856	1551
miR529b	LOC_Os08g04920.4	178	-27.88	178	-27.88	243498	21	2201	1896
miR529b	LOC_Os08g04920.5	178	-27.88	178	-27.88	243499	21	2314	2009
miR529b	LOC_Os08g04920.6	178	-27.88	178	-27.88	243500	21	2174	1869
miR529b	LOC_Os08g04920.7	178	-27.88	178	-27.88	243501	21	2211	1906
miR529b	LOC_Os08g04920.8	178	-27.88	178	-27.88	243502	21	2337	2032
miR529b	LOC_Os08g05130.1	157	-22.29	157	-22.29	243524	21	2484	2459
miR529b	LOC_Os08g05440.1	166	-25.57	166	-25.57	243554	21	4497	1133
miR529b	LOC_Os08g05910.1	166	-25.26	166	-25.26	243605	21	2356	2254

miR529b	LOC_Os08g06010.1	165	-20.36	165	-20.36	243616	21	2316	77
miR529b	LOC_Os08g06440.1	179	-26.82	179	-26.82	243673	21	732	377
miR529b	LOC_Os08g06460.1	162	-20.81	162	-20.81	243675	21	1692	1468
miR529b	LOC_Os08g06460.2	162	-20.81	162	-20.81	243676	21	1696	1475
miR529b	LOC_Os08g06470.1	156	-22.3	156	-22.3	243677	21	2496	1546
miR529b	LOC_Os08g06470.2	156	-22.3	156	-22.3	243678	21	2902	1950
miR529b	LOC_Os08g06600.1	169	-23.26	169	-23.26	243695	21	1750	896
miR529b	LOC_Os08g06640.1	168	-23.18	168	-23.18	243700	21	2001	1
miR529b	LOC_Os08g07300.1	170	-26.12	170	-26.12	243760	21	1323	3
miR529b	LOC_Os08g07300.2	170	-26.12	170	-26.12	243761	21	1061	3
miR529b	LOC_Os08g07400.1	153	-20.08	153	-20.08	243770	21	2582	233
miR529b	LOC_Os08g07460.1	173	-24.66	173	-24.66	243776	21	1971	499
miR529b	LOC_Os08g08110.1	162	-20.56	162	-20.56	243844	21	1953	716
miR529b	LOC_Os08g08540.1	154	-20.29	154	-20.29	243888	21	1758	14
miR529b	LOC_Os08g08780.1	161	-25	161	-25	243920	21	2436	2411
miR529b	LOC_Os08g08830.1	162	-21.08	162	-21.08	243926	21	3782	910
miR529b	LOC_Os08g08840.1	173	-26.11	173	-26.11	243927	21	1819	913
miR529b	LOC_Os08g08840.2	173	-26.11	173	-26.11	243928	21	1816	910
miR529b	LOC_Os08g11830.1	167	-21.93	167	-21.93	244258	21	1987	1104
miR529b	LOC_Os08g11850.1	167	-21.93	167	-21.93	244260	21	4332	3849
miR529b	LOC_Os08g12400.1	149	-20.5	149	-20.5	244312	21	2466	2441
miR529b	LOC_Os08g12910.1	166	-21.94	166	-21.94	244372	21	2208	204
miR529b	LOC_Os08g13350.1	164	-22.36	164	-22.36	244416	21	2261	1948
miR529b	LOC_Os08g13350.2	164	-22.36	164	-22.36	244417	21	2540	2227
miR529b	LOC_Os08g13760.1	161	-25	161	-25	244461	21	2481	2456
miR529b	LOC_Os08g14195.1	161	-21.93	161	-21.93	244503	21	2464	159
miR529b	LOC_Os08g14610.1	165	-21.5	165	-21.5	244549	21	3447	2524
miR529b	LOC_Os08g14940.1	158	-22.07	158	-22.07	244588	21	2904	24
miR529b	LOC_Os08g14950.1	162	-23.16	162	-23.16	244589	21	3169	16
miR529b	LOC_Os08g14990.1	160	-20.85	160	-20.85	244593	21	3390	3224
miR529b	LOC_Os08g17130.1	160	-21.76	160	-21.76	244835	21	3447	3421
miR529b	LOC_Os08g17390.1	150	-20.22	150	-20.22	244857	21	2443	1256

miR529b	LOC_Os08g17500.1	161	-22.02	161	-22.02	244868	21	1420	84
miR529b	LOC_Os08g19374.1	165	-22.3	165	-22.3	245015	21	2232	352
miR529b	LOC_Os08g19374.2	165	-22.3	165	-22.3	245016	21	1725	352
miR529b	LOC_Os08g19374.3	165	-22.3	165	-22.3	245017	21	1033	352
miR529b	LOC_Os08g19374.4	165	-22.3	165	-22.3	245018	21	1130	352
miR529b	LOC_Os08g19530.1	153	-20.34	153	-20.34	245033	21	1464	723
miR529b	LOC_Os08g19610.1	165	-22.01	165	-22.01	245041	21	1468	421
miR529b	LOC_Os08g19610.2	165	-22.01	165	-22.01	245042	21	2182	1135
miR529b	LOC_Os08g19610.3	165	-22.01	165	-22.01	245043	21	1568	521
miR529b	LOC_Os08g19610.4	165	-22.01	165	-22.01	245044	21	3303	1135
miR529b	LOC_Os08g19694.1	153	-20.85	153	-20.85	245053	21	3636	1526
miR529b	LOC_Os08g19860.1	150	-20.94	150	-20.94	245070	21	1854	445
miR529b	LOC_Os08g20580.1	165	-24	165	-24	245139	21	675	105
miR529b	LOC_Os08g21110.1	159	-23.12	159	-23.12	245179	21	6117	143
miR529b	LOC_Os08g21541.1	168	-22.44	168	-22.44	245218	21	4751	476
miR529b	LOC_Os08g22180.1	153	-20.33	153	-20.33	245285	21	4056	240
miR529b	LOC_Os08g22200.1	152	-20.24	152	-20.24	245287	21	1088	381
miR529b	LOC_Os08g22284.1	168	-23.18	168	-23.18	245292	21	2502	2460
miR529b	LOC_Os08g22630.1	161	-25	161	-25	245322	21	2055	2030
miR529b	LOC_Os08g22760.1	154	-20.71	154	-20.71	245333	21	5106	3643
miR529b	LOC_Os08g25330.1	157	-21.69	157	-21.69	245596	21	4575	531
miR529b	LOC_Os08g25440.1	156	-20.02	156	-20.02	245608	21	1302	1273
miR529b	LOC_Os08g25700.1	153	-20.25	153	-20.25	245636	21	1747	1081
miR529b	LOC_Os08g26510.1	163	-20.86	163	-20.86	245718	21	1203	488
miR529b	LOC_Os08g27570.1	154	-21	154	-21	245830	21	2562	1324
miR529b	LOC_Os08g27790.1	322	-42.01	166	-21.94	245855	21	1926	204 295
miR529b	LOC_Os08g28080.1	170	-23.08	170	-23.08	245885	21	2514	1139
miR529b	LOC_Os08g28310.1	169	-23.84	169	-23.84	245912	21	1848	1535
miR529b	LOC_Os08g28319.1	154	-20.29	154	-20.29	245913	21	1464	14
miR529b	LOC_Os08g29615.1	161	-20.08	161	-20.08	246046	21	2608	45
miR529b	LOC_Os08g29700.1	145	-20.29	145	-20.29	246054	21	1314	695
miR529b	LOC_Os08g30770.1	161	-21.98	161	-21.98	246163	21	3444	1092

miR529b	LOC_Os08g31630.1	168	-22.91	168	-22.91	246254	21	2698	62
miR529b	LOC_Os08g31630.2	168	-22.91	168	-22.91	246255	21	2891	53
miR529b	LOC_Os08g31940.1	322	-46.17	164	-23.37	246292	21	1747	1247 29
miR529b	LOC_Os08g32490.1	163	-22.03	163	-22.03	246350	21	360	254
miR529b	LOC_Os08g33082.1	168	-23.18	168	-23.18	246419	21	3200	80
miR529b	LOC_Os08g34070.1	165	-20.36	165	-20.36	246536	21	1470	7
miR529b	LOC_Os08g34070.2	165	-20.36	165	-20.36	246537	21	1389	7
miR529b	LOC_Os08g34320.1	161	-21.02	161	-21.02	246564	21	2419	1138
miR529b	LOC_Os08g34660.1	165	-22.28	165	-22.28	246600	21	780	47
miR529b	LOC_Os08g35310.1	162	-21.37	162	-21.37	246679	21	1041	83
miR529b	LOC_Os08g35990.1	167	-23.67	167	-23.67	246753	21	381	6
miR529b	LOC_Os08g36110.1	161	-20.09	161	-20.09	246765	21	1244	74
miR529b	LOC_Os08g37470.1	153	-20.31	153	-20.31	246918	21	1089	34
miR529b	LOC_Os08g37540.1	157	-22.29	157	-22.29	246926	21	2466	2441
miR529b	LOC_Os08g37710.1	149	-20.42	149	-20.42	246949	21	1308	1234
miR529b	LOC_Os08g37780.1	171	-23.02	171	-23.02	246957	21	2781	1965
miR529b	LOC_Os08g37780.2	171	-23.02	171	-23.02	246958	21	2776	1965
miR529b	LOC_Os08g37920.1	157	-23.61	157	-23.61	246977	21	3230	2964
miR529b	LOC_Os08g37920.2	157	-23.61	157	-23.61	246978	21	3101	2835
miR529b	LOC_Os08g38040.1	156	-21.71	156	-21.71	246994	21	2964	2526
miR529b	LOC_Os08g38100.1	168	-24.51	168	-24.51	247002	21	1113	60
miR529b	LOC_Os08g38560.1	168	-24.98	168	-24.98	247057	21	3625	52
miR529b	LOC_Os08g39310.1	162	-21.7	162	-21.7	247158	21	1862	1500
miR529b	LOC_Os08g39760.1	145	-20.29	145	-20.29	247208	21	5856	5063
miR529b	LOC_Os08g39980.1	164	-23.12	164	-23.12	247237	21	2545	2508
miR529b	LOC_Os08g40290.1	311	-43.61	166	-21.97	247272	21	3791	2710 2808
miR529b	LOC_Os08g40310.1	166	-20.05	166	-20.05	247273	21	3132	3101
miR529b	LOC_Os08g40420.1	161	-20.05	161	-20.05	247282	21	3593	2378
miR529b	LOC_Os08g40420.2	161	-20.05	161	-20.05	247283	21	3744	2529
miR529b	LOC_Os08g40430.1	161	-20.05	161	-20.05	247284	21	2237	877
miR529b	LOC_Os08g40480.1	151	-20.15	151	-20.15	247289	21	1133	948
miR529b	LOC_Os08g40620.1	165	-21.81	165	-21.81	247311	21	3411	85

miR529b	LOC_Os08g40620.2	165	-21.81	165	-21.81	247312	21	3483	85
miR529b	LOC_Os08g40890.1	165	-27.42	165	-27.42	247347	21	3193	1122
miR529b	LOC_Os08g40930.1	157	-21.1	157	-21.1	247353	21	3772	693
miR529b	LOC_Os08g41620.1	166	-20.01	166	-20.01	247439	21	2253	704
miR529b	LOC_Os08g41900.1	159	-20.35	159	-20.35	247468	21	3189	2038
miR529b	LOC_Os08g41940.1	182	-32.61	182	-32.61	247472	21	1708	1049
miR529b	LOC_Os08g42050.1	165	-21.76	165	-21.76	247485	21	1106	655
miR529b	LOC_Os08g43010.1	162	-23.85	162	-23.85	247608	21	3990	2398
miR529b	LOC_Os08g43130.1	153	-20.36	153	-20.36	247623	21	4634	1362
miR529b	LOC_Os08g43710.1	161	-20.19	161	-20.19	247692	21	957	883
miR529b	LOC_Os08g44760.1	171	-21.15	171	-21.15	247818	21	2520	196
miR529b	LOC_Os08g44760.2	171	-21.15	171	-21.15	247819	21	2505	184
miR529b	LOC_Os09g01100.1	167	-21.93	167	-21.93	247911	21	4434	3906
miR529b	LOC_Os09g01540.1	161	-25	161	-25	247958	21	2583	2558
miR529b	LOC_Os09g01830.1	154	-20.29	154	-20.29	247994	21	1998	14
miR529b	LOC_Os09g01840.1	154	-20.29	154	-20.29	247995	21	1998	14
miR529b	LOC_Os09g02360.1	178	-26.85	178	-26.85	248048	21	1835	271
miR529b	LOC_Os09g03160.1	162	-20.9	162	-20.9	248130	21	2724	554
miR529b	LOC_Os09g03620.1	164	-24.49	164	-24.49	248178	21	2412	24
miR529b	LOC_Os09g03810.1	170	-23.03	170	-23.03	248193	21	357	101
miR529b	LOC_Os09g04540.1	161	-20.5	161	-20.5	248272	21	2067	2042
miR529b	LOC_Os09g04740.1	157	-21.69	157	-21.69	248292	21	4383	546
miR529b	LOC_Os09g06230.1	168	-22.71	168	-22.71	248345	21	1722	266
miR529b	LOC_Os09g06499.1	156	-20.49	156	-20.49	248370	21	2703	673
miR529b	LOC_Os09g06499.2	156	-20.49	156	-20.49	248371	21	2161	673
miR529b	LOC_Os09g06740.1	162	-21.27	162	-21.27	248394	21	1258	25
miR529b	LOC_Os09g07970.1	169	-23.84	169	-23.84	248535	21	2802	1835
miR529b	LOC_Os09g08020.1	145	-20.29	145	-20.29	248540	21	4923	4346
miR529b	LOC_Os09g08510.1	151	-20.15	151	-20.15	248591	21	1341	948
miR529b	LOC_Os09g08930.1	169	-23.84	169	-23.84	248633	21	2934	2006
miR529b	LOC_Os09g09100.1	167	-21.93	167	-21.93	248650	21	2691	1587
miR529b	LOC_Os09g09290.1	168	-23.18	168	-23.18	248668	21	7384	4462

miR529b	LOC_Os09g09290.2	168	-23.18	168	-23.18	248669	21	6473	4455
miR529b	LOC_Os09g10080.1	161	-25	161	-25	248748	21	2484	2459
miR529b	LOC_Os09g10120.1	164	-23.85	164	-23.85	248752	21	1626	147
miR529b	LOC_Os09g10140.1	164	-23.85	164	-23.85	248754	21	1626	147
miR529b	LOC_Os09g10160.1	164	-23.85	164	-23.85	248756	21	1626	147
miR529b	LOC_Os09g10750.1	153	-21.38	153	-21.38	248818	21	1370	64
miR529b	LOC_Os09g11390.1	164	-24.01	164	-24.01	248887	21	2184	1199
miR529b	LOC_Os09g11820.1	156	-21.71	156	-21.71	248933	21	3594	2349
miR529b	LOC_Os09g11950.1	161	-25	161	-25	248946	21	2058	2033
miR529b	LOC_Os09g12750.1	157	-20.1	157	-20.1	249029	21	1420	254
miR529b	LOC_Os09g12750.2	157	-20.1	157	-20.1	249030	21	2820	254
miR529b	LOC_Os09g12890.1	167	-21.93	167	-21.93	249043	21	4776	3819
miR529b	LOC_Os09g13050.1	165	-21.55	165	-21.55	249058	21	540	41
miR529b	LOC_Os09g13890.1	164	-24.87	164	-24.87	249147	21	1644	12
miR529b	LOC_Os09g14300.1	154	-20.29	154	-20.29	249189	21	438	72
miR529b	LOC_Os09g14410.1	159	-23.92	159	-23.92	249200	21	3784	2521
miR529b	LOC_Os09g14930.1	163	-20.91	163	-20.91	249259	21	1734	30
miR529b	LOC_Os09g15800.1	170	-27.09	170	-27.09	249356	21	2268	217
miR529b	LOC_Os09g16430.1	157	-21.69	157	-21.69	249421	21	3786	531
miR529b	LOC_Os09g16850.1	156	-23.58	156	-23.58	249465	21	1152	52
miR529b	LOC_Os09g16995.1	157	-21.65	157	-21.65	249480	21	3285	2201
miR529b	LOC_Os09g17600.1	159	-21.27	159	-21.27	249541	21	2154	20
miR529b	LOC_Os09g17600.2	159	-21.27	159	-21.27	249542	21	2131	1
miR529b	LOC_Os09g17600.3	159	-21.27	159	-21.27	249543	21	2315	22
miR529b	LOC_Os09g19600.1	161	-25	161	-25	249686	21	2436	2411
miR529b	LOC_Os09g19830.1	326	-41.6	170	-20.96	249710	21	3844	3109 3744
miR529b	LOC_Os09g19840.1	342	-48.8	174	-24.54	249711	21	435	28 57
miR529b	LOC_Os09g20740.1	153	-20.46	153	-20.46	249817	21	1317	96
miR529b	LOC_Os09g21120.1	174	-24.54	174	-24.54	249858	21	2592	29
miR529b	LOC_Os09g21250.1	162	-20.9	162	-20.9	249874	21	1085	581
miR529b	LOC_Os09g22090.1	162	-20.54	162	-20.54	249960	21	3525	2922
miR529b	LOC_Os09g23840.1	154	-20.29	154	-20.29	250092	21	1818	14

miR529b	LOC_Os09g24820.1	174	-25.45	174	-25.45	250197	21	4291	3585
miR529b	LOC_Os09g24840.1	166	-20.74	166	-20.74	250199	21	696	86
miR529b	LOC_Os09g24840.2	166	-20.74	166	-20.74	250200	21	679	86
miR529b	LOC_Os09g26390.1	168	-23.18	168	-23.18	250368	21	3659	2624
miR529b	LOC_Os09g26390.2	168	-23.18	168	-23.18	250369	21	4010	2975
miR529b	LOC_Os09g26390.3	168	-23.18	168	-23.18	250370	21	4062	3027
miR529b	LOC_Os09g26580.1	156	-21.09	156	-21.09	250394	21	2379	15
miR529b	LOC_Os09g27700.1	158	-20.55	158	-20.55	250536	21	2717	1341
miR529b	LOC_Os09g27700.2	158	-20.55	158	-20.55	250537	21	2855	1479
miR529b	LOC_Os09g28370.1	144	-20.23	144	-20.23	250616	21	6942	2020
miR529b	LOC_Os09g28710.1	172	-24.1	172	-24.1	250654	21	864	634
miR529b	LOC_Os09g29510.1	164	-24.7	164	-24.7	250746	21	2271	1096
miR529b	LOC_Os09g29600.1	152	-20.32	152	-20.32	250754	21	2475	418
miR529b	LOC_Os09g29720.1	169	-23.84	169	-23.84	250766	21	2775	1847
miR529b	LOC_Os09g30150.1	162	-20.32	162	-20.32	250822	21	2463	1435
miR529b	LOC_Os09g30150.2	162	-20.32	162	-20.32	250823	21	2174	1146
miR529b	LOC_Os09g30150.3	162	-20.32	162	-20.32	250824	21	2313	1285
miR529b	LOC_Os09g30400.1	164	-20.5	164	-20.5	250855	21	2835	2230
miR529b	LOC_Os09g30411.1	156	-21.65	156	-21.65	250860	21	1321	512
miR529b	LOC_Os09g30412.1	166	-20.09	166	-20.09	250861	21	2613	1740
miR529b	LOC_Os09g30418.1	166	-20.09	166	-20.09	250865	21	2493	1745
miR529b	LOC_Os09g31300.1	165	-20.36	165	-20.36	250927	21	2112	215
miR529b	LOC_Os09g31300.2	165	-20.36	165	-20.36	250928	21	2106	215
miR529b	LOC_Os09g31390.1	166	-21.77	166	-21.77	250936	21	2479	2180
miR529b	LOC_Os09g31390.2	166	-21.77	166	-21.77	250937	21	2473	2174
miR529b	LOC_Os09g32080.1	159	-21.76	159	-21.76	250992	21	1850	45
miR529b	LOC_Os09g32080.2	159	-21.76	159	-21.76	250993	21	1726	45
miR529b	LOC_Os09g32510.1	165	-23.27	165	-23.27	251043	21	1656	939
miR529b	LOC_Os09g32510.2	165	-23.27	165	-23.27	251044	21	1741	1024
miR529b	LOC_Os09g32510.3	165	-23.27	165	-23.27	251045	21	1555	838
miR529b	LOC_Os09g32510.4	165	-23.27	165	-23.27	251046	21	1577	860
miR529b	LOC_Os09g32510.5	165	-23.27	165	-23.27	251047	21	1669	860

miR529b	LOC_Os09g32944.1	182	-32.61	182	-32.61	251105	21	1419	1029
miR529b	LOC_Os09g32964.1	152	-23.18	152	-23.18	251112	21	1212	154
miR529b	LOC_Os09g33660.1	165	-22.38	165	-22.38	251151	21	171	43
miR529b	LOC_Os09g33860.1	151	-21.68	151	-21.68	251181	21	1756	16
miR529b	LOC_Os09g34180.1	168	-24.98	168	-24.98	251233	21	2888	73
miR529b	LOC_Os09g34860.1	155	-20.07	155	-20.07	251259	21	1453	1272
miR529b	LOC_Os09g36010.1	168	-24.98	168	-24.98	251338	21	936	42
miR529b	LOC_Os09g36450.1	164	-23.43	164	-23.43	251394	21	1459	736
miR529b	LOC_Os09g36450.2	164	-23.43	164	-23.43	251395	21	1438	715
miR529b	LOC_Os09g37510.1	164	-22.28	164	-22.28	251521	21	1845	1826
miR529b	LOC_Os09g37910.1	171	-21.22	171	-21.22	251563	21	956	738
miR529b	LOC_Os09g37930.1	314	-43.15	162	-22.75	251565	21	3690	3414 3390
miR529b	LOC_Os09g38620.1	158	-20.99	158	-20.99	251643	21	2678	870
miR529b	LOC_Os09g38970.1	161	-21.84	161	-21.84	251690	21	2753	2700
miR529b	LOC_Os09g39230.1	158	-20.91	158	-20.91	251716	21	1682	1431
miR529b	LOC_Os09g39410.1	153	-20.52	153	-20.52	251737	21	2078	60
miR529b	LOC_Os10g01020.1	145	-20.29	145	-20.29	251829	21	5976	5390
miR529b	LOC_Os10g01200.1	155	-20.37	155	-20.37	251846	21	873	405
miR529b	LOC_Os10g01610.1	160	-21.57	160	-21.57	251886	21	3893	2250
miR529b	LOC_Os10g01750.1	165	-21.47	165	-21.47	251899	21	4053	3273
miR529b	LOC_Os10g01850.1	160	-20.25	160	-20.25	251910	21	3147	2370
miR529b	LOC_Os10g01900.1	157	-21.83	157	-21.83	251915	21	1077	594
miR529b	LOC_Os10g02230.1	156	-20.4	156	-20.4	251951	21	708	21
miR529b	LOC_Os10g02500.1	161	-21.98	161	-21.98	251979	21	3948	3839
miR529b	LOC_Os10g02760.1	163	-20.23	163	-20.23	252006	21	2365	1518
miR529b	LOC_Os10g02760.2	163	-20.23	163	-20.23	252007	21	2362	1518
miR529b	LOC_Os10g02910.1	159	-21.27	159	-21.27	252022	21	2821	961
miR529b	LOC_Os10g03330.1	169	-23.84	169	-23.84	252064	21	3081	1892
miR529b	LOC_Os10g04850.1	165	-23.07	165	-23.07	252214	21	2187	615
miR529b	LOC_Os10g05130.1	166	-21.48	166	-21.48	252241	21	1384	102
miR529b	LOC_Os10g05610.1	173	-24.6	173	-24.6	252294	21	210	58
miR529b	LOC_Os10g05695.1	336	-48.64	168	-24.38	252302	21	3975	3667 3690

miR529b	LOC_Os10g06520.1	167	-21.93	167	-21.93	252386	21	4893	3735
miR529b	LOC_Os10g06630.1	174	-22.74	174	-22.74	252395	21	1015	91
miR529b	LOC_Os10g07510.1	154	-20.53	154	-20.53	252474	21	1030	830
miR529b	LOC_Os10g09060.1	167	-21.93	167	-21.93	252625	21	3423	3354
miR529b	LOC_Os10g09970.1	167	-21.93	167	-21.93	252712	21	3735	2403
miR529b	LOC_Os10g10290.1	153	-20.34	153	-20.34	252750	21	1509	1275
miR529b	LOC_Os10g10400.1	164	-22.7	164	-22.7	252763	21	363	38
miR529b	LOC_Os10g10820.1	161	-25	161	-25	252803	21	2580	2555
miR529b	LOC_Os10g10840.1	161	-25	161	-25	252805	21	2436	2411
miR529b	LOC_Os10g11220.1	178	-28.3	178	-28.3	252841	21	2751	1517
miR529b	LOC_Os10g11310.1	165	-22	165	-22	252852	21	2833	1116
miR529b	LOC_Os10g11310.2	165	-22	165	-22	252853	21	2891	1116
miR529b	LOC_Os10g11310.3	165	-22	165	-22	252854	21	2841	1116
miR529b	LOC_Os10g12280.1	157	-22.29	157	-22.29	252936	21	2538	2513
miR529b	LOC_Os10g12750.1	170	-23.76	170	-23.76	252974	21	1409	1373
miR529b	LOC_Os10g13340.1	154	-20.29	154	-20.29	252987	21	1632	14
miR529b	LOC_Os10g13940.1	157	-21.63	157	-21.63	253048	21	2053	1208
miR529b	LOC_Os10g13990.1	161	-25	161	-25	253054	21	2481	2456
miR529b	LOC_Os10g14030.1	178	-28.3	178	-28.3	253058	21	1692	989
miR529b	LOC_Os10g15390.1	158	-20.59	158	-20.59	253127	21	1045	395
miR529b	LOC_Os10g15390.2	158	-20.59	158	-20.59	253128	21	1004	364
miR529b	LOC_Os10g16620.1	167	-22.36	167	-22.36	253177	21	537	5
miR529b	LOC_Os10g17310.1	164	-20.53	164	-20.53	253240	21	2106	1065
miR529b	LOC_Os10g17410.1	154	-21	154	-21	253250	21	3336	1381
miR529b	LOC_Os10g19310.1	158	-20.48	158	-20.48	253421	21	1257	600
miR529b	LOC_Os10g20710.1	161	-20.64	161	-20.64	253536	21	2550	21
miR529b	LOC_Os10g21460.1	164	-21.13	164	-21.13	253642	21	1512	114
miR529b	LOC_Os10g21470.1	170	-22.93	170	-22.93	253643	21	3784	2194
miR529b	LOC_Os10g21470.2	170	-22.93	170	-22.93	253644	21	1879	289
miR529b	LOC_Os10g22100.1	163	-20.77	163	-20.77	253708	21	2503	667
miR529b	LOC_Os10g22300.1	163	-23.19	163	-23.19	253728	21	2166	1074
miR529b	LOC_Os10g22410.1	325	-44.54	164	-23.17	253742	21	811	384 167

miR529b	LOC_Os10g22950.1	164	-21.49	164	-21.49	253802	21	3560	3281
miR529b	LOC_Os10g23090.1	164	-20.56	164	-20.56	253817	21	1440	153
miR529b	LOC_Os10g23100.1	165	-24.26	165	-24.26	253819	21	1286	1064
miR529b	LOC_Os10g23190.1	156	-21.71	156	-21.71	253830	21	2772	1026
miR529b	LOC_Os10g23880.1	167	-23.13	167	-23.13	253850	21	3528	2988
miR529b	LOC_Os10g24230.1	151	-21.1	151	-21.1	253888	21	3234	2755
miR529b	LOC_Os10g24450.1	164	-20.53	164	-20.53	253909	21	2798	918
miR529b	LOC_Os10g25200.1	160	-25.47	160	-25.47	253981	21	4170	1550
miR529b	LOC_Os10g25550.1	146	-22.27	146	-22.27	254013	21	1485	471
miR529b	LOC_Os10g25690.1	166	-22.69	166	-22.69	254032	21	405	341
miR529b	LOC_Os10g26510.1	168	-24.51	168	-24.51	254116	21	1002	76
miR529b	LOC_Os10g26540.1	168	-24.98	168	-24.98	254119	21	866	5
miR529b	LOC_Os10g26730.1	152	-22.44	152	-22.44	254141	21	1702	585
miR529b	LOC_Os10g26730.2	152	-22.44	152	-22.44	254142	21	1610	585
miR529b	LOC_Os10g28060.1	157	-20.83	157	-20.83	254232	21	1856	1807
miR529b	LOC_Os10g28254.1	316	-42.26	159	-21.55	254251	21	902	12 23
miR529b	LOC_Os10g28790.1	157	-20.51	157	-20.51	254315	21	1122	184
miR529b	LOC_Os10g29670.1	166	-21.55	166	-21.55	254403	21	1249	447
miR529b	LOC_Os10g29670.2	166	-21.55	166	-21.55	254404	21	1478	676
miR529b	LOC_Os10g30580.1	166	-24.54	166	-24.54	254489	21	2882	40
miR529b	LOC_Os10g31040.1	161	-22.11	161	-22.11	254540	21	2525	156
miR529b	LOC_Os10g31040.5	161	-22.11	161	-22.11	254544	21	2445	154
miR529b	LOC_Os10g31600.1	161	-23.87	161	-23.87	254597	21	2544	2519
miR529b	LOC_Os10g31830.1	164	-22.08	164	-22.08	254620	21	1468	938
miR529b	LOC_Os10g33720.1	168	-24.98	168	-24.98	254806	21	2580	14
miR529b	LOC_Os10g33750.1	155	-20.1	155	-20.1	254811	21	1309	1077
miR529b	LOC_Os10g33850.1	161	-21.35	161	-21.35	254827	21	1386	28
miR529b	LOC_Os10g35630.1	158	-22.8	158	-22.8	255031	21	1625	101
miR529b	LOC_Os10g37510.1	164	-22.28	164	-22.28	255222	21	708	23
miR529b	LOC_Os10g37520.1	168	-23.18	168	-23.18	255223	21	1883	29
miR529b	LOC_Os10g37520.2	168	-23.18	168	-23.18	255224	21	1829	29
miR529b	LOC_Os10g37960.1	168	-25.5	168	-25.5	255280	21	1692	1234

miR529b	LOC_Os10g38196.1	153	-21.5	153	-21.5	255304	21	402	138
miR529b	LOC_Os10g38960.1	155	-20.45	155	-20.45	255373	21	2025	60
miR529b	LOC_Os10g39710.1	174	-25.45	174	-25.45	255471	21	1865	1542
miR529b	LOC_Os10g39780.1	173	-25.25	173	-25.25	255480	21	2295	248
miR529b	LOC_Os10g39780.3	173	-25.25	173	-25.25	255482	21	2284	248
miR529b	LOC_Os10g39780.4	173	-25.25	173	-25.25	255483	21	1978	248
miR529b	LOC_Os10g40160.1	157	-22.29	157	-22.29	255544	21	2496	2471
miR529b	LOC_Os10g40280.1	158	-20.25	158	-20.25	255557	21	1658	1197
miR529b	LOC_Os10g40640.1	155	-20.8	155	-20.8	255592	21	2374	6
miR529b	LOC_Os10g40730.1	161	-22.23	161	-22.23	255599	21	1637	309
miR529b	LOC_Os10g40790.1	165	-22.01	165	-22.01	255604	21	843	36
miR529b	LOC_Os10g41040.1	158	-21.4	158	-21.4	255635	21	2671	2557
miR529b	LOC_Os10g41040.2	158	-21.4	158	-21.4	255636	21	2770	2656
miR529b	LOC_Os10g41224.1	165	-21.84	165	-21.84	255656	21	970	360
miR529b	LOC_Os10g41440.1	166	-25.37	166	-25.37	255683	21	1823	15
miR529b	LOC_Os10g41480.1	159	-20.6	159	-20.6	255687	21	1619	34
miR529b	LOC_Os10g41580.1	165	-20.36	165	-20.36	255697	21	1643	1498
miR529b	LOC_Os10g41960.1	170	-23.73	170	-23.73	255751	21	2628	349
miR529b	LOC_Os10g42700.1	162	-20.89	162	-20.89	255834	21	1586	208
miR529b	LOC_Os10g42754.1	165	-22.01	165	-22.01	255848	21	1473	232
miR529b	LOC_Os11g01170.1	168	-24.98	168	-24.98	255916	21	1383	102
miR529b	LOC_Os11g01470.1	174	-22.84	174	-22.84	255948	21	5073	4736
miR529b	LOC_Os11g01480.1	160	-22.17	160	-22.17	255949	21	1710	378
miR529b	LOC_Os11g01820.1	164	-20.31	164	-20.31	255986	21	3023	2570
miR529b	LOC_Os11g01836.1	169	-23.56	169	-23.56	255988	21	2594	874
miR529b	LOC_Os11g02464.1	165	-21.26	165	-21.26	256071	21	2615	1873
miR529b	LOC_Os11g03420.1	154	-21.28	154	-21.28	256176	21	744	2
miR529b	LOC_Os11g03700.1	153	-21.69	153	-21.69	256208	21	1995	1636
miR529b	LOC_Os11g03970.1	155	-20.14	155	-20.14	256238	21	1940	1476
miR529b	LOC_Os11g03970.2	155	-20.14	155	-20.14	256239	21	1924	1460
miR529b	LOC_Os11g04220.1	154	-20.16	154	-20.16	256270	21	5526	936
miR529b	LOC_Os11g04440.2	166	-20.3	166	-20.3	256301	21	936	168

miR529b	LOC_Os11g04450.1	166	-20.57	166	-20.57	256303	21	471	60
miR529b	LOC_Os11g05300.1	167	-21.68	167	-21.68	256405	21	1105	616
miR529b	LOC_Os11g05930.1	153	-20.86	153	-20.86	256474	21	2358	2216
miR529b	LOC_Os11g05930.2	153	-20.86	153	-20.86	256475	21	2372	2230
miR529b	LOC_Os11g05930.3	153	-20.86	153	-20.86	256476	21	2506	2364
miR529b	LOC_Os11g05930.4	153	-20.86	153	-20.86	256477	21	2490	2355
miR529b	LOC_Os11g05930.5	153	-20.86	153	-20.86	256478	21	2651	2515
miR529b	LOC_Os11g06200.1	151	-20.44	151	-20.44	256510	21	888	97
miR529b	LOC_Os11g06420.1	159	-20.11	159	-20.11	256541	21	2107	241
miR529b	LOC_Os11g06420.2	159	-20.11	159	-20.11	256542	21	2103	241
miR529b	LOC_Os11g06500.1	168	-21.4	168	-21.4	256550	21	993	384
miR529b	LOC_Os11g06910.1	161	-20.37	161	-20.37	256595	21	3414	3241
miR529b	LOC_Os11g06910.2	161	-20.37	161	-20.37	256596	21	3511	3338
miR529b	LOC_Os11g06910.3	161	-20.37	161	-20.37	256597	21	3521	3348
miR529b	LOC_Os11g07880.1	164	-21.56	164	-21.56	256714	21	772	646
miR529b	LOC_Os11g08050.1	161	-22.38	161	-22.38	256734	21	2130	696
miR529b	LOC_Os11g08210.1	162	-20.28	162	-20.28	256752	21	1998	409
miR529b	LOC_Os11g08480.1	165	-24	165	-24	256786	21	651	18
miR529b	LOC_Os11g09900.1	166	-20.56	166	-20.56	256932	21	1664	701
miR529b	LOC_Os11g10090.1	148	-20.25	148	-20.25	256952	21	2046	236
miR529b	LOC_Os11g10420.1	157	-20.38	157	-20.38	256986	21	4891	3011
miR529b	LOC_Os11g10420.2	157	-20.38	157	-20.38	256987	21	5159	3279
miR529b	LOC_Os11g10970.1	157	-21.69	157	-21.69	257044	21	3300	531
miR529b	LOC_Os11g12130.1	167	-21.93	167	-21.93	257163	21	3960	3477
miR529b	LOC_Os11g13780.1	174	-26.46	174	-26.46	257276	21	1017	937
miR529b	LOC_Os11g14140.1	160	-22.33	160	-22.33	257322	21	1654	267
miR529b	LOC_Os11g14510.1	156	-21.71	156	-21.71	257361	21	4059	2220
miR529b	LOC_Os11g14630.1	155	-21.7	155	-21.7	257382	21	1857	1682
miR529b	LOC_Os11g15100.1	157	-22.29	157	-22.29	257436	21	2448	2423
miR529b	LOC_Os11g16550.1	158	-23.36	158	-23.36	257549	21	1573	516
miR529b	LOC_Os11g16580.1	146	-20.79	146	-20.79	257552	21	1656	596
miR529b	LOC_Os11g17230.1	165	-22.26	165	-22.26	257609	21	3858	3043

miR529b	LOC_Os11g17530.1	161	-22.38	161	-22.38	257640	21	2705	2395
miR529b	LOC_Os11g17600.1	161	-23.99	161	-23.99	257646	21	3207	125
miR529b	LOC_Os11g17600.2	161	-23.99	161	-23.99	257647	21	3270	125
miR529b	LOC_Os11g17600.3	161	-23.99	161	-23.99	257648	21	3048	125
miR529b	LOC_Os11g17970.1	153	-21.47	153	-21.47	257681	21	2197	1839
miR529b	LOC_Os11g18930.1	166	-23.01	166	-23.01	257768	21	306	1
miR529b	LOC_Os11g19780.1	160	-20.31	160	-20.31	257861	21	305	204
miR529b	LOC_Os11g19810.1	169	-21.99	169	-21.99	257864	21	3438	1680
miR529b	LOC_Os11g19810.2	169	-21.99	169	-21.99	257865	21	3642	1686
miR529b	LOC_Os11g20610.1	316	-41.82	161	-21.52	257941	21	3786	2977 2526
miR529b	LOC_Os11g21790.1	167	-23.13	167	-23.13	257960	21	3216	2361
miR529b	LOC_Os11g22020.1	161	-24.99	161	-24.99	257983	21	2205	2180
miR529b	LOC_Os11g22150.1	149	-20.68	149	-20.68	257996	21	651	436
miR529b	LOC_Os11g22150.2	149	-20.68	149	-20.68	257997	21	679	459
miR529b	LOC_Os11g22550.1	164	-23.56	164	-23.56	258039	21	3138	2681
miR529b	LOC_Os11g23830.1	157	-21.69	157	-21.69	258128	21	1689	135
miR529b	LOC_Os11g26010.1	162	-20.54	162	-20.54	258334	21	2798	110
miR529b	LOC_Os11g26520.1	161	-20.3	161	-20.3	258381	21	3621	2488
miR529b	LOC_Os11g28140.1	169	-23.84	169	-23.84	258538	21	3162	1973
miR529b	LOC_Os11g28970.1	164	-22.81	164	-22.81	258623	21	1227	450
miR529b	LOC_Os11g30170.1	156	-21.71	156	-21.71	258749	21	4053	2763
miR529b	LOC_Os11g30280.1	155	-20.1	155	-20.1	258760	21	498	132
miR529b	LOC_Os11g30300.1	170	-25.32	170	-25.32	258762	21	387	1
miR529b	LOC_Os11g31900.1	154	-20.27	154	-20.27	258918	21	877	269
miR529b	LOC_Os11g31900.2	154	-20.27	154	-20.27	258919	21	827	331
miR529b	LOC_Os11g31900.3	154	-20.27	154	-20.27	258920	21	1072	269
miR529b	LOC_Os11g32900.1	162	-20.95	162	-20.95	259027	21	3416	10
miR529b	LOC_Os11g32900.2	162	-20.95	162	-20.95	259028	21	3508	8
miR529b	LOC_Os11g33030.1	149	-22.93	149	-22.93	259044	21	1618	1544
miR529b	LOC_Os11g34750.1	156	-24.29	156	-24.29	259185	21	2274	1532
miR529b	LOC_Os11g35220.1	161	-20.63	161	-20.63	259235	21	2284	1722
miR529b	LOC_Os11g35350.1	158	-20.75	158	-20.75	259247	21	816	382

miR529b	LOC_Os11g35650.1	178	-28.3	178	-28.3	259281	21	2751	1517
miR529b	LOC_Os11g36430.1	160	-21.25	160	-21.25	259370	21	1750	1068
miR529b	LOC_Os11g36430.2	160	-21.25	160	-21.25	259371	21	1627	1068
miR529b	LOC_Os11g36430.3	160	-21.25	160	-21.25	259372	21	1665	983
miR529b	LOC_Os11g36430.5	160	-21.25	160	-21.25	259373	21	1833	1151
miR529b	LOC_Os11g36470.1	174	-26.62	174	-26.62	259377	21	3463	2312
miR529b	LOC_Os11g36510.1	157	-20.12	157	-20.12	259382	21	2802	1195
miR529b	LOC_Os11g36630.1	166	-21.94	166	-21.94	259393	21	2184	204
miR529b	LOC_Os11g37090.1	156	-21.1	156	-21.1	259436	21	3185	3147
miR529b	LOC_Os11g37260.1	168	-22.91	168	-22.91	259452	21	2897	35
miR529b	LOC_Os11g37520.1	328	-46.71	168	-24.98	259477	21	3712	15 3626
miR529b	LOC_Os11g37740.1	152	-20.89	152	-20.89	259500	21	3330	1951
miR529b	LOC_Os11g38520.1	166	-21.86	166	-21.86	259585	21	4343	2839
miR529b	LOC_Os11g39640.1	156	-20.61	156	-20.61	259713	21	2007	1866
miR529b	LOC_Os11g40315.1	166	-20.4	166	-20.4	259782	21	2838	1820
miR529b	LOC_Os11g40400.1	161	-22.93	161	-22.93	259792	21	891	44
miR529b	LOC_Os11g40490.1	158	-25.74	158	-25.74	259801	21	1116	1071
miR529b	LOC_Os11g40510.1	165	-20.94	165	-20.94	259805	21	2088	1036
miR529b	LOC_Os11g40510.2	165	-20.94	165	-20.94	259806	21	1974	1036
miR529b	LOC_Os11g41390.1	150	-21.82	150	-21.82	259898	21	1305	1036
miR529b	LOC_Os11g41600.1	161	-20.95	161	-20.95	259922	21	1112	943
miR529b	LOC_Os11g41600.2	161	-20.95	161	-20.95	259923	21	1043	874
miR529b	LOC_Os11g42330.1	157	-22.29	157	-22.29	259998	21	2466	2441
miR529b	LOC_Os11g42340.1	336	-48.17	168	-24.38	259999	21	423	22 47
miR529b	LOC_Os11g43380.1	161	-25	161	-25	260107	21	2436	2411
miR529b	LOC_Os11g43390.1	159	-20.19	159	-20.19	260108	21	2800	1448
miR529b	LOC_Os11g43404.1	165	-25.12	165	-25.12	260110	21	2264	1386
miR529b	LOC_Os11g43404.2	165	-25.12	165	-25.12	260111	21	2347	1468
miR529b	LOC_Os11g43404.5	165	-25.12	165	-25.12	260114	21	1409	530
miR529b	LOC_Os11g44280.1	160	-21.55	160	-21.55	260207	21	183	18
miR529b	LOC_Os11g45000.1	165	-20.87	165	-20.87	260283	21	2868	501
miR529b	LOC_Os11g45370.1	164	-22.97	164	-22.97	260320	21	414	5

miR529b	LOC_Os11g45620.1	166	-23.63	166	-23.63	260347	21	2838	1580
miR529b	LOC_Os11g45660.1	178	-28.3	178	-28.3	260349	21	2751	1517
miR529b	LOC_Os11g46120.1	168	-24.98	168	-24.98	260403	21	2870	2093
miR529b	LOC_Os11g46200.1	161	-20.63	161	-20.63	260409	21	3378	1672
miR529b	LOC_Os11g46290.1	156	-21.71	156	-21.71	260419	21	2598	1026
miR529b	LOC_Os11g47430.1	154	-20.23	154	-20.23	260484	21	5769	4558
miR529b	LOC_Os11g47432.1	166	-25.87	166	-25.87	260488	21	4251	723
miR529b	LOC_Os11g47438.1	166	-25.87	166	-25.87	260492	21	4020	723
miR529b	LOC_Os11g47574.1	158	-22.5	158	-22.5	260521	21	1010	19
miR529b	LOC_Os11g47650.1	162	-20.5	162	-20.5	260529	21	1830	61
miR529b	LOC_Os11g47650.2	162	-20.5	162	-20.5	260530	21	1911	61
miR529b	LOC_Os12g01170.1	168	-24.98	168	-24.98	260607	21	1389	96
miR529b	LOC_Os12g01490.1	158	-22.28	158	-22.28	260643	21	1803	473
miR529b	LOC_Os12g01820.1	165	-21.92	165	-21.92	260685	21	2994	2737
miR529b	LOC_Os12g01850.1	169	-23.56	169	-23.56	260687	21	2535	782
miR529b	LOC_Os12g01955.1	171	-26.04	171	-26.04	260705	21	1435	694
miR529b	LOC_Os12g02390.1	165	-21.26	165	-21.26	260757	21	2626	1865
miR529b	LOC_Os12g02730.1	155	-20.23	155	-20.23	260806	21	779	611
miR529b	LOC_Os12g02840.1	153	-20.33	153	-20.33	260824	21	2891	2015
miR529b	LOC_Os12g02960.1	165	-20.42	165	-20.42	260836	21	911	780
miR529b	LOC_Os12g03110.1	154	-21.28	154	-21.28	260856	21	742	5
miR529b	LOC_Os12g03810.1	155	-20.14	155	-20.14	260929	21	2183	1721
miR529b	LOC_Os12g03810.2	155	-20.14	155	-20.14	260930	21	1934	1472
miR529b	LOC_Os12g04030.1	154	-20.16	154	-20.16	260962	21	3078	1112
miR529b	LOC_Os12g04130.1	165	-22.27	165	-22.27	260972	21	2105	1495
miR529b	LOC_Os12g05500.1	161	-24.99	161	-24.99	261135	21	2079	2054
miR529b	LOC_Os12g05810.1	160	-22.87	160	-22.87	261170	21	1439	123
miR529b	LOC_Os12g05810.2	160	-22.87	160	-22.87	261171	21	1509	123
miR529b	LOC_Os12g05810.3	160	-22.87	160	-22.87	261172	21	1156	123
miR529b	LOC_Os12g06040.1	156	-21.71	156	-21.71	261193	21	4671	2994
miR529b	LOC_Os12g06260.1	165	-20.52	165	-20.52	261217	21	1397	1205
miR529b	LOC_Os12g06410.1	160	-21.05	160	-21.05	261233	21	1314	521

miR529b	LOC_Os12g06980.1	145	-20.08	145	-20.08	261303	21	7811	5349
miR529b	LOC_Os12g07840.1	167	-21.81	167	-21.81	261395	21	3569	6
miR529b	LOC_Os12g07940.1	161	-21.83	161	-21.83	261406	21	2873	1861
miR529b	LOC_Os12g09530.1	166	-24.94	166	-24.94	261580	21	3303	3049
miR529b	LOC_Os12g09870.1	157	-22.29	157	-22.29	261614	21	2466	2441
miR529b	LOC_Os12g11200.1	163	-21.21	163	-21.21	261757	21	2034	852
miR529b	LOC_Os12g11680.1	175	-20.79	175	-20.79	261801	21	3282	3049
miR529b	LOC_Os12g11720.1	175	-20.79	175	-20.79	261805	21	3060	2960
miR529b	LOC_Os12g11860.1	175	-20.99	175	-20.99	261818	21	3018	2918
miR529b	LOC_Os12g11930.1	165	-22.15	165	-22.15	261823	21	3273	996
miR529b	LOC_Os12g12010.1	175	-20.79	175	-20.79	261830	21	3000	2900
miR529b	LOC_Os12g12120.1	175	-20.79	175	-20.79	261842	21	3297	2967
miR529b	LOC_Os12g12130.1	179	-23.49	179	-23.49	261843	21	3329	2994
miR529b	LOC_Os12g12230.1	312	-41.3	161	-21.13	261854	21	540	140 437
miR529b	LOC_Os12g12664.1	166	-24.87	166	-24.87	261900	21	1797	449
miR529b	LOC_Os12g12664.2	166	-24.87	166	-24.87	261901	21	3510	449
miR529b	LOC_Os12g12880.1	169	-25.74	169	-25.74	261925	21	1198	1022
miR529b	LOC_Os12g13130.1	157	-20.16	157	-20.16	261949	21	1455	104
miR529b	LOC_Os12g13130.2	157	-20.16	157	-20.16	261950	21	1462	127
miR529b	LOC_Os12g13130.3	157	-20.16	157	-20.16	261951	21	1669	127
miR529b	LOC_Os12g13130.4	157	-20.16	157	-20.16	261952	21	1419	127
miR529b	LOC_Os12g13290.1	157	-21.46	157	-21.46	261971	21	2419	306
miR529b	LOC_Os12g13400.1	161	-20.44	161	-20.44	261985	21	1585	210
miR529b	LOC_Os12g14480.1	159	-23.91	159	-23.91	262098	21	1095	1000
miR529b	LOC_Os12g15620.1	166	-21.94	166	-21.94	262202	21	1902	204
miR529b	LOC_Os12g15780.1	160	-21.31	160	-21.31	262216	21	3660	1569
miR529b	LOC_Os12g16350.1	160	-20.16	160	-20.16	262270	21	2778	546
miR529b	LOC_Os12g16350.13	165	-24.08	165	-24.08	262279	21	4262	932
miR529b	LOC_Os12g17300.1	164	-20.14	164	-20.14	262381	21	2370	2345
miR529b	LOC_Os12g17580.1	157	-20.43	157	-20.43	262409	21	333	289
miR529b	LOC_Os12g18410.1	149	-20.45	149	-20.45	262500	21	1164	146
miR529b	LOC_Os12g18410.2	149	-20.45	149	-20.45	262501	21	1009	86

miR529b	LOC_Os12g19290.1	150	-21.26	150	-21.26	262588	21	2503	175
miR529b	LOC_Os12g19770.1	157	-21.69	157	-21.69	262634	21	4674	531
miR529b	LOC_Os12g20300.1	168	-21.13	168	-21.13	262687	21	4695	1724
miR529b	LOC_Os12g20350.1	161	-25	161	-25	262694	21	2490	2465
miR529b	LOC_Os12g21640.1	155	-20.37	155	-20.37	262727	21	4551	4014
miR529b	LOC_Os12g23010.1	161	-25	161	-25	262868	21	1947	1922
miR529b	LOC_Os12g23130.1	158	-23.63	158	-23.63	262878	21	3732	3157
miR529b	LOC_Os12g23350.1	166	-21.94	166	-21.94	262902	21	2136	204
miR529b	LOC_Os12g23540.1	153	-20.48	153	-20.48	262919	21	2328	2303
miR529b	LOC_Os12g24540.1	154	-22.3	154	-22.3	263011	21	4617	4315
miR529b	LOC_Os12g24770.1	161	-25	161	-25	263032	21	1521	1496
miR529b	LOC_Os12g25170.1	164	-22.27	164	-22.27	263071	21	1754	1710
miR529b	LOC_Os12g25690.1	154	-22.32	154	-22.32	263123	21	1920	32
miR529b	LOC_Os12g26444.1	150	-20.16	150	-20.16	263202	21	4269	2685
miR529b	LOC_Os12g26890.1	155	-20.72	155	-20.72	263245	21	435	224
miR529b	LOC_Os12g27490.1	155	-20.37	155	-20.37	263307	21	4836	3960
miR529b	LOC_Os12g27880.1	167	-21.93	167	-21.93	263346	21	3846	3363
miR529b	LOC_Os12g28340.1	168	-23.18	168	-23.18	263388	21	2166	2142
miR529b	LOC_Os12g29060.1	154	-20.29	154	-20.29	263457	21	1983	14
miR529b	LOC_Os12g29130.1	157	-21.69	157	-21.69	263464	21	4689	531
miR529b	LOC_Os12g29200.1	161	-25	161	-25	263471	21	2436	2411
miR529b	LOC_Os12g29380.1	313	-44.17	157	-22.48	263486	21	4569	531 3998
miR529b	LOC_Os12g29520.1	165	-24.08	165	-24.08	263499	21	3622	3552
miR529b	LOC_Os12g29520.2	165	-24.08	165	-24.08	263500	21	3639	3569
miR529b	LOC_Os12g29520.3	165	-24.08	165	-24.08	263501	21	3619	3549
miR529b	LOC_Os12g29520.4	165	-24.08	165	-24.08	263502	21	3619	3549
miR529b	LOC_Os12g29520.5	165	-24.08	165	-24.08	263503	21	3701	3631
miR529b	LOC_Os12g29530.1	174	-24.07	174	-24.07	263504	21	5167	4765
miR529b	LOC_Os12g29740.1	169	-26.26	169	-26.26	263529	21	1698	1121
miR529b	LOC_Os12g29820.1	164	-25.78	164	-25.78	263537	21	1704	173
miR529b	LOC_Os12g30250.1	161	-20.3	161	-20.3	263581	21	528	40
miR529b	LOC_Os12g30560.1	330	-47.73	168	-24.98	263613	21	546	94 47

miR529b	LOC_Os12g30590.1	166	-25.36	166	-25.36	263616	21	3414	40
miR529b	LOC_Os12g31440.1	145	-20.03	145	-20.03	263693	21	2050	1189
miR529b	LOC_Os12g31860.2	141	-20.65	141	-20.65	263739	21	3029	2557
miR529b	LOC_Os12g32190.1	155	-21.29	155	-21.29	263772	21	2325	826
miR529b	LOC_Os12g32190.2	155	-21.29	155	-21.29	263773	21	2240	826
miR529b	LOC_Os12g33340.1	161	-25	161	-25	263891	21	2355	2330
miR529b	LOC_Os12g33470.1	174	-25.26	174	-25.26	263902	21	432	40
miR529b	LOC_Os12g33560.1	161	-24.99	161	-24.99	263911	21	1776	1751
miR529b	LOC_Os12g34140.1	154	-20.29	154	-20.29	263980	21	1632	14
miR529b	LOC_Os12g34419.1	161	-21.59	161	-21.59	264014	21	231	145
miR529b	LOC_Os12g35490.1	157	-20.35	157	-20.35	264132	21	1906	1089
miR529b	LOC_Os12g35670.1	165	-24.08	165	-24.08	264156	21	1087	138
miR529b	LOC_Os12g36120.1	166	-21.94	166	-21.94	264201	21	2007	204
miR529b	LOC_Os12g36180.1	163	-22.18	163	-22.18	264207	21	3664	1752
miR529b	LOC_Os12g36900.1	165	-22.28	165	-22.28	264280	21	1561	72
miR529b	LOC_Os12g37720.1	163	-20.14	163	-20.14	264379	21	4346	1128
miR529b	LOC_Os12g38310.1	164	-20.29	164	-20.29	264439	21	700	15
miR529b	LOC_Os12g38550.1	167	-21.06	167	-21.06	264466	21	1410	931
miR529b	LOC_Os12g39630.1	168	-23.18	168	-23.18	264606	21	2684	2596
miR529b	LOC_Os12g40840.2	166	-27.67	166	-27.67	264740	21	1554	167
miR529b	LOC_Os12g40840.3	166	-27.67	166	-27.67	264741	21	1928	167
miR529b	LOC_Os12g41060.1	170	-24.39	170	-24.39	264764	21	2141	378
miR529b	LOC_Os12g41124.1	328	-45.35	165	-24.08	264770	21	2710	39 2387
miR529b	LOC_Os12g41680.1	170	-23.69	170	-23.69	264837	21	1532	115
miR529b	LOC_Os12g41870.1	156	-21.71	156	-21.71	264862	21	4710	3198
miR529b	LOC_Os12g42220.1	157	-20.48	157	-20.48	264915	21	1474	1405
miR529b	LOC_Os12g42450.1	159	-21.26	159	-21.26	264943	21	1044	468
miR529b	LOC_Os12g42500.1	158	-23.18	158	-23.18	264947	21	741	697
miR529b	LOC_Os12g42900.1	161	-22.2	161	-22.2	264998	21	1493	332
miR529b	LOC_Os12g43300.1	158	-21	158	-21	265035	21	1993	1652
miR529b	LOC_Os12g44100.1	160	-20.47	160	-20.47	265126	21	2301	254
miR529b	ChrUn.fgenesh.mRNA.49	178	-28.3	178	-28.3	265216	21	2007	1223

miR529b	ChrUn.fgenesh.mRNA.85	167	-22.66	167	-22.66	265252	21	3369	701
miR529b	ChrSy.fgenesh.mRNA.23	161	-25	161	-25	265286	21	2427	2402
miR529b	ChrSy.fgenesh.mRNA.48	159	-21.55	159	-21.55	265311	21	3657	3308
miR399i	LOC_Os01g01080.1	153	-21.18	153	-21.18	331705	21	1955	63
miR399i	LOC_Os01g01080.2	153	-21.18	153	-21.18	331706	21	1976	63
miR399i	LOC_Os01g01080.3	153	-21.18	153	-21.18	331707	21	2042	63
miR399i	LOC_Os01g01100.1	161	-21.95	161	-21.95	331709	21	4017	3254
miR399i	LOC_Os01g01650.1	155	-26.04	155	-26.04	331781	21	1313	661
miR399i	LOC_Os01g01680.1	159	-26.57	159	-26.57	331785	21	1023	111
miR399i	LOC_Os01g01689.1	162	-21.37	162	-21.37	331786	21	8736	6153
miR399i	LOC_Os01g01689.3	162	-21.37	162	-21.37	331787	21	9026	6153
miR399i	LOC_Os01g02000.1	168	-27.63	168	-27.63	331823	21	2767	308
miR399i	LOC_Os01g02230.1	153	-20.56	153	-20.56	331854	21	2898	1153
miR399i	LOC_Os01g02310.1	149	-23.83	149	-23.83	331863	21	1884	1548
miR399i	LOC_Os01g02334.1	149	-23.83	149	-23.83	331865	21	632	239
miR399i	LOC_Os01g02670.1	153	-20.95	153	-20.95	331900	21	3312	589
miR399i	LOC_Os01g03070.1	153	-22.52	153	-22.52	331949	21	3512	2165
miR399i	LOC_Os01g03070.2	153	-22.52	153	-22.52	331950	21	3493	2159
miR399i	LOC_Os01g04950.1	157	-20.34	157	-20.34	332153	21	2778	305
miR399i	LOC_Os01g04950.4	157	-20.34	157	-20.34	332156	21	3836	243
miR399i	LOC_Os01g05710.1	154	-20.29	154	-20.29	332243	21	784	468
miR399i	LOC_Os01g05800.1	149	-24.58	149	-24.58	332251	21	2443	765
miR399i	LOC_Os01g05800.2	149	-24.58	149	-24.58	332252	21	2440	765
miR399i	LOC_Os01g06330.1	165	-23.31	165	-23.31	332307	21	1287	369
miR399i	LOC_Os01g06410.1	151	-21.16	151	-21.16	332315	21	561	208
miR399i	LOC_Os01g06530.1	147	-25.72	147	-25.72	332329	21	2805	53
miR399i	LOC_Os01g07640.1	156	-22.12	156	-22.12	332460	21	1608	321
miR399i	LOC_Os01g08750.1	158	-22.76	158	-22.76	332595	21	10065	3610
miR399i	LOC_Os01g08750.2	158	-22.76	158	-22.76	332596	21	9417	2962
miR399i	LOC_Os01g08890.1	151	-20.57	151	-20.57	332616	21	2771	355
miR399i	LOC_Os01g09550.4	155	-23.39	155	-23.39	332688	21	5715	2064
miR399i	LOC_Os01g09640.1	163	-24.56	163	-24.56	332698	21	1769	1371

miR399i	LOC_Os01g12350.1	152	-20.9	152	-20.9	332986	21	3117	324
miR399i	LOC_Os01g12660.1	146	-23.21	146	-23.21	333023	21	2967	2403
miR399i	LOC_Os01g12790.1	140	-22.85	140	-22.85	333044	21	3756	95
miR399i	LOC_Os01g13060.2	165	-23.23	165	-23.23	333081	21	3190	2349
miR399i	LOC_Os01g13540.1	161	-20.76	161	-20.76	333138	21	3360	1234
miR399i	LOC_Os01g13730.1	152	-20.39	152	-20.39	333162	21	1948	1920
miR399i	LOC_Os01g15029.1	157	-21.76	157	-21.76	333304	21	676	429
miR399i	LOC_Os01g15690.1	154	-23.23	154	-23.23	333376	21	4635	3326
miR399i	LOC_Os01g15890.1	154	-25.79	154	-25.79	333397	21	2637	2315
miR399i	LOC_Os01g15900.1	156	-23.57	156	-23.57	333398	21	2228	1405
miR399i	LOC_Os01g16130.1	149	-21.41	149	-21.41	333427	21	2027	327
miR399i	LOC_Os01g16220.1	160	-25.98	160	-25.98	333444	21	1693	482
miR399i	LOC_Os01g16690.1	159	-20.88	159	-20.88	333499	21	1692	668
miR399i	LOC_Os01g18470.1	149	-20.5	149	-20.5	333635	21	4038	1682
miR399i	LOC_Os01g21160.3	161	-21.15	161	-21.15	333881	21	2026	166
miR399i	LOC_Os01g21450.1	147	-26.26	147	-26.26	333909	21	2470	319
miR399i	LOC_Os01g21450.2	147	-26.26	147	-26.26	333910	21	3220	447
miR399i	LOC_Os01g21450.3	147	-26.26	147	-26.26	333911	21	3512	739
miR399i	LOC_Os01g21500.1	155	-24.66	155	-24.66	333914	21	1164	971
miR399i	LOC_Os01g22640.1	157	-20.71	157	-20.71	334022	21	1926	1663
miR399i	LOC_Os01g23730.1	168	-25.71	168	-25.71	334133	21	804	126
miR399i	LOC_Os01g23940.1	154	-20.84	154	-20.84	334154	21	1353	1225
miR399i	LOC_Os01g24294.1	151	-22.42	151	-22.42	334190	21	5229	184
miR399i	LOC_Os01g25610.1	153	-20.81	153	-20.81	334333	21	1242	1
miR399i	LOC_Os01g27040.1	154	-21.32	154	-21.32	334430	21	3750	3118
miR399i	LOC_Os01g27180.1	150	-23.24	150	-23.24	334445	21	456	241
miR399i	LOC_Os01g27440.1	163	-25.57	163	-25.57	334468	21	3384	360
miR399i	LOC_Os01g27640.1	160	-21.61	160	-21.61	334486	21	1647	1026
miR399i	LOC_Os01g27900.1	149	-20.48	149	-20.48	334510	21	2379	532
miR399i	LOC_Os01g28020.1	155	-20.23	155	-20.23	334524	21	1425	1238
miR399i	LOC_Os01g28540.1	159	-25.56	159	-25.56	334571	21	1315	242
miR399i	LOC_Os01g28540.2	159	-25.56	159	-25.56	334572	21	1288	242

miR399i	LOC_Os01g28680.8	159	-21.15	159	-21.15	334591	21	4068	2418
miR399i	LOC_Os01g28680.9	159	-21.15	159	-21.15	334592	21	3921	2408
miR399i	LOC_Os01g28740.1	149	-20.5	149	-20.5	334602	21	3969	1478
miR399i	LOC_Os01g29070.1	155	-25.34	155	-25.34	334638	21	1233	911
miR399i	LOC_Os01g29750.1	149	-20.5	149	-20.5	334701	21	4698	2480
miR399i	LOC_Os01g31280.1	167	-26.96	167	-26.96	334755	21	384	32
miR399i	LOC_Os01g31730.1	157	-21.13	157	-21.13	334802	21	4719	3006
miR399i	LOC_Os01g32350.1	166	-27.79	166	-27.79	334871	21	1281	835
miR399i	LOC_Os01g32350.2	166	-27.79	166	-27.79	334872	21	1438	835
miR399i	LOC_Os01g32720.1	143	-23.45	143	-23.45	334909	21	3548	3486
miR399i	LOC_Os01g32830.1	161	-20.16	161	-20.16	334921	21	2718	2657
miR399i	LOC_Os01g32830.2	161	-20.16	161	-20.16	334922	21	947	886
miR399i	LOC_Os01g33260.1	157	-20.35	157	-20.35	334967	21	7063	3732
miR399i	LOC_Os01g33869.1	166	-20.03	166	-20.03	335024	21	2391	2347
miR399i	LOC_Os01g34030.1	308	-45.5	155	-24.38	335038	21	2607	2298 255
miR399i	LOC_Os01g34190.1	159	-28.16	159	-28.16	335050	21	1531	1184
miR399i	LOC_Os01g34490.1	145	-20.25	145	-20.25	335080	21	3000	2179
miR399i	LOC_Os01g34630.1	148	-21.32	148	-21.32	335104	21	2389	412
miR399i	LOC_Os01g34890.1	145	-23.11	145	-23.11	335131	21	3675	915
miR399i	LOC_Os01g35090.1	314	-42.22	161	-21.35	335152	21	3960	2572 2623
miR399i	LOC_Os01g35480.1	153	-20.56	153	-20.56	335191	21	6039	4258
miR399i	LOC_Os01g35740.1	153	-20.56	153	-20.56	335222	21	6039	4258
miR399i	LOC_Os01g36080.1	145	-20.64	145	-20.64	335255	21	2886	2621
miR399i	LOC_Os01g36080.2	145	-20.64	145	-20.64	335256	21	2902	2606
miR399i	LOC_Os01g36080.3	145	-20.64	145	-20.64	335257	21	2323	2059
miR399i	LOC_Os01g36080.4	150	-20.48	150	-20.48	335258	21	2944	2680
miR399i	LOC_Os01g36410.1	152	-23.4	152	-23.4	335289	21	711	192
miR399i	LOC_Os01g36730.1	157	-25.26	157	-25.26	335320	21	2538	2183
miR399i	LOC_Os01g37250.1	157	-21.13	157	-21.13	335378	21	2220	776
miR399i	LOC_Os01g37360.1	156	-23.31	156	-23.31	335397	21	2103	1734
miR399i	LOC_Os01g37560.1	159	-23.16	159	-23.16	335416	21	2012	1023
miR399i	LOC_Os01g37560.2	159	-23.16	159	-23.16	335417	21	1836	1023

miR399i	LOC_Os01g37560.3	159	-23.16	159	-23.16	335418	21	2132	1143
miR399i	LOC_Os01g37570.1	155	-25.34	155	-25.34	335419	21	1368	911
miR399i	LOC_Os01g37580.1	154	-21.35	154	-21.35	335420	21	5799	3307
miR399i	LOC_Os01g38260.1	154	-22.63	154	-22.63	335499	21	2067	1972
miR399i	LOC_Os01g38300.1	157	-23.88	157	-23.88	335503	21	1131	7
miR399i	LOC_Os01g38410.1	149	-23.81	149	-23.81	335513	21	1482	835
miR399i	LOC_Os01g38880.1	162	-21.61	162	-21.61	335563	21	1797	527
miR399i	LOC_Os01g38930.1	159	-20.48	159	-20.48	335568	21	849	415
miR399i	LOC_Os01g39370.1	161	-21.95	161	-21.95	335615	21	5430	3722
miR399i	LOC_Os01g39399.1	165	-23.45	165	-23.45	335620	21	519	1
miR399i	LOC_Os01g40630.1	151	-22.66	151	-22.66	335753	21	1429	337
miR399i	LOC_Os01g40630.2	151	-22.66	151	-22.66	335754	21	1856	337
miR399i	LOC_Os01g40630.3	151	-22.66	151	-22.66	335755	21	1404	337
miR399i	LOC_Os01g41400.1	167	-21.97	167	-21.97	335839	21	1332	335
miR399i	LOC_Os01g41660.1	179	-33.62	179	-33.62	335867	21	1641	398
miR399i	LOC_Os01g41950.1	151	-20.04	151	-20.04	335896	21	3201	1222
miR399i	LOC_Os01g42490.1	151	-23.19	151	-23.19	335953	21	3092	2997
miR399i	LOC_Os01g42490.2	151	-23.19	151	-23.19	335954	21	4032	3937
miR399i	LOC_Os01g42490.3	151	-23.19	151	-23.19	335955	21	3426	3331
miR399i	LOC_Os01g42700.1	163	-28.02	163	-28.02	335979	21	2576	1552
miR399i	LOC_Os01g42700.2	163	-28.02	163	-28.02	335980	21	1875	1552
miR399i	LOC_Os01g42700.5	163	-28.02	163	-28.02	335983	21	1837	1552
miR399i	LOC_Os01g42710.1	152	-22.07	152	-22.07	335984	21	4918	3090
miR399i	LOC_Os01g43090.1	155	-21.93	155	-21.93	336031	21	1353	205
miR399i	LOC_Os01g43170.1	148	-20.55	148	-20.55	336041	21	2132	1379
miR399i	LOC_Os01g43170.2	148	-20.55	148	-20.55	336042	21	2190	1379
miR399i	LOC_Os01g43170.3	148	-20.55	148	-20.55	336043	21	2166	1358
miR399i	LOC_Os01g46810.1	157	-20.48	157	-20.48	336394	21	4832	4246
miR399i	LOC_Os01g47690.1	160	-28.95	160	-28.95	336506	21	1559	456
miR399i	LOC_Os01g47690.2	160	-28.95	160	-28.95	336507	21	1385	282
miR399i	LOC_Os01g47820.1	158	-27.56	158	-27.56	336525	21	2425	559
miR399i	LOC_Os01g48700.1	164	-21.96	164	-21.96	336625	21	1185	814

miR399i	LOC_Os01g48900.1	145	-21.66	145	-21.66	336646	21	2691	1309
miR399i	LOC_Os01g49060.1	162	-22.08	162	-22.08	336670	21	1961	542
miR399i	LOC_Os01g49060.2	162	-22.08	162	-22.08	336671	21	1955	532
miR399i	LOC_Os01g49060.3	162	-22.08	162	-22.08	336672	21	2044	532
miR399i	LOC_Os01g49370.1	166	-27.49	166	-27.49	336704	21	981	936
miR399i	LOC_Os01g50360.1	150	-22.34	150	-22.34	336812	21	1551	392
miR399i	LOC_Os01g50530.1	141	-23.86	141	-23.86	336828	21	1236	99
miR399i	LOC_Os01g51890.1	161	-22.93	161	-22.93	336995	21	2687	2013
miR399i	LOC_Os01g52250.1	154	-21.9	154	-21.9	337036	21	5093	5056
miR399i	LOC_Os01g52250.2	154	-21.9	154	-21.9	337037	21	4841	4804
miR399i	LOC_Os01g52250.3	154	-21.9	154	-21.9	337038	21	4452	4415
miR399i	LOC_Os01g52250.4	154	-21.9	154	-21.9	337039	21	5084	5047
miR399i	LOC_Os01g52250.5	154	-21.9	154	-21.9	337040	21	5082	5045
miR399i	LOC_Os01g52250.6	154	-21.9	154	-21.9	337041	21	5163	5126
miR399i	LOC_Os01g52550.1	152	-22.81	152	-22.81	337075	21	4255	4128
miR399i	LOC_Os01g53200.1	164	-23.98	164	-23.98	337143	21	2027	583
miR399i	LOC_Os01g53390.1	144	-24.87	144	-24.87	337163	21	1949	1580
miR399i	LOC_Os01g53420.1	155	-21.04	155	-21.04	337164	21	1752	953
miR399i	LOC_Os01g53480.1	161	-21.55	161	-21.55	337171	21	2301	1199
miR399i	LOC_Os01g53750.1	152	-23.62	152	-23.62	337201	21	1425	1269
miR399i	LOC_Os01g54080.1	155	-23.65	155	-23.65	337246	21	3356	492
miR399i	LOC_Os01g54600.1	151	-21.72	151	-21.72	337306	21	1731	676
miR399i	LOC_Os01g55120.1	158	-22.55	158	-22.55	337369	21	1767	816
miR399i	LOC_Os01g55360.1	147	-23.35	147	-23.35	337399	21	1892	725
miR399i	LOC_Os01g55360.2	147	-23.35	147	-23.35	337400	21	1897	725
miR399i	LOC_Os01g55360.3	147	-23.35	147	-23.35	337401	21	1806	725
miR399i	LOC_Os01g57280.1	169	-26.68	169	-26.68	337649	21	4172	1320
miR399i	LOC_Os01g57280.2	169	-26.68	169	-26.68	337650	21	4423	1571
miR399i	LOC_Os01g57280.3	169	-26.68	169	-26.68	337651	21	4944	1571
miR399i	LOC_Os01g57810.1	157	-25.26	157	-25.26	337715	21	2337	2033
miR399i	LOC_Os01g58400.1	155	-21.88	155	-21.88	337784	21	2032	1116
miR399i	LOC_Os01g58400.2	155	-21.88	155	-21.88	337785	21	1999	1116

miR399i	LOC_Os01g58470.1	150	-26.44	150	-26.44	337794	21	1126	436
miR399i	LOC_Os01g58740.1	157	-20.26	157	-20.26	337824	21	1989	1135
miR399i	LOC_Os01g58740.2	157	-20.26	157	-20.26	337825	21	2188	1041
miR399i	LOC_Os01g59060.1	152	-20.66	152	-20.66	337859	21	756	262
miR399i	LOC_Os01g59670.1	151	-21.93	151	-21.93	337932	21	4538	723
miR399i	LOC_Os01g60520.1	162	-30.93	162	-30.93	338028	21	852	100
miR399i	LOC_Os01g61710.1	150	-22.8	150	-22.8	338162	21	1842	1041
miR399i	LOC_Os01g61710.2	150	-22.8	150	-22.8	338163	21	1954	1153
miR399i	LOC_Os01g61710.3	150	-22.8	150	-22.8	338164	21	2143	1153
miR399i	LOC_Os01g61710.4	150	-22.8	150	-22.8	338165	21	1928	1153
miR399i	LOC_Os01g61710.5	150	-22.8	150	-22.8	338166	21	2224	1151
miR399i	LOC_Os01g61710.6	150	-22.8	150	-22.8	338167	21	2134	1153
miR399i	LOC_Os01g62100.1	158	-30.13	158	-30.13	338229	21	5409	5011
miR399i	LOC_Os01g62340.1	157	-25.26	157	-25.26	338255	21	2397	2210
miR399i	LOC_Os01g62440.1	149	-20.63	149	-20.63	338273	21	3075	2156
miR399i	LOC_Os01g62440.2	149	-20.63	149	-20.63	338274	21	2005	1086
miR399i	LOC_Os01g62440.3	149	-20.63	149	-20.63	338275	21	3154	2235
miR399i	LOC_Os01g63400.1	162	-21.19	162	-21.19	338389	21	3097	2893
miR399i	LOC_Os01g63400.2	162	-21.19	162	-21.19	338390	21	3190	2988
miR399i	LOC_Os01g63410.1	162	-21.19	162	-21.19	338391	21	1378	1176
miR399i	LOC_Os01g63710.1	147	-20.1	147	-20.1	338420	21	2163	112
miR399i	LOC_Os01g63710.3	147	-20.1	147	-20.1	338422	21	3745	1579
miR399i	LOC_Os01g63720.1	163	-22.48	163	-22.48	338423	21	258	218
miR399i	LOC_Os01g64270.1	149	-20.38	149	-20.38	338502	21	1214	1069
miR399i	LOC_Os01g64280.1	149	-20.38	149	-20.38	338503	21	2580	1254
miR399i	LOC_Os01g65190.1	167	-25.02	167	-25.02	338622	21	2156	1185
miR399i	LOC_Os01g65230.1	151	-20.29	151	-20.29	338628	21	2258	419
miR399i	LOC_Os01g65320.1	302	-40.52	152	-20.45	338635	21	4563	3809 4538
miR399i	LOC_Os01g65330.1	162	-23.52	162	-23.52	338636	21	2336	336
miR399i	LOC_Os01g65330.2	162	-23.52	162	-23.52	338637	21	2422	336
miR399i	LOC_Os01g65460.1	150	-27.58	150	-27.58	338654	21	3664	1062
miR399i	LOC_Os01g65460.2	150	-27.58	150	-27.58	338655	21	3787	1062

miR399i	LOC_Os01g66840.1	151	-20.5	151	-20.5	338812	21	1203	451
miR399i	LOC_Os01g66910.1	169	-33.46	169	-33.46	338822	21	378	245
miR399i	LOC_Os01g67170.1	157	-28.3	157	-28.3	338853	21	2118	1129
miR399i	LOC_Os01g68179.1	182	-33.24	182	-33.24	338982	21	743	658
miR399i	LOC_Os01g68200.1	154	-20.29	154	-20.29	338983	21	915	827
miR399i	LOC_Os01g68490.1	154	-25.24	154	-25.24	339020	21	4040	2386
miR399i	LOC_Os01g68750.1	146	-20.44	146	-20.44	339052	21	3582	852
miR399i	LOC_Os01g70260.1	165	-24.35	165	-24.35	339181	21	2715	2532
miR399i	LOC_Os01g70260.2	165	-24.35	165	-24.35	339182	21	2867	2684
miR399i	LOC_Os01g70320.1	163	-22.99	163	-22.99	339194	21	2213	1185
miR399i	LOC_Os01g70630.1	161	-21.83	161	-21.83	339233	21	3217	2703
miR399i	LOC_Os01g70710.1	165	-24	165	-24	339242	21	1669	1036
miR399i	LOC_Os01g70710.2	165	-24	165	-24	339243	21	1917	1015
miR399i	LOC_Os01g71100.1	146	-22.43	146	-22.43	339293	21	3575	1054
miR399i	LOC_Os01g71106.2	157	-22.57	157	-22.57	339294	21	4440	888
miR399i	LOC_Os01g71630.1	143	-21.56	143	-21.56	339355	21	2405	1113
miR399i	LOC_Os01g71630.2	143	-21.56	143	-21.56	339356	21	2437	1145
miR399i	LOC_Os01g71630.4	143	-21.56	143	-21.56	339357	21	2432	1140
miR399i	LOC_Os01g72350.1	145	-23.43	145	-23.43	339432	21	2418	1553
miR399i	LOC_Os01g72350.2	145	-23.43	145	-23.43	339433	21	2378	1553
miR399i	LOC_Os01g72870.1	146	-20.27	146	-20.27	339494	21	1319	952
miR399i	LOC_Os01g72870.2	146	-20.27	146	-20.27	339495	21	1271	952
miR399i	LOC_Os01g72870.3	146	-20.27	146	-20.27	339496	21	1217	952
miR399i	LOC_Os01g73000.1	163	-24.82	163	-24.82	339512	21	2202	999
miR399i	LOC_Os01g73000.2	163	-24.82	163	-24.82	339513	21	1983	780
miR399i	LOC_Os01g73250.1	156	-21.48	156	-21.48	339541	21	1340	1186
miR399i	LOC_Os01g73380.1	159	-20.6	159	-20.6	339553	21	3393	3057
miR399i	LOC_Os01g73410.1	150	-26.17	150	-26.17	339556	21	3788	1997
miR399i	LOC_Os01g73680.1	144	-22.76	144	-22.76	339590	21	2524	618
miR399i	LOC_Os01g74460.1	160	-21.4	160	-21.4	339683	21	2281	2173
miR399i	LOC_Os01g74460.2	160	-21.4	160	-21.4	339684	21	2364	2256
miR399i	LOC_Os01g74530.2	153	-21.49	153	-21.49	339692	21	2557	2088

miR399i	LOC_Os02g01220.1	151	-22.2	151	-22.2	339746	21	1369	1338
miR399i	LOC_Os02g01230.1	151	-22.2	151	-22.2	339747	21	2378	836
miR399i	LOC_Os02g01230.2	151	-22.2	151	-22.2	339748	21	2257	715
miR399i	LOC_Os02g01490.1	147	-22.11	147	-22.11	339779	21	4545	414
miR399i	LOC_Os02g01640.1	156	-24.57	156	-24.57	339796	21	4461	2881
miR399i	LOC_Os02g01670.1	156	-24.57	156	-24.57	339798	21	4461	2881
miR399i	LOC_Os02g02700.1	162	-25.5	162	-25.5	339918	21	2034	2008
miR399i	LOC_Os02g02810.1	152	-22.44	152	-22.44	339938	21	1170	545
miR399i	LOC_Os02g02970.1	149	-20.98	149	-20.98	339962	21	1801	314
miR399i	LOC_Os02g03540.1	168	-29.86	168	-29.86	340037	21	1456	503
miR399i	LOC_Os02g03980.1	164	-25.55	164	-25.55	340097	21	1185	752
miR399i	LOC_Os02g04230.1	162	-20.14	162	-20.14	340132	21	2104	1463
miR399i	LOC_Os02g04230.2	162	-20.14	162	-20.14	340133	21	2004	1463
miR399i	LOC_Os02g04230.3	162	-20.14	162	-20.14	340134	21	2021	1463
miR399i	LOC_Os02g04590.1	174	-32.7	174	-32.7	340184	21	429	244
miR399i	LOC_Os02g04630.1	167	-23.63	167	-23.63	340188	21	1089	304
miR399i	LOC_Os02g04850.1	165	-26.49	165	-26.49	340221	21	1535	1496
miR399i	LOC_Os02g05510.1	163	-24.96	163	-24.96	340306	21	1469	1296
miR399i	LOC_Os02g05510.3	163	-24.96	163	-24.96	340307	21	1397	1224
miR399i	LOC_Os02g06060.1	157	-26.48	157	-26.48	340371	21	255	184
miR399i	LOC_Os02g06570.1	149	-23.34	149	-23.34	340427	21	2420	1335
miR399i	LOC_Os02g06570.2	149	-23.34	149	-23.34	340428	21	2492	1335
miR399i	LOC_Os02g06605.1	159	-21.89	159	-21.89	340436	21	781	625
miR399i	LOC_Os02g06910.1	151	-20.86	151	-20.86	340473	21	3929	3757
miR399i	LOC_Os02g07540.1	153	-21.08	153	-21.08	340560	21	5895	4111
miR399i	LOC_Os02g07654.1	160	-21.58	160	-21.58	340574	21	795	518
miR399i	LOC_Os02g08130.1	168	-25	168	-25	340641	21	2798	1365
miR399i	LOC_Os02g08130.2	168	-25	168	-25	340642	21	4810	1365
miR399i	LOC_Os02g08130.3	168	-25	168	-25	340643	21	4638	1348
miR399i	LOC_Os02g09960.1	156	-21.21	156	-21.21	340800	21	2372	928
miR399i	LOC_Os02g10400.1	150	-21.18	150	-21.18	340856	21	1446	643
miR399i	LOC_Os02g10990.1	150	-22.43	150	-22.43	340933	21	1963	975

miR399i	LOC_Os02g12660.1	144	-21.53	144	-21.53	341080	21	2993	2118
miR399i	LOC_Os02g12730.1	155	-21.74	155	-21.74	341087	21	3169	1489
miR399i	LOC_Os02g12730.2	155	-21.74	155	-21.74	341088	21	2319	999
miR399i	LOC_Os02g12730.3	155	-21.74	155	-21.74	341089	21	3257	1577
miR399i	LOC_Os02g13640.1	158	-22.14	158	-22.14	341206	21	1645	926
miR399i	LOC_Os02g13960.1	143	-21.86	143	-21.86	341240	21	3311	2398
miR399i	LOC_Os02g14130.1	156	-21.74	156	-21.74	341262	21	2112	653
miR399i	LOC_Os02g14210.1	153	-24.89	153	-24.89	341270	21	1979	833
miR399i	LOC_Os02g14340.1	150	-21.11	150	-21.11	341283	21	576	148
miR399i	LOC_Os02g15310.1	150	-20.28	150	-20.28	341395	21	1269	920
miR399i	LOC_Os02g15310.2	150	-20.28	150	-20.28	341396	21	2241	1891
miR399i	LOC_Os02g15310.3	150	-20.28	150	-20.28	341397	21	1822	1473
miR399i	LOC_Os02g15310.4	150	-20.28	150	-20.28	341398	21	2071	1721
miR399i	LOC_Os02g15550.1	162	-23.85	162	-23.85	341422	21	2139	552
miR399i	LOC_Os02g15550.2	162	-23.85	162	-23.85	341423	21	1699	552
miR399i	LOC_Os02g15550.3	162	-23.85	162	-23.85	341424	21	1953	552
miR399i	LOC_Os02g16270.1	151	-22.52	151	-22.52	341508	21	4510	4425
miR399i	LOC_Os02g16270.2	151	-22.52	151	-22.52	341509	21	4520	4435
miR399i	LOC_Os02g17980.1	166	-25.16	166	-25.16	341694	21	1725	308
miR399i	LOC_Os02g18080.1	154	-21.05	154	-21.05	341708	21	4117	3140
miR399i	LOC_Os02g18090.1	158	-20.47	158	-20.47	341709	21	1128	370
miR399i	LOC_Os02g18140.1	147	-20.81	147	-20.81	341715	21	2709	1219
miR399i	LOC_Os02g18210.1	314	-41.1	157	-20.97	341724	21	2970	2578 2631
miR399i	LOC_Os02g18240.1	155	-24.66	155	-24.66	341727	21	1224	848
miR399i	LOC_Os02g18760.1	151	-24.91	151	-24.91	341781	21	2733	2371
miR399i	LOC_Os02g19100.1	154	-20.54	154	-20.54	341817	21	2301	1177
miR399i	LOC_Os02g19170.1	147	-22.43	147	-22.43	341823	21	2509	771
miR399i	LOC_Os02g19170.2	147	-22.43	147	-22.43	341824	21	2576	838
miR399i	LOC_Os02g19170.3	147	-22.43	147	-22.43	341825	21	2524	824
miR399i	LOC_Os02g19320.1	157	-21.4	157	-21.4	341841	21	426	259
miR399i	LOC_Os02g19550.1	158	-22.47	158	-22.47	341868	21	2284	1699
miR399i	LOC_Os02g19690.1	150	-25.46	150	-25.46	341881	21	732	430

miR399i	LOC_Os02g19780.1	319	-48.94	168	-26	341890	21	537	227 489
miR399i	LOC_Os02g20100.1	318	-46.13	161	-25.26	341921	21	5415	4036 5045
miR399i	LOC_Os02g20120.1	157	-25.26	157	-25.26	341923	21	2205	1925
miR399i	LOC_Os02g20450.1	157	-22.71	157	-22.71	341958	21	462	378
miR399i	LOC_Os02g20460.1	157	-29.89	157	-29.89	341959	21	2103	1390
miR399i	LOC_Os02g20570.1	153	-20.95	153	-20.95	341971	21	3369	40
miR399i	LOC_Os02g20640.1	159	-25.65	159	-25.65	341976	21	1230	33
miR399i	LOC_Os02g21530.1	144	-22.74	144	-22.74	342075	21	609	114
miR399i	LOC_Os02g21570.1	140	-21.12	140	-21.12	342079	21	671	161
miR399i	LOC_Os02g22000.1	150	-20.01	150	-20.01	342126	21	564	138
miR399i	LOC_Os02g22120.1	154	-20.97	154	-20.97	342146	21	675	216
miR399i	LOC_Os02g22210.1	327	-47.53	164	-26.1	342154	21	3396	2981 1248
miR399i	LOC_Os02g22260.1	159	-21.12	159	-21.12	342159	21	1324	986
miR399i	LOC_Os02g22260.2	159	-21.12	159	-21.12	342160	21	1644	1306
miR399i	LOC_Os02g22320.1	149	-27.19	149	-27.19	342164	21	837	90
miR399i	LOC_Os02g24090.1	321	-46.66	163	-25.96	342267	21	4725	2759 52
miR399i	LOC_Os02g25560.1	166	-24.42	166	-24.42	342419	21	2388	1454
miR399i	LOC_Os02g26060.1	158	-20.06	158	-20.06	342470	21	3768	3112
miR399i	LOC_Os02g26290.1	154	-24.11	154	-24.11	342494	21	1066	383
miR399i	LOC_Os02g26370.1	156	-23.63	156	-23.63	342509	21	1491	768
miR399i	LOC_Os02g27650.1	318	-42.45	161	-21.58	342666	21	2781	1105 1156
miR399i	LOC_Os02g28780.1	157	-20.91	157	-20.91	342775	21	3483	2503
miR399i	LOC_Os02g29030.1	146	-22.48	146	-22.48	342801	21	5010	204
miR399i	LOC_Os02g29040.1	157	-20.1	157	-20.1	342802	21	1636	439
miR399i	LOC_Os02g29040.2	157	-20.1	157	-20.1	342803	21	1597	439
miR399i	LOC_Os02g29190.1	158	-21.63	158	-21.63	342819	21	1395	171
miR399i	LOC_Os02g29210.1	158	-21.63	158	-21.63	342821	21	1674	664
miR399i	LOC_Os02g29210.2	158	-21.63	158	-21.63	342822	21	1600	637
miR399i	LOC_Os02g29210.3	158	-21.63	158	-21.63	342823	21	2226	637
miR399i	LOC_Os02g29210.4	158	-21.63	158	-21.63	342824	21	1603	637
miR399i	LOC_Os02g29380.1	157	-21.25	157	-21.25	342840	21	3992	3713
miR399i	LOC_Os02g29480.1	157	-22.57	157	-22.57	342848	21	2123	1202

miR399i	LOC_Os02g30040.1	157	-20.91	157	-20.91	342907	21	2751	1150
miR399i	LOC_Os02g30060.1	152	-21.78	152	-21.78	342909	21	1110	1060
miR399i	LOC_Os02g30290.1	149	-20.5	149	-20.5	342939	21	4161	1805
miR399i	LOC_Os02g30730.1	155	-22.27	155	-22.27	342992	21	3090	666
miR399i	LOC_Os02g30730.2	155	-22.27	155	-22.27	342993	21	3164	740
miR399i	LOC_Os02g31180.1	152	-21.4	152	-21.4	343042	21	5520	4189
miR399i	LOC_Os02g31926.1	152	-22.13	152	-22.13	343073	21	1071	464
miR399i	LOC_Os02g31960.1	165	-25.07	165	-25.07	343076	21	1834	643
miR399i	LOC_Os02g31960.3	165	-25.07	165	-25.07	343077	21	1739	643
miR399i	LOC_Os02g31960.4	165	-25.07	165	-25.07	343078	21	1819	643
miR399i	LOC_Os02g32220.1	151	-20.86	151	-20.86	343105	21	732	706
miR399i	LOC_Os02g32465.1	158	-20.05	158	-20.05	343133	21	447	369
miR399i	LOC_Os02g32880.1	151	-21.18	151	-21.18	343184	21	6558	4296
miR399i	LOC_Os02g33280.1	157	-20.97	157	-20.97	343228	21	3936	2536
miR399i	LOC_Os02g33490.1	156	-21.25	156	-21.25	343254	21	3299	655
miR399i	LOC_Os02g34490.1	150	-21.96	150	-21.96	343349	21	3225	2263
miR399i	LOC_Os02g34650.1	157	-22.77	157	-22.77	343369	21	1820	1592
miR399i	LOC_Os02g34660.1	140	-23.66	140	-23.66	343370	21	2790	2391
miR399i	LOC_Os02g35190.1	145	-23.3	145	-23.3	343433	21	2934	379
miR399i	LOC_Os02g35190.2	145	-23.3	145	-23.3	343434	21	2896	379
miR399i	LOC_Os02g35230.1	148	-21.61	148	-21.61	343438	21	2360	463
miR399i	LOC_Os02g35290.1	314	-41.1	157	-20.97	343444	21	4305	3479 3532
miR399i	LOC_Os02g35820.1	147	-21.75	147	-21.75	343503	21	4812	1111
miR399i	LOC_Os02g35820.2	147	-21.75	147	-21.75	343504	21	4518	1111
miR399i	LOC_Os02g36340.1	162	-22.19	162	-22.19	343559	21	2247	389
miR399i	LOC_Os02g36340.2	162	-22.19	162	-22.19	343560	21	2220	389
miR399i	LOC_Os02g37960.1	155	-22.69	155	-22.69	343766	21	1836	857
miR399i	LOC_Os02g38640.1	147	-20.09	147	-20.09	343849	21	873	167
miR399i	LOC_Os02g38790.1	150	-21.18	150	-21.18	343866	21	1416	1084
miR399i	LOC_Os02g39064.1	153	-20.79	153	-20.79	343905	21	525	25
miR399i	LOC_Os02g39764.1	158	-21.64	158	-21.64	343999	21	1338	944
miR399i	LOC_Os02g40450.1	156	-22.54	156	-22.54	344090	21	3946	945

miR399i	LOC_Os02g41590.1	166	-26.56	166	-26.56	344168	21	1736	761
miR399i	LOC_Os02g41890.1	161	-22.55	161	-22.55	344203	21	3612	458
miR399i	LOC_Os02g42040.1	164	-24.01	164	-24.01	344222	21	4437	3312
miR399i	LOC_Os02g42180.1	153	-27.56	153	-27.56	344236	21	4578	2829
miR399i	LOC_Os02g42330.1	154	-21.79	154	-21.79	344260	21	1747	375
miR399i	LOC_Os02g42330.2	154	-21.79	154	-21.79	344261	21	1498	306
miR399i	LOC_Os02g42950.1	149	-26.74	149	-26.74	344335	21	1971	915
miR399i	LOC_Os02g42950.2	149	-26.74	149	-26.74	344336	21	1409	915
miR399i	LOC_Os02g43170.2	151	-21.72	151	-21.72	344365	21	1610	842
miR399i	LOC_Os02g43860.1	162	-22.93	162	-22.93	344447	21	2416	1062
miR399i	LOC_Os02g43860.2	162	-22.93	162	-22.93	344448	21	2677	1062
miR399i	LOC_Os02g44642.1	159	-21.59	159	-21.59	344565	21	3757	3220
miR399i	LOC_Os02g44720.1	151	-21.54	151	-21.54	344575	21	851	188
miR399i	LOC_Os02g44870.1	158	-22.31	158	-22.31	344595	21	1371	1056
miR399i	LOC_Os02g45150.2	157	-20.38	157	-20.38	344632	21	2026	620
miR399i	LOC_Os02g45390.1	148	-33.67	148	-33.67	344657	21	1261	1041
miR399i	LOC_Os02g46030.1	303	-45.11	152	-24.11	344730	21	2083	1068 1715
miR399i	LOC_Os02g46962.1	152	-26.44	152	-26.44	344844	21	1790	329
miR399i	LOC_Os02g46962.2	152	-26.44	152	-26.44	344845	21	1702	241
miR399i	LOC_Os02g46962.3	152	-26.44	152	-26.44	344846	21	1530	69
miR399i	LOC_Os02g47380.1	142	-23.41	142	-23.41	344897	21	2868	844
miR399i	LOC_Os02g47390.1	158	-23.34	158	-23.34	344898	21	3305	2608
miR399i	LOC_Os02g47570.1	161	-22.68	161	-22.68	344926	21	1219	243
miR399i	LOC_Os02g47570.2	161	-22.68	161	-22.68	344927	21	940	243
miR399i	LOC_Os02g48340.1	151	-21	151	-21	345022	21	2237	619
miR399i	LOC_Os02g48340.2	151	-21	151	-21	345023	21	2213	592
miR399i	LOC_Os02g48340.3	151	-21	151	-21	345024	21	2210	592
miR399i	LOC_Os02g48340.5	151	-21	151	-21	345025	21	1719	592
miR399i	LOC_Os02g48340.6	151	-21	151	-21	345026	21	2304	592
miR399i	LOC_Os02g48900.1	157	-20.44	157	-20.44	345098	21	2024	1794
miR399i	LOC_Os02g48900.2	157	-20.44	157	-20.44	345099	21	2565	2335
miR399i	LOC_Os02g49050.1	151	-21.12	151	-21.12	345112	21	2523	1622

miR399i	LOC_Os02g49050.2	151	-21.12	151	-21.12	345113	21	2501	1615
miR399i	LOC_Os02g49280.1	165	-24.15	165	-24.15	345145	21	786	263
miR399i	LOC_Os02g49320.1	155	-20.54	155	-20.54	345149	21	1710	618
miR399i	LOC_Os02g49332.2	159	-21.36	159	-21.36	345152	21	4273	1427
miR399i	LOC_Os02g49332.3	159	-21.36	159	-21.36	345153	21	4042	1419
miR399i	LOC_Os02g49670.1	173	-34.32	173	-34.32	345193	21	4050	3247
miR399i	LOC_Os02g49730.1	161	-21.9	161	-21.9	345205	21	1452	487
miR399i	LOC_Os02g49800.1	150	-20.77	150	-20.77	345215	21	2215	1328
miR399i	LOC_Os02g50380.1	160	-21.06	160	-21.06	345283	21	414	130
miR399i	LOC_Os02g50530.1	164	-21.43	164	-21.43	345299	21	2460	1904
miR399i	LOC_Os02g50540.1	149	-22.01	149	-22.01	345300	21	1735	1348
miR399i	LOC_Os02g50570.1	150	-25.11	150	-25.11	345304	21	3448	2037
miR399i	LOC_Os02g52120.1	155	-24.66	155	-24.66	345496	21	354	121
miR399i	LOC_Os02g52380.1	150	-24.01	150	-24.01	345535	21	1623	793
miR399i	LOC_Os02g52744.1	147	-23.22	147	-23.22	345576	21	1127	815
miR399i	LOC_Os02g52744.2	147	-23.22	147	-23.22	345577	21	1123	811
miR399i	LOC_Os02g53850.1	161	-22.33	161	-22.33	345727	21	2187	1379
miR399i	LOC_Os02g54100.1	147	-21.26	147	-21.26	345755	21	2516	1296
miR399i	LOC_Os02g54810.1	174	-34.8	174	-34.8	345836	21	453	87
miR399i	LOC_Os02g55010.1	158	-23.19	158	-23.19	345859	21	3772	3082
miR399i	LOC_Os02g56740.1	157	-22.26	157	-22.26	346074	21	3142	3004
miR399i	LOC_Os02g56740.2	157	-22.26	157	-22.26	346075	21	3244	3106
miR399i	LOC_Os02g57340.1	158	-24.14	158	-24.14	346161	21	3061	1658
miR399i	LOC_Os02g57670.1	150	-20.57	150	-20.57	346220	21	1087	811
miR399i	LOC_Os02g57880.1	149	-20.93	149	-20.93	346250	21	1172	1056
miR399i	LOC_Os02g58030.1	154	-24.35	154	-24.35	346270	21	2311	29
miR399i	LOC_Os02g58480.1	140	-21.49	140	-21.49	346321	21	3550	3200
miR399i	LOC_Os02g58490.1	140	-21.49	140	-21.49	346322	21	3647	299
miR399i	LOC_Os02g58710.1	161	-21.95	161	-21.95	346353	21	4191	3737
miR399i	LOC_Os03g02150.1	154	-24.56	154	-24.56	346532	21	3083	1740
miR399i	LOC_Os03g02150.2	154	-24.56	154	-24.56	346533	21	3870	1740
miR399i	LOC_Os03g02630.1	154	-20.95	154	-20.95	346598	21	672	24

miR399i	LOC_Os03g02890.1	152	-20.33	152	-20.33	346640	21	2373	1614
miR399i	LOC_Os03g03250.1	153	-20.56	153	-20.56	346680	21	5862	4192
miR399i	LOC_Os03g03420.1	173	-28.4	173	-28.4	346699	21	727	420
miR399i	LOC_Os03g03420.2	173	-28.4	173	-28.4	346700	21	814	420
miR399i	LOC_Os03g03650.1	164	-23.37	164	-23.37	346730	21	1985	824
miR399i	LOC_Os03g04560.1	155	-23.5	155	-23.5	346843	21	3431	1385
miR399i	LOC_Os03g05334.1	166	-30.63	166	-30.63	346938	21	656	356
miR399i	LOC_Os03g05350.1	153	-24.27	153	-24.27	346940	21	4386	2104
miR399i	LOC_Os03g06180.1	156	-20.88	156	-20.88	347049	21	2398	590
miR399i	LOC_Os03g06220.1	151	-20.67	151	-20.67	347053	21	2525	1461
miR399i	LOC_Os03g06420.2	159	-22.39	159	-22.39	347076	21	2487	2012
miR399i	LOC_Os03g06710.1	159	-21.62	159	-21.62	347112	21	3133	777
miR399i	LOC_Os03g07130.1	147	-21.99	147	-21.99	347164	21	2388	1311
miR399i	LOC_Os03g07130.2	147	-21.99	147	-21.99	347165	21	2181	1104
miR399i	LOC_Os03g07130.3	147	-21.99	147	-21.99	347166	21	2292	1311
miR399i	LOC_Os03g07130.4	147	-21.99	147	-21.99	347167	21	2167	1207
miR399i	LOC_Os03g07130.5	147	-21.99	147	-21.99	347168	21	1528	451
miR399i	LOC_Os03g07420.1	169	-25.72	169	-25.72	347202	21	1502	46
miR399i	LOC_Os03g07580.1	164	-21.09	164	-21.09	347228	21	4354	3081
miR399i	LOC_Os03g07620.1	156	-20.18	156	-20.18	347232	21	396	309
miR399i	LOC_Os03g08120.1	158	-22.13	158	-22.13	347289	21	1278	50
miR399i	LOC_Os03g08430.1	159	-20.7	159	-20.7	347333	21	4128	2432
miR399i	LOC_Os03g08430.2	159	-20.7	159	-20.7	347334	21	4125	2432
miR399i	LOC_Os03g09850.1	157	-20.59	157	-20.59	347456	21	1781	94
miR399i	LOC_Os03g09970.1	151	-21.29	151	-21.29	347470	21	2809	1818
miR399i	LOC_Os03g09970.2	151	-21.29	151	-21.29	347471	21	2621	1630
miR399i	LOC_Os03g09970.3	151	-21.29	151	-21.29	347472	21	2529	1538
miR399i	LOC_Os03g09970.4	151	-21.29	151	-21.29	347473	21	2536	1545
miR399i	LOC_Os03g11720.1	147	-20.35	147	-20.35	347685	21	3129	1916
miR399i	LOC_Os03g11780.1	152	-21.66	152	-21.66	347692	21	646	392
miR399i	LOC_Os03g11890.1	150	-22.88	150	-22.88	347704	21	5003	1165
miR399i	LOC_Os03g12180.1	152	-20.15	152	-20.15	347747	21	2587	2525

miR399i	LOC_Os03g12490.1	157	-20.13	157	-20.13	347791	21	2292	95
miR399i	LOC_Os03g12680.1	164	-26.18	164	-26.18	347815	21	1700	804
miR399i	LOC_Os03g12680.2	164	-26.18	164	-26.18	347816	21	1555	659
miR399i	LOC_Os03g13080.1	155	-22.1	155	-22.1	347874	21	1875	1020
miR399i	LOC_Os03g13460.1	145	-21.68	145	-21.68	347917	21	3291	2566
miR399i	LOC_Os03g13460.2	145	-21.68	145	-21.68	347918	21	3715	2990
miR399i	LOC_Os03g13960.2	155	-20.53	155	-20.53	347981	21	1370	1046
miR399i	LOC_Os03g15270.1	168	-24.68	168	-24.68	348141	21	1703	965
miR399i	LOC_Os03g15960.1	158	-23.5	158	-23.5	348232	21	1035	742
miR399i	LOC_Os03g15960.2	158	-23.5	158	-23.5	348233	21	993	700
miR399i	LOC_Os03g16540.1	154	-23.34	154	-23.34	348317	21	1536	1162
miR399i	LOC_Os03g16700.1	153	-23.16	153	-23.16	348334	21	2980	2616
miR399i	LOC_Os03g16700.2	153	-23.16	153	-23.16	348335	21	2808	2444
miR399i	LOC_Os03g16700.3	153	-23.16	153	-23.16	348336	21	2801	2437
miR399i	LOC_Os03g16700.4	153	-23.16	153	-23.16	348337	21	2967	2603
miR399i	LOC_Os03g17020.1	154	-21.96	154	-21.96	348385	21	1976	633
miR399i	LOC_Os03g17020.2	154	-21.96	154	-21.96	348386	21	2171	828
miR399i	LOC_Os03g18060.1	149	-20.5	149	-20.5	348508	21	5067	2840
miR399i	LOC_Os03g18440.1	149	-20.26	149	-20.26	348555	21	4114	3563
miR399i	LOC_Os03g18454.1	149	-20.26	149	-20.26	348556	21	2178	902
miR399i	LOC_Os03g18454.2	149	-20.26	149	-20.26	348557	21	2081	902
miR399i	LOC_Os03g18470.1	468	-68.5	158	-23.78	348558	21	1605	482 289 671
miR399i	LOC_Os03g19570.1	162	-23.16	162	-23.16	348686	21	3258	2395
miR399i	LOC_Os03g20060.1	152	-25.97	152	-25.97	348747	21	1887	418
miR399i	LOC_Os03g21090.1	159	-22.52	159	-22.52	348872	21	1949	484
miR399i	LOC_Os03g22490.1	159	-26.63	159	-26.63	349047	21	1667	973
miR399i	LOC_Os03g22490.2	159	-26.63	159	-26.63	349048	21	1766	973
miR399i	LOC_Os03g22490.3	159	-26.63	159	-26.63	349049	21	1731	973
miR399i	LOC_Os03g22490.4	159	-26.63	159	-26.63	349050	21	1309	973
miR399i	LOC_Os03g22560.1	331	-52.41	166	-26.3	349056	21	3248	891 973
miR399i	LOC_Os03g24410.1	154	-22.96	154	-22.96	349201	21	2274	1282
miR399i	LOC_Os03g24960.1	161	-21.81	161	-21.81	349258	21	1200	187

miR399i	LOC_Os03g25420.1	324	-47.96	162	-25.07	349314	21	3077	1009 1045
miR399i	LOC_Os03g26044.1	167	-22.62	167	-22.62	349380	21	3221	2645
miR399i	LOC_Os03g26080.1	162	-24.97	162	-24.97	349383	21	1899	1052
miR399i	LOC_Os03g26630.1	146	-20.48	146	-20.48	349430	21	2928	1099
miR399i	LOC_Os03g26630.2	146	-20.48	146	-20.48	349431	21	3026	1197
miR399i	LOC_Os03g27310.1	151	-23.28	151	-23.28	349502	21	935	741
miR399i	LOC_Os03g27310.2	151	-23.28	151	-23.28	349503	21	879	685
miR399i	LOC_Os03g27390.1	149	-20.6	149	-20.6	349518	21	2558	889
miR399i	LOC_Os03g27390.2	149	-20.6	149	-20.6	349519	21	2581	889
miR399i	LOC_Os03g27480.1	155	-22.72	155	-22.72	349531	21	1748	1548
miR399i	LOC_Os03g27480.2	155	-22.72	155	-22.72	349532	21	1582	1385
miR399i	LOC_Os03g27820.1	159	-24.84	159	-24.84	349565	21	3028	399
miR399i	LOC_Os03g27960.1	149	-26.59	149	-26.59	349580	21	2080	559
miR399i	LOC_Os03g27960.2	149	-26.59	149	-26.59	349581	21	2110	592
miR399i	LOC_Os03g28270.1	146	-20.91	146	-20.91	349611	21	4432	3301
miR399i	LOC_Os03g29220.1	157	-22.97	157	-22.97	349667	21	876	804
miR399i	LOC_Os03g29340.1	167	-24.85	167	-24.85	349680	21	3720	2205
miR399i	LOC_Os03g29340.2	167	-24.85	167	-24.85	349681	21	3756	2241
miR399i	LOC_Os03g29350.1	167	-24.85	167	-24.85	349682	21	2931	880
miR399i	LOC_Os03g29350.2	167	-24.85	167	-24.85	349683	21	2984	880
miR399i	LOC_Os03g29350.4	167	-24.85	167	-24.85	349684	21	2923	880
miR399i	LOC_Os03g29520.1	147	-21.73	147	-21.73	349698	21	5769	1648
miR399i	LOC_Os03g30510.1	160	-29.39	160	-29.39	349793	21	2442	690
miR399i	LOC_Os03g31520.1	165	-21.22	165	-21.22	349905	21	1536	477
miR399i	LOC_Os03g31750.1	158	-23.46	158	-23.46	349930	21	3082	2357
miR399i	LOC_Os03g31750.2	158	-23.46	158	-23.46	349931	21	2743	2357
miR399i	LOC_Os03g31750.3	158	-23.46	158	-23.46	349932	21	3097	2372
miR399i	LOC_Os03g31986.1	140	-20.83	140	-20.83	349949	21	3954	2861
miR399i	LOC_Os03g32620.1	149	-21.19	149	-21.19	350006	21	4010	3637
miR399i	LOC_Os03g33012.1	149	-22.08	149	-22.08	350044	21	1926	1862
miR399i	LOC_Os03g33770.1	149	-28.65	149	-28.65	350117	21	2397	298
miR399i	LOC_Os03g34102.1	149	-20.48	149	-20.48	350148	21	1107	37

miR399i	LOC_Os03g34170.1	152	-23.77	152	-23.77	350154	21	1167	1006
miR399i	LOC_Os03g34270.1	148	-21.42	148	-21.42	350164	21	2796	285
miR399i	LOC_Os03g35390.1	155	-23.01	155	-23.01	350184	21	5727	639
miR399i	LOC_Os03g35540.1	152	-20.4	152	-20.4	350196	21	4707	3580
miR399i	LOC_Os03g35886.1	155	-25.16	155	-25.16	350223	21	4339	182
miR399i	LOC_Os03g36560.1	141	-22.89	141	-22.89	350282	21	1497	1150
miR399i	LOC_Os03g36570.1	143	-21.52	143	-21.52	350283	21	6849	3642
miR399i	LOC_Os03g37500.1	153	-20.65	153	-20.65	350363	21	4860	3536
miR399i	LOC_Os03g37720.1	161	-21.12	161	-21.12	350383	21	3801	1372
miR399i	LOC_Os03g37760.1	163	-25.4	163	-25.4	350388	21	411	61
miR399i	LOC_Os03g38740.1	160	-21.09	160	-21.09	350493	21	5009	1403
miR399i	LOC_Os03g38750.1	159	-20.38	159	-20.38	350495	21	414	144
miR399i	LOC_Os03g39730.1	157	-25.26	157	-25.26	350595	21	1272	947
miR399i	LOC_Os03g39780.1	146	-21.78	146	-21.78	350599	21	2388	1337
miR399i	LOC_Os03g39850.1	161	-20.64	161	-20.64	350603	21	902	219
miR399i	LOC_Os03g40930.1	151	-24.35	151	-24.35	350696	21	2045	776
miR399i	LOC_Os03g41380.1	165	-23.13	165	-23.13	350736	21	861	548
miR399i	LOC_Os03g41510.1	146	-22.09	146	-22.09	350745	21	1670	1328
miR399i	LOC_Os03g41510.2	146	-22.09	146	-22.09	350746	21	1690	1348
miR399i	LOC_Os03g41510.3	146	-22.09	146	-22.09	350747	21	1698	1371
miR399i	LOC_Os03g41510.4	146	-22.09	146	-22.09	350748	21	1641	1299
miR399i	LOC_Os03g41510.5	146	-22.09	146	-22.09	350749	21	1683	1341
miR399i	LOC_Os03g41510.6	146	-22.09	146	-22.09	350750	21	1646	1319
miR399i	LOC_Os03g41510.7	146	-22.09	146	-22.09	350751	21	1757	1430
miR399i	LOC_Os03g41510.8	146	-22.09	146	-22.09	350752	21	1991	1649
miR399i	LOC_Os03g41710.1	157	-20.24	157	-20.24	350770	21	1881	107
miR399i	LOC_Os03g41830.1	155	-24.7	155	-24.7	350780	21	2730	969
miR399i	LOC_Os03g42180.1	152	-21.52	152	-21.52	350813	21	369	307
miR399i	LOC_Os03g43800.1	152	-20.17	152	-20.17	350983	21	4253	945
miR399i	LOC_Os03g43800.2	152	-20.17	152	-20.17	350984	21	4224	945
miR399i	LOC_Os03g44240.1	154	-22.64	154	-22.64	351030	21	3066	2870
miR399i	LOC_Os03g44530.1	148	-20.87	148	-20.87	351063	21	2162	1329

miR399i	LOC_Os03g44530.2	148	-20.87	148	-20.87	351064	21	2123	1290
miR399i	LOC_Os03g45220.1	145	-24.36	145	-24.36	351144	21	604	199
miR399i	LOC_Os03g45970.1	157	-21.18	157	-21.18	351212	21	2513	1780
miR399i	LOC_Os03g45970.2	157	-21.18	157	-21.18	351213	21	2190	1457
miR399i	LOC_Os03g45970.3	157	-21.18	157	-21.18	351214	21	2358	1625
miR399i	LOC_Os03g45990.1	168	-30.2	168	-30.2	351216	21	4865	4568
miR399i	LOC_Os03g46340.1	155	-21.23	155	-21.23	351257	21	3131	2658
miR399i	LOC_Os03g46930.1	147	-23.52	147	-23.52	351319	21	384	307
miR399i	LOC_Os03g47390.1	163	-23.94	163	-23.94	351364	21	282	163
miR399i	LOC_Os03g47650.1	159	-20.9	159	-20.9	351399	21	1824	415
miR399i	LOC_Os03g48450.1	148	-20.17	148	-20.17	351489	21	3638	275
miR399i	LOC_Os03g48450.3	148	-20.17	148	-20.17	351491	21	3022	275
miR399i	LOC_Os03g48450.4	148	-20.17	148	-20.17	351492	21	3112	275
miR399i	LOC_Os03g48970.1	156	-21.44	156	-21.44	351555	21	1594	497
miR399i	LOC_Os03g48970.2	156	-21.44	156	-21.44	351556	21	2123	1026
miR399i	LOC_Os03g48970.3	156	-21.44	156	-21.44	351557	21	2293	1196
miR399i	LOC_Os03g48970.4	156	-21.44	156	-21.44	351558	21	1586	497
miR399i	LOC_Os03g49340.1	160	-21.42	160	-21.42	351599	21	1698	994
miR399i	LOC_Os03g49524.1	159	-20.8	159	-20.8	351627	21	2574	332
miR399i	LOC_Os03g49640.1	152	-20.21	152	-20.21	351649	21	2537	1644
miR399i	LOC_Os03g49640.2	152	-20.21	152	-20.21	351650	21	2631	1738
miR399i	LOC_Os03g50670.1	158	-21.12	158	-21.12	351765	21	4350	1346
miR399i	LOC_Os03g51440.1	155	-21.25	155	-21.25	351843	21	3315	1375
miR399i	LOC_Os03g51570.1	160	-25.05	160	-25.05	351862	21	255	197
miR399i	LOC_Os03g51990.1	158	-26.37	158	-26.37	351910	21	1656	1616
miR399i	LOC_Os03g52700.1	156	-24.76	156	-24.76	351995	21	3359	2829
miR399i	LOC_Os03g52780.1	156	-21.36	156	-21.36	352003	21	1886	1238
miR399i	LOC_Os03g52950.1	159	-25.46	159	-25.46	352021	21	3183	2179
miR399i	LOC_Os03g53280.1	158	-21.12	158	-21.12	352059	21	11747	864
miR399i	LOC_Os03g53750.1	160	-24.04	160	-24.04	352118	21	1878	537
miR399i	LOC_Os03g53750.2	160	-24.04	160	-24.04	352119	21	1861	537
miR399i	LOC_Os03g55820.1	150	-20.07	150	-20.07	352319	21	1361	1039

miR399i	LOC_Os03g55890.1	150	-21.36	150	-21.36	352328	21	2451	1760
miR399i	LOC_Os03g56530.1	147	-20.6	147	-20.6	352407	21	4185	3300
miR399i	LOC_Os03g56572.1	161	-21.95	161	-21.95	352411	21	3957	2690
miR399i	LOC_Os03g56890.1	155	-21.96	155	-21.96	352457	21	767	713
miR399i	LOC_Os03g58190.1	166	-25.9	166	-25.9	352633	21	1542	1466
miR399i	LOC_Os03g58240.1	157	-21.05	157	-21.05	352638	21	2646	920
miR399i	LOC_Os03g58490.1	153	-22.09	153	-22.09	352663	21	2903	537
miR399i	LOC_Os03g58490.2	153	-22.09	153	-22.09	352664	21	2935	527
miR399i	LOC_Os03g58890.1	158	-20.25	158	-20.25	352707	21	1162	537
miR399i	LOC_Os03g58890.2	158	-20.25	158	-20.25	352708	21	1168	537
miR399i	LOC_Os03g59680.1	163	-22.43	163	-22.43	352792	21	2266	949
miR399i	LOC_Os03g59730.1	146	-20.82	146	-20.82	352797	21	1491	1427
miR399i	LOC_Os03g60460.1	151	-22.5	151	-22.5	352874	21	2197	1315
miR399i	LOC_Os03g60740.1	155	-21.57	155	-21.57	352909	21	1114	721
miR399i	LOC_Os03g60750.1	161	-20.64	161	-20.64	352910	21	1399	1121
miR399i	LOC_Os03g60750.2	161	-20.64	161	-20.64	352911	21	1488	1210
miR399i	LOC_Os03g60750.3	161	-20.64	161	-20.64	352912	21	1474	1196
miR399i	LOC_Os03g60920.1	158	-21.87	158	-21.87	352933	21	1431	940
miR399i	LOC_Os03g61130.1	163	-26.69	163	-26.69	352954	21	2206	795
miR399i	LOC_Os03g61380.1	153	-24.9	153	-24.9	352992	21	987	879
miR399i	LOC_Os03g61860.1	143	-21.55	143	-21.55	353047	21	630	324
miR399i	LOC_Os03g61950.1	155	-20.65	155	-20.65	353060	21	2690	464
miR399i	LOC_Os03g61950.2	155	-20.65	155	-20.65	353061	21	3095	464
miR399i	LOC_Os03g62314.1	154	-20.8	154	-20.8	353114	21	2010	1578
miR399i	LOC_Os03g64210.1	157	-20.51	157	-20.51	353359	21	2439	523
miR399i	LOC_Os03g64210.2	157	-20.51	157	-20.51	353360	21	2599	523
miR399i	LOC_Os03g64210.3	157	-20.51	157	-20.51	353361	21	2401	523
miR399i	LOC_Os04g01240.1	162	-20.91	162	-20.91	353418	21	1043	346
miR399i	LOC_Os04g01340.1	153	-24.96	153	-24.96	353435	21	2100	713
miR399i	LOC_Os04g02140.1	171	-29.98	171	-29.98	353523	21	1958	1497
miR399i	LOC_Os04g02500.1	149	-22.06	149	-22.06	353557	21	1751	41
miR399i	LOC_Os04g02820.1	156	-22.79	156	-22.79	353592	21	3228	1578

miR399i	LOC_Os04g02820.2	156	-22.79	156	-22.79	353593	21	3000	1350
miR399i	LOC_Os04g02820.3	156	-22.79	156	-22.79	353594	21	2556	1352
miR399i	LOC_Os04g02860.1	153	-20.63	153	-20.63	353598	21	2928	1131
miR399i	LOC_Os04g03110.1	141	-25.41	141	-25.41	353626	21	4098	3435
miR399i	LOC_Os04g03230.1	155	-23.06	155	-23.06	353637	21	525	47
miR399i	LOC_Os04g03300.1	155	-23.06	155	-23.06	353644	21	3981	3949
miR399i	LOC_Os04g03630.1	162	-20.75	162	-20.75	353673	21	4899	3592
miR399i	LOC_Os04g03950.1	157	-22.93	157	-22.93	353705	21	2376	1321
miR399i	LOC_Os04g04020.1	156	-26.5	156	-26.5	353713	21	3676	468
miR399i	LOC_Os04g05180.1	155	-24.66	155	-24.66	353822	21	1530	1142
miR399i	LOC_Os04g05210.1	156	-20.19	156	-20.19	353825	21	2511	2084
miR399i	LOC_Os04g05470.1	149	-23.2	149	-23.2	353848	21	2076	1435
miR399i	LOC_Os04g06610.1	159	-20.27	159	-20.27	353956	21	4362	1664
miR399i	LOC_Os04g07020.1	153	-21.08	153	-21.08	353993	21	5898	4099
miR399i	LOC_Os04g07300.1	153	-21.08	153	-21.08	354017	21	5997	4201
miR399i	LOC_Os04g07400.1	162	-21.61	162	-21.61	354027	21	816	262
miR399i	LOC_Os04g07510.1	151	-21.76	151	-21.76	354037	21	4209	2122
miR399i	LOC_Os04g09284.1	156	-22.81	156	-22.81	354186	21	4470	3382
miR399i	LOC_Os04g09350.1	167	-22.11	167	-22.11	354191	21	5391	1297
miR399i	LOC_Os04g09380.1	153	-20.49	153	-20.49	354197	21	2934	40
miR399i	LOC_Os04g09690.1	165	-25.01	165	-25.01	354224	21	2172	268
miR399i	LOC_Os04g09720.1	159	-23.94	159	-23.94	354227	21	1693	277
miR399i	LOC_Os04g09720.2	159	-23.94	159	-23.94	354228	21	1615	197
miR399i	LOC_Os04g09750.1	150	-23.56	150	-23.56	354231	21	903	644
miR399i	LOC_Os04g10090.1	149	-20.48	149	-20.48	354263	21	447	37
miR399i	LOC_Os04g10200.1	153	-20.56	153	-20.56	354274	21	5976	4192
miR399i	LOC_Os04g10850.1	153	-20.56	153	-20.56	354339	21	5592	3853
miR399i	LOC_Os04g11090.1	155	-23.01	155	-23.01	354360	21	3876	645
miR399i	LOC_Os04g11330.1	146	-24.06	146	-24.06	354385	21	924	229
miR399i	LOC_Os04g11620.1	311	-40.89	157	-20.46	354413	21	5544	5421 5376
miR399i	LOC_Os04g12130.1	327	-49.04	164	-27.61	354466	21	3024	2468 1101
miR399i	LOC_Os04g12720.1	163	-23.46	163	-23.46	354519	21	1732	973

miR399i	LOC_Os04g12950.1	163	-23.46	163	-23.46	354535	21	1266	661
miR399i	LOC_Os04g12970.1	163	-23.46	163	-23.46	354537	21	1841	1092
miR399i	LOC_Os04g13010.1	150	-21.77	150	-21.77	354541	21	846	129
miR399i	LOC_Os04g13030.1	153	-20.56	153	-20.56	354543	21	6027	4243
miR399i	LOC_Os04g13080.1	166	-27.18	166	-27.18	354548	21	1815	1422
miR399i	LOC_Os04g13510.1	162	-22.42	162	-22.42	354587	21	4560	4490
miR399i	LOC_Os04g13560.1	156	-21.19	156	-21.19	354592	21	1548	796
miR399i	LOC_Os04g13750.1	157	-27.94	157	-27.94	354611	21	2719	2349
miR399i	LOC_Os04g14000.1	153	-23.45	153	-23.45	354636	21	1881	118
miR399i	LOC_Os04g14510.1	168	-25.09	168	-25.09	354689	21	3582	1485
miR399i	LOC_Os04g14510.2	168	-25.09	168	-25.09	354690	21	3625	1485
miR399i	LOC_Os04g14654.1	159	-20.71	159	-20.71	354704	21	2328	839
miR399i	LOC_Os04g14654.2	159	-20.71	159	-20.71	354705	21	2338	839
miR399i	LOC_Os04g14654.3	159	-20.71	159	-20.71	354706	21	2240	751
miR399i	LOC_Os04g17130.1	151	-20.78	151	-20.78	354929	21	4533	4029
miR399i	LOC_Os04g17200.1	156	-20.44	156	-20.44	354936	21	534	185
miR399i	LOC_Os04g17320.1	140	-23.66	140	-23.66	354948	21	4863	972
miR399i	LOC_Os04g17410.1	156	-24.57	156	-24.57	354957	21	4368	2788
miR399i	LOC_Os04g17650.1	151	-20.89	151	-20.89	354979	21	2633	1577
miR399i	LOC_Os04g18570.1	163	-21.83	163	-21.83	355070	21	3039	425
miR399i	LOC_Os04g18770.1	140	-23.66	140	-23.66	355090	21	4650	2475
miR399i	LOC_Os04g18884.1	140	-20.45	140	-20.45	355101	21	1788	1083
miR399i	LOC_Os04g19170.1	160	-22.64	160	-22.64	355128	21	1083	970
miR399i	LOC_Os04g20810.1	157	-20.43	157	-20.43	355290	21	1359	1267
miR399i	LOC_Os04g20810.2	157	-20.43	157	-20.43	355291	21	1418	1326
miR399i	LOC_Os04g20810.3	157	-20.43	157	-20.43	355292	21	1240	1148
miR399i	LOC_Os04g21100.1	154	-22.84	154	-22.84	355316	21	2229	1874
miR399i	LOC_Os04g22500.1	157	-22.55	157	-22.55	355455	21	1809	1545
miR399i	LOC_Os04g22580.1	149	-21.16	149	-21.16	355461	21	2973	1479
miR399i	LOC_Os04g22960.1	160	-21.87	160	-21.87	355497	21	1833	78
miR399i	LOC_Os04g23310.1	153	-20.56	153	-20.56	355529	21	5211	3712
miR399i	LOC_Os04g23799.1	157	-21.58	157	-21.58	355569	21	3915	2395

miR399i	LOC_Os04g24290.1	157	-24.79	157	-24.79	355618	21	1611	371
miR399i	LOC_Os04g24590.1	153	-20.56	153	-20.56	355653	21	5979	4198
miR399i	LOC_Os04g24700.1	173	-25.97	173	-25.97	355664	21	2280	1255
miR399i	LOC_Os04g24980.1	147	-22.07	147	-22.07	355690	21	966	547
miR399i	LOC_Os04g25020.1	147	-22.07	147	-22.07	355696	21	5466	5047
miR399i	LOC_Os04g25030.1	147	-22.07	147	-22.07	355697	21	5196	4945
miR399i	LOC_Os04g26460.1	155	-20.54	155	-20.54	355843	21	321	77
miR399i	LOC_Os04g28090.1	151	-20.24	151	-20.24	355996	21	3576	1567
miR399i	LOC_Os04g28450.1	167	-28.53	167	-28.53	356030	21	450	242
miR399i	LOC_Os04g28560.1	142	-22.13	142	-22.13	356042	21	4824	2260
miR399i	LOC_Os04g28880.1	165	-24.86	165	-24.86	356074	21	4584	1019
miR399i	LOC_Os04g28900.1	153	-23.38	153	-23.38	356076	21	2442	843
miR399i	LOC_Os04g29500.1	155	-24.66	155	-24.66	356126	21	1165	848
miR399i	LOC_Os04g30010.1	160	-22.38	160	-22.38	356173	21	2522	2372
miR399i	LOC_Os04g30210.1	157	-20.21	157	-20.21	356192	21	2140	1203
miR399i	LOC_Os04g30230.1	157	-20.21	157	-20.21	356194	21	909	817
miR399i	LOC_Os04g30320.1	157	-20.21	157	-20.21	356205	21	1849	1102
miR399i	LOC_Os04g30530.1	159	-22.11	159	-22.11	356224	21	1704	1585
miR399i	LOC_Os04g30610.1	159	-21.61	159	-21.61	356232	21	1518	1021
miR399i	LOC_Os04g30660.1	159	-22.11	159	-22.11	356237	21	3231	1276
miR399i	LOC_Os04g30690.1	159	-22.11	159	-22.11	356240	21	2811	1276
miR399i	LOC_Os04g30860.1	163	-23.26	163	-23.26	356260	21	2928	826
miR399i	LOC_Os04g30930.1	147	-20.35	147	-20.35	356269	21	3240	1663
miR399i	LOC_Os04g31030.1	154	-22.01	154	-22.01	356280	21	1858	138
miR399i	LOC_Os04g32270.1	150	-22.6	150	-22.6	356407	21	978	355
miR399i	LOC_Os04g32440.1	154	-20.65	154	-20.65	356425	21	1122	283
miR399i	LOC_Os04g32650.1	159	-22.41	159	-22.41	356449	21	2496	1549
miR399i	LOC_Os04g32920.1	161	-20.14	161	-20.14	356482	21	3094	99
miR399i	LOC_Os04g32920.2	161	-20.14	161	-20.14	356483	21	3174	99
miR399i	LOC_Os04g32920.3	161	-20.14	161	-20.14	356484	21	3069	101
miR399i	LOC_Os04g32920.5	161	-20.14	161	-20.14	356486	21	3082	99
miR399i	LOC_Os04g33120.1	150	-27.61	150	-27.61	356512	21	2805	269

miR399i	LOC_Os04g33160.1	168	-27.15	168	-27.15	356516	21	399	174
miR399i	LOC_Os04g33360.1	157	-20.39	157	-20.39	356540	21	1814	133
miR399i	LOC_Os04g33690.1	147	-20.68	147	-20.68	356579	21	2798	2748
miR399i	LOC_Os04g34080.1	162	-21.91	162	-21.91	356619	21	2774	1430
miR399i	LOC_Os04g34260.1	153	-20.44	153	-20.44	356637	21	4551	3810
miR399i	LOC_Os04g34290.1	154	-22.31	154	-22.31	356640	21	2451	806
miR399i	LOC_Os04g35090.1	298	-41.85	157	-21.18	356721	21	552	478 502
miR399i	LOC_Os04g35260.1	152	-22.43	152	-22.43	356739	21	4533	3377
miR399i	LOC_Os04g35800.1	159	-25.77	159	-25.77	356807	21	2169	987
miR399i	LOC_Os04g35900.1	161	-24.11	161	-24.11	356818	21	1988	1170
miR399i	LOC_Os04g35920.2	154	-20.05	154	-20.05	356820	21	2643	1615
miR399i	LOC_Os04g35940.1	150	-20.26	150	-20.26	356822	21	1638	437
miR399i	LOC_Os04g36720.1	161	-22.11	161	-22.11	356856	21	2761	394
miR399i	LOC_Os04g36740.1	150	-22.9	150	-22.9	356858	21	2303	2103
miR399i	LOC_Os04g37970.1	165	-24.67	165	-24.67	356943	21	1563	100
miR399i	LOC_Os04g38420.1	154	-23.35	154	-23.35	356987	21	1289	33
miR399i	LOC_Os04g38630.1	313	-44.15	158	-22.65	357015	21	2789	748 920
miR399i	LOC_Os04g38940.1	165	-23.5	165	-23.5	357057	21	1212	1081
miR399i	LOC_Os04g38940.2	165	-23.5	165	-23.5	357058	21	1344	1213
miR399i	LOC_Os04g39190.1	168	-28.42	168	-28.42	357090	21	3530	2171
miR399i	LOC_Os04g40020.1	154	-25.8	154	-25.8	357186	21	858	750
miR399i	LOC_Os04g41220.1	153	-23.46	153	-23.46	357344	21	3158	2703
miR399i	LOC_Os04g41560.2	155	-22.17	155	-22.17	357380	21	1272	366
miR399i	LOC_Os04g41560.4	155	-22.17	155	-22.17	357381	21	1367	366
miR399i	LOC_Os04g41690.1	167	-28.81	167	-28.81	357394	21	3324	3130
miR399i	LOC_Os04g42100.1	150	-27.64	150	-27.64	357443	21	2247	811
miR399i	LOC_Os04g42610.1	147	-20.7	147	-20.7	357509	21	2784	759
miR399i	LOC_Os04g43150.1	154	-20.1	154	-20.1	357574	21	2004	1330
miR399i	LOC_Os04g43150.2	154	-20.1	154	-20.1	357575	21	2787	1434
miR399i	LOC_Os04g43164.1	152	-21.21	152	-21.21	357577	21	1378	888
miR399i	LOC_Os04g43164.2	152	-21.21	152	-21.21	357578	21	1536	1051
miR399i	LOC_Os04g43220.1	162	-20.18	162	-20.18	357587	21	2911	1887

miR399i	LOC_Os04g43220.2	162	-20.18	162	-20.18	357588	21	2950	1812
miR399i	LOC_Os04g43220.3	162	-20.18	162	-20.18	357589	21	2759	1812
miR399i	LOC_Os04g43220.4	162	-20.18	162	-20.18	357590	21	4011	1873
miR399i	LOC_Os04g43230.1	157	-25.87	157	-25.87	357591	21	1703	628
miR399i	LOC_Os04g43230.2	157	-25.87	157	-25.87	357592	21	1778	703
miR399i	LOC_Os04g44900.1	156	-25.14	156	-25.14	357787	21	2558	1559
miR399i	LOC_Os04g45130.1	161	-22.74	161	-22.74	357821	21	752	212
miR399i	LOC_Os04g45970.1	153	-25.25	153	-25.25	357913	21	2102	524
miR399i	LOC_Os04g45970.2	153	-25.25	153	-25.25	357914	21	1508	525
miR399i	LOC_Os04g45990.1	162	-23.02	162	-23.02	357916	21	2714	1573
miR399i	LOC_Os04g46710.1	151	-21.94	151	-21.94	358003	21	696	79
miR399i	LOC_Os04g46860.1	165	-22.68	165	-22.68	358026	21	2663	264
miR399i	LOC_Os04g49240.1	161	-20.12	161	-20.12	358310	21	450	52
miR399i	LOC_Os04g49290.1	150	-21.21	150	-21.21	358315	21	4335	3191
miR399i	LOC_Os04g49530.1	155	-20.13	155	-20.13	358342	21	2175	2136
miR399i	LOC_Os04g49540.1	155	-20.13	155	-20.13	358343	21	2011	1101
miR399i	LOC_Os04g50830.1	158	-20.67	158	-20.67	358447	21	3570	1831
miR399i	LOC_Os04g51120.1	150	-20.36	150	-20.36	358481	21	2391	1674
miR399i	LOC_Os04g51120.3	150	-20.36	150	-20.36	358482	21	2623	1906
miR399i	LOC_Os04g51454.1	154	-20.48	154	-20.48	358530	21	516	151
miR399i	LOC_Os04g51650.1	166	-28.78	166	-28.78	358554	21	309	225
miR399i	LOC_Os04g51809.1	158	-22.69	158	-22.69	358576	21	2132	1662
miR399i	LOC_Os04g51809.3	158	-22.69	158	-22.69	358578	21	1658	1188
miR399i	LOC_Os04g52380.1	155	-21.83	155	-21.83	358649	21	2155	402
miR399i	LOC_Os04g52380.2	155	-21.83	155	-21.83	358650	21	3260	402
miR399i	LOC_Os04g52380.3	155	-21.83	155	-21.83	358651	21	2083	402
miR399i	LOC_Os04g52380.4	155	-21.83	155	-21.83	358652	21	2048	402
miR399i	LOC_Os04g52390.1	155	-20.48	155	-20.48	358653	21	3493	3430
miR399i	LOC_Os04g53660.1	150	-20.51	150	-20.51	358817	21	3132	125
miR399i	LOC_Os04g53660.3	150	-20.51	150	-20.51	358819	21	3047	107
miR399i	LOC_Os04g53890.1	153	-20.44	153	-20.44	358847	21	4551	3810
miR399i	LOC_Os04g53900.1	155	-24.66	155	-24.66	358848	21	1284	812

miR399i	LOC_Os04g53990.1	155	-20.6	155	-20.6	358860	21	1976	778
miR399i	LOC_Os04g54710.1	152	-20.95	152	-20.95	358945	21	2139	67
miR399i	LOC_Os04g55140.1	325	-44.27	164	-22.84	358993	21	3546	2990 1131
miR399i	LOC_Os04g55190.1	150	-21.52	150	-21.52	358999	21	3245	2953
miR399i	LOC_Os04g55230.1	182	-33.21	182	-33.21	359003	21	5705	4032
miR399i	LOC_Os04g55330.1	147	-25.24	147	-25.24	359017	21	588	25
miR399i	LOC_Os04g55960.1	154	-22.45	154	-22.45	359089	21	2578	1207
miR399i	LOC_Os04g56160.1	157	-20.7	157	-20.7	359114	21	3639	3501
miR399i	LOC_Os04g56160.2	157	-20.7	157	-20.7	359115	21	2393	2257
miR399i	LOC_Os04g56160.3	157	-20.7	157	-20.7	359116	21	3417	3279
miR399i	LOC_Os04g56510.1	154	-20.06	154	-20.06	359159	21	2865	336
miR399i	LOC_Os04g58250.1	152	-25.82	152	-25.82	359392	21	2608	1667
miR399i	LOC_Os04g58410.1	169	-24.8	169	-24.8	359410	21	3345	2619
miR399i	LOC_Os04g58410.2	169	-24.8	169	-24.8	359411	21	3440	2697
miR399i	LOC_Os04g58470.1	152	-23.14	152	-23.14	359418	21	2522	2004
miR399i	LOC_Os04g58504.1	152	-23.14	152	-23.14	359421	21	2463	1973
miR399i	LOC_Os04g58600.2	145	-22.75	145	-22.75	359436	21	1643	969
miR399i	LOC_Os04g58840.1	163	-26.75	163	-26.75	359471	21	2098	1957
miR399i	LOC_Os04g58970.1	158	-23.55	158	-23.55	359484	21	608	519
miR399i	LOC_Os04g58980.1	158	-23.55	158	-23.55	359485	21	2649	344
miR399i	LOC_Os04g59090.1	162	-24.61	162	-24.61	359498	21	1947	1592
miR399i	LOC_Os04g59180.1	153	-20.56	153	-20.56	359508	21	5457	3661
miR399i	LOC_Os04g59320.1	147	-22.4	147	-22.4	359522	21	2756	1967
miR399i	LOC_Os04g59540.1	167	-26.38	167	-26.38	359548	21	4528	2231
miR399i	LOC_Os04g59624.2	157	-24.9	157	-24.9	359568	21	3412	2627
miR399i	LOC_Os04g59624.3	157	-24.9	157	-24.9	359569	21	3481	2630
miR399i	LOC_Os05g01030.1	159	-23.71	159	-23.71	359584	21	3570	920
miR399i	LOC_Os05g01350.1	153	-22.33	153	-22.33	359634	21	3053	2085
miR399i	LOC_Os05g01520.1	156	-20.28	156	-20.28	359660	21	2496	1622
miR399i	LOC_Os05g02040.1	144	-20.6	144	-20.6	359729	21	3409	904
miR399i	LOC_Os05g02340.1	157	-21.13	157	-21.13	359767	21	4074	2678
miR399i	LOC_Os05g02790.1	165	-25.25	165	-25.25	359824	21	3105	141

miR399i	LOC_Os05g03000.1	141	-20.24	141	-20.24	359844	21	1818	1008
miR399i	LOC_Os05g03000.2	141	-20.24	141	-20.24	359845	21	1835	1045
miR399i	LOC_Os05g03310.1	157	-20.65	157	-20.65	359882	21	1989	385
miR399i	LOC_Os05g03400.1	164	-21.09	164	-21.09	359894	21	3112	1577
miR399i	LOC_Os05g03420.1	298	-41.66	150	-21.49	359896	21	912	773 696
miR399i	LOC_Os05g03480.2	156	-20.09	156	-20.09	359903	21	1654	517
miR399i	LOC_Os05g03480.4	156	-20.09	156	-20.09	359904	21	1644	517
miR399i	LOC_Os05g03480.5	156	-20.09	156	-20.09	359905	21	1185	517
miR399i	LOC_Os05g03650.1	153	-24.37	153	-24.37	359925	21	3084	516
miR399i	LOC_Os05g03810.1	169	-26.08	169	-26.08	359940	21	819	472
miR399i	LOC_Os05g04420.1	156	-20.19	156	-20.19	360010	21	5379	2522
miR399i	LOC_Os05g04520.1	157	-22.51	157	-22.51	360021	21	1632	501
miR399i	LOC_Os05g04980.1	153	-21.08	153	-21.08	360070	21	5910	4111
miR399i	LOC_Os05g05130.1	163	-29.25	163	-29.25	360085	21	1350	674
miR399i	LOC_Os05g05310.1	152	-20.37	152	-20.37	360107	21	3863	2869
miR399i	LOC_Os05g05310.2	152	-20.37	152	-20.37	360108	21	3859	2869
miR399i	LOC_Os05g05490.1	170	-28.1	170	-28.1	360130	21	2268	1468
miR399i	LOC_Os05g05620.1	152	-23.16	152	-23.16	360149	21	700	195
miR399i	LOC_Os05g06280.1	147	-22.03	147	-22.03	360221	21	3584	285
miR399i	LOC_Os05g06280.2	147	-22.03	147	-22.03	360222	21	3599	573
miR399i	LOC_Os05g06330.1	160	-22.36	160	-22.36	360229	21	1404	428
miR399i	LOC_Os05g06910.1	168	-23.65	168	-23.65	360300	21	1787	1607
miR399i	LOC_Os05g07020.1	143	-20.74	143	-20.74	360311	21	585	532
miR399i	LOC_Os05g07560.1	157	-21.98	157	-21.98	360373	21	4291	217
miR399i	LOC_Os05g08310.1	158	-20.06	158	-20.06	360462	21	4404	3775
miR399i	LOC_Os05g09420.1	157	-23.48	157	-23.48	360599	21	516	486
miR399i	LOC_Os05g10440.1	164	-27.68	164	-27.68	360665	21	2346	1204
miR399i	LOC_Os05g10590.1	149	-24.34	149	-24.34	360681	21	2052	1851
miR399i	LOC_Os05g10590.2	149	-24.34	149	-24.34	360682	21	1126	843
miR399i	LOC_Os05g10800.1	156	-20.72	156	-20.72	360711	21	1417	1
miR399i	LOC_Os05g11400.1	167	-28.59	167	-28.59	360778	21	1329	514
miR399i	LOC_Os05g12570.1	333	-51.88	173	-28.01	360901	21	3315	1117 669

miR399i	LOC_Os05g12804.1	173	-27.8	173	-27.8	360928	21	1659	1344
miR399i	LOC_Os05g13830.1	312	-46.17	161	-24.45	360984	21	762	388 329
miR399i	LOC_Os05g13850.1	161	-24.45	161	-24.45	360986	21	762	388
miR399i	LOC_Os05g13940.1	616	-84.38	155	-21.79	360996	21	1900	912 1050 897 1035
miR399i	LOC_Os05g15530.1	155	-25.76	155	-25.76	361165	21	1328	773
miR399i	LOC_Os05g15920.1	149	-21.11	149	-21.11	361204	21	1169	248
miR399i	LOC_Os05g15990.1	153	-21.08	153	-21.08	361211	21	5940	4144
miR399i	LOC_Os05g16410.1	159	-23.38	159	-23.38	361252	21	1377	970
miR399i	LOC_Os05g16700.1	153	-20.56	153	-20.56	361284	21	1785	10
miR399i	LOC_Os05g17400.1	161	-25.05	161	-25.05	361354	21	921	716
miR399i	LOC_Os05g17650.1	155	-22.21	155	-22.21	361381	21	1902	704
miR399i	LOC_Os05g18294.2	159	-22.76	159	-22.76	361445	21	1660	870
miR399i	LOC_Os05g18294.3	159	-22.76	159	-22.76	361446	21	1380	870
miR399i	LOC_Os05g18930.1	161	-21.95	161	-21.95	361513	21	2118	404
miR399i	LOC_Os05g19010.1	150	-20.1	150	-20.1	361521	21	937	191
miR399i	LOC_Os05g19050.1	157	-20.82	157	-20.82	361526	21	4152	3294
miR399i	LOC_Os05g19330.1	153	-20.44	153	-20.44	361554	21	4488	3747
miR399i	LOC_Os05g19400.1	165	-21.22	165	-21.22	361560	21	1152	582
miR399i	LOC_Os05g19910.1	157	-21.78	157	-21.78	361616	21	1602	271
miR399i	LOC_Os05g20000.1	157	-23.7	157	-23.7	361629	21	3147	481
miR399i	LOC_Os05g20050.1	172	-29.16	172	-29.16	361634	21	1209	542
miR399i	LOC_Os05g20050.2	172	-29.16	172	-29.16	361635	21	1119	542
miR399i	LOC_Os05g20210.1	158	-27.35	158	-27.35	361650	21	759	286
miR399i	LOC_Os05g20270.1	155	-20.23	155	-20.23	361656	21	2604	2366
miR399i	LOC_Os05g20360.1	161	-23.19	161	-23.19	361665	21	4542	137
miR399i	LOC_Os05g20640.1	149	-20.48	149	-20.48	361693	21	447	37
miR399i	LOC_Os05g20730.1	149	-20.48	149	-20.48	361702	21	1746	37
miR399i	LOC_Os05g21090.1	149	-20.48	149	-20.48	361744	21	1419	771
miR399i	LOC_Os05g22300.1	146	-24.14	146	-24.14	361767	21	1395	969
miR399i	LOC_Os05g22500.1	153	-21.13	153	-21.13	361788	21	1845	37
miR399i	LOC_Os05g22660.1	171	-25.65	171	-25.65	361804	21	2568	90
miR399i	LOC_Os05g22920.1	164	-25.28	164	-25.28	361833	21	2405	1171

miR399i	LOC_Os05g22920.2	164	-25.28	164	-25.28	361834	21	2272	1080
miR399i	LOC_Os05g22920.4	164	-25.28	164	-25.28	361835	21	2031	1171
miR399i	LOC_Os05g23240.1	168	-26.96	168	-26.96	361868	21	894	273
miR399i	LOC_Os05g23260.1	145	-20.59	145	-20.59	361871	21	1181	487
miR399i	LOC_Os05g23290.1	161	-20.87	161	-20.87	361874	21	2946	2626
miR399i	LOC_Os05g23700.1	154	-23.42	154	-23.42	361926	21	1393	226
miR399i	LOC_Os05g23740.1	150	-21.04	150	-21.04	361928	21	2505	782
miR399i	LOC_Os05g23880.1	154	-20.46	154	-20.46	361942	21	3815	2389
miR399i	LOC_Os05g23970.1	151	-23.65	151	-23.65	361951	21	2106	455
miR399i	LOC_Os05g23980.1	153	-24.79	153	-24.79	361952	21	2187	283
miR399i	LOC_Os05g24610.1	153	-24.36	153	-24.36	362023	21	5970	518
miR399i	LOC_Os05g24790.1	145	-23.03	145	-23.03	362041	21	1434	1044
miR399i	LOC_Os05g24860.1	310	-42.72	157	-21.37	362047	21	2574	926 979
miR399i	LOC_Os05g25470.1	149	-20.5	149	-20.5	362116	21	4704	2180
miR399i	LOC_Os05g25974.1	169	-28.66	169	-28.66	362169	21	2654	552
miR399i	LOC_Os05g25974.2	169	-28.66	169	-28.66	362170	21	2590	476
miR399i	LOC_Os05g27160.1	173	-27.57	173	-27.57	362287	21	291	102
miR399i	LOC_Os05g27400.1	155	-23.01	155	-23.01	362310	21	3729	642
miR399i	LOC_Os05g27860.1	147	-20.56	147	-20.56	362358	21	2418	338
miR399i	LOC_Os05g28090.1	161	-24.38	161	-24.38	362386	21	923	324
miR399i	LOC_Os05g28090.2	161	-24.38	161	-24.38	362387	21	1479	362
miR399i	LOC_Os05g28090.3	161	-24.38	161	-24.38	362388	21	1018	324
miR399i	LOC_Os05g28170.1	154	-20.27	154	-20.27	362394	21	3423	993
miR399i	LOC_Os05g28170.2	154	-20.27	154	-20.27	362395	21	4616	1990
miR399i	LOC_Os05g28250.1	149	-20.48	149	-20.48	362405	21	2049	37
miR399i	LOC_Os05g29110.1	166	-26.64	166	-26.64	362501	21	4722	464
miR399i	LOC_Os05g30070.1	142	-23.41	142	-23.41	362557	21	4530	2620
miR399i	LOC_Os05g30270.1	153	-20.56	153	-20.56	362579	21	5205	4192
miR399i	LOC_Os05g30660.1	140	-20.25	140	-20.25	362624	21	2133	595
miR399i	LOC_Os05g31120.1	153	-24.89	153	-24.89	362684	21	1158	381
miR399i	LOC_Os05g31440.1	153	-20.56	153	-20.56	362716	21	5961	4171
miR399i	LOC_Os05g31860.1	151	-20.93	151	-20.93	362761	21	3171	2530

miR399i	LOC_Os05g32030.1	153	-20.65	153	-20.65	362776	21	2013	66
miR399i	LOC_Os05g32050.1	157	-20.91	157	-20.91	362778	21	4278	2530
miR399i	LOC_Os05g33260.2	150	-20.43	150	-20.43	362916	21	1618	916
miR399i	LOC_Os05g33260.3	150	-20.43	150	-20.43	362917	21	1555	916
miR399i	LOC_Os05g34650.1	161	-24.38	161	-24.38	363077	21	2156	1190
miR399i	LOC_Os05g34650.2	161	-24.38	161	-24.38	363078	21	1658	692
miR399i	LOC_Os05g34720.1	150	-20.01	150	-20.01	363088	21	1358	839
miR399i	LOC_Os05g34760.2	152	-23.61	152	-23.61	363093	21	3677	2618
miR399i	LOC_Os05g34870.1	159	-22.72	159	-22.72	363106	21	1008	50
miR399i	LOC_Os05g35180.1	168	-26.16	168	-26.16	363143	21	3075	292
miR399i	LOC_Os05g35380.1	171	-26.31	171	-26.31	363168	21	1145	320
miR399i	LOC_Os05g36090.1	147	-20.39	147	-20.39	363245	21	1967	1199
miR399i	LOC_Os05g36360.1	153	-21.62	153	-21.62	363283	21	1623	1165
miR399i	LOC_Os05g36940.1	153	-20.16	153	-20.16	363294	21	4308	3053
miR399i	LOC_Os05g37500.1	148	-20.68	148	-20.68	363366	21	6524	2255
miR399i	LOC_Os05g37740.1	153	-24.9	153	-24.9	363392	21	672	615
miR399i	LOC_Os05g37770.1	158	-22.51	158	-22.51	363395	21	414	99
miR399i	LOC_Os05g37940.1	160	-22.27	160	-22.27	363416	21	297	249
miR399i	LOC_Os05g38190.2	163	-23.79	163	-23.79	363450	21	4395	2674
miR399i	LOC_Os05g38640.1	159	-22.41	159	-22.41	363516	21	1134	555
miR399i	LOC_Os05g38950.1	161	-22.3	161	-22.3	363552	21	3127	486
miR399i	LOC_Os05g38950.2	161	-22.3	161	-22.3	363553	21	3034	393
miR399i	LOC_Os05g39390.1	149	-22.21	149	-22.21	363607	21	3323	1644
miR399i	LOC_Os05g39410.1	140	-24.77	140	-24.77	363609	21	2660	1524
miR399i	LOC_Os05g39410.2	140	-24.77	140	-24.77	363610	21	2908	1775
miR399i	LOC_Os05g39410.3	140	-24.77	140	-24.77	363611	21	2739	1524
miR399i	LOC_Os05g39670.1	160	-23.82	160	-23.82	363641	21	1125	784
miR399i	LOC_Os05g40050.1	143	-20.83	143	-20.83	363679	21	3044	1916
miR399i	LOC_Os05g40050.2	143	-20.83	143	-20.83	363680	21	3094	1916
miR399i	LOC_Os05g40070.1	156	-24.14	156	-24.14	363682	21	1174	1067
miR399i	LOC_Os05g40810.1	152	-20.39	152	-20.39	363776	21	2326	1157
miR399i	LOC_Os05g41510.1	145	-21.53	145	-21.53	363858	21	6761	4916

miR399i	LOC_Os05g41520.1	145	-21.53	145	-21.53	363859	21	1891	46
miR399i	LOC_Os05g41830.1	327	-49.04	164	-27.61	363895	21	3906	2780 1134
miR399i	LOC_Os05g42400.1	157	-20.23	157	-20.23	363959	21	156	132
miR399i	LOC_Os05g42448.1	166	-29.57	166	-29.57	363966	21	3933	890
miR399i	LOC_Os05g43220.1	153	-20.95	153	-20.95	363994	21	3636	40
miR399i	LOC_Os05g43350.1	165	-24.73	165	-24.73	364009	21	3924	2186
miR399i	LOC_Os05g43390.1	162	-25.99	162	-25.99	364015	21	2068	1035
miR399i	LOC_Os05g43550.1	155	-20.01	155	-20.01	364032	21	1299	493
miR399i	LOC_Os05g43700.1	148	-20.32	148	-20.32	364050	21	435	352
miR399i	LOC_Os05g43800.1	145	-20.03	145	-20.03	364062	21	2096	1519
miR399i	LOC_Os05g43910.1	153	-24.52	153	-24.52	364077	21	2396	1977
miR399i	LOC_Os05g44360.1	146	-21.59	146	-21.59	364137	21	2831	2265
miR399i	LOC_Os05g44360.2	146	-21.59	146	-21.59	364138	21	2410	2352
miR399i	LOC_Os05g45340.1	146	-21.85	146	-21.85	364254	21	3239	1864
miR399i	LOC_Os05g45350.2	174	-31.64	174	-31.64	364256	21	1602	404
miR399i	LOC_Os05g45360.1	165	-24.68	165	-24.68	364258	21	957	372
miR399i	LOC_Os05g45830.1	162	-24.44	162	-24.44	364313	21	3058	2339
miR399i	LOC_Os05g45950.1	141	-21.64	141	-21.64	364330	21	1424	782
miR399i	LOC_Os05g46040.2	162	-22.21	162	-22.21	364340	21	2459	926
miR399i	LOC_Os05g47520.1	157	-20.78	157	-20.78	364467	21	831	229
miR399i	LOC_Os05g47560.1	151	-20.55	151	-20.55	364476	21	2160	1364
miR399i	LOC_Os05g47920.1	327	-49.04	164	-27.61	364511	21	4734	3443 1422
miR399i	LOC_Os05g48570.1	152	-21.15	152	-21.15	364598	21	579	479
miR399i	LOC_Os05g49130.1	162	-30.94	162	-30.94	364676	21	3232	2666
miR399i	LOC_Os05g49130.2	162	-30.94	162	-30.94	364677	21	2752	2186
miR399i	LOC_Os05g49250.1	157	-21.61	157	-21.61	364694	21	1360	1176
miR399i	LOC_Os05g49250.2	157	-21.61	157	-21.61	364695	21	3128	2943
miR399i	LOC_Os05g49470.1	152	-21.14	152	-21.14	364724	21	1192	1000
miR399i	LOC_Os05g49720.1	171	-25.65	171	-25.65	364753	21	1299	20
miR399i	LOC_Os05g50080.1	144	-21.19	144	-21.19	364801	21	1620	190
miR399i	LOC_Os05g50560.1	154	-24.23	154	-24.23	364856	21	2630	1316
miR399i	LOC_Os05g51220.1	148	-20.18	148	-20.18	364946	21	1721	184

miR399i	LOC_Os05g51480.1	152	-20.36	152	-20.36	364978	21	3831	3107
miR399i	LOC_Os05g51480.2	152	-20.36	152	-20.36	364979	21	3825	3103
miR399i	LOC_Os05g51550.1	162	-21.14	162	-21.14	364989	21	2700	1259
miR399i	LOC_Os05g51570.1	157	-26.86	157	-26.86	364993	21	1655	512
miR399i	LOC_Os05g51590.1	159	-23.57	159	-23.57	364995	21	1439	753
miR399i	LOC_Os06g01320.1	156	-20.39	156	-20.39	365090	21	7009	4750
miR399i	LOC_Os06g01500.1	152	-21.08	152	-21.08	365107	21	1877	707
miR399i	LOC_Os06g01500.3	152	-21.08	152	-21.08	365109	21	1787	707
miR399i	LOC_Os06g01650.1	161	-22.93	161	-22.93	365127	21	3936	140
miR399i	LOC_Os06g01650.2	161	-22.93	161	-22.93	365128	21	4037	140
miR399i	LOC_Os06g01750.1	149	-22.28	149	-22.28	365141	21	840	699
miR399i	LOC_Os06g02180.1	147	-26.67	147	-26.67	365191	21	4214	2764
miR399i	LOC_Os06g02820.1	161	-24.38	161	-24.38	365266	21	990	858
miR399i	LOC_Os06g03380.1	146	-22.76	146	-22.76	365319	21	2575	560
miR399i	LOC_Os06g04010.1	158	-22.84	158	-22.84	365397	21	1620	801
miR399i	LOC_Os06g04010.2	158	-22.84	158	-22.84	365398	21	1644	797
miR399i	LOC_Os06g04280.1	165	-23.41	165	-23.41	365431	21	1911	859
miR399i	LOC_Os06g05240.1	158	-22.59	158	-22.59	365550	21	2516	1527
miR399i	LOC_Os06g05980.1	146	-21.11	146	-21.11	365647	21	2211	197
miR399i	LOC_Os06g05980.2	146	-21.11	146	-21.11	365648	21	2277	255
miR399i	LOC_Os06g05990.1	174	-26.91	174	-26.91	365649	21	1779	716
miR399i	LOC_Os06g06690.1	164	-27.76	164	-27.76	365742	21	360	274
miR399i	LOC_Os06g06920.1	146	-20.52	146	-20.52	365768	21	2433	23
miR399i	LOC_Os06g07070.1	163	-29.83	163	-29.83	365786	21	1651	971
miR399i	LOC_Os06g08070.1	149	-20.5	149	-20.5	365893	21	4011	2093
miR399i	LOC_Os06g08380.1	317	-43.44	160	-22.89	365922	21	6114	5171 2728
miR399i	LOC_Os06g08480.1	163	-23.58	163	-23.58	365932	21	4808	1320
miR399i	LOC_Os06g08860.1	153	-22.82	153	-22.82	365987	21	1343	289
miR399i	LOC_Os06g10480.1	148	-20.81	148	-20.81	366179	21	3205	654
miR399i	LOC_Os06g10620.1	321	-43.28	169	-22.9	366192	21	2477	287 2349
miR399i	LOC_Os06g10900.1	151	-23.72	151	-23.72	366223	21	672	337
miR399i	LOC_Os06g11170.1	161	-22.26	161	-22.26	366251	21	1350	1050

miR399i	LOC_Os06g11170.2	161	-22.26	161	-22.26	366252	21	1594	1294
miR399i	LOC_Os06g11410.1	151	-20.63	151	-20.63	366278	21	1580	742
miR399i	LOC_Os06g11410.2	151	-20.63	151	-20.63	366279	21	1698	852
miR399i	LOC_Os06g11560.1	165	-22.6	165	-22.6	366296	21	2118	977
miR399i	LOC_Os06g11990.1	163	-22.07	163	-22.07	366343	21	3815	2163
miR399i	LOC_Os06g12260.1	155	-20.66	155	-20.66	366381	21	3048	2248
miR399i	LOC_Os06g12260.2	155	-20.66	155	-20.66	366382	21	3089	2248
miR399i	LOC_Os06g12260.3	155	-20.66	155	-20.66	366383	21	2094	1294
miR399i	LOC_Os06g12610.1	160	-24.6	160	-24.6	366421	21	2469	1844
miR399i	LOC_Os06g13860.1	151	-20.07	151	-20.07	366563	21	2877	2518
miR399i	LOC_Os06g13860.2	151	-20.07	151	-20.07	366564	21	2768	2409
miR399i	LOC_Os06g13970.1	165	-23.31	165	-23.31	366574	21	690	549
miR399i	LOC_Os06g14140.1	153	-21.08	153	-21.08	366592	21	5961	4177
miR399i	LOC_Os06g16980.1	165	-25.79	165	-25.79	366890	21	4102	725
miR399i	LOC_Os06g16980.2	165	-25.79	165	-25.79	366891	21	3986	609
miR399i	LOC_Os06g17800.1	157	-25.78	157	-25.78	366970	21	2514	2342
miR399i	LOC_Os06g18820.2	153	-23.07	153	-23.07	367021	21	2715	2231
miR399i	LOC_Os06g18970.1	150	-22.28	150	-22.28	367036	21	1995	357
miR399i	LOC_Os06g19900.1	159	-23.38	159	-23.38	367131	21	3870	412
miR399i	LOC_Os06g20390.1	162	-30.09	162	-30.09	367185	21	2009	722
miR399i	LOC_Os06g20420.1	148	-20.9	148	-20.9	367192	21	441	233
miR399i	LOC_Os06g22030.1	152	-26.52	152	-26.52	367362	21	2253	1348
miR399i	LOC_Os06g22870.1	163	-25.09	163	-25.09	367457	21	1414	683
miR399i	LOC_Os06g22870.2	163	-25.09	163	-25.09	367458	21	1383	683
miR399i	LOC_Os06g23020.1	153	-20.92	153	-20.92	367474	21	639	244
miR399i	LOC_Os06g23080.1	163	-25.98	163	-25.98	367480	21	1998	873
miR399i	LOC_Os06g23100.1	294	-46.27	148	-23.72	367482	21	468	414 440
miR399i	LOC_Os06g23200.1	157	-20.97	157	-20.97	367491	21	1857	934
miR399i	LOC_Os06g23504.1	157	-21.91	157	-21.91	367526	21	2023	1105
miR399i	LOC_Os06g24090.1	151	-22.42	151	-22.42	367585	21	1503	115
miR399i	LOC_Os06g24680.1	151	-20.93	151	-20.93	367644	21	1902	394
miR399i	LOC_Os06g24730.2	165	-25.96	165	-25.96	367650	21	3743	2512

miR399i	LOC_Os06g24730.3	165	-25.96	165	-25.96	367651	21	3081	1850
miR399i	LOC_Os06g25160.1	157	-22.01	157	-22.01	367691	21	738	171
miR399i	LOC_Os06g25210.1	149	-20.48	149	-20.48	367696	21	2703	1092
miR399i	LOC_Os06g25230.1	153	-21.31	153	-21.31	367698	21	1134	876
miR399i	LOC_Os06g25280.1	153	-20.93	153	-20.93	367703	21	2136	915
miR399i	LOC_Os06g25890.1	159	-20.02	159	-20.02	367761	21	4704	2015
miR399i	LOC_Os06g26070.1	140	-20.89	140	-20.89	367779	21	3171	818
miR399i	LOC_Os06g27450.1	149	-20.48	149	-20.48	367817	21	1125	445
miR399i	LOC_Os06g28080.1	149	-20.48	149	-20.48	367889	21	1728	306
miR399i	LOC_Os06g29120.1	294	-42.74	147	-21.37	367994	21	3173	2655 2782
miR399i	LOC_Os06g29120.2	147	-21.37	147	-21.37	367995	21	2949	2568
miR399i	LOC_Os06g29250.1	154	-26.13	154	-26.13	368008	21	913	548
miR399i	LOC_Os06g29760.1	153	-20.02	153	-20.02	368063	21	6015	4219
miR399i	LOC_Os06g30080.1	157	-25.26	157	-25.26	368095	21	2736	2366
miR399i	LOC_Os06g30770.1	152	-22.06	152	-22.06	368167	21	489	166
miR399i	LOC_Os06g31750.1	155	-24.66	155	-24.66	368266	21	1269	797
miR399i	LOC_Os06g32390.1	155	-24.66	155	-24.66	368327	21	990	518
miR399i	LOC_Os06g33100.1	162	-20.68	162	-20.68	368393	21	1598	711
miR399i	LOC_Os06g33280.1	153	-20.56	153	-20.56	368410	21	2868	1153
miR399i	LOC_Os06g35110.1	327	-49.04	164	-27.61	368594	21	1284	1001 841
miR399i	LOC_Os06g35270.1	157	-25.26	157	-25.26	368611	21	2232	1877
miR399i	LOC_Os06g35390.1	161	-21.95	161	-21.95	368622	21	1650	698
miR399i	LOC_Os06g35660.1	156	-20.45	156	-20.45	368650	21	1587	55
miR399i	LOC_Os06g36500.1	163	-27.61	163	-27.61	368740	21	3756	1842
miR399i	LOC_Os06g37000.1	148	-20.72	148	-20.72	368792	21	1543	462
miR399i	LOC_Os06g37000.2	148	-20.72	148	-20.72	368793	21	1535	454
miR399i	LOC_Os06g37400.1	160	-22.66	160	-22.66	368833	21	7053	6339
miR399i	LOC_Os06g37540.1	160	-21.32	160	-21.32	368849	21	237	37
miR399i	LOC_Os06g37850.1	164	-27.68	164	-27.68	368881	21	1518	601
miR399i	LOC_Os06g38420.1	153	-20.56	153	-20.56	368935	21	5988	4192
miR399i	LOC_Os06g38480.1	151	-20.86	151	-20.86	368943	21	2210	1103
miR399i	LOC_Os06g38480.2	151	-20.86	151	-20.86	368944	21	2126	1019

miR399i	LOC_Os06g38900.1	149	-20.5	149	-20.5	368985	21	2154	122
miR399i	LOC_Os06g38950.1	158	-20.25	158	-20.25	368991	21	3289	349
miR399i	LOC_Os06g39980.1	152	-23.41	152	-23.41	369114	21	1722	78
miR399i	LOC_Os06g40000.1	141	-20.7	141	-20.7	369116	21	2742	348
miR399i	LOC_Os06g40190.1	161	-24.71	161	-24.71	369137	21	3157	1580
miR399i	LOC_Os06g40560.1	147	-22.76	147	-22.76	369176	21	1731	1321
miR399i	LOC_Os06g40650.1	156	-20.07	156	-20.07	369189	21	2172	716
miR399i	LOC_Os06g40650.2	156	-20.07	156	-20.07	369190	21	2360	716
miR399i	LOC_Os06g40650.4	156	-20.07	156	-20.07	369192	21	2613	1248
miR399i	LOC_Os06g40950.1	161	-26.48	161	-26.48	369227	21	1500	1010
miR399i	LOC_Os06g41010.1	154	-22.31	154	-22.31	369233	21	1345	222
miR399i	LOC_Os06g41010.3	154	-22.31	154	-22.31	369234	21	1246	123
miR399i	LOC_Os06g41010.4	154	-22.31	154	-22.31	369235	21	1428	305
miR399i	LOC_Os06g41510.1	161	-22.7	161	-22.7	369287	21	2496	148
miR399i	LOC_Os06g41710.1	163	-25.78	163	-25.78	369308	21	3305	417
miR399i	LOC_Os06g42420.1	157	-20.13	157	-20.13	369392	21	2730	95
miR399i	LOC_Os06g42640.1	169	-25.72	169	-25.72	369420	21	2924	566
miR399i	LOC_Os06g42640.2	169	-25.72	169	-25.72	369421	21	2940	566
miR399i	LOC_Os06g42640.3	169	-25.72	169	-25.72	369422	21	2929	566
miR399i	LOC_Os06g42860.1	163	-23.17	163	-23.17	369445	21	1668	216
miR399i	LOC_Os06g43290.1	153	-20.95	153	-20.95	369489	21	3711	40
miR399i	LOC_Os06g43540.1	153	-20.16	153	-20.16	369513	21	4220	2657
miR399i	LOC_Os06g43800.1	166	-20.68	166	-20.68	369547	21	1671	350
miR399i	LOC_Os06g43800.2	166	-20.68	166	-20.68	369548	21	1836	350
miR399i	LOC_Os06g43820.1	140	-22.64	140	-22.64	369550	21	765	210
miR399i	LOC_Os06g44200.1	166	-26.45	166	-26.45	369596	21	2224	662
miR399i	LOC_Os06g44200.2	166	-26.45	166	-26.45	369597	21	2203	662
miR399i	LOC_Os06g44200.3	166	-26.45	166	-26.45	369598	21	2282	662
miR399i	LOC_Os06g44770.1	151	-20.67	151	-20.67	369658	21	516	145
miR399i	LOC_Os06g45020.1	147	-20.53	147	-20.53	369686	21	2795	190
miR399i	LOC_Os06g45030.1	149	-21.69	149	-21.69	369687	21	3229	2080
miR399i	LOC_Os06g45800.1	153	-25.14	153	-25.14	369784	21	4464	3161

miR399i	LOC_Os06g45850.1	149	-20.14	149	-20.14	369793	21	1279	466
miR399i	LOC_Os06g45850.2	149	-20.14	149	-20.14	369794	21	1292	479
miR399i	LOC_Os06g45850.3	149	-20.14	149	-20.14	369795	21	1251	433
miR399i	LOC_Os06g45850.4	149	-20.14	149	-20.14	369796	21	1250	437
miR399i	LOC_Os06g45850.5	149	-20.14	149	-20.14	369797	21	1246	433
miR399i	LOC_Os06g46380.1	154	-22.6	154	-22.6	369853	21	2503	861
miR399i	LOC_Os06g46870.1	167	-23.64	167	-23.64	369914	21	813	312
miR399i	LOC_Os06g46940.1	155	-20.71	155	-20.71	369926	21	3151	1617
miR399i	LOC_Os06g46940.2	155	-20.71	155	-20.71	369927	21	1506	76
miR399i	LOC_Os06g47380.1	153	-20.56	153	-20.56	369986	21	6033	4258
miR399i	LOC_Os06g48140.1	157	-25.78	157	-25.78	370075	21	2514	2342
miR399i	LOC_Os06g48470.1	153	-20.56	153	-20.56	370113	21	6033	4258
miR399i	LOC_Os06g48520.1	155	-21.21	155	-21.21	370119	21	2235	102
miR399i	LOC_Os06g49060.1	145	-28.94	145	-28.94	370190	21	2143	663
miR399i	LOC_Os06g49130.1	150	-22.63	150	-22.63	370201	21	5280	2166
miR399i	LOC_Os06g49130.2	150	-22.63	150	-22.63	370202	21	4413	1299
miR399i	LOC_Os06g49250.5	148	-20.12	148	-20.12	370219	21	2215	438
miR399i	LOC_Os06g49540.1	151	-21.38	151	-21.38	370261	21	4221	1122
miR399i	LOC_Os06g49670.1	142	-23.39	142	-23.39	370278	21	2060	1495
miR399i	LOC_Os06g49670.2	142	-23.39	142	-23.39	370279	21	1740	1495
miR399i	LOC_Os06g49980.1	159	-21.84	159	-21.84	370318	21	1116	1094
miR399i	LOC_Os06g50110.1	157	-22.41	157	-22.41	370334	21	1586	873
miR399i	LOC_Os06g50270.1	153	-21.08	153	-21.08	370352	21	5910	4111
miR399i	LOC_Os06g50789.1	149	-25.08	149	-25.08	370408	21	1485	272
miR399i	LOC_Os06g50930.1	149	-24.6	149	-24.6	370426	21	1287	470
miR399i	LOC_Os06g50930.2	149	-24.6	149	-24.6	370427	21	1447	470
miR399i	LOC_Os06g51330.1	162	-21.89	162	-21.89	370485	21	4277	2711
miR399i	LOC_Os06g51330.2	162	-21.89	162	-21.89	370486	21	4223	2657
miR399i	LOC_Os06g51430.1	157	-25	157	-25	370501	21	2037	698
miR399i	LOC_Os06g51460.1	142	-22.04	142	-22.04	370504	21	3495	1211
miR399i	LOC_Os06g51460.2	142	-22.04	142	-22.04	370505	21	3483	1169
miR399i	LOC_Os07g01320.1	159	-23.31	159	-23.31	370563	21	2104	1540

miR399i	LOC_Os07g02600.1	327	-49.04	164	-27.61	370704	21	2619	2063 828
miR399i	LOC_Os07g02760.1	150	-20.1	150	-20.1	370721	21	1979	1762
miR399i	LOC_Os07g02760.2	150	-20.1	150	-20.1	370722	21	1927	1710
miR399i	LOC_Os07g03030.1	161	-21.63	161	-21.63	370754	21	1895	327
miR399i	LOC_Os07g03310.1	156	-24.31	156	-24.31	370794	21	3054	156
miR399i	LOC_Os07g03490.1	156	-24.31	156	-24.31	370803	21	2946	156
miR399i	LOC_Os07g03790.1	140	-20.66	140	-20.66	370832	21	1971	1122
miR399i	LOC_Os07g03830.1	143	-20.65	143	-20.65	370835	21	2016	1170
miR399i	LOC_Os07g05500.1	147	-21.7	147	-21.7	371012	21	966	510
miR399i	LOC_Os07g06274.1	153	-21.08	153	-21.08	371099	21	5895	4111
miR399i	LOC_Os07g06610.1	157	-21.03	157	-21.03	371129	21	3023	920
miR399i	LOC_Os07g07020.2	150	-21.47	150	-21.47	371181	21	1385	1144
miR399i	LOC_Os07g07840.1	153	-21.08	153	-21.08	371273	21	2799	1000
miR399i	LOC_Os07g07910.1	168	-21.02	168	-21.02	371281	21	3016	1124
miR399i	LOC_Os07g08140.1	159	-21.32	159	-21.32	371301	21	1967	257
miR399i	LOC_Os07g08660.1	161	-21.14	161	-21.14	371358	21	838	691
miR399i	LOC_Os07g09010.1	159	-23.98	159	-23.98	371399	21	2437	1484
miR399i	LOC_Os07g09090.1	166	-26.65	166	-26.65	371410	21	1431	1108
miR399i	LOC_Os07g09900.1	142	-21.6	142	-21.6	371494	21	2082	183
miR399i	LOC_Os07g10150.1	163	-21.07	163	-21.07	371519	21	3204	1192
miR399i	LOC_Os07g10254.1	151	-21.81	151	-21.81	371530	21	701	228
miR399i	LOC_Os07g10470.1	149	-20.5	149	-20.5	371555	21	2130	1784
miR399i	LOC_Os07g10610.2	148	-22.05	148	-22.05	371574	21	4614	1052
miR399i	LOC_Os07g11240.1	140	-23.66	140	-23.66	371652	21	5226	1845
miR399i	LOC_Os07g11270.1	157	-22.78	157	-22.78	371655	21	2682	2327
miR399i	LOC_Os07g12040.1	150	-20.21	150	-20.21	371725	21	459	130
miR399i	LOC_Os07g12130.1	165	-25.82	165	-25.82	371734	21	1482	1100
miR399i	LOC_Os07g12700.1	155	-25.12	155	-25.12	371800	21	282	65
miR399i	LOC_Os07g13130.1	157	-25.76	157	-25.76	371844	21	2358	1988
miR399i	LOC_Os07g13534.1	156	-20.79	156	-20.79	371885	21	697	272
miR399i	LOC_Os07g13680.1	156	-22.53	156	-22.53	371897	21	3288	166
miR399i	LOC_Os07g13980.1	157	-22.64	157	-22.64	371925	21	2753	429

miR399i	LOC_Os07g13969.1	157	-22.64	157	-22.64	371926	21	1095	429
miR399i	LOC_Os07g14650.1	161	-23.7	161	-23.7	372010	21	984	5
miR399i	LOC_Os07g14650.2	161	-23.7	161	-23.7	372011	21	1084	5
miR399i	LOC_Os07g14990.1	314	-41.1	157	-20.97	372044	21	3213	1601 1654
miR399i	LOC_Os07g15050.1	141	-20.33	141	-20.33	372050	21	2043	1295
miR399i	LOC_Os07g15500.1	161	-24.4	161	-24.4	372096	21	1355	690
miR399i	LOC_Os07g15610.1	160	-21.24	160	-21.24	372107	21	258	19
miR399i	LOC_Os07g16000.1	163	-22.99	163	-22.99	372147	21	4893	437
miR399i	LOC_Os07g16030.1	155	-21.71	155	-21.71	372149	21	3314	1213
miR399i	LOC_Os07g16560.1	150	-21.02	150	-21.02	372195	21	5169	3767
miR399i	LOC_Os07g17210.1	150	-25.33	150	-25.33	372263	21	2193	2086
miR399i	LOC_Os07g17360.1	154	-20.86	154	-20.86	372280	21	2672	231
miR399i	LOC_Os07g17360.2	154	-20.86	154	-20.86	372281	21	2636	231
miR399i	LOC_Os07g17980.1	160	-21.47	160	-21.47	372345	21	732	33
miR399i	LOC_Os07g18370.1	157	-25.26	157	-25.26	372386	21	2499	2168
miR399i	LOC_Os07g18660.1	168	-21.96	168	-21.96	372412	21	432	356
miR399i	LOC_Os07g18944.1	157	-22.12	157	-22.12	372436	21	2462	1921
miR399i	LOC_Os07g19390.1	151	-21.35	151	-21.35	372475	21	954	1
miR399i	LOC_Os07g19470.1	153	-20.25	153	-20.25	372482	21	1300	891
miR399i	LOC_Os07g19470.2	153	-20.25	153	-20.25	372483	21	1286	891
miR399i	LOC_Os07g19470.3	153	-20.25	153	-20.25	372484	21	1399	891
miR399i	LOC_Os07g19580.1	172	-27.86	172	-27.86	372495	21	543	218
miR399i	LOC_Os07g20270.1	163	-25.33	163	-25.33	372515	21	1444	598
miR399i	LOC_Os07g20690.1	161	-26.46	161	-26.46	372557	21	2211	1219
miR399i	LOC_Os07g22390.2	150	-20.69	150	-20.69	372626	21	1661	928
miR399i	LOC_Os07g22400.1	158	-22.03	158	-22.03	372627	21	1941	1048
miR399i	LOC_Os07g22400.2	158	-22.03	158	-22.03	372628	21	1741	848
miR399i	LOC_Os07g22460.1	149	-22.35	149	-22.35	372634	21	588	344
miR399i	LOC_Os07g23040.1	166	-26.64	166	-26.64	372712	21	1323	443
miR399i	LOC_Os07g23200.1	150	-21.22	150	-21.22	372728	21	1365	1028
miR399i	LOC_Os07g23260.1	157	-23.09	157	-23.09	372733	21	1218	955
miR399i	LOC_Os07g24010.1	159	-22.9	159	-22.9	372817	21	465	178

miR399i	LOC_Os07g25090.1	153	-20.56	153	-20.56	372927	21	6018	4222
miR399i	LOC_Os07g25440.1	158	-22.13	158	-22.13	372962	21	2733	1925
miR399i	LOC_Os07g25440.2	158	-22.13	158	-22.13	372963	21	2658	1925
miR399i	LOC_Os07g25680.1	159	-21.59	159	-21.59	372986	21	4485	3894
miR399i	LOC_Os07g26090.1	159	-21.69	159	-21.69	373027	21	879	483
miR399i	LOC_Os07g26140.1	153	-21.48	153	-21.48	373032	21	564	13
miR399i	LOC_Os07g26810.1	153	-20.56	153	-20.56	373105	21	3942	3412
miR399i	LOC_Os07g26840.1	157	-25.76	157	-25.76	373108	21	2310	1940
miR399i	LOC_Os07g27150.1	144	-20.65	144	-20.65	373145	21	1077	384
miR399i	LOC_Os07g27150.2	144	-20.65	144	-20.65	373146	21	1056	363
miR399i	LOC_Os07g27150.3	144	-20.65	144	-20.65	373147	21	1001	308
miR399i	LOC_Os07g27490.1	159	-22.59	159	-22.59	373180	21	2259	1292
miR399i	LOC_Os07g27490.2	159	-22.59	159	-22.59	373181	21	1796	829
miR399i	LOC_Os07g27720.1	142	-22.48	142	-22.48	373202	21	3018	648
miR399i	LOC_Os07g28080.1	159	-21.38	159	-21.38	373241	21	393	143
miR399i	LOC_Os07g28810.1	160	-26.43	160	-26.43	373320	21	966	182
miR399i	LOC_Os07g29150.1	148	-29.41	148	-29.41	373351	21	639	271
miR399i	LOC_Os07g29770.1	151	-24.2	151	-24.2	373423	21	3806	1969
miR399i	LOC_Os07g29770.2	151	-24.2	151	-24.2	373424	21	4270	2433
miR399i	LOC_Os07g29770.3	151	-24.2	151	-24.2	373425	21	4035	2198
miR399i	LOC_Os07g30160.1	170	-28.55	170	-28.55	373466	21	4014	3628
miR399i	LOC_Os07g30170.1	170	-28.55	170	-28.55	373467	21	1700	292
miR399i	LOC_Os07g30420.1	172	-28.89	172	-28.89	373491	21	264	38
miR399i	LOC_Os07g30640.1	167	-23.54	167	-23.54	373511	21	702	235
miR399i	LOC_Os07g30730.1	156	-24.57	156	-24.57	373521	21	4566	2986
miR399i	LOC_Os07g30880.1	168	-27.12	168	-27.12	373540	21	5415	82
miR399i	LOC_Os07g30910.1	156	-24.57	156	-24.57	373543	21	5358	2986
miR399i	LOC_Os07g31590.1	155	-20.52	155	-20.52	373618	21	3315	2217
miR399i	LOC_Os07g31920.1	167	-25.41	167	-25.41	373647	21	1761	1602
miR399i	LOC_Os07g32360.1	167	-24.33	167	-24.33	373694	21	366	40
miR399i	LOC_Os07g32390.1	158	-20.8	158	-20.8	373697	21	2961	1720
miR399i	LOC_Os07g32680.1	149	-25.37	149	-25.37	373732	21	1424	612

miR399i	LOC_Os07g32700.1	143	-21.87	143	-21.87	373734	21	891	301
miR399i	LOC_Os07g32710.1	144	-21.84	144	-21.84	373735	21	1402	312
miR399i	LOC_Os07g33010.1	143	-20.48	143	-20.48	373763	21	6084	5961
miR399i	LOC_Os07g33420.1	143	-20.99	143	-20.99	373809	21	1963	1716
miR399i	LOC_Os07g33420.2	143	-20.99	143	-20.99	373810	21	2047	1723
miR399i	LOC_Os07g33420.3	143	-20.99	143	-20.99	373811	21	2117	1723
miR399i	LOC_Os07g33420.4	143	-20.99	143	-20.99	373812	21	2042	1723
miR399i	LOC_Os07g33450.1	163	-21.83	163	-21.83	373814	21	4422	1311
miR399i	LOC_Os07g33480.1	158	-22.96	158	-22.96	373817	21	1931	540
miR399i	LOC_Os07g33480.2	158	-22.96	158	-22.96	373818	21	2307	540
miR399i	LOC_Os07g33480.4	159	-23.56	159	-23.56	373819	21	2440	1048
miR399i	LOC_Os07g34190.1	154	-26.19	154	-26.19	373887	21	1197	361
miR399i	LOC_Os07g34710.1	152	-24.24	152	-24.24	373949	21	1338	529
miR399i	LOC_Os07g34710.2	152	-24.24	152	-24.24	373950	21	1086	277
miR399i	LOC_Os07g34710.3	152	-24.24	152	-24.24	373951	21	1068	259
miR399i	LOC_Os07g34930.1	165	-27.34	165	-27.34	373972	21	609	153
miR399i	LOC_Os07g35300.1	149	-20.93	149	-20.93	374011	21	2040	1582
miR399i	LOC_Os07g35760.1	159	-22.29	159	-22.29	374072	21	567	22
miR399i	LOC_Os07g36600.1	157	-20.48	157	-20.48	374164	21	1816	238
miR399i	LOC_Os07g36600.2	157	-20.48	157	-20.48	374165	21	1682	238
miR399i	LOC_Os07g36780.1	150	-27.8	150	-27.8	374182	21	2817	1002
miR399i	LOC_Os07g36860.1	152	-21.27	152	-21.27	374192	21	1041	180
miR399i	LOC_Os07g37300.1	152	-24.9	152	-24.9	374243	21	474	309
miR399i	LOC_Os07g37610.1	171	-31.5	171	-31.5	374277	21	3506	3375
miR399i	LOC_Os07g37790.1	162	-22.8	162	-22.8	374295	21	1207	406
miR399i	LOC_Os07g38270.1	153	-22.77	153	-22.77	374351	21	3242	3181
miR399i	LOC_Os07g38360.2	161	-24.14	161	-24.14	374363	21	5071	4396
miR399i	LOC_Os07g38370.1	164	-20.96	164	-20.96	374366	21	1027	777
miR399i	LOC_Os07g38620.1	141	-21.44	141	-21.44	374392	21	1569	839
miR399i	LOC_Os07g38890.1	155	-21.44	155	-21.44	374422	21	2031	559
miR399i	LOC_Os07g39260.1	153	-22.7	153	-22.7	374464	21	1478	899
miR399i	LOC_Os07g39390.1	160	-23.38	160	-23.38	374479	21	2023	1756

miR399i	LOC_Os07g40310.1	157	-25.25	157	-25.25	374587	21	480	455
miR399i	LOC_Os07g40320.1	159	-20.98	159	-20.98	374588	21	1119	887
miR399i	LOC_Os07g40620.1	170	-30.19	170	-30.19	374619	21	1688	1036
miR399i	LOC_Os07g40670.1	157	-23.09	157	-23.09	374624	21	375	192
miR399i	LOC_Os07g40750.1	155	-25.11	155	-25.11	374635	21	1827	1387
miR399i	LOC_Os07g40850.1	467	-69.63	157	-23.98	374646	21	1012	415 619 754
miR399i	LOC_Os07g40860.1	314	-46.94	157	-24.87	374647	21	1145	547 766
miR399i	LOC_Os07g40860.2	314	-46.94	157	-24.87	374648	21	1099	501 720
miR399i	LOC_Os07g40890.1	165	-29.14	165	-29.14	374651	21	699	424
miR399i	LOC_Os07g40950.1	159	-25.14	159	-25.14	374657	21	984	327
miR399i	LOC_Os07g41400.1	157	-20.93	157	-20.93	374705	21	460	192
miR399i	LOC_Os07g41430.1	149	-20.5	149	-20.5	374708	21	5007	2420
miR399i	LOC_Os07g41694.1	153	-23.92	153	-23.92	374741	21	1878	1087
miR399i	LOC_Os07g42234.1	151	-20.18	151	-20.18	374799	21	4335	1611
miR399i	LOC_Os07g42370.1	144	-20.04	144	-20.04	374811	21	1405	479
miR399i	LOC_Os07g42370.2	144	-20.04	144	-20.04	374812	21	1712	479
miR399i	LOC_Os07g42370.3	144	-20.04	144	-20.04	374813	21	1056	479
miR399i	LOC_Os07g42680.1	140	-22.49	140	-22.49	374858	21	3408	1606
miR399i	LOC_Os07g42900.1	155	-23.01	155	-23.01	374885	21	4108	1721
miR399i	LOC_Os07g43380.1	154	-20.66	154	-20.66	374950	21	1672	1180
miR399i	LOC_Os07g44540.1	161	-21.95	161	-21.95	375073	21	2961	1103
miR399i	LOC_Os07g44630.1	155	-22.47	155	-22.47	375086	21	1180	278
miR399i	LOC_Os07g44630.2	155	-22.47	155	-22.47	375087	21	1273	278
miR399i	LOC_Os07g44670.1	158	-20.47	158	-20.47	375091	21	2895	277
miR399i	LOC_Os07g45330.1	159	-20.47	159	-20.47	375158	21	1663	489
miR399i	LOC_Os07g45650.1	156	-20.19	156	-20.19	375191	21	5967	2501
miR399i	LOC_Os07g45850.1	157	-20.24	157	-20.24	375211	21	1116	113
miR399i	LOC_Os07g46270.1	153	-21.08	153	-21.08	375253	21	2949	1150
miR399i	LOC_Os07g46590.1	157	-20.18	157	-20.18	375298	21	3775	1188
miR399i	LOC_Os07g47010.1	149	-20.5	149	-20.5	375361	21	2337	2162
miR399i	LOC_Os07g47201.1	151	-22.74	151	-22.74	375392	21	1582	624
miR399i	LOC_Os07g47490.1	151	-20.21	151	-20.21	375431	21	3384	2076

miR399i	LOC_Os07g47490.2	151	-20.21	151	-20.21	375432	21	5148	2103
miR399i	LOC_Os07g47490.3	151	-20.21	151	-20.21	375433	21	2509	2076
miR399i	LOC_Os07g47510.1	151	-22.15	151	-22.15	375439	21	1464	1209
miR399i	LOC_Os07g47510.2	151	-22.15	151	-22.15	375440	21	993	738
miR399i	LOC_Os07g47580.1	159	-24.01	159	-24.01	375448	21	1229	791
miR399i	LOC_Os07g47580.2	159	-24.01	159	-24.01	375449	21	1509	1071
miR399i	LOC_Os07g47580.3	159	-24.01	159	-24.01	375450	21	1366	791
miR399i	LOC_Os07g47720.1	165	-25.28	165	-25.28	375472	21	2436	311
miR399i	LOC_Os07g47740.1	153	-21.08	153	-21.08	375474	21	5895	4111
miR399i	LOC_Os07g47780.1	150	-22.13	150	-22.13	375478	21	901	517
miR399i	LOC_Os07g47780.2	150	-22.13	150	-22.13	375479	21	877	493
miR399i	LOC_Os07g47820.1	151	-20.97	151	-20.97	375484	21	3787	540
miR399i	LOC_Os07g47820.2	151	-20.97	151	-20.97	375485	21	3765	540
miR399i	LOC_Os07g47920.1	166	-24.99	166	-24.99	375496	21	2601	1729
miR399i	LOC_Os07g48290.1	153	-21.04	153	-21.04	375538	21	2436	676
miR399i	LOC_Os07g48290.2	153	-21.04	153	-21.04	375539	21	2412	676
miR399i	LOC_Os07g48340.1	150	-23.51	150	-23.51	375545	21	851	620
miR399i	LOC_Os07g48340.2	150	-23.51	150	-23.51	375546	21	1184	750
miR399i	LOC_Os07g48720.1	154	-20.64	154	-20.64	375597	21	3146	1908
miR399i	LOC_Os07g48720.2	154	-20.64	154	-20.64	375598	21	3033	1795
miR399i	LOC_Os07g48720.3	154	-20.64	154	-20.64	375599	21	3343	1961
miR399i	LOC_Os08g03090.1	152	-20.26	152	-20.26	375956	21	1899	1504
miR399i	LOC_Os08g03220.1	157	-20.79	157	-20.79	375969	21	5520	3662
miR399i	LOC_Os08g03360.1	153	-20.07	153	-20.07	375989	21	1949	778
miR399i	LOC_Os08g04070.1	158	-20.98	158	-20.98	376072	21	693	599
miR399i	LOC_Os08g04300.1	162	-25.05	162	-25.05	376103	21	2415	262
miR399i	LOC_Os08g04630.1	163	-24.23	163	-24.23	376140	21	2636	1067
miR399i	LOC_Os08g04950.1	156	-24.57	156	-24.57	376180	21	5187	2701
miR399i	LOC_Os08g05120.1	313	-40.35	157	-20.19	376199	21	5976	569 2510
miR399i	LOC_Os08g05560.1	160	-22.2	160	-22.2	376243	21	2319	2209
miR399i	LOC_Os08g05590.1	153	-24.7	153	-24.7	376247	21	1302	675
miR399i	LOC_Os08g05600.1	150	-22.91	150	-22.91	376248	21	837	205

miR399i	LOC_Os08g05930.1	153	-20.56	153	-20.56	376284	21	5592	3808
miR399i	LOC_Os08g06360.1	152	-21.14	152	-21.14	376337	21	1389	1021
miR399i	LOC_Os08g06760.1	160	-21.46	160	-21.46	376385	21	504	106
miR399i	LOC_Os08g07380.1	151	-20.09	151	-20.09	376444	21	5334	3108
miR399i	LOC_Os08g08140.1	159	-23.27	159	-23.27	376525	21	3297	1706
miR399i	LOC_Os08g08910.1	157	-25.05	157	-25.05	376611	21	162	103
miR399i	LOC_Os08g10060.1	153	-20.56	153	-20.56	376740	21	6042	4258
miR399i	LOC_Os08g10140.1	157	-22.26	157	-22.26	376749	21	2436	1166
miR399i	LOC_Os08g11010.1	151	-21.16	151	-21.16	376846	21	1509	546
miR399i	LOC_Os08g11150.1	332	-52.64	170	-30.08	376860	21	4680	2754 2063
miR399i	LOC_Os08g11190.1	152	-22.01	152	-22.01	376864	21	2094	1676
miR399i	LOC_Os08g11500.1	159	-22.4	159	-22.4	376902	21	1335	125
miR399i	LOC_Os08g11810.1	146	-20.55	146	-20.55	376932	21	2097	182
miR399i	LOC_Os08g12140.1	157	-21.11	157	-21.11	376964	21	2220	2024
miR399i	LOC_Os08g12170.1	161	-24.39	161	-24.39	376967	21	561	515
miR399i	LOC_Os08g12380.1	157	-25.76	157	-25.76	376986	21	1659	1289
miR399i	LOC_Os08g12960.1	152	-21.89	152	-21.89	377053	21	921	612
miR399i	LOC_Os08g13520.1	157	-20.97	157	-20.97	377113	21	1845	865
miR399i	LOC_Os08g13620.1	153	-20.56	153	-20.56	377123	21	5736	4150
miR399i	LOC_Os08g13640.1	162	-23.41	162	-23.41	377125	21	1155	159
miR399i	LOC_Os08g14390.1	159	-26.96	159	-26.96	377200	21	1173	97
miR399i	LOC_Os08g15300.1	160	-22.04	160	-22.04	377314	21	3561	2566
miR399i	LOC_Os08g15900.1	155	-20.34	155	-20.34	377378	21	2008	1425
miR399i	LOC_Os08g16010.1	158	-20.84	158	-20.84	377389	21	1186	972
miR399i	LOC_Os08g16010.2	158	-20.84	158	-20.84	377390	21	1044	851
miR399i	LOC_Os08g16280.1	151	-20.47	151	-20.47	377419	21	2736	2028
miR399i	LOC_Os08g16680.1	327	-49.04	164	-27.61	377463	21	3558	3074 1266
miR399i	LOC_Os08g17020.1	146	-20.26	146	-20.26	377500	21	825	229
miR399i	LOC_Os08g19540.1	159	-22.7	159	-22.7	377710	21	3897	1862
miR399i	LOC_Os08g19800.1	166	-25.11	166	-25.11	377739	21	1104	483
miR399i	LOC_Os08g19980.1	167	-22.88	167	-22.88	377757	21	3393	2053
miR399i	LOC_Os08g21110.1	142	-23.41	142	-23.41	377855	21	6117	5728

miR399i	LOC_Os08g21400.1	167	-26.23	167	-26.23	377881	21	192	65
miR399i	LOC_Os08g21420.1	167	-26.23	167	-26.23	377883	21	1593	65
miR399i	LOC_Os08g21800.1	142	-23.41	142	-23.41	377917	21	3618	3229
miR399i	LOC_Os08g21970.1	157	-25.26	157	-25.26	377934	21	2334	1964
miR399i	LOC_Os08g22006.1	149	-20.48	149	-20.48	377939	21	2094	1015
miR399i	LOC_Os08g22028.1	157	-25.26	157	-25.26	377948	21	2334	1964
miR399i	LOC_Os08g22020.1	149	-20.48	149	-20.48	377952	21	1842	745
miR399i	LOC_Os08g22080.1	149	-20.48	149	-20.48	377955	21	2196	1015
miR399i	LOC_Os08g22480.1	140	-20.89	140	-20.89	377985	21	2334	1451
miR399i	LOC_Os08g22630.1	153	-23.38	153	-23.38	377998	21	2055	882
miR399i	LOC_Os08g22864.1	151	-25.34	151	-25.34	378018	21	3559	2372
miR399i	LOC_Os08g22864.2	151	-25.34	151	-25.34	378019	21	3664	2362
miR399i	LOC_Os08g23240.1	153	-24.38	153	-24.38	378058	21	1719	207
miR399i	LOC_Os08g24270.1	153	-20.56	153	-20.56	378167	21	5925	4192
miR399i	LOC_Os08g24310.1	155	-21.35	155	-21.35	378171	21	3277	3213
miR399i	LOC_Os08g24650.1	153	-20.56	153	-20.56	378206	21	5895	4111
miR399i	LOC_Os08g24680.1	153	-24.9	153	-24.9	378209	21	2880	492
miR399i	LOC_Os08g25640.1	172	-28.87	172	-28.87	378306	21	3330	207
miR399i	LOC_Os08g26400.1	149	-20.48	149	-20.48	378383	21	1764	37
miR399i	LOC_Os08g27000.1	149	-20.5	149	-20.5	378448	21	4197	2102
miR399i	LOC_Os08g27280.1	153	-21.08	153	-21.08	378477	21	5892	4111
miR399i	LOC_Os08g27560.1	148	-24.94	148	-24.94	378505	21	465	81
miR399i	LOC_Os08g28360.1	145	-26.87	145	-26.87	378592	21	1944	1373
miR399i	LOC_Os08g28600.1	144	-20.64	144	-20.64	378615	21	2901	345
miR399i	LOC_Os08g29400.1	145	-20.9	145	-20.9	378698	21	3608	2708
miR399i	LOC_Os08g29400.2	145	-20.9	145	-20.9	378699	21	3139	2239
miR399i	LOC_Os08g29480.1	153	-24.9	153	-24.9	378706	21	2415	615
miR399i	LOC_Os08g29930.1	153	-20.56	153	-20.56	378750	21	5988	4192
miR399i	LOC_Os08g30550.1	158	-20.57	158	-20.57	378816	21	2712	2563
miR399i	LOC_Os08g31219.1	154	-23.53	154	-23.53	378884	21	944	808
miR399i	LOC_Os08g31228.1	154	-23.53	154	-23.53	378885	21	1546	1076
miR399i	LOC_Os08g31370.1	164	-26.13	164	-26.13	378898	21	2670	1043

miR399i	LOC_Os08g31980.1	169	-26.08	169	-26.08	378973	21	3289	875
miR399i	LOC_Os08g32300.1	152	-26.16	152	-26.16	379007	21	2280	2074
miR399i	LOC_Os08g34690.1	162	-26.37	162	-26.37	379279	21	1695	1363
miR399i	LOC_Os08g35020.1	157	-26.03	157	-26.03	379318	21	5099	4840
miR399i	LOC_Os08g35080.1	156	-23.39	156	-23.39	379325	21	285	30
miR399i	LOC_Os08g36490.1	170	-24.65	170	-24.65	379485	21	2848	2473
miR399i	LOC_Os08g36510.1	158	-21.69	158	-21.69	379487	21	915	84
miR399i	LOC_Os08g36774.1	161	-20.75	161	-20.75	379514	21	1953	725
miR399i	LOC_Os08g37750.1	173	-32.29	173	-32.29	379630	21	1614	1440
miR399i	LOC_Os08g38070.1	154	-20.84	154	-20.84	379673	21	558	325
miR399i	LOC_Os08g38640.1	153	-20.16	153	-20.16	379743	21	4116	2861
miR399i	LOC_Os08g38970.1	148	-20.18	148	-20.18	379788	21	3554	2715
miR399i	LOC_Os08g39950.1	147	-27.82	147	-27.82	379909	21	2739	1461
miR399i	LOC_Os08g40090.1	168	-23.61	168	-23.61	379924	21	2804	447
miR399i	LOC_Os08g40530.1	153	-20.33	153	-20.33	379970	21	2394	1314
miR399i	LOC_Os08g40530.2	153	-20.33	153	-20.33	379971	21	2804	1724
miR399i	LOC_Os08g40640.1	327	-49.04	164	-27.61	379991	21	5181	3572 1551
miR399i	LOC_Os08g40650.1	150	-22.81	150	-22.81	379992	21	2370	568
miR399i	LOC_Os08g41070.1	158	-20.47	158	-20.47	380048	21	4194	400
miR399i	LOC_Os08g41550.1	164	-27.38	164	-27.38	380108	21	4852	2091
miR399i	LOC_Os08g41630.1	153	-21.71	153	-21.71	380116	21	3265	990
miR399i	LOC_Os08g41660.1	151	-22.11	151	-22.11	380118	21	582	151
miR399i	LOC_Os08g43290.1	155	-20.03	155	-20.03	380316	21	601	560
miR399i	LOC_Os08g43300.1	157	-24.63	157	-24.63	380317	21	2181	556
miR399i	LOC_Os08g43400.1	157	-22.09	157	-22.09	380329	21	3403	1038
miR399i	LOC_Os08g43400.2	157	-22.09	157	-22.09	380330	21	3381	1038
miR399i	LOC_Os08g43400.3	157	-22.09	157	-22.09	380331	21	3561	1035
miR399i	LOC_Os08g43400.4	157	-22.09	157	-22.09	380332	21	3368	1038
miR399i	LOC_Os08g43910.1	167	-28.05	167	-28.05	380386	21	609	69
miR399i	LOC_Os08g44150.1	171	-25.59	171	-25.59	380415	21	1611	1226
miR399i	LOC_Os08g44740.1	167	-24.73	167	-24.73	380492	21	444	305
miR399i	LOC_Os08g44780.1	150	-22.64	150	-22.64	380499	21	1700	1134

miR399i	LOC_Os08g45000.1	354	-62.08	183	-34.98	380535	21	1827	292 386
miR399i	LOC_Os08g45260.1	155	-24.66	155	-24.66	380571	21	1467	941
miR399i	LOC_Os09g01030.1	151	-20.41	151	-20.41	380580	21	624	313
miR399i	LOC_Os09g01310.1	153	-20.89	153	-20.89	380610	21	1056	85
miR399i	LOC_Os09g01340.1	154	-22.63	154	-22.63	380613	21	5541	2059
miR399i	LOC_Os09g01370.1	161	-22.93	161	-22.93	380616	21	2520	1817
miR399i	LOC_Os09g01560.1	153	-20.56	153	-20.56	380636	21	5835	4051
miR399i	LOC_Os09g01570.1	153	-20.95	153	-20.95	380637	21	3246	40
miR399i	LOC_Os09g01850.1	153	-20.56	153	-20.56	380672	21	2937	1153
miR399i	LOC_Os09g01880.1	154	-23.84	154	-23.84	380675	21	1023	107
miR399i	LOC_Os09g02040.1	163	-26.69	163	-26.69	380690	21	1599	781
miR399i	LOC_Os09g02150.1	150	-22.65	150	-22.65	380701	21	651	61
miR399i	LOC_Os09g02820.1	155	-25.8	155	-25.8	380773	21	1116	44
miR399i	LOC_Os09g03210.1	155	-23.82	155	-23.82	380811	21	1280	195
miR399i	LOC_Os09g03500.1	157	-24.92	157	-24.92	380839	21	1596	205
miR399i	LOC_Os09g03620.1	161	-24.68	161	-24.68	380854	21	2412	1802
miR399i	LOC_Os09g03630.1	162	-23.71	162	-23.71	380855	21	1785	1346
miR399i	LOC_Os09g03860.1	158	-21.7	158	-21.7	380874	21	3492	1988
miR399i	LOC_Os09g03900.1	150	-21.09	150	-21.09	380878	21	2418	51
miR399i	LOC_Os09g04220.1	162	-22.19	162	-22.19	380911	21	1365	398
miR399i	LOC_Os09g04680.1	165	-25.47	165	-25.47	380961	21	1155	66
miR399i	LOC_Os09g04830.1	146	-24.06	146	-24.06	380978	21	3432	3067
miR399i	LOC_Os09g06150.1	154	-20.43	154	-20.43	381013	21	6033	5808
miR399i	LOC_Os09g06450.1	153	-21.17	153	-21.17	381043	21	2130	813
miR399i	LOC_Os09g07070.1	140	-20.83	140	-20.83	381105	21	4188	1595
miR399i	LOC_Os09g07120.1	153	-21.07	153	-21.07	381110	21	3754	2636
miR399i	LOC_Os09g07120.2	153	-21.07	153	-21.07	381111	21	3650	2532
miR399i	LOC_Os09g07230.1	140	-23.66	140	-23.66	381122	21	3693	642
miR399i	LOC_Os09g07300.1	166	-23.82	166	-23.82	381130	21	13113	7844
miR399i	LOC_Os09g07590.1	151	-20.3	151	-20.3	381167	21	834	288
miR399i	LOC_Os09g07610.1	148	-20.83	148	-20.83	381169	21	3486	3173
miR399i	LOC_Os09g07640.1	157	-25.04	157	-25.04	381173	21	3891	2481

miR399i	LOC_Os09g07690.1	157	-24.73	157	-24.73	381179	21	2223	712
miR399i	LOC_Os09g07960.1	161	-21.95	161	-21.95	381210	21	4074	2237
miR399i	LOC_Os09g08030.1	161	-23.73	161	-23.73	381217	21	621	40
miR399i	LOC_Os09g08340.1	147	-20.78	147	-20.78	381243	21	4083	3621
miR399i	LOC_Os09g08440.1	163	-21.35	163	-21.35	381259	21	1128	43
miR399i	LOC_Os09g09000.1	310	-49.32	155	-24.66	381316	21	8022	6584 7550
miR399i	LOC_Os09g09020.1	155	-24.66	155	-24.66	381318	21	1383	911
miR399i	LOC_Os09g09080.1	155	-24.66	155	-24.66	381324	21	1467	941
miR399i	LOC_Os09g09220.1	144	-21.54	144	-21.54	381338	21	2529	610
miR399i	LOC_Os09g09470.1	158	-20.94	158	-20.94	381364	21	3002	1429
miR399i	LOC_Os09g09470.2	158	-20.94	158	-20.94	381365	21	2874	1316
miR399i	LOC_Os09g10190.1	162	-22.55	162	-22.55	381435	21	348	119
miR399i	LOC_Os09g10390.1	140	-23.66	140	-23.66	381458	21	2562	2334
miR399i	LOC_Os09g10540.1	140	-20.45	140	-20.45	381472	21	4686	3981
miR399i	LOC_Os09g10840.1	163	-20.76	163	-20.76	381503	21	2069	1196
miR399i	LOC_Os09g10840.2	163	-20.76	163	-20.76	381504	21	2093	1220
miR399i	LOC_Os09g11530.1	165	-29.83	165	-29.83	381580	21	828	537
miR399i	LOC_Os09g11550.1	316	-41.5	163	-20.95	381582	21	3246	1268 40
miR399i	LOC_Os09g11610.1	316	-43.27	161	-22.4	381588	21	3738	3118 3169
miR399i	LOC_Os09g12090.1	164	-21.43	164	-21.43	381634	21	2889	2333
miR399i	LOC_Os09g13140.1	155	-24.66	155	-24.66	381743	21	1413	941
miR399i	LOC_Os09g13830.1	157	-20.29	157	-20.29	381815	21	669	279
miR399i	LOC_Os09g14610.1	160	-21.09	160	-21.09	381897	21	2410	1292
miR399i	LOC_Os09g14610.2	160	-21.09	160	-21.09	381898	21	3004	1292
miR399i	LOC_Os09g14610.3	160	-21.09	160	-21.09	381899	21	3306	1292
miR399i	LOC_Os09g14770.1	157	-22.55	157	-22.55	381919	21	2373	1286
miR399i	LOC_Os09g15010.1	142	-22.13	142	-22.13	381944	21	3999	1792
miR399i	LOC_Os09g15090.1	157	-23.87	157	-23.87	381952	21	2514	2076
miR399i	LOC_Os09g15140.1	155	-22.98	155	-22.98	381956	21	2259	1233
miR399i	LOC_Os09g15480.1	159	-23.68	159	-23.68	381998	21	1194	718
miR399i	LOC_Os09g15480.2	159	-23.68	159	-23.68	381999	21	1168	692
miR399i	LOC_Os09g16030.1	146	-22.4	146	-22.4	382057	21	1777	768

miR399i	LOC_Os09g16290.1	145	-21.52	145	-21.52	382083	21	4062	1743
miR399i	LOC_Os09g16600.1	156	-22.37	156	-22.37	382116	21	943	619
miR399i	LOC_Os09g17030.1	156	-24.57	156	-24.57	382161	21	5409	2986
miR399i	LOC_Os09g17240.1	150	-20.19	150	-20.19	382183	21	567	435
miR399i	LOC_Os09g17660.1	158	-25.38	158	-25.38	382225	21	758	203
miR399i	LOC_Os09g18450.1	157	-24	157	-24	382302	21	1073	578
miR399i	LOC_Os09g18490.1	157	-20.26	157	-20.26	382306	21	2640	1021
miR399i	LOC_Os09g19190.1	309	-49.24	163	-27.61	382323	21	2472	795 2393
miR399i	LOC_Os09g19260.1	320	-48.1	166	-27.61	382328	21	1668	911 39
miR399i	LOC_Os09g20710.1	143	-20.38	143	-20.38	382490	21	651	514
miR399i	LOC_Os09g20780.1	142	-20.43	142	-20.43	382497	21	288	152
miR399i	LOC_Os09g21340.1	163	-21.66	163	-21.66	382558	21	2834	1438
miR399i	LOC_Os09g21610.1	153	-20.56	153	-20.56	382585	21	6042	4258
miR399i	LOC_Os09g21710.1	149	-21.1	149	-21.1	382597	21	890	168
miR399i	LOC_Os09g22060.1	157	-22.55	157	-22.55	382633	21	783	140
miR399i	LOC_Os09g23070.1	156	-22.69	156	-22.69	382683	21	2042	385
miR399i	LOC_Os09g23070.2	156	-22.69	156	-22.69	382684	21	2169	385
miR399i	LOC_Os09g23140.1	162	-23.44	162	-23.44	382690	21	1678	850
miR399i	LOC_Os09g23140.2	162	-23.44	162	-23.44	382691	21	1668	850
miR399i	LOC_Os09g23690.1	165	-26.11	165	-26.11	382752	21	1738	930
miR399i	LOC_Os09g23899.1	144	-21.61	144	-21.61	382775	21	811	723
miR399i	LOC_Os09g23908.1	144	-21.61	144	-21.61	382776	21	645	573
miR399i	LOC_Os09g23930.1	144	-21.61	144	-21.61	382778	21	615	595
miR399i	LOC_Os09g23957.1	144	-21.61	144	-21.61	382779	21	738	718
miR399i	LOC_Os09g23966.1	144	-21.61	144	-21.61	382780	21	909	821
miR399i	LOC_Os09g23948.1	144	-21.61	144	-21.61	382781	21	661	607
miR399i	LOC_Os09g23939.1	144	-21.61	144	-21.61	382782	21	405	385
miR399i	LOC_Os09g23999.1	144	-21.61	144	-21.61	382785	21	769	681
miR399i	LOC_Os09g24020.1	144	-21.61	144	-21.61	382786	21	815	743
miR399i	LOC_Os09g24008.1	144	-21.61	144	-21.61	382787	21	736	650
miR399i	LOC_Os09g24038.1	144	-21.61	144	-21.61	382788	21	789	703
miR399i	LOC_Os09g24029.1	144	-21.61	144	-21.61	382789	21	405	385

miR399i	LOC_Os09g24110.1	144	-21.61	144	-21.61	382796	21	753	688
miR399i	LOC_Os09g24120.1	144	-21.61	144	-21.61	382797	21	754	680
miR399i	LOC_Os09g24130.1	144	-21.61	144	-21.61	382798	21	731	711
miR399i	LOC_Os09g24140.1	144	-21.61	144	-21.61	382799	21	808	720
miR399i	LOC_Os09g24150.1	144	-21.61	144	-21.61	382800	21	591	571
miR399i	LOC_Os09g24159.1	144	-21.61	144	-21.61	382801	21	683	609
miR399i	LOC_Os09g24240.1	158	-21.81	158	-21.81	382810	21	2790	1929
miR399i	LOC_Os09g24320.1	155	-20.83	155	-20.83	382818	21	1791	1043
miR399i	LOC_Os09g25960.1	158	-23.42	158	-23.42	383000	21	2802	603
miR399i	LOC_Os09g26560.1	169	-24.59	169	-24.59	383067	21	1927	1105
miR399i	LOC_Os09g26560.2	169	-24.59	169	-24.59	383068	21	1850	1028
miR399i	LOC_Os09g26720.1	147	-21.25	147	-21.25	383089	21	963	154
miR399i	LOC_Os09g27040.1	167	-21.04	167	-21.04	383125	21	2429	1664
miR399i	LOC_Os09g27250.1	146	-23.3	146	-23.3	383150	21	1552	741
miR399i	LOC_Os09g27840.1	160	-21.34	160	-21.34	383231	21	582	236
miR399i	LOC_Os09g27970.1	163	-20.32	163	-20.32	383247	21	2683	2024
miR399i	LOC_Os09g27970.2	163	-20.32	163	-20.32	383248	21	2824	2165
miR399i	LOC_Os09g27970.3	163	-20.32	163	-20.32	383249	21	3153	2494
miR399i	LOC_Os09g27970.4	163	-20.32	163	-20.32	383250	21	3167	2508
miR399i	LOC_Os09g27970.5	163	-20.32	163	-20.32	383251	21	3078	2419
miR399i	LOC_Os09g27980.1	150	-20.16	150	-20.16	383252	21	1243	594
miR399i	LOC_Os09g27990.1	150	-20.16	150	-20.16	383253	21	1693	31
miR399i	LOC_Os09g28300.1	318	-47.02	161	-25.81	383285	21	2908	429 713
miR399i	LOC_Os09g28300.2	316	-46.43	159	-25.81	383286	21	2570	92 375
miR399i	LOC_Os09g28880.1	147	-25.49	147	-25.49	383348	21	3624	2675
miR399i	LOC_Os09g29430.1	169	-26.69	169	-26.69	383411	21	1943	1505
miR399i	LOC_Os09g29600.1	164	-24.23	164	-24.23	383430	21	2475	1392
miR399i	LOC_Os09g30380.1	158	-22.52	158	-22.52	383529	21	3415	1903
miR399i	LOC_Os09g30458.1	164	-21.65	164	-21.65	383545	21	2684	217
miR399i	LOC_Os09g32300.1	149	-23.43	149	-23.43	383697	21	2763	2336
miR399i	LOC_Os09g32300.2	149	-23.43	149	-23.43	383698	21	2690	2263
miR399i	LOC_Os09g32300.3	149	-23.43	149	-23.43	383699	21	2993	2566

miR399i	LOC_Os09g32450.1	159	-21.2	159	-21.2	383713	21	3083	1941
miR399i	LOC_Os09g32650.1	151	-21.68	151	-21.68	383741	21	3713	290
miR399i	LOC_Os09g32740.1	162	-24.2	162	-24.2	383754	21	3952	3809
miR399i	LOC_Os09g32930.1	151	-21.77	151	-21.77	383775	21	2197	1812
miR399i	LOC_Os09g32930.3	151	-21.77	151	-21.77	383776	21	2190	1809
miR399i	LOC_Os09g33510.1	156	-21.92	156	-21.92	383807	21	2403	1779
miR399i	LOC_Os09g33510.2	156	-21.92	156	-21.92	383808	21	2315	1779
miR399i	LOC_Os09g33530.1	154	-21.44	154	-21.44	383810	21	2152	1198
miR399i	LOC_Os09g33530.2	154	-21.44	154	-21.44	383811	21	2315	1383
miR399i	LOC_Os09g33720.1	152	-22.15	152	-22.15	383837	21	1738	1522
miR399i	LOC_Os09g34060.1	146	-23.26	146	-23.26	383891	21	2099	2044
miR399i	LOC_Os09g34070.1	152	-23.68	152	-23.68	383892	21	3540	2076
miR399i	LOC_Os09g34070.2	152	-23.68	152	-23.68	383893	21	3216	1752
miR399i	LOC_Os09g35540.1	160	-21.07	160	-21.07	383955	21	1467	695
miR399i	LOC_Os09g35830.1	158	-21.51	158	-21.51	383991	21	2590	847
miR399i	LOC_Os09g35830.2	158	-21.51	158	-21.51	383992	21	2536	847
miR399i	LOC_Os09g35830.3	158	-21.51	158	-21.51	383993	21	1903	847
miR399i	LOC_Os09g35830.4	158	-21.51	158	-21.51	383994	21	1920	847
miR399i	LOC_Os09g36040.1	157	-29.12	157	-29.12	384017	21	1216	541
miR399i	LOC_Os09g36300.1	150	-20.81	150	-20.81	384052	21	3317	805
miR399i	LOC_Os09g36300.2	150	-20.81	150	-20.81	384053	21	3433	921
miR399i	LOC_Os09g36300.3	150	-20.81	150	-20.81	384054	21	3430	805
miR399i	LOC_Os09g36300.4	150	-20.81	150	-20.81	384055	21	3070	805
miR399i	LOC_Os09g37012.1	166	-25.06	166	-25.06	384140	21	1941	468
miR399i	LOC_Os09g37380.1	147	-23.25	147	-23.25	384180	21	423	193
miR399i	LOC_Os09g37446.1	148	-22.87	148	-22.87	384187	21	2405	1838
miR399i	LOC_Os09g37446.2	148	-22.87	148	-22.87	384188	21	2326	1759
miR399i	LOC_Os09g37949.1	162	-20.88	162	-20.88	384243	21	2054	1500
miR399i	LOC_Os09g38350.1	160	-25.2	160	-25.2	384282	21	1998	1966
miR399i	LOC_Os09g38790.1	162	-21.46	162	-21.46	384346	21	2997	2468
miR399i	LOC_Os09g38790.2	162	-21.46	162	-21.46	384347	21	2865	2336
miR399i	LOC_Os09g38790.3	162	-21.46	162	-21.46	384348	21	1627	1098

miR399i	LOC_Os09g38834.1	148	-20.83	148	-20.83	384351	21	2313	1286
miR399i	LOC_Os09g38970.1	162	-26.98	162	-26.98	384366	21	2753	2046
miR399i	LOC_Os09g39600.1	154	-24.66	154	-24.66	384437	21	636	206
miR399i	LOC_Os09g39640.1	168	-27.06	168	-27.06	384441	21	2636	438
miR399i	LOC_Os09g39650.1	153	-25.64	153	-25.64	384442	21	2298	632
miR399i	LOC_Os09g39650.2	153	-25.64	153	-25.64	384443	21	2269	603
miR399i	LOC_Os09g39650.3	153	-25.64	153	-25.64	384444	21	2324	658
miR399i	LOC_Os10g01110.1	152	-20.78	152	-20.78	384515	21	2015	542
miR399i	LOC_Os10g01460.1	171	-27.47	171	-27.47	384545	21	414	267
miR399i	LOC_Os10g01570.1	154	-22.33	154	-22.33	384558	21	1600	1244
miR399i	LOC_Os10g02700.1	152	-23.38	152	-23.38	384677	21	2178	730
miR399i	LOC_Os10g04240.1	161	-21.95	161	-21.95	384832	21	2784	1103
miR399i	LOC_Os10g05970.1	162	-25.03	162	-25.03	385005	21	1143	572
miR399i	LOC_Os10g05970.2	162	-25.03	162	-25.03	385006	21	981	572
miR399i	LOC_Os10g06130.3	166	-24.99	166	-24.99	385022	21	3655	1774
miR399i	LOC_Os10g06130.4	166	-24.99	166	-24.99	385023	21	2521	640
miR399i	LOC_Os10g06130.5	166	-24.99	166	-24.99	385024	21	2501	620
miR399i	LOC_Os10g06200.1	151	-21.63	151	-21.63	385030	21	2475	2183
miR399i	LOC_Os10g06350.1	165	-22.97	165	-22.97	385045	21	2523	2153
miR399i	LOC_Os10g07010.1	152	-23.94	152	-23.94	385107	21	1896	381
miR399i	LOC_Os10g07200.1	146	-21.83	146	-21.83	385125	21	1343	212
miR399i	LOC_Os10g08014.1	162	-21.64	162	-21.64	385198	21	1521	334
miR399i	LOC_Os10g08474.1	169	-29.86	169	-29.86	385242	21	1706	58
miR399i	LOC_Os10g08540.1	319	-48.39	161	-25.46	385248	21	2968	1056 50
miR399i	LOC_Os10g09410.1	157	-25.26	157	-25.26	385335	21	978	608
miR399i	LOC_Os10g09810.1	153	-20.44	153	-20.44	385370	21	3795	1551
miR399i	LOC_Os10g10420.1	165	-27.45	165	-27.45	385441	21	1347	138
miR399i	LOC_Os10g10798.1	158	-23.34	158	-23.34	385477	21	2560	1897
miR399i	LOC_Os10g11240.1	146	-20.26	146	-20.26	385519	21	828	229
miR399i	LOC_Os10g11454.1	147	-20.09	147	-20.09	385540	21	5238	167
miR399i	LOC_Os10g11860.1	155	-20.12	155	-20.12	385577	21	1744	1272
miR399i	LOC_Os10g12232.1	153	-20.56	153	-20.56	385608	21	2928	1153

miR399i	LOC_Os10g12240.1	156	-20.19	156	-20.19	385609	21	2427	2270
miR399i	LOC_Os10g12260.1	153	-21.1	153	-21.1	385610	21	1278	71
miR399i	LOC_Os10g13370.1	156	-23.73	156	-23.73	385666	21	5379	1372
miR399i	LOC_Os10g13400.1	153	-20.56	153	-20.56	385669	21	5802	4006
miR399i	LOC_Os10g13530.1	140	-20.45	140	-20.45	385682	21	5142	4449
miR399i	LOC_Os10g13980.1	156	-20.19	156	-20.19	385729	21	5895	2507
miR399i	LOC_Os10g14795.1	146	-24.06	146	-24.06	385766	21	4161	2128
miR399i	LOC_Os10g16640.1	140	-25.04	140	-25.04	385855	21	5479	2509
miR399i	LOC_Os10g16640.2	140	-25.04	140	-25.04	385856	21	4865	1895
miR399i	LOC_Os10g17130.1	164	-24.1	164	-24.1	385901	21	387	244
miR399i	LOC_Os10g18294.1	140	-20.45	140	-20.45	386002	21	5052	4278
miR399i	LOC_Os10g18340.1	147	-20.61	147	-20.61	386005	21	892	649
miR399i	LOC_Os10g18340.2	147	-20.61	147	-20.61	386006	21	664	425
miR399i	LOC_Os10g18380.1	169	-29.03	169	-29.03	386011	21	996	790
miR399i	LOC_Os10g18940.1	159	-22.88	159	-22.88	386065	21	2034	1381
miR399i	LOC_Os10g19130.1	153	-20.56	153	-20.56	386082	21	5988	4192
miR399i	LOC_Os10g20710.1	297	-43.36	152	-22.7	386212	21	2550	1292 2220
miR399i	LOC_Os10g20780.1	166	-24.4	166	-24.4	386218	21	1053	402
miR399i	LOC_Os10g20940.1	161	-20.87	161	-20.87	386232	21	1452	1024
miR399i	LOC_Os10g21080.1	153	-20.95	153	-20.95	386244	21	3789	481
miR399i	LOC_Os10g21310.1	165	-25.47	165	-25.47	386285	21	1662	438
miR399i	LOC_Os10g21380.1	162	-28.3	162	-28.3	386310	21	1755	601
miR399i	LOC_Os10g21690.1	165	-23.15	165	-23.15	386339	21	621	555
miR399i	LOC_Os10g21850.1	160	-21.61	160	-21.61	386355	21	4518	4029
miR399i	LOC_Os10g22030.1	165	-21.22	165	-21.22	386376	21	2643	585
miR399i	LOC_Os10g22680.1	146	-20.63	146	-20.63	386447	21	609	503
miR399i	LOC_Os10g22800.1	157	-20.51	157	-20.51	386461	21	1404	580
miR399i	LOC_Os10g24140.1	148	-20.49	148	-20.49	386555	21	390	290
miR399i	LOC_Os10g24660.1	322	-41.69	163	-21.57	386606	21	756	455 346
miR399i	LOC_Os10g24670.1	165	-28.24	165	-28.24	386607	21	693	16
miR399i	LOC_Os10g25674.1	165	-23.95	165	-23.95	386705	21	1179	872
miR399i	LOC_Os10g25674.2	165	-23.95	165	-23.95	386706	21	1037	872

miR399i	LOC_Os10g26230.1	157	-25.26	157	-25.26	386765	21	1212	842
miR399i	LOC_Os10g26360.1	149	-20.5	149	-20.5	386776	21	4779	2300
miR399i	LOC_Os10g26380.1	159	-20.88	159	-20.88	386778	21	432	374
miR399i	LOC_Os10g26570.1	147	-22.17	147	-22.17	386798	21	2369	2334
miR399i	LOC_Os10g27020.1	163	-24.32	163	-24.32	386843	21	903	845
miR399i	LOC_Os10g27040.1	146	-24.06	146	-24.06	386845	21	3783	2080
miR399i	LOC_Os10g27460.1	145	-21.63	145	-21.63	386893	21	555	169
miR399i	LOC_Os10g28150.1	151	-22.19	151	-22.19	386916	21	2403	910
miR399i	LOC_Os10g28340.1	147	-20.74	147	-20.74	386938	21	1682	923
miR399i	LOC_Os10g28340.2	147	-20.74	147	-20.74	386939	21	2309	1457
miR399i	LOC_Os10g28340.3	147	-20.74	147	-20.74	386940	21	2095	1243
miR399i	LOC_Os10g28340.5	147	-20.74	147	-20.74	386941	21	2002	1243
miR399i	LOC_Os10g29400.1	169	-31.48	169	-31.48	387048	21	2334	62
miR399i	LOC_Os10g29560.1	157	-26.04	157	-26.04	387067	21	3798	440
miR399i	LOC_Os10g30020.1	155	-21.94	155	-21.94	387110	21	2529	588
miR399i	LOC_Os10g30130.1	167	-24.71	167	-24.71	387120	21	4959	820
miR399i	LOC_Os10g30690.1	165	-22.82	165	-22.82	387180	21	1301	197
miR399i	LOC_Os10g31170.1	144	-20.65	144	-20.65	387230	21	1596	1423
miR399i	LOC_Os10g32710.1	155	-22.3	155	-22.3	387372	21	2967	1149
miR399i	LOC_Os10g32710.2	155	-22.3	155	-22.3	387373	21	2972	1154
miR399i	LOC_Os10g32710.3	155	-22.3	155	-22.3	387374	21	3050	1232
miR399i	LOC_Os10g32760.1	159	-23.23	159	-23.23	387380	21	2403	8
miR399i	LOC_Os10g33010.1	157	-23.38	157	-23.38	387408	21	5205	3623
miR399i	LOC_Os10g33350.1	161	-22.81	161	-22.81	387441	21	1081	231
miR399i	LOC_Os10g33350.3	161	-22.81	161	-22.81	387442	21	1215	365
miR399i	LOC_Os10g33500.1	150	-20.73	150	-20.73	387458	21	1977	1151
miR399i	LOC_Os10g33720.1	157	-21.75	157	-21.75	387482	21	2580	1005
miR399i	LOC_Os10g33720.3	157	-21.75	157	-21.75	387483	21	2533	958
miR399i	LOC_Os10g34120.1	153	-20.95	153	-20.95	387538	21	3939	145
miR399i	LOC_Os10g34230.1	150	-20.84	150	-20.84	387547	21	2800	2351
miR399i	LOC_Os10g34440.1	156	-24.13	156	-24.13	387569	21	972	407
miR399i	LOC_Os10g35150.1	154	-21.33	154	-21.33	387649	21	1573	1541

miR399i	LOC_Os10g35150.2	154	-21.33	154	-21.33	387650	21	1344	1302
miR399i	LOC_Os10g35520.1	147	-21.95	147	-21.95	387697	21	1387	561
miR399i	LOC_Os10g35530.1	147	-21.95	147	-21.95	387698	21	1182	561
miR399i	LOC_Os10g35530.2	147	-21.95	147	-21.95	387699	21	1275	654
miR399i	LOC_Os10g35540.1	148	-22.22	148	-22.22	387700	21	1200	598
miR399i	LOC_Os10g36229.1	149	-20.66	149	-20.66	387777	21	3729	1307
miR399i	LOC_Os10g36490.1	155	-21.21	155	-21.21	387802	21	3881	2597
miR399i	LOC_Os10g36870.1	145	-20.03	145	-20.03	387845	21	1771	1138
miR399i	LOC_Os10g37720.1	159	-22.25	159	-22.25	387922	21	2242	182
miR399i	LOC_Os10g38234.1	148	-22.06	148	-22.06	387986	21	2205	549
miR399i	LOC_Os10g38350.1	175	-29.34	175	-29.34	387994	21	930	541
miR399i	LOC_Os10g39170.1	154	-20.61	154	-20.61	388082	21	1434	1288
miR399i	LOC_Os10g39910.1	146	-28.17	146	-28.17	388176	21	3845	1779
miR399i	LOC_Os10g39950.1	166	-26.24	166	-26.24	388184	21	5257	3537
miR399i	LOC_Os10g41060.1	154	-20.4	154	-20.4	388314	21	2660	1056
miR399i	LOC_Os10g41180.1	154	-21.52	154	-21.52	388325	21	1920	879
miR399i	LOC_Os10g41430.1	155	-21.43	155	-21.43	388358	21	1080	528
miR399i	LOC_Os10g41490.1	160	-23.05	160	-23.05	388364	21	1520	534
miR399i	LOC_Os10g41490.2	160	-23.05	160	-23.05	388365	21	1495	512
miR399i	LOC_Os10g41490.3	160	-23.05	160	-23.05	388366	21	1334	534
miR399i	LOC_Os10g41620.1	156	-22.06	156	-22.06	388377	21	2082	1117
miR399i	LOC_Os10g41900.1	155	-23.14	155	-23.14	388415	21	2082	191
miR399i	LOC_Os10g42220.1	154	-23.24	154	-23.24	388455	21	630	460
miR399i	LOC_Os10g42410.1	155	-21.27	155	-21.27	388477	21	1928	9
miR399i	LOC_Os10g42410.2	155	-21.27	155	-21.27	388478	21	2394	9
miR399i	LOC_Os10g42410.3	155	-21.27	155	-21.27	388479	21	2021	9
miR399i	LOC_Os11g01280.1	161	-20.47	161	-20.47	388603	21	2646	132
miR399i	LOC_Os11g01330.1	165	-25.09	165	-25.09	388608	21	4019	2303
miR399i	LOC_Os11g02670.1	159	-22.05	159	-22.05	388772	21	3358	2817
miR399i	LOC_Os11g02740.1	145	-20.12	145	-20.12	388779	21	2552	765
miR399i	LOC_Os11g02830.1	156	-26.62	156	-26.62	388790	21	1924	968
miR399i	LOC_Os11g02830.2	156	-26.62	156	-26.62	388791	21	1919	968

miR399i	LOC_Os11g03830.1	161	-23.16	161	-23.16	388900	21	3417	1335
miR399i	LOC_Os11g04190.1	163	-27.65	163	-27.65	388942	21	2281	1023
miR399i	LOC_Os11g04570.1	157	-26.04	157	-26.04	388993	21	2665	553
miR399i	LOC_Os11g04800.1	147	-23.09	147	-23.09	389018	21	2535	127
miR399i	LOC_Os11g04980.1	144	-20.34	144	-20.34	389040	21	2788	540
miR399i	LOC_Os11g04980.2	144	-20.34	144	-20.34	389041	21	2782	540
miR399i	LOC_Os11g05030.1	140	-20.13	140	-20.13	389049	21	2985	2466
miR399i	LOC_Os11g05340.1	155	-21.43	155	-21.43	389084	21	3558	3106
miR399i	LOC_Os11g05730.1	146	-20.66	146	-20.66	389130	21	622	592
miR399i	LOC_Os11g05840.1	163	-27.61	163	-27.61	389141	21	7244	4046
miR399i	LOC_Os11g06610.1	141	-20.02	141	-20.02	389237	21	660	418
miR399i	LOC_Os11g06870.1	146	-21.97	146	-21.97	389266	21	1734	1711
miR399i	LOC_Os11g07730.1	161	-21.95	161	-21.95	389375	21	4815	3497
miR399i	LOC_Os11g07910.1	158	-20.16	158	-20.16	389394	21	2460	1779
miR399i	LOC_Os11g08090.1	158	-28.92	158	-28.92	389415	21	6768	1826
miR399i	LOC_Os11g08370.1	147	-21.2	147	-21.2	389449	21	2096	1673
miR399i	LOC_Os11g10760.1	151	-21.51	151	-21.51	389699	21	3141	729
miR399i	LOC_Os11g11040.1	143	-20.07	143	-20.07	389729	21	501	207
miR399i	LOC_Os11g11262.1	152	-23.45	152	-23.45	389751	21	681	144
miR399i	LOC_Os11g11400.1	157	-28.4	157	-28.4	389765	21	390	105
miR399i	LOC_Os11g11580.1	157	-21.13	157	-21.13	389786	21	4468	1520
miR399i	LOC_Os11g12010.1	168	-25.33	168	-25.33	389827	21	1781	1275
miR399i	LOC_Os11g12100.1	310	-41.9	161	-21.73	389836	21	4941	3085 3136
miR399i	LOC_Os11g12120.1	151	-22.59	151	-22.59	389838	21	3297	1488
miR399i	LOC_Os11g13420.1	159	-21.05	159	-21.05	389920	21	1267	975
miR399i	LOC_Os11g13420.2	159	-21.05	159	-21.05	389921	21	770	471
miR399i	LOC_Os11g13420.3	159	-21.05	159	-21.05	389922	21	1056	975
miR399i	LOC_Os11g14270.1	149	-20.89	149	-20.89	390012	21	3153	2110
miR399i	LOC_Os11g14380.1	166	-24.21	166	-24.21	390023	21	3038	1195
miR399i	LOC_Os11g15010.1	161	-23.18	161	-23.18	390099	21	1838	310
miR399i	LOC_Os11g15070.1	155	-22.79	155	-22.79	390109	21	2052	1207
miR399i	LOC_Os11g15400.1	164	-22.17	164	-22.17	390138	21	3513	2409

miR399i	LOC_Os11g15750.1	159	-21.36	159	-21.36	390175	21	3072	2567
miR399i	LOC_Os11g16750.1	160	-22.29	160	-22.29	390243	21	2265	640
miR399i	LOC_Os11g17040.1	167	-30.47	167	-30.47	390268	21	354	8
miR399i	LOC_Os11g17650.1	153	-23.3	153	-23.3	390329	21	564	348
miR399i	LOC_Os11g17780.1	162	-20.56	162	-20.56	390340	21	1662	764
miR399i	LOC_Os11g18710.1	154	-23.9	154	-23.9	390420	21	1944	1802
miR399i	LOC_Os11g18720.1	155	-23.02	155	-23.02	390421	21	2391	600
miR399i	LOC_Os11g19350.1	166	-25.16	166	-25.16	390489	21	2732	223
miR399i	LOC_Os11g19590.1	140	-20.83	140	-20.83	390516	21	1761	1076
miR399i	LOC_Os11g19710.1	142	-21.25	142	-21.25	390528	21	861	12
miR399i	LOC_Os11g19810.1	159	-20.47	159	-20.47	390540	21	3438	2597
miR399i	LOC_Os11g19810.2	159	-20.47	159	-20.47	390541	21	3642	2603
miR399i	LOC_Os11g21840.1	159	-25.55	159	-25.55	390635	21	2034	1804
miR399i	LOC_Os11g21850.1	153	-21.35	153	-21.35	390641	21	3846	2692
miR399i	LOC_Os11g22040.1	152	-23.4	152	-23.4	390661	21	942	600
miR399i	LOC_Os11g22220.1	157	-21.07	157	-21.07	390682	21	3330	458
miR399i	LOC_Os11g22240.1	159	-23.64	159	-23.64	390684	21	3822	294
miR399i	LOC_Os11g22404.1	169	-23.52	169	-23.52	390702	21	2159	1058
miR399i	LOC_Os11g22600.1	144	-24.45	144	-24.45	390720	21	1776	1696
miR399i	LOC_Os11g22740.1	314	-42.22	161	-21.35	390733	21	1944	649 700
miR399i	LOC_Os11g22750.1	156	-22.81	156	-22.81	390734	21	2142	751
miR399i	LOC_Os11g22930.1	157	-23.57	157	-23.57	390752	21	2664	438
miR399i	LOC_Os11g22960.1	149	-26.56	149	-26.56	390755	21	1159	259
miR399i	LOC_Os11g23100.1	161	-22.83	161	-22.83	390769	21	5196	3685
miR399i	LOC_Os11g23170.1	157	-21.2	157	-21.2	390776	21	1411	477
miR399i	LOC_Os11g23170.2	157	-21.2	157	-21.2	390777	21	1430	477
miR399i	LOC_Os11g23170.3	157	-21.2	157	-21.2	390778	21	1503	477
miR399i	LOC_Os11g23820.1	161	-23.72	161	-23.72	390803	21	3147	1494
miR399i	LOC_Os11g24090.1	164	-27	164	-27	390834	21	1671	76
miR399i	LOC_Os11g26130.1	150	-21.33	150	-21.33	391022	21	3392	1137
miR399i	LOC_Os11g26670.1	154	-22.47	154	-22.47	391072	21	4704	3173
miR399i	LOC_Os11g27620.1	150	-20.41	150	-20.41	391165	21	483	325

miR399i	LOC_Os11g27830.1	146	-20.88	146	-20.88	391187	21	1848	820
miR399i	LOC_Os11g27920.1	157	-24.89	157	-24.89	391196	21	1800	501
miR399i	LOC_Os11g27940.1	154	-21.76	154	-21.76	391198	21	1026	850
miR399i	LOC_Os11g28230.1	160	-21.87	160	-21.87	391222	21	2982	78
miR399i	LOC_Os11g28550.1	163	-27.68	163	-27.68	391261	21	1641	237
miR399i	LOC_Os11g28700.1	153	-20.56	153	-20.56	391275	21	5739	3955
miR399i	LOC_Os11g28730.1	153	-20.56	153	-20.56	391278	21	5286	3490
miR399i	LOC_Os11g28840.1	157	-20.97	157	-20.97	391288	21	831	451
miR399i	LOC_Os11g29200.1	162	-20.61	162	-20.61	391325	21	2934	2168
miR399i	LOC_Os11g29240.1	146	-22.2	146	-22.2	391330	21	3404	278
miR399i	LOC_Os11g29700.1	170	-24.81	170	-24.81	391375	21	848	440
miR399i	LOC_Os11g29700.2	170	-24.81	170	-24.81	391376	21	797	440
miR399i	LOC_Os11g29850.1	156	-21.17	156	-21.17	391392	21	1559	777
miR399i	LOC_Os11g29850.2	156	-21.17	156	-21.17	391393	21	1762	777
miR399i	LOC_Os11g31180.1	147	-20.67	147	-20.67	391515	21	2568	536
miR399i	LOC_Os11g33240.1	170	-26.01	170	-26.01	391743	21	2233	682
miR399i	LOC_Os11g33240.2	170	-26.01	170	-26.01	391744	21	2219	668
miR399i	LOC_Os11g33240.3	170	-26.01	170	-26.01	391745	21	2162	611
miR399i	LOC_Os11g35090.1	152	-22.23	152	-22.23	391900	21	2410	708
miR399i	LOC_Os11g35170.1	155	-24.66	155	-24.66	391907	21	1257	941
miR399i	LOC_Os11g35274.1	152	-22.46	152	-22.46	391916	21	2429	714
miR399i	LOC_Os11g35700.1	157	-20.13	157	-20.13	391962	21	4029	2213
miR399i	LOC_Os11g35780.1	156	-24.74	156	-24.74	391971	21	390	83
miR399i	LOC_Os11g35930.1	157	-25.97	157	-25.97	391989	21	1427	1195
miR399i	LOC_Os11g36970.1	147	-20.28	147	-20.28	392100	21	2493	2397
miR399i	LOC_Os11g37090.1	152	-23.87	152	-23.87	392112	21	3185	2680
miR399i	LOC_Os11g38020.1	150	-20.38	150	-20.38	392210	21	1970	800
miR399i	LOC_Os11g38530.1	152	-24.22	152	-24.22	392262	21	2649	135
miR399i	LOC_Os11g38900.1	158	-25.78	158	-25.78	392301	21	3318	2810
miR399i	LOC_Os11g39250.1	149	-20.5	149	-20.5	392347	21	3042	482
miR399i	LOC_Os11g39320.1	159	-21.01	159	-21.01	392355	21	3408	1318
miR399i	LOC_Os11g39350.1	158	-24.34	158	-24.34	392357	21	2532	358

miR399i	LOC_Os11g39350.2	158	-24.34	158	-24.34	392358	21	2384	358
miR399i	LOC_Os11g40284.1	151	-23.65	151	-23.65	392455	21	3732	300
miR399i	LOC_Os11g40430.1	163	-27.42	163	-27.42	392472	21	1416	384
miR399i	LOC_Os11g40590.1	150	-23.16	150	-23.16	392494	21	3972	625
miR399i	LOC_Os11g41400.1	153	-26.75	153	-26.75	392575	21	651	480
miR399i	LOC_Os11g41410.1	146	-21.58	146	-21.58	392576	21	1480	491
miR399i	LOC_Os11g41760.1	153	-21.08	153	-21.08	392615	21	5985	4201
miR399i	LOC_Os11g42420.1	155	-22.77	155	-22.77	392685	21	3884	3738
miR399i	LOC_Os11g42420.2	155	-22.77	155	-22.77	392686	21	4240	4094
miR399i	LOC_Os11g42430.1	155	-22.77	155	-22.77	392687	21	1856	376
miR399i	LOC_Os11g43640.1	314	-47.51	157	-24.63	392819	21	2841	704 803
miR399i	LOC_Os11g43700.1	151	-21.61	151	-21.61	392825	21	3570	770
miR399i	LOC_Os11g44014.1	158	-20.47	158	-20.47	392858	21	4932	277
miR399i	LOC_Os11g44070.1	153	-20.56	153	-20.56	392863	21	2472	895
miR399i	LOC_Os11g44960.1	167	-26.07	167	-26.07	392955	21	3270	1882
miR399i	LOC_Os11g44990.1	160	-21.29	160	-21.29	392958	21	2931	2186
miR399i	LOC_Os11g45050.1	167	-26.07	167	-26.07	392963	21	3102	1879
miR399i	LOC_Os11g45130.1	160	-24.25	160	-24.25	392971	21	3303	1867
miR399i	LOC_Os11g45180.1	160	-22.93	160	-22.93	392976	21	3096	1879
miR399i	LOC_Os11g45240.1	153	-21.08	153	-21.08	392982	21	6870	4267
miR399i	LOC_Os11g45380.1	152	-22.17	152	-22.17	392997	21	1662	928
miR399i	LOC_Os11g45510.1	148	-20.42	148	-20.42	393010	21	1971	98
miR399i	LOC_Os11g45620.1	155	-24.71	155	-24.71	393023	21	2838	1643
miR399i	LOC_Os11g46000.2	162	-20.93	162	-20.93	393064	21	2376	300
miR399i	LOC_Os11g46120.1	153	-23.97	153	-23.97	393079	21	2870	2392
miR399i	LOC_Os11g47300.1	142	-21.63	142	-21.63	393148	21	3324	2819
miR399i	LOC_Os11g47910.1	155	-21.65	155	-21.65	393238	21	1788	722
miR399i	LOC_Os11g47920.1	155	-21.65	155	-21.65	393239	21	1782	716
miR399i	LOC_Os12g01360.1	165	-25.09	165	-25.09	393307	21	3908	2302
miR399i	LOC_Os12g02710.1	145	-20.11	145	-20.11	393480	21	2603	877
miR399i	LOC_Os12g02770.1	157	-22.56	157	-22.56	393488	21	648	565
miR399i	LOC_Os12g02810.1	156	-26.62	156	-26.62	393494	21	1780	963

miR399i	LOC_Os12g03080.1	166	-20.95	166	-20.95	393528	21	6121	5061
miR399i	LOC_Os12g03680.1	151	-22.42	151	-22.42	393591	21	2499	958
miR399i	LOC_Os12g04000.1	155	-22.36	155	-22.36	393634	21	1530	955
miR399i	LOC_Os12g04770.1	147	-22.88	147	-22.88	393727	21	2251	263
miR399i	LOC_Os12g04990.1	144	-20.34	144	-20.34	393753	21	2652	535
miR399i	LOC_Os12g04990.2	144	-20.34	144	-20.34	393754	21	2647	530
miR399i	LOC_Os12g04990.3	144	-20.34	144	-20.34	393755	21	2580	535
miR399i	LOC_Os12g05050.1	157	-20.01	157	-20.01	393766	21	1210	372
miR399i	LOC_Os12g05420.1	155	-21.43	155	-21.43	393804	21	1881	1222
miR399i	LOC_Os12g05830.1	171	-29.77	171	-29.77	393850	21	1691	1011
miR399i	LOC_Os12g06400.1	168	-26.04	168	-26.04	393908	21	279	126
miR399i	LOC_Os12g06670.1	149	-20.23	149	-20.23	393941	21	2136	283
miR399i	LOC_Os12g06680.1	158	-20.06	158	-20.06	393942	21	3540	3016
miR399i	LOC_Os12g06870.1	306	-41.33	154	-21.27	393968	21	3466	743 894
miR399i	LOC_Os12g06890.1	145	-21.18	145	-21.18	393969	21	3370	494
miR399i	LOC_Os12g06910.1	157	-22.51	157	-22.51	393971	21	3411	1778
miR399i	LOC_Os12g06910.2	157	-22.51	157	-22.51	393972	21	3511	1778
miR399i	LOC_Os12g07530.1	142	-23.67	142	-23.67	394038	21	5389	850
miR399i	LOC_Os12g07670.1	175	-30.29	175	-30.29	394053	21	2430	1486
miR399i	LOC_Os12g07860.1	161	-23.73	161	-23.73	394073	21	723	40
miR399i	LOC_Os12g08170.1	162	-30.37	162	-30.37	394111	21	1845	1027
miR399i	LOC_Os12g08410.1	157	-21.58	157	-21.58	394140	21	1455	37
miR399i	LOC_Os12g08940.1	160	-25.53	160	-25.53	394195	21	1344	698
miR399i	LOC_Os12g09130.1	158	-20.18	158	-20.18	394213	21	816	43
miR399i	LOC_Os12g09190.1	158	-20.18	158	-20.18	394218	21	2898	416
miR399i	LOC_Os12g09580.1	162	-20.84	162	-20.84	394262	21	2181	674
miR399i	LOC_Os12g09600.1	146	-21.72	146	-21.72	394265	21	459	53
miR399i	LOC_Os12g10270.1	145	-20.14	145	-20.14	394330	21	1737	1599
miR399i	LOC_Os12g10410.1	168	-30.55	168	-30.55	394344	21	3259	2782
miR399i	LOC_Os12g10440.1	157	-23.28	157	-23.28	394347	21	2598	1487
miR399i	LOC_Os12g10600.1	157	-23.26	157	-23.26	394369	21	768	12
miR399i	LOC_Os12g11660.1	159	-23.87	159	-23.87	394475	21	725	266

miR399i	LOC_Os12g12800.1	164	-21.73	164	-21.73	394591	21	2400	861
miR399i	LOC_Os12g13150.1	161	-28.67	161	-28.67	394630	21	7573	3989
miR399i	LOC_Os12g14610.1	161	-20.71	161	-20.71	394785	21	1458	1064
miR399i	LOC_Os12g16450.1	140	-21.65	140	-21.65	394969	21	4008	2634
miR399i	LOC_Os12g16570.1	156	-23.92	156	-23.92	394983	21	309	17
miR399i	LOC_Os12g16840.1	318	-41.33	161	-20.97	395010	21	4200	2734 2785
miR399i	LOC_Os12g17170.1	152	-21.32	152	-21.32	395044	21	1884	1784
miR399i	LOC_Os12g17420.1	146	-22.21	146	-22.21	395068	21	1545	293
miR399i	LOC_Os12g17690.1	162	-24.56	162	-24.56	395097	21	3201	1766
miR399i	LOC_Os12g18050.1	155	-24.66	155	-24.66	395133	21	1326	944
miR399i	LOC_Os12g18290.1	155	-24.66	155	-24.66	395163	21	969	317
miR399i	LOC_Os12g19470.1	160	-26.41	160	-26.41	395279	21	2122	158
miR399i	LOC_Os12g19470.2	160	-26.41	160	-26.41	395280	21	2178	296
miR399i	LOC_Os12g20010.1	159	-23.36	159	-23.36	395334	21	477	147
miR399i	LOC_Os12g20210.1	161	-21.95	161	-21.95	395354	21	4140	2879
miR399i	LOC_Os12g20410.1	140	-21.24	140	-21.24	395379	21	1551	327
miR399i	LOC_Os12g20410.2	140	-21.24	140	-21.24	395380	21	1580	371
miR399i	LOC_Os12g20410.3	140	-21.24	140	-21.24	395381	21	1624	327
miR399i	LOC_Os12g21780.1	161	-20.87	161	-20.87	395417	21	4626	3475
miR399i	LOC_Os12g22240.1	165	-25.58	165	-25.58	395469	21	1119	871
miR399i	LOC_Os12g23100.1	154	-20.43	154	-20.43	395551	21	1941	417
miR399i	LOC_Os12g23230.1	153	-20.62	153	-20.62	395565	21	6033	4258
miR399i	LOC_Os12g23550.1	167	-24.01	167	-24.01	395596	21	1326	167
miR399i	LOC_Os12g23660.1	314	-41.71	157	-21.58	395607	21	3249	1403 1456
miR399i	LOC_Os12g23770.1	153	-20.18	153	-20.18	395618	21	2958	1991
miR399i	LOC_Os12g23869.1	164	-23.21	164	-23.21	395626	21	453	5
miR399i	LOC_Os12g23920.1	153	-20.62	153	-20.62	395630	21	5955	4180
miR399i	LOC_Os12g24450.1	166	-24.34	166	-24.34	395679	21	3144	361
miR399i	LOC_Os12g24510.1	153	-22.83	153	-22.83	395684	21	3213	2025
miR399i	LOC_Os12g25130.1	147	-20.1	147	-20.1	395743	21	608	130
miR399i	LOC_Os12g25590.1	155	-24.66	155	-24.66	395789	21	1260	857
miR399i	LOC_Os12g25600.1	155	-24.66	155	-24.66	395790	21	1437	911

miR399i	LOC_Os12g26830.1	140	-20.45	140	-20.45	395914	21	1855	1263
miR399i	LOC_Os12g26840.1	157	-21.13	157	-21.13	395915	21	1923	413
miR399i	LOC_Os12g26850.1	151	-20.57	151	-20.57	395916	21	3968	466
miR399i	LOC_Os12g27120.1	141	-21.38	141	-21.38	395948	21	2715	254
miR399i	LOC_Os12g27370.1	151	-24.3	151	-24.3	395972	21	2927	664
miR399i	LOC_Os12g27400.1	155	-25.69	155	-25.69	395974	21	1920	355
miR399i	LOC_Os12g27710.1	153	-20.56	153	-20.56	396005	21	5871	4075
miR399i	LOC_Os12g27910.1	159	-21.28	159	-21.28	396025	21	1989	390
miR399i	LOC_Os12g28150.1	156	-20.19	156	-20.19	396046	21	3816	2522
miR399i	LOC_Os12g28190.1	156	-20.19	156	-20.19	396049	21	5823	2522
miR399i	LOC_Os12g28290.1	318	-42.45	161	-21.58	396060	21	3951	2914 2965
miR399i	LOC_Os12g28470.1	156	-24.57	156	-24.57	396077	21	5478	2986
miR399i	LOC_Os12g28550.1	162	-21.41	162	-21.41	396084	21	1984	377
miR399i	LOC_Os12g29440.1	162	-22.74	162	-22.74	396169	21	615	266
miR399i	LOC_Os12g29530.1	149	-20.5	149	-20.5	396180	21	5167	2378
miR399i	LOC_Os12g29700.1	160	-21.61	160	-21.61	396200	21	4866	4206
miR399i	LOC_Os12g29980.1	154	-20.5	154	-20.5	396227	21	1859	1130
miR399i	LOC_Os12g29980.2	154	-20.5	154	-20.5	396228	21	1817	1088
miR399i	LOC_Os12g30040.1	154	-24.11	154	-24.11	396237	21	2147	817
miR399i	LOC_Os12g30070.1	310	-50.16	155	-25.08	396241	21	2208	384 930
miR399i	LOC_Os12g30670.1	151	-23.15	151	-23.15	396301	21	1602	1418
miR399i	LOC_Os12g30690.1	141	-20.7	141	-20.7	396303	21	2667	531
miR399i	LOC_Os12g30740.1	150	-20.15	150	-20.15	396307	21	240	72
miR399i	LOC_Os12g31000.1	149	-21.43	149	-21.43	396329	21	2010	321
miR399i	LOC_Os12g31190.1	153	-20.56	153	-20.56	396346	21	5955	4225
miR399i	LOC_Os12g31390.1	148	-21.09	148	-21.09	396364	21	591	410
miR399i	LOC_Os12g31560.1	156	-22.73	156	-22.73	396381	21	1569	751
miR399i	LOC_Os12g31570.1	156	-24.57	156	-24.57	396382	21	5328	2824
miR399i	LOC_Os12g31920.1	150	-20.13	150	-20.13	396425	21	4002	1265
miR399i	LOC_Os12g34094.1	158	-22.82	158	-22.82	396646	21	1368	1244
miR399i	LOC_Os12g34770.1	150	-20.13	150	-20.13	396722	21	4002	1265
miR399i	LOC_Os12g34890.1	161	-24.76	161	-24.76	396741	21	874	264

miR399i	LOC_Os12g34920.1	156	-20.23	156	-20.23	396744	21	1266	677
miR399i	LOC_Os12g34990.1	157	-22.12	157	-22.12	396751	21	3182	2589
miR399i	LOC_Os12g34990.2	157	-22.12	157	-22.12	396752	21	1207	614
miR399i	LOC_Os12g34990.3	157	-22.12	157	-22.12	396753	21	1872	1279
miR399i	LOC_Os12g34990.4	157	-22.12	157	-22.12	396754	21	1153	560
miR399i	LOC_Os12g35510.1	164	-28.04	164	-28.04	396809	21	387	257
miR399i	LOC_Os12g35790.1	150	-21.16	150	-21.16	396843	21	312	161
miR399i	LOC_Os12g35810.1	153	-20.95	153	-20.95	396844	21	2952	40
miR399i	LOC_Os12g36440.1	161	-24.85	161	-24.85	396907	21	2541	209
miR399i	LOC_Os12g36770.2	159	-21.53	159	-21.53	396943	21	2233	593
miR399i	LOC_Os12g37160.1	151	-22.37	151	-22.37	396983	21	2003	122
miR399i	LOC_Os12g37160.2	151	-22.37	151	-22.37	396984	21	1941	122
miR399i	LOC_Os12g37340.1	153	-20.56	153	-20.56	397003	21	5976	4192
miR399i	LOC_Os12g37400.1	156	-20.48	156	-20.48	397009	21	2679	1919
miR399i	LOC_Os12g37400.2	156	-20.48	156	-20.48	397010	21	2781	1912
miR399i	LOC_Os12g37890.1	159	-20.71	159	-20.71	397073	21	5573	1768
miR399i	LOC_Os12g39610.1	157	-21.13	157	-21.13	397276	21	5259	3674
miR399i	LOC_Os12g39870.1	162	-22.76	162	-22.76	397306	21	1407	1151
miR399i	LOC_Os12g39900.1	160	-21.42	160	-21.42	397309	21	1650	940
miR399i	LOC_Os12g40440.1	160	-23.49	160	-23.49	397369	21	2702	777
miR399i	LOC_Os12g41760.1	159	-23.1	159	-23.1	397525	21	522	290
miR399i	LOC_Os12g41810.1	161	-21.73	161	-21.73	397532	21	2390	954
miR399i	LOC_Os12g42140.1	148	-20.86	148	-20.86	397583	21	2869	1183
miR399i	LOC_Os12g42250.1	152	-21.61	152	-21.61	397594	21	1118	377
miR399i	LOC_Os12g42284.1	159	-21.36	159	-21.36	397599	21	807	93
miR399i	LOC_Os12g42430.1	160	-24.45	160	-24.45	397615	21	1867	855
miR399i	LOC_Os12g42430.2	160	-24.45	160	-24.45	397616	21	1869	855
miR399i	LOC_Os12g42430.3	160	-24.45	160	-24.45	397617	21	1849	855
miR399i	LOC_Os12g42900.1	455	-63.63	157	-21.5	397674	21	1493	733 577 670
miR399i	LOC_Os12g43060.1	157	-22.56	157	-22.56	397690	21	4884	2270
miR399i	LOC_Os12g43810.1	158	-25.47	158	-25.47	397765	21	705	469
miR399i	LOC_Os12g44120.1	149	-25.03	149	-25.03	397804	21	903	154

miR399i	LOC_Os12g44190.1	140	-21.31	140	-21.31	397814	21	2073	1274
miR399i	ChrUn.fgenesh.mRNA.12	163	-26.76	163	-26.76	397855	21	5379	2182
miR399i	ChrUn.fgenesh.mRNA.85	154	-21.32	154	-21.32	397928	21	3369	2829
miR399i	ChrSy.fgenesh.mRNA.22	157	-20.16	157	-20.16	397961	21	5973	566
miR399i	ChrSy.fgenesh.mRNA.52	159	-23.38	159	-23.38	397991	21	2985	1675
miR3979-3p	LOC_Os01g02040.1	164	-24.97	164	-24.97	398165	20	3085	1086
miR3979-3p	LOC_Os01g02040.2	164	-24.97	164	-24.97	398166	20	2608	609
miR3979-3p	LOC_Os01g02890.1	152	-20.7	152	-20.7	398261	20	2918	2328
miR3979-3p	LOC_Os01g03780.1	146	-22.27	146	-22.27	398359	20	5694	1333
miR3979-3p	LOC_Os01g03980.1	168	-31.77	168	-31.77	398379	20	1018	532
miR3979-3p	LOC_Os01g04090.1	142	-21.36	142	-21.36	398391	20	1685	541
miR3979-3p	LOC_Os01g04540.1	142	-20.93	142	-20.93	398440	20	2208	878
miR3979-3p	LOC_Os01g04850.1	145	-22.39	145	-22.39	398478	20	873	627
miR3979-3p	LOC_Os01g05060.1	154	-25.35	154	-25.35	398506	20	1178	947
miR3979-3p	LOC_Os01g07860.1	157	-27.76	157	-27.76	398828	20	2475	1074
miR3979-3p	LOC_Os01g08050.1	140	-20.94	140	-20.94	398849	20	453	9
miR3979-3p	LOC_Os01g08350.1	155	-21.01	155	-21.01	398882	20	351	295
miR3979-3p	LOC_Os01g08650.1	146	-23.3	146	-23.3	398914	20	1755	607
miR3979-3p	LOC_Os01g08650.2	146	-23.3	146	-23.3	398915	20	1725	607
miR3979-3p	LOC_Os01g09180.1	144	-22.42	144	-22.42	398980	20	156	8
miR3979-3p	LOC_Os01g09880.1	161	-24.05	161	-24.05	399062	20	1448	479
miR3979-3p	LOC_Os01g10050.1	157	-25.54	157	-25.54	399080	20	2110	1816
miR3979-3p	LOC_Os01g10050.2	157	-25.54	157	-25.54	399081	20	2225	1931
miR3979-3p	LOC_Os01g11054.1	141	-22.61	141	-22.61	399191	20	3535	2398
miR3979-3p	LOC_Os01g11054.2	141	-22.61	141	-22.61	399192	20	3007	1892
miR3979-3p	LOC_Os01g11054.3	141	-22.61	141	-22.61	399193	20	2397	1260
miR3979-3p	LOC_Os01g11054.4	141	-22.61	141	-22.61	399194	20	2177	1040
miR3979-3p	LOC_Os01g11350.1	152	-20.5	152	-20.5	399220	20	1483	1341
miR3979-3p	LOC_Os01g12310.1	151	-26.47	151	-26.47	399318	20	396	139
miR3979-3p	LOC_Os01g12440.1	162	-25.59	162	-25.59	399333	20	1833	96
miR3979-3p	LOC_Os01g12590.1	145	-23.09	145	-23.09	399354	20	6111	4793
miR3979-3p	LOC_Os01g13170.1	141	-20.34	141	-20.34	399435	20	1865	1222

miR3979-3p	LOC_Os01g13180.1	149	-20.6	149	-20.6	399436	20	1389	935
miR3979-3p	LOC_Os01g13420.1	156	-23.98	156	-23.98	399463	20	1042	463
miR3979-3p	LOC_Os01g13490.1	178	-32.82	178	-32.82	399470	20	1216	75
miR3979-3p	LOC_Os01g13620.1	145	-25.15	145	-25.15	399484	20	3438	187
miR3979-3p	LOC_Os01g15100.1	155	-20.48	155	-20.48	399648	20	525	368
miR3979-3p	LOC_Os01g15770.1	143	-21.91	143	-21.91	399721	20	1139	104
miR3979-3p	LOC_Os01g15880.1	151	-22.4	151	-22.4	399734	20	1551	376
miR3979-3p	LOC_Os01g15890.1	140	-20.92	140	-20.92	399735	20	2637	309
miR3979-3p	LOC_Os01g16060.1	152	-20.3	152	-20.3	399756	20	3105	2434
miR3979-3p	LOC_Os01g16370.1	152	-20.75	152	-20.75	399802	20	3454	3132
miR3979-3p	LOC_Os01g16370.2	152	-20.75	152	-20.75	399803	20	3286	2964
miR3979-3p	LOC_Os01g17370.1	145	-23.03	145	-23.03	399909	20	2295	748
miR3979-3p	LOC_Os01g19130.1	170	-30.21	170	-30.21	400044	20	1676	1387
miR3979-3p	LOC_Os01g19160.1	146	-22.23	146	-22.23	400047	20	1475	533
miR3979-3p	LOC_Os01g19160.2	146	-22.23	146	-22.23	400048	20	1602	533
miR3979-3p	LOC_Os01g19190.1	150	-22.75	150	-22.75	400055	20	5553	994
miR3979-3p	LOC_Os01g19840.1	158	-23.47	158	-23.47	400124	20	551	466
miR3979-3p	LOC_Os01g21090.1	152	-26.66	152	-26.66	400210	20	1797	1480
miR3979-3p	LOC_Os01g21530.1	145	-21.44	145	-21.44	400255	20	2988	2132
miR3979-3p	LOC_Os01g22770.1	144	-22.42	144	-22.42	400374	20	318	8
miR3979-3p	LOC_Os01g24260.1	156	-27.84	156	-27.84	400525	20	882	793
miR3979-3p	LOC_Os01g24820.1	157	-25.82	157	-25.82	400587	20	2550	204
miR3979-3p	LOC_Os01g24840.1	156	-27.62	156	-27.62	400589	20	657	84
miR3979-3p	LOC_Os01g25386.1	147	-22.36	147	-22.36	400650	20	5280	2726
miR3979-3p	LOC_Os01g25990.2	146	-23.12	146	-23.12	400707	20	2745	2358
miR3979-3p	LOC_Os01g25990.3	146	-23.12	146	-23.12	400708	20	2840	2453
miR3979-3p	LOC_Os01g26330.1	148	-21.13	148	-21.13	400739	20	525	450
miR3979-3p	LOC_Os01g27790.1	150	-22.75	150	-22.75	400840	20	5181	1363
miR3979-3p	LOC_Os01g27960.1	146	-20.1	146	-20.1	400855	20	3036	1284
miR3979-3p	LOC_Os01g29040.1	145	-25.15	145	-25.15	400973	20	804	425
miR3979-3p	LOC_Os01g32400.1	145	-25.15	145	-25.15	401214	20	1596	251
miR3979-3p	LOC_Os01g32574.1	145	-21.45	145	-21.45	401231	20	1422	1228

miR3979-3p	LOC_Os01g34030.1	146	-29.22	146	-29.22	401376	20	2607	866
miR3979-3p	LOC_Os01g34050.1	150	-25.48	150	-25.48	401378	20	1196	815
miR3979-3p	LOC_Os01g34920.1	160	-22.11	160	-22.11	401472	20	2864	132
miR3979-3p	LOC_Os01g35770.1	141	-21.74	141	-21.74	401562	20	3972	3925
miR3979-3p	LOC_Os01g36920.2	160	-23.25	160	-23.25	401682	20	1977	27
miR3979-3p	LOC_Os01g37070.1	157	-28.72	157	-28.72	401698	20	6276	4013
miR3979-3p	LOC_Os01g37740.1	165	-25.61	165	-25.61	401772	20	615	338
miR3979-3p	LOC_Os01g37820.1	146	-23.49	146	-23.49	401781	20	1746	1380
miR3979-3p	LOC_Os01g37860.1	152	-20.85	152	-20.85	401792	20	4020	3966
miR3979-3p	LOC_Os01g38380.1	154	-20.91	154	-20.91	401848	20	1194	892
miR3979-3p	LOC_Os01g40450.1	147	-22.42	147	-22.42	402073	20	2051	1773
miR3979-3p	LOC_Os01g40800.1	142	-21.24	142	-21.24	402114	20	2787	759
miR3979-3p	LOC_Os01g41280.1	161	-26.27	161	-26.27	402166	20	1512	29
miR3979-3p	LOC_Os01g42070.1	158	-24.63	158	-24.63	402248	20	2610	1041
miR3979-3p	LOC_Os01g42970.1	150	-24.11	150	-24.11	402352	20	1776	1679
miR3979-3p	LOC_Os01g42970.2	150	-24.11	150	-24.11	402353	20	1773	1676
miR3979-3p	LOC_Os01g43050.1	170	-30.22	170	-30.22	402364	20	2897	94
miR3979-3p	LOC_Os01g43820.1	152	-26.66	152	-26.66	402458	20	2877	1414
miR3979-3p	LOC_Os01g43920.1	151	-23.01	151	-23.01	402472	20	1035	997
miR3979-3p	LOC_Os01g44310.1	141	-26.15	141	-26.15	402516	20	4787	4487
miR3979-3p	LOC_Os01g44310.2	141	-26.15	141	-26.15	402517	20	4784	4484
miR3979-3p	LOC_Os01g45260.1	150	-20.24	150	-20.24	402561	20	468	375
miR3979-3p	LOC_Os01g46250.1	149	-20.33	149	-20.33	402668	20	1405	133
miR3979-3p	LOC_Os01g47850.1	145	-25.15	145	-25.15	402866	20	2178	668
miR3979-3p	LOC_Os01g47860.1	168	-33.07	168	-33.07	402867	20	2400	585
miR3979-3p	LOC_Os01g48330.1	153	-23.23	153	-23.23	402921	20	3973	3074
miR3979-3p	LOC_Os01g48500.1	171	-30.69	171	-30.69	402942	20	1085	89
miR3979-3p	LOC_Os01g49200.1	145	-22.43	145	-22.43	403024	20	2553	1869
miR3979-3p	LOC_Os01g50200.1	157	-28.11	157	-28.11	403135	20	1868	1192
miR3979-3p	LOC_Os01g50840.1	148	-22.6	148	-22.6	403204	20	1281	1227
miR3979-3p	LOC_Os01g51230.1	152	-25.18	152	-25.18	403252	20	1676	1555
miR3979-3p	LOC_Os01g51370.1	150	-20.73	150	-20.73	403269	20	892	512

miR3979-3p	LOC_Os01g51370.2	150	-20.73	150	-20.73	403270	20	1682	1301
miR3979-3p	LOC_Os01g51660.1	150	-20.39	150	-20.39	403303	20	516	471
miR3979-3p	LOC_Os01g51960.1	150	-33.97	150	-33.97	403339	20	303	221
miR3979-3p	LOC_Os01g52710.1	153	-23.01	153	-23.01	403427	20	2494	1672
miR3979-3p	LOC_Os01g52930.1	142	-21.24	142	-21.24	403454	20	2685	760
miR3979-3p	LOC_Os01g53060.1	149	-20.97	149	-20.97	403466	20	1288	355
miR3979-3p	LOC_Os01g53060.2	149	-20.97	149	-20.97	403467	20	1488	345
miR3979-3p	LOC_Os01g53720.1	140	-20.57	140	-20.57	403535	20	3493	455
miR3979-3p	LOC_Os01g53720.2	140	-20.57	140	-20.57	403536	20	3204	455
miR3979-3p	LOC_Os01g54190.1	161	-25.29	161	-25.29	403594	20	1607	163
miR3979-3p	LOC_Os01g54240.1	145	-20.28	145	-20.28	403598	20	2666	44
miR3979-3p	LOC_Os01g54250.1	154	-27.47	154	-27.47	403600	20	2490	1213
miR3979-3p	LOC_Os01g54540.1	158	-22.36	158	-22.36	403635	20	1087	28
miR3979-3p	LOC_Os01g54540.2	158	-22.36	158	-22.36	403636	20	1072	28
miR3979-3p	LOC_Os01g55040.1	167	-26.45	167	-26.45	403694	20	6528	5851
miR3979-3p	LOC_Os01g55549.1	148	-28.12	148	-28.12	403766	20	2451	153
miR3979-3p	LOC_Os01g55610.1	144	-20.41	144	-20.41	403776	20	2119	1577
miR3979-3p	LOC_Os01g55910.1	150	-22.75	150	-22.75	403813	20	4443	1333
miR3979-3p	LOC_Os01g55920.1	152	-26.66	152	-26.66	403814	20	2712	1444
miR3979-3p	LOC_Os01g56150.1	145	-22.64	145	-22.64	403841	20	1951	1268
miR3979-3p	LOC_Os01g56330.1	153	-22.29	153	-22.29	403866	20	3189	861
miR3979-3p	LOC_Os01g56630.2	155	-21.85	155	-21.85	403906	20	3513	1
miR3979-3p	LOC_Os01g56750.1	146	-20.22	146	-20.22	403924	20	1466	349
miR3979-3p	LOC_Os01g56980.1	149	-20.68	149	-20.68	403955	20	3528	3162
miR3979-3p	LOC_Os01g57082.1	151	-25.22	151	-25.22	403965	20	3125	1681
miR3979-3p	LOC_Os01g57690.1	156	-22.14	156	-22.14	404036	20	1180	413
miR3979-3p	LOC_Os01g58550.1	141	-20.99	141	-20.99	404140	20	2264	1307
miR3979-3p	LOC_Os01g58690.1	153	-23.82	153	-23.82	404155	20	1390	203
miR3979-3p	LOC_Os01g58690.2	153	-23.82	153	-23.82	404156	20	1387	191
miR3979-3p	LOC_Os01g58690.3	153	-23.82	153	-23.82	404157	20	1364	168
miR3979-3p	LOC_Os01g58740.1	146	-24.94	146	-24.94	404162	20	1989	1195
miR3979-3p	LOC_Os01g58740.2	146	-24.94	146	-24.94	404163	20	2188	1101

miR3979-3p	LOC_Os01g59980.1	150	-22.29	150	-22.29	404306	20	3246	418
miR3979-3p	LOC_Os01g60420.1	145	-23.2	145	-23.2	404356	20	1942	1149
miR3979-3p	LOC_Os01g60560.1	141	-23.54	141	-23.54	404369	20	1512	120
miR3979-3p	LOC_Os01g60820.1	160	-30.17	160	-30.17	404399	20	591	495
miR3979-3p	LOC_Os01g60890.1	151	-21.67	151	-21.67	404405	20	468	235
miR3979-3p	LOC_Os01g60910.2	162	-29.91	162	-29.91	404408	20	3096	2484
miR3979-3p	LOC_Os01g61440.1	148	-23.67	148	-23.67	404464	20	1489	757
miR3979-3p	LOC_Os01g61720.6	155	-20.57	155	-20.57	404510	20	2670	2437
miR3979-3p	LOC_Os01g62560.1	152	-20.34	152	-20.34	404627	20	1146	969
miR3979-3p	LOC_Os01g62570.1	156	-20.49	156	-20.49	404628	20	1905	228
miR3979-3p	LOC_Os01g63100.1	150	-22.75	150	-22.75	404691	20	5355	994
miR3979-3p	LOC_Os01g63110.1	150	-25.33	150	-25.33	404692	20	1581	1480
miR3979-3p	LOC_Os01g63160.1	173	-35.28	173	-35.28	404697	20	1394	1088
miR3979-3p	LOC_Os01g63220.1	312	-42.1	160	-21.76	404704	20	1872	134 1250
miR3979-3p	LOC_Os01g63220.2	312	-42.1	160	-21.76	404705	20	2358	134 1250
miR3979-3p	LOC_Os01g63220.3	312	-42.1	160	-21.76	404706	20	2125	134 1250
miR3979-3p	LOC_Os01g63780.1	140	-21.09	140	-21.09	404768	20	1319	133
miR3979-3p	LOC_Os01g63780.2	140	-21.09	140	-21.09	404769	20	1389	133
miR3979-3p	LOC_Os01g63854.1	147	-22.87	147	-22.87	404779	20	1807	1184
miR3979-3p	LOC_Os01g64120.1	161	-26.88	161	-26.88	404818	20	949	404
miR3979-3p	LOC_Os01g64540.1	151	-20.57	151	-20.57	404870	20	1607	210
miR3979-3p	LOC_Os01g65080.1	161	-24.01	161	-24.01	404944	20	2170	444
miR3979-3p	LOC_Os01g65080.2	161	-24.01	161	-24.01	404945	20	3915	2189
miR3979-3p	LOC_Os01g65080.3	161	-24.01	161	-24.01	404946	20	2384	658
miR3979-3p	LOC_Os01g65080.4	161	-24.01	161	-24.01	404947	20	2349	623
miR3979-3p	LOC_Os01g65760.1	140	-22.56	140	-22.56	405029	20	1894	1061
miR3979-3p	LOC_Os01g66330.1	144	-22.76	144	-22.76	405099	20	2349	495
miR3979-3p	LOC_Os01g66940.1	146	-22.95	146	-22.95	405163	20	1793	1517
miR3979-3p	LOC_Os01g67250.1	300	-44.65	155	-22.72	405200	20	4173	3917 3906
miR3979-3p	LOC_Os01g67250.2	300	-44.65	155	-22.72	405201	20	4331	4076 4065
miR3979-3p	LOC_Os01g67970.1	167	-28.06	167	-28.06	405293	20	4251	64
miR3979-3p	LOC_Os01g68610.1	149	-20.83	149	-20.83	405372	20	1782	892

miR3979-3p	LOC_Os01g68620.1	148	-23.08	148	-23.08	405373	20	2032	427
miR3979-3p	LOC_Os01g69930.1	141	-23.18	141	-23.18	405482	20	2854	1726
miR3979-3p	LOC_Os01g71106.2	141	-22.47	141	-22.47	405632	20	4440	3603
miR3979-3p	LOC_Os01g71400.1	163	-30	163	-30	405664	20	1069	504
miR3979-3p	LOC_Os01g71650.1	163	-30	163	-30	405697	20	693	267
miR3979-3p	LOC_Os01g72880.1	153	-29.54	153	-29.54	405835	20	2623	2215
miR3979-3p	LOC_Os01g73344.1	146	-20.1	146	-20.1	405888	20	5472	2571
miR3979-3p	LOC_Os01g73990.1	147	-22.04	147	-22.04	405968	20	968	471
miR3979-3p	LOC_Os01g74200.1	145	-21.52	145	-21.52	405992	20	1916	705
miR3979-3p	LOC_Os02g01970.1	157	-25.35	157	-25.35	406172	20	2547	141
miR3979-3p	LOC_Os02g02020.1	152	-20.06	152	-20.06	406177	20	2665	1773
miR3979-3p	LOC_Os02g02370.1	151	-22.28	151	-22.28	406217	20	894	142
miR3979-3p	LOC_Os02g02790.1	147	-29.75	147	-29.75	406274	20	2894	22
miR3979-3p	LOC_Os02g02880.1	140	-21.22	140	-21.22	406284	20	2131	2054
miR3979-3p	LOC_Os02g03090.1	152	-20.61	152	-20.61	406319	20	2636	2171
miR3979-3p	LOC_Os02g03090.2	152	-20.61	152	-20.61	406320	20	2618	2153
miR3979-3p	LOC_Os02g03260.1	313	-50.6	162	-29.23	406339	20	1932	623 1463
miR3979-3p	LOC_Os02g03260.2	313	-50.6	162	-29.23	406340	20	1729	623 1463
miR3979-3p	LOC_Os02g03260.3	313	-50.6	162	-29.23	406341	20	1968	623 1463
miR3979-3p	LOC_Os02g04610.1	169	-28.32	169	-28.32	406524	20	1607	360
miR3979-3p	LOC_Os02g04690.1	148	-23.81	148	-23.81	406534	20	2370	694
miR3979-3p	LOC_Os02g04690.2	148	-23.81	148	-23.81	406535	20	2532	856
miR3979-3p	LOC_Os02g04700.2	156	-26.65	156	-26.65	406537	20	2378	154
miR3979-3p	LOC_Os02g04840.1	145	-21.66	145	-21.66	406558	20	4103	3694
miR3979-3p	LOC_Os02g04900.1	158	-20.89	158	-20.89	406564	20	1278	907
miR3979-3p	LOC_Os02g05850.1	150	-22.75	150	-22.75	406688	20	4035	1018
miR3979-3p	LOC_Os02g07810.1	147	-25.59	147	-25.59	406937	20	2079	671
miR3979-3p	LOC_Os02g09120.1	156	-26.11	156	-26.11	407040	20	1092	30
miR3979-3p	LOC_Os02g09560.1	152	-25.01	152	-25.01	407086	20	2736	1273
miR3979-3p	LOC_Os02g10010.1	141	-20.97	141	-20.97	407143	20	1519	883
miR3979-3p	LOC_Os02g10010.2	141	-20.97	141	-20.97	407144	20	933	297
miR3979-3p	LOC_Os02g10380.1	146	-22.45	146	-22.45	407190	20	1337	1204

miR3979-3p	LOC_Os02g11665.1	148	-21.64	148	-21.64	407297	20	4228	433
miR3979-3p	LOC_Os02g11720.1	148	-23.04	148	-23.04	407303	20	1415	482
miR3979-3p	LOC_Os02g13110.1	152	-22.71	152	-22.71	407481	20	2340	2038
miR3979-3p	LOC_Os02g13110.2	152	-22.71	152	-22.71	407482	20	2268	1966
miR3979-3p	LOC_Os02g13400.1	147	-24.83	147	-24.83	407515	20	3060	498
miR3979-3p	LOC_Os02g13400.2	147	-24.83	147	-24.83	407516	20	3168	498
miR3979-3p	LOC_Os02g13510.1	155	-28.41	155	-28.41	407527	20	4059	42
miR3979-3p	LOC_Os02g14210.1	140	-20.07	140	-20.07	407608	20	1979	1448
miR3979-3p	LOC_Os02g15620.1	148	-22.47	148	-22.47	407770	20	444	296
miR3979-3p	LOC_Os02g16270.1	154	-25.92	154	-25.92	407846	20	4510	4086
miR3979-3p	LOC_Os02g16270.2	154	-25.92	154	-25.92	407847	20	4520	4086
miR3979-3p	LOC_Os02g16550.1	165	-27.01	165	-27.01	407877	20	1254	958
miR3979-3p	LOC_Os02g16740.1	153	-24.2	153	-24.2	407898	20	231	160
miR3979-3p	LOC_Os02g17360.1	152	-20.17	152	-20.17	407962	20	2732	2180
miR3979-3p	LOC_Os02g17360.2	152	-20.17	152	-20.17	407963	20	2992	2441
miR3979-3p	LOC_Os02g17720.1	151	-22.17	151	-22.17	408002	20	4497	2755
miR3979-3p	LOC_Os02g17770.1	156	-20.7	156	-20.7	408007	20	402	189
miR3979-3p	LOC_Os02g17960.1	147	-20.23	147	-20.23	408029	20	5343	2969
miR3979-3p	LOC_Os02g19270.1	162	-25.34	162	-25.34	408174	20	3708	155
miR3979-3p	LOC_Os02g19390.1	145	-25.08	145	-25.08	408187	20	1521	116
miR3979-3p	LOC_Os02g21070.1	147	-23.36	147	-23.36	408363	20	717	422
miR3979-3p	LOC_Os02g21590.1	149	-23.72	149	-23.72	408419	20	3999	3297
miR3979-3p	LOC_Os02g22210.1	157	-20.67	157	-20.67	408492	20	3396	2915
miR3979-3p	LOC_Os02g22810.1	141	-23.54	141	-23.54	408558	20	1368	209
miR3979-3p	LOC_Os02g26300.1	157	-27.52	157	-27.52	408841	20	2523	1
miR3979-3p	LOC_Os02g26370.2	148	-20.1	148	-20.1	408848	20	2098	1371
miR3979-3p	LOC_Os02g27240.1	152	-26.66	152	-26.66	408954	20	2475	1384
miR3979-3p	LOC_Os02g27950.1	161	-25.58	161	-25.58	409035	20	2610	936
miR3979-3p	LOC_Os02g27950.2	161	-25.58	161	-25.58	409036	20	2604	930
miR3979-3p	LOC_Os02g28160.1	149	-26.33	149	-26.33	409056	20	642	118
miR3979-3p	LOC_Os02g28330.1	145	-22.15	145	-22.15	409072	20	4398	2475
miR3979-3p	LOC_Os02g28680.1	154	-23.87	154	-23.87	409105	20	1269	280

miR3979-3p	LOC_Os02g28850.1	150	-23.09	150	-23.09	409120	20	3517	1695
miR3979-3p	LOC_Os02g30490.1	163	-29.99	163	-29.99	409300	20	819	357
miR3979-3p	LOC_Os02g31920.1	174	-30.82	174	-30.82	409408	20	691	478
miR3979-3p	LOC_Os02g32420.1	153	-23.67	153	-23.67	409465	20	1568	1406
miR3979-3p	LOC_Os02g33320.1	176	-32.91	176	-32.91	409571	20	3164	69
miR3979-3p	LOC_Os02g33320.2	176	-32.91	176	-32.91	409572	20	2427	62
miR3979-3p	LOC_Os02g33320.3	176	-32.91	176	-32.91	409573	20	2600	62
miR3979-3p	LOC_Os02g33840.1	160	-25.34	160	-25.34	409629	20	2297	1063
miR3979-3p	LOC_Os02g34900.1	150	-25.66	150	-25.66	409731	20	2649	219
miR3979-3p	LOC_Os02g35560.1	144	-21.63	144	-21.63	409806	20	3445	3034
miR3979-3p	LOC_Os02g35740.1	143	-23.18	143	-23.18	409829	20	693	209
miR3979-3p	LOC_Os02g36720.1	140	-22.26	140	-22.26	409947	20	840	697
miR3979-3p	LOC_Os02g37140.1	147	-22.02	147	-22.02	410008	20	3511	2626
miR3979-3p	LOC_Os02g37140.2	147	-22.02	147	-22.02	410009	20	3506	2621
miR3979-3p	LOC_Os02g37620.1	164	-29.07	164	-29.07	410058	20	2352	709
miR3979-3p	LOC_Os02g38140.1	145	-22.69	145	-22.69	410128	20	1728	1528
miR3979-3p	LOC_Os02g38140.2	145	-22.69	145	-22.69	410129	20	1960	1528
miR3979-3p	LOC_Os02g38160.1	145	-24.2	145	-24.2	410130	20	2089	1578
miR3979-3p	LOC_Os02g38160.2	145	-24.2	145	-24.2	410131	20	3337	1578
miR3979-3p	LOC_Os02g38880.1	303	-49.25	152	-25.01	410216	20	432	153 180
miR3979-3p	LOC_Os02g39710.1	164	-21.94	164	-21.94	410327	20	1463	802
miR3979-3p	LOC_Os02g39780.1	154	-27.64	154	-27.64	410338	20	5106	3324
miR3979-3p	LOC_Os02g40610.1	152	-26.6	152	-26.6	410448	20	1140	1109
miR3979-3p	LOC_Os02g40664.1	148	-23.69	148	-23.69	410453	20	2965	767
miR3979-3p	LOC_Os02g41480.1	140	-20.95	140	-20.95	410492	20	2514	673
miR3979-3p	LOC_Os02g41954.1	150	-22.34	150	-22.34	410548	20	1557	441
miR3979-3p	LOC_Os02g42290.1	149	-23.38	149	-23.38	410584	20	1449	74
miR3979-3p	LOC_Os02g42290.2	149	-23.38	149	-23.38	410585	20	1384	74
miR3979-3p	LOC_Os02g42290.3	149	-23.38	149	-23.38	410586	20	1357	74
miR3979-3p	LOC_Os02g43590.1	144	-22.35	144	-22.35	410750	20	1936	1023
miR3979-3p	LOC_Os02g43750.1	155	-20.01	155	-20.01	410769	20	3456	1998
miR3979-3p	LOC_Os02g44370.1	161	-23.59	161	-23.59	410864	20	2986	1220

miR3979-3p	LOC_Os02g45110.1	154	-24.47	154	-24.47	410962	20	2549	278
miR3979-3p	LOC_Os02g45344.1	164	-25.66	164	-25.66	410991	20	1943	1043
miR3979-3p	LOC_Os02g46460.1	158	-21.06	158	-21.06	411123	20	2347	2005
miR3979-3p	LOC_Os02g46460.2	158	-21.06	158	-21.06	411124	20	2545	2005
miR3979-3p	LOC_Os02g46480.1	156	-22.82	156	-22.82	411126	20	710	115
miR3979-3p	LOC_Os02g46600.1	141	-21.64	141	-21.64	411137	20	2936	1000
miR3979-3p	LOC_Os02g47140.1	153	-29.27	153	-29.27	411205	20	984	207
miR3979-3p	LOC_Os02g47140.2	153	-29.27	153	-29.27	411206	20	980	207
miR3979-3p	LOC_Os02g47230.1	160	-24.54	160	-24.54	411218	20	2130	2097
miR3979-3p	LOC_Os02g47230.2	160	-24.54	160	-24.54	411219	20	2274	2241
miR3979-3p	LOC_Os02g47680.1	294	-47.3	150	-25.76	411281	20	570	145 235
miR3979-3p	LOC_Os02g47690.1	146	-20.1	146	-20.1	411282	20	4879	2320
miR3979-3p	LOC_Os02g47730.1	143	-20.08	143	-20.08	411286	20	6282	6008
miR3979-3p	LOC_Os02g47790.1	141	-21.45	141	-21.45	411294	20	1626	507
miR3979-3p	LOC_Os02g48060.1	158	-26.14	158	-26.14	411327	20	1397	606
miR3979-3p	LOC_Os02g48610.1	162	-27.08	162	-27.08	411400	20	1057	66
miR3979-3p	LOC_Os02g48610.2	162	-27.08	162	-27.08	411401	20	919	66
miR3979-3p	LOC_Os02g49270.1	146	-22.94	146	-22.94	411482	20	2556	133
miR3979-3p	LOC_Os02g49570.1	147	-23.78	147	-23.78	411519	20	2478	1947
miR3979-3p	LOC_Os02g49940.1	151	-21.12	151	-21.12	411571	20	738	192
miR3979-3p	LOC_Os02g49970.1	153	-21.5	153	-21.5	411574	20	2059	1764
miR3979-3p	LOC_Os02g50230.1	154	-20.87	154	-20.87	411601	20	1976	52
miR3979-3p	LOC_Os02g50530.1	157	-20.67	157	-20.67	411637	20	2460	1838
miR3979-3p	LOC_Os02g51030.1	148	-23.37	148	-23.37	411699	20	1067	293
miR3979-3p	LOC_Os02g51130.1	149	-21.74	149	-21.74	411710	20	1923	185
miR3979-3p	LOC_Os02g51200.1	164	-25.34	164	-25.34	411720	20	5130	827
miR3979-3p	LOC_Os02g52250.1	150	-24.33	150	-24.33	411858	20	2480	2121
miR3979-3p	LOC_Os02g52490.1	156	-24.7	156	-24.7	411889	20	1680	1420
miR3979-3p	LOC_Os02g52490.2	156	-24.7	156	-24.7	411890	20	1790	1530
miR3979-3p	LOC_Os02g52520.1	297	-51.53	152	-28.44	411893	20	5820	1224 4502
miR3979-3p	LOC_Os02g52700.1	149	-24.15	149	-24.15	411910	20	1631	1279
miR3979-3p	LOC_Os02g52730.1	148	-23.89	148	-23.89	411913	20	2213	235

miR3979-3p	LOC_Os02g52744.1	176	-33.3	176	-33.3	411914	20	1127	670
miR3979-3p	LOC_Os02g52744.2	176	-33.3	176	-33.3	411915	20	1123	666
miR3979-3p	LOC_Os02g53300.1	142	-24.27	142	-24.27	411991	20	414	197
miR3979-3p	LOC_Os02g53750.1	140	-21.04	140	-21.04	412051	20	1884	932
miR3979-3p	LOC_Os02g53750.2	140	-21.04	140	-21.04	412052	20	3121	932
miR3979-3p	LOC_Os02g54160.1	145	-25.19	145	-25.19	412105	20	1703	1397
miR3979-3p	LOC_Os02g54160.2	145	-25.19	145	-25.19	412106	20	1778	1472
miR3979-3p	LOC_Os02g54510.1	142	-24	142	-24	412143	20	4517	238
miR3979-3p	LOC_Os02g54690.1	145	-21.75	145	-21.75	412161	20	870	566
miR3979-3p	LOC_Os02g54890.1	161	-24.16	161	-24.16	412185	20	2090	276
miR3979-3p	LOC_Os02g56060.1	150	-22.75	150	-22.75	412333	20	5562	1333
miR3979-3p	LOC_Os02g56310.1	154	-23.09	154	-23.09	412362	20	1324	722
miR3979-3p	LOC_Os02g56360.1	151	-21.87	151	-21.87	412368	20	1607	1146
miR3979-3p	LOC_Os02g56565.1	148	-23.93	148	-23.93	412394	20	2238	127
miR3979-3p	LOC_Os02g57770.1	143	-22.06	143	-22.06	412571	20	1227	850
miR3979-3p	LOC_Os03g01060.1	152	-25.61	152	-25.61	412717	20	3297	1681
miR3979-3p	LOC_Os03g01390.1	152	-26.04	152	-26.04	412765	20	795	151
miR3979-3p	LOC_Os03g01770.1	143	-21.96	143	-21.96	412816	20	729	492
miR3979-3p	LOC_Os03g02640.1	154	-31.42	154	-31.42	412937	20	1338	161
miR3979-3p	LOC_Os03g04120.1	162	-21.78	162	-21.78	413130	20	2154	197
miR3979-3p	LOC_Os03g04390.1	149	-24.5	149	-24.5	413157	20	3547	1019
miR3979-3p	LOC_Os03g04380.1	149	-24.5	149	-24.5	413158	20	2081	1019
miR3979-3p	LOC_Os03g04520.2	161	-24.98	161	-24.98	413174	20	3629	3372
miR3979-3p	LOC_Os03g04520.3	161	-24.98	161	-24.98	413175	20	2464	2207
miR3979-3p	LOC_Os03g04520.4	161	-24.98	161	-24.98	413176	20	3573	3316
miR3979-3p	LOC_Os03g04550.1	152	-25.33	152	-25.33	413180	20	1715	202
miR3979-3p	LOC_Os03g05110.1	149	-20.1	149	-20.1	413247	20	2581	559
miR3979-3p	LOC_Os03g05700.1	168	-33.31	168	-33.31	413328	20	602	523
miR3979-3p	LOC_Os03g05940.1	148	-21.76	148	-21.76	413361	20	399	1
miR3979-3p	LOC_Os03g06270.1	152	-26.66	152	-26.66	413397	20	2715	1384
miR3979-3p	LOC_Os03g07050.1	152	-21.37	152	-21.37	413490	20	3654	3240
miR3979-3p	LOC_Os03g07250.1	157	-26.87	157	-26.87	413523	20	2037	1070

miR3979-3p	LOC_Os03g08370.1	152	-20.47	152	-20.47	413664	20	3109	87
miR3979-3p	LOC_Os03g09230.1	145	-23.03	145	-23.03	413772	20	1157	338
miR3979-3p	LOC_Os03g09250.1	153	-20.72	153	-20.72	413774	20	2208	1452
miR3979-3p	LOC_Os03g09250.2	153	-20.72	153	-20.72	413775	20	2228	1472
miR3979-3p	LOC_Os03g09250.3	153	-20.72	153	-20.72	413776	20	2318	1562
miR3979-3p	LOC_Os03g09250.4	153	-20.72	153	-20.72	413777	20	2411	1655
miR3979-3p	LOC_Os03g09960.1	151	-21.57	151	-21.57	413807	20	624	191
miR3979-3p	LOC_Os03g10090.1	151	-22.36	151	-22.36	413825	20	2355	542
miR3979-3p	LOC_Os03g10100.1	147	-21.73	147	-21.73	413826	20	2256	819
miR3979-3p	LOC_Os03g10640.1	148	-23.61	148	-23.61	413891	20	3878	1611
miR3979-3p	LOC_Os03g11420.1	158	-24.81	158	-24.81	413986	20	2076	1323
miR3979-3p	LOC_Os03g12230.1	154	-21.44	154	-21.44	414090	20	1202	155
miR3979-3p	LOC_Os03g12230.2	154	-21.44	154	-21.44	414091	20	1366	319
miR3979-3p	LOC_Os03g12820.1	166	-27.96	166	-27.96	414168	20	1903	1330
miR3979-3p	LOC_Os03g12820.2	166	-27.96	166	-27.96	414169	20	1557	1330
miR3979-3p	LOC_Os03g13300.1	157	-21.98	157	-21.98	414241	20	2149	27
miR3979-3p	LOC_Os03g13680.1	154	-27.64	154	-27.64	414279	20	2109	702
miR3979-3p	LOC_Os03g13910.1	152	-25.01	152	-25.01	414311	20	2826	1282
miR3979-3p	LOC_Os03g16170.1	140	-20.73	140	-20.73	414612	20	1719	1253
miR3979-3p	LOC_Os03g16170.2	140	-20.73	140	-20.73	414613	20	1872	1406
miR3979-3p	LOC_Os03g16310.1	144	-22.42	144	-22.42	414633	20	2613	8
miR3979-3p	LOC_Os03g16480.1	168	-27.17	168	-27.17	414651	20	1486	33
miR3979-3p	LOC_Os03g17000.2	140	-20.45	140	-20.45	414717	20	2667	1796
miR3979-3p	LOC_Os03g17000.3	140	-20.45	140	-20.45	414718	20	2490	1619
miR3979-3p	LOC_Os03g17000.4	140	-20.45	140	-20.45	414719	20	2762	1796
miR3979-3p	LOC_Os03g18840.1	140	-20.67	140	-20.67	414939	20	2271	2079
miR3979-3p	LOC_Os03g20949.2	150	-21.19	150	-21.19	415194	20	2688	682
miR3979-3p	LOC_Os03g21180.1	156	-30.09	156	-30.09	415220	20	5781	3944
miR3979-3p	LOC_Os03g21419.1	163	-24.34	163	-24.34	415245	20	3274	2888
miR3979-3p	LOC_Os03g22450.1	155	-22.31	155	-22.31	415380	20	1663	826
miR3979-3p	LOC_Os03g22550.1	164	-27.56	164	-27.56	415393	20	1215	23
miR3979-3p	LOC_Os03g22990.1	150	-21.38	150	-21.38	415449	20	2051	919

miR3979-3p	LOC_Os03g24590.1	152	-23.1	152	-23.1	415558	20	1202	43
miR3979-3p	LOC_Os03g24610.1	153	-27.04	153	-27.04	415560	20	1572	431
miR3979-3p	LOC_Os03g25480.1	149	-22.36	149	-22.36	415660	20	1557	290
miR3979-3p	LOC_Os03g26430.1	140	-20.73	140	-20.73	415749	20	1381	1030
miR3979-3p	LOC_Os03g26860.1	158	-22.5	158	-22.5	415790	20	1657	696
miR3979-3p	LOC_Os03g27280.1	144	-24.27	144	-24.27	415837	20	1720	1406
miR3979-3p	LOC_Os03g30092.1	154	-28.05	154	-28.05	416090	20	631	477
miR3979-3p	LOC_Os03g30420.1	158	-23.14	158	-23.14	416123	20	1771	758
miR3979-3p	LOC_Os03g30510.1	146	-24.01	146	-24.01	416131	20	2442	1362
miR3979-3p	LOC_Os03g30710.1	146	-20.1	146	-20.1	416153	20	5283	2382
miR3979-3p	LOC_Os03g30810.1	155	-26.52	155	-26.52	416164	20	3411	1552
miR3979-3p	LOC_Os03g31570.1	154	-26.32	154	-26.32	416252	20	1912	100
miR3979-3p	LOC_Os03g32380.1	146	-20.1	146	-20.1	416320	20	2805	126
miR3979-3p	LOC_Os03g32930.1	159	-20.78	159	-20.78	416374	20	2139	1831
miR3979-3p	LOC_Os03g33530.1	154	-27.64	154	-27.64	416431	20	6084	3909
miR3979-3p	LOC_Os03g33590.1	152	-20.78	152	-20.78	416437	20	1839	1633
miR3979-3p	LOC_Os03g33590.2	152	-20.78	152	-20.78	416438	20	1815	1609
miR3979-3p	LOC_Os03g33760.1	140	-22.57	140	-22.57	416454	20	4608	282
miR3979-3p	LOC_Os03g34096.1	157	-21.94	157	-21.94	416485	20	663	182
miR3979-3p	LOC_Os03g34170.1	148	-20.69	148	-20.69	416492	20	1167	122
miR3979-3p	LOC_Os03g34240.1	143	-22.57	143	-22.57	416499	20	2727	322
miR3979-3p	LOC_Os03g35710.1	144	-24.84	144	-24.84	416547	20	1521	1416
miR3979-3p	LOC_Os03g35788.1	154	-27.64	154	-27.64	416552	20	5724	3447
miR3979-3p	LOC_Os03g36030.1	146	-20.1	146	-20.1	416574	20	4386	1665
miR3979-3p	LOC_Os03g36200.1	143	-21.65	143	-21.65	416589	20	735	392
miR3979-3p	LOC_Os03g36320.1	151	-25.61	151	-25.61	416598	20	414	159
miR3979-3p	LOC_Os03g36660.1	150	-22.75	150	-22.75	416630	20	4626	229
miR3979-3p	LOC_Os03g36670.1	152	-26.66	152	-26.66	416631	20	3129	1480
miR3979-3p	LOC_Os03g36860.1	145	-25.15	145	-25.15	416648	20	1884	470
miR3979-3p	LOC_Os03g36880.1	164	-28.26	164	-28.26	416650	20	2136	231
miR3979-3p	LOC_Os03g39380.1	143	-20.1	143	-20.1	416895	20	841	183
miR3979-3p	LOC_Os03g39380.2	143	-20.1	143	-20.1	416896	20	973	183

miR3979-3p	LOC_Os03g39380.3	143	-20.1	143	-20.1	416897	20	577	200
miR3979-3p	LOC_Os03g39920.1	150	-27.34	150	-27.34	416949	20	5736	1333
miR3979-3p	LOC_Os03g39960.1	157	-28.62	157	-28.62	416952	20	2978	223
miR3979-3p	LOC_Os03g39960.2	157	-28.62	157	-28.62	416953	20	2981	223
miR3979-3p	LOC_Os03g40310.1	148	-22.99	148	-22.99	416985	20	1037	668
miR3979-3p	LOC_Os03g40600.1	145	-20.38	145	-20.38	417010	20	1016	617
miR3979-3p	LOC_Os03g41350.1	147	-25.52	147	-25.52	417072	20	2743	699
miR3979-3p	LOC_Os03g41350.2	147	-25.52	147	-25.52	417073	20	3433	699
miR3979-3p	LOC_Os03g41980.1	150	-22.75	150	-22.75	417131	20	5637	1333
miR3979-3p	LOC_Os03g41990.1	152	-26.66	152	-26.66	417132	20	2736	1480
miR3979-3p	LOC_Os03g42090.1	158	-28.03	158	-28.03	417143	20	414	384
miR3979-3p	LOC_Os03g42200.1	153	-21.33	153	-21.33	417153	20	1416	33
miR3979-3p	LOC_Os03g42670.1	144	-22.42	144	-22.42	417199	20	1530	156
miR3979-3p	LOC_Os03g42750.1	147	-27.02	147	-27.02	417206	20	3078	2888
miR3979-3p	LOC_Os03g42750.2	147	-27.02	147	-27.02	417207	20	3333	3143
miR3979-3p	LOC_Os03g43220.1	161	-25.74	161	-25.74	417259	20	5073	1681
miR3979-3p	LOC_Os03g44400.1	152	-26.66	152	-26.66	417387	20	3015	1363
miR3979-3p	LOC_Os03g44500.1	147	-25.51	147	-25.51	417398	20	3743	3380
miR3979-3p	LOC_Os03g44510.1	155	-26.87	155	-26.87	417399	20	1926	1668
miR3979-3p	LOC_Os03g44974.1	144	-22.91	144	-22.91	417462	20	264	224
miR3979-3p	LOC_Os03g45300.1	161	-24.14	161	-24.14	417489	20	4183	1544
miR3979-3p	LOC_Os03g46170.1	145	-20.64	145	-20.64	417575	20	3885	135
miR3979-3p	LOC_Os03g46190.1	148	-23.06	148	-23.06	417577	20	1487	822
miR3979-3p	LOC_Os03g46190.2	148	-23.06	148	-23.06	417578	20	1669	1004
miR3979-3p	LOC_Os03g46220.1	150	-22.75	150	-22.75	417580	20	5673	1150
miR3979-3p	LOC_Os03g46280.1	160	-22.79	160	-22.79	417588	20	2505	932
miR3979-3p	LOC_Os03g46440.1	153	-20.7	153	-20.7	417605	20	3220	2772
miR3979-3p	LOC_Os03g46440.2	153	-20.7	153	-20.7	417606	20	2857	2409
miR3979-3p	LOC_Os03g46440.3	153	-20.7	153	-20.7	417607	20	3770	3322
miR3979-3p	LOC_Os03g47730.1	150	-24.78	150	-24.78	417742	20	1628	964
miR3979-3p	LOC_Os03g48160.1	146	-22.81	146	-22.81	417796	20	828	325
miR3979-3p	LOC_Os03g48190.1	141	-20.24	141	-20.24	417800	20	1742	975

miR3979-3p	LOC_Os03g48190.2	141	-20.24	141	-20.24	417801	20	1681	914
miR3979-3p	LOC_Os03g48870.1	152	-23.86	152	-23.86	417880	20	953	29
miR3979-3p	LOC_Os03g49040.1	160	-22.79	160	-22.79	417903	20	2394	932
miR3979-3p	LOC_Os03g49170.1	327	-53.86	170	-27.97	417917	20	3605	178 2381
miR3979-3p	LOC_Os03g49170.3	327	-53.86	170	-27.97	417918	20	3310	178 2086
miR3979-3p	LOC_Os03g50355.1	156	-22.65	156	-22.65	418066	20	1520	343
miR3979-3p	LOC_Os03g50480.1	157	-20.57	157	-20.57	418079	20	2222	1982
miR3979-3p	LOC_Os03g50480.2	157	-20.57	157	-20.57	418080	20	2066	1826
miR3979-3p	LOC_Os03g51440.1	147	-22.01	147	-22.01	418181	20	3315	224
miR3979-3p	LOC_Os03g51520.1	152	-24.91	152	-24.91	418193	20	4728	281
miR3979-3p	LOC_Os03g51520.2	152	-24.91	152	-24.91	418194	20	5070	407
miR3979-3p	LOC_Os03g51690.1	140	-25.56	140	-25.56	418214	20	2604	1501
miR3979-3p	LOC_Os03g51690.2	140	-25.56	140	-25.56	418215	20	2522	1501
miR3979-3p	LOC_Os03g51870.1	158	-22.87	158	-22.87	418233	20	2230	261
miR3979-3p	LOC_Os03g52190.1	150	-22.75	150	-22.75	418270	20	5556	1333
miR3979-3p	LOC_Os03g52200.1	152	-26.66	152	-26.66	418271	20	2700	1135
miR3979-3p	LOC_Os03g52810.1	155	-27.51	155	-27.51	418343	20	5964	4251
miR3979-3p	LOC_Os03g53430.1	145	-25.08	145	-25.08	418415	20	3207	713
miR3979-3p	LOC_Os03g53700.1	151	-20.09	151	-20.09	418449	20	1923	22
miR3979-3p	LOC_Os03g53700.2	151	-20.09	151	-20.09	418450	20	1033	22
miR3979-3p	LOC_Os03g53730.1	153	-24.7	153	-24.7	418454	20	964	357
miR3979-3p	LOC_Os03g54990.1	152	-27.66	152	-27.66	418552	20	588	377
miR3979-3p	LOC_Os03g55200.1	148	-24.08	148	-24.08	418580	20	2110	282
miR3979-3p	LOC_Os03g56250.1	155	-26.22	155	-26.22	418706	20	2809	1589
miR3979-3p	LOC_Os03g57470.1	142	-21.24	142	-21.24	418878	20	3141	759
miR3979-3p	LOC_Os03g57520.1	163	-27.96	163	-27.96	418884	20	504	446
miR3979-3p	LOC_Os03g57690.1	147	-22.13	147	-22.13	418904	20	4068	2631
miR3979-3p	LOC_Os03g59510.1	148	-23.83	148	-23.83	419111	20	3036	1384
miR3979-3p	LOC_Os03g60030.1	156	-22.82	156	-22.82	419169	20	1554	368
miR3979-3p	LOC_Os03g61270.1	155	-26.15	155	-26.15	419317	20	1740	1463
miR3979-3p	LOC_Os03g61760.1	156	-20.37	156	-20.37	419372	20	3926	3400
miR3979-3p	LOC_Os03g62100.1	152	-23.59	152	-23.59	419424	20	639	454

miR3979-3p	LOC_Os03g62720.2	156	-22.32	156	-22.32	419512	20	1322	8
miR3979-3p	LOC_Os03g62720.3	156	-22.32	156	-22.32	419513	20	1688	7
miR3979-3p	LOC_Os03g63330.1	140	-21.86	140	-21.86	419592	20	2067	488
miR3979-3p	LOC_Os03g63760.1	156	-21	156	-21	419642	20	1464	1184
miR3979-3p	LOC_Os04g02900.1	150	-26	150	-26	419944	20	2173	79
miR3979-3p	LOC_Os04g03390.1	150	-23.54	150	-23.54	419991	20	438	129
miR3979-3p	LOC_Os04g03680.1	148	-23.02	148	-23.02	420015	20	975	616
miR3979-3p	LOC_Os04g03890.1	146	-20.35	146	-20.35	420038	20	1455	184
miR3979-3p	LOC_Os04g04270.1	148	-28.3	148	-28.3	420074	20	2703	1017
miR3979-3p	LOC_Os04g04730.1	152	-26.66	152	-26.66	420118	20	2016	949
miR3979-3p	LOC_Os04g05090.1	141	-20.9	141	-20.9	420152	20	1938	518
miR3979-3p	LOC_Os04g05290.1	156	-21.34	156	-21.34	420171	20	2890	1509
miR3979-3p	LOC_Os04g06070.1	154	-25.55	154	-25.55	420241	20	1905	711
miR3979-3p	LOC_Os04g06520.1	155	-20.89	155	-20.89	420285	20	1062	885
miR3979-3p	LOC_Os04g06780.1	154	-27.66	154	-27.66	420307	20	321	127
miR3979-3p	LOC_Os04g07720.1	150	-26.15	150	-26.15	420396	20	1119	848
miR3979-3p	LOC_Os04g07760.1	149	-20.74	149	-20.74	420400	20	3033	1091
miR3979-3p	LOC_Os04g08670.1	154	-26.98	154	-26.98	420491	20	2316	734
miR3979-3p	LOC_Os04g08772.1	154	-27.64	154	-27.64	420505	20	1209	777
miR3979-3p	LOC_Os04g10360.1	154	-21.03	154	-21.03	420630	20	2426	1881
miR3979-3p	LOC_Os04g10530.1	149	-20.35	149	-20.35	420644	20	1920	645
miR3979-3p	LOC_Os04g10700.1	155	-29.17	155	-29.17	420661	20	3042	1543
miR3979-3p	LOC_Os04g12080.1	150	-20.18	150	-20.18	420799	20	2036	1828
miR3979-3p	LOC_Os04g12130.1	157	-21.33	157	-21.33	420804	20	3024	2402
miR3979-3p	LOC_Os04g12700.1	148	-26.97	148	-26.97	420855	20	810	207
miR3979-3p	LOC_Os04g12760.1	150	-22.75	150	-22.75	420860	20	5667	1108
miR3979-3p	LOC_Os04g12770.1	293	-47.13	148	-24.1	420861	20	2115	2029 1480
miR3979-3p	LOC_Os04g12800.1	143	-22.12	143	-22.12	420864	20	470	234
miR3979-3p	LOC_Os04g13310.1	142	-21.24	142	-21.24	420908	20	2715	621
miR3979-3p	LOC_Os04g13880.1	149	-26.33	149	-26.33	420963	20	1935	498
miR3979-3p	LOC_Os04g14340.1	147	-22.38	147	-22.38	421009	20	3398	330
miR3979-3p	LOC_Os04g14410.1	141	-23.91	141	-23.91	421016	20	1691	674

miR3979-3p	LOC_Os04g14460.1	161	-30.7	161	-30.7	421022	20	696	297
miR3979-3p	LOC_Os04g14710.1	140	-20.35	140	-20.35	421049	20	1734	1449
miR3979-3p	LOC_Os04g14710.2	140	-20.35	140	-20.35	421050	20	2315	1560
miR3979-3p	LOC_Os04g16850.1	157	-27.46	157	-27.46	421241	20	1677	1591
miR3979-3p	LOC_Os04g16860.1	141	-20.85	141	-20.85	421242	20	1755	320
miR3979-3p	LOC_Os04g16870.1	157	-27.46	157	-27.46	421243	20	864	778
miR3979-3p	LOC_Os04g17620.1	145	-20.82	145	-20.82	421314	20	2160	1953
miR3979-3p	LOC_Os04g18930.1	143	-20.08	143	-20.08	421443	20	891	613
miR3979-3p	LOC_Os04g19390.1	145	-25.08	145	-25.08	421488	20	1743	230
miR3979-3p	LOC_Os04g20054.1	152	-26.66	152	-26.66	421552	20	3015	1876
miR3979-3p	LOC_Os04g20940.1	152	-20.61	152	-20.61	421641	20	5781	3138
miR3979-3p	LOC_Os04g20950.1	152	-25.01	152	-25.01	421642	20	2697	1303
miR3979-3p	LOC_Os04g21520.1	160	-27.69	160	-27.69	421696	20	1410	1139
miR3979-3p	LOC_Os04g21870.1	142	-20.33	142	-20.33	421730	20	1496	998
miR3979-3p	LOC_Os04g24030.1	144	-22.91	144	-22.91	421928	20	450	410
miR3979-3p	LOC_Os04g24130.1	144	-22.8	144	-22.8	421937	20	1302	687
miR3979-3p	LOC_Os04g24530.1	147	-28.21	147	-28.21	421985	20	1877	422
miR3979-3p	LOC_Os04g27630.1	142	-22.35	142	-22.35	422291	20	4846	340
miR3979-3p	LOC_Os04g27950.1	157	-26.86	157	-26.86	422319	20	991	186
miR3979-3p	LOC_Os04g29990.1	154	-20.49	154	-20.49	422509	20	2124	1560
miR3979-3p	LOC_Os04g30760.1	158	-27.91	158	-27.91	422585	20	1515	859
miR3979-3p	LOC_Os04g30910.1	156	-21.75	156	-21.75	422603	20	1193	45
miR3979-3p	LOC_Os04g30910.2	156	-21.75	156	-21.75	422604	20	1271	45
miR3979-3p	LOC_Os04g30910.3	156	-21.75	156	-21.75	422605	20	1349	30
miR3979-3p	LOC_Os04g32970.1	166	-24.53	166	-24.53	422832	20	1378	435
miR3979-3p	LOC_Os04g32980.1	145	-22.08	145	-22.08	422833	20	1957	1580
miR3979-3p	LOC_Os04g33670.1	141	-23.87	141	-23.87	422915	20	1101	480
miR3979-3p	LOC_Os04g33970.1	163	-29.64	163	-29.64	422946	20	1391	61
miR3979-3p	LOC_Os04g35220.1	147	-21.76	147	-21.76	423072	20	1095	54
miR3979-3p	LOC_Os04g35220.2	147	-21.76	147	-21.76	423073	20	1468	54
miR3979-3p	LOC_Os04g35280.1	146	-23.42	146	-23.42	423079	20	2256	1399
miR3979-3p	LOC_Os04g35390.1	152	-22.39	152	-22.39	423092	20	675	96

miR3979-3p	LOC_Os04g35470.1	152	-26.66	152	-26.66	423101	20	1335	781
miR3979-3p	LOC_Os04g37560.1	160	-27.81	160	-27.81	423234	20	249	119
miR3979-3p	LOC_Os04g38010.1	157	-25.39	157	-25.39	423285	20	1401	15
miR3979-3p	LOC_Os04g39090.1	151	-20.32	151	-20.32	423415	20	1668	77
miR3979-3p	LOC_Os04g39090.2	151	-20.32	151	-20.32	423416	20	1771	77
miR3979-3p	LOC_Os04g39320.1	143	-22.07	143	-22.07	423444	20	1578	1005
miR3979-3p	LOC_Os04g39320.2	143	-22.07	143	-22.07	423445	20	1204	1005
miR3979-3p	LOC_Os04g39320.4	143	-22.07	143	-22.07	423446	20	1300	1005
miR3979-3p	LOC_Os04g40150.1	141	-20.99	141	-20.99	423540	20	2661	2242
miR3979-3p	LOC_Os04g40860.1	145	-22.29	145	-22.29	423631	20	2466	510
miR3979-3p	LOC_Os04g41470.1	148	-20.85	148	-20.85	423707	20	2439	61
miR3979-3p	LOC_Os04g41470.2	148	-20.85	148	-20.85	423708	20	2520	61
miR3979-3p	LOC_Os04g41670.1	160	-27.69	160	-27.69	423730	20	2666	827
miR3979-3p	LOC_Os04g42740.1	147	-21.7	147	-21.7	423858	20	2382	170
miR3979-3p	LOC_Os04g43070.1	149	-23.59	149	-23.59	423905	20	2476	127
miR3979-3p	LOC_Os04g44150.1	306	-46.32	154	-24.18	424030	20	1425	80 291
miR3979-3p	LOC_Os04g44440.1	149	-21.11	149	-21.11	424064	20	1071	174
miR3979-3p	LOC_Os04g44700.1	147	-20.54	147	-20.54	424101	20	3143	25
miR3979-3p	LOC_Os04g44700.2	147	-20.54	147	-20.54	424102	20	2672	25
miR3979-3p	LOC_Os04g45550.1	152	-25.01	152	-25.01	424204	20	2655	1192
miR3979-3p	LOC_Os04g45730.1	288	-43.3	148	-23.23	424222	20	1637	1355 553
miR3979-3p	LOC_Os04g45930.1	158	-28.68	158	-28.68	424244	20	3903	1218
miR3979-3p	LOC_Os04g45930.2	158	-28.68	158	-28.68	424245	20	3979	1218
miR3979-3p	LOC_Os04g45930.3	158	-28.68	158	-28.68	424246	20	2615	1218
miR3979-3p	LOC_Os04g45950.1	157	-26.52	157	-26.52	424248	20	1841	1551
miR3979-3p	LOC_Os04g45950.2	157	-26.52	157	-26.52	424249	20	2182	1551
miR3979-3p	LOC_Os04g46440.1	144	-22.76	144	-22.76	424308	20	1052	129
miR3979-3p	LOC_Os04g46790.1	147	-23.93	147	-23.93	424356	20	672	268
miR3979-3p	LOC_Os04g47780.1	141	-22.05	141	-22.05	424476	20	1678	1239
miR3979-3p	LOC_Os04g49000.1	161	-25.08	161	-25.08	424624	20	1240	316
miR3979-3p	LOC_Os04g49140.1	140	-21.58	140	-21.58	424639	20	1175	340
miR3979-3p	LOC_Os04g49410.1	148	-20.16	148	-20.16	424663	20	2163	439

miR3979-3p	LOC_Os04g49510.1	168	-26.87	168	-26.87	424675	20	3137	156
miR3979-3p	LOC_Os04g49510.2	168	-26.87	168	-26.87	424676	20	1862	156
miR3979-3p	LOC_Os04g49510.3	168	-26.87	168	-26.87	424677	20	1378	156
miR3979-3p	LOC_Os04g49510.4	168	-26.87	168	-26.87	424678	20	1797	156
miR3979-3p	LOC_Os04g50184.1	148	-24.9	148	-24.9	424758	20	402	129
miR3979-3p	LOC_Os04g50910.1	150	-25.02	150	-25.02	424794	20	564	485
miR3979-3p	LOC_Os04g51100.1	161	-23.63	161	-23.63	424816	20	1596	65
miR3979-3p	LOC_Os04g51360.1	146	-22.95	146	-22.95	424850	20	1047	883
miR3979-3p	LOC_Os04g52050.1	155	-26.52	155	-26.52	424952	20	3461	1468
miR3979-3p	LOC_Os04g52390.1	145	-21.62	145	-21.62	424991	20	3493	361
miR3979-3p	LOC_Os04g52640.1	156	-25.92	156	-25.92	425021	20	4444	3083
miR3979-3p	LOC_Os04g52640.2	156	-25.92	156	-25.92	425022	20	4899	3083
miR3979-3p	LOC_Os04g52640.3	156	-25.92	156	-25.92	425023	20	4418	3083
miR3979-3p	LOC_Os04g52780.1	141	-21.74	141	-21.74	425044	20	4046	766
miR3979-3p	LOC_Os04g53210.1	144	-23.15	144	-23.15	425093	20	1539	329
miR3979-3p	LOC_Os04g53210.3	144	-23.15	144	-23.15	425094	20	1476	329
miR3979-3p	LOC_Os04g53490.1	156	-22.32	156	-22.32	425132	20	1251	550
miR3979-3p	LOC_Os04g53660.1	157	-26.31	157	-26.31	425155	20	3132	2264
miR3979-3p	LOC_Os04g53660.2	157	-26.31	157	-26.31	425156	20	2993	2125
miR3979-3p	LOC_Os04g53660.3	157	-26.31	157	-26.31	425157	20	3047	2219
miR3979-3p	LOC_Os04g53870.1	156	-25.5	156	-25.5	425183	20	5784	4055
miR3979-3p	LOC_Os04g54660.1	309	-54.77	164	-29.69	425280	20	3489	1371 581
miR3979-3p	LOC_Os04g56110.1	147	-23.53	147	-23.53	425443	20	1873	1058
miR3979-3p	LOC_Os04g56110.2	147	-23.53	147	-23.53	425444	20	2568	1753
miR3979-3p	LOC_Os04g56110.3	147	-23.53	147	-23.53	425445	20	1669	854
miR3979-3p	LOC_Os04g56720.1	160	-27.66	160	-27.66	425523	20	2215	62
miR3979-3p	LOC_Os04g57780.1	154	-26.01	154	-26.01	425671	20	1062	927
miR3979-3p	LOC_Os04g57780.2	154	-26.01	154	-26.01	425672	20	1149	1018
miR3979-3p	LOC_Os04g58620.1	156	-24.27	156	-24.27	425776	20	4333	963
miR3979-3p	LOC_Os04g58780.1	176	-32.16	176	-32.16	425801	20	3519	233
miR3979-3p	LOC_Os04g58780.2	176	-32.16	176	-32.16	425802	20	3499	233
miR3979-3p	LOC_Os05g01430.1	151	-28.05	151	-28.05	425980	20	1020	501

miR3979-3p	LOC_Os05g01444.1	151	-28.05	151	-28.05	425982	20	1654	969
miR3979-3p	LOC_Os05g02040.1	160	-25.15	160	-25.15	426067	20	3409	1
miR3979-3p	LOC_Os05g02300.1	154	-25.98	154	-25.98	426101	20	797	26
miR3979-3p	LOC_Os05g02350.1	325	-52.57	166	-28.48	426106	20	1542	333 183
miR3979-3p	LOC_Os05g02950.1	150	-22.75	150	-22.75	426175	20	4116	1149
miR3979-3p	LOC_Os05g03060.1	153	-26.08	153	-26.08	426191	20	2258	669
miR3979-3p	LOC_Os05g03650.1	146	-29.22	146	-29.22	426263	20	3084	1127
miR3979-3p	LOC_Os05g03690.1	146	-20.1	146	-20.1	426267	20	5061	2253
miR3979-3p	LOC_Os05g04270.1	144	-22.91	144	-22.91	426331	20	264	224
miR3979-3p	LOC_Os05g04370.1	140	-20.13	140	-20.13	426342	20	2800	2348
miR3979-3p	LOC_Os05g04540.1	147	-21.76	147	-21.76	426361	20	978	641
miR3979-3p	LOC_Os05g04570.1	148	-22.95	148	-22.95	426363	20	951	453
miR3979-3p	LOC_Os05g04940.1	149	-21.39	149	-21.39	426403	20	1275	1102
miR3979-3p	LOC_Os05g05300.1	154	-20.34	154	-20.34	426444	20	3778	94
miR3979-3p	LOC_Os05g05354.1	146	-22.6	146	-22.6	426452	20	2435	2294
miR3979-3p	LOC_Os05g05354.2	146	-22.6	146	-22.6	426453	20	1513	1372
miR3979-3p	LOC_Os05g05590.1	154	-22.14	154	-22.14	426479	20	2135	397
miR3979-3p	LOC_Os05g05740.1	147	-20.99	147	-20.99	426501	20	975	696
miR3979-3p	LOC_Os05g05780.1	140	-21.82	140	-21.82	426505	20	4228	1302
miR3979-3p	LOC_Os05g05810.1	152	-25.29	152	-25.29	426508	20	3152	923
miR3979-3p	LOC_Os05g06480.1	149	-21.49	149	-21.49	426584	20	2793	689
miR3979-3p	LOC_Os05g07090.3	152	-20.11	152	-20.11	426665	20	1951	1749
miR3979-3p	LOC_Os05g07210.1	151	-24.01	151	-24.01	426676	20	1498	800
miR3979-3p	LOC_Os05g08040.1	156	-21.6	156	-21.6	426770	20	2103	409
miR3979-3p	LOC_Os05g08240.1	141	-21.64	141	-21.64	426793	20	456	202
miR3979-3p	LOC_Os05g09490.1	153	-26.09	153	-26.09	426944	20	1263	248
miR3979-3p	LOC_Os05g09490.2	153	-26.09	153	-26.09	426945	20	1340	325
miR3979-3p	LOC_Os05g10250.1	150	-22.75	150	-22.75	426983	20	5391	1399
miR3979-3p	LOC_Os05g10960.1	146	-20.1	146	-20.1	427067	20	4257	1665
miR3979-3p	LOC_Os05g11140.1	157	-21.99	157	-21.99	427087	20	2540	1619
miR3979-3p	LOC_Os05g11140.2	157	-21.99	157	-21.99	427088	20	2720	1799
miR3979-3p	LOC_Os05g11140.3	157	-21.99	157	-21.99	427089	20	2589	1668

miR3979-3p	LOC_Os05g13920.1	152	-21.75	152	-21.75	427332	20	3969	650
miR3979-3p	LOC_Os05g14194.1	152	-26.28	152	-26.28	427363	20	1821	1619
miR3979-3p	LOC_Os05g14194.2	152	-26.28	152	-26.28	427364	20	1419	1257
miR3979-3p	LOC_Os05g15180.1	151	-24.17	151	-24.17	427463	20	744	582
miR3979-3p	LOC_Os05g15180.2	151	-24.17	151	-24.17	427464	20	748	586
miR3979-3p	LOC_Os05g15580.1	156	-22.42	156	-22.42	427508	20	5370	2213
miR3979-3p	LOC_Os05g16360.1	144	-21.57	144	-21.57	427584	20	960	447
miR3979-3p	LOC_Os05g18310.1	149	-26.33	149	-26.33	427787	20	1101	118
miR3979-3p	LOC_Os05g18520.1	146	-20.1	146	-20.1	427805	20	3864	1665
miR3979-3p	LOC_Os05g18920.1	144	-22.8	144	-22.8	427850	20	3099	2442
miR3979-3p	LOC_Os05g19310.1	146	-20.1	146	-20.1	427890	20	2887	2374
miR3979-3p	LOC_Os05g20340.1	152	-25	152	-25	428001	20	2418	955
miR3979-3p	LOC_Os05g20750.1	141	-20.47	141	-20.47	428042	20	3747	320
miR3979-3p	LOC_Os05g20810.1	150	-24.51	150	-24.51	428048	20	711	283
miR3979-3p	LOC_Os05g20910.1	160	-29.3	160	-29.3	428058	20	294	157
miR3979-3p	LOC_Os05g21100.1	150	-22.75	150	-22.75	428083	20	5910	1333
miR3979-3p	LOC_Os05g21120.1	146	-20.72	146	-20.72	428085	20	5943	3651
miR3979-3p	LOC_Os05g22500.1	157	-25.96	157	-25.96	428126	20	1845	1316
miR3979-3p	LOC_Os05g22660.1	152	-20.5	152	-20.5	428142	20	2568	1521
miR3979-3p	LOC_Os05g22840.1	151	-22.72	151	-22.72	428162	20	516	99
miR3979-3p	LOC_Os05g22870.1	152	-21.06	152	-21.06	428166	20	2499	1987
miR3979-3p	LOC_Os05g23600.1	148	-23.91	148	-23.91	428251	20	2213	488
miR3979-3p	LOC_Os05g23600.2	148	-23.91	148	-23.91	428252	20	1544	488
miR3979-3p	LOC_Os05g24310.1	146	-20.1	146	-20.1	428326	20	3444	669
miR3979-3p	LOC_Os05g25075.1	149	-20.42	149	-20.42	428411	20	2626	2443
miR3979-3p	LOC_Os05g25170.1	160	-22.79	160	-22.79	428421	20	2778	1085
miR3979-3p	LOC_Os05g25300.1	145	-20.62	145	-20.62	428434	20	3276	1411
miR3979-3p	LOC_Os05g25540.1	158	-22.68	158	-22.68	428461	20	1838	1607
miR3979-3p	LOC_Os05g26570.1	150	-22.75	150	-22.75	428571	20	3516	1333
miR3979-3p	LOC_Os05g26780.1	145	-24.76	145	-24.76	428591	20	1191	185
miR3979-3p	LOC_Os05g27580.1	144	-22.76	144	-22.76	428668	20	759	250
miR3979-3p	LOC_Os05g28290.1	158	-22.96	158	-22.96	428747	20	1240	81

miR3979-3p	LOC_Os05g28300.1	161	-31.55	161	-31.55	428748	20	4481	33
miR3979-3p	LOC_Os05g29110.1	161	-22.04	161	-22.04	428839	20	4722	1910
miR3979-3p	LOC_Os05g29680.1	147	-23.27	147	-23.27	428848	20	429	15
miR3979-3p	LOC_Os05g31830.1	156	-26.12	156	-26.12	429096	20	1443	318
miR3979-3p	LOC_Os05g32060.1	299	-49.79	153	-26.59	429117	20	1958	739 176
miR3979-3p	LOC_Os05g32280.1	143	-20.08	143	-20.08	429143	20	489	202
miR3979-3p	LOC_Os05g33630.1	140	-20.98	140	-20.98	429298	20	3449	2430
miR3979-3p	LOC_Os05g33630.2	140	-20.98	140	-20.98	429299	20	3737	2430
miR3979-3p	LOC_Os05g33630.3	140	-20.98	140	-20.98	429300	20	3516	2430
miR3979-3p	LOC_Os05g33630.4	140	-20.98	140	-20.98	429301	20	3725	2430
miR3979-3p	LOC_Os05g33630.5	140	-20.98	140	-20.98	429302	20	3825	2530
miR3979-3p	LOC_Os05g33630.6	140	-20.98	140	-20.98	429303	20	2890	1595
miR3979-3p	LOC_Os05g33980.1	146	-22.5	146	-22.5	429338	20	5592	3038
miR3979-3p	LOC_Os05g33990.1	145	-22.39	145	-22.39	429339	20	3030	1276
miR3979-3p	LOC_Os05g34520.1	150	-20.23	150	-20.23	429400	20	2289	1760
miR3979-3p	LOC_Os05g34890.1	153	-21.73	153	-21.73	429447	20	687	526
miR3979-3p	LOC_Os05g35266.1	158	-21.3	158	-21.3	429492	20	1825	844
miR3979-3p	LOC_Os05g35266.2	158	-21.3	158	-21.3	429493	20	2109	844
miR3979-3p	LOC_Os05g35266.3	158	-21.3	158	-21.3	429494	20	1908	947
miR3979-3p	LOC_Os05g35770.1	141	-20.26	141	-20.26	429550	20	570	509
miR3979-3p	LOC_Os05g35860.1	144	-27.36	144	-27.36	429558	20	3795	2794
miR3979-3p	LOC_Os05g35960.1	148	-26.28	148	-26.28	429568	20	663	84
miR3979-3p	LOC_Os05g36020.1	152	-22	152	-22	429574	20	324	42
miR3979-3p	LOC_Os05g36170.1	155	-27.51	155	-27.51	429597	20	6018	4248
miR3979-3p	LOC_Os05g37230.1	145	-20.91	145	-20.91	429675	20	4110	402
miR3979-3p	LOC_Os05g38100.1	150	-22.75	150	-22.75	429775	20	5511	1333
miR3979-3p	LOC_Os05g38110.1	152	-26.66	152	-26.66	429776	20	2754	1441
miR3979-3p	LOC_Os05g38180.1	150	-24.63	150	-24.63	429784	20	1319	246
miR3979-3p	LOC_Os05g38180.2	150	-24.63	150	-24.63	429785	20	1174	246
miR3979-3p	LOC_Os05g38180.3	150	-24.63	150	-24.63	429786	20	1485	246
miR3979-3p	LOC_Os05g38180.4	150	-24.63	150	-24.63	429787	20	1487	246
miR3979-3p	LOC_Os05g38390.1	140	-22.52	140	-22.52	429818	20	1542	921

miR3979-3p	LOC_Os05g39410.1	156	-20.18	156	-20.18	429947	20	2660	2433
miR3979-3p	LOC_Os05g39410.2	156	-20.18	156	-20.18	429948	20	2908	2681
miR3979-3p	LOC_Os05g39410.3	156	-20.18	156	-20.18	429949	20	2739	2512
miR3979-3p	LOC_Os05g40490.1	150	-20.92	150	-20.92	430078	20	2226	557
miR3979-3p	LOC_Os05g40770.1	145	-22.74	145	-22.74	430108	20	3359	653
miR3979-3p	LOC_Os05g40770.2	145	-22.74	145	-22.74	430109	20	3031	653
miR3979-3p	LOC_Os05g41795.1	141	-20.75	141	-20.75	430228	20	2062	289
miR3979-3p	LOC_Os05g41830.1	161	-26.6	161	-26.6	430233	20	3906	2712
miR3979-3p	LOC_Os05g42448.1	142	-22.95	142	-22.95	430304	20	3933	498
miR3979-3p	LOC_Os05g43530.1	156	-27.24	156	-27.24	430368	20	2651	1228
miR3979-3p	LOC_Os05g44360.1	153	-24.05	153	-24.05	430475	20	2831	419
miR3979-3p	LOC_Os05g44360.2	153	-24.05	153	-24.05	430476	20	2410	419
miR3979-3p	LOC_Os05g44760.1	140	-21.04	140	-21.04	430522	20	2740	2670
miR3979-3p	LOC_Os05g44770.1	140	-21.04	140	-21.04	430523	20	4042	1294
miR3979-3p	LOC_Os05g45200.1	146	-26.8	146	-26.8	430575	20	1517	1037
miR3979-3p	LOC_Os05g45430.1	143	-23.27	143	-23.27	430605	20	1842	1510
miR3979-3p	LOC_Os05g45510.1	155	-24.11	155	-24.11	430613	20	1848	779
miR3979-3p	LOC_Os05g47510.1	156	-27.36	156	-27.36	430804	20	1323	450
miR3979-3p	LOC_Os05g48060.1	147	-23.36	147	-23.36	430867	20	1980	60
miR3979-3p	LOC_Os05g48700.1	145	-21.05	145	-21.05	430953	20	1708	947
miR3979-3p	LOC_Os05g48810.1	145	-20.55	145	-20.55	430969	20	1624	986
miR3979-3p	LOC_Os05g49290.1	142	-20.04	142	-20.04	431038	20	2581	1718
miR3979-3p	LOC_Os05g49290.2	142	-20.04	142	-20.04	431039	20	2413	1550
miR3979-3p	LOC_Os05g49290.3	142	-20.04	142	-20.04	431040	20	2443	1623
miR3979-3p	LOC_Os05g50290.1	160	-23.62	160	-23.62	431164	20	1487	10
miR3979-3p	LOC_Os05g50450.1	140	-22.62	140	-22.62	431181	20	429	54
miR3979-3p	LOC_Os05g50520.1	146	-20.87	146	-20.87	431190	20	4215	1291
miR3979-3p	LOC_Os05g50750.1	167	-27.08	167	-27.08	431215	20	3003	1133
miR3979-3p	LOC_Os05g50750.2	167	-27.08	167	-27.08	431216	20	3085	1215
miR3979-3p	LOC_Os05g50750.3	167	-27.08	167	-27.08	431217	20	2810	1120
miR3979-3p	LOC_Os05g51560.1	142	-20.26	142	-20.26	431328	20	2188	1904
miR3979-3p	LOC_Os05g51560.2	142	-20.26	142	-20.26	431329	20	2349	1904

miR3979-3p	LOC_Os05g51560.3	142	-20.26	142	-20.26	431330	20	2275	2013
miR3979-3p	LOC_Os05g51890.1	149	-26.33	149	-26.33	431376	20	1512	118
miR3979-3p	LOC_Os05g52070.1	145	-20.91	145	-20.91	431390	20	5016	399
miR3979-3p	LOC_Os06g01150.1	164	-30.36	164	-30.36	431407	20	570	198
miR3979-3p	LOC_Os06g01430.1	148	-23.83	148	-23.83	431438	20	2748	1174
miR3979-3p	LOC_Os06g04610.1	154	-21.88	154	-21.88	431811	20	4005	794
miR3979-3p	LOC_Os06g05359.1	145	-21.76	145	-21.76	431902	20	4325	3097
miR3979-3p	LOC_Os06g05680.1	151	-22.17	151	-22.17	431946	20	4803	2899
miR3979-3p	LOC_Os06g06290.1	141	-20.67	141	-20.67	432035	20	1929	1298
miR3979-3p	LOC_Os06g06802.1	151	-22.17	151	-22.17	432094	20	5712	3808
miR3979-3p	LOC_Os06g07380.1	144	-20.34	144	-20.34	432157	20	1458	720
miR3979-3p	LOC_Os06g07470.1	140	-22.18	140	-22.18	432166	20	1614	625
miR3979-3p	LOC_Os06g07474.1	151	-21.06	151	-21.06	432167	20	1924	1570
miR3979-3p	LOC_Os06g07474.2	151	-21.06	151	-21.06	432168	20	1935	1581
miR3979-3p	LOC_Os06g07860.1	146	-20.35	146	-20.35	432209	20	1754	101
miR3979-3p	LOC_Os06g08790.1	148	-21.81	148	-21.81	432310	20	2838	336
miR3979-3p	LOC_Os06g09450.2	151	-23.95	151	-23.95	432396	20	3816	3522
miR3979-3p	LOC_Os06g09760.1	162	-25.57	162	-25.57	432431	20	375	1
miR3979-3p	LOC_Os06g10710.1	149	-21.65	149	-21.65	432538	20	1530	145
miR3979-3p	LOC_Os06g10930.1	157	-20.44	157	-20.44	432564	20	1921	1652
miR3979-3p	LOC_Os06g11270.1	148	-23.21	148	-23.21	432599	20	1338	276
miR3979-3p	LOC_Os06g12110.1	160	-23.96	160	-23.96	432695	20	1479	179
miR3979-3p	LOC_Os06g12110.2	160	-23.96	160	-23.96	432696	20	1629	179
miR3979-3p	LOC_Os06g14270.1	153	-30.14	153	-30.14	432943	20	675	643
miR3979-3p	LOC_Os06g14540.1	140	-20.17	140	-20.17	432981	20	2092	1633
miR3979-3p	LOC_Os06g14550.1	163	-26.69	163	-26.69	432982	20	733	305
miR3979-3p	LOC_Os06g14560.1	143	-20.08	143	-20.08	432983	20	804	753
miR3979-3p	LOC_Os06g14570.1	146	-20.1	146	-20.1	432984	20	3456	2496
miR3979-3p	LOC_Os06g15260.1	157	-21.33	157	-21.33	433051	20	960	326
miR3979-3p	LOC_Os06g15490.1	150	-20.24	150	-20.24	433076	20	441	357
miR3979-3p	LOC_Os06g15510.1	142	-21.5	142	-21.5	433078	20	3909	450
miR3979-3p	LOC_Os06g15550.1	151	-25.29	151	-25.29	433082	20	1182	6

miR3979-3p	LOC_Os06g18000.1	152	-20.32	152	-20.32	433327	20	1930	1437
miR3979-3p	LOC_Os06g20240.1	148	-23.69	148	-23.69	433505	20	648	622
miR3979-3p	LOC_Os06g20840.1	150	-23.35	150	-23.35	433570	20	1449	674
miR3979-3p	LOC_Os06g21360.1	141	-20.35	141	-20.35	433626	20	1845	1364
miR3979-3p	LOC_Os06g22170.1	148	-24.33	148	-24.33	433720	20	411	305
miR3979-3p	LOC_Os06g22320.1	140	-22.08	140	-22.08	433733	20	2667	1621
miR3979-3p	LOC_Os06g23340.1	147	-20.06	147	-20.06	433847	20	2388	212
miR3979-3p	LOC_Os06g23890.1	154	-26.59	154	-26.59	433903	20	2592	816
miR3979-3p	LOC_Os06g25280.1	162	-23.59	162	-23.59	434041	20	2136	769
miR3979-3p	LOC_Os06g26260.1	152	-27.1	152	-27.1	434134	20	2016	87
miR3979-3p	LOC_Os06g30200.1	157	-26.53	157	-26.53	434443	20	453	4
miR3979-3p	LOC_Os06g30440.1	150	-21.52	150	-21.52	434464	20	2330	1447
miR3979-3p	LOC_Os06g30440.2	150	-21.52	150	-21.52	434465	20	1482	624
miR3979-3p	LOC_Os06g30710.1	148	-20.12	148	-20.12	434496	20	2164	37
miR3979-3p	LOC_Os06g30710.2	148	-20.12	148	-20.12	434497	20	1885	64
miR3979-3p	LOC_Os06g30710.3	148	-20.12	148	-20.12	434498	20	2125	64
miR3979-3p	LOC_Os06g30930.1	148	-25.13	148	-25.13	434525	20	1374	451
miR3979-3p	LOC_Os06g31630.1	148	-25.12	148	-25.12	434592	20	2034	1871
miR3979-3p	LOC_Os06g32970.1	141	-20.3	141	-20.3	434718	20	717	92
miR3979-3p	LOC_Os06g35470.1	149	-24.02	149	-24.02	434968	20	1747	189
miR3979-3p	LOC_Os06g37460.1	145	-20.49	145	-20.49	435179	20	1812	187
miR3979-3p	LOC_Os06g39470.1	147	-26.71	147	-26.71	435383	20	1562	413
miR3979-3p	LOC_Os06g39660.1	152	-21.78	152	-21.78	435404	20	1534	571
miR3979-3p	LOC_Os06g39820.1	145	-23.38	145	-23.38	435431	20	3387	201
miR3979-3p	LOC_Os06g40620.1	161	-21.22	161	-21.22	435520	20	1443	206
miR3979-3p	LOC_Os06g40620.2	161	-21.22	161	-21.22	435521	20	1409	206
miR3979-3p	LOC_Os06g40940.1	141	-21.39	141	-21.39	435562	20	3559	424
miR3979-3p	LOC_Os06g40940.3	141	-21.39	141	-21.39	435564	20	2245	424
miR3979-3p	LOC_Os06g41730.1	145	-24.38	145	-24.38	435648	20	2361	1006
miR3979-3p	LOC_Os06g41730.2	145	-24.38	145	-24.38	435649	20	2172	1006
miR3979-3p	LOC_Os06g42590.1	150	-22.75	150	-22.75	435753	20	4815	1213
miR3979-3p	LOC_Os06g42600.1	152	-25	152	-25	435754	20	2664	1135

miR3979-3p	LOC_Os06g43430.1	165	-27.01	165	-27.01	435838	20	1748	323
miR3979-3p	LOC_Os06g43450.1	142	-21.24	142	-21.24	435840	20	3198	759
miR3979-3p	LOC_Os06g43520.1	145	-20.38	145	-20.38	435849	20	2698	476
miR3979-3p	LOC_Os06g43850.1	152	-20.7	152	-20.7	435891	20	1155	618
miR3979-3p	LOC_Os06g44060.1	156	-23.22	156	-23.22	435918	20	2053	856
miR3979-3p	LOC_Os06g44060.2	156	-23.22	156	-23.22	435919	20	1973	776
miR3979-3p	LOC_Os06g44620.1	153	-21.08	153	-21.08	435980	20	2152	1489
miR3979-3p	LOC_Os06g44620.2	153	-21.08	153	-21.08	435981	20	3213	2550
miR3979-3p	LOC_Os06g45190.1	162	-21.32	162	-21.32	436052	20	574	50
miR3979-3p	LOC_Os06g45730.1	152	-23.33	152	-23.33	436115	20	1495	758
miR3979-3p	LOC_Os06g45740.1	146	-22.8	146	-22.8	436116	20	1599	339
miR3979-3p	LOC_Os06g45830.1	150	-20.56	150	-20.56	436128	20	3058	2819
miR3979-3p	LOC_Os06g46330.1	173	-35	173	-35	436184	20	1849	1773
miR3979-3p	LOC_Os06g47570.1	149	-22.37	149	-22.37	436346	20	2976	428
miR3979-3p	LOC_Os06g47580.1	153	-21.04	153	-21.04	436347	20	3687	1460
miR3979-3p	LOC_Os06g47580.2	153	-21.04	153	-21.04	436348	20	3833	1606
miR3979-3p	LOC_Os06g47580.3	153	-21.04	153	-21.04	436349	20	3822	1595
miR3979-3p	LOC_Os06g47950.1	148	-20.03	148	-20.03	436391	20	1881	1383
miR3979-3p	LOC_Os06g48440.1	145	-20.91	145	-20.91	436448	20	4608	402
miR3979-3p	LOC_Os06g48900.1	146	-20.1	146	-20.1	436503	20	5472	2571
miR3979-3p	LOC_Os06g49110.1	145	-20.1	145	-20.1	436534	20	2198	1928
miR3979-3p	LOC_Os06g49110.2	145	-20.1	145	-20.1	436535	20	2357	2016
miR3979-3p	LOC_Os06g49110.3	145	-20.1	145	-20.1	436536	20	2253	1928
miR3979-3p	LOC_Os06g49480.1	158	-22.4	158	-22.4	436588	20	1041	854
miR3979-3p	LOC_Os06g49480.2	158	-22.4	158	-22.4	436589	20	1067	880
miR3979-3p	LOC_Os06g51530.1	169	-34.77	169	-34.77	436856	20	872	508
miR3979-3p	LOC_Os07g02600.1	157	-21.33	157	-21.33	437042	20	2619	1997
miR3979-3p	LOC_Os07g02760.1	150	-24.7	150	-24.7	437059	20	1979	229
miR3979-3p	LOC_Os07g02760.2	150	-24.7	150	-24.7	437060	20	1927	242
miR3979-3p	LOC_Os07g02760.3	150	-24.7	150	-24.7	437061	20	1502	242
miR3979-3p	LOC_Os07g04200.1	155	-26.93	155	-26.93	437210	20	1235	1045
miR3979-3p	LOC_Os07g07470.1	156	-25.08	156	-25.08	437570	20	1451	1114

miR3979-3p	LOC_Os07g09100.1	140	-22.26	140	-22.26	437749	20	807	23
miR3979-3p	LOC_Os07g09384.1	149	-20.62	149	-20.62	437777	20	3349	343
miR3979-3p	LOC_Os07g10630.1	288	-43.22	148	-22.18	437914	20	3838	1029 848
miR3979-3p	LOC_Os07g11480.1	159	-24.36	159	-24.36	438015	20	3153	304
miR3979-3p	LOC_Os07g12480.1	140	-21.14	140	-21.14	438112	20	1983	546
miR3979-3p	LOC_Os07g12790.1	156	-25.13	156	-25.13	438147	20	1011	23
miR3979-3p	LOC_Os07g13140.1	144	-22.2	144	-22.2	438183	20	3753	668
miR3979-3p	LOC_Os07g13234.1	160	-27.61	160	-27.61	438193	20	2566	1887
miR3979-3p	LOC_Os07g13234.2	160	-27.61	160	-27.61	438194	20	2285	1593
miR3979-3p	LOC_Os07g14050.1	152	-21.75	152	-21.75	438271	20	2514	461
miR3979-3p	LOC_Os07g15370.1	152	-24.97	152	-24.97	438420	20	2213	1518
miR3979-3p	LOC_Os07g15370.2	152	-24.97	152	-24.97	438421	20	2486	1518
miR3979-3p	LOC_Os07g16700.1	156	-21.34	156	-21.34	438547	20	1968	1019
miR3979-3p	LOC_Os07g17270.1	140	-21.32	140	-21.32	438609	20	5502	1884
miR3979-3p	LOC_Os07g17330.1	140	-21.15	140	-21.15	438615	20	595	218
miR3979-3p	LOC_Os07g18890.1	154	-27.64	154	-27.64	438769	20	4953	2805
miR3979-3p	LOC_Os07g18970.1	151	-22.17	151	-22.17	438776	20	4947	2899
miR3979-3p	LOC_Os07g19250.1	149	-24.66	149	-24.66	438802	20	528	178
miR3979-3p	LOC_Os07g20690.1	146	-23.37	146	-23.37	438895	20	2211	1772
miR3979-3p	LOC_Os07g20810.1	146	-20.1	146	-20.1	438906	20	3021	831
miR3979-3p	LOC_Os07g22060.1	162	-25.34	162	-25.34	438929	20	3363	842
miR3979-3p	LOC_Os07g22070.1	144	-21.55	144	-21.55	438930	20	432	383
miR3979-3p	LOC_Os07g22210.1	168	-34.83	168	-34.83	438944	20	492	98
miR3979-3p	LOC_Os07g22830.1	157	-27.46	157	-27.46	439024	20	684	631
miR3979-3p	LOC_Os07g23050.1	161	-22.04	161	-22.04	439051	20	1656	251
miR3979-3p	LOC_Os07g23310.1	152	-26.66	152	-26.66	439076	20	3117	1468
miR3979-3p	LOC_Os07g25190.1	156	-21.34	156	-21.34	439275	20	3768	1430
miR3979-3p	LOC_Os07g26030.1	158	-26.71	158	-26.71	439360	20	2382	1738
miR3979-3p	LOC_Os07g26960.1	154	-22.71	154	-22.71	439460	20	672	203
miR3979-3p	LOC_Os07g27040.1	169	-26.06	169	-26.06	439467	20	375	249
miR3979-3p	LOC_Os07g27460.1	158	-20.49	158	-20.49	439514	20	1198	159
miR3979-3p	LOC_Os07g27470.1	153	-26.56	153	-26.56	439516	20	2163	1791

miR3979-3p	LOC_Os07g27780.1	160	-25.33	160	-25.33	439549	20	1148	424
miR3979-3p	LOC_Os07g27820.1	145	-22.16	145	-22.16	439553	20	2283	1094
miR3979-3p	LOC_Os07g28280.1	160	-27.34	160	-27.34	439601	20	730	477
miR3979-3p	LOC_Os07g28490.1	158	-24.73	158	-24.73	439623	20	2202	1333
miR3979-3p	LOC_Os07g28600.1	160	-22.79	160	-22.79	439633	20	2685	1169
miR3979-3p	LOC_Os07g29070.1	145	-20.64	145	-20.64	439683	20	666	334
miR3979-3p	LOC_Os07g30020.1	140	-20.32	140	-20.32	439786	20	3694	3673
miR3979-3p	LOC_Os07g30050.1	149	-26.33	149	-26.33	439789	20	1545	282
miR3979-3p	LOC_Os07g31630.1	150	-22.75	150	-22.75	439961	20	5046	520
miR3979-3p	LOC_Os07g31640.1	152	-26.66	152	-26.66	439962	20	2724	1336
miR3979-3p	LOC_Os07g31760.1	153	-27.21	153	-27.21	439973	20	243	199
miR3979-3p	LOC_Os07g32100.1	144	-22.03	144	-22.03	440001	20	1017	522
miR3979-3p	LOC_Os07g32420.1	155	-28.03	155	-28.03	440041	20	2223	169
miR3979-3p	LOC_Os07g32420.2	155	-28.03	155	-28.03	440042	20	2150	157
miR3979-3p	LOC_Os07g32420.3	155	-28.03	155	-28.03	440043	20	2065	169
miR3979-3p	LOC_Os07g32560.1	140	-21.35	140	-21.35	440055	20	1505	1077
miR3979-3p	LOC_Os07g34980.1	165	-28.83	165	-28.83	440315	20	1977	30
miR3979-3p	LOC_Os07g35620.1	148	-23.99	148	-23.99	440390	20	663	515
miR3979-3p	LOC_Os07g36170.1	144	-20.79	144	-20.79	440454	20	2833	1454
miR3979-3p	LOC_Os07g36170.2	144	-20.79	144	-20.79	440455	20	2906	1538
miR3979-3p	LOC_Os07g36510.1	146	-22.95	146	-22.95	440493	20	684	52
miR3979-3p	LOC_Os07g37040.1	152	-25	152	-25	440552	20	1793	1461
miR3979-3p	LOC_Os07g37620.1	156	-23.92	156	-23.92	440616	20	1589	1384
miR3979-3p	LOC_Os07g37650.1	147	-21.39	147	-21.39	440619	20	2202	1885
miR3979-3p	LOC_Os07g37830.1	150	-26.32	150	-26.32	440640	20	2957	1908
miR3979-3p	LOC_Os07g37830.2	150	-26.32	150	-26.32	440641	20	2814	1765
miR3979-3p	LOC_Os07g37840.1	150	-26.32	150	-26.32	440642	20	1336	287
miR3979-3p	LOC_Os07g37920.1	155	-27.87	155	-27.87	440650	20	2325	1849
miR3979-3p	LOC_Os07g38420.1	152	-22.44	152	-22.44	440709	20	2114	1424
miR3979-3p	LOC_Os07g38500.1	150	-22.75	150	-22.75	440717	20	5796	1333
miR3979-3p	LOC_Os07g38510.1	152	-26.66	152	-26.66	440718	20	2601	1330
miR3979-3p	LOC_Os07g39510.1	158	-23.36	158	-23.36	440829	20	868	118

miR3979-3p	LOC_Os07g39510.2	158	-23.36	158	-23.36	440830	20	929	118
miR3979-3p	LOC_Os07g39510.3	158	-23.36	158	-23.36	440831	20	929	118
miR3979-3p	LOC_Os07g39630.1	162	-31.57	162	-31.57	440846	20	2549	102
miR3979-3p	LOC_Os07g39700.1	140	-21.31	140	-21.31	440853	20	1032	602
miR3979-3p	LOC_Os07g40290.1	150	-22.68	150	-22.68	440923	20	2337	2174
miR3979-3p	LOC_Os07g40404.1	152	-24.89	152	-24.89	440933	20	2259	2134
miR3979-3p	LOC_Os07g40790.1	154	-24.89	154	-24.89	440977	20	1597	1211
miR3979-3p	LOC_Os07g42400.1	155	-21.68	155	-21.68	441157	20	3228	2431
miR3979-3p	LOC_Os07g42400.2	155	-21.68	155	-21.68	441158	20	3319	2522
miR3979-3p	LOC_Os07g42510.1	152	-20.43	152	-20.43	441172	20	1557	1077
miR3979-3p	LOC_Os07g42670.1	293	-47.49	148	-25.15	441195	20	1065	873 338
miR3979-3p	LOC_Os07g42680.1	164	-29.69	164	-29.69	441196	20	3408	1536
miR3979-3p	LOC_Os07g42940.1	162	-25.46	162	-25.46	441228	20	1696	1642
miR3979-3p	LOC_Os07g42940.5	162	-25.46	162	-25.46	441232	20	1807	1753
miR3979-3p	LOC_Os07g42940.6	162	-25.46	162	-25.46	441233	20	1887	1833
miR3979-3p	LOC_Os07g42940.7	162	-25.46	162	-25.46	441234	20	1770	1724
miR3979-3p	LOC_Os07g42940.9	162	-25.46	162	-25.46	441236	20	1637	1583
miR3979-3p	LOC_Os07g43490.1	140	-21.24	140	-21.24	441299	20	648	447
miR3979-3p	LOC_Os07g43760.1	152	-25.61	152	-25.61	441324	20	2679	1282
miR3979-3p	LOC_Os07g45830.1	156	-21.34	156	-21.34	441547	20	4632	1907
miR3979-3p	LOC_Os07g46180.1	152	-21.31	152	-21.31	441582	20	5321	1357
miR3979-3p	LOC_Os07g46560.1	152	-25	152	-25	441633	20	1596	980
miR3979-3p	LOC_Os07g46640.1	141	-25.16	141	-25.16	441641	20	1959	274
miR3979-3p	LOC_Os07g46640.2	141	-25.16	141	-25.16	441642	20	2057	274
miR3979-3p	LOC_Os07g46700.1	151	-21.8	151	-21.8	441650	20	1429	1318
miR3979-3p	LOC_Os07g46700.2	151	-21.8	151	-21.8	441651	20	1486	1375
miR3979-3p	LOC_Os07g46700.3	151	-21.8	151	-21.8	441652	20	1751	1640
miR3979-3p	LOC_Os07g46700.4	151	-21.8	151	-21.8	441653	20	3522	3411
miR3979-3p	LOC_Os07g47010.1	163	-26.71	163	-26.71	441699	20	2337	388
miR3979-3p	LOC_Os07g47030.1	173	-28.99	173	-28.99	441701	20	2260	2226
miR3979-3p	LOC_Os07g47030.3	173	-28.99	173	-28.99	441703	20	2330	2296
miR3979-3p	LOC_Os07g47030.4	173	-28.99	173	-28.99	441704	20	2403	2339

miR3979-3p	LOC_Os07g48080.1	161	-31.36	161	-31.36	441851	20	1966	296
miR3979-3p	LOC_Os07g48180.1	148	-22.29	148	-22.29	441864	20	1168	735
miR3979-3p	LOC_Os07g48670.1	157	-27.76	157	-27.76	441930	20	1002	507
miR3979-3p	LOC_Os07g49100.1	145	-22.24	145	-22.24	441986	20	1988	901
miR3979-3p	LOC_Os07g49370.1	145	-22.7	145	-22.7	442019	20	1647	853
miR3979-3p	LOC_Os07g49430.1	145	-25.15	145	-25.15	442031	20	3543	752
miR3979-3p	LOC_Os08g01200.1	150	-24.85	150	-24.85	442070	20	1080	1001
miR3979-3p	LOC_Os08g02190.1	157	-24.37	157	-24.37	442193	20	2013	1585
miR3979-3p	LOC_Os08g02550.1	140	-20.64	140	-20.64	442232	20	1794	1104
miR3979-3p	LOC_Os08g02850.1	296	-48.07	153	-24.14	442266	20	2270	1956 1652
miR3979-3p	LOC_Os08g03270.1	147	-22.33	147	-22.33	442311	20	2115	138
miR3979-3p	LOC_Os08g03990.1	148	-26.72	148	-26.72	442402	20	1706	78
miR3979-3p	LOC_Os08g04800.1	148	-23.69	148	-23.69	442495	20	1746	833
miR3979-3p	LOC_Os08g04900.1	144	-24.13	144	-24.13	442507	20	213	68
miR3979-3p	LOC_Os08g05140.1	158	-23.56	158	-23.56	442539	20	978	471
miR3979-3p	LOC_Os08g05550.1	156	-28.33	156	-28.33	442580	20	1241	510
miR3979-3p	LOC_Os08g05840.1	149	-22.08	149	-22.08	442612	20	3448	1906
miR3979-3p	LOC_Os08g06240.1	162	-30.07	162	-30.07	442656	20	1550	462
miR3979-3p	LOC_Os08g06344.1	155	-29.98	155	-29.98	442672	20	4796	4261
miR3979-3p	LOC_Os08g06344.2	155	-29.98	155	-29.98	442673	20	4487	3996
miR3979-3p	LOC_Os08g06344.3	155	-29.98	155	-29.98	442674	20	4655	4164
miR3979-3p	LOC_Os08g06580.1	152	-26.66	152	-26.66	442707	20	3078	1324
miR3979-3p	LOC_Os08g08140.1	150	-21.44	150	-21.44	442863	20	3297	1314
miR3979-3p	LOC_Os08g09340.1	148	-22.33	148	-22.33	442998	20	1913	1
miR3979-3p	LOC_Os08g09340.2	148	-22.33	148	-22.33	442999	20	2000	1
miR3979-3p	LOC_Os08g09790.1	145	-21.75	145	-21.75	443046	20	1767	7
miR3979-3p	LOC_Os08g10670.1	162	-24.64	162	-24.64	443150	20	1927	805
miR3979-3p	LOC_Os08g10670.2	162	-24.64	162	-24.64	443151	20	1770	678
miR3979-3p	LOC_Os08g11070.1	149	-22.5	149	-22.5	443190	20	1113	846
miR3979-3p	LOC_Os08g11150.1	147	-20.19	147	-20.19	443198	20	4680	3663
miR3979-3p	LOC_Os08g11180.1	155	-21.73	155	-21.73	443201	20	2760	2546
miR3979-3p	LOC_Os08g11440.1	151	-22.64	151	-22.64	443234	20	930	810

miR3979-3p	LOC_Os08g11960.1	156	-21.34	156	-21.34	443285	20	5238	1898
miR3979-3p	LOC_Os08g12190.1	146	-20.1	146	-20.1	443307	20	2386	697
miR3979-3p	LOC_Os08g12370.1	145	-20.47	145	-20.47	443323	20	4518	402
miR3979-3p	LOC_Os08g12560.1	142	-22.31	142	-22.31	443343	20	1638	492
miR3979-3p	LOC_Os08g13960.1	152	-24.66	152	-24.66	443499	20	3534	2785
miR3979-3p	LOC_Os08g16190.1	144	-23.01	144	-23.01	443747	20	3348	257
miR3979-3p	LOC_Os08g16610.1	151	-25.23	151	-25.23	443795	20	2856	1528
miR3979-3p	LOC_Os08g16660.1	150	-22.57	150	-22.57	443799	20	2077	595
miR3979-3p	LOC_Os08g17080.1	151	-24.67	151	-24.67	443844	20	2207	213
miR3979-3p	LOC_Os08g17740.1	160	-27.69	160	-27.69	443907	20	1749	125
miR3979-3p	LOC_Os08g19300.1	144	-22.91	144	-22.91	444020	20	381	341
miR3979-3p	LOC_Os08g20180.1	157	-20.6	157	-20.6	444116	20	2640	264
miR3979-3p	LOC_Os08g20370.1	146	-20.1	146	-20.1	444136	20	5535	2634
miR3979-3p	LOC_Os08g21280.1	141	-20.91	141	-20.91	444206	20	3750	194
miR3979-3p	LOC_Os08g21740.1	151	-26.27	151	-26.27	444250	20	1107	58
miR3979-3p	LOC_Os08g22310.1	140	-22.57	140	-22.57	444308	20	4320	282
miR3979-3p	LOC_Os08g22320.1	147	-20.5	147	-20.5	444309	20	2376	2340
miR3979-3p	LOC_Os08g22790.1	145	-21.75	145	-21.75	444350	20	2831	130
miR3979-3p	LOC_Os08g23400.1	156	-21.34	156	-21.34	444412	20	4452	1025
miR3979-3p	LOC_Os08g23630.1	150	-22.75	150	-22.75	444437	20	4875	1333
miR3979-3p	LOC_Os08g23829.1	150	-23.12	150	-23.12	444461	20	528	182
miR3979-3p	LOC_Os08g24400.1	158	-29.72	158	-29.72	444521	20	1935	127
miR3979-3p	LOC_Os08g27250.1	155	-26.53	155	-26.53	444812	20	1179	247
miR3979-3p	LOC_Os08g27360.1	164	-29.07	164	-29.07	444823	20	2496	535
miR3979-3p	LOC_Os08g27520.1	144	-30.26	144	-30.26	444837	20	1464	689
miR3979-3p	LOC_Os08g27540.3	160	-25.44	160	-25.44	444841	20	1913	216
miR3979-3p	LOC_Os08g27590.1	153	-24.08	153	-24.08	444849	20	1668	985
miR3979-3p	LOC_Os08g27630.1	159	-25.37	159	-25.37	444853	20	1473	897
miR3979-3p	LOC_Os08g29030.1	166	-27.4	166	-27.4	444998	20	2496	1126
miR3979-3p	LOC_Os08g29340.1	152	-25.49	152	-25.49	445029	20	1434	216
miR3979-3p	LOC_Os08g29809.1	161	-23.69	161	-23.69	445079	20	3204	1746
miR3979-3p	LOC_Os08g30010.1	146	-20.1	146	-20.1	445095	20	5409	2508

miR3979-3p	LOC_Os08g30014.1	165	-22.8	165	-22.8	445096	20	671	328
miR3979-3p	LOC_Os08g30680.1	152	-21.28	152	-21.28	445168	20	4860	4378
miR3979-3p	LOC_Os08g31350.1	165	-29.36	165	-29.36	445234	20	2055	1198
miR3979-3p	LOC_Os08g31470.1	156	-22.52	156	-22.52	445252	20	2630	1132
miR3979-3p	LOC_Os08g31470.2	156	-22.52	156	-22.52	445253	20	2524	1026
miR3979-3p	LOC_Os08g31470.3	156	-22.52	156	-22.52	445254	20	3435	1937
miR3979-3p	LOC_Os08g31470.4	156	-22.52	156	-22.52	445255	20	2706	1208
miR3979-3p	LOC_Os08g33390.1	165	-26.4	165	-26.4	445470	20	1761	1547
miR3979-3p	LOC_Os08g35520.1	148	-21.63	148	-21.63	445713	20	1998	1581
miR3979-3p	LOC_Os08g35620.1	158	-25.55	158	-25.55	445723	20	2965	6
miR3979-3p	LOC_Os08g35620.2	158	-25.55	158	-25.55	445724	20	2949	6
miR3979-3p	LOC_Os08g36420.1	161	-26.75	161	-26.75	445812	20	2750	1899
miR3979-3p	LOC_Os08g36420.2	161	-26.75	161	-26.75	445813	20	2828	1977
miR3979-3p	LOC_Os08g36420.3	161	-26.75	161	-26.75	445814	20	1993	1142
miR3979-3p	LOC_Os08g36420.4	161	-26.75	161	-26.75	445815	20	2415	1142
miR3979-3p	LOC_Os08g36420.5	161	-26.75	161	-26.75	445816	20	2070	1219
miR3979-3p	LOC_Os08g36450.1	143	-21.37	143	-21.37	445819	20	1692	51
miR3979-3p	LOC_Os08g37940.1	152	-21.75	152	-21.75	445994	20	1489	1152
miR3979-3p	LOC_Os08g38200.1	156	-20.56	156	-20.56	446028	20	2629	1773
miR3979-3p	LOC_Os08g38990.1	169	-27.14	169	-27.14	446128	20	2424	85
miR3979-3p	LOC_Os08g38990.2	169	-27.14	169	-27.14	446129	20	2608	65
miR3979-3p	LOC_Os08g38990.3	169	-27.14	169	-27.14	446130	20	2502	85
miR3979-3p	LOC_Os08g38990.4	169	-27.14	169	-27.14	446131	20	2659	85
miR3979-3p	LOC_Os08g39170.1	148	-21.26	148	-21.26	446153	20	1854	1675
miR3979-3p	LOC_Os08g39240.1	156	-24.59	156	-24.59	446161	20	2653	789
miR3979-3p	LOC_Os08g39240.2	156	-24.59	156	-24.59	446162	20	4088	789
miR3979-3p	LOC_Os08g39660.1	157	-22.83	157	-22.83	446211	20	1668	312
miR3979-3p	LOC_Os08g39760.1	150	-24.14	150	-24.14	446222	20	5856	3588
miR3979-3p	LOC_Os08g40600.1	159	-24.37	159	-24.37	446322	20	1152	486
miR3979-3p	LOC_Os08g40860.1	164	-25.97	164	-25.97	446358	20	2139	1685
miR3979-3p	LOC_Os08g40960.1	146	-22.91	146	-22.91	446370	20	4605	3014
miR3979-3p	LOC_Os08g40980.1	152	-26.66	152	-26.66	446372	20	3054	1471

miR3979-3p	LOC_Os08g44470.1	162	-21.15	162	-21.15	446799	20	871	251
miR3979-3p	LOC_Os08g44570.1	142	-21.5	142	-21.5	446812	20	6093	2307
miR3979-3p	LOC_Os08g44820.5	158	-20.81	158	-20.81	446845	20	2648	2553
miR3979-3p	LOC_Os08g45080.1	143	-24.15	143	-24.15	446882	20	4445	472
miR3979-3p	LOC_Os09g01020.1	146	-29.22	146	-29.22	446917	20	1491	398
miR3979-3p	LOC_Os09g01730.1	144	-22.91	144	-22.91	446998	20	450	410
miR3979-3p	LOC_Os09g02020.1	141	-22.13	141	-22.13	447026	20	5262	3804
miR3979-3p	LOC_Os09g02060.1	166	-33.11	166	-33.11	447030	20	570	343
miR3979-3p	LOC_Os09g02630.1	143	-20.35	143	-20.35	447090	20	2499	175
miR3979-3p	LOC_Os09g03420.1	146	-22.35	146	-22.35	447169	20	5215	2494
miR3979-3p	LOC_Os09g03450.1	160	-28.24	160	-28.24	447172	20	1407	375
miR3979-3p	LOC_Os09g03650.1	145	-25.15	145	-25.15	447195	20	3993	89
miR3979-3p	LOC_Os09g03900.1	150	-23.54	150	-23.54	447216	20	2418	4
miR3979-3p	LOC_Os09g04070.1	157	-28.72	157	-28.72	447231	20	6015	3752
miR3979-3p	LOC_Os09g04650.1	144	-22.91	144	-22.91	447296	20	264	224
miR3979-3p	LOC_Os09g04890.1	146	-20.98	146	-20.98	447323	20	3601	242
miR3979-3p	LOC_Os09g04990.1	162	-25.44	162	-25.44	447333	20	2656	638
miR3979-3p	LOC_Os09g06464.1	152	-25.83	152	-25.83	447382	20	1527	840
miR3979-3p	LOC_Os09g06540.1	145	-25.15	145	-25.15	447388	20	1725	263
miR3979-3p	LOC_Os09g07540.1	146	-22.09	146	-22.09	447499	20	2370	2302
miR3979-3p	LOC_Os09g08020.1	154	-23.05	154	-23.05	447554	20	4923	2859
miR3979-3p	LOC_Os09g08900.1	295	-43.92	149	-23.82	447644	20	5411	5342 2433
miR3979-3p	LOC_Os09g10220.1	164	-29.07	164	-29.07	447778	20	4605	709
miR3979-3p	LOC_Os09g10860.1	153	-26.47	153	-26.47	447844	20	303	193
miR3979-3p	LOC_Os09g11580.1	152	-21.62	152	-21.62	447923	20	4764	4600
miR3979-3p	LOC_Os09g12090.1	157	-21.33	157	-21.33	447972	20	2889	2270
miR3979-3p	LOC_Os09g12720.1	147	-20.69	147	-20.69	448038	20	2246	787
miR3979-3p	LOC_Os09g12720.2	147	-20.69	147	-20.69	448039	20	1873	787
miR3979-3p	LOC_Os09g14610.3	149	-22.84	149	-22.84	448237	20	3306	2932
miR3979-3p	LOC_Os09g15270.1	145	-25.15	145	-25.15	448307	20	4674	437
miR3979-3p	LOC_Os09g15430.1	151	-21.76	151	-21.76	448329	20	2004	83
miR3979-3p	LOC_Os09g15430.2	151	-21.76	151	-21.76	448330	20	2008	83

miR3979-3p	LOC_Os09g15430.3	151	-21.76	151	-21.76	448331	20	1999	83
miR3979-3p	LOC_Os09g15430.4	151	-21.76	151	-21.76	448332	20	1832	83
miR3979-3p	LOC_Os09g16350.1	146	-20.1	146	-20.1	448427	20	3291	1665
miR3979-3p	LOC_Os09g16920.1	144	-23.15	144	-23.15	448487	20	2140	487
miR3979-3p	LOC_Os09g17170.1	149	-26.33	149	-26.33	448514	20	1446	118
miR3979-3p	LOC_Os09g17450.1	146	-20.1	146	-20.1	448540	20	5334	2433
miR3979-3p	LOC_Os09g17570.1	152	-25.02	152	-25.02	448552	20	849	632
miR3979-3p	LOC_Os09g19900.1	163	-29.89	163	-29.89	448732	20	1593	1119
miR3979-3p	LOC_Os09g20110.1	159	-24.58	159	-24.58	448758	20	660	343
miR3979-3p	LOC_Os09g21350.1	164	-31.02	164	-31.02	448897	20	1605	1495
miR3979-3p	LOC_Os09g21460.1	145	-27.16	145	-27.16	448907	20	751	375
miR3979-3p	LOC_Os09g26390.1	162	-25.32	162	-25.32	449382	20	3659	251
miR3979-3p	LOC_Os09g26390.2	162	-25.32	162	-25.32	449383	20	4010	196
miR3979-3p	LOC_Os09g26390.3	162	-25.32	162	-25.32	449384	20	4062	251
miR3979-3p	LOC_Os09g27430.1	143	-20.09	143	-20.09	449507	20	774	296
miR3979-3p	LOC_Os09g27470.1	141	-20.66	141	-20.66	449511	20	573	239
miR3979-3p	LOC_Os09g27500.1	154	-23.08	154	-23.08	449514	20	2036	656
miR3979-3p	LOC_Os09g27810.1	148	-22.66	148	-22.66	449564	20	2460	1211
miR3979-3p	LOC_Os09g28560.1	151	-21.69	151	-21.69	449649	20	1778	1741
miR3979-3p	LOC_Os09g28890.1	156	-22.5	156	-22.5	449687	20	1663	23
miR3979-3p	LOC_Os09g28980.1	145	-23.8	145	-23.8	449701	20	537	379
miR3979-3p	LOC_Os09g30494.1	146	-20.1	146	-20.1	449890	20	4713	2412
miR3979-3p	LOC_Os09g31970.1	141	-22.65	141	-22.65	449992	20	2577	549
miR3979-3p	LOC_Os09g33450.1	161	-30.86	161	-30.86	450136	20	2086	1879
miR3979-3p	LOC_Os09g33450.2	161	-30.86	161	-30.86	450137	20	2132	1925
miR3979-3p	LOC_Os09g33610.1	157	-27.76	157	-27.76	450158	20	5946	1224
miR3979-3p	LOC_Os09g35590.1	153	-22.37	153	-22.37	450298	20	411	200
miR3979-3p	LOC_Os09g36320.1	140	-21.04	140	-21.04	450395	20	1818	774
miR3979-3p	LOC_Os09g36650.1	161	-22.64	161	-22.64	450433	20	1884	1786
miR3979-3p	LOC_Os09g36810.1	148	-25.16	148	-25.16	450452	20	2736	2253
miR3979-3p	LOC_Os09g37300.1	169	-24.93	169	-24.93	450509	20	2809	25
miR3979-3p	LOC_Os09g37860.1	147	-25.08	147	-25.08	450573	20	2578	158

miR3979-3p	LOC_Os09g38268.1	148	-23.55	148	-23.55	450612	20	1395	437
miR3979-3p	LOC_Os09g38500.1	152	-24.3	152	-24.3	450637	20	1113	867
miR3979-3p	LOC_Os09g38500.2	152	-24.3	152	-24.3	450638	20	1302	1056
miR3979-3p	LOC_Os09g38500.3	152	-24.3	152	-24.3	450639	20	1190	944
miR3979-3p	LOC_Os09g39270.1	145	-21.86	145	-21.86	450734	20	4198	356
miR3979-3p	LOC_Os09g39820.1	145	-25.15	145	-25.15	450806	20	4734	689
miR3979-3p	LOC_Os10g02080.1	146	-24.43	146	-24.43	450946	20	1551	1465
miR3979-3p	LOC_Os10g02440.1	145	-24.02	145	-24.02	450986	20	1374	379
miR3979-3p	LOC_Os10g02850.1	142	-21.24	142	-21.24	451030	20	2943	504
miR3979-3p	LOC_Os10g04220.1	145	-22.16	145	-22.16	451168	20	1971	578
miR3979-3p	LOC_Os10g04710.1	151	-21.25	151	-21.25	451217	20	519	426
miR3979-3p	LOC_Os10g05329.1	144	-22.91	144	-22.91	451279	20	384	344
miR3979-3p	LOC_Os10g05380.1	154	-27.64	154	-27.64	451283	20	4224	2013
miR3979-3p	LOC_Os10g05540.1	145	-20.61	145	-20.61	451301	20	1509	866
miR3979-3p	LOC_Os10g05690.1	145	-21.99	145	-21.99	451315	20	2129	1207
miR3979-3p	LOC_Os10g06270.1	146	-20.1	146	-20.1	451375	20	5160	2406
miR3979-3p	LOC_Os10g07004.1	143	-21.18	143	-21.18	451444	20	1992	150
miR3979-3p	LOC_Os10g07998.1	156	-23.39	156	-23.39	451531	20	1372	851
miR3979-3p	LOC_Os10g08540.1	155	-20.33	155	-20.33	451586	20	2968	2859
miR3979-3p	LOC_Os10g08820.1	146	-20.1	146	-20.1	451617	20	4341	1665
miR3979-3p	LOC_Os10g09590.1	145	-21.86	145	-21.86	451687	20	486	178
miR3979-3p	LOC_Os10g09610.1	156	-26.09	156	-26.09	451689	20	5562	2493
miR3979-3p	LOC_Os10g10460.1	146	-20.1	146	-20.1	451782	20	5460	2559
miR3979-3p	LOC_Os10g12740.1	155	-31.88	155	-31.88	451987	20	4428	2993
miR3979-3p	LOC_Os10g13730.1	157	-25.96	157	-25.96	452044	20	1323	935
miR3979-3p	LOC_Os10g14860.1	149	-26.33	149	-26.33	452110	20	1566	163
miR3979-3p	LOC_Os10g17820.1	146	-20.1	146	-20.1	452302	20	5358	2457
miR3979-3p	LOC_Os10g18560.1	151	-23.87	151	-23.87	452367	20	2970	2690
miR3979-3p	LOC_Os10g18740.1	148	-21.38	148	-21.38	452384	20	2811	112
miR3979-3p	LOC_Os10g22720.1	151	-25.07	151	-25.07	452788	20	945	6
miR3979-3p	LOC_Os10g23120.1	141	-21.57	141	-21.57	452835	20	2299	1483
miR3979-3p	LOC_Os10g25060.1	148	-20.42	148	-20.42	452981	20	1443	1093

miR3979-3p	LOC_Os10g25502.1	145	-25.15	145	-25.15	453022	20	1629	116
miR3979-3p	LOC_Os10g25720.1	146	-20.1	146	-20.1	453048	20	3738	1080
miR3979-3p	LOC_Os10g26110.1	141	-22.14	141	-22.14	453088	20	1548	1350
miR3979-3p	LOC_Os10g26280.1	154	-26.03	154	-26.03	453108	20	2560	2235
miR3979-3p	LOC_Os10g27310.1	152	-23.67	152	-23.67	453216	20	2158	2054
miR3979-3p	LOC_Os10g29180.1	169	-30.46	169	-30.46	453364	20	2656	27
miR3979-3p	LOC_Os10g29180.2	169	-30.46	169	-30.46	453365	20	1413	27
miR3979-3p	LOC_Os10g30380.1	156	-23.74	156	-23.74	453486	20	1569	612
miR3979-3p	LOC_Os10g30460.1	156	-21.54	156	-21.54	453493	20	1077	182
miR3979-3p	LOC_Os10g31000.1	145	-24.29	145	-24.29	453549	20	1170	694
miR3979-3p	LOC_Os10g31000.2	145	-24.29	145	-24.29	453550	20	3505	3077
miR3979-3p	LOC_Os10g33310.1	162	-27.65	162	-27.65	453777	20	1251	815
miR3979-3p	LOC_Os10g33440.2	141	-21.78	141	-21.78	453789	20	5941	3351
miR3979-3p	LOC_Os10g34810.1	154	-21.15	154	-21.15	453947	20	1507	983
miR3979-3p	LOC_Os10g35030.1	168	-29.79	168	-29.79	453969	20	2461	1969
miR3979-3p	LOC_Os10g35030.2	168	-29.79	168	-29.79	453970	20	2451	1959
miR3979-3p	LOC_Os10g35030.3	168	-29.79	168	-29.79	453971	20	2428	1936
miR3979-3p	LOC_Os10g36229.1	140	-20.94	140	-20.94	454115	20	3729	1530
miR3979-3p	LOC_Os10g36703.1	140	-23.85	140	-23.85	454166	20	1019	587
miR3979-3p	LOC_Os10g36848.1	157	-25.07	157	-25.07	454181	20	2132	992
miR3979-3p	LOC_Os10g36870.1	152	-20.51	152	-20.51	454183	20	1771	1399
miR3979-3p	LOC_Os10g37260.1	154	-20.69	154	-20.69	454215	20	2673	1850
miR3979-3p	LOC_Os10g38120.1	156	-22.68	156	-22.68	454311	20	1802	1342
miR3979-3p	LOC_Os10g38800.1	140	-21.04	140	-21.04	454372	20	2671	529
miR3979-3p	LOC_Os10g39030.1	153	-21.31	153	-21.31	454400	20	2604	2029
miR3979-3p	LOC_Os10g39100.1	147	-22.16	147	-22.16	454408	20	1358	577
miR3979-3p	LOC_Os10g39430.1	149	-21.76	149	-21.76	454446	20	2891	1529
miR3979-3p	LOC_Os10g39730.1	154	-24.89	154	-24.89	454488	20	999	525
miR3979-3p	LOC_Os10g40824.1	157	-20.33	157	-20.33	454624	20	2019	1684
miR3979-3p	LOC_Os10g40830.1	157	-20.33	157	-20.33	454625	20	2250	162
miR3979-3p	LOC_Os10g41490.1	142	-22.74	142	-22.74	454702	20	1520	1285
miR3979-3p	LOC_Os10g41490.2	142	-22.74	142	-22.74	454703	20	1495	1260

miR3979-3p	LOC_Os10g42090.1	143	-20.29	143	-20.29	454779	20	914	223
miR3979-3p	LOC_Os10g42090.2	143	-20.29	143	-20.29	454780	20	1846	223
miR3979-3p	LOC_Os11g02774.1	161	-24.88	161	-24.88	455121	20	520	344
miR3979-3p	LOC_Os11g03200.1	145	-22.24	145	-22.24	455165	20	1222	1191
miR3979-3p	LOC_Os11g03260.1	146	-23.29	146	-23.29	455173	20	642	369
miR3979-3p	LOC_Os11g03360.1	140	-20.42	140	-20.42	455183	20	1563	346
miR3979-3p	LOC_Os11g03570.1	154	-26.44	154	-26.44	455209	20	2516	2309
miR3979-3p	LOC_Os11g03830.1	161	-26.6	161	-26.6	455238	20	3417	2967
miR3979-3p	LOC_Os11g03840.1	174	-28.98	174	-28.98	455239	20	2794	642
miR3979-3p	LOC_Os11g05130.1	157	-23.85	157	-23.85	455399	20	6552	6170
miR3979-3p	LOC_Os11g05300.1	155	-21.7	155	-21.7	455419	20	1105	978
miR3979-3p	LOC_Os11g05600.1	152	-22.86	152	-22.86	455455	20	516	14
miR3979-3p	LOC_Os11g06220.1	146	-20.37	146	-20.37	455526	20	2207	605
miR3979-3p	LOC_Os11g06220.2	146	-20.37	146	-20.37	455527	20	3284	1682
miR3979-3p	LOC_Os11g06234.1	146	-20.37	146	-20.37	455529	20	1796	264
miR3979-3p	LOC_Os11g06320.1	153	-26.58	153	-26.58	455538	20	2681	2430
miR3979-3p	LOC_Os11g06970.1	148	-22.25	148	-22.25	455619	20	459	78
miR3979-3p	LOC_Os11g07790.1	146	-20.1	146	-20.1	455719	20	5275	2416
miR3979-3p	LOC_Os11g08340.1	152	-20.65	152	-20.65	455784	20	1842	810
miR3979-3p	LOC_Os11g10490.1	150	-21.8	150	-21.8	456010	20	2232	2089
miR3979-3p	LOC_Os11g10610.1	141	-21.46	141	-21.46	456026	20	3543	2129
miR3979-3p	LOC_Os11g10900.1	146	-20.1	146	-20.1	456051	20	5334	2433
miR3979-3p	LOC_Os11g11490.1	140	-21.04	140	-21.04	456114	20	4172	1604
miR3979-3p	LOC_Os11g12340.1	149	-23.46	149	-23.46	456199	20	3426	1632
miR3979-3p	LOC_Os11g12340.2	149	-23.46	149	-23.46	456200	20	3390	1603
miR3979-3p	LOC_Os11g12840.1	143	-20.08	143	-20.08	456250	20	1611	1568
miR3979-3p	LOC_Os11g13890.1	144	-21.38	144	-21.38	456305	20	1305	225
miR3979-3p	LOC_Os11g13890.2	144	-21.38	144	-21.38	456306	20	1737	225
miR3979-3p	LOC_Os11g13890.4	144	-21.38	144	-21.38	456307	20	1300	225
miR3979-3p	LOC_Os11g13890.5	144	-21.38	144	-21.38	456308	20	959	225
miR3979-3p	LOC_Os11g13890.6	144	-21.38	144	-21.38	456309	20	1949	225
miR3979-3p	LOC_Os11g14130.1	287	-42.14	144	-21.41	456335	20	2024	566 297

miR3979-3p	LOC_Os11g14850.1	148	-25.42	148	-25.42	456417	20	927	481
miR3979-3p	LOC_Os11g15400.1	157	-21.33	157	-21.33	456476	20	3513	3035
miR3979-3p	LOC_Os11g15430.1	160	-22.79	160	-22.79	456479	20	2211	821
miR3979-3p	LOC_Os11g16924.1	147	-20.29	147	-20.29	456597	20	2619	1158
miR3979-3p	LOC_Os11g16924.2	147	-20.29	147	-20.29	456598	20	2619	1158
miR3979-3p	LOC_Os11g17440.1	151	-25.03	151	-25.03	456646	20	3108	2317
miR3979-3p	LOC_Os11g22050.1	142	-21.51	142	-21.51	457000	20	291	224
miR3979-3p	LOC_Os11g22220.1	145	-20.91	145	-20.91	457020	20	3330	402
miR3979-3p	LOC_Os11g23890.1	145	-22.76	145	-22.76	457151	20	1959	689
miR3979-3p	LOC_Os11g24520.1	155	-21.87	155	-21.87	457209	20	327	98
miR3979-3p	LOC_Os11g25970.1	159	-23.16	159	-23.16	457343	20	501	22
miR3979-3p	LOC_Os11g26700.1	147	-22.09	147	-22.09	457413	20	744	212
miR3979-3p	LOC_Os11g27410.1	146	-20.1	146	-20.1	457482	20	5064	2301
miR3979-3p	LOC_Os11g28050.1	160	-27.69	160	-27.69	457545	20	1992	902
miR3979-3p	LOC_Os11g29380.1	160	-27.95	160	-27.95	457681	20	3216	33
miR3979-3p	LOC_Os11g29880.1	143	-22.57	143	-22.57	457734	20	4284	256
miR3979-3p	LOC_Os11g30310.1	141	-21.04	141	-21.04	457777	20	1623	883
miR3979-3p	LOC_Os11g30540.1	152	-26.66	152	-26.66	457798	20	2733	1081
miR3979-3p	LOC_Os11g31320.1	150	-22.28	150	-22.28	457865	20	825	264
miR3979-3p	LOC_Os11g31740.1	143	-20.75	143	-20.75	457914	20	1386	243
miR3979-3p	LOC_Os11g31760.1	160	-27.87	160	-27.87	457916	20	4858	1561
miR3979-3p	LOC_Os11g32950.1	161	-28.1	161	-28.1	458050	20	882	432
miR3979-3p	LOC_Os11g33370.1	145	-21.76	145	-21.76	458093	20	762	80
miR3979-3p	LOC_Os11g34050.1	146	-21.54	146	-21.54	458117	20	2784	2740
miR3979-3p	LOC_Os11g34240.1	145	-25.08	145	-25.08	458145	20	834	190
miR3979-3p	LOC_Os11g34420.1	152	-26.66	152	-26.66	458167	20	2505	1468
miR3979-3p	LOC_Os11g34520.1	152	-26.66	152	-26.66	458179	20	1821	1348
miR3979-3p	LOC_Os11g34880.1	145	-21.75	145	-21.75	458214	20	2822	2288
miR3979-3p	LOC_Os11g35870.3	152	-24.38	152	-24.38	458320	20	2896	1890
miR3979-3p	LOC_Os11g36220.1	143	-22.22	143	-22.22	458364	20	5007	4557
miR3979-3p	LOC_Os11g36450.1	145	-23.34	145	-23.34	458389	20	2029	869
miR3979-3p	LOC_Os11g37660.1	144	-23.33	144	-23.33	458505	20	2978	2482

miR3979-3p	LOC_Os11g39260.1	152	-20.71	152	-20.71	458687	20	723	214
miR3979-3p	LOC_Os11g40220.1	158	-25.46	158	-25.46	458786	20	1050	628
miR3979-3p	LOC_Os11g40510.1	155	-22.98	155	-22.98	458819	20	2088	1930
miR3979-3p	LOC_Os11g40510.2	155	-22.98	155	-22.98	458820	20	1974	1816
miR3979-3p	LOC_Os11g40780.1	148	-20.75	148	-20.75	458855	20	3165	2351
miR3979-3p	LOC_Os11g41034.1	157	-27.53	157	-27.53	458878	20	3311	3056
miR3979-3p	LOC_Os11g41380.1	156	-26.2	156	-26.2	458911	20	1968	1789
miR3979-3p	LOC_Os11g42290.1	140	-21.4	140	-21.4	459008	20	1575	1142
miR3979-3p	LOC_Os11g42790.1	158	-22.76	158	-22.76	459062	20	2362	1394
miR3979-3p	LOC_Os11g43150.1	149	-23.72	149	-23.72	459099	20	2178	1218
miR3979-3p	LOC_Os11g43390.1	150	-20.11	150	-20.11	459122	20	2800	520
miR3979-3p	LOC_Os11g43710.1	280	-43.15	140	-22.35	459164	20	495	91 110
miR3979-3p	LOC_Os11g43900.1	163	-24.74	163	-24.74	459183	20	979	873
miR3979-3p	LOC_Os11g43900.2	163	-24.74	163	-24.74	459184	20	1169	1063
miR3979-3p	LOC_Os11g44170.1	145	-23.2	145	-23.2	459210	20	1441	1099
miR3979-3p	LOC_Os11g44920.1	154	-22.84	154	-22.84	459287	20	2011	87
miR3979-3p	LOC_Os11g45290.1	168	-30.63	168	-30.63	459327	20	785	30
miR3979-3p	LOC_Os11g45290.2	168	-30.63	168	-30.63	459328	20	840	30
miR3979-3p	LOC_Os11g45750.1	140	-22.33	140	-22.33	459377	20	4821	2246
miR3979-3p	LOC_Os11g45750.2	140	-22.33	140	-22.33	459378	20	4259	2155
miR3979-3p	LOC_Os11g47110.1	152	-23.06	152	-23.06	459467	20	2214	931
miR3979-3p	LOC_Os11g47451.1	150	-23.48	150	-23.48	459517	20	2469	1420
miR3979-3p	LOC_Os11g47980.1	140	-20.75	140	-20.75	459584	20	1101	270
miR3979-3p	LOC_Os12g01610.1	140	-20.24	140	-20.24	459678	20	627	584
miR3979-3p	LOC_Os12g01880.1	161	-25.4	161	-25.4	459704	20	1630	1015
miR3979-3p	LOC_Os12g01970.1	148	-22.95	148	-22.95	459721	20	849	294
miR3979-3p	LOC_Os12g02750.1	161	-24.88	161	-24.88	459821	20	1677	840
miR3979-3p	LOC_Os12g02750.2	161	-24.88	161	-24.88	459822	20	1178	865
miR3979-3p	LOC_Os12g02750.3	161	-24.88	161	-24.88	459823	20	1217	840
miR3979-3p	LOC_Os12g02750.4	161	-24.88	161	-24.88	459824	20	1221	865
miR3979-3p	LOC_Os12g03340.1	154	-26.44	154	-26.44	459893	20	2551	2297
miR3979-3p	LOC_Os12g03630.1	147	-22.05	147	-22.05	459924	20	1641	1371

miR3979-3p	LOC_Os12g03650.1	174	-28.98	174	-28.98	459926	20	2508	374
miR3979-3p	LOC_Os12g03670.1	170	-26.77	170	-26.77	459928	20	2511	374
miR3979-3p	LOC_Os12g04800.1	147	-25.81	147	-25.81	460067	20	1038	465
miR3979-3p	LOC_Os12g04810.1	146	-20.1	146	-20.1	460068	20	4951	2395
miR3979-3p	LOC_Os12g04840.1	149	-24.61	149	-24.61	460071	20	4419	3591
miR3979-3p	LOC_Os12g04970.1	148	-21.5	148	-21.5	460089	20	885	748
miR3979-3p	LOC_Os12g05120.1	143	-22.77	143	-22.77	460110	20	2706	1397
miR3979-3p	LOC_Os12g05120.2	143	-22.77	143	-22.77	460111	20	2799	1400
miR3979-3p	LOC_Os12g05529.1	153	-20.68	153	-20.68	460152	20	636	266
miR3979-3p	LOC_Os12g06180.1	140	-21.93	140	-21.93	460221	20	877	775
miR3979-3p	LOC_Os12g06560.1	143	-22.44	143	-22.44	460265	20	3392	3007
miR3979-3p	LOC_Os12g06690.1	153	-22.82	153	-22.82	460281	20	2301	464
miR3979-3p	LOC_Os12g06850.1	154	-21.11	154	-21.11	460301	20	1967	1374
miR3979-3p	LOC_Os12g06850.2	154	-21.11	154	-21.11	460302	20	2143	1550
miR3979-3p	LOC_Os12g06850.3	154	-21.11	154	-21.11	460303	20	2083	1490
miR3979-3p	LOC_Os12g06850.4	154	-21.11	154	-21.11	460304	20	1545	952
miR3979-3p	LOC_Os12g07880.1	146	-23.09	146	-23.09	460414	20	2612	1702
miR3979-3p	LOC_Os12g09089.1	148	-24.59	148	-24.59	460548	20	1764	968
miR3979-3p	LOC_Os12g09089.2	148	-24.59	148	-24.59	460549	20	5101	4305
miR3979-3p	LOC_Os12g09610.1	154	-29.42	154	-29.42	460604	20	1248	8
miR3979-3p	LOC_Os12g09850.1	148	-23.83	148	-23.83	460626	20	2781	1384
miR3979-3p	LOC_Os12g10010.1	152	-21.5	152	-21.5	460640	20	714	52
miR3979-3p	LOC_Os12g10650.1	152	-20.04	152	-20.04	460713	20	989	578
miR3979-3p	LOC_Os12g12130.1	151	-24.02	151	-24.02	460857	20	3329	751
miR3979-3p	LOC_Os12g12800.1	145	-21.75	145	-21.75	460929	20	2400	1446
miR3979-3p	LOC_Os12g13340.1	152	-21.44	152	-21.44	460990	20	3086	2916
miR3979-3p	LOC_Os12g13340.2	152	-21.44	152	-21.44	460991	20	3658	3488
miR3979-3p	LOC_Os12g13440.1	147	-23.14	147	-23.14	461003	20	8268	5264
miR3979-3p	LOC_Os12g13440.2	147	-23.14	147	-23.14	461004	20	8226	5252
miR3979-3p	LOC_Os12g13520.1	141	-20.04	141	-20.04	461012	20	537	158
miR3979-3p	LOC_Os12g14059.1	160	-21.25	160	-21.25	461069	20	2112	473
miR3979-3p	LOC_Os12g14126.1	163	-22.81	163	-22.81	461077	20	4166	48

miR3979-3p	LOC_Os12g15340.1	145	-24.4	145	-24.4	461185	20	5049	1068
miR3979-3p	LOC_Os12g15530.1	148	-26.97	148	-26.97	461206	20	2818	1576
miR3979-3p	LOC_Os12g18340.1	148	-23.8	148	-23.8	461506	20	417	180
miR3979-3p	LOC_Os12g19700.1	156	-21.34	156	-21.34	461641	20	6525	1031
miR3979-3p	LOC_Os12g20290.1	145	-24.84	145	-24.84	461700	20	2295	1160
miR3979-3p	LOC_Os12g21770.1	155	-25.64	155	-25.64	461754	20	1788	1759
miR3979-3p	LOC_Os12g22340.1	160	-31.95	160	-31.95	461816	20	897	129
miR3979-3p	LOC_Os12g23980.1	151	-23.05	151	-23.05	461974	20	2482	1820
miR3979-3p	LOC_Os12g25020.1	160	-27.43	160	-27.43	462070	20	1506	1211
miR3979-3p	LOC_Os12g25070.1	157	-27.51	157	-27.51	462074	20	1752	864
miR3979-3p	LOC_Os12g25670.1	152	-28.63	152	-28.63	462135	20	468	389
miR3979-3p	LOC_Os12g25710.1	143	-21.48	143	-21.48	462141	20	2052	1646
miR3979-3p	LOC_Os12g25710.2	143	-21.48	143	-21.48	462142	20	1835	1646
miR3979-3p	LOC_Os12g25710.3	143	-21.48	143	-21.48	462143	20	1803	1646
miR3979-3p	LOC_Os12g25710.4	143	-21.48	143	-21.48	462144	20	1989	1583
miR3979-3p	LOC_Os12g26780.1	156	-21.34	156	-21.34	462247	20	3681	1013
miR3979-3p	LOC_Os12g27490.1	145	-20.91	145	-20.91	462321	20	4836	402
miR3979-3p	LOC_Os12g27530.1	145	-25.15	145	-25.15	462327	20	1752	665
miR3979-3p	LOC_Os12g28360.1	156	-25.5	156	-25.5	462404	20	6093	4256
miR3979-3p	LOC_Os12g28630.1	158	-27.46	158	-27.46	462430	20	960	774
miR3979-3p	LOC_Os12g28830.1	141	-20.58	141	-20.58	462448	20	2493	1687
miR3979-3p	LOC_Os12g29100.1	146	-20.1	146	-20.1	462475	20	4566	1665
miR3979-3p	LOC_Os12g29520.1	164	-26.8	164	-26.8	462513	20	3622	3309
miR3979-3p	LOC_Os12g29520.2	164	-26.8	164	-26.8	462514	20	3639	3326
miR3979-3p	LOC_Os12g29520.3	164	-26.8	164	-26.8	462515	20	3619	3306
miR3979-3p	LOC_Os12g29520.4	164	-26.8	164	-26.8	462516	20	3619	3306
miR3979-3p	LOC_Os12g29520.5	164	-26.8	164	-26.8	462517	20	3701	3388
miR3979-3p	LOC_Os12g29920.1	156	-27.65	156	-27.65	462559	20	900	657
miR3979-3p	LOC_Os12g30660.1	144	-23.01	144	-23.01	462638	20	849	257
miR3979-3p	LOC_Os12g31990.1	144	-22.91	144	-22.91	462768	20	915	875
miR3979-3p	LOC_Os12g32060.1	148	-22.6	148	-22.6	462775	20	780	666
miR3979-3p	LOC_Os12g32110.1	158	-24.65	158	-24.65	462778	20	2196	1064

miR3979-3p	LOC_Os12g35070.1	160	-26.03	160	-26.03	463103	20	534	450
miR3979-3p	LOC_Os12g36180.1	141	-20.34	141	-20.34	463221	20	3664	3126
miR3979-3p	LOC_Os12g36560.1	152	-26.66	152	-26.66	463256	20	3123	1471
miR3979-3p	LOC_Os12g36580.1	159	-26.23	159	-26.23	463258	20	3196	1548
miR3979-3p	LOC_Os12g36770.2	149	-21.98	149	-21.98	463281	20	2233	1635
miR3979-3p	LOC_Os12g37380.1	152	-25.02	152	-25.02	463345	20	1669	466
miR3979-3p	LOC_Os12g37470.1	144	-22.42	144	-22.42	463358	20	2478	8
miR3979-3p	LOC_Os12g39580.1	154	-27.47	154	-27.47	463611	20	2709	1067
miR3979-3p	LOC_Os12g39990.1	149	-25.73	149	-25.73	463660	20	798	196
miR3979-3p	LOC_Os12g40320.1	152	-23.97	152	-23.97	463695	20	1843	867
miR3979-3p	LOC_Os12g40360.1	169	-32.61	169	-32.61	463699	20	1941	196
miR3979-3p	LOC_Os12g40510.1	166	-27.91	166	-27.91	463717	20	1556	84
miR3979-3p	LOC_Os12g41170.1	154	-21.47	154	-21.47	463791	20	3112	671
miR3979-3p	LOC_Os12g41950.1	152	-20.83	152	-20.83	463891	20	3960	3265
miR3979-3p	LOC_Os12g41962.1	163	-25.7	163	-25.7	463893	20	2828	13
miR3979-3p	LOC_Os12g41962.2	163	-25.7	163	-25.7	463894	20	2750	13
miR3979-3p	LOC_Os12g41962.3	163	-25.7	163	-25.7	463895	20	1467	13
miR3979-3p	LOC_Os12g41962.4	163	-25.7	163	-25.7	463896	20	1377	16
miR3979-3p	LOC_Os12g42680.1	150	-22.75	150	-22.75	463980	20	5274	1330
miR3979-3p	LOC_Os12g42690.1	152	-26.66	152	-26.66	463981	20	2826	1528
miR3979-3p	LOC_Os12g43020.1	140	-22.57	140	-22.57	464025	20	5304	282
miR3979-3p	LOC_Os12g44010.1	148	-20.19	148	-20.19	464127	20	2435	1616
miR3979-3p	ChrUn.fgenesh.mRNA.29	146	-20.37	146	-20.37	464210	20	2769	1281
miR3979-3p	ChrSy.fgenesh.mRNA.7	151	-22.17	151	-22.17	464284	20	2670	859
miR3979-3p	ChrSy.fgenesh.mRNA.34	146	-20.1	146	-20.1	464311	20	3366	564
miR3979-3p	ChrSy.fgenesh.mRNA.56	146	-26.01	146	-26.01	464333	20	1755	1315
miR3979-3p	ChrSy.fgenesh.mRNA.72	150	-22.75	150	-22.75	464349	20	2583	1333
miR3979-3p	ChrSy.fgenesh.mRNA.73	152	-26.66	152	-26.66	464350	20	2013	1312
miR3979-3p	ChrSy.fgenesh.mRNA.77	143	-22.53	143	-22.53	464354	20	4137	256
miR2873c	LOC_Os01g07360.1	157	-20.43	157	-20.43	465102	21	2224	994
miR2873c	LOC_Os01g07360.2	157	-20.43	157	-20.43	465103	21	2030	994
miR2873c	LOC_Os01g07360.3	157	-20.43	157	-20.43	465104	21	1949	994

miR2873c	LOC_Os01g07360.4	157	-20.43	157	-20.43	465105	21	1840	994
miR2873c	LOC_Os01g10490.1	157	-20.2	157	-20.2	465472	21	1216	35
miR2873c	LOC_Os01g12400.2	174	-20.69	174	-20.69	465667	21	2692	1763
miR2873c	LOC_Os01g12410.1	159	-21.32	159	-21.32	465668	21	2289	1359
miR2873c	LOC_Os01g13090.1	147	-21.15	147	-21.15	465761	21	1398	859
miR2873c	LOC_Os01g13090.2	147	-21.15	147	-21.15	465762	21	1402	863
miR2873c	LOC_Os01g13090.3	147	-21.15	147	-21.15	465763	21	1387	848
miR2873c	LOC_Os01g13090.4	147	-21.15	147	-21.15	465764	21	1396	857
miR2873c	LOC_Os01g13200.1	171	-20.97	171	-20.97	465776	21	2015	859
miR2873c	LOC_Os01g16740.1	163	-21.44	163	-21.44	466179	21	3639	2073
miR2873c	LOC_Os01g33370.1	183	-26.34	183	-26.34	467650	21	896	339
miR2873c	LOC_Os01g33740.1	173	-21.93	173	-21.93	467687	21	2799	1003
miR2873c	LOC_Os01g37240.1	163	-21.56	163	-21.56	468053	21	3099	1164
miR2873c	LOC_Os01g39570.1	148	-21.9	148	-21.9	468311	21	3822	3780
miR2873c	LOC_Os01g44200.1	163	-20.56	163	-20.56	468838	21	6264	4104
miR2873c	LOC_Os01g46710.1	168	-21.87	168	-21.87	469057	21	4742	2155
miR2873c	LOC_Os01g51340.1	167	-20.84	167	-20.84	469604	21	1350	330
miR2873c	LOC_Os01g65320.1	163	-22.68	163	-22.68	471311	21	4563	1908
miR2873c	LOC_Os01g68990.1	183	-22.4	183	-22.4	471758	21	2376	1975
miR2873c	LOC_Os02g01450.1	175	-25.64	175	-25.64	472451	21	1263	907
miR2873c	LOC_Os02g02290.1	170	-23.06	170	-23.06	472546	21	7021	28
miR2873c	LOC_Os02g03670.1	170	-21.07	170	-21.07	472731	21	2051	1492
miR2873c	LOC_Os02g03670.3	170	-21.07	170	-21.07	472732	21	2831	2082
miR2873c	LOC_Os02g03670.5	170	-21.07	170	-21.07	472733	21	2358	1609
miR2873c	LOC_Os02g03670.6	170	-21.07	170	-21.07	472734	21	2259	1609
miR2873c	LOC_Os02g03670.7	170	-21.07	170	-21.07	472735	21	2190	1492
miR2873c	LOC_Os02g13120.1	154	-20.22	154	-20.22	473821	21	261	81
miR2873c	LOC_Os02g18170.1	170	-22.46	170	-22.46	474396	21	552	39
miR2873c	LOC_Os02g18840.1	174	-22.02	174	-22.02	474468	21	2692	298
miR2873c	LOC_Os02g20410.1	171	-20.87	171	-20.87	474631	21	3728	1996
miR2873c	LOC_Os02g21400.1	158	-20.24	158	-20.24	474737	21	4965	980
miR2873c	LOC_Os02g24520.1	167	-20.84	167	-20.84	474984	21	1836	819

miR2873c	LOC_Os02g25170.1	167	-20.84	167	-20.84	475057	21	6420	5592
miR2873c	LOC_Os02g39540.1	162	-20.69	162	-20.69	476640	21	3250	2955
miR2873c	LOC_Os02g39540.2	162	-20.69	162	-20.69	476641	21	3246	2951
miR2873c	LOC_Os02g46260.1	163	-20.87	163	-20.87	477431	21	2097	870
miR2873c	LOC_Os02g46260.2	163	-20.87	163	-20.87	477432	21	2102	870
miR2873c	LOC_Os02g46260.3	163	-20.87	163	-20.87	477433	21	2217	870
miR2873c	LOC_Os02g46260.4	163	-20.87	163	-20.87	477434	21	2040	773
miR2873c	LOC_Os02g46260.5	163	-20.87	163	-20.87	477435	21	1978	870
miR2873c	LOC_Os02g46260.6	163	-20.87	163	-20.87	477436	21	1864	870
miR2873c	LOC_Os02g46830.1	166	-21.85	166	-21.85	477504	21	750	134
miR2873c	LOC_Os02g48880.1	174	-24.83	174	-24.83	477772	21	2955	2398
miR2873c	LOC_Os02g48880.2	174	-24.83	174	-24.83	477773	21	2334	1777
miR2873c	LOC_Os02g49900.1	163	-21.56	163	-21.56	477905	21	4764	972
miR2873c	LOC_Os02g53770.1	167	-23.96	167	-23.96	478391	21	3611	3353
miR2873c	LOC_Os02g58710.1	163	-21.56	163	-21.56	479029	21	4191	954
miR2873c	LOC_Os03g03860.1	162	-21.09	162	-21.09	479436	21	3829	2727
miR2873c	LOC_Os03g03860.2	162	-21.09	162	-21.09	479437	21	2953	2460
miR2873c	LOC_Os03g03860.3	162	-21.09	162	-21.09	479438	21	3814	2727
miR2873c	LOC_Os03g04800.1	167	-22	167	-22	479549	21	2952	1017
miR2873c	LOC_Os03g08860.1	163	-24.64	163	-24.64	480068	21	1248	1131
miR2873c	LOC_Os03g08860.2	163	-24.64	163	-24.64	480069	21	1546	1429
miR2873c	LOC_Os03g11690.1	159	-20.65	159	-20.65	480357	21	2523	2112
miR2873c	LOC_Os03g11690.2	159	-20.65	159	-20.65	480358	21	2848	2467
miR2873c	LOC_Os03g13830.1	168	-22.2	168	-22.2	480639	21	2433	1040
miR2873c	LOC_Os03g15650.1	172	-22.46	172	-22.46	480857	21	2218	2146
miR2873c	LOC_Os03g15650.2	172	-22.46	172	-22.46	480858	21	3137	3065
miR2873c	LOC_Os03g15650.3	172	-22.46	172	-22.46	480859	21	2555	2483
miR2873c	LOC_Os03g16530.1	167	-20.84	167	-20.84	480992	21	6336	5319
miR2873c	LOC_Os03g20080.1	166	-20.65	166	-20.65	481425	21	3837	3241
miR2873c	LOC_Os03g29520.1	163	-20.6	163	-20.6	482374	21	5769	4755
miR2873c	LOC_Os03g33828.1	167	-20.84	167	-20.84	482799	21	1452	1281
miR2873c	LOC_Os03g37250.1	167	-22.21	167	-22.21	483021	21	5322	963

miR2873c	LOC_Os03g39680.1	170	-22.71	170	-22.71	483266	21	1107	454
miR2873c	LOC_Os03g43720.3	167	-20.02	167	-20.02	483648	21	2428	70
miR2873c	LOC_Os03g47970.1	171	-25.52	171	-25.52	484111	21	1479	419
miR2873c	LOC_Os03g49210.1	168	-22.55	168	-22.55	484261	21	2288	1881
miR2873c	LOC_Os03g52280.1	174	-21.74	174	-21.74	484616	21	829	239
miR2873c	LOC_Os03g62379.1	163	-23.53	163	-23.53	485796	21	657	165
miR2873c	LOC_Os04g06402.1	148	-21.9	148	-21.9	486609	21	1494	1425
miR2873c	LOC_Os04g06450.1	148	-21.9	148	-21.9	486614	21	1779	1710
miR2873c	LOC_Os04g10590.1	166	-20.85	166	-20.85	486985	21	1668	866
miR2873c	LOC_Os04g11260.1	145	-20.87	145	-20.87	487054	21	369	286
miR2873c	LOC_Os04g16530.1	172	-22.2	172	-22.2	487511	21	2805	1554
miR2873c	LOC_Os04g16990.1	174	-24.82	174	-24.82	487591	21	2577	452
miR2873c	LOC_Os04g27260.1	148	-21.47	148	-21.47	488594	21	1389	1347
miR2873c	LOC_Os04g29630.1	169	-21.33	169	-21.33	488814	21	1674	300
miR2873c	LOC_Os04g30440.1	167	-20.84	167	-20.84	488891	21	3414	2673
miR2873c	LOC_Os04g33810.1	174	-22.66	174	-22.66	489266	21	1005	976
miR2873c	LOC_Os04g42700.1	169	-23.52	169	-23.52	490192	21	3997	1945
miR2873c	LOC_Os04g48590.1	167	-21.44	167	-21.44	490912	21	1680	699
miR2873c	LOC_Os04g51120.1	175	-24.94	175	-24.94	491157	21	2391	844
miR2873c	LOC_Os04g51120.3	175	-24.94	175	-24.94	491158	21	2623	844
miR2873c	LOC_Os04g52074.1	153	-21.89	153	-21.89	491292	21	3306	1737
miR2873c	LOC_Os04g54010.1	166	-20.22	166	-20.22	491541	21	2157	807
miR2873c	LOC_Os04g54370.1	159	-20.25	159	-20.25	491581	21	1717	1315
miR2873c	LOC_Os04g54530.1	162	-23.75	162	-23.75	491600	21	1101	750
miR2873c	LOC_Os05g02330.1	163	-21.56	163	-21.56	492442	21	1272	1164
miR2873c	LOC_Os05g03090.1	159	-20.1	159	-20.1	492533	21	2823	1332
miR2873c	LOC_Os05g16950.1	148	-21.9	148	-21.9	493985	21	2034	1965
miR2873c	LOC_Os05g17604.3	162	-21.64	162	-21.64	494051	21	5099	3724
miR2873c	LOC_Os05g18920.1	163	-21.56	163	-21.56	494188	21	3099	1164
miR2873c	LOC_Os05g19460.1	167	-20.84	167	-20.84	494242	21	2346	1329
miR2873c	LOC_Os05g24930.1	160	-20.77	160	-20.77	494731	21	3281	1766
miR2873c	LOC_Os05g33390.1	174	-21.64	174	-21.64	495606	21	4278	1976

miR2873c	LOC_Os05g47790.1	157	-20.33	157	-20.33	497173	21	3803	2504
miR2873c	LOC_Os05g50910.1	164	-20.89	164	-20.89	497578	21	3119	2805
miR2873c	LOC_Os06g11950.1	162	-23.75	162	-23.75	499015	21	1161	621
miR2873c	LOC_Os06g14510.1	159	-20.07	159	-20.07	499314	21	2344	1284
miR2873c	LOC_Os06g14510.2	159	-20.07	159	-20.07	499315	21	2097	1037
miR2873c	LOC_Os06g14510.3	159	-20.07	159	-20.07	499316	21	2078	1018
miR2873c	LOC_Os06g19170.1	172	-20.91	172	-20.91	499733	21	2925	2636
miR2873c	LOC_Os06g25560.1	166	-21.53	166	-21.53	500403	21	600	429
miR2873c	LOC_Os06g25800.1	167	-21.12	167	-21.12	500428	21	3346	523
miR2873c	LOC_Os06g25830.1	167	-21.12	167	-21.12	500431	21	840	222
miR2873c	LOC_Os06g25950.1	162	-20.16	162	-20.16	500443	21	1387	1269
miR2873c	LOC_Os06g29020.3	158	-21.51	158	-21.51	500661	21	3424	1292
miR2873c	LOC_Os06g32350.1	166	-21.13	166	-21.13	500999	21	399	15
miR2873c	LOC_Os06g32355.1	166	-21.13	166	-21.13	501000	21	237	15
miR2873c	LOC_Os06g32470.1	167	-20.84	167	-20.84	501011	21	4302	3483
miR2873c	LOC_Os06g36170.1	170	-20.31	170	-20.31	501378	21	2050	1268
miR2873c	LOC_Os06g41410.1	165	-22.06	165	-22.06	501955	21	885	469
miR2873c	LOC_Os06g49350.1	173	-24.12	173	-24.12	502906	21	6657	6433
miR2873c	LOC_Os07g20480.1	148	-21.9	148	-21.9	505211	21	1020	978
miR2873c	LOC_Os07g26290.1	163	-21.56	163	-21.56	505724	21	3003	1164
miR2873c	LOC_Os07g27834.1	163	-21.56	163	-21.56	505892	21	4266	288
miR2873c	LOC_Os07g29360.1	157	-20.7	157	-20.7	506050	21	4236	3024
miR2873c	LOC_Os07g33480.4	187	-24.69	187	-24.69	506495	21	2440	885
miR2873c	LOC_Os07g33630.1	160	-20.61	160	-20.61	506510	21	3197	508
miR2873c	LOC_Os07g36770.1	172	-22.2	172	-22.2	506857	21	4407	705
miR2873c	LOC_Os07g49270.1	172	-20.11	172	-20.11	508344	21	4420	1396
miR2873c	LOC_Os07g49270.2	172	-20.11	172	-20.11	508345	21	3753	1396
miR2873c	LOC_Os08g02930.1	162	-21.3	162	-21.3	508613	21	318	69
miR2873c	LOC_Os08g03220.1	163	-21.56	163	-21.56	508645	21	5520	1164
miR2873c	LOC_Os08g10900.1	171	-20.75	171	-20.75	509511	21	1053	39
miR2873c	LOC_Os08g16110.1	165	-20.36	165	-20.36	510076	21	531	244
miR2873c	LOC_Os08g19540.1	167	-20.84	167	-20.84	510386	21	3897	2976

miR2873c	LOC_Os08g20500.1	150	-21.7	150	-21.7	510487	21	3128	317
miR2873c	LOC_Os08g31150.1	162	-20.31	162	-20.31	511553	21	5976	5708
miR2873c	LOC_Os08g33690.1	161	-21.13	161	-21.13	511844	21	576	483
miR2873c	LOC_Os08g35210.1	159	-21.94	159	-21.94	512018	21	3945	2390
miR2873c	LOC_Os08g38620.1	154	-20.36	154	-20.36	512415	21	1748	1638
miR2873c	LOC_Os08g38620.2	154	-20.36	154	-20.36	512416	21	1720	1610
miR2873c	LOC_Os08g38620.3	154	-20.36	154	-20.36	512417	21	2019	1909
miR2873c	LOC_Os09g02700.1	171	-21.17	171	-21.17	513435	21	2523	109
miR2873c	LOC_Os09g02960.1	167	-20.84	167	-20.84	513463	21	4074	3702
miR2873c	LOC_Os09g04160.1	157	-20.52	157	-20.52	513579	21	2130	1644
miR2873c	LOC_Os09g04160.2	157	-20.52	157	-20.52	513580	21	2681	1644
miR2873c	LOC_Os09g08470.1	159	-21.09	159	-21.09	513939	21	4581	625
miR2873c	LOC_Os09g14040.1	165	-21.12	165	-21.12	514515	21	1179	373
miR2873c	LOC_Os09g23370.2	175	-20.06	175	-20.06	515395	21	826	92
miR2873c	LOC_Os09g26430.1	163	-21.11	163	-21.11	515728	21	4041	1045
miR2873c	LOC_Os09g38790.1	165	-21.72	165	-21.72	517022	21	2997	1912
miR2873c	LOC_Os09g38790.2	165	-21.72	165	-21.72	517023	21	2865	1780
miR2873c	LOC_Os09g38790.3	165	-21.72	165	-21.72	517024	21	1627	542
miR2873c	LOC_Os09g39590.1	158	-22.45	158	-22.45	517111	21	2552	1425
miR2873c	LOC_Os10g02720.1	183	-26.07	183	-26.07	517354	21	2371	9
miR2873c	LOC_Os10g02720.2	183	-26.07	183	-26.07	517355	21	1778	9
miR2873c	LOC_Os10g04230.1	163	-21.56	163	-21.56	517507	21	2484	1164
miR2873c	LOC_Os10g07998.1	161	-21.52	161	-21.52	517869	21	1372	4
miR2873c	LOC_Os10g09940.1	162	-21.9	162	-21.9	518061	21	3258	2973
miR2873c	LOC_Os10g15190.1	154	-20.3	154	-20.3	518502	21	954	756
miR2873c	LOC_Os10g27450.1	157	-20.64	157	-20.64	519567	21	1647	782
miR2873c	LOC_Os10g27450.2	157	-20.64	157	-20.64	519568	21	1545	681
miR2873c	LOC_Os10g28410.1	166	-22.4	166	-22.4	519624	21	1255	643
miR2873c	LOC_Os10g28410.2	166	-22.4	166	-22.4	519625	21	1245	633
miR2873c	LOC_Os10g33010.1	163	-21.56	163	-21.56	520084	21	5205	1077
miR2873c	LOC_Os11g03500.1	163	-20.04	163	-20.04	521538	21	1561	495
miR2873c	LOC_Os11g03780.1	158	-21.61	158	-21.61	521571	21	3015	2121

miR2873c	LOC_Os11g03780.2	158	-21.61	158	-21.61	521572	21	2416	2117
miR2873c	LOC_Os11g03794.1	158	-21.61	158	-21.61	521573	21	2372	61
miR2873c	LOC_Os11g05050.1	161	-20.01	161	-20.01	521727	21	714	53
miR2873c	LOC_Os11g08542.1	178	-27.25	178	-27.25	522144	21	2793	1184
miR2873c	LOC_Os11g09474.1	161	-23.99	161	-23.99	522237	21	2964	1908
miR2873c	LOC_Os11g09919.1	167	-20.84	167	-20.84	522286	21	6567	3243
miR2873c	LOC_Os11g12210.1	148	-21.47	148	-21.47	522523	21	5550	5481
miR2873c	LOC_Os11g14390.1	169	-20.39	169	-20.39	522700	21	1712	969
miR2873c	LOC_Os11g19830.1	163	-21.56	163	-21.56	523219	21	5298	1239
miR2873c	LOC_Os11g25050.1	166	-21.52	166	-21.52	523595	21	3192	2022
miR2873c	LOC_Os11g29990.1	174	-22.35	174	-22.35	524085	21	3033	924
miR2873c	LOC_Os11g31870.1	179	-23.8	179	-23.8	524267	21	1209	1036
miR2873c	LOC_Os11g35320.1	155	-23.55	155	-23.55	524596	21	1779	632
miR2873c	LOC_Os11g36630.1	154	-21.21	154	-21.21	524745	21	2184	211
miR2873c	LOC_Os11g37860.1	153	-21.06	153	-21.06	524867	21	3581	2807
miR2873c	LOC_Os12g01790.1	179	-23.8	179	-23.8	526034	21	3987	3814
miR2873c	LOC_Os12g03200.1	166	-20.25	166	-20.25	526217	21	2273	973
miR2873c	LOC_Os12g03200.2	166	-20.25	166	-20.25	526218	21	3519	974
miR2873c	LOC_Os12g03200.3	166	-20.25	166	-20.25	526219	21	2099	973
miR2873c	LOC_Os12g03260.1	163	-20.04	163	-20.04	526225	21	2120	964
miR2873c	LOC_Os12g05290.1	173	-24.12	173	-24.12	526466	21	4767	4543
miR2873c	LOC_Os12g06890.1	183	-29.87	183	-29.87	526645	21	3370	1031
miR2873c	LOC_Os12g09739.1	175	-21.67	175	-21.67	526955	21	4259	2610
miR2873c	LOC_Os12g19910.1	148	-21.9	148	-21.9	528000	21	993	930
miR2873c	LOC_Os12g23230.1	163	-21.56	163	-21.56	528241	21	6033	1164
miR2873c	LOC_Os12g23920.1	163	-21.56	163	-21.56	528306	21	5955	1164
miR2873c	LOC_Os12g26310.1	167	-20.84	167	-20.84	528542	21	5799	4839
miR2873c	LOC_Os12g30720.1	148	-21.03	148	-21.03	528981	21	2865	1806
miR2873c	LOC_Os12g30760.1	152	-21.01	152	-21.01	528985	21	3168	1800
miR2873c	LOC_Os12g34100.1	158	-20.19	158	-20.19	529326	21	2815	823
miR2873c	LOC_Os12g39610.1	163	-21.56	163	-21.56	529952	21	5259	639
miR2873c	LOC_Os12g42064.1	336	-40.28	168	-20.14	530250	21	2969	2384 2502

miR2873c	LOC_Os12g43060.1	156	-23.24	156	-23.24	530366	21	4884	4574
miR2863a	LOC_Os01g01830.1	179	-28.75	179	-28.75	530817	21	3274	1234
miR2863a	LOC_Os01g02250.1	157	-20.89	157	-20.89	530870	21	4035	2304
miR2863a	LOC_Os01g02280.1	157	-20.89	157	-20.89	530873	21	5367	3636
miR2863a	LOC_Os01g02960.1	161	-22.36	161	-22.36	530949	21	1861	1774
miR2863a	LOC_Os01g04160.1	167	-24.82	167	-24.82	531074	21	3091	859
miR2863a	LOC_Os01g04180.1	161	-23.43	161	-23.43	531076	21	885	355
miR2863a	LOC_Os01g05640.1	170	-21.11	170	-21.11	531249	21	3307	1177
miR2863a	LOC_Os01g06130.1	146	-21.73	146	-21.73	531296	21	1066	586
miR2863a	LOC_Os01g06130.2	146	-21.73	146	-21.73	531297	21	993	520
miR2863a	LOC_Os01g06150.1	146	-20.9	146	-20.9	531299	21	1041	638
miR2863a	LOC_Os01g09010.1	157	-20.1	157	-20.1	531640	21	1873	884
miR2863a	LOC_Os01g10050.2	155	-21.28	155	-21.28	531757	21	2225	246
miR2863a	LOC_Os01g12720.1	155	-21.92	155	-21.92	532050	21	2306	1000
miR2863a	LOC_Os01g15350.1	155	-20.59	155	-20.59	532349	21	2323	2227
miR2863a	LOC_Os01g15979.1	158	-26.49	158	-26.49	532419	21	849	406
miR2863a	LOC_Os01g22039.1	154	-22.52	154	-22.52	532983	21	1641	949
miR2863a	LOC_Os01g22090.1	157	-21.86	157	-21.86	532987	21	2772	2499
miR2863a	LOC_Os01g25060.1	158	-23.96	158	-23.96	533289	21	2451	442
miR2863a	LOC_Os01g25740.1	169	-22.81	169	-22.81	533360	21	4397	119
miR2863a	LOC_Os01g26160.1	158	-21.45	158	-21.45	533401	21	4232	923
miR2863a	LOC_Os01g26160.2	158	-21.45	158	-21.45	533402	21	4257	923
miR2863a	LOC_Os01g26224.1	150	-20.13	150	-20.13	533406	21	5145	3155
miR2863a	LOC_Os01g28750.1	162	-28.96	162	-28.96	533618	21	783	44
miR2863a	LOC_Os01g32930.1	165	-20.74	165	-20.74	533948	21	672	417
miR2863a	LOC_Os01g33740.1	152	-21.36	152	-21.36	534025	21	2799	482
miR2863a	LOC_Os01g35870.1	146	-22.44	146	-22.44	534250	21	306	283
miR2863a	LOC_Os01g36064.1	153	-23.16	153	-23.16	534267	21	2694	2218
miR2863a	LOC_Os01g37520.1	153	-20.38	153	-20.38	534426	21	609	385
miR2863a	LOC_Os01g37580.1	160	-20.58	160	-20.58	534434	21	5799	2168
miR2863a	LOC_Os01g38100.1	152	-21.83	152	-21.83	534495	21	2764	625
miR2863a	LOC_Os01g38180.1	161	-23.09	161	-23.09	534503	21	2036	886

miR2863a	LOC_Os01g39380.1	168	-22.5	168	-22.5	534630	21	3927	3514
miR2863a	LOC_Os01g39380.2	168	-22.5	168	-22.5	534631	21	3936	3332
miR2863a	LOC_Os01g39380.3	168	-22.5	168	-22.5	534632	21	3930	3517
miR2863a	LOC_Os01g39740.1	156	-20.86	156	-20.86	534668	21	2115	1386
miR2863a	LOC_Os01g41350.1	158	-21.02	158	-21.02	534849	21	1233	714
miR2863a	LOC_Os01g41710.1	159	-21.89	159	-21.89	534886	21	1236	294
miR2863a	LOC_Os01g43340.1	161	-20.56	161	-20.56	535075	21	3942	42
miR2863a	LOC_Os01g44394.1	149	-20.41	149	-20.41	535203	21	1451	477
miR2863a	LOC_Os01g46380.1	165	-23.87	165	-23.87	535357	21	2263	532
miR2863a	LOC_Os01g47490.3	154	-20.77	154	-20.77	535491	21	2704	588
miR2863a	LOC_Os01g47830.1	152	-20.09	152	-20.09	535540	21	1812	1061
miR2863a	LOC_Os01g48850.1	157	-26.75	157	-26.75	535658	21	1993	1104
miR2863a	LOC_Os01g51540.1	164	-24.99	164	-24.99	535965	21	1445	987
miR2863a	LOC_Os01g52500.1	153	-24.32	153	-24.32	536081	21	2604	1528
miR2863a	LOC_Os01g52500.2	153	-24.32	153	-24.32	536082	21	2378	1289
miR2863a	LOC_Os01g52500.3	153	-24.32	153	-24.32	536083	21	2617	1528
miR2863a	LOC_Os01g52500.4	153	-24.32	153	-24.32	536084	21	2106	1017
miR2863a	LOC_Os01g52500.5	153	-24.32	153	-24.32	536085	21	3179	2090
miR2863a	LOC_Os01g54515.1	150	-21.4	150	-21.4	536307	21	1965	622
miR2863a	LOC_Os01g55040.1	164	-20.67	164	-20.67	536370	21	6528	6384
miR2863a	LOC_Os01g55190.1	162	-25.11	162	-25.11	536390	21	1781	482
miR2863a	LOC_Os01g57945.1	164	-23.95	164	-23.95	536744	21	483	54
miR2863a	LOC_Os01g58240.1	157	-20.72	157	-20.72	536781	21	2511	1054
miR2863a	LOC_Os01g58280.1	157	-21.41	157	-21.41	536783	21	2316	1056
miR2863a	LOC_Os01g60060.1	166	-22.26	166	-22.26	536991	21	3495	1272
miR2863a	LOC_Os01g60270.1	160	-24.16	160	-24.16	537017	21	2025	744
miR2863a	LOC_Os01g60270.2	160	-24.16	160	-24.16	537018	21	2416	737
miR2863a	LOC_Os01g61460.1	154	-21.94	154	-21.94	537142	21	2174	1540
miR2863a	LOC_Os01g61670.1	153	-24.86	153	-24.86	537170	21	1094	2
miR2863a	LOC_Os01g62070.1	145	-20.19	145	-20.19	537237	21	1776	330
miR2863a	LOC_Os01g62420.1	162	-20.81	162	-20.81	537280	21	1420	259
miR2863a	LOC_Os01g62420.2	162	-20.81	162	-20.81	537281	21	919	259

miR2863a	LOC_Os01g62420.3	162	-20.81	162	-20.81	537282	21	853	259
miR2863a	LOC_Os01g62420.4	160	-23.99	160	-23.99	537283	21	1415	255
miR2863a	LOC_Os01g62530.1	165	-23.32	165	-23.32	537300	21	936	153
miR2863a	LOC_Os01g63240.1	146	-21.62	146	-21.62	537384	21	2018	1426
miR2863a	LOC_Os01g66940.1	161	-22.74	161	-22.74	537839	21	1793	810
miR2863a	LOC_Os01g68330.1	157	-23.55	157	-23.55	538014	21	1647	535
miR2863a	LOC_Os01g69030.1	158	-22.14	158	-22.14	538101	21	3739	427
miR2863a	LOC_Os01g69030.2	158	-22.14	158	-22.14	538102	21	3805	427
miR2863a	LOC_Os01g69030.3	158	-22.14	158	-22.14	538103	21	3801	427
miR2863a	LOC_Os01g69030.4	158	-22.14	158	-22.14	538104	21	3771	427
miR2863a	LOC_Os01g69120.1	161	-22.59	161	-22.59	538117	21	1998	1520
miR2863a	LOC_Os01g69120.2	161	-22.59	161	-22.59	538118	21	2231	1520
miR2863a	LOC_Os01g69120.3	161	-22.59	161	-22.59	538119	21	2109	1520
miR2863a	LOC_Os01g69900.1	153	-20.58	153	-20.58	538150	21	2348	2075
miR2863a	LOC_Os01g71190.1	155	-21.06	155	-21.06	538316	21	945	587
miR2863a	LOC_Os01g73430.1	158	-20.19	158	-20.19	538572	21	4713	2831
miR2863a	LOC_Os01g74040.1	144	-20.57	144	-20.57	538650	21	1526	775
miR2863a	LOC_Os02g01280.1	158	-21.84	158	-21.84	538769	21	2335	539
miR2863a	LOC_Os02g01800.1	149	-20.37	149	-20.37	538830	21	3549	510
miR2863a	LOC_Os02g02390.1	156	-21.96	156	-21.96	538895	21	2736	208
miR2863a	LOC_Os02g03250.1	160	-21.4	160	-21.4	539014	21	1194	514
miR2863a	LOC_Os02g03560.2	159	-20.99	159	-20.99	539054	21	2498	571
miR2863a	LOC_Os02g05030.1	162	-20.22	162	-20.22	539254	21	1688	436
miR2863a	LOC_Os02g05180.1	153	-23.16	153	-23.16	539269	21	4851	4022
miR2863a	LOC_Os02g05700.1	157	-23.69	157	-23.69	539346	21	2657	214
miR2863a	LOC_Os02g05700.2	157	-23.69	157	-23.69	539347	21	2903	214
miR2863a	LOC_Os02g06230.1	170	-22.08	170	-22.08	539405	21	4362	1994
miR2863a	LOC_Os02g07180.1	159	-20.81	159	-20.81	539526	21	1699	1071
miR2863a	LOC_Os02g08310.1	171	-23.37	171	-23.37	539677	21	1479	39
miR2863a	LOC_Os02g10540.1	155	-21.28	155	-21.28	539890	21	1260	12
miR2863a	LOC_Os02g12360.1	145	-21.68	145	-21.68	540049	21	3183	328
miR2863a	LOC_Os02g13560.1	155	-22.08	155	-22.08	540209	21	2965	335

miR2863a	LOC_Os02g13560.2	155	-22.08	155	-22.08	540210	21	3159	529
miR2863a	LOC_Os02g13560.3	155	-22.08	155	-22.08	540211	21	3025	395
miR2863a	LOC_Os02g13560.4	155	-22.08	155	-22.08	540212	21	2761	131
miR2863a	LOC_Os02g14450.1	158	-21.31	158	-21.31	540309	21	675	242
miR2863a	LOC_Os02g17304.1	158	-22.3	158	-22.3	540632	21	3188	286
miR2863a	LOC_Os02g17720.1	154	-22.23	154	-22.23	540678	21	4497	2633
miR2863a	LOC_Os02g24840.1	161	-20.9	161	-20.9	541361	21	972	689
miR2863a	LOC_Os02g25530.1	149	-22.87	149	-22.87	541430	21	381	168
miR2863a	LOC_Os02g25880.1	154	-22.23	154	-22.23	541468	21	3606	2345
miR2863a	LOC_Os02g30300.1	160	-23.22	160	-23.22	541954	21	1412	1264
miR2863a	LOC_Os02g30730.1	146	-20.46	146	-20.46	542006	21	3090	359
miR2863a	LOC_Os02g30730.2	146	-20.46	146	-20.46	542007	21	3164	357
miR2863a	LOC_Os02g30800.1	167	-22.78	167	-22.78	542014	21	6861	3752
miR2863a	LOC_Os02g33620.1	154	-22.23	154	-22.23	542282	21	3042	1779
miR2863a	LOC_Os02g38000.1	150	-22.7	150	-22.7	542783	21	4635	2777
miR2863a	LOC_Os02g38440.1	151	-20.01	151	-20.01	542838	21	1311	439
miR2863a	LOC_Os02g41780.1	158	-20.9	158	-20.9	543202	21	1675	468
miR2863a	LOC_Os02g43740.1	162	-20.93	162	-20.93	543444	21	3018	1460
miR2863a	LOC_Os02g46580.1	158	-25.05	158	-25.05	543812	21	3702	855
miR2863a	LOC_Os02g47380.1	165	-24.13	165	-24.13	543911	21	2868	2367
miR2863a	LOC_Os02g47820.1	163	-23.56	163	-23.56	543973	21	333	108
miR2863a	LOC_Os02g48340.1	170	-24.53	170	-24.53	544036	21	2237	732
miR2863a	LOC_Os02g48340.2	170	-24.53	170	-24.53	544037	21	2213	705
miR2863a	LOC_Os02g48340.3	170	-24.53	170	-24.53	544038	21	2210	705
miR2863a	LOC_Os02g48340.5	170	-24.53	170	-24.53	544039	21	1719	705
miR2863a	LOC_Os02g48340.6	170	-24.53	170	-24.53	544040	21	2304	705
miR2863a	LOC_Os02g48500.1	147	-21.02	147	-21.02	544059	21	480	165
miR2863a	LOC_Os02g48790.1	166	-21.04	166	-21.04	544097	21	3231	2100
miR2863a	LOC_Os02g48790.2	166	-21.04	166	-21.04	544098	21	3078	1947
miR2863a	LOC_Os02g48790.3	166	-21.04	166	-21.04	544099	21	2985	1854
miR2863a	LOC_Os02g48790.4	166	-21.04	166	-21.04	544100	21	3177	2046
miR2863a	LOC_Os02g48790.5	166	-21.04	166	-21.04	544101	21	3220	1947

miR2863a	LOC_Os02g48830.1	161	-21.86	161	-21.86	544105	21	2304	707
miR2863a	LOC_Os02g50240.1	157	-20.75	157	-20.75	544278	21	1744	104
miR2863a	LOC_Os02g50240.2	157	-20.75	157	-20.75	544279	21	1453	104
miR2863a	LOC_Os02g51680.1	158	-23.32	158	-23.32	544458	21	1423	920
miR2863a	LOC_Os02g52280.1	155	-20.73	155	-20.73	544538	21	3805	2945
miR2863a	LOC_Os02g53310.1	158	-22.14	158	-22.14	544668	21	486	445
miR2863a	LOC_Os02g53990.1	164	-21.02	164	-21.02	544754	21	3869	2079
miR2863a	LOC_Os02g55020.1	157	-20.64	157	-20.64	544874	21	2219	1167
miR2863a	LOC_Os02g55070.1	169	-22.15	169	-22.15	544881	21	1741	252
miR2863a	LOC_Os02g56740.1	154	-21.58	154	-21.58	545088	21	3142	1139
miR2863a	LOC_Os02g56740.2	154	-21.58	154	-21.58	545089	21	3244	1241
miR2863a	LOC_Os02g57200.1	309	-44.79	159	-22.73	545150	21	3116	2012 1924
miR2863a	LOC_Os02g58110.1	175	-25.68	175	-25.68	545293	21	2174	1212
miR2863a	LOC_Os02g58250.1	160	-20.75	160	-20.75	545311	21	3533	3395
miR2863a	LOC_Os02g58530.1	154	-21.84	154	-21.84	545340	21	1959	45
miR2863a	LOC_Os03g02010.4	170	-20.17	170	-20.17	545522	21	2275	230
miR2863a	LOC_Os03g02440.1	156	-20.4	156	-20.4	545586	21	4464	3972
miR2863a	LOC_Os03g02440.2	156	-20.4	156	-20.4	545587	21	4051	3559
miR2863a	LOC_Os03g03680.1	166	-23.28	166	-23.28	545751	21	2423	365
miR2863a	LOC_Os03g04920.1	159	-23.93	159	-23.93	545898	21	5531	4783
miR2863a	LOC_Os03g05100.1	153	-20.2	153	-20.2	545922	21	874	451
miR2863a	LOC_Os03g06580.1	165	-20.84	165	-20.84	546108	21	1365	740
miR2863a	LOC_Os03g06580.2	165	-20.84	165	-20.84	546109	21	1065	569
miR2863a	LOC_Os03g06580.3	165	-20.84	165	-20.84	546110	21	1193	697
miR2863a	LOC_Os03g06580.4	165	-20.84	165	-20.84	546111	21	1194	569
miR2863a	LOC_Os03g07170.1	154	-20.59	154	-20.59	546187	21	3033	1510
miR2863a	LOC_Os03g07180.1	154	-20.59	154	-20.59	546188	21	2079	41
miR2863a	LOC_Os03g07180.3	154	-20.59	154	-20.59	546190	21	1953	41
miR2863a	LOC_Os03g07310.1	150	-20.29	150	-20.29	546204	21	1881	1413
miR2863a	LOC_Os03g08000.1	154	-24.35	154	-24.35	546286	21	1848	1766
miR2863a	LOC_Os03g11320.1	157	-23.54	157	-23.54	546645	21	2527	968
miR2863a	LOC_Os03g11320.2	157	-23.54	157	-23.54	546646	21	2520	961

miR2863a	LOC_Os03g11980.1	154	-22.71	154	-22.71	546729	21	2434	2133
miR2863a	LOC_Os03g12064.6	152	-21.3	152	-21.3	546747	21	2698	849
miR2863a	LOC_Os03g13600.1	151	-20.33	151	-20.33	546946	21	1337	288
miR2863a	LOC_Os03g13600.2	151	-20.33	151	-20.33	546947	21	1322	288
miR2863a	LOC_Os03g14110.1	161	-20.35	161	-20.35	547021	21	1200	25
miR2863a	LOC_Os03g14520.1	160	-20.69	160	-20.69	547069	21	1316	1198
miR2863a	LOC_Os03g14980.1	155	-21.06	155	-21.06	547118	21	4170	3005
miR2863a	LOC_Os03g14980.2	155	-21.06	155	-21.06	547119	21	4255	3005
miR2863a	LOC_Os03g15910.1	163	-21.09	163	-21.09	547240	21	1767	1416
miR2863a	LOC_Os03g17030.1	169	-23.83	169	-23.83	547401	21	2466	641
miR2863a	LOC_Os03g17590.1	154	-20.82	154	-20.82	547467	21	1766	1437
miR2863a	LOC_Os03g17780.1	169	-27.34	169	-27.34	547486	21	1909	462
miR2863a	LOC_Os03g18290.1	152	-21.52	152	-21.52	547550	21	1131	297
miR2863a	LOC_Os03g18350.1	152	-20.25	152	-20.25	547558	21	3021	813
miR2863a	LOC_Os03g18729.1	161	-22.75	161	-22.75	547602	21	300	255
miR2863a	LOC_Os03g19040.1	154	-20.19	154	-20.19	547636	21	1018	823
miR2863a	LOC_Os03g20450.1	161	-20.97	161	-20.97	547812	21	4346	1205
miR2863a	LOC_Os03g21850.1	161	-20.14	161	-20.14	547978	21	3700	832
miR2863a	LOC_Os03g21850.2	161	-20.14	161	-20.14	547979	21	3424	831
miR2863a	LOC_Os03g22730.1	154	-21.03	154	-21.03	548091	21	1707	1269
miR2863a	LOC_Os03g22740.1	158	-23.57	158	-23.57	548092	21	1707	1269
miR2863a	LOC_Os03g22900.1	165	-23.14	165	-23.14	548117	21	4929	492
miR2863a	LOC_Os03g23960.1	162	-28.24	162	-28.24	548170	21	1506	680
miR2863a	LOC_Os03g25700.1	161	-25.06	161	-25.06	548359	21	1728	1222
miR2863a	LOC_Os03g27330.1	169	-22.61	169	-22.61	548523	21	549	359
miR2863a	LOC_Os03g28910.1	156	-22.99	156	-22.99	548645	21	867	136
miR2863a	LOC_Os03g29440.1	153	-20.67	153	-20.67	548706	21	2457	964
miR2863a	LOC_Os03g29980.2	140	-21.88	140	-21.88	548753	21	2758	161
miR2863a	LOC_Os03g30700.1	154	-22.6	154	-22.6	548828	21	1230	374
miR2863a	LOC_Os03g36470.1	154	-22.65	154	-22.65	549286	21	2259	2092
miR2863a	LOC_Os03g39610.1	164	-22.5	164	-22.5	549595	21	1684	366
miR2863a	LOC_Os03g39610.2	164	-22.5	164	-22.5	549596	21	1773	455

miR2863a	LOC_Os03g40250.1	157	-21.85	157	-21.85	549656	21	2027	203
miR2863a	LOC_Os03g40260.1	153	-21.38	153	-21.38	549657	21	2094	1102
miR2863a	LOC_Os03g43200.1	157	-20.98	157	-20.98	549933	21	648	390
miR2863a	LOC_Os03g43410.1	151	-22.63	151	-22.63	549954	21	1096	186
miR2863a	LOC_Os03g45400.1	151	-20.13	151	-20.13	550175	21	1647	697
miR2863a	LOC_Os03g47460.1	165	-21.51	165	-21.51	550383	21	1677	440
miR2863a	LOC_Os03g48560.1	162	-23.81	162	-23.81	550515	21	1459	824
miR2863a	LOC_Os03g49110.1	165	-23.76	165	-23.76	550585	21	1545	1016
miR2863a	LOC_Os03g49220.1	158	-21.43	158	-21.43	550600	21	1764	1126
miR2863a	LOC_Os03g49220.2	158	-21.43	158	-21.43	550601	21	1805	1167
miR2863a	LOC_Os03g50440.1	154	-20.29	154	-20.29	550751	21	1197	363
miR2863a	LOC_Os03g51080.1	163	-21.24	163	-21.24	550821	21	2184	996
miR2863a	LOC_Os03g53140.1	152	-22.73	152	-22.73	551054	21	3251	1379
miR2863a	LOC_Os03g53140.2	152	-22.73	152	-22.73	551055	21	2805	1400
miR2863a	LOC_Os03g53140.3	152	-22.73	152	-22.73	551056	21	3463	1377
miR2863a	LOC_Os03g53700.1	157	-22.42	157	-22.42	551125	21	1923	634
miR2863a	LOC_Os03g53700.2	157	-22.42	157	-22.42	551126	21	1033	634
miR2863a	LOC_Os03g54760.1	145	-22.51	145	-22.51	551201	21	1727	550
miR2863a	LOC_Os03g55470.1	161	-24.06	161	-24.06	551287	21	2382	1817
miR2863a	LOC_Os03g56830.1	157	-20.87	157	-20.87	551464	21	2127	1560
miR2863a	LOC_Os03g56830.2	157	-20.87	157	-20.87	551465	21	1236	698
miR2863a	LOC_Os03g63040.1	316	-43.13	165	-22.79	552236	21	3297	1252 1060
miR2863a	LOC_Os03g63124.1	165	-25.81	165	-25.81	552244	21	5910	4308
miR2863a	LOC_Os03g64030.1	144	-20.09	144	-20.09	552355	21	1018	976
miR2863a	LOC_Os04g01120.1	149	-21.66	149	-21.66	552418	21	2142	1441
miR2863a	LOC_Os04g01310.1	157	-22.76	157	-22.76	552446	21	2758	2653
miR2863a	LOC_Os04g02820.1	157	-20.75	157	-20.75	552606	21	3228	2276
miR2863a	LOC_Os04g02820.2	157	-20.75	157	-20.75	552607	21	3000	2048
miR2863a	LOC_Os04g02980.1	158	-22.82	158	-22.82	552630	21	2159	611
miR2863a	LOC_Os04g03860.1	157	-20.02	157	-20.02	552710	21	3344	2606
miR2863a	LOC_Os04g07850.1	148	-20.7	148	-20.7	553087	21	360	125
miR2863a	LOC_Os04g08400.1	153	-21.68	153	-21.68	553139	21	3423	3205

miR2863a	LOC_Os04g09590.1	161	-21.42	161	-21.42	553231	21	1296	1088
miR2863a	LOC_Os04g10380.1	173	-20.07	173	-20.07	553308	21	5131	2336
miR2863a	LOC_Os04g13470.1	153	-22.82	153	-22.82	553598	21	3764	3021
miR2863a	LOC_Os04g13650.1	148	-20.82	148	-20.82	553615	21	2289	282
miR2863a	LOC_Os04g14370.1	150	-21.58	150	-21.58	553688	21	1073	693
miR2863a	LOC_Os04g18570.1	166	-21.08	166	-21.08	554084	21	3039	2973
miR2863a	LOC_Os04g18690.1	165	-23.7	165	-23.7	554096	21	2505	1576
miR2863a	LOC_Os04g19740.1	147	-20.57	147	-20.57	554199	21	2567	1218
miR2863a	LOC_Os04g22000.1	154	-20.41	154	-20.41	554419	21	2280	1944
miR2863a	LOC_Os04g24294.2	152	-21.19	152	-21.19	554634	21	2260	1638
miR2863a	LOC_Os04g24300.1	152	-21.19	152	-21.19	554635	21	2789	377
miR2863a	LOC_Os04g25210.1	160	-20.42	160	-20.42	554727	21	1682	29
miR2863a	LOC_Os04g27850.1	151	-20.09	151	-20.09	554987	21	1271	1146
miR2863a	LOC_Os04g32020.1	162	-21.89	162	-21.89	555393	21	3697	1579
miR2863a	LOC_Os04g32020.2	162	-21.89	162	-21.89	555394	21	3852	1579
miR2863a	LOC_Os04g32020.3	162	-21.89	162	-21.89	555395	21	2669	1579
miR2863a	LOC_Os04g32440.1	154	-21.97	154	-21.97	555439	21	1122	372
miR2863a	LOC_Os04g33530.1	145	-22.11	145	-22.11	555574	21	1705	1153
miR2863a	LOC_Os04g34290.1	171	-25.72	171	-25.72	555654	21	2451	483
miR2863a	LOC_Os04g34300.1	171	-25.72	171	-25.72	555655	21	2034	486
miR2863a	LOC_Os04g35870.1	154	-22.23	154	-22.23	555829	21	4806	2780
miR2863a	LOC_Os04g36910.1	168	-22.85	168	-22.85	555892	21	2728	1221
miR2863a	LOC_Os04g36910.2	168	-22.85	168	-22.85	555893	21	2816	1309
miR2863a	LOC_Os04g39864.1	162	-24.24	162	-24.24	556181	21	1878	819
miR2863a	LOC_Os04g39864.2	162	-24.24	162	-24.24	556182	21	1751	819
miR2863a	LOC_Os04g39864.3	162	-24.24	162	-24.24	556183	21	1954	819
miR2863a	LOC_Os04g39864.4	162	-24.24	162	-24.24	556184	21	1681	817
miR2863a	LOC_Os04g40370.1	177	-24.55	177	-24.55	556241	21	2028	1166
miR2863a	LOC_Os04g40370.2	177	-24.55	177	-24.55	556242	21	2025	1163
miR2863a	LOC_Os04g40370.3	177	-24.55	177	-24.55	556243	21	1729	867
miR2863a	LOC_Os04g41320.1	157	-21.24	157	-21.24	556370	21	2500	1389
miR2863a	LOC_Os04g41910.1	162	-20.11	162	-20.11	556434	21	1956	1057

miR2863a	LOC_Os04g41910.2	162	-20.11	162	-20.11	556435	21	1606	1057
miR2863a	LOC_Os04g45330.1	160	-20.66	160	-20.66	556859	21	1345	341
miR2863a	LOC_Os04g45800.1	171	-22.68	171	-22.68	556906	21	3152	2389
miR2863a	LOC_Os04g46180.1	162	-20.93	162	-20.93	556951	21	3026	1426
miR2863a	LOC_Os04g46180.2	162	-20.93	162	-20.93	556952	21	2982	1382
miR2863a	LOC_Os04g49757.2	153	-23.16	153	-23.16	557385	21	1950	99
miR2863a	LOC_Os04g49900.1	163	-22.4	163	-22.4	557399	21	4248	3692
miR2863a	LOC_Os04g49910.1	154	-22.23	154	-22.23	557400	21	4056	2030
miR2863a	LOC_Os04g51090.1	156	-22.59	156	-22.59	557491	21	8044	2237
miR2863a	LOC_Os04g51930.1	158	-26.23	158	-26.23	557612	21	5355	2433
miR2863a	LOC_Os04g51970.1	154	-20.67	154	-20.67	557618	21	2004	1053
miR2863a	LOC_Os04g51970.2	154	-20.67	154	-20.67	557619	21	1915	1053
miR2863a	LOC_Os04g52240.1	158	-21.58	158	-21.58	557648	21	2741	1358
miR2863a	LOC_Os04g53640.1	154	-25.99	154	-25.99	557829	21	1048	493
miR2863a	LOC_Os04g54110.1	158	-21.01	158	-21.01	557889	21	2064	1722
miR2863a	LOC_Os04g54120.1	146	-20.63	146	-20.63	557890	21	2469	1387
miR2863a	LOC_Os04g55560.2	167	-20.06	167	-20.06	558055	21	2490	1945
miR2863a	LOC_Os04g55560.3	167	-20.06	167	-20.06	558056	21	2501	1956
miR2863a	LOC_Os04g55560.4	167	-20.06	167	-20.06	558057	21	1674	1129
miR2863a	LOC_Os04g56280.1	154	-22.77	154	-22.77	558140	21	1002	54
miR2863a	LOC_Os04g57570.1	165	-25.81	165	-25.81	558317	21	6834	5232
miR2863a	LOC_Os04g58180.1	159	-20.34	159	-20.34	558395	21	3028	1698
miR2863a	LOC_Os04g59420.1	153	-21.15	153	-21.15	558546	21	1488	671
miR2863a	LOC_Os04g59420.2	153	-21.15	153	-21.15	558547	21	1344	527
miR2863a	LOC_Os05g01730.1	144	-20.13	144	-20.13	558703	21	1449	749
miR2863a	LOC_Os05g01730.2	144	-20.13	144	-20.13	558704	21	994	784
miR2863a	LOC_Os05g01730.3	144	-20.13	144	-20.13	558705	21	1330	630
miR2863a	LOC_Os05g04740.1	151	-20.69	151	-20.69	559060	21	1795	96
miR2863a	LOC_Os05g04740.2	151	-20.69	151	-20.69	559061	21	1885	96
miR2863a	LOC_Os05g05300.1	152	-21.78	152	-21.78	559120	21	3778	1790
miR2863a	LOC_Os05g05310.1	152	-21.78	152	-21.78	559121	21	3863	415
miR2863a	LOC_Os05g05310.2	152	-21.78	152	-21.78	559122	21	3859	415

miR2863a	LOC_Os05g07220.1	156	-20.62	156	-20.62	559353	21	3840	266
miR2863a	LOC_Os05g07220.2	156	-20.62	156	-20.62	559354	21	3683	266
miR2863a	LOC_Os05g09320.1	150	-21.77	150	-21.77	559597	21	2601	584
miR2863a	LOC_Os05g10730.1	164	-22.28	164	-22.28	559713	21	6041	4584
miR2863a	LOC_Os05g10730.2	164	-22.28	164	-22.28	559714	21	6094	4637
miR2863a	LOC_Os05g10730.3	164	-22.28	164	-22.28	559715	21	6127	4670
miR2863a	LOC_Os05g10740.1	164	-22.28	164	-22.28	559716	21	1845	169
miR2863a	LOC_Os05g10740.2	164	-22.28	164	-22.28	559717	21	1945	169
miR2863a	LOC_Os05g15720.1	164	-21.93	164	-21.93	560198	21	3641	3197
miR2863a	LOC_Os05g15850.1	153	-20.22	153	-20.22	560209	21	1149	899
miR2863a	LOC_Os05g15950.1	164	-22.22	164	-22.22	560221	21	1557	1230
miR2863a	LOC_Os05g18170.1	154	-21.11	154	-21.11	560445	21	1500	56
miR2863a	LOC_Os05g20180.1	152	-21.29	152	-21.29	560661	21	4404	426
miR2863a	LOC_Os05g22940.1	162	-21.23	162	-21.23	560851	21	7687	4273
miR2863a	LOC_Os05g24930.1	158	-20.05	158	-20.05	561069	21	3281	2106
miR2863a	LOC_Os05g28590.1	175	-28.26	175	-28.26	561457	21	609	271
miR2863a	LOC_Os05g30454.1	152	-20.09	152	-20.09	561617	21	1211	1043
miR2863a	LOC_Os05g30610.1	157	-20.01	157	-20.01	561633	21	4776	3387
miR2863a	LOC_Os05g31160.1	149	-20.06	149	-20.06	561703	21	1523	378
miR2863a	LOC_Os05g31160.2	149	-20.06	149	-20.06	561704	21	1546	394
miR2863a	LOC_Os05g31210.1	154	-22.23	154	-22.23	561708	21	3660	1739
miR2863a	LOC_Os05g31600.1	158	-23.43	158	-23.43	561748	21	4182	3643
miR2863a	LOC_Os05g33120.1	170	-22.08	170	-22.08	561917	21	2666	2201
miR2863a	LOC_Os05g33810.1	152	-20.29	152	-20.29	561997	21	3045	709
miR2863a	LOC_Os05g35110.1	151	-22.22	151	-22.22	562147	21	1861	1240
miR2863a	LOC_Os05g35630.1	158	-22.28	158	-22.28	562210	21	450	353
miR2863a	LOC_Os05g38810.1	148	-20.6	148	-20.6	562551	21	4711	3879
miR2863a	LOC_Os05g39330.1	173	-22.49	173	-22.49	562612	21	3492	539
miR2863a	LOC_Os05g39390.1	161	-20.12	161	-20.12	562621	21	3323	447
miR2863a	LOC_Os05g39620.1	156	-21.25	156	-21.25	562650	21	4176	1649
miR2863a	LOC_Os05g39680.1	150	-20.02	150	-20.02	562656	21	4334	4261
miR2863a	LOC_Os05g40110.1	155	-20.25	155	-20.25	562700	21	4845	601

miR2863a	LOC_Os05g40180.1	156	-20.42	156	-20.42	562707	21	3982	3454
miR2863a	LOC_Os05g41150.1	163	-21.43	163	-21.43	562829	21	2562	1895
miR2863a	LOC_Os05g44250.1	150	-20.13	150	-20.13	563131	21	1812	155
miR2863a	LOC_Os05g44340.1	150	-20.94	150	-20.94	563149	21	3264	750
miR2863a	LOC_Os05g44380.1	178	-22.36	178	-22.36	563154	21	1693	16
miR2863a	LOC_Os05g45850.1	175	-22.32	175	-22.32	563330	21	5614	5259
miR2863a	LOC_Os05g45860.1	157	-20.46	157	-20.46	563331	21	2092	1447
miR2863a	LOC_Os05g46630.1	149	-21.12	149	-21.12	563431	21	1534	858
miR2863a	LOC_Os05g46630.2	149	-21.12	149	-21.12	563432	21	1891	1215
miR2863a	LOC_Os05g51860.1	157	-23.85	157	-23.85	564050	21	1734	1042
miR2863a	LOC_Os06g01390.1	145	-20.67	145	-20.67	564110	21	2785	1149
miR2863a	LOC_Os06g03030.1	154	-20.2	154	-20.2	564298	21	652	418
miR2863a	LOC_Os06g03380.1	163	-20.65	163	-20.65	564333	21	2575	1976
miR2863a	LOC_Os06g03550.1	152	-20.12	152	-20.12	564350	21	216	58
miR2863a	LOC_Os06g03560.1	157	-21.8	157	-21.8	564351	21	2666	288
miR2863a	LOC_Os06g03990.1	157	-22.42	157	-22.42	564409	21	2386	932
miR2863a	LOC_Os06g05680.1	154	-22.23	154	-22.23	564622	21	4803	2780
miR2863a	LOC_Os06g06802.1	154	-22.23	154	-22.23	564770	21	5712	3689
miR2863a	LOC_Os06g06920.1	174	-23.58	174	-23.58	564782	21	2433	987
miR2863a	LOC_Os06g07070.1	160	-20.98	160	-20.98	564800	21	1651	418
miR2863a	LOC_Os06g09370.1	150	-20.21	150	-20.21	565061	21	2221	726
miR2863a	LOC_Os06g09370.2	150	-20.21	150	-20.21	565062	21	2121	726
miR2863a	LOC_Os06g09370.3	150	-20.21	150	-20.21	565063	21	1990	726
miR2863a	LOC_Os06g09830.1	165	-21.65	165	-21.65	565114	21	928	60
miR2863a	LOC_Os06g10620.1	155	-20.24	155	-20.24	565206	21	2477	2253
miR2863a	LOC_Os06g10680.1	154	-21.3	154	-21.3	565210	21	297	12
miR2863a	LOC_Os06g15990.1	158	-24.03	158	-24.03	565799	21	2355	676
miR2863a	LOC_Os06g16240.1	151	-20.34	151	-20.34	565825	21	1722	1156
miR2863a	LOC_Os06g16640.1	145	-21.65	145	-21.65	565870	21	1485	863
miR2863a	LOC_Os06g16970.1	160	-20.97	160	-20.97	565903	21	276	139
miR2863a	LOC_Os06g19470.1	162	-24.01	162	-24.01	566098	21	3094	883
miR2863a	LOC_Os06g19470.2	162	-24.01	162	-24.01	566099	21	2968	883

miR2863a	LOC_Os06g19470.3	162	-24.01	162	-24.01	566100	21	3253	1168
miR2863a	LOC_Os06g19470.4	162	-24.01	162	-24.01	566101	21	2793	1168
miR2863a	LOC_Os06g20090.1	158	-21.49	158	-21.49	566167	21	5669	2193
miR2863a	LOC_Os06g20710.1	150	-21.43	150	-21.43	566234	21	2613	881
miR2863a	LOC_Os06g23410.1	167	-25.93	167	-25.93	566530	21	552	78
miR2863a	LOC_Os06g24120.1	165	-20.3	165	-20.3	566602	21	528	65
miR2863a	LOC_Os06g26150.1	161	-22.06	161	-22.06	566801	21	4839	3824
miR2863a	LOC_Os06g31710.1	149	-20.01	149	-20.01	567276	21	366	201
miR2863a	LOC_Os06g32590.1	159	-21.32	159	-21.32	567360	21	225	13
miR2863a	LOC_Os06g34910.1	144	-20.64	144	-20.64	567586	21	3702	2272
miR2863a	LOC_Os06g37085.1	156	-22.39	156	-22.39	567816	21	3193	515
miR2863a	LOC_Os06g37420.1	168	-23.33	168	-23.33	567849	21	1460	20
miR2863a	LOC_Os06g37420.2	168	-23.33	168	-23.33	567850	21	1499	45
miR2863a	LOC_Os06g37560.1	154	-21.28	154	-21.28	567865	21	3071	1701
miR2863a	LOC_Os06g38760.1	160	-20.56	160	-20.56	567985	21	3615	3572
miR2863a	LOC_Os06g39480.1	152	-21.46	152	-21.46	568060	21	3620	501
miR2863a	LOC_Os06g40090.1	157	-21.48	157	-21.48	568142	21	1062	1030
miR2863a	LOC_Os06g41340.1	165	-25.81	165	-25.81	568278	21	6168	4086
miR2863a	LOC_Os06g41480.1	161	-21.27	161	-21.27	568298	21	3902	3487
miR2863a	LOC_Os06g44340.1	149	-21.95	149	-21.95	568628	21	1281	672
miR2863a	LOC_Os06g46720.1	163	-20.2	163	-20.2	568910	21	4410	569
miR2863a	LOC_Os06g48320.1	150	-21.5	150	-21.5	569110	21	1609	16
miR2863a	LOC_Os06g49810.1	154	-22.71	154	-22.71	569310	21	2286	1701
miR2863a	LOC_Os06g51210.1	156	-20.63	156	-20.63	569477	21	1623	1137
miR2863a	LOC_Os06g51250.1	160	-20.78	160	-20.78	569484	21	4122	2310
miR2863a	LOC_Os06g51270.1	157	-20.31	157	-20.31	569487	21	5949	3670
miR2863a	LOC_Os06g51540.1	154	-23.19	154	-23.19	569533	21	2493	1986
miR2863a	LOC_Os07g04210.1	162	-21.38	162	-21.38	569887	21	2979	2798
miR2863a	LOC_Os07g05380.1	168	-21.15	168	-21.15	570011	21	1371	1069
miR2863a	LOC_Os07g05410.1	159	-20.55	159	-20.55	570014	21	450	191
miR2863a	LOC_Os07g05690.1	154	-20.01	154	-20.01	570050	21	1515	1339
miR2863a	LOC_Os07g06400.1	156	-20.69	156	-20.69	570118	21	603	55

miR2863a	LOC_Os07g11010.1	168	-21.8	168	-21.8	570640	21	2458	811
miR2863a	LOC_Os07g11010.2	168	-21.8	168	-21.8	570641	21	2383	736
miR2863a	LOC_Os07g11840.1	150	-21.77	150	-21.77	570724	21	1365	335
miR2863a	LOC_Os07g12640.1	166	-21.24	166	-21.24	570808	21	1822	1238
miR2863a	LOC_Os07g13430.1	167	-23.5	167	-23.5	570888	21	2247	547
miR2863a	LOC_Os07g15430.1	144	-21.85	144	-21.85	571102	21	1834	887
miR2863a	LOC_Os07g15710.1	160	-21.3	160	-21.3	571133	21	1632	630
miR2863a	LOC_Os07g17260.1	308	-42.7	154	-22.23	571283	21	5431	2208 3480
miR2863a	LOC_Os07g17260.2	308	-42.7	154	-22.23	571284	21	5288	2065 3337
miR2863a	LOC_Os07g18970.1	154	-22.23	154	-22.23	571452	21	4947	2780
miR2863a	LOC_Os07g22460.1	156	-21.82	156	-21.82	571648	21	588	460
miR2863a	LOC_Os07g22710.4	158	-26.75	158	-26.75	571689	21	4311	1134
miR2863a	LOC_Os07g23770.1	146	-20.62	146	-20.62	571798	21	630	427
miR2863a	LOC_Os07g24190.1	157	-25.09	157	-25.09	571847	21	4525	746
miR2863a	LOC_Os07g24190.2	157	-25.09	157	-25.09	571848	21	3487	746
miR2863a	LOC_Os07g24190.3	157	-25.09	157	-25.09	571849	21	4622	746
miR2863a	LOC_Os07g24590.1	154	-20.38	154	-20.38	571886	21	5211	4612
miR2863a	LOC_Os07g24690.1	149	-21.66	149	-21.66	571895	21	2022	1888
miR2863a	LOC_Os07g25000.1	146	-20.57	146	-20.57	571933	21	1089	358
miR2863a	LOC_Os07g26800.1	151	-22.27	151	-22.27	572118	21	2202	2036
miR2863a	LOC_Os07g27690.1	147	-21.66	147	-21.66	572213	21	1620	1462
miR2863a	LOC_Os07g27730.1	169	-23.24	169	-23.24	572217	21	2250	919
miR2863a	LOC_Os07g29240.1	161	-22.84	161	-22.84	572375	21	898	294
miR2863a	LOC_Os07g30250.1	158	-20.22	158	-20.22	572491	21	3206	2703
miR2863a	LOC_Os07g30670.1	157	-20.02	157	-20.02	572528	21	972	700
miR2863a	LOC_Os07g30670.2	157	-20.02	157	-20.02	572529	21	1008	736
miR2863a	LOC_Os07g30670.3	157	-20.02	157	-20.02	572530	21	1194	922
miR2863a	LOC_Os07g32650.1	159	-25.22	159	-25.22	572743	21	3763	1875
miR2863a	LOC_Os07g33450.1	330	-42.76	165	-21.38	572828	21	4422	516 4104
miR2863a	LOC_Os07g35050.1	154	-20.49	154	-20.49	572997	21	1793	1250
miR2863a	LOC_Os07g35060.1	154	-20.49	154	-20.49	572998	21	1737	1212
miR2863a	LOC_Os07g35060.2	154	-20.49	154	-20.49	572999	21	1718	1193

miR2863a	LOC_Os07g35100.1	165	-25.81	165	-25.81	573003	21	5322	3720
miR2863a	LOC_Os07g37550.1	159	-22.29	159	-22.29	573281	21	1194	296
miR2863a	LOC_Os07g38890.1	158	-22.72	158	-22.72	573436	21	2031	139
miR2863a	LOC_Os07g39780.1	147	-21.54	147	-21.54	573541	21	2466	599
miR2863a	LOC_Os07g39780.2	147	-21.54	147	-21.54	573542	21	2514	647
miR2863a	LOC_Os07g39780.3	147	-21.54	147	-21.54	573543	21	2701	647
miR2863a	LOC_Os07g41380.1	170	-21.01	170	-21.01	573717	21	3186	1472
miR2863a	LOC_Os07g42310.1	154	-21.63	154	-21.63	573821	21	2022	1718
miR2863a	LOC_Os07g42640.1	147	-22.13	147	-22.13	573866	21	1482	1427
miR2863a	LOC_Os07g43200.1	159	-21.66	159	-21.66	573943	21	4737	207
miR2863a	LOC_Os07g43220.1	155	-21.39	155	-21.39	573945	21	666	504
miR2863a	LOC_Os07g43230.1	159	-21.66	159	-21.66	573946	21	786	468
miR2863a	LOC_Os07g44210.1	159	-21.43	159	-21.43	574052	21	2499	2089
miR2863a	LOC_Os07g44960.2	171	-21.49	171	-21.49	574136	21	1525	1201
miR2863a	LOC_Os07g45090.1	161	-22.78	161	-22.78	574151	21	2424	1208
miR2863a	LOC_Os07g45570.1	162	-20.24	162	-20.24	574197	21	3395	2370
miR2863a	LOC_Os07g48970.1	157	-22.67	157	-22.67	574648	21	2287	867
miR2863a	LOC_Os07g48970.2	157	-22.67	157	-22.67	574649	21	3104	905
miR2863a	LOC_Os07g49230.1	165	-28.36	165	-28.36	574676	21	3642	3181
miR2863a	LOC_Os07g49230.2	165	-28.36	165	-28.36	574677	21	3581	3060
miR2863a	LOC_Os08g01470.1	154	-21.03	154	-21.03	574786	21	1023	8
miR2863a	LOC_Os08g01780.2	148	-20.06	148	-20.06	574826	21	3103	876
miR2863a	LOC_Os08g05640.2	175	-21.59	175	-21.59	575266	21	2107	138
miR2863a	LOC_Os08g07390.1	158	-21.1	158	-21.1	575459	21	2055	706
miR2863a	LOC_Os08g07774.1	163	-20.2	163	-20.2	575497	21	4109	1232
miR2863a	LOC_Os08g07890.1	163	-20.2	163	-20.2	575510	21	5061	1002
miR2863a	LOC_Os08g07940.1	171	-22.66	171	-22.66	575515	21	3399	1473
miR2863a	LOC_Os08g08210.1	159	-21.04	159	-21.04	575548	21	3259	256
miR2863a	LOC_Os08g09230.1	168	-21.53	168	-21.53	575658	21	5766	3953
miR2863a	LOC_Os08g09230.2	168	-21.53	168	-21.53	575659	21	5764	3953
miR2863a	LOC_Os08g09990.1	153	-20.22	153	-20.22	575744	21	663	132
miR2863a	LOC_Os08g10070.1	159	-21.13	159	-21.13	575755	21	1720	367

miR2863a	LOC_Os08g10070.2	159	-21.13	159	-21.13	575756	21	2133	780
miR2863a	LOC_Os08g10920.1	159	-20.16	159	-20.16	575851	21	1933	789
miR2863a	LOC_Os08g10970.1	315	-44.22	161	-22.73	575856	21	1368	826 342
miR2863a	LOC_Os08g11930.1	152	-21.29	152	-21.29	575958	21	5352	1386
miR2863a	LOC_Os08g13460.1	149	-20.4	149	-20.4	576121	21	640	282
miR2863a	LOC_Os08g15010.1	154	-22.23	154	-22.23	576285	21	5484	2780
miR2863a	LOC_Os08g15090.1	167	-22.44	167	-22.44	576294	21	3550	1733
miR2863a	LOC_Os08g16160.1	163	-23.26	163	-23.26	576420	21	2259	553
miR2863a	LOC_Os08g16550.1	157	-23.79	157	-23.79	576464	21	990	516
miR2863a	LOC_Os08g19860.1	165	-22.93	165	-22.93	576760	21	1854	1078
miR2863a	LOC_Os08g29720.2	158	-22.15	158	-22.15	577747	21	1846	1473
miR2863a	LOC_Os08g30580.1	161	-20.84	161	-20.84	577833	21	207	96
miR2863a	LOC_Os08g30730.1	162	-24.88	162	-24.88	577848	21	1358	808
miR2863a	LOC_Os08g30740.1	162	-24.88	162	-24.88	577850	21	3300	423
miR2863a	LOC_Os08g31900.1	146	-20.37	146	-20.37	577978	21	687	429
miR2863a	LOC_Os08g32980.2	157	-22.81	157	-22.81	578097	21	957	65
miR2863a	LOC_Os08g34370.1	160	-22.85	160	-22.85	578258	21	1572	975
miR2863a	LOC_Os08g34460.1	159	-20.55	159	-20.55	578268	21	1062	474
miR2863a	LOC_Os08g39120.1	165	-21.6	165	-21.6	578821	21	2382	1083
miR2863a	LOC_Os08g40590.1	149	-21.3	149	-21.3	578996	21	1777	1109
miR2863a	LOC_Os08g40590.2	149	-21.3	149	-21.3	578997	21	1590	922
miR2863a	LOC_Os08g41440.1	152	-24.03	152	-24.03	579104	21	2014	1734
miR2863a	LOC_Os08g41830.1	152	-20.07	152	-20.07	579149	21	2080	440
miR2863a	LOC_Os08g43000.1	156	-21.02	156	-21.02	579297	21	3633	425
miR2863a	LOC_Os09g02700.1	158	-22.15	158	-22.15	579773	21	2523	1116
miR2863a	LOC_Os09g03100.1	169	-23.27	169	-23.27	579814	21	2283	1141
miR2863a	LOC_Os09g03600.1	153	-20.84	153	-20.84	579863	21	3054	524
miR2863a	LOC_Os09g03620.1	153	-20.28	153	-20.28	579868	21	2412	1269
miR2863a	LOC_Os09g04710.1	151	-20.77	151	-20.77	579978	21	1400	946
miR2863a	LOC_Os09g04710.2	151	-20.77	151	-20.77	579979	21	1376	922
miR2863a	LOC_Os09g04840.1	161	-21.59	161	-21.59	579993	21	930	397
miR2863a	LOC_Os09g04920.1	163	-23.56	163	-23.56	580002	21	490	151

miR2863a	LOC_Os09g06150.1	161	-20.19	161	-20.19	580027	21	6033	5296
miR2863a	LOC_Os09g06400.1	158	-21.79	158	-21.79	580052	21	1104	204
miR2863a	LOC_Os09g11590.1	172	-22.36	172	-22.36	580600	21	162	60
miR2863a	LOC_Os09g14120.1	155	-20.06	155	-20.06	580861	21	4125	3227
miR2863a	LOC_Os09g14500.1	154	-22.23	154	-22.23	580898	21	4806	2780
miR2863a	LOC_Os09g15010.1	161	-21.41	161	-21.41	580958	21	3999	3551
miR2863a	LOC_Os09g17710.1	153	-20.25	153	-20.25	581244	21	1212	356
miR2863a	LOC_Os09g17740.1	159	-22.29	159	-22.29	581248	21	1479	379
miR2863a	LOC_Os09g17810.1	145	-20.84	145	-20.84	581256	21	2176	1079
miR2863a	LOC_Os09g20020.1	152	-21.75	152	-21.75	581424	21	3681	588
miR2863a	LOC_Os09g22030.1	157	-23.79	157	-23.79	581644	21	4512	4038
miR2863a	LOC_Os09g22540.1	170	-22.44	170	-22.44	581693	21	1290	1190
miR2863a	LOC_Os09g24260.1	166	-21.86	166	-21.86	581826	21	3369	2414
miR2863a	LOC_Os09g25580.1	157	-20.25	157	-20.25	581971	21	1943	1042
miR2863a	LOC_Os09g28050.2	153	-20.96	153	-20.96	582273	21	2027	452
miR2863a	LOC_Os09g28050.3	153	-20.96	153	-20.96	582274	21	2255	680
miR2863a	LOC_Os09g28680.1	165	-25.81	165	-25.81	582341	21	5460	3375
miR2863a	LOC_Os09g29060.1	164	-23.94	164	-23.94	582385	21	1502	751
miR2863a	LOC_Os09g29404.1	152	-20.56	152	-20.56	582421	21	3443	3407
miR2863a	LOC_Os09g29510.1	154	-21.6	154	-21.6	582436	21	2271	810
miR2863a	LOC_Os09g29810.1	154	-22.23	154	-22.23	582465	21	5283	2780
miR2863a	LOC_Os09g32210.1	150	-20.67	150	-20.67	582697	21	3589	1982
miR2863a	LOC_Os09g32650.1	148	-20.86	148	-20.86	582755	21	3713	1591
miR2863a	LOC_Os09g34110.2	167	-22.99	167	-22.99	582914	21	4663	2069
miR2863a	LOC_Os09g36090.1	162	-22.17	162	-22.17	583036	21	5924	5014
miR2863a	LOC_Os09g38200.1	169	-23.5	169	-23.5	583285	21	3888	1902
miR2863a	LOC_Os09g38570.1	161	-21.58	161	-21.58	583325	21	2434	1647
miR2863a	LOC_Os09g39660.1	161	-24.41	161	-24.41	583459	21	2284	896
miR2863a	LOC_Os09g39660.2	161	-24.41	161	-24.41	583460	21	2305	917
miR2863a	LOC_Os09g39660.3	161	-24.41	161	-24.41	583461	21	2498	1110
miR2863a	LOC_Os09g39680.1	161	-22.21	161	-22.21	583463	21	2608	562
miR2863a	LOC_Os09g39710.1	161	-20.74	161	-20.74	583466	21	1158	504

miR2863a	LOC_Os10g01890.1	160	-22.67	160	-22.67	583604	21	3144	2621
miR2863a	LOC_Os10g05710.1	158	-20.14	158	-20.14	583994	21	3678	2318
miR2863a	LOC_Os10g07380.1	156	-21.4	156	-21.4	584151	21	707	657
miR2863a	LOC_Os10g12220.1	147	-21.66	147	-21.66	584620	21	2499	2338
miR2863a	LOC_Os10g12460.1	158	-23.83	158	-23.83	584638	21	1305	730
miR2863a	LOC_Os10g13800.1	148	-20.54	148	-20.54	584726	21	1556	65
miR2863a	LOC_Os10g13800.2	148	-20.54	148	-20.54	584727	21	767	81
miR2863a	LOC_Os10g14230.1	326	-45.25	163	-23.05	584773	21	2214	1824 2189
miR2863a	LOC_Os10g14795.1	165	-23.76	165	-23.76	584780	21	4161	3688
miR2863a	LOC_Os10g16800.1	150	-20.8	150	-20.8	584884	21	1962	779
miR2863a	LOC_Os10g17300.1	173	-24.75	173	-24.75	584929	21	2472	1096
miR2863a	LOC_Os10g17570.1	154	-22.23	154	-22.23	584954	21	2805	899
miR2863a	LOC_Os10g18099.1	161	-20.76	161	-20.76	585004	21	831	713
miR2863a	LOC_Os10g21326.1	164	-23.95	164	-23.95	585302	21	478	49
miR2863a	LOC_Os10g22010.1	165	-20.19	165	-20.19	585388	21	6108	5249
miR2863a	LOC_Os10g24220.1	165	-23.56	165	-23.56	585577	21	2463	1009
miR2863a	LOC_Os10g24900.1	163	-22.94	163	-22.94	585644	21	1654	1331
miR2863a	LOC_Os10g25080.1	154	-20.38	154	-20.38	585659	21	5352	4753
miR2863a	LOC_Os10g25487.1	159	-22.19	159	-22.19	585697	21	4894	2185
miR2863a	LOC_Os10g26540.1	152	-20.71	152	-20.71	585809	21	866	246
miR2863a	LOC_Os10g29810.1	157	-23.11	157	-23.11	586106	21	1194	1134
miR2863a	LOC_Os10g29920.1	154	-21.23	154	-21.23	586114	21	1116	1056
miR2863a	LOC_Os10g31290.1	161	-28.25	161	-28.25	586252	21	3042	2751
miR2863a	LOC_Os10g31690.1	167	-24.36	167	-24.36	586284	21	609	520
miR2863a	LOC_Os10g31930.1	166	-21.23	166	-21.23	586321	21	1714	418
miR2863a	LOC_Os10g33440.2	161	-20.36	161	-20.36	586465	21	5941	2345
miR2863a	LOC_Os10g33640.1	174	-25.91	174	-25.91	586485	21	1587	1242
miR2863a	LOC_Os10g33650.1	167	-22.01	167	-22.01	586486	21	2373	1432
miR2863a	LOC_Os10g33650.2	167	-22.01	167	-22.01	586487	21	2254	1380
miR2863a	LOC_Os10g33650.3	167	-22.01	167	-22.01	586488	21	2310	1436
miR2863a	LOC_Os10g33680.1	151	-20.77	151	-20.77	586491	21	2615	1972
miR2863a	LOC_Os10g35090.1	160	-20.75	160	-20.75	586655	21	3694	2208

miR2863a	LOC_Os10g36650.1	162	-25.92	162	-25.92	586833	21	1840	332
miR2863a	LOC_Os10g37760.1	163	-23.01	163	-23.01	586942	21	1468	427
miR2863a	LOC_Os10g37760.2	163	-23.01	163	-23.01	586943	21	1332	427
miR2863a	LOC_Os10g39150.1	174	-24.87	174	-24.87	587094	21	965	525
miR2863a	LOC_Os10g39520.1	165	-25.18	165	-25.18	587135	21	2075	317
miR2863a	LOC_Os10g39640.1	165	-21.37	165	-21.37	587155	21	1653	1605
miR2863a	LOC_Os10g40410.1	161	-23.27	161	-23.27	587255	21	958	225
miR2863a	LOC_Os10g40410.2	161	-23.27	161	-23.27	587256	21	862	225
miR2863a	LOC_Os10g41240.1	156	-21.28	156	-21.28	587348	21	1741	96
miR2863a	LOC_Os10g41838.1	153	-23.16	153	-23.16	587422	21	1813	99
miR2863a	LOC_Os10g42299.1	156	-20.73	156	-20.73	587480	21	2934	728
miR2863a	LOC_Os10g42299.2	156	-20.73	156	-20.73	587481	21	3298	728
miR2863a	LOC_Os10g42299.3	156	-20.73	156	-20.73	587482	21	2619	413
miR2863a	LOC_Os10g42299.4	156	-20.73	156	-20.73	587483	21	2863	728
miR2863a	LOC_Os10g42299.5	156	-20.73	156	-20.73	587484	21	3192	728
miR2863a	LOC_Os11g01154.4	157	-22.48	157	-22.48	587605	21	1978	143
miR2863a	LOC_Os11g02780.1	158	-20.23	158	-20.23	587799	21	5157	1706
miR2863a	LOC_Os11g03734.1	154	-20.04	154	-20.04	587904	21	2432	837
miR2863a	LOC_Os11g05590.1	141	-20.85	141	-20.85	588130	21	1362	1132
miR2863a	LOC_Os11g07060.1	160	-20.24	160	-20.24	588309	21	3785	2110
miR2863a	LOC_Os11g07060.2	160	-20.24	160	-20.24	588310	21	3699	2024
miR2863a	LOC_Os11g07060.3	160	-20.24	160	-20.24	588311	21	3815	2140
miR2863a	LOC_Os11g07060.4	160	-20.24	160	-20.24	588312	21	3789	2114
miR2863a	LOC_Os11g07060.5	160	-20.24	160	-20.24	588313	21	3703	2110
miR2863a	LOC_Os11g08090.1	161	-20.4	161	-20.4	588429	21	6768	434
miR2863a	LOC_Os11g08620.1	167	-24.04	167	-24.04	588487	21	2355	1017
miR2863a	LOC_Os11g09670.1	162	-22.16	162	-22.16	588592	21	1634	749
miR2863a	LOC_Os11g09670.2	162	-22.16	162	-22.16	588593	21	1751	866
miR2863a	LOC_Os11g11000.1	149	-21.62	149	-21.62	588737	21	2596	1008
miR2863a	LOC_Os11g11000.2	149	-21.62	149	-21.62	588738	21	2484	1008
miR2863a	LOC_Os11g11000.3	149	-21.62	149	-21.62	588739	21	2591	1008
miR2863a	LOC_Os11g11350.1	153	-20.64	153	-20.64	588774	21	2532	1501

miR2863a	LOC_Os11g12760.1	151	-20.6	151	-20.6	588917	21	1137	314
miR2863a	LOC_Os11g13694.1	157	-21.3	157	-21.3	588958	21	1578	811
miR2863a	LOC_Os11g15070.1	169	-24.88	169	-24.88	589123	21	2052	1102
miR2863a	LOC_Os11g17630.1	153	-20.71	153	-20.71	589341	21	591	337
miR2863a	LOC_Os11g18800.1	155	-20.54	155	-20.54	589444	21	582	9
miR2863a	LOC_Os11g19330.1	160	-21.3	160	-21.3	589501	21	2106	753
miR2863a	LOC_Os11g19380.1	161	-21.11	161	-21.11	589506	21	2625	1718
miR2863a	LOC_Os11g22290.1	174	-25.09	174	-25.09	589703	21	363	230
miR2863a	LOC_Os11g23060.1	162	-21.82	162	-21.82	589778	21	2817	60
miR2863a	LOC_Os11g24080.1	147	-20.13	147	-20.13	589847	21	189	74
miR2863a	LOC_Os11g25630.1	160	-23.25	160	-23.25	589989	21	1278	401
miR2863a	LOC_Os11g30210.1	156	-21.32	156	-21.32	590443	21	2820	322
miR2863a	LOC_Os11g31210.1	153	-22.9	153	-22.9	590532	21	1665	288
miR2863a	LOC_Os11g31590.1	152	-22.11	152	-22.11	590570	21	1428	1239
miR2863a	LOC_Os11g33260.1	162	-24.24	162	-24.24	590761	21	1708	396
miR2863a	LOC_Os11g34350.1	167	-24.13	167	-24.13	590835	21	2567	1337
miR2863a	LOC_Os11g34660.1	159	-20.83	159	-20.83	590867	21	1415	163
miR2863a	LOC_Os11g35530.1	163	-23.26	163	-23.26	590960	21	2175	553
miR2863a	LOC_Os11g35660.1	147	-20.29	147	-20.29	590972	21	2931	2857
miR2863a	LOC_Os11g35910.1	165	-24.54	165	-24.54	591001	21	2682	1186
miR2863a	LOC_Os11g36240.1	161	-28.01	161	-28.01	591042	21	1116	889
miR2863a	LOC_Os11g36760.1	150	-20.62	150	-20.62	591093	21	2550	1262
miR2863a	LOC_Os11g37260.1	161	-24.07	161	-24.07	591142	21	2897	1410
miR2863a	LOC_Os11g37940.1	170	-23.52	170	-23.52	591215	21	958	474
miR2863a	LOC_Os11g37960.1	166	-23.49	166	-23.49	591218	21	737	410
miR2863a	LOC_Os11g38610.2	159	-21.11	159	-21.11	591283	21	3509	259
miR2863a	LOC_Os11g40410.1	172	-20.9	172	-20.9	591483	21	2619	1774
miR2863a	LOC_Os11g40410.2	172	-20.9	172	-20.9	591484	21	2916	2067
miR2863a	LOC_Os11g40420.1	172	-20.9	172	-20.9	591485	21	2803	1617
miR2863a	LOC_Os11g40590.1	154	-20.45	154	-20.45	591508	21	3972	2515
miR2863a	LOC_Os11g40590.2	154	-20.45	154	-20.45	591509	21	3236	1779
miR2863a	LOC_Os11g40590.3	154	-20.45	154	-20.45	591510	21	3241	1779

miR2863a	LOC_Os11g40810.1	158	-23.03	158	-23.03	591534	21	2211	1060
miR2863a	LOC_Os11g41470.1	161	-24.13	161	-24.13	591596	21	2196	1860
miR2863a	LOC_Os11g42080.1	141	-23.06	141	-23.06	591663	21	2196	1980
miR2863a	LOC_Os11g42630.1	154	-21.8	154	-21.8	591722	21	6291	3113
miR2863a	LOC_Os11g44050.1	147	-21.66	147	-21.66	591875	21	2466	2305
miR2863a	LOC_Os11g44190.1	154	-22.23	154	-22.23	591888	21	4167	2372
miR2863a	LOC_Os11g46040.1	153	-21.76	153	-21.76	592083	21	3957	2055
miR2863a	LOC_Os11g47330.1	153	-20.15	153	-20.15	592166	21	1761	585
miR2863a	LOC_Os11g48060.1	154	-20.47	154	-20.47	592268	21	2496	1614
miR2863a	LOC_Os12g01150.1	157	-22.48	157	-22.48	592295	21	363	129
miR2863a	LOC_Os12g04840.1	165	-25.58	165	-25.58	592747	21	4419	3959
miR2863a	LOC_Os12g04910.1	146	-20.71	146	-20.71	592756	21	4056	3325
miR2863a	LOC_Os12g05310.1	334	-42.61	170	-22.08	592805	21	7326	3050 931
miR2863a	LOC_Os12g06630.1	161	-23.25	161	-23.25	592948	21	1978	918
miR2863a	LOC_Os12g06630.2	161	-23.25	161	-23.25	592949	21	2025	918
miR2863a	LOC_Os12g06630.3	161	-23.25	161	-23.25	592950	21	2076	918
miR2863a	LOC_Os12g07750.1	162	-20.36	162	-20.36	593075	21	4671	1187
miR2863a	LOC_Os12g07810.1	158	-22.42	158	-22.42	593082	21	1712	583
miR2863a	LOC_Os12g08780.1	153	-20.12	153	-20.12	593192	21	1156	859
miR2863a	LOC_Os12g09460.1	158	-20.68	158	-20.68	593262	21	405	271
miR2863a	LOC_Os12g10900.1	154	-22.23	154	-22.23	593418	21	5388	3362
miR2863a	LOC_Os12g11210.1	150	-20.13	150	-20.13	593448	21	5610	3980
miR2863a	LOC_Os12g16650.1	165	-22.13	165	-22.13	594005	21	3110	1964
miR2863a	LOC_Os12g16650.2	165	-22.13	165	-22.13	594006	21	3127	1981
miR2863a	LOC_Os12g20144.1	154	-22.69	154	-22.69	594362	21	2288	1337
miR2863a	LOC_Os12g20169.1	159	-21.58	159	-21.58	594365	21	3504	2151
miR2863a	LOC_Os12g20470.1	146	-20.57	146	-20.57	594401	21	4575	3844
miR2863a	LOC_Os12g22240.1	158	-20.32	158	-20.32	594483	21	1119	498
miR2863a	LOC_Os12g22730.1	155	-20.03	155	-20.03	594531	21	666	68
miR2863a	LOC_Os12g28100.1	164	-20.16	164	-20.16	595057	21	4547	1213
miR2863a	LOC_Os12g32110.1	163	-23.26	163	-23.26	595454	21	2196	382
miR2863a	LOC_Os12g32440.1	151	-21.21	151	-21.21	595488	21	2490	2462

miR2863a	LOC_Os12g34022.1	154	-21.63	154	-21.63	595649	21	1062	274
miR2863a	LOC_Os12g34096.1	154	-20.06	154	-20.06	595661	21	913	433
miR2863a	LOC_Os12g34260.1	150	-20.13	150	-20.13	595684	21	2619	290
miR2863a	LOC_Os12g34650.1	154	-21.07	154	-21.07	595725	21	4896	4076
miR2863a	LOC_Os12g34860.1	153	-21.28	153	-21.28	595747	21	2115	706
miR2863a	LOC_Os12g34860.2	153	-21.28	153	-21.28	595748	21	1072	778
miR2863a	LOC_Os12g35040.1	161	-20.2	161	-20.2	595773	21	1772	1007
miR2863a	LOC_Os12g35380.1	329	-45.02	166	-23.26	595809	21	2067	1691 553
miR2863a	LOC_Os12g35895.1	161	-21.16	161	-21.16	595867	21	4294	1419
miR2863a	LOC_Os12g37519.2	169	-22.23	169	-22.23	596040	21	880	704
miR2863a	LOC_Os12g38210.1	157	-20.85	157	-20.85	596118	21	2519	1157
miR2863a	LOC_Os12g40790.1	159	-20.82	159	-20.82	596423	21	3756	1700
miR2863a	LOC_Os12g43160.1	161	-23.36	161	-23.36	596715	21	1257	895
miR2863a	ChrUn.fgenesh.mRNA.20	162	-24.14	162	-24.14	596877	21	1803	1346
miR2863a	ChrSy.fgenesh.mRNA.7	154	-22.23	154	-22.23	596960	21	2670	737
miR2863a	ChrSy.fgenesh.mRNA.69	149	-23.77	149	-23.77	597022	21	3090	2077
miR1874-3p	LOC_Os01g01070.1	160	-23.77	160	-23.77	729730	24	1568	654
miR1874-3p	LOC_Os01g01070.2	160	-23.77	160	-23.77	729731	24	1562	648
miR1874-3p	LOC_Os01g01070.3	160	-23.77	160	-23.77	729732	24	1369	648
miR1874-3p	LOC_Os01g01312.1	165	-26.73	165	-26.73	729763	24	4165	84
miR1874-3p	LOC_Os01g01369.1	155	-20.29	155	-20.29	729771	24	1545	435
miR1874-3p	LOC_Os01g01430.1	164	-25.94	164	-25.94	729784	24	1425	792
miR1874-3p	LOC_Os01g01620.1	166	-23.4	166	-23.4	729807	24	1609	186
miR1874-3p	LOC_Os01g01680.1	160	-20.39	160	-20.39	729813	24	1023	937
miR1874-3p	LOC_Os01g01850.1	151	-25.25	151	-25.25	729833	24	6084	2556
miR1874-3p	LOC_Os01g01860.1	141	-22.41	141	-22.41	729834	24	3144	2890
miR1874-3p	LOC_Os01g01900.1	157	-21.89	157	-21.89	729838	24	3243	1677
miR1874-3p	LOC_Os01g01970.1	156	-22.42	156	-22.42	729847	24	1151	13
miR1874-3p	LOC_Os01g01970.2	156	-22.42	156	-22.42	729848	24	1089	13
miR1874-3p	LOC_Os01g02000.1	155	-24.13	155	-24.13	729851	24	2767	1557
miR1874-3p	LOC_Os01g02040.1	149	-20.64	149	-20.64	729855	24	3085	820
miR1874-3p	LOC_Os01g02040.2	149	-20.64	149	-20.64	729856	24	2608	343

miR1874-3p	LOC_Os01g02050.1	157	-27	157	-27	729857	24	3492	2547
miR1874-3p	LOC_Os01g02210.1	156	-20.49	156	-20.49	729880	24	1935	280
miR1874-3p	LOC_Os01g02240.1	154	-23.53	154	-23.53	729883	24	4758	4379
miR1874-3p	LOC_Os01g02270.1	170	-21.94	170	-21.94	729886	24	3504	1816
miR1874-3p	LOC_Os01g02740.1	458	-73.08	157	-28.9	729936	24	8799	6605 4024 3045
miR1874-3p	LOC_Os01g02770.1	180	-30.02	180	-30.02	729939	24	1992	668
miR1874-3p	LOC_Os01g02940.1	157	-20.7	157	-20.7	729957	24	2623	396
miR1874-3p	LOC_Os01g02940.2	157	-20.7	157	-20.7	729958	24	2650	396
miR1874-3p	LOC_Os01g02940.3	157	-20.7	157	-20.7	729959	24	2663	396
miR1874-3p	LOC_Os01g02940.4	157	-20.7	157	-20.7	729960	24	2715	396
miR1874-3p	LOC_Os01g02940.6	157	-20.7	157	-20.7	729961	24	2709	396
miR1874-3p	LOC_Os01g03050.1	166	-26.59	166	-26.59	729973	24	1494	825
miR1874-3p	LOC_Os01g03110.1	161	-20.83	161	-20.83	729984	24	2056	925
miR1874-3p	LOC_Os01g03144.1	158	-25.29	158	-25.29	729986	24	3584	2277
miR1874-3p	LOC_Os01g03452.1	159	-22.62	159	-22.62	730017	24	3684	159
miR1874-3p	LOC_Os01g03750.1	155	-20.42	155	-20.42	730045	24	895	595
miR1874-3p	LOC_Os01g04190.1	156	-22.3	156	-22.3	730091	24	2317	1039
miR1874-3p	LOC_Os01g04190.2	156	-22.3	156	-22.3	730092	24	2295	1017
miR1874-3p	LOC_Os01g04190.3	156	-22.3	156	-22.3	730093	24	2339	1061
miR1874-3p	LOC_Os01g04190.4	156	-22.3	156	-22.3	730094	24	2414	1061
miR1874-3p	LOC_Os01g04510.1	162	-23.56	162	-23.56	730127	24	567	500
miR1874-3p	LOC_Os01g04520.1	152	-30.12	152	-30.12	730128	24	1881	1432
miR1874-3p	LOC_Os01g04630.1	161	-20.3	161	-20.3	730143	24	1746	644
miR1874-3p	LOC_Os01g04660.1	160	-23.6	160	-23.6	730146	24	1577	280
miR1874-3p	LOC_Os01g04660.2	160	-23.6	160	-23.6	730147	24	1703	280
miR1874-3p	LOC_Os01g04780.1	141	-22.41	141	-22.41	730160	24	8220	7966
miR1874-3p	LOC_Os01g05960.1	149	-21.91	149	-21.91	730293	24	3430	2629
miR1874-3p	LOC_Os01g06300.1	154	-23.58	154	-23.58	730332	24	4458	4079
miR1874-3p	LOC_Os01g06510.1	160	-20.87	160	-20.87	730355	24	2153	1613
miR1874-3p	LOC_Os01g06720.1	146	-21.56	146	-21.56	730382	24	2721	1497
miR1874-3p	LOC_Os01g06900.1	157	-22.54	157	-22.54	730399	24	3093	76
miR1874-3p	LOC_Os01g07110.1	156	-24.07	156	-24.07	730423	24	4550	4255

miR1874-3p	LOC_Os01g07170.1	153	-20.16	153	-20.16	730431	24	1659	1355
miR1874-3p	LOC_Os01g07400.1	159	-21.63	159	-21.63	730464	24	2011	1880
miR1874-3p	LOC_Os01g07400.2	159	-21.63	159	-21.63	730465	24	2156	2025
miR1874-3p	LOC_Os01g07700.1	164	-22.83	164	-22.83	730493	24	1676	909
miR1874-3p	LOC_Os01g07700.2	164	-22.83	164	-22.83	730494	24	1673	896
miR1874-3p	LOC_Os01g07700.3	164	-22.83	164	-22.83	730495	24	1757	896
miR1874-3p	LOC_Os01g08190.3	160	-25.92	160	-25.92	730554	24	3503	2491
miR1874-3p	LOC_Os01g08520.1	155	-26.55	155	-26.55	730590	24	1648	760
miR1874-3p	LOC_Os01g08540.1	148	-20.24	148	-20.24	730592	24	4059	1837
miR1874-3p	LOC_Os01g08550.1	159	-22.33	159	-22.33	730593	24	1968	1552
miR1874-3p	LOC_Os01g09050.1	151	-20.89	151	-20.89	730657	24	3321	1400
miR1874-3p	LOC_Os01g09420.1	158	-20.63	158	-20.63	730698	24	1890	57
miR1874-3p	LOC_Os01g09665.1	158	-26.39	158	-26.39	730729	24	1275	292
miR1874-3p	LOC_Os01g09770.1	154	-22.01	154	-22.01	730742	24	2078	1625
miR1874-3p	LOC_Os01g10000.1	152	-21.47	152	-21.47	730763	24	393	295
miR1874-3p	LOC_Os01g10030.1	161	-25.84	161	-25.84	730767	24	2923	2518
miR1874-3p	LOC_Os01g10110.1	152	-20.26	152	-20.26	730778	24	2586	1293
miR1874-3p	LOC_Os01g10320.1	154	-21.16	154	-21.16	730806	24	2158	2020
miR1874-3p	LOC_Os01g10890.1	153	-20.24	153	-20.24	730865	24	2061	1995
miR1874-3p	LOC_Os01g11230.1	144	-20.48	144	-20.48	730898	24	1055	340
miR1874-3p	LOC_Os01g11230.2	144	-20.48	144	-20.48	730899	24	990	340
miR1874-3p	LOC_Os01g11390.1	158	-21.64	158	-21.64	730913	24	408	387
miR1874-3p	LOC_Os01g11780.1	156	-29.23	156	-29.23	730951	24	648	395
miR1874-3p	LOC_Os01g11952.1	152	-23.26	152	-23.26	730969	24	3659	2558
miR1874-3p	LOC_Os01g12790.1	168	-22.73	168	-22.73	731072	24	3756	526
miR1874-3p	LOC_Os01g13030.2	169	-23.99	169	-23.99	731105	24	1395	1158
miR1874-3p	LOC_Os01g13150.1	159	-22.42	159	-22.42	731123	24	3469	3021
miR1874-3p	LOC_Os01g13350.1	157	-21.77	157	-21.77	731142	24	2643	1375
miR1874-3p	LOC_Os01g13350.2	157	-21.77	157	-21.77	731143	24	2645	1375
miR1874-3p	LOC_Os01g13370.1	147	-25.42	147	-25.42	731147	24	1783	1222
miR1874-3p	LOC_Os01g13440.1	157	-20.21	157	-20.21	731155	24	824	31
miR1874-3p	LOC_Os01g13900.1	141	-22.25	141	-22.25	731208	24	1254	1000

miR1874-3p	LOC_Os01g14490.1	156	-22.37	156	-22.37	731270	24	2616	1761
miR1874-3p	LOC_Os01g14620.1	148	-20.52	148	-20.52	731283	24	387	17
miR1874-3p	LOC_Os01g14850.1	165	-23.84	165	-23.84	731306	24	1009	463
miR1874-3p	LOC_Os01g14860.1	159	-21.08	159	-21.08	731307	24	2032	827
miR1874-3p	LOC_Os01g14860.2	159	-21.08	159	-21.08	731308	24	2070	865
miR1874-3p	LOC_Os01g14860.3	159	-21.08	159	-21.08	731309	24	2816	1611
miR1874-3p	LOC_Os01g14860.4	159	-21.08	159	-21.08	731310	24	2028	823
miR1874-3p	LOC_Os01g14950.1	170	-25.59	170	-25.59	731322	24	2193	1054
miR1874-3p	LOC_Os01g15000.1	170	-28.42	170	-28.42	731328	24	2738	2467
miR1874-3p	LOC_Os01g15029.1	149	-23.26	149	-23.26	731332	24	676	96
miR1874-3p	LOC_Os01g15320.1	166	-24.25	166	-24.25	731360	24	736	90
miR1874-3p	LOC_Os01g15900.1	162	-24.26	162	-24.26	731426	24	2228	835
miR1874-3p	LOC_Os01g16280.1	161	-21.54	161	-21.54	731480	24	276	7
miR1874-3p	LOC_Os01g16370.1	156	-24.89	156	-24.89	731492	24	3454	1824
miR1874-3p	LOC_Os01g16370.2	156	-24.89	156	-24.89	731493	24	3286	1656
miR1874-3p	LOC_Os01g16500.1	160	-28.19	160	-28.19	731508	24	4542	226
miR1874-3p	LOC_Os01g16700.1	162	-22.03	162	-22.03	731528	24	1191	1095
miR1874-3p	LOC_Os01g17080.1	164	-25.19	164	-25.19	731575	24	1275	352
miR1874-3p	LOC_Os01g18000.1	176	-33.4	176	-33.4	731617	24	4224	2241
miR1874-3p	LOC_Os01g18390.1	310	-47.02	158	-26.13	731656	24	873	817 733
miR1874-3p	LOC_Os01g18470.1	162	-21.53	162	-21.53	731663	24	4038	4016
miR1874-3p	LOC_Os01g18650.1	167	-21.85	167	-21.85	731680	24	2253	1572
miR1874-3p	LOC_Os01g18670.1	152	-20.46	152	-20.46	731682	24	3858	1203
miR1874-3p	LOC_Os01g19150.1	153	-21.02	153	-21.02	731739	24	2270	1450
miR1874-3p	LOC_Os01g19150.3	153	-21.02	153	-21.02	731741	24	2343	1523
miR1874-3p	LOC_Os01g19150.4	153	-21.02	153	-21.02	731742	24	2309	1489
miR1874-3p	LOC_Os01g19150.5	153	-21.02	153	-21.02	731743	24	2266	1446
miR1874-3p	LOC_Os01g19894.1	320	-40.67	160	-20.49	731819	24	5877	1817 3940
miR1874-3p	LOC_Os01g19990.1	156	-24.58	156	-24.58	731828	24	920	39
miR1874-3p	LOC_Os01g20100.1	145	-22.14	145	-22.14	731839	24	354	165
miR1874-3p	LOC_Os01g20140.1	158	-21.78	158	-21.78	731845	24	495	56
miR1874-3p	LOC_Os01g20150.1	169	-28.72	169	-28.72	731846	24	2553	2118

miR1874-3p	LOC_Os01g20860.1	156	-20.22	156	-20.22	731869	24	2976	509
miR1874-3p	LOC_Os01g21144.1	166	-23.53	166	-23.53	731906	24	2753	159
miR1874-3p	LOC_Os01g21190.1	154	-23.58	154	-23.58	731914	24	3489	3227
miR1874-3p	LOC_Os01g21360.1	157	-20.21	157	-20.21	731928	24	2544	1914
miR1874-3p	LOC_Os01g21400.1	160	-21.17	160	-21.17	731931	24	1278	1029
miR1874-3p	LOC_Os01g21536.1	144	-22.3	144	-22.3	731946	24	4482	3978
miR1874-3p	LOC_Os01g21580.1	160	-26.11	160	-26.11	731950	24	710	585
miR1874-3p	LOC_Os01g22540.1	157	-21.4	157	-21.4	732040	24	630	606
miR1874-3p	LOC_Os01g22630.1	155	-31.73	155	-31.73	732048	24	3482	3273
miR1874-3p	LOC_Os01g22630.2	155	-31.73	155	-31.73	732049	24	3355	3146
miR1874-3p	LOC_Os01g22840.1	168	-24.46	168	-24.46	732071	24	2823	2084
miR1874-3p	LOC_Os01g22900.1	165	-24.07	165	-24.07	732077	24	2540	2193
miR1874-3p	LOC_Os01g22900.3	165	-24.07	165	-24.07	732079	24	2479	2132
miR1874-3p	LOC_Os01g23040.1	154	-22.07	154	-22.07	732091	24	4995	4973
miR1874-3p	LOC_Os01g23130.1	153	-24.49	153	-24.49	732100	24	3795	1217
miR1874-3p	LOC_Os01g23590.1	142	-24.74	142	-24.74	732141	24	3479	805
miR1874-3p	LOC_Os01g23750.1	143	-23.38	143	-23.38	732164	24	1221	1121
miR1874-3p	LOC_Os01g23930.1	320	-40.67	160	-20.49	732181	24	4650	2264 4405
miR1874-3p	LOC_Os01g24030.1	162	-20.63	162	-20.63	732189	24	2071	1960
miR1874-3p	LOC_Os01g24030.2	162	-20.63	162	-20.63	732190	24	1586	1500
miR1874-3p	LOC_Os01g24050.1	165	-21.03	165	-21.03	732192	24	2506	123
miR1874-3p	LOC_Os01g24130.1	158	-20.45	158	-20.45	732203	24	1377	755
miR1874-3p	LOC_Os01g24550.1	155	-21.5	155	-21.5	732244	24	1342	111
miR1874-3p	LOC_Os01g24660.1	169	-28.72	169	-28.72	732254	24	2184	1701
miR1874-3p	LOC_Os01g24680.1	147	-22.55	147	-22.55	732256	24	2819	2249
miR1874-3p	LOC_Os01g24680.2	147	-22.55	147	-22.55	732257	24	3352	2782
miR1874-3p	LOC_Os01g25090.1	160	-23.82	160	-23.82	732307	24	846	600
miR1874-3p	LOC_Os01g25330.1	163	-21.03	163	-21.03	732332	24	3856	250
miR1874-3p	LOC_Os01g25330.2	163	-21.03	163	-21.03	732333	24	4137	250
miR1874-3p	LOC_Os01g25330.3	163	-21.03	163	-21.03	732334	24	3885	250
miR1874-3p	LOC_Os01g25670.1	162	-20.36	162	-20.36	732366	24	498	447
miR1874-3p	LOC_Os01g25720.1	154	-21.76	154	-21.76	732371	24	5381	2161

miR1874-3p	LOC_Os01g25720.2	154	-21.76	154	-21.76	732372	24	5288	2161
miR1874-3p	LOC_Os01g25760.1	163	-21.63	163	-21.63	732375	24	4099	1761
miR1874-3p	LOC_Os01g25820.1	147	-22.63	147	-22.63	732380	24	3414	2462
miR1874-3p	LOC_Os01g25820.2	147	-22.63	147	-22.63	732381	24	3497	2545
miR1874-3p	LOC_Os01g25860.1	154	-22.07	154	-22.07	732383	24	5379	5357
miR1874-3p	LOC_Os01g26000.1	170	-23.36	170	-23.36	732399	24	2264	222
miR1874-3p	LOC_Os01g26930.1	153	-24.49	153	-24.49	732439	24	3855	1199
miR1874-3p	LOC_Os01g27210.1	150	-21.98	150	-21.98	732476	24	1006	840
miR1874-3p	LOC_Os01g27320.1	147	-23.72	147	-23.72	732484	24	3786	3080
miR1874-3p	LOC_Os01g27520.1	152	-21.53	152	-21.53	732503	24	2121	532
miR1874-3p	LOC_Os01g27520.2	152	-21.53	152	-21.53	732504	24	2096	532
miR1874-3p	LOC_Os01g27670.1	161	-27.79	161	-27.79	732517	24	2598	2559
miR1874-3p	LOC_Os01g27680.1	153	-21.19	153	-21.19	732518	24	1353	241
miR1874-3p	LOC_Os01g27730.1	150	-20.08	150	-20.08	732523	24	2600	482
miR1874-3p	LOC_Os01g28590.1	144	-21.69	144	-21.69	732605	24	519	425
miR1874-3p	LOC_Os01g29000.1	145	-21.11	145	-21.11	732660	24	3396	517
miR1874-3p	LOC_Os01g29900.1	155	-21.33	155	-21.33	732745	24	4698	1545
miR1874-3p	LOC_Os01g30970.1	156	-20.44	156	-20.44	732751	24	971	206
miR1874-3p	LOC_Os01g31260.1	147	-23.52	147	-23.52	732781	24	2808	2534
miR1874-3p	LOC_Os01g31810.2	161	-20.45	161	-20.45	732839	24	1153	455
miR1874-3p	LOC_Os01g31900.1	154	-23.58	154	-23.58	732855	24	2547	2300
miR1874-3p	LOC_Os01g32470.1	151	-21.7	151	-21.7	732911	24	759	721
miR1874-3p	LOC_Os01g32750.1	158	-27.03	158	-27.03	732941	24	2260	538
miR1874-3p	LOC_Os01g32990.1	141	-22.07	141	-22.07	732967	24	4116	2668
miR1874-3p	LOC_Os01g33110.1	176	-32.12	176	-32.12	732981	24	3653	440
miR1874-3p	LOC_Os01g33136.1	147	-22.89	147	-22.89	732984	24	2235	2105
miR1874-3p	LOC_Os01g33220.1	155	-21.33	155	-21.33	732991	24	3864	1317
miR1874-3p	LOC_Os01g33240.1	319	-48.79	160	-28.3	732993	24	1761	1099 1498
miR1874-3p	LOC_Os01g33684.1	308	-42.25	155	-21.19	733033	24	4094	2446 1469
miR1874-3p	LOC_Os01g33684.2	308	-42.25	155	-21.19	733034	24	4085	2437 1460
miR1874-3p	LOC_Os01g33684.3	308	-42.25	155	-21.19	733035	24	4111	2446 1469
miR1874-3p	LOC_Os01g33784.1	168	-21.42	168	-21.42	733045	24	1533	1213

miR1874-3p	LOC_Os01g33960.1	165	-20.87	165	-20.87	733059	24	2142	905
miR1874-3p	LOC_Os01g34140.1	155	-22.44	155	-22.44	733076	24	5442	5410
miR1874-3p	LOC_Os01g34350.1	167	-22.5	167	-22.5	733093	24	4046	1002
miR1874-3p	LOC_Os01g34850.1	157	-21.45	157	-21.45	733155	24	1628	451
miR1874-3p	LOC_Os01g34860.1	145	-20.59	145	-20.59	733156	24	2396	2252
miR1874-3p	LOC_Os01g34880.1	152	-21.28	152	-21.28	733158	24	1725	207
miR1874-3p	LOC_Os01g34980.1	168	-26.01	168	-26.01	733169	24	738	435
miR1874-3p	LOC_Os01g35070.1	144	-22.3	144	-22.3	733178	24	4173	3747
miR1874-3p	LOC_Os01g35560.1	154	-21.23	154	-21.23	733226	24	2292	296
miR1874-3p	LOC_Os01g35710.1	147	-23.89	147	-23.89	733247	24	3729	3365
miR1874-3p	LOC_Os01g36040.1	154	-22.07	154	-22.07	733278	24	5385	5363
miR1874-3p	LOC_Os01g36250.1	320	-40.67	160	-20.49	733300	24	4701	2099 4456
miR1874-3p	LOC_Os01g36260.1	320	-40.67	160	-20.49	733301	24	4548	2129 4303
miR1874-3p	LOC_Os01g36370.1	170	-24.2	170	-24.2	733313	24	2724	402
miR1874-3p	LOC_Os01g36390.1	156	-25.35	156	-25.35	733315	24	2878	2568
miR1874-3p	LOC_Os01g36480.1	147	-20.96	147	-20.96	733324	24	570	548
miR1874-3p	LOC_Os01g36500.1	160	-21.91	160	-21.91	733326	24	2547	1528
miR1874-3p	LOC_Os01g36760.1	166	-20.5	166	-20.5	733351	24	510	233
miR1874-3p	LOC_Os01g36850.1	150	-22.49	150	-22.49	733363	24	1895	1447
miR1874-3p	LOC_Os01g37000.1	164	-23.6	164	-23.6	733382	24	1559	932
miR1874-3p	LOC_Os01g37060.1	154	-22.07	154	-22.07	733387	24	5376	5354
miR1874-3p	LOC_Os01g37080.1	156	-20.16	156	-20.16	733389	24	3957	528
miR1874-3p	LOC_Os01g37110.1	164	-27.93	164	-27.93	733392	24	1325	533
miR1874-3p	LOC_Os01g37260.1	156	-23.93	156	-23.93	733407	24	1819	39
miR1874-3p	LOC_Os01g37260.2	156	-23.93	156	-23.93	733408	24	1821	54
miR1874-3p	LOC_Os01g37325.1	149	-23.3	149	-23.3	733421	24	4121	397
miR1874-3p	LOC_Os01g37430.1	169	-28.72	169	-28.72	733432	24	1965	1530
miR1874-3p	LOC_Os01g37460.1	163	-21.6	163	-21.6	733433	24	941	1
miR1874-3p	LOC_Os01g37580.1	155	-20.33	155	-20.33	733448	24	5799	647
miR1874-3p	LOC_Os01g37610.1	176	-33.4	176	-33.4	733452	24	4206	2241
miR1874-3p	LOC_Os01g37842.1	162	-20.4	162	-20.4	733480	24	1646	28
miR1874-3p	LOC_Os01g38170.1	150	-21.02	150	-21.02	733516	24	1932	1343

miR1874-3p	LOC_Os01g38180.1	157	-23.56	157	-23.56	733517	24	2036	788
miR1874-3p	LOC_Os01g38860.1	322	-49.26	166	-26.44	733589	24	612	182 198
miR1874-3p	LOC_Os01g39385.1	163	-20.16	163	-20.16	733647	24	6262	3982
miR1874-3p	LOC_Os01g39530.1	159	-22.53	159	-22.53	733659	24	1608	299
miR1874-3p	LOC_Os01g39640.1	158	-22.37	158	-22.37	733670	24	909	353
miR1874-3p	LOC_Os01g39700.1	159	-20.38	159	-20.38	733678	24	627	345
miR1874-3p	LOC_Os01g39770.1	322	-48.84	163	-27.7	733685	24	1435	34 577
miR1874-3p	LOC_Os01g39770.2	322	-48.84	163	-27.7	733686	24	1616	34 577
miR1874-3p	LOC_Os01g39770.3	322	-48.84	163	-27.7	733687	24	1517	30 573
miR1874-3p	LOC_Os01g39830.1	164	-26.68	164	-26.68	733698	24	3257	148
miR1874-3p	LOC_Os01g39970.1	179	-31.07	179	-31.07	733711	24	2832	198
miR1874-3p	LOC_Os01g40120.1	319	-47.03	165	-24.35	733725	24	1341	708 22
miR1874-3p	LOC_Os01g40200.1	161	-22.8	161	-22.8	733733	24	2897	2637
miR1874-3p	LOC_Os01g40200.2	161	-22.8	161	-22.8	733734	24	2988	2728
miR1874-3p	LOC_Os01g40310.1	165	-20.73	165	-20.73	733745	24	885	35
miR1874-3p	LOC_Os01g40460.1	160	-25.04	160	-25.04	733764	24	1059	751
miR1874-3p	LOC_Os01g41090.1	159	-24.65	159	-24.65	733836	24	4122	2644
miR1874-3p	LOC_Os01g41530.1	140	-20.63	140	-20.63	733881	24	1900	1072
miR1874-3p	LOC_Os01g41620.1	320	-40.67	160	-20.49	733891	24	4860	2258 4615
miR1874-3p	LOC_Os01g41660.1	161	-26.56	161	-26.56	733895	24	1641	548
miR1874-3p	LOC_Os01g42350.1	324	-47.63	171	-26.89	733967	24	4440	138 3306
miR1874-3p	LOC_Os01g42370.1	312	-45.37	159	-24.63	733969	24	4335	138 3213
miR1874-3p	LOC_Os01g42380.1	160	-24.44	160	-24.44	733970	24	4916	1321
miR1874-3p	LOC_Os01g42410.1	309	-43.26	155	-21.92	733972	24	4616	3559 1264
miR1874-3p	LOC_Os01g42510.1	159	-24.1	159	-24.1	733985	24	761	465
miR1874-3p	LOC_Os01g42510.2	159	-24.1	159	-24.1	733986	24	670	374
miR1874-3p	LOC_Os01g42560.1	156	-21.55	156	-21.55	733990	24	1083	992
miR1874-3p	LOC_Os01g42850.1	161	-20.7	161	-20.7	734028	24	3483	1787
miR1874-3p	LOC_Os01g42850.2	161	-20.7	161	-20.7	734029	24	3480	1787
miR1874-3p	LOC_Os01g43550.1	155	-21.83	155	-21.83	734117	24	1468	367
miR1874-3p	LOC_Os01g43550.2	155	-21.83	155	-21.83	734118	24	1466	371
miR1874-3p	LOC_Os01g43690.1	156	-20.25	156	-20.25	734137	24	522	197

miR1874-3p	LOC_Os01g43800.1	159	-25.66	159	-25.66	734147	24	5175	2114
miR1874-3p	LOC_Os01g43851.1	147	-21.02	147	-21.02	734151	24	1760	34
miR1874-3p	LOC_Os01g44030.1	156	-21.19	156	-21.19	734173	24	558	315
miR1874-3p	LOC_Os01g44970.1	163	-21.95	163	-21.95	734222	24	2178	1843
miR1874-3p	LOC_Os01g45050.1	176	-33.4	176	-33.4	734231	24	4161	2241
miR1874-3p	LOC_Os01g45174.1	154	-23.58	154	-23.58	734244	24	2178	1931
miR1874-3p	LOC_Os01g45500.1	158	-21.02	158	-21.02	734277	24	873	367
miR1874-3p	LOC_Os01g45880.1	145	-24.67	145	-24.67	734316	24	5462	4301
miR1874-3p	LOC_Os01g45880.2	145	-24.67	145	-24.67	734317	24	5408	4247
miR1874-3p	LOC_Os01g46080.1	167	-26.57	167	-26.57	734337	24	1730	1111
miR1874-3p	LOC_Os01g46080.2	167	-26.57	167	-26.57	734338	24	1721	1102
miR1874-3p	LOC_Os01g46090.1	163	-23.74	163	-23.74	734339	24	933	769
miR1874-3p	LOC_Os01g46110.1	150	-21.18	150	-21.18	734341	24	3849	3309
miR1874-3p	LOC_Os01g46120.1	167	-26.57	167	-26.57	734342	24	1428	1118
miR1874-3p	LOC_Os01g46120.2	167	-26.57	167	-26.57	734343	24	1125	815
miR1874-3p	LOC_Os01g46120.3	167	-26.57	167	-26.57	734344	24	1146	841
miR1874-3p	LOC_Os01g46140.1	159	-20.53	159	-20.53	734347	24	731	227
miR1874-3p	LOC_Os01g46169.1	163	-23.74	163	-23.74	734348	24	1688	1159
miR1874-3p	LOC_Os01g46169.2	163	-23.74	163	-23.74	734349	24	2192	1663
miR1874-3p	LOC_Os01g46590.1	155	-22.14	155	-22.14	734394	24	2335	923
miR1874-3p	LOC_Os01g46590.2	155	-22.14	155	-22.14	734395	24	2215	803
miR1874-3p	LOC_Os01g46632.1	158	-21.02	158	-21.02	734401	24	1728	1222
miR1874-3p	LOC_Os01g47100.1	161	-26.06	161	-26.06	734453	24	1074	580
miR1874-3p	LOC_Os01g47400.1	152	-24.26	152	-24.26	734488	24	1776	163
miR1874-3p	LOC_Os01g47400.2	152	-24.26	152	-24.26	734489	24	1676	151
miR1874-3p	LOC_Os01g47410.1	154	-20.71	154	-20.71	734490	24	1946	595
miR1874-3p	LOC_Os01g47410.2	154	-20.71	154	-20.71	734491	24	1887	595
miR1874-3p	LOC_Os01g47440.1	157	-20.97	157	-20.97	734497	24	657	22
miR1874-3p	LOC_Os01g47550.1	177	-32.32	177	-32.32	734518	24	1514	1329
miR1874-3p	LOC_Os01g47740.1	163	-24.62	163	-24.62	734540	24	2513	1645
miR1874-3p	LOC_Os01g47740.2	163	-24.62	163	-24.62	734541	24	2579	1645
miR1874-3p	LOC_Os01g47740.3	163	-24.62	163	-24.62	734542	24	2510	1642

miR1874-3p	LOC_Os01g47740.4	163	-24.62	163	-24.62	734543	24	2407	1645
miR1874-3p	LOC_Os01g48070.1	159	-23.3	159	-23.3	734578	24	1278	1030
miR1874-3p	LOC_Os01g48180.1	146	-22.75	146	-22.75	734590	24	5142	888
miR1874-3p	LOC_Os01g48410.1	158	-25.88	158	-25.88	734621	24	2338	86
miR1874-3p	LOC_Os01g48490.1	169	-27.36	169	-27.36	734631	24	4872	1775
miR1874-3p	LOC_Os01g48550.1	153	-23.32	153	-23.32	734637	24	1483	1035
miR1874-3p	LOC_Os01g48610.1	160	-24.97	160	-24.97	734643	24	3130	28
miR1874-3p	LOC_Os01g49110.1	161	-22.14	161	-22.14	734705	24	2530	69
miR1874-3p	LOC_Os01g49110.2	161	-22.14	161	-22.14	734706	24	2434	67
miR1874-3p	LOC_Os01g50010.1	155	-25.6	155	-25.6	734805	24	2395	484
miR1874-3p	LOC_Os01g50320.1	163	-22.19	163	-22.19	734834	24	3010	126
miR1874-3p	LOC_Os01g50616.1	164	-23.55	164	-23.55	734865	24	2817	266
miR1874-3p	LOC_Os01g51060.1	142	-24.42	142	-24.42	734921	24	2674	2198
miR1874-3p	LOC_Os01g51340.1	156	-20.7	156	-20.7	734956	24	1350	830
miR1874-3p	LOC_Os01g51490.1	171	-26.52	171	-26.52	734974	24	930	61
miR1874-3p	LOC_Os01g51550.1	172	-30.02	172	-30.02	734980	24	1818	1085
miR1874-3p	LOC_Os01g52000.1	172	-25.17	172	-25.17	735033	24	4302	2943
miR1874-3p	LOC_Os01g52170.1	154	-21	154	-21	735058	24	2043	1923
miR1874-3p	LOC_Os01g52304.1	164	-22.53	164	-22.53	735075	24	2900	2773
miR1874-3p	LOC_Os01g52550.1	151	-21.46	151	-21.46	735103	24	4255	807
miR1874-3p	LOC_Os01g52720.1	153	-20.5	153	-20.5	735118	24	1095	18
miR1874-3p	LOC_Os01g53160.1	160	-20.89	160	-20.89	735167	24	1391	33
miR1874-3p	LOC_Os01g53330.1	147	-23.3	147	-23.3	735184	24	1836	1729
miR1874-3p	LOC_Os01g53420.1	302	-45.76	152	-23.39	735192	24	1752	339 1531
miR1874-3p	LOC_Os01g53630.1	151	-21.37	151	-21.37	735213	24	1960	319
miR1874-3p	LOC_Os01g53900.1	168	-23.99	168	-23.99	735250	24	2858	73
miR1874-3p	LOC_Os01g54050.1	157	-22.81	157	-22.81	735271	24	1819	1117
miR1874-3p	LOC_Os01g54119.1	154	-21.12	154	-21.12	735278	24	2491	1250
miR1874-3p	LOC_Os01g54150.1	149	-20.12	149	-20.12	735280	24	2841	1339
miR1874-3p	LOC_Os01g54420.1	163	-20.52	163	-20.52	735309	24	2790	1095
miR1874-3p	LOC_Os01g54760.1	155	-20.39	155	-20.39	735351	24	756	458
miR1874-3p	LOC_Os01g55090.1	156	-24.3	156	-24.3	735390	24	2107	776

miR1874-3p	LOC_Os01g55090.2	156	-24.3	156	-24.3	735391	24	2242	774
miR1874-3p	LOC_Os01g55120.1	311	-46.26	163	-24.61	735397	24	1767	454 760
miR1874-3p	LOC_Os01g55480.1	161	-27.79	161	-27.79	735442	24	2766	2601
miR1874-3p	LOC_Os01g55580.1	155	-20.98	155	-20.98	735462	24	2448	1539
miR1874-3p	LOC_Os01g55580.2	155	-20.98	155	-20.98	735463	24	2523	1614
miR1874-3p	LOC_Os01g55610.1	149	-21.21	149	-21.21	735466	24	2119	163
miR1874-3p	LOC_Os01g55660.1	151	-20.89	151	-20.89	735471	24	4143	1328
miR1874-3p	LOC_Os01g55880.1	164	-24.72	164	-24.72	735495	24	1581	281
miR1874-3p	LOC_Os01g56000.1	145	-20.73	145	-20.73	735513	24	4417	1177
miR1874-3p	LOC_Os01g56130.1	165	-21.38	165	-21.38	735529	24	2466	1196
miR1874-3p	LOC_Os01g56190.1	156	-23.29	156	-23.29	735535	24	4670	2532
miR1874-3p	LOC_Os01g56320.1	148	-21.68	148	-21.68	735555	24	3214	1140
miR1874-3p	LOC_Os01g56490.1	155	-20.94	155	-20.94	735573	24	4246	3723
miR1874-3p	LOC_Os01g56790.1	164	-22.06	164	-22.06	735619	24	3473	1037
miR1874-3p	LOC_Os01g56960.1	165	-20.92	165	-20.92	735644	24	1044	473
miR1874-3p	LOC_Os01g56980.1	165	-20.92	165	-20.92	735645	24	3528	473
miR1874-3p	LOC_Os01g57110.1	149	-22.01	149	-22.01	735657	24	4335	3205
miR1874-3p	LOC_Os01g57110.2	149	-22.01	149	-22.01	735658	24	4290	3205
miR1874-3p	LOC_Os01g57210.1	160	-20.62	160	-20.62	735667	24	3209	706
miR1874-3p	LOC_Os01g57210.2	160	-20.62	160	-20.62	735668	24	3219	493
miR1874-3p	LOC_Os01g57420.1	310	-41.71	156	-21.37	735695	24	3197	2230 1503
miR1874-3p	LOC_Os01g57420.2	310	-41.71	156	-21.37	735696	24	2348	1381 616
miR1874-3p	LOC_Os01g57490.1	154	-21.33	154	-21.33	735703	24	2394	1869
miR1874-3p	LOC_Os01g57610.1	160	-22.72	160	-22.72	735717	24	2692	843
miR1874-3p	LOC_Os01g57730.1	157	-20.72	157	-20.72	735734	24	1739	45
miR1874-3p	LOC_Os01g57840.1	160	-22.52	160	-22.52	735745	24	919	839
miR1874-3p	LOC_Os01g57870.1	148	-20.18	148	-20.18	735747	24	3660	2287
miR1874-3p	LOC_Os01g57870.2	148	-20.18	148	-20.18	735748	24	3429	2287
miR1874-3p	LOC_Os01g58114.1	152	-23.85	152	-23.85	735784	24	2179	1046
miR1874-3p	LOC_Os01g58114.2	152	-23.85	152	-23.85	735785	24	2214	1046
miR1874-3p	LOC_Os01g58170.1	147	-22.21	147	-22.21	735790	24	528	504
miR1874-3p	LOC_Os01g58490.1	141	-24.81	141	-24.81	735824	24	579	124

miR1874-3p	LOC_Os01g58640.1	148	-24.05	148	-24.05	735840	24	2503	1060
miR1874-3p	LOC_Os01g58730.1	149	-20.22	149	-20.22	735851	24	1288	1185
miR1874-3p	LOC_Os01g58890.1	163	-23.22	163	-23.22	735869	24	1080	36
miR1874-3p	LOC_Os01g58890.2	163	-23.22	163	-23.22	735870	24	1449	36
miR1874-3p	LOC_Os01g58890.3	163	-23.22	163	-23.22	735871	24	882	34
miR1874-3p	LOC_Os01g58890.4	163	-23.22	163	-23.22	735872	24	1414	34
miR1874-3p	LOC_Os01g59510.1	168	-25.69	168	-25.69	735939	24	2165	1674
miR1874-3p	LOC_Os01g59520.1	147	-21.78	147	-21.78	735940	24	1423	124
miR1874-3p	LOC_Os01g59520.2	147	-21.78	147	-21.78	735941	24	1335	124
miR1874-3p	LOC_Os01g59520.3	147	-21.78	147	-21.78	735942	24	1510	124
miR1874-3p	LOC_Os01g59580.1	161	-23.07	161	-23.07	735949	24	2748	116
miR1874-3p	LOC_Os01g60190.1	164	-20.39	164	-20.39	736020	24	2130	221
miR1874-3p	LOC_Os01g60190.2	164	-20.39	164	-20.39	736021	24	2350	441
miR1874-3p	LOC_Os01g60330.1	147	-20.91	147	-20.91	736036	24	2230	1382
miR1874-3p	LOC_Os01g60700.1	147	-20.26	147	-20.26	736074	24	2134	925
miR1874-3p	LOC_Os01g60700.2	147	-20.26	147	-20.26	736075	24	1482	194
miR1874-3p	LOC_Os01g60920.1	160	-20.4	160	-20.4	736099	24	2188	528
miR1874-3p	LOC_Os01g61180.1	158	-23.78	158	-23.78	736125	24	2458	1678
miR1874-3p	LOC_Os01g61940.1	148	-27.57	148	-27.57	736231	24	2762	986
miR1874-3p	LOC_Os01g62220.1	156	-22.34	156	-22.34	736270	24	1371	564
miR1874-3p	LOC_Os01g62420.1	150	-23.1	150	-23.1	736294	24	1420	208
miR1874-3p	LOC_Os01g62420.2	150	-23.1	150	-23.1	736295	24	919	208
miR1874-3p	LOC_Os01g62420.3	150	-23.1	150	-23.1	736296	24	853	208
miR1874-3p	LOC_Os01g62420.4	150	-23.1	150	-23.1	736297	24	1415	84
miR1874-3p	LOC_Os01g62770.1	146	-20.52	146	-20.52	736340	24	3437	1984
miR1874-3p	LOC_Os01g62900.1	152	-20.7	152	-20.7	736362	24	3441	3123
miR1874-3p	LOC_Os01g62910.1	152	-20.7	152	-20.7	736363	24	3712	1293
miR1874-3p	LOC_Os01g63080.1	147	-23.72	147	-23.72	736379	24	5004	4346
miR1874-3p	LOC_Os01g63290.1	162	-20.23	162	-20.23	736407	24	2466	1308
miR1874-3p	LOC_Os01g63290.2	162	-20.23	162	-20.23	736408	24	2086	1308
miR1874-3p	LOC_Os01g63690.1	158	-20.47	158	-20.47	736447	24	1998	408
miR1874-3p	LOC_Os01g64310.1	145	-22.33	145	-22.33	736534	24	1446	888

miR1874-3p	LOC_Os01g64310.2	145	-22.33	145	-22.33	736535	24	1696	1138
miR1874-3p	LOC_Os01g64330.1	160	-20.46	160	-20.46	736537	24	2695	2058
miR1874-3p	LOC_Os01g64610.1	156	-20.06	156	-20.06	736568	24	2988	180
miR1874-3p	LOC_Os01g64700.1	145	-20.6	145	-20.6	736585	24	2035	1309
miR1874-3p	LOC_Os01g64700.2	145	-20.6	145	-20.6	736586	24	2043	1317
miR1874-3p	LOC_Os01g64810.1	171	-28.91	171	-28.91	736605	24	2294	1403
miR1874-3p	LOC_Os01g64810.2	171	-28.91	171	-28.91	736606	24	1971	1080
miR1874-3p	LOC_Os01g64820.1	152	-20.02	152	-20.02	736607	24	4966	4945
miR1874-3p	LOC_Os01g64980.1	144	-22.31	144	-22.31	736623	24	4142	1960
miR1874-3p	LOC_Os01g64980.2	144	-22.31	144	-22.31	736624	24	3317	1135
miR1874-3p	LOC_Os01g65030.1	160	-27.96	160	-27.96	736629	24	2728	1817
miR1874-3p	LOC_Os01g65090.1	157	-22.49	157	-22.49	736638	24	1696	47
miR1874-3p	LOC_Os01g65420.1	145	-22.55	145	-22.55	736678	24	1228	869
miR1874-3p	LOC_Os01g65480.1	157	-21.91	157	-21.91	736685	24	1720	136
miR1874-3p	LOC_Os01g65520.1	155	-21.31	155	-21.31	736689	24	3128	1591
miR1874-3p	LOC_Os01g65890.1	165	-21.89	165	-21.89	736737	24	2496	970
miR1874-3p	LOC_Os01g66080.1	149	-20.12	149	-20.12	736759	24	2892	1336
miR1874-3p	LOC_Os01g66580.1	168	-22.41	168	-22.41	736811	24	861	837
miR1874-3p	LOC_Os01g66920.1	158	-23.87	158	-23.87	736851	24	2668	1795
miR1874-3p	LOC_Os01g67120.1	162	-22.25	162	-22.25	736876	24	2345	921
miR1874-3p	LOC_Os01g67120.2	162	-22.25	162	-22.25	736877	24	1825	401
miR1874-3p	LOC_Os01g67500.1	160	-20.31	160	-20.31	736928	24	2935	2621
miR1874-3p	LOC_Os01g67500.3	160	-20.31	160	-20.31	736929	24	2840	2763
miR1874-3p	LOC_Os01g67600.1	160	-22.58	160	-22.58	736942	24	1880	379
miR1874-3p	LOC_Os01g67990.1	154	-23.58	154	-23.58	736985	24	2634	2255
miR1874-3p	LOC_Os01g68070.1	165	-24.36	165	-24.36	736998	24	2930	986
miR1874-3p	LOC_Os01g68160.3	155	-20.63	155	-20.63	737007	24	5135	1572
miR1874-3p	LOC_Os01g68310.1	156	-22.74	156	-22.74	737021	24	1008	400
miR1874-3p	LOC_Os01g68320.1	167	-20.55	167	-20.55	737022	24	2805	840
miR1874-3p	LOC_Os01g68320.2	167	-20.55	167	-20.55	737023	24	2887	922
miR1874-3p	LOC_Os01g68320.3	167	-20.55	167	-20.55	737024	24	4120	2155
miR1874-3p	LOC_Os01g68320.4	167	-20.55	167	-20.55	737025	24	3030	1065

miR1874-3p	LOC_Os01g68320.5	167	-20.55	167	-20.55	737026	24	4068	2103
miR1874-3p	LOC_Os01g68324.3	152	-21.53	152	-21.53	737027	24	2732	925
miR1874-3p	LOC_Os01g68390.1	167	-24.05	167	-24.05	737037	24	1689	914
miR1874-3p	LOC_Os01g68524.1	170	-23.48	170	-23.48	737052	24	3327	698
miR1874-3p	LOC_Os01g68524.2	170	-23.48	170	-23.48	737053	24	1133	698
miR1874-3p	LOC_Os01g68720.1	155	-21.05	155	-21.05	737076	24	1496	235
miR1874-3p	LOC_Os01g68760.1	152	-20.49	152	-20.49	737081	24	2406	1829
miR1874-3p	LOC_Os01g69840.1	167	-23.05	167	-23.05	737159	24	582	160
miR1874-3p	LOC_Os01g69920.1	159	-24.17	159	-24.17	737169	24	4082	740
miR1874-3p	LOC_Os01g69920.2	159	-24.17	159	-24.17	737170	24	4153	740
miR1874-3p	LOC_Os01g69920.3	159	-24.17	159	-24.17	737171	24	4184	740
miR1874-3p	LOC_Os01g69970.1	165	-23.57	165	-23.57	737176	24	1903	1154
miR1874-3p	LOC_Os01g70110.1	166	-29.31	166	-29.31	737190	24	1109	286
miR1874-3p	LOC_Os01g70190.1	164	-24.23	164	-24.23	737201	24	2200	132
miR1874-3p	LOC_Os01g70260.1	164	-21.62	164	-21.62	737209	24	2715	1814
miR1874-3p	LOC_Os01g70260.2	164	-21.62	164	-21.62	737210	24	2867	1966
miR1874-3p	LOC_Os01g70340.1	166	-24.92	166	-24.92	737225	24	1569	1268
miR1874-3p	LOC_Os01g70560.1	152	-24.21	152	-24.21	737252	24	1425	273
miR1874-3p	LOC_Os01g70780.1	170	-31.79	170	-31.79	737279	24	1725	347
miR1874-3p	LOC_Os01g70850.1	145	-22.8	145	-22.8	737288	24	1139	375
miR1874-3p	LOC_Os01g70850.2	145	-22.8	145	-22.8	737289	24	1343	358
miR1874-3p	LOC_Os01g70860.1	149	-21.27	149	-21.27	737290	24	1047	282
miR1874-3p	LOC_Os01g71330.1	161	-23.95	161	-23.95	737347	24	857	46
miR1874-3p	LOC_Os01g71624.1	160	-22.87	160	-22.87	737378	24	828	22
miR1874-3p	LOC_Os01g71624.2	160	-22.87	160	-22.87	737379	24	1046	26
miR1874-3p	LOC_Os01g71624.3	160	-22.87	160	-22.87	737380	24	756	26
miR1874-3p	LOC_Os01g71624.4	160	-22.87	160	-22.87	737381	24	782	26
miR1874-3p	LOC_Os01g71624.5	160	-22.87	160	-22.87	737382	24	1829	26
miR1874-3p	LOC_Os01g71970.1	171	-27.96	171	-27.96	737418	24	1544	350
miR1874-3p	LOC_Os01g72000.1	143	-21.03	143	-21.03	737422	24	2782	667
miR1874-3p	LOC_Os01g72250.1	154	-21.14	154	-21.14	737449	24	1710	1292
miR1874-3p	LOC_Os01g72340.1	155	-24.53	155	-24.53	737459	24	3965	2356

miR1874-3p	LOC_Os01g72390.1	148	-23.59	148	-23.59	737467	24	2574	1367
miR1874-3p	LOC_Os01g72420.1	150	-22.16	150	-22.16	737470	24	2586	2041
miR1874-3p	LOC_Os01g72460.1	157	-24.14	157	-24.14	737474	24	849	453
miR1874-3p	LOC_Os01g72570.1	154	-21.22	154	-21.22	737486	24	2500	141
miR1874-3p	LOC_Os01g72570.2	154	-21.22	154	-21.22	737487	24	2227	141
miR1874-3p	LOC_Os01g72570.3	154	-21.22	154	-21.22	737488	24	2110	141
miR1874-3p	LOC_Os01g72620.1	159	-21.32	159	-21.32	737492	24	1233	358
miR1874-3p	LOC_Os01g72720.1	149	-26.39	149	-26.39	737505	24	1155	390
miR1874-3p	LOC_Os01g72880.1	158	-22.86	158	-22.86	737525	24	2623	2188
miR1874-3p	LOC_Os01g72940.1	160	-21.79	160	-21.79	737532	24	2180	1612
miR1874-3p	LOC_Os01g72990.1	158	-22.67	158	-22.67	737538	24	3992	3613
miR1874-3p	LOC_Os01g73070.1	160	-21.38	160	-21.38	737546	24	1450	40
miR1874-3p	LOC_Os01g73070.2	160	-21.38	160	-21.38	737547	24	2938	40
miR1874-3p	LOC_Os01g73150.1	164	-20.37	164	-20.37	737557	24	2154	378
miR1874-3p	LOC_Os01g73580.1	159	-23.01	159	-23.01	737603	24	2186	1176
miR1874-3p	LOC_Os01g73910.1	168	-24.05	168	-24.05	737642	24	1591	165
miR1874-3p	LOC_Os01g73910.2	168	-24.05	168	-24.05	737643	24	1488	169
miR1874-3p	LOC_Os01g73910.3	168	-24.05	168	-24.05	737644	24	1414	104
miR1874-3p	LOC_Os01g73910.4	168	-24.05	168	-24.05	737645	24	1665	165
miR1874-3p	LOC_Os01g73910.5	168	-24.05	168	-24.05	737646	24	1593	165
miR1874-3p	LOC_Os01g73910.6	168	-24.05	168	-24.05	737647	24	1486	165
miR1874-3p	LOC_Os01g73930.1	154	-23.58	154	-23.58	737649	24	3249	3002
miR1874-3p	LOC_Os01g73970.1	153	-21.16	153	-21.16	737655	24	1956	552
miR1874-3p	LOC_Os01g74410.1	156	-20.37	156	-20.37	737706	24	1363	897
miR1874-3p	LOC_Os01g74410.2	156	-20.37	156	-20.37	737707	24	1583	1117
miR1874-3p	LOC_Os01g74510.1	145	-25.05	145	-25.05	737717	24	3404	2100
miR1874-3p	LOC_Os01g74690.1	156	-20.06	156	-20.06	737741	24	3105	180
miR1874-3p	LOC_Os02g01010.1	153	-22.17	153	-22.17	737742	24	2164	607
miR1874-3p	LOC_Os02g01060.1	149	-23.85	149	-23.85	737751	24	939	132
miR1874-3p	LOC_Os02g01250.1	162	-21.85	162	-21.85	737779	24	930	185
miR1874-3p	LOC_Os02g01275.1	496	-81.7	169	-29.63	737782	24	1095	287 166 329
miR1874-3p	LOC_Os02g01400.1	156	-20.06	156	-20.06	737798	24	2973	180

miR1874-3p	LOC_Os02g01610.1	156	-21.26	156	-21.26	737820	24	2997	1841
miR1874-3p	LOC_Os02g01610.2	156	-21.26	156	-21.26	737821	24	3319	2163
miR1874-3p	LOC_Os02g01790.1	154	-21.85	154	-21.85	737842	24	3199	296
miR1874-3p	LOC_Os02g01790.2	154	-21.85	154	-21.85	737843	24	3093	287
miR1874-3p	LOC_Os02g01870.1	151	-20.4	151	-20.4	737852	24	1537	1136
miR1874-3p	LOC_Os02g01940.1	171	-24.53	171	-24.53	737859	24	2994	2696
miR1874-3p	LOC_Os02g02020.1	148	-20.09	148	-20.09	737867	24	2665	1450
miR1874-3p	LOC_Os02g02040.1	152	-20.46	152	-20.46	737869	24	2299	2263
miR1874-3p	LOC_Os02g02050.1	153	-21.03	153	-21.03	737875	24	8431	5201
miR1874-3p	LOC_Os02g02120.1	154	-23.09	154	-23.09	737882	24	2858	2657
miR1874-3p	LOC_Os02g02610.1	154	-20.1	154	-20.1	737938	24	3378	2999
miR1874-3p	LOC_Os02g02650.1	155	-24.2	155	-24.2	737942	24	279	142
miR1874-3p	LOC_Os02g02780.1	150	-20.15	150	-20.15	737963	24	2250	766
miR1874-3p	LOC_Os02g03020.1	159	-26.53	159	-26.53	737996	24	856	2
miR1874-3p	LOC_Os02g03240.1	163	-23.56	163	-23.56	738027	24	2697	783
miR1874-3p	LOC_Os02g03480.1	159	-23.32	159	-23.32	738058	24	1111	852
miR1874-3p	LOC_Os02g03480.2	159	-23.32	159	-23.32	738059	24	1029	785
miR1874-3p	LOC_Os02g03560.2	155	-20.83	155	-20.83	738068	24	2498	1241
miR1874-3p	LOC_Os02g03780.1	168	-26.04	168	-26.04	738099	24	165	137
miR1874-3p	LOC_Os02g03950.3	150	-22.96	150	-22.96	738120	24	2112	1312
miR1874-3p	LOC_Os02g04040.1	158	-20.77	158	-20.77	738136	24	1819	1777
miR1874-3p	LOC_Os02g04410.1	145	-20.48	145	-20.48	738184	24	771	737
miR1874-3p	LOC_Os02g04440.2	169	-32.69	169	-32.69	738189	24	4787	652
miR1874-3p	LOC_Os02g04570.1	160	-24.28	160	-24.28	738210	24	2103	1942
miR1874-3p	LOC_Os02g04660.1	153	-21.98	153	-21.98	738219	24	2384	1713
miR1874-3p	LOC_Os02g04840.1	161	-23.85	161	-23.85	738248	24	4103	1934
miR1874-3p	LOC_Os02g04910.1	308	-47.16	154	-23.58	738255	24	5157	4301 4778
miR1874-3p	LOC_Os02g04924.1	156	-20.44	156	-20.44	738257	24	2621	567
miR1874-3p	LOC_Os02g04924.2	156	-20.44	156	-20.44	738258	24	2625	567
miR1874-3p	LOC_Os02g04950.1	158	-22.23	158	-22.23	738261	24	4361	207
miR1874-3p	LOC_Os02g05260.1	176	-29.95	176	-29.95	738298	24	3996	2670
miR1874-3p	LOC_Os02g05390.1	148	-23.72	148	-23.72	738319	24	1227	725

miR1874-3p	LOC_Os02g05630.1	164	-21.65	164	-21.65	738347	24	1773	102
miR1874-3p	LOC_Os02g05630.2	164	-21.65	164	-21.65	738348	24	1961	102
miR1874-3p	LOC_Os02g05630.3	164	-21.65	164	-21.65	738349	24	2010	102
miR1874-3p	LOC_Os02g05630.4	164	-21.65	164	-21.65	738350	24	2525	102
miR1874-3p	LOC_Os02g05880.1	159	-22.28	159	-22.28	738381	24	6900	1351
miR1874-3p	LOC_Os02g06000.1	150	-21.12	150	-21.12	738393	24	2493	2246
miR1874-3p	LOC_Os02g06100.1	160	-20.87	160	-20.87	738403	24	570	179
miR1874-3p	LOC_Os02g06550.1	146	-22.29	146	-22.29	738453	24	441	373
miR1874-3p	LOC_Os02g06830.1	165	-21.06	165	-21.06	738492	24	3642	3446
miR1874-3p	LOC_Os02g06830.2	165	-21.06	165	-21.06	738493	24	3528	3332
miR1874-3p	LOC_Os02g07200.1	140	-22.66	140	-22.66	738542	24	2254	1119
miR1874-3p	LOC_Os02g07200.2	140	-22.66	140	-22.66	738543	24	2348	1213
miR1874-3p	LOC_Os02g07200.3	140	-22.66	140	-22.66	738544	24	2336	1201
miR1874-3p	LOC_Os02g07350.1	154	-24.07	154	-24.07	738566	24	1338	333
miR1874-3p	LOC_Os02g07480.1	160	-29.69	160	-29.69	738580	24	1976	1014
miR1874-3p	LOC_Os02g07480.2	160	-29.69	160	-29.69	738581	24	1971	1014
miR1874-3p	LOC_Os02g07520.1	164	-24.18	164	-24.18	738586	24	5766	2126
miR1874-3p	LOC_Os02g07910.1	154	-23.62	154	-23.62	738640	24	785	79
miR1874-3p	LOC_Os02g07910.2	154	-23.62	154	-23.62	738641	24	803	87
miR1874-3p	LOC_Os02g08210.1	157	-21.07	157	-21.07	738679	24	435	197
miR1874-3p	LOC_Os02g08360.1	153	-20.5	153	-20.5	738696	24	3688	135
miR1874-3p	LOC_Os02g08364.1	156	-22.21	156	-22.21	738697	24	2590	664
miR1874-3p	LOC_Os02g08440.1	150	-23.32	150	-23.32	738705	24	1876	1436
miR1874-3p	LOC_Os02g08440.2	150	-23.32	150	-23.32	738706	24	1833	1393
miR1874-3p	LOC_Os02g08440.3	150	-23.32	150	-23.32	738707	24	1987	1547
miR1874-3p	LOC_Os02g08440.4	150	-23.32	150	-23.32	738708	24	1313	873
miR1874-3p	LOC_Os02g08450.1	156	-20.96	156	-20.96	738709	24	1968	1160
miR1874-3p	LOC_Os02g09450.1	161	-20.92	161	-20.92	738764	24	2540	131
miR1874-3p	LOC_Os02g09450.3	161	-20.92	161	-20.92	738765	24	2387	80
miR1874-3p	LOC_Os02g09990.1	147	-20.43	147	-20.43	738831	24	1317	275
miR1874-3p	LOC_Os02g10950.1	158	-20.6	158	-20.6	738957	24	851	185
miR1874-3p	LOC_Os02g11050.1	151	-20.23	151	-20.23	738972	24	1770	802

miR1874-3p	LOC_Os02g11070.1	150	-22.69	150	-22.69	738974	24	2035	396
miR1874-3p	LOC_Os02g11070.2	150	-22.69	150	-22.69	738975	24	2054	396
miR1874-3p	LOC_Os02g11780.1	156	-20.97	156	-20.97	739002	24	3997	7
miR1874-3p	LOC_Os02g12300.1	142	-20.96	142	-20.96	739057	24	1881	1691
miR1874-3p	LOC_Os02g12810.1	144	-21.47	144	-21.47	739129	24	3222	475
miR1874-3p	LOC_Os02g12810.2	144	-21.47	144	-21.47	739130	24	3487	475
miR1874-3p	LOC_Os02g12810.4	144	-21.47	144	-21.47	739132	24	2987	475
miR1874-3p	LOC_Os02g12970.1	176	-33.4	176	-33.4	739158	24	3933	2043
miR1874-3p	LOC_Os02g13030.1	171	-26.48	171	-26.48	739164	24	450	277
miR1874-3p	LOC_Os02g13260.1	160	-26.64	160	-26.64	739190	24	1608	102
miR1874-3p	LOC_Os02g13380.1	153	-24.67	153	-24.67	739203	24	839	211
miR1874-3p	LOC_Os02g13460.1	152	-21.26	152	-21.26	739212	24	1605	406
miR1874-3p	LOC_Os02g13480.1	163	-26.53	163	-26.53	739214	24	783	125
miR1874-3p	LOC_Os02g13500.1	317	-45.51	160	-25.02	739216	24	1920	1372 310
miR1874-3p	LOC_Os02g13560.3	157	-22.07	157	-22.07	739225	24	3025	46
miR1874-3p	LOC_Os02g14440.1	150	-21.91	150	-21.91	739321	24	1300	1115
miR1874-3p	LOC_Os02g14440.2	150	-21.91	150	-21.91	739322	24	1435	1250
miR1874-3p	LOC_Os02g14510.1	157	-22.94	157	-22.94	739329	24	327	190
miR1874-3p	LOC_Os02g14520.1	166	-26.14	166	-26.14	739330	24	2940	1304
miR1874-3p	LOC_Os02g14880.1	158	-22.29	158	-22.29	739378	24	387	42
miR1874-3p	LOC_Os02g14890.1	161	-21.48	161	-21.48	739379	24	792	17
miR1874-3p	LOC_Os02g15060.1	160	-20.77	160	-20.77	739393	24	903	316
miR1874-3p	LOC_Os02g15580.1	148	-21.52	148	-21.52	739455	24	2128	1134
miR1874-3p	LOC_Os02g15580.3	148	-21.52	148	-21.52	739457	24	3157	1134
miR1874-3p	LOC_Os02g15810.1	158	-20.73	158	-20.73	739479	24	1899	295
miR1874-3p	LOC_Os02g15950.1	150	-21.43	150	-21.43	739500	24	3187	941
miR1874-3p	LOC_Os02g16270.1	464	-65.06	157	-23.96	739536	24	4510	433 1198 88
miR1874-3p	LOC_Os02g16270.2	464	-65.06	157	-23.96	739537	24	4520	433 1198 88
miR1874-3p	LOC_Os02g16450.1	159	-20.9	159	-20.9	739555	24	2018	758
miR1874-3p	LOC_Os02g16909.1	149	-20.21	149	-20.21	739604	24	1449	371
miR1874-3p	LOC_Os02g17250.1	157	-21.59	157	-21.59	739640	24	1592	678
miR1874-3p	LOC_Os02g17390.1	163	-21.37	163	-21.37	739656	24	2871	95

miR1874-3p	LOC_Os02g17500.1	147	-21.41	147	-21.41	739668	24	2242	1950
miR1874-3p	LOC_Os02g17500.2	147	-21.41	147	-21.41	739669	24	2030	1738
miR1874-3p	LOC_Os02g17500.3	147	-21.41	147	-21.41	739670	24	2406	2114
miR1874-3p	LOC_Os02g18115.1	147	-20.34	147	-20.34	739740	24	1737	1209
miR1874-3p	LOC_Os02g18320.1	145	-21.98	145	-21.98	739763	24	2459	1864
miR1874-3p	LOC_Os02g18370.1	160	-24.13	160	-24.13	739768	24	3659	832
miR1874-3p	LOC_Os02g18620.1	160	-25.86	160	-25.86	739796	24	777	405
miR1874-3p	LOC_Os02g18820.1	140	-20.11	140	-20.11	739817	24	2923	1789
miR1874-3p	LOC_Os02g18820.2	140	-20.11	140	-20.11	739818	24	2742	1608
miR1874-3p	LOC_Os02g18890.1	149	-22.12	149	-22.12	739825	24	1554	686
miR1874-3p	LOC_Os02g19030.1	148	-20.99	148	-20.99	739838	24	570	238
miR1874-3p	LOC_Os02g19140.1	152	-24.26	152	-24.26	739848	24	1970	1612
miR1874-3p	LOC_Os02g19750.1	153	-25.07	153	-25.07	739915	24	2703	1626
miR1874-3p	LOC_Os02g19890.1	162	-22.06	162	-22.06	739927	24	3881	759
miR1874-3p	LOC_Os02g19890.2	162	-22.06	162	-22.06	739928	24	5805	701
miR1874-3p	LOC_Os02g19890.3	162	-22.06	162	-22.06	739929	24	5400	701
miR1874-3p	LOC_Os02g20140.1	313	-46.4	160	-24.93	739953	24	2100	1468 1784
miR1874-3p	LOC_Os02g20260.1	156	-20.06	156	-20.06	739965	24	2886	180
miR1874-3p	LOC_Os02g20420.1	164	-23.73	164	-23.73	739984	24	6192	753
miR1874-3p	LOC_Os02g20460.1	314	-47.62	158	-26.98	739987	24	2103	1116 104
miR1874-3p	LOC_Os02g20600.1	157	-20.43	157	-20.43	740001	24	708	683
miR1874-3p	LOC_Os02g20620.1	157	-24.2	157	-24.2	740002	24	786	215
miR1874-3p	LOC_Os02g20720.1	149	-21.68	149	-21.68	740011	24	1173	143
miR1874-3p	LOC_Os02g20970.1	167	-23.67	167	-23.67	740044	24	2217	1143
miR1874-3p	LOC_Os02g21090.1	155	-20.44	155	-20.44	740055	24	2383	362
miR1874-3p	LOC_Os02g21090.2	155	-20.44	155	-20.44	740056	24	2622	362
miR1874-3p	LOC_Os02g21090.3	155	-20.44	155	-20.44	740057	24	2836	362
miR1874-3p	LOC_Os02g21340.1	153	-21.34	153	-21.34	740085	24	3507	3168
miR1874-3p	LOC_Os02g21850.1	148	-22.37	148	-22.37	740138	24	492	48
miR1874-3p	LOC_Os02g22080.1	164	-24.99	164	-24.99	740162	24	3207	1867
miR1874-3p	LOC_Os02g22090.1	173	-27.07	173	-27.07	740165	24	4768	1132
miR1874-3p	LOC_Os02g22180.1	145	-20.37	145	-20.37	740179	24	2400	1232

miR1874-3p	LOC_Os02g22340.1	151	-21.67	151	-21.67	740194	24	273	33
miR1874-3p	LOC_Os02g23823.1	156	-22.76	156	-22.76	740253	24	1810	727
miR1874-3p	LOC_Os02g23840.1	151	-24.57	151	-24.57	740263	24	4794	4286
miR1874-3p	LOC_Os02g23930.1	156	-21.14	156	-21.14	740272	24	2583	1096
miR1874-3p	LOC_Os02g23970.1	162	-20.65	162	-20.65	740279	24	1728	1522
miR1874-3p	LOC_Os02g24070.1	166	-33.86	166	-33.86	740289	24	1206	191
miR1874-3p	LOC_Os02g24090.1	152	-20.77	152	-20.77	740295	24	4725	2608
miR1874-3p	LOC_Os02g24250.1	156	-25.26	156	-25.26	740311	24	5142	2001
miR1874-3p	LOC_Os02g24280.1	467	-63.97	160	-23.28	740314	24	3279	1179 3034 769
miR1874-3p	LOC_Os02g24850.1	152	-29.34	152	-29.34	740376	24	342	47
miR1874-3p	LOC_Os02g24890.1	160	-21.28	160	-21.28	740380	24	2154	411
miR1874-3p	LOC_Os02g24950.1	168	-23.99	168	-23.99	740386	24	3249	2910
miR1874-3p	LOC_Os02g24980.1	140	-20.17	140	-20.17	740389	24	1092	400
miR1874-3p	LOC_Os02g25060.1	154	-21.46	154	-21.46	740397	24	2107	728
miR1874-3p	LOC_Os02g25060.2	154	-21.46	154	-21.46	740398	24	2133	701
miR1874-3p	LOC_Os02g25210.1	159	-20.66	159	-20.66	740413	24	501	442
miR1874-3p	LOC_Os02g25580.1	150	-22.22	150	-22.22	740448	24	2700	1535
miR1874-3p	LOC_Os02g25580.2	150	-22.22	150	-22.22	740449	24	2403	1535
miR1874-3p	LOC_Os02g25590.1	164	-27.89	164	-27.89	740450	24	1116	889
miR1874-3p	LOC_Os02g25600.1	160	-20.74	160	-20.74	740451	24	1260	564
miR1874-3p	LOC_Os02g25830.1	151	-21.71	151	-21.71	740477	24	819	475
miR1874-3p	LOC_Os02g25930.1	156	-22.73	156	-22.73	740488	24	3936	3627
miR1874-3p	LOC_Os02g25980.1	159	-20.76	159	-20.76	740493	24	213	68
miR1874-3p	LOC_Os02g26014.1	164	-26.5	164	-26.5	740495	24	1997	1471
miR1874-3p	LOC_Os02g26160.1	162	-23.5	162	-23.5	740507	24	2240	50
miR1874-3p	LOC_Os02g26160.2	162	-23.5	162	-23.5	740508	24	2534	50
miR1874-3p	LOC_Os02g26660.1	325	-45.96	168	-24.49	740571	24	1469	678 281
miR1874-3p	LOC_Os02g26660.2	325	-45.96	168	-24.49	740572	24	1335	680 283
miR1874-3p	LOC_Os02g27110.1	155	-20.58	155	-20.58	740631	24	9893	4220
miR1874-3p	LOC_Os02g27160.1	153	-20.51	153	-20.51	740636	24	2151	418
miR1874-3p	LOC_Os02g27870.1	145	-24.67	145	-24.67	740716	24	2396	525
miR1874-3p	LOC_Os02g28560.1	167	-22.77	167	-22.77	740783	24	537	515

miR1874-3p	LOC_Os02g28820.1	159	-25.98	159	-25.98	740807	24	1379	534
miR1874-3p	LOC_Os02g28970.1	150	-21.2	150	-21.2	740822	24	2023	35
miR1874-3p	LOC_Os02g28970.2	150	-21.2	150	-21.2	740823	24	1986	35
miR1874-3p	LOC_Os02g28990.1	155	-20.03	155	-20.03	740825	24	579	161
miR1874-3p	LOC_Os02g29410.1	148	-22	148	-22	740871	24	2799	2664
miR1874-3p	LOC_Os02g29530.1	159	-22.23	159	-22.23	740882	24	2049	816
miR1874-3p	LOC_Os02g29600.1	161	-24.56	161	-24.56	740890	24	1104	275
miR1874-3p	LOC_Os02g29640.1	156	-21.28	156	-21.28	740894	24	1956	861
miR1874-3p	LOC_Os02g29890.1	150	-23.01	150	-23.01	740920	24	3063	1831
miR1874-3p	LOC_Os02g29940.1	159	-24.69	159	-24.69	740925	24	642	201
miR1874-3p	LOC_Os02g29950.1	169	-28.72	169	-28.72	740926	24	2310	1839
miR1874-3p	LOC_Os02g30210.1	159	-20.71	159	-20.71	740957	24	2150	1941
miR1874-3p	LOC_Os02g30210.2	159	-20.71	159	-20.71	740958	24	2046	1941
miR1874-3p	LOC_Os02g30400.1	151	-20.1	151	-20.1	740980	24	2430	2390
miR1874-3p	LOC_Os02g30680.1	320	-40.67	160	-20.49	741016	24	2877	302 2527
miR1874-3p	LOC_Os02g30730.1	169	-26.29	169	-26.29	741020	24	3090	1363
miR1874-3p	LOC_Os02g30730.2	169	-26.29	169	-26.29	741021	24	3164	1437
miR1874-3p	LOC_Os02g30780.1	150	-22.72	150	-22.72	741026	24	816	41
miR1874-3p	LOC_Os02g30800.1	320	-41.66	162	-21.13	741028	24	6861	2070 1569
miR1874-3p	LOC_Os02g30880.1	157	-21.8	157	-21.8	741037	24	3891	1890
miR1874-3p	LOC_Os02g32060.1	157	-26.24	157	-26.24	741116	24	1413	688
miR1874-3p	LOC_Os02g32060.2	157	-26.24	157	-26.24	741117	24	1419	685
miR1874-3p	LOC_Os02g32060.3	157	-26.24	157	-26.24	741118	24	1248	685
miR1874-3p	LOC_Os02g32590.1	161	-22.64	161	-22.64	741180	24	2166	621
miR1874-3p	LOC_Os02g32590.2	161	-22.64	161	-22.64	741181	24	2022	621
miR1874-3p	LOC_Os02g32600.1	154	-24.99	154	-24.99	741182	24	1386	284
miR1874-3p	LOC_Os02g32860.1	163	-25.21	163	-25.21	741210	24	2918	1514
miR1874-3p	LOC_Os02g33000.1	169	-25.88	169	-25.88	741224	24	2215	1790
miR1874-3p	LOC_Os02g33010.1	169	-25.88	169	-25.88	741225	24	2443	387
miR1874-3p	LOC_Os02g33110.1	160	-25.57	160	-25.57	741237	24	2192	1093
miR1874-3p	LOC_Os02g33110.2	160	-25.57	160	-25.57	741238	24	2329	1093
miR1874-3p	LOC_Os02g33110.3	160	-25.57	160	-25.57	741239	24	2372	1093

miR1874-3p	LOC_Os02g33280.1	167	-23.96	167	-23.96	741256	24	3936	2831
miR1874-3p	LOC_Os02g33630.1	151	-21.89	151	-21.89	741297	24	2019	1346
miR1874-3p	LOC_Os02g33680.1	151	-22.32	151	-22.32	741302	24	1518	135
miR1874-3p	LOC_Os02g34330.1	145	-20.07	145	-20.07	741361	24	1323	575
miR1874-3p	LOC_Os02g34430.1	158	-21.47	158	-21.47	741370	24	2482	268
miR1874-3p	LOC_Os02g34650.1	165	-22.47	165	-22.47	741397	24	1820	181
miR1874-3p	LOC_Os02g34940.1	157	-23.93	157	-23.93	741425	24	1944	423
miR1874-3p	LOC_Os02g34940.2	157	-23.93	157	-23.93	741426	24	1812	423
miR1874-3p	LOC_Os02g34990.2	162	-20.4	162	-20.4	741432	24	2390	97
miR1874-3p	LOC_Os02g35270.1	150	-21.18	150	-21.18	741470	24	1569	1530
miR1874-3p	LOC_Os02g35329.1	158	-21.57	158	-21.57	741480	24	1198	16
miR1874-3p	LOC_Os02g35570.1	158	-21.02	158	-21.02	741497	24	906	373
miR1874-3p	LOC_Os02g35870.1	156	-22.31	156	-22.31	741536	24	2518	1307
miR1874-3p	LOC_Os02g35870.2	156	-22.31	156	-22.31	741537	24	2094	1307
miR1874-3p	LOC_Os02g36060.1	157	-28.9	157	-28.9	741556	24	3207	851
miR1874-3p	LOC_Os02g36280.1	151	-24.99	151	-24.99	741580	24	1997	1785
miR1874-3p	LOC_Os02g36600.1	161	-25.99	161	-25.99	741627	24	1421	1286
miR1874-3p	LOC_Os02g36974.1	148	-20.91	148	-20.91	741672	24	1447	272
miR1874-3p	LOC_Os02g36974.2	148	-20.91	148	-20.91	741673	24	1470	295
miR1874-3p	LOC_Os02g36974.3	148	-20.91	148	-20.91	741674	24	2659	1484
miR1874-3p	LOC_Os02g36974.4	148	-20.91	148	-20.91	741675	24	1434	259
miR1874-3p	LOC_Os02g36974.5	148	-20.91	148	-20.91	741676	24	1152	295
miR1874-3p	LOC_Os02g37050.1	152	-22.05	152	-22.05	741683	24	3179	1379
miR1874-3p	LOC_Os02g37109.1	159	-28.06	159	-28.06	741691	24	1170	124
miR1874-3p	LOC_Os02g37109.2	159	-28.06	159	-28.06	741692	24	1105	59
miR1874-3p	LOC_Os02g37140.1	169	-21.24	169	-21.24	741698	24	3511	1529
miR1874-3p	LOC_Os02g37140.2	169	-21.24	169	-21.24	741699	24	3506	1524
miR1874-3p	LOC_Os02g37510.1	146	-22.19	146	-22.19	741736	24	1434	377
miR1874-3p	LOC_Os02g37690.1	140	-20.05	140	-20.05	741752	24	1999	1866
miR1874-3p	LOC_Os02g37790.1	151	-21.7	151	-21.7	741763	24	1227	841
miR1874-3p	LOC_Os02g38220.1	149	-21.82	149	-21.82	741827	24	2091	1753
miR1874-3p	LOC_Os02g38250.1	156	-22.52	156	-22.52	741830	24	2697	1952

miR1874-3p	LOC_Os02g38570.1	156	-20.17	156	-20.17	741869	24	3141	180
miR1874-3p	LOC_Os02g38640.1	158	-21.02	158	-21.02	741877	24	873	367
miR1874-3p	LOC_Os02g38670.1	156	-20.17	156	-20.17	741880	24	2985	180
miR1874-3p	LOC_Os02g38840.1	161	-21.19	161	-21.19	741901	24	2114	155
miR1874-3p	LOC_Os02g38840.2	161	-21.19	161	-21.19	741902	24	2278	49
miR1874-3p	LOC_Os02g39160.1	148	-25.02	148	-25.02	741946	24	2688	1491
miR1874-3p	LOC_Os02g39670.1	172	-28.25	172	-28.25	742014	24	1695	1281
miR1874-3p	LOC_Os02g39720.2	165	-23.09	165	-23.09	742018	24	1405	1038
miR1874-3p	LOC_Os02g39720.3	165	-23.09	165	-23.09	742019	24	1396	1029
miR1874-3p	LOC_Os02g39720.4	165	-23.09	165	-23.09	742020	24	1369	1002
miR1874-3p	LOC_Os02g39720.6	165	-23.09	165	-23.09	742021	24	1568	1029
miR1874-3p	LOC_Os02g39900.1	154	-22.17	154	-22.17	742046	24	3081	2852
miR1874-3p	LOC_Os02g39930.1	153	-22.16	153	-22.16	742049	24	962	148
miR1874-3p	LOC_Os02g40080.1	165	-20.87	165	-20.87	742074	24	1324	519
miR1874-3p	LOC_Os02g40140.1	148	-22.81	148	-22.81	742083	24	1392	378
miR1874-3p	LOC_Os02g40410.1	155	-21.63	155	-21.63	742113	24	3133	367
miR1874-3p	LOC_Os02g40410.2	155	-21.63	155	-21.63	742114	24	3109	366
miR1874-3p	LOC_Os02g40420.1	152	-20.26	152	-20.26	742115	24	2112	1117
miR1874-3p	LOC_Os02g40784.1	153	-22.71	153	-22.71	742154	24	2484	481
miR1874-3p	LOC_Os02g40784.3	153	-22.71	153	-22.71	742155	24	2363	481
miR1874-3p	LOC_Os02g40940.1	159	-26.11	159	-26.11	742175	24	2625	1485
miR1874-3p	LOC_Os02g41610.1	161	-24.69	161	-24.69	742197	24	3058	2516
miR1874-3p	LOC_Os02g41750.1	154	-24.99	154	-24.99	742213	24	828	311
miR1874-3p	LOC_Os02g41850.1	153	-20.99	153	-20.99	742224	24	1062	191
miR1874-3p	LOC_Os02g42020.1	166	-21.05	166	-21.05	742246	24	2660	1168
miR1874-3p	LOC_Os02g42020.2	166	-21.05	166	-21.05	742247	24	2085	1168
miR1874-3p	LOC_Os02g42020.3	166	-21.05	166	-21.05	742248	24	1863	371
miR1874-3p	LOC_Os02g42150.1	164	-22.73	164	-22.73	742260	24	3218	2154
miR1874-3p	LOC_Os02g42150.2	164	-22.73	164	-22.73	742261	24	2969	2049
miR1874-3p	LOC_Os02g42200.1	161	-25.21	161	-25.21	742266	24	5793	324
miR1874-3p	LOC_Os02g42300.1	155	-23.19	155	-23.19	742277	24	3003	1992
miR1874-3p	LOC_Os02g42370.1	144	-20.72	144	-20.72	742294	24	3371	2904

miR1874-3p	LOC_Os02g42530.1	158	-23.96	158	-23.96	742315	24	2719	1757
miR1874-3p	LOC_Os02g42530.2	158	-23.96	158	-23.96	742316	24	3080	1757
miR1874-3p	LOC_Os02g42530.3	158	-23.96	158	-23.96	742317	24	2801	1839
miR1874-3p	LOC_Os02g44360.1	154	-23.52	154	-23.52	742553	24	2747	1592
miR1874-3p	LOC_Os02g44780.2	151	-21.44	151	-21.44	742611	24	2155	805
miR1874-3p	LOC_Os02g45130.1	145	-21.69	145	-21.69	742656	24	2664	1146
miR1874-3p	LOC_Os02g45130.2	145	-21.69	145	-21.69	742657	24	2011	1142
miR1874-3p	LOC_Os02g45200.1	142	-20.33	142	-20.33	742664	24	2355	2043
miR1874-3p	LOC_Os02g45230.1	164	-24.23	164	-24.23	742671	24	366	291
miR1874-3p	LOC_Os02g45250.1	153	-20.32	153	-20.32	742673	24	3437	1789
miR1874-3p	LOC_Os02g46200.1	153	-24.49	153	-24.49	742777	24	2880	902
miR1874-3p	LOC_Os02g46680.1	160	-25.29	160	-25.29	742835	24	4342	2618
miR1874-3p	LOC_Os02g47570.1	145	-21.79	145	-21.79	742954	24	1219	907
miR1874-3p	LOC_Os02g47590.1	158	-25.35	158	-25.35	742957	24	1481	665
miR1874-3p	LOC_Os02g47730.1	164	-24.18	164	-24.18	742976	24	6282	2597
miR1874-3p	LOC_Os02g47980.1	162	-20.84	162	-20.84	743007	24	1300	1066
miR1874-3p	LOC_Os02g48260.1	177	-27.25	177	-27.25	743041	24	3060	17
miR1874-3p	LOC_Os02g48300.1	162	-23.33	162	-23.33	743045	24	1368	193
miR1874-3p	LOC_Os02g48930.1	161	-21.39	161	-21.39	743130	24	2460	1227
miR1874-3p	LOC_Os02g49090.1	144	-22.86	144	-22.86	743145	24	4487	3227
miR1874-3p	LOC_Os02g49090.2	144	-22.86	144	-22.86	743146	24	4797	3537
miR1874-3p	LOC_Os02g49640.1	159	-20.84	159	-20.84	743218	24	898	67
miR1874-3p	LOC_Os02g49720.1	148	-22.94	148	-22.94	743228	24	2823	1708
miR1874-3p	LOC_Os02g49720.2	148	-22.94	148	-22.94	743229	24	2757	1618
miR1874-3p	LOC_Os02g49720.3	148	-22.94	148	-22.94	743230	24	2733	1618
miR1874-3p	LOC_Os02g49720.6	148	-22.94	148	-22.94	743231	24	2942	1618
miR1874-3p	LOC_Os02g49720.7	148	-22.94	148	-22.94	743232	24	2815	1700
miR1874-3p	LOC_Os02g50270.1	183	-26.94	183	-26.94	743296	24	1980	1894
miR1874-3p	LOC_Os02g50360.1	143	-21.55	143	-21.55	743307	24	2689	219
miR1874-3p	LOC_Os02g50799.1	149	-21.78	149	-21.78	743360	24	2851	1999
miR1874-3p	LOC_Os02g51119.1	159	-22.61	159	-22.61	743398	24	1732	951
miR1874-3p	LOC_Os02g51150.1	159	-23.38	159	-23.38	743403	24	1577	807

miR1874-3p	LOC_Os02g51150.2	159	-23.38	159	-23.38	743404	24	1550	780
miR1874-3p	LOC_Os02g51330.1	160	-21.77	160	-21.77	743424	24	2707	1541
miR1874-3p	LOC_Os02g51330.2	160	-21.77	160	-21.77	743425	24	2698	1541
miR1874-3p	LOC_Os02g51390.1	154	-23.94	154	-23.94	743431	24	3752	1072
miR1874-3p	LOC_Os02g51390.2	154	-23.94	154	-23.94	743432	24	3374	1072
miR1874-3p	LOC_Os02g51510.1	158	-22.82	158	-22.82	743454	24	684	577
miR1874-3p	LOC_Os02g51530.1	156	-20.06	156	-20.06	743456	24	3135	180
miR1874-3p	LOC_Os02g51580.1	160	-20.91	160	-20.91	743461	24	534	316
miR1874-3p	LOC_Os02g51890.1	164	-20.82	164	-20.82	743501	24	2398	2097
miR1874-3p	LOC_Os02g51890.2	318	-42.2	164	-21.38	743502	24	4440	4139 2638
miR1874-3p	LOC_Os02g51900.1	164	-20.82	164	-20.82	743503	24	2142	791
miR1874-3p	LOC_Os02g52440.1	154	-22.38	154	-22.38	743571	24	321	66
miR1874-3p	LOC_Os02g53110.1	324	-45.75	168	-24.73	743652	24	3884	1114 2053
miR1874-3p	LOC_Os02g53130.1	160	-20.31	160	-20.31	743655	24	3060	77
miR1874-3p	LOC_Os02g53220.1	152	-20.54	152	-20.54	743672	24	276	50
miR1874-3p	LOC_Os02g53440.1	168	-25.07	168	-25.07	743700	24	858	125
miR1874-3p	LOC_Os02g53450.2	147	-23.79	147	-23.79	743701	24	2344	1001
miR1874-3p	LOC_Os02g53970.1	450	-63.29	152	-21.82	743767	24	2624	110 1674 1491
miR1874-3p	LOC_Os02g54600.1	161	-23.56	161	-23.56	743844	24	1880	601
miR1874-3p	LOC_Os02g54650.1	156	-20.24	156	-20.24	743848	24	2107	591
miR1874-3p	LOC_Os02g54770.1	159	-20.02	159	-20.02	743859	24	1398	1025
miR1874-3p	LOC_Os02g54770.2	159	-20.02	159	-20.02	743860	24	991	618
miR1874-3p	LOC_Os02g54820.1	148	-21.41	148	-21.41	743865	24	3664	2537
miR1874-3p	LOC_Os02g54820.2	148	-21.41	148	-21.41	743866	24	3154	2537
miR1874-3p	LOC_Os02g54820.3	148	-21.41	148	-21.41	743867	24	3554	2937
miR1874-3p	LOC_Os02g54870.1	156	-20.15	156	-20.15	743873	24	1541	400
miR1874-3p	LOC_Os02g54950.1	158	-24.05	158	-24.05	743881	24	1167	788
miR1874-3p	LOC_Os02g54960.1	167	-22.53	167	-22.53	743882	24	1191	794
miR1874-3p	LOC_Os02g55030.1	170	-20.57	170	-20.57	743889	24	2811	788
miR1874-3p	LOC_Os02g55310.1	156	-25.56	156	-25.56	743928	24	1982	1646
miR1874-3p	LOC_Os02g55440.1	158	-25.57	158	-25.57	743942	24	2613	743
miR1874-3p	LOC_Os02g55440.2	158	-25.57	158	-25.57	743943	24	2508	638

miR1874-3p	LOC_Os02g55649.1	160	-24.62	160	-24.62	743967	24	710	438
miR1874-3p	LOC_Os02g55800.1	160	-22.62	160	-22.62	743987	24	600	558
miR1874-3p	LOC_Os02g55810.1	160	-24.62	160	-24.62	743988	24	681	412
miR1874-3p	LOC_Os02g56219.1	163	-20.33	163	-20.33	744044	24	1288	736
miR1874-3p	LOC_Os02g56480.1	155	-20.34	155	-20.34	744070	24	2222	238
miR1874-3p	LOC_Os02g56550.1	162	-28.13	162	-28.13	744081	24	1311	564
miR1874-3p	LOC_Os02g56660.1	156	-20.06	156	-20.06	744094	24	3186	180
miR1874-3p	LOC_Os02g56900.1	171	-28.79	171	-28.79	744122	24	1422	190
miR1874-3p	LOC_Os02g57080.1	155	-20.05	155	-20.05	744147	24	3209	1619
miR1874-3p	LOC_Os02g57080.2	155	-20.05	155	-20.05	744148	24	2741	1619
miR1874-3p	LOC_Os02g57110.2	164	-27.98	164	-27.98	744152	24	1668	1224
miR1874-3p	LOC_Os02g57140.2	164	-24.37	164	-24.37	744156	24	3772	448
miR1874-3p	LOC_Os02g57240.1	165	-23.32	165	-23.32	744168	24	1365	1320
miR1874-3p	LOC_Os02g57250.1	166	-23.03	166	-23.03	744169	24	1302	650
miR1874-3p	LOC_Os02g57250.2	166	-23.03	166	-23.03	744170	24	1299	650
miR1874-3p	LOC_Os02g57350.1	327	-44.69	167	-23.67	744190	24	1096	97 1
miR1874-3p	LOC_Os02g57460.1	165	-22.28	165	-22.28	744214	24	1624	553
miR1874-3p	LOC_Os02g57530.3	159	-20.77	159	-20.77	744226	24	4184	246
miR1874-3p	LOC_Os02g57820.1	151	-21.59	151	-21.59	744267	24	2350	1664
miR1874-3p	LOC_Os02g58139.1	153	-21.25	153	-21.25	744309	24	3370	2219
miR1874-3p	LOC_Os02g58139.2	153	-21.25	153	-21.25	744310	24	2899	1748
miR1874-3p	LOC_Os02g58214.1	157	-20.21	157	-20.21	744320	24	1765	1197
miR1874-3p	LOC_Os02g58280.1	143	-22.43	143	-22.43	744328	24	1656	319
miR1874-3p	LOC_Os02g58330.1	159	-20.32	159	-20.32	744334	24	2148	1580
miR1874-3p	LOC_Os02g58340.1	159	-20.32	159	-20.32	744335	24	3247	400
miR1874-3p	LOC_Os02g58460.1	316	-43.74	164	-22.14	744347	24	1911	502 1777
miR1874-3p	LOC_Os02g58480.1	161	-25.77	161	-25.77	744349	24	3550	1025
miR1874-3p	LOC_Os02g58550.1	153	-21.59	153	-21.59	744356	24	928	275
miR1874-3p	LOC_Os02g58550.2	153	-21.59	153	-21.59	744357	24	922	269
miR1874-3p	LOC_Os02g58580.5	170	-28.24	170	-28.24	744366	24	1094	141
miR1874-3p	LOC_Os02g58590.1	165	-22.3	165	-22.3	744367	24	1347	227
miR1874-3p	LOC_Os02g58670.2	155	-21.2	155	-21.2	744377	24	1225	656

miR1874-3p	LOC_Os03g01014.1	157	-26.55	157	-26.55	744400	24	2010	828
miR1874-3p	LOC_Os03g01570.1	146	-21.51	146	-21.51	744472	24	1857	1263
miR1874-3p	LOC_Os03g01570.2	146	-21.51	146	-21.51	744473	24	1713	1119
miR1874-3p	LOC_Os03g01590.1	165	-22.72	165	-22.72	744476	24	1879	758
miR1874-3p	LOC_Os03g01590.2	165	-22.72	165	-22.72	744477	24	1702	584
miR1874-3p	LOC_Os03g01710.1	149	-20.81	149	-20.81	744489	24	4964	4740
miR1874-3p	LOC_Os03g01760.1	150	-20.25	150	-20.25	744504	24	1886	1511
miR1874-3p	LOC_Os03g01760.2	150	-20.25	150	-20.25	744505	24	1881	1506
miR1874-3p	LOC_Os03g01950.1	154	-23.53	154	-23.53	744531	24	4686	4307
miR1874-3p	LOC_Os03g02140.1	160	-20.49	160	-20.49	744559	24	3318	3073
miR1874-3p	LOC_Os03g02210.1	147	-22.57	147	-22.57	744573	24	1715	585
miR1874-3p	LOC_Os03g02240.1	150	-23.56	150	-23.56	744575	24	3313	2251
miR1874-3p	LOC_Os03g02340.1	163	-23.56	163	-23.56	744587	24	1548	1221
miR1874-3p	LOC_Os03g02360.1	156	-20.06	156	-20.06	744589	24	3165	180
miR1874-3p	LOC_Os03g02440.1	164	-25.76	164	-25.76	744600	24	4464	1812
miR1874-3p	LOC_Os03g02440.2	164	-25.76	164	-25.76	744601	24	4051	1503
miR1874-3p	LOC_Os03g02590.2	149	-22.8	149	-22.8	744620	24	2567	1701
miR1874-3p	LOC_Os03g02590.3	149	-22.8	149	-22.8	744621	24	2495	1701
miR1874-3p	LOC_Os03g02590.4	149	-22.8	149	-22.8	744622	24	2796	1701
miR1874-3p	LOC_Os03g02740.1	157	-24.19	157	-24.19	744644	24	2888	2645
miR1874-3p	LOC_Os03g02890.1	152	-21.24	152	-21.24	744668	24	2373	2146
miR1874-3p	LOC_Os03g03060.1	160	-20.49	160	-20.49	744686	24	4311	4066
miR1874-3p	LOC_Os03g03070.1	160	-23.12	160	-23.12	744687	24	1278	565
miR1874-3p	LOC_Os03g03240.1	154	-23.05	154	-23.05	744707	24	2466	281
miR1874-3p	LOC_Os03g03460.1	167	-24.16	167	-24.16	744734	24	1531	1234
miR1874-3p	LOC_Os03g03470.1	150	-20.26	150	-20.26	744735	24	1269	249
miR1874-3p	LOC_Os03g03470.2	150	-20.26	150	-20.26	744736	24	1769	269
miR1874-3p	LOC_Os03g03470.3	150	-20.26	150	-20.26	744737	24	1675	269
miR1874-3p	LOC_Os03g03470.4	150	-20.26	150	-20.26	744738	24	1759	269
miR1874-3p	LOC_Os03g03690.1	310	-42.38	157	-21.32	744766	24	2630	139 71
miR1874-3p	LOC_Os03g03690.2	310	-42.38	157	-21.32	744767	24	2449	140 72
miR1874-3p	LOC_Os03g03690.3	310	-42.38	157	-21.32	744768	24	2671	183 115

miR1874-3p	LOC_Os03g03810.1	164	-24.17	164	-24.17	744783	24	579	455
miR1874-3p	LOC_Os03g03830.1	301	-42.49	155	-22.04	744785	24	3905	1975 1259
miR1874-3p	LOC_Os03g04300.3	161	-21.72	161	-21.72	744838	24	1922	630
miR1874-3p	LOC_Os03g04550.1	154	-20.06	154	-20.06	744870	24	1715	1503
miR1874-3p	LOC_Os03g04950.1	163	-24.39	163	-24.39	744916	24	2585	137
miR1874-3p	LOC_Os03g05340.1	157	-20.46	157	-20.46	744967	24	5763	4709
miR1874-3p	LOC_Os03g05400.1	168	-20.86	168	-20.86	744985	24	1230	1042
miR1874-3p	LOC_Os03g05430.1	154	-22.44	154	-22.44	744989	24	977	160
miR1874-3p	LOC_Os03g05430.2	154	-22.44	154	-22.44	744990	24	750	160
miR1874-3p	LOC_Os03g05710.1	163	-27.4	163	-27.4	745019	24	1385	617
miR1874-3p	LOC_Os03g05850.1	172	-25.17	172	-25.17	745038	24	4230	2871
miR1874-3p	LOC_Os03g06070.1	153	-21.98	153	-21.98	745065	24	2246	995
miR1874-3p	LOC_Os03g06070.2	153	-21.98	153	-21.98	745066	24	1511	995
miR1874-3p	LOC_Os03g06440.1	172	-25.17	172	-25.17	745108	24	4023	76
miR1874-3p	LOC_Os03g06440.2	172	-25.17	172	-25.17	745109	24	3903	72
miR1874-3p	LOC_Os03g06460.1	152	-21.15	152	-21.15	745110	24	2732	76
miR1874-3p	LOC_Os03g06720.1	157	-22.83	157	-22.83	745141	24	2983	977
miR1874-3p	LOC_Os03g06750.1	153	-20.12	153	-20.12	745144	24	1489	301
miR1874-3p	LOC_Os03g06840.1	147	-24.54	147	-24.54	745154	24	765	609
miR1874-3p	LOC_Os03g06860.1	154	-21.21	154	-21.21	745160	24	2327	131
miR1874-3p	LOC_Os03g06910.1	147	-20.81	147	-20.81	745165	24	1947	1169
miR1874-3p	LOC_Os03g07226.1	175	-29.72	175	-29.72	745210	24	1170	321
miR1874-3p	LOC_Os03g07226.2	175	-29.72	175	-29.72	745211	24	1647	321
miR1874-3p	LOC_Os03g07234.1	157	-20.37	157	-20.37	745212	24	1548	98
miR1874-3p	LOC_Os03g07780.1	156	-20.06	156	-20.06	745277	24	3126	180
miR1874-3p	LOC_Os03g08560.1	160	-25.82	160	-25.82	745381	24	3643	3508
miR1874-3p	LOC_Os03g08740.1	154	-22.12	154	-22.12	745406	24	4013	2837
miR1874-3p	LOC_Os03g08740.2	154	-22.12	154	-22.12	745407	24	3955	2837
miR1874-3p	LOC_Os03g08770.1	160	-21.21	160	-21.21	745410	24	1226	301
miR1874-3p	LOC_Os03g08770.2	160	-21.21	160	-21.21	745411	24	1223	298
miR1874-3p	LOC_Os03g08850.1	157	-22.71	157	-22.71	745419	24	2299	931
miR1874-3p	LOC_Os03g09100.1	318	-46.55	159	-24.13	745442	24	3630	372 1371

miR1874-3p	LOC_Os03g09190.1	145	-20.56	145	-20.56	745452	24	2253	306
miR1874-3p	LOC_Os03g09190.2	145	-20.56	145	-20.56	745453	24	2244	297
miR1874-3p	LOC_Os03g09900.1	152	-22.69	152	-22.69	745489	24	1713	783
miR1874-3p	LOC_Os03g10180.1	151	-20.14	151	-20.14	745525	24	2537	665
miR1874-3p	LOC_Os03g10600.1	153	-20.46	153	-20.46	745575	24	3601	1396
miR1874-3p	LOC_Os03g10740.1	152	-20.3	152	-20.3	745589	24	773	109
miR1874-3p	LOC_Os03g10800.1	146	-20.28	146	-20.28	745596	24	3189	1972
miR1874-3p	LOC_Os03g10800.2	146	-20.28	146	-20.28	745597	24	3298	2091
miR1874-3p	LOC_Os03g10800.3	146	-20.28	146	-20.28	745598	24	3518	2311
miR1874-3p	LOC_Os03g10800.4	146	-20.28	146	-20.28	745599	24	3417	1972
miR1874-3p	LOC_Os03g11310.1	157	-25.38	157	-25.38	745658	24	2113	290
miR1874-3p	LOC_Os03g11420.1	168	-25.67	168	-25.67	745676	24	2076	625
miR1874-3p	LOC_Os03g11630.1	155	-21.44	155	-21.44	745703	24	2547	1757
miR1874-3p	LOC_Os03g12064.3	151	-20.14	151	-20.14	745758	24	2210	1179
miR1874-3p	LOC_Os03g12064.6	151	-20.14	151	-20.14	745761	24	2698	1296
miR1874-3p	LOC_Os03g12110.1	153	-21.14	153	-21.14	745764	24	1429	504
miR1874-3p	LOC_Os03g12110.2	153	-21.14	153	-21.14	745765	24	1587	504
miR1874-3p	LOC_Os03g12350.1	171	-29.9	171	-29.9	745799	24	2697	1664
miR1874-3p	LOC_Os03g12350.2	171	-29.9	171	-29.9	745800	24	2628	1664
miR1874-3p	LOC_Os03g12350.4	171	-29.9	171	-29.9	745801	24	3333	1664
miR1874-3p	LOC_Os03g12810.1	147	-20.08	147	-20.08	745855	24	1006	6
miR1874-3p	LOC_Os03g12810.2	147	-20.08	147	-20.08	745856	24	2461	6
miR1874-3p	LOC_Os03g12900.1	143	-20.82	143	-20.82	745873	24	2563	2466
miR1874-3p	LOC_Os03g12900.2	143	-20.82	143	-20.82	745874	24	2658	2462
miR1874-3p	LOC_Os03g12900.3	143	-20.82	143	-20.82	745875	24	2838	2462
miR1874-3p	LOC_Os03g12900.4	143	-20.82	143	-20.82	745876	24	3148	1817
miR1874-3p	LOC_Os03g13340.1	160	-26.91	160	-26.91	745935	24	1149	150
miR1874-3p	LOC_Os03g13720.1	167	-24.09	167	-24.09	745973	24	3002	1720
miR1874-3p	LOC_Os03g13790.1	149	-21.52	149	-21.52	745983	24	2917	1497
miR1874-3p	LOC_Os03g13790.2	149	-21.52	149	-21.52	745984	24	2937	1517
miR1874-3p	LOC_Os03g13960.2	179	-27.85	179	-27.85	746009	24	1370	370
miR1874-3p	LOC_Os03g14080.1	152	-24.13	152	-24.13	746032	24	2004	760

miR1874-3p	LOC_Os03g14790.1	155	-25.88	155	-25.88	746111	24	3007	1874
miR1874-3p	LOC_Os03g15060.1	142	-20.07	142	-20.07	746147	24	396	255
miR1874-3p	LOC_Os03g15770.1	145	-20.59	145	-20.59	746226	24	3059	1663
miR1874-3p	LOC_Os03g15770.2	145	-20.59	145	-20.59	746227	24	2481	1555
miR1874-3p	LOC_Os03g15770.3	145	-20.59	145	-20.59	746228	24	2664	1738
miR1874-3p	LOC_Os03g15810.1	154	-20.25	154	-20.25	746235	24	3009	958
miR1874-3p	LOC_Os03g15930.1	162	-22.94	162	-22.94	746255	24	954	869
miR1874-3p	LOC_Os03g15930.2	162	-22.94	162	-22.94	746256	24	946	780
miR1874-3p	LOC_Os03g15930.3	162	-22.94	162	-22.94	746257	24	1174	814
miR1874-3p	LOC_Os03g16000.1	151	-21.76	151	-21.76	746265	24	2133	1610
miR1874-3p	LOC_Os03g16140.1	149	-21.5	149	-21.5	746294	24	2008	1796
miR1874-3p	LOC_Os03g16140.2	149	-21.5	149	-21.5	746295	24	1996	1796
miR1874-3p	LOC_Os03g16140.3	149	-21.5	149	-21.5	746296	24	2223	1796
miR1874-3p	LOC_Os03g16140.4	149	-21.5	149	-21.5	746297	24	2027	1815
miR1874-3p	LOC_Os03g16530.1	156	-20.7	156	-20.7	746344	24	6336	5819
miR1874-3p	LOC_Os03g16670.1	159	-21.37	159	-21.37	746359	24	1377	1051
miR1874-3p	LOC_Os03g16740.1	164	-23.86	164	-23.86	746369	24	2546	1099
miR1874-3p	LOC_Os03g16740.2	164	-23.86	164	-23.86	746370	24	2538	1099
miR1874-3p	LOC_Os03g16900.1	159	-24.17	159	-24.17	746393	24	1715	1579
miR1874-3p	LOC_Os03g17164.1	177	-27.58	177	-27.58	746428	24	2448	256
miR1874-3p	LOC_Os03g17300.1	174	-24.23	174	-24.23	746444	24	3983	120
miR1874-3p	LOC_Os03g17300.2	174	-24.23	174	-24.23	746445	24	4115	120
miR1874-3p	LOC_Os03g17300.3	174	-24.23	174	-24.23	746446	24	4023	120
miR1874-3p	LOC_Os03g17300.4	174	-24.23	174	-24.23	746447	24	4026	120
miR1874-3p	LOC_Os03g17310.1	333	-46.86	174	-24.23	746448	24	6948	120 5427
miR1874-3p	LOC_Os03g17340.1	142	-20.62	142	-20.62	746450	24	2611	1675
miR1874-3p	LOC_Os03g17730.1	159	-20.41	159	-20.41	746495	24	2074	1589
miR1874-3p	LOC_Os03g17910.1	143	-22.51	143	-22.51	746514	24	1133	384
miR1874-3p	LOC_Os03g18190.1	149	-21.75	149	-21.75	746553	24	3099	958
miR1874-3p	LOC_Os03g18210.1	157	-26.89	157	-26.89	746556	24	2540	1994
miR1874-3p	LOC_Os03g18264.1	159	-20.72	159	-20.72	746562	24	4154	1316
miR1874-3p	LOC_Os03g18540.2	161	-21.96	161	-21.96	746595	24	2896	1056

miR1874-3p	LOC_Os03g19275.1	158	-25.06	158	-25.06	746678	24	1077	1025
miR1874-3p	LOC_Os03g19280.1	143	-20.61	143	-20.61	746679	24	1871	404
miR1874-3p	LOC_Os03g19300.1	164	-21.09	164	-21.09	746682	24	1891	1308
miR1874-3p	LOC_Os03g19370.1	167	-28.49	167	-28.49	746688	24	1406	308
miR1874-3p	LOC_Os03g19380.1	156	-22.32	156	-22.32	746690	24	1005	469
miR1874-3p	LOC_Os03g20949.2	149	-21.11	149	-21.11	746884	24	2688	662
miR1874-3p	LOC_Os03g21100.1	154	-22.49	154	-22.49	746901	24	2424	875
miR1874-3p	LOC_Os03g21210.1	160	-23.08	160	-23.08	746913	24	2325	1072
miR1874-3p	LOC_Os03g21360.1	147	-23.72	147	-23.72	746929	24	1113	368
miR1874-3p	LOC_Os03g21800.1	163	-20.47	163	-20.47	746986	24	1694	1329
miR1874-3p	LOC_Os03g22030.1	149	-22.49	149	-22.49	747014	24	543	30
miR1874-3p	LOC_Os03g22040.1	162	-23.35	162	-23.35	747015	24	2101	82
miR1874-3p	LOC_Os03g22090.1	155	-21.06	155	-21.06	747024	24	2823	2660
miR1874-3p	LOC_Os03g22100.1	313	-41.34	162	-20.87	747025	24	3228	3206 2579
miR1874-3p	LOC_Os03g22220.1	149	-23.3	149	-23.3	747040	24	1140	1076
miR1874-3p	LOC_Os03g22270.1	155	-20.41	155	-20.41	747045	24	1122	216
miR1874-3p	LOC_Os03g22270.2	155	-20.41	155	-20.41	747046	24	1494	216
miR1874-3p	LOC_Os03g22270.3	155	-20.41	155	-20.41	747047	24	2330	216
miR1874-3p	LOC_Os03g22600.1	157	-22.02	157	-22.02	747090	24	2393	80
miR1874-3p	LOC_Os03g22634.1	162	-23.57	162	-23.57	747094	24	2506	2331
miR1874-3p	LOC_Os03g23060.1	154	-22.42	154	-22.42	747150	24	987	782
miR1874-3p	LOC_Os03g23070.1	154	-22.42	154	-22.42	747151	24	2973	2945
miR1874-3p	LOC_Os03g23080.1	162	-20.65	162	-20.65	747152	24	1839	2
miR1874-3p	LOC_Os03g23220.1	154	-22.07	154	-22.07	747165	24	5415	5393
miR1874-3p	LOC_Os03g23760.1	156	-25.26	156	-25.26	747168	24	3822	2028
miR1874-3p	LOC_Os03g23800.1	154	-22.07	154	-22.07	747171	24	5247	5225
miR1874-3p	LOC_Os03g23830.1	154	-22.07	154	-22.07	747173	24	4605	4583
miR1874-3p	LOC_Os03g23840.1	154	-22.07	154	-22.07	747174	24	5415	5393
miR1874-3p	LOC_Os03g23850.1	154	-22.07	154	-22.07	747175	24	876	854
miR1874-3p	LOC_Os03g23860.1	156	-25.26	156	-25.26	747176	24	5295	1962
miR1874-3p	LOC_Os03g24180.1	158	-21.3	158	-21.3	747206	24	3285	1919
miR1874-3p	LOC_Os03g24190.1	149	-22.12	149	-22.12	747208	24	5118	575

miR1874-3p	LOC_Os03g24220.1	168	-21.95	168	-21.95	747211	24	3695	3483
miR1874-3p	LOC_Os03g24220.2	168	-21.95	168	-21.95	747212	24	3674	3462
miR1874-3p	LOC_Os03g24450.1	155	-21.24	155	-21.24	747233	24	2385	428
miR1874-3p	LOC_Os03g24550.1	155	-21.42	155	-21.42	747244	24	285	45
miR1874-3p	LOC_Os03g24960.1	166	-23.02	166	-23.02	747286	24	1200	1116
miR1874-3p	LOC_Os03g25070.1	161	-24.23	161	-24.23	747298	24	2897	159
miR1874-3p	LOC_Os03g25470.1	166	-22.23	166	-22.23	747349	24	552	509
miR1874-3p	LOC_Os03g25960.1	159	-24.9	159	-24.9	747397	24	1186	328
miR1874-3p	LOC_Os03g25980.1	149	-24.57	149	-24.57	747400	24	1795	1540
miR1874-3p	LOC_Os03g25980.2	149	-24.57	149	-24.57	747401	24	1829	1540
miR1874-3p	LOC_Os03g26020.1	152	-26.31	152	-26.31	747406	24	2691	574
miR1874-3p	LOC_Os03g26260.1	150	-22.98	150	-22.98	747426	24	2484	21
miR1874-3p	LOC_Os03g26310.1	142	-21.3	142	-21.3	747430	24	864	491
miR1874-3p	LOC_Os03g26760.1	158	-21.02	158	-21.02	747470	24	5469	4720
miR1874-3p	LOC_Os03g26784.1	155	-22.31	155	-22.31	747472	24	1266	1147
miR1874-3p	LOC_Os03g26870.1	163	-26.31	163	-26.31	747481	24	2055	200
miR1874-3p	LOC_Os03g26870.2	163	-26.31	163	-26.31	747482	24	3358	200
miR1874-3p	LOC_Os03g26890.1	164	-26.24	164	-26.24	747484	24	4119	2027
miR1874-3p	LOC_Os03g27340.1	154	-20.49	154	-20.49	747538	24	2371	1327
miR1874-3p	LOC_Os03g27460.1	156	-20.46	156	-20.46	747555	24	1803	762
miR1874-3p	LOC_Os03g27460.2	156	-20.46	156	-20.46	747556	24	2970	1929
miR1874-3p	LOC_Os03g27460.3	156	-20.46	156	-20.46	747557	24	1828	787
miR1874-3p	LOC_Os03g27890.1	157	-25.18	157	-25.18	747600	24	447	303
miR1874-3p	LOC_Os03g28010.1	149	-20.73	149	-20.73	747614	24	5928	2873
miR1874-3p	LOC_Os03g28030.1	156	-21.01	156	-21.01	747616	24	468	203
miR1874-3p	LOC_Os03g28090.1	165	-25.84	165	-25.84	747622	24	2246	193
miR1874-3p	LOC_Os03g28170.1	174	-29.56	174	-29.56	747630	24	817	535
miR1874-3p	LOC_Os03g28389.1	154	-21.24	154	-21.24	747654	24	818	319
miR1874-3p	LOC_Os03g28400.1	154	-21.24	154	-21.24	747655	24	1823	319
miR1874-3p	LOC_Os03g28950.1	156	-25.7	156	-25.7	747663	24	1338	577
miR1874-3p	LOC_Os03g28969.1	152	-20.71	152	-20.71	747665	24	387	137
miR1874-3p	LOC_Os03g29260.2	157	-22.01	157	-22.01	747702	24	1717	91

miR1874-3p	LOC_Os03g29310.1	161	-27.28	161	-27.28	747706	24	2580	2415
miR1874-3p	LOC_Os03g29520.1	305	-42.35	156	-21.65	747726	24	5769	5255 4083
miR1874-3p	LOC_Os03g29900.1	154	-23.58	154	-23.58	747756	24	1995	1364
miR1874-3p	LOC_Os03g30050.1	153	-23.68	153	-23.68	747774	24	774	565
miR1874-3p	LOC_Os03g30530.1	155	-20.83	155	-20.83	747824	24	3312	1266
miR1874-3p	LOC_Os03g30530.2	155	-20.83	155	-20.83	747825	24	3309	1263
miR1874-3p	LOC_Os03g30530.3	155	-20.83	155	-20.83	747826	24	2320	1263
miR1874-3p	LOC_Os03g30630.1	156	-27.84	156	-27.84	747835	24	686	486
miR1874-3p	LOC_Os03g31000.1	160	-24.73	160	-24.73	747874	24	2079	1996
miR1874-3p	LOC_Os03g31460.1	159	-26.31	159	-26.31	747928	24	918	855
miR1874-3p	LOC_Os03g31880.1	159	-21.35	159	-21.35	747966	24	2475	1952
miR1874-3p	LOC_Os03g32526.1	146	-20.15	146	-20.15	748024	24	2820	395
miR1874-3p	LOC_Os03g32620.1	144	-20.3	144	-20.3	748034	24	4010	3057
miR1874-3p	LOC_Os03g32650.1	154	-23.58	154	-23.58	748038	24	4593	4220
miR1874-3p	LOC_Os03g32910.1	165	-21.2	165	-21.2	748062	24	807	224
miR1874-3p	LOC_Os03g33074.1	162	-23.7	162	-23.7	748080	24	180	115
miR1874-3p	LOC_Os03g33200.1	169	-21.7	169	-21.7	748092	24	711	147
miR1874-3p	LOC_Os03g33250.1	160	-21.62	160	-21.62	748096	24	2520	1448
miR1874-3p	LOC_Os03g33590.1	146	-20.07	146	-20.07	748127	24	1839	1153
miR1874-3p	LOC_Os03g33590.2	146	-20.07	146	-20.07	748128	24	1815	1129
miR1874-3p	LOC_Os03g33837.1	156	-20.97	156	-20.97	748152	24	576	59
miR1874-3p	LOC_Os03g35400.1	159	-21.53	159	-21.53	748213	24	1653	558
miR1874-3p	LOC_Os03g35440.1	144	-22.3	144	-22.3	748216	24	796	369
miR1874-3p	LOC_Os03g35830.1	147	-23.72	147	-23.72	748244	24	4428	3854
miR1874-3p	LOC_Os03g36000.1	312	-41.73	161	-21.61	748262	24	3555	2498 2823
miR1874-3p	LOC_Os03g36150.1	154	-21.2	154	-21.2	748275	24	846	824
miR1874-3p	LOC_Os03g36220.1	297	-46.87	156	-26.54	748280	24	3810	2500 2903
miR1874-3p	LOC_Os03g37100.1	162	-28.37	162	-28.37	748361	24	1745	21
miR1874-3p	LOC_Os03g38650.1	152	-22.58	152	-22.58	748510	24	495	309
miR1874-3p	LOC_Os03g38970.1	166	-25.83	166	-25.83	748548	24	4644	450
miR1874-3p	LOC_Os03g39020.1	467	-61.35	165	-20.73	748556	24	3842	1063 2188 2100
miR1874-3p	LOC_Os03g39210.1	162	-20.65	162	-20.65	748570	24	1956	1753

miR1874-3p	LOC_Os03g39432.1	164	-20.63	164	-20.63	748591	24	2028	200
miR1874-3p	LOC_Os03g39500.1	163	-24.51	163	-24.51	748596	24	3930	2267
miR1874-3p	LOC_Os03g39520.1	149	-23.49	149	-23.49	748598	24	1749	1206
miR1874-3p	LOC_Os03g40580.1	161	-21.79	161	-21.79	748699	24	2178	770
miR1874-3p	LOC_Os03g41510.1	167	-27.06	167	-27.06	748773	24	1670	1512
miR1874-3p	LOC_Os03g41510.2	167	-27.06	167	-27.06	748774	24	1690	1532
miR1874-3p	LOC_Os03g41510.3	167	-27.06	167	-27.06	748775	24	1698	1555
miR1874-3p	LOC_Os03g41510.4	167	-27.06	167	-27.06	748776	24	1641	1483
miR1874-3p	LOC_Os03g41510.5	167	-27.06	167	-27.06	748777	24	1683	1525
miR1874-3p	LOC_Os03g41510.6	167	-27.06	167	-27.06	748778	24	1646	1503
miR1874-3p	LOC_Os03g41510.7	167	-27.06	167	-27.06	748779	24	1757	1614
miR1874-3p	LOC_Os03g41510.8	167	-27.06	167	-27.06	748780	24	1991	1833
miR1874-3p	LOC_Os03g41780.1	140	-20.5	140	-20.5	748803	24	3054	1291
miR1874-3p	LOC_Os03g41980.1	148	-21.28	148	-21.28	748821	24	5637	2346
miR1874-3p	LOC_Os03g42530.1	163	-23.31	163	-23.31	748877	24	2148	1895
miR1874-3p	LOC_Os03g42730.1	156	-20.86	156	-20.86	748894	24	1245	1040
miR1874-3p	LOC_Os03g42930.1	148	-20.99	148	-20.99	748920	24	978	163
miR1874-3p	LOC_Os03g43010.1	147	-22.37	147	-22.37	748927	24	1979	1916
miR1874-3p	LOC_Os03g43220.1	168	-26.41	168	-26.41	748949	24	5073	1652
miR1874-3p	LOC_Os03g43240.1	154	-22.42	154	-22.42	748952	24	387	359
miR1874-3p	LOC_Os03g43350.1	159	-22.56	159	-22.56	748960	24	1360	552
miR1874-3p	LOC_Os03g43430.1	153	-23.69	153	-23.69	748970	24	824	170
miR1874-3p	LOC_Os03g43490.1	161	-25.42	161	-25.42	748976	24	558	429
miR1874-3p	LOC_Os03g43500.1	146	-20.82	146	-20.82	748977	24	333	272
miR1874-3p	LOC_Os03g43720.2	151	-22.45	151	-22.45	748999	24	2405	158
miR1874-3p	LOC_Os03g44010.1	154	-20.99	154	-20.99	749037	24	1538	72
miR1874-3p	LOC_Os03g44010.2	154	-20.99	154	-20.99	749038	24	1610	72
miR1874-3p	LOC_Os03g44370.1	155	-20.27	155	-20.27	749074	24	2607	2395
miR1874-3p	LOC_Os03g44500.1	157	-20.21	157	-20.21	749088	24	3743	804
miR1874-3p	LOC_Os03g44540.1	145	-20.12	145	-20.12	749093	24	2188	2115
miR1874-3p	LOC_Os03g44930.1	149	-21.09	149	-21.09	749146	24	636	213
miR1874-3p	LOC_Os03g45000.1	162	-23.31	162	-23.31	749155	24	969	433

miR1874-3p	LOC_Os03g45140.1	320	-40.67	160	-20.49	749164	24	4536	2096 4291
miR1874-3p	LOC_Os03g45160.1	164	-23.66	164	-23.66	749166	24	219	4
miR1874-3p	LOC_Os03g45194.1	143	-21.01	143	-21.01	749170	24	1872	241
miR1874-3p	LOC_Os03g45900.1	154	-24.13	154	-24.13	749235	24	402	21
miR1874-3p	LOC_Os03g46170.1	144	-22.57	144	-22.57	749265	24	3885	3510
miR1874-3p	LOC_Os03g46510.1	147	-25.8	147	-25.8	749306	24	2343	1477
miR1874-3p	LOC_Os03g46530.1	147	-25.8	147	-25.8	749307	24	1608	1416
miR1874-3p	LOC_Os03g46640.1	161	-22.26	161	-22.26	749317	24	1033	269
miR1874-3p	LOC_Os03g46770.1	153	-23.48	153	-23.48	749332	24	991	699
miR1874-3p	LOC_Os03g46870.1	154	-23.4	154	-23.4	749342	24	999	86
miR1874-3p	LOC_Os03g47410.1	176	-29.95	176	-29.95	749393	24	4020	2808
miR1874-3p	LOC_Os03g47740.1	164	-21.08	164	-21.08	749433	24	2894	1851
miR1874-3p	LOC_Os03g47740.2	164	-21.08	164	-21.08	749434	24	2989	1946
miR1874-3p	LOC_Os03g47740.3	164	-21.08	164	-21.08	749435	24	2652	1946
miR1874-3p	LOC_Os03g47890.1	160	-20.49	160	-20.49	749454	24	1905	895
miR1874-3p	LOC_Os03g48320.1	156	-22.62	156	-22.62	749504	24	3540	2587
miR1874-3p	LOC_Os03g48370.1	168	-23.09	168	-23.09	749510	24	2865	2323
miR1874-3p	LOC_Os03g48810.1	143	-22.94	143	-22.94	749561	24	1951	871
miR1874-3p	LOC_Os03g48990.1	147	-23.72	147	-23.72	749588	24	5313	4775
miR1874-3p	LOC_Os03g49420.1	152	-23.47	152	-23.47	749635	24	3862	1546
miR1874-3p	LOC_Os03g49420.2	152	-23.47	152	-23.47	749636	24	3818	1507
miR1874-3p	LOC_Os03g49500.1	162	-22.56	162	-22.56	749650	24	2538	2156
miR1874-3p	LOC_Os03g49500.2	162	-22.56	162	-22.56	749651	24	2304	2156
miR1874-3p	LOC_Os03g49540.1	154	-23.58	154	-23.58	749657	24	2190	2051
miR1874-3p	LOC_Os03g49960.1	153	-23.4	153	-23.4	749710	24	2443	924
miR1874-3p	LOC_Os03g49960.2	153	-23.4	153	-23.4	749711	24	2459	940
miR1874-3p	LOC_Os03g49960.5	153	-23.4	153	-23.4	749712	24	2438	919
miR1874-3p	LOC_Os03g49980.1	149	-22.12	149	-22.12	749714	24	5262	305
miR1874-3p	LOC_Os03g50100.1	157	-22.73	157	-22.73	749728	24	3451	2234
miR1874-3p	LOC_Os03g50364.1	163	-20.4	163	-20.4	749757	24	1371	821
miR1874-3p	LOC_Os03g50390.1	154	-20.09	154	-20.09	749759	24	4833	2240
miR1874-3p	LOC_Os03g50390.2	154	-20.09	154	-20.09	749760	24	3323	2216

miR1874-3p	LOC_Os03g50480.1	150	-25.09	150	-25.09	749769	24	2222	2185
miR1874-3p	LOC_Os03g50480.2	150	-25.09	150	-25.09	749770	24	2066	2029
miR1874-3p	LOC_Os03g50490.1	168	-23.83	168	-23.83	749771	24	1714	1273
miR1874-3p	LOC_Os03g51380.1	159	-20.86	159	-20.86	749866	24	1930	856
miR1874-3p	LOC_Os03g51690.1	155	-22.12	155	-22.12	749904	24	2604	280
miR1874-3p	LOC_Os03g51690.2	155	-22.12	155	-22.12	749905	24	2522	280
miR1874-3p	LOC_Os03g51690.3	155	-22.12	155	-22.12	749906	24	1272	280
miR1874-3p	LOC_Os03g51970.1	162	-23.31	162	-23.31	749935	24	1811	1020
miR1874-3p	LOC_Os03g52110.1	156	-21.34	156	-21.34	749952	24	3756	668
miR1874-3p	LOC_Os03g52239.1	160	-21.9	160	-21.9	749964	24	3575	2971
miR1874-3p	LOC_Os03g52239.2	160	-21.9	160	-21.9	749965	24	1695	1091
miR1874-3p	LOC_Os03g52239.3	160	-21.9	160	-21.9	749966	24	2114	1510
miR1874-3p	LOC_Os03g52360.1	151	-23.03	151	-23.03	749980	24	739	174
miR1874-3p	LOC_Os03g52794.1	326	-49.28	166	-28.79	750032	24	11361	1101 2770
miR1874-3p	LOC_Os03g53390.1	146	-20.37	146	-20.37	750101	24	1074	692
miR1874-3p	LOC_Os03g53820.1	156	-20.06	156	-20.06	750158	24	3165	180
miR1874-3p	LOC_Os03g54150.1	149	-20.11	149	-20.11	750202	24	3624	3143
miR1874-3p	LOC_Os03g54150.2	149	-20.11	149	-20.11	750203	24	3391	2910
miR1874-3p	LOC_Os03g54160.1	149	-20.11	149	-20.11	750204	24	2007	941
miR1874-3p	LOC_Os03g54160.2	149	-20.11	149	-20.11	750205	24	1986	941
miR1874-3p	LOC_Os03g54930.1	156	-22.88	156	-22.88	750236	24	1818	161
miR1874-3p	LOC_Os03g55020.1	148	-25.81	148	-25.81	750246	24	1487	1036
miR1874-3p	LOC_Os03g55034.1	148	-25.81	148	-25.81	750249	24	691	434
miR1874-3p	LOC_Os03g55610.1	159	-23.14	159	-23.14	750316	24	1687	89
miR1874-3p	LOC_Os03g56160.1	155	-21.94	155	-21.94	750387	24	2259	1857
miR1874-3p	LOC_Os03g56270.1	174	-31.46	174	-31.46	750398	24	3741	1800
miR1874-3p	LOC_Os03g56270.2	174	-31.46	174	-31.46	750399	24	3306	1794
miR1874-3p	LOC_Os03g56270.3	174	-31.46	174	-31.46	750400	24	3553	1794
miR1874-3p	LOC_Os03g56520.1	151	-21.96	151	-21.96	750434	24	510	35
miR1874-3p	LOC_Os03g56714.1	157	-23.99	157	-23.99	750458	24	744	307
miR1874-3p	LOC_Os03g56840.1	152	-21.24	152	-21.24	750480	24	1748	875
miR1874-3p	LOC_Os03g56840.2	152	-21.24	152	-21.24	750481	24	1828	955

miR1874-3p	LOC_Os03g56960.1	154	-21.46	154	-21.46	750502	24	2830	994
miR1874-3p	LOC_Os03g57160.1	329	-48.04	169	-25.61	750530	24	3376	515 1084
miR1874-3p	LOC_Os03g57330.1	151	-20.16	151	-20.16	750553	24	2063	887
miR1874-3p	LOC_Os03g57330.3	151	-20.16	151	-20.16	750554	24	1973	923
miR1874-3p	LOC_Os03g57500.1	155	-20.78	155	-20.78	750572	24	1974	1661
miR1874-3p	LOC_Os03g57880.1	154	-20.15	154	-20.15	750615	24	2262	22
miR1874-3p	LOC_Os03g57880.2	154	-20.15	154	-20.15	750616	24	2346	22
miR1874-3p	LOC_Os03g57880.3	154	-20.15	154	-20.15	750617	24	2148	22
miR1874-3p	LOC_Os03g58060.1	309	-41.12	162	-20.77	750644	24	3697	3373 2089
miR1874-3p	LOC_Os03g58060.2	309	-41.12	162	-20.77	750645	24	4048	3724 1999
miR1874-3p	LOC_Os03g58060.3	309	-41.12	162	-20.77	750646	24	3684	3360 1999
miR1874-3p	LOC_Os03g58090.1	168	-25.11	168	-25.11	750649	24	1399	585
miR1874-3p	LOC_Os03g58260.2	153	-22.59	153	-22.59	750669	24	1815	528
miR1874-3p	LOC_Os03g58270.1	152	-22.2	152	-22.2	750670	24	3573	1105
miR1874-3p	LOC_Os03g59060.1	158	-22.22	158	-22.22	750754	24	1650	670
miR1874-3p	LOC_Os03g59060.2	158	-22.22	158	-22.22	750755	24	1509	670
miR1874-3p	LOC_Os03g59560.1	159	-24.5	159	-24.5	750806	24	300	257
miR1874-3p	LOC_Os03g59874.1	169	-24.41	169	-24.41	750844	24	234	8
miR1874-3p	LOC_Os03g60140.1	153	-21.91	153	-21.91	750870	24	1226	293
miR1874-3p	LOC_Os03g60430.1	153	-20.87	153	-20.87	750898	24	2123	250
miR1874-3p	LOC_Os03g60430.2	153	-20.87	153	-20.87	750899	24	2135	250
miR1874-3p	LOC_Os03g61140.1	165	-21.76	165	-21.76	750983	24	1236	63
miR1874-3p	LOC_Os03g61140.2	165	-21.76	165	-21.76	750984	24	1353	63
miR1874-3p	LOC_Os03g61150.3	159	-21.87	159	-21.87	750987	24	1224	468
miR1874-3p	LOC_Os03g61730.1	155	-24.64	155	-24.64	751059	24	2723	527
miR1874-3p	LOC_Os03g61920.1	149	-20.04	149	-20.04	751082	24	1777	745
miR1874-3p	LOC_Os03g62090.1	151	-21.28	151	-21.28	751113	24	4365	2956
miR1874-3p	LOC_Os03g62110.2	161	-24.77	161	-24.77	751115	24	2232	1581
miR1874-3p	LOC_Os03g62110.3	161	-24.77	161	-24.77	751116	24	2157	1506
miR1874-3p	LOC_Os03g62290.1	302	-47.23	151	-23.77	751140	24	772	527 543
miR1874-3p	LOC_Os03g62314.1	147	-20.13	147	-20.13	751142	24	2010	1276
miR1874-3p	LOC_Os03g62379.1	165	-21.16	165	-21.16	751148	24	657	550

miR1874-3p	LOC_Os03g62388.1	155	-21.76	155	-21.76	751149	24	1995	410
miR1874-3p	LOC_Os03g62440.1	159	-23.62	159	-23.62	751155	24	513	50
miR1874-3p	LOC_Os03g62460.1	169	-28.72	169	-28.72	751157	24	2631	2196
miR1874-3p	LOC_Os03g62522.1	156	-23.54	156	-23.54	751164	24	1590	303
miR1874-3p	LOC_Os03g62640.2	160	-29.69	160	-29.69	751184	24	1618	1442
miR1874-3p	LOC_Os03g62640.3	160	-29.69	160	-29.69	751185	24	2506	2330
miR1874-3p	LOC_Os03g62640.4	160	-29.69	160	-29.69	751186	24	2282	2106
miR1874-3p	LOC_Os03g62640.5	160	-29.69	160	-29.69	751187	24	2384	2208
miR1874-3p	LOC_Os03g62950.1	157	-22.86	157	-22.86	751239	24	1183	448
miR1874-3p	LOC_Os03g63040.1	148	-20.93	148	-20.93	751250	24	3297	2667
miR1874-3p	LOC_Os03g63150.1	154	-26.44	154	-26.44	751261	24	4553	2955
miR1874-3p	LOC_Os03g63150.2	154	-26.44	154	-26.44	751262	24	4643	3045
miR1874-3p	LOC_Os03g63200.1	152	-24.31	152	-24.31	751266	24	4380	3023
miR1874-3p	LOC_Os03g63240.1	151	-22.35	151	-22.35	751270	24	2238	871
miR1874-3p	LOC_Os03g63410.1	163	-21.86	163	-21.86	751290	24	1918	1352
miR1874-3p	LOC_Os03g63410.2	163	-21.86	163	-21.86	751291	24	2318	1752
miR1874-3p	LOC_Os03g63510.1	151	-26.19	151	-26.19	751303	24	2315	1770
miR1874-3p	LOC_Os03g63660.1	157	-21.58	157	-21.58	751320	24	2085	1261
miR1874-3p	LOC_Os03g63720.1	169	-24.02	169	-24.02	751328	24	1205	267
miR1874-3p	LOC_Os03g63880.1	165	-22.98	165	-22.98	751351	24	621	25
miR1874-3p	LOC_Os03g63910.1	162	-20.08	162	-20.08	751354	24	2793	5
miR1874-3p	LOC_Os03g63920.1	152	-21.76	152	-21.76	751355	24	2722	275
miR1874-3p	LOC_Os03g64230.1	156	-21.01	156	-21.01	751392	24	2422	1903
miR1874-3p	LOC_Os03g64230.2	156	-21.01	156	-21.01	751393	24	2082	1882
miR1874-3p	LOC_Os03g64300.1	151	-21.87	151	-21.87	751401	24	3276	2229
miR1874-3p	LOC_Os03g64350.1	143	-21.55	143	-21.55	751406	24	1696	59
miR1874-3p	LOC_Os03g64350.2	143	-21.55	143	-21.55	751407	24	1690	58
miR1874-3p	LOC_Os04g01190.1	154	-20.7	154	-20.7	751441	24	1983	209
miR1874-3p	LOC_Os04g01380.1	156	-21.42	156	-21.42	751466	24	1614	623
miR1874-3p	LOC_Os04g01874.1	152	-21.84	152	-21.84	751518	24	2317	1458
miR1874-3p	LOC_Os04g02000.2	152	-20.13	152	-20.13	751531	24	2552	1267
miR1874-3p	LOC_Os04g02160.1	159	-22.46	159	-22.46	751554	24	432	33

miR1874-3p	LOC_Os04g02420.1	160	-20.18	160	-20.18	751578	24	2778	1322
miR1874-3p	LOC_Os04g02434.1	160	-20.49	160	-20.49	751579	24	1355	790
miR1874-3p	LOC_Os04g02450.1	147	-20.57	147	-20.57	751580	24	2283	654
miR1874-3p	LOC_Os04g02520.1	146	-23.28	146	-23.28	751591	24	3474	2715
miR1874-3p	LOC_Os04g02730.1	155	-24.93	155	-24.93	751614	24	2506	504
miR1874-3p	LOC_Os04g02840.1	154	-21.74	154	-21.74	751624	24	927	373
miR1874-3p	LOC_Os04g02900.1	162	-26.04	162	-26.04	751634	24	2173	34
miR1874-3p	LOC_Os04g03630.1	156	-21.34	156	-21.34	751701	24	4899	998
miR1874-3p	LOC_Os04g04060.1	147	-22.26	147	-22.26	751745	24	2995	1207
miR1874-3p	LOC_Os04g04160.1	158	-21.02	158	-21.02	751753	24	5001	4423
miR1874-3p	LOC_Os04g04540.1	150	-21.2	150	-21.2	751791	24	783	756
miR1874-3p	LOC_Os04g04560.1	147	-23.72	147	-23.72	751793	24	792	365
miR1874-3p	LOC_Os04g04670.1	151	-22.62	151	-22.62	751802	24	1260	85
miR1874-3p	LOC_Os04g04750.1	150	-21.35	150	-21.35	751810	24	1017	804
miR1874-3p	LOC_Os04g04990.1	144	-23.67	144	-23.67	751831	24	2529	1837
miR1874-3p	LOC_Os04g05050.1	312	-43.82	157	-23.02	751837	24	2066	1338 704
miR1874-3p	LOC_Os04g05050.2	312	-43.82	157	-23.02	751838	24	2153	1425 791
miR1874-3p	LOC_Os04g05120.1	153	-20.26	153	-20.26	751844	24	760	475
miR1874-3p	LOC_Os04g05260.1	154	-22.44	154	-22.44	751858	24	597	575
miR1874-3p	LOC_Os04g05330.1	145	-22.05	145	-22.05	751864	24	1935	133
miR1874-3p	LOC_Os04g05470.1	145	-22.05	145	-22.05	751876	24	2076	133
miR1874-3p	LOC_Os04g05820.1	169	-23.14	169	-23.14	751908	24	2220	178
miR1874-3p	LOC_Os04g05880.1	165	-20.52	165	-20.52	751914	24	6381	4926
miR1874-3p	LOC_Os04g06000.1	152	-21.28	152	-21.28	751926	24	3843	2791
miR1874-3p	LOC_Os04g06130.1	153	-20.94	153	-20.94	751937	24	4980	4505
miR1874-3p	LOC_Os04g06200.1	161	-20.93	161	-20.93	751943	24	1338	410
miR1874-3p	LOC_Os04g06230.1	150	-21.18	150	-21.18	751946	24	2322	2283
miR1874-3p	LOC_Os04g06244.1	164	-20.94	164	-20.94	751947	24	1770	744
miR1874-3p	LOC_Os04g06280.1	169	-28.72	169	-28.72	751950	24	2673	333
miR1874-3p	LOC_Os04g06500.1	163	-28.2	163	-28.2	751977	24	2330	1035
miR1874-3p	LOC_Os04g06700.1	163	-28.2	163	-28.2	751992	24	2310	1029
miR1874-3p	LOC_Os04g06850.1	153	-21.6	153	-21.6	752005	24	2686	1572

miR1874-3p	LOC_Os04g06920.1	144	-20.5	144	-20.5	752011	24	1239	936
miR1874-3p	LOC_Os04g06960.1	343	-54.84	176	-29.95	752014	24	2682	1890 1092
miR1874-3p	LOC_Os04g07060.1	154	-22.07	154	-22.07	752018	24	5295	5273
miR1874-3p	LOC_Os04g07320.1	151	-24.57	151	-24.57	752047	24	4995	4616
miR1874-3p	LOC_Os04g07830.1	156	-21.34	156	-21.34	752099	24	1221	575
miR1874-3p	LOC_Os04g08210.1	176	-33.4	176	-33.4	752134	24	4218	2241
miR1874-3p	LOC_Os04g08460.1	148	-21.26	148	-21.26	752161	24	1575	1357
miR1874-3p	LOC_Os04g08540.1	158	-21.02	158	-21.02	752168	24	5340	4609
miR1874-3p	LOC_Os04g08610.1	156	-21.83	156	-21.83	752174	24	603	446
miR1874-3p	LOC_Os04g08816.1	150	-21.18	150	-21.18	752204	24	3399	3309
miR1874-3p	LOC_Os04g09270.1	154	-23.05	154	-23.05	752213	24	2175	281
miR1874-3p	LOC_Os04g09330.1	169	-28.72	169	-28.72	752217	24	2226	1335
miR1874-3p	LOC_Os04g09810.1	156	-23.42	156	-23.42	752264	24	2337	1061
miR1874-3p	LOC_Os04g10070.1	161	-22.06	161	-22.06	752289	24	2577	1068
miR1874-3p	LOC_Os04g10100.1	154	-22.07	154	-22.07	752292	24	5415	5393
miR1874-3p	LOC_Os04g10700.1	156	-22	156	-22	752351	24	3042	1020
miR1874-3p	LOC_Os04g10760.1	174	-21.25	174	-21.25	752360	24	972	105
miR1874-3p	LOC_Os04g10840.1	154	-23.05	154	-23.05	752366	24	2466	281
miR1874-3p	LOC_Os04g11340.1	176	-33.4	176	-33.4	752414	24	1494	354
miR1874-3p	LOC_Os04g11950.1	169	-28.72	169	-28.72	752473	24	2472	2037
miR1874-3p	LOC_Os04g12310.1	151	-23.72	151	-23.72	752511	24	4878	4301
miR1874-3p	LOC_Os04g12320.1	151	-24.57	151	-24.57	752512	24	4290	3830
miR1874-3p	LOC_Os04g12520.1	163	-23.56	163	-23.56	752530	24	4728	921
miR1874-3p	LOC_Os04g12580.1	163	-22.81	163	-22.81	752536	24	2346	1060
miR1874-3p	LOC_Os04g12600.1	153	-20.82	153	-20.82	752538	24	2415	1247
miR1874-3p	LOC_Os04g13020.1	154	-20.7	154	-20.7	752570	24	2361	281
miR1874-3p	LOC_Os04g13050.1	160	-27.13	160	-27.13	752573	24	1242	206
miR1874-3p	LOC_Os04g13160.1	164	-22.68	164	-22.68	752584	24	2013	507
miR1874-3p	LOC_Os04g13510.1	160	-23.74	160	-23.74	752615	24	4560	1022
miR1874-3p	LOC_Os04g13640.1	162	-23.84	162	-23.84	752628	24	975	160
miR1874-3p	LOC_Os04g13780.1	150	-22.69	150	-22.69	752642	24	2694	701
miR1874-3p	LOC_Os04g13990.1	158	-20.31	158	-20.31	752663	24	4391	3874

miR1874-3p	LOC_Os04g14060.1	158	-23.76	158	-23.76	752669	24	726	700
miR1874-3p	LOC_Os04g14180.1	164	-23.37	164	-23.37	752681	24	621	332
miR1874-3p	LOC_Os04g14560.1	162	-23.84	162	-23.84	752723	24	5973	5506
miR1874-3p	LOC_Os04g14654.1	152	-21.17	152	-21.17	752732	24	2328	1419
miR1874-3p	LOC_Os04g14654.2	152	-21.17	152	-21.17	752733	24	2338	1419
miR1874-3p	LOC_Os04g14654.3	152	-21.17	152	-21.17	752734	24	2240	1331
miR1874-3p	LOC_Os04g14760.1	143	-20.67	143	-20.67	752744	24	943	862
miR1874-3p	LOC_Os04g14840.1	162	-20.65	162	-20.65	752750	24	2133	1465
miR1874-3p	LOC_Os04g14890.1	149	-21.88	149	-21.88	752755	24	4494	4046
miR1874-3p	LOC_Os04g15570.1	172	-28.71	172	-28.71	752774	24	4182	2205
miR1874-3p	LOC_Os04g15660.1	168	-23.22	168	-23.22	752783	24	2676	1532
miR1874-3p	LOC_Os04g16040.1	173	-28.72	173	-28.72	752816	24	564	156
miR1874-3p	LOC_Os04g16150.1	149	-20.51	149	-20.51	752827	24	3603	2432
miR1874-3p	LOC_Os04g16540.1	156	-20.49	156	-20.49	752864	24	2502	280
miR1874-3p	LOC_Os04g16600.1	155	-21.33	155	-21.33	752870	24	7167	1218
miR1874-3p	LOC_Os04g16660.1	155	-21.33	155	-21.33	752875	24	399	144
miR1874-3p	LOC_Os04g17280.1	168	-26.41	168	-26.41	752972	24	1962	794
miR1874-3p	LOC_Os04g17360.1	156	-22.33	156	-22.33	752980	24	469	352
miR1874-3p	LOC_Os04g17380.1	153	-20.79	153	-20.79	752982	24	519	43
miR1874-3p	LOC_Os04g17574.1	149	-22.65	149	-22.65	753000	24	1851	295
miR1874-3p	LOC_Os04g17630.1	155	-24.85	155	-24.85	753005	24	1770	186
miR1874-3p	LOC_Os04g17669.1	151	-20.35	151	-20.35	753011	24	564	159
miR1874-3p	LOC_Os04g18030.1	142	-20.19	142	-20.19	753048	24	2850	778
miR1874-3p	LOC_Os04g18030.2	142	-20.19	142	-20.19	753049	24	2775	703
miR1874-3p	LOC_Os04g18030.3	142	-20.19	142	-20.19	753050	24	2747	675
miR1874-3p	LOC_Os04g18030.4	142	-20.19	142	-20.19	753051	24	2860	788
miR1874-3p	LOC_Os04g18340.1	154	-22.42	154	-22.42	753076	24	2592	2564
miR1874-3p	LOC_Os04g18400.1	155	-21.33	155	-21.33	753081	24	3009	1863
miR1874-3p	LOC_Os04g18690.1	312	-43.74	159	-22.06	753110	24	2505	774 1073
miR1874-3p	LOC_Os04g18750.1	147	-23.72	147	-23.72	753116	24	5892	4202
miR1874-3p	LOC_Os04g18790.1	157	-20.83	157	-20.83	753120	24	1461	506
miR1874-3p	LOC_Os04g18810.1	162	-21.23	162	-21.23	753122	24	1074	23

miR1874-3p	LOC_Os04g18884.1	158	-21.02	158	-21.02	753129	24	1788	1282
miR1874-3p	LOC_Os04g19440.1	162	-20.65	162	-20.65	753183	24	402	58
miR1874-3p	LOC_Os04g19810.1	144	-23.37	144	-23.37	753220	24	1548	1025
miR1874-3p	LOC_Os04g19830.1	158	-24.07	158	-24.07	753222	24	684	224
miR1874-3p	LOC_Os04g19840.1	169	-28.72	169	-28.72	753223	24	2187	1752
miR1874-3p	LOC_Os04g20140.1	144	-25.12	144	-25.12	753250	24	2376	675
miR1874-3p	LOC_Os04g20270.1	158	-27.12	158	-27.12	753262	24	1916	388
miR1874-3p	LOC_Os04g20270.2	158	-27.12	158	-27.12	753263	24	1918	388
miR1874-3p	LOC_Os04g20720.1	140	-20.17	140	-20.17	753310	24	3138	676
miR1874-3p	LOC_Os04g20850.1	154	-21.75	154	-21.75	753322	24	4617	4111
miR1874-3p	LOC_Os04g21160.1	156	-20.46	156	-20.46	753350	24	1407	1056
miR1874-3p	LOC_Os04g21220.1	154	-22.07	154	-22.07	753356	24	2865	2843
miR1874-3p	LOC_Os04g21640.1	160	-21.36	160	-21.36	753395	24	294	86
miR1874-3p	LOC_Os04g21760.1	165	-21.7	165	-21.7	753409	24	2034	536
miR1874-3p	LOC_Os04g21900.1	166	-22.19	166	-22.19	753423	24	1038	125
miR1874-3p	LOC_Os04g22200.1	159	-24.23	159	-24.23	753453	24	4737	2258
miR1874-3p	LOC_Os04g22230.1	157	-22.27	157	-22.27	753456	24	567	58
miR1874-3p	LOC_Os04g22570.1	158	-20.18	158	-20.18	753488	24	4557	2873
miR1874-3p	LOC_Os04g22690.1	164	-24.18	164	-24.18	753499	24	4392	1388
miR1874-3p	LOC_Os04g22910.1	315	-46.4	158	-23.28	753520	24	1795	407 1471
miR1874-3p	LOC_Os04g23689.1	151	-26.92	151	-26.92	753590	24	303	282
miR1874-3p	LOC_Os04g23720.1	152	-22.57	152	-22.57	753593	24	2469	1572
miR1874-3p	LOC_Os04g24430.1	151	-23	151	-23	753665	24	2844	892
miR1874-3p	LOC_Os04g24970.1	157	-22.79	157	-22.79	753717	24	2472	110
miR1874-3p	LOC_Os04g25030.1	154	-22.07	154	-22.07	753725	24	5196	5174
miR1874-3p	LOC_Os04g25470.1	151	-20.14	151	-20.14	753765	24	5133	247
miR1874-3p	LOC_Os04g25840.1	172	-27.76	172	-27.76	753809	24	1596	360
miR1874-3p	LOC_Os04g25920.1	159	-20.72	159	-20.72	753818	24	630	200
miR1874-3p	LOC_Os04g26240.1	160	-25.17	160	-25.17	753849	24	1746	1157
miR1874-3p	LOC_Os04g26240.2	160	-25.17	160	-25.17	753850	24	1781	1157
miR1874-3p	LOC_Os04g26280.1	149	-25.92	149	-25.92	753854	24	1212	161
miR1874-3p	LOC_Os04g26660.1	156	-25.26	156	-25.26	753890	24	5040	2091

miR1874-3p	LOC_Os04g26700.1	157	-23.82	157	-23.82	753894	24	5472	1434
miR1874-3p	LOC_Os04g26980.1	154	-21.24	154	-21.24	753918	24	1389	1350
miR1874-3p	LOC_Os04g27070.1	158	-20.41	158	-20.41	753925	24	1572	1521
miR1874-3p	LOC_Os04g27200.1	157	-23.03	157	-23.03	753937	24	375	213
miR1874-3p	LOC_Os04g27270.1	141	-21.63	141	-21.63	753947	24	1486	1004
miR1874-3p	LOC_Os04g27540.1	327	-47.13	171	-26.02	753972	24	1503	1377 720
miR1874-3p	LOC_Os04g27760.1	151	-22.2	151	-22.2	753994	24	1240	786
miR1874-3p	LOC_Os04g28190.1	159	-21.78	159	-21.78	754034	24	1155	1020
miR1874-3p	LOC_Os04g28970.1	163	-23.86	163	-23.86	754109	24	393	200
miR1874-3p	LOC_Os04g29000.1	155	-21.96	155	-21.96	754112	24	1461	1041
miR1874-3p	LOC_Os04g29600.1	154	-23.58	154	-23.58	754163	24	2436	2057
miR1874-3p	LOC_Os04g29820.1	156	-23.08	156	-23.08	754182	24	2457	1383
miR1874-3p	LOC_Os04g29950.1	148	-21.53	148	-21.53	754195	24	1197	840
miR1874-3p	LOC_Os04g30190.1	154	-23.53	154	-23.53	754218	24	4686	4307
miR1874-3p	LOC_Os04g30310.1	152	-25.48	152	-25.48	754232	24	2007	21
miR1874-3p	LOC_Os04g30380.1	176	-33.4	176	-33.4	754238	24	3891	1989
miR1874-3p	LOC_Os04g30440.1	156	-20.7	156	-20.7	754243	24	3414	3173
miR1874-3p	LOC_Os04g30540.1	155	-21.28	155	-21.28	754253	24	1092	547
miR1874-3p	LOC_Os04g30660.1	152	-23.21	152	-23.21	754265	24	3231	2648
miR1874-3p	LOC_Os04g30840.1	180	-30.71	180	-30.71	754286	24	321	36
miR1874-3p	LOC_Os04g30920.1	161	-20.88	161	-20.88	754296	24	855	109
miR1874-3p	LOC_Os04g31000.1	160	-26.43	160	-26.43	754304	24	3104	56
miR1874-3p	LOC_Os04g31924.1	317	-41.32	160	-20.94	754396	24	2760	24 621
miR1874-3p	LOC_Os04g31924.2	317	-41.32	160	-20.94	754397	24	3785	24 621
miR1874-3p	LOC_Os04g32510.1	159	-21.68	159	-21.68	754460	24	975	917
miR1874-3p	LOC_Os04g32880.1	161	-20.13	161	-20.13	754502	24	2139	1442
miR1874-3p	LOC_Os04g32880.2	161	-20.13	161	-20.13	754503	24	1850	1153
miR1874-3p	LOC_Os04g32880.3	161	-20.13	161	-20.13	754504	24	1772	1075
miR1874-3p	LOC_Os04g32880.4	161	-20.13	161	-20.13	754505	24	1947	1250
miR1874-3p	LOC_Os04g32880.5	161	-20.13	161	-20.13	754506	24	1884	1187
miR1874-3p	LOC_Os04g32960.1	160	-27.75	160	-27.75	754521	24	3492	288
miR1874-3p	LOC_Os04g32970.1	153	-23.32	153	-23.32	754522	24	1378	1183

miR1874-3p	LOC_Os04g33080.1	164	-21.27	164	-21.27	754532	24	2573	2145
miR1874-3p	LOC_Os04g33080.3	164	-21.27	164	-21.27	754534	24	2619	2152
miR1874-3p	LOC_Os04g33430.1	169	-28.38	169	-28.38	754575	24	366	134
miR1874-3p	LOC_Os04g33530.1	147	-25.19	147	-25.19	754588	24	1705	1020
miR1874-3p	LOC_Os04g34220.1	158	-22.96	158	-22.96	754661	24	546	286
miR1874-3p	LOC_Os04g34470.1	160	-22.99	160	-22.99	754684	24	459	182
miR1874-3p	LOC_Os04g34590.1	158	-22.08	158	-22.08	754698	24	1616	1549
miR1874-3p	LOC_Os04g34720.1	149	-21.25	149	-21.25	754708	24	2256	985
miR1874-3p	LOC_Os04g34720.2	149	-21.25	149	-21.25	754709	24	2353	1082
miR1874-3p	LOC_Os04g34984.1	154	-20.99	154	-20.99	754738	24	2224	81
miR1874-3p	LOC_Os04g34984.2	154	-20.99	154	-20.99	754739	24	2771	81
miR1874-3p	LOC_Os04g34984.3	154	-20.99	154	-20.99	754740	24	2314	81
miR1874-3p	LOC_Os04g35260.1	322	-48.88	162	-24.95	754767	24	4533	2981 3617
miR1874-3p	LOC_Os04g35430.1	153	-21.04	153	-21.04	754787	24	4486	3255
miR1874-3p	LOC_Os04g35600.1	169	-26.19	169	-26.19	754812	24	1877	512
miR1874-3p	LOC_Os04g35700.1	156	-22.45	156	-22.45	754822	24	2743	1726
miR1874-3p	LOC_Os04g35700.2	156	-22.45	156	-22.45	754823	24	2046	1029
miR1874-3p	LOC_Os04g35910.1	152	-21.5	152	-21.5	754847	24	1734	379
miR1874-3p	LOC_Os04g36570.1	149	-20.67	149	-20.67	754868	24	1626	1399
miR1874-3p	LOC_Os04g36740.1	141	-26.19	141	-26.19	754886	24	2303	948
miR1874-3p	LOC_Os04g36740.2	141	-26.19	141	-26.19	754887	24	1646	948
miR1874-3p	LOC_Os04g36790.1	146	-23.74	146	-23.74	754892	24	1937	113
miR1874-3p	LOC_Os04g37460.1	152	-23.67	152	-23.67	754913	24	1925	1537
miR1874-3p	LOC_Os04g37860.1	158	-21.78	158	-21.78	754957	24	738	224
miR1874-3p	LOC_Os04g37870.1	169	-28.72	169	-28.72	754958	24	2631	2196
miR1874-3p	LOC_Os04g37920.1	155	-21.11	155	-21.11	754963	24	2896	1760
miR1874-3p	LOC_Os04g37920.3	155	-21.11	155	-21.11	754964	24	2889	1760
miR1874-3p	LOC_Os04g37920.4	155	-21.11	155	-21.11	754965	24	2913	1760
miR1874-3p	LOC_Os04g37920.6	155	-21.11	155	-21.11	754966	24	4778	2783
miR1874-3p	LOC_Os04g38120.1	158	-21.02	158	-21.02	754983	24	1698	1270
miR1874-3p	LOC_Os04g38140.1	154	-22.07	154	-22.07	754985	24	1389	1367
miR1874-3p	LOC_Os04g38240.1	154	-23.53	154	-23.53	754994	24	4758	4379

miR1874-3p	LOC_Os04g38620.1	156	-23.92	156	-23.92	755042	24	1244	471
miR1874-3p	LOC_Os04g38780.1	166	-20.13	166	-20.13	755061	24	648	624
miR1874-3p	LOC_Os04g38830.1	158	-24.62	158	-24.62	755066	24	2346	1965
miR1874-3p	LOC_Os04g39629.1	163	-28.7	163	-28.7	755174	24	2915	305
miR1874-3p	LOC_Os04g40060.1	151	-20.9	151	-20.9	755219	24	3277	753
miR1874-3p	LOC_Os04g40130.1	166	-20.96	166	-20.96	755226	24	2567	1756
miR1874-3p	LOC_Os04g40360.1	154	-23.58	154	-23.58	755254	24	4110	3797
miR1874-3p	LOC_Os04g40370.1	170	-27.24	170	-27.24	755255	24	2028	1200
miR1874-3p	LOC_Os04g40370.2	170	-27.24	170	-27.24	755256	24	2025	1197
miR1874-3p	LOC_Os04g40370.3	170	-27.24	170	-27.24	755257	24	1729	901
miR1874-3p	LOC_Os04g40900.1	150	-24.07	150	-24.07	755328	24	1122	892
miR1874-3p	LOC_Os04g41250.1	163	-24.63	163	-24.63	755375	24	2939	864
miR1874-3p	LOC_Os04g41599.1	155	-20.27	155	-20.27	755414	24	5862	5830
miR1874-3p	LOC_Os04g41690.1	163	-23.56	163	-23.56	755422	24	3324	1062
miR1874-3p	LOC_Os04g41840.1	320	-40.67	160	-20.49	755440	24	4668	2195 4318
miR1874-3p	LOC_Os04g42030.1	164	-24.07	164	-24.07	755461	24	2146	158
miR1874-3p	LOC_Os04g42130.1	160	-24.98	160	-24.98	755474	24	2126	1780
miR1874-3p	LOC_Os04g42600.1	150	-21.34	150	-21.34	755535	24	3135	2028
miR1874-3p	LOC_Os04g42600.2	150	-21.34	150	-21.34	755536	24	2637	1530
miR1874-3p	LOC_Os04g42610.1	160	-20.25	160	-20.25	755537	24	2784	2265
miR1874-3p	LOC_Os04g42620.1	148	-21.14	148	-21.14	755538	24	1883	1520
miR1874-3p	LOC_Os04g42634.1	158	-21.78	158	-21.78	755539	24	702	315
miR1874-3p	LOC_Os04g42770.2	165	-23.88	165	-23.88	755552	24	3631	3537
miR1874-3p	LOC_Os04g42770.5	165	-23.88	165	-23.88	755555	24	3628	3534
miR1874-3p	LOC_Os04g42770.6	165	-23.88	165	-23.88	755556	24	3645	3551
miR1874-3p	LOC_Os04g42840.1	158	-23.05	158	-23.05	755565	24	4899	2567
miR1874-3p	LOC_Os04g43270.1	172	-25.27	172	-25.27	755624	24	2391	317
miR1874-3p	LOC_Os04g43340.1	151	-22.54	151	-22.54	755632	24	3630	1139
miR1874-3p	LOC_Os04g43500.1	154	-21.04	154	-21.04	755653	24	4251	4229
miR1874-3p	LOC_Os04g43840.1	169	-26.68	169	-26.68	755688	24	1109	18
miR1874-3p	LOC_Os04g43850.1	154	-21.04	154	-21.04	755689	24	3315	3293
miR1874-3p	LOC_Os04g44250.1	158	-21.66	158	-21.66	755733	24	1705	1034

miR1874-3p	LOC_Os04g44354.4	160	-22.45	160	-22.45	755746	24	3650	1644
miR1874-3p	LOC_Os04g44610.1	141	-23.27	141	-23.27	755782	24	2680	1443
miR1874-3p	LOC_Os04g44730.1	149	-21.26	149	-21.26	755795	24	1498	467
miR1874-3p	LOC_Os04g44970.1	156	-20.06	156	-20.06	755827	24	3090	180
miR1874-3p	LOC_Os04g44980.1	155	-21.1	155	-21.1	755828	24	1248	780
miR1874-3p	LOC_Os04g45080.1	158	-20.13	158	-20.13	755843	24	922	444
miR1874-3p	LOC_Os04g45970.1	160	-27.06	160	-27.06	755941	24	2102	1211
miR1874-3p	LOC_Os04g46240.1	156	-25.26	156	-25.26	755972	24	957	752
miR1874-3p	LOC_Os04g46370.1	153	-22.44	153	-22.44	755985	24	1523	1117
miR1874-3p	LOC_Os04g46370.2	153	-20.29	153	-20.29	755986	24	1636	1231
miR1874-3p	LOC_Os04g46720.1	157	-21.83	157	-21.83	756032	24	165	140
miR1874-3p	LOC_Os04g47059.1	142	-20.07	142	-20.07	756081	24	1791	236
miR1874-3p	LOC_Os04g47650.1	155	-22.96	155	-22.96	756151	24	424	102
miR1874-3p	LOC_Os04g47990.1	147	-20.44	147	-20.44	756195	24	2345	2070
miR1874-3p	LOC_Os04g48070.1	152	-26.38	152	-26.38	756205	24	3110	1771
miR1874-3p	LOC_Os04g48070.2	152	-26.38	152	-26.38	756206	24	3089	1750
miR1874-3p	LOC_Os04g48070.3	152	-26.38	152	-26.38	756207	24	2246	1010
miR1874-3p	LOC_Os04g48280.1	156	-21.08	156	-21.08	756231	24	795	771
miR1874-3p	LOC_Os04g48520.1	156	-20.44	156	-20.44	756257	24	2430	1373
miR1874-3p	LOC_Os04g48590.1	156	-20.7	156	-20.7	756264	24	1680	1166
miR1874-3p	LOC_Os04g48760.1	156	-22.04	156	-22.04	756283	24	4296	81
miR1874-3p	LOC_Os04g48870.1	151	-20.05	151	-20.05	756297	24	873	492
miR1874-3p	LOC_Os04g49110.1	154	-25.23	154	-25.23	756325	24	2244	225
miR1874-3p	LOC_Os04g49490.1	159	-22.18	159	-22.18	756362	24	2828	1670
miR1874-3p	LOC_Os04g50172.1	154	-21.23	154	-21.23	756445	24	2355	395
miR1874-3p	LOC_Os04g50670.1	155	-25.93	155	-25.93	756459	24	924	63
miR1874-3p	LOC_Os04g50940.1	147	-20.98	147	-20.98	756487	24	2265	2073
miR1874-3p	LOC_Os04g50940.2	147	-20.98	147	-20.98	756488	24	3072	2867
miR1874-3p	LOC_Os04g51210.1	150	-21.2	150	-21.2	756524	24	603	576
miR1874-3p	LOC_Os04g51320.1	155	-21.71	155	-21.71	756536	24	1153	406
miR1874-3p	LOC_Os04g52280.1	144	-20.63	144	-20.63	756667	24	3280	940
miR1874-3p	LOC_Os04g52390.1	151	-20.45	151	-20.45	756681	24	3493	820

miR1874-3p	LOC_Os04g52630.1	147	-20.08	147	-20.08	756710	24	3111	2755
miR1874-3p	LOC_Os04g53210.1	150	-20.18	150	-20.18	756783	24	1539	17
miR1874-3p	LOC_Os04g53210.3	150	-20.18	150	-20.18	756784	24	1476	17
miR1874-3p	LOC_Os04g53270.1	154	-20.7	154	-20.7	756795	24	2499	281
miR1874-3p	LOC_Os04g53310.1	165	-22.18	165	-22.18	756799	24	5084	4341
miR1874-3p	LOC_Os04g53330.1	150	-23.04	150	-23.04	756801	24	1435	174
miR1874-3p	LOC_Os04g53330.2	150	-23.04	150	-23.04	756802	24	1426	167
miR1874-3p	LOC_Os04g53380.1	150	-20.24	150	-20.24	756807	24	8342	6176
miR1874-3p	LOC_Os04g53496.1	460	-69.89	160	-23.39	756823	24	5424	548 4504 5272
miR1874-3p	LOC_Os04g53760.1	153	-20.36	153	-20.36	756860	24	3200	2401
miR1874-3p	LOC_Os04g53760.2	153	-20.36	153	-20.36	756861	24	3125	2326
miR1874-3p	LOC_Os04g53790.1	176	-33.4	176	-33.4	756865	24	4218	2241
miR1874-3p	LOC_Os04g54002.1	147	-20.64	147	-20.64	756892	24	3002	2152
miR1874-3p	LOC_Os04g54040.1	146	-22.24	146	-22.24	756896	24	756	26
miR1874-3p	LOC_Os04g54260.1	151	-25.25	151	-25.25	756921	24	6087	2556
miR1874-3p	LOC_Os04g54270.1	141	-22.41	141	-22.41	756922	24	3144	2890
miR1874-3p	LOC_Os04g54280.1	173	-31.5	173	-31.5	756923	24	2499	303
miR1874-3p	LOC_Os04g54720.1	150	-20.62	150	-20.62	756974	24	672	355
miR1874-3p	LOC_Os04g55210.1	151	-20.16	151	-20.16	757029	24	2805	1301
miR1874-3p	LOC_Os04g55240.1	164	-22.51	164	-22.51	757032	24	1287	1076
miR1874-3p	LOC_Os04g55540.1	156	-20.06	156	-20.06	757066	24	3174	147
miR1874-3p	LOC_Os04g55730.1	158	-25.45	158	-25.45	757090	24	2892	1893
miR1874-3p	LOC_Os04g56140.1	153	-21.35	153	-21.35	757138	24	2275	1471
miR1874-3p	LOC_Os04g56140.2	153	-21.35	153	-21.35	757139	24	2249	1462
miR1874-3p	LOC_Os04g56140.3	153	-21.35	153	-21.35	757140	24	2194	1471
miR1874-3p	LOC_Os04g56240.1	153	-22.29	153	-22.29	757151	24	2019	205
miR1874-3p	LOC_Os04g56560.1	147	-21.09	147	-21.09	757192	24	2051	69
miR1874-3p	LOC_Os04g56890.1	147	-23.52	147	-23.52	757232	24	1767	1340
miR1874-3p	LOC_Os04g56990.1	148	-20.1	148	-20.1	757244	24	2626	1214
miR1874-3p	LOC_Os04g56990.2	148	-20.1	148	-20.1	757245	24	2765	1353
miR1874-3p	LOC_Os04g56990.3	148	-20.1	148	-20.1	757246	24	2620	1208
miR1874-3p	LOC_Os04g57170.1	153	-25.81	153	-25.81	757270	24	516	70

miR1874-3p	LOC_Os04g57180.1	159	-22.52	159	-22.52	757271	24	2727	366
miR1874-3p	LOC_Os04g57480.1	155	-21	155	-21	757315	24	1875	1010
miR1874-3p	LOC_Os04g57480.2	155	-21	155	-21	757316	24	1830	1010
miR1874-3p	LOC_Os04g57480.3	155	-21	155	-21	757317	24	2501	1629
miR1874-3p	LOC_Os04g57480.4	155	-21	155	-21	757318	24	1848	1012
miR1874-3p	LOC_Os04g57480.5	155	-21	155	-21	757319	24	1772	936
miR1874-3p	LOC_Os04g57670.1	155	-21.03	155	-21.03	757345	24	3206	2427
miR1874-3p	LOC_Os04g57890.1	150	-20.84	150	-20.84	757378	24	3552	1497
miR1874-3p	LOC_Os04g58040.1	153	-20.15	153	-20.15	757391	24	804	500
miR1874-3p	LOC_Os04g58140.1	155	-21.38	155	-21.38	757403	24	2491	1304
miR1874-3p	LOC_Os04g58140.2	155	-21.38	155	-21.38	757404	24	3022	1275
miR1874-3p	LOC_Os04g58160.2	156	-21.3	156	-21.3	757407	24	1150	192
miR1874-3p	LOC_Os04g58180.1	143	-22.06	143	-22.06	757409	24	3028	1325
miR1874-3p	LOC_Os04g58780.1	153	-21.54	153	-21.54	757491	24	3519	2194
miR1874-3p	LOC_Os04g58780.2	153	-21.54	153	-21.54	757492	24	3499	2174
miR1874-3p	LOC_Os04g59000.1	151	-21.79	151	-21.79	757515	24	2473	320
miR1874-3p	LOC_Os04g59140.1	156	-25.62	156	-25.62	757531	24	1894	96
miR1874-3p	LOC_Os04g59170.1	154	-23.05	154	-23.05	757535	24	2466	281
miR1874-3p	LOC_Os04g59290.1	173	-27.75	173	-27.75	757547	24	5616	456
miR1874-3p	LOC_Os04g59320.1	145	-22.08	145	-22.08	757550	24	2756	1906
miR1874-3p	LOC_Os04g59480.1	151	-20.64	151	-20.64	757569	24	1917	524
miR1874-3p	LOC_Os04g59510.1	160	-20.91	160	-20.91	757573	24	2541	2489
miR1874-3p	LOC_Os04g59600.2	155	-20.23	155	-20.23	757590	24	2293	2016
miR1874-3p	LOC_Os05g00990.1	159	-21.67	159	-21.67	757600	24	1692	1359
miR1874-3p	LOC_Os05g01040.3	150	-20.93	150	-20.93	757615	24	2417	749
miR1874-3p	LOC_Os05g01110.1	171	-21.46	171	-21.46	757628	24	845	38
miR1874-3p	LOC_Os05g01240.1	173	-26.31	173	-26.31	757642	24	3944	216
miR1874-3p	LOC_Os05g01240.2	173	-26.31	173	-26.31	757643	24	3543	216
miR1874-3p	LOC_Os05g01240.3	173	-26.31	173	-26.31	757644	24	3508	216
miR1874-3p	LOC_Os05g01240.4	333	-52.63	173	-26.32	757645	24	3578	216 3398
miR1874-3p	LOC_Os05g01240.5	338	-50.9	173	-26.31	757646	24	3676	216 3496
miR1874-3p	LOC_Os05g01280.1	176	-27.82	176	-27.82	757653	24	950	515

miR1874-3p	LOC_Os05g01520.1	154	-22.81	154	-22.81	757688	24	2496	1605
miR1874-3p	LOC_Os05g01950.1	160	-23.8	160	-23.8	757740	24	829	503
miR1874-3p	LOC_Os05g01990.1	163	-25.62	163	-25.62	757747	24	2501	2419
miR1874-3p	LOC_Os05g01994.1	163	-25.62	163	-25.62	757749	24	758	238
miR1874-3p	LOC_Os05g02110.1	148	-25.19	148	-25.19	757765	24	1782	173
miR1874-3p	LOC_Os05g02580.1	151	-21.53	151	-21.53	757826	24	3255	1918
miR1874-3p	LOC_Os05g03000.1	151	-20.6	151	-20.6	757872	24	1818	1640
miR1874-3p	LOC_Os05g03000.2	151	-20.6	151	-20.6	757873	24	1835	1677
miR1874-3p	LOC_Os05g03200.1	164	-26.36	164	-26.36	757898	24	1376	877
miR1874-3p	LOC_Os05g03550.1	151	-20.25	151	-20.25	757942	24	5167	1554
miR1874-3p	LOC_Os05g04480.1	154	-23.53	154	-23.53	758044	24	2898	2519
miR1874-3p	LOC_Os05g04600.1	153	-22.08	153	-22.08	758055	24	1763	1237
miR1874-3p	LOC_Os05g04640.1	146	-23.01	146	-23.01	758064	24	1551	1214
miR1874-3p	LOC_Os05g04680.1	163	-24.08	163	-24.08	758068	24	877	569
miR1874-3p	LOC_Os05g04740.1	159	-23.31	159	-23.31	758074	24	1795	12
miR1874-3p	LOC_Os05g04740.2	159	-23.31	159	-23.31	758075	24	1885	12
miR1874-3p	LOC_Os05g05050.1	176	-33.4	176	-33.4	758105	24	4113	2241
miR1874-3p	LOC_Os05g05090.1	163	-23.53	163	-23.53	758109	24	3114	1392
miR1874-3p	LOC_Os05g05230.1	159	-20.88	159	-20.88	758126	24	3806	3003
miR1874-3p	LOC_Os05g05270.1	157	-21.76	157	-21.76	758131	24	2369	1129
miR1874-3p	LOC_Os05g05600.1	177	-30.89	177	-30.89	758170	24	2691	1304
miR1874-3p	LOC_Os05g05600.2	177	-30.89	177	-30.89	758171	24	2798	1411
miR1874-3p	LOC_Os05g05600.3	177	-30.89	177	-30.89	758172	24	2681	1294
miR1874-3p	LOC_Os05g05600.4	177	-30.89	177	-30.89	758173	24	2720	1333
miR1874-3p	LOC_Os05g05600.5	177	-30.89	177	-30.89	758174	24	2630	1244
miR1874-3p	LOC_Os05g05600.6	177	-30.89	177	-30.89	758175	24	2893	1506
miR1874-3p	LOC_Os05g05780.1	152	-25.23	152	-25.23	758195	24	4228	1876
miR1874-3p	LOC_Os05g05860.1	150	-24.77	150	-24.77	758205	24	5950	5927
miR1874-3p	LOC_Os05g05950.1	149	-23.36	149	-23.36	758214	24	4276	2669
miR1874-3p	LOC_Os05g05980.1	168	-31.92	168	-31.92	758217	24	636	165
miR1874-3p	LOC_Os05g06200.1	332	-55.22	168	-30.03	758240	24	2475	2451 430
miR1874-3p	LOC_Os05g06300.1	156	-27.12	156	-27.12	758253	24	2808	2697

miR1874-3p	LOC_Os05g06320.2	324	-46.82	162	-23.41	758255	24	3466	447 620
miR1874-3p	LOC_Os05g06320.3	324	-46.82	162	-23.41	758256	24	1915	447 620
miR1874-3p	LOC_Os05g06460.1	160	-24.67	160	-24.67	758272	24	2160	982
miR1874-3p	LOC_Os05g06710.1	164	-26.26	164	-26.26	758305	24	1841	387
miR1874-3p	LOC_Os05g07070.3	154	-20.7	154	-20.7	758348	24	6422	5691
miR1874-3p	LOC_Os05g07070.8	154	-20.7	154	-20.7	758352	24	5982	5251
miR1874-3p	LOC_Os05g07280.1	155	-20.93	155	-20.93	758377	24	2680	295
miR1874-3p	LOC_Os05g07290.1	176	-33.4	176	-33.4	758378	24	3705	2241
miR1874-3p	LOC_Os05g07350.1	154	-22.07	154	-22.07	758385	24	5415	5393
miR1874-3p	LOC_Os05g07560.1	149	-22.34	149	-22.34	758401	24	4291	4030
miR1874-3p	LOC_Os05g07890.1	163	-21.7	163	-21.7	758440	24	781	634
miR1874-3p	LOC_Os05g07920.1	163	-23.56	163	-23.56	758444	24	4137	447
miR1874-3p	LOC_Os05g08190.1	156	-21.23	156	-21.23	758478	24	1077	1010
miR1874-3p	LOC_Os05g08554.1	155	-22.09	155	-22.09	758519	24	676	132
miR1874-3p	LOC_Os05g09180.1	150	-21.18	150	-21.18	758598	24	3504	3309
miR1874-3p	LOC_Os05g10610.1	153	-22.43	153	-22.43	758712	24	1506	673
miR1874-3p	LOC_Os05g10650.1	159	-22.58	159	-22.58	758719	24	2101	1502
miR1874-3p	LOC_Os05g10730.1	150	-21.33	150	-21.33	758727	24	6041	3528
miR1874-3p	LOC_Os05g10730.2	150	-21.33	150	-21.33	758728	24	6094	3581
miR1874-3p	LOC_Os05g10730.3	150	-21.33	150	-21.33	758729	24	6127	3528
miR1874-3p	LOC_Os05g10770.1	153	-20.21	153	-20.21	758736	24	4353	94
miR1874-3p	LOC_Os05g10920.1	163	-24.22	163	-24.22	758753	24	2800	2671
miR1874-3p	LOC_Os05g11110.1	170	-27.26	170	-27.26	758774	24	3999	748
miR1874-3p	LOC_Os05g11120.1	168	-20.75	168	-20.75	758775	24	1186	467
miR1874-3p	LOC_Os05g11370.1	153	-22.3	153	-22.3	758802	24	1801	72
miR1874-3p	LOC_Os05g11810.1	156	-24.61	156	-24.61	758845	24	2572	539
miR1874-3p	LOC_Os05g11810.2	156	-24.61	156	-24.61	758846	24	2036	328
miR1874-3p	LOC_Os05g11970.1	140	-20.14	140	-20.14	758861	24	1302	1031
miR1874-3p	LOC_Os05g12620.1	163	-23.31	163	-23.31	758934	24	3336	1526
miR1874-3p	LOC_Os05g12680.1	148	-21.96	148	-21.96	758944	24	2528	97
miR1874-3p	LOC_Os05g12770.1	156	-21.11	156	-21.11	758952	24	3498	2616
miR1874-3p	LOC_Os05g12790.1	149	-20.04	149	-20.04	758954	24	387	250

miR1874-3p	LOC_Os05g13780.1	162	-26.5	162	-26.5	759006	24	4224	3249
miR1874-3p	LOC_Os05g13790.1	162	-26.5	162	-26.5	759007	24	2212	1417
miR1874-3p	LOC_Os05g13800.1	152	-22.63	152	-22.63	759008	24	2334	1284
miR1874-3p	LOC_Os05g14170.1	149	-23.73	149	-23.73	759050	24	3540	1812
miR1874-3p	LOC_Os05g14270.1	146	-20.31	146	-20.31	759062	24	1574	81
miR1874-3p	LOC_Os05g14440.1	177	-26.23	177	-26.23	759080	24	2256	604
miR1874-3p	LOC_Os05g14560.1	161	-22.77	161	-22.77	759090	24	2580	2035
miR1874-3p	LOC_Os05g14800.1	160	-21.69	160	-21.69	759113	24	996	608
miR1874-3p	LOC_Os05g14860.1	152	-24.98	152	-24.98	759119	24	1468	51
miR1874-3p	LOC_Os05g14930.1	145	-20.5	145	-20.5	759125	24	2304	628
miR1874-3p	LOC_Os05g15470.1	154	-22.07	154	-22.07	759187	24	5052	5030
miR1874-3p	LOC_Os05g15560.1	158	-21.02	158	-21.02	759196	24	2964	2458
miR1874-3p	LOC_Os05g15720.1	172	-30.68	172	-30.68	759212	24	3641	2510
miR1874-3p	LOC_Os05g15980.1	154	-23.05	154	-23.05	759238	24	2487	281
miR1874-3p	LOC_Os05g16280.1	147	-21.89	147	-21.89	759266	24	1923	1897
miR1874-3p	LOC_Os05g16680.1	154	-20.7	154	-20.7	759310	24	2481	281
miR1874-3p	LOC_Os05g16720.1	154	-22.07	154	-22.07	759314	24	5412	5390
miR1874-3p	LOC_Os05g16770.1	147	-23.72	147	-23.72	759318	24	816	368
miR1874-3p	LOC_Os05g16950.1	154	-22.42	154	-22.42	759337	24	2034	2006
miR1874-3p	LOC_Os05g17010.1	158	-21.26	158	-21.26	759343	24	1440	246
miR1874-3p	LOC_Os05g17070.1	147	-20.82	147	-20.82	759349	24	483	327
miR1874-3p	LOC_Os05g17100.1	154	-22.07	154	-22.07	759352	24	5361	5339
miR1874-3p	LOC_Os05g17200.1	154	-22.42	154	-22.42	759362	24	852	824
miR1874-3p	LOC_Os05g17220.1	154	-22.42	154	-22.42	759364	24	1401	1373
miR1874-3p	LOC_Os05g17240.1	154	-22.42	154	-22.42	759366	24	1401	1373
miR1874-3p	LOC_Os05g17310.1	154	-22.42	154	-22.42	759373	24	420	392
miR1874-3p	LOC_Os05g17320.1	154	-22.07	154	-22.07	759374	24	5379	5357
miR1874-3p	LOC_Os05g17370.1	153	-20.94	153	-20.94	759379	24	805	338
miR1874-3p	LOC_Os05g17430.1	153	-20.94	153	-20.94	759385	24	885	338
miR1874-3p	LOC_Os05g17604.3	155	-20.01	155	-20.01	759403	24	5099	4711
miR1874-3p	LOC_Os05g17630.1	153	-20.94	153	-20.94	759407	24	5134	4706
miR1874-3p	LOC_Os05g17849.1	154	-20.69	154	-20.69	759428	24	1565	81

miR1874-3p	LOC_Os05g18000.1	170	-25.56	170	-25.56	759443	24	3510	58
miR1874-3p	LOC_Os05g18240.1	152	-20.33	152	-20.33	759466	24	663	119
miR1874-3p	LOC_Os05g18294.2	156	-26.05	156	-26.05	759473	24	1660	1285
miR1874-3p	LOC_Os05g18294.5	156	-26.05	156	-26.05	759476	24	2256	1881
miR1874-3p	LOC_Os05g18500.1	153	-20.94	153	-20.94	759493	24	1200	596
miR1874-3p	LOC_Os05g18530.1	160	-22.72	160	-22.72	759497	24	648	211
miR1874-3p	LOC_Os05g18604.3	152	-21.39	152	-21.39	759504	24	1850	223
miR1874-3p	LOC_Os05g19080.1	150	-21.29	150	-21.29	759556	24	1305	1079
miR1874-3p	LOC_Os05g19140.1	154	-22.07	154	-22.07	759562	24	5214	5192
miR1874-3p	LOC_Os05g19200.1	154	-22.07	154	-22.07	759568	24	3342	3320
miR1874-3p	LOC_Os05g19440.1	154	-22.42	154	-22.42	759592	24	711	683
miR1874-3p	LOC_Os05g19560.1	154	-22.07	154	-22.07	759611	24	5832	5810
miR1874-3p	LOC_Os05g19650.1	158	-21.02	158	-21.02	759621	24	5226	4609
miR1874-3p	LOC_Os05g19870.1	154	-22.07	154	-22.07	759640	24	5373	5351
miR1874-3p	LOC_Os05g20129.1	158	-20.8	158	-20.8	759671	24	5355	4609
miR1874-3p	LOC_Os05g20230.1	154	-22.07	154	-22.07	759680	24	5196	5174
miR1874-3p	LOC_Os05g20520.1	158	-21.02	158	-21.02	759709	24	870	364
miR1874-3p	LOC_Os05g21000.1	154	-22.42	154	-22.42	759764	24	1743	1715
miR1874-3p	LOC_Os05g22370.1	154	-28.26	154	-28.26	759801	24	1229	890
miR1874-3p	LOC_Os05g22370.2	154	-28.26	154	-28.26	759802	24	2756	2417
miR1874-3p	LOC_Os05g22370.4	154	-28.26	154	-28.26	759804	24	1665	1326
miR1874-3p	LOC_Os05g22820.1	158	-21.02	158	-21.02	759850	24	786	358
miR1874-3p	LOC_Os05g22870.1	153	-21.43	153	-21.43	759856	24	2499	1655
miR1874-3p	LOC_Os05g22930.1	159	-26.53	159	-26.53	759864	24	1218	682
miR1874-3p	LOC_Os05g22960.1	145	-23.42	145	-23.42	759866	24	522	300
miR1874-3p	LOC_Os05g23530.1	154	-20.28	154	-20.28	759931	24	2639	2450
miR1874-3p	LOC_Os05g23530.2	154	-20.28	154	-20.28	759932	24	2661	2472
miR1874-3p	LOC_Os05g23530.4	154	-20.28	154	-20.28	759934	24	2485	2450
miR1874-3p	LOC_Os05g23550.1	158	-21.1	158	-21.1	759935	24	1074	181
miR1874-3p	LOC_Os05g23560.1	160	-22.63	160	-22.63	759936	24	258	5
miR1874-3p	LOC_Os05g23700.1	158	-20.36	158	-20.36	759954	24	1393	20
miR1874-3p	LOC_Os05g23770.1	169	-28.72	169	-28.72	759959	24	2631	2196

miR1874-3p	LOC_Os05g23870.1	155	-21.44	155	-21.44	759969	24	1629	1142
miR1874-3p	LOC_Os05g23980.1	154	-22.4	154	-22.4	759980	24	2187	2072
miR1874-3p	LOC_Os05g24240.1	165	-22.91	165	-22.91	760009	24	1659	1291
miR1874-3p	LOC_Os05g24470.1	156	-20.06	156	-20.06	760031	24	3165	180
miR1874-3p	LOC_Os05g24680.1	153	-20.94	153	-20.94	760059	24	5664	4946
miR1874-3p	LOC_Os05g24860.1	159	-22.12	159	-22.12	760075	24	2574	1499
miR1874-3p	LOC_Os05g24930.1	158	-20.63	158	-20.63	760083	24	3281	410
miR1874-3p	LOC_Os05g25040.1	154	-23.33	154	-23.33	760095	24	1437	1381
miR1874-3p	LOC_Os05g25050.1	149	-20.2	149	-20.2	760098	24	747	692
miR1874-3p	LOC_Os05g25070.1	320	-46.37	168	-26.34	760100	24	1553	311 1213
miR1874-3p	LOC_Os05g25320.1	156	-21.6	156	-21.6	760127	24	3200	1758
miR1874-3p	LOC_Os05g25370.1	155	-21.53	155	-21.53	760132	24	3039	1633
miR1874-3p	LOC_Os05g25390.1	150	-24.24	150	-24.24	760134	24	2325	1913
miR1874-3p	LOC_Os05g25610.1	143	-24.28	143	-24.28	760158	24	1542	609
miR1874-3p	LOC_Os05g25860.1	174	-27.2	174	-27.2	760184	24	2177	1
miR1874-3p	LOC_Os05g26410.1	158	-20.31	158	-20.31	760244	24	4005	3559
miR1874-3p	LOC_Os05g26470.1	162	-25.86	162	-25.86	760250	24	4167	3663
miR1874-3p	LOC_Os05g26660.1	158	-21.88	158	-21.88	760269	24	2194	960
miR1874-3p	LOC_Os05g27350.1	145	-22.83	145	-22.83	760333	24	1502	1296
miR1874-3p	LOC_Os05g28120.1	154	-23.58	154	-23.58	760418	24	5565	5186
miR1874-3p	LOC_Os05g28300.1	152	-21.8	152	-21.8	760438	24	4481	973
miR1874-3p	LOC_Os05g28460.1	151	-23.95	151	-23.95	760454	24	1501	193
miR1874-3p	LOC_Os05g30010.1	146	-20.67	146	-20.67	760579	24	4251	1290
miR1874-3p	LOC_Os05g30130.1	156	-23.23	156	-23.23	760591	24	1904	1664
miR1874-3p	LOC_Os05g30170.1	154	-23.58	154	-23.58	760596	24	1665	1286
miR1874-3p	LOC_Os05g30240.1	162	-26.17	162	-26.17	760603	24	3218	2315
miR1874-3p	LOC_Os05g30340.1	155	-22.83	155	-22.83	760614	24	1837	1232
miR1874-3p	LOC_Os05g30340.2	155	-22.83	155	-22.83	760615	24	1888	1437
miR1874-3p	LOC_Os05g30530.1	145	-20.11	145	-20.11	760638	24	1166	705
miR1874-3p	LOC_Os05g30870.2	147	-20.4	147	-20.4	760683	24	2938	17
miR1874-3p	LOC_Os05g31230.3	160	-24.98	160	-24.98	760726	24	3583	2538
miR1874-3p	LOC_Os05g31430.1	154	-23.05	154	-23.05	760743	24	2466	281

miR1874-3p	LOC_Os05g31570.1	157	-22.09	157	-22.09	760759	24	4693	54
miR1874-3p	LOC_Os05g31610.1	147	-23.31	147	-23.31	760763	24	4329	649
miR1874-3p	LOC_Os05g31620.1	151	-22.42	151	-22.42	760764	24	979	226
miR1874-3p	LOC_Os05g31970.1	153	-22.49	153	-22.49	760799	24	871	23
miR1874-3p	LOC_Os05g32000.1	148	-21.1	148	-21.1	760801	24	1257	1032
miR1874-3p	LOC_Os05g32060.1	160	-23.83	160	-23.83	760807	24	1958	1223
miR1874-3p	LOC_Os05g32190.1	154	-20.12	154	-20.12	760823	24	886	249
miR1874-3p	LOC_Os05g32220.1	166	-22.47	166	-22.47	760826	24	1591	1284
miR1874-3p	LOC_Os05g32730.1	155	-25.02	155	-25.02	760885	24	1662	786
miR1874-3p	LOC_Os05g32960.1	161	-22.23	161	-22.23	760909	24	2541	459
miR1874-3p	LOC_Os05g33370.1	154	-23.58	154	-23.58	760956	24	4437	4058
miR1874-3p	LOC_Os05g33390.1	156	-21.57	156	-21.57	760958	24	4278	491
miR1874-3p	LOC_Os05g34050.1	148	-20.6	148	-20.6	761036	24	2624	1664
miR1874-3p	LOC_Os05g34210.1	171	-22.8	171	-22.8	761053	24	2794	1061
miR1874-3p	LOC_Os05g34670.1	157	-26.81	157	-26.81	761109	24	1876	1320
miR1874-3p	LOC_Os05g34830.3	162	-25.51	162	-25.51	761130	24	1592	285
miR1874-3p	LOC_Os05g34900.1	158	-26.59	158	-26.59	761138	24	2683	752
miR1874-3p	LOC_Os05g34900.2	158	-26.59	158	-26.59	761139	24	2678	752
miR1874-3p	LOC_Os05g35360.1	168	-20.01	168	-20.01	761194	24	2543	1867
miR1874-3p	LOC_Os05g35410.1	151	-27.8	151	-27.8	761199	24	2626	2406
miR1874-3p	LOC_Os05g36070.1	166	-22.69	166	-22.69	761269	24	1713	94
miR1874-3p	LOC_Os05g36070.2	166	-22.69	166	-22.69	761270	24	1819	87
miR1874-3p	LOC_Os05g36070.3	166	-22.69	166	-22.69	761271	24	1633	91
miR1874-3p	LOC_Os05g36360.1	147	-20.69	147	-20.69	761311	24	1623	1365
miR1874-3p	LOC_Os05g37500.1	167	-27.86	167	-27.86	761394	24	6524	477
miR1874-3p	LOC_Os05g37520.1	173	-26.7	173	-26.7	761396	24	2127	145
miR1874-3p	LOC_Os05g37520.2	173	-26.7	173	-26.7	761397	24	1999	145
miR1874-3p	LOC_Os05g37820.1	161	-23.12	161	-23.12	761428	24	2087	842
miR1874-3p	LOC_Os05g38150.1	151	-20.01	151	-20.01	761470	24	2985	1027
miR1874-3p	LOC_Os05g38150.2	151	-20.01	151	-20.01	761471	24	2989	1031
miR1874-3p	LOC_Os05g38790.1	158	-25.73	158	-25.73	761562	24	2041	1024
miR1874-3p	LOC_Os05g38920.1	169	-28.72	169	-28.72	761577	24	1824	1374

miR1874-3p	LOC_Os05g38980.1	154	-20.32	154	-20.32	761583	24	3094	903
miR1874-3p	LOC_Os05g39080.1	143	-20.49	143	-20.49	761597	24	2509	1719
miR1874-3p	LOC_Os05g39240.1	161	-24.06	161	-24.06	761617	24	2032	35
miR1874-3p	LOC_Os05g39530.1	148	-23.36	148	-23.36	761651	24	1474	769
miR1874-3p	LOC_Os05g39530.2	148	-23.36	148	-23.36	761652	24	1465	760
miR1874-3p	LOC_Os05g39720.1	150	-20.1	150	-20.1	761674	24	2273	386
miR1874-3p	LOC_Os05g40080.1	152	-21.24	152	-21.24	761711	24	987	97
miR1874-3p	LOC_Os05g40090.1	171	-25.73	171	-25.73	761712	24	2361	2339
miR1874-3p	LOC_Os05g40110.1	158	-20.6	158	-20.6	761714	24	4845	3722
miR1874-3p	LOC_Os05g40270.1	311	-44.07	163	-22.86	761731	24	3817	1614 2884
miR1874-3p	LOC_Os05g40270.2	311	-44.07	163	-22.86	761732	24	3884	1681 2951
miR1874-3p	LOC_Os05g40270.3	311	-44.07	163	-22.86	761733	24	3838	1635 2905
miR1874-3p	LOC_Os05g40270.4	311	-44.07	163	-22.86	761734	24	4118	1915 3185
miR1874-3p	LOC_Os05g40270.5	311	-44.07	163	-22.86	761735	24	3887	1684 2954
miR1874-3p	LOC_Os05g40270.6	311	-44.07	163	-22.86	761736	24	3791	1588 2858
miR1874-3p	LOC_Os05g40270.7	311	-44.07	163	-22.86	761737	24	3689	1486 2756
miR1874-3p	LOC_Os05g40270.8	311	-44.07	163	-22.86	761738	24	3817	1684 2884
miR1874-3p	LOC_Os05g40270.9	311	-44.07	163	-22.86	761739	24	4106	1684 3173
miR1874-3p	LOC_Os05g40270.10	163	-22.86	163	-22.86	761740	24	2222	1684
miR1874-3p	LOC_Os05g40270.12	148	-21.21	148	-21.21	761741	24	1751	818
miR1874-3p	LOC_Os05g40280.1	148	-21.21	148	-21.21	761742	24	1852	118
miR1874-3p	LOC_Os05g40340.1	158	-22.94	158	-22.94	761752	24	612	396
miR1874-3p	LOC_Os05g40420.1	158	-23.25	158	-23.25	761759	24	1992	1800
miR1874-3p	LOC_Os05g40420.3	158	-23.25	158	-23.25	761761	24	1914	1725
miR1874-3p	LOC_Os05g40700.1	154	-22.34	154	-22.34	761788	24	1319	64
miR1874-3p	LOC_Os05g40890.2	171	-28.77	171	-28.77	761814	24	928	76
miR1874-3p	LOC_Os05g41200.1	156	-22.3	156	-22.3	761851	24	941	129
miR1874-3p	LOC_Os05g41560.1	154	-23.58	154	-23.58	761893	24	4062	3683
miR1874-3p	LOC_Os05g41795.1	153	-20.4	153	-20.4	761918	24	2062	1471
miR1874-3p	LOC_Os05g42030.1	154	-23.58	154	-23.58	761950	24	1839	1460
miR1874-3p	LOC_Os05g42436.1	169	-31.18	169	-31.18	761992	24	261	162
miR1874-3p	LOC_Os05g43252.1	156	-20.92	156	-20.92	762027	24	3747	3671

miR1874-3p	LOC_Os05g43252.2	156	-20.92	156	-20.92	762028	24	3781	3706
miR1874-3p	LOC_Os05g43570.1	167	-25.29	167	-25.29	762062	24	2632	507
miR1874-3p	LOC_Os05g43570.2	167	-25.29	167	-25.29	762063	24	2587	406
miR1874-3p	LOC_Os05g43570.3	167	-25.29	167	-25.29	762064	24	2591	406
miR1874-3p	LOC_Os05g44030.1	162	-23.79	162	-23.79	762120	24	945	726
miR1874-3p	LOC_Os05g44380.1	160	-25.98	160	-25.98	762168	24	1693	1322
miR1874-3p	LOC_Os05g44740.1	174	-25.84	174	-25.84	762210	24	3738	1637
miR1874-3p	LOC_Os05g44786.1	164	-23.13	164	-23.13	762215	24	4905	2107
miR1874-3p	LOC_Os05g44810.1	147	-25.52	147	-25.52	762221	24	1396	544
miR1874-3p	LOC_Os05g44810.2	147	-25.52	147	-25.52	762222	24	1353	544
miR1874-3p	LOC_Os05g44810.3	147	-25.52	147	-25.52	762223	24	1333	544
miR1874-3p	LOC_Os05g45210.1	147	-23.97	147	-23.97	762266	24	3529	1612
miR1874-3p	LOC_Os05g45210.2	147	-23.97	147	-23.97	762267	24	3646	1730
miR1874-3p	LOC_Os05g45210.3	147	-23.97	147	-23.97	762268	24	3738	1822
miR1874-3p	LOC_Os05g45400.1	313	-45.87	165	-24.63	762290	24	2125	1015 968
miR1874-3p	LOC_Os05g45690.1	156	-20.15	156	-20.15	762321	24	1569	673
miR1874-3p	LOC_Os05g45720.1	157	-21.17	157	-21.17	762324	24	3413	2477
miR1874-3p	LOC_Os05g45720.2	157	-21.17	157	-21.17	762325	24	3282	2346
miR1874-3p	LOC_Os05g45875.1	157	-20.03	157	-20.03	762347	24	1495	896
miR1874-3p	LOC_Os05g45875.2	157	-20.03	157	-20.03	762348	24	1613	1014
miR1874-3p	LOC_Os05g46220.1	148	-25.86	148	-25.86	762388	24	2175	2063
miR1874-3p	LOC_Os05g46230.1	148	-25.86	148	-25.86	762389	24	2753	1581
miR1874-3p	LOC_Os05g46240.1	157	-26.64	157	-26.64	762390	24	1163	92
miR1874-3p	LOC_Os05g46240.2	157	-26.64	157	-26.64	762391	24	1171	92
miR1874-3p	LOC_Os05g46240.3	157	-26.64	157	-26.64	762392	24	1290	92
miR1874-3p	LOC_Os05g46240.4	157	-26.64	157	-26.64	762393	24	1303	92
miR1874-3p	LOC_Os05g46260.1	156	-20.95	156	-20.95	762397	24	3603	169
miR1874-3p	LOC_Os05g46580.1	150	-21.08	150	-21.08	762440	24	1674	1041
miR1874-3p	LOC_Os05g46690.1	150	-20.01	150	-20.01	762452	24	2142	296
miR1874-3p	LOC_Os05g46700.1	142	-23.41	142	-23.41	762453	24	1317	2
miR1874-3p	LOC_Os05g46750.1	295	-43.46	151	-22.88	762460	24	1842	605 1755
miR1874-3p	LOC_Os05g48030.1	152	-21.69	152	-21.69	762551	24	3047	1974

miR1874-3p	LOC_Os05g48200.1	298	-42.98	150	-22.42	762573	24	6926	4265 5971
miR1874-3p	LOC_Os05g48500.2	153	-22.04	153	-22.04	762617	24	3307	148
miR1874-3p	LOC_Os05g48590.1	162	-25.69	162	-25.69	762628	24	1574	926
miR1874-3p	LOC_Os05g48660.1	144	-20.38	144	-20.38	762637	24	2079	1173
miR1874-3p	LOC_Os05g48660.2	144	-20.38	144	-20.38	762638	24	2118	1173
miR1874-3p	LOC_Os05g48790.3	168	-25.41	168	-25.41	762654	24	2134	1058
miR1874-3p	LOC_Os05g49140.1	160	-22.44	160	-22.44	762706	24	2498	443
miR1874-3p	LOC_Os05g49140.2	160	-22.44	160	-22.44	762707	24	2377	443
miR1874-3p	LOC_Os05g49140.3	160	-22.44	160	-22.44	762708	24	2596	443
miR1874-3p	LOC_Os05g49164.1	501	-85.23	169	-31.48	762713	24	1902	80 197 239
miR1874-3p	LOC_Os05g49200.1	156	-21.13	156	-21.13	762716	24	2254	1554
miR1874-3p	LOC_Os05g49200.2	156	-21.13	156	-21.13	762717	24	2672	1972
miR1874-3p	LOC_Os05g49210.1	156	-22.18	156	-22.18	762718	24	1878	837
miR1874-3p	LOC_Os05g49390.1	152	-24.79	152	-24.79	762741	24	1338	282
miR1874-3p	LOC_Os05g49600.1	169	-23.06	169	-23.06	762766	24	2925	24
miR1874-3p	LOC_Os05g49720.1	157	-22.53	157	-22.53	762781	24	1299	760
miR1874-3p	LOC_Os05g49810.1	159	-24.13	159	-24.13	762793	24	1137	1114
miR1874-3p	LOC_Os05g49820.1	159	-24.13	159	-24.13	762794	24	2799	313
miR1874-3p	LOC_Os05g49820.2	159	-24.13	159	-24.13	762795	24	2900	313
miR1874-3p	LOC_Os05g50050.1	158	-21.78	158	-21.78	762826	24	570	56
miR1874-3p	LOC_Os05g50060.1	169	-28.72	169	-28.72	762827	24	2559	2196
miR1874-3p	LOC_Os05g50770.2	161	-21.02	161	-21.02	762909	24	2241	37
miR1874-3p	LOC_Os05g50990.1	159	-21.95	159	-21.95	762940	24	3238	1918
miR1874-3p	LOC_Os05g51090.1	154	-21.47	154	-21.47	762953	24	1432	177
miR1874-3p	LOC_Os05g51180.1	162	-22.76	162	-22.76	762967	24	1717	1522
miR1874-3p	LOC_Os05g51350.1	151	-24.25	151	-24.25	762987	24	1362	16
miR1874-3p	LOC_Os05g51690.1	147	-20.31	147	-20.31	763039	24	1598	1473
miR1874-3p	LOC_Os05g51690.3	147	-20.31	147	-20.31	763041	24	1048	923
miR1874-3p	LOC_Os06g01230.2	161	-28.88	161	-28.88	763107	24	2308	465
miR1874-3p	LOC_Os06g01360.1	158	-24.68	158	-24.68	763121	24	2203	184
miR1874-3p	LOC_Os06g01360.2	158	-24.68	158	-24.68	763122	24	2271	184
miR1874-3p	LOC_Os06g01500.1	165	-27.07	165	-27.07	763135	24	1877	1823

miR1874-3p	LOC_Os06g01500.2	165	-27.07	165	-27.07	763136	24	1775	1721
miR1874-3p	LOC_Os06g01500.3	165	-27.07	165	-27.07	763137	24	1787	1733
miR1874-3p	LOC_Os06g01630.1	167	-24.66	167	-24.66	763149	24	2195	472
miR1874-3p	LOC_Os06g01630.2	167	-24.66	167	-24.66	763150	24	2264	472
miR1874-3p	LOC_Os06g01630.3	167	-24.66	167	-24.66	763151	24	2019	316
miR1874-3p	LOC_Os06g01630.5	167	-24.66	167	-24.66	763152	24	2243	472
miR1874-3p	LOC_Os06g01630.6	167	-24.66	167	-24.66	763153	24	2221	316
miR1874-3p	LOC_Os06g01680.1	165	-21.14	165	-21.14	763159	24	4059	218
miR1874-3p	LOC_Os06g01680.2	165	-21.14	165	-21.14	763160	24	4496	215
miR1874-3p	LOC_Os06g01680.3	165	-21.14	165	-21.14	763161	24	4050	218
miR1874-3p	LOC_Os06g01680.4	165	-21.14	165	-21.14	763162	24	4567	218
miR1874-3p	LOC_Os06g01740.1	145	-21.44	145	-21.44	763168	24	1817	1601
miR1874-3p	LOC_Os06g02160.1	159	-25.37	159	-25.37	763213	24	4193	150
miR1874-3p	LOC_Os06g02160.2	159	-25.37	159	-25.37	763214	24	4110	150
miR1874-3p	LOC_Os06g02160.3	159	-25.37	159	-25.37	763215	24	4102	150
miR1874-3p	LOC_Os06g02160.4	159	-25.37	159	-25.37	763216	24	4118	150
miR1874-3p	LOC_Os06g02304.1	154	-21.23	154	-21.23	763232	24	3543	1664
miR1874-3p	LOC_Os06g02400.1	165	-27.43	165	-27.43	763243	24	2810	2237
miR1874-3p	LOC_Os06g02430.1	166	-30.11	166	-30.11	763246	24	1729	1108
miR1874-3p	LOC_Os06g02560.1	169	-27.17	169	-27.17	763266	24	1703	505
miR1874-3p	LOC_Os06g02560.2	169	-27.17	169	-27.17	763267	24	1540	342
miR1874-3p	LOC_Os06g02560.3	169	-27.17	169	-27.17	763268	24	1703	485
miR1874-3p	LOC_Os06g02630.1	160	-24.04	160	-24.04	763275	24	1877	1307
miR1874-3p	LOC_Os06g02700.1	165	-22.14	165	-22.14	763282	24	1011	951
miR1874-3p	LOC_Os06g03040.1	160	-20.9	160	-20.9	763313	24	657	415
miR1874-3p	LOC_Os06g03270.1	149	-21.68	149	-21.68	763336	24	624	205
miR1874-3p	LOC_Os06g03440.1	158	-21.02	158	-21.02	763353	24	613	184
miR1874-3p	LOC_Os06g03474.1	158	-21.02	158	-21.02	763356	24	3933	3427
miR1874-3p	LOC_Os06g03500.1	148	-20.78	148	-20.78	763358	24	3024	2467
miR1874-3p	LOC_Os06g03640.1	157	-21.37	157	-21.37	763379	24	2110	64
miR1874-3p	LOC_Os06g04000.1	150	-23.66	150	-23.66	763424	24	845	428
miR1874-3p	LOC_Os06g04360.1	157	-23.56	157	-23.56	763467	24	2101	106

miR1874-3p	LOC_Os06g04760.1	146	-20.76	146	-20.76	763518	24	432	130
miR1874-3p	LOC_Os06g04840.1	158	-25.61	158	-25.61	763526	24	3516	1854
miR1874-3p	LOC_Os06g04840.2	158	-25.61	158	-25.61	763527	24	3505	1854
miR1874-3p	LOC_Os06g04920.1	152	-21.56	152	-21.56	763535	24	1304	205
miR1874-3p	LOC_Os06g04920.2	152	-21.56	152	-21.56	763536	24	1348	320
miR1874-3p	LOC_Os06g04980.1	178	-23.91	178	-23.91	763543	24	1539	946
miR1874-3p	LOC_Os06g05050.1	145	-20.32	145	-20.32	763552	24	2535	2062
miR1874-3p	LOC_Os06g05100.1	152	-21.66	152	-21.66	763558	24	3297	2485
miR1874-3p	LOC_Os06g05100.2	152	-21.66	152	-21.66	763559	24	3279	2485
miR1874-3p	LOC_Os06g05100.3	152	-21.66	152	-21.66	763560	24	2117	1305
miR1874-3p	LOC_Os06g05100.4	152	-21.66	152	-21.66	763561	24	3319	2507
miR1874-3p	LOC_Os06g05100.5	152	-21.66	152	-21.66	763562	24	3469	2666
miR1874-3p	LOC_Os06g05359.1	157	-21.23	157	-21.23	763592	24	4325	3536
miR1874-3p	LOC_Os06g06400.1	159	-20.77	159	-20.77	763737	24	4985	2434
miR1874-3p	LOC_Os06g06440.1	151	-20.94	151	-20.94	763743	24	4814	1992
miR1874-3p	LOC_Os06g06470.1	165	-21.92	165	-21.92	763746	24	1638	41
miR1874-3p	LOC_Os06g06520.1	158	-20.06	158	-20.06	763752	24	1484	710
miR1874-3p	LOC_Os06g06560.1	160	-21.54	160	-21.54	763756	24	2860	2283
miR1874-3p	LOC_Os06g06620.1	151	-20.68	151	-20.68	763763	24	306	283
miR1874-3p	LOC_Os06g06760.1	159	-21.68	159	-21.68	763777	24	3388	2215
miR1874-3p	LOC_Os06g06760.2	159	-21.68	159	-21.68	763778	24	3165	1992
miR1874-3p	LOC_Os06g06760.3	159	-21.68	159	-21.68	763779	24	3015	1842
miR1874-3p	LOC_Os06g06840.1	156	-20.06	156	-20.06	763788	24	2961	180
miR1874-3p	LOC_Os06g06880.1	152	-22.78	152	-22.78	763792	24	1854	1676
miR1874-3p	LOC_Os06g07080.1	162	-20.42	162	-20.42	763815	24	3710	775
miR1874-3p	LOC_Os06g07230.1	166	-21.93	166	-21.93	763833	24	1977	684
miR1874-3p	LOC_Os06g07420.1	145	-21.85	145	-21.85	763851	24	2625	21
miR1874-3p	LOC_Os06g07474.1	153	-21.54	153	-21.54	763857	24	1924	128
miR1874-3p	LOC_Os06g07474.2	153	-21.54	153	-21.54	763858	24	1935	128
miR1874-3p	LOC_Os06g07720.1	156	-21.34	156	-21.34	763884	24	4695	2105
miR1874-3p	LOC_Os06g08400.1	147	-23.46	147	-23.46	763952	24	2176	901
miR1874-3p	LOC_Os06g08400.2	147	-23.46	147	-23.46	763953	24	2506	1277

miR1874-3p	LOC_Os06g08884.1	165	-21	165	-21	764018	24	1726	580
miR1874-3p	LOC_Os06g08884.2	165	-21	165	-21	764019	24	979	580
miR1874-3p	LOC_Os06g09450.1	147	-20.76	147	-20.76	764085	24	3191	301
miR1874-3p	LOC_Os06g09450.2	147	-20.76	147	-20.76	764086	24	3816	301
miR1874-3p	LOC_Os06g09450.3	147	-20.76	147	-20.76	764087	24	3122	301
miR1874-3p	LOC_Os06g09450.4	147	-20.76	147	-20.76	764088	24	3634	301
miR1874-3p	LOC_Os06g09450.5	147	-20.76	147	-20.76	764089	24	3169	301
miR1874-3p	LOC_Os06g09450.6	147	-20.76	147	-20.76	764090	24	3219	301
miR1874-3p	LOC_Os06g09450.7	147	-20.76	147	-20.76	764091	24	3216	301
miR1874-3p	LOC_Os06g09450.8	147	-20.76	147	-20.76	764092	24	3288	301
miR1874-3p	LOC_Os06g09620.1	151	-22.77	151	-22.77	764108	24	2213	476
miR1874-3p	LOC_Os06g09980.1	163	-22.6	163	-22.6	764151	24	1082	632
miR1874-3p	LOC_Os06g10280.1	147	-20.08	147	-20.08	764184	24	1768	1260
miR1874-3p	LOC_Os06g10400.1	162	-29.77	162	-29.77	764197	24	375	296
miR1874-3p	LOC_Os06g11380.1	155	-20.38	155	-20.38	764303	24	2071	1165
miR1874-3p	LOC_Os06g11830.1	144	-21.38	144	-21.38	764354	24	2640	1279
miR1874-3p	LOC_Os06g11970.1	157	-21.52	157	-21.52	764369	24	1396	509
miR1874-3p	LOC_Os06g12170.1	157	-24.78	157	-24.78	764393	24	3403	17
miR1874-3p	LOC_Os06g12170.3	157	-24.78	157	-24.78	764395	24	1545	14
miR1874-3p	LOC_Os06g12170.4	157	-24.78	157	-24.78	764396	24	3029	123
miR1874-3p	LOC_Os06g12170.5	157	-24.78	157	-24.78	764397	24	2927	17
miR1874-3p	LOC_Os06g12170.6	157	-24.78	157	-24.78	764398	24	2056	6
miR1874-3p	LOC_Os06g12170.7	157	-24.78	157	-24.78	764399	24	3399	17
miR1874-3p	LOC_Os06g12170.8	157	-24.78	157	-24.78	764400	24	1807	14
miR1874-3p	LOC_Os06g12680.1	152	-20.24	152	-20.24	764458	24	1783	225
miR1874-3p	LOC_Os06g12740.1	158	-23.04	158	-23.04	764466	24	1606	1296
miR1874-3p	LOC_Os06g12890.1	172	-29.95	172	-29.95	764482	24	735	218
miR1874-3p	LOC_Os06g13160.1	149	-20.81	149	-20.81	764514	24	1431	664
miR1874-3p	LOC_Os06g13560.1	156	-20.81	156	-20.81	764553	24	1529	925
miR1874-3p	LOC_Os06g13560.2	156	-20.81	156	-20.81	764554	24	1523	925
miR1874-3p	LOC_Os06g13560.3	156	-20.81	156	-20.81	764555	24	1735	925
miR1874-3p	LOC_Os06g13560.4	156	-20.81	156	-20.81	764556	24	1338	925

miR1874-3p	LOC_Os06g13560.5	156	-20.81	156	-20.81	764557	24	1637	1033
miR1874-3p	LOC_Os06g13950.1	160	-26.32	160	-26.32	764600	24	1815	339
miR1874-3p	LOC_Os06g14110.1	157	-23.3	157	-23.3	764617	24	2751	110
miR1874-3p	LOC_Os06g14400.1	154	-22.06	154	-22.06	764649	24	1498	49
miR1874-3p	LOC_Os06g14740.1	160	-22.67	160	-22.67	764691	24	4664	750
miR1874-3p	LOC_Os06g15050.1	152	-22.83	152	-22.83	764720	24	1782	485
miR1874-3p	LOC_Os06g15090.1	161	-20.93	161	-20.93	764724	24	1692	770
miR1874-3p	LOC_Os06g15120.1	154	-22.42	154	-22.42	764727	24	282	254
miR1874-3p	LOC_Os06g15310.1	161	-23.47	161	-23.47	764744	24	4740	2015
miR1874-3p	LOC_Os06g15480.1	183	-31.31	183	-31.31	764764	24	1583	200
miR1874-3p	LOC_Os06g15480.2	183	-31.31	183	-31.31	764765	24	1565	200
miR1874-3p	LOC_Os06g15700.1	157	-22.67	157	-22.67	764787	24	6494	2368
miR1874-3p	LOC_Os06g16010.1	169	-28.72	169	-28.72	764816	24	2712	2277
miR1874-3p	LOC_Os06g16160.1	168	-24.43	168	-24.43	764831	24	2739	1501
miR1874-3p	LOC_Os06g16600.1	153	-20.72	153	-20.72	764880	24	1188	1016
miR1874-3p	LOC_Os06g16690.1	169	-28.72	169	-28.72	764888	24	2631	2196
miR1874-3p	LOC_Os06g17440.1	172	-28.54	172	-28.54	764964	24	1285	24
miR1874-3p	LOC_Os06g17629.1	149	-21.68	149	-21.68	764985	24	489	167
miR1874-3p	LOC_Os06g17750.1	154	-22.07	154	-22.07	764994	24	5418	5396
miR1874-3p	LOC_Os06g17840.1	148	-20.95	148	-20.95	765002	24	5406	2406
miR1874-3p	LOC_Os06g17900.1	153	-21.66	153	-21.66	765008	24	2862	1589
miR1874-3p	LOC_Os06g17920.1	150	-21.91	150	-21.91	765010	24	3003	1566
miR1874-3p	LOC_Os06g18000.1	153	-21.18	153	-21.18	765017	24	1930	32
miR1874-3p	LOC_Os06g18030.1	156	-25.26	156	-25.26	765020	24	2565	2013
miR1874-3p	LOC_Os06g18800.1	158	-21.02	158	-21.02	765046	24	5229	4609
miR1874-3p	LOC_Os06g18820.2	165	-23.96	165	-23.96	765049	24	2715	1438
miR1874-3p	LOC_Os06g18950.1	159	-24.24	159	-24.24	765062	24	5535	2702
miR1874-3p	LOC_Os06g18970.1	162	-23.59	162	-23.59	765064	24	1995	1973
miR1874-3p	LOC_Os06g19130.1	146	-21.56	146	-21.56	765081	24	3261	2403
miR1874-3p	LOC_Os06g19160.1	160	-20.49	160	-20.49	765084	24	2685	1297
miR1874-3p	LOC_Os06g19310.1	154	-22.07	154	-22.07	765095	24	3207	3185
miR1874-3p	LOC_Os06g19330.1	154	-22.07	154	-22.07	765097	24	1584	1562

miR1874-3p	LOC_Os06g19350.1	140	-20.5	140	-20.5	765099	24	3882	670
miR1874-3p	LOC_Os06g19410.1	164	-20.64	164	-20.64	765106	24	987	509
miR1874-3p	LOC_Os06g19420.1	149	-23.42	149	-23.42	765107	24	372	136
miR1874-3p	LOC_Os06g19470.1	151	-20.17	151	-20.17	765112	24	3094	2147
miR1874-3p	LOC_Os06g19470.2	151	-20.17	151	-20.17	765113	24	2968	2147
miR1874-3p	LOC_Os06g19470.3	151	-20.17	151	-20.17	765114	24	3253	2432
miR1874-3p	LOC_Os06g19470.4	151	-20.17	151	-20.17	765115	24	2793	2432
miR1874-3p	LOC_Os06g20250.1	148	-20.99	148	-20.99	765196	24	636	280
miR1874-3p	LOC_Os06g20390.1	159	-20.67	159	-20.67	765213	24	2009	131
miR1874-3p	LOC_Os06g20500.1	146	-20.15	146	-20.15	765229	24	2490	65
miR1874-3p	LOC_Os06g20580.1	150	-20.49	150	-20.49	765235	24	2604	882
miR1874-3p	LOC_Os06g20590.1	161	-20.42	161	-20.42	765236	24	4575	2000
miR1874-3p	LOC_Os06g21059.1	158	-21.02	158	-21.02	765283	24	4422	364
miR1874-3p	LOC_Os06g21068.1	158	-21.02	158	-21.02	765284	24	870	364
miR1874-3p	LOC_Os06g21140.1	151	-22.17	151	-22.17	765292	24	849	600
miR1874-3p	LOC_Os06g21260.1	156	-20.12	156	-20.12	765304	24	4116	3174
miR1874-3p	LOC_Os06g21570.1	145	-21.11	145	-21.11	765339	24	1515	89
miR1874-3p	LOC_Os06g21610.1	154	-23.58	154	-23.58	765344	24	4341	3962
miR1874-3p	LOC_Os06g21620.2	161	-20.99	161	-20.99	765346	24	1361	275
miR1874-3p	LOC_Os06g21740.1	165	-24.54	165	-24.54	765359	24	3981	1529
miR1874-3p	LOC_Os06g22040.1	148	-21.44	148	-21.44	765391	24	543	89
miR1874-3p	LOC_Os06g22050.1	162	-22.18	162	-22.18	765392	24	1133	421
miR1874-3p	LOC_Os06g22230.1	164	-26.24	164	-26.24	765416	24	4293	2420
miR1874-3p	LOC_Os06g22520.1	156	-21.34	156	-21.34	765447	24	5496	2561
miR1874-3p	LOC_Os06g22810.1	156	-25.58	156	-25.58	765477	24	3193	2588
miR1874-3p	LOC_Os06g22829.1	145	-20.24	145	-20.24	765479	24	2709	1430
miR1874-3p	LOC_Os06g22840.1	170	-24.22	170	-24.22	765482	24	642	58
miR1874-3p	LOC_Os06g23230.1	154	-23.58	154	-23.58	765522	24	4530	3866
miR1874-3p	LOC_Os06g23800.2	157	-20.51	157	-20.51	765583	24	1210	314
miR1874-3p	LOC_Os06g23940.1	169	-28.72	169	-28.72	765598	24	1926	1740
miR1874-3p	LOC_Os06g24370.1	144	-22.93	144	-22.93	765638	24	951	520
miR1874-3p	LOC_Os06g25190.1	162	-20.65	162	-20.65	765722	24	384	181

miR1874-3p	LOC_Os06g25320.1	146	-22.4	146	-22.4	765736	24	3660	3295
miR1874-3p	LOC_Os06g25510.1	154	-21.93	154	-21.93	765750	24	348	236
miR1874-3p	LOC_Os06g25605.1	158	-20.06	158	-20.06	765760	24	2519	2197
miR1874-3p	LOC_Os06g25710.1	156	-20.7	156	-20.7	765771	24	2151	1634
miR1874-3p	LOC_Os06g25800.1	156	-21.84	156	-21.84	765780	24	3346	21
miR1874-3p	LOC_Os06g25840.1	160	-25.36	160	-25.36	765784	24	2694	1748
miR1874-3p	LOC_Os06g26150.1	160	-21.61	160	-21.61	765815	24	4839	1793
miR1874-3p	LOC_Os06g26170.1	154	-22.9	154	-22.9	765817	24	1077	764
miR1874-3p	LOC_Os06g27460.1	159	-24.95	159	-24.95	765846	24	2985	1943
miR1874-3p	LOC_Os06g27540.1	159	-24.23	159	-24.23	765854	24	3210	633
miR1874-3p	LOC_Os06g27830.1	156	-20.94	156	-20.94	765885	24	1867	1460
miR1874-3p	LOC_Os06g27880.1	151	-20.14	151	-20.14	765890	24	5580	250
miR1874-3p	LOC_Os06g27890.1	153	-20.33	153	-20.33	765891	24	2340	1525
miR1874-3p	LOC_Os06g27890.2	153	-20.33	153	-20.33	765892	24	2283	1508
miR1874-3p	LOC_Os06g27890.3	153	-20.33	153	-20.33	765893	24	2305	1525
miR1874-3p	LOC_Os06g27890.4	153	-20.33	153	-20.33	765894	24	3122	2342
miR1874-3p	LOC_Os06g28430.1	150	-21.67	150	-21.67	765952	24	699	294
miR1874-3p	LOC_Os06g28440.1	155	-24.85	155	-24.85	765953	24	483	8
miR1874-3p	LOC_Os06g28450.1	143	-21.07	143	-21.07	765954	24	2214	970
miR1874-3p	LOC_Os06g28790.1	149	-20.41	149	-20.41	765988	24	810	788
miR1874-3p	LOC_Os06g28820.1	160	-21.69	160	-21.69	765990	24	5189	3631
miR1874-3p	LOC_Os06g29050.1	163	-23.56	163	-23.56	766016	24	3432	1041
miR1874-3p	LOC_Os06g29080.1	157	-20.86	157	-20.86	766019	24	3011	1708
miR1874-3p	LOC_Os06g29270.1	158	-20.18	158	-20.18	766041	24	3801	3005
miR1874-3p	LOC_Os06g29650.1	157	-21.46	157	-21.46	766080	24	1439	119
miR1874-3p	LOC_Os06g29650.2	157	-21.46	157	-21.46	766081	24	1355	119
miR1874-3p	LOC_Os06g29679.1	163	-26.3	163	-26.3	766084	24	758	396
miR1874-3p	LOC_Os06g31020.1	154	-22.07	154	-22.07	766225	24	5430	5408
miR1874-3p	LOC_Os06g31200.1	155	-21.97	155	-21.97	766243	24	1938	1101
miR1874-3p	LOC_Os06g32090.1	154	-22.42	154	-22.42	766326	24	2226	2198
miR1874-3p	LOC_Os06g32130.1	154	-22.07	154	-22.07	766330	24	690	668
miR1874-3p	LOC_Os06g32470.1	156	-20.7	156	-20.7	766363	24	4302	3983

miR1874-3p	LOC_Os06g32490.1	162	-23.96	162	-23.96	766365	24	4983	4477
miR1874-3p	LOC_Os06g32760.1	169	-28.72	169	-28.72	766388	24	2238	1803
miR1874-3p	LOC_Os06g32820.1	154	-22.07	154	-22.07	766394	24	4506	4484
miR1874-3p	LOC_Os06g33070.1	169	-28.72	169	-28.72	766418	24	2649	2106
miR1874-3p	LOC_Os06g33250.1	164	-20.11	164	-20.11	766436	24	2439	483
miR1874-3p	LOC_Os06g33360.1	152	-20.8	152	-20.8	766448	24	3060	2812
miR1874-3p	LOC_Os06g33370.1	152	-20.8	152	-20.8	766449	24	1283	215
miR1874-3p	LOC_Os06g33450.1	150	-22.51	150	-22.51	766457	24	3123	631
miR1874-3p	LOC_Os06g33450.2	150	-22.51	150	-22.51	766458	24	1615	631
miR1874-3p	LOC_Os06g33960.1	151	-24.57	151	-24.57	766508	24	801	371
miR1874-3p	LOC_Os06g34090.1	151	-20.89	151	-20.89	766521	24	4317	1400
miR1874-3p	LOC_Os06g34410.1	166	-28.05	166	-28.05	766551	24	288	16
miR1874-3p	LOC_Os06g34510.1	155	-20.27	155	-20.27	766561	24	2790	2758
miR1874-3p	LOC_Os06g34590.1	160	-20.98	160	-20.98	766570	24	1293	449
miR1874-3p	LOC_Os06g34920.1	162	-24.09	162	-24.09	766601	24	738	493
miR1874-3p	LOC_Os06g35000.1	144	-21.71	144	-21.71	766609	24	3057	295
miR1874-3p	LOC_Os06g35080.1	146	-20.14	146	-20.14	766619	24	1050	407
miR1874-3p	LOC_Os06g35430.1	156	-20.49	156	-20.49	766654	24	2442	280
miR1874-3p	LOC_Os06g35530.1	163	-22.37	163	-22.37	766665	24	2031	1766
miR1874-3p	LOC_Os06g35970.1	155	-20.88	155	-20.88	766709	24	1174	314
miR1874-3p	LOC_Os06g35970.2	155	-20.88	155	-20.88	766710	24	1078	314
miR1874-3p	LOC_Os06g36070.1	158	-20.48	158	-20.48	766720	24	1414	307
miR1874-3p	LOC_Os06g36090.1	163	-21.69	163	-21.69	766722	24	4330	3636
miR1874-3p	LOC_Os06g36090.2	163	-21.69	163	-21.69	766723	24	4312	3618
miR1874-3p	LOC_Os06g36180.1	155	-23.53	155	-23.53	766731	24	2367	835
miR1874-3p	LOC_Os06g36480.1	154	-20.48	154	-20.48	766766	24	2040	33
miR1874-3p	LOC_Os06g36530.1	177	-26.23	177	-26.23	766771	24	2550	604
miR1874-3p	LOC_Os06g36550.1	158	-21.02	158	-21.02	766773	24	873	367
miR1874-3p	LOC_Os06g36650.1	146	-20.84	146	-20.84	766783	24	3588	1205
miR1874-3p	LOC_Os06g36770.1	156	-27.79	156	-27.79	766795	24	2479	124
miR1874-3p	LOC_Os06g36770.2	156	-27.79	156	-27.79	766796	24	2476	124
miR1874-3p	LOC_Os06g37440.1	158	-22.2	158	-22.2	766867	24	1831	532

miR1874-3p	LOC_Os06g37760.1	164	-20.13	164	-20.13	766900	24	2064	1774
miR1874-3p	LOC_Os06g37970.1	154	-23.58	154	-23.58	766917	24	891	662
miR1874-3p	LOC_Os06g38062.1	156	-23.08	156	-23.08	766926	24	2808	1518
miR1874-3p	LOC_Os06g38080.1	145	-20.82	145	-20.82	766928	24	2126	401
miR1874-3p	LOC_Os06g38240.1	166	-25.15	166	-25.15	766945	24	3117	858
miR1874-3p	LOC_Os06g38320.1	141	-21.87	141	-21.87	766951	24	1355	311
miR1874-3p	LOC_Os06g38320.2	141	-21.87	141	-21.87	766952	24	1920	311
miR1874-3p	LOC_Os06g38320.3	141	-21.87	141	-21.87	766953	24	1801	311
miR1874-3p	LOC_Os06g38380.1	156	-29.72	156	-29.72	766959	24	372	203
miR1874-3p	LOC_Os06g38390.1	154	-30.7	154	-30.7	766960	24	375	205
miR1874-3p	LOC_Os06g38730.1	158	-20.08	158	-20.08	766996	24	3009	1057
miR1874-3p	LOC_Os06g39090.1	304	-42.26	156	-21.28	767034	24	3573	2820 559
miR1874-3p	LOC_Os06g39640.1	151	-24.52	151	-24.52	767092	24	3193	2109
miR1874-3p	LOC_Os06g39790.1	159	-22.78	159	-22.78	767118	24	2985	814
miR1874-3p	LOC_Os06g39880.1	142	-26.71	142	-26.71	767128	24	2151	1347
miR1874-3p	LOC_Os06g39940.1	141	-20.71	141	-20.71	767138	24	3201	2952
miR1874-3p	LOC_Os06g39970.1	145	-23.18	145	-23.18	767141	24	2583	1553
miR1874-3p	LOC_Os06g40030.1	157	-22.31	157	-22.31	767147	24	2807	1896
miR1874-3p	LOC_Os06g40220.1	144	-20.59	144	-20.59	767169	24	1470	1003
miR1874-3p	LOC_Os06g40490.1	164	-20.87	164	-20.87	767197	24	2003	145
miR1874-3p	LOC_Os06g40600.1	321	-45.64	167	-23.03	767208	24	3563	1657 98
miR1874-3p	LOC_Os06g40630.1	153	-21.46	153	-21.46	767212	24	1190	801
miR1874-3p	LOC_Os06g40680.1	162	-21.98	162	-21.98	767223	24	3486	1731
miR1874-3p	LOC_Os06g40940.1	163	-22.21	163	-22.21	767252	24	3559	1183
miR1874-3p	LOC_Os06g40940.2	163	-22.21	163	-22.21	767253	24	3142	766
miR1874-3p	LOC_Os06g40940.3	163	-22.21	163	-22.21	767254	24	2245	1183
miR1874-3p	LOC_Os06g41100.1	173	-30.1	173	-30.1	767271	24	1892	1321
miR1874-3p	LOC_Os06g41390.1	155	-22.4	155	-22.4	767303	24	1539	71
miR1874-3p	LOC_Os06g41390.2	155	-22.4	155	-22.4	767304	24	1539	71
miR1874-3p	LOC_Os06g41390.3	155	-22.4	155	-22.4	767305	24	1576	71
miR1874-3p	LOC_Os06g41390.4	155	-22.4	155	-22.4	767306	24	2037	72
miR1874-3p	LOC_Os06g41470.1	169	-28.72	169	-28.72	767311	24	2529	2094

miR1874-3p	LOC_Os06g41570.1	140	-21.56	140	-21.56	767320	24	483	10
miR1874-3p	LOC_Os06g41690.1	165	-22.54	165	-22.54	767334	24	7773	7435
miR1874-3p	LOC_Os06g41750.1	154	-22.45	154	-22.45	767341	24	2554	387
miR1874-3p	LOC_Os06g41780.1	156	-20.42	156	-20.42	767344	24	2693	1675
miR1874-3p	LOC_Os06g41780.2	156	-20.42	156	-20.42	767345	24	3440	1675
miR1874-3p	LOC_Os06g41930.1	167	-25.7	167	-25.7	767363	24	1852	60
miR1874-3p	LOC_Os06g41930.3	167	-25.7	167	-25.7	767365	24	1933	77
miR1874-3p	LOC_Os06g41930.4	167	-25.7	167	-25.7	767366	24	2152	76
miR1874-3p	LOC_Os06g41990.1	165	-25.75	165	-25.75	767373	24	4292	2245
miR1874-3p	LOC_Os06g41990.2	165	-25.75	165	-25.75	767374	24	4385	2338
miR1874-3p	LOC_Os06g42020.1	169	-30.89	169	-30.89	767377	24	2147	1281
miR1874-3p	LOC_Os06g42030.1	169	-28.63	169	-28.63	767378	24	5864	1501
miR1874-3p	LOC_Os06g42100.1	158	-25.28	158	-25.28	767386	24	459	181
miR1874-3p	LOC_Os06g42200.1	156	-20.06	156	-20.06	767397	24	3165	180
miR1874-3p	LOC_Os06g42310.1	172	-28.71	172	-28.71	767409	24	2430	1301
miR1874-3p	LOC_Os06g42490.1	161	-24.51	161	-24.51	767429	24	3137	2497
miR1874-3p	LOC_Os06g42770.1	156	-23.97	156	-23.97	767463	24	2712	895
miR1874-3p	LOC_Os06g42800.1	171	-28.74	171	-28.74	767468	24	2844	601
miR1874-3p	LOC_Os06g43750.1	151	-23.91	151	-23.91	767570	24	696	641
miR1874-3p	LOC_Os06g44440.1	157	-23.3	157	-23.3	767653	24	633	385
miR1874-3p	LOC_Os06g44930.1	148	-26.12	148	-26.12	767703	24	1060	626
miR1874-3p	LOC_Os06g45000.1	163	-21.15	163	-21.15	767711	24	1609	888
miR1874-3p	LOC_Os06g45000.2	163	-21.15	163	-21.15	767712	24	1578	857
miR1874-3p	LOC_Os06g45000.3	163	-21.15	163	-21.15	767713	24	1605	1039
miR1874-3p	LOC_Os06g45130.1	170	-27.27	170	-27.27	767729	24	204	164
miR1874-3p	LOC_Os06g45510.1	154	-21.29	154	-21.29	767781	24	1158	784
miR1874-3p	LOC_Os06g45510.2	154	-21.29	154	-21.29	767782	24	1158	784
miR1874-3p	LOC_Os06g45510.3	154	-21.29	154	-21.29	767783	24	1270	896
miR1874-3p	LOC_Os06g45610.1	156	-20.9	156	-20.9	767793	24	825	140
miR1874-3p	LOC_Os06g46250.1	155	-24.16	155	-24.16	767866	24	2354	1287
miR1874-3p	LOC_Os06g46500.1	163	-23.51	163	-23.51	767899	24	2118	603
miR1874-3p	LOC_Os06g46720.1	317	-46.66	166	-24.61	767924	24	4410	451 2342

miR1874-3p	LOC_Os06g47150.1	149	-21.8	149	-21.8	767981	24	3224	566
miR1874-3p	LOC_Os06g47150.2	149	-21.8	149	-21.8	767982	24	3026	368
miR1874-3p	LOC_Os06g47150.3	149	-21.8	149	-21.8	767983	24	2993	335
miR1874-3p	LOC_Os06g47150.4	149	-21.8	149	-21.8	767984	24	3562	368
miR1874-3p	LOC_Os06g47170.1	149	-20.12	149	-20.12	767986	24	2676	1174
miR1874-3p	LOC_Os06g47370.1	156	-20.49	156	-20.49	768013	24	2502	280
miR1874-3p	LOC_Os06g47470.1	154	-23.22	154	-23.22	768023	24	1740	1213
miR1874-3p	LOC_Os06g47800.1	150	-20.68	150	-20.68	768059	24	2955	1536
miR1874-3p	LOC_Os06g47820.1	145	-20.78	145	-20.78	768061	24	1568	1204
miR1874-3p	LOC_Os06g48100.1	156	-20.06	156	-20.06	768099	24	3048	180
miR1874-3p	LOC_Os06g48250.1	160	-23.61	160	-23.61	768117	24	1937	86
miR1874-3p	LOC_Os06g48350.1	152	-25.47	152	-25.47	768128	24	2193	1437
miR1874-3p	LOC_Os06g48355.1	152	-25.47	152	-25.47	768129	24	1353	1029
miR1874-3p	LOC_Os06g48460.1	156	-20.49	156	-20.49	768140	24	2481	280
miR1874-3p	LOC_Os06g48770.1	154	-20.89	154	-20.89	768181	24	3470	2432
miR1874-3p	LOC_Os06g48770.2	154	-20.89	154	-20.89	768182	24	3369	2342
miR1874-3p	LOC_Os06g48940.1	147	-23.92	147	-23.92	768197	24	3726	46
miR1874-3p	LOC_Os06g48960.1	149	-20.53	149	-20.53	768199	24	994	395
miR1874-3p	LOC_Os06g48990.1	146	-21.26	146	-21.26	768203	24	3409	2436
miR1874-3p	LOC_Os06g48990.2	146	-21.26	146	-21.26	768204	24	3451	2478
miR1874-3p	LOC_Os06g49810.1	168	-23.33	168	-23.33	768324	24	2286	1791
miR1874-3p	LOC_Os06g49860.1	159	-27.58	159	-27.58	768331	24	3164	1506
miR1874-3p	LOC_Os06g49900.1	149	-22.12	149	-22.12	768336	24	1278	578
miR1874-3p	LOC_Os06g49960.1	156	-20.06	156	-20.06	768344	24	3153	180
miR1874-3p	LOC_Os06g49980.1	146	-20.21	146	-20.21	768346	24	1116	696
miR1874-3p	LOC_Os06g50130.1	149	-24.54	149	-24.54	768365	24	1061	98
miR1874-3p	LOC_Os06g50310.1	160	-25.29	160	-25.29	768384	24	1551	1217
miR1874-3p	LOC_Os06g50520.1	141	-20.77	141	-20.77	768410	24	2263	568
miR1874-3p	LOC_Os06g50520.2	141	-20.77	141	-20.77	768411	24	2307	568
miR1874-3p	LOC_Os06g50620.1	157	-22.18	157	-22.18	768422	24	750	228
miR1874-3p	LOC_Os06g50770.1	157	-22.18	157	-22.18	768434	24	750	228
miR1874-3p	LOC_Os06g50860.1	146	-21.1	146	-21.1	768444	24	1808	1203

miR1874-3p	LOC_Os06g50860.2	146	-21.1	146	-21.1	768445	24	1892	1203
miR1874-3p	LOC_Os06g50910.1	323	-42.68	164	-22.29	768449	24	8483	3224 5092
miR1874-3p	LOC_Os06g50910.2	323	-42.68	164	-22.29	768450	24	8236	2977 4845
miR1874-3p	LOC_Os06g50910.3	323	-42.68	164	-22.29	768451	24	8081	2822 4690
miR1874-3p	LOC_Os06g51029.1	155	-20.84	155	-20.84	768464	24	2409	1264
miR1874-3p	LOC_Os06g51029.2	155	-20.84	155	-20.84	768465	24	1981	836
miR1874-3p	LOC_Os06g51070.1	161	-26.89	161	-26.89	768468	24	1047	192
miR1874-3p	LOC_Os06g51160.1	161	-21.78	161	-21.78	768486	24	1806	1708
miR1874-3p	LOC_Os06g51250.1	160	-26.1	160	-26.1	768498	24	4122	1647
miR1874-3p	LOC_Os06g51460.1	159	-21.56	159	-21.56	768532	24	3495	3025
miR1874-3p	LOC_Os06g51460.2	159	-21.56	159	-21.56	768533	24	3483	2983
miR1874-3p	LOC_Os07g01214.1	159	-20.01	159	-20.01	768579	24	3255	2705
miR1874-3p	LOC_Os07g01540.1	148	-22.88	148	-22.88	768615	24	3664	3595
miR1874-3p	LOC_Os07g01820.1	151	-21.95	151	-21.95	768644	24	1493	427
miR1874-3p	LOC_Os07g01820.2	151	-21.95	151	-21.95	768645	24	1409	427
miR1874-3p	LOC_Os07g01820.3	151	-21.95	151	-21.95	768646	24	1406	427
miR1874-3p	LOC_Os07g01820.4	151	-21.95	151	-21.95	768647	24	1419	427
miR1874-3p	LOC_Os07g01820.5	151	-21.95	151	-21.95	768648	24	2686	373
miR1874-3p	LOC_Os07g01950.1	165	-24.14	165	-24.14	768666	24	716	532
miR1874-3p	LOC_Os07g01950.2	165	-24.14	165	-24.14	768667	24	794	610
miR1874-3p	LOC_Os07g02560.1	149	-20.18	149	-20.18	768727	24	1906	811
miR1874-3p	LOC_Os07g02570.1	167	-27.55	167	-27.55	768728	24	1533	520
miR1874-3p	LOC_Os07g02624.1	159	-25.07	159	-25.07	768735	24	1668	502
miR1874-3p	LOC_Os07g02640.1	163	-26.59	163	-26.59	768738	24	1290	487
miR1874-3p	LOC_Os07g02810.1	160	-20.09	160	-20.09	768758	24	2302	165
miR1874-3p	LOC_Os07g03110.1	168	-25.45	168	-25.45	768794	24	1834	149
miR1874-3p	LOC_Os07g03180.1	154	-23.09	154	-23.09	768803	24	900	222
miR1874-3p	LOC_Os07g03840.1	155	-23.59	155	-23.59	768864	24	2355	1411
miR1874-3p	LOC_Os07g03910.1	161	-29.11	161	-29.11	768871	24	1483	735
miR1874-3p	LOC_Os07g03960.1	161	-29.11	161	-29.11	768876	24	2003	1167
miR1874-3p	LOC_Os07g03990.1	161	-25.5	161	-25.5	768879	24	3481	380
miR1874-3p	LOC_Os07g04020.1	161	-25.53	161	-25.53	768882	24	1092	239

miR1874-3p	LOC_Os07g04690.1	156	-22.61	156	-22.61	768947	24	2033	540
miR1874-3p	LOC_Os07g04690.2	156	-22.61	156	-22.61	768948	24	1817	324
miR1874-3p	LOC_Os07g04700.1	147	-20.66	147	-20.66	768949	24	3570	1959
miR1874-3p	LOC_Os07g04700.2	147	-20.66	147	-20.66	768950	24	3632	1959
miR1874-3p	LOC_Os07g04700.3	147	-20.66	147	-20.66	768951	24	4016	2346
miR1874-3p	LOC_Os07g04700.5	147	-20.66	147	-20.66	768952	24	3751	2081
miR1874-3p	LOC_Os07g04700.6	147	-20.66	147	-20.66	768953	24	3941	2271
miR1874-3p	LOC_Os07g05140.1	154	-23.58	154	-23.58	769000	24	3708	3329
miR1874-3p	LOC_Os07g05330.1	149	-20.94	149	-20.94	769019	24	3723	3188
miR1874-3p	LOC_Os07g05430.1	172	-25.17	172	-25.17	769030	24	4302	2943
miR1874-3p	LOC_Os07g05840.1	143	-21.57	143	-21.57	769080	24	2105	439
miR1874-3p	LOC_Os07g05850.1	159	-20.63	159	-20.63	769081	24	2724	856
miR1874-3p	LOC_Os07g06262.1	154	-20.7	154	-20.7	769120	24	2436	281
miR1874-3p	LOC_Os07g06355.1	154	-20.7	154	-20.7	769126	24	2436	281
miR1874-3p	LOC_Os07g06550.1	153	-20.92	153	-20.92	769149	24	1799	901
miR1874-3p	LOC_Os07g06680.1	147	-22.62	147	-22.62	769169	24	2045	886
miR1874-3p	LOC_Os07g06830.1	158	-20.23	158	-20.23	769186	24	1390	4
miR1874-3p	LOC_Os07g06990.2	170	-22.8	170	-22.8	769205	24	1911	1135
miR1874-3p	LOC_Os07g07030.1	147	-24.94	147	-24.94	769210	24	2164	282
miR1874-3p	LOC_Os07g07030.2	147	-24.94	147	-24.94	769211	24	2023	60
miR1874-3p	LOC_Os07g07030.3	147	-24.94	147	-24.94	769212	24	2164	282
miR1874-3p	LOC_Os07g07030.4	147	-24.94	147	-24.94	769213	24	2020	60
miR1874-3p	LOC_Os07g07730.1	153	-24.76	153	-24.76	769291	24	330	230
miR1874-3p	LOC_Os07g07950.1	151	-20.78	151	-20.78	769313	24	2916	1611
miR1874-3p	LOC_Os07g07950.2	151	-20.78	151	-20.78	769314	24	2968	1663
miR1874-3p	LOC_Os07g08070.1	153	-22.83	153	-22.83	769324	24	1212	39
miR1874-3p	LOC_Os07g08460.1	158	-22.43	158	-22.43	769367	24	1166	649
miR1874-3p	LOC_Os07g08460.2	158	-22.43	158	-22.43	769368	24	1278	761
miR1874-3p	LOC_Os07g08490.1	149	-21.65	149	-21.65	769371	24	441	264
miR1874-3p	LOC_Os07g08550.1	169	-31.82	169	-31.82	769377	24	444	345
miR1874-3p	LOC_Os07g08710.1	141	-21.89	141	-21.89	769391	24	1189	410
miR1874-3p	LOC_Os07g08710.2	141	-21.89	141	-21.89	769392	24	1009	410

miR1874-3p	LOC_Os07g08820.1	164	-21.35	164	-21.35	769404	24	869	431
miR1874-3p	LOC_Os07g09020.1	159	-26.75	159	-26.75	769428	24	3528	2036
miR1874-3p	LOC_Os07g09060.1	152	-20.26	152	-20.26	769433	24	1944	1737
miR1874-3p	LOC_Os07g09060.2	152	-20.26	152	-20.26	769434	24	2018	1811
miR1874-3p	LOC_Os07g09060.3	152	-20.26	152	-20.26	769435	24	2033	1826
miR1874-3p	LOC_Os07g09384.1	160	-24.77	160	-24.77	769467	24	3349	3103
miR1874-3p	LOC_Os07g09460.1	153	-27.1	153	-27.1	769473	24	768	230
miR1874-3p	LOC_Os07g09460.2	153	-27.1	153	-27.1	769474	24	822	304
miR1874-3p	LOC_Os07g09740.1	156	-25.95	156	-25.95	769506	24	1995	1242
miR1874-3p	LOC_Os07g09830.1	291	-44.19	147	-22.19	769515	24	2121	469 1206
miR1874-3p	LOC_Os07g09860.1	441	-65.24	149	-22.87	769518	24	2037	73 436 607
miR1874-3p	LOC_Os07g09880.1	161	-20.32	161	-20.32	769520	24	1146	708
miR1874-3p	LOC_Os07g10200.1	148	-21.69	148	-21.69	769552	24	573	53
miR1874-3p	LOC_Os07g10300.1	148	-20.63	148	-20.63	769564	24	1656	1258
miR1874-3p	LOC_Os07g10300.3	148	-20.63	148	-20.63	769566	24	1796	1398
miR1874-3p	LOC_Os07g10520.1	173	-22.43	173	-22.43	769589	24	2638	664
miR1874-3p	LOC_Os07g10530.1	164	-24.97	164	-24.97	769590	24	1845	93
miR1874-3p	LOC_Os07g10580.1	165	-21.24	165	-21.24	769597	24	694	436
miR1874-3p	LOC_Os07g11150.1	144	-20.86	144	-20.86	769669	24	2930	2627
miR1874-3p	LOC_Os07g11150.3	144	-20.86	144	-20.86	769671	24	2932	2629
miR1874-3p	LOC_Os07g11680.1	157	-28.9	157	-28.9	769725	24	5976	3272
miR1874-3p	LOC_Os07g11720.1	156	-23.36	156	-23.36	769729	24	798	508
miR1874-3p	LOC_Os07g11790.1	158	-20.3	158	-20.3	769733	24	2016	1914
miR1874-3p	LOC_Os07g11810.1	154	-21.74	154	-21.74	769735	24	819	376
miR1874-3p	LOC_Os07g12130.1	150	-21.4	150	-21.4	769762	24	1482	74
miR1874-3p	LOC_Os07g12140.1	153	-22.16	153	-22.16	769763	24	2529	1243
miR1874-3p	LOC_Os07g12140.2	153	-22.16	153	-22.16	769764	24	2411	1106
miR1874-3p	LOC_Os07g12180.1	169	-23.73	169	-23.73	769768	24	1872	662
miR1874-3p	LOC_Os07g12290.1	162	-20.65	162	-20.65	769780	24	246	43
miR1874-3p	LOC_Os07g12470.1	165	-20.88	165	-20.88	769801	24	3176	428
miR1874-3p	LOC_Os07g12600.1	155	-23.08	155	-23.08	769818	24	2584	2446
miR1874-3p	LOC_Os07g12650.1	157	-20.29	157	-20.29	769823	24	2018	1485

miR1874-3p	LOC_Os07g12800.1	156	-21.53	156	-21.53	769838	24	2064	98
miR1874-3p	LOC_Os07g12800.2	156	-21.53	156	-21.53	769839	24	2181	215
miR1874-3p	LOC_Os07g12810.1	155	-21.95	155	-21.95	769840	24	1881	1215
miR1874-3p	LOC_Os07g12830.1	167	-24.52	167	-24.52	769842	24	1385	1214
miR1874-3p	LOC_Os07g12830.2	167	-24.52	167	-24.52	769843	24	1709	1538
miR1874-3p	LOC_Os07g12830.3	167	-24.52	167	-24.52	769844	24	1426	1255
miR1874-3p	LOC_Os07g12980.1	164	-24.18	164	-24.18	769859	24	4557	2702
miR1874-3p	LOC_Os07g13170.1	158	-20.2	158	-20.2	769875	24	2229	1456
miR1874-3p	LOC_Os07g13170.2	158	-20.2	158	-20.2	769876	24	2195	1515
miR1874-3p	LOC_Os07g13530.1	161	-23.73	161	-23.73	769911	24	1355	650
miR1874-3p	LOC_Os07g13530.2	161	-23.73	161	-23.73	769912	24	1470	765
miR1874-3p	LOC_Os07g13790.1	161	-24.83	161	-24.83	769935	24	366	263
miR1874-3p	LOC_Os07g15340.1	144	-21.38	144	-21.38	770107	24	2694	1279
miR1874-3p	LOC_Os07g15490.1	159	-23.14	159	-23.14	770123	24	1174	1
miR1874-3p	LOC_Os07g15600.1	150	-22.71	150	-22.71	770134	24	2150	136
miR1874-3p	LOC_Os07g15630.1	156	-20.06	156	-20.06	770137	24	3147	180
miR1874-3p	LOC_Os07g15680.1	151	-23.8	151	-23.8	770144	24	3278	708
miR1874-3p	LOC_Os07g15850.1	141	-22.07	141	-22.07	770160	24	2394	1762
miR1874-3p	LOC_Os07g16520.1	153	-20.94	153	-20.94	770219	24	3352	2924
miR1874-3p	LOC_Os07g16580.1	305	-44.47	154	-22.73	770225	24	861	355 539
miR1874-3p	LOC_Os07g16760.1	169	-26.97	169	-26.97	770243	24	927	125
miR1874-3p	LOC_Os07g17040.1	157	-26.27	157	-26.27	770275	24	798	36
miR1874-3p	LOC_Os07g17280.1	152	-22.65	152	-22.65	770300	24	2559	1299
miR1874-3p	LOC_Os07g17420.1	154	-20.86	154	-20.86	770316	24	1365	24
miR1874-3p	LOC_Os07g17580.1	155	-21.48	155	-21.48	770332	24	3807	610
miR1874-3p	LOC_Os07g17610.1	156	-25.26	156	-25.26	770335	24	5019	2124
miR1874-3p	LOC_Os07g17810.1	149	-21.88	149	-21.88	770355	24	2151	1787
miR1874-3p	LOC_Os07g17850.1	171	-25.73	171	-25.73	770359	24	2331	2309
miR1874-3p	LOC_Os07g19510.1	148	-21.59	148	-21.59	770515	24	3390	3111
miR1874-3p	LOC_Os07g20130.1	150	-21.43	150	-21.43	770528	24	978	568
miR1874-3p	LOC_Os07g20370.1	159	-20.96	159	-20.96	770552	24	1734	1358
miR1874-3p	LOC_Os07g20430.1	154	-23.58	154	-23.58	770558	24	5045	4666

miR1874-3p	LOC_Os07g20470.1	158	-21.02	158	-21.02	770562	24	807	361
miR1874-3p	LOC_Os07g20510.1	168	-31.72	168	-31.72	770566	24	3840	468
miR1874-3p	LOC_Os07g20670.1	168	-26.52	168	-26.52	770583	24	5637	1997
miR1874-3p	LOC_Os07g21980.1	151	-20.03	151	-20.03	770614	24	1605	69
miR1874-3p	LOC_Os07g22200.1	160	-21.69	160	-21.69	770633	24	4686	1067
miR1874-3p	LOC_Os07g22390.1	156	-21.62	156	-21.62	770653	24	1950	315
miR1874-3p	LOC_Os07g22390.2	156	-21.62	156	-21.62	770654	24	1661	315
miR1874-3p	LOC_Os07g22494.1	149	-20.64	149	-20.64	770665	24	2207	1359
miR1874-3p	LOC_Os07g22494.2	149	-20.64	149	-20.64	770666	24	1646	798
miR1874-3p	LOC_Os07g22494.3	149	-20.64	149	-20.64	770667	24	2206	1359
miR1874-3p	LOC_Os07g22750.1	155	-24.85	155	-24.85	770708	24	1692	186
miR1874-3p	LOC_Os07g22960.1	158	-22.62	158	-22.62	770731	24	8239	998
miR1874-3p	LOC_Os07g23480.1	167	-20.11	167	-20.11	770781	24	6171	4318
miR1874-3p	LOC_Os07g23990.1	164	-21.86	164	-21.86	770842	24	6206	957
miR1874-3p	LOC_Os07g23990.2	164	-21.86	164	-21.86	770843	24	6190	957
miR1874-3p	LOC_Os07g24100.1	151	-20.14	151	-20.14	770853	24	5727	727
miR1874-3p	LOC_Os07g24190.1	158	-22.04	158	-22.04	770861	24	4525	3347
miR1874-3p	LOC_Os07g24190.2	158	-22.04	158	-22.04	770862	24	3487	3347
miR1874-3p	LOC_Os07g24190.3	158	-22.04	158	-22.04	770863	24	4622	3347
miR1874-3p	LOC_Os07g24790.1	147	-22.98	147	-22.98	770919	24	1713	1145
miR1874-3p	LOC_Os07g24910.1	152	-23.31	152	-23.31	770930	24	3254	2828
miR1874-3p	LOC_Os07g24950.1	158	-21.78	158	-21.78	770934	24	336	243
miR1874-3p	LOC_Os07g24960.1	169	-28.72	169	-28.72	770935	24	2736	2196
miR1874-3p	LOC_Os07g25010.1	147	-23.89	147	-23.89	770948	24	2886	2459
miR1874-3p	LOC_Os07g25040.1	149	-21.88	149	-21.88	770950	24	3309	2945
miR1874-3p	LOC_Os07g25080.1	154	-20.7	154	-20.7	770954	24	2496	281
miR1874-3p	LOC_Os07g25110.1	149	-21.88	149	-21.88	770957	24	1383	1019
miR1874-3p	LOC_Os07g25200.1	153	-20.94	153	-20.94	770966	24	2174	1655
miR1874-3p	LOC_Os07g25240.1	162	-20.65	162	-20.65	770970	24	1020	58
miR1874-3p	LOC_Os07g25260.1	155	-21.74	155	-21.74	770972	24	1383	71
miR1874-3p	LOC_Os07g25310.1	154	-23.14	154	-23.14	770977	24	1056	968
miR1874-3p	LOC_Os07g25330.1	153	-20.73	153	-20.73	770979	24	3960	3659

miR1874-3p	LOC_Os07g25440.1	155	-20.28	155	-20.28	770990	24	2733	417
miR1874-3p	LOC_Os07g25440.2	155	-20.28	155	-20.28	770991	24	2658	417
miR1874-3p	LOC_Os07g25540.1	165	-21.42	165	-21.42	771001	24	1738	246
miR1874-3p	LOC_Os07g25650.1	149	-23.03	149	-23.03	771011	24	1890	1453
miR1874-3p	LOC_Os07g26110.1	160	-21.51	160	-21.51	771057	24	1330	22
miR1874-3p	LOC_Os07g26490.1	158	-25.14	158	-25.14	771099	24	2378	850
miR1874-3p	LOC_Os07g26490.2	158	-25.14	158	-25.14	771100	24	2370	850
miR1874-3p	LOC_Os07g26780.1	157	-28.9	157	-28.9	771130	24	4989	2072
miR1874-3p	LOC_Os07g27330.1	304	-46.8	152	-24.99	771192	24	2716	409 579
miR1874-3p	LOC_Os07g27590.1	141	-21.99	141	-21.99	771219	24	897	562
miR1874-3p	LOC_Os07g27740.1	159	-23.12	159	-23.12	771232	24	4593	1427
miR1874-3p	LOC_Os07g28200.1	148	-24.19	148	-24.19	771281	24	4902	4251
miR1874-3p	LOC_Os07g28260.1	158	-22.9	158	-22.9	771287	24	2112	1970
miR1874-3p	LOC_Os07g28260.4	158	-22.9	158	-22.9	771288	24	5353	2321
miR1874-3p	LOC_Os07g28260.5	158	-22.9	158	-22.9	771289	24	4317	2224
miR1874-3p	LOC_Os07g28260.6	158	-22.9	158	-22.9	771290	24	2248	2106
miR1874-3p	LOC_Os07g28310.1	155	-21.24	155	-21.24	771294	24	1815	515
miR1874-3p	LOC_Os07g28370.1	143	-20.87	143	-20.87	771299	24	5385	4923
miR1874-3p	LOC_Os07g28810.1	164	-23.99	164	-23.99	771348	24	966	48
miR1874-3p	LOC_Os07g28900.1	153	-25.14	153	-25.14	771357	24	3048	1103
miR1874-3p	LOC_Os07g28920.1	304	-42.16	158	-21.49	771359	24	2626	774 1263
miR1874-3p	LOC_Os07g29190.1	155	-21.07	155	-21.07	771383	24	1196	1099
miR1874-3p	LOC_Os07g29450.1	158	-20.31	158	-20.31	771416	24	3166	128
miR1874-3p	LOC_Os07g29530.1	160	-21.89	160	-21.89	771424	24	465	319
miR1874-3p	LOC_Os07g30540.1	158	-24.07	158	-24.07	771529	24	699	215
miR1874-3p	LOC_Os07g30550.1	169	-28.72	169	-28.72	771530	24	2631	2196
miR1874-3p	LOC_Os07g30570.1	154	-23.05	154	-23.05	771532	24	2391	281
miR1874-3p	LOC_Os07g30790.1	151	-20.71	151	-20.71	771554	24	1769	852
miR1874-3p	LOC_Os07g30880.1	154	-22.07	154	-22.07	771568	24	5415	5393
miR1874-3p	LOC_Os07g31130.1	145	-20.87	145	-20.87	771593	24	2828	2459
miR1874-3p	LOC_Os07g31360.1	149	-21.28	149	-21.28	771621	24	480	391
miR1874-3p	LOC_Os07g31420.1	154	-21.97	154	-21.97	771627	24	3241	60

miR1874-3p	LOC_Os07g31420.2	154	-21.97	154	-21.97	771628	24	3040	60
miR1874-3p	LOC_Os07g31490.1	149	-21.51	149	-21.51	771637	24	790	522
miR1874-3p	LOC_Os07g31490.2	149	-21.51	149	-21.51	771638	24	689	422
miR1874-3p	LOC_Os07g32060.1	151	-20.59	151	-20.59	771687	24	1848	1515
miR1874-3p	LOC_Os07g32090.1	159	-26.2	159	-26.2	771690	24	1479	1455
miR1874-3p	LOC_Os07g32350.1	174	-22.61	174	-22.61	771720	24	1647	337
miR1874-3p	LOC_Os07g32350.2	174	-22.61	174	-22.61	771721	24	2476	337
miR1874-3p	LOC_Os07g32550.1	162	-24.84	162	-24.84	771744	24	1532	1169
miR1874-3p	LOC_Os07g32774.1	158	-21.54	158	-21.54	771768	24	1171	1081
miR1874-3p	LOC_Os07g32800.1	164	-23.87	164	-23.87	771771	24	1019	408
miR1874-3p	LOC_Os07g32800.2	164	-23.87	164	-23.87	771772	24	933	408
miR1874-3p	LOC_Os07g33110.1	153	-22.83	153	-22.83	771801	24	2043	160
miR1874-3p	LOC_Os07g33110.2	153	-22.83	153	-22.83	771802	24	2382	160
miR1874-3p	LOC_Os07g33110.3	153	-22.83	153	-22.83	771803	24	2406	160
miR1874-3p	LOC_Os07g33110.4	153	-22.83	153	-22.83	771804	24	2220	160
miR1874-3p	LOC_Os07g33110.5	153	-22.83	153	-22.83	771805	24	2452	160
miR1874-3p	LOC_Os07g33200.1	153	-22.71	153	-22.71	771813	24	2107	1
miR1874-3p	LOC_Os07g33210.1	153	-21.37	153	-21.37	771814	24	1539	378
miR1874-3p	LOC_Os07g33670.1	321	-40.59	162	-20.57	771868	24	4687	2849 884
miR1874-3p	LOC_Os07g33850.1	146	-24.39	146	-24.39	771885	24	1542	444
miR1874-3p	LOC_Os07g33850.2	146	-24.39	146	-24.39	771886	24	1991	444
miR1874-3p	LOC_Os07g34020.1	157	-22.95	157	-22.95	771896	24	1009	51
miR1874-3p	LOC_Os07g34020.2	157	-22.95	157	-22.95	771897	24	1005	51
miR1874-3p	LOC_Os07g34460.1	154	-22.42	154	-22.42	771942	24	510	482
miR1874-3p	LOC_Os07g34510.1	147	-24.08	147	-24.08	771947	24	5373	277
miR1874-3p	LOC_Os07g34550.1	155	-20.54	155	-20.54	771953	24	2058	909
miR1874-3p	LOC_Os07g34598.1	156	-21.78	156	-21.78	771963	24	2382	1254
miR1874-3p	LOC_Os07g34598.2	156	-21.78	156	-21.78	771964	24	2514	1389
miR1874-3p	LOC_Os07g34750.1	159	-20.94	159	-20.94	771983	24	3222	2081
miR1874-3p	LOC_Os07g35260.1	159	-24.1	159	-24.1	772034	24	2426	1837
miR1874-3p	LOC_Os07g35260.2	159	-24.1	159	-24.1	772035	24	2480	1939
miR1874-3p	LOC_Os07g35630.1	169	-27.03	169	-27.03	772081	24	1242	921

miR1874-3p	LOC_Os07g35960.1	146	-20.12	146	-20.12	772117	24	2991	1757
miR1874-3p	LOC_Os07g36000.1	176	-33.4	176	-33.4	772122	24	4206	2241
miR1874-3p	LOC_Os07g36020.1	158	-21.78	158	-21.78	772124	24	615	72
miR1874-3p	LOC_Os07g36030.1	169	-28.72	169	-28.72	772125	24	2631	2196
miR1874-3p	LOC_Os07g36250.1	172	-25.7	172	-25.7	772157	24	1774	673
miR1874-3p	LOC_Os07g36250.2	172	-25.7	172	-25.7	772158	24	1694	673
miR1874-3p	LOC_Os07g36250.3	172	-25.7	172	-25.7	772159	24	1790	673
miR1874-3p	LOC_Os07g36370.1	151	-20.98	151	-20.98	772171	24	1802	1034
miR1874-3p	LOC_Os07g36710.1	156	-20.48	156	-20.48	772203	24	2412	1824
miR1874-3p	LOC_Os07g36740.1	156	-22	156	-22	772206	24	2670	1718
miR1874-3p	LOC_Os07g36860.1	140	-22.11	140	-22.11	772220	24	1041	578
miR1874-3p	LOC_Os07g37650.1	156	-22.46	156	-22.46	772309	24	2202	1164
miR1874-3p	LOC_Os07g38120.1	145	-20.27	145	-20.27	772365	24	2370	771
miR1874-3p	LOC_Os07g38560.1	154	-21.01	154	-21.01	772414	24	1485	1428
miR1874-3p	LOC_Os07g38730.1	157	-20.54	157	-20.54	772432	24	1783	906
miR1874-3p	LOC_Os07g39020.1	140	-20.06	140	-20.06	772468	24	2447	1642
miR1874-3p	LOC_Os07g39030.1	153	-23.12	153	-23.12	772469	24	312	244
miR1874-3p	LOC_Os07g39090.1	318	-43.08	167	-23.03	772474	24	3036	763 108
miR1874-3p	LOC_Os07g39150.1	149	-22.12	149	-22.12	772482	24	1443	575
miR1874-3p	LOC_Os07g39240.1	155	-21.84	155	-21.84	772490	24	3658	1730
miR1874-3p	LOC_Os07g39410.1	148	-27.39	148	-27.39	772509	24	2379	501
miR1874-3p	LOC_Os07g39750.1	144	-23.8	144	-23.8	772550	24	2350	1015
miR1874-3p	LOC_Os07g39910.1	158	-20.46	158	-20.46	772572	24	2427	1197
miR1874-3p	LOC_Os07g39990.1	165	-21.07	165	-21.07	772580	24	492	423
miR1874-3p	LOC_Os07g40030.1	161	-25.09	161	-25.09	772583	24	1479	214
miR1874-3p	LOC_Os07g40030.2	161	-25.09	161	-25.09	772584	24	1457	214
miR1874-3p	LOC_Os07g40030.3	161	-25.09	161	-25.09	772585	24	1619	214
miR1874-3p	LOC_Os07g40910.1	170	-23.92	170	-23.92	772681	24	633	69
miR1874-3p	LOC_Os07g41240.1	151	-22.26	151	-22.26	772713	24	1945	1670
miR1874-3p	LOC_Os07g41310.1	165	-20.75	165	-20.75	772723	24	1988	69
miR1874-3p	LOC_Os07g41410.1	154	-27.06	154	-27.06	772734	24	720	617
miR1874-3p	LOC_Os07g42020.1	165	-27.99	165	-27.99	772810	24	2058	1623

miR1874-3p	LOC_Os07g42030.1	150	-20.55	150	-20.55	772811	24	711	134
miR1874-3p	LOC_Os07g42070.1	150	-22.74	150	-22.74	772815	24	901	255
miR1874-3p	LOC_Os07g42410.1	151	-21.13	151	-21.13	772849	24	4850	3364
miR1874-3p	LOC_Os07g42440.4	160	-21.98	160	-21.98	772855	24	1155	852
miR1874-3p	LOC_Os07g42490.1	314	-47.46	158	-24.95	772859	24	2820	2622 7
miR1874-3p	LOC_Os07g42490.2	314	-47.46	158	-24.95	772860	24	2956	2763 7
miR1874-3p	LOC_Os07g43640.1	161	-24.7	161	-24.7	773002	24	783	153
miR1874-3p	LOC_Os07g44060.1	152	-22.93	152	-22.93	773049	24	1136	1006
miR1874-3p	LOC_Os07g44330.3	152	-21.02	152	-21.02	773081	24	2228	1633
miR1874-3p	LOC_Os07g44340.1	140	-20.29	140	-20.29	773082	24	2199	576
miR1874-3p	LOC_Os07g44390.1	176	-33.4	176	-33.4	773088	24	3408	1548
miR1874-3p	LOC_Os07g44640.1	152	-25.05	152	-25.05	773116	24	2289	768
miR1874-3p	LOC_Os07g44820.1	324	-46.5	169	-23.7	773135	24	2226	986 1071
miR1874-3p	LOC_Os07g44850.1	152	-24.96	152	-24.96	773138	24	1459	963
miR1874-3p	LOC_Os07g44920.1	165	-20.3	165	-20.3	773146	24	1016	658
miR1874-3p	LOC_Os07g45750.1	157	-20.21	157	-20.21	773229	24	5637	3520
miR1874-3p	LOC_Os07g46039.1	158	-20.64	158	-20.64	773258	24	3192	2263
miR1874-3p	LOC_Os07g46039.2	158	-20.64	158	-20.64	773259	24	2877	1934
miR1874-3p	LOC_Os07g46060.1	142	-20.91	142	-20.91	773260	24	3620	2141
miR1874-3p	LOC_Os07g46220.1	155	-20.84	155	-20.84	773276	24	1558	584
miR1874-3p	LOC_Os07g46500.1	162	-20.3	162	-20.3	773315	24	861	448
miR1874-3p	LOC_Os07g46500.2	327	-42.43	165	-22.13	773316	24	1125	567 702
miR1874-3p	LOC_Os07g46650.1	160	-25.16	160	-25.16	773333	24	2076	562
miR1874-3p	LOC_Os07g46770.1	165	-22.09	165	-22.09	773355	24	1860	1266
miR1874-3p	LOC_Os07g47110.1	166	-27.36	166	-27.36	773407	24	2168	274
miR1874-3p	LOC_Os07g47160.1	341	-62.14	179	-32.65	773413	24	897	236 277
miR1874-3p	LOC_Os07g47360.1	168	-25.01	168	-25.01	773442	24	5838	919
miR1874-3p	LOC_Os07g47360.2	168	-25.01	168	-25.01	773443	24	6067	919
miR1874-3p	LOC_Os07g47360.3	168	-25.01	168	-25.01	773444	24	6070	919
miR1874-3p	LOC_Os07g47480.1	166	-21.08	166	-21.08	773457	24	1270	91
miR1874-3p	LOC_Os07g47480.2	166	-21.08	166	-21.08	773458	24	1378	91
miR1874-3p	LOC_Os07g47590.1	157	-23.08	157	-23.08	773479	24	1723	1192

miR1874-3p	LOC_Os07g47590.2	157	-23.08	157	-23.08	773480	24	1514	1192
miR1874-3p	LOC_Os07g47590.3	157	-23.08	157	-23.08	773481	24	1443	1192
miR1874-3p	LOC_Os07g48370.1	155	-22.7	155	-22.7	773577	24	2910	138
miR1874-3p	LOC_Os07g48520.1	156	-22.3	156	-22.3	773602	24	4592	524
miR1874-3p	LOC_Os07g48520.2	156	-22.3	156	-22.3	773603	24	4421	524
miR1874-3p	LOC_Os07g48520.3	156	-22.3	156	-22.3	773604	24	3506	524
miR1874-3p	LOC_Os07g48620.1	169	-27.61	169	-27.61	773613	24	3096	1790
miR1874-3p	LOC_Os07g49120.1	142	-22.71	142	-22.71	773679	24	1220	63
miR1874-3p	LOC_Os07g49210.1	307	-42.12	156	-21.66	773688	24	6079	5184 242
miR1874-3p	LOC_Os07g49410.1	155	-21.81	155	-21.81	773717	24	1444	668
miR1874-3p	LOC_Os07g49410.2	155	-21.81	155	-21.81	773718	24	1466	690
miR1874-3p	LOC_Os07g49410.3	155	-21.81	155	-21.81	773719	24	1440	664
miR1874-3p	LOC_Os07g49520.1	151	-23.51	151	-23.51	773733	24	3644	1434
miR1874-3p	LOC_Os08g01090.2	169	-23.81	169	-23.81	773749	24	1608	1411
miR1874-3p	LOC_Os08g01100.1	165	-20.03	165	-20.03	773750	24	1021	332
miR1874-3p	LOC_Os08g01190.1	152	-20.91	152	-20.91	773759	24	1164	169
miR1874-3p	LOC_Os08g01200.1	146	-22.23	146	-22.23	773760	24	1080	420
miR1874-3p	LOC_Os08g01380.1	159	-21.56	159	-21.56	773790	24	956	43
miR1874-3p	LOC_Os08g01380.2	159	-21.56	159	-21.56	773791	24	863	43
miR1874-3p	LOC_Os08g01520.1	159	-24.4	159	-24.4	773806	24	1575	866
miR1874-3p	LOC_Os08g01650.1	161	-22.33	161	-22.33	773821	24	2680	809
miR1874-3p	LOC_Os08g01660.2	162	-23.29	162	-23.29	773823	24	677	647
miR1874-3p	LOC_Os08g01900.1	161	-20.24	161	-20.24	773852	24	2962	1981
miR1874-3p	LOC_Os08g01910.1	150	-20.88	150	-20.88	773853	24	998	892
miR1874-3p	LOC_Os08g01940.1	159	-23.65	159	-23.65	773856	24	3263	2801
miR1874-3p	LOC_Os08g01940.2	159	-23.65	159	-23.65	773857	24	3254	2801
miR1874-3p	LOC_Os08g02130.1	154	-22.78	154	-22.78	773876	24	3192	2109
miR1874-3p	LOC_Os08g02130.2	154	-22.78	154	-22.78	773877	24	3196	2113
miR1874-3p	LOC_Os08g02390.1	158	-22.18	158	-22.18	773908	24	1219	37
miR1874-3p	LOC_Os08g02420.1	156	-21.49	156	-21.49	773911	24	1265	126
miR1874-3p	LOC_Os08g02990.1	161	-21.92	161	-21.92	773972	24	6355	5058
miR1874-3p	LOC_Os08g03002.1	156	-28.47	156	-28.47	773975	24	1783	670

miR1874-3p	LOC_Os08g03010.1	158	-21.02	158	-21.02	773976	24	5265	4759
miR1874-3p	LOC_Os08g03430.1	158	-21.45	158	-21.45	774028	24	1937	1779
miR1874-3p	LOC_Os08g03440.1	148	-22.19	148	-22.19	774030	24	1977	1641
miR1874-3p	LOC_Os08g03579.1	148	-22.95	148	-22.95	774045	24	1709	334
miR1874-3p	LOC_Os08g03610.1	149	-20.81	149	-20.81	774049	24	873	241
miR1874-3p	LOC_Os08g03630.1	147	-23.64	147	-23.64	774051	24	1888	616
miR1874-3p	LOC_Os08g04050.1	157	-20.28	157	-20.28	774098	24	849	51
miR1874-3p	LOC_Os08g04110.1	163	-22.83	163	-22.83	774105	24	1581	800
miR1874-3p	LOC_Os08g04110.2	163	-22.83	163	-22.83	774106	24	1623	761
miR1874-3p	LOC_Os08g04180.1	149	-22.02	149	-22.02	774116	24	1829	856
miR1874-3p	LOC_Os08g04400.1	159	-24.74	159	-24.74	774140	24	3410	109
miR1874-3p	LOC_Os08g04440.1	164	-24.59	164	-24.59	774144	24	1080	709
miR1874-3p	LOC_Os08g04440.2	164	-20.95	164	-20.95	774145	24	2636	2286
miR1874-3p	LOC_Os08g04500.1	153	-21.82	153	-21.82	774154	24	1949	310
miR1874-3p	LOC_Os08g04500.2	153	-21.82	153	-21.82	774155	24	2127	310
miR1874-3p	LOC_Os08g05190.1	158	-21.02	158	-21.02	774234	24	798	370
miR1874-3p	LOC_Os08g05250.1	155	-21.41	155	-21.41	774239	24	4254	347
miR1874-3p	LOC_Os08g05810.1	159	-22.06	159	-22.06	774296	24	2844	1501
miR1874-3p	LOC_Os08g06130.1	156	-23.01	156	-23.01	774333	24	2577	450
miR1874-3p	LOC_Os08g06140.1	166	-20.56	166	-20.56	774334	24	2944	535
miR1874-3p	LOC_Os08g06140.2	166	-20.56	166	-20.56	774335	24	2942	536
miR1874-3p	LOC_Os08g06140.3	166	-20.56	166	-20.56	774336	24	2906	536
miR1874-3p	LOC_Os08g06250.1	151	-20.39	151	-20.39	774347	24	1449	558
miR1874-3p	LOC_Os08g06320.1	152	-20.9	152	-20.9	774360	24	1387	360
miR1874-3p	LOC_Os08g06344.1	321	-45.71	162	-23.65	774362	24	4796	3861 2939
miR1874-3p	LOC_Os08g06344.2	321	-45.71	162	-23.65	774363	24	4487	3596 2674
miR1874-3p	LOC_Os08g06344.3	321	-45.71	162	-23.65	774364	24	4655	3764 2842
miR1874-3p	LOC_Os08g06740.1	152	-23.08	152	-23.08	774412	24	1062	709
miR1874-3p	LOC_Os08g07040.1	151	-20.05	151	-20.05	774441	24	1584	1300
miR1874-3p	LOC_Os08g07130.1	151	-25.25	151	-25.25	774449	24	6036	2556
miR1874-3p	LOC_Os08g07140.1	141	-22.41	141	-22.41	774450	24	3144	2890
miR1874-3p	LOC_Os08g07290.1	316	-44.58	159	-22.53	774462	24	6519	5003 3105

miR1874-3p	LOC_Os08g07290.2	157	-22.53	157	-22.53	774463	24	5090	3105
miR1874-3p	LOC_Os08g07330.2	150	-20.48	150	-20.48	774467	24	3507	499
miR1874-3p	LOC_Os08g07660.1	161	-20.59	161	-20.59	774501	24	866	19
miR1874-3p	LOC_Os08g08080.1	158	-24.97	158	-24.97	774543	24	2229	86
miR1874-3p	LOC_Os08g08080.2	158	-24.97	158	-24.97	774544	24	2186	81
miR1874-3p	LOC_Os08g08110.1	163	-21.5	163	-21.5	774548	24	1953	1465
miR1874-3p	LOC_Os08g08130.1	150	-22.49	150	-22.49	774552	24	1567	1148
miR1874-3p	LOC_Os08g08460.1	163	-24.55	163	-24.55	774587	24	5964	1988
miR1874-3p	LOC_Os08g08550.1	154	-22.07	154	-22.07	774593	24	5274	5252
miR1874-3p	LOC_Os08g08660.1	167	-24.03	167	-24.03	774612	24	504	32
miR1874-3p	LOC_Os08g09100.1	159	-22.65	159	-22.65	774658	24	1305	966
miR1874-3p	LOC_Os08g09230.1	176	-27.64	176	-27.64	774672	24	5766	4468
miR1874-3p	LOC_Os08g09230.2	176	-27.64	176	-27.64	774673	24	5764	4468
miR1874-3p	LOC_Os08g09350.1	160	-22.11	160	-22.11	774690	24	2105	1525
miR1874-3p	LOC_Os08g09410.1	154	-24.92	154	-24.92	774696	24	1344	506
miR1874-3p	LOC_Os08g09450.1	141	-21.14	141	-21.14	774700	24	1582	516
miR1874-3p	LOC_Os08g09670.1	141	-21.04	141	-21.04	774719	24	1276	529
miR1874-3p	LOC_Os08g10210.1	155	-20.58	155	-20.58	774783	24	291	110
miR1874-3p	LOC_Os08g10730.1	159	-20.43	159	-20.43	774846	24	1641	1081
miR1874-3p	LOC_Os08g10880.1	153	-22.21	153	-22.21	774861	24	603	66
miR1874-3p	LOC_Os08g11110.1	149	-21.88	149	-21.88	774884	24	2889	2540
miR1874-3p	LOC_Os08g11440.1	154	-22.07	154	-22.07	774924	24	930	908
miR1874-3p	LOC_Os08g11690.1	172	-21.15	172	-21.15	774947	24	543	465
miR1874-3p	LOC_Os08g11770.1	154	-22.42	154	-22.42	774956	24	321	293
miR1874-3p	LOC_Os08g11940.1	154	-22.07	154	-22.07	774973	24	969	947
miR1874-3p	LOC_Os08g11980.1	158	-21.02	158	-21.02	774977	24	5214	4339
miR1874-3p	LOC_Os08g12090.1	154	-22.42	154	-22.42	774987	24	381	353
miR1874-3p	LOC_Os08g12180.1	156	-26.32	156	-26.32	774996	24	1827	820
miR1874-3p	LOC_Os08g12220.1	156	-26.32	156	-26.32	774999	24	1767	1027
miR1874-3p	LOC_Os08g12240.1	163	-27.68	163	-27.68	775001	24	336	245
miR1874-3p	LOC_Os08g12440.1	154	-23.58	154	-23.58	775020	24	4203	3824
miR1874-3p	LOC_Os08g12470.1	158	-20.34	158	-20.34	775025	24	5250	4696

miR1874-3p	LOC_Os08g13380.1	324	-42.78	165	-22.35	775124	24	741	6 628
miR1874-3p	LOC_Os08g13890.1	150	-22.21	150	-22.21	775180	24	1307	859
miR1874-3p	LOC_Os08g13920.1	159	-20.7	159	-20.7	775184	24	1436	1175
miR1874-3p	LOC_Os08g13970.1	160	-20.49	160	-20.49	775190	24	4506	4147
miR1874-3p	LOC_Os08g14400.1	168	-22.25	168	-22.25	775229	24	1250	186
miR1874-3p	LOC_Os08g14450.1	154	-22.93	154	-22.93	775236	24	2786	108
miR1874-3p	LOC_Os08g14560.1	156	-20.06	156	-20.06	775248	24	3093	180
miR1874-3p	LOC_Os08g14790.1	146	-20.04	146	-20.04	775270	24	420	176
miR1874-3p	LOC_Os08g14850.1	143	-23.86	143	-23.86	775276	24	4134	1182
miR1874-3p	LOC_Os08g14940.1	153	-21.98	153	-21.98	775292	24	2904	485
miR1874-3p	LOC_Os08g14950.1	156	-23.99	156	-23.99	775293	24	3169	552
miR1874-3p	LOC_Os08g15020.1	149	-20.53	149	-20.53	775300	24	1293	16
miR1874-3p	LOC_Os08g15090.1	157	-23.22	157	-23.22	775308	24	3550	2080
miR1874-3p	LOC_Os08g15230.1	150	-20.78	150	-20.78	775320	24	3579	3488
miR1874-3p	LOC_Os08g15310.1	160	-22.96	160	-22.96	775343	24	1908	1547
miR1874-3p	LOC_Os08g15760.1	149	-20.94	149	-20.94	775390	24	516	275
miR1874-3p	LOC_Os08g15990.1	155	-20.23	155	-20.23	775415	24	2628	2157
miR1874-3p	LOC_Os08g16070.1	156	-22.06	156	-22.06	775424	24	2388	2191
miR1874-3p	LOC_Os08g16090.1	157	-22.79	157	-22.79	775426	24	2751	110
miR1874-3p	LOC_Os08g16240.1	163	-24.56	163	-24.56	775442	24	4482	1334
miR1874-3p	LOC_Os08g16280.1	154	-21.23	154	-21.23	775447	24	2736	959
miR1874-3p	LOC_Os08g16440.1	154	-23.58	154	-23.58	775465	24	1350	1103
miR1874-3p	LOC_Os08g16480.1	141	-21.3	141	-21.3	775469	24	2440	2137
miR1874-3p	LOC_Os08g16480.2	141	-21.3	141	-21.3	775470	24	2239	1936
miR1874-3p	LOC_Os08g16480.3	141	-21.3	141	-21.3	775471	24	2436	2133
miR1874-3p	LOC_Os08g16700.1	151	-21.52	151	-21.52	775493	24	537	242
miR1874-3p	LOC_Os08g16710.1	155	-22	155	-22	775494	24	1802	587
miR1874-3p	LOC_Os08g16980.1	152	-25.46	152	-25.46	775524	24	2196	826
miR1874-3p	LOC_Os08g17030.1	169	-29.39	169	-29.39	775529	24	3102	1078
miR1874-3p	LOC_Os08g17050.1	155	-21.66	155	-21.66	775531	24	453	172
miR1874-3p	LOC_Os08g17820.1	159	-26.96	159	-26.96	775605	24	6047	40
miR1874-3p	LOC_Os08g17820.2	159	-26.96	159	-26.96	775606	24	5913	34

miR1874-3p	LOC_Os08g17870.1	160	-20.87	160	-20.87	775610	24	2465	63
miR1874-3p	LOC_Os08g17910.1	161	-24.75	161	-24.75	775614	24	4248	1104
miR1874-3p	LOC_Os08g18000.1	154	-20.92	154	-20.92	775623	24	1660	1166
miR1874-3p	LOC_Os08g19040.1	155	-23.09	155	-23.09	775681	24	621	600
miR1874-3p	LOC_Os08g19114.1	163	-21.9	163	-21.9	775689	24	3449	1522
miR1874-3p	LOC_Os08g19114.2	163	-21.9	163	-21.9	775690	24	1100	177
miR1874-3p	LOC_Os08g19374.1	151	-20.49	151	-20.49	775719	24	2232	1510
miR1874-3p	LOC_Os08g19540.1	156	-20.7	156	-20.7	775738	24	3897	3476
miR1874-3p	LOC_Os08g19610.4	170	-20.07	170	-20.07	775748	24	3303	2125
miR1874-3p	LOC_Os08g20040.1	140	-20.5	140	-20.5	775792	24	1020	379
miR1874-3p	LOC_Os08g20290.1	316	-46.44	162	-25.78	775818	24	2724	1489 788
miR1874-3p	LOC_Os08g20410.1	164	-23.03	164	-23.03	775830	24	1183	347
miR1874-3p	LOC_Os08g20780.1	158	-21.02	158	-21.02	775862	24	879	370
miR1874-3p	LOC_Os08g20849.1	154	-22.07	154	-22.07	775868	24	5415	5393
miR1874-3p	LOC_Os08g21110.1	151	-24.57	151	-24.57	775883	24	6117	4403
miR1874-3p	LOC_Os08g21210.1	161	-20.93	161	-20.93	775890	24	2547	2114
miR1874-3p	LOC_Os08g21350.1	322	-49.74	172	-27.14	775902	24	4078	2351 3741
miR1874-3p	LOC_Os08g21350.2	322	-49.74	172	-27.14	775903	24	3840	2351 3741
miR1874-3p	LOC_Os08g21350.3	322	-49.74	172	-27.14	775904	24	4066	2351 3729
miR1874-3p	LOC_Os08g21420.1	143	-20.79	143	-20.79	775911	24	1593	750
miR1874-3p	LOC_Os08g21541.1	154	-23.94	154	-23.94	775922	24	4751	1089
miR1874-3p	LOC_Os08g21990.1	154	-21.23	154	-21.23	775965	24	3054	875
miR1874-3p	LOC_Os08g22012.1	154	-21.23	154	-21.23	775969	24	4035	1646
miR1874-3p	LOC_Os08g22036.1	154	-21.23	154	-21.23	775979	24	2646	692
miR1874-3p	LOC_Os08g22054.1	154	-21.23	154	-21.23	775982	24	4083	1646
miR1874-3p	LOC_Os08g22340.1	300	-42.96	155	-21.63	776001	24	3705	1380 3503
miR1874-3p	LOC_Os08g22700.1	155	-20.16	155	-20.16	776033	24	534	315
miR1874-3p	LOC_Os08g22960.1	320	-41.2	162	-21.02	776055	24	5608	4724 4139
miR1874-3p	LOC_Os08g22990.1	147	-23.72	147	-23.72	776058	24	4728	4283
miR1874-3p	LOC_Os08g23600.1	152	-20.42	152	-20.42	776124	24	381	158
miR1874-3p	LOC_Os08g23740.1	158	-23.33	158	-23.33	776139	24	2652	18
miR1874-3p	LOC_Os08g24260.1	154	-20.7	154	-20.7	776194	24	2577	281

miR1874-3p	LOC_Os08g24380.1	172	-27.54	172	-27.54	776206	24	2793	1289
miR1874-3p	LOC_Os08g25280.1	155	-27.7	155	-27.7	776296	24	3076	265
miR1874-3p	LOC_Os08g25734.1	160	-21.29	160	-21.29	776343	24	2063	1201
miR1874-3p	LOC_Os08g25734.2	160	-21.29	160	-21.29	776344	24	1944	1201
miR1874-3p	LOC_Os08g25864.1	165	-21	165	-21	776356	24	1717	768
miR1874-3p	LOC_Os08g25870.1	151	-20.14	151	-20.14	776357	24	1428	250
miR1874-3p	LOC_Os08g26020.1	169	-28.72	169	-28.72	776368	24	2631	2196
miR1874-3p	LOC_Os08g26500.1	150	-20.23	150	-20.23	776421	24	3231	988
miR1874-3p	LOC_Os08g26650.1	140	-20.17	140	-20.17	776432	24	2643	874
miR1874-3p	LOC_Os08g26875.1	160	-27.15	160	-27.15	776463	24	3718	1167
miR1874-3p	LOC_Os08g27120.1	153	-21.54	153	-21.54	776489	24	1023	383
miR1874-3p	LOC_Os08g27640.1	155	-20.48	155	-20.48	776544	24	645	351
miR1874-3p	LOC_Os08g27759.1	166	-22.58	166	-22.58	776557	24	2541	566
miR1874-3p	LOC_Os08g28030.1	157	-22.06	157	-22.06	776584	24	2378	1049
miR1874-3p	LOC_Os08g28070.1	162	-20.53	162	-20.53	776588	24	642	439
miR1874-3p	LOC_Os08g28310.1	154	-22.44	154	-22.44	776616	24	1848	1826
miR1874-3p	LOC_Os08g28350.1	150	-21.18	150	-21.18	776619	24	3669	3246
miR1874-3p	LOC_Os08g28570.1	154	-20.81	154	-20.81	776640	24	2652	125
miR1874-3p	LOC_Os08g28784.1	154	-22.22	154	-22.22	776662	24	885	507
miR1874-3p	LOC_Os08g28930.1	154	-21.54	154	-21.54	776676	24	1419	757
miR1874-3p	LOC_Os08g29030.1	168	-26.06	168	-26.06	776688	24	2496	10
miR1874-3p	LOC_Os08g29420.1	156	-21.34	156	-21.34	776728	24	3129	2522
miR1874-3p	LOC_Os08g29490.1	147	-21.35	147	-21.35	776735	24	5181	951
miR1874-3p	LOC_Os08g29570.1	162	-25.22	162	-25.22	776745	24	2701	1248
miR1874-3p	LOC_Os08g29660.1	150	-20.9	150	-20.9	776754	24	2274	1122
miR1874-3p	LOC_Os08g29669.1	150	-20.9	150	-20.9	776755	24	2000	613
miR1874-3p	LOC_Os08g29809.1	308	-42.63	159	-21.44	776769	24	3204	188 2579
miR1874-3p	LOC_Os08g30080.1	161	-20.62	161	-20.62	776799	24	1542	105
miR1874-3p	LOC_Os08g31160.1	160	-20.72	160	-20.72	776906	24	519	2
miR1874-3p	LOC_Os08g31470.1	149	-23.5	149	-23.5	776942	24	2630	260
miR1874-3p	LOC_Os08g31470.4	149	-23.5	149	-23.5	776945	24	2706	260
miR1874-3p	LOC_Os08g31870.1	147	-21.38	147	-21.38	776989	24	2822	2354

miR1874-3p	LOC_Os08g31890.1	159	-22.17	159	-22.17	776991	24	1248	47
miR1874-3p	LOC_Os08g31920.1	144	-20.68	144	-20.68	776994	24	1901	1298
miR1874-3p	LOC_Os08g31990.1	153	-23.17	153	-23.17	777002	24	249	133
miR1874-3p	LOC_Os08g32240.1	159	-23.12	159	-23.12	777029	24	1791	1106
miR1874-3p	LOC_Os08g32260.1	160	-20.81	160	-20.81	777031	24	2913	1
miR1874-3p	LOC_Os08g32470.1	154	-24.99	154	-24.99	777052	24	693	533
miR1874-3p	LOC_Os08g32590.1	161	-28.46	161	-28.46	777064	24	1237	660
miR1874-3p	LOC_Os08g32680.1	161	-26.08	161	-26.08	777079	24	1587	303
miR1874-3p	LOC_Os08g32760.1	151	-25.25	151	-25.25	777086	24	6087	2559
miR1874-3p	LOC_Os08g32970.1	150	-21.58	150	-21.58	777109	24	1274	15
miR1874-3p	LOC_Os08g33210.1	168	-25.08	168	-25.08	777140	24	1339	512
miR1874-3p	LOC_Os08g33280.1	145	-21.44	145	-21.44	777147	24	2736	2550
miR1874-3p	LOC_Os08g33330.1	155	-21.45	155	-21.45	777151	24	1649	263
miR1874-3p	LOC_Os08g33710.1	148	-23.84	148	-23.84	777198	24	1420	118
miR1874-3p	LOC_Os08g33780.1	169	-28.72	169	-28.72	777206	24	1884	1449
miR1874-3p	LOC_Os08g33870.1	155	-21.03	155	-21.03	777218	24	2484	2200
miR1874-3p	LOC_Os08g34010.1	157	-24.23	157	-24.23	777230	24	1086	1047
miR1874-3p	LOC_Os08g34550.1	153	-20.92	153	-20.92	777291	24	2344	1934
miR1874-3p	LOC_Os08g34700.1	158	-20.56	158	-20.56	777308	24	1083	90
miR1874-3p	LOC_Os08g34860.1	153	-20.64	153	-20.64	777324	24	2139	315
miR1874-3p	LOC_Os08g35600.1	163	-28.05	163	-28.05	777411	24	1935	1168
miR1874-3p	LOC_Os08g35840.1	153	-20.28	153	-20.28	777437	24	579	324
miR1874-3p	LOC_Os08g36340.1	159	-21.07	159	-21.07	777495	24	2584	174
miR1874-3p	LOC_Os08g36940.1	156	-20.06	156	-20.06	777561	24	3165	180
miR1874-3p	LOC_Os08g37115.1	160	-26.7	160	-26.7	777578	24	1841	256
miR1874-3p	LOC_Os08g37400.1	159	-25.75	159	-25.75	777609	24	2058	533
miR1874-3p	LOC_Os08g37470.1	156	-25.25	156	-25.25	777622	24	1089	109
miR1874-3p	LOC_Os08g37800.1	153	-20.5	153	-20.5	777664	24	2315	197
miR1874-3p	LOC_Os08g37874.1	309	-47.16	163	-24.89	777671	24	1983	810 1800
miR1874-3p	LOC_Os08g37874.2	163	-24.89	163	-24.89	777672	24	1970	826
miR1874-3p	LOC_Os08g37874.3	163	-24.89	163	-24.89	777673	24	1630	1032
miR1874-3p	LOC_Os08g38050.1	154	-23.58	154	-23.58	777699	24	1224	326

miR1874-3p	LOC_Os08g38086.3	160	-20.31	160	-20.31	777703	24	2652	1566
miR1874-3p	LOC_Os08g38086.4	160	-20.31	160	-20.31	777704	24	2722	1569
miR1874-3p	LOC_Os08g38610.1	154	-20.53	154	-20.53	777766	24	1869	1526
miR1874-3p	LOC_Os08g39000.1	149	-20.65	149	-20.65	777822	24	1265	783
miR1874-3p	LOC_Os08g39070.1	163	-23.29	163	-23.29	777830	24	1541	153
miR1874-3p	LOC_Os08g39230.1	169	-28.66	169	-28.66	777850	24	684	11
miR1874-3p	LOC_Os08g39280.1	165	-23.26	165	-23.26	777856	24	993	280
miR1874-3p	LOC_Os08g39310.1	154	-20.64	154	-20.64	777862	24	1862	1
miR1874-3p	LOC_Os08g39370.1	169	-20.65	169	-20.65	777868	24	2532	309
miR1874-3p	LOC_Os08g39400.1	153	-20.99	153	-20.99	777871	24	414	229
miR1874-3p	LOC_Os08g39570.1	156	-20.87	156	-20.87	777892	24	4223	604
miR1874-3p	LOC_Os08g39810.1	176	-27.46	176	-27.46	777917	24	3127	2609
miR1874-3p	LOC_Os08g40240.1	153	-24.29	153	-24.29	777971	24	645	446
miR1874-3p	LOC_Os08g40360.1	169	-29.39	169	-29.39	777981	24	3891	1129
miR1874-3p	LOC_Os08g40420.1	157	-24.67	157	-24.67	777986	24	3593	1781
miR1874-3p	LOC_Os08g40420.2	157	-24.67	157	-24.67	777987	24	3744	1932
miR1874-3p	LOC_Os08g40430.1	157	-24.67	157	-24.67	777988	24	2237	286
miR1874-3p	LOC_Os08g40560.1	163	-22.14	163	-22.14	778003	24	2773	1306
miR1874-3p	LOC_Os08g40560.2	163	-22.14	163	-22.14	778004	24	2703	1306
miR1874-3p	LOC_Os08g40900.1	164	-21.5	164	-21.5	778052	24	4066	1966
miR1874-3p	LOC_Os08g40900.2	164	-21.5	164	-21.5	778053	24	3870	1966
miR1874-3p	LOC_Os08g41010.1	165	-28.62	165	-28.62	778065	24	1862	449
miR1874-3p	LOC_Os08g41010.2	165	-28.62	165	-28.62	778066	24	1789	438
miR1874-3p	LOC_Os08g41010.3	165	-28.62	165	-28.62	778067	24	1859	446
miR1874-3p	LOC_Os08g41010.4	165	-28.62	165	-28.62	778068	24	2016	446
miR1874-3p	LOC_Os08g41010.5	165	-28.62	165	-28.62	778069	24	1851	449
miR1874-3p	LOC_Os08g41010.6	165	-28.62	165	-28.62	778070	24	1848	446
miR1874-3p	LOC_Os08g41010.7	165	-28.62	165	-28.62	778071	24	1839	437
miR1874-3p	LOC_Os08g41580.1	154	-20.31	154	-20.31	778139	24	3520	764
miR1874-3p	LOC_Os08g41590.1	153	-23.83	153	-23.83	778140	24	1283	867
miR1874-3p	LOC_Os08g41620.1	162	-26.23	162	-26.23	778143	24	2253	818
miR1874-3p	LOC_Os08g41760.1	158	-22.09	158	-22.09	778156	24	1396	137

miR1874-3p	LOC_Os08g41830.1	162	-20.36	162	-20.36	778163	24	2080	52
miR1874-3p	LOC_Os08g41880.1	155	-20.79	155	-20.79	778169	24	2280	1
miR1874-3p	LOC_Os08g42110.1	178	-33.34	178	-33.34	778194	24	1069	103
miR1874-3p	LOC_Os08g42120.1	154	-23.58	154	-23.58	778195	24	4377	4148
miR1874-3p	LOC_Os08g42170.1	156	-23.19	156	-23.19	778200	24	421	312
miR1874-3p	LOC_Os08g42210.1	178	-33.34	178	-33.34	778204	24	1107	178
miR1874-3p	LOC_Os08g42290.1	178	-33.34	178	-33.34	778211	24	836	132
miR1874-3p	LOC_Os08g42640.1	147	-22.77	147	-22.77	778267	24	1906	382
miR1874-3p	LOC_Os08g42830.1	154	-23.58	154	-23.58	778292	24	2877	2498
miR1874-3p	LOC_Os08g43000.1	307	-46.38	156	-23.58	778311	24	3633	224 1235
miR1874-3p	LOC_Os08g43010.1	310	-46.64	157	-25.65	778312	24	3990	1181 1434
miR1874-3p	LOC_Os08g43050.1	159	-22.35	159	-22.35	778317	24	3966	1535
miR1874-3p	LOC_Os08g43110.1	155	-25.41	155	-25.41	778325	24	4524	148
miR1874-3p	LOC_Os08g43130.1	159	-20.37	159	-20.37	778327	24	4634	974
miR1874-3p	LOC_Os08g43160.1	165	-28.68	165	-28.68	778330	24	1959	667
miR1874-3p	LOC_Os08g43380.1	155	-28.77	155	-28.77	778355	24	2226	5
miR1874-3p	LOC_Os08g44050.1	165	-21.79	165	-21.79	778434	24	2319	176
miR1874-3p	LOC_Os08g44050.2	165	-21.79	165	-21.79	778435	24	2613	184
miR1874-3p	LOC_Os08g44050.3	165	-21.79	165	-21.79	778436	24	2730	184
miR1874-3p	LOC_Os08g44050.4	165	-21.79	165	-21.79	778437	24	2478	184
miR1874-3p	LOC_Os08g44990.1	169	-28.72	169	-28.72	778562	24	2748	2196
miR1874-3p	LOC_Os08g45080.1	158	-24.1	158	-24.1	778572	24	4445	4281
miR1874-3p	LOC_Os08g45140.1	159	-20.82	159	-20.82	778578	24	816	50
miR1874-3p	LOC_Os09g01110.1	153	-20.94	153	-20.94	778616	24	766	338
miR1874-3p	LOC_Os09g01480.1	154	-21.23	154	-21.23	778656	24	4980	3497
miR1874-3p	LOC_Os09g01550.1	156	-20.49	156	-20.49	778663	24	2502	280
miR1874-3p	LOC_Os09g01590.1	162	-20.63	162	-20.63	778667	24	1531	946
miR1874-3p	LOC_Os09g01810.1	154	-20.7	154	-20.7	778696	24	2481	281
miR1874-3p	LOC_Os09g02260.1	147	-20.66	147	-20.66	778742	24	2784	252
miR1874-3p	LOC_Os09g02360.1	170	-27.74	170	-27.74	778752	24	1835	377
miR1874-3p	LOC_Os09g02420.1	142	-24.93	142	-24.93	778761	24	402	260
miR1874-3p	LOC_Os09g02650.1	163	-20.05	163	-20.05	778782	24	4300	286

miR1874-3p	LOC_Os09g02690.1	147	-23.29	147	-23.29	778785	24	967	234
miR1874-3p	LOC_Os09g02690.2	147	-23.29	147	-23.29	778786	24	691	256
miR1874-3p	LOC_Os09g02790.1	163	-27.72	163	-27.72	778796	24	815	558
miR1874-3p	LOC_Os09g02990.1	141	-21.63	141	-21.63	778818	24	3912	3230
miR1874-3p	LOC_Os09g03110.1	156	-21.34	156	-21.34	778829	24	6336	2108
miR1874-3p	LOC_Os09g03140.1	158	-22.85	158	-22.85	778832	24	1579	1548
miR1874-3p	LOC_Os09g03380.1	162	-21.77	162	-21.77	778855	24	2151	1319
miR1874-3p	LOC_Os09g03630.1	140	-22.34	140	-22.34	778883	24	1785	1400
miR1874-3p	LOC_Os09g03890.1	149	-20.94	149	-20.94	778905	24	3406	1361
miR1874-3p	LOC_Os09g04080.1	156	-20.06	156	-20.06	778922	24	3105	180
miR1874-3p	LOC_Os09g04110.1	155	-21.89	155	-21.89	778925	24	4442	479
miR1874-3p	LOC_Os09g04110.2	155	-21.89	155	-21.89	778926	24	4455	479
miR1874-3p	LOC_Os09g04260.1	169	-28.72	169	-28.72	778943	24	5772	1641
miR1874-3p	LOC_Os09g04660.1	151	-20.05	151	-20.05	778987	24	2283	519
miR1874-3p	LOC_Os09g04760.1	158	-26.28	158	-26.28	778998	24	387	63
miR1874-3p	LOC_Os09g06560.1	154	-24.81	154	-24.81	779080	24	4298	1022
miR1874-3p	LOC_Os09g06710.1	161	-20.93	161	-20.93	779095	24	2241	1364
miR1874-3p	LOC_Os09g06850.1	154	-22.42	154	-22.42	779109	24	282	254
miR1874-3p	LOC_Os09g07180.1	160	-24.13	160	-24.13	779146	24	999	190
miR1874-3p	LOC_Os09g07250.1	154	-20.7	154	-20.7	779152	24	1977	209
miR1874-3p	LOC_Os09g07300.1	148	-24.73	148	-24.73	779158	24	13113	9507
miR1874-3p	LOC_Os09g07430.1	149	-21.45	149	-21.45	779174	24	3180	295
miR1874-3p	LOC_Os09g07540.1	154	-21.23	154	-21.23	779189	24	2370	770
miR1874-3p	LOC_Os09g07810.1	167	-24.61	167	-24.61	779219	24	531	437
miR1874-3p	LOC_Os09g07890.1	159	-24.23	159	-24.23	779231	24	3867	929
miR1874-3p	LOC_Os09g07980.1	150	-21.18	150	-21.18	779240	24	3567	3309
miR1874-3p	LOC_Os09g08000.1	147	-23.1	147	-23.1	779242	24	498	33
miR1874-3p	LOC_Os09g08670.1	153	-25.61	153	-25.61	779311	24	3189	1186
miR1874-3p	LOC_Os09g08940.1	150	-21.18	150	-21.18	779338	24	3349	3309
miR1874-3p	LOC_Os09g09100.1	153	-20.94	153	-20.94	779354	24	2691	2183
miR1874-3p	LOC_Os09g09650.1	156	-20.29	156	-20.29	779412	24	1759	11
miR1874-3p	LOC_Os09g09830.1	150	-21.08	150	-21.08	779430	24	802	563

miR1874-3p	LOC_Os09g09830.2	150	-21.08	150	-21.08	779431	24	796	563
miR1874-3p	LOC_Os09g10180.1	166	-30.76	166	-30.76	779462	24	513	1
miR1874-3p	LOC_Os09g10440.1	162	-24.09	162	-24.09	779491	24	495	274
miR1874-3p	LOC_Os09g10540.1	158	-21.02	158	-21.02	779500	24	4686	4180
miR1874-3p	LOC_Os09g10650.1	151	-22.68	151	-22.68	779510	24	4575	4554
miR1874-3p	LOC_Os09g11020.1	164	-20.17	164	-20.17	779555	24	3495	1526
miR1874-3p	LOC_Os09g11480.2	163	-21.19	163	-21.19	779603	24	1566	639
miR1874-3p	LOC_Os09g11610.1	157	-20.16	157	-20.16	779616	24	3738	3686
miR1874-3p	LOC_Os09g11820.1	154	-23.58	154	-23.58	779637	24	3594	3362
miR1874-3p	LOC_Os09g11850.1	148	-20.98	148	-20.98	779640	24	2022	664
miR1874-3p	LOC_Os09g11870.1	156	-21.66	156	-21.66	779642	24	2376	981
miR1874-3p	LOC_Os09g12350.1	146	-20.95	146	-20.95	779686	24	2001	1328
miR1874-3p	LOC_Os09g12660.1	157	-20.72	157	-20.72	779721	24	2029	1199
miR1874-3p	LOC_Os09g12660.2	157	-20.72	157	-20.72	779722	24	1973	1143
miR1874-3p	LOC_Os09g12830.1	169	-28.72	169	-28.72	779742	24	1362	1122
miR1874-3p	LOC_Os09g12870.1	168	-23.99	168	-23.99	779746	24	3423	3084
miR1874-3p	LOC_Os09g12890.1	153	-20.94	153	-20.94	779747	24	4776	4412
miR1874-3p	LOC_Os09g12930.1	176	-33.4	176	-33.4	779751	24	2970	1878
miR1874-3p	LOC_Os09g13070.1	147	-23.72	147	-23.72	779764	24	1485	992
miR1874-3p	LOC_Os09g13230.1	147	-23.72	147	-23.72	779780	24	4950	4415
miR1874-3p	LOC_Os09g14010.1	298	-43.19	157	-22.8	779865	24	3051	2849 466
miR1874-3p	LOC_Os09g14060.1	141	-22.8	141	-22.8	779869	24	2688	58
miR1874-3p	LOC_Os09g14360.1	154	-22.44	154	-22.44	779899	24	2286	2264
miR1874-3p	LOC_Os09g14470.1	156	-20.7	156	-20.7	779909	24	3540	3023
miR1874-3p	LOC_Os09g14670.1	162	-22.46	162	-22.46	779936	24	3449	405
miR1874-3p	LOC_Os09g15250.1	151	-20.36	151	-20.36	779995	24	5211	628
miR1874-3p	LOC_Os09g15680.1	168	-26.22	168	-26.22	780048	24	855	133
miR1874-3p	LOC_Os09g15810.1	156	-20.89	156	-20.89	780061	24	1405	699
miR1874-3p	LOC_Os09g15950.1	153	-21.71	153	-21.71	780077	24	2292	1870
miR1874-3p	LOC_Os09g16090.1	160	-20.96	160	-20.96	780091	24	1404	257
miR1874-3p	LOC_Os09g16200.1	157	-23.37	157	-23.37	780102	24	2175	1880
miR1874-3p	LOC_Os09g16270.1	159	-22.28	159	-22.28	780109	24	609	167

miR1874-3p	LOC_Os09g16330.2	320	-48.52	163	-27.15	780115	24	4341	3278 3662
miR1874-3p	LOC_Os09g16380.1	497	-76.71	170	-30.03	780120	24	4161	3491 973 3161
miR1874-3p	LOC_Os09g16458.1	163	-21.37	163	-21.37	780130	24	1474	422
miR1874-3p	LOC_Os09g16760.1	168	-26.76	168	-26.76	780160	24	2543	1230
miR1874-3p	LOC_Os09g17130.1	154	-20.17	154	-20.17	780199	24	1107	1
miR1874-3p	LOC_Os09g17200.1	160	-20.49	160	-20.49	780207	24	4554	4162
miR1874-3p	LOC_Os09g17250.1	154	-22.42	154	-22.42	780212	24	1587	1559
miR1874-3p	LOC_Os09g17329.1	313	-45.34	166	-23.47	780218	24	2957	1031 1718
miR1874-3p	LOC_Os09g17329.2	313	-45.34	166	-23.47	780219	24	2530	1005 1692
miR1874-3p	LOC_Os09g17400.1	154	-22.42	154	-22.42	780225	24	3903	3875
miR1874-3p	LOC_Os09g17490.1	141	-21.35	141	-21.35	780234	24	4302	1807
miR1874-3p	LOC_Os09g17920.1	151	-20.49	151	-20.49	780282	24	3453	3107
miR1874-3p	LOC_Os09g18250.1	166	-24.33	166	-24.33	780310	24	1119	542
miR1874-3p	LOC_Os09g18350.1	149	-21.35	149	-21.35	780320	24	786	21
miR1874-3p	LOC_Os09g19150.1	160	-20.25	160	-20.25	780347	24	2440	215
miR1874-3p	LOC_Os09g19150.2	160	-20.25	160	-20.25	780348	24	1254	215
miR1874-3p	LOC_Os09g19274.1	153	-20.99	153	-20.99	780358	24	2549	2425
miR1874-3p	LOC_Os09g19280.1	153	-20.99	153	-20.99	780359	24	1440	321
miR1874-3p	LOC_Os09g19430.1	153	-24.22	153	-24.22	780373	24	1269	1052
miR1874-3p	LOC_Os09g19530.1	151	-25.25	151	-25.25	780383	24	6054	2526
miR1874-3p	LOC_Os09g19540.1	141	-22.41	141	-22.41	780384	24	3144	2890
miR1874-3p	LOC_Os09g19640.1	156	-21.35	156	-21.35	780394	24	1159	713
miR1874-3p	LOC_Os09g19750.2	167	-27.48	167	-27.48	780406	24	1667	233
miR1874-3p	LOC_Os09g19850.1	163	-22.16	163	-22.16	780416	24	3395	3141
miR1874-3p	LOC_Os09g20410.1	164	-22.6	164	-22.6	780480	24	519	164
miR1874-3p	LOC_Os09g20650.1	158	-25.23	158	-25.23	780510	24	1661	1312
miR1874-3p	LOC_Os09g20650.2	158	-25.23	158	-25.23	780511	24	1695	1312
miR1874-3p	LOC_Os09g21110.1	318	-42.81	160	-22.41	780561	24	4023	1011 1551
miR1874-3p	LOC_Os09g21420.1	152	-20.14	152	-20.14	780593	24	3757	1663
miR1874-3p	LOC_Os09g21689.1	159	-21.49	159	-21.49	780621	24	1285	936
miR1874-3p	LOC_Os09g21689.2	159	-21.49	159	-21.49	780622	24	4777	4413
miR1874-3p	LOC_Os09g21689.3	159	-21.49	159	-21.49	780623	24	6566	6202

miR1874-3p	LOC_Os09g21930.1	153	-21.67	153	-21.67	780648	24	414	229
miR1874-3p	LOC_Os09g21980.1	161	-20.93	161	-20.93	780653	24	3444	1694
miR1874-3p	LOC_Os09g22060.1	146	-20.37	146	-20.37	780661	24	783	538
miR1874-3p	LOC_Os09g23360.1	150	-20.14	150	-20.14	780744	24	2175	1470
miR1874-3p	LOC_Os09g23360.2	150	-20.14	150	-20.14	780745	24	2386	1470
miR1874-3p	LOC_Os09g23740.1	146	-20.33	146	-20.33	780786	24	5435	2958
miR1874-3p	LOC_Os09g23860.1	158	-21.02	158	-21.02	780798	24	1908	1402
miR1874-3p	LOC_Os09g24220.1	160	-20.63	160	-20.63	780836	24	3831	1621
miR1874-3p	LOC_Os09g24700.1	154	-20.64	154	-20.64	780888	24	4185	3703
miR1874-3p	LOC_Os09g25290.1	158	-20.62	158	-20.62	780954	24	1807	452
miR1874-3p	LOC_Os09g25490.1	145	-23.69	145	-23.69	780976	24	3698	2304
miR1874-3p	LOC_Os09g25560.1	155	-26.73	155	-26.73	780983	24	2327	1126
miR1874-3p	LOC_Os09g25640.1	155	-20.24	155	-20.24	780995	24	2202	1537
miR1874-3p	LOC_Os09g25640.2	155	-20.24	155	-20.24	780996	24	2112	1447
miR1874-3p	LOC_Os09g25860.1	168	-25.02	168	-25.02	781015	24	1233	903
miR1874-3p	LOC_Os09g26530.1	155	-21.86	155	-21.86	781089	24	2273	325
miR1874-3p	LOC_Os09g26550.2	153	-22.03	153	-22.03	781092	24	2040	284
miR1874-3p	LOC_Os09g26830.1	160	-20.42	160	-20.42	781129	24	2121	207
miR1874-3p	LOC_Os09g27110.1	168	-23.75	168	-23.75	781161	24	2316	2294
miR1874-3p	LOC_Os09g27120.1	156	-22.33	156	-22.33	781162	24	444	65
miR1874-3p	LOC_Os09g27240.1	160	-28.94	160	-28.94	781176	24	1675	499
miR1874-3p	LOC_Os09g27240.2	160	-28.94	160	-28.94	781177	24	1451	305
miR1874-3p	LOC_Os09g27350.1	155	-25.77	155	-25.77	781187	24	3045	1754
miR1874-3p	LOC_Os09g27420.1	164	-22.94	164	-22.94	781195	24	2758	2292
miR1874-3p	LOC_Os09g27420.2	164	-22.94	164	-22.94	781196	24	2746	2280
miR1874-3p	LOC_Os09g27970.1	165	-20.31	165	-20.31	781275	24	2683	1471
miR1874-3p	LOC_Os09g27970.2	165	-20.31	165	-20.31	781276	24	2824	1142
miR1874-3p	LOC_Os09g27970.3	165	-20.31	165	-20.31	781277	24	3153	1471
miR1874-3p	LOC_Os09g27970.4	165	-20.31	165	-20.31	781278	24	3167	1471
miR1874-3p	LOC_Os09g27970.5	165	-20.31	165	-20.31	781279	24	3078	1471
miR1874-3p	LOC_Os09g28580.1	152	-20.95	152	-20.95	781343	24	566	259
miR1874-3p	LOC_Os09g28940.1	160	-23.29	160	-23.29	781387	24	2814	777

miR1874-3p	LOC_Os09g28950.1	156	-20.14	156	-20.14	781388	24	1131	1
miR1874-3p	LOC_Os09g28990.1	152	-22.11	152	-22.11	781392	24	753	298
miR1874-3p	LOC_Os09g29000.1	164	-24.18	164	-24.18	781393	24	3579	2198
miR1874-3p	LOC_Os09g29584.1	158	-20.2	158	-20.2	781457	24	2998	2673
miR1874-3p	LOC_Os09g30020.1	149	-23.53	149	-23.53	781511	24	4770	710
miR1874-3p	LOC_Os09g31230.1	147	-21.64	147	-21.64	781622	24	3196	2431
miR1874-3p	LOC_Os09g31230.2	147	-21.64	147	-21.64	781623	24	2945	2175
miR1874-3p	LOC_Os09g31310.1	162	-22.17	162	-22.17	781633	24	748	124
miR1874-3p	LOC_Os09g31390.1	174	-28.25	174	-28.25	781640	24	2479	244
miR1874-3p	LOC_Os09g31390.2	174	-28.25	174	-28.25	781641	24	2473	244
miR1874-3p	LOC_Os09g31390.3	330	-49.51	174	-28.25	781642	24	1654	167 1582
miR1874-3p	LOC_Os09g31990.1	169	-23.08	169	-23.08	781684	24	697	438
miR1874-3p	LOC_Os09g32010.1	144	-20.57	144	-20.57	781686	24	2779	1940
miR1874-3p	LOC_Os09g32010.2	144	-20.57	144	-20.57	781687	24	3027	1940
miR1874-3p	LOC_Os09g32300.1	163	-22.56	163	-22.56	781725	24	2763	674
miR1874-3p	LOC_Os09g32300.2	163	-22.56	163	-22.56	781726	24	2690	669
miR1874-3p	LOC_Os09g32300.3	163	-22.56	163	-22.56	781727	24	2993	669
miR1874-3p	LOC_Os09g32310.1	166	-20.06	166	-20.06	781728	24	1931	1423
miR1874-3p	LOC_Os09g32380.1	155	-21	155	-21	781735	24	3307	2309
miR1874-3p	LOC_Os09g32540.1	157	-20.47	157	-20.47	781757	24	3225	2433
miR1874-3p	LOC_Os09g32992.1	316	-48.68	158	-26.18	781825	24	2312	756 1632
miR1874-3p	LOC_Os09g33460.1	155	-24.42	155	-24.42	781828	24	2007	1872
miR1874-3p	LOC_Os09g33460.2	155	-24.42	155	-24.42	781829	24	1916	1781
miR1874-3p	LOC_Os09g33876.1	156	-25.51	156	-25.51	781887	24	2322	57
miR1874-3p	LOC_Os09g33940.2	159	-23.97	159	-23.97	781901	24	2264	188
miR1874-3p	LOC_Os09g33980.1	152	-23.58	152	-23.58	781905	24	4160	698
miR1874-3p	LOC_Os09g34060.1	146	-22.2	146	-22.2	781919	24	2099	1297
miR1874-3p	LOC_Os09g34180.1	145	-20.88	145	-20.88	781937	24	2888	939
miR1874-3p	LOC_Os09g35910.1	164	-20.86	164	-20.86	782030	24	1435	206
miR1874-3p	LOC_Os09g35920.1	162	-22.56	162	-22.56	782031	24	3110	1212
miR1874-3p	LOC_Os09g35920.2	162	-22.56	162	-22.56	782032	24	2390	1209
miR1874-3p	LOC_Os09g36110.1	152	-23.59	152	-23.59	782053	24	2310	1976

miR1874-3p	LOC_Os09g36220.1	146	-22.48	146	-22.48	782065	24	2416	1316
miR1874-3p	LOC_Os09g36220.2	146	-22.48	146	-22.48	782066	24	2475	1316
miR1874-3p	LOC_Os09g36600.2	166	-25.39	166	-25.39	782116	24	3728	1753
miR1874-3p	LOC_Os09g36760.1	160	-20.89	160	-20.89	782136	24	2341	49
miR1874-3p	LOC_Os09g37520.1	158	-22.27	158	-22.27	782226	24	4169	97
miR1874-3p	LOC_Os09g37590.1	154	-24.46	154	-24.46	782233	24	2880	376
miR1874-3p	LOC_Os09g37890.1	173	-28.69	173	-28.69	782266	24	2325	534
miR1874-3p	LOC_Os09g37930.1	154	-22.14	154	-22.14	782269	24	3690	1210
miR1874-3p	LOC_Os09g37967.1	160	-21.36	160	-21.36	782273	24	1380	4
miR1874-3p	LOC_Os09g37990.1	155	-20.32	155	-20.32	782275	24	624	283
miR1874-3p	LOC_Os09g38170.1	160	-21.58	160	-21.58	782297	24	1278	1048
miR1874-3p	LOC_Os09g38490.1	152	-20.15	152	-20.15	782326	24	1446	529
miR1874-3p	LOC_Os09g38630.1	154	-24.38	154	-24.38	782348	24	1578	1478
miR1874-3p	LOC_Os09g38870.1	158	-21.8	158	-21.8	782382	24	2070	356
miR1874-3p	LOC_Os09g39280.1	148	-20.1	148	-20.1	782425	24	1350	28
miR1874-3p	LOC_Os09g39320.1	300	-43.17	152	-23.07	782429	24	3747	28 1700
miR1874-3p	LOC_Os09g39462.3	152	-20.36	152	-20.36	782449	24	2867	1329
miR1874-3p	LOC_Os09g39920.1	164	-22.44	164	-22.44	782509	24	1973	1919
miR1874-3p	LOC_Os09g40060.1	156	-20.06	156	-20.06	782527	24	2988	153
miR1874-3p	LOC_Os09g40085.1	156	-20.06	156	-20.06	782529	24	1503	33
miR1874-3p	LOC_Os10g01550.1	158	-23.98	158	-23.98	782583	24	3145	286
miR1874-3p	LOC_Os10g01770.1	153	-20.76	153	-20.76	782605	24	3255	2539
miR1874-3p	LOC_Os10g01860.1	153	-22.49	153	-22.49	782615	24	867	345
miR1874-3p	LOC_Os10g01920.1	162	-25.9	162	-25.9	782621	24	1530	219
miR1874-3p	LOC_Os10g01950.1	167	-25.31	167	-25.31	782624	24	2889	1934
miR1874-3p	LOC_Os10g02520.1	154	-23.58	154	-23.58	782685	24	2307	1928
miR1874-3p	LOC_Os10g02960.1	151	-20.89	151	-20.89	782732	24	4353	1400
miR1874-3p	LOC_Os10g03220.1	155	-23.6	155	-23.6	782757	24	1292	1085
miR1874-3p	LOC_Os10g03340.1	150	-21.18	150	-21.18	782769	24	3345	3306
miR1874-3p	LOC_Os10g04060.1	152	-21.7	152	-21.7	782846	24	3825	3721
miR1874-3p	LOC_Os10g04316.1	169	-28.72	169	-28.72	782866	24	3447	1647
miR1874-3p	LOC_Os10g04850.1	158	-23.66	158	-23.66	782918	24	2187	467

miR1874-3p	LOC_Os10g05280.1	154	-23.01	154	-23.01	782964	24	324	302
miR1874-3p	LOC_Os10g06030.1	151	-21.04	151	-21.04	783041	24	2082	212
miR1874-3p	LOC_Os10g06090.1	151	-21.04	151	-21.04	783045	24	2247	218
miR1874-3p	LOC_Os10g06170.1	156	-25.26	156	-25.26	783055	24	5367	2136
miR1874-3p	LOC_Os10g06520.1	153	-20.94	153	-20.94	783090	24	4893	4172
miR1874-3p	LOC_Os10g07229.1	161	-23.98	161	-23.98	783155	24	1865	663
miR1874-3p	LOC_Os10g07248.1	163	-23.56	163	-23.56	783156	24	4383	1236
miR1874-3p	LOC_Os10g07520.1	158	-25.76	158	-25.76	783179	24	369	102
miR1874-3p	LOC_Os10g07578.1	148	-22.95	148	-22.95	783204	24	3957	1405
miR1874-3p	LOC_Os10g08290.1	157	-21.48	157	-21.48	783255	24	348	62
miR1874-3p	LOC_Os10g08530.1	159	-22.39	159	-22.39	783275	24	912	371
miR1874-3p	LOC_Os10g08750.1	162	-21.43	162	-21.43	783300	24	345	55
miR1874-3p	LOC_Os10g09070.1	156	-20.99	156	-20.99	783330	24	813	336
miR1874-3p	LOC_Os10g09190.1	155	-20.24	155	-20.24	783342	24	360	327
miR1874-3p	LOC_Os10g09240.1	154	-20.49	154	-20.49	783346	24	663	615
miR1874-3p	LOC_Os10g09310.1	149	-21.88	149	-21.88	783353	24	2082	1718
miR1874-3p	LOC_Os10g09440.1	153	-20.94	153	-20.94	783365	24	4985	4403
miR1874-3p	LOC_Os10g09460.1	153	-20.94	153	-20.94	783367	24	5337	4769
miR1874-3p	LOC_Os10g09529.1	148	-20.79	148	-20.79	783373	24	1596	1480
miR1874-3p	LOC_Os10g09700.1	151	-23.59	151	-23.59	783389	24	1395	366
miR1874-3p	LOC_Os10g09810.1	170	-23.25	170	-23.25	783398	24	3795	3677
miR1874-3p	LOC_Os10g09940.1	164	-20.4	164	-20.4	783413	24	3258	2074
miR1874-3p	LOC_Os10g09970.1	153	-20.94	153	-20.94	783416	24	3735	3245
miR1874-3p	LOC_Os10g10030.1	164	-26.57	164	-26.57	783421	24	3038	1971
miR1874-3p	LOC_Os10g10300.1	156	-20.7	156	-20.7	783455	24	4062	3539
miR1874-3p	LOC_Os10g10780.1	155	-20.27	155	-20.27	783503	24	3192	3160
miR1874-3p	LOC_Os10g11250.1	327	-52.04	169	-29.39	783548	24	3504	1057 2946
miR1874-3p	LOC_Os10g11340.1	159	-21.63	159	-21.63	783561	24	2921	91
miR1874-3p	LOC_Os10g11454.1	158	-21.02	158	-21.02	783568	24	5238	367
miR1874-3p	LOC_Os10g11580.1	169	-20.78	169	-20.78	783578	24	1449	1280
miR1874-3p	LOC_Os10g11870.1	152	-20.67	152	-20.67	783606	24	4845	1430
miR1874-3p	LOC_Os10g12050.1	161	-28.42	161	-28.42	783621	24	1900	1384

miR1874-3p	LOC_Os10g12050.2	161	-28.42	161	-28.42	783622	24	1774	1368
miR1874-3p	LOC_Os10g12149.1	161	-22.87	161	-22.87	783629	24	360	142
miR1874-3p	LOC_Os10g12200.1	158	-24.62	158	-24.62	783633	24	1344	12
miR1874-3p	LOC_Os10g12530.1	158	-21.02	158	-21.02	783657	24	3792	3352
miR1874-3p	LOC_Os10g12580.1	156	-20.37	156	-20.37	783662	24	1614	1558
miR1874-3p	LOC_Os10g12760.1	154	-22.42	154	-22.42	783679	24	3195	3167
miR1874-3p	LOC_Os10g12770.1	154	-22.42	154	-22.42	783680	24	5550	5522
miR1874-3p	LOC_Os10g13530.1	154	-21.74	154	-21.74	783710	24	5142	4648
miR1874-3p	LOC_Os10g13770.1	160	-20.49	160	-20.49	783737	24	2550	799
miR1874-3p	LOC_Os10g13870.1	154	-21.95	154	-21.95	783747	24	447	188
miR1874-3p	LOC_Os10g14960.1	153	-20.12	153	-20.12	783814	24	918	123
miR1874-3p	LOC_Os10g15079.1	154	-21.23	154	-21.23	783825	24	4815	1529
miR1874-3p	LOC_Os10g15370.1	161	-22.88	161	-22.88	783833	24	204	14
miR1874-3p	LOC_Os10g15250.1	157	-20.28	157	-20.28	783848	24	4992	981
miR1874-3p	LOC_Os10g16480.1	154	-22.07	154	-22.07	783868	24	5433	5411
miR1874-3p	LOC_Os10g16560.1	155	-21.33	155	-21.33	783876	24	7344	1545
miR1874-3p	LOC_Os10g16750.1	167	-23.96	167	-23.96	783893	24	3981	3275
miR1874-3p	LOC_Os10g17170.1	147	-23.72	147	-23.72	783932	24	4734	4307
miR1874-3p	LOC_Os10g17230.1	156	-20.49	156	-20.49	783936	24	2298	280
miR1874-3p	LOC_Os10g17440.1	147	-24.57	147	-24.57	783956	24	900	476
miR1874-3p	LOC_Os10g17690.1	145	-20.17	145	-20.17	783980	24	1884	1583
miR1874-3p	LOC_Os10g17900.1	147	-23.72	147	-23.72	784000	24	4785	4247
miR1874-3p	LOC_Os10g18294.1	158	-21.02	158	-21.02	784030	24	5052	4477
miR1874-3p	LOC_Os10g18560.1	156	-20.55	156	-20.55	784057	24	2970	1952
miR1874-3p	LOC_Os10g18660.1	158	-21.02	158	-21.02	784067	24	3585	370
miR1874-3p	LOC_Os10g18770.1	150	-22.15	150	-22.15	784077	24	2205	1268
miR1874-3p	LOC_Os10g18830.1	172	-26.05	172	-26.05	784083	24	2670	1756
miR1874-3p	LOC_Os10g19160.1	326	-48.45	174	-27.51	784113	24	3299	497 1385
miR1874-3p	LOC_Os10g19331.1	153	-22.96	153	-22.96	784131	24	2111	193
miR1874-3p	LOC_Os10g19331.2	153	-22.96	153	-22.96	784132	24	682	166
miR1874-3p	LOC_Os10g19902.1	166	-23.69	166	-23.69	784147	24	237	212
miR1874-3p	LOC_Os10g20030.1	154	-22.44	154	-22.44	784167	24	3138	3116

miR1874-3p	LOC_Os10g20190.1	158	-21.02	158	-21.02	784181	24	5052	4624
miR1874-3p	LOC_Os10g20480.1	151	-21.3	151	-21.3	784214	24	2566	2382
miR1874-3p	LOC_Os10g20480.2	151	-21.3	151	-21.3	784215	24	2676	2492
miR1874-3p	LOC_Os10g20480.3	151	-21.3	151	-21.3	784216	24	3911	1743
miR1874-3p	LOC_Os10g20920.1	157	-20.49	157	-20.49	784258	24	1503	280
miR1874-3p	LOC_Os10g21000.1	157	-24.01	157	-24.01	784266	24	3436	1976
miR1874-3p	LOC_Os10g21540.1	145	-20.31	145	-20.31	784353	24	1272	749
miR1874-3p	LOC_Os10g21810.1	146	-22.71	146	-22.71	784378	24	3561	222
miR1874-3p	LOC_Os10g21810.2	146	-22.71	146	-22.71	784379	24	5006	1030
miR1874-3p	LOC_Os10g21920.1	155	-21.72	155	-21.72	784391	24	1539	860
miR1874-3p	LOC_Os10g21940.1	159	-21.45	159	-21.45	784394	24	6276	5248
miR1874-3p	LOC_Os10g21950.1	176	-33.4	176	-33.4	784395	24	5655	4053
miR1874-3p	LOC_Os10g22270.1	145	-24.97	145	-24.97	784430	24	228	143
miR1874-3p	LOC_Os10g22350.1	147	-20.19	147	-20.19	784439	24	1287	485
miR1874-3p	LOC_Os10g22460.1	154	-21.11	154	-21.11	784452	24	1970	11
miR1874-3p	LOC_Os10g22630.1	158	-22.82	158	-22.82	784469	24	1089	308
miR1874-3p	LOC_Os10g22630.2	158	-22.82	158	-22.82	784470	24	1030	391
miR1874-3p	LOC_Os10g22730.1	159	-22.62	159	-22.62	784479	24	1981	344
miR1874-3p	LOC_Os10g22730.2	159	-22.62	159	-22.62	784480	24	2376	344
miR1874-3p	LOC_Os10g22730.3	159	-22.62	159	-22.62	784481	24	2261	229
miR1874-3p	LOC_Os10g22730.4	159	-22.62	159	-22.62	784482	24	2367	344
miR1874-3p	LOC_Os10g22869.1	165	-22.25	165	-22.25	784495	24	4109	2583
miR1874-3p	LOC_Os10g22869.2	165	-22.25	165	-22.25	784496	24	4119	2583
miR1874-3p	LOC_Os10g22869.3	165	-22.25	165	-22.25	784497	24	4319	2793
miR1874-3p	LOC_Os10g22860.1	165	-22.25	165	-22.25	784498	24	3204	2176
miR1874-3p	LOC_Os10g22910.1	149	-20.64	149	-20.64	784502	24	231	48
miR1874-3p	LOC_Os10g22950.1	174	-22.68	174	-22.68	784506	24	3560	844
miR1874-3p	LOC_Os10g23050.1	154	-22.05	154	-22.05	784518	24	1425	576
miR1874-3p	LOC_Os10g23190.1	154	-23.58	154	-23.58	784534	24	2772	2393
miR1874-3p	LOC_Os10g23270.1	151	-25.6	151	-25.6	784542	24	981	924
miR1874-3p	LOC_Os10g24230.1	161	-20.71	161	-20.71	784592	24	3234	1805
miR1874-3p	LOC_Os10g24450.1	164	-20.07	164	-20.07	784613	24	2798	1463

miR1874-3p	LOC_Os10g24470.1	161	-21.16	161	-21.16	784615	24	1452	822
miR1874-3p	LOC_Os10g24810.1	162	-22.42	162	-22.42	784649	24	1946	1528
miR1874-3p	LOC_Os10g24870.1	159	-21.65	159	-21.65	784655	24	435	175
miR1874-3p	LOC_Os10g24890.1	159	-20.64	159	-20.64	784657	24	429	276
miR1874-3p	LOC_Os10g25040.1	157	-23.45	157	-23.45	784669	24	3692	2556
miR1874-3p	LOC_Os10g25140.1	159	-20.87	159	-20.87	784679	24	1745	23
miR1874-3p	LOC_Os10g25450.1	163	-27.17	163	-27.17	784710	24	2843	456
miR1874-3p	LOC_Os10g25980.1	150	-21.18	150	-21.18	784762	24	3468	3309
miR1874-3p	LOC_Os10g26650.1	160	-27.59	160	-27.59	784834	24	3813	1646
miR1874-3p	LOC_Os10g26810.1	151	-24.51	151	-24.51	784853	24	1140	26
miR1874-3p	LOC_Os10g27460.1	149	-21.5	149	-21.5	784921	24	555	18
miR1874-3p	LOC_Os10g27480.1	157	-20.64	157	-20.64	784923	24	2679	227
miR1874-3p	LOC_Os10g28130.1	149	-21.65	149	-21.65	784942	24	642	239
miR1874-3p	LOC_Os10g28310.1	311	-42.05	162	-21.28	784960	24	4836	3887 1006
miR1874-3p	LOC_Os10g28350.1	153	-20.01	153	-20.01	784970	24	943	773
miR1874-3p	LOC_Os10g28510.1	173	-37.87	173	-37.87	784986	24	4305	4165
miR1874-3p	LOC_Os10g28510.2	173	-37.87	173	-37.87	784987	24	4403	4260
miR1874-3p	LOC_Os10g28665.1	305	-46.83	155	-26.3	785007	24	2384	2039 90
miR1874-3p	LOC_Os10g28665.2	305	-46.83	155	-26.3	785008	24	2888	2245 133
miR1874-3p	LOC_Os10g28975.1	163	-23.56	163	-23.56	785036	24	775	325
miR1874-3p	LOC_Os10g29070.1	148	-21.99	148	-21.99	785045	24	2244	2109
miR1874-3p	LOC_Os10g29390.1	150	-22.05	150	-22.05	785075	24	1280	258
miR1874-3p	LOC_Os10g30460.1	150	-20.6	150	-20.6	785183	24	1077	1053
miR1874-3p	LOC_Os10g30540.1	149	-23.55	149	-23.55	785190	24	2381	1537
miR1874-3p	LOC_Os10g30630.1	154	-23.58	154	-23.58	785202	24	261	164
miR1874-3p	LOC_Os10g30800.1	161	-23.08	161	-23.08	785217	24	990	52
miR1874-3p	LOC_Os10g31400.1	150	-21.2	150	-21.2	785282	24	495	468
miR1874-3p	LOC_Os10g31864.1	304	-41.01	152	-20.67	785330	24	11520	1492 10361
miR1874-3p	LOC_Os10g32124.1	149	-22.12	149	-22.12	785352	24	6339	575
miR1874-3p	LOC_Os10g32710.1	163	-21.65	163	-21.65	785400	24	2967	997
miR1874-3p	LOC_Os10g32710.2	163	-21.65	163	-21.65	785401	24	2972	1002
miR1874-3p	LOC_Os10g32710.3	163	-21.65	163	-21.65	785402	24	3050	1080

miR1874-3p	LOC_Os10g33350.1	140	-24.05	140	-24.05	785469	24	1081	627
miR1874-3p	LOC_Os10g33350.3	140	-24.05	140	-24.05	785470	24	1215	761
miR1874-3p	LOC_Os10g33660.1	158	-20.21	158	-20.21	785503	24	1121	199
miR1874-3p	LOC_Os10g33740.1	153	-21.01	153	-21.01	785514	24	2817	2540
miR1874-3p	LOC_Os10g33900.1	154	-21.97	154	-21.97	785537	24	1350	591
miR1874-3p	LOC_Os10g33970.1	150	-21.61	150	-21.61	785550	24	2342	330
miR1874-3p	LOC_Os10g33970.2	304	-42.42	154	-21.61	785551	24	2007	1981 330
miR1874-3p	LOC_Os10g34220.1	148	-36.84	148	-36.84	785574	24	1970	1188
miR1874-3p	LOC_Os10g34230.1	311	-46.61	160	-23.43	785575	24	2800	705 1526
miR1874-3p	LOC_Os10g34430.1	148	-23.32	148	-23.32	785596	24	5198	590
miR1874-3p	LOC_Os10g34520.1	155	-25.72	155	-25.72	785603	24	804	697
miR1874-3p	LOC_Os10g34560.1	145	-24.27	145	-24.27	785607	24	627	528
miR1874-3p	LOC_Os10g34660.1	153	-20.5	153	-20.5	785620	24	1582	82
miR1874-3p	LOC_Os10g34670.2	173	-24.6	173	-24.6	785622	24	1978	809
miR1874-3p	LOC_Os10g34960.1	147	-22.17	147	-22.17	785651	24	924	759
miR1874-3p	LOC_Os10g35080.1	164	-23.61	164	-23.61	785668	24	753	10
miR1874-3p	LOC_Os10g35540.1	164	-23.63	164	-23.63	785728	24	1200	522
miR1874-3p	LOC_Os10g35930.1	151	-20.37	151	-20.37	785773	24	1272	894
miR1874-3p	LOC_Os10g36250.1	157	-23.8	157	-23.8	785806	24	2725	1323
miR1874-3p	LOC_Os10g36550.1	164	-23.81	164	-23.81	785838	24	2075	600
miR1874-3p	LOC_Os10g36550.2	164	-23.81	164	-23.81	785839	24	2117	642
miR1874-3p	LOC_Os10g36550.3	164	-23.81	164	-23.81	785840	24	2241	766
miR1874-3p	LOC_Os10g36770.1	156	-29.45	156	-29.45	785863	24	405	93
miR1874-3p	LOC_Os10g36810.1	150	-21.66	150	-21.66	785869	24	5532	111
miR1874-3p	LOC_Os10g37090.1	152	-20.02	152	-20.02	785891	24	2039	1276
miR1874-3p	LOC_Os10g37410.1	157	-22.16	157	-22.16	785914	24	2244	34
miR1874-3p	LOC_Os10g37740.1	160	-22.99	160	-22.99	785955	24	2192	402
miR1874-3p	LOC_Os10g37880.1	149	-24.48	149	-24.48	785976	24	1445	1010
miR1874-3p	LOC_Os10g37880.2	149	-24.48	149	-24.48	785977	24	1527	1092
miR1874-3p	LOC_Os10g37880.3	149	-24.48	149	-24.48	785978	24	1526	1091
miR1874-3p	LOC_Os10g37880.4	149	-24.48	149	-24.48	785979	24	1348	913
miR1874-3p	LOC_Os10g38664.1	159	-20.14	159	-20.14	786048	24	345	29

miR1874-3p	LOC_Os10g38890.1	156	-20.86	156	-20.86	786069	24	2308	1675
miR1874-3p	LOC_Os10g39020.1	145	-21.19	145	-21.19	786088	24	2772	2312
miR1874-3p	LOC_Os10g39020.2	145	-21.19	145	-21.19	786089	24	2769	2309
miR1874-3p	LOC_Os10g39500.1	162	-24.55	162	-24.55	786147	24	3386	1005
miR1874-3p	LOC_Os10g39610.1	157	-20.01	157	-20.01	786160	24	681	333
miR1874-3p	LOC_Os10g39610.2	157	-20.01	157	-20.01	786161	24	660	312
miR1874-3p	LOC_Os10g39640.1	151	-20.76	151	-20.76	786169	24	1653	1166
miR1874-3p	LOC_Os10g39670.1	154	-21.38	154	-21.38	786171	24	2132	1227
miR1874-3p	LOC_Os10g40324.1	175	-27.18	175	-27.18	786264	24	4651	3133
miR1874-3p	LOC_Os10g40840.1	301	-45.11	154	-25.08	786316	24	2238	1171 1843
miR1874-3p	LOC_Os10g40859.1	158	-20.5	158	-20.5	786317	24	2721	917
miR1874-3p	LOC_Os10g41000.1	161	-21.66	161	-21.66	786333	24	2720	883
miR1874-3p	LOC_Os10g41650.1	148	-20.82	148	-20.82	786407	24	1733	456
miR1874-3p	LOC_Os10g41770.1	169	-25.08	169	-25.08	786424	24	3671	1221
miR1874-3p	LOC_Os10g41999.1	153	-24.8	153	-24.8	786458	24	918	866
miR1874-3p	LOC_Os10g42490.1	175	-32.43	175	-32.43	786515	24	3751	2127
miR1874-3p	LOC_Os10g42490.2	175	-32.43	175	-32.43	786516	24	3635	2127
miR1874-3p	LOC_Os10g42510.1	157	-20.71	157	-20.71	786518	24	3999	1017
miR1874-3p	LOC_Os10g42650.1	149	-21.12	149	-21.12	786534	24	1953	1810
miR1874-3p	LOC_Os10g42840.1	154	-22.15	154	-22.15	786566	24	832	429
miR1874-3p	LOC_Os10g42840.2	154	-22.15	154	-22.15	786567	24	611	208
miR1874-3p	LOC_Os10g42850.1	154	-20.29	154	-20.29	786568	24	1068	609
miR1874-3p	LOC_Os10g42960.1	158	-20.44	158	-20.44	786585	24	2533	2272
miR1874-3p	LOC_Os10g43075.1	170	-29.26	170	-29.26	786595	24	5631	3458
miR1874-3p	LOC_Os10g43075.2	170	-29.26	170	-29.26	786596	24	5718	3545
miR1874-3p	LOC_Os11g01340.2	167	-26.9	167	-26.9	786637	24	1851	1200
miR1874-3p	LOC_Os11g01340.3	167	-26.9	167	-26.9	786638	24	1847	1209
miR1874-3p	LOC_Os11g01340.4	167	-26.9	167	-26.9	786639	24	1931	1200
miR1874-3p	LOC_Os11g01875.3	152	-23.44	152	-23.44	786702	24	3492	200
miR1874-3p	LOC_Os11g01875.4	152	-23.44	152	-23.44	786703	24	3431	200
miR1874-3p	LOC_Os11g01875.5	152	-23.44	152	-23.44	786704	24	3651	200
miR1874-3p	LOC_Os11g02290.1	160	-20.6	160	-20.6	786749	24	873	288

miR1874-3p	LOC_Os11g02550.1	162	-23.84	162	-23.84	786785	24	3584	2534
miR1874-3p	LOC_Os11g02600.1	158	-21.38	158	-21.38	786789	24	1497	673
miR1874-3p	LOC_Os11g02770.1	152	-21.56	152	-21.56	786810	24	450	113
miR1874-3p	LOC_Os11g02800.1	158	-22.68	158	-22.68	786815	24	582	446
miR1874-3p	LOC_Os11g02900.1	151	-26.63	151	-26.63	786826	24	948	805
miR1874-3p	LOC_Os11g03050.1	149	-22.84	149	-22.84	786839	24	1871	1441
miR1874-3p	LOC_Os11g03100.1	156	-20.06	156	-20.06	786845	24	3207	180
miR1874-3p	LOC_Os11g03360.1	153	-20.72	153	-20.72	786873	24	1563	1223
miR1874-3p	LOC_Os11g03400.1	146	-20.07	146	-20.07	786878	24	900	162
miR1874-3p	LOC_Os11g03580.1	155	-21.96	155	-21.96	786900	24	1047	371
miR1874-3p	LOC_Os11g03710.1	147	-27.23	147	-27.23	786913	24	564	449
miR1874-3p	LOC_Os11g03810.1	154	-20.58	154	-20.58	786926	24	877	683
miR1874-3p	LOC_Os11g03940.1	154	-20.39	154	-20.39	786939	24	6084	1261
miR1874-3p	LOC_Os11g03960.1	154	-24.99	154	-24.99	786941	24	4317	3506
miR1874-3p	LOC_Os11g04000.1	145	-20.18	145	-20.18	786946	24	543	519
miR1874-3p	LOC_Os11g04010.1	145	-20.18	145	-20.18	786947	24	1653	281
miR1874-3p	LOC_Os11g04104.1	161	-21.74	161	-21.74	786959	24	2299	1133
miR1874-3p	LOC_Os11g04104.2	161	-21.74	161	-21.74	786960	24	2315	1149
miR1874-3p	LOC_Os11g04104.3	161	-21.74	161	-21.74	786961	24	2231	1149
miR1874-3p	LOC_Os11g04730.1	167	-23.38	167	-23.38	787039	24	1043	319
miR1874-3p	LOC_Os11g04810.1	155	-20.16	155	-20.16	787047	24	390	206
miR1874-3p	LOC_Os11g05140.1	145	-20.13	145	-20.13	787091	24	1564	734
miR1874-3p	LOC_Os11g05310.1	165	-21.07	165	-21.07	787110	24	931	688
miR1874-3p	LOC_Os11g05650.1	160	-23.12	160	-23.12	787149	24	1541	984
miR1874-3p	LOC_Os11g05700.1	159	-22.98	159	-22.98	787155	24	2787	2158
miR1874-3p	LOC_Os11g05760.1	168	-30	168	-30	787161	24	1802	1144
miR1874-3p	LOC_Os11g05920.1	161	-27.79	161	-27.79	787177	24	3408	3243
miR1874-3p	LOC_Os11g06010.1	142	-26.05	142	-26.05	787192	24	3607	761
miR1874-3p	LOC_Os11g06130.1	297	-43.63	149	-22.02	787204	24	3018	1825 1910
miR1874-3p	LOC_Os11g06130.2	297	-43.63	149	-22.02	787205	24	3061	1912 1997
miR1874-3p	LOC_Os11g06440.1	166	-22.73	166	-22.73	787248	24	1855	6
miR1874-3p	LOC_Os11g06700.1	169	-27.25	169	-27.25	787275	24	2273	2140

miR1874-3p	LOC_Os11g06770.1	162	-20.69	162	-20.69	787283	24	2381	1616
miR1874-3p	LOC_Os11g06780.1	154	-23.65	154	-23.65	787285	24	1472	1110
miR1874-3p	LOC_Os11g06900.1	147	-24.66	147	-24.66	787298	24	2321	1488
miR1874-3p	LOC_Os11g07020.1	162	-21.67	162	-21.67	787315	24	1757	1410
miR1874-3p	LOC_Os11g07020.4	162	-21.67	162	-21.67	787316	24	1738	1391
miR1874-3p	LOC_Os11g07020.5	162	-21.67	162	-21.67	787317	24	1858	1511
miR1874-3p	LOC_Os11g07020.6	162	-21.67	162	-21.67	787318	24	1086	739
miR1874-3p	LOC_Os11g07020.7	162	-21.67	162	-21.67	787319	24	1933	1586
miR1874-3p	LOC_Os11g07170.1	161	-22.61	161	-22.61	787340	24	3012	2626
miR1874-3p	LOC_Os11g07180.1	171	-27.48	171	-27.48	787341	24	3355	2712
miR1874-3p	LOC_Os11g07230.1	155	-20.92	155	-20.92	787347	24	3946	2082
miR1874-3p	LOC_Os11g07230.2	155	-20.92	155	-20.92	787348	24	3586	1664
miR1874-3p	LOC_Os11g07230.3	155	-20.92	155	-20.92	787349	24	3617	1695
miR1874-3p	LOC_Os11g07280.1	146	-20.13	146	-20.13	787354	24	3414	2288
miR1874-3p	LOC_Os11g07280.2	146	-20.13	146	-20.13	787355	24	4176	3050
miR1874-3p	LOC_Os11g07310.1	169	-22.5	169	-22.5	787358	24	573	161
miR1874-3p	LOC_Os11g07350.1	157	-21.95	157	-21.95	787362	24	993	52
miR1874-3p	LOC_Os11g07470.1	152	-25.52	152	-25.52	787378	24	4323	1751
miR1874-3p	LOC_Os11g07912.1	160	-22.04	160	-22.04	787424	24	2183	863
miR1874-3p	LOC_Os11g08080.1	170	-25.25	170	-25.25	787441	24	2737	1867
miR1874-3p	LOC_Os11g08080.2	170	-25.25	170	-25.25	787442	24	2221	1857
miR1874-3p	LOC_Os11g08200.1	149	-22.6	149	-22.6	787455	24	1693	463
miR1874-3p	LOC_Os11g08265.1	156	-20.44	156	-20.44	787464	24	315	285
miR1874-3p	LOC_Os11g08390.1	152	-20.78	152	-20.78	787479	24	1512	1492
miR1874-3p	LOC_Os11g08550.1	157	-21.9	157	-21.9	787497	24	3906	2351
miR1874-3p	LOC_Os11g09250.1	156	-20.06	156	-20.06	787567	24	3165	180
miR1874-3p	LOC_Os11g09260.1	153	-22.21	153	-22.21	787568	24	1887	1203
miR1874-3p	LOC_Os11g09280.1	157	-22.92	157	-22.92	787570	24	1966	555
miR1874-3p	LOC_Os11g09280.2	157	-22.92	157	-22.92	787571	24	2045	634
miR1874-3p	LOC_Os11g09590.1	157	-22.5	157	-22.5	787598	24	1520	1207
miR1874-3p	LOC_Os11g09600.1	156	-23.15	156	-23.15	787599	24	1242	867
miR1874-3p	LOC_Os11g09720.1	153	-22.32	153	-22.32	787614	24	2031	110

miR1874-3p	LOC_Os11g09900.1	141	-20.89	141	-20.89	787636	24	1664	500
miR1874-3p	LOC_Os11g10160.1	148	-25.93	148	-25.93	787664	24	597	356
miR1874-3p	LOC_Os11g10200.1	160	-21.26	160	-21.26	787668	24	1903	611
miR1874-3p	LOC_Os11g10420.1	315	-44.1	162	-24.05	787690	24	4891	857 1867
miR1874-3p	LOC_Os11g10420.2	315	-44.1	162	-24.05	787691	24	5159	857 2135
miR1874-3p	LOC_Os11g10510.1	157	-24.37	157	-24.37	787702	24	1702	657
miR1874-3p	LOC_Os11g11070.1	145	-21.31	145	-21.31	787760	24	2772	669
miR1874-3p	LOC_Os11g11070.2	145	-21.31	145	-21.31	787761	24	3376	665
miR1874-3p	LOC_Os11g11180.1	160	-23.12	160	-23.12	787772	24	804	21
miR1874-3p	LOC_Os11g11510.1	316	-50.83	161	-25.64	787806	24	975	443 887
miR1874-3p	LOC_Os11g11940.1	152	-20.89	152	-20.89	787848	24	3509	2686
miR1874-3p	LOC_Os11g11950.1	153	-21.84	153	-21.84	787849	24	2325	177
miR1874-3p	LOC_Os11g11960.1	161	-24.71	161	-24.71	787850	24	2661	2164
miR1874-3p	LOC_Os11g12070.1	153	-20.94	153	-20.94	787861	24	766	338
miR1874-3p	LOC_Os11g12120.1	154	-21.23	154	-21.23	787866	24	3297	656
miR1874-3p	LOC_Os11g12140.1	153	-20.94	153	-20.94	787868	24	766	338
miR1874-3p	LOC_Os11g12360.1	159	-20.17	159	-20.17	787892	24	6948	6415
miR1874-3p	LOC_Os11g12870.1	154	-22.07	154	-22.07	787943	24	5388	5366
miR1874-3p	LOC_Os11g13640.1	320	-40.67	160	-20.49	787967	24	4701	2099 4456
miR1874-3p	LOC_Os11g13810.1	155	-20.91	155	-20.91	787985	24	3384	2536
miR1874-3p	LOC_Os11g13920.1	157	-20.21	157	-20.21	788002	24	6474	4084
miR1874-3p	LOC_Os11g14050.1	154	-25.51	154	-25.51	788017	24	3664	3355
miR1874-3p	LOC_Os11g14050.2	154	-25.51	154	-25.51	788018	24	3630	3321
miR1874-3p	LOC_Os11g14090.1	157	-28.9	157	-28.9	788022	24	6369	3554
miR1874-3p	LOC_Os11g14400.1	155	-20.16	155	-20.16	788053	24	1463	413
miR1874-3p	LOC_Os11g14520.1	157	-21.02	157	-21.02	788066	24	1716	1338
miR1874-3p	LOC_Os11g14570.1	156	-22.36	156	-22.36	788077	24	2735	1939
miR1874-3p	LOC_Os11g14570.2	156	-22.36	156	-22.36	788078	24	2726	1939
miR1874-3p	LOC_Os11g14570.3	156	-22.36	156	-22.36	788079	24	1823	1049
miR1874-3p	LOC_Os11g14570.4	156	-22.36	156	-22.36	788080	24	1845	1049
miR1874-3p	LOC_Os11g14910.1	162	-28.74	162	-28.74	788117	24	1470	1384
miR1874-3p	LOC_Os11g15030.1	153	-21.37	153	-21.37	788129	24	1164	931

miR1874-3p	LOC_Os11g15140.1	151	-24.59	151	-24.59	788144	24	1779	1011
miR1874-3p	LOC_Os11g15710.1	158	-20.81	158	-20.81	788199	24	291	6
miR1874-3p	LOC_Os11g16290.3	180	-33	180	-33	788212	24	3014	2775
miR1874-3p	LOC_Os11g16290.4	180	-33	180	-33	788213	24	2918	2685
miR1874-3p	LOC_Os11g16420.1	162	-20.26	162	-20.26	788236	24	1437	462
miR1874-3p	LOC_Os11g16420.2	162	-20.26	162	-20.26	788237	24	1468	549
miR1874-3p	LOC_Os11g17090.1	159	-22.27	159	-22.27	788302	24	774	713
miR1874-3p	LOC_Os11g18044.1	164	-24.89	164	-24.89	788391	24	2223	291
miR1874-3p	LOC_Os11g18120.1	161	-22.82	161	-22.82	788396	24	3045	254
miR1874-3p	LOC_Os11g18194.1	156	-20.31	156	-20.31	788401	24	2582	1759
miR1874-3p	LOC_Os11g18320.1	158	-21.83	158	-21.83	788412	24	3471	3026
miR1874-3p	LOC_Os11g18810.1	161	-24.75	161	-24.75	788459	24	4776	552
miR1874-3p	LOC_Os11g18947.1	160	-23.6	160	-23.6	788474	24	1521	627
miR1874-3p	LOC_Os11g18947.2	160	-23.6	160	-23.6	788475	24	2921	627
miR1874-3p	LOC_Os11g19350.1	155	-22.97	155	-22.97	788517	24	2732	1225
miR1874-3p	LOC_Os11g19760.1	163	-26.1	163	-26.1	788560	24	267	220
miR1874-3p	LOC_Os11g19780.1	149	-20.83	149	-20.83	788565	24	305	122
miR1874-3p	LOC_Os11g19810.1	292	-40.18	146	-20.09	788568	24	3438	1966 2230
miR1874-3p	LOC_Os11g19810.2	292	-40.18	146	-20.09	788569	24	3642	1972 2236
miR1874-3p	LOC_Os11g19840.1	157	-22.36	157	-22.36	788572	24	1404	350
miR1874-3p	LOC_Os11g20260.1	166	-23.98	166	-23.98	788612	24	1242	719
miR1874-3p	LOC_Os11g20300.1	156	-20.06	156	-20.06	788616	24	3027	153
miR1874-3p	LOC_Os11g20510.1	157	-20.28	157	-20.28	788637	24	3612	729
miR1874-3p	LOC_Os11g22090.1	164	-21.89	164	-21.89	788694	24	552	81
miR1874-3p	LOC_Os11g22336.1	156	-21.34	156	-21.34	788722	24	2916	1859
miR1874-3p	LOC_Os11g22810.1	157	-20.28	157	-20.28	788768	24	1029	609
miR1874-3p	LOC_Os11g22820.1	162	-20.19	162	-20.19	788769	24	417	214
miR1874-3p	LOC_Os11g22830.1	150	-21	150	-21	788770	24	891	752
miR1874-3p	LOC_Os11g23830.1	175	-35.53	175	-35.53	788832	24	1689	214
miR1874-3p	LOC_Os11g24180.1	153	-23.34	153	-23.34	788870	24	1581	24
miR1874-3p	LOC_Os11g24320.1	167	-24.11	167	-24.11	788882	24	486	414
miR1874-3p	LOC_Os11g24540.1	153	-20.96	153	-20.96	788901	24	2225	45

miR1874-3p	LOC_Os11g24540.2	153	-20.96	153	-20.96	788902	24	2398	45
miR1874-3p	LOC_Os11g25440.1	143	-21.61	143	-21.61	788986	24	693	276
miR1874-3p	LOC_Os11g25670.1	159	-24.23	159	-24.23	789007	24	3792	1628
miR1874-3p	LOC_Os11g25980.1	153	-21.01	153	-21.01	789034	24	3810	56
miR1874-3p	LOC_Os11g25980.2	153	-21.01	153	-21.01	789035	24	3555	56
miR1874-3p	LOC_Os11g26130.1	159	-22.73	159	-22.73	789050	24	3392	2096
miR1874-3p	LOC_Os11g26300.1	144	-20.13	144	-20.13	789065	24	2574	1396
miR1874-3p	LOC_Os11g27400.1	151	-20.79	151	-20.79	789171	24	1594	184
miR1874-3p	LOC_Os11g27600.1	158	-21.3	158	-21.3	789191	24	570	56
miR1874-3p	LOC_Os11g27610.1	169	-28.72	169	-28.72	789192	24	2499	1935
miR1874-3p	LOC_Os11g27730.1	155	-20.39	155	-20.39	789204	24	1931	1866
miR1874-3p	LOC_Os11g27730.2	155	-20.39	155	-20.39	789205	24	1855	1790
miR1874-3p	LOC_Os11g27870.1	157	-20.1	157	-20.1	789219	24	1473	569
miR1874-3p	LOC_Os11g28060.1	158	-26.36	158	-26.36	789236	24	846	387
miR1874-3p	LOC_Os11g28150.1	150	-21.18	150	-21.18	789243	24	3405	3309
miR1874-3p	LOC_Os11g28184.1	145	-22.02	145	-22.02	789245	24	3674	86
miR1874-3p	LOC_Os11g28184.2	145	-22.02	145	-22.02	789246	24	2914	86
miR1874-3p	LOC_Os11g28184.3	145	-22.02	145	-22.02	789247	24	3812	86
miR1874-3p	LOC_Os11g28540.1	163	-22.52	163	-22.52	789288	24	759	737
miR1874-3p	LOC_Os11g28560.1	156	-20.49	156	-20.49	789290	24	1836	277
miR1874-3p	LOC_Os11g28780.1	156	-20.17	156	-20.17	789310	24	2994	180
miR1874-3p	LOC_Os11g28910.1	146	-25.29	146	-25.29	789323	24	2142	1473
miR1874-3p	LOC_Os11g29030.1	156	-23.84	156	-23.84	789332	24	915	598
miR1874-3p	LOC_Os11g29710.1	156	-21.23	156	-21.23	789406	24	1786	1173
miR1874-3p	LOC_Os11g30170.1	154	-23.58	154	-23.58	789453	24	4053	3824
miR1874-3p	LOC_Os11g30270.1	153	-21.01	153	-21.01	789463	24	2562	2222
miR1874-3p	LOC_Os11g30640.1	158	-25.41	158	-25.41	789496	24	477	455
miR1874-3p	LOC_Os11g30760.1	158	-20.07	158	-20.07	789506	24	1009	176
miR1874-3p	LOC_Os11g31340.1	329	-53.1	165	-28.65	789557	24	1273	719 273
miR1874-3p	LOC_Os11g31400.1	155	-20.95	155	-20.95	789562	24	765	29
miR1874-3p	LOC_Os11g31500.1	151	-21.6	151	-21.6	789575	24	5025	1094
miR1874-3p	LOC_Os11g32040.1	144	-21.53	144	-21.53	789637	24	354	53

miR1874-3p	LOC_Os11g32060.1	165	-25.97	165	-25.97	789639	24	4458	3429
miR1874-3p	LOC_Os11g32110.1	150	-20.1	150	-20.1	789644	24	3570	1462
miR1874-3p	LOC_Os11g32110.2	150	-20.1	150	-20.1	789645	24	3562	1457
miR1874-3p	LOC_Os11g32110.3	150	-20.1	150	-20.1	789646	24	3567	1462
miR1874-3p	LOC_Os11g32110.4	150	-20.1	150	-20.1	789647	24	3519	1462
miR1874-3p	LOC_Os11g32270.1	164	-24.33	164	-24.33	789663	24	2379	53
miR1874-3p	LOC_Os11g32270.2	164	-24.33	164	-24.33	789664	24	2325	49
miR1874-3p	LOC_Os11g32300.1	153	-23.17	153	-23.17	789669	24	366	250
miR1874-3p	LOC_Os11g32759.1	142	-20.35	142	-20.35	789715	24	3341	2844
miR1874-3p	LOC_Os11g32859.1	142	-20.35	142	-20.35	789716	24	699	311
miR1874-3p	LOC_Os11g32900.1	157	-20.94	157	-20.94	789731	24	3416	736
miR1874-3p	LOC_Os11g32900.2	157	-20.94	157	-20.94	789732	24	3508	734
miR1874-3p	LOC_Os11g33240.1	155	-23.13	155	-23.13	789771	24	2233	71
miR1874-3p	LOC_Os11g33240.2	155	-23.13	155	-23.13	789772	24	2219	71
miR1874-3p	LOC_Os11g33240.3	155	-23.13	155	-23.13	789773	24	2162	71
miR1874-3p	LOC_Os11g33960.1	168	-24.77	168	-24.77	789796	24	4236	3897
miR1874-3p	LOC_Os11g34160.1	155	-20.96	155	-20.96	789821	24	2587	785
miR1874-3p	LOC_Os11g34200.1	154	-20.61	154	-20.61	789825	24	1234	630
miR1874-3p	LOC_Os11g34270.1	156	-20.08	156	-20.08	789838	24	3992	30
miR1874-3p	LOC_Os11g34500.1	159	-25.66	159	-25.66	789867	24	5403	1847
miR1874-3p	LOC_Os11g34710.1	161	-20.99	161	-20.99	789885	24	1312	259
miR1874-3p	LOC_Os11g34760.1	145	-23.42	145	-23.42	789890	24	1488	1098
miR1874-3p	LOC_Os11g34840.1	145	-23.42	145	-23.42	789899	24	996	762
miR1874-3p	LOC_Os11g35030.1	174	-30.97	174	-30.97	789920	24	1708	704
miR1874-3p	LOC_Os11g35030.2	174	-30.97	174	-30.97	789921	24	2524	704
miR1874-3p	LOC_Os11g35390.1	169	-22.36	169	-22.36	789956	24	1490	676
miR1874-3p	LOC_Os11g35540.1	168	-20.17	168	-20.17	789975	24	3158	1122
miR1874-3p	LOC_Os11g35560.1	307	-43.8	160	-23.52	789977	24	5289	817 4274
miR1874-3p	LOC_Os11g35850.1	161	-21.22	161	-21.22	790006	24	3195	2882
miR1874-3p	LOC_Os11g36150.1	161	-20.52	161	-20.52	790044	24	3618	531
miR1874-3p	LOC_Os11g36340.1	158	-21.51	158	-21.51	790065	24	1968	128
miR1874-3p	LOC_Os11g36930.1	159	-21.78	159	-21.78	790124	24	1864	267

miR1874-3p	LOC_Os11g37270.1	152	-27.18	152	-27.18	790157	24	1727	489
miR1874-3p	LOC_Os11g37600.1	152	-21.7	152	-21.7	790190	24	2469	1218
miR1874-3p	LOC_Os11g37700.1	313	-41.85	158	-21.44	790199	24	4824	1332 3553
miR1874-3p	LOC_Os11g37830.1	156	-20.06	156	-20.06	790216	24	3093	180
miR1874-3p	LOC_Os11g37860.1	158	-26.15	158	-26.15	790219	24	3581	1768
miR1874-3p	LOC_Os11g38200.1	168	-21.46	168	-21.46	790256	24	1523	556
miR1874-3p	LOC_Os11g38520.1	150	-26.77	150	-26.77	790289	24	4343	2177
miR1874-3p	LOC_Os11g38660.1	156	-20.96	156	-20.96	790303	24	1938	1160
miR1874-3p	LOC_Os11g38750.1	162	-28.29	162	-28.29	790310	24	4044	1094
miR1874-3p	LOC_Os11g38810.1	149	-20.64	149	-20.64	790318	24	1738	1213
miR1874-3p	LOC_Os11g38810.2	149	-20.64	149	-20.64	790319	24	1733	1208
miR1874-3p	LOC_Os11g39020.1	157	-25.74	157	-25.74	790347	24	2595	83
miR1874-3p	LOC_Os11g39130.3	159	-21.29	159	-21.29	790359	24	1073	740
miR1874-3p	LOC_Os11g39170.1	155	-22.91	155	-22.91	790364	24	2901	1621
miR1874-3p	LOC_Os11g39310.1	152	-21.08	152	-21.08	790382	24	3655	2472
miR1874-3p	LOC_Os11g39880.1	151	-21.07	151	-21.07	790441	24	1041	1019
miR1874-3p	LOC_Os11g40390.1	164	-22.9	164	-22.9	790495	24	4227	3698
miR1874-3p	LOC_Os11g40410.1	167	-22.55	167	-22.55	790497	24	2619	805
miR1874-3p	LOC_Os11g40410.2	167	-22.55	167	-22.55	790498	24	2916	807
miR1874-3p	LOC_Os11g40450.1	156	-23.05	156	-23.05	790501	24	7341	1845
miR1874-3p	LOC_Os11g40460.1	173	-31.5	173	-31.5	790502	24	3435	1830
miR1874-3p	LOC_Os11g40540.1	154	-20.04	154	-20.04	790513	24	2514	1534
miR1874-3p	LOC_Os11g40540.2	154	-20.04	154	-20.04	790514	24	2660	1522
miR1874-3p	LOC_Os11g40640.1	311	-46.96	159	-24.17	790533	24	2421	60 701
miR1874-3p	LOC_Os11g40760.1	163	-30.03	163	-30.03	790543	24	1112	134
miR1874-3p	LOC_Os11g40920.1	157	-20.21	157	-20.21	790558	24	6474	4084
miR1874-3p	LOC_Os11g41490.1	155	-21	155	-21	790612	24	1732	570
miR1874-3p	LOC_Os11g41500.1	157	-25.48	157	-25.48	790613	24	471	204
miR1874-3p	LOC_Os11g41590.1	166	-27.77	166	-27.77	790625	24	1389	32
miR1874-3p	LOC_Os11g41600.1	165	-22.7	165	-22.7	790626	24	1112	84
miR1874-3p	LOC_Os11g41600.2	165	-22.7	165	-22.7	790627	24	1043	84
miR1874-3p	LOC_Os11g41750.1	154	-20.7	154	-20.7	790642	24	2436	281

miR1874-3p	LOC_Os11g41940.1	178	-25.78	178	-25.78	790662	24	586	450
miR1874-3p	LOC_Os11g42200.1	156	-21.17	156	-21.17	790689	24	2234	317
miR1874-3p	LOC_Os11g42210.1	150	-20.76	150	-20.76	790690	24	1344	948
miR1874-3p	LOC_Os11g42260.1	158	-21.02	158	-21.02	790695	24	960	367
miR1874-3p	LOC_Os11g42580.1	325	-47.18	168	-24.09	790731	24	4335	632 3095
miR1874-3p	LOC_Os11g42770.1	157	-23.09	157	-23.09	790750	24	4008	2771
miR1874-3p	LOC_Os11g42800.1	158	-22.06	158	-22.06	790753	24	2012	1201
miR1874-3p	LOC_Os11g42830.1	297	-44.67	150	-22.62	790756	24	1896	1547 431
miR1874-3p	LOC_Os11g43030.1	158	-27.39	158	-27.39	790778	24	2120	1880
miR1874-3p	LOC_Os11g43250.1	165	-21.79	165	-21.79	790798	24	3217	1697
miR1874-3p	LOC_Os11g43390.1	158	-22.97	158	-22.97	790812	24	2800	1407
miR1874-3p	LOC_Os11g43900.1	154	-22.02	154	-22.02	790873	24	979	201
miR1874-3p	LOC_Os11g43900.2	154	-22.02	154	-22.02	790874	24	1169	201
miR1874-3p	LOC_Os11g44240.1	157	-24.01	157	-24.01	790907	24	5718	4053
miR1874-3p	LOC_Os11g44310.1	159	-23.87	159	-23.87	790914	24	2312	1630
miR1874-3p	LOC_Os11g44340.1	162	-25.2	162	-25.2	790916	24	2418	1303
miR1874-3p	LOC_Os11g44530.1	157	-21.8	157	-21.8	790936	24	4224	2223
miR1874-3p	LOC_Os11g44780.1	156	-20.48	156	-20.48	790958	24	2811	1824
miR1874-3p	LOC_Os11g44800.1	157	-22.9	157	-22.9	790960	24	1530	1316
miR1874-3p	LOC_Os11g44880.1	156	-21.44	156	-21.44	790971	24	3220	2044
miR1874-3p	LOC_Os11g45530.1	158	-21.14	158	-21.14	791040	24	2585	1454
miR1874-3p	LOC_Os11g45540.1	150	-22.01	150	-22.01	791041	24	1878	1247
miR1874-3p	LOC_Os11g45740.1	140	-20.34	140	-20.34	791066	24	1402	782
miR1874-3p	LOC_Os11g45780.1	145	-22.87	145	-22.87	791070	24	735	363
miR1874-3p	LOC_Os11g45790.1	151	-20.96	151	-20.96	791071	24	3249	2345
miR1874-3p	LOC_Os11g45840.1	149	-21.15	149	-21.15	791075	24	2004	928
miR1874-3p	LOC_Os11g45990.1	151	-21.21	151	-21.21	791090	24	2457	781
miR1874-3p	LOC_Os11g46040.1	144	-21.37	144	-21.37	791097	24	3957	225
miR1874-3p	LOC_Os11g46290.1	154	-23.58	154	-23.58	791123	24	2598	2351
miR1874-3p	LOC_Os11g47070.1	169	-28.72	169	-28.72	791154	24	2169	1710
miR1874-3p	LOC_Os11g47120.1	159	-20.71	159	-20.71	791158	24	1676	233
miR1874-3p	LOC_Os11g47448.1	161	-24.5	161	-24.5	791205	24	852	17

miR1874-3p	LOC_Os11g47460.1	162	-24.53	162	-24.53	791210	24	1201	571
miR1874-3p	LOC_Os11g47460.2	162	-24.53	162	-24.53	791211	24	1143	571
miR1874-3p	LOC_Os11g47460.3	162	-24.53	162	-24.53	791212	24	1199	571
miR1874-3p	LOC_Os11g47460.4	162	-24.53	162	-24.53	791213	24	1144	571
miR1874-3p	LOC_Os11g47470.1	176	-33.4	176	-33.4	791214	24	3000	1569
miR1874-3p	LOC_Os11g47520.1	155	-25.23	155	-25.23	791220	24	1179	655
miR1874-3p	LOC_Os11g47560.1	155	-25.23	155	-25.23	791223	24	1095	619
miR1874-3p	LOC_Os11g47590.1	157	-22.06	157	-22.06	791227	24	1375	61
miR1874-3p	LOC_Os11g47680.1	324	-46.79	168	-25.07	791237	24	835	9 674
miR1874-3p	LOC_Os11g47730.1	158	-23.87	158	-23.87	791241	24	1575	191
miR1874-3p	LOC_Os11g47730.2	158	-23.87	158	-23.87	791242	24	1821	437
miR1874-3p	LOC_Os11g47760.1	157	-21.87	157	-21.87	791245	24	2389	57
miR1874-3p	LOC_Os11g47760.2	157	-21.87	157	-21.87	791246	24	2287	57
miR1874-3p	LOC_Os11g47760.3	157	-21.87	157	-21.87	791247	24	2352	57
miR1874-3p	LOC_Os11g47760.5	157	-21.87	157	-21.87	791249	24	2348	57
miR1874-3p	LOC_Os11g47760.6	157	-21.87	157	-21.87	791250	24	1627	57
miR1874-3p	LOC_Os11g47890.1	167	-29.09	167	-29.09	791264	24	1917	1894
miR1874-3p	LOC_Os11g47900.1	155	-21.53	155	-21.53	791265	24	2285	1688
miR1874-3p	LOC_Os12g01370.1	163	-24.56	163	-24.56	791336	24	1814	1179
miR1874-3p	LOC_Os12g01620.1	159	-24.91	159	-24.91	791369	24	1502	1457
miR1874-3p	LOC_Os12g01930.3	150	-22.76	150	-22.76	791402	24	2311	1910
miR1874-3p	LOC_Os12g02490.1	162	-23.84	162	-23.84	791474	24	3684	2640
miR1874-3p	LOC_Os12g02760.1	158	-22.68	158	-22.68	791515	24	993	637
miR1874-3p	LOC_Os12g02820.1	149	-22.84	149	-22.84	791524	24	2067	1642
miR1874-3p	LOC_Os12g03040.1	443	-62.41	157	-21.37	791548	24	1945	19 1446 906
miR1874-3p	LOC_Os12g03200.1	149	-23.3	149	-23.3	791569	24	2273	271
miR1874-3p	LOC_Os12g03200.2	149	-23.3	149	-23.3	791570	24	3519	272
miR1874-3p	LOC_Os12g03200.3	149	-23.3	149	-23.3	791571	24	2099	271
miR1874-3p	LOC_Os12g03350.1	155	-21.96	155	-21.96	791584	24	843	197
miR1874-3p	LOC_Os12g03630.1	154	-20.58	154	-20.58	791614	24	1641	85
miR1874-3p	LOC_Os12g03790.1	154	-20.39	154	-20.39	791631	24	2109	1270
miR1874-3p	LOC_Os12g03899.1	161	-20.34	161	-20.34	791650	24	2216	1222

miR1874-3p	LOC_Os12g03899.2	161	-20.34	161	-20.34	791651	24	2204	1222
miR1874-3p	LOC_Os12g03899.3	161	-20.34	161	-20.34	791652	24	2094	1112
miR1874-3p	LOC_Os12g03899.4	161	-20.34	161	-20.34	791653	24	2462	1066
miR1874-3p	LOC_Os12g03920.1	152	-27.49	152	-27.49	791654	24	996	357
miR1874-3p	LOC_Os12g04510.1	167	-23.38	167	-23.38	791724	24	1321	223
miR1874-3p	LOC_Os12g04590.1	159	-21.02	159	-21.02	791731	24	1494	821
miR1874-3p	LOC_Os12g04590.2	159	-21.02	159	-21.02	791732	24	1591	918
miR1874-3p	LOC_Os12g04650.1	161	-20.84	161	-20.84	791738	24	1811	210
miR1874-3p	LOC_Os12g04650.2	161	-20.84	161	-20.84	791739	24	1930	210
miR1874-3p	LOC_Os12g04650.3	161	-20.84	161	-20.84	791740	24	1840	255
miR1874-3p	LOC_Os12g04650.4	161	-20.84	161	-20.84	791741	24	1871	255
miR1874-3p	LOC_Os12g04660.1	159	-21.02	159	-21.02	791742	24	867	400
miR1874-3p	LOC_Os12g04790.1	164	-22.31	164	-22.31	791756	24	1174	136
miR1874-3p	LOC_Os12g04880.1	164	-21.55	164	-21.55	791766	24	3133	2341
miR1874-3p	LOC_Os12g05200.1	162	-20.89	162	-20.89	791809	24	1048	741
miR1874-3p	LOC_Os12g05330.1	162	-20.68	162	-20.68	791822	24	1524	255
miR1874-3p	LOC_Os12g05340.1	164	-27.62	164	-27.62	791824	24	360	119
miR1874-3p	LOC_Os12g05370.1	145	-21.53	145	-21.53	791827	24	963	168
miR1874-3p	LOC_Os12g06040.1	150	-21.12	150	-21.12	791897	24	4671	4166
miR1874-3p	LOC_Os12g06200.1	157	-23.31	157	-23.31	791913	24	2100	655
miR1874-3p	LOC_Os12g06200.2	157	-23.31	157	-23.31	791914	24	2207	655
miR1874-3p	LOC_Os12g06790.1	146	-21.07	146	-21.07	791982	24	432	409
miR1874-3p	LOC_Os12g07050.1	166	-24.23	166	-24.23	792013	24	1215	649
miR1874-3p	LOC_Os12g07050.2	166	-24.23	166	-24.23	792014	24	738	172
miR1874-3p	LOC_Os12g07050.3	166	-24.23	166	-24.23	792015	24	1552	649
miR1874-3p	LOC_Os12g07740.1	155	-20.86	155	-20.86	792088	24	1908	1303
miR1874-3p	LOC_Os12g08040.1	147	-25.85	147	-25.85	792126	24	465	339
miR1874-3p	LOC_Os12g08140.1	163	-23.06	163	-23.06	792136	24	4510	4411
miR1874-3p	LOC_Os12g08510.1	151	-22.37	151	-22.37	792178	24	5637	1889
miR1874-3p	LOC_Os12g09584.1	157	-23.94	157	-23.94	792291	24	1559	245
miR1874-3p	LOC_Os12g09990.1	152	-21.18	152	-21.18	792328	24	3864	337
miR1874-3p	LOC_Os12g10070.1	164	-26.25	164	-26.25	792336	24	2028	619

miR1874-3p	LOC_Os12g10500.1	151	-20.89	151	-20.89	792381	24	4074	1400
miR1874-3p	LOC_Os12g10650.1	153	-21.28	153	-21.28	792403	24	989	90
miR1874-3p	LOC_Os12g10700.1	149	-24.25	149	-24.25	792408	24	3932	1071
miR1874-3p	LOC_Os12g10740.1	152	-24.09	152	-24.09	792416	24	3436	387
miR1874-3p	LOC_Os12g11170.1	158	-21.02	158	-21.02	792458	24	1485	370
miR1874-3p	LOC_Os12g11260.1	169	-28.72	169	-28.72	792467	24	2694	2196
miR1874-3p	LOC_Os12g11820.1	158	-21.02	158	-21.02	792519	24	881	364
miR1874-3p	LOC_Os12g12664.1	148	-22.7	148	-22.7	792604	24	1797	1153
miR1874-3p	LOC_Os12g12664.2	148	-22.7	148	-22.7	792605	24	3510	2866
miR1874-3p	LOC_Os12g12860.1	143	-20.09	143	-20.09	792627	24	2427	1497
miR1874-3p	LOC_Os12g12970.1	163	-22.49	163	-22.49	792636	24	1359	319
miR1874-3p	LOC_Os12g13000.1	304	-45.5	152	-23.22	792639	24	789	276 705
miR1874-3p	LOC_Os12g13174.1	155	-22.49	155	-22.49	792663	24	3959	929
miR1874-3p	LOC_Os12g13440.1	163	-27.28	163	-27.28	792693	24	8268	5490
miR1874-3p	LOC_Os12g13440.2	163	-27.28	163	-27.28	792694	24	8226	5478
miR1874-3p	LOC_Os12g13570.1	155	-20.59	155	-20.59	792707	24	3382	2205
miR1874-3p	LOC_Os12g14290.1	167	-25.31	167	-25.31	792785	24	4311	1277
miR1874-3p	LOC_Os12g14830.1	150	-21.14	150	-21.14	792829	24	570	189
miR1874-3p	LOC_Os12g15290.1	141	-22.07	141	-22.07	792871	24	2763	1249
miR1874-3p	LOC_Os12g16000.1	161	-23.26	161	-23.26	792940	24	3357	1074
miR1874-3p	LOC_Os12g16080.1	156	-22.28	156	-22.28	792948	24	3369	3295
miR1874-3p	LOC_Os12g16150.1	164	-26.24	164	-26.24	792954	24	4233	1997
miR1874-3p	LOC_Os12g16160.1	160	-21.45	160	-21.45	792955	24	663	474
miR1874-3p	LOC_Os12g16460.1	141	-22.25	141	-22.25	792998	24	1554	1300
miR1874-3p	LOC_Os12g16480.1	145	-20.2	145	-20.2	793000	24	720	521
miR1874-3p	LOC_Os12g16810.1	147	-23.72	147	-23.72	793035	24	885	368
miR1874-3p	LOC_Os12g16980.1	147	-22.88	147	-22.88	793051	24	1467	1202
miR1874-3p	LOC_Os12g17360.1	161	-24.66	161	-24.66	793091	24	1689	1222
miR1874-3p	LOC_Os12g17450.1	151	-22.84	151	-22.84	793100	24	3162	15
miR1874-3p	LOC_Os12g17490.1	155	-20.32	155	-20.32	793104	24	2978	2769
miR1874-3p	LOC_Os12g17620.1	157	-28.9	157	-28.9	793118	24	3372	2558
miR1874-3p	LOC_Os12g17690.1	159	-20.29	159	-20.29	793125	24	3201	1592

miR1874-3p	LOC_Os12g17770.1	160	-25.43	160	-25.43	793131	24	408	30
miR1874-3p	LOC_Os12g18050.1	154	-22.44	154	-22.44	793161	24	1326	1304
miR1874-3p	LOC_Os12g18460.1	154	-22.42	154	-22.42	793209	24	5544	5516
miR1874-3p	LOC_Os12g18750.1	156	-22.84	156	-22.84	793243	24	1011	650
miR1874-3p	LOC_Os12g19260.1	165	-21.66	165	-21.66	793288	24	2271	2
miR1874-3p	LOC_Os12g19470.1	155	-20.03	155	-20.03	793307	24	2122	1625
miR1874-3p	LOC_Os12g19470.2	155	-20.03	155	-20.03	793308	24	2178	1660
miR1874-3p	LOC_Os12g19760.1	176	-33.4	176	-33.4	793337	24	4137	2202
miR1874-3p	LOC_Os12g19910.1	154	-22.07	154	-22.07	793352	24	993	971
miR1874-3p	LOC_Os12g20040.1	144	-20.48	144	-20.48	793365	24	3060	231
miR1874-3p	LOC_Os12g20150.1	140	-20.63	140	-20.63	793377	24	4232	381
miR1874-3p	LOC_Os12g20420.1	159	-22.2	159	-22.2	793410	24	1458	120
miR1874-3p	LOC_Os12g22080.1	156	-22.78	156	-22.78	793480	24	1539	623
miR1874-3p	LOC_Os12g22090.1	494	-80.56	165	-29.4	793481	24	3192	1 552 54
miR1874-3p	LOC_Os12g22090.2	494	-80.56	165	-29.4	793482	24	2045	1 552 54
miR1874-3p	LOC_Os12g22090.4	494	-80.56	165	-29.4	793483	24	2035	1 552 54
miR1874-3p	LOC_Os12g22410.1	152	-20.26	152	-20.26	793513	24	1266	971
miR1874-3p	LOC_Os12g23220.1	156	-20.49	156	-20.49	793592	24	2502	280
miR1874-3p	LOC_Os12g23660.1	159	-22.53	159	-22.53	793635	24	3249	1976
miR1874-3p	LOC_Os12g23910.1	156	-20.49	156	-20.49	793657	24	2502	280
miR1874-3p	LOC_Os12g24050.1	146	-20.61	146	-20.61	793672	24	4863	2514
miR1874-3p	LOC_Os12g24120.1	160	-20.8	160	-20.8	793679	24	2763	624
miR1874-3p	LOC_Os12g24830.1	156	-21.34	156	-21.34	793742	24	1809	1202
miR1874-3p	LOC_Os12g25040.1	154	-21.23	154	-21.23	793762	24	2762	2026
miR1874-3p	LOC_Os12g25190.1	176	-33.4	176	-33.4	793777	24	3045	1359
miR1874-3p	LOC_Os12g25340.1	169	-28.72	169	-28.72	793791	24	2466	2031
miR1874-3p	LOC_Os12g25420.1	162	-21.06	162	-21.06	793799	24	294	272
miR1874-3p	LOC_Os12g25580.1	163	-25.03	163	-25.03	793816	24	2529	1052
miR1874-3p	LOC_Os12g25830.1	154	-21.23	154	-21.23	793844	24	5148	2690
miR1874-3p	LOC_Os12g26020.1	316	-46.44	162	-25.78	793860	24	2724	1489 788
miR1874-3p	LOC_Os12g26250.1	156	-20.06	156	-20.06	793883	24	3123	180
miR1874-3p	LOC_Os12g26480.1	153	-21.59	153	-21.59	793909	24	1312	732

miR1874-3p	LOC_Os12g26480.2	153	-21.59	153	-21.59	793910	24	1413	833
miR1874-3p	LOC_Os12g26590.1	150	-24.24	150	-24.24	793919	24	684	225
miR1874-3p	LOC_Os12g26830.1	158	-21.02	158	-21.02	793942	24	1855	1462
miR1874-3p	LOC_Os12g26860.1	158	-21.02	158	-21.02	793945	24	6069	3223
miR1874-3p	LOC_Os12g26870.1	158	-21.02	158	-21.02	793946	24	1974	364
miR1874-3p	LOC_Os12g27060.1	159	-27.31	159	-27.31	793967	24	3354	75
miR1874-3p	LOC_Os12g27096.1	159	-21.53	159	-21.53	793971	24	5238	3917
miR1874-3p	LOC_Os12g27240.1	311	-48.97	161	-27.79	793988	24	3243	2769 3084
miR1874-3p	LOC_Os12g27290.1	160	-20.07	160	-20.07	793992	24	369	311
miR1874-3p	LOC_Os12g27310.1	148	-21.93	148	-21.93	793994	24	1710	592
miR1874-3p	LOC_Os12g27370.1	163	-28.66	163	-28.66	794000	24	2927	784
miR1874-3p	LOC_Os12g27390.1	159	-22.62	159	-22.62	794001	24	3990	159
miR1874-3p	LOC_Os12g27600.1	144	-22.57	144	-22.57	794024	24	1725	1119
miR1874-3p	LOC_Os12g27700.1	154	-20.7	154	-20.7	794032	24	2517	281
miR1874-3p	LOC_Os12g27710.1	149	-20.75	149	-20.75	794033	24	5871	284
miR1874-3p	LOC_Os12g27890.1	153	-20.94	153	-20.94	794051	24	857	338
miR1874-3p	LOC_Os12g28400.1	156	-20.06	156	-20.06	794098	24	3207	180
miR1874-3p	LOC_Os12g28490.1	154	-22.07	154	-22.07	794107	24	5334	5312
miR1874-3p	LOC_Os12g28980.1	312	-43.09	158	-22.07	794153	24	1050	373 1028
miR1874-3p	LOC_Os12g28990.1	154	-22.07	154	-22.07	794154	24	5280	5258
miR1874-3p	LOC_Os12g29010.1	312	-43.09	158	-22.07	794156	24	1050	373 1028
miR1874-3p	LOC_Os12g29240.1	158	-21.78	158	-21.78	794178	24	615	72
miR1874-3p	LOC_Os12g29250.1	169	-28.72	169	-28.72	794179	24	2631	2196
miR1874-3p	LOC_Os12g29560.1	147	-23.4	147	-23.4	794211	24	1723	983
miR1874-3p	LOC_Os12g30160.1	156	-21.51	156	-21.51	794277	24	465	100
miR1874-3p	LOC_Os12g30410.1	158	-21.02	158	-21.02	794300	24	984	364
miR1874-3p	LOC_Os12g30700.1	159	-24.28	159	-24.28	794332	24	3939	557
miR1874-3p	LOC_Os12g30750.1	160	-20.87	160	-20.87	794336	24	357	216
miR1874-3p	LOC_Os12g30880.1	164	-22.3	164	-22.3	794347	24	1011	609
miR1874-3p	LOC_Os12g30900.1	160	-20.49	160	-20.49	794349	24	1740	1615
miR1874-3p	LOC_Os12g31180.1	154	-20.7	154	-20.7	794373	24	2481	281
miR1874-3p	LOC_Os12g31940.1	154	-23.58	154	-23.58	794455	24	591	212

miR1874-3p	LOC_Os12g32000.1	162	-22.93	162	-22.93	794459	24	1872	778
miR1874-3p	LOC_Os12g32040.1	151	-21.29	151	-21.29	794463	24	809	690
miR1874-3p	LOC_Os12g32120.1	169	-29.39	169	-29.39	794469	24	3045	898
miR1874-3p	LOC_Os12g32230.1	161	-20.97	161	-20.97	794481	24	1790	4
miR1874-3p	LOC_Os12g32230.2	161	-20.97	161	-20.97	794482	24	1795	4
miR1874-3p	LOC_Os12g32680.1	157	-22.74	157	-22.74	794529	24	3627	3526
miR1874-3p	LOC_Os12g32710.1	152	-22.99	152	-22.99	794532	24	3652	1779
miR1874-3p	LOC_Os12g32730.1	157	-22.79	157	-22.79	794534	24	2610	110
miR1874-3p	LOC_Os12g33240.1	148	-21.04	148	-21.04	794584	24	2296	68
miR1874-3p	LOC_Os12g33240.2	148	-21.04	148	-21.04	794585	24	1017	72
miR1874-3p	LOC_Os12g33240.3	148	-21.04	148	-21.04	794586	24	1032	72
miR1874-3p	LOC_Os12g33280.1	164	-20.87	164	-20.87	794590	24	1728	1612
miR1874-3p	LOC_Os12g33450.1	158	-21.02	158	-21.02	794604	24	5037	4531
miR1874-3p	LOC_Os12g33570.1	159	-20.12	159	-20.12	794616	24	369	164
miR1874-3p	LOC_Os12g33630.1	153	-20.15	153	-20.15	794622	24	705	589
miR1874-3p	LOC_Os12g33650.1	148	-21.07	148	-21.07	794624	24	1629	43
miR1874-3p	LOC_Os12g33710.1	160	-24.6	160	-24.6	794630	24	696	621
miR1874-3p	LOC_Os12g33920.1	158	-20.31	158	-20.31	794647	24	1047	364
miR1874-3p	LOC_Os12g34090.1	154	-22.07	154	-22.07	794671	24	5373	5351
miR1874-3p	LOC_Os12g34180.1	154	-22.07	154	-22.07	794690	24	5418	5396
miR1874-3p	LOC_Os12g34330.1	160	-20.15	160	-20.15	794706	24	2782	830
miR1874-3p	LOC_Os12g34650.1	141	-21.63	141	-21.63	794739	24	4896	4307
miR1874-3p	LOC_Os12g34740.1	160	-21.69	160	-21.69	794747	24	4101	1547
miR1874-3p	LOC_Os12g35130.1	154	-22.07	154	-22.07	794798	24	4413	4391
miR1874-3p	LOC_Os12g35170.1	158	-21.02	158	-21.02	794802	24	879	373
miR1874-3p	LOC_Os12g35490.1	172	-24.96	172	-24.96	794836	24	1906	209
miR1874-3p	LOC_Os12g35630.1	161	-24.47	161	-24.47	794855	24	4512	3220
miR1874-3p	LOC_Os12g35840.1	161	-27.79	161	-27.79	794875	24	2970	2805
miR1874-3p	LOC_Os12g35940.1	158	-20.72	158	-20.72	794886	24	4274	3408
miR1874-3p	LOC_Os12g36020.1	157	-23.3	157	-23.3	794894	24	1278	1030
miR1874-3p	LOC_Os12g36170.1	152	-20.25	152	-20.25	794910	24	4119	715
miR1874-3p	LOC_Os12g36260.1	151	-20.89	151	-20.89	794918	24	4359	1388

miR1874-3p	LOC_Os12g36420.1	149	-21.1	149	-21.1	794932	24	2367	741
miR1874-3p	LOC_Os12g36500.1	167	-26.13	167	-26.13	794940	24	609	255
miR1874-3p	LOC_Os12g36550.1	159	-23.47	159	-23.47	794945	24	3969	1616
miR1874-3p	LOC_Os12g36680.1	149	-23.19	149	-23.19	794958	24	2090	1173
miR1874-3p	LOC_Os12g36735.1	151	-22.26	151	-22.26	794964	24	567	132
miR1874-3p	LOC_Os12g37330.1	154	-20.7	154	-20.7	795030	24	2439	281
miR1874-3p	LOC_Os12g37430.1	165	-21.93	165	-21.93	795042	24	2285	896
miR1874-3p	LOC_Os12g37430.2	165	-21.93	165	-21.93	795043	24	2431	896
miR1874-3p	LOC_Os12g37430.3	165	-21.93	165	-21.93	795044	24	2572	1037
miR1874-3p	LOC_Os12g37519.1	162	-24.25	162	-24.25	795053	24	826	180
miR1874-3p	LOC_Os12g37519.2	162	-24.25	162	-24.25	795054	24	880	180
miR1874-3p	LOC_Os12g37560.1	150	-20.59	150	-20.59	795060	24	2253	107
miR1874-3p	LOC_Os12g37560.2	150	-20.59	150	-20.59	795061	24	2339	90
miR1874-3p	LOC_Os12g37690.1	140	-24.83	140	-24.83	795080	24	1304	595
miR1874-3p	LOC_Os12g37720.1	169	-27.36	169	-27.36	795083	24	4346	1158
miR1874-3p	LOC_Os12g37810.1	149	-22.12	149	-22.12	795092	24	4821	575
miR1874-3p	LOC_Os12g37830.1	165	-22.86	165	-22.86	795094	24	2640	1860
miR1874-3p	LOC_Os12g37840.1	157	-24.59	157	-24.59	795095	24	2828	112
miR1874-3p	LOC_Os12g37840.2	157	-24.59	157	-24.59	795096	24	2971	112
miR1874-3p	LOC_Os12g37970.1	304	-44.67	157	-23.38	795109	24	1313	555 585
miR1874-3p	LOC_Os12g37970.2	304	-44.67	157	-23.38	795110	24	1324	555 585
miR1874-3p	LOC_Os12g37970.3	304	-44.67	157	-23.38	795111	24	1436	555 585
miR1874-3p	LOC_Os12g38180.1	166	-26.76	166	-26.76	795129	24	3650	532
miR1874-3p	LOC_Os12g38210.1	151	-20.29	151	-20.29	795132	24	2519	863
miR1874-3p	LOC_Os12g38590.1	166	-25.45	166	-25.45	795174	24	4143	3262
miR1874-3p	LOC_Os12g38860.1	150	-22.59	150	-22.59	795213	24	2792	239
miR1874-3p	LOC_Os12g38940.1	141	-24.25	141	-24.25	795223	24	2057	707
miR1874-3p	LOC_Os12g39330.1	173	-28.07	173	-28.07	795263	24	1441	56
miR1874-3p	LOC_Os12g39380.1	165	-24.79	165	-24.79	795270	24	3073	1273
miR1874-3p	LOC_Os12g39880.1	157	-24.41	157	-24.41	795335	24	2840	564
miR1874-3p	LOC_Os12g39980.1	150	-20.79	150	-20.79	795349	24	8636	6708
miR1874-3p	LOC_Os12g40490.1	142	-20.84	142	-20.84	795403	24	2502	1200

miR1874-3p	LOC_Os12g40530.1	298	-46.01	153	-24.22	795409	24	3433	341 1131
miR1874-3p	LOC_Os12g40660.1	156	-23.08	156	-23.08	795424	24	2319	1755
miR1874-3p	LOC_Os12g41200.1	166	-20.74	166	-20.74	795484	24	5496	283
miR1874-3p	LOC_Os12g41570.1	149	-24.1	149	-24.1	795526	24	873	74
miR1874-3p	LOC_Os12g41800.1	157	-26.12	157	-26.12	795558	24	1837	86
miR1874-3p	LOC_Os12g41800.2	157	-26.12	157	-26.12	795559	24	1816	143
miR1874-3p	LOC_Os12g41820.1	153	-23.58	153	-23.58	795561	24	3126	1443
miR1874-3p	LOC_Os12g41860.1	160	-22.08	160	-22.08	795565	24	3154	1015
miR1874-3p	LOC_Os12g41870.1	154	-23.58	154	-23.58	795566	24	4710	4331
miR1874-3p	LOC_Os12g41900.1	156	-23.06	156	-23.06	795570	24	4329	1932
miR1874-3p	LOC_Os12g42044.1	155	-23.08	155	-23.08	795594	24	4172	1200
miR1874-3p	LOC_Os12g42044.2	155	-23.08	155	-23.08	795595	24	2305	1197
miR1874-3p	LOC_Os12g42044.3	155	-23.08	155	-23.08	795596	24	2279	1161
miR1874-3p	LOC_Os12g42044.4	155	-23.08	155	-23.08	795597	24	4172	1200
miR1874-3p	LOC_Os12g42044.5	155	-23.08	155	-23.08	795598	24	3655	1200
miR1874-3p	LOC_Os12g42064.1	306	-41.87	165	-21.15	795602	24	2969	619 2865
miR1874-3p	LOC_Os12g42200.1	153	-23.99	153	-23.99	795617	24	3020	1080
miR1874-3p	LOC_Os12g42280.1	154	-25.23	154	-25.23	795626	24	2565	1932
miR1874-3p	LOC_Os12g42310.1	156	-21.71	156	-21.71	795630	24	4179	873
miR1874-3p	LOC_Os12g42330.1	146	-21.09	146	-21.09	795631	24	1569	1253
miR1874-3p	LOC_Os12g42520.1	150	-21.23	150	-21.23	795653	24	3165	40
miR1874-3p	LOC_Os12g42870.1	151	-22.42	151	-22.42	795695	24	1805	494
miR1874-3p	LOC_Os12g42930.1	163	-21.06	163	-21.06	795705	24	2322	1010
miR1874-3p	LOC_Os12g42960.1	147	-21.57	147	-21.57	795708	24	2111	65
miR1874-3p	LOC_Os12g43050.1	144	-22.3	144	-22.3	795717	24	4788	4284
miR1874-3p	LOC_Os12g43363.1	147	-20.77	147	-20.77	795745	24	1428	109
miR1874-3p	LOC_Os12g43363.2	147	-20.77	147	-20.77	795746	24	1447	112
miR1874-3p	LOC_Os12g43363.3	147	-20.77	147	-20.77	795747	24	1437	109
miR1874-3p	LOC_Os12g43520.1	157	-22.74	157	-22.74	795762	24	1454	315
miR1874-3p	LOC_Os12g43720.1	156	-20.36	156	-20.36	795785	24	3149	640
miR1874-3p	LOC_Os12g43790.1	167	-21.24	167	-21.24	795792	24	819	195
miR1874-3p	LOC_Os12g44080.1	151	-22.09	151	-22.09	795828	24	732	169

miR1874-3p	LOC_Os12g44100.1	165	-28.06	165	-28.06	795830	24	2301	2034
miR1874-3p	LOC_Os12g44330.1	147	-21.53	147	-21.53	795859	24	3663	3428
miR1874-3p	LOC_Os12g44330.2	147	-21.53	147	-21.53	795860	24	3147	2826
miR1874-3p	ChrUn.fgenesh.mRNA.9	159	-26.39	159	-26.39	795880	24	432	183
miR1874-3p	ChrUn.fgenesh.mRNA.12	162	-25.78	162	-25.78	795883	24	5379	5357
miR1874-3p	ChrUn.fgenesh.mRNA.27	157	-23.82	157	-23.82	795898	24	3765	288
miR1874-3p	ChrUn.fgenesh.mRNA.30	154	-22.07	154	-22.07	795901	24	1323	1301
miR1874-3p	ChrUn.fgenesh.mRNA.36	150	-22.15	150	-22.15	795907	24	2337	1268
miR1874-3p	ChrUn.fgenesh.mRNA.51	158	-20.34	158	-20.34	795922	24	5313	4798
miR1874-3p	ChrUn.fgenesh.mRNA.52	157	-23.34	157	-23.34	795923	24	3678	2297
miR1874-3p	ChrUn.fgenesh.mRNA.80	152	-22.63	152	-22.63	795951	24	5409	1707
miR1874-3p	ChrSy.fgenesh.mRNA.9	147	-20.02	147	-20.02	795976	24	483	250
miR1874-3p	ChrSy.fgenesh.mRNA.52	159	-21.53	159	-21.53	796019	24	2985	335
miR1874-3p	ChrSy.fgenesh.mRNA.60	165	-28.73	165	-28.73	796027	24	2190	1271
miR1874-3p	ChrSy.fgenesh.mRNA.82	157	-26.55	157	-26.55	796049	24	1143	402
miR1870-3p	LOC_Os01g03370.1	164	-20.18	164	-20.18	796347	24	2895	674
miR1870-3p	LOC_Os01g05660.1	162	-21.36	162	-21.36	796603	24	2993	243
miR1870-3p	LOC_Os01g05660.2	162	-21.36	162	-21.36	796604	24	2863	237
miR1870-3p	LOC_Os01g05660.3	162	-21.36	162	-21.36	796605	24	2913	237
miR1870-3p	LOC_Os01g12480.1	156	-20.74	156	-20.74	797365	24	920	321
miR1870-3p	LOC_Os01g21350.1	154	-20.08	154	-20.08	798265	24	1347	548
miR1870-3p	LOC_Os01g21530.1	147	-23.66	147	-23.66	798283	24	2988	1136
miR1870-3p	LOC_Os01g22900.1	157	-20.66	157	-20.66	798415	24	2540	2396
miR1870-3p	LOC_Os01g22900.3	157	-20.66	157	-20.66	798417	24	2479	2335
miR1870-3p	LOC_Os01g33090.1	184	-25.08	184	-25.08	799317	24	2623	1608
miR1870-3p	LOC_Os01g36020.1	176	-22.7	176	-22.7	799614	24	2058	926
miR1870-3p	LOC_Os01g41770.1	166	-21.41	166	-21.41	800244	24	3227	1806
miR1870-3p	LOC_Os01g42210.1	168	-21.46	168	-21.46	800290	24	666	608
miR1870-3p	LOC_Os01g43380.1	160	-20.83	160	-20.83	800433	24	2458	251
miR1870-3p	LOC_Os01g44150.1	167	-22.62	167	-22.62	800523	24	4446	306
miR1870-3p	LOC_Os01g49110.1	154	-21.45	154	-21.45	801043	24	2530	2121
miR1870-3p	LOC_Os01g49110.2	154	-21.45	154	-21.45	801044	24	2434	2025

miR1870-3p	LOC_Os01g49120.1	154	-21.45	154	-21.45	801045	24	2367	310
miR1870-3p	LOC_Os01g50960.1	166	-20.06	166	-20.06	801248	24	1144	402
miR1870-3p	LOC_Os01g50970.1	156	-20.5	156	-20.5	801249	24	2101	1644
miR1870-3p	LOC_Os01g51370.1	169	-23.24	169	-23.24	801297	24	892	107
miR1870-3p	LOC_Os01g51370.2	169	-23.24	169	-23.24	801298	24	1682	814
miR1870-3p	LOC_Os01g52550.1	160	-21.45	160	-21.45	801441	24	4255	2142
miR1870-3p	LOC_Os01g66130.1	161	-21.41	161	-21.41	803104	24	2774	288
miR1870-3p	LOC_Os01g66520.1	163	-22.31	163	-22.31	803144	24	2547	1886
miR1870-3p	LOC_Os01g68230.1	150	-20.1	150	-20.1	803352	24	2733	2231
miR1870-3p	LOC_Os01g70720.1	170	-24.33	170	-24.33	803610	24	3013	978
miR1870-3p	LOC_Os01g70720.2	170	-24.33	170	-24.33	803611	24	2901	978
miR1870-3p	LOC_Os01g70820.1	147	-20.26	147	-20.26	803622	24	852	533
miR1870-3p	LOC_Os02g03320.1	154	-20.22	154	-20.22	804375	24	2146	447
miR1870-3p	LOC_Os02g06700.1	148	-22.12	148	-22.12	804812	24	948	482
miR1870-3p	LOC_Os02g06700.2	148	-22.12	148	-22.12	804813	24	802	336
miR1870-3p	LOC_Os02g07450.1	147	-20.73	147	-20.73	804915	24	1481	393
miR1870-3p	LOC_Os02g07450.2	147	-20.73	147	-20.73	804916	24	1454	393
miR1870-3p	LOC_Os02g07670.3	142	-20.54	142	-20.54	804943	24	2748	2107
miR1870-3p	LOC_Os02g18180.1	157	-21.89	157	-21.89	806087	24	2734	2589
miR1870-3p	LOC_Os02g24170.1	167	-20.01	167	-20.01	806641	24	1252	68
miR1870-3p	LOC_Os02g33360.1	167	-22.62	167	-22.62	807605	24	5040	399
miR1870-3p	LOC_Os02g37930.1	162	-20.1	162	-20.1	808126	24	819	285
miR1870-3p	LOC_Os02g42134.2	164	-22.89	164	-22.89	808597	24	3038	2271
miR1870-3p	LOC_Os02g42180.1	160	-21.16	160	-21.16	808602	24	4578	3809
miR1870-3p	LOC_Os02g43890.1	166	-20.11	166	-20.11	808818	24	339	306
miR1870-3p	LOC_Os02g44740.2	149	-20.17	149	-20.17	808944	24	3177	658
miR1870-3p	LOC_Os02g45054.3	170	-23.98	170	-23.98	808982	24	1349	374
miR1870-3p	LOC_Os02g46130.1	160	-21.84	160	-21.84	809108	24	2519	1822
miR1870-3p	LOC_Os02g51030.1	165	-20.57	165	-20.57	809727	24	1067	472
miR1870-3p	LOC_Os02g53100.1	152	-20.08	152	-20.08	809989	24	1815	134
miR1870-3p	LOC_Os02g55970.1	156	-22.95	156	-22.95	810348	24	2929	230
miR1870-3p	LOC_Os02g55970.2	156	-22.95	156	-22.95	810349	24	2942	243

miR1870-3p	LOC_Os02g56900.1	156	-22.05	156	-22.05	810460	24	1422	57
miR1870-3p	LOC_Os02g57080.1	153	-22.86	153	-22.86	810485	24	3209	2573
miR1870-3p	LOC_Os02g57080.2	153	-22.86	153	-22.86	810486	24	2741	2573
miR1870-3p	LOC_Os03g01930.1	147	-21.99	147	-21.99	810865	24	2282	932
miR1870-3p	LOC_Os03g07360.1	145	-21.75	145	-21.75	811561	24	1992	1538
miR1870-3p	LOC_Os03g11890.1	162	-22.02	162	-22.02	812070	24	5003	4679
miR1870-3p	LOC_Os03g13900.1	154	-20.53	154	-20.53	812338	24	5745	3188
miR1870-3p	LOC_Os03g14590.1	155	-23.14	155	-23.14	812429	24	1729	469
miR1870-3p	LOC_Os03g14590.2	155	-23.14	155	-23.14	812430	24	2210	469
miR1870-3p	LOC_Os03g14590.3	155	-23.14	155	-23.14	812431	24	3461	469
miR1870-3p	LOC_Os03g15900.2	162	-20.08	162	-20.08	812588	24	3764	1960
miR1870-3p	LOC_Os03g18420.1	159	-22.87	159	-22.87	812919	24	923	715
miR1870-3p	LOC_Os03g21740.1	170	-21.72	170	-21.72	813318	24	3624	2815
miR1870-3p	LOC_Os03g26229.1	164	-22.35	164	-22.35	813761	24	992	226
miR1870-3p	LOC_Os03g26329.1	150	-22.62	150	-22.62	813769	24	3933	41
miR1870-3p	LOC_Os03g27140.1	152	-20.17	152	-20.17	813854	24	5526	3181
miR1870-3p	LOC_Os03g28110.1	164	-21	164	-21	813962	24	3912	2618
miR1870-3p	LOC_Os03g31730.1	159	-21.22	159	-21.22	814294	24	3243	2035
miR1870-3p	LOC_Os03g31986.1	159	-21.34	159	-21.34	814315	24	3954	459
miR1870-3p	LOC_Os03g33770.1	154	-20.21	154	-20.21	814483	24	2397	1767
miR1870-3p	LOC_Os03g36239.1	153	-21.31	153	-21.31	814619	24	8958	2787
miR1870-3p	LOC_Os03g39660.1	158	-21.31	158	-21.31	814954	24	873	342
miR1870-3p	LOC_Os03g39910.1	169	-21.5	169	-21.5	814976	24	2886	1664
miR1870-3p	LOC_Os03g41870.1	149	-20.06	149	-20.06	815150	24	474	362
miR1870-3p	LOC_Os03g55870.1	176	-20.68	176	-20.68	816690	24	1463	1245
miR1870-3p	LOC_Os03g55870.2	176	-20.68	176	-20.68	816691	24	1540	1353
miR1870-3p	LOC_Os03g60620.1	149	-20.49	149	-20.49	817262	24	2564	2042
miR1870-3p	LOC_Os04g01439.1	154	-21	154	-21	817809	24	5712	4968
miR1870-3p	LOC_Os04g11280.1	170	-20.87	170	-20.87	818746	24	1503	509
miR1870-3p	LOC_Os04g18610.1	154	-22.47	154	-22.47	819440	24	2058	249
miR1870-3p	LOC_Os04g20940.1	154	-20.53	154	-20.53	819669	24	5781	2738
miR1870-3p	LOC_Os04g31680.1	155	-20.66	155	-20.66	820715	24	384	33

miR1870-3p	LOC_Os04g33010.1	177	-23.87	177	-23.87	820862	24	2820	2061
miR1870-3p	LOC_Os04g33490.1	150	-21.18	150	-21.18	820921	24	2165	1187
miR1870-3p	LOC_Os04g43840.1	158	-22.01	158	-22.01	822026	24	1109	1014
miR1870-3p	LOC_Os04g44070.1	162	-20.19	162	-20.19	822050	24	357	260
miR1870-3p	LOC_Os04g57480.1	151	-21.29	151	-21.29	823653	24	1875	1547
miR1870-3p	LOC_Os04g59100.1	152	-20.54	152	-20.54	823865	24	5130	2842
miR1870-3p	LOC_Os04g59460.1	159	-23.96	159	-23.96	823905	24	5636	3289
miR1870-3p	LOC_Os05g01040.1	320	-42.79	164	-22.22	823951	24	2443	793 551
miR1870-3p	LOC_Os05g01040.2	156	-20.57	156	-20.57	823952	24	2250	551
miR1870-3p	LOC_Os05g01040.4	164	-22.22	164	-22.22	823954	24	1056	137
miR1870-3p	LOC_Os05g07470.1	163	-21.2	163	-21.2	824732	24	1647	509
miR1870-3p	LOC_Os05g10920.1	161	-22.13	161	-22.13	825091	24	2800	1576
miR1870-3p	LOC_Os05g14590.1	167	-21.1	167	-21.1	825431	24	2975	405
miR1870-3p	LOC_Os05g16560.1	164	-21	164	-21	825633	24	1884	629
miR1870-3p	LOC_Os05g20160.1	167	-22.62	167	-22.62	826011	24	4413	122
miR1870-3p	LOC_Os05g33050.1	154	-20.93	154	-20.93	827260	24	2896	380
miR1870-3p	LOC_Os05g34380.1	152	-20.51	152	-20.51	827413	24	1410	954
miR1870-3p	LOC_Os05g38100.1	152	-20.54	152	-20.54	827803	24	5511	3058
miR1870-3p	LOC_Os05g52019.1	166	-20.57	166	-20.57	829416	24	987	261
miR1870-3p	LOC_Os06g03640.1	162	-25.64	162	-25.64	829717	24	2110	271
miR1870-3p	LOC_Os06g07080.1	173	-20.91	173	-20.91	830153	24	3710	1078
miR1870-3p	LOC_Os06g11830.1	161	-21.57	161	-21.57	830692	24	2640	2179
miR1870-3p	LOC_Os06g12720.1	176	-22.7	176	-22.7	830802	24	2352	848
miR1870-3p	LOC_Os06g25880.1	154	-21	154	-21	832126	24	4713	4314
miR1870-3p	LOC_Os06g28490.1	163	-20.96	163	-20.96	832296	24	1047	558
miR1870-3p	LOC_Os06g30020.1	163	-23.72	163	-23.72	832455	24	2542	1236
miR1870-3p	LOC_Os06g30300.1	163	-21.82	163	-21.82	832480	24	267	53
miR1870-3p	LOC_Os06g31220.1	147	-20.3	147	-20.3	832583	24	3612	776
miR1870-3p	LOC_Os06g33180.2	167	-29.32	167	-29.32	832767	24	2951	1911
miR1870-3p	LOC_Os06g34380.1	166	-22.54	166	-22.54	832886	24	1230	855
miR1870-3p	LOC_Os06g37520.1	166	-20.57	166	-20.57	833213	24	741	261
miR1870-3p	LOC_Os06g37850.1	169	-20.88	169	-20.88	833247	24	1518	787

miR1870-3p	LOC_Os06g38650.1	160	-20.22	160	-20.22	833328	24	3336	2547
miR1870-3p	LOC_Os06g39090.1	159	-20.24	159	-20.24	833372	24	3573	3135
miR1870-3p	LOC_Os06g40450.1	165	-20.81	165	-20.81	833529	24	1782	711
miR1870-3p	LOC_Os06g40450.2	165	-20.81	165	-20.81	833530	24	1765	711
miR1870-3p	LOC_Os06g40450.3	165	-20.81	165	-20.81	833531	24	1779	711
miR1870-3p	LOC_Os06g47130.1	172	-22.78	172	-22.78	834315	24	2232	1977
miR1870-3p	LOC_Os06g47130.3	172	-22.78	172	-22.78	834317	24	2245	1990
miR1870-3p	LOC_Os06g49120.1	160	-21.05	160	-21.05	834565	24	2206	1170
miR1870-3p	LOC_Os06g49120.2	160	-21.05	160	-21.05	834566	24	2211	1175
miR1870-3p	LOC_Os07g05840.1	167	-22.6	167	-22.6	835418	24	2105	1195
miR1870-3p	LOC_Os07g10120.1	164	-20.29	164	-20.29	835882	24	1646	1167
miR1870-3p	LOC_Os07g15340.1	161	-21.57	161	-21.57	836445	24	2694	2179
miR1870-3p	LOC_Os07g17660.1	176	-22.7	176	-22.7	836678	24	1083	740
miR1870-3p	LOC_Os07g34020.1	168	-24.25	168	-24.25	838234	24	1009	617
miR1870-3p	LOC_Os07g34020.2	168	-24.25	168	-24.25	838235	24	1005	617
miR1870-3p	LOC_Os07g35870.2	159	-20.51	159	-20.51	838447	24	2726	1781
miR1870-3p	LOC_Os07g36780.1	156	-21.47	156	-21.47	838548	24	2817	777
miR1870-3p	LOC_Os07g41750.1	156	-20.46	156	-20.46	839114	24	1096	562
miR1870-3p	LOC_Os07g41750.2	156	-20.46	156	-20.46	839115	24	1054	562
miR1870-3p	LOC_Os07g41750.3	156	-20.46	156	-20.46	839116	24	802	562
miR1870-3p	LOC_Os07g43200.1	153	-22.53	153	-22.53	839295	24	4737	2837
miR1870-3p	LOC_Os07g45620.1	148	-20.45	148	-20.45	839554	24	4620	1369
miR1870-3p	LOC_Os07g45880.1	155	-20.61	155	-20.61	839580	24	2697	382
miR1870-3p	LOC_Os08g02350.1	177	-22.84	177	-22.84	840240	24	8490	4340
miR1870-3p	LOC_Os08g03390.1	161	-20.38	161	-20.38	840358	24	2105	1590
miR1870-3p	LOC_Os08g06800.1	161	-20.69	161	-20.69	840755	24	3441	2042
miR1870-3p	LOC_Os08g11960.1	155	-20.54	155	-20.54	841313	24	5238	1314
miR1870-3p	LOC_Os08g18900.1	162	-21.85	162	-21.85	842002	24	981	177
miR1870-3p	LOC_Os08g21124.1	147	-22.2	147	-22.2	842222	24	3009	2441
miR1870-3p	LOC_Os08g26850.1	156	-20.87	156	-20.87	842794	24	1827	130
miR1870-3p	LOC_Os09g01670.1	150	-21.02	150	-21.02	845018	24	1251	188
miR1870-3p	LOC_Os09g02630.1	149	-20.48	149	-20.48	845118	24	2499	1287

miR1870-3p	LOC_Os09g03160.1	180	-24.05	180	-24.05	845172	24	2724	1563
miR1870-3p	LOC_Os09g15330.1	164	-22.37	164	-22.37	846343	24	2215	2025
miR1870-3p	LOC_Os09g15330.2	164	-22.37	164	-22.37	846344	24	2247	2056
miR1870-3p	LOC_Os09g15570.1	178	-22.29	178	-22.29	846374	24	1812	1350
miR1870-3p	LOC_Os09g15835.1	168	-21.02	168	-21.02	846403	24	1479	224
miR1870-3p	LOC_Os09g19830.1	160	-24.92	160	-24.92	846752	24	3844	2153
miR1870-3p	LOC_Os09g22320.1	171	-24.07	171	-24.07	847022	24	4746	4289
miR1870-3p	LOC_Os09g23300.1	170	-21.79	170	-21.79	847075	24	1230	1046
miR1870-3p	LOC_Os09g26000.1	158	-21.92	158	-21.92	847370	24	2745	686
miR1870-3p	LOC_Os09g26430.1	163	-21.08	163	-21.08	847418	24	4041	1928
miR1870-3p	LOC_Os09g36540.1	149	-20.39	149	-20.39	848448	24	5037	3555
miR1870-3p	LOC_Os09g38940.1	171	-22.77	171	-22.77	848727	24	1149	867
miR1870-3p	LOC_Os10g05740.1	156	-26.24	156	-26.24	849351	24	564	168
miR1870-3p	LOC_Os10g10244.1	172	-22.67	172	-22.67	849788	24	3500	199
miR1870-3p	LOC_Os10g19160.1	159	-22.15	159	-22.15	850451	24	3299	2355
miR1870-3p	LOC_Os10g21470.1	160	-25.11	160	-25.11	850685	24	3784	850
miR1870-3p	LOC_Os10g25340.1	165	-24.31	165	-24.31	851040	24	1261	1143
miR1870-3p	LOC_Os10g39480.1	153	-20.98	153	-20.98	852483	24	630	392
miR1870-3p	LOC_Os10g41300.1	162	-21.89	162	-21.89	852707	24	1388	1079
miR1870-3p	LOC_Os11g04920.1	161	-20.05	161	-20.05	853398	24	922	257
miR1870-3p	LOC_Os11g12290.1	159	-22.51	159	-22.51	854222	24	1867	150
miR1870-3p	LOC_Os11g13510.1	158	-21.82	158	-21.82	854295	24	2304	20
miR1870-3p	LOC_Os11g19970.1	159	-21.22	159	-21.22	854922	24	636	107
miR1870-3p	LOC_Os11g20580.1	162	-20.57	162	-20.57	854980	24	282	166
miR1870-3p	LOC_Os11g24920.1	166	-22.21	166	-22.21	855274	24	2469	494
miR1870-3p	LOC_Os11g25830.1	151	-20.72	151	-20.72	855357	24	990	681
miR1870-3p	LOC_Os11g31230.1	153	-21.31	153	-21.31	855886	24	2145	936
miR1870-3p	LOC_Os11g32510.1	161	-20.49	161	-20.49	856028	24	1057	109
miR1870-3p	LOC_Os11g34110.1	154	-20.08	154	-20.08	856151	24	1644	1125
miR1870-3p	LOC_Os11g41770.1	170	-25.3	170	-25.3	856982	24	1617	530
miR1870-3p	LOC_Os11g42160.1	153	-20.38	153	-20.38	857023	24	1845	768
miR1870-3p	LOC_Os11g44360.1	152	-20.39	152	-20.39	857256	24	378	8

miR1870-3p	LOC_Os12g09250.1	157	-20.09	157	-20.09	858590	24	2347	459
miR1870-3p	LOC_Os12g10810.1	316	-41.93	169	-21.75	858762	24	1422	631 1
miR1870-3p	LOC_Os12g12560.1	163	-20.43	163	-20.43	858931	24	1303	721
miR1870-3p	LOC_Os12g12650.1	150	-20.92	150	-20.92	858941	24	1098	418
miR1870-3p	LOC_Os12g13990.1	163	-22.28	163	-22.28	859090	24	2163	624
miR1870-3p	LOC_Os12g15780.1	170	-20.62	170	-20.62	859258	24	3660	2262
miR1870-3p	LOC_Os12g16880.1	168	-22.47	168	-22.47	859380	24	696	423
miR1870-3p	LOC_Os12g16890.1	168	-22.47	168	-22.47	859381	24	730	426
miR1870-3p	LOC_Os12g17010.1	168	-22.47	168	-22.47	859392	24	697	363
miR1870-3p	LOC_Os12g17030.1	168	-22.47	168	-22.47	859394	24	270	196
miR1870-3p	LOC_Os12g17690.1	158	-20.08	158	-20.08	859463	24	3201	1158
miR1870-3p	LOC_Os12g18390.1	162	-24.45	162	-24.45	859540	24	582	30
miR1870-3p	LOC_Os12g19000.1	154	-20.53	154	-20.53	859605	24	4512	2420
miR1870-3p	LOC_Os12g19180.1	167	-27.13	167	-27.13	859619	24	2278	1792
miR1870-3p	LOC_Os12g20030.1	150	-25.01	150	-25.01	859702	24	855	393
miR1870-3p	LOC_Os12g20190.1	166	-22.66	166	-22.66	859718	24	5133	647
miR1870-3p	LOC_Os12g24050.1	177	-20.62	177	-20.62	860010	24	4863	4673
miR1870-3p	LOC_Os12g28100.1	171	-20.44	171	-20.44	860409	24	4547	1829
miR1870-3p	LOC_Os12g32470.1	151	-20.02	151	-20.02	860842	24	1993	55
miR1870-3p	LOC_Os12g39580.1	154	-22.8	154	-22.8	861639	24	2709	1233
miR1870-3p	LOC_Os12g43990.1	156	-20.29	156	-20.29	862151	24	837	804
miR1846d-3p	LOC_Os01g01120.1	151	-28.85	151	-28.85	862416	20	1206	428
miR1846d-3p	LOC_Os01g01250.1	166	-26.45	166	-26.45	862430	20	2499	14
miR1846d-3p	LOC_Os01g01295.1	159	-27.25	159	-27.25	862435	20	1793	563
miR1846d-3p	LOC_Os01g01560.1	146	-23.14	146	-23.14	862477	20	2240	1690
miR1846d-3p	LOC_Os01g01650.1	159	-34.21	159	-34.21	862485	20	1313	755
miR1846d-3p	LOC_Os01g01770.1	152	-25.26	152	-25.26	862500	20	1005	806
miR1846d-3p	LOC_Os01g01850.1	148	-20.3	148	-20.3	862509	20	6084	507
miR1846d-3p	LOC_Os01g01860.1	144	-24.33	144	-24.33	862510	20	3144	1829
miR1846d-3p	LOC_Os01g02210.1	152	-20.54	152	-20.54	862556	20	1935	397
miR1846d-3p	LOC_Os01g02230.1	145	-20.08	145	-20.08	862558	20	2898	1317
miR1846d-3p	LOC_Os01g02310.1	160	-33.06	160	-33.06	862567	20	1884	432

miR1846d-3p	LOC_Os01g02334.1	157	-28.73	157	-28.73	862569	20	632	563
miR1846d-3p	LOC_Os01g02590.1	154	-21.25	154	-21.25	862598	20	2487	2451
miR1846d-3p	LOC_Os01g02650.1	295	-41.79	148	-21.62	862602	20	6084	507 2546
miR1846d-3p	LOC_Os01g02660.1	172	-35.42	172	-35.42	862603	20	3084	1750
miR1846d-3p	LOC_Os01g02920.1	146	-20.36	146	-20.36	862631	20	2288	705
miR1846d-3p	LOC_Os01g02930.1	161	-26.45	161	-26.45	862632	20	1950	751
miR1846d-3p	LOC_Os01g03080.1	147	-23.19	147	-23.19	862655	20	1001	569
miR1846d-3p	LOC_Os01g03270.1	155	-25	155	-25	862675	20	870	805
miR1846d-3p	LOC_Os01g03330.1	152	-31.68	152	-31.68	862681	20	1863	1052
miR1846d-3p	LOC_Os01g03780.1	305	-47.07	158	-25.45	862725	20	5694	507 2546
miR1846d-3p	LOC_Os01g03810.1	145	-21.97	145	-21.97	862728	20	1025	524
miR1846d-3p	LOC_Os01g03820.1	145	-21.97	145	-21.97	862729	20	3187	524
miR1846d-3p	LOC_Os01g03840.1	141	-21	141	-21	862731	20	989	39
miR1846d-3p	LOC_Os01g03914.1	151	-24.08	151	-24.08	862737	20	2241	1022
miR1846d-3p	LOC_Os01g03914.2	151	-24.08	151	-24.08	862738	20	2233	1022
miR1846d-3p	LOC_Os01g03914.3	151	-24.08	151	-24.08	862739	20	2314	1022
miR1846d-3p	LOC_Os01g04020.1	149	-24.3	149	-24.3	862750	20	1958	1204
miR1846d-3p	LOC_Os01g04020.2	149	-24.3	149	-24.3	862751	20	2132	1378
miR1846d-3p	LOC_Os01g04090.1	144	-21.02	144	-21.02	862757	20	1685	921
miR1846d-3p	LOC_Os01g04250.1	146	-26	146	-26	862778	20	2991	2554
miR1846d-3p	LOC_Os01g04409.1	141	-22.69	141	-22.69	862794	20	2460	2129
miR1846d-3p	LOC_Os01g04580.1	167	-32.5	167	-32.5	862811	20	2292	1799
miR1846d-3p	LOC_Os01g04720.1	148	-21.3	148	-21.3	862829	20	3149	523
miR1846d-3p	LOC_Os01g04720.2	148	-21.3	148	-21.3	862830	20	3869	523
miR1846d-3p	LOC_Os01g04780.1	144	-24.33	144	-24.33	862836	20	8220	6905
miR1846d-3p	LOC_Os01g04814.1	144	-21.18	144	-21.18	862839	20	1888	1675
miR1846d-3p	LOC_Os01g04814.3	144	-21.18	144	-21.18	862840	20	1970	1757
miR1846d-3p	LOC_Os01g04814.4	144	-21.18	144	-21.18	862841	20	1912	1699
miR1846d-3p	LOC_Os01g04910.1	155	-25.09	155	-25.09	862852	20	1057	212
miR1846d-3p	LOC_Os01g04980.1	156	-23.52	156	-23.52	862863	20	2274	504
miR1846d-3p	LOC_Os01g05650.1	148	-23.22	148	-23.22	862940	20	757	593
miR1846d-3p	LOC_Os01g06040.1	298	-43.56	155	-22.55	862976	20	927	96 470

miR1846d-3p	LOC_Os01g06100.1	156	-21.95	156	-21.95	862983	20	2427	507
miR1846d-3p	LOC_Os01g06110.1	172	-36.72	172	-36.72	862984	20	3102	1765
miR1846d-3p	LOC_Os01g06160.1	160	-25.64	160	-25.64	862990	20	2991	1051
miR1846d-3p	LOC_Os01g06170.1	166	-26.59	166	-26.59	862991	20	2688	470
miR1846d-3p	LOC_Os01g06280.1	145	-23.25	145	-23.25	863002	20	3911	1641
miR1846d-3p	LOC_Os01g06280.2	145	-23.25	145	-23.25	863003	20	3315	1853
miR1846d-3p	LOC_Os01g06340.1	156	-27.48	156	-27.48	863012	20	860	225
miR1846d-3p	LOC_Os01g06350.1	156	-26.63	156	-26.63	863013	20	573	1
miR1846d-3p	LOC_Os01g06370.1	156	-26.63	156	-26.63	863015	20	561	1
miR1846d-3p	LOC_Os01g06550.1	156	-25.32	156	-25.32	863035	20	3225	2915
miR1846d-3p	LOC_Os01g07280.1	157	-21.95	157	-21.95	863116	20	2952	283
miR1846d-3p	LOC_Os01g07420.1	150	-24.45	150	-24.45	863143	20	1519	603
miR1846d-3p	LOC_Os01g07560.1	148	-24.57	148	-24.57	863157	20	4026	3152
miR1846d-3p	LOC_Os01g07610.1	150	-21.21	150	-21.21	863161	20	1913	651
miR1846d-3p	LOC_Os01g07930.1	156	-26.36	156	-26.36	863202	20	1212	739
miR1846d-3p	LOC_Os01g07940.1	161	-25.72	161	-25.72	863203	20	2543	1694
miR1846d-3p	LOC_Os01g08170.1	314	-52.57	162	-28.55	863226	20	3604	1801 2071
miR1846d-3p	LOC_Os01g08330.1	150	-21.99	150	-21.99	863246	20	1365	1033
miR1846d-3p	LOC_Os01g08470.1	142	-22.8	142	-22.8	863261	20	2276	512
miR1846d-3p	LOC_Os01g08570.1	157	-27.79	157	-27.79	863272	20	1488	266
miR1846d-3p	LOC_Os01g08750.3	149	-22.33	149	-22.33	863301	20	2706	2251
miR1846d-3p	LOC_Os01g09140.1	157	-25.83	157	-25.83	863342	20	453	150
miR1846d-3p	LOC_Os01g09160.1	166	-26.29	166	-26.29	863344	20	3144	659
miR1846d-3p	LOC_Os01g09290.1	154	-22.44	154	-22.44	863359	20	579	173
miR1846d-3p	LOC_Os01g09520.1	147	-21.62	147	-21.62	863386	20	6042	2546
miR1846d-3p	LOC_Os01g09530.1	316	-61.05	172	-36.72	863387	20	3144	1807 1829
miR1846d-3p	LOC_Os01g09890.1	149	-22.31	149	-22.31	863429	20	1102	1056
miR1846d-3p	LOC_Os01g10250.2	147	-20.99	147	-20.99	863470	20	2646	2020
miR1846d-3p	LOC_Os01g10350.1	157	-24.4	157	-24.4	863484	20	1482	60
miR1846d-3p	LOC_Os01g10420.1	141	-20.59	141	-20.59	863491	20	738	341
miR1846d-3p	LOC_Os01g10710.1	157	-26.88	157	-26.88	863524	20	2538	603
miR1846d-3p	LOC_Os01g10900.1	148	-20.86	148	-20.86	863542	20	2835	2140

miR1846d-3p	LOC_Os01g10900.2	148	-20.86	148	-20.86	863543	20	2420	2122
miR1846d-3p	LOC_Os01g10920.1	160	-29.31	160	-29.31	863545	20	1851	1655
miR1846d-3p	LOC_Os01g11120.1	160	-27.04	160	-27.04	863564	20	1557	1005
miR1846d-3p	LOC_Os01g11220.1	161	-31.25	161	-31.25	863573	20	1661	989
miR1846d-3p	LOC_Os01g11230.1	162	-24.38	162	-24.38	863574	20	1055	828
miR1846d-3p	LOC_Os01g11230.2	162	-24.38	162	-24.38	863575	20	990	763
miR1846d-3p	LOC_Os01g11550.1	145	-22.31	145	-22.31	863602	20	2473	2242
miR1846d-3p	LOC_Os01g11820.1	144	-21.47	144	-21.47	863632	20	1023	517
miR1846d-3p	LOC_Os01g11940.1	151	-25.18	151	-25.18	863642	20	1240	64
miR1846d-3p	LOC_Os01g12530.1	160	-30.65	160	-30.65	863708	20	2270	602
miR1846d-3p	LOC_Os01g12530.2	160	-30.65	160	-30.65	863709	20	2742	602
miR1846d-3p	LOC_Os01g12530.3	160	-30.65	160	-30.65	863710	20	2400	602
miR1846d-3p	LOC_Os01g12530.4	160	-30.65	160	-30.65	863711	20	1658	602
miR1846d-3p	LOC_Os01g12530.5	160	-30.65	160	-30.65	863712	20	3540	602
miR1846d-3p	LOC_Os01g12540.1	158	-27.44	158	-27.44	863714	20	2348	158
miR1846d-3p	LOC_Os01g12540.2	158	-27.44	158	-27.44	863715	20	3336	172
miR1846d-3p	LOC_Os01g12590.1	305	-49.7	158	-28.91	863720	20	6111	507 2573
miR1846d-3p	LOC_Os01g12930.1	153	-26.98	153	-26.98	863769	20	4179	2097
miR1846d-3p	LOC_Os01g13024.1	153	-23.5	153	-23.5	863779	20	1024	477
miR1846d-3p	LOC_Os01g13100.1	141	-21.12	141	-21.12	863793	20	1953	1738
miR1846d-3p	LOC_Os01g13100.2	141	-21.12	141	-21.12	863794	20	1892	1717
miR1846d-3p	LOC_Os01g13100.3	141	-21.12	141	-21.12	863795	20	1885	1670
miR1846d-3p	LOC_Os01g13200.1	145	-20.91	145	-20.91	863804	20	2015	557
miR1846d-3p	LOC_Os01g13890.1	305	-45.5	158	-23.88	863883	20	5178	168 2207
miR1846d-3p	LOC_Os01g14040.1	156	-26.87	156	-26.87	863899	20	965	832
miR1846d-3p	LOC_Os01g14060.1	301	-52.73	154	-26.82	863902	20	807	785 147
miR1846d-3p	LOC_Os01g14420.1	154	-26.16	154	-26.16	863940	20	1390	1341
miR1846d-3p	LOC_Os01g14440.1	147	-22.1	147	-22.1	863942	20	2114	336
miR1846d-3p	LOC_Os01g14700.1	148	-22.91	148	-22.91	863967	20	455	209
miR1846d-3p	LOC_Os01g14720.1	157	-22.54	157	-22.54	863968	20	1209	1083
miR1846d-3p	LOC_Os01g14880.1	149	-24.41	149	-24.41	863988	20	243	107
miR1846d-3p	LOC_Os01g15070.1	152	-22.43	152	-22.43	864011	20	261	72

miR1846d-3p	LOC_Os01g15090.1	152	-22.43	152	-22.43	864013	20	255	66
miR1846d-3p	LOC_Os01g15390.1	162	-30.19	162	-30.19	864042	20	1174	79
miR1846d-3p	LOC_Os01g15570.1	152	-22.43	152	-22.43	864065	20	240	53
miR1846d-3p	LOC_Os01g16040.1	152	-26.16	152	-26.16	864120	20	2029	1868
miR1846d-3p	LOC_Os01g16310.1	145	-21.38	145	-21.38	864160	20	3111	1585
miR1846d-3p	LOC_Os01g16590.1	159	-29.62	159	-29.62	864193	20	408	92
miR1846d-3p	LOC_Os01g16820.1	146	-20.01	146	-20.01	864214	20	3894	17
miR1846d-3p	LOC_Os01g17040.1	144	-23.38	144	-23.38	864244	20	636	378
miR1846d-3p	LOC_Os01g17120.1	151	-21.79	151	-21.79	864255	20	3366	2916
miR1846d-3p	LOC_Os01g17330.1	143	-21.41	143	-21.41	864272	20	1161	1022
miR1846d-3p	LOC_Os01g17330.2	143	-21.41	143	-21.41	864273	20	1288	1149
miR1846d-3p	LOC_Os01g17360.1	147	-21.62	147	-21.62	864274	20	4752	2273
miR1846d-3p	LOC_Os01g17430.1	166	-32.12	166	-32.12	864284	20	3419	3278
miR1846d-3p	LOC_Os01g17450.1	166	-26.59	166	-26.59	864286	20	2610	128
miR1846d-3p	LOC_Os01g17980.1	312	-50.64	156	-25.32	864291	20	855	525 702
miR1846d-3p	LOC_Os01g18780.1	152	-23.16	152	-23.16	864368	20	882	344
miR1846d-3p	LOC_Os01g18930.1	154	-21	154	-21	864391	20	1026	501
miR1846d-3p	LOC_Os01g18980.1	156	-28.42	156	-28.42	864396	20	318	47
miR1846d-3p	LOC_Os01g19190.1	305	-47.07	158	-25.45	864421	20	5553	168 2207
miR1846d-3p	LOC_Os01g19230.1	1055	-157.05	157	-22.94	864425	20	564	372 184 23 123 223 3
miR1846d-3p	LOC_Os01g19240.1	159	-31.29	159	-31.29	864426	20	1077	353
miR1846d-3p	LOC_Os01g19270.1	445	-69.19	150	-24	864429	20	981	354 534 378
miR1846d-3p	LOC_Os01g19390.1	151	-25.37	151	-25.37	864441	20	1362	435
miR1846d-3p	LOC_Os01g19460.1	144	-20.1	144	-20.1	864451	20	3396	3277
miR1846d-3p	LOC_Os01g19580.1	147	-20.9	147	-20.9	864460	20	675	64
miR1846d-3p	LOC_Os01g19620.1	156	-23.52	156	-23.52	864464	20	2226	507
miR1846d-3p	LOC_Os01g19880.1	146	-21.88	146	-21.88	864494	20	764	140
miR1846d-3p	LOC_Os01g20040.1	157	-24.4	157	-24.4	864509	20	903	350
miR1846d-3p	LOC_Os01g20160.1	169	-28.36	169	-28.36	864523	20	2164	1019
miR1846d-3p	LOC_Os01g20710.1	145	-24.1	145	-24.1	864531	20	747	208
miR1846d-3p	LOC_Os01g20730.1	146	-26.39	146	-26.39	864533	20	1130	486
miR1846d-3p	LOC_Os01g20920.1	145	-21.48	145	-21.48	864558	20	363	41

miR1846d-3p	LOC_Os01g21135.1	166	-32.53	166	-32.53	864581	20	1769	1634
miR1846d-3p	LOC_Os01g21144.1	149	-24.74	149	-24.74	864582	20	2753	2248
miR1846d-3p	LOC_Os01g21410.1	153	-23.13	153	-23.13	864608	20	1079	699
miR1846d-3p	LOC_Os01g21640.1	161	-26.42	161	-26.42	864633	20	603	206
miR1846d-3p	LOC_Os01g22030.1	152	-22.21	152	-22.21	864672	20	366	119
miR1846d-3p	LOC_Os01g22100.1	145	-20.13	145	-20.13	864678	20	1569	519
miR1846d-3p	LOC_Os01g22410.1	166	-26.29	166	-26.29	864703	20	3144	659
miR1846d-3p	LOC_Os01g22420.1	148	-27.59	148	-27.59	864704	20	6087	4511
miR1846d-3p	LOC_Os01g22650.1	309	-48.94	158	-25.15	864727	20	429	307 212
miR1846d-3p	LOC_Os01g22740.1	166	-26.29	166	-26.29	864737	20	3072	659
miR1846d-3p	LOC_Os01g22890.1	146	-20.01	146	-20.01	864752	20	3921	17
miR1846d-3p	LOC_Os01g22910.1	599	-105.55	154	-29.54	864756	20	1395	854 1040 243 721
miR1846d-3p	LOC_Os01g23100.1	147	-22.55	147	-22.55	864773	20	981	904
miR1846d-3p	LOC_Os01g23890.1	151	-28.31	151	-28.31	864853	20	930	491
miR1846d-3p	LOC_Os01g24030.1	156	-23.03	156	-23.03	864865	20	2071	625
miR1846d-3p	LOC_Os01g24030.2	156	-23.03	156	-23.03	864866	20	1586	625
miR1846d-3p	LOC_Os01g24040.1	146	-20.01	146	-20.01	864867	20	2322	17
miR1846d-3p	LOC_Os01g24310.1	142	-22.55	142	-22.55	864895	20	3288	1970
miR1846d-3p	LOC_Os01g24350.1	147	-23.96	147	-23.96	864899	20	1299	1121
miR1846d-3p	LOC_Os01g24620.1	166	-26.29	166	-26.29	864926	20	3144	659
miR1846d-3p	LOC_Os01g24764.1	148	-20.57	148	-20.57	864947	20	1360	482
miR1846d-3p	LOC_Os01g25484.1	154	-30.16	154	-30.16	865023	20	2618	184
miR1846d-3p	LOC_Os01g25484.2	154	-30.16	154	-30.16	865024	20	2525	184
miR1846d-3p	LOC_Os01g25520.1	155	-27.34	155	-27.34	865028	20	588	347
miR1846d-3p	LOC_Os01g25580.1	147	-21.62	147	-21.62	865033	20	5775	2552
miR1846d-3p	LOC_Os01g26130.1	149	-26.36	149	-26.36	865086	20	1803	127
miR1846d-3p	LOC_Os01g26130.3	149	-26.36	149	-26.36	865087	20	1511	127
miR1846d-3p	LOC_Os01g26130.4	149	-26.36	149	-26.36	865088	20	1543	127
miR1846d-3p	LOC_Os01g26130.5	149	-26.36	149	-26.36	865089	20	1663	127
miR1846d-3p	LOC_Os01g26980.1	292	-48.58	150	-25.2	865124	20	5739	128 3336
miR1846d-3p	LOC_Os01g27410.1	148	-23.93	148	-23.93	865169	20	297	36
miR1846d-3p	LOC_Os01g27420.1	146	-20.01	146	-20.01	865170	20	4659	17

miR1846d-3p	LOC_Os01g27790.1	305	-47.07	158	-25.45	865206	20	5181	585 2531
miR1846d-3p	LOC_Os01g27930.1	145	-20.08	145	-20.08	865217	20	2325	1959
miR1846d-3p	LOC_Os01g28360.1	162	-24.83	162	-24.83	865260	20	1317	390
miR1846d-3p	LOC_Os01g29080.1	154	-24.76	154	-24.76	865343	20	807	560
miR1846d-3p	LOC_Os01g29260.1	151	-24.69	151	-24.69	865363	20	2220	2007
miR1846d-3p	LOC_Os01g29320.1	148	-26.12	148	-26.12	865369	20	2172	570
miR1846d-3p	LOC_Os01g29580.1	158	-22.95	158	-22.95	865389	20	2622	659
miR1846d-3p	LOC_Os01g29590.1	169	-30.47	169	-30.47	865390	20	5010	774
miR1846d-3p	LOC_Os01g29670.1	143	-21.79	143	-21.79	865397	20	3042	2133
miR1846d-3p	LOC_Os01g29850.1	159	-24.36	159	-24.36	865416	20	1149	63
miR1846d-3p	LOC_Os01g31350.1	153	-23.35	153	-23.35	865466	20	1578	1059
miR1846d-3p	LOC_Os01g31580.1	156	-22.85	156	-22.85	865491	20	2881	2717
miR1846d-3p	LOC_Os01g32184.1	144	-21.52	144	-21.52	865560	20	2117	1975
miR1846d-3p	LOC_Os01g32184.2	144	-21.52	144	-21.52	865561	20	2084	1942
miR1846d-3p	LOC_Os01g32470.1	143	-24.27	143	-24.27	865587	20	759	324
miR1846d-3p	LOC_Os01g32550.1	153	-22.46	153	-22.46	865595	20	1908	358
miR1846d-3p	LOC_Os01g32940.1	303	-49.06	153	-25.48	865639	20	339	282 143
miR1846d-3p	LOC_Os01g33000.1	158	-28.62	158	-28.62	865644	20	1947	127
miR1846d-3p	LOC_Os01g33560.1	147	-21.23	147	-21.23	865700	20	786	358
miR1846d-3p	LOC_Os01g33750.1	151	-20.5	151	-20.5	865716	20	3696	795
miR1846d-3p	LOC_Os01g33800.1	157	-23.5	157	-23.5	865722	20	2278	466
miR1846d-3p	LOC_Os01g33800.2	157	-23.5	157	-23.5	865723	20	2294	466
miR1846d-3p	LOC_Os01g34310.1	154	-31.34	154	-31.34	865765	20	4551	1815
miR1846d-3p	LOC_Os01g34590.1	313	-55.72	159	-29.97	865794	20	844	363 614
miR1846d-3p	LOC_Os01g34614.1	145	-20.6	145	-20.6	865798	20	555	279
miR1846d-3p	LOC_Os01g34770.1	322	-63.78	165	-34.15	865822	20	2143	709 663
miR1846d-3p	LOC_Os01g34800.1	147	-21.62	147	-21.62	865826	20	3282	2546
miR1846d-3p	LOC_Os01g34890.1	144	-23.02	144	-23.02	865835	20	3675	693
miR1846d-3p	LOC_Os01g34910.1	159	-29.06	159	-29.06	865837	20	738	288
miR1846d-3p	LOC_Os01g35110.1	148	-23.45	148	-23.45	865858	20	363	55
miR1846d-3p	LOC_Os01g35390.1	145	-20.08	145	-20.08	865886	20	3513	2055
miR1846d-3p	LOC_Os01g35470.1	165	-28.85	165	-28.85	865894	20	2580	338

miR1846d-3p	LOC_Os01g35480.1	145	-20.08	145	-20.08	865895	20	6039	4422
miR1846d-3p	LOC_Os01g35600.1	150	-20.26	150	-20.26	865912	20	561	136
miR1846d-3p	LOC_Os01g35730.1	165	-28.85	165	-28.85	865925	20	2565	338
miR1846d-3p	LOC_Os01g35740.1	145	-20.08	145	-20.08	865926	20	6039	4422
miR1846d-3p	LOC_Os01g35830.1	161	-26.95	161	-26.95	865936	20	1223	1047
miR1846d-3p	LOC_Os01g36120.1	156	-23.03	156	-23.03	865966	20	408	153
miR1846d-3p	LOC_Os01g36140.1	152	-20.54	152	-20.54	865968	20	1647	397
miR1846d-3p	LOC_Os01g36180.1	145	-20.08	145	-20.08	865971	20	1917	285
miR1846d-3p	LOC_Os01g36390.1	165	-34.38	165	-34.38	865991	20	2878	75
miR1846d-3p	LOC_Os01g36460.1	151	-24.63	151	-24.63	865998	20	1498	1075
miR1846d-3p	LOC_Os01g36540.1	146	-22.95	146	-22.95	866006	20	1710	533
miR1846d-3p	LOC_Os01g36680.1	146	-20.01	146	-20.01	866019	20	3963	17
miR1846d-3p	LOC_Os01g36900.1	149	-23.09	149	-23.09	866046	20	1545	735
miR1846d-3p	LOC_Os01g36984.1	157	-25.27	157	-25.27	866057	20	600	577
miR1846d-3p	LOC_Os01g37050.1	161	-35.95	161	-35.95	866062	20	1480	1263
miR1846d-3p	LOC_Os01g37070.1	147	-21.62	147	-21.62	866064	20	6276	2546
miR1846d-3p	LOC_Os01g37080.1	172	-34.12	172	-34.12	866065	20	3957	2260
miR1846d-3p	LOC_Os01g37130.1	151	-22.3	151	-22.3	866070	20	1438	345
miR1846d-3p	LOC_Os01g37130.2	151	-22.3	151	-22.3	866071	20	1435	345
miR1846d-3p	LOC_Os01g37380.1	148	-21.23	148	-21.23	866103	20	1845	11
miR1846d-3p	LOC_Os01g37540.1	303	-45.14	156	-23.52	866118	20	6084	507 2546
miR1846d-3p	LOC_Os01g37550.1	172	-36.72	172	-36.72	866119	20	3144	1807
miR1846d-3p	LOC_Os01g37780.1	144	-20.28	144	-20.28	866143	20	729	303
miR1846d-3p	LOC_Os01g37880.1	166	-26.29	166	-26.29	866163	20	3144	659
miR1846d-3p	LOC_Os01g37920.1	152	-20.01	152	-20.01	866169	20	357	176
miR1846d-3p	LOC_Os01g37930.1	166	-27.37	166	-27.37	866170	20	3207	659
miR1846d-3p	LOC_Os01g38520.1	152	-21.79	152	-21.79	866229	20	1483	762
miR1846d-3p	LOC_Os01g38540.1	303	-45.14	156	-23.52	866232	20	6081	504 2543
miR1846d-3p	LOC_Os01g38930.1	163	-27.79	163	-27.79	866272	20	849	88
miR1846d-3p	LOC_Os01g38950.1	148	-29.64	148	-29.64	866274	20	2285	161
miR1846d-3p	LOC_Os01g39050.1	151	-27.23	151	-27.23	866287	20	5391	4926
miR1846d-3p	LOC_Os01g39060.1	293	-47.51	149	-26.26	866288	20	2812	136 106

miR1846d-3p	LOC_Os01g39070.1	150	-23.23	150	-23.23	866289	20	665	325
miR1846d-3p	LOC_Os01g39350.1	146	-22.12	146	-22.12	866317	20	1554	1277
miR1846d-3p	LOC_Os01g39380.2	152	-24.43	152	-24.43	866321	20	3936	3741
miR1846d-3p	LOC_Os01g39530.1	145	-21.14	145	-21.14	866335	20	1608	1394
miR1846d-3p	LOC_Os01g39550.1	146	-20.01	146	-20.01	866337	20	3963	17
miR1846d-3p	LOC_Os01g39830.1	143	-24.14	143	-24.14	866374	20	3257	323
miR1846d-3p	LOC_Os01g40640.1	151	-24.25	151	-24.25	866460	20	1932	1645
miR1846d-3p	LOC_Os01g40640.3	151	-24.25	151	-24.25	866461	20	1936	1649
miR1846d-3p	LOC_Os01g40800.1	162	-24.8	162	-24.8	866480	20	2787	659
miR1846d-3p	LOC_Os01g41060.1	166	-26.59	166	-26.59	866509	20	2820	659
miR1846d-3p	LOC_Os01g41080.1	151	-24.69	151	-24.69	866511	20	2298	1983
miR1846d-3p	LOC_Os01g41160.1	152	-20.26	152	-20.26	866518	20	1140	632
miR1846d-3p	LOC_Os01g41220.1	156	-26.53	156	-26.53	866525	20	1277	310
miR1846d-3p	LOC_Os01g41220.2	156	-26.53	156	-26.53	866526	20	1279	306
miR1846d-3p	LOC_Os01g41340.1	144	-22.17	144	-22.17	866538	20	1383	942
miR1846d-3p	LOC_Os01g41370.1	151	-22.15	151	-22.15	866541	20	1896	124
miR1846d-3p	LOC_Os01g41600.1	153	-23.24	153	-23.24	866564	20	1992	224
miR1846d-3p	LOC_Os01g41690.1	303	-45.14	156	-23.52	866574	20	5871	504 2333
miR1846d-3p	LOC_Os01g41890.1	143	-31.71	143	-31.71	866594	20	2964	2474
miR1846d-3p	LOC_Os01g41950.1	147	-21.36	147	-21.36	866600	20	3201	2500
miR1846d-3p	LOC_Os01g41970.1	159	-22.3	159	-22.3	866602	20	618	41
miR1846d-3p	LOC_Os01g42190.1	157	-28.83	157	-28.83	866626	20	1274	765
miR1846d-3p	LOC_Os01g42294.1	156	-22.67	156	-22.67	866638	20	2777	2689
miR1846d-3p	LOC_Os01g42340.1	145	-22.25	145	-22.25	866642	20	2109	766
miR1846d-3p	LOC_Os01g42350.1	143	-21.21	143	-21.21	866643	20	4440	2887
miR1846d-3p	LOC_Os01g42370.1	143	-21.21	143	-21.21	866645	20	4335	2794
miR1846d-3p	LOC_Os01g42710.1	152	-26.16	152	-26.16	866688	20	4918	492
miR1846d-3p	LOC_Os01g42770.1	154	-26.04	154	-26.04	866694	20	754	108
miR1846d-3p	LOC_Os01g42830.1	161	-25.2	161	-25.2	866702	20	1363	156
miR1846d-3p	LOC_Os01g42990.1	152	-27.41	152	-27.41	866724	20	1950	58
miR1846d-3p	LOC_Os01g43210.1	155	-25.58	155	-25.58	866750	20	2970	29
miR1846d-3p	LOC_Os01g43400.1	160	-25.77	160	-25.77	866774	20	477	204

miR1846d-3p	LOC_Os01g43430.1	168	-33.01	168	-33.01	866777	20	858	419
miR1846d-3p	LOC_Os01g43440.1	161	-24.92	161	-24.92	866778	20	1485	1295
miR1846d-3p	LOC_Os01g43800.1	305	-47.07	158	-25.45	866823	20	5175	492 2474
miR1846d-3p	LOC_Os01g43890.1	163	-33.46	163	-33.46	866835	20	2066	1068
miR1846d-3p	LOC_Os01g43895.1	302	-48.69	154	-25.04	866836	20	738	283 428
miR1846d-3p	LOC_Os01g43910.1	158	-26.23	158	-26.23	866837	20	2798	2356
miR1846d-3p	LOC_Os01g44050.1	140	-20.45	140	-20.45	866853	20	1956	764
miR1846d-3p	LOC_Os01g44100.1	152	-30.38	152	-30.38	866856	20	732	324
miR1846d-3p	LOC_Os01g45260.1	151	-27.46	151	-27.46	866927	20	468	338
miR1846d-3p	LOC_Os01g45450.1	311	-50.16	167	-27.96	866948	20	1542	77 1086
miR1846d-3p	LOC_Os01g45460.1	161	-30.66	161	-30.66	866949	20	1517	218
miR1846d-3p	LOC_Os01g45580.1	166	-26.29	166	-26.29	866962	20	3144	659
miR1846d-3p	LOC_Os01g45800.1	166	-27.37	166	-27.37	866984	20	3126	659
miR1846d-3p	LOC_Os01g45860.1	152	-25.43	152	-25.43	866990	20	1678	1483
miR1846d-3p	LOC_Os01g46030.1	157	-25.01	157	-25.01	867008	20	1551	1224
miR1846d-3p	LOC_Os01g46340.1	146	-21.33	146	-21.33	867043	20	3297	2041
miR1846d-3p	LOC_Os01g46350.1	146	-21.33	146	-21.33	867044	20	2658	661
miR1846d-3p	LOC_Os01g46670.1	150	-22.65	150	-22.65	867081	20	699	544
miR1846d-3p	LOC_Os01g46770.1	296	-43.23	149	-21.62	867094	20	6084	507 2546
miR1846d-3p	LOC_Os01g47020.1	166	-26.29	166	-26.29	867122	20	3144	659
miR1846d-3p	LOC_Os01g47030.1	148	-27.59	148	-27.59	867123	20	6087	4511
miR1846d-3p	LOC_Os01g47300.1	145	-20.65	145	-20.65	867152	20	2685	2549
miR1846d-3p	LOC_Os01g47300.2	145	-20.65	145	-20.65	867153	20	823	684
miR1846d-3p	LOC_Os01g47300.3	145	-20.65	145	-20.65	867154	20	786	647
miR1846d-3p	LOC_Os01g47300.4	145	-20.65	145	-20.65	867155	20	849	710
miR1846d-3p	LOC_Os01g47380.1	150	-23.43	150	-23.43	867163	20	612	374
miR1846d-3p	LOC_Os01g47780.1	148	-25.7	148	-25.7	867225	20	1052	827
miR1846d-3p	LOC_Os01g48030.1	149	-22.6	149	-22.6	867249	20	786	497
miR1846d-3p	LOC_Os01g48250.1	165	-32.02	165	-32.02	867275	20	1818	161
miR1846d-3p	LOC_Os01g48260.1	150	-22.62	150	-22.62	867276	20	270	110
miR1846d-3p	LOC_Os01g48270.1	166	-31.41	166	-31.41	867277	20	1706	1383
miR1846d-3p	LOC_Os01g48320.1	145	-23.47	145	-23.47	867286	20	1799	1528

miR1846d-3p	LOC_Os01g49370.1	305	-45.42	153	-24.06	867408	20	981	556 294
miR1846d-3p	LOC_Os01g49970.1	166	-26.59	166	-26.59	867477	20	2712	614
miR1846d-3p	LOC_Os01g50180.1	146	-20.01	146	-20.01	867499	20	4596	650
miR1846d-3p	LOC_Os01g50370.1	154	-20.25	154	-20.25	867517	20	1588	1069
miR1846d-3p	LOC_Os01g50400.1	320	-64.15	165	-34.55	867519	20	1366	730 1037
miR1846d-3p	LOC_Os01g50500.1	143	-21.79	143	-21.79	867529	20	4575	3666
miR1846d-3p	LOC_Os01g50590.1	152	-24.47	152	-24.47	867537	20	1551	164
miR1846d-3p	LOC_Os01g50640.1	147	-21.62	147	-21.62	867548	20	5589	2555
miR1846d-3p	LOC_Os01g50650.1	145	-26.45	145	-26.45	867549	20	3141	1770
miR1846d-3p	LOC_Os01g50740.1	146	-22.12	146	-22.12	867558	20	462	48
miR1846d-3p	LOC_Os01g50770.1	145	-25.25	145	-25.25	867561	20	1941	145
miR1846d-3p	LOC_Os01g50770.2	145	-25.25	145	-25.25	867562	20	1927	145
miR1846d-3p	LOC_Os01g50790.1	166	-27.37	166	-27.37	867564	20	1953	659
miR1846d-3p	LOC_Os01g50840.1	159	-23.79	159	-23.79	867570	20	1281	524
miR1846d-3p	LOC_Os01g50849.1	145	-20.71	145	-20.71	867571	20	2230	1689
miR1846d-3p	LOC_Os01g50849.2	145	-20.71	145	-20.71	867572	20	2304	1763
miR1846d-3p	LOC_Os01g50849.3	145	-20.71	145	-20.71	867573	20	2239	1698
miR1846d-3p	LOC_Os01g51080.1	305	-50.53	158	-28.91	867598	20	2634	507 2471
miR1846d-3p	LOC_Os01g51390.1	147	-20.76	147	-20.76	867639	20	1853	1214
miR1846d-3p	LOC_Os01g51400.1	156	-27.47	156	-27.47	867640	20	2252	742
miR1846d-3p	LOC_Os01g51430.1	158	-24.6	158	-24.6	867644	20	1189	210
miR1846d-3p	LOC_Os01g51754.1	166	-28.15	166	-28.15	867681	20	4408	598
miR1846d-3p	LOC_Os01g51754.2	166	-28.15	166	-28.15	867682	20	4270	598
miR1846d-3p	LOC_Os01g51754.4	166	-28.15	166	-28.15	867684	20	1470	598
miR1846d-3p	LOC_Os01g51820.1	149	-20.35	149	-20.35	867690	20	5964	1149
miR1846d-3p	LOC_Os01g52000.1	157	-27.27	157	-27.27	867709	20	4302	4253
miR1846d-3p	LOC_Os01g52110.1	160	-33.15	160	-33.15	867720	20	3601	3462
miR1846d-3p	LOC_Os01g52110.2	160	-33.15	160	-33.15	867721	20	2021	1882
miR1846d-3p	LOC_Os01g52110.3	160	-33.15	160	-33.15	867722	20	1974	1852
miR1846d-3p	LOC_Os01g52110.4	160	-33.15	160	-33.15	867723	20	3651	3512
miR1846d-3p	LOC_Os01g52110.5	160	-33.15	160	-33.15	867724	20	3761	3622
miR1846d-3p	LOC_Os01g52110.7	160	-33.15	160	-33.15	867725	20	3755	3616

miR1846d-3p	LOC_Os01g52110.8	160	-33.15	160	-33.15	867726	20	1984	1846
miR1846d-3p	LOC_Os01g52110.9	160	-33.15	160	-33.15	867727	20	3847	3708
miR1846d-3p	LOC_Os01g52370.1	156	-23.03	156	-23.03	867755	20	1207	968
miR1846d-3p	LOC_Os01g52390.1	155	-21.87	155	-21.87	867757	20	1614	1489
miR1846d-3p	LOC_Os01g52570.1	140	-24.62	140	-24.62	867781	20	1017	657
miR1846d-3p	LOC_Os01g52770.1	151	-21.9	151	-21.9	867800	20	1712	566
miR1846d-3p	LOC_Os01g52840.1	150	-25.16	150	-25.16	867809	20	1641	1480
miR1846d-3p	LOC_Os01g52851.1	155	-26.83	155	-26.83	867810	20	960	337
miR1846d-3p	LOC_Os01g52880.1	153	-23.93	153	-23.93	867815	20	1519	682
miR1846d-3p	LOC_Os01g52930.1	162	-24.8	162	-24.8	867820	20	2685	659
miR1846d-3p	LOC_Os01g52970.1	145	-22.24	145	-22.24	867824	20	1532	1482
miR1846d-3p	LOC_Os01g53000.1	164	-29.45	164	-29.45	867826	20	3251	1004
miR1846d-3p	LOC_Os01g53000.2	164	-29.45	164	-29.45	867827	20	2713	1004
miR1846d-3p	LOC_Os01g53020.1	149	-25.29	149	-25.29	867829	20	1424	349
miR1846d-3p	LOC_Os01g53130.1	151	-26.11	151	-26.11	867838	20	1395	1034
miR1846d-3p	LOC_Os01g53130.2	151	-26.11	151	-26.11	867839	20	3233	2872
miR1846d-3p	LOC_Os01g53294.1	147	-22.34	147	-22.34	867857	20	2782	219
miR1846d-3p	LOC_Os01g53480.1	143	-26.03	143	-26.03	867875	20	2301	967
miR1846d-3p	LOC_Os01g53660.1	151	-27.97	151	-27.97	867892	20	2736	5
miR1846d-3p	LOC_Os01g53840.1	150	-25.77	150	-25.77	867914	20	2393	1877
miR1846d-3p	LOC_Os01g53920.1	165	-27.7	165	-27.7	867928	20	4265	1966
miR1846d-3p	LOC_Os01g53920.2	165	-27.7	165	-27.7	867929	20	2887	1969
miR1846d-3p	LOC_Os01g53960.1	151	-21.79	151	-21.79	867934	20	4704	3666
miR1846d-3p	LOC_Os01g53990.1	151	-21.63	151	-21.63	867937	20	1372	393
miR1846d-3p	LOC_Os01g53990.2	151	-21.63	151	-21.63	867938	20	1842	863
miR1846d-3p	LOC_Os01g54100.1	156	-26.79	156	-26.79	867952	20	2389	2297
miR1846d-3p	LOC_Os01g54100.2	156	-26.79	156	-26.79	867953	20	2772	2388
miR1846d-3p	LOC_Os01g54119.1	143	-25.39	143	-25.39	867954	20	2491	2419
miR1846d-3p	LOC_Os01g54560.1	293	-49.97	152	-28.1	868004	20	3472	1969 2600
miR1846d-3p	LOC_Os01g54560.2	293	-49.97	152	-28.1	868005	20	3385	1969 2600
miR1846d-3p	LOC_Os01g54560.3	293	-49.97	152	-28.1	868006	20	3241	1969 2600
miR1846d-3p	LOC_Os01g55000.1	167	-31.05	167	-31.05	868051	20	1493	353

miR1846d-3p	LOC_Os01g55050.1	152	-24.54	152	-24.54	868061	20	2225	1918
miR1846d-3p	LOC_Os01g55050.2	160	-23.91	160	-23.91	868062	20	2127	1915
miR1846d-3p	LOC_Os01g55420.1	152	-22.8	152	-22.8	868112	20	1106	668
miR1846d-3p	LOC_Os01g55549.1	157	-26.96	157	-26.96	868132	20	2451	990
miR1846d-3p	LOC_Os01g55610.1	146	-23.69	146	-23.69	868142	20	2119	1594
miR1846d-3p	LOC_Os01g56170.1	144	-22.93	144	-22.93	868209	20	915	681
miR1846d-3p	LOC_Os01g56200.1	166	-35.45	166	-35.45	868212	20	2906	1094
miR1846d-3p	LOC_Os01g56200.2	166	-35.45	166	-35.45	868213	20	2155	1094
miR1846d-3p	LOC_Os01g56460.1	152	-24.25	152	-24.25	868246	20	1266	994
miR1846d-3p	LOC_Os01g56710.1	158	-30.17	158	-30.17	868286	20	1053	794
miR1846d-3p	LOC_Os01g56740.1	150	-26.49	150	-26.49	868289	20	1638	1089
miR1846d-3p	LOC_Os01g56750.1	152	-24.02	152	-24.02	868290	20	1466	506
miR1846d-3p	LOC_Os01g56930.1	152	-26.78	152	-26.78	868316	20	2164	242
miR1846d-3p	LOC_Os01g56930.2	152	-26.78	152	-26.78	868317	20	1546	242
miR1846d-3p	LOC_Os01g57130.1	162	-24.8	162	-24.8	868336	20	2994	659
miR1846d-3p	LOC_Os01g57160.1	151	-20.29	151	-20.29	868339	20	3174	1989
miR1846d-3p	LOC_Os01g57220.2	154	-27.89	154	-27.89	868346	20	2000	1630
miR1846d-3p	LOC_Os01g57230.1	154	-27.89	154	-27.89	868347	20	2646	637
miR1846d-3p	LOC_Os01g57240.1	145	-20.67	145	-20.67	868348	20	1886	1596
miR1846d-3p	LOC_Os01g57430.1	150	-21.32	150	-21.32	868373	20	246	114
miR1846d-3p	LOC_Os01g57630.1	154	-24.33	154	-24.33	868395	20	2084	840
miR1846d-3p	LOC_Os01g57630.2	154	-24.33	154	-24.33	868396	20	1792	835
miR1846d-3p	LOC_Os01g57630.3	154	-24.33	154	-24.33	868397	20	1694	836
miR1846d-3p	LOC_Os01g57940.1	145	-21.04	145	-21.04	868432	20	1727	195
miR1846d-3p	LOC_Os01g58160.1	151	-25.21	151	-25.21	868465	20	795	67
miR1846d-3p	LOC_Os01g58590.1	151	-21.09	151	-21.09	868511	20	459	210
miR1846d-3p	LOC_Os01g58640.1	154	-20.78	154	-20.78	868516	20	2503	1489
miR1846d-3p	LOC_Os01g58710.1	157	-27.18	157	-27.18	868525	20	4023	113
miR1846d-3p	LOC_Os01g58950.1	141	-22.9	141	-22.9	868552	20	1494	1164
miR1846d-3p	LOC_Os01g59070.1	152	-26.21	152	-26.21	868564	20	1783	50
miR1846d-3p	LOC_Os01g59260.1	142	-22.55	142	-22.55	868586	20	3288	1970
miR1846d-3p	LOC_Os01g59690.1	149	-21.68	149	-21.68	868638	20	1763	498

miR1846d-3p	LOC_Os01g59819.1	141	-21.72	141	-21.72	868654	20	1757	283
miR1846d-3p	LOC_Os01g60000.1	150	-24.99	150	-24.99	868675	20	3534	225
miR1846d-3p	LOC_Os01g60020.1	168	-31.8	168	-31.8	868677	20	1364	360
miR1846d-3p	LOC_Os01g60860.1	156	-23.81	156	-23.81	868768	20	2494	2296
miR1846d-3p	LOC_Os01g60970.1	156	-23.52	156	-23.52	868779	20	4989	510
miR1846d-3p	LOC_Os01g61010.1	140	-20.81	140	-20.81	868783	20	2910	157
miR1846d-3p	LOC_Os01g61010.2	140	-20.81	140	-20.81	868784	20	3248	157
miR1846d-3p	LOC_Os01g61020.1	164	-31.71	164	-31.71	868785	20	1551	569
miR1846d-3p	LOC_Os01g61300.1	144	-21.44	144	-21.44	868812	20	399	128
miR1846d-3p	LOC_Os01g61570.1	159	-27.4	159	-27.4	868846	20	1526	703
miR1846d-3p	LOC_Os01g61650.1	158	-22.95	158	-22.95	868858	20	2874	659
miR1846d-3p	LOC_Os01g61930.1	318	-57.72	162	-32.4	868906	20	4610	251 953
miR1846d-3p	LOC_Os01g62010.1	148	-21.38	148	-21.38	868916	20	1044	207
miR1846d-3p	LOC_Os01g62320.1	148	-22.38	148	-22.38	868957	20	3801	844
miR1846d-3p	LOC_Os01g62490.1	156	-25.64	156	-25.64	868985	20	2213	1850
miR1846d-3p	LOC_Os01g62780.1	149	-21.2	149	-21.2	869017	20	1227	172
miR1846d-3p	LOC_Os01g63010.1	159	-31.17	159	-31.17	869049	20	1697	1433
miR1846d-3p	LOC_Os01g63060.1	147	-21.72	147	-21.72	869054	20	1824	481
miR1846d-3p	LOC_Os01g63100.1	305	-47.07	158	-25.45	869057	20	5355	168 2216
miR1846d-3p	LOC_Os01g63170.1	144	-23.56	144	-23.56	869064	20	2892	2424
miR1846d-3p	LOC_Os01g63280.1	153	-22.6	153	-22.6	869080	20	2332	415
miR1846d-3p	LOC_Os01g63280.2	153	-22.6	153	-22.6	869081	20	1786	415
miR1846d-3p	LOC_Os01g63280.3	153	-22.6	153	-22.6	869082	20	2046	415
miR1846d-3p	LOC_Os01g63500.1	144	-23.62	144	-23.62	869105	20	1016	348
miR1846d-3p	LOC_Os01g63580.1	156	-31.65	156	-31.65	869113	20	1715	543
miR1846d-3p	LOC_Os01g63640.1	166	-26.59	166	-26.59	869118	20	2484	470
miR1846d-3p	LOC_Os01g63680.1	156	-24.61	156	-24.61	869122	20	1675	870
miR1846d-3p	LOC_Os01g63890.1	164	-30.27	164	-30.27	869148	20	937	774
miR1846d-3p	LOC_Os01g64280.1	325	-57.04	163	-34.46	869207	20	2580	2445 2186
miR1846d-3p	LOC_Os01g64510.1	144	-22.93	144	-22.93	869232	20	2459	24
miR1846d-3p	LOC_Os01g64540.1	154	-25.5	154	-25.5	869236	20	1607	894
miR1846d-3p	LOC_Os01g64600.1	147	-20.79	147	-20.79	869243	20	5958	2450

miR1846d-3p	LOC_Os01g64610.1	172	-34.12	172	-34.12	869244	20	2988	1753
miR1846d-3p	LOC_Os01g64740.1	152	-24.29	152	-24.29	869268	20	873	738
miR1846d-3p	LOC_Os01g64800.1	160	-33.88	160	-33.88	869280	20	270	194
miR1846d-3p	LOC_Os01g64820.1	153	-23.97	153	-23.97	869283	20	4966	3486
miR1846d-3p	LOC_Os01g64900.1	146	-24.49	146	-24.49	869291	20	5074	4127
miR1846d-3p	LOC_Os01g65000.1	153	-25.22	153	-25.22	869303	20	1497	1090
miR1846d-3p	LOC_Os01g65100.3	173	-35.18	173	-35.18	869319	20	2715	114
miR1846d-3p	LOC_Os01g65300.1	152	-20.66	152	-20.66	869337	20	615	191
miR1846d-3p	LOC_Os01g65500.1	168	-31.71	168	-31.71	869363	20	2956	787
miR1846d-3p	LOC_Os01g65610.1	148	-23.02	148	-23.02	869376	20	1642	1365
miR1846d-3p	LOC_Os01g65650.1	140	-20.01	140	-20.01	869381	20	4110	3553
miR1846d-3p	LOC_Os01g65660.1	140	-20.01	140	-20.01	869382	20	2469	863
miR1846d-3p	LOC_Os01g65660.2	140	-20.01	140	-20.01	869383	20	2095	809
miR1846d-3p	LOC_Os01g65800.1	146	-22.44	146	-22.44	869402	20	2051	1700
miR1846d-3p	LOC_Os01g65904.1	308	-50.08	156	-27.88	869416	20	1522	569 1053
miR1846d-3p	LOC_Os01g66110.1	146	-20.75	146	-20.75	869438	20	3112	2603
miR1846d-3p	LOC_Os01g66110.2	146	-20.75	146	-20.75	869439	20	3150	2603
miR1846d-3p	LOC_Os01g66160.1	157	-24.72	157	-24.72	869445	20	1615	413
miR1846d-3p	LOC_Os01g66200.1	165	-28.16	165	-28.16	869451	20	2682	2303
miR1846d-3p	LOC_Os01g66230.2	146	-21.58	146	-21.58	869454	20	3083	814
miR1846d-3p	LOC_Os01g66440.1	167	-34.69	167	-34.69	869474	20	1041	903
miR1846d-3p	LOC_Os01g66560.1	157	-23.32	157	-23.32	869485	20	2405	2155
miR1846d-3p	LOC_Os01g66720.1	158	-26.78	158	-26.78	869504	20	1265	261
miR1846d-3p	LOC_Os01g66740.1	153	-23.82	153	-23.82	869507	20	1218	791
miR1846d-3p	LOC_Os01g66780.1	151	-24.69	151	-24.69	869510	20	366	315
miR1846d-3p	LOC_Os01g67080.1	151	-28.37	151	-28.37	869548	20	741	557
miR1846d-3p	LOC_Os01g67270.1	151	-24.66	151	-24.66	869569	20	387	95
miR1846d-3p	LOC_Os01g67890.1	153	-20.03	153	-20.03	869650	20	1452	848
miR1846d-3p	LOC_Os01g68040.1	294	-49.09	148	-26.24	869670	20	1911	1858 1137
miR1846d-3p	LOC_Os01g68130.1	142	-20.24	142	-20.24	869679	20	1792	308
miR1846d-3p	LOC_Os01g68320.1	154	-23.31	154	-23.31	869698	20	2805	1048
miR1846d-3p	LOC_Os01g68320.2	154	-23.31	154	-23.31	869699	20	2887	1130

miR1846d-3p	LOC_Os01g68320.3	154	-23.31	154	-23.31	869700	20	4120	2363
miR1846d-3p	LOC_Os01g68320.4	154	-23.31	154	-23.31	869701	20	3030	1273
miR1846d-3p	LOC_Os01g68320.5	154	-23.31	154	-23.31	869702	20	4068	2311
miR1846d-3p	LOC_Os01g68390.1	152	-21.91	152	-21.91	869713	20	1689	1546
miR1846d-3p	LOC_Os01g68410.1	153	-27.86	153	-27.86	869715	20	4629	2664
miR1846d-3p	LOC_Os01g68450.1	141	-21.99	141	-21.99	869720	20	1100	844
miR1846d-3p	LOC_Os01g68450.2	141	-21.99	141	-21.99	869721	20	1200	944
miR1846d-3p	LOC_Os01g68560.1	156	-25.75	156	-25.75	869732	20	1939	848
miR1846d-3p	LOC_Os01g68700.1	156	-29.05	156	-29.05	869747	20	2432	1307
miR1846d-3p	LOC_Os01g68700.2	156	-29.05	156	-29.05	869748	20	2428	1303
miR1846d-3p	LOC_Os01g68740.1	152	-24.33	152	-24.33	869755	20	1294	997
miR1846d-3p	LOC_Os01g68920.1	158	-28.91	158	-28.91	869777	20	1380	489
miR1846d-3p	LOC_Os01g69030.1	152	-23.03	152	-23.03	869791	20	3739	818
miR1846d-3p	LOC_Os01g69030.2	152	-23.03	152	-23.03	869792	20	3805	818
miR1846d-3p	LOC_Os01g69030.3	152	-23.03	152	-23.03	869793	20	3801	818
miR1846d-3p	LOC_Os01g69030.4	152	-23.03	152	-23.03	869794	20	3771	818
miR1846d-3p	LOC_Os01g69090.1	145	-21.32	145	-21.32	869803	20	1553	336
miR1846d-3p	LOC_Os01g70230.1	144	-25.5	144	-25.5	869881	20	897	858
miR1846d-3p	LOC_Os01g70250.1	156	-24.91	156	-24.91	869884	20	2951	2237
miR1846d-3p	LOC_Os01g70730.1	150	-26.21	150	-26.21	869950	20	906	607
miR1846d-3p	LOC_Os01g70790.1	147	-22.75	147	-22.75	869956	20	1528	979
miR1846d-3p	LOC_Os01g70900.1	148	-20.3	148	-20.3	869970	20	2634	507
miR1846d-3p	LOC_Os01g70980.1	158	-25.32	158	-25.32	869984	20	1041	836
miR1846d-3p	LOC_Os01g71020.1	147	-23.89	147	-23.89	869989	20	309	15
miR1846d-3p	LOC_Os01g71510.1	166	-26.29	166	-26.29	870039	20	3135	659
miR1846d-3p	LOC_Os01g71780.1	148	-21.65	148	-21.65	870076	20	1721	462
miR1846d-3p	LOC_Os01g72150.1	150	-21.16	150	-21.16	870114	20	845	629
miR1846d-3p	LOC_Os01g72340.1	154	-21.02	154	-21.02	870135	20	3965	2977
miR1846d-3p	LOC_Os01g72370.1	165	-34.47	165	-34.47	870138	20	4096	3367
miR1846d-3p	LOC_Os01g72370.2	165	-34.47	165	-34.47	870139	20	1800	1071
miR1846d-3p	LOC_Os01g72370.3	165	-34.47	165	-34.47	870140	20	2693	1964
miR1846d-3p	LOC_Os01g72520.1	308	-47.05	157	-25.82	870157	20	1955	1326 1564

miR1846d-3p	LOC_Os01g72720.1	148	-29.84	148	-29.84	870181	20	1155	876
miR1846d-3p	LOC_Os01g72760.1	146	-23.87	146	-23.87	870184	20	1656	690
miR1846d-3p	LOC_Os01g72910.1	160	-26.44	160	-26.44	870205	20	670	335
miR1846d-3p	LOC_Os01g73180.1	157	-25.43	157	-25.43	870236	20	270	78
miR1846d-3p	LOC_Os01g73410.1	308	-59.87	156	-31.06	870260	20	3788	108 432
miR1846d-3p	LOC_Os01g73440.1	151	-28.36	151	-28.36	870263	20	735	77
miR1846d-3p	LOC_Os01g73470.1	152	-22.6	152	-22.6	870266	20	1424	1382
miR1846d-3p	LOC_Os01g73580.1	147	-20.01	147	-20.01	870279	20	2186	1014
miR1846d-3p	LOC_Os01g74050.1	166	-26.45	166	-26.45	870341	20	966	659
miR1846d-3p	LOC_Os01g74080.1	148	-20.3	148	-20.3	870344	20	2226	507
miR1846d-3p	LOC_Os01g74220.1	140	-21.04	140	-21.04	870360	20	3165	1480
miR1846d-3p	LOC_Os01g74260.1	305	-50.53	158	-28.91	870364	20	5682	507 2474
miR1846d-3p	LOC_Os01g74360.1	155	-25.28	155	-25.28	870377	20	306	210
miR1846d-3p	LOC_Os01g74650.3	148	-22.72	148	-22.72	870413	20	2194	1971
miR1846d-3p	LOC_Os01g74680.1	147	-21.62	147	-21.62	870416	20	5454	2546
miR1846d-3p	LOC_Os01g74690.1	172	-34.12	172	-34.12	870417	20	3105	1828
miR1846d-3p	LOC_Os02g01160.1	146	-23.53	146	-23.53	870442	20	306	189
miR1846d-3p	LOC_Os02g01270.1	153	-24.32	153	-24.32	870457	20	2954	2279
miR1846d-3p	LOC_Os02g01390.1	147	-20.79	147	-20.79	870473	20	5913	2546
miR1846d-3p	LOC_Os02g01400.1	172	-34.12	172	-34.12	870474	20	2973	1786
miR1846d-3p	LOC_Os02g01580.1	168	-32.43	168	-32.43	870493	20	2502	1270
miR1846d-3p	LOC_Os02g02080.1	152	-23.13	152	-23.13	870554	20	426	79
miR1846d-3p	LOC_Os02g02140.1	145	-24.12	145	-24.12	870560	20	3442	1904
miR1846d-3p	LOC_Os02g02550.1	165	-26.88	165	-26.88	870608	20	1390	287
miR1846d-3p	LOC_Os02g02580.1	141	-20.68	141	-20.68	870611	20	1570	407
miR1846d-3p	LOC_Os02g02900.1	161	-27.19	161	-27.19	870652	20	1222	389
miR1846d-3p	LOC_Os02g02970.1	152	-22.91	152	-22.91	870666	20	1801	727
miR1846d-3p	LOC_Os02g03160.1	146	-20.01	146	-20.01	870695	20	3963	17
miR1846d-3p	LOC_Os02g03450.1	146	-22	146	-22	870731	20	1150	82
miR1846d-3p	LOC_Os02g03600.1	158	-20.76	158	-20.76	870752	20	1967	1596
miR1846d-3p	LOC_Os02g03750.1	151	-24.13	151	-24.13	870771	20	2002	1273
miR1846d-3p	LOC_Os02g03910.1	152	-22.26	152	-22.26	870790	20	987	740

miR1846d-3p	LOC_Os02g03950.1	145	-21.6	145	-21.6	870794	20	1886	206
miR1846d-3p	LOC_Os02g03950.2	145	-21.6	145	-21.6	870795	20	1589	206
miR1846d-3p	LOC_Os02g03950.3	145	-21.6	145	-21.6	870796	20	2112	206
miR1846d-3p	LOC_Os02g03950.4	145	-21.6	145	-21.6	870797	20	1415	170
miR1846d-3p	LOC_Os02g03950.5	145	-21.6	145	-21.6	870798	20	1528	170
miR1846d-3p	LOC_Os02g04140.1	166	-27.37	166	-27.37	870822	20	3207	659
miR1846d-3p	LOC_Os02g04290.1	166	-26.59	166	-26.59	870845	20	3024	659
miR1846d-3p	LOC_Os02g04380.1	157	-30.64	157	-30.64	870857	20	2250	1593
miR1846d-3p	LOC_Os02g04430.1	147	-21.37	147	-21.37	870864	20	2379	888
miR1846d-3p	LOC_Os02g04680.1	147	-23.55	147	-23.55	870898	20	2517	611
miR1846d-3p	LOC_Os02g04680.2	147	-23.55	147	-23.55	870899	20	2514	611
miR1846d-3p	LOC_Os02g05420.1	154	-21.47	154	-21.47	870999	20	1686	237
miR1846d-3p	LOC_Os02g05450.1	144	-25.07	144	-25.07	871002	20	2750	463
miR1846d-3p	LOC_Os02g05590.1	161	-28.39	161	-28.39	871019	20	669	79
miR1846d-3p	LOC_Os02g05600.1	160	-26.79	160	-26.79	871020	20	1714	438
miR1846d-3p	LOC_Os02g05680.1	151	-23.33	151	-23.33	871031	20	1477	158
miR1846d-3p	LOC_Os02g05680.2	151	-23.33	151	-23.33	871032	20	2423	158
miR1846d-3p	LOC_Os02g05700.1	149	-21.7	149	-21.7	871036	20	2657	397
miR1846d-3p	LOC_Os02g05700.2	149	-21.7	149	-21.7	871037	20	2903	397
miR1846d-3p	LOC_Os02g05850.1	158	-25.45	158	-25.45	871054	20	4035	301
miR1846d-3p	LOC_Os02g06040.1	166	-26.29	166	-26.29	871073	20	3132	659
miR1846d-3p	LOC_Os02g06205.1	157	-26.03	157	-26.03	871091	20	2373	1679
miR1846d-3p	LOC_Os02g06210.1	157	-26.03	157	-26.03	871092	20	5466	1679
miR1846d-3p	LOC_Os02g06220.1	145	-21.62	145	-21.62	871094	20	795	111
miR1846d-3p	LOC_Os02g06260.1	157	-26.03	157	-26.03	871098	20	2295	1856
miR1846d-3p	LOC_Os02g06430.1	150	-25.52	150	-25.52	871116	20	2629	2221
miR1846d-3p	LOC_Os02g06480.1	154	-23.77	154	-23.77	871121	20	3602	2804
miR1846d-3p	LOC_Os02g06480.2	154	-23.77	154	-23.77	871122	20	3596	2798
miR1846d-3p	LOC_Os02g06584.2	161	-27.11	161	-27.11	871134	20	2258	53
miR1846d-3p	LOC_Os02g06584.3	161	-27.11	161	-27.11	871135	20	2079	53
miR1846d-3p	LOC_Os02g06584.4	161	-27.11	161	-27.11	871136	20	2233	33
miR1846d-3p	LOC_Os02g06630.1	147	-20.39	147	-20.39	871142	20	2034	1922

miR1846d-3p	LOC_Os02g06880.1	296	-48.84	151	-24.55	871175	20	2521	1760 1966
miR1846d-3p	LOC_Os02g06980.1	149	-26.91	149	-26.91	871188	20	693	83
miR1846d-3p	LOC_Os02g07180.1	148	-26.43	148	-26.43	871216	20	1699	191
miR1846d-3p	LOC_Os02g07190.1	156	-23.52	156	-23.52	871217	20	2889	776
miR1846d-3p	LOC_Os02g07260.2	160	-29.28	160	-29.28	871232	20	1841	211
miR1846d-3p	LOC_Os02g07370.1	158	-28.91	158	-28.91	871244	20	1752	507
miR1846d-3p	LOC_Os02g07380.1	147	-21.62	147	-21.62	871245	20	4716	1445
miR1846d-3p	LOC_Os02g07490.1	308	-53.58	160	-31.76	871258	20	1670	150 1067
miR1846d-3p	LOC_Os02g07530.1	157	-23.73	157	-23.73	871263	20	2436	338
miR1846d-3p	LOC_Os02g07540.1	145	-20.08	145	-20.08	871264	20	5895	4275
miR1846d-3p	LOC_Os02g07560.1	166	-26.29	166	-26.29	871266	20	3144	659
miR1846d-3p	LOC_Os02g07750.1	148	-20.96	148	-20.96	871295	20	1720	1268
miR1846d-3p	LOC_Os02g07940.1	158	-28.91	158	-28.91	871320	20	1398	507
miR1846d-3p	LOC_Os02g08120.1	158	-26.55	158	-26.55	871343	20	2291	2194
miR1846d-3p	LOC_Os02g08120.2	158	-26.55	158	-26.55	871344	20	2276	2179
miR1846d-3p	LOC_Os02g08420.1	145	-20.56	145	-20.56	871380	20	1697	888
miR1846d-3p	LOC_Os02g08500.1	148	-23.11	148	-23.11	871390	20	2485	570
miR1846d-3p	LOC_Os02g09410.1	299	-47.91	152	-26.14	871436	20	1572	730 702
miR1846d-3p	LOC_Os02g09440.1	152	-25.49	152	-25.49	871439	20	1233	50
miR1846d-3p	LOC_Os02g09550.1	305	-45.5	158	-23.88	871451	20	5619	507 2555
miR1846d-3p	LOC_Os02g09690.1	145	-22.24	145	-22.24	871470	20	2442	718
miR1846d-3p	LOC_Os02g09720.1	290	-46.15	147	-24.73	871472	20	4676	3284 1615
miR1846d-3p	LOC_Os02g09780.1	150	-24.36	150	-24.36	871482	20	702	406
miR1846d-3p	LOC_Os02g10180.1	144	-21.52	144	-21.52	871531	20	2016	1788
miR1846d-3p	LOC_Os02g10180.2	144	-21.52	144	-21.52	871532	20	2005	1791
miR1846d-3p	LOC_Os02g10270.1	140	-20.04	140	-20.04	871543	20	5370	3744
miR1846d-3p	LOC_Os02g10280.1	148	-21.64	148	-21.64	871544	20	1332	1144
miR1846d-3p	LOC_Os02g10440.1	152	-22.53	152	-22.53	871565	20	2228	173
miR1846d-3p	LOC_Os02g10770.2	159	-28.7	159	-28.7	871605	20	2132	2012
miR1846d-3p	LOC_Os02g11090.1	146	-21.51	146	-21.51	871653	20	1653	1519
miR1846d-3p	LOC_Os02g12430.1	291	-46.04	146	-23.43	871748	20	550	300 112
miR1846d-3p	LOC_Os02g12520.1	145	-20.8	145	-20.8	871767	20	348	87

miR1846d-3p	LOC_Os02g12570.1	149	-22.26	149	-22.26	871772	20	1632	1275
miR1846d-3p	LOC_Os02g12620.1	152	-23.26	152	-23.26	871777	20	1632	504
miR1846d-3p	LOC_Os02g12810.1	161	-23.9	161	-23.9	871805	20	3222	2597
miR1846d-3p	LOC_Os02g12810.2	161	-23.9	161	-23.9	871806	20	3487	2617
miR1846d-3p	LOC_Os02g12810.3	161	-23.9	161	-23.9	871807	20	2331	1706
miR1846d-3p	LOC_Os02g12810.4	161	-23.9	161	-23.9	871808	20	2987	2704
miR1846d-3p	LOC_Os02g12980.1	147	-21.62	147	-21.62	871835	20	5025	2555
miR1846d-3p	LOC_Os02g13250.1	151	-23.12	151	-23.12	871865	20	2526	1314
miR1846d-3p	LOC_Os02g13410.1	165	-29.42	165	-29.42	871883	20	1527	1421
miR1846d-3p	LOC_Os02g13420.1	155	-33.63	155	-33.63	871884	20	1836	1104
miR1846d-3p	LOC_Os02g13430.1	148	-23.88	148	-23.88	871885	20	3203	1305
miR1846d-3p	LOC_Os02g13440.1	154	-24.57	154	-24.57	871886	20	684	573
miR1846d-3p	LOC_Os02g13510.1	283	-43.04	142	-23.02	871893	20	4059	2431 3574
miR1846d-3p	LOC_Os02g13800.1	172	-30.02	172	-30.02	871926	20	1489	451
miR1846d-3p	LOC_Os02g13950.1	150	-22.51	150	-22.51	871943	20	2090	910
miR1846d-3p	LOC_Os02g14090.1	142	-22.85	142	-22.85	871962	20	2013	1356
miR1846d-3p	LOC_Os02g14100.1	159	-25.09	159	-25.09	871963	20	1464	909
miR1846d-3p	LOC_Os02g14110.1	151	-22.57	151	-22.57	871964	20	2459	397
miR1846d-3p	LOC_Os02g14220.1	162	-30	162	-30	871975	20	4134	1080
miR1846d-3p	LOC_Os02g14290.1	144	-20.25	144	-20.25	871981	20	1371	1211
miR1846d-3p	LOC_Os02g14290.2	144	-20.25	144	-20.25	871982	20	1068	908
miR1846d-3p	LOC_Os02g14300.1	144	-20.25	144	-20.25	871983	20	630	38
miR1846d-3p	LOC_Os02g14440.1	156	-31.19	156	-31.19	871997	20	1300	309
miR1846d-3p	LOC_Os02g14440.2	156	-31.19	156	-31.19	871998	20	1435	309
miR1846d-3p	LOC_Os02g14460.1	151	-24.76	151	-24.76	872000	20	1005	178
miR1846d-3p	LOC_Os02g14700.1	160	-28.28	160	-28.28	872023	20	2076	802
miR1846d-3p	LOC_Os02g14790.1	155	-24	155	-24	872038	20	1426	1351
miR1846d-3p	LOC_Os02g15720.1	153	-22.44	153	-22.44	872146	20	951	505
miR1846d-3p	LOC_Os02g15770.1	445	-65.89	151	-25.27	872151	20	615	183 82 350
miR1846d-3p	LOC_Os02g16040.1	153	-23.6	153	-23.6	872189	20	1016	131
miR1846d-3p	LOC_Os02g16040.2	153	-23.6	153	-23.6	872190	20	1539	131
miR1846d-3p	LOC_Os02g16760.1	155	-26.02	155	-26.02	872266	20	1173	1085

miR1846d-3p	LOC_Os02g16770.1	299	-46.29	155	-26.02	872267	20	1341	766 1246
miR1846d-3p	LOC_Os02g16990.1	148	-20.37	148	-20.37	872289	20	2625	441
miR1846d-3p	LOC_Os02g17180.1	159	-33.5	159	-33.5	872309	20	1143	384
miR1846d-3p	LOC_Os02g17292.1	149	-27.26	149	-27.26	872320	20	5638	629
miR1846d-3p	LOC_Os02g17420.1	170	-30.64	170	-30.64	872335	20	3027	599
miR1846d-3p	LOC_Os02g17620.1	156	-24.15	156	-24.15	872356	20	952	745
miR1846d-3p	LOC_Os02g17640.1	145	-20.74	145	-20.74	872358	20	1311	374
miR1846d-3p	LOC_Os02g18020.1	152	-26.88	152	-26.88	872405	20	849	702
miR1846d-3p	LOC_Os02g18190.1	312	-57.4	160	-29.95	872426	20	339	255 227
miR1846d-3p	LOC_Os02g18210.1	150	-22.95	150	-22.95	872428	20	2970	2755
miR1846d-3p	LOC_Os02g18260.1	157	-27.73	157	-27.73	872433	20	555	381
miR1846d-3p	LOC_Os02g18280.1	151	-24.69	151	-24.69	872435	20	1404	1104
miR1846d-3p	LOC_Os02g18380.1	153	-27.54	153	-27.54	872445	20	767	743
miR1846d-3p	LOC_Os02g18400.1	146	-20.04	146	-20.04	872447	20	462	184
miR1846d-3p	LOC_Os02g18510.1	147	-21.69	147	-21.69	872458	20	2709	679
miR1846d-3p	LOC_Os02g18550.1	157	-30.49	157	-30.49	872463	20	1233	599
miR1846d-3p	LOC_Os02g18770.1	141	-21.83	141	-21.83	872486	20	324	239
miR1846d-3p	LOC_Os02g18784.1	608	-104.06	156	-28.39	872488	20	917	592 646 416 543
miR1846d-3p	LOC_Os02g19100.1	153	-26.82	153	-26.82	872521	20	2301	1515
miR1846d-3p	LOC_Os02g19200.1	152	-27.62	152	-27.62	872531	20	4684	4099
miR1846d-3p	LOC_Os02g19380.1	153	-31.46	153	-31.46	872552	20	1038	509
miR1846d-3p	LOC_Os02g19390.1	154	-28.81	154	-28.81	872553	20	1521	1188
miR1846d-3p	LOC_Os02g19470.1	164	-30.74	164	-30.74	872563	20	2692	303
miR1846d-3p	LOC_Os02g19540.1	157	-31.48	157	-31.48	872571	20	723	632
miR1846d-3p	LOC_Os02g19950.1	145	-20.71	145	-20.71	872608	20	1236	881
miR1846d-3p	LOC_Os02g19960.1	159	-22.08	159	-22.08	872609	20	192	145
miR1846d-3p	LOC_Os02g20220.1	147	-21.62	147	-21.62	872637	20	2691	2264
miR1846d-3p	LOC_Os02g20260.1	172	-34.12	172	-34.12	872641	20	2886	1753
miR1846d-3p	LOC_Os02g20420.1	147	-22.02	147	-22.02	872660	20	6192	5289
miR1846d-3p	LOC_Os02g21260.1	160	-26.1	160	-26.1	872752	20	1609	92
miR1846d-3p	LOC_Os02g21840.1	149	-21.69	149	-21.69	872813	20	3063	799
miR1846d-3p	LOC_Os02g22020.1	153	-21.3	153	-21.3	872832	20	1711	630

miR1846d-3p	LOC_Os02g22410.1	147	-21.62	147	-21.62	872883	20	3279	719
miR1846d-3p	LOC_Os02g22550.1	157	-24.4	157	-24.4	872897	20	531	314
miR1846d-3p	LOC_Os02g23939.1	158	-30.67	158	-30.67	872950	20	3666	181
miR1846d-3p	LOC_Os02g23939.2	158	-30.67	158	-30.67	872951	20	2970	181
miR1846d-3p	LOC_Os02g23939.3	158	-30.67	158	-30.67	872952	20	2879	181
miR1846d-3p	LOC_Os02g24040.1	152	-22.43	152	-22.43	872962	20	255	66
miR1846d-3p	LOC_Os02g24550.1	148	-22.32	148	-22.32	873015	20	3261	1341
miR1846d-3p	LOC_Os02g24780.1	151	-24.26	151	-24.26	873045	20	3258	2964
miR1846d-3p	LOC_Os02g25590.1	166	-27.94	166	-27.94	873126	20	1116	904
miR1846d-3p	LOC_Os02g25624.1	162	-28.72	162	-28.72	873130	20	1379	1132
miR1846d-3p	LOC_Os02g25800.1	144	-22.93	144	-22.93	873150	20	2208	24
miR1846d-3p	LOC_Os02g25930.1	153	-22.64	153	-22.64	873164	20	3936	1152
miR1846d-3p	LOC_Os02g25990.1	157	-24.93	157	-24.93	873170	20	642	504
miR1846d-3p	LOC_Os02g26190.1	156	-28.52	156	-28.52	873187	20	6728	1017
miR1846d-3p	LOC_Os02g26190.2	156	-28.52	156	-28.52	873188	20	6844	1017
miR1846d-3p	LOC_Os02g26200.1	148	-25.29	148	-25.29	873189	20	732	134
miR1846d-3p	LOC_Os02g26600.1	145	-21.67	145	-21.67	873242	20	3141	717
miR1846d-3p	LOC_Os02g26890.1	147	-24.75	147	-24.75	873284	20	1521	984
miR1846d-3p	LOC_Os02g26920.1	305	-45.5	158	-23.88	873287	20	5619	507 2546
miR1846d-3p	LOC_Os02g27230.1	305	-47.07	158	-25.45	873319	20	5661	489 2537
miR1846d-3p	LOC_Os02g27300.1	157	-30.73	157	-30.73	873326	20	624	528
miR1846d-3p	LOC_Os02g27650.1	157	-27.73	157	-27.73	873370	20	2781	2388
miR1846d-3p	LOC_Os02g27790.1	152	-26.97	152	-26.97	873385	20	3621	489
miR1846d-3p	LOC_Os02g28010.1	151	-21.79	151	-21.79	873408	20	4554	3378
miR1846d-3p	LOC_Os02g28290.1	143	-21.22	143	-21.22	873434	20	312	182
miR1846d-3p	LOC_Os02g28730.1	938	-154.79	162	-29.61	873475	20	570	434 273 3 123 363 39
miR1846d-3p	LOC_Os02g28840.1	147	-20.45	147	-20.45	873485	20	453	264
miR1846d-3p	LOC_Os02g28970.1	157	-27.08	157	-27.08	873498	20	2023	461
miR1846d-3p	LOC_Os02g28970.2	157	-27.08	157	-27.08	873499	20	1986	461
miR1846d-3p	LOC_Os02g28980.1	144	-22.52	144	-22.52	873500	20	2427	267
miR1846d-3p	LOC_Os02g29050.1	155	-24.1	155	-24.1	873508	20	1695	1019
miR1846d-3p	LOC_Os02g29590.1	144	-22.11	144	-22.11	873565	20	1285	507

miR1846d-3p	LOC_Os02g29660.1	141	-20.12	141	-20.12	873572	20	1764	393
miR1846d-3p	LOC_Os02g29850.1	153	-32.64	153	-32.64	873592	20	1137	152
miR1846d-3p	LOC_Os02g30120.1	166	-27.37	166	-27.37	873621	20	2718	659
miR1846d-3p	LOC_Os02g30320.1	147	-21.71	147	-21.71	873648	20	1193	485
miR1846d-3p	LOC_Os02g31100.1	161	-31.97	161	-31.97	873739	20	859	670
miR1846d-3p	LOC_Os02g31160.1	153	-26.44	153	-26.44	873744	20	1125	321
miR1846d-3p	LOC_Os02g31180.1	145	-20.08	145	-20.08	873746	20	5520	4356
miR1846d-3p	LOC_Os02g31840.1	158	-27.44	158	-27.44	873764	20	1107	807
miR1846d-3p	LOC_Os02g32660.1	145	-22.86	145	-22.86	873867	20	2958	2837
miR1846d-3p	LOC_Os02g32660.2	145	-22.86	145	-22.86	873868	20	2268	2147
miR1846d-3p	LOC_Os02g32690.1	145	-22.61	145	-22.61	873870	20	4495	2619
miR1846d-3p	LOC_Os02g32690.2	145	-22.61	145	-22.61	873871	20	4755	2879
miR1846d-3p	LOC_Os02g32870.1	147	-20.45	147	-20.45	873887	20	1917	1
miR1846d-3p	LOC_Os02g32930.1	149	-21.02	149	-21.02	873893	20	1955	248
miR1846d-3p	LOC_Os02g32960.1	148	-25.58	148	-25.58	873896	20	633	550
miR1846d-3p	LOC_Os02g33210.1	144	-21.09	144	-21.09	873925	20	1812	225
miR1846d-3p	LOC_Os02g33280.1	140	-21.54	140	-21.54	873932	20	3936	3563
miR1846d-3p	LOC_Os02g33300.1	148	-20.79	148	-20.79	873935	20	315	45
miR1846d-3p	LOC_Os02g33335.1	153	-25.56	153	-25.56	873941	20	1078	781
miR1846d-3p	LOC_Os02g33720.1	156	-30.42	156	-30.42	873983	20	609	229
miR1846d-3p	LOC_Os02g34140.1	157	-27.12	157	-27.12	874019	20	366	17
miR1846d-3p	LOC_Os02g34170.1	166	-26.29	166	-26.29	874022	20	3132	659
miR1846d-3p	LOC_Os02g34180.1	145	-25.97	145	-25.97	874023	20	6087	4509
miR1846d-3p	LOC_Os02g34550.1	142	-23.38	142	-23.38	874059	20	4371	2304
miR1846d-3p	LOC_Os02g34860.1	148	-22.2	148	-22.2	874094	20	1757	1295
miR1846d-3p	LOC_Os02g34960.1	164	-30.37	164	-30.37	874104	20	2503	1269
miR1846d-3p	LOC_Os02g35010.1	150	-24.73	150	-24.73	874110	20	2636	497
miR1846d-3p	LOC_Os02g35020.1	154	-26.04	154	-26.04	874111	20	2307	1152
miR1846d-3p	LOC_Os02g35140.1	148	-27.31	148	-27.31	874129	20	3399	237
miR1846d-3p	LOC_Os02g35144.1	158	-25.37	158	-25.37	874130	20	1888	1773
miR1846d-3p	LOC_Os02g35220.1	145	-22.42	145	-22.42	874141	20	2065	1412
miR1846d-3p	LOC_Os02g35290.1	140	-20.76	140	-20.76	874148	20	4305	4217

miR1846d-3p	LOC_Os02g35800.1	158	-28.91	158	-28.91	874205	20	2304	507
miR1846d-3p	LOC_Os02g35840.1	151	-28.71	151	-28.71	874210	20	1820	1696
miR1846d-3p	LOC_Os02g35980.1	340	-61.77	173	-32.74	874225	20	465	322 352
miR1846d-3p	LOC_Os02g36440.1	157	-26.14	157	-26.14	874280	20	1912	1494
miR1846d-3p	LOC_Os02g36450.1	157	-25.2	157	-25.2	874281	20	1866	1452
miR1846d-3p	LOC_Os02g36450.2	157	-25.2	157	-25.2	874282	20	1753	1341
miR1846d-3p	LOC_Os02g36520.1	144	-20.44	144	-20.44	874292	20	2797	2128
miR1846d-3p	LOC_Os02g36570.1	163	-32.72	163	-32.72	874297	20	2858	268
miR1846d-3p	LOC_Os02g36800.1	146	-20.01	146	-20.01	874327	20	4068	797
miR1846d-3p	LOC_Os02g36850.1	148	-27.8	148	-27.8	874333	20	926	402
miR1846d-3p	LOC_Os02g36870.1	151	-24.34	151	-24.34	874335	20	1011	378
miR1846d-3p	LOC_Os02g36924.1	146	-21.22	146	-21.22	874342	20	1085	296
miR1846d-3p	LOC_Os02g36990.1	158	-29.58	158	-29.58	874354	20	1116	925
miR1846d-3p	LOC_Os02g37254.1	151	-25.32	151	-25.32	874387	20	751	485
miR1846d-3p	LOC_Os02g37420.1	145	-25.41	145	-25.41	874403	20	1283	84
miR1846d-3p	LOC_Os02g37440.1	156	-26.35	156	-26.35	874405	20	1893	441
miR1846d-3p	LOC_Os02g37790.1	141	-25.59	141	-25.59	874439	20	1227	326
miR1846d-3p	LOC_Os02g37830.1	147	-21.54	147	-21.54	874443	20	2190	1736
miR1846d-3p	LOC_Os02g37830.2	147	-21.54	147	-21.54	874444	20	2273	1819
miR1846d-3p	LOC_Os02g37880.1	148	-22.89	148	-22.89	874457	20	2574	2123
miR1846d-3p	LOC_Os02g37880.2	148	-22.89	148	-22.89	874458	20	2723	2272
miR1846d-3p	LOC_Os02g37880.3	148	-22.89	148	-22.89	874459	20	2722	2271
miR1846d-3p	LOC_Os02g37960.1	160	-23.97	160	-23.97	874470	20	1836	698
miR1846d-3p	LOC_Os02g38040.1	144	-20.55	144	-20.55	874478	20	1978	747
miR1846d-3p	LOC_Os02g38190.1	161	-27.47	161	-27.47	874500	20	1030	543
miR1846d-3p	LOC_Os02g38240.1	160	-28.29	160	-28.29	874505	20	1256	1145
miR1846d-3p	LOC_Os02g38310.1	299	-45.97	151	-23.94	874514	20	1044	687 78
miR1846d-3p	LOC_Os02g38370.1	166	-27.37	166	-27.37	874521	20	2970	659
miR1846d-3p	LOC_Os02g38520.1	147	-21.62	147	-21.62	874540	20	5007	2411
miR1846d-3p	LOC_Os02g38660.1	147	-21.62	147	-21.62	874555	20	5097	2027
miR1846d-3p	LOC_Os02g38930.1	154	-28.05	154	-28.05	874587	20	1389	570
miR1846d-3p	LOC_Os02g38970.1	157	-25.59	157	-25.59	874591	20	3019	710

miR1846d-3p	LOC_Os02g38970.2	157	-25.59	157	-25.59	874592	20	2769	460
miR1846d-3p	LOC_Os02g38980.1	151	-24.74	151	-24.74	874593	20	3191	2883
miR1846d-3p	LOC_Os02g38980.3	151	-24.74	151	-24.74	874594	20	3355	3047
miR1846d-3p	LOC_Os02g38980.4	151	-24.74	151	-24.74	874595	20	3287	2981
miR1846d-3p	LOC_Os02g38980.5	151	-24.74	151	-24.74	874596	20	2217	1909
miR1846d-3p	LOC_Os02g38980.6	151	-24.74	151	-24.74	874597	20	1978	1670
miR1846d-3p	LOC_Os02g39110.1	295	-41.92	148	-21.62	874615	20	6084	507 2546
miR1846d-3p	LOC_Os02g39120.1	172	-35.42	172	-35.42	874616	20	3168	1831
miR1846d-3p	LOC_Os02g39140.1	146	-21.67	146	-21.67	874618	20	1555	460
miR1846d-3p	LOC_Os02g39140.2	146	-21.67	146	-21.67	874619	20	1662	460
miR1846d-3p	LOC_Os02g39200.1	159	-27.8	159	-27.8	874626	20	2113	125
miR1846d-3p	LOC_Os02g39220.1	318	-47.17	159	-25.14	874628	20	4626	1773 3039
miR1846d-3p	LOC_Os02g39470.1	157	-27.52	157	-27.52	874658	20	2916	2792
miR1846d-3p	LOC_Os02g39490.1	143	-21.97	143	-21.97	874660	20	1124	805
miR1846d-3p	LOC_Os02g39880.1	160	-29.83	160	-29.83	874718	20	708	180
miR1846d-3p	LOC_Os02g40100.1	152	-24.08	152	-24.08	874755	20	3162	252
miR1846d-3p	LOC_Os02g40360.1	166	-27.37	166	-27.37	874784	20	3195	659
miR1846d-3p	LOC_Os02g40680.1	153	-25.56	153	-25.56	874820	20	1108	1079
miR1846d-3p	LOC_Os02g41690.1	151	-24.69	151	-24.69	874882	20	1779	1662
miR1846d-3p	LOC_Os02g41910.1	147	-21.55	147	-21.55	874910	20	1517	42
miR1846d-3p	LOC_Os02g42000.1	153	-20.46	153	-20.46	874921	20	974	154
miR1846d-3p	LOC_Os02g42180.1	623	-107.82	162	-28.31	874940	20	4578	1641 3960 2748 4453
miR1846d-3p	LOC_Os02g42470.1	152	-28.02	152	-28.02	874986	20	246	120
miR1846d-3p	LOC_Os02g42660.1	147	-21.29	147	-21.29	875009	20	1564	1240
miR1846d-3p	LOC_Os02g42850.1	151	-22.91	151	-22.91	875028	20	1800	1118
miR1846d-3p	LOC_Os02g42850.2	151	-22.91	151	-22.91	875029	20	1458	776
miR1846d-3p	LOC_Os02g42870.1	159	-23.49	159	-23.49	875031	20	1432	491
miR1846d-3p	LOC_Os02g42970.1	144	-23.08	144	-23.08	875042	20	1113	92
miR1846d-3p	LOC_Os02g43040.1	141	-20.27	141	-20.27	875050	20	2441	1596
miR1846d-3p	LOC_Os02g43280.1	151	-21.26	151	-21.26	875080	20	2054	1314
miR1846d-3p	LOC_Os02g43280.2	151	-21.26	151	-21.26	875081	20	2160	1420
miR1846d-3p	LOC_Os02g43330.1	164	-32.1	164	-32.1	875087	20	1336	823

miR1846d-3p	LOC_Os02g43620.1	148	-26.01	148	-26.01	875122	20	2060	491
miR1846d-3p	LOC_Os02g43660.1	152	-25.07	152	-25.07	875125	20	876	688
miR1846d-3p	LOC_Os02g43710.1	166	-25.4	166	-25.4	875130	20	1053	142
miR1846d-3p	LOC_Os02g43720.1	150	-22.01	150	-22.01	875131	20	1158	837
miR1846d-3p	LOC_Os02g43720.2	150	-22.01	150	-22.01	875132	20	1015	694
miR1846d-3p	LOC_Os02g43790.1	321	-60.22	164	-32.37	875139	20	1304	1217 1181
miR1846d-3p	LOC_Os02g43830.1	149	-22.83	149	-22.83	875143	20	1259	538
miR1846d-3p	LOC_Os02g43830.2	149	-22.83	149	-22.83	875144	20	1313	593
miR1846d-3p	LOC_Os02g43950.1	162	-29.06	162	-29.06	875165	20	240	26
miR1846d-3p	LOC_Os02g44030.1	148	-26.53	148	-26.53	875174	20	687	130
miR1846d-3p	LOC_Os02g44440.1	144	-21.47	144	-21.47	875237	20	2409	870
miR1846d-3p	LOC_Os02g44599.1	148	-23.56	148	-23.56	875260	20	3763	2831
miR1846d-3p	LOC_Os02g44599.2	148	-23.56	148	-23.56	875261	20	3917	2985
miR1846d-3p	LOC_Os02g44599.3	148	-23.56	148	-23.56	875262	20	3383	2831
miR1846d-3p	LOC_Os02g44599.4	148	-23.56	148	-23.56	875263	20	3681	2748
miR1846d-3p	LOC_Os02g45110.1	150	-20.7	150	-20.7	875328	20	2549	842
miR1846d-3p	LOC_Os02g45160.1	149	-21.34	149	-21.34	875337	20	3084	2332
miR1846d-3p	LOC_Os02g45180.1	153	-22.15	153	-22.15	875339	20	1113	313
miR1846d-3p	LOC_Os02g45490.1	302	-52.3	157	-28.89	875373	20	3428	2984 3202
miR1846d-3p	LOC_Os02g45490.2	302	-52.3	157	-28.89	875374	20	3145	2705 2923
miR1846d-3p	LOC_Os02g45490.3	302	-52.3	157	-28.89	875375	20	2916	2476 2694
miR1846d-3p	LOC_Os02g45490.4	302	-52.3	157	-28.89	875376	20	3346	2906 3124
miR1846d-3p	LOC_Os02g45880.1	157	-23.5	157	-23.5	875419	20	708	660
miR1846d-3p	LOC_Os02g46040.1	309	-53.08	162	-31.46	875435	20	5955	507 2417
miR1846d-3p	LOC_Os02g46180.1	295	-42.54	149	-21.84	875451	20	1051	945 789
miR1846d-3p	LOC_Os02g46270.1	159	-23.33	159	-23.33	875465	20	525	193
miR1846d-3p	LOC_Os02g46820.1	150	-25.72	150	-25.72	875531	20	297	218
miR1846d-3p	LOC_Os02g47040.1	292	-44.33	148	-23.02	875560	20	567	302 479
miR1846d-3p	LOC_Os02g47220.2	157	-27.69	157	-27.69	875582	20	3030	478
miR1846d-3p	LOC_Os02g47380.1	156	-23.89	156	-23.89	875601	20	2868	2265
miR1846d-3p	LOC_Os02g47410.1	157	-24.69	157	-24.69	875605	20	3747	3539
miR1846d-3p	LOC_Os02g47410.2	157	-24.69	157	-24.69	875606	20	3744	3536

miR1846d-3p	LOC_Os02g47410.3	157	-24.69	157	-24.69	875607	20	3741	3533
miR1846d-3p	LOC_Os02g47420.1	152	-26.65	152	-26.65	875608	20	2390	415
miR1846d-3p	LOC_Os02g47420.2	152	-26.65	152	-26.65	875609	20	2485	415
miR1846d-3p	LOC_Os02g47420.3	152	-26.65	152	-26.65	875610	20	2070	276
miR1846d-3p	LOC_Os02g47420.4	152	-26.65	152	-26.65	875611	20	2520	415
miR1846d-3p	LOC_Os02g48150.1	142	-20.38	142	-20.38	875705	20	1159	269
miR1846d-3p	LOC_Os02g48160.1	478	-85.16	169	-33.14	875706	20	417	295 253 273
miR1846d-3p	LOC_Os02g48184.1	153	-26.87	153	-26.87	875708	20	1304	778
miR1846d-3p	LOC_Os02g48340.1	156	-29.25	156	-29.25	875726	20	2237	189
miR1846d-3p	LOC_Os02g48340.2	156	-29.25	156	-29.25	875727	20	2213	189
miR1846d-3p	LOC_Os02g48340.3	156	-29.25	156	-29.25	875728	20	2210	189
miR1846d-3p	LOC_Os02g48340.5	156	-29.25	156	-29.25	875729	20	1719	189
miR1846d-3p	LOC_Os02g48340.6	156	-29.25	156	-29.25	875730	20	2304	189
miR1846d-3p	LOC_Os02g48410.1	166	-26.59	166	-26.59	875740	20	2796	659
miR1846d-3p	LOC_Os02g48650.1	141	-22.18	141	-22.18	875772	20	1143	384
miR1846d-3p	LOC_Os02g49332.2	164	-31.61	164	-31.61	875856	20	4273	2020
miR1846d-3p	LOC_Os02g49332.3	164	-31.61	164	-31.61	875857	20	4042	2012
miR1846d-3p	LOC_Os02g49420.1	165	-28.84	165	-28.84	875868	20	1256	452
miR1846d-3p	LOC_Os02g49440.1	171	-36.05	171	-36.05	875870	20	1343	1054
miR1846d-3p	LOC_Os02g49880.1	294	-44.89	152	-23.51	875931	20	2892	2209 2014
miR1846d-3p	LOC_Os02g49940.1	153	-27.08	153	-27.08	875937	20	738	526
miR1846d-3p	LOC_Os02g49960.1	158	-25.61	158	-25.61	875939	20	1186	708
miR1846d-3p	LOC_Os02g50280.1	146	-20.57	146	-20.57	875973	20	2626	504
miR1846d-3p	LOC_Os02g50360.1	147	-24.86	147	-24.86	875983	20	2689	2662
miR1846d-3p	LOC_Os02g50540.1	149	-22.97	149	-22.97	876004	20	1735	291
miR1846d-3p	LOC_Os02g50600.1	156	-27.72	156	-27.72	876011	20	1767	1566
miR1846d-3p	LOC_Os02g50730.1	145	-24.35	145	-24.35	876027	20	2095	334
miR1846d-3p	LOC_Os02g50770.1	148	-20.76	148	-20.76	876033	20	2019	657
miR1846d-3p	LOC_Os02g50940.1	145	-23.38	145	-23.38	876053	20	938	647
miR1846d-3p	LOC_Os02g51119.1	150	-26.18	150	-26.18	876074	20	1732	1527
miR1846d-3p	LOC_Os02g51119.2	150	-26.18	150	-26.18	876075	20	2694	2489
miR1846d-3p	LOC_Os02g51290.1	148	-24.6	148	-24.6	876095	20	1122	1063

miR1846d-3p	LOC_Os02g51440.1	146	-20.38	146	-20.38	876118	20	2076	1615
miR1846d-3p	LOC_Os02g51520.1	147	-21.62	147	-21.62	876131	20	5772	2513
miR1846d-3p	LOC_Os02g51530.1	172	-34.12	172	-34.12	876132	20	3135	1828
miR1846d-3p	LOC_Os02g51700.1	164	-34	164	-34	876149	20	1016	122
miR1846d-3p	LOC_Os02g51880.1	145	-24.34	145	-24.34	876176	20	3081	2974
miR1846d-3p	LOC_Os02g51890.1	151	-26.67	151	-26.67	876177	20	2398	2302
miR1846d-3p	LOC_Os02g51890.2	151	-26.67	151	-26.67	876178	20	4440	4344
miR1846d-3p	LOC_Os02g51900.1	300	-49.25	151	-26.67	876179	20	2142	996 1526
miR1846d-3p	LOC_Os02g51980.1	146	-20.01	146	-20.01	876186	20	3459	17
miR1846d-3p	LOC_Os02g52070.1	166	-26.59	166	-26.59	876195	20	2370	659
miR1846d-3p	LOC_Os02g52100.1	305	-50.53	158	-28.91	876198	20	5964	489 2528
miR1846d-3p	LOC_Os02g52210.1	145	-20.25	145	-20.25	876219	20	1558	1158
miR1846d-3p	LOC_Os02g52210.2	145	-20.25	145	-20.25	876220	20	2241	1158
miR1846d-3p	LOC_Os02g52230.1	148	-24.15	148	-24.15	876222	20	2790	370
miR1846d-3p	LOC_Os02g52380.1	152	-21	152	-21	876239	20	1623	984
miR1846d-3p	LOC_Os02g52450.1	147	-20.06	147	-20.06	876248	20	2015	1958
miR1846d-3p	LOC_Os02g52450.3	147	-20.06	147	-20.06	876250	20	972	768
miR1846d-3p	LOC_Os02g52520.1	305	-47.51	158	-26.72	876259	20	5820	507 2546
miR1846d-3p	LOC_Os02g52610.1	151	-24.67	151	-24.67	876266	20	1893	142
miR1846d-3p	LOC_Os02g52840.1	165	-34.12	165	-34.12	876292	20	1221	675
miR1846d-3p	LOC_Os02g52850.1	144	-21.61	144	-21.61	876293	20	3127	2553
miR1846d-3p	LOC_Os02g53030.1	158	-23.58	158	-23.58	876318	20	983	483
miR1846d-3p	LOC_Os02g53150.1	160	-33.26	160	-33.26	876333	20	1524	1080
miR1846d-3p	LOC_Os02g53360.1	151	-25.65	151	-25.65	876367	20	861	378
miR1846d-3p	LOC_Os02g53380.1	153	-22.95	153	-22.95	876369	20	2849	2119
miR1846d-3p	LOC_Os02g53530.1	150	-25.82	150	-25.82	876385	20	1300	988
miR1846d-3p	LOC_Os02g53540.1	162	-24.8	162	-24.8	876386	20	3198	659
miR1846d-3p	LOC_Os02g53680.1	155	-24.26	155	-24.26	876402	20	2591	2128
miR1846d-3p	LOC_Os02g53820.1	148	-20.38	148	-20.38	876426	20	2292	1164
miR1846d-3p	LOC_Os02g53820.2	148	-20.38	148	-20.38	876427	20	2286	1158
miR1846d-3p	LOC_Os02g53835.1	146	-25.62	146	-25.62	876429	20	1304	676
miR1846d-3p	LOC_Os02g54050.1	148	-23.91	148	-23.91	876451	20	669	518

miR1846d-3p	LOC_Os02g54100.1	153	-24.06	153	-24.06	876459	20	2516	1078
miR1846d-3p	LOC_Os02g54270.1	152	-23.22	152	-23.22	876484	20	1938	832
miR1846d-3p	LOC_Os02g54330.1	156	-31.92	156	-31.92	876490	20	1242	456
miR1846d-3p	LOC_Os02g54510.1	145	-21.15	145	-21.15	876509	20	4517	280
miR1846d-3p	LOC_Os02g54600.1	157	-21.39	157	-21.39	876520	20	1880	618
miR1846d-3p	LOC_Os02g54790.1	148	-25.74	148	-25.74	876538	20	2346	113
miR1846d-3p	LOC_Os02g54820.1	145	-23.28	145	-23.28	876541	20	3664	2810
miR1846d-3p	LOC_Os02g54860.1	164	-27.42	164	-27.42	876547	20	2330	20
miR1846d-3p	LOC_Os02g54980.1	152	-29.02	152	-29.02	876560	20	2139	1164
miR1846d-3p	LOC_Os02g55440.1	140	-20.69	140	-20.69	876618	20	2613	1747
miR1846d-3p	LOC_Os02g55440.2	140	-20.69	140	-20.69	876619	20	2508	1642
miR1846d-3p	LOC_Os02g55840.1	160	-25.98	160	-25.98	876667	20	1501	769
miR1846d-3p	LOC_Os02g55840.2	160	-25.98	160	-25.98	876668	20	1481	769
miR1846d-3p	LOC_Os02g55840.3	160	-25.98	160	-25.98	876669	20	1534	769
miR1846d-3p	LOC_Os02g55840.4	160	-25.98	160	-25.98	876670	20	1554	769
miR1846d-3p	LOC_Os02g55840.5	160	-25.98	160	-25.98	876671	20	1534	769
miR1846d-3p	LOC_Os02g55840.6	160	-25.98	160	-25.98	876672	20	1650	767
miR1846d-3p	LOC_Os02g55940.1	157	-27.47	157	-27.47	876683	20	1870	299
miR1846d-3p	LOC_Os02g56060.1	305	-47.07	158	-25.45	876699	20	5562	507 2486
miR1846d-3p	LOC_Os02g56110.1	159	-25.8	159	-25.8	876704	20	2971	2764
miR1846d-3p	LOC_Os02g56110.2	159	-25.8	159	-25.8	876705	20	3211	3004
miR1846d-3p	LOC_Os02g56640.1	147	-21.62	147	-21.62	876768	20	3366	2546
miR1846d-3p	LOC_Os02g56660.1	172	-34.12	172	-34.12	876770	20	3186	1921
miR1846d-3p	LOC_Os02g56690.1	152	-26.75	152	-26.75	876773	20	1065	267
miR1846d-3p	LOC_Os02g56840.1	145	-21.97	145	-21.97	876789	20	1360	66
miR1846d-3p	LOC_Os02g56850.1	161	-25.41	161	-25.41	876790	20	2060	2017
miR1846d-3p	LOC_Os02g56850.2	161	-25.41	161	-25.41	876791	20	2176	2133
miR1846d-3p	LOC_Os02g56850.3	161	-25.41	161	-25.41	876792	20	2160	2117
miR1846d-3p	LOC_Os02g56850.4	161	-25.41	161	-25.41	876793	20	1628	1585
miR1846d-3p	LOC_Os02g57340.1	140	-21.25	140	-21.25	876865	20	3061	645
miR1846d-3p	LOC_Os02g57710.1	145	-22.02	145	-22.02	876930	20	2186	1149
miR1846d-3p	LOC_Os02g57770.1	152	-26.89	152	-26.89	876937	20	1227	548

miR1846d-3p	LOC_Os02g57870.1	152	-21.52	152	-21.52	876952	20	1751	1544
miR1846d-3p	LOC_Os02g57870.2	152	-21.52	152	-21.52	876953	20	1873	1687
miR1846d-3p	LOC_Os02g58350.1	158	-31.91	158	-31.91	877012	20	746	387
miR1846d-3p	LOC_Os02g58380.1	154	-25.08	154	-25.08	877015	20	552	390
miR1846d-3p	LOC_Os03g01040.1	297	-42.37	150	-21.62	877081	20	3114	507 2456
miR1846d-3p	LOC_Os03g01070.1	166	-25.39	166	-25.39	877084	20	2748	425
miR1846d-3p	LOC_Os03g01380.1	158	-25.45	158	-25.45	877130	20	2331	507
miR1846d-3p	LOC_Os03g01470.1	156	-24.05	156	-24.05	877139	20	1530	201
miR1846d-3p	LOC_Os03g01650.1	159	-21.3	159	-21.3	877160	20	1230	347
miR1846d-3p	LOC_Os03g01720.1	148	-21.15	148	-21.15	877166	20	2874	18
miR1846d-3p	LOC_Os03g01960.1	140	-22.39	140	-22.39	877208	20	1041	517
miR1846d-3p	LOC_Os03g02310.1	152	-26.04	152	-26.04	877259	20	893	784
miR1846d-3p	LOC_Os03g02350.1	147	-21.62	147	-21.62	877264	20	6084	2546
miR1846d-3p	LOC_Os03g02360.1	172	-34.12	172	-34.12	877265	20	3165	1828
miR1846d-3p	LOC_Os03g02390.1	157	-25.04	157	-25.04	877269	20	1295	1166
miR1846d-3p	LOC_Os03g02560.1	141	-22.29	141	-22.29	877293	20	1631	1147
miR1846d-3p	LOC_Os03g02762.1	301	-45.37	151	-23.61	877322	20	3252	2926 2976
miR1846d-3p	LOC_Os03g02770.1	301	-45.37	151	-23.61	877323	20	1914	1588 1638
miR1846d-3p	LOC_Os03g02770.2	301	-45.37	151	-23.61	877324	20	1999	1673 1723
miR1846d-3p	LOC_Os03g02874.1	145	-23.12	145	-23.12	877338	20	3500	3301
miR1846d-3p	LOC_Os03g02874.2	145	-23.12	145	-23.12	877339	20	984	785
miR1846d-3p	LOC_Os03g02874.3	145	-23.12	145	-23.12	877340	20	1471	1272
miR1846d-3p	LOC_Os03g02874.4	145	-23.12	145	-23.12	877341	20	1113	914
miR1846d-3p	LOC_Os03g02874.5	145	-23.12	145	-23.12	877342	20	1379	1180
miR1846d-3p	LOC_Os03g02874.6	145	-23.12	145	-23.12	877343	20	1101	902
miR1846d-3p	LOC_Os03g03034.1	149	-20.34	149	-20.34	877356	20	1499	1467
miR1846d-3p	LOC_Os03g03034.2	149	-20.34	149	-20.34	877357	20	1284	1252
miR1846d-3p	LOC_Os03g03150.1	169	-28.26	169	-28.26	877371	20	2269	289
miR1846d-3p	LOC_Os03g03150.2	169	-28.26	169	-28.26	877372	20	2047	289
miR1846d-3p	LOC_Os03g03210.1	143	-21.21	143	-21.21	877380	20	3162	44
miR1846d-3p	LOC_Os03g03230.1	164	-28.95	164	-28.95	877382	20	1722	636
miR1846d-3p	LOC_Os03g03250.1	145	-20.08	145	-20.08	877384	20	5862	4356

miR1846d-3p	LOC_Os03g03260.1	148	-23.81	148	-23.81	877385	20	2580	2121
miR1846d-3p	LOC_Os03g03290.1	312	-48.82	160	-25.22	877389	20	1833	432 801
miR1846d-3p	LOC_Os03g03540.1	148	-24.93	148	-24.93	877422	20	1399	907
miR1846d-3p	LOC_Os03g03654.1	165	-33.65	165	-33.65	877435	20	776	434
miR1846d-3p	LOC_Os03g03910.1	157	-23.23	157	-23.23	877471	20	2126	434
miR1846d-3p	LOC_Os03g04000.1	161	-27.9	161	-27.9	877482	20	2198	289
miR1846d-3p	LOC_Os03g04195.1	148	-21.85	148	-21.85	877503	20	3507	2826
miR1846d-3p	LOC_Os03g04520.2	154	-25.14	154	-25.14	877540	20	3629	784
miR1846d-3p	LOC_Os03g04520.4	154	-25.14	154	-25.14	877542	20	3573	784
miR1846d-3p	LOC_Os03g04545.1	165	-32.97	165	-32.97	877545	20	1199	49
miR1846d-3p	LOC_Os03g05140.1	150	-25.5	150	-25.5	877616	20	3093	1540
miR1846d-3p	LOC_Os03g05210.1	168	-31.59	168	-31.59	877623	20	1554	1350
miR1846d-3p	LOC_Os03g05350.1	300	-49.66	153	-25.5	877644	20	4386	1304 1260
miR1846d-3p	LOC_Os03g05360.1	157	-24.58	157	-24.58	877645	20	7362	1694
miR1846d-3p	LOC_Os03g05450.1	150	-25.75	150	-25.75	877668	20	642	410
miR1846d-3p	LOC_Os03g05460.1	160	-27.44	160	-27.44	877669	20	1638	1284
miR1846d-3p	LOC_Os03g05790.1	144	-23.56	144	-23.56	877703	20	3390	3132
miR1846d-3p	LOC_Os03g05850.1	151	-25.79	151	-25.79	877714	20	4230	4183
miR1846d-3p	LOC_Os03g06010.1	149	-23.57	149	-23.57	877735	20	818	747
miR1846d-3p	LOC_Os03g06260.1	147	-21.62	147	-21.62	877762	20	5190	2348
miR1846d-3p	LOC_Os03g06550.1	166	-27.37	166	-27.37	877795	20	3198	659
miR1846d-3p	LOC_Os03g06630.1	148	-27.7	148	-27.7	877805	20	1409	20
miR1846d-3p	LOC_Os03g06630.2	148	-27.7	148	-27.7	877806	20	1349	20
miR1846d-3p	LOC_Os03g06710.1	158	-23.12	158	-23.12	877816	20	3133	708
miR1846d-3p	LOC_Os03g06730.1	142	-20.66	142	-20.66	877818	20	1983	1393
miR1846d-3p	LOC_Os03g07020.1	166	-26.59	166	-26.59	877853	20	3306	659
miR1846d-3p	LOC_Os03g07130.1	150	-25.48	150	-25.48	877868	20	2388	88
miR1846d-3p	LOC_Os03g07130.3	150	-25.48	150	-25.48	877870	20	2292	88
miR1846d-3p	LOC_Os03g07170.1	160	-24.68	160	-24.68	877877	20	3033	109
miR1846d-3p	LOC_Os03g07200.1	163	-31.36	163	-31.36	877882	20	1589	935
miR1846d-3p	LOC_Os03g07200.2	163	-31.36	163	-31.36	877883	20	1491	935
miR1846d-3p	LOC_Os03g07200.3	163	-31.36	163	-31.36	877884	20	1481	935

miR1846d-3p	LOC_Os03g07770.1	147	-21.62	147	-21.62	877952	20	5988	2501
miR1846d-3p	LOC_Os03g07780.1	316	-59.75	172	-35.42	877953	20	3126	1789 1811
miR1846d-3p	LOC_Os03g08040.1	145	-20.79	145	-20.79	877980	20	669	274
miR1846d-3p	LOC_Os03g08130.1	157	-25.89	157	-25.89	877994	20	1188	1109
miR1846d-3p	LOC_Os03g08270.2	143	-21.52	143	-21.52	878010	20	2177	66
miR1846d-3p	LOC_Os03g08280.1	157	-24.12	157	-24.12	878014	20	1209	1088
miR1846d-3p	LOC_Os03g08280.2	157	-24.12	157	-24.12	878015	20	1107	968
miR1846d-3p	LOC_Os03g08280.3	157	-24.12	157	-24.12	878016	20	1194	1055
miR1846d-3p	LOC_Os03g08284.1	154	-24.99	154	-24.99	878017	20	2070	346
miR1846d-3p	LOC_Os03g08284.2	154	-24.99	154	-24.99	878018	20	2010	346
miR1846d-3p	LOC_Os03g08284.3	154	-24.99	154	-24.99	878019	20	1509	346
miR1846d-3p	LOC_Os03g08410.1	148	-23.23	148	-23.23	878035	20	1620	388
miR1846d-3p	LOC_Os03g08470.1	152	-27.54	152	-27.54	878043	20	1651	1188
miR1846d-3p	LOC_Os03g08470.2	152	-27.54	152	-27.54	878044	20	1763	1300
miR1846d-3p	LOC_Os03g08470.3	152	-27.54	152	-27.54	878045	20	1676	1213
miR1846d-3p	LOC_Os03g08540.1	145	-23.42	145	-23.42	878054	20	1219	326
miR1846d-3p	LOC_Os03g08660.1	156	-26.06	156	-26.06	878072	20	906	34
miR1846d-3p	LOC_Os03g09190.1	140	-23.16	140	-23.16	878128	20	2253	857
miR1846d-3p	LOC_Os03g09190.2	140	-23.16	140	-23.16	878129	20	2244	848
miR1846d-3p	LOC_Os03g09220.1	157	-26.46	157	-26.46	878132	20	2306	275
miR1846d-3p	LOC_Os03g09220.2	157	-26.46	157	-26.46	878133	20	2215	275
miR1846d-3p	LOC_Os03g09220.3	157	-26.46	157	-26.46	878134	20	2209	275
miR1846d-3p	LOC_Os03g09220.4	157	-26.46	157	-26.46	878135	20	1807	275
miR1846d-3p	LOC_Os03g09220.5	157	-26.46	157	-26.46	878136	20	2211	275
miR1846d-3p	LOC_Os03g09220.6	157	-26.46	157	-26.46	878137	20	1801	275
miR1846d-3p	LOC_Os03g09940.1	151	-23.43	151	-23.43	878171	20	2677	784
miR1846d-3p	LOC_Os03g09960.1	149	-27.34	149	-27.34	878173	20	624	511
miR1846d-3p	LOC_Os03g10220.1	157	-22.08	157	-22.08	878206	20	2214	2131
miR1846d-3p	LOC_Os03g10224.1	157	-22.08	157	-22.08	878207	20	1833	1723
miR1846d-3p	LOC_Os03g10230.1	157	-22.08	157	-22.08	878208	20	2116	66
miR1846d-3p	LOC_Os03g10250.1	145	-22.39	145	-22.39	878211	20	1640	727
miR1846d-3p	LOC_Os03g10360.1	153	-24.83	153	-24.83	878225	20	1492	1081

miR1846d-3p	LOC_Os03g10420.1	157	-26.59	157	-26.59	878231	20	2391	1026
miR1846d-3p	LOC_Os03g10700.1	157	-22.54	157	-22.54	878262	20	1668	1373
miR1846d-3p	LOC_Os03g10820.1	148	-25.74	148	-25.74	878278	20	1825	1426
miR1846d-3p	LOC_Os03g10870.1	156	-23.03	156	-23.03	878284	20	947	169
miR1846d-3p	LOC_Os03g11220.1	158	-25.25	158	-25.25	878323	20	1541	1139
miR1846d-3p	LOC_Os03g11320.1	147	-27.52	147	-27.52	878335	20	2527	381
miR1846d-3p	LOC_Os03g11320.2	147	-27.52	147	-27.52	878336	20	2520	381
miR1846d-3p	LOC_Os03g11630.1	145	-20.47	145	-20.47	878379	20	2547	91
miR1846d-3p	LOC_Os03g12050.1	287	-41.97	146	-21.9	878431	20	2060	1725 1379
miR1846d-3p	LOC_Os03g12600.1	145	-23.22	145	-23.22	878507	20	6036	4128
miR1846d-3p	LOC_Os03g12640.1	152	-26.43	152	-26.43	878513	20	291	238
miR1846d-3p	LOC_Os03g12730.1	157	-28.92	157	-28.92	878526	20	3871	1150
miR1846d-3p	LOC_Os03g12820.1	156	-24.09	156	-24.09	878534	20	1903	696
miR1846d-3p	LOC_Os03g12820.2	156	-24.09	156	-24.09	878535	20	1557	696
miR1846d-3p	LOC_Os03g12879.1	331	-57.2	168	-30.19	878543	20	1193	576 495
miR1846d-3p	LOC_Os03g13080.1	149	-22.47	149	-22.47	878578	20	1875	1527
miR1846d-3p	LOC_Os03g13230.1	302	-46.83	156	-26.41	878597	20	2250	1331 867
miR1846d-3p	LOC_Os03g13325.1	159	-26.7	159	-26.7	878610	20	657	419
miR1846d-3p	LOC_Os03g13520.1	147	-22.64	147	-22.64	878628	20	1828	1659
miR1846d-3p	LOC_Os03g13730.1	168	-34.74	168	-34.74	878650	20	1427	240
miR1846d-3p	LOC_Os03g13810.1	153	-22.05	153	-22.05	878662	20	2361	102
miR1846d-3p	LOC_Os03g13810.2	153	-22.05	153	-22.05	878663	20	2353	100
miR1846d-3p	LOC_Os03g13810.3	153	-22.05	153	-22.05	878664	20	3344	102
miR1846d-3p	LOC_Os03g13900.1	305	-45.49	158	-23.88	878676	20	5745	507 2555
miR1846d-3p	LOC_Os03g14060.1	146	-20.01	146	-20.01	878706	20	3855	17
miR1846d-3p	LOC_Os03g14170.1	321	-57.09	165	-30.59	878720	20	2139	1891 1832
miR1846d-3p	LOC_Os03g14250.1	152	-22.48	152	-22.48	878727	20	4183	553
miR1846d-3p	LOC_Os03g14710.1	161	-28.98	161	-28.98	878780	20	2043	566
miR1846d-3p	LOC_Os03g14800.1	147	-23.82	147	-23.82	878788	20	2247	2226
miR1846d-3p	LOC_Os03g14800.3	147	-23.82	147	-23.82	878789	20	2077	2056
miR1846d-3p	LOC_Os03g14800.4	147	-23.82	147	-23.82	878790	20	2363	2322
miR1846d-3p	LOC_Os03g14800.5	147	-23.82	147	-23.82	878791	20	1543	1502

miR1846d-3p	LOC_Os03g14800.6	147	-23.82	147	-23.82	878792	20	1587	1546
miR1846d-3p	LOC_Os03g14930.1	158	-28.91	158	-28.91	878805	20	1398	507
miR1846d-3p	LOC_Os03g15050.1	317	-55.59	161	-28.73	878820	20	2670	2258 1760
miR1846d-3p	LOC_Os03g15050.2	317	-55.59	161	-28.73	878821	20	2483	2071 1573
miR1846d-3p	LOC_Os03g15050.4	156	-28.73	156	-28.73	878822	20	2001	1573
miR1846d-3p	LOC_Os03g15100.1	298	-42.57	150	-22.12	878828	20	849	168 330
miR1846d-3p	LOC_Os03g15380.1	160	-27.45	160	-27.45	878857	20	999	94
miR1846d-3p	LOC_Os03g15400.1	165	-31.59	165	-31.59	878859	20	822	164
miR1846d-3p	LOC_Os03g15410.1	152	-33.31	152	-33.31	878860	20	3628	821
miR1846d-3p	LOC_Os03g15440.1	143	-22.85	143	-22.85	878864	20	1655	659
miR1846d-3p	LOC_Os03g15590.1	166	-33.29	166	-33.29	878879	20	684	330
miR1846d-3p	LOC_Os03g15740.1	154	-30.07	154	-30.07	878899	20	3227	159
miR1846d-3p	LOC_Os03g15920.1	151	-28.69	151	-28.69	878927	20	1476	883
miR1846d-3p	LOC_Os03g15920.2	151	-28.69	151	-28.69	878928	20	1561	883
miR1846d-3p	LOC_Os03g15920.3	151	-28.69	151	-28.69	878929	20	1667	883
miR1846d-3p	LOC_Os03g15910.1	151	-28.69	151	-28.69	878930	20	1767	883
miR1846d-3p	LOC_Os03g16120.1	151	-23.15	151	-23.15	878959	20	1819	1524
miR1846d-3p	LOC_Os03g16120.2	151	-23.15	151	-23.15	878960	20	1810	1515
miR1846d-3p	LOC_Os03g16120.3	151	-23.15	151	-23.15	878961	20	1857	1562
miR1846d-3p	LOC_Os03g16120.4	151	-23.15	151	-23.15	878962	20	1898	1603
miR1846d-3p	LOC_Os03g16120.5	151	-23.15	151	-23.15	878963	20	1939	1644
miR1846d-3p	LOC_Os03g16160.1	156	-23.03	156	-23.03	878977	20	330	224
miR1846d-3p	LOC_Os03g16170.1	169	-29.64	169	-29.64	878978	20	1719	1469
miR1846d-3p	LOC_Os03g16170.2	169	-29.64	169	-29.64	878979	20	1872	1622
miR1846d-3p	LOC_Os03g16280.1	158	-28.91	158	-28.91	878996	20	1317	196
miR1846d-3p	LOC_Os03g16390.1	147	-20.55	147	-20.55	879005	20	1937	860
miR1846d-3p	LOC_Os03g16718.1	152	-29.19	152	-29.19	879043	20	1617	1354
miR1846d-3p	LOC_Os03g16824.1	152	-27.52	152	-27.52	879057	20	2893	422
miR1846d-3p	LOC_Os03g16890.1	314	-54.15	169	-32.66	879068	20	1572	1193 1165
miR1846d-3p	LOC_Os03g17020.1	156	-22.77	156	-22.77	879089	20	1976	1864
miR1846d-3p	LOC_Os03g17020.2	156	-22.77	156	-22.77	879090	20	2171	2059
miR1846d-3p	LOC_Os03g17432.1	150	-24.89	150	-24.89	879134	20	1215	912

miR1846d-3p	LOC_Os03g17510.1	144	-22.76	144	-22.76	879143	20	2560	2413
miR1846d-3p	LOC_Os03g17610.1	148	-22.77	148	-22.77	879159	20	2564	881
miR1846d-3p	LOC_Os03g17634.1	148	-22.77	148	-22.77	879160	20	4852	881
miR1846d-3p	LOC_Os03g17860.1	166	-31.17	166	-31.17	879184	20	772	619
miR1846d-3p	LOC_Os03g17860.2	166	-31.17	166	-31.17	879185	20	665	512
miR1846d-3p	LOC_Os03g18030.1	151	-23.93	151	-23.93	879208	20	2180	748
miR1846d-3p	LOC_Os03g18030.2	151	-23.93	151	-23.93	879209	20	2226	748
miR1846d-3p	LOC_Os03g18270.1	314	-51.46	167	-27.56	879239	20	1566	1189 1344
miR1846d-3p	LOC_Os03g18310.1	154	-22.94	154	-22.94	879242	20	3048	1399
miR1846d-3p	LOC_Os03g18310.2	154	-22.94	154	-22.94	879243	20	3002	1339
miR1846d-3p	LOC_Os03g18310.3	154	-22.94	154	-22.94	879244	20	1989	1339
miR1846d-3p	LOC_Os03g18490.1	155	-25.41	155	-25.41	879264	20	983	879
miR1846d-3p	LOC_Os03g18510.1	153	-27.63	153	-27.63	879267	20	1537	208
miR1846d-3p	LOC_Os03g18690.1	145	-20.75	145	-20.75	879287	20	1739	1298
miR1846d-3p	LOC_Os03g18870.1	147	-22.7	147	-22.7	879308	20	1157	925
miR1846d-3p	LOC_Os03g19120.1	159	-26.91	159	-26.91	879335	20	1077	99
miR1846d-3p	LOC_Os03g19436.1	500	-92.65	168	-32.42	879372	20	747	479 539 509
miR1846d-3p	LOC_Os03g19490.1	156	-25.7	156	-25.7	879378	20	2626	537
miR1846d-3p	LOC_Os03g19520.1	153	-24.22	153	-24.22	879381	20	3852	2256
miR1846d-3p	LOC_Os03g19520.2	153	-24.22	153	-24.22	879382	20	3855	2259
miR1846d-3p	LOC_Os03g19520.3	153	-24.22	153	-24.22	879383	20	2866	1270
miR1846d-3p	LOC_Os03g19670.1	148	-22.11	148	-22.11	879405	20	1711	1394
miR1846d-3p	LOC_Os03g19670.2	148	-22.11	148	-22.11	879406	20	1801	1565
miR1846d-3p	LOC_Os03g19680.1	294	-43.74	149	-22.27	879407	20	1472	474 457
miR1846d-3p	LOC_Os03g19770.1	149	-23.19	149	-23.19	879417	20	621	566
miR1846d-3p	LOC_Os03g20140.1	141	-20.32	141	-20.32	879458	20	2244	1708
miR1846d-3p	LOC_Os03g20210.1	159	-24.7	159	-24.7	879465	20	1507	223
miR1846d-3p	LOC_Os03g20340.1	159	-24.51	159	-24.51	879483	20	1496	325
miR1846d-3p	LOC_Os03g20410.1	147	-22.44	147	-22.44	879496	20	1179	963
miR1846d-3p	LOC_Os03g20420.1	145	-21.61	145	-21.61	879497	20	2661	2510
miR1846d-3p	LOC_Os03g20420.2	145	-21.61	145	-21.61	879498	20	2944	2793
miR1846d-3p	LOC_Os03g20420.3	145	-21.61	145	-21.61	879499	20	3158	3007

miR1846d-3p	LOC_Os03g20850.1	145	-24.74	145	-24.74	879548	20	1936	449
miR1846d-3p	LOC_Os03g20990.1	301	-47.07	159	-25.44	879564	20	615	411 246
miR1846d-3p	LOC_Os03g21060.1	144	-23.5	144	-23.5	879573	20	1846	1375
miR1846d-3p	LOC_Os03g21100.1	152	-25.91	152	-25.91	879577	20	2424	2064
miR1846d-3p	LOC_Os03g21180.1	147	-21.62	147	-21.62	879586	20	5781	2426
miR1846d-3p	LOC_Os03g21190.1	172	-34.12	172	-34.12	879587	20	3129	1792
miR1846d-3p	LOC_Os03g21210.1	143	-22	143	-22	879589	20	2325	1638
miR1846d-3p	LOC_Os03g21230.1	144	-21.55	144	-21.55	879591	20	3658	2125
miR1846d-3p	LOC_Os03g21260.1	165	-31.32	165	-31.32	879594	20	1677	87
miR1846d-3p	LOC_Os03g21570.1	159	-27.61	159	-27.61	879630	20	936	392
miR1846d-3p	LOC_Os03g22140.1	148	-21.15	148	-21.15	879707	20	1864	864
miR1846d-3p	LOC_Os03g22160.1	148	-22.23	148	-22.23	879709	20	3452	3128
miR1846d-3p	LOC_Os03g22450.1	153	-27.67	153	-27.67	879746	20	1663	1088
miR1846d-3p	LOC_Os03g22600.1	147	-23.17	147	-23.17	879766	20	2393	2020
miR1846d-3p	LOC_Os03g22650.1	311	-50.31	156	-27.14	879771	20	579	326 133
miR1846d-3p	LOC_Os03g22720.1	162	-24.29	162	-24.29	879778	20	1930	1625
miR1846d-3p	LOC_Os03g22720.2	162	-24.29	162	-24.29	879779	20	1624	1319
miR1846d-3p	LOC_Os03g22720.3	162	-24.29	162	-24.29	879780	20	1513	1208
miR1846d-3p	LOC_Os03g23940.1	148	-23.83	148	-23.83	879858	20	838	804
miR1846d-3p	LOC_Os03g24110.1	305	-48.96	158	-27.34	879876	20	5934	426 2462
miR1846d-3p	LOC_Os03g24120.1	146	-22.32	146	-22.32	879877	20	3084	1396
miR1846d-3p	LOC_Os03g24270.1	303	-45.13	156	-23.52	879893	20	6084	507 2546
miR1846d-3p	LOC_Os03g24360.1	303	-45.14	156	-23.52	879900	20	6084	507 2546
miR1846d-3p	LOC_Os03g24370.1	316	-61.05	172	-36.72	879901	20	3078	1741 1763
miR1846d-3p	LOC_Os03g24440.1	169	-29.7	169	-29.7	879908	20	987	255
miR1846d-3p	LOC_Os03g24610.1	158	-23.29	158	-23.29	879926	20	1572	279
miR1846d-3p	LOC_Os03g25080.1	151	-26.46	151	-26.46	879975	20	1907	1262
miR1846d-3p	LOC_Os03g25920.1	156	-24.97	156	-24.97	880067	20	1500	855
miR1846d-3p	LOC_Os03g25970.1	151	-29.73	151	-29.73	880074	20	2987	2858
miR1846d-3p	LOC_Os03g25970.2	151	-29.73	151	-29.73	880075	20	3154	3025
miR1846d-3p	LOC_Os03g26080.1	159	-28.39	159	-28.39	880087	20	1899	362
miR1846d-3p	LOC_Os03g26520.1	150	-21.81	150	-21.81	880125	20	606	243

miR1846d-3p	LOC_Os03g26760.1	299	-44.29	154	-23.72	880146	20	5469	5355 5375
miR1846d-3p	LOC_Os03g26870.1	151	-20.96	151	-20.96	880157	20	2055	1223
miR1846d-3p	LOC_Os03g26870.2	151	-20.96	151	-20.96	880158	20	3358	2526
miR1846d-3p	LOC_Os03g27140.1	297	-42.37	150	-21.62	880192	20	5526	507 2546
miR1846d-3p	LOC_Os03g27250.1	140	-21.42	140	-21.42	880200	20	3297	114
miR1846d-3p	LOC_Os03g27830.1	309	-50.9	165	-30.31	880270	20	2715	2426 2404
miR1846d-3p	LOC_Os03g28010.1	140	-20.04	140	-20.04	880290	20	5928	5289
miR1846d-3p	LOC_Os03g28090.1	146	-20.99	146	-20.99	880298	20	2246	1486
miR1846d-3p	LOC_Os03g28110.1	153	-20.86	153	-20.86	880300	20	3912	2447
miR1846d-3p	LOC_Os03g28920.1	148	-22.13	148	-22.13	880336	20	378	206
miR1846d-3p	LOC_Os03g29020.1	148	-23.14	148	-23.14	880347	20	2766	2728
miR1846d-3p	LOC_Os03g29184.1	155	-30.3	155	-30.3	880364	20	774	119
miR1846d-3p	LOC_Os03g29222.1	160	-25.64	160	-25.64	880372	20	1695	394
miR1846d-3p	LOC_Os03g29790.1	143	-21.79	143	-21.79	880426	20	4647	3624
miR1846d-3p	LOC_Os03g29950.1	153	-24.51	153	-24.51	880439	20	1993	536
miR1846d-3p	LOC_Os03g29950.2	153	-24.51	153	-24.51	880440	20	1983	526
miR1846d-3p	LOC_Os03g29990.1	154	-22.93	154	-22.93	880444	20	294	217
miR1846d-3p	LOC_Os03g30250.1	145	-21.96	145	-21.96	880473	20	2565	708
miR1846d-3p	LOC_Os03g30390.1	149	-22.27	149	-22.27	880486	20	1833	391
miR1846d-3p	LOC_Os03g30410.1	145	-21.13	145	-21.13	880488	20	294	226
miR1846d-3p	LOC_Os03g30470.1	175	-38.01	175	-38.01	880495	20	1082	214
miR1846d-3p	LOC_Os03g30720.1	144	-25.6	144	-25.6	880520	20	1086	738
miR1846d-3p	LOC_Os03g30810.1	157	-23.98	157	-23.98	880530	20	3411	1709
miR1846d-3p	LOC_Os03g30980.1	151	-24.69	151	-24.69	880548	20	1887	1587
miR1846d-3p	LOC_Os03g31134.1	146	-20.01	146	-20.01	880563	20	4557	665
miR1846d-3p	LOC_Os03g31390.1	152	-21.56	152	-21.56	880598	20	324	169
miR1846d-3p	LOC_Os03g31650.1	152	-21.65	152	-21.65	880625	20	3684	3311
miR1846d-3p	LOC_Os03g31790.1	142	-21.29	142	-21.29	880639	20	291	135
miR1846d-3p	LOC_Os03g32314.1	469	-79.5	161	-29.81	880681	20	2669	2301 1068 2149
miR1846d-3p	LOC_Os03g32490.1	157	-24.56	157	-24.56	880698	20	1230	1078
miR1846d-3p	LOC_Os03g32499.1	153	-21.63	153	-21.63	880699	20	708	108
miR1846d-3p	LOC_Os03g32526.1	157	-30.58	157	-30.58	880700	20	2820	2788

miR1846d-3p	LOC_Os03g32580.1	149	-25.36	149	-25.36	880705	20	1183	147
miR1846d-3p	LOC_Os03g32850.1	142	-20.57	142	-20.57	880732	20	2430	37
miR1846d-3p	LOC_Os03g33280.1	160	-27.35	160	-27.35	880775	20	3399	1060
miR1846d-3p	LOC_Os03g33640.1	146	-20.01	146	-20.01	880808	20	3594	17
miR1846d-3p	LOC_Os03g33690.1	144	-22.93	144	-22.93	880813	20	2250	24
miR1846d-3p	LOC_Os03g33900.1	156	-23.88	156	-23.88	880834	20	1428	1113
miR1846d-3p	LOC_Os03g34190.1	148	-21.47	148	-21.47	880860	20	4959	95
miR1846d-3p	LOC_Os03g36660.1	147	-21.62	147	-21.62	880996	20	4626	1391
miR1846d-3p	LOC_Os03g37010.1	165	-31.22	165	-31.22	881027	20	2824	2594
miR1846d-3p	LOC_Os03g38210.1	145	-23.33	145	-23.33	881144	20	1829	486
miR1846d-3p	LOC_Os03g38510.1	152	-29.88	152	-29.88	881169	20	330	151
miR1846d-3p	LOC_Os03g38560.1	166	-27.37	166	-27.37	881177	20	2742	470
miR1846d-3p	LOC_Os03g38670.1	309	-53.11	156	-26.79	881188	20	516	365 193
miR1846d-3p	LOC_Os03g38710.1	156	-25.77	156	-25.77	881192	20	1563	1203
miR1846d-3p	LOC_Os03g38800.1	309	-45.25	157	-23.68	881204	20	1990	699 1716
miR1846d-3p	LOC_Os03g39350.1	154	-21.7	154	-21.7	881257	20	5136	8
miR1846d-3p	LOC_Os03g39580.1	162	-24.8	162	-24.8	881280	20	3036	659
miR1846d-3p	LOC_Os03g39720.1	155	-22.68	155	-22.68	881298	20	966	425
miR1846d-3p	LOC_Os03g39740.1	298	-45.09	153	-22.74	881300	20	3485	3189 3158
miR1846d-3p	LOC_Os03g39740.2	298	-45.09	153	-22.74	881301	20	3394	3102 3071
miR1846d-3p	LOC_Os03g39910.1	298	-48.77	154	-24.78	881314	20	2886	2657 709
miR1846d-3p	LOC_Os03g39920.1	301	-45.61	154	-23.99	881315	20	5736	507 2555
miR1846d-3p	LOC_Os03g40070.1	151	-26.11	151	-26.11	881328	20	1202	426
miR1846d-3p	LOC_Os03g40410.1	150	-25.62	150	-25.62	881359	20	1854	1610
miR1846d-3p	LOC_Os03g40410.2	150	-25.62	150	-25.62	881360	20	1840	1613
miR1846d-3p	LOC_Os03g40410.3	150	-25.62	150	-25.62	881361	20	1710	1483
miR1846d-3p	LOC_Os03g40560.1	144	-21.65	144	-21.65	881373	20	3519	1794
miR1846d-3p	LOC_Os03g40800.1	151	-26.6	151	-26.6	881390	20	246	161
miR1846d-3p	LOC_Os03g40830.1	149	-23.05	149	-23.05	881392	20	3246	2354
miR1846d-3p	LOC_Os03g41675.1	158	-26.15	158	-26.15	881471	20	1192	1073
miR1846d-3p	LOC_Os03g41910.1	151	-21.2	151	-21.2	881491	20	237	147
miR1846d-3p	LOC_Os03g41980.1	154	-23.99	154	-23.99	881497	20	5637	507

miR1846d-3p	LOC_Os03g42020.1	153	-27.41	153	-27.41	881502	20	3516	89
miR1846d-3p	LOC_Os03g42130.1	145	-21.02	145	-21.02	881513	20	1360	677
miR1846d-3p	LOC_Os03g42190.1	165	-33.17	165	-33.17	881518	20	726	593
miR1846d-3p	LOC_Os03g42334.1	162	-32.74	162	-32.74	881535	20	537	44
miR1846d-3p	LOC_Os03g42569.1	152	-24.4	152	-24.4	881556	20	656	156
miR1846d-3p	LOC_Os03g42750.1	154	-22.75	154	-22.75	881572	20	3078	2720
miR1846d-3p	LOC_Os03g42750.2	154	-22.75	154	-22.75	881573	20	3333	2975
miR1846d-3p	LOC_Os03g43510.1	143	-24.56	143	-24.56	881654	20	2247	1675
miR1846d-3p	LOC_Os03g43510.2	143	-24.56	143	-24.56	881655	20	2341	1769
miR1846d-3p	LOC_Os03g43590.1	152	-26.49	152	-26.49	881665	20	2662	2214
miR1846d-3p	LOC_Os03g44030.1	586	-95.06	151	-25.82	881716	20	704	277 308 368 398
miR1846d-3p	LOC_Os03g44270.1	146	-20.01	146	-20.01	881737	20	2316	1127
miR1846d-3p	LOC_Os03g44300.1	158	-25.32	158	-25.32	881742	20	1663	326
miR1846d-3p	LOC_Os03g44360.1	146	-21.31	146	-21.31	881749	20	3900	2026
miR1846d-3p	LOC_Os03g44390.1	299	-45.66	152	-24.04	881752	20	5646	396 2504
miR1846d-3p	LOC_Os03g44420.1	157	-24.29	157	-24.29	881755	20	2204	730
miR1846d-3p	LOC_Os03g44510.1	152	-22.05	152	-22.05	881765	20	1926	1504
miR1846d-3p	LOC_Os03g44540.1	165	-30.48	165	-30.48	881769	20	2188	1404
miR1846d-3p	LOC_Os03g44800.1	320	-55.67	160	-28.92	881800	20	1973	583 689
miR1846d-3p	LOC_Os03g44910.1	148	-21.28	148	-21.28	881820	20	639	406
miR1846d-3p	LOC_Os03g45350.1	152	-25.49	152	-25.49	881862	20	324	218
miR1846d-3p	LOC_Os03g45370.1	153	-23.99	153	-23.99	881863	20	2390	849
miR1846d-3p	LOC_Os03g45800.1	149	-23.38	149	-23.38	881902	20	357	327
miR1846d-3p	LOC_Os03g46040.1	151	-25.61	151	-25.61	881923	20	2193	135
miR1846d-3p	LOC_Os03g46052.1	152	-24.2	152	-24.2	881924	20	615	321
miR1846d-3p	LOC_Os03g46100.1	145	-22.56	145	-22.56	881930	20	1965	1383
miR1846d-3p	LOC_Os03g46100.2	145	-22.56	145	-22.56	881931	20	1890	1308
miR1846d-3p	LOC_Os03g46100.4	145	-22.56	145	-22.56	881932	20	923	293
miR1846d-3p	LOC_Os03g46100.5	145	-22.56	145	-22.56	881933	20	1008	378
miR1846d-3p	LOC_Os03g46220.1	305	-47.07	158	-25.45	881946	20	5673	324 2360
miR1846d-3p	LOC_Os03g46330.1	140	-20.14	140	-20.14	881960	20	6123	4901
miR1846d-3p	LOC_Os03g46340.1	140	-20.14	140	-20.14	881961	20	3131	599

miR1846d-3p	LOC_Os03g46454.1	149	-23.34	149	-23.34	881975	20	826	486
miR1846d-3p	LOC_Os03g46460.1	149	-25.53	149	-25.53	881976	20	3674	3340
miR1846d-3p	LOC_Os03g46470.1	149	-25.53	149	-25.53	881977	20	1508	688
miR1846d-3p	LOC_Os03g46770.1	165	-31.22	165	-31.22	882008	20	991	110
miR1846d-3p	LOC_Os03g46970.1	305	-50.53	158	-28.91	882026	20	6087	507 2549
miR1846d-3p	LOC_Os03g47380.1	301	-45.38	153	-23.07	882067	20	804	678 708
miR1846d-3p	LOC_Os03g47470.1	150	-21.87	150	-21.87	882074	20	1605	1
miR1846d-3p	LOC_Os03g47550.1	159	-26.98	159	-26.98	882082	20	1357	1097
miR1846d-3p	LOC_Os03g47550.2	159	-27.29	159	-27.29	882083	20	1254	992
miR1846d-3p	LOC_Os03g47580.1	304	-50.12	159	-28.7	882088	20	2492	2407 2350
miR1846d-3p	LOC_Os03g47590.1	166	-26.59	166	-26.59	882090	20	3135	659
miR1846d-3p	LOC_Os03g47686.1	156	-28.21	156	-28.21	882105	20	1655	6
miR1846d-3p	LOC_Os03g47830.1	146	-22.71	146	-22.71	882124	20	2679	2517
miR1846d-3p	LOC_Os03g47970.1	157	-26.84	157	-26.84	882139	20	1479	1150
miR1846d-3p	LOC_Os03g47970.2	157	-26.84	157	-26.84	882140	20	1002	673
miR1846d-3p	LOC_Os03g48150.1	159	-27.14	159	-27.14	882161	20	489	37
miR1846d-3p	LOC_Os03g48550.1	154	-21.35	154	-21.35	882204	20	426	172
miR1846d-3p	LOC_Os03g48970.1	154	-26.26	154	-26.26	882259	20	1594	947
miR1846d-3p	LOC_Os03g48970.2	154	-26.26	154	-26.26	882260	20	2123	1476
miR1846d-3p	LOC_Os03g48970.3	154	-26.26	154	-26.26	882261	20	2293	1646
miR1846d-3p	LOC_Os03g48970.4	154	-26.26	154	-26.26	882262	20	1586	939
miR1846d-3p	LOC_Os03g49040.1	151	-23.12	151	-23.12	882269	20	2394	1446
miR1846d-3p	LOC_Os03g49080.1	144	-21.22	144	-21.22	882272	20	495	37
miR1846d-3p	LOC_Os03g49430.1	156	-29.25	156	-29.25	882314	20	1861	1188
miR1846d-3p	LOC_Os03g49430.3	156	-29.25	156	-29.25	882315	20	2891	2409
miR1846d-3p	LOC_Os03g49430.4	156	-29.25	156	-29.25	882316	20	2280	1607
miR1846d-3p	LOC_Os03g49485.1	145	-29.03	145	-29.03	882323	20	866	543
miR1846d-3p	LOC_Os03g49520.1	144	-20.89	144	-20.89	882329	20	1491	616
miR1846d-3p	LOC_Os03g49520.2	144	-20.89	144	-20.89	882330	20	1656	616
miR1846d-3p	LOC_Os03g49550.1	147	-25.43	147	-25.43	882334	20	1884	668
miR1846d-3p	LOC_Os03g49630.1	157	-26.55	157	-26.55	882351	20	2240	2175
miR1846d-3p	LOC_Os03g49630.2	157	-26.55	157	-26.55	882352	20	2235	2170

miR1846d-3p	LOC_Os03g49740.1	156	-25.93	156	-25.93	882366	20	573	153
miR1846d-3p	LOC_Os03g49790.1	153	-20.72	153	-20.72	882370	20	1056	335
miR1846d-3p	LOC_Os03g49880.1	159	-29.38	159	-29.38	882379	20	1959	1490
miR1846d-3p	LOC_Os03g49910.1	146	-20.01	146	-20.01	882382	20	3633	17
miR1846d-3p	LOC_Os03g50030.1	151	-22.92	151	-22.92	882394	20	1360	908
miR1846d-3p	LOC_Os03g50070.1	169	-28.26	169	-28.26	882398	20	1608	54
miR1846d-3p	LOC_Os03g50230.1	148	-22.77	148	-22.77	882416	20	2854	2682
miR1846d-3p	LOC_Os03g50310.1	148	-24.44	148	-24.44	882426	20	2055	303
miR1846d-3p	LOC_Os03g50355.1	156	-26.01	156	-26.01	882432	20	1520	1085
miR1846d-3p	LOC_Os03g50420.1	152	-29.28	152	-29.28	882439	20	1368	333
miR1846d-3p	LOC_Os03g51220.1	157	-26.48	157	-26.48	882524	20	3028	488
miR1846d-3p	LOC_Os03g51230.1	151	-21.8	151	-21.8	882525	20	3121	2384
miR1846d-3p	LOC_Os03g51230.2	151	-21.8	151	-21.8	882526	20	3230	2493
miR1846d-3p	LOC_Os03g51250.1	145	-20.67	145	-20.67	882530	20	1895	1675
miR1846d-3p	LOC_Os03g51310.1	305	-50.53	158	-28.91	882536	20	5247	507 2375
miR1846d-3p	LOC_Os03g51360.1	158	-28.91	158	-28.91	882541	20	2208	507
miR1846d-3p	LOC_Os03g51610.1	146	-22.26	146	-22.26	882571	20	2672	2076
miR1846d-3p	LOC_Os03g51620.1	160	-32.33	160	-32.33	882572	20	2867	2703
miR1846d-3p	LOC_Os03g51724.1	146	-20.01	146	-20.01	882588	20	786	131
miR1846d-3p	LOC_Os03g51770.1	162	-24.8	162	-24.8	882593	20	3222	659
miR1846d-3p	LOC_Os03g52020.1	150	-24.61	150	-24.61	882617	20	578	168
miR1846d-3p	LOC_Os03g52170.1	164	-35.23	164	-35.23	882634	20	2524	2414
miR1846d-3p	LOC_Os03g52190.1	305	-47.07	158	-25.45	882636	20	5556	507 2546
miR1846d-3p	LOC_Os03g52360.1	157	-24.93	157	-24.93	882656	20	739	223
miR1846d-3p	LOC_Os03g52510.1	151	-24.69	151	-24.69	882672	20	1584	1284
miR1846d-3p	LOC_Os03g52530.1	164	-28.95	164	-28.95	882674	20	2292	846
miR1846d-3p	LOC_Os03g52570.1	155	-24.65	155	-24.65	882678	20	1404	440
miR1846d-3p	LOC_Os03g52620.1	296	-45.2	154	-25.07	882681	20	1636	635 732
miR1846d-3p	LOC_Os03g52660.1	150	-23.52	150	-23.52	882688	20	1771	605
miR1846d-3p	LOC_Os03g52670.1	150	-23.52	150	-23.52	882689	20	2243	136
miR1846d-3p	LOC_Os03g52810.1	305	-50.53	158	-28.91	882709	20	5964	507 2549
miR1846d-3p	LOC_Os03g52910.1	149	-22.11	149	-22.11	882721	20	1300	558

miR1846d-3p	LOC_Os03g53050.1	153	-21.62	153	-21.62	882736	20	1951	1350
miR1846d-3p	LOC_Os03g53170.1	152	-21.92	152	-21.92	882750	20	3041	1187
miR1846d-3p	LOC_Os03g53190.2	152	-24.32	152	-24.32	882753	20	1054	118
miR1846d-3p	LOC_Os03g53210.1	160	-28.49	160	-28.49	882755	20	1476	1214
miR1846d-3p	LOC_Os03g53419.1	296	-44.22	149	-22.83	882780	20	681	599 45
miR1846d-3p	LOC_Os03g53560.1	146	-20.01	146	-20.01	882794	20	3963	17
miR1846d-3p	LOC_Os03g53700.1	301	-44.54	152	-23.74	882815	20	1923	113 60
miR1846d-3p	LOC_Os03g53700.2	301	-44.54	152	-23.74	882816	20	1033	113 60
miR1846d-3p	LOC_Os03g53790.1	148	-25.24	148	-25.24	882827	20	2260	261
miR1846d-3p	LOC_Os03g53800.1	152	-22.19	152	-22.19	882828	20	2695	676
miR1846d-3p	LOC_Os03g53800.2	152	-22.19	152	-22.19	882829	20	2258	239
miR1846d-3p	LOC_Os03g53800.3	152	-22.19	152	-22.19	882830	20	3103	1084
miR1846d-3p	LOC_Os03g53800.4	152	-22.19	152	-22.19	882831	20	2283	264
miR1846d-3p	LOC_Os03g53800.5	152	-22.19	152	-22.19	882832	20	2832	813
miR1846d-3p	LOC_Os03g53810.1	147	-21.62	147	-21.62	882833	20	6039	2552
miR1846d-3p	LOC_Os03g53820.1	172	-34.12	172	-34.12	882834	20	3165	1828
miR1846d-3p	LOC_Os03g53910.1	148	-20.32	148	-20.32	882850	20	1641	8
miR1846d-3p	LOC_Os03g54240.1	157	-29.44	157	-29.44	882889	20	2007	437
miR1846d-3p	LOC_Os03g54900.1	146	-21.3	146	-21.3	882908	20	4555	3164
miR1846d-3p	LOC_Os03g54900.2	146	-21.3	146	-21.3	882909	20	4594	3261
miR1846d-3p	LOC_Os03g54950.1	166	-26.59	166	-26.59	882913	20	2280	191
miR1846d-3p	LOC_Os03g55150.1	155	-23.95	155	-23.95	882939	20	989	4
miR1846d-3p	LOC_Os03g55150.4	155	-23.95	155	-23.95	882940	20	950	4
miR1846d-3p	LOC_Os03g55150.5	155	-23.95	155	-23.95	882941	20	490	4
miR1846d-3p	LOC_Os03g55150.6	155	-23.95	155	-23.95	882942	20	950	4
miR1846d-3p	LOC_Os03g55240.1	145	-20.55	145	-20.55	882951	20	2545	1031
miR1846d-3p	LOC_Os03g55260.1	165	-29.71	165	-29.71	882953	20	1941	609
miR1846d-3p	LOC_Os03g55420.1	147	-20.77	147	-20.77	882971	20	1426	752
miR1846d-3p	LOC_Os03g55600.1	159	-24.46	159	-24.46	882991	20	1709	217
miR1846d-3p	LOC_Os03g56110.1	158	-25.62	158	-25.62	883057	20	1844	611
miR1846d-3p	LOC_Os03g56110.2	158	-25.62	158	-25.62	883058	20	1673	455
miR1846d-3p	LOC_Os03g56140.1	158	-25.62	158	-25.62	883061	20	1350	309

miR1846d-3p	LOC_Os03g56140.2	158	-25.62	158	-25.62	883062	20	1245	309
miR1846d-3p	LOC_Os03g56220.1	157	-27.13	157	-27.13	883068	20	2025	1733
miR1846d-3p	LOC_Os03g56270.1	155	-24.68	155	-24.68	883074	20	3741	664
miR1846d-3p	LOC_Os03g56270.2	155	-24.68	155	-24.68	883075	20	3306	658
miR1846d-3p	LOC_Os03g56270.3	155	-24.68	155	-24.68	883076	20	3553	658
miR1846d-3p	LOC_Os03g56380.1	303	-49.02	153	-25.29	883092	20	2776	488 452
miR1846d-3p	LOC_Os03g56940.1	165	-32.99	165	-32.99	883168	20	1155	402
miR1846d-3p	LOC_Os03g57170.1	145	-22.66	145	-22.66	883207	20	2143	72
miR1846d-3p	LOC_Os03g57170.2	145	-22.66	145	-22.66	883208	20	1525	72
miR1846d-3p	LOC_Os03g57170.3	145	-22.66	145	-22.66	883209	20	2011	72
miR1846d-3p	LOC_Os03g57210.1	165	-29.64	165	-29.64	883213	20	1297	43
miR1846d-3p	LOC_Os03g57210.2	165	-29.64	165	-29.64	883214	20	782	43
miR1846d-3p	LOC_Os03g57340.1	147	-24.74	147	-24.74	883231	20	1558	23
miR1846d-3p	LOC_Os03g57430.1	145	-20.39	145	-20.39	883241	20	4080	3817
miR1846d-3p	LOC_Os03g57470.1	162	-24.8	162	-24.8	883244	20	3141	659
miR1846d-3p	LOC_Os03g57530.1	156	-27.18	156	-27.18	883251	20	1526	1239
miR1846d-3p	LOC_Os03g57545.1	150	-22.25	150	-22.25	883253	20	591	181
miR1846d-3p	LOC_Os03g57820.1	149	-22.27	149	-22.27	883284	20	2580	505
miR1846d-3p	LOC_Os03g58000.1	151	-25.59	151	-25.59	883312	20	1461	1093
miR1846d-3p	LOC_Os03g58650.2	161	-28.75	161	-28.75	883387	20	1226	214
miR1846d-3p	LOC_Os03g58700.1	144	-21.7	144	-21.7	883392	20	3038	207
miR1846d-3p	LOC_Os03g58790.1	157	-25.19	157	-25.19	883402	20	2122	1571
miR1846d-3p	LOC_Os03g58820.1	156	-25.16	156	-25.16	883405	20	1494	1355
miR1846d-3p	LOC_Os03g59080.1	148	-25.9	148	-25.9	883433	20	2718	2592
miR1846d-3p	LOC_Os03g59340.1	155	-26.2	155	-26.2	883461	20	4488	3719
miR1846d-3p	LOC_Os03g59350.1	152	-25.77	152	-25.77	883462	20	1658	1305
miR1846d-3p	LOC_Os03g59410.1	305	-50.53	158	-28.91	883468	20	5979	507 2546
miR1846d-3p	LOC_Os03g59430.2	159	-26.47	159	-26.47	883470	20	2707	1935
miR1846d-3p	LOC_Os03g59500.1	301	-44.34	154	-22.72	883476	20	5523	279 2318
miR1846d-3p	LOC_Os03g59600.1	158	-29.35	158	-29.35	883486	20	762	480
miR1846d-3p	LOC_Os03g59770.1	146	-20.54	146	-20.54	883509	20	884	652
miR1846d-3p	LOC_Os03g59790.1	150	-20.54	150	-20.54	883512	20	603	541

miR1846d-3p	LOC_Os03g59870.1	150	-20.54	150	-20.54	883519	20	624	562
miR1846d-3p	LOC_Os03g59990.1	144	-21.08	144	-21.08	883531	20	531	333
miR1846d-3p	LOC_Os03g60230.1	146	-20.01	146	-20.01	883556	20	3675	17
miR1846d-3p	LOC_Os03g60300.1	156	-26.39	156	-26.39	883563	20	1619	649
miR1846d-3p	LOC_Os03g60670.1	154	-28.51	154	-28.51	883605	20	2382	1016
miR1846d-3p	LOC_Os03g60939.1	145	-24.12	145	-24.12	883639	20	1237	304
miR1846d-3p	LOC_Os03g61140.1	149	-28.61	149	-28.61	883659	20	1236	744
miR1846d-3p	LOC_Os03g61140.2	149	-28.61	149	-28.61	883660	20	1353	744
miR1846d-3p	LOC_Os03g61330.4	156	-22.72	156	-22.72	883692	20	1398	1120
miR1846d-3p	LOC_Os03g61720.1	162	-22.6	162	-22.6	883734	20	1938	1596
miR1846d-3p	LOC_Os03g61780.1	157	-24.57	157	-24.57	883740	20	2125	432
miR1846d-3p	LOC_Os03g61780.2	157	-24.57	157	-24.57	883741	20	2080	375
miR1846d-3p	LOC_Os03g62250.1	155	-26.32	155	-26.32	883811	20	2112	2052
miR1846d-3p	LOC_Os03g62260.1	155	-26.32	155	-26.32	883812	20	2137	1946
miR1846d-3p	LOC_Os03g62370.1	146	-22.66	146	-22.66	883823	20	2371	1423
miR1846d-3p	LOC_Os03g62630.1	144	-22.92	144	-22.92	883859	20	1055	172
miR1846d-3p	LOC_Os03g62670.1	175	-37.96	175	-37.96	883869	20	1013	284
miR1846d-3p	LOC_Os03g62670.2	175	-37.96	175	-37.96	883870	20	1072	278
miR1846d-3p	LOC_Os03g62670.3	175	-37.96	175	-37.96	883871	20	1308	278
miR1846d-3p	LOC_Os03g63190.1	155	-27.29	155	-27.29	883941	20	549	518
miR1846d-3p	LOC_Os03g63510.1	146	-23.01	146	-23.01	883979	20	2315	1707
miR1846d-3p	LOC_Os03g63540.1	158	-25.8	158	-25.8	883983	20	1390	727
miR1846d-3p	LOC_Os03g63630.1	303	-45.14	156	-23.52	883992	20	6081	504 2543
miR1846d-3p	LOC_Os03g63660.1	148	-24.07	148	-24.07	883996	20	2085	1416
miR1846d-3p	LOC_Os03g63790.1	303	-45.14	156	-23.52	884016	20	6084	507 2546
miR1846d-3p	LOC_Os03g63810.1	149	-23.72	149	-23.72	884018	20	1624	975
miR1846d-3p	LOC_Os03g64170.1	161	-28.38	161	-28.38	884057	20	1697	557
miR1846d-3p	LOC_Os03g64170.2	161	-28.38	161	-28.38	884058	20	1305	557
miR1846d-3p	LOC_Os03g64400.1	143	-21.23	143	-21.23	884090	20	3257	1874
miR1846d-3p	LOC_Os03g64400.2	143	-21.23	143	-21.23	884091	20	3148	1765
miR1846d-3p	LOC_Os03g64400.3	143	-21.23	143	-21.23	884092	20	2416	1765
miR1846d-3p	LOC_Os03g64400.4	143	-21.23	143	-21.23	884093	20	3271	1888

miR1846d-3p	LOC_Os04g01160.1	159	-27.64	159	-27.64	884114	20	2638	1144
miR1846d-3p	LOC_Os04g01190.1	149	-21.28	149	-21.28	884117	20	1983	325
miR1846d-3p	LOC_Os04g01380.1	154	-22.58	154	-22.58	884142	20	1614	639
miR1846d-3p	LOC_Os04g01620.1	305	-50.53	158	-28.91	884168	20	5799	507 2546
miR1846d-3p	LOC_Os04g01900.1	165	-31.59	165	-31.59	884196	20	831	296
miR1846d-3p	LOC_Os04g02110.1	145	-21.26	145	-21.26	884222	20	5222	5040
miR1846d-3p	LOC_Os04g02110.2	145	-21.26	145	-21.26	884223	20	3912	3730
miR1846d-3p	LOC_Os04g02110.3	145	-21.26	145	-21.26	884224	20	4947	4765
miR1846d-3p	LOC_Os04g02375.1	144	-23.56	144	-23.56	884249	20	653	132
miR1846d-3p	LOC_Os04g02470.1	149	-22.8	149	-22.8	884258	20	528	376
miR1846d-3p	LOC_Os04g02590.1	154	-25.03	154	-25.03	884276	20	384	286
miR1846d-3p	LOC_Os04g02960.1	146	-20.01	146	-20.01	884318	20	8196	4220
miR1846d-3p	LOC_Os04g03040.1	142	-22.55	142	-22.55	884326	20	3288	1970
miR1846d-3p	LOC_Os04g03100.1	156	-26.46	156	-26.46	884329	20	2532	485
miR1846d-3p	LOC_Os04g03150.1	158	-23.73	158	-23.73	884334	20	3159	659
miR1846d-3p	LOC_Os04g03320.1	147	-26.49	147	-26.49	884350	20	552	490
miR1846d-3p	LOC_Os04g03440.1	297	-46.2	152	-26.12	884362	20	1554	599 1257
miR1846d-3p	LOC_Os04g03490.1	152	-25.43	152	-25.43	884367	20	4005	3309
miR1846d-3p	LOC_Os04g03550.1	140	-21.04	140	-21.04	884371	20	3072	1506
miR1846d-3p	LOC_Os04g03870.1	160	-31.65	160	-31.65	884401	20	1443	837
miR1846d-3p	LOC_Os04g03880.1	312	-53.06	159	-28.15	884402	20	594	253 170
miR1846d-3p	LOC_Os04g03910.1	148	-23.45	148	-23.45	884405	20	1005	797
miR1846d-3p	LOC_Os04g03950.1	161	-26.38	161	-26.38	884409	20	2376	1213
miR1846d-3p	LOC_Os04g04060.1	156	-23.73	156	-23.73	884421	20	2995	2684
miR1846d-3p	LOC_Os04g04330.1	153	-22.91	153	-22.91	884447	20	4151	3271
miR1846d-3p	LOC_Os04g04330.2	153	-22.91	153	-22.91	884448	20	4039	3248
miR1846d-3p	LOC_Os04g04390.1	159	-27.03	159	-27.03	884453	20	5394	5239
miR1846d-3p	LOC_Os04g04490.1	143	-21.79	143	-21.79	884462	20	4401	3378
miR1846d-3p	LOC_Os04g04720.1	158	-25.45	158	-25.45	884483	20	4632	168
miR1846d-3p	LOC_Os04g04770.1	155	-24.03	155	-24.03	884487	20	866	278
miR1846d-3p	LOC_Os04g04900.1	154	-25.77	154	-25.77	884500	20	4125	2296
miR1846d-3p	LOC_Os04g05520.1	303	-45.14	156	-23.52	884557	20	6084	507 2546

miR1846d-3p	LOC_Os04g05610.1	142	-22.55	142	-22.55	884566	20	3117	1814
miR1846d-3p	LOC_Os04g05680.1	152	-21.82	152	-21.82	884571	20	2418	1252
miR1846d-3p	LOC_Os04g05760.1	144	-23.34	144	-23.34	884578	20	1143	55
miR1846d-3p	LOC_Os04g05770.1	161	-23.94	161	-23.94	884579	20	2019	1754
miR1846d-3p	LOC_Os04g06920.1	143	-20.5	143	-20.5	884687	20	1239	643
miR1846d-3p	LOC_Os04g07030.1	157	-23.73	157	-23.73	884696	20	2481	338
miR1846d-3p	LOC_Os04g07090.1	147	-21.62	147	-21.62	884702	20	4113	575
miR1846d-3p	LOC_Os04g07290.1	152	-20.54	152	-20.54	884720	20	2445	397
miR1846d-3p	LOC_Os04g07300.1	145	-20.08	145	-20.08	884721	20	5997	4365
miR1846d-3p	LOC_Os04g08150.1	142	-22.55	142	-22.55	884804	20	2484	1601
miR1846d-3p	LOC_Os04g08320.1	151	-23.37	151	-23.37	884819	20	1349	1218
miR1846d-3p	LOC_Os04g08360.1	889	-145.59	157	-26.52	884826	20	507	387 209 240 270 300
miR1846d-3p	LOC_Os04g08430.1	166	-27.37	166	-27.37	884832	20	3126	659
miR1846d-3p	LOC_Os04g08650.1	159	-27.87	159	-27.87	884855	20	201	81
miR1846d-3p	LOC_Os04g08690.1	149	-22.54	149	-22.54	884859	20	489	240
miR1846d-3p	LOC_Os04g08780.1	146	-20.01	146	-20.01	884873	20	3615	17
miR1846d-3p	LOC_Os04g08815.1	153	-26.37	153	-26.37	884882	20	270	203
miR1846d-3p	LOC_Os04g09340.1	151	-23.8	151	-23.8	884894	20	285	173
miR1846d-3p	LOC_Os04g09590.1	315	-56.65	162	-30.71	884921	20	1296	306 902
miR1846d-3p	LOC_Os04g09830.1	140	-21.04	140	-21.04	884942	20	3663	1480
miR1846d-3p	LOC_Os04g09930.1	147	-21.62	147	-21.62	884952	20	4155	710
miR1846d-3p	LOC_Os04g09950.1	146	-20.74	146	-20.74	884954	20	654	342
miR1846d-3p	LOC_Os04g10160.1	151	-37.74	151	-37.74	884974	20	1916	1561
miR1846d-3p	LOC_Os04g10180.1	162	-26.71	162	-26.71	884976	20	534	374
miR1846d-3p	LOC_Os04g10200.1	145	-20.08	145	-20.08	884978	20	5976	4356
miR1846d-3p	LOC_Os04g10270.1	140	-21.04	140	-21.04	884987	20	2652	1116
miR1846d-3p	LOC_Os04g10420.1	142	-23.42	142	-23.42	885002	20	1736	1652
miR1846d-3p	LOC_Os04g10470.1	157	-26.2	157	-26.2	885006	20	372	193
miR1846d-3p	LOC_Os04g10750.1	153	-26.68	153	-26.68	885032	20	2055	1547
miR1846d-3p	LOC_Os04g10750.2	153	-26.68	153	-26.68	885033	20	3105	2597
miR1846d-3p	LOC_Os04g10750.3	153	-26.68	153	-26.68	885034	20	2090	1582
miR1846d-3p	LOC_Os04g10750.4	153	-26.68	153	-26.68	885035	20	2055	1547

miR1846d-3p	LOC_Os04g10840.1	152	-20.54	152	-20.54	885042	20	2466	397
miR1846d-3p	LOC_Os04g10850.1	145	-20.08	145	-20.08	885043	20	5592	4017
miR1846d-3p	LOC_Os04g11170.1	161	-31.11	161	-31.11	885073	20	2637	1722
miR1846d-3p	LOC_Os04g11350.1	140	-21.26	140	-21.26	885091	20	5017	2542
miR1846d-3p	LOC_Os04g11590.1	156	-28.42	156	-28.42	885114	20	693	612
miR1846d-3p	LOC_Os04g11610.1	144	-22.94	144	-22.94	885116	20	2274	24
miR1846d-3p	LOC_Os04g11890.1	156	-28.19	156	-28.19	885144	20	1628	1266
miR1846d-3p	LOC_Os04g11956.1	149	-20.16	149	-20.16	885150	20	648	214
miR1846d-3p	LOC_Os04g11962.1	296	-46.21	149	-24.41	885151	20	354	96 126
miR1846d-3p	LOC_Os04g12100.1	148	-20.1	148	-20.1	885167	20	629	407
miR1846d-3p	LOC_Os04g12420.1	145	-28.53	145	-28.53	885198	20	2877	1419
miR1846d-3p	LOC_Os04g12760.1	309	-48.25	158	-24.45	885226	20	5667	507 2321
miR1846d-3p	LOC_Os04g12960.1	150	-22	150	-22	885240	20	1673	877
miR1846d-3p	LOC_Os04g13020.1	161	-25.78	161	-25.78	885246	20	2361	338
miR1846d-3p	LOC_Os04g13030.1	145	-20.08	145	-20.08	885247	20	6027	4407
miR1846d-3p	LOC_Os04g13220.1	157	-30.75	157	-30.75	885266	20	4974	4217
miR1846d-3p	LOC_Os04g13280.1	154	-22.25	154	-22.25	885271	20	417	253
miR1846d-3p	LOC_Os04g13310.1	162	-24.8	162	-24.8	885274	20	2715	521
miR1846d-3p	LOC_Os04g13620.1	142	-22.55	142	-22.55	885302	20	3237	1919
miR1846d-3p	LOC_Os04g14140.1	154	-24.72	154	-24.72	885353	20	627	170
miR1846d-3p	LOC_Os04g14520.1	144	-21.74	144	-21.74	885395	20	1968	42
miR1846d-3p	LOC_Os04g14540.1	140	-23.11	140	-23.11	885397	20	2298	1224
miR1846d-3p	LOC_Os04g14610.1	152	-25.45	152	-25.45	885404	20	766	91
miR1846d-3p	LOC_Os04g14940.1	162	-24.8	162	-24.8	885435	20	3198	659
miR1846d-3p	LOC_Os04g15670.1	140	-20.22	140	-20.22	885460	20	627	230
miR1846d-3p	LOC_Os04g16380.1	148	-21.6	148	-21.6	885524	20	2469	1240
miR1846d-3p	LOC_Os04g16550.1	145	-20.02	145	-20.02	885541	20	5787	4356
miR1846d-3p	LOC_Os04g16630.1	140	-21.12	140	-21.12	885548	20	3669	3467
miR1846d-3p	LOC_Os04g16910.1	148	-21.47	148	-21.47	885613	20	4203	95
miR1846d-3p	LOC_Os04g17020.1	146	-20.01	146	-20.01	885622	20	3939	17
miR1846d-3p	LOC_Os04g17064.1	152	-20.94	152	-20.94	885626	20	1569	265
miR1846d-3p	LOC_Os04g17064.2	152	-20.94	152	-20.94	885627	20	1561	265

miR1846d-3p	LOC_Os04g17260.1	156	-23.52	156	-23.52	885646	20	5979	510
miR1846d-3p	LOC_Os04g17430.1	140	-20.15	140	-20.15	885663	20	3093	1333
miR1846d-3p	LOC_Os04g17680.1	311	-51.63	160	-26.25	885688	20	2232	904 609
miR1846d-3p	LOC_Os04g17680.2	311	-51.63	160	-26.25	885689	20	2000	904 609
miR1846d-3p	LOC_Os04g17680.3	311	-51.63	160	-26.25	885690	20	1901	904 609
miR1846d-3p	LOC_Os04g17680.4	311	-51.63	160	-26.25	885691	20	2133	904 609
miR1846d-3p	LOC_Os04g17750.1	162	-24.8	162	-24.8	885697	20	3048	659
miR1846d-3p	LOC_Os04g17900.1	144	-22.93	144	-22.93	885712	20	2148	24
miR1846d-3p	LOC_Os04g18244.1	152	-25.6	152	-25.6	885744	20	4929	2498
miR1846d-3p	LOC_Os04g18380.1	155	-24.94	155	-24.94	885755	20	1857	1534
miR1846d-3p	LOC_Os04g18420.1	172	-34.12	172	-34.12	885759	20	2466	1129
miR1846d-3p	LOC_Os04g18860.1	152	-21.82	152	-21.82	885803	20	2496	1234
miR1846d-3p	LOC_Os04g19550.1	156	-29.93	156	-29.93	885869	20	405	78
miR1846d-3p	LOC_Os04g19590.1	148	-21.47	148	-21.47	885873	20	5013	341
miR1846d-3p	LOC_Os04g19640.1	152	-20.69	152	-20.69	885878	20	1230	29
miR1846d-3p	LOC_Os04g19720.1	305	-50.53	158	-28.91	885887	20	5589	507 2546
miR1846d-3p	LOC_Os04g19740.1	145	-26.31	145	-26.31	885889	20	2567	433
miR1846d-3p	LOC_Os04g19880.1	145	-21.48	145	-21.48	885902	20	699	477
miR1846d-3p	LOC_Os04g19930.1	146	-20.01	146	-20.01	885907	20	888	17
miR1846d-3p	LOC_Os04g20010.1	158	-25.45	158	-25.45	885915	20	2715	507
miR1846d-3p	LOC_Os04g20260.1	164	-30.85	164	-30.85	885937	20	1632	640
miR1846d-3p	LOC_Os04g20340.1	166	-25.39	166	-25.39	885947	20	2391	14
miR1846d-3p	LOC_Os04g20474.1	144	-21.84	144	-21.84	885961	20	4313	3206
miR1846d-3p	LOC_Os04g20474.2	144	-21.84	144	-21.84	885962	20	4181	3075
miR1846d-3p	LOC_Os04g20474.3	144	-21.84	144	-21.84	885963	20	3843	2737
miR1846d-3p	LOC_Os04g20474.4	144	-21.84	144	-21.84	885964	20	4085	2979
miR1846d-3p	LOC_Os04g20620.1	146	-20.01	146	-20.01	885977	20	3705	17
miR1846d-3p	LOC_Os04g20860.1	140	-20.04	140	-20.04	885999	20	2538	1902
miR1846d-3p	LOC_Os04g20880.1	156	-26.69	156	-26.69	886001	20	1271	599
miR1846d-3p	LOC_Os04g20930.1	158	-22.94	158	-22.94	886006	20	420	165
miR1846d-3p	LOC_Os04g20940.1	147	-21.61	147	-21.61	886007	20	5781	2105
miR1846d-3p	LOC_Os04g21260.1	166	-26.29	166	-26.29	886036	20	2904	659

miR1846d-3p	LOC_Os04g21400.1	140	-20.77	140	-20.77	886051	20	1878	1505
miR1846d-3p	LOC_Os04g21560.1	151	-21.79	151	-21.79	886064	20	4578	3669
miR1846d-3p	LOC_Os04g21670.1	142	-22.55	142	-22.55	886075	20	564	521
miR1846d-3p	LOC_Os04g21700.1	148	-23.14	148	-23.14	886078	20	1218	1180
miR1846d-3p	LOC_Os04g21900.1	316	-62.88	169	-32.36	886099	20	1038	918 894
miR1846d-3p	LOC_Os04g21970.1	143	-28.79	143	-28.79	886106	20	1044	894
miR1846d-3p	LOC_Os04g22040.1	157	-26.29	157	-26.29	886113	20	264	46
miR1846d-3p	LOC_Os04g22290.1	152	-20.91	152	-20.91	886139	20	711	268
miR1846d-3p	LOC_Os04g22500.1	154	-21.71	154	-21.71	886159	20	1809	1464
miR1846d-3p	LOC_Os04g22960.1	148	-23.38	148	-23.38	886201	20	1833	547
miR1846d-3p	LOC_Os04g23000.1	152	-26.38	152	-26.38	886205	20	597	161
miR1846d-3p	LOC_Os04g23040.1	154	-22.07	154	-22.07	886209	20	1160	741
miR1846d-3p	LOC_Os04g23200.1	149	-20.3	149	-20.3	886222	20	2313	2279
miR1846d-3p	LOC_Os04g23310.1	145	-20.08	145	-20.08	886233	20	5211	3876
miR1846d-3p	LOC_Os04g23319.1	152	-27.91	152	-27.91	886234	20	924	308
miR1846d-3p	LOC_Os04g23580.1	161	-32.95	161	-32.95	886256	20	2110	1731
miR1846d-3p	LOC_Os04g23870.1	156	-23.52	156	-23.52	886277	20	6087	510
miR1846d-3p	LOC_Os04g23890.1	146	-21.76	146	-21.76	886279	20	3330	1809
miR1846d-3p	LOC_Os04g23890.2	146	-21.76	146	-21.76	886280	20	3248	1897
miR1846d-3p	LOC_Os04g23890.3	146	-21.76	146	-21.76	886281	20	3418	1897
miR1846d-3p	LOC_Os04g23890.4	146	-21.76	146	-21.76	886282	20	3285	1903
miR1846d-3p	LOC_Os04g24080.1	152	-21.82	152	-21.82	886298	20	2484	1252
miR1846d-3p	LOC_Os04g24414.1	156	-24.08	156	-24.08	886338	20	2310	1745
miR1846d-3p	LOC_Os04g24414.2	156	-24.08	156	-24.08	886339	20	2187	1622
miR1846d-3p	LOC_Os04g24414.3	156	-24.08	156	-24.08	886340	20	2207	1642
miR1846d-3p	LOC_Os04g24430.1	154	-23.04	154	-23.04	886341	20	2844	963
miR1846d-3p	LOC_Os04g24590.1	145	-20.08	145	-20.08	886357	20	5979	4362
miR1846d-3p	LOC_Os04g24670.1	144	-22.19	144	-22.19	886365	20	417	361
miR1846d-3p	LOC_Os04g24730.1	144	-23.56	144	-23.56	886371	20	4842	4316
miR1846d-3p	LOC_Os04g24860.1	145	-20.08	145	-20.08	886383	20	1179	1095
miR1846d-3p	LOC_Os04g24940.1	153	-22.46	153	-22.46	886390	20	1635	417
miR1846d-3p	LOC_Os04g25170.1	157	-24.4	157	-24.4	886414	20	438	300

miR1846d-3p	LOC_Os04g25280.1	302	-44.11	152	-22.92	886423	20	2196	213 123
miR1846d-3p	LOC_Os04g25470.1	314	-40.42	157	-20.21	886441	20	5133	2697 3120
miR1846d-3p	LOC_Os04g25600.1	295	-44.39	148	-22.77	886454	20	6054	507 2546
miR1846d-3p	LOC_Os04g25610.1	172	-35.42	172	-35.42	886455	20	3093	1756
miR1846d-3p	LOC_Os04g25790.1	296	-48.17	151	-26.41	886480	20	771	538 470
miR1846d-3p	LOC_Os04g25830.1	156	-21.39	156	-21.39	886484	20	651	349
miR1846d-3p	LOC_Os04g25840.1	148	-26.37	148	-26.37	886485	20	1596	1485
miR1846d-3p	LOC_Os04g25900.1	150	-27.55	150	-27.55	886491	20	3152	2848
miR1846d-3p	LOC_Os04g25900.2	150	-27.55	150	-27.55	886492	20	3074	2767
miR1846d-3p	LOC_Os04g25960.1	154	-26.23	154	-26.23	886498	20	1950	265
miR1846d-3p	LOC_Os04g26090.1	154	-20.05	154	-20.05	886511	20	654	170
miR1846d-3p	LOC_Os04g26480.1	146	-20.01	146	-20.01	886549	20	4071	17
miR1846d-3p	LOC_Os04g26620.1	149	-21.98	149	-21.98	886562	20	474	18
miR1846d-3p	LOC_Os04g26690.1	142	-21.04	142	-21.04	886569	20	2967	1970
miR1846d-3p	LOC_Os04g26810.1	147	-20.45	147	-20.45	886576	20	444	366
miR1846d-3p	LOC_Os04g26960.1	146	-21.59	146	-21.59	886592	20	1554	830
miR1846d-3p	LOC_Os04g27050.1	144	-21.24	144	-21.24	886599	20	321	257
miR1846d-3p	LOC_Os04g27200.1	156	-27.34	156	-27.34	886613	20	375	280
miR1846d-3p	LOC_Os04g27220.1	166	-26.29	166	-26.29	886615	20	3102	659
miR1846d-3p	LOC_Os04g27330.1	156	-27.15	156	-27.15	886628	20	264	60
miR1846d-3p	LOC_Os04g27850.1	145	-20.75	145	-20.75	886677	20	1271	236
miR1846d-3p	LOC_Os04g27870.1	166	-26.59	166	-26.59	886679	20	774	659
miR1846d-3p	LOC_Os04g28234.1	152	-26.92	152	-26.92	886713	20	3591	834
miR1846d-3p	LOC_Os04g28234.2	152	-26.92	152	-26.92	886714	20	3165	834
miR1846d-3p	LOC_Os04g28234.3	152	-26.92	152	-26.92	886715	20	4103	834
miR1846d-3p	LOC_Os04g28300.1	152	-27.46	152	-27.46	886721	20	2535	614
miR1846d-3p	LOC_Os04g28420.1	158	-25.46	158	-25.46	886730	20	2218	1919
miR1846d-3p	LOC_Os04g28420.2	158	-25.46	158	-25.46	886731	20	2214	1915
miR1846d-3p	LOC_Os04g28550.1	144	-22.93	144	-22.93	886745	20	2058	24
miR1846d-3p	LOC_Os04g28640.1	154	-25.04	154	-25.04	886753	20	468	280
miR1846d-3p	LOC_Os04g29010.1	460	-72.4	154	-25.8	886789	20	348	111 193 141
miR1846d-3p	LOC_Os04g29090.1	160	-28.05	160	-28.05	886796	20	1989	1737

miR1846d-3p	LOC_Os04g29190.1	144	-22.93	144	-22.93	886806	20	3693	24
miR1846d-3p	LOC_Os04g29469.1	151	-26.72	151	-26.72	886828	20	7083	2987
miR1846d-3p	LOC_Os04g30630.1	157	-32.55	157	-32.55	886938	20	2172	1081
miR1846d-3p	LOC_Os04g30780.1	158	-26.48	158	-26.48	886953	20	1553	1403
miR1846d-3p	LOC_Os04g30780.2	158	-26.48	158	-26.48	886954	20	1647	1497
miR1846d-3p	LOC_Os04g31080.1	146	-20.01	146	-20.01	886989	20	3213	17
miR1846d-3p	LOC_Os04g31310.1	152	-28.03	152	-28.03	887019	20	198	166
miR1846d-3p	LOC_Os04g31330.1	159	-26.69	159	-26.69	887021	20	2744	1095
miR1846d-3p	LOC_Os04g31500.1	305	-50.53	158	-28.91	887035	20	6072	507 2531
miR1846d-3p	LOC_Os04g31850.1	158	-22.95	158	-22.95	887065	20	3126	659
miR1846d-3p	LOC_Os04g31940.1	148	-25.14	148	-25.14	887074	20	2881	545
miR1846d-3p	LOC_Os04g31960.1	309	-49.06	161	-25.82	887076	20	1863	230 1775
miR1846d-3p	LOC_Os04g32030.1	145	-23.52	145	-23.52	887086	20	1631	1047
miR1846d-3p	LOC_Os04g32320.1	143	-23.3	143	-23.3	887117	20	1305	849
miR1846d-3p	LOC_Os04g32720.1	166	-26.59	166	-26.59	887160	20	2313	341
miR1846d-3p	LOC_Os04g32840.1	145	-20.53	145	-20.53	887172	20	1305	329
miR1846d-3p	LOC_Os04g32970.1	141	-20.52	141	-20.52	887198	20	1378	1081
miR1846d-3p	LOC_Os04g32980.1	156	-25.72	156	-25.72	887199	20	1957	865
miR1846d-3p	LOC_Os04g33020.1	155	-23.33	155	-23.33	887201	20	561	5
miR1846d-3p	LOC_Os04g33460.1	145	-22.36	145	-22.36	887255	20	1266	1024
miR1846d-3p	LOC_Os04g33580.1	298	-47.22	150	-25.8	887269	20	639	398 446
miR1846d-3p	LOC_Os04g33660.1	148	-20.71	148	-20.71	887279	20	1202	620
miR1846d-3p	LOC_Os04g33660.2	148	-20.71	148	-20.71	887280	20	1343	761
miR1846d-3p	LOC_Os04g33885.1	447	-66.77	157	-23.65	887302	20	1536	1131 1112 1275
miR1846d-3p	LOC_Os04g34030.1	149	-23.89	149	-23.89	887318	20	1356	779
miR1846d-3p	LOC_Os04g34500.1	148	-23.59	148	-23.59	887363	20	1413	897
miR1846d-3p	LOC_Os04g34650.1	143	-21.83	143	-21.83	887380	20	3741	1614
miR1846d-3p	LOC_Os04g34910.1	158	-26.1	158	-26.1	887405	20	1042	635
miR1846d-3p	LOC_Os04g35010.1	459	-85.97	157	-31.05	887418	20	3171	2749 2876 2704
miR1846d-3p	LOC_Os04g35030.1	156	-27.24	156	-27.24	887420	20	2554	2474
miR1846d-3p	LOC_Os04g35070.1	152	-23.72	152	-23.72	887423	20	1022	497
miR1846d-3p	LOC_Os04g35320.1	166	-26.59	166	-26.59	887450	20	2916	377

miR1846d-3p	LOC_Os04g35430.1	146	-21.02	146	-21.02	887463	20	4486	3707
miR1846d-3p	LOC_Os04g35440.1	147	-21.62	147	-21.62	887464	20	1338	1214
miR1846d-3p	LOC_Os04g35670.1	792	-128.33	161	-27.47	887495	20	1008	255 381 768 684 76
miR1846d-3p	LOC_Os04g35950.1	297	-46.58	150	-24.96	887527	20	5751	507 2375
miR1846d-3p	LOC_Os04g37450.1	156	-27.34	156	-27.34	887588	20	796	466
miR1846d-3p	LOC_Os04g37650.1	166	-27.96	166	-27.96	887610	20	2068	315
miR1846d-3p	LOC_Os04g37690.1	153	-25.85	153	-25.85	887615	20	1686	345
miR1846d-3p	LOC_Os04g37700.1	150	-31.24	150	-31.24	887616	20	1005	562
miR1846d-3p	LOC_Os04g37790.1	149	-23.11	149	-23.11	887624	20	2534	2087
miR1846d-3p	LOC_Os04g37790.2	149	-23.11	149	-23.11	887625	20	2331	2087
miR1846d-3p	LOC_Os04g37890.1	158	-21.52	158	-21.52	887636	20	1470	178
miR1846d-3p	LOC_Os04g38070.1	312	-49.53	166	-27.37	887655	20	2577	638 360
miR1846d-3p	LOC_Os04g38370.1	325	-52.27	170	-29.59	887686	20	573	350 53
miR1846d-3p	LOC_Os04g38390.1	153	-29.46	153	-29.46	887688	20	1014	432
miR1846d-3p	LOC_Os04g38570.1	149	-21.89	149	-21.89	887709	20	4244	724
miR1846d-3p	LOC_Os04g38570.2	149	-21.89	149	-21.89	887710	20	4335	724
miR1846d-3p	LOC_Os04g38810.1	146	-21.13	146	-21.13	887740	20	2761	1848
miR1846d-3p	LOC_Os04g38930.1	150	-23.7	150	-23.7	887760	20	1830	909
miR1846d-3p	LOC_Os04g38980.1	141	-20.72	141	-20.72	887769	20	2787	2668
miR1846d-3p	LOC_Os04g39680.1	155	-22.39	155	-22.39	887854	20	2803	2588
miR1846d-3p	LOC_Os04g39720.1	166	-25.39	166	-25.39	887857	20	2925	572
miR1846d-3p	LOC_Os04g39780.1	154	-22.62	154	-22.62	887863	20	7452	6891
miR1846d-3p	LOC_Os04g40460.1	150	-23.88	150	-23.88	887943	20	1767	900
miR1846d-3p	LOC_Os04g40470.1	150	-23.88	150	-23.88	887944	20	1733	810
miR1846d-3p	LOC_Os04g40570.1	309	-51.16	155	-25.97	887957	20	3917	3128 933
miR1846d-3p	LOC_Os04g40770.1	142	-24.33	142	-24.33	887988	20	2106	368
miR1846d-3p	LOC_Os04g41030.1	149	-20.8	149	-20.8	888023	20	2851	1746
miR1846d-3p	LOC_Os04g41100.1	145	-22.15	145	-22.15	888031	20	4376	137
miR1846d-3p	LOC_Os04g41100.2	145	-22.15	145	-22.15	888032	20	4281	137
miR1846d-3p	LOC_Os04g41100.3	145	-22.15	145	-22.15	888033	20	4302	137
miR1846d-3p	LOC_Os04g41100.4	145	-22.15	145	-22.15	888034	20	4379	137
miR1846d-3p	LOC_Os04g41100.5	145	-22.15	145	-22.15	888035	20	4448	137

miR1846d-3p	LOC_Os04g41100.6	145	-22.15	145	-22.15	888036	20	4259	134
miR1846d-3p	LOC_Os04g41430.1	149	-23.02	149	-23.02	888070	20	1263	1111
miR1846d-3p	LOC_Os04g41670.1	151	-23.12	151	-23.12	888096	20	2666	1341
miR1846d-3p	LOC_Os04g41860.1	156	-28.23	156	-28.23	888118	20	1044	786
miR1846d-3p	LOC_Os04g41970.1	145	-20.14	145	-20.14	888130	20	2407	1780
miR1846d-3p	LOC_Os04g42330.1	147	-20.56	147	-20.56	888173	20	2630	181
miR1846d-3p	LOC_Os04g42330.2	147	-20.56	147	-20.56	888174	20	2615	181
miR1846d-3p	LOC_Os04g42600.1	145	-21.21	145	-21.21	888211	20	3135	1318
miR1846d-3p	LOC_Os04g42600.2	145	-21.21	145	-21.21	888212	20	2637	820
miR1846d-3p	LOC_Os04g42670.1	154	-20.86	154	-20.86	888217	20	2621	2457
miR1846d-3p	LOC_Os04g42690.1	302	-44.66	153	-22.6	888219	20	1741	1481 1331
miR1846d-3p	LOC_Os04g42760.1	159	-27.65	159	-27.65	888226	20	1511	181
miR1846d-3p	LOC_Os04g43164.1	149	-21.34	149	-21.34	888281	20	1378	1248
miR1846d-3p	LOC_Os04g43164.2	149	-21.34	149	-21.34	888282	20	1536	1406
miR1846d-3p	LOC_Os04g43170.1	150	-21.03	150	-21.03	888283	20	1236	310
miR1846d-3p	LOC_Os04g43880.1	144	-21.4	144	-21.4	888368	20	435	92
miR1846d-3p	LOC_Os04g43970.1	166	-26.59	166	-26.59	888379	20	2268	14
miR1846d-3p	LOC_Os04g44080.1	150	-23.93	150	-23.93	888389	20	264	21
miR1846d-3p	LOC_Os04g44100.1	155	-25.23	155	-25.23	888391	20	1067	32
miR1846d-3p	LOC_Os04g44170.1	148	-24.52	148	-24.52	888398	20	621	516
miR1846d-3p	LOC_Os04g44270.1	149	-23.02	149	-23.02	888411	20	1311	1012
miR1846d-3p	LOC_Os04g44480.1	305	-50.53	158	-28.91	888433	20	5970	489 2528
miR1846d-3p	LOC_Os04g44530.1	152	-24.88	152	-24.88	888438	20	2741	1914
miR1846d-3p	LOC_Os04g44530.2	152	-24.88	152	-24.88	888439	20	2930	2103
miR1846d-3p	LOC_Os04g44530.3	152	-24.88	152	-24.88	888440	20	2434	1607
miR1846d-3p	LOC_Os04g44530.4	152	-24.88	152	-24.88	888441	20	2063	1236
miR1846d-3p	LOC_Os04g44530.5	152	-24.88	152	-24.88	888442	20	2835	2008
miR1846d-3p	LOC_Os04g44530.7	152	-24.88	152	-24.88	888443	20	2841	2014
miR1846d-3p	LOC_Os04g44530.8	152	-24.88	152	-24.88	888444	20	2929	2102
miR1846d-3p	LOC_Os04g44960.1	147	-21.62	147	-21.62	888502	20	6084	2546
miR1846d-3p	LOC_Os04g44970.1	172	-34.12	172	-34.12	888503	20	3090	1753
miR1846d-3p	LOC_Os04g45040.1	157	-26.03	157	-26.03	888513	20	2361	170

miR1846d-3p	LOC_Os04g45040.2	157	-26.03	157	-26.03	888514	20	2260	117
miR1846d-3p	LOC_Os04g45040.3	157	-26.03	157	-26.03	888515	20	2321	170
miR1846d-3p	LOC_Os04g45290.1	146	-21.94	146	-21.94	888544	20	2454	914
miR1846d-3p	LOC_Os04g45290.2	146	-21.94	146	-21.94	888545	20	2706	914
miR1846d-3p	LOC_Os04g45480.1	147	-23.71	147	-23.71	888564	20	2012	545
miR1846d-3p	LOC_Os04g45540.1	158	-23.88	158	-23.88	888569	20	5733	507
miR1846d-3p	LOC_Os04g45700.1	148	-21.95	148	-21.95	888585	20	1119	995
miR1846d-3p	LOC_Os04g45730.1	152	-26.14	152	-26.14	888588	20	1637	1228
miR1846d-3p	LOC_Os04g45780.1	145	-21.51	145	-21.51	888594	20	2499	1492
miR1846d-3p	LOC_Os04g45990.1	165	-32.73	165	-32.73	888620	20	2714	2226
miR1846d-3p	LOC_Os04g46210.1	145	-21.48	145	-21.48	888646	20	1362	179
miR1846d-3p	LOC_Os04g46220.1	149	-24.79	149	-24.79	888647	20	2768	1844
miR1846d-3p	LOC_Os04g46300.1	155	-25.54	155	-25.54	888654	20	3048	2528
miR1846d-3p	LOC_Os04g46500.1	150	-20.75	150	-20.75	888683	20	2049	207
miR1846d-3p	LOC_Os04g46540.1	146	-23.17	146	-23.17	888687	20	366	58
miR1846d-3p	LOC_Os04g46880.1	143	-22.82	143	-22.82	888732	20	2290	1499
miR1846d-3p	LOC_Os04g46880.2	143	-22.82	143	-22.82	888733	20	2229	1499
miR1846d-3p	LOC_Os04g46970.1	291	-42.15	148	-21.83	888747	20	3137	525 568
miR1846d-3p	LOC_Os04g47400.1	147	-20.37	147	-20.37	888796	20	2147	977
miR1846d-3p	LOC_Os04g47730.1	162	-24.8	162	-24.8	888837	20	2304	659
miR1846d-3p	LOC_Os04g47990.1	156	-24.07	156	-24.07	888871	20	2345	1980
miR1846d-3p	LOC_Os04g47990.2	156	-24.07	156	-24.07	888872	20	2175	1980
miR1846d-3p	LOC_Os04g48080.1	166	-26.59	166	-26.59	888884	20	3132	659
miR1846d-3p	LOC_Os04g48210.1	151	-25.56	151	-25.56	888895	20	1937	1649
miR1846d-3p	LOC_Os04g48210.2	151	-25.56	151	-25.56	888896	20	2026	1738
miR1846d-3p	LOC_Os04g48250.1	153	-29.97	153	-29.97	888901	20	468	339
miR1846d-3p	LOC_Os04g48416.1	155	-27.1	155	-27.1	888922	20	2331	827
miR1846d-3p	LOC_Os04g49090.1	142	-22.55	142	-22.55	888999	20	3288	1970
miR1846d-3p	LOC_Os04g49210.1	148	-20.16	148	-20.16	889011	20	1891	1425
miR1846d-3p	LOC_Os04g49530.1	157	-22.72	157	-22.72	889046	20	2175	124
miR1846d-3p	LOC_Os04g49748.1	145	-22.53	145	-22.53	889073	20	1494	317
miR1846d-3p	LOC_Os04g49790.1	146	-20.82	146	-20.82	889078	20	3338	526

miR1846d-3p	LOC_Os04g49850.1	175	-34.44	175	-34.44	889084	20	508	129
miR1846d-3p	LOC_Os04g50150.1	166	-26.29	166	-26.29	889117	20	2685	500
miR1846d-3p	LOC_Os04g50168.1	166	-26.59	166	-26.59	889120	20	1572	470
miR1846d-3p	LOC_Os04g50820.1	149	-20.48	149	-20.48	889150	20	2607	1825
miR1846d-3p	LOC_Os04g50930.1	142	-22.32	142	-22.32	889162	20	2047	1239
miR1846d-3p	LOC_Os04g51100.1	155	-23.65	155	-23.65	889182	20	1596	1225
miR1846d-3p	LOC_Os04g51110.1	143	-23.98	143	-23.98	889183	20	2570	624
miR1846d-3p	LOC_Os04g51110.2	143	-23.98	143	-23.98	889184	20	2342	624
miR1846d-3p	LOC_Os04g51410.1	150	-22.44	150	-22.44	889228	20	829	186
miR1846d-3p	LOC_Os04g51440.1	144	-27.33	144	-27.33	889231	20	3302	3082
miR1846d-3p	LOC_Os04g51440.2	144	-27.33	144	-27.33	889232	20	3384	3082
miR1846d-3p	LOC_Os04g51450.1	154	-21.82	154	-21.82	889233	20	1566	716
miR1846d-3p	LOC_Os04g51530.1	166	-27.37	166	-27.37	889242	20	2850	659
miR1846d-3p	LOC_Os04g51570.1	153	-25.33	153	-25.33	889246	20	800	146
miR1846d-3p	LOC_Os04g51620.1	149	-20.44	149	-20.44	889253	20	486	132
miR1846d-3p	LOC_Os04g51720.1	166	-27.37	166	-27.37	889264	20	3114	659
miR1846d-3p	LOC_Os04g51809.1	320	-55.48	167	-29.86	889280	20	2132	331 683
miR1846d-3p	LOC_Os04g51809.2	320	-55.48	167	-29.86	889281	20	1785	331 683
miR1846d-3p	LOC_Os04g51809.3	320	-55.48	167	-29.86	889282	20	1658	331 683
miR1846d-3p	LOC_Os04g51990.1	149	-23.17	149	-23.17	889311	20	1374	866
miR1846d-3p	LOC_Os04g52140.1	152	-24.05	152	-24.05	889326	20	2866	1154
miR1846d-3p	LOC_Os04g52190.1	164	-28	164	-28	889333	20	2824	1803
miR1846d-3p	LOC_Os04g52370.1	157	-28.23	157	-28.23	889351	20	2073	1650
miR1846d-3p	LOC_Os04g52370.2	157	-28.23	157	-28.23	889352	20	2067	1644
miR1846d-3p	LOC_Os04g52420.1	148	-25.71	148	-25.71	889361	20	2067	411
miR1846d-3p	LOC_Os04g52540.1	151	-26.04	151	-26.04	889377	20	3478	1153
miR1846d-3p	LOC_Os04g52950.1	157	-29.6	157	-29.6	889432	20	632	439
miR1846d-3p	LOC_Os04g53130.1	148	-21.72	148	-21.72	889448	20	1068	849
miR1846d-3p	LOC_Os04g53240.1	147	-25.82	147	-25.82	889465	20	826	696
miR1846d-3p	LOC_Os04g53240.2	145	-22.38	145	-22.38	889466	20	718	589
miR1846d-3p	LOC_Os04g53240.3	156	-31.67	156	-31.67	889467	20	701	569
miR1846d-3p	LOC_Os04g53240.4	147	-25.82	147	-25.82	889468	20	932	802

miR1846d-3p	LOC_Os04g53270.1	157	-24.09	157	-24.09	889471	20	2499	338
miR1846d-3p	LOC_Os04g53280.1	145	-22.39	145	-22.39	889472	20	5967	4356
miR1846d-3p	LOC_Os04g53320.1	149	-20.01	149	-20.01	889476	20	797	624
miR1846d-3p	LOC_Os04g53670.1	152	-22.15	152	-22.15	889524	20	4138	4051
miR1846d-3p	LOC_Os04g53760.1	156	-30.05	156	-30.05	889536	20	3200	2886
miR1846d-3p	LOC_Os04g53760.2	156	-30.05	156	-30.05	889537	20	3125	2811
miR1846d-3p	LOC_Os04g53840.1	144	-23.13	144	-23.13	889546	20	480	192
miR1846d-3p	LOC_Os04g53870.1	144	-22.5	144	-22.5	889549	20	5784	2349
miR1846d-3p	LOC_Os04g53880.1	172	-34.12	172	-34.12	889550	20	3081	1828
miR1846d-3p	LOC_Os04g53930.1	152	-24.42	152	-24.42	889555	20	1762	43
miR1846d-3p	LOC_Os04g53990.1	150	-22.24	150	-22.24	889564	20	1976	920
miR1846d-3p	LOC_Os04g54150.1	151	-24.69	151	-24.69	889583	20	2343	2043
miR1846d-3p	LOC_Os04g54260.1	156	-23.52	156	-23.52	889597	20	6087	507
miR1846d-3p	LOC_Os04g54270.1	144	-24.33	144	-24.33	889598	20	3144	1829
miR1846d-3p	LOC_Os04g54390.1	151	-24.16	151	-24.16	889611	20	1285	272
miR1846d-3p	LOC_Os04g54440.1	145	-21.84	145	-21.84	889616	20	3418	3290
miR1846d-3p	LOC_Os04g54440.2	145	-21.84	145	-21.84	889617	20	3280	3152
miR1846d-3p	LOC_Os04g54440.3	145	-21.84	145	-21.84	889618	20	1676	1548
miR1846d-3p	LOC_Os04g54600.1	152	-27.05	152	-27.05	889637	20	1142	380
miR1846d-3p	LOC_Os04g54690.1	166	-27.37	166	-27.37	889644	20	3150	545
miR1846d-3p	LOC_Os04g54800.1	149	-21.89	149	-21.89	889658	20	1338	1201
miR1846d-3p	LOC_Os04g54800.2	149	-21.89	149	-21.89	889659	20	1411	1274
miR1846d-3p	LOC_Os04g54800.3	149	-21.89	149	-21.89	889660	20	1262	1125
miR1846d-3p	LOC_Os04g55210.1	161	-30.09	161	-30.09	889705	20	2805	385
miR1846d-3p	LOC_Os04g55290.1	164	-30.27	164	-30.27	889713	20	1300	750
miR1846d-3p	LOC_Os04g55290.3	164	-30.27	164	-30.27	889715	20	959	829
miR1846d-3p	LOC_Os04g55290.5	164	-30.27	164	-30.27	889717	20	1295	750
miR1846d-3p	LOC_Os04g55360.1	156	-28.25	156	-28.25	889724	20	2319	1954
miR1846d-3p	LOC_Os04g55450.1	144	-23.56	144	-23.56	889733	20	4794	4305
miR1846d-3p	LOC_Os04g55530.1	147	-21.62	147	-21.62	889741	20	5655	2417
miR1846d-3p	LOC_Os04g55540.1	172	-34.12	172	-34.12	889742	20	3174	1837
miR1846d-3p	LOC_Os04g55820.1	147	-21.62	147	-21.62	889775	20	5262	2546

miR1846d-3p	LOC_Os04g55830.1	172	-35.42	172	-35.42	889776	20	3165	1831
miR1846d-3p	LOC_Os04g55870.1	166	-26.29	166	-26.29	889781	20	3135	659
miR1846d-3p	LOC_Os04g56060.1	162	-28.03	162	-28.03	889803	20	1669	851
miR1846d-3p	LOC_Os04g56090.1	153	-26.46	153	-26.46	889807	20	2445	1180
miR1846d-3p	LOC_Os04g56920.1	150	-30.3	150	-30.3	889911	20	1926	474
miR1846d-3p	LOC_Os04g57100.1	151	-24.69	151	-24.69	889936	20	2370	2073
miR1846d-3p	LOC_Os04g57180.1	152	-28.62	152	-28.62	889947	20	2727	922
miR1846d-3p	LOC_Os04g57230.1	152	-24.28	152	-24.28	889961	20	1724	986
miR1846d-3p	LOC_Os04g57230.2	152	-24.28	152	-24.28	889962	20	1654	924
miR1846d-3p	LOC_Os04g57290.1	168	-32.62	168	-32.62	889968	20	1888	695
miR1846d-3p	LOC_Os04g57450.1	162	-24.8	162	-24.8	889988	20	3339	659
miR1846d-3p	LOC_Os04g57550.1	144	-24.63	144	-24.63	890005	20	2207	1035
miR1846d-3p	LOC_Os04g57680.1	150	-22.94	150	-22.94	890022	20	1440	326
miR1846d-3p	LOC_Os04g57680.2	150	-22.94	150	-22.94	890023	20	1887	300
miR1846d-3p	LOC_Os04g57700.1	150	-24.01	150	-24.01	890025	20	2039	1889
miR1846d-3p	LOC_Os04g57700.4	150	-24.01	150	-24.01	890028	20	2076	1926
miR1846d-3p	LOC_Os04g57770.1	148	-21.42	148	-21.42	890036	20	1753	450
miR1846d-3p	LOC_Os04g57880.1	148	-23.66	148	-23.66	890053	20	2456	1489
miR1846d-3p	LOC_Os04g57890.1	148	-23.66	148	-23.66	890054	20	3552	377
miR1846d-3p	LOC_Os04g57910.1	156	-25.23	156	-25.23	890056	20	555	418
miR1846d-3p	LOC_Os04g57940.1	151	-23.83	151	-23.83	890059	20	2109	1204
miR1846d-3p	LOC_Os04g58180.1	145	-21.06	145	-21.06	890085	20	3028	785
miR1846d-3p	LOC_Os04g58280.1	148	-23.91	148	-23.91	890098	20	2620	1107
miR1846d-3p	LOC_Os04g58280.2	148	-23.91	148	-23.91	890099	20	2409	896
miR1846d-3p	LOC_Os04g58520.1	150	-28.23	150	-28.23	890126	20	594	200
miR1846d-3p	LOC_Os04g58530.1	150	-28.13	150	-28.13	890127	20	652	246
miR1846d-3p	LOC_Os04g58720.1	145	-22.18	145	-22.18	890157	20	3860	2796
miR1846d-3p	LOC_Os04g58980.1	152	-29.79	152	-29.79	890189	20	2649	911
miR1846d-3p	LOC_Os04g59100.1	297	-42.37	150	-21.62	890203	20	5130	168 2207
miR1846d-3p	LOC_Os04g59170.1	152	-20.54	152	-20.54	890211	20	2466	397
miR1846d-3p	LOC_Os04g59180.1	145	-20.08	145	-20.08	890212	20	5457	3825
miR1846d-3p	LOC_Os04g59220.1	158	-28.91	158	-28.91	890216	20	1398	507

miR1846d-3p	LOC_Os05g01060.1	156	-30.93	156	-30.93	890296	20	2774	2436
miR1846d-3p	LOC_Os05g01060.2	156	-30.93	156	-30.93	890297	20	2399	2061
miR1846d-3p	LOC_Os05g01130.1	146	-20.01	146	-20.01	890306	20	3801	17
miR1846d-3p	LOC_Os05g01150.1	148	-23.14	148	-23.14	890308	20	837	769
miR1846d-3p	LOC_Os05g01200.1	165	-27.35	165	-27.35	890313	20	951	744
miR1846d-3p	LOC_Os05g01400.1	297	-42.37	150	-21.62	890343	20	5607	468 2294
miR1846d-3p	LOC_Os05g01660.1	162	-24.8	162	-24.8	890384	20	2553	14
miR1846d-3p	LOC_Os05g02110.1	148	-25.18	148	-25.18	890441	20	1782	846
miR1846d-3p	LOC_Os05g02140.1	165	-30.17	165	-30.17	890444	20	2024	1314
miR1846d-3p	LOC_Os05g02280.1	140	-20.04	140	-20.04	890465	20	1189	553
miR1846d-3p	LOC_Os05g02550.1	145	-20.04	145	-20.04	890498	20	927	126
miR1846d-3p	LOC_Os05g02560.1	160	-31.18	160	-31.18	890499	20	1398	92
miR1846d-3p	LOC_Os05g02700.1	151	-23.2	151	-23.2	890515	20	1087	225
miR1846d-3p	LOC_Os05g02740.1	157	-26.42	157	-26.42	890519	20	586	78
miR1846d-3p	LOC_Os05g02920.1	156	-29.28	156	-29.28	890538	20	582	323
miR1846d-3p	LOC_Os05g02950.1	305	-47.07	158	-25.45	890541	20	4116	507 1406
miR1846d-3p	LOC_Os05g02980.1	145	-23.79	145	-23.79	890544	20	1247	654
miR1846d-3p	LOC_Os05g03174.1	153	-25.98	153	-25.98	890571	20	2286	852
miR1846d-3p	LOC_Os05g03174.2	153	-25.98	153	-25.98	890572	20	2015	581
miR1846d-3p	LOC_Os05g03430.1	156	-28.54	156	-28.54	890601	20	3386	105
miR1846d-3p	LOC_Os05g03430.2	156	-28.54	156	-28.54	890602	20	3385	105
miR1846d-3p	LOC_Os05g03500.1	320	-56.84	164	-31.13	890611	20	1092	852 903
miR1846d-3p	LOC_Os05g03560.1	148	-27.16	148	-27.16	890619	20	327	227
miR1846d-3p	LOC_Os05g03710.1	166	-26.59	166	-26.59	890635	20	2811	659
miR1846d-3p	LOC_Os05g03920.1	155	-26.88	155	-26.88	890658	20	2323	476
miR1846d-3p	LOC_Os05g04030.1	166	-25.39	166	-25.39	890668	20	2748	659
miR1846d-3p	LOC_Os05g04290.1	166	-26.59	166	-26.59	890698	20	2166	659
miR1846d-3p	LOC_Os05g04730.1	146	-20.01	146	-20.01	890749	20	3963	17
miR1846d-3p	LOC_Os05g04950.1	141	-20.11	141	-20.11	890770	20	3248	2308
miR1846d-3p	LOC_Os05g04950.2	141	-20.11	141	-20.11	890771	20	3319	2308
miR1846d-3p	LOC_Os05g04970.1	157	-23.73	157	-23.73	890773	20	2436	338
miR1846d-3p	LOC_Os05g05010.1	148	-20.6	148	-20.6	890777	20	2496	1252

miR1846d-3p	LOC_Os05g05346.1	145	-20.38	145	-20.38	890817	20	2900	442
miR1846d-3p	LOC_Os05g05420.1	151	-21.79	151	-21.79	890825	20	4935	3666
miR1846d-3p	LOC_Os05g05510.1	147	-22.22	147	-22.22	890836	20	1362	210
miR1846d-3p	LOC_Os05g05590.1	145	-20.86	145	-20.86	890845	20	2135	1898
miR1846d-3p	LOC_Os05g05950.1	143	-20.78	143	-20.78	890890	20	4276	3754
miR1846d-3p	LOC_Os05g06060.1	314	-59.07	170	-30.4	890902	20	1131	96 1008
miR1846d-3p	LOC_Os05g06100.1	152	-30.8	152	-30.8	890906	20	1134	1020
miR1846d-3p	LOC_Os05g06140.1	153	-22.71	153	-22.71	890910	20	1948	1402
miR1846d-3p	LOC_Os05g06250.1	145	-20.58	145	-20.58	890921	20	1113	335
miR1846d-3p	LOC_Os05g06280.2	157	-29.29	157	-29.29	890926	20	3599	49
miR1846d-3p	LOC_Os05g06390.1	143	-22.19	143	-22.19	890939	20	5811	3251
miR1846d-3p	LOC_Os05g06540.1	144	-20.33	144	-20.33	890958	20	1687	157
miR1846d-3p	LOC_Os05g06540.2	144	-20.33	144	-20.33	890959	20	1705	157
miR1846d-3p	LOC_Os05g06630.1	144	-20.33	144	-20.33	890968	20	2462	143
miR1846d-3p	LOC_Os05g06814.1	157	-23.94	157	-23.94	890994	20	1790	1369
miR1846d-3p	LOC_Os05g06814.2	157	-23.94	157	-23.94	890995	20	1856	1435
miR1846d-3p	LOC_Os05g06860.1	166	-26.29	166	-26.29	890999	20	3135	659
miR1846d-3p	LOC_Os05g07210.1	142	-21.4	142	-21.4	891042	20	1498	574
miR1846d-3p	LOC_Os05g07330.1	140	-21.04	140	-21.04	891059	20	3282	1522
miR1846d-3p	LOC_Os05g07590.1	155	-24.46	155	-24.46	891079	20	777	211
miR1846d-3p	LOC_Os05g07670.1	152	-26.09	152	-26.09	891087	20	1892	1654
miR1846d-3p	LOC_Os05g07740.1	152	-28.36	152	-28.36	891101	20	3965	3031
miR1846d-3p	LOC_Os05g07950.1	152	-24.71	152	-24.71	891127	20	504	434
miR1846d-3p	LOC_Os05g07960.1	152	-24.71	152	-24.71	891128	20	1826	1683
miR1846d-3p	LOC_Os05g08000.1	152	-23.93	152	-23.93	891132	20	771	724
miR1846d-3p	LOC_Os05g08010.1	152	-24.71	152	-24.71	891133	20	1545	1360
miR1846d-3p	LOC_Os05g08200.1	156	-28.42	156	-28.42	891155	20	1425	1073
miR1846d-3p	LOC_Os05g08250.1	145	-20.82	145	-20.82	891160	20	1506	665
miR1846d-3p	LOC_Os05g08450.1	151	-22.51	151	-22.51	891185	20	1678	43
miR1846d-3p	LOC_Os05g08560.1	156	-26.79	156	-26.79	891196	20	2078	1704
miR1846d-3p	LOC_Os05g08694.1	140	-20.04	140	-20.04	891217	20	4824	3453
miR1846d-3p	LOC_Os05g08860.1	156	-26.1	156	-26.1	891237	20	837	466

miR1846d-3p	LOC_Os05g08990.1	145	-23.55	145	-23.55	891254	20	2841	2470
miR1846d-3p	LOC_Os05g09030.1	166	-26.29	166	-26.29	891260	20	3144	659
miR1846d-3p	LOC_Os05g09070.1	166	-26.59	166	-26.59	891264	20	3141	659
miR1846d-3p	LOC_Os05g10250.1	301	-45.61	154	-23.99	891349	20	5391	573 2579
miR1846d-3p	LOC_Os05g10290.1	145	-21.19	145	-21.19	891352	20	351	275
miR1846d-3p	LOC_Os05g10730.1	148	-24.06	148	-24.06	891403	20	6041	232
miR1846d-3p	LOC_Os05g10730.2	148	-24.06	148	-24.06	891404	20	6094	285
miR1846d-3p	LOC_Os05g10730.3	148	-24.06	148	-24.06	891405	20	6127	232
miR1846d-3p	LOC_Os05g10870.1	147	-21.62	147	-21.62	891424	20	5889	2492
miR1846d-3p	LOC_Os05g11005.1	304	-48.11	154	-25.45	891438	20	694	260 504
miR1846d-3p	LOC_Os05g11040.1	155	-23.91	155	-23.91	891441	20	3840	339
miR1846d-3p	LOC_Os05g11500.1	153	-22.66	153	-22.66	891488	20	774	92
miR1846d-3p	LOC_Os05g12020.1	144	-26.25	144	-26.25	891543	20	216	128
miR1846d-3p	LOC_Os05g12300.1	156	-25.64	156	-25.64	891577	20	2481	562
miR1846d-3p	LOC_Os05g12300.2	156	-25.64	156	-25.64	891578	20	3285	562
miR1846d-3p	LOC_Os05g12300.3	156	-25.64	156	-25.64	891579	20	3184	562
miR1846d-3p	LOC_Os05g12310.1	156	-23.89	156	-23.89	891580	20	1821	1325
miR1846d-3p	LOC_Os05g12550.1	148	-23.24	148	-23.24	891603	20	1185	260
miR1846d-3p	LOC_Os05g12570.1	148	-21.05	148	-21.05	891605	20	3315	3180
miR1846d-3p	LOC_Os05g12610.1	145	-21.49	145	-21.49	891609	20	1128	366
miR1846d-3p	LOC_Os05g12620.1	150	-20.92	150	-20.92	891610	20	3336	2922
miR1846d-3p	LOC_Os05g12700.1	149	-23.43	149	-23.43	891621	20	522	423
miR1846d-3p	LOC_Os05g13410.1	145	-20.32	145	-20.32	891644	20	927	27
miR1846d-3p	LOC_Os05g13480.1	155	-30.75	155	-30.75	891652	20	573	334
miR1846d-3p	LOC_Os05g13740.1	140	-20.04	140	-20.04	891678	20	3126	2490
miR1846d-3p	LOC_Os05g13780.1	148	-25.96	148	-25.96	891682	20	4224	3587
miR1846d-3p	LOC_Os05g13790.1	148	-25.96	148	-25.96	891683	20	2212	1755
miR1846d-3p	LOC_Os05g13800.1	142	-23.38	142	-23.38	891684	20	2334	2004
miR1846d-3p	LOC_Os05g13810.2	151	-23.16	151	-23.16	891686	20	664	221
miR1846d-3p	LOC_Os05g14340.1	147	-21.62	147	-21.62	891746	20	6084	2546
miR1846d-3p	LOC_Os05g14350.1	172	-35.42	172	-35.42	891747	20	3207	1873
miR1846d-3p	LOC_Os05g14470.1	148	-21.64	148	-21.64	891759	20	372	73

miR1846d-3p	LOC_Os05g14640.1	159	-28.39	159	-28.39	891773	20	1686	950
miR1846d-3p	LOC_Os05g15120.1	150	-22.28	150	-22.28	891821	20	312	152
miR1846d-3p	LOC_Os05g15160.1	149	-27.36	149	-27.36	891827	20	1679	232
miR1846d-3p	LOC_Os05g15320.1	151	-23.12	151	-23.12	891843	20	1878	621
miR1846d-3p	LOC_Os05g15680.1	156	-24.4	156	-24.4	891884	20	309	80
miR1846d-3p	LOC_Os05g15850.1	143	-20.88	143	-20.88	891899	20	1149	762
miR1846d-3p	LOC_Os05g15980.1	152	-20.54	152	-20.54	891914	20	2487	397
miR1846d-3p	LOC_Os05g15990.1	145	-20.08	145	-20.08	891915	20	5940	4308
miR1846d-3p	LOC_Os05g16120.1	295	-43.06	148	-22.76	891928	20	6084	507 2546
miR1846d-3p	LOC_Os05g16315.1	159	-29.97	159	-29.97	891945	20	522	181
miR1846d-3p	LOC_Os05g16370.1	146	-21.24	146	-21.24	891951	20	3633	3398
miR1846d-3p	LOC_Os05g16664.1	145	-21.86	145	-21.86	891984	20	946	469
miR1846d-3p	LOC_Os05g16700.1	145	-20.08	145	-20.08	891988	20	1785	174
miR1846d-3p	LOC_Os05g17020.1	153	-22.58	153	-22.58	892020	20	477	329
miR1846d-3p	LOC_Os05g17040.1	141	-23.08	141	-23.08	892022	20	1893	1757
miR1846d-3p	LOC_Os05g17180.1	166	-27.37	166	-27.37	892036	20	3054	470
miR1846d-3p	LOC_Os05g17540.1	140	-20.04	140	-20.04	892071	20	4665	4029
miR1846d-3p	LOC_Os05g18274.1	148	-21.32	148	-21.32	892146	20	1819	90
miR1846d-3p	LOC_Os05g18700.1	165	-28.33	165	-28.33	892191	20	225	201
miR1846d-3p	LOC_Os05g18840.1	156	-23.89	156	-23.89	892208	20	1209	1044
miR1846d-3p	LOC_Os05g18900.1	145	-26.82	145	-26.82	892214	20	2706	1541
miR1846d-3p	LOC_Os05g19000.1	168	-31.01	168	-31.01	892224	20	1770	167
miR1846d-3p	LOC_Os05g19040.1	165	-32.41	165	-32.41	892229	20	1254	691
miR1846d-3p	LOC_Os05g19050.1	144	-22.39	144	-22.39	892230	20	4152	3661
miR1846d-3p	LOC_Os05g19160.1	166	-26.29	166	-26.29	892240	20	2982	659
miR1846d-3p	LOC_Os05g19220.1	148	-21.47	148	-21.47	892246	20	4695	128
miR1846d-3p	LOC_Os05g19550.1	145	-23.22	145	-23.22	892286	20	3792	3153
miR1846d-3p	LOC_Os05g19560.1	166	-26.59	166	-26.59	892287	20	5832	353
miR1846d-3p	LOC_Os05g20330.1	305	-45.5	158	-23.88	892366	20	5358	21 2069
miR1846d-3p	LOC_Os05g20390.1	166	-26.29	166	-26.29	892372	20	3144	659
miR1846d-3p	LOC_Os05g20470.1	154	-27.54	154	-27.54	892380	20	576	451
miR1846d-3p	LOC_Os05g20710.1	144	-22.11	144	-22.11	892404	20	2925	2718

miR1846d-3p	LOC_Os05g20850.1	144	-21.65	144	-21.65	892418	20	1743	111
miR1846d-3p	LOC_Os05g21080.1	156	-28.42	156	-28.42	892447	20	1230	878
miR1846d-3p	LOC_Os05g21090.1	142	-21.9	142	-21.9	892448	20	1419	1193
miR1846d-3p	LOC_Os05g21100.1	305	-47.07	158	-25.45	892449	20	5910	507 2546
miR1846d-3p	LOC_Os05g21120.1	144	-21.8	144	-21.8	892451	20	5943	4752
miR1846d-3p	LOC_Os05g22580.1	152	-23.71	152	-23.71	892499	20	987	332
miR1846d-3p	LOC_Os05g22650.1	147	-25.11	147	-25.11	892507	20	3300	1399
miR1846d-3p	LOC_Os05g22870.1	143	-20.22	143	-20.22	892532	20	2499	2288
miR1846d-3p	LOC_Os05g22894.1	155	-29.07	155	-29.07	892534	20	2290	137
miR1846d-3p	LOC_Os05g23150.1	295	-41.92	148	-21.62	892562	20	5721	507 2546
miR1846d-3p	LOC_Os05g23190.1	172	-34.12	172	-34.12	892566	20	3222	1885
miR1846d-3p	LOC_Os05g23200.1	161	-27.79	161	-27.79	892568	20	1044	826
miR1846d-3p	LOC_Os05g23210.1	146	-20.21	146	-20.21	892569	20	4572	2872
miR1846d-3p	LOC_Os05g23250.1	146	-21.31	146	-21.31	892573	20	3921	2696
miR1846d-3p	LOC_Os05g23320.2	147	-22.14	147	-22.14	892582	20	3066	347
miR1846d-3p	LOC_Os05g23360.1	166	-26.29	166	-26.29	892586	20	1509	659
miR1846d-3p	LOC_Os05g23450.1	149	-21.2	149	-21.2	892597	20	2597	1579
miR1846d-3p	LOC_Os05g23450.2	149	-21.2	149	-21.2	892598	20	2456	1579
miR1846d-3p	LOC_Os05g23530.1	151	-23.64	151	-23.64	892607	20	2639	608
miR1846d-3p	LOC_Os05g23530.2	151	-23.64	151	-23.64	892608	20	2661	542
miR1846d-3p	LOC_Os05g23530.3	151	-23.64	151	-23.64	892609	20	1517	608
miR1846d-3p	LOC_Os05g23530.4	151	-23.64	151	-23.64	892610	20	2485	608
miR1846d-3p	LOC_Os05g23590.1	152	-22.6	152	-22.6	892615	20	2034	1860
miR1846d-3p	LOC_Os05g24090.1	307	-50.25	162	-28.09	892670	20	705	145 114
miR1846d-3p	LOC_Os05g24360.1	149	-20.75	149	-20.75	892697	20	843	272
miR1846d-3p	LOC_Os05g24460.1	147	-21.62	147	-21.62	892706	20	5907	2546
miR1846d-3p	LOC_Os05g24470.1	172	-34.12	172	-34.12	892707	20	3165	1828
miR1846d-3p	LOC_Os05g24490.1	157	-24.61	157	-24.61	892709	20	624	538
miR1846d-3p	LOC_Os05g25280.1	151	-24.69	151	-24.69	892798	20	2010	1896
miR1846d-3p	LOC_Os05g25360.1	162	-30.07	162	-30.07	892807	20	474	133
miR1846d-3p	LOC_Os05g25620.1	159	-26.65	159	-26.65	892835	20	643	245
miR1846d-3p	LOC_Os05g25640.1	145	-20.46	145	-20.46	892837	20	1839	1226

miR1846d-3p	LOC_Os05g25670.1	308	-57.37	163	-33.49	892840	20	300	261 92
miR1846d-3p	LOC_Os05g25840.1	144	-21.23	144	-21.23	892856	20	2598	2491
miR1846d-3p	LOC_Os05g25840.3	144	-21.23	144	-21.23	892857	20	2508	2401
miR1846d-3p	LOC_Os05g25900.1	150	-21.35	150	-21.35	892865	20	225	21
miR1846d-3p	LOC_Os05g25960.1	161	-29.31	161	-29.31	892872	20	1704	431
miR1846d-3p	LOC_Os05g26160.1	140	-20.46	140	-20.46	892894	20	2760	25
miR1846d-3p	LOC_Os05g26194.1	156	-25.58	156	-25.58	892898	20	607	358
miR1846d-3p	LOC_Os05g26200.1	151	-20.53	151	-20.53	892899	20	837	754
miR1846d-3p	LOC_Os05g26290.1	148	-20.51	148	-20.51	892910	20	2139	944
miR1846d-3p	LOC_Os05g26570.1	305	-45.73	158	-25.45	892937	20	3516	507 2540
miR1846d-3p	LOC_Os05g26850.1	160	-23.74	160	-23.74	892964	20	300	242
miR1846d-3p	LOC_Os05g26990.2	144	-22.94	144	-22.94	892975	20	3109	2827
miR1846d-3p	LOC_Os05g26990.3	144	-22.94	144	-22.94	892976	20	2219	1937
miR1846d-3p	LOC_Os05g26990.4	144	-22.94	144	-22.94	892977	20	1860	1578
miR1846d-3p	LOC_Os05g27540.1	153	-20.72	153	-20.72	893030	20	894	335
miR1846d-3p	LOC_Os05g27810.1	313	-49.77	157	-25.61	893056	20	722	469 1
miR1846d-3p	LOC_Os05g28390.1	153	-24.63	153	-24.63	893124	20	483	229
miR1846d-3p	LOC_Os05g28430.1	153	-22.46	153	-22.46	893127	20	2427	505
miR1846d-3p	LOC_Os05g28670.1	151	-24.69	151	-24.69	893153	20	1380	1080
miR1846d-3p	LOC_Os05g28770.1	159	-23.52	159	-23.52	893161	20	1188	954
miR1846d-3p	LOC_Os05g28900.1	147	-21.62	147	-21.62	893174	20	5067	1862
miR1846d-3p	LOC_Os05g29040.1	145	-20.36	145	-20.36	893197	20	453	181
miR1846d-3p	LOC_Os05g30030.1	166	-33.79	166	-33.79	893257	20	775	515
miR1846d-3p	LOC_Os05g30060.1	144	-21.22	144	-21.22	893260	20	2460	37
miR1846d-3p	LOC_Os05g30070.1	156	-23.89	156	-23.89	893261	20	4530	4475
miR1846d-3p	LOC_Os05g30220.1	148	-24.37	148	-24.37	893277	20	4913	4760
miR1846d-3p	LOC_Os05g30250.1	157	-24.03	157	-24.03	893280	20	2257	114
miR1846d-3p	LOC_Os05g30250.2	157	-24.03	157	-24.03	893281	20	2185	114
miR1846d-3p	LOC_Os05g30260.1	152	-20.54	152	-20.54	893282	20	2466	397
miR1846d-3p	LOC_Os05g30270.1	145	-20.08	145	-20.08	893283	20	5205	4356
miR1846d-3p	LOC_Os05g30390.1	152	-23.86	152	-23.86	893297	20	1573	250
miR1846d-3p	LOC_Os05g30550.1	166	-26.59	166	-26.59	893317	20	3027	659

miR1846d-3p	LOC_Os05g30760.1	146	-20.04	146	-20.04	893345	20	1079	424
miR1846d-3p	LOC_Os05g30770.1	302	-41.94	157	-21.3	893346	20	864	637 239
miR1846d-3p	LOC_Os05g31000.1	155	-26.07	155	-26.07	893376	20	1131	483
miR1846d-3p	LOC_Os05g31320.1	142	-20.95	142	-20.95	893408	20	720	478
miR1846d-3p	LOC_Os05g31430.1	152	-20.54	152	-20.54	893419	20	2466	397
miR1846d-3p	LOC_Os05g31440.1	145	-20.08	145	-20.08	893420	20	5961	4335
miR1846d-3p	LOC_Os05g31525.1	156	-24.61	156	-24.61	893430	20	546	517
miR1846d-3p	LOC_Os05g31530.1	142	-21.24	142	-21.24	893431	20	5545	146
miR1846d-3p	LOC_Os05g31744.1	145	-23.47	145	-23.47	893452	20	898	705
miR1846d-3p	LOC_Os05g31785.1	150	-24.69	150	-24.69	893458	20	588	405
miR1846d-3p	LOC_Os05g32030.1	146	-22.58	146	-22.58	893480	20	2013	259
miR1846d-3p	LOC_Os05g32140.1	148	-20.15	148	-20.15	893494	20	2290	913
miR1846d-3p	LOC_Os05g32220.1	157	-25.71	157	-25.71	893502	20	1591	792
miR1846d-3p	LOC_Os05g32460.1	145	-22.09	145	-22.09	893529	20	939	419
miR1846d-3p	LOC_Os05g32544.1	145	-20.98	145	-20.98	893539	20	2638	2370
miR1846d-3p	LOC_Os05g32544.2	145	-20.98	145	-20.98	893540	20	2558	2290
miR1846d-3p	LOC_Os05g32960.1	167	-27.27	167	-27.27	893585	20	2541	2391
miR1846d-3p	LOC_Os05g33070.1	151	-25.73	151	-25.73	893600	20	606	32
miR1846d-3p	LOC_Os05g33080.1	164	-26.97	164	-26.97	893601	20	2049	731
miR1846d-3p	LOC_Os05g33190.1	153	-22.09	153	-22.09	893614	20	1396	82
miR1846d-3p	LOC_Os05g33280.1	151	-20.62	151	-20.62	893623	20	1415	1369
miR1846d-3p	LOC_Os05g33320.1	165	-31.67	165	-31.67	893627	20	912	246
miR1846d-3p	LOC_Os05g33760.1	297	-47.68	150	-24.43	893682	20	1758	1024 511
miR1846d-3p	LOC_Os05g33790.1	146	-20.01	146	-20.01	893685	20	3567	17
miR1846d-3p	LOC_Os05g33840.1	159	-27.8	159	-27.8	893690	20	2685	1291
miR1846d-3p	LOC_Os05g33980.1	147	-21.62	147	-21.62	893704	20	5592	2246
miR1846d-3p	LOC_Os05g34080.1	162	-24.8	162	-24.8	893715	20	2592	659
miR1846d-3p	LOC_Os05g34130.1	316	-53.68	160	-26.93	893720	20	1930	1639 1580
miR1846d-3p	LOC_Os05g34325.1	149	-21.6	149	-21.6	893745	20	1914	318
miR1846d-3p	LOC_Os05g35444.1	150	-22.18	150	-22.18	893880	20	1708	328
miR1846d-3p	LOC_Os05g35560.1	153	-22.11	153	-22.11	893894	20	333	257
miR1846d-3p	LOC_Os05g35780.1	290	-44.67	145	-23.42	893917	20	489	328 349

miR1846d-3p	LOC_Os05g35950.1	150	-20.98	150	-20.98	893933	20	732	669
miR1846d-3p	LOC_Os05g36170.1	147	-21.62	147	-21.62	893963	20	6018	2546
miR1846d-3p	LOC_Os05g36190.1	159	-28.7	159	-28.7	893967	20	2345	2039
miR1846d-3p	LOC_Os05g36200.1	146	-20.01	146	-20.01	893968	20	2228	17
miR1846d-3p	LOC_Os05g36350.1	164	-29.93	164	-29.93	893986	20	1823	484
miR1846d-3p	LOC_Os05g36980.1	151	-23.2	151	-23.2	894002	20	960	372
miR1846d-3p	LOC_Os05g37250.1	143	-23.05	143	-23.05	894042	20	2451	1409
miR1846d-3p	LOC_Os05g37270.1	147	-21.62	147	-21.62	894044	20	6084	2546
miR1846d-3p	LOC_Os05g37280.1	172	-34.12	172	-34.12	894045	20	3093	1756
miR1846d-3p	LOC_Os05g37300.1	172	-34.12	172	-34.12	894047	20	3210	1873
miR1846d-3p	LOC_Os05g37520.1	154	-27.62	154	-27.62	894072	20	2127	252
miR1846d-3p	LOC_Os05g37520.2	154	-27.62	154	-27.62	894073	20	1999	252
miR1846d-3p	LOC_Os05g37780.1	149	-21.42	149	-21.42	894100	20	918	131
miR1846d-3p	LOC_Os05g37860.1	159	-29.97	159	-29.97	894108	20	609	529
miR1846d-3p	LOC_Os05g37940.1	159	-22.08	159	-22.08	894120	20	297	265
miR1846d-3p	LOC_Os05g38100.1	305	-47.07	158	-25.45	894141	20	5511	507 2434
miR1846d-3p	LOC_Os05g38130.1	148	-21.47	148	-21.47	894144	20	486	358
miR1846d-3p	LOC_Os05g38210.1	156	-25.82	156	-25.82	894155	20	1141	1024
miR1846d-3p	LOC_Os05g38280.1	165	-29.31	165	-29.31	894167	20	2017	930
miR1846d-3p	LOC_Os05g38410.1	156	-22.61	156	-22.61	894188	20	1716	1367
miR1846d-3p	LOC_Os05g38630.2	152	-24.99	152	-24.99	894219	20	1334	1141
miR1846d-3p	LOC_Os05g39130.1	148	-20.72	148	-20.72	894279	20	4260	3986
miR1846d-3p	LOC_Os05g39170.1	147	-21.68	147	-21.68	894285	20	1224	1185
miR1846d-3p	LOC_Os05g39180.1	150	-23.75	150	-23.75	894286	20	1728	773
miR1846d-3p	LOC_Os05g39600.1	152	-23.98	152	-23.98	894337	20	2937	2256
miR1846d-3p	LOC_Os05g39730.1	151	-25.69	151	-25.69	894351	20	2280	2092
miR1846d-3p	LOC_Os05g39930.1	155	-27.51	155	-27.51	894369	20	2633	380
miR1846d-3p	LOC_Os05g40070.1	169	-31.12	169	-31.12	894386	20	1174	811
miR1846d-3p	LOC_Os05g40220.1	148	-21.07	148	-21.07	894401	20	2525	2233
miR1846d-3p	LOC_Os05g40220.2	148	-21.07	148	-21.07	894402	20	2528	2236
miR1846d-3p	LOC_Os05g40420.1	318	-54.72	160	-28.29	894435	20	1992	1963 434
miR1846d-3p	LOC_Os05g40420.2	158	-26.43	158	-26.43	894436	20	1691	355

miR1846d-3p	LOC_Os05g40420.3	318	-54.72	160	-28.29	894437	20	1914	1882 355
miR1846d-3p	LOC_Os05g40860.1	166	-26.59	166	-26.59	894487	20	2853	470
miR1846d-3p	LOC_Os05g40950.1	150	-20.44	150	-20.44	894497	20	1148	756
miR1846d-3p	LOC_Os05g41000.1	149	-28.25	149	-28.25	894503	20	2761	599
miR1846d-3p	LOC_Os05g41100.1	157	-24.69	157	-24.69	894513	20	2292	1896
miR1846d-3p	LOC_Os05g41510.1	164	-29.66	164	-29.66	894562	20	6761	77
miR1846d-3p	LOC_Os05g41660.1	142	-21.38	142	-21.38	894580	20	1362	1026
miR1846d-3p	LOC_Os05g41690.1	166	-26.59	166	-26.59	894583	20	2727	659
miR1846d-3p	LOC_Os05g41930.1	144	-22.25	144	-22.25	894612	20	1658	1401
miR1846d-3p	LOC_Os05g41930.3	144	-22.25	144	-22.25	894614	20	1670	1401
miR1846d-3p	LOC_Os05g41950.1	146	-21.02	146	-21.02	894616	20	1861	1708
miR1846d-3p	LOC_Os05g42010.1	156	-32.35	156	-32.35	894623	20	2386	2109
miR1846d-3p	LOC_Os05g42010.2	156	-32.35	156	-32.35	894624	20	2471	2194
miR1846d-3p	LOC_Os05g42020.1	148	-20.13	148	-20.13	894625	20	2341	635
miR1846d-3p	LOC_Os05g42250.1	153	-27.61	153	-27.61	894646	20	2485	842
miR1846d-3p	LOC_Os05g42270.1	149	-25.65	149	-25.65	894648	20	1549	48
miR1846d-3p	LOC_Os05g42310.1	162	-24.8	162	-24.8	894653	20	3105	614
miR1846d-3p	LOC_Os05g43060.1	152	-27.59	152	-27.59	894684	20	5324	4612
miR1846d-3p	LOC_Os05g43240.1	163	-27.64	163	-27.64	894700	20	2436	47
miR1846d-3p	LOC_Os05g43240.2	163	-27.64	163	-27.64	894701	20	2531	53
miR1846d-3p	LOC_Os05g43410.1	151	-24.13	151	-24.13	894721	20	5832	2384
miR1846d-3p	LOC_Os05g43420.1	172	-34.12	172	-34.12	894722	20	3072	1739
miR1846d-3p	LOC_Os05g43730.1	320	-52.11	162	-28.91	894756	20	5295	1929 507
miR1846d-3p	LOC_Os05g43780.1	150	-22.77	150	-22.77	894761	20	1314	146
miR1846d-3p	LOC_Os05g43780.2	150	-22.77	150	-22.77	894762	20	2116	165
miR1846d-3p	LOC_Os05g43900.1	151	-21.46	151	-21.46	894780	20	267	81
miR1846d-3p	LOC_Os05g44000.1	151	-23.5	151	-23.5	894790	20	2589	26
miR1846d-3p	LOC_Os05g44010.1	152	-21.82	152	-21.82	894791	20	2184	1252
miR1846d-3p	LOC_Os05g44110.1	164	-30.26	164	-30.26	894804	20	951	858
miR1846d-3p	LOC_Os05g44130.1	162	-35.88	162	-35.88	894806	20	1483	1265
miR1846d-3p	LOC_Os05g44130.2	162	-35.88	162	-35.88	894807	20	1886	1668
miR1846d-3p	LOC_Os05g44130.3	162	-35.88	162	-35.88	894808	20	1676	1458

miR1846d-3p	LOC_Os05g44220.1	143	-20.85	143	-20.85	894818	20	507	257
miR1846d-3p	LOC_Os05g44290.1	150	-24.47	150	-24.47	894825	20	2736	413
miR1846d-3p	LOC_Os05g44290.3	150	-24.47	150	-24.47	894827	20	3154	485
miR1846d-3p	LOC_Os05g44290.4	150	-24.47	150	-24.47	894828	20	3778	881
miR1846d-3p	LOC_Os05g44650.1	152	-23	152	-23	894877	20	666	469
miR1846d-3p	LOC_Os05g44780.1	307	-49.46	154	-25.3	894890	20	1573	1381 1401
miR1846d-3p	LOC_Os05g44800.1	159	-31.66	159	-31.66	894893	20	631	190
miR1846d-3p	LOC_Os05g44800.2	159	-31.66	159	-31.66	894894	20	815	190
miR1846d-3p	LOC_Os05g44800.3	159	-31.66	159	-31.66	894895	20	689	190
miR1846d-3p	LOC_Os05g44890.1	172	-35.42	172	-35.42	894907	20	3243	1906
miR1846d-3p	LOC_Os05g44930.1	154	-21.62	154	-21.62	894914	20	3302	523
miR1846d-3p	LOC_Os05g44990.1	158	-24.15	158	-24.15	894920	20	2739	459
miR1846d-3p	LOC_Os05g45000.1	154	-21.62	154	-21.62	894921	20	816	453
miR1846d-3p	LOC_Os05g45180.1	147	-21.68	147	-21.68	894940	20	1689	693
miR1846d-3p	LOC_Os05g45430.1	148	-24.63	148	-24.63	894971	20	1842	398
miR1846d-3p	LOC_Os05g45520.1	152	-25.48	152	-25.48	894980	20	1146	1052
miR1846d-3p	LOC_Os05g45620.1	305	-47.07	158	-25.45	894990	20	4467	507 2546
miR1846d-3p	LOC_Os05g45630.1	151	-21.79	151	-21.79	894991	20	4575	3666
miR1846d-3p	LOC_Os05g45680.1	156	-28.17	156	-28.17	894996	20	1158	943
miR1846d-3p	LOC_Os05g45760.1	155	-20.25	155	-20.25	895009	20	2209	1069
miR1846d-3p	LOC_Os05g45790.1	166	-27.37	166	-27.37	895013	20	3117	659
miR1846d-3p	LOC_Os05g46020.1	157	-29.57	157	-29.57	895041	20	1383	530
miR1846d-3p	LOC_Os05g46110.1	156	-31.01	156	-31.01	895050	20	1413	646
miR1846d-3p	LOC_Os05g46440.1	145	-20.49	145	-20.49	895093	20	270	96
miR1846d-3p	LOC_Os05g46500.1	145	-20.49	145	-20.49	895107	20	687	45
miR1846d-3p	LOC_Os05g46550.1	146	-20.58	146	-20.58	895112	20	1949	1848
miR1846d-3p	LOC_Os05g47640.1	153	-23.07	153	-23.07	895187	20	2028	1058
miR1846d-3p	LOC_Os05g47700.1	156	-24.57	156	-24.57	895193	20	707	316
miR1846d-3p	LOC_Os05g47740.1	146	-20.01	146	-20.01	895197	20	3963	17
miR1846d-3p	LOC_Os05g47870.1	148	-22.67	148	-22.67	895209	20	3077	1107
miR1846d-3p	LOC_Os05g47870.2	148	-22.67	148	-22.67	895210	20	3074	1107
miR1846d-3p	LOC_Os05g48030.1	152	-25.2	152	-25.2	895227	20	3047	1962

miR1846d-3p	LOC_Os05g48360.1	152	-22.98	152	-22.98	895271	20	2353	803
miR1846d-3p	LOC_Os05g48360.2	152	-22.98	152	-22.98	895272	20	2282	732
miR1846d-3p	LOC_Os05g48550.1	142	-22.55	142	-22.55	895300	20	2916	1595
miR1846d-3p	LOC_Os05g48630.1	144	-21.11	144	-21.11	895309	20	908	731
miR1846d-3p	LOC_Os05g48630.2	144	-21.11	144	-21.11	895310	20	858	681
miR1846d-3p	LOC_Os05g48810.1	161	-30.31	161	-30.31	895335	20	1624	288
miR1846d-3p	LOC_Os05g49210.1	154	-24.17	154	-24.17	895394	20	1878	291
miR1846d-3p	LOC_Os05g49250.1	152	-24.97	152	-24.97	895398	20	1360	638
miR1846d-3p	LOC_Os05g49250.2	152	-24.97	152	-24.97	895399	20	3128	2405
miR1846d-3p	LOC_Os05g49490.1	293	-43.56	148	-22.44	895430	20	1377	1258 1061
miR1846d-3p	LOC_Os05g49540.1	146	-20.01	146	-20.01	895435	20	6342	1259
miR1846d-3p	LOC_Os05g49550.1	146	-20.01	146	-20.01	895436	20	4302	17
miR1846d-3p	LOC_Os05g49560.1	166	-25.39	166	-25.39	895437	20	3168	659
miR1846d-3p	LOC_Os05g50270.1	159	-28.71	159	-28.71	895528	20	1147	637
miR1846d-3p	LOC_Os05g50300.1	156	-27.08	156	-27.08	895531	20	2184	1582
miR1846d-3p	LOC_Os05g50440.1	152	-23.28	152	-23.28	895546	20	1137	229
miR1846d-3p	LOC_Os05g50460.1	152	-24.2	152	-24.2	895548	20	1905	394
miR1846d-3p	LOC_Os05g50520.1	158	-25.45	158	-25.45	895556	20	4215	507
miR1846d-3p	LOC_Os05g50590.1	157	-25.84	157	-25.84	895563	20	366	1
miR1846d-3p	LOC_Os05g50730.1	145	-22.41	145	-22.41	895579	20	1385	792
miR1846d-3p	LOC_Os05g50960.1	151	-24.58	151	-24.58	895613	20	1629	356
miR1846d-3p	LOC_Os05g50990.1	148	-21.64	148	-21.64	895616	20	3238	2418
miR1846d-3p	LOC_Os05g51300.1	147	-22.24	147	-22.24	895658	20	2313	1988
miR1846d-3p	LOC_Os05g51380.1	160	-27	160	-27	895665	20	3561	209
miR1846d-3p	LOC_Os05g51380.2	160	-27	160	-27	895666	20	3088	69
miR1846d-3p	LOC_Os05g51390.1	154	-24.35	154	-24.35	895667	20	1131	236
miR1846d-3p	LOC_Os05g51480.1	142	-20.86	142	-20.86	895682	20	3831	2548
miR1846d-3p	LOC_Os05g51480.2	142	-20.86	142	-20.86	895683	20	3825	2544
miR1846d-3p	LOC_Os05g51510.1	154	-24.59	154	-24.59	895687	20	2206	552
miR1846d-3p	LOC_Os05g51510.2	154	-24.59	154	-24.59	895688	20	2195	552
miR1846d-3p	LOC_Os05g51740.1	161	-27.01	161	-27.01	895724	20	3844	1073
miR1846d-3p	LOC_Os05g51770.1	151	-21.79	151	-21.79	895729	20	3762	3015

miR1846d-3p	LOC_Os05g51810.1	143	-20.48	143	-20.48	895734	20	333	189
miR1846d-3p	LOC_Os05g51830.1	160	-27.41	160	-27.41	895736	20	1448	603
miR1846d-3p	LOC_Os06g01420.1	297	-42.37	150	-21.62	895803	20	5754	507 2408
miR1846d-3p	LOC_Os06g01560.1	305	-50.53	158	-28.91	895818	20	5871	507 2408
miR1846d-3p	LOC_Os06g01590.1	147	-23.18	147	-23.18	895821	20	1532	593
miR1846d-3p	LOC_Os06g01800.1	147	-20.88	147	-20.88	895851	20	1295	134
miR1846d-3p	LOC_Os06g01950.1	152	-23.7	152	-23.7	895863	20	1575	802
miR1846d-3p	LOC_Os06g02120.1	146	-20.55	146	-20.55	895882	20	3318	3196
miR1846d-3p	LOC_Os06g02120.2	146	-20.55	146	-20.55	895883	20	2307	2185
miR1846d-3p	LOC_Os06g02120.3	146	-20.55	146	-20.55	895884	20	2490	2368
miR1846d-3p	LOC_Os06g02740.1	148	-24.33	148	-24.33	895962	20	2160	354
miR1846d-3p	LOC_Os06g02750.1	140	-20.04	140	-20.04	895963	20	2580	1347
miR1846d-3p	LOC_Os06g02770.1	455	-71.05	159	-26.23	895965	20	585	18 79 285
miR1846d-3p	LOC_Os06g02780.1	159	-30.63	159	-30.63	895966	20	1884	1322
miR1846d-3p	LOC_Os06g02830.1	148	-20.93	148	-20.93	895971	20	2741	151
miR1846d-3p	LOC_Os06g03220.1	144	-23.55	144	-23.55	896007	20	1062	593
miR1846d-3p	LOC_Os06g03530.1	146	-22.49	146	-22.49	896038	20	2936	2676
miR1846d-3p	LOC_Os06g03930.1	148	-20.1	148	-20.1	896093	20	1997	1602
miR1846d-3p	LOC_Os06g03930.2	148	-20.1	148	-20.1	896094	20	1646	1251
miR1846d-3p	LOC_Os06g04150.1	151	-23.67	151	-23.67	896119	20	1201	438
miR1846d-3p	LOC_Os06g04410.1	147	-22.86	147	-22.86	896149	20	1038	702
miR1846d-3p	LOC_Os06g04530.1	148	-22.41	148	-22.41	896167	20	1318	675
miR1846d-3p	LOC_Os06g04540.1	169	-30.77	169	-30.77	896168	20	1335	915
miR1846d-3p	LOC_Os06g04650.1	150	-21.08	150	-21.08	896183	20	1213	542
miR1846d-3p	LOC_Os06g04870.1	168	-38.11	168	-38.11	896206	20	1541	269
miR1846d-3p	LOC_Os06g04880.1	151	-22.26	151	-22.26	896207	20	2955	1291
miR1846d-3p	LOC_Os06g05030.1	140	-21.04	140	-21.04	896226	20	3165	1480
miR1846d-3p	LOC_Os06g05260.1	152	-23.45	152	-23.45	896258	20	1867	1471
miR1846d-3p	LOC_Os06g05359.1	150	-23.56	150	-23.56	896268	20	4325	1682
miR1846d-3p	LOC_Os06g05400.1	146	-21.52	146	-21.52	896274	20	561	532
miR1846d-3p	LOC_Os06g05710.1	140	-21.34	140	-21.34	896315	20	711	203
miR1846d-3p	LOC_Os06g05750.1	141	-21.09	141	-21.09	896319	20	1924	1132

miR1846d-3p	LOC_Os06g05820.1	155	-31.51	155	-31.51	896331	20	4435	258
miR1846d-3p	LOC_Os06g05860.1	152	-21.78	152	-21.78	896337	20	2043	1977
miR1846d-3p	LOC_Os06g06014.1	155	-25.82	155	-25.82	896355	20	1345	1085
miR1846d-3p	LOC_Os06g06014.2	155	-25.82	155	-25.82	896356	20	1470	1209
miR1846d-3p	LOC_Os06g06014.3	155	-25.82	155	-25.82	896357	20	1347	1117
miR1846d-3p	LOC_Os06g06014.4	155	-25.82	155	-25.82	896358	20	1269	1039
miR1846d-3p	LOC_Os06g06060.1	147	-22.22	147	-22.22	896368	20	3045	126
miR1846d-3p	LOC_Os06g06090.1	148	-24.61	148	-24.61	896373	20	1815	1647
miR1846d-3p	LOC_Os06g06090.2	148	-24.61	148	-24.61	896374	20	2427	2259
miR1846d-3p	LOC_Os06g06190.1	152	-32.09	152	-32.09	896389	20	2484	166
miR1846d-3p	LOC_Os06g06310.1	156	-25.7	156	-25.7	896403	20	2592	2392
miR1846d-3p	LOC_Os06g06510.1	154	-27.78	154	-27.78	896427	20	774	455
miR1846d-3p	LOC_Os06g06720.1	153	-25.11	153	-25.11	896449	20	1292	426
miR1846d-3p	LOC_Os06g06830.1	147	-21.62	147	-21.62	896463	20	5811	2324
miR1846d-3p	LOC_Os06g06880.1	158	-24.08	158	-24.08	896468	20	1854	1652
miR1846d-3p	LOC_Os06g07520.1	151	-21.79	151	-21.79	896539	20	4575	3666
miR1846d-3p	LOC_Os06g07590.1	145	-23.48	145	-23.48	896549	20	693	535
miR1846d-3p	LOC_Os06g07600.1	153	-22.71	153	-22.71	896550	20	2952	891
miR1846d-3p	LOC_Os06g07923.1	142	-21.62	142	-21.62	896582	20	1573	278
miR1846d-3p	LOC_Os06g07896.1	153	-24.34	153	-24.34	896590	20	1401	1105
miR1846d-3p	LOC_Os06g08090.1	151	-23.12	151	-23.12	896599	20	2112	1437
miR1846d-3p	LOC_Os06g08090.2	151	-23.12	151	-23.12	896600	20	2138	1437
miR1846d-3p	LOC_Os06g08170.1	146	-20.69	146	-20.69	896607	20	2017	1036
miR1846d-3p	LOC_Os06g08380.1	152	-29.32	152	-29.32	896626	20	6114	6017
miR1846d-3p	LOC_Os06g08400.1	146	-21.68	146	-21.68	896628	20	2176	768
miR1846d-3p	LOC_Os06g08400.2	146	-21.68	146	-21.68	896629	20	2506	1144
miR1846d-3p	LOC_Os06g08450.1	154	-25.33	154	-25.33	896633	20	3147	2673
miR1846d-3p	LOC_Os06g08500.1	152	-24.4	152	-24.4	896638	20	1104	932
miR1846d-3p	LOC_Os06g08540.1	157	-30.33	157	-30.33	896646	20	2266	127
miR1846d-3p	LOC_Os06g08540.2	157	-30.33	157	-30.33	896647	20	2172	127
miR1846d-3p	LOC_Os06g08560.1	151	-23.56	151	-23.56	896649	20	4629	2975
miR1846d-3p	LOC_Os06g09240.1	155	-25.11	155	-25.11	896736	20	1497	1235

miR1846d-3p	LOC_Os06g09450.1	148	-22.33	148	-22.33	896761	20	3191	2244
miR1846d-3p	LOC_Os06g09450.2	148	-22.33	148	-22.33	896762	20	3816	2244
miR1846d-3p	LOC_Os06g09450.3	148	-22.33	148	-22.33	896763	20	3122	2244
miR1846d-3p	LOC_Os06g09450.4	148	-22.33	148	-22.33	896764	20	3634	2244
miR1846d-3p	LOC_Os06g09450.5	148	-22.33	148	-22.33	896765	20	3169	2244
miR1846d-3p	LOC_Os06g09450.6	148	-22.33	148	-22.33	896766	20	3219	2244
miR1846d-3p	LOC_Os06g09450.7	148	-22.33	148	-22.33	896767	20	3216	2244
miR1846d-3p	LOC_Os06g09450.8	148	-22.33	148	-22.33	896768	20	3288	2341
miR1846d-3p	LOC_Os06g09640.1	166	-27.37	166	-27.37	896786	20	2796	659
miR1846d-3p	LOC_Os06g10300.1	153	-26.51	153	-26.51	896862	20	1557	390
miR1846d-3p	LOC_Os06g10355.1	456	-71.39	160	-27.38	896868	20	1534	1283 1131 926
miR1846d-3p	LOC_Os06g10420.1	147	-26.45	147	-26.45	896875	20	864	230
miR1846d-3p	LOC_Os06g10450.1	155	-22.68	155	-22.68	896880	20	909	455
miR1846d-3p	LOC_Os06g10950.1	151	-23.33	151	-23.33	896932	20	1776	1753
miR1846d-3p	LOC_Os06g11160.1	145	-22.33	145	-22.33	896954	20	357	148
miR1846d-3p	LOC_Os06g11300.1	157	-29.42	157	-29.42	896968	20	1970	729
miR1846d-3p	LOC_Os06g11304.1	157	-29.42	157	-29.42	896969	20	1569	447
miR1846d-3p	LOC_Os06g11450.1	153	-27.01	153	-27.01	896989	20	1816	1499
miR1846d-3p	LOC_Os06g11840.1	149	-24.57	149	-24.57	897031	20	1638	1426
miR1846d-3p	LOC_Os06g11860.1	160	-26.21	160	-26.21	897033	20	2074	830
miR1846d-3p	LOC_Os06g12090.1	145	-21.33	145	-21.33	897059	20	748	570
miR1846d-3p	LOC_Os06g12170.1	157	-27.21	157	-27.21	897069	20	3403	1616
miR1846d-3p	LOC_Os06g12170.2	157	-27.21	157	-27.21	897070	20	2135	348
miR1846d-3p	LOC_Os06g12170.4	157	-27.21	157	-27.21	897072	20	3029	1242
miR1846d-3p	LOC_Os06g12170.5	157	-27.21	157	-27.21	897073	20	2927	1140
miR1846d-3p	LOC_Os06g12170.6	157	-27.21	157	-27.21	897074	20	2056	269
miR1846d-3p	LOC_Os06g12170.7	157	-27.21	157	-27.21	897075	20	3399	1612
miR1846d-3p	LOC_Os06g12330.1	160	-27.79	160	-27.79	897094	20	1752	310
miR1846d-3p	LOC_Os06g12350.1	151	-25.52	151	-25.52	897096	20	1759	740
miR1846d-3p	LOC_Os06g12640.1	145	-20.91	145	-20.91	897130	20	2327	873
miR1846d-3p	LOC_Os06g12830.1	148	-22.86	148	-22.86	897151	20	1737	276
miR1846d-3p	LOC_Os06g12950.1	160	-27.6	160	-27.6	897164	20	951	570

miR1846d-3p	LOC_Os06g13440.1	154	-21.53	154	-21.53	897217	20	258	142
miR1846d-3p	LOC_Os06g13670.1	157	-24.46	157	-24.46	897247	20	1530	316
miR1846d-3p	LOC_Os06g14080.1	153	-25.1	153	-25.1	897290	20	2760	2676
miR1846d-3p	LOC_Os06g14130.1	157	-23.73	157	-23.73	897295	20	2439	338
miR1846d-3p	LOC_Os06g14150.1	140	-20.15	140	-20.15	897297	20	3324	1564
miR1846d-3p	LOC_Os06g14190.1	162	-25.89	162	-25.89	897301	20	3559	2001
miR1846d-3p	LOC_Os06g14210.1	143	-20.81	143	-20.81	897303	20	783	648
miR1846d-3p	LOC_Os06g14450.1	154	-25.72	154	-25.72	897332	20	3602	156
miR1846d-3p	LOC_Os06g14450.2	154	-25.72	154	-25.72	897333	20	2193	156
miR1846d-3p	LOC_Os06g14450.3	154	-25.72	154	-25.72	897334	20	2200	156
miR1846d-3p	LOC_Os06g14600.1	168	-32.43	168	-32.43	897353	20	2502	1270
miR1846d-3p	LOC_Os06g14660.1	149	-21.32	149	-21.32	897359	20	387	274
miR1846d-3p	LOC_Os06g14830.1	152	-22.27	152	-22.27	897376	20	714	336
miR1846d-3p	LOC_Os06g15100.1	164	-28.95	164	-28.95	897401	20	3261	846
miR1846d-3p	LOC_Os06g15490.1	151	-27.47	151	-27.47	897442	20	441	320
miR1846d-3p	LOC_Os06g15510.1	147	-21.62	147	-21.62	897444	20	3909	698
miR1846d-3p	LOC_Os06g15540.1	172	-34.12	172	-34.12	897447	20	2067	730
miR1846d-3p	LOC_Os06g15640.1	144	-21.65	144	-21.65	897458	20	4107	1578
miR1846d-3p	LOC_Os06g15700.1	152	-23.3	152	-23.3	897463	20	6494	5760
miR1846d-3p	LOC_Os06g15820.1	149	-28.96	149	-28.96	897473	20	2160	1448
miR1846d-3p	LOC_Os06g15920.1	166	-26.29	166	-26.29	897483	20	3132	659
miR1846d-3p	LOC_Os06g15970.1	145	-25.97	145	-25.97	897487	20	4167	2589
miR1846d-3p	LOC_Os06g16000.1	148	-20.13	148	-20.13	897491	20	1564	659
miR1846d-3p	LOC_Os06g16120.1	164	-30.24	164	-30.24	897503	20	1231	564
miR1846d-3p	LOC_Os06g16230.1	148	-27.59	148	-27.59	897514	20	6018	4442
miR1846d-3p	LOC_Os06g16340.1	149	-21.65	149	-21.65	897527	20	549	483
miR1846d-3p	LOC_Os06g16919.1	144	-20.29	144	-20.29	897585	20	1072	631
miR1846d-3p	LOC_Os06g16919.2	144	-20.29	144	-20.29	897586	20	1976	1535
miR1846d-3p	LOC_Os06g16919.3	144	-20.29	144	-20.29	897587	20	2071	1630
miR1846d-3p	LOC_Os06g16919.4	144	-20.29	144	-20.29	897588	20	1078	637
miR1846d-3p	LOC_Os06g17030.1	166	-27.37	166	-27.37	897599	20	3168	659
miR1846d-3p	LOC_Os06g17100.1	155	-26.05	155	-26.05	897606	20	3066	2829

miR1846d-3p	LOC_Os06g17150.1	295	-41.91	148	-21.61	897611	20	6084	507 2546
miR1846d-3p	LOC_Os06g17450.1	149	-23.43	149	-23.43	897641	20	665	465
miR1846d-3p	LOC_Os06g17490.1	148	-20.15	148	-20.15	897645	20	2562	466
miR1846d-3p	LOC_Os06g18150.1	152	-21.61	152	-21.61	897707	20	745	664
miR1846d-3p	LOC_Os06g18150.2	152	-21.61	152	-21.61	897708	20	732	651
miR1846d-3p	LOC_Os06g18160.1	157	-29.69	157	-29.69	897709	20	762	23
miR1846d-3p	LOC_Os06g18680.1	161	-26.95	161	-26.95	897712	20	489	105
miR1846d-3p	LOC_Os06g18960.1	154	-26.25	154	-26.25	897739	20	2860	1138
miR1846d-3p	LOC_Os06g19500.1	148	-24.65	148	-24.65	897794	20	987	765
miR1846d-3p	LOC_Os06g19550.1	145	-22.46	145	-22.46	897799	20	1278	1097
miR1846d-3p	LOC_Os06g19550.2	145	-22.46	145	-22.46	897800	20	1289	1108
miR1846d-3p	LOC_Os06g19570.1	155	-25.03	155	-25.03	897801	20	984	235
miR1846d-3p	LOC_Os06g19750.1	146	-22.61	146	-22.61	897822	20	1163	448
miR1846d-3p	LOC_Os06g20440.1	157	-30.58	157	-30.58	897899	20	1266	1234
miR1846d-3p	LOC_Os06g20500.1	157	-30.58	157	-30.58	897905	20	2490	2458
miR1846d-3p	LOC_Os06g20600.1	159	-22.86	159	-22.86	897913	20	864	1
miR1846d-3p	LOC_Os06g20620.1	156	-26.4	156	-26.4	897915	20	2000	409
miR1846d-3p	LOC_Os06g20660.1	317	-51.8	163	-26.69	897919	20	6465	5684 5441
miR1846d-3p	LOC_Os06g20720.1	146	-20.01	146	-20.01	897925	20	1104	17
miR1846d-3p	LOC_Os06g20740.1	145	-25.38	145	-25.38	897927	20	291	264
miR1846d-3p	LOC_Os06g21040.1	153	-22.46	153	-22.46	897957	20	1932	448
miR1846d-3p	LOC_Os06g21180.1	147	-21.62	147	-21.62	897972	20	4917	1760
miR1846d-3p	LOC_Os06g21300.1	149	-22.27	149	-22.27	897985	20	2517	505
miR1846d-3p	LOC_Os06g21890.1	157	-26.56	157	-26.56	898051	20	1749	1486
miR1846d-3p	LOC_Os06g21980.1	153	-20.72	153	-20.72	898059	20	1740	39
miR1846d-3p	LOC_Os06g22060.1	163	-27.06	163	-27.06	898069	20	2616	461
miR1846d-3p	LOC_Os06g22060.2	163	-27.06	163	-27.06	898070	20	2590	461
miR1846d-3p	LOC_Os06g22060.3	163	-27.06	163	-27.06	898071	20	2676	461
miR1846d-3p	LOC_Os06g22060.4	163	-27.06	163	-27.06	898072	20	2288	159
miR1846d-3p	LOC_Os06g22060.5	163	-27.06	163	-27.06	898073	20	2304	149
miR1846d-3p	LOC_Os06g22100.1	165	-26.88	165	-26.88	898078	20	1996	378
miR1846d-3p	LOC_Os06g22100.2	165	-26.88	165	-26.88	898079	20	2018	400

miR1846d-3p	LOC_Os06g22120.1	150	-21.07	150	-21.07	898081	20	441	207
miR1846d-3p	LOC_Os06g22330.1	158	-26.05	158	-26.05	898100	20	2209	93
miR1846d-3p	LOC_Os06g22640.1	153	-22.37	153	-22.37	898135	20	237	58
miR1846d-3p	LOC_Os06g22850.2	157	-22.89	157	-22.89	898159	20	1485	376
miR1846d-3p	LOC_Os06g23090.1	149	-21.04	149	-21.04	898185	20	1092	382
miR1846d-3p	LOC_Os06g23600.1	146	-21.91	146	-21.91	898238	20	760	624
miR1846d-3p	LOC_Os06g23790.1	156	-23.57	156	-23.57	898257	20	1187	943
miR1846d-3p	LOC_Os06g23790.2	156	-23.57	156	-23.57	898258	20	1219	975
miR1846d-3p	LOC_Os06g23820.1	166	-26.59	166	-26.59	898261	20	1836	659
miR1846d-3p	LOC_Os06g25210.1	144	-22.6	144	-22.6	898400	20	2703	1512
miR1846d-3p	LOC_Os06g25605.1	155	-26.56	155	-26.56	898436	20	2519	2043
miR1846d-3p	LOC_Os06g27560.1	153	-26.21	153	-26.21	898532	20	2026	870
miR1846d-3p	LOC_Os06g27670.1	145	-21.68	145	-21.68	898543	20	804	234
miR1846d-3p	LOC_Os06g27820.1	159	-30.98	159	-30.98	898560	20	1328	540
miR1846d-3p	LOC_Os06g27910.1	164	-28.41	164	-28.41	898572	20	1658	735
miR1846d-3p	LOC_Os06g28090.1	140	-21.04	140	-21.04	898594	20	3165	1480
miR1846d-3p	LOC_Os06g28124.1	150	-21.91	150	-21.91	898597	20	2174	1557
miR1846d-3p	LOC_Os06g28420.1	146	-22.47	146	-22.47	898627	20	747	584
miR1846d-3p	LOC_Os06g28550.1	155	-30.3	155	-30.3	898641	20	1180	636
miR1846d-3p	LOC_Os06g28550.2	155	-30.3	155	-30.3	898642	20	1305	708
miR1846d-3p	LOC_Os06g28700.1	326	-48.83	165	-26.47	898656	20	279	40 146
miR1846d-3p	LOC_Os06g29200.1	166	-26.59	166	-26.59	898707	20	2502	470
miR1846d-3p	LOC_Os06g29460.1	157	-23.65	157	-23.65	898737	20	2210	1783
miR1846d-3p	LOC_Os06g29460.2	157	-23.65	157	-23.65	898738	20	1972	1545
miR1846d-3p	LOC_Os06g29460.3	157	-23.65	157	-23.65	898739	20	2102	1675
miR1846d-3p	LOC_Os06g29679.1	153	-24.22	153	-24.22	898760	20	758	284
miR1846d-3p	LOC_Os06g29750.1	152	-20.54	152	-20.54	898766	20	2412	397
miR1846d-3p	LOC_Os06g29760.1	145	-20.08	145	-20.08	898767	20	6015	4383
miR1846d-3p	LOC_Os06g29860.1	157	-22.89	157	-22.89	898780	20	231	47
miR1846d-3p	LOC_Os06g30470.1	299	-52.74	151	-28.65	898838	20	1432	952 853
miR1846d-3p	LOC_Os06g30820.1	306	-52.61	153	-28.43	898876	20	1935	1626 1672
miR1846d-3p	LOC_Os06g30820.2	306	-52.61	153	-28.43	898877	20	788	488 534

miR1846d-3p	LOC_Os06g30820.3	306	-52.61	153	-28.43	898878	20	797	497 543
miR1846d-3p	LOC_Os06g30820.4	306	-52.61	153	-28.43	898879	20	1703	1394 1440
miR1846d-3p	LOC_Os06g30840.1	146	-20.01	146	-20.01	898881	20	3675	17
miR1846d-3p	LOC_Os06g30860.1	157	-27.87	157	-27.87	898883	20	1041	591
miR1846d-3p	LOC_Os06g30940.1	151	-27.47	151	-27.47	898892	20	2955	281
miR1846d-3p	LOC_Os06g31000.1	147	-21.62	147	-21.62	898899	20	6222	2408
miR1846d-3p	LOC_Os06g31030.1	172	-34.12	172	-34.12	898902	20	2103	766
miR1846d-3p	LOC_Os06g31190.1	164	-32.52	164	-32.52	898918	20	2677	2056
miR1846d-3p	LOC_Os06g31570.1	144	-23.65	144	-23.65	898954	20	1302	897
miR1846d-3p	LOC_Os06g31630.1	152	-21.82	152	-21.82	898958	20	2034	1234
miR1846d-3p	LOC_Os06g31920.1	309	-51.49	157	-31.48	898987	20	2148	1970 1939
miR1846d-3p	LOC_Os06g31950.1	305	-52.43	157	-31.48	898990	20	2148	1970 1938
miR1846d-3p	LOC_Os06g32150.1	156	-28.87	156	-28.87	899008	20	390	281
miR1846d-3p	LOC_Os06g32230.1	308	-48.88	156	-28.87	899015	20	405	350 320
miR1846d-3p	LOC_Os06g32690.1	165	-32.53	165	-32.53	899057	20	282	164
miR1846d-3p	LOC_Os06g32790.1	147	-21.62	147	-21.62	899067	20	6084	2546
miR1846d-3p	LOC_Os06g32800.1	164	-30.76	164	-30.76	899068	20	2523	1831
miR1846d-3p	LOC_Os06g32960.1	151	-26.08	151	-26.08	899083	20	969	284
miR1846d-3p	LOC_Os06g33170.1	160	-35.33	160	-35.33	899104	20	986	623
miR1846d-3p	LOC_Os06g33280.1	145	-20.08	145	-20.08	899114	20	2868	1317
miR1846d-3p	LOC_Os06g33290.1	152	-23.89	152	-23.89	899115	20	4380	949
miR1846d-3p	LOC_Os06g33360.1	151	-22.14	151	-22.14	899124	20	3060	2059
miR1846d-3p	LOC_Os06g33430.1	295	-41.92	148	-21.62	899131	20	6036	507 2546
miR1846d-3p	LOC_Os06g33580.1	157	-25.79	157	-25.79	899144	20	912	288
miR1846d-3p	LOC_Os06g33640.1	142	-23.01	142	-23.01	899150	20	961	732
miR1846d-3p	LOC_Os06g33640.2	142	-23.01	142	-23.01	899151	20	677	448
miR1846d-3p	LOC_Os06g33690.1	146	-24.44	146	-24.44	899155	20	926	782
miR1846d-3p	LOC_Os06g34310.1	151	-24.69	151	-24.69	899217	20	2043	1377
miR1846d-3p	LOC_Os06g34320.1	151	-24.69	151	-24.69	899218	20	5820	1
miR1846d-3p	LOC_Os06g34610.1	168	-35.53	168	-35.53	899248	20	672	39
miR1846d-3p	LOC_Os06g34640.1	143	-21.41	143	-21.41	899251	20	471	439
miR1846d-3p	LOC_Os06g35030.1	159	-30.54	159	-30.54	899288	20	1725	257

miR1846d-3p	LOC_Os06g35030.2	159	-30.54	159	-30.54	899289	20	915	256
miR1846d-3p	LOC_Os06g35030.3	159	-30.54	159	-30.54	899290	20	1735	257
miR1846d-3p	LOC_Os06g35080.1	152	-22.81	152	-22.81	899295	20	1050	315
miR1846d-3p	LOC_Os06g35390.1	295	-42.58	150	-22.08	899326	20	1650	915 1403
miR1846d-3p	LOC_Os06g35430.1	161	-25.78	161	-25.78	899330	20	2442	338
miR1846d-3p	LOC_Os06g35460.1	145	-20.36	145	-20.36	899333	20	3699	2247
miR1846d-3p	LOC_Os06g35510.1	300	-52.76	152	-26.53	899338	20	858	289 507
miR1846d-3p	LOC_Os06g35524.1	148	-28.09	148	-28.09	899340	20	1996	1687
miR1846d-3p	LOC_Os06g35900.1	154	-22.89	154	-22.89	899377	20	1721	1068
miR1846d-3p	LOC_Os06g36020.1	161	-24.18	161	-24.18	899391	20	2043	1094
miR1846d-3p	LOC_Os06g36030.1	148	-23.43	148	-23.43	899392	20	1188	245
miR1846d-3p	LOC_Os06g36110.1	160	-25.64	160	-25.64	899400	20	3336	1060
miR1846d-3p	LOC_Os06g36270.1	159	-31.9	159	-31.9	899415	20	4377	119
miR1846d-3p	LOC_Os06g36270.2	159	-31.9	159	-31.9	899416	20	4514	118
miR1846d-3p	LOC_Os06g36270.3	159	-31.9	159	-31.9	899417	20	4393	118
miR1846d-3p	LOC_Os06g36270.4	159	-31.9	159	-31.9	899418	20	3762	119
miR1846d-3p	LOC_Os06g36270.5	159	-31.9	159	-31.9	899419	20	4256	119
miR1846d-3p	LOC_Os06g36340.1	162	-28.52	162	-28.52	899425	20	717	588
miR1846d-3p	LOC_Os06g36440.1	142	-22.55	142	-22.55	899438	20	1818	500
miR1846d-3p	LOC_Os06g37040.1	303	-45.14	156	-23.52	899502	20	6084	507 2546
miR1846d-3p	LOC_Os06g37050.1	172	-36.72	172	-36.72	899503	20	3144	1807
miR1846d-3p	LOC_Os06g37090.1	166	-26.29	166	-26.29	899507	20	3135	659
miR1846d-3p	LOC_Os06g37400.1	142	-21.05	142	-21.05	899537	20	7053	5714
miR1846d-3p	LOC_Os06g37430.1	158	-24.54	158	-24.54	899541	20	2262	692
miR1846d-3p	LOC_Os06g37430.2	158	-24.54	158	-24.54	899542	20	2149	579
miR1846d-3p	LOC_Os06g37670.1	147	-21.51	147	-21.51	899567	20	3411	1461
miR1846d-3p	LOC_Os06g37730.1	148	-21.6	148	-21.6	899573	20	2469	1240
miR1846d-3p	LOC_Os06g37840.1	145	-21.07	145	-21.07	899584	20	1245	90
miR1846d-3p	LOC_Os06g38040.1	156	-26.15	156	-26.15	899599	20	1869	155
miR1846d-3p	LOC_Os06g38410.1	152	-20.54	152	-20.54	899638	20	2238	397
miR1846d-3p	LOC_Os06g38420.1	145	-20.08	145	-20.08	899639	20	5988	4356
miR1846d-3p	LOC_Os06g38612.1	166	-27.37	166	-27.37	899663	20	3000	659

miR1846d-3p	LOC_Os06g38620.1	172	-35.42	172	-35.42	899664	20	3243	1906
miR1846d-3p	LOC_Os06g38980.1	156	-27.47	156	-27.47	899699	20	3121	2867
miR1846d-3p	LOC_Os06g39170.1	166	-26.59	166	-26.59	899719	20	2586	425
miR1846d-3p	LOC_Os06g39590.1	163	-32.08	163	-32.08	899760	20	1239	930
miR1846d-3p	LOC_Os06g39590.2	163	-32.08	163	-32.08	899761	20	1318	1019
miR1846d-3p	LOC_Os06g39708.1	299	-46.26	154	-24.43	899777	20	1527	557 965
miR1846d-3p	LOC_Os06g39930.1	147	-21.62	147	-21.62	899813	20	6036	2546
miR1846d-3p	LOC_Os06g39950.1	140	-20.04	140	-20.04	899815	20	1989	237
miR1846d-3p	LOC_Os06g40040.1	320	-61.48	165	-31.06	899824	20	835	35 72
miR1846d-3p	LOC_Os06g40040.2	320	-61.48	165	-31.06	899825	20	917	35 72
miR1846d-3p	LOC_Os06g40470.1	162	-24.8	162	-24.8	899871	20	3198	659
miR1846d-3p	LOC_Os06g40550.1	146	-23.41	146	-23.41	899879	20	2399	862
miR1846d-3p	LOC_Os06g40580.1	148	-25.59	148	-25.59	899882	20	876	460
miR1846d-3p	LOC_Os06g41240.1	751	-121.27	154	-27.36	899958	20	951	736 559 668 589 797
miR1846d-3p	LOC_Os06g41290.1	154	-25.75	154	-25.75	899963	20	735	370
miR1846d-3p	LOC_Os06g41390.1	156	-26.71	156	-26.71	899979	20	1539	1184
miR1846d-3p	LOC_Os06g41390.2	156	-26.71	156	-26.71	899980	20	1539	1184
miR1846d-3p	LOC_Os06g41390.3	156	-26.71	156	-26.71	899981	20	1576	1221
miR1846d-3p	LOC_Os06g41390.4	156	-26.71	156	-26.71	899982	20	2037	1682
miR1846d-3p	LOC_Os06g41500.1	144	-23.94	144	-23.94	899990	20	1863	975
miR1846d-3p	LOC_Os06g41520.1	156	-25.53	156	-25.53	899992	20	984	481
miR1846d-3p	LOC_Os06g41680.1	152	-22	152	-22	900009	20	489	422
miR1846d-3p	LOC_Os06g41800.1	147	-20.38	147	-20.38	900023	20	1507	503
miR1846d-3p	LOC_Os06g41970.1	305	-50.53	158	-28.91	900047	20	2934	507 2312
miR1846d-3p	LOC_Os06g42190.1	305	-50.53	158	-28.91	900072	20	5928	507 2546
miR1846d-3p	LOC_Os06g42200.1	172	-34.12	172	-34.12	900073	20	3165	1828
miR1846d-3p	LOC_Os06g42250.1	148	-21.47	148	-21.47	900079	20	2886	176
miR1846d-3p	LOC_Os06g42540.1	149	-24.52	149	-24.52	900110	20	997	302
miR1846d-3p	LOC_Os06g42540.2	149	-24.52	149	-24.52	900111	20	1663	113
miR1846d-3p	LOC_Os06g42590.1	147	-21.62	147	-21.62	900119	20	4815	1805
miR1846d-3p	LOC_Os06g42860.1	148	-21.75	148	-21.75	900149	20	1668	1058
miR1846d-3p	LOC_Os06g42910.1	158	-28	158	-28	900154	20	2110	118

miR1846d-3p	LOC_Os06g43320.1	151	-24.48	151	-24.48	900195	20	1814	1399
miR1846d-3p	LOC_Os06g43450.1	166	-26.59	166	-26.59	900206	20	3198	659
miR1846d-3p	LOC_Os06g43480.1	154	-21.98	154	-21.98	900209	20	1560	716
miR1846d-3p	LOC_Os06g43490.1	154	-24.32	154	-24.32	900210	20	1515	716
miR1846d-3p	LOC_Os06g43550.1	146	-22.11	146	-22.11	900218	20	1783	1382
miR1846d-3p	LOC_Os06g43550.2	146	-22.11	146	-22.11	900219	20	1687	1286
miR1846d-3p	LOC_Os06g43630.1	149	-23.15	149	-23.15	900230	20	3490	2553
miR1846d-3p	LOC_Os06g43875.1	163	-29.2	163	-29.2	900262	20	1349	1264
miR1846d-3p	LOC_Os06g43880.1	151	-23.14	151	-23.14	900263	20	1961	1819
miR1846d-3p	LOC_Os06g43980.1	145	-23.07	145	-23.07	900274	20	912	14
miR1846d-3p	LOC_Os06g44260.1	148	-23.95	148	-23.95	900309	20	1081	375
miR1846d-3p	LOC_Os06g44660.1	144	-21.2	144	-21.2	900351	20	1076	322
miR1846d-3p	LOC_Os06g44770.1	151	-25.28	151	-25.28	900362	20	516	303
miR1846d-3p	LOC_Os06g44780.1	153	-22.6	153	-22.6	900363	20	674	224
miR1846d-3p	LOC_Os06g44810.1	149	-22.19	149	-22.19	900366	20	1990	150
miR1846d-3p	LOC_Os06g44920.1	143	-21.71	143	-21.71	900378	20	1368	742
miR1846d-3p	LOC_Os06g44950.1	305	-50.53	158	-28.91	900381	20	6084	507 2546
miR1846d-3p	LOC_Os06g45260.1	166	-26.29	166	-26.29	900425	20	3132	659
miR1846d-3p	LOC_Os06g45530.1	152	-21.82	152	-21.82	900461	20	2412	1180
miR1846d-3p	LOC_Os06g46690.1	151	-24.66	151	-24.66	900597	20	1956	1366
miR1846d-3p	LOC_Os06g46754.1	150	-23.47	150	-23.47	900603	20	1206	468
miR1846d-3p	LOC_Os06g46754.2	150	-23.47	150	-23.47	900604	20	1387	468
miR1846d-3p	LOC_Os06g47120.1	304	-43.28	152	-21.64	900652	20	2026	1761 1899
miR1846d-3p	LOC_Os06g47310.1	153	-30.62	153	-30.62	900681	20	1476	293
miR1846d-3p	LOC_Os06g47370.1	152	-20.54	152	-20.54	900689	20	2502	397
miR1846d-3p	LOC_Os06g47380.1	145	-20.08	145	-20.08	900690	20	6033	4422
miR1846d-3p	LOC_Os06g47720.1	148	-20.3	148	-20.3	900728	20	3231	2905
miR1846d-3p	LOC_Os06g47800.1	145	-20.58	145	-20.58	900735	20	2955	439
miR1846d-3p	LOC_Os06g47920.1	149	-22.88	149	-22.88	900754	20	2075	1475
miR1846d-3p	LOC_Os06g48090.1	147	-20.79	147	-20.79	900774	20	5526	2210
miR1846d-3p	LOC_Os06g48100.1	172	-34.12	172	-34.12	900775	20	3048	1711
miR1846d-3p	LOC_Os06g48330.1	158	-30.38	158	-30.38	900802	20	2941	348

miR1846d-3p	LOC_Os06g48450.1	152	-21.82	152	-21.82	900815	20	2049	1270
miR1846d-3p	LOC_Os06g48460.1	152	-20.54	152	-20.54	900816	20	2481	397
miR1846d-3p	LOC_Os06g48470.1	145	-20.08	145	-20.08	900817	20	6033	4422
miR1846d-3p	LOC_Os06g48980.1	148	-24.8	148	-24.8	900878	20	1518	343
miR1846d-3p	LOC_Os06g49160.1	148	-23.77	148	-23.77	900909	20	1216	172
miR1846d-3p	LOC_Os06g49450.1	155	-22.95	155	-22.95	900949	20	1617	275
miR1846d-3p	LOC_Os06g49450.2	155	-22.95	155	-22.95	900950	20	1611	275
miR1846d-3p	LOC_Os06g49450.3	155	-22.95	155	-22.95	900951	20	2167	275
miR1846d-3p	LOC_Os06g49520.1	152	-25.84	152	-25.84	900963	20	1532	1157
miR1846d-3p	LOC_Os06g49580.1	142	-22.55	142	-22.55	900969	20	3288	1970
miR1846d-3p	LOC_Os06g49590.1	147	-20.45	147	-20.45	900970	20	213	135
miR1846d-3p	LOC_Os06g49790.1	160	-29.09	160	-29.09	900998	20	2201	1713
miR1846d-3p	LOC_Os06g49950.1	147	-21.62	147	-21.62	901019	20	6156	2618
miR1846d-3p	LOC_Os06g49960.1	172	-34.12	172	-34.12	901020	20	3153	1816
miR1846d-3p	LOC_Os06g50120.1	160	-29.26	160	-29.26	901039	20	1370	1209
miR1846d-3p	LOC_Os06g50120.2	160	-29.26	160	-29.26	901040	20	1362	1201
miR1846d-3p	LOC_Os06g50260.1	157	-23.73	157	-23.73	901055	20	2436	338
miR1846d-3p	LOC_Os06g50270.1	145	-20.08	145	-20.08	901056	20	5910	4275
miR1846d-3p	LOC_Os06g50390.1	143	-20.16	143	-20.16	901071	20	1974	903
miR1846d-3p	LOC_Os06g50510.1	152	-23.71	152	-23.71	901085	20	2115	299
miR1846d-3p	LOC_Os06g50659.1	145	-20.54	145	-20.54	901101	20	687	638
miR1846d-3p	LOC_Os06g50809.1	145	-20.54	145	-20.54	901114	20	687	638
miR1846d-3p	LOC_Os06g51250.1	150	-22.3	150	-22.3	901174	20	4122	190
miR1846d-3p	LOC_Os06g51390.1	153	-25.34	153	-25.34	901197	20	1878	665
miR1846d-3p	LOC_Os06g51390.2	153	-25.34	153	-25.34	901198	20	1884	671
miR1846d-3p	LOC_Os06g51480.1	147	-26.21	147	-26.21	901211	20	1644	386
miR1846d-3p	LOC_Os06g51480.2	147	-26.21	147	-26.21	901212	20	1697	318
miR1846d-3p	LOC_Os07g01050.1	172	-34.12	172	-34.12	901231	20	2670	1435
miR1846d-3p	LOC_Os07g01170.1	304	-53.4	153	-28.07	901250	20	390	96 211
miR1846d-3p	LOC_Os07g01390.1	153	-22.46	153	-22.46	901274	20	4312	2849
miR1846d-3p	LOC_Os07g01500.1	147	-21.62	147	-21.62	901287	20	4881	1487
miR1846d-3p	LOC_Os07g01510.1	172	-34.12	172	-34.12	901288	20	2955	1828

miR1846d-3p	LOC_Os07g01520.1	145	-20.48	145	-20.48	901289	20	2565	2102
miR1846d-3p	LOC_Os07g01550.1	155	-21.84	155	-21.84	901292	20	4010	3625
miR1846d-3p	LOC_Os07g01560.1	155	-21.84	155	-21.84	901293	20	2657	1244
miR1846d-3p	LOC_Os07g01560.2	155	-21.84	155	-21.84	901294	20	2120	1244
miR1846d-3p	LOC_Os07g01650.1	145	-25.97	145	-25.97	901302	20	5235	4509
miR1846d-3p	LOC_Os07g01890.1	149	-23.5	149	-23.5	901333	20	2022	1731
miR1846d-3p	LOC_Os07g01890.2	149	-23.5	149	-23.5	901334	20	2488	1731
miR1846d-3p	LOC_Os07g01910.1	152	-25.86	152	-25.86	901338	20	676	590
miR1846d-3p	LOC_Os07g02290.1	147	-20.06	147	-20.06	901376	20	1398	838
miR1846d-3p	LOC_Os07g03670.1	146	-20.01	146	-20.01	901524	20	3894	17
miR1846d-3p	LOC_Os07g03720.1	146	-20.01	146	-20.01	901529	20	3615	17
miR1846d-3p	LOC_Os07g03840.1	152	-24.25	152	-24.25	901540	20	2355	2071
miR1846d-3p	LOC_Os07g03910.1	150	-20.73	150	-20.73	901547	20	1483	182
miR1846d-3p	LOC_Os07g03940.1	146	-20.01	146	-20.01	901550	20	3963	17
miR1846d-3p	LOC_Os07g03960.1	150	-20.73	150	-20.73	901552	20	2003	335
miR1846d-3p	LOC_Os07g04130.1	145	-21.16	145	-21.16	901568	20	2289	1997
miR1846d-3p	LOC_Os07g04190.1	161	-26.84	161	-26.84	901575	20	3723	1362
miR1846d-3p	LOC_Os07g04300.1	145	-21.3	145	-21.3	901586	20	342	150
miR1846d-3p	LOC_Os07g04560.1	146	-21.32	146	-21.32	901613	20	1032	876
miR1846d-3p	LOC_Os07g04650.1	312	-51.72	156	-26.36	901620	20	1491	954 1365
miR1846d-3p	LOC_Os07g04830.1	148	-23.83	148	-23.83	901641	20	930	896
miR1846d-3p	LOC_Os07g04910.1	145	-27.8	145	-27.8	901650	20	1225	528
miR1846d-3p	LOC_Os07g05190.1	145	-20.21	145	-20.21	901683	20	4130	475
miR1846d-3p	LOC_Os07g05420.1	166	-32.83	166	-32.83	901705	20	1645	295
miR1846d-3p	LOC_Os07g05430.1	151	-25.79	151	-25.79	901706	20	4302	4255
miR1846d-3p	LOC_Os07g05440.1	299	-47.8	153	-26.65	901707	20	5102	2812 4539
miR1846d-3p	LOC_Os07g05440.2	299	-47.8	153	-26.65	901708	20	4620	2330 4057
miR1846d-3p	LOC_Os07g06060.1	161	-32.08	161	-32.08	901777	20	760	591
miR1846d-3p	LOC_Os07g06262.1	157	-23.73	157	-23.73	901796	20	2436	338
miR1846d-3p	LOC_Os07g06355.1	157	-23.73	157	-23.73	901802	20	2436	338
miR1846d-3p	LOC_Os07g06274.1	145	-20.08	145	-20.08	901803	20	5895	4275
miR1846d-3p	LOC_Os07g06420.1	155	-28.16	155	-28.16	901813	20	219	132

miR1846d-3p	LOC_Os07g06470.1	155	-25.18	155	-25.18	901818	20	1587	635
miR1846d-3p	LOC_Os07g06470.2	155	-25.18	155	-25.18	901819	20	1376	635
miR1846d-3p	LOC_Os07g06500.1	142	-23.24	142	-23.24	901821	20	2587	2374
miR1846d-3p	LOC_Os07g06550.1	151	-23.72	151	-23.72	901825	20	1799	1592
miR1846d-3p	LOC_Os07g06990.1	160	-31.27	160	-31.27	901880	20	1047	138
miR1846d-3p	LOC_Os07g06990.2	160	-31.27	160	-31.27	901881	20	1911	138
miR1846d-3p	LOC_Os07g07000.1	141	-20.18	141	-20.18	901882	20	1296	708
miR1846d-3p	LOC_Os07g07020.1	148	-27.49	148	-27.49	901884	20	1190	140
miR1846d-3p	LOC_Os07g07020.2	148	-27.49	148	-27.49	901885	20	1385	140
miR1846d-3p	LOC_Os07g07060.1	151	-20.64	151	-20.64	901893	20	2367	1702
miR1846d-3p	LOC_Os07g07070.1	151	-20.64	151	-20.64	901894	20	3892	632
miR1846d-3p	LOC_Os07g07130.1	154	-30.26	154	-30.26	901897	20	2092	153
miR1846d-3p	LOC_Os07g07194.1	144	-20.25	144	-20.25	901902	20	2591	2360
miR1846d-3p	LOC_Os07g07194.2	144	-20.25	144	-20.25	901903	20	2510	2279
miR1846d-3p	LOC_Os07g07440.1	158	-25.37	158	-25.37	901933	20	786	433
miR1846d-3p	LOC_Os07g07715.1	147	-22.36	147	-22.36	901965	20	581	555
miR1846d-3p	LOC_Os07g07830.1	157	-23.73	157	-23.73	901976	20	1938	338
miR1846d-3p	LOC_Os07g07920.1	156	-23.73	156	-23.73	901986	20	998	555
miR1846d-3p	LOC_Os07g09230.1	158	-22.95	158	-22.95	902129	20	3243	659
miR1846d-3p	LOC_Os07g09420.1	152	-25.11	152	-25.11	902146	20	1784	273
miR1846d-3p	LOC_Os07g09490.1	165	-29.19	165	-29.19	902153	20	1772	523
miR1846d-3p	LOC_Os07g09500.1	160	-26.54	160	-26.54	902154	20	606	471
miR1846d-3p	LOC_Os07g09760.1	154	-24.68	154	-24.68	902184	20	4944	4603
miR1846d-3p	LOC_Os07g10050.1	305	-50.53	158	-28.91	902213	20	5505	507 2414
miR1846d-3p	LOC_Os07g10160.1	145	-23.62	145	-23.62	902224	20	1512	307
miR1846d-3p	LOC_Os07g10560.1	152	-24.64	152	-24.64	902271	20	1110	995
miR1846d-3p	LOC_Os07g10610.1	147	-20.24	147	-20.24	902277	20	1253	671
miR1846d-3p	LOC_Os07g10610.2	147	-20.24	147	-20.24	902278	20	4614	4040
miR1846d-3p	LOC_Os07g10720.1	151	-25.6	151	-25.6	902295	20	789	593
miR1846d-3p	LOC_Os07g10720.2	151	-25.6	151	-25.6	902296	20	784	593
miR1846d-3p	LOC_Os07g10860.1	153	-27.34	153	-27.34	902310	20	1119	97
miR1846d-3p	LOC_Os07g10950.1	149	-23.39	149	-23.39	902324	20	1437	1160

miR1846d-3p	LOC_Os07g11450.1	159	-26.71	159	-26.71	902379	20	567	498
miR1846d-3p	LOC_Os07g11540.1	142	-22.55	142	-22.55	902389	20	2835	1970
miR1846d-3p	LOC_Os07g11600.1	166	-27.37	166	-27.37	902394	20	2955	572
miR1846d-3p	LOC_Os07g11780.1	151	-25.36	151	-25.36	902408	20	723	251
miR1846d-3p	LOC_Os07g12370.1	154	-24.86	154	-24.86	902467	20	633	485
miR1846d-3p	LOC_Os07g12390.1	616	-96.04	154	-24.26	902469	20	607	288 347 396 455
miR1846d-3p	LOC_Os07g12440.1	164	-31.42	164	-31.42	902474	20	243	187
miR1846d-3p	LOC_Os07g12550.1	160	-27.49	160	-27.49	902487	20	729	434
miR1846d-3p	LOC_Os07g12560.1	145	-23.18	145	-23.18	902488	20	2190	1621
miR1846d-3p	LOC_Os07g12560.2	145	-23.18	145	-23.18	902489	20	1979	1410
miR1846d-3p	LOC_Os07g12590.1	148	-22.93	148	-22.93	902492	20	2978	2454
miR1846d-3p	LOC_Os07g12590.2	148	-22.93	148	-22.93	902493	20	2353	1829
miR1846d-3p	LOC_Os07g12600.1	151	-28.93	151	-28.93	902494	20	2584	1718
miR1846d-3p	LOC_Os07g12610.1	303	-54.42	152	-28.93	902495	20	1996	1487 1468
miR1846d-3p	LOC_Os07g12710.1	157	-24.45	157	-24.45	902505	20	1446	1155
miR1846d-3p	LOC_Os07g12770.1	152	-25.16	152	-25.16	902511	20	1840	428
miR1846d-3p	LOC_Os07g12900.1	152	-22.74	152	-22.74	902527	20	3015	416
miR1846d-3p	LOC_Os07g13234.1	159	-22.54	159	-22.54	902559	20	2566	2088
miR1846d-3p	LOC_Os07g13234.2	159	-22.54	159	-22.54	902560	20	2285	1794
miR1846d-3p	LOC_Os07g13420.1	151	-21.79	151	-21.79	902577	20	4545	3636
miR1846d-3p	LOC_Os07g13680.1	164	-28.95	164	-28.95	902601	20	3288	846
miR1846d-3p	LOC_Os07g13770.1	141	-20.41	141	-20.41	902609	20	1620	1063
miR1846d-3p	LOC_Os07g13810.1	157	-27.41	157	-27.41	902613	20	1758	571
miR1846d-3p	LOC_Os07g13860.1	142	-22.55	142	-22.55	902618	20	2733	1328
miR1846d-3p	LOC_Os07g13930.1	148	-24.4	148	-24.4	902625	20	1263	1221
miR1846d-3p	LOC_Os07g13940.1	161	-32.46	161	-32.46	902626	20	1218	564
miR1846d-3p	LOC_Os07g14110.1	143	-23.55	143	-23.55	902643	20	990	6
miR1846d-3p	LOC_Os07g14280.1	159	-28.7	159	-28.7	902671	20	1483	1360
miR1846d-3p	LOC_Os07g14330.1	148	-23.29	148	-23.29	902676	20	450	58
miR1846d-3p	LOC_Os07g14380.1	146	-24.55	146	-24.55	902682	20	1728	977
miR1846d-3p	LOC_Os07g14890.1	156	-25.26	156	-25.26	902737	20	755	510
miR1846d-3p	LOC_Os07g14890.2	156	-25.26	156	-25.26	902738	20	767	522

miR1846d-3p	LOC_Os07g14940.1	147	-23.82	147	-23.82	902743	20	2598	1959
miR1846d-3p	LOC_Os07g14990.1	456	-70.82	157	-27.73	902748	20	3213	2820 1818 2839
miR1846d-3p	LOC_Os07g15030.1	152	-28.05	152	-28.05	902752	20	3090	1419
miR1846d-3p	LOC_Os07g15230.1	141	-22.35	141	-22.35	902772	20	1568	1494
miR1846d-3p	LOC_Os07g15260.1	146	-20.01	146	-20.01	902775	20	3390	17
miR1846d-3p	LOC_Os07g15590.1	169	-35.79	169	-35.79	902809	20	351	213
miR1846d-3p	LOC_Os07g15620.1	147	-20.79	147	-20.79	902812	20	5577	2141
miR1846d-3p	LOC_Os07g15630.1	172	-34.12	172	-34.12	902813	20	3147	1810
miR1846d-3p	LOC_Os07g15690.1	147	-25.24	147	-25.24	902821	20	1998	260
miR1846d-3p	LOC_Os07g15860.1	167	-29	167	-29	902837	20	255	73
miR1846d-3p	LOC_Os07g16030.1	154	-26.03	154	-26.03	902853	20	3314	235
miR1846d-3p	LOC_Os07g16490.1	152	-21.82	152	-21.82	902892	20	2331	1099
miR1846d-3p	LOC_Os07g16790.1	153	-22.44	153	-22.44	902922	20	903	715
miR1846d-3p	LOC_Os07g17030.1	146	-20.01	146	-20.01	902950	20	3963	17
miR1846d-3p	LOC_Os07g17040.1	149	-21.9	149	-21.9	902951	20	798	753
miR1846d-3p	LOC_Os07g17290.1	146	-23.14	146	-23.14	902977	20	1851	1690
miR1846d-3p	LOC_Os07g17640.1	147	-21.56	147	-21.56	903014	20	804	332
miR1846d-3p	LOC_Os07g18100.1	156	-31.19	156	-31.19	903061	20	411	337
miR1846d-3p	LOC_Os07g18250.1	147	-26.75	147	-26.75	903077	20	2085	1879
miR1846d-3p	LOC_Os07g18250.2	147	-26.75	147	-26.75	903078	20	1815	1609
miR1846d-3p	LOC_Os07g18260.1	156	-26.56	156	-26.56	903079	20	963	217
miR1846d-3p	LOC_Os07g18610.1	163	-28.56	163	-28.56	903112	20	1467	497
miR1846d-3p	LOC_Os07g19410.1	296	-44.26	148	-22.99	903181	20	1392	855 972
miR1846d-3p	LOC_Os07g20200.1	306	-48.41	166	-27.37	903212	20	3207	659 1522
miR1846d-3p	LOC_Os07g20240.1	144	-23.56	144	-23.56	903216	20	3092	2472
miR1846d-3p	LOC_Os07g20450.1	160	-31.25	160	-31.25	903236	20	954	883
miR1846d-3p	LOC_Os07g20670.1	152	-22.57	152	-22.57	903259	20	5637	5501
miR1846d-3p	LOC_Os07g22610.1	144	-23.56	144	-23.56	903361	20	4340	3971
miR1846d-3p	LOC_Os07g22880.1	142	-22.55	142	-22.55	903395	20	2887	1520
miR1846d-3p	LOC_Os07g23190.1	147	-21.38	147	-21.38	903431	20	1107	972
miR1846d-3p	LOC_Os07g23220.1	140	-20.04	140	-20.04	903434	20	3684	3048
miR1846d-3p	LOC_Os07g23300.1	147	-21.62	147	-21.62	903441	20	5358	2243

miR1846d-3p	LOC_Os07g23400.1	145	-24.3	145	-24.3	903449	20	2820	2740
miR1846d-3p	LOC_Os07g23770.1	140	-20.28	140	-20.28	903488	20	630	564
miR1846d-3p	LOC_Os07g24320.1	148	-20.37	148	-20.37	903552	20	2097	75
miR1846d-3p	LOC_Os07g24340.1	146	-20.01	146	-20.01	903554	20	4290	17
miR1846d-3p	LOC_Os07g25090.1	145	-20.08	145	-20.08	903631	20	6018	4386
miR1846d-3p	LOC_Os07g25484.1	152	-23.82	152	-23.82	903671	20	252	189
miR1846d-3p	LOC_Os07g25670.1	149	-21.98	149	-21.98	903689	20	726	441
miR1846d-3p	LOC_Os07g25820.1	141	-20.06	141	-20.06	903705	20	432	216
miR1846d-3p	LOC_Os07g26250.1	156	-28.42	156	-28.42	903748	20	528	47
miR1846d-3p	LOC_Os07g26720.1	140	-20.08	140	-20.08	903799	20	621	593
miR1846d-3p	LOC_Os07g26720.2	140	-20.08	140	-20.08	903800	20	633	605
miR1846d-3p	LOC_Os07g26750.1	156	-28.89	156	-28.89	903803	20	555	225
miR1846d-3p	LOC_Os07g26760.1	140	-21.6	140	-21.6	903804	20	312	276
miR1846d-3p	LOC_Os07g26920.1	155	-24.01	155	-24.01	903820	20	708	433
miR1846d-3p	LOC_Os07g27120.1	152	-25.64	152	-25.64	903841	20	1497	436
miR1846d-3p	LOC_Os07g27120.2	152	-25.64	152	-25.64	903842	20	3835	436
miR1846d-3p	LOC_Os07g27120.3	152	-25.64	152	-25.64	903843	20	1840	387
miR1846d-3p	LOC_Os07g27120.4	152	-25.64	152	-25.64	903844	20	1328	387
miR1846d-3p	LOC_Os07g27120.5	152	-25.64	152	-25.64	903845	20	1732	382
miR1846d-3p	LOC_Os07g27120.6	152	-25.64	152	-25.64	903846	20	1639	382
miR1846d-3p	LOC_Os07g27240.1	151	-21.02	151	-21.02	903859	20	522	377
miR1846d-3p	LOC_Os07g27320.1	156	-25.64	156	-25.64	903867	20	3792	290
miR1846d-3p	LOC_Os07g27330.1	296	-49.69	152	-24.94	903868	20	2716	1755 2130
miR1846d-3p	LOC_Os07g27670.1	157	-25.95	157	-25.95	903901	20	791	756
miR1846d-3p	LOC_Os07g27690.1	153	-21.65	153	-21.65	903903	20	1620	196
miR1846d-3p	LOC_Os07g28180.1	147	-20.51	147	-20.51	903955	20	477	376
miR1846d-3p	LOC_Os07g28370.1	141	-20.78	141	-20.78	903975	20	5385	3946
miR1846d-3p	LOC_Os07g28440.1	159	-29.17	159	-29.17	903982	20	1609	1398
miR1846d-3p	LOC_Os07g28530.1	147	-21.62	147	-21.62	903992	20	3366	128
miR1846d-3p	LOC_Os07g28850.1	150	-22.39	150	-22.39	904028	20	3538	867
miR1846d-3p	LOC_Os07g29010.1	149	-22.27	149	-22.27	904043	20	1302	436
miR1846d-3p	LOC_Os07g29280.1	302	-47.92	153	-24.51	904066	20	228	33 142

miR1846d-3p	LOC_Os07g29560.1	157	-22.54	157	-22.54	904103	20	660	632
miR1846d-3p	LOC_Os07g29630.1	157	-26.91	157	-26.91	904112	20	1006	892
miR1846d-3p	LOC_Os07g29700.1	155	-21.67	155	-21.67	904120	20	477	301
miR1846d-3p	LOC_Os07g29910.1	148	-24.15	148	-24.15	904142	20	627	42
miR1846d-3p	LOC_Os07g30060.1	151	-24.69	151	-24.69	904156	20	681	381
miR1846d-3p	LOC_Os07g30350.1	306	-46.74	166	-26.59	904190	20	3156	614 1396
miR1846d-3p	LOC_Os07g30410.1	154	-25.92	154	-25.92	904194	20	2064	1981
miR1846d-3p	LOC_Os07g30510.1	154	-25.92	154	-25.92	904203	20	2064	1981
miR1846d-3p	LOC_Os07g30570.1	152	-20.54	152	-20.54	904208	20	2391	397
miR1846d-3p	LOC_Os07g30890.1	166	-26.29	166	-26.29	904245	20	3189	659
miR1846d-3p	LOC_Os07g31040.1	158	-28.91	158	-28.91	904261	20	2322	507
miR1846d-3p	LOC_Os07g31050.1	147	-21.62	147	-21.62	904262	20	3246	128
miR1846d-3p	LOC_Os07g31150.1	154	-26.61	154	-26.61	904271	20	657	99
miR1846d-3p	LOC_Os07g31410.1	155	-29.5	155	-29.5	904302	20	636	253
miR1846d-3p	LOC_Os07g31630.1	147	-21.62	147	-21.62	904327	20	5046	1733
miR1846d-3p	LOC_Os07g31670.1	153	-29.66	153	-29.66	904331	20	414	154
miR1846d-3p	LOC_Os07g31750.1	308	-51.46	156	-28.43	904338	20	1365	1292 976
miR1846d-3p	LOC_Os07g31770.1	160	-27.91	160	-27.91	904340	20	1726	1479
miR1846d-3p	LOC_Os07g32220.1	153	-22.46	153	-22.46	904380	20	2121	505
miR1846d-3p	LOC_Os07g32310.1	147	-21.62	147	-21.62	904390	20	5673	2555
miR1846d-3p	LOC_Os07g32412.2	174	-32.17	174	-32.17	904406	20	2335	466
miR1846d-3p	LOC_Os07g32470.1	145	-21.19	145	-21.19	904414	20	531	195
miR1846d-3p	LOC_Os07g32630.1	145	-20.93	145	-20.93	904431	20	1962	302
miR1846d-3p	LOC_Os07g32640.1	158	-29.59	158	-29.59	904432	20	297	242
miR1846d-3p	LOC_Os07g32900.1	157	-28	157	-28	904457	20	3187	84
miR1846d-3p	LOC_Os07g33000.1	162	-24.8	162	-24.8	904466	20	2691	659
miR1846d-3p	LOC_Os07g33440.1	170	-30.16	170	-30.16	904517	20	1873	281
miR1846d-3p	LOC_Os07g33450.1	148	-20.72	148	-20.72	904518	20	4422	3999
miR1846d-3p	LOC_Os07g33570.1	157	-24.09	157	-24.09	904532	20	2565	1681
miR1846d-3p	LOC_Os07g33790.1	161	-26.6	161	-26.6	904557	20	3865	172
miR1846d-3p	LOC_Os07g33830.1	148	-22.77	148	-22.77	904559	20	1410	507
miR1846d-3p	LOC_Os07g34130.1	142	-21.82	142	-21.82	904585	20	2103	509

miR1846d-3p	LOC_Os07g34170.1	152	-24.27	152	-24.27	904589	20	1344	1141
miR1846d-3p	LOC_Os07g34390.1	312	-56.29	158	-28.78	904611	20	2036	635 112
miR1846d-3p	LOC_Os07g34650.1	141	-25.15	141	-25.15	904647	20	1850	232
miR1846d-3p	LOC_Os07g34700.1	158	-30.27	158	-30.27	904652	20	1395	1148
miR1846d-3p	LOC_Os07g34830.1	151	-24.95	151	-24.95	904666	20	2383	2070
miR1846d-3p	LOC_Os07g34860.1	146	-20.01	146	-20.01	904669	20	3963	17
miR1846d-3p	LOC_Os07g34980.1	148	-24.97	148	-24.97	904681	20	1977	465
miR1846d-3p	LOC_Os07g35130.1	143	-21.15	143	-21.15	904696	20	1812	1043
miR1846d-3p	LOC_Os07g35140.1	150	-22.61	150	-22.61	904697	20	2575	802
miR1846d-3p	LOC_Os07g35140.2	150	-22.61	150	-22.61	904698	20	1999	802
miR1846d-3p	LOC_Os07g35240.1	142	-22.55	142	-22.55	904708	20	4551	2894
miR1846d-3p	LOC_Os07g35510.1	157	-24.04	157	-24.04	904743	20	2395	635
miR1846d-3p	LOC_Os07g35680.1	153	-24.03	153	-24.03	904762	20	2557	694
miR1846d-3p	LOC_Os07g35700.1	147	-21.68	147	-21.68	904765	20	2359	2326
miR1846d-3p	LOC_Os07g35979.1	144	-22.93	144	-22.93	904795	20	1699	434
miR1846d-3p	LOC_Os07g36010.1	156	-23.52	156	-23.52	904799	20	5439	3273
miR1846d-3p	LOC_Os07g36150.1	148	-22.9	148	-22.9	904818	20	3891	3749
miR1846d-3p	LOC_Os07g36230.1	299	-50.77	150	-28.99	904828	20	2342	1952 1677
miR1846d-3p	LOC_Os07g36230.2	299	-50.77	150	-28.99	904829	20	2032	1620 1345
miR1846d-3p	LOC_Os07g36230.3	299	-50.77	150	-28.99	904830	20	2229	1839 1564
miR1846d-3p	LOC_Os07g36230.4	299	-50.77	150	-28.99	904831	20	2085	1695 1420
miR1846d-3p	LOC_Os07g36660.1	166	-26.59	166	-26.59	904874	20	2772	659
miR1846d-3p	LOC_Os07g36830.1	152	-24.35	152	-24.35	904893	20	1758	1163
miR1846d-3p	LOC_Os07g36990.1	146	-20.01	146	-20.01	904912	20	3891	17
miR1846d-3p	LOC_Os07g37156.1	157	-25.82	157	-25.82	904933	20	1008	528
miR1846d-3p	LOC_Os07g37190.1	166	-26.29	166	-26.29	904936	20	3135	659
miR1846d-3p	LOC_Os07g37454.1	158	-28.8	158	-28.8	904961	20	1978	883
miR1846d-3p	LOC_Os07g37550.1	148	-23.72	148	-23.72	904971	20	1194	217
miR1846d-3p	LOC_Os07g37630.1	151	-20.96	151	-20.96	904983	20	3669	3566
miR1846d-3p	LOC_Os07g38280.1	143	-20.85	143	-20.85	905056	20	3630	3599
miR1846d-3p	LOC_Os07g38280.2	143	-20.85	143	-20.85	905057	20	3543	3512
miR1846d-3p	LOC_Os07g38300.1	141	-20.38	141	-20.38	905059	20	1277	1033

miR1846d-3p	LOC_Os07g38300.2	141	-20.38	141	-20.38	905060	20	1290	1046
miR1846d-3p	LOC_Os07g38300.5	141	-20.38	141	-20.38	905061	20	1285	1041
miR1846d-3p	LOC_Os07g38490.1	153	-20.7	153	-20.7	905082	20	1982	755
miR1846d-3p	LOC_Os07g38500.1	305	-47.07	158	-25.45	905083	20	5796	507 2516
miR1846d-3p	LOC_Os07g38660.1	157	-22.87	157	-22.87	905100	20	393	340
miR1846d-3p	LOC_Os07g39260.1	149	-21.02	149	-21.02	905168	20	1478	1388
miR1846d-3p	LOC_Os07g39390.1	155	-28.59	155	-28.59	905183	20	2023	730
miR1846d-3p	LOC_Os07g39560.3	148	-20.58	148	-20.58	905204	20	2569	525
miR1846d-3p	LOC_Os07g39680.1	149	-20.15	149	-20.15	905217	20	729	69
miR1846d-3p	LOC_Os07g39750.1	141	-22.26	141	-22.26	905226	20	2350	2212
miR1846d-3p	LOC_Os07g39750.2	141	-22.26	141	-22.26	905227	20	1317	1179
miR1846d-3p	LOC_Os07g39750.3	141	-22.26	141	-22.26	905228	20	1412	1274
miR1846d-3p	LOC_Os07g39810.1	156	-29.24	156	-29.24	905236	20	1168	726
miR1846d-3p	LOC_Os07g39990.1	146	-23.59	146	-23.59	905256	20	492	159
miR1846d-3p	LOC_Os07g40050.1	149	-20.37	149	-20.37	905263	20	5910	94
miR1846d-3p	LOC_Os07g40120.1	302	-47.02	156	-25.72	905270	20	2355	706 1233
miR1846d-3p	LOC_Os07g40150.1	146	-21.53	146	-21.53	905273	20	2217	912
miR1846d-3p	LOC_Os07g40404.1	149	-26.54	149	-26.54	905299	20	2259	1805
miR1846d-3p	LOC_Os07g40550.1	141	-20.23	141	-20.23	905314	20	3193	819
miR1846d-3p	LOC_Os07g40550.2	141	-20.23	141	-20.23	905315	20	3231	819
miR1846d-3p	LOC_Os07g40550.3	141	-20.23	141	-20.23	905316	20	3752	819
miR1846d-3p	LOC_Os07g40590.1	140	-21.31	140	-21.31	905320	20	3342	1480
miR1846d-3p	LOC_Os07g40700.1	147	-20.2	147	-20.2	905331	20	1726	384
miR1846d-3p	LOC_Os07g40700.2	147	-20.2	147	-20.2	905332	20	1537	384
miR1846d-3p	LOC_Os07g40880.1	144	-21.31	144	-21.31	905354	20	447	359
miR1846d-3p	LOC_Os07g41110.1	306	-51.34	158	-28.91	905376	20	5073	507 2445
miR1846d-3p	LOC_Os07g41240.1	155	-29.3	155	-29.3	905389	20	1945	1346
miR1846d-3p	LOC_Os07g41610.1	146	-20.01	146	-20.01	905432	20	1095	17
miR1846d-3p	LOC_Os07g41830.1	148	-22.92	148	-22.92	905467	20	1161	1135
miR1846d-3p	LOC_Os07g41870.1	166	-27.37	166	-27.37	905471	20	3207	659
miR1846d-3p	LOC_Os07g42050.1	166	-27.37	166	-27.37	905489	20	3249	659
miR1846d-3p	LOC_Os07g42200.1	154	-23.29	154	-23.29	905501	20	1875	1337

miR1846d-3p	LOC_Os07g42324.1	145	-22.32	145	-22.32	905512	20	1233	656
miR1846d-3p	LOC_Os07g42510.1	152	-25.35	152	-25.35	905538	20	1557	968
miR1846d-3p	LOC_Os07g42590.1	146	-22.84	146	-22.84	905546	20	1842	867
miR1846d-3p	LOC_Os07g42650.1	145	-20.67	145	-20.67	905558	20	1334	236
miR1846d-3p	LOC_Os07g42650.2	145	-20.67	145	-20.67	905559	20	1525	236
miR1846d-3p	LOC_Os07g42650.3	145	-20.67	145	-20.67	905560	20	1244	236
miR1846d-3p	LOC_Os07g42680.1	154	-30.87	154	-30.87	905562	20	3408	564
miR1846d-3p	LOC_Os07g42714.1	143	-22.22	143	-22.22	905568	20	3539	3017
miR1846d-3p	LOC_Os07g42800.1	153	-22.94	153	-22.94	905577	20	2012	1025
miR1846d-3p	LOC_Os07g42880.1	161	-29.03	161	-29.03	905584	20	1875	849
miR1846d-3p	LOC_Os07g43350.1	160	-24.53	160	-24.53	905651	20	3633	2882
miR1846d-3p	LOC_Os07g43400.1	143	-23.31	143	-23.31	905656	20	1832	336
miR1846d-3p	LOC_Os07g43460.1	150	-25.28	150	-25.28	905662	20	1781	1463
miR1846d-3p	LOC_Os07g43750.1	147	-21.62	147	-21.62	905689	20	5166	2156
miR1846d-3p	LOC_Os07g43820.1	158	-25.95	158	-25.95	905697	20	1164	781
miR1846d-3p	LOC_Os07g44950.1	314	-52.69	158	-28.57	905825	20	1974	312 267
miR1846d-3p	LOC_Os07g45660.1	168	-32.42	168	-32.42	905896	20	2481	1249
miR1846d-3p	LOC_Os07g45670.1	149	-23.81	149	-23.81	905897	20	1389	996
miR1846d-3p	LOC_Os07g45830.1	152	-25.45	152	-25.45	905913	20	4632	534
miR1846d-3p	LOC_Os07g45900.1	142	-22.55	142	-22.55	905920	20	3288	1724
miR1846d-3p	LOC_Os07g46250.1	157	-23.73	157	-23.73	905955	20	2436	338
miR1846d-3p	LOC_Os07g46330.1	161	-32.08	161	-32.08	905965	20	1378	521
miR1846d-3p	LOC_Os07g46440.1	292	-48.19	149	-27.08	905982	20	1888	1593 724
miR1846d-3p	LOC_Os07g46730.2	144	-21.52	144	-21.52	906026	20	2408	2289
miR1846d-3p	LOC_Os07g46750.1	145	-20.18	145	-20.18	906028	20	1181	73
miR1846d-3p	LOC_Os07g46770.1	313	-52.35	159	-27.01	906031	20	1860	831 494
miR1846d-3p	LOC_Os07g46780.1	154	-26.97	154	-26.97	906032	20	1700	1454
miR1846d-3p	LOC_Os07g46780.2	154	-26.97	154	-26.97	906033	20	1774	1534
miR1846d-3p	LOC_Os07g46780.3	154	-26.97	154	-26.97	906034	20	2172	1932
miR1846d-3p	LOC_Os07g46880.1	147	-21.62	147	-21.62	906050	20	6084	2546
miR1846d-3p	LOC_Os07g47194.1	152	-21.65	152	-21.65	906093	20	2642	1521
miR1846d-3p	LOC_Os07g47194.2	152	-21.65	152	-21.65	906094	20	2509	1388

miR1846d-3p	LOC_Os07g47194.3	152	-21.65	152	-21.65	906095	20	3000	1843
miR1846d-3p	LOC_Os07g47470.1	153	-27.86	153	-27.86	906132	20	1656	138
miR1846d-3p	LOC_Os07g47670.2	156	-27.55	156	-27.55	906171	20	823	75
miR1846d-3p	LOC_Os07g47680.1	149	-24.7	149	-24.7	906172	20	582	166
miR1846d-3p	LOC_Os07g47730.1	157	-23.73	157	-23.73	906177	20	2481	338
miR1846d-3p	LOC_Os07g47870.1	148	-20.89	148	-20.89	906195	20	2376	2073
miR1846d-3p	LOC_Os07g47970.1	145	-20.15	145	-20.15	906205	20	2157	629
miR1846d-3p	LOC_Os07g48170.1	150	-26.53	150	-26.53	906229	20	3415	3128
miR1846d-3p	LOC_Os07g48280.1	153	-21.16	153	-21.16	906239	20	2677	1498
miR1846d-3p	LOC_Os07g48280.2	153	-21.16	153	-21.16	906240	20	2315	1446
miR1846d-3p	LOC_Os07g48280.3	153	-21.16	153	-21.16	906241	20	2726	1446
miR1846d-3p	LOC_Os07g48650.1	147	-21.65	147	-21.65	906294	20	2601	444
miR1846d-3p	LOC_Os07g49090.1	152	-28.57	152	-28.57	906351	20	915	441
miR1846d-3p	LOC_Os07g49250.1	145	-23.27	145	-23.27	906370	20	2163	252
miR1846d-3p	LOC_Os07g49330.1	332	-61.23	168	-31.6	906380	20	2380	1844 1730
miR1846d-3p	LOC_Os07g49330.3	332	-61.23	168	-31.6	906381	20	2561	2028 1914
miR1846d-3p	LOC_Os07g49410.1	143	-21.64	143	-21.64	906393	20	1444	566
miR1846d-3p	LOC_Os07g49410.2	143	-21.64	143	-21.64	906394	20	1466	588
miR1846d-3p	LOC_Os07g49410.3	141	-23.22	141	-23.22	906395	20	1440	563
miR1846d-3p	LOC_Os07g49530.1	144	-21.9	144	-21.9	906410	20	1623	522
miR1846d-3p	LOC_Os08g01054.1	164	-28.28	164	-28.28	906417	20	5588	249
miR1846d-3p	LOC_Os08g01054.2	164	-28.28	164	-28.28	906418	20	5082	249
miR1846d-3p	LOC_Os08g01100.1	164	-33.89	164	-33.89	906426	20	1021	952
miR1846d-3p	LOC_Os08g01514.1	302	-45.85	153	-23.16	906481	20	945	690 572
miR1846d-3p	LOC_Os08g01950.1	156	-25.77	156	-25.77	906534	20	1838	598
miR1846d-3p	LOC_Os08g02030.1	148	-25.19	148	-25.19	906542	20	1644	1562
miR1846d-3p	LOC_Os08g02204.1	147	-22.87	147	-22.87	906561	20	904	434
miR1846d-3p	LOC_Os08g02370.1	147	-20.69	147	-20.69	906581	20	1529	1054
miR1846d-3p	LOC_Os08g02470.1	147	-21.62	147	-21.62	906592	20	5049	2315
miR1846d-3p	LOC_Os08g02750.1	155	-21.15	155	-21.15	906623	20	1300	454
miR1846d-3p	LOC_Os08g02880.1	141	-20.09	141	-20.09	906635	20	679	7
miR1846d-3p	LOC_Os08g03002.1	156	-25.81	156	-25.81	906651	20	1783	1596

miR1846d-3p	LOC_Os08g03180.1	164	-27.84	164	-27.84	906669	20	4808	124
miR1846d-3p	LOC_Os08g03190.1	164	-27.84	164	-27.84	906670	20	480	57
miR1846d-3p	LOC_Os08g03270.1	151	-23.55	151	-23.55	906677	20	2115	1256
miR1846d-3p	LOC_Os08g03310.1	144	-22.8	144	-22.8	906684	20	2735	1207
miR1846d-3p	LOC_Os08g03310.2	144	-22.8	144	-22.8	906685	20	2687	1159
miR1846d-3p	LOC_Os08g03310.3	144	-22.8	144	-22.8	906686	20	2921	1308
miR1846d-3p	LOC_Os08g03310.4	144	-22.8	144	-22.8	906687	20	2788	1260
miR1846d-3p	LOC_Os08g03310.5	144	-22.8	144	-22.8	906688	20	2833	1308
miR1846d-3p	LOC_Os08g03310.6	144	-22.8	144	-22.8	906689	20	3135	1308
miR1846d-3p	LOC_Os08g03370.1	151	-27.22	151	-27.22	906694	20	1040	759
miR1846d-3p	LOC_Os08g03520.1	152	-22.21	152	-22.21	906714	20	1194	918
miR1846d-3p	LOC_Os08g03710.1	146	-24.67	146	-24.67	906737	20	423	270
miR1846d-3p	LOC_Os08g03960.1	166	-27.37	166	-27.37	906765	20	2346	14
miR1846d-3p	LOC_Os08g04310.1	153	-25.13	153	-25.13	906808	20	1055	373
miR1846d-3p	LOC_Os08g04330.1	142	-22.55	142	-22.55	906810	20	2064	1157
miR1846d-3p	LOC_Os08g04440.1	152	-25.21	152	-25.21	906820	20	1080	1014
miR1846d-3p	LOC_Os08g04440.2	152	-25.21	152	-25.21	906821	20	2636	2592
miR1846d-3p	LOC_Os08g04510.1	163	-28.08	163	-28.08	906832	20	5013	3395
miR1846d-3p	LOC_Os08g04560.1	310	-50.28	165	-29.81	906837	20	2122	674 1326
miR1846d-3p	LOC_Os08g05280.1	146	-25.35	146	-25.35	906918	20	2538	1361
miR1846d-3p	LOC_Os08g05300.1	145	-21.21	145	-21.21	906920	20	498	383
miR1846d-3p	LOC_Os08g05400.1	142	-22.55	142	-22.55	906930	20	1479	158
miR1846d-3p	LOC_Os08g05510.1	152	-22.01	152	-22.01	906941	20	1290	142
miR1846d-3p	LOC_Os08g05510.2	152	-22.01	152	-22.01	906942	20	1256	142
miR1846d-3p	LOC_Os08g05520.1	148	-28.06	148	-28.06	906943	20	1759	1332
miR1846d-3p	LOC_Os08g05530.1	149	-21.84	149	-21.84	906944	20	336	84
miR1846d-3p	LOC_Os08g05550.1	160	-24.78	160	-24.78	906946	20	1241	454
miR1846d-3p	LOC_Os08g05560.1	145	-23.87	145	-23.87	906947	20	2319	61
miR1846d-3p	LOC_Os08g05770.1	161	-29.55	161	-29.55	906967	20	1040	980
miR1846d-3p	LOC_Os08g05780.1	161	-29.55	161	-29.55	906968	20	1132	947
miR1846d-3p	LOC_Os08g05930.1	145	-20.02	145	-20.02	906988	20	5592	3972
miR1846d-3p	LOC_Os08g06220.1	155	-23.33	155	-23.33	907020	20	1438	536

miR1846d-3p	LOC_Os08g06370.1	166	-28.37	166	-28.37	907043	20	1605	109
miR1846d-3p	LOC_Os08g06415.1	317	-52.77	161	-26.68	907049	20	1114	763 803
miR1846d-3p	LOC_Os08g06420.1	145	-20.9	145	-20.9	907050	20	2183	606
miR1846d-3p	LOC_Os08g06420.2	299	-50.05	154	-29.15	907051	20	2064	397 487
miR1846d-3p	LOC_Os08g06480.1	305	-51.29	156	-26.72	907061	20	4506	2841 3644
miR1846d-3p	LOC_Os08g06570.1	305	-47.17	158	-25.55	907072	20	6030	507 2540
miR1846d-3p	LOC_Os08g06659.1	149	-20.76	149	-20.76	907081	20	1651	1137
miR1846d-3p	LOC_Os08g06830.1	153	-22.44	153	-22.44	907096	20	1329	1
miR1846d-3p	LOC_Os08g07130.1	156	-23.52	156	-23.52	907125	20	6036	507
miR1846d-3p	LOC_Os08g07140.1	144	-24.33	144	-24.33	907126	20	3144	1829
miR1846d-3p	LOC_Os08g07170.1	152	-20.08	152	-20.08	907128	20	654	310
miR1846d-3p	LOC_Os08g07220.1	160	-26.93	160	-26.93	907132	20	2940	855
miR1846d-3p	LOC_Os08g07380.1	147	-27.05	147	-27.05	907148	20	5334	3999
miR1846d-3p	LOC_Os08g07410.1	166	-27.37	166	-27.37	907151	20	3306	659
miR1846d-3p	LOC_Os08g07910.1	157	-26.1	157	-26.1	907202	20	459	169
miR1846d-3p	LOC_Os08g08205.1	160	-29.04	160	-29.04	907237	20	1811	177
miR1846d-3p	LOC_Os08g08360.1	152	-30.21	152	-30.21	907253	20	1015	277
miR1846d-3p	LOC_Os08g08586.1	149	-28.79	149	-28.79	907273	20	2286	1922
miR1846d-3p	LOC_Os08g08586.2	149	-28.79	149	-28.79	907274	20	1596	1232
miR1846d-3p	LOC_Os08g08586.3	149	-28.79	149	-28.79	907275	20	2364	2000
miR1846d-3p	LOC_Os08g08586.4	149	-28.79	149	-28.79	907276	20	1686	1322
miR1846d-3p	LOC_Os08g08592.1	150	-20.54	150	-20.54	907277	20	2225	1817
miR1846d-3p	LOC_Os08g08592.2	150	-20.54	150	-20.54	907278	20	2229	1817
miR1846d-3p	LOC_Os08g08592.3	150	-20.54	150	-20.54	907279	20	2322	1817
miR1846d-3p	LOC_Os08g08600.1	145	-20.08	145	-20.08	907280	20	2019	1722
miR1846d-3p	LOC_Os08g08780.1	148	-21.6	148	-21.6	907300	20	2436	1207
miR1846d-3p	LOC_Os08g08790.1	168	-31.71	168	-31.71	907301	20	525	469
miR1846d-3p	LOC_Os08g09030.1	149	-22.7	149	-22.7	907327	20	429	83
miR1846d-3p	LOC_Os08g09110.1	153	-21.15	153	-21.15	907335	20	3096	2044
miR1846d-3p	LOC_Os08g09150.1	162	-24.8	162	-24.8	907339	20	3048	659
miR1846d-3p	LOC_Os08g09340.1	157	-23.85	157	-23.85	907364	20	1913	313
miR1846d-3p	LOC_Os08g09340.2	157	-23.85	157	-23.85	907365	20	2000	313

miR1846d-3p	LOC_Os08g09720.1	156	-27.55	156	-27.55	907403	20	1760	507
miR1846d-3p	LOC_Os08g10050.1	165	-28.85	165	-28.85	907443	20	2451	338
miR1846d-3p	LOC_Os08g10060.1	145	-20.08	145	-20.08	907444	20	6042	4422
miR1846d-3p	LOC_Os08g10130.1	149	-24.89	149	-24.89	907452	20	814	84
miR1846d-3p	LOC_Os08g10240.1	157	-22.89	157	-22.89	907462	20	330	301
miR1846d-3p	LOC_Os08g10520.1	144	-20.02	144	-20.02	907498	20	420	1
miR1846d-3p	LOC_Os08g11200.1	294	-46.51	150	-23.63	907569	20	5385	3678 2365
miR1846d-3p	LOC_Os08g11240.1	147	-20.45	147	-20.45	907579	20	333	146
miR1846d-3p	LOC_Os08g11480.1	304	-46.94	152	-23.87	907604	20	3496	883 1089
miR1846d-3p	LOC_Os08g11510.1	155	-25.97	155	-25.97	907607	20	240	118
miR1846d-3p	LOC_Os08g11590.1	154	-23.65	154	-23.65	907613	20	3447	178
miR1846d-3p	LOC_Os08g12040.1	154	-23.65	154	-23.65	907658	20	582	178
miR1846d-3p	LOC_Os08g12300.1	148	-21.47	148	-21.47	907683	20	5259	1361
miR1846d-3p	LOC_Os08g12680.1	157	-26.1	157	-26.1	907721	20	4721	64
miR1846d-3p	LOC_Os08g12760.1	149	-24.74	149	-24.74	907729	20	2401	2283
miR1846d-3p	LOC_Os08g12760.2	149	-24.74	149	-24.74	907730	20	2380	2262
miR1846d-3p	LOC_Os08g12760.3	149	-24.74	149	-24.74	907731	20	2326	2238
miR1846d-3p	LOC_Os08g12760.4	149	-24.74	149	-24.74	907732	20	2348	2248
miR1846d-3p	LOC_Os08g12780.1	177	-35.18	177	-35.18	907733	20	2012	1808
miR1846d-3p	LOC_Os08g12780.2	177	-35.18	177	-35.18	907734	20	2493	2289
miR1846d-3p	LOC_Os08g12780.3	177	-35.18	177	-35.18	907735	20	2488	2284
miR1846d-3p	LOC_Os08g12780.4	177	-35.18	177	-35.18	907736	20	2218	1801
miR1846d-3p	LOC_Os08g12780.5	177	-35.18	177	-35.18	907737	20	1048	844
miR1846d-3p	LOC_Os08g12830.1	145	-22.46	145	-22.46	907743	20	1932	942
miR1846d-3p	LOC_Os08g12830.2	145	-22.46	145	-22.46	907744	20	1822	942
miR1846d-3p	LOC_Os08g13060.1	152	-23.13	152	-23.13	907768	20	1504	533
miR1846d-3p	LOC_Os08g13080.1	153	-21.13	153	-21.13	907770	20	5670	1137
miR1846d-3p	LOC_Os08g13200.1	153	-26.3	153	-26.3	907782	20	774	661
miR1846d-3p	LOC_Os08g13320.1	149	-20.98	149	-20.98	907793	20	1345	422
miR1846d-3p	LOC_Os08g13500.1	149	-21.28	149	-21.28	907815	20	2184	397
miR1846d-3p	LOC_Os08g13530.1	145	-22.15	145	-22.15	907818	20	273	163
miR1846d-3p	LOC_Os08g13610.1	152	-20.54	152	-20.54	907826	20	2547	397

miR1846d-3p	LOC_Os08g13795.1	324	-58.19	169	-32.14	907845	20	828	449 244
miR1846d-3p	LOC_Os08g14010.1	148	-22.93	148	-22.93	907870	20	465	306
miR1846d-3p	LOC_Os08g14470.1	303	-43.95	156	-22.33	907915	20	6084	507 2546
miR1846d-3p	LOC_Os08g14480.1	316	-61.05	172	-36.72	907916	20	3057	1720 1742
miR1846d-3p	LOC_Os08g14550.1	147	-21.62	147	-21.62	907923	20	4701	1445
miR1846d-3p	LOC_Os08g14560.1	172	-34.12	172	-34.12	907924	20	3093	1945
miR1846d-3p	LOC_Os08g14610.1	148	-24.8	148	-24.8	907929	20	3447	2718
miR1846d-3p	LOC_Os08g14850.1	151	-20.61	151	-20.61	907952	20	4134	2773
miR1846d-3p	LOC_Os08g14860.1	162	-34.49	162	-34.49	907953	20	2067	161
miR1846d-3p	LOC_Os08g14860.2	162	-34.49	162	-34.49	907954	20	1430	161
miR1846d-3p	LOC_Os08g14860.3	162	-34.49	162	-34.49	907955	20	1390	161
miR1846d-3p	LOC_Os08g14860.4	162	-34.49	162	-34.49	907956	20	1387	161
miR1846d-3p	LOC_Os08g14860.5	162	-34.49	162	-34.49	907957	20	1474	161
miR1846d-3p	LOC_Os08g14880.3	308	-46.6	156	-25.32	907961	20	4115	135 165
miR1846d-3p	LOC_Os08g14880.4	308	-46.6	156	-25.32	907962	20	2906	135 165
miR1846d-3p	LOC_Os08g14900.1	148	-23.3	148	-23.3	907964	20	309	246
miR1846d-3p	LOC_Os08g15204.1	164	-31.01	164	-31.01	907993	20	1224	234
miR1846d-3p	LOC_Os08g15204.2	164	-31.01	164	-31.01	907994	20	1324	334
miR1846d-3p	LOC_Os08g15330.1	152	-23.91	152	-23.91	908021	20	321	192
miR1846d-3p	LOC_Os08g15920.1	143	-25.76	143	-25.76	908084	20	2173	1261
miR1846d-3p	LOC_Os08g16350.1	148	-21.02	148	-21.02	908130	20	1802	1581
miR1846d-3p	LOC_Os08g16350.2	148	-21.02	148	-21.02	908131	20	1775	1554
miR1846d-3p	LOC_Os08g16350.3	148	-21.02	148	-21.02	908132	20	1896	1675
miR1846d-3p	LOC_Os08g16470.1	159	-23.33	159	-23.33	908144	20	432	394
miR1846d-3p	LOC_Os08g16600.1	164	-33.65	164	-33.65	908160	20	1632	341
miR1846d-3p	LOC_Os08g17040.1	149	-25.27	149	-25.27	908206	20	6993	4013
miR1846d-3p	LOC_Os08g17180.1	141	-20.12	141	-20.12	908220	20	1656	822
miR1846d-3p	LOC_Os08g17400.1	159	-28.59	159	-28.59	908238	20	2554	2406
miR1846d-3p	LOC_Os08g17450.1	151	-24.69	151	-24.69	908243	20	1725	1602
miR1846d-3p	LOC_Os08g17630.1	155	-26.75	155	-26.75	908262	20	213	104
miR1846d-3p	LOC_Os08g17990.1	156	-26.34	156	-26.34	908298	20	4986	3659
miR1846d-3p	LOC_Os08g18044.1	152	-25.32	152	-25.32	908301	20	2015	601

miR1846d-3p	LOC_Os08g18044.2	152	-25.32	152	-25.32	908302	20	1444	601
miR1846d-3p	LOC_Os08g18940.1	303	-45.14	156	-23.52	908344	20	6084	507 2546
miR1846d-3p	LOC_Os08g18950.1	164	-30.23	164	-30.23	908345	20	3039	1807
miR1846d-3p	LOC_Os08g19210.1	305	-51.2	156	-26.36	908376	20	1515	630 246
miR1846d-3p	LOC_Os08g19210.2	305	-51.2	156	-26.36	908377	20	1518	600 216
miR1846d-3p	LOC_Os08g19350.1	295	-41.91	148	-21.61	908392	20	6084	507 2546
miR1846d-3p	LOC_Os08g19500.1	166	-26.59	166	-26.59	908410	20	3060	659
miR1846d-3p	LOC_Os08g19850.1	150	-24.61	150	-24.61	908449	20	2498	2151
miR1846d-3p	LOC_Os08g20210.1	146	-20.01	146	-20.01	908486	20	3435	17
miR1846d-3p	LOC_Os08g20280.1	166	-26.59	166	-26.59	908493	20	2166	659
miR1846d-3p	LOC_Os08g20800.1	146	-21.78	146	-21.78	908540	20	2508	1484
miR1846d-3p	LOC_Os08g21110.1	156	-21.74	156	-21.74	908559	20	6117	5741
miR1846d-3p	LOC_Os08g21220.1	295	-41.79	148	-21.62	908567	20	6084	507 2546
miR1846d-3p	LOC_Os08g21230.1	172	-35.42	172	-35.42	908568	20	3150	1816
miR1846d-3p	LOC_Os08g21930.1	155	-23.43	155	-23.43	908634	20	2193	2045
miR1846d-3p	LOC_Os08g22380.1	148	-22.32	148	-22.32	908681	20	2895	1269
miR1846d-3p	LOC_Os08g23160.1	150	-21.43	150	-21.43	908754	20	1119	72
miR1846d-3p	LOC_Os08g23260.1	152	-28.36	152	-28.36	908764	20	1065	519
miR1846d-3p	LOC_Os08g23300.1	166	-26.29	166	-26.29	908768	20	3144	659
miR1846d-3p	LOC_Os08g23500.1	156	-28.42	156	-28.42	908790	20	441	51
miR1846d-3p	LOC_Os08g23620.1	148	-21.47	148	-21.47	908802	20	1572	626
miR1846d-3p	LOC_Os08g23630.1	301	-45.61	154	-23.99	908803	20	4875	507 2285
miR1846d-3p	LOC_Os08g23760.1	166	-26.29	166	-26.29	908819	20	3135	659
miR1846d-3p	LOC_Os08g23910.1	142	-22.55	142	-22.55	908835	20	2910	1871
miR1846d-3p	LOC_Os08g23930.1	160	-27.53	160	-27.53	908837	20	2206	197
miR1846d-3p	LOC_Os08g23930.2	160	-27.53	160	-27.53	908838	20	2203	197
miR1846d-3p	LOC_Os08g24270.1	145	-20.08	145	-20.08	908871	20	5925	4356
miR1846d-3p	LOC_Os08g24290.1	151	-21.68	151	-21.68	908873	20	2220	1518
miR1846d-3p	LOC_Os08g24390.1	149	-20.45	149	-20.45	908883	20	2197	1713
miR1846d-3p	LOC_Os08g24390.2	149	-20.45	149	-20.45	908884	20	2265	1796
miR1846d-3p	LOC_Os08g24390.3	149	-20.45	149	-20.45	908885	20	1102	595
miR1846d-3p	LOC_Os08g24390.4	149	-20.45	149	-20.45	908886	20	2013	1529

miR1846d-3p	LOC_Os08g24410.1	171	-36.05	171	-36.05	908888	20	4146	2198
miR1846d-3p	LOC_Os08g24434.1	157	-26.8	157	-26.8	908890	20	1618	1277
miR1846d-3p	LOC_Os08g24530.1	156	-28.42	156	-28.42	908898	20	3084	2732
miR1846d-3p	LOC_Os08g24640.1	157	-23.73	157	-23.73	908909	20	2436	338
miR1846d-3p	LOC_Os08g24660.1	165	-32.76	165	-32.76	908911	20	780	240
miR1846d-3p	LOC_Os08g24720.1	166	-26.59	166	-26.59	908917	20	2793	659
miR1846d-3p	LOC_Os08g24980.1	144	-21.33	144	-21.33	908941	20	3747	376
miR1846d-3p	LOC_Os08g25080.1	156	-31.23	156	-31.23	908951	20	918	233
miR1846d-3p	LOC_Os08g25080.2	156	-31.23	156	-31.23	908952	20	904	233
miR1846d-3p	LOC_Os08g25110.1	156	-23.52	156	-23.52	908956	20	6087	510
miR1846d-3p	LOC_Os08g25140.1	164	-29.12	164	-29.12	908960	20	1279	471
miR1846d-3p	LOC_Os08g25330.1	151	-21.79	151	-21.79	908976	20	4575	3666
miR1846d-3p	LOC_Os08g25710.1	158	-24.56	158	-24.56	909017	20	3680	2165
miR1846d-3p	LOC_Os08g25760.1	143	-20.27	143	-20.27	909022	20	843	767
miR1846d-3p	LOC_Os08g25780.1	148	-24.8	148	-24.8	909024	20	474	35
miR1846d-3p	LOC_Os08g25900.1	151	-31.21	151	-31.21	909036	20	1192	764
miR1846d-3p	LOC_Os08g25940.1	303	-43.57	156	-21.95	909037	20	6009	507 2471
miR1846d-3p	LOC_Os08g26040.1	156	-23.58	156	-23.58	909046	20	702	420
miR1846d-3p	LOC_Os08g26090.1	142	-22.55	142	-22.55	909051	20	3288	1970
miR1846d-3p	LOC_Os08g26490.1	154	-24.31	154	-24.31	909096	20	738	157
miR1846d-3p	LOC_Os08g26530.1	607	-97.22	152	-25.8	909100	20	618	97 306 365 198
miR1846d-3p	LOC_Os08g26700.1	156	-23.57	156	-23.57	909114	20	1241	984
miR1846d-3p	LOC_Os08g26700.2	156	-23.57	156	-23.57	909115	20	1320	1070
miR1846d-3p	LOC_Os08g26700.3	156	-23.57	156	-23.57	909116	20	1317	1067
miR1846d-3p	LOC_Os08g26930.1	153	-22.46	153	-22.46	909145	20	2313	379
miR1846d-3p	LOC_Os08g27270.1	157	-23.73	157	-23.73	909180	20	2436	338
miR1846d-3p	LOC_Os08g27280.1	145	-20.08	145	-20.08	909181	20	5892	4275
miR1846d-3p	LOC_Os08g27320.1	145	-20.08	145	-20.08	909185	20	2310	1173
miR1846d-3p	LOC_Os08g27470.1	645	-102.61	170	-27.44	909200	20	312	225 16 163 94
miR1846d-3p	LOC_Os08g27810.1	161	-31.07	161	-31.07	909237	20	1277	409
miR1846d-3p	LOC_Os08g27950.1	178	-35.35	178	-35.35	909252	20	963	672
miR1846d-3p	LOC_Os08g28380.1	147	-20.45	147	-20.45	909298	20	156	6

miR1846d-3p	LOC_Os08g28680.1	159	-28.7	159	-28.7	909326	20	1266	1099
miR1846d-3p	LOC_Os08g28784.1	153	-26.26	153	-26.26	909338	20	885	240
miR1846d-3p	LOC_Os08g28810.1	165	-37.11	165	-37.11	909341	20	318	163
miR1846d-3p	LOC_Os08g28850.1	152	-25.21	152	-25.21	909345	20	777	454
miR1846d-3p	LOC_Os08g29320.1	155	-21.2	155	-21.2	909393	20	351	275
miR1846d-3p	LOC_Os08g29615.1	151	-27.26	151	-27.26	909426	20	2608	264
miR1846d-3p	LOC_Os08g29669.1	152	-25.66	152	-25.66	909431	20	2000	1796
miR1846d-3p	LOC_Os08g29930.1	145	-20.08	145	-20.08	909454	20	5988	4356
miR1846d-3p	LOC_Os08g30060.1	162	-31.34	162	-31.34	909472	20	2387	111
miR1846d-3p	LOC_Os08g30490.1	161	-29.47	161	-29.47	909515	20	1190	217
miR1846d-3p	LOC_Os08g30719.1	152	-20.85	152	-20.85	909537	20	851	283
miR1846d-3p	LOC_Os08g30770.1	157	-23.32	157	-23.32	909543	20	3444	65
miR1846d-3p	LOC_Os08g31070.1	162	-35.25	162	-35.25	909572	20	990	326
miR1846d-3p	LOC_Os08g31830.1	141	-20.1	141	-20.1	909658	20	848	321
miR1846d-3p	LOC_Os08g31850.1	160	-27.42	160	-27.42	909663	20	1552	99
miR1846d-3p	LOC_Os08g32020.1	151	-24.69	151	-24.69	909681	20	2346	2046
miR1846d-3p	LOC_Os08g32060.1	161	-31.17	161	-31.17	909685	20	2434	99
miR1846d-3p	LOC_Os08g32260.1	141	-22.84	141	-22.84	909707	20	2913	619
miR1846d-3p	LOC_Os08g32530.1	140	-20.04	140	-20.04	909734	20	891	519
miR1846d-3p	LOC_Os08g32620.1	155	-27.14	155	-27.14	909743	20	1209	17
miR1846d-3p	LOC_Os08g32620.2	155	-27.14	155	-27.14	909744	20	1340	17
miR1846d-3p	LOC_Os08g32620.3	155	-27.14	155	-27.14	909745	20	1191	9
miR1846d-3p	LOC_Os08g32620.4	155	-27.14	155	-27.14	909746	20	1327	17
miR1846d-3p	LOC_Os08g32710.1	155	-20.76	155	-20.76	909757	20	240	83
miR1846d-3p	LOC_Os08g32760.1	156	-23.52	156	-23.52	909762	20	6087	510
miR1846d-3p	LOC_Os08g32830.1	142	-22.55	142	-22.55	909769	20	3288	1970
miR1846d-3p	LOC_Os08g33060.1	166	-26.29	166	-26.29	909796	20	2724	311
miR1846d-3p	LOC_Os08g33076.1	148	-24.17	148	-24.17	909798	20	2060	123
miR1846d-3p	LOC_Os08g33090.1	145	-23.48	145	-23.48	909800	20	1941	1600
miR1846d-3p	LOC_Os08g33150.1	151	-21.54	151	-21.54	909809	20	1329	375
miR1846d-3p	LOC_Os08g33300.1	145	-22.64	145	-22.64	909825	20	1605	1476
miR1846d-3p	LOC_Os08g33820.1	156	-26.71	156	-26.71	909886	20	1282	730

miR1846d-3p	LOC_Os08g33970.1	152	-22.43	152	-22.43	909903	20	294	105
miR1846d-3p	LOC_Os08g34010.1	157	-23.18	157	-23.18	909906	20	1086	770
miR1846d-3p	LOC_Os08g34060.1	302	-49.8	154	-27.3	909912	20	3310	412 166
miR1846d-3p	LOC_Os08g34060.2	302	-49.8	154	-27.3	909913	20	3249	412 166
miR1846d-3p	LOC_Os08g34060.3	302	-49.8	154	-27.3	909914	20	3281	412 166
miR1846d-3p	LOC_Os08g34060.4	154	-22.5	154	-22.5	909915	20	3479	610
miR1846d-3p	LOC_Os08g34240.1	160	-29.11	160	-29.11	909935	20	1869	1353
miR1846d-3p	LOC_Os08g34249.1	149	-24.24	149	-24.24	909936	20	201	113
miR1846d-3p	LOC_Os08g34258.1	149	-24.24	149	-24.24	909937	20	201	113
miR1846d-3p	LOC_Os08g34310.1	161	-25.86	161	-25.86	909943	20	1386	1274
miR1846d-3p	LOC_Os08g34420.1	152	-23.62	152	-23.62	909954	20	3798	1434
miR1846d-3p	LOC_Os08g34490.1	146	-20.01	146	-20.01	909961	20	1926	1400
miR1846d-3p	LOC_Os08g34630.1	145	-20.49	145	-20.49	909977	20	888	354
miR1846d-3p	LOC_Os08g34850.1	147	-24.28	147	-24.28	909999	20	4902	3752
miR1846d-3p	LOC_Os08g34879.1	149	-24.65	149	-24.65	910002	20	1293	1128
miR1846d-3p	LOC_Os08g34950.1	157	-23.32	157	-23.32	910010	20	6347	313
miR1846d-3p	LOC_Os08g35170.1	146	-20.01	146	-20.01	910040	20	4101	233
miR1846d-3p	LOC_Os08g35190.1	159	-24.73	159	-24.73	910042	20	1146	283
miR1846d-3p	LOC_Os08g35190.2	159	-24.73	159	-24.73	910043	20	1502	283
miR1846d-3p	LOC_Os08g35319.1	152	-21.75	152	-21.75	910060	20	1212	717
miR1846d-3p	LOC_Os08g35380.1	158	-22.95	158	-22.95	910065	20	2301	14
miR1846d-3p	LOC_Os08g35610.1	153	-25.56	153	-25.56	910088	20	885	661
miR1846d-3p	LOC_Os08g35700.1	160	-28.77	160	-28.77	910098	20	3091	2685
miR1846d-3p	LOC_Os08g35710.1	156	-24.06	156	-24.06	910099	20	1184	994
miR1846d-3p	LOC_Os08g35740.1	152	-21.26	152	-21.26	910102	20	1624	138
miR1846d-3p	LOC_Os08g35740.2	152	-21.26	152	-21.26	910103	20	1483	138
miR1846d-3p	LOC_Os08g35750.1	148	-21.14	148	-21.14	910104	20	976	591
miR1846d-3p	LOC_Os08g35800.1	166	-26.59	166	-26.59	910109	20	2574	659
miR1846d-3p	LOC_Os08g35939.1	150	-26.73	150	-26.73	910125	20	1744	1290
miR1846d-3p	LOC_Os08g36170.1	152	-27.41	152	-27.41	910152	20	1640	1370
miR1846d-3p	LOC_Os08g36280.1	142	-22.55	142	-22.55	910161	20	3273	1970
miR1846d-3p	LOC_Os08g36500.1	156	-24.76	156	-24.76	910190	20	3279	2926

miR1846d-3p	LOC_Os08g36930.1	147	-21.62	147	-21.62	910236	20	5925	2546
miR1846d-3p	LOC_Os08g36940.1	172	-34.12	172	-34.12	910237	20	3165	1828
miR1846d-3p	LOC_Os08g37080.1	282	-42.49	142	-22.15	910251	20	702	109 447
miR1846d-3p	LOC_Os08g37260.1	156	-27.21	156	-27.21	910266	20	1992	1461
miR1846d-3p	LOC_Os08g37260.2	156	-27.21	156	-27.21	910267	20	1623	1092
miR1846d-3p	LOC_Os08g37550.1	166	-26.59	166	-26.59	910307	20	3081	659
miR1846d-3p	LOC_Os08g37874.1	162	-31.45	162	-31.45	910347	20	1983	155
miR1846d-3p	LOC_Os08g37874.2	162	-31.45	162	-31.45	910348	20	1970	171
miR1846d-3p	LOC_Os08g37874.3	162	-31.45	162	-31.45	910349	20	1630	361
miR1846d-3p	LOC_Os08g38480.1	150	-22.94	150	-22.94	910429	20	1915	774
miR1846d-3p	LOC_Os08g38520.1	157	-23.86	157	-23.86	910433	20	846	555
miR1846d-3p	LOC_Os08g38710.1	162	-28.31	162	-28.31	910454	20	2727	663
miR1846d-3p	LOC_Os08g38980.1	156	-22.78	156	-22.78	910493	20	2805	844
miR1846d-3p	LOC_Os08g39030.1	144	-24.97	144	-24.97	910500	20	1384	247
miR1846d-3p	LOC_Os08g39090.1	160	-28.82	160	-28.82	910509	20	1951	286
miR1846d-3p	LOC_Os08g39220.1	174	-34.34	174	-34.34	910525	20	2193	702
miR1846d-3p	LOC_Os08g39280.1	149	-20.42	149	-20.42	910532	20	993	382
miR1846d-3p	LOC_Os08g39340.1	145	-23.41	145	-23.41	910541	20	741	192
miR1846d-3p	LOC_Os08g39470.1	153	-25.95	153	-25.95	910556	20	456	103
miR1846d-3p	LOC_Os08g39630.1	146	-21.83	146	-21.83	910574	20	2607	920
miR1846d-3p	LOC_Os08g39680.1	145	-24.27	145	-24.27	910579	20	801	634
miR1846d-3p	LOC_Os08g39770.1	151	-24.69	151	-24.69	910589	20	735	435
miR1846d-3p	LOC_Os08g39970.1	154	-22	154	-22	910616	20	1644	177
miR1846d-3p	LOC_Os08g39980.1	152	-27.05	152	-27.05	910617	20	2545	1780
miR1846d-3p	LOC_Os08g40110.2	147	-20.81	147	-20.81	910631	20	1750	1304
miR1846d-3p	LOC_Os08g40150.2	147	-20.41	147	-20.41	910637	20	2328	1872
miR1846d-3p	LOC_Os08g40270.1	165	-30.85	165	-30.85	910650	20	2220	1997
miR1846d-3p	LOC_Os08g40580.1	153	-24	153	-24	910682	20	1082	1024
miR1846d-3p	LOC_Os08g40580.2	153	-24	153	-24	910683	20	970	912
miR1846d-3p	LOC_Os08g40580.3	153	-24	153	-24	910684	20	1028	970
miR1846d-3p	LOC_Os08g40580.4	153	-24	153	-24	910685	20	1060	1002
miR1846d-3p	LOC_Os08g40800.1	305	-50.53	158	-28.91	910714	20	5826	507 2546

miR1846d-3p	LOC_Os08g40900.1	146	-20.77	146	-20.77	910728	20	4066	3984
miR1846d-3p	LOC_Os08g40960.1	147	-21.62	147	-21.62	910736	20	4605	2222
miR1846d-3p	LOC_Os08g40990.1	150	-24.19	150	-24.19	910739	20	2594	1654
miR1846d-3p	LOC_Os08g41010.1	165	-29.01	165	-29.01	910741	20	1862	79
miR1846d-3p	LOC_Os08g41010.2	165	-29.01	165	-29.01	910742	20	1789	68
miR1846d-3p	LOC_Os08g41010.3	165	-29.01	165	-29.01	910743	20	1859	79
miR1846d-3p	LOC_Os08g41010.4	165	-29.01	165	-29.01	910744	20	2016	79
miR1846d-3p	LOC_Os08g41010.5	165	-29.01	165	-29.01	910745	20	1851	79
miR1846d-3p	LOC_Os08g41010.6	165	-29.01	165	-29.01	910746	20	1848	79
miR1846d-3p	LOC_Os08g41010.7	165	-29.01	165	-29.01	910747	20	1839	79
miR1846d-3p	LOC_Os08g41020.1	154	-22.52	154	-22.52	910748	20	4320	4091
miR1846d-3p	LOC_Os08g41120.1	148	-20.11	148	-20.11	910757	20	1026	354
miR1846d-3p	LOC_Os08g41250.1	148	-20.14	148	-20.14	910773	20	1270	1003
miR1846d-3p	LOC_Os08g41250.2	148	-20.14	148	-20.14	910774	20	1376	1003
miR1846d-3p	LOC_Os08g41630.1	152	-25.23	152	-25.23	910820	20	3265	2972
miR1846d-3p	LOC_Os08g41680.1	155	-30.42	155	-30.42	910824	20	596	545
miR1846d-3p	LOC_Os08g42020.1	145	-20.07	145	-20.07	910860	20	2258	1625
miR1846d-3p	LOC_Os08g42020.2	145	-20.07	145	-20.07	910861	20	2206	1625
miR1846d-3p	LOC_Os08g42090.1	146	-20.01	146	-20.01	910868	20	3963	17
miR1846d-3p	LOC_Os08g42130.1	166	-26.59	166	-26.59	910872	20	3057	599
miR1846d-3p	LOC_Os08g42390.1	153	-22.55	153	-22.55	910901	20	1507	55
miR1846d-3p	LOC_Os08g42560.1	155	-24.61	155	-24.61	910931	20	2651	103
miR1846d-3p	LOC_Os08g42580.1	145	-23.2	145	-23.2	910933	20	2604	2009
miR1846d-3p	LOC_Os08g42580.4	145	-23.2	145	-23.2	910934	20	2492	1897
miR1846d-3p	LOC_Os08g42650.1	305	-50.53	158	-28.91	910944	20	4953	168 2075
miR1846d-3p	LOC_Os08g43000.1	150	-21.38	150	-21.38	910987	20	3633	625
miR1846d-3p	LOC_Os08g43310.1	163	-34.06	163	-34.06	911022	20	507	196
miR1846d-3p	LOC_Os08g43430.1	162	-24.88	162	-24.88	911039	20	1661	547
miR1846d-3p	LOC_Os08g43670.1	161	-26.16	161	-26.16	911068	20	1304	681
miR1846d-3p	LOC_Os08g44030.1	148	-22.04	148	-22.04	911108	20	327	157
miR1846d-3p	LOC_Os08g44260.1	148	-20.09	148	-20.09	911130	20	3118	2257
miR1846d-3p	LOC_Os08g44260.2	148	-20.09	148	-20.09	911131	20	4437	3576

miR1846d-3p	LOC_Os08g44490.1	166	-27.37	166	-27.37	911168	20	3015	659
miR1846d-3p	LOC_Os08g44570.1	147	-21.62	147	-21.62	911178	20	6093	2555
miR1846d-3p	LOC_Os08g44580.1	172	-35.42	172	-35.42	911179	20	3054	1717
miR1846d-3p	LOC_Os08g44700.1	147	-21.62	147	-21.62	911192	20	6084	2546
miR1846d-3p	LOC_Os08g44710.1	172	-35.42	172	-35.42	911193	20	3252	1873
miR1846d-3p	LOC_Os08g44750.1	288	-46.65	147	-23.63	911197	20	1622	920 1196
miR1846d-3p	LOC_Os08g44780.1	152	-25.58	152	-25.58	911203	20	1700	450
miR1846d-3p	LOC_Os08g45090.1	161	-24.85	161	-24.85	911249	20	570	471
miR1846d-3p	LOC_Os09g01000.1	147	-23.55	147	-23.55	911279	20	2927	2723
miR1846d-3p	LOC_Os09g01350.1	148	-23.28	148	-23.28	911318	20	2463	1263
miR1846d-3p	LOC_Os09g01540.1	152	-21.82	152	-21.82	911338	20	2583	1270
miR1846d-3p	LOC_Os09g01560.1	145	-20.02	145	-20.02	911340	20	5835	4215
miR1846d-3p	LOC_Os09g01600.1	141	-20.14	141	-20.14	911344	20	2432	1886
miR1846d-3p	LOC_Os09g01600.2	141	-20.14	141	-20.14	911345	20	2263	1882
miR1846d-3p	LOC_Os09g01600.3	141	-20.14	141	-20.14	911346	20	2265	1886
miR1846d-3p	LOC_Os09g01600.4	141	-20.14	141	-20.14	911347	20	2188	1883
miR1846d-3p	LOC_Os09g01810.1	166	-30.42	166	-30.42	911372	20	2481	338
miR1846d-3p	LOC_Os09g02150.1	150	-24.28	150	-24.28	911405	20	651	355
miR1846d-3p	LOC_Os09g02200.1	151	-24.52	151	-24.52	911410	20	297	67
miR1846d-3p	LOC_Os09g02300.1	153	-22.35	153	-22.35	911422	20	1238	617
miR1846d-3p	LOC_Os09g02370.1	155	-29.99	155	-29.99	911429	20	807	579
miR1846d-3p	LOC_Os09g02460.1	151	-21.79	151	-21.79	911441	20	3546	2694
miR1846d-3p	LOC_Os09g02620.1	161	-27.96	161	-27.96	911455	20	231	123
miR1846d-3p	LOC_Os09g02760.1	146	-20.01	146	-20.01	911468	20	3981	17
miR1846d-3p	LOC_Os09g02830.1	156	-28.21	156	-28.21	911478	20	627	551
miR1846d-3p	LOC_Os09g03180.1	141	-20.9	141	-20.9	911512	20	2883	314
miR1846d-3p	LOC_Os09g03230.1	146	-20.58	146	-20.58	911517	20	1141	240
miR1846d-3p	LOC_Os09g03939.1	156	-24.9	156	-24.9	911585	20	2495	2312
miR1846d-3p	LOC_Os09g03939.2	156	-24.9	156	-24.9	911586	20	1948	1760
miR1846d-3p	LOC_Os09g03980.1	152	-24.78	152	-24.78	911589	20	2595	176
miR1846d-3p	LOC_Os09g04070.1	147	-21.62	147	-21.62	911597	20	6015	2546
miR1846d-3p	LOC_Os09g04080.1	315	-57.69	172	-34.12	911598	20	3105	1816 1838

miR1846d-3p	LOC_Os09g04290.1	319	-55.24	160	-28.99	911622	20	1783	888 594
miR1846d-3p	LOC_Os09g04290.2	319	-55.24	160	-28.99	911623	20	1787	888 594
miR1846d-3p	LOC_Os09g04300.1	165	-32.53	165	-32.53	911624	20	1766	851
miR1846d-3p	LOC_Os09g04300.2	165	-32.53	165	-32.53	911625	20	2590	851
miR1846d-3p	LOC_Os09g04300.3	165	-32.53	165	-32.53	911626	20	2886	851
miR1846d-3p	LOC_Os09g04540.1	148	-20.15	148	-20.15	911652	20	2067	966
miR1846d-3p	LOC_Os09g04740.1	151	-21.79	151	-21.79	911672	20	4383	3510
miR1846d-3p	LOC_Os09g04940.1	162	-24.8	162	-24.8	911694	20	3015	659
miR1846d-3p	LOC_Os09g06950.1	301	-49.03	157	-27.59	911795	20	800	98 276
miR1846d-3p	LOC_Os09g07090.1	140	-20.04	140	-20.04	911811	20	1815	1179
miR1846d-3p	LOC_Os09g07250.1	149	-21.28	149	-21.28	911828	20	1977	325
miR1846d-3p	LOC_Os09g07270.1	150	-24.06	150	-24.06	911830	20	3726	2946
miR1846d-3p	LOC_Os09g07510.1	160	-28.96	160	-28.96	911860	20	2370	2191
miR1846d-3p	LOC_Os09g07510.2	170	-32.37	170	-32.37	911861	20	2348	2179
miR1846d-3p	LOC_Os09g07510.3	160	-28.96	160	-28.96	911862	20	2344	2165
miR1846d-3p	LOC_Os09g07750.1	305	-50.53	158	-28.91	911888	20	5253	507 2546
miR1846d-3p	LOC_Os09g08480.1	144	-22.09	144	-22.09	911968	20	837	16
miR1846d-3p	LOC_Os09g08525.1	150	-23.62	150	-23.62	911973	20	1125	415
miR1846d-3p	LOC_Os09g08710.2	156	-24.46	156	-24.46	911991	20	909	298
miR1846d-3p	LOC_Os09g08710.3	156	-24.46	156	-24.46	911992	20	991	298
miR1846d-3p	LOC_Os09g08790.1	151	-23.12	151	-23.12	912000	20	2793	1791
miR1846d-3p	LOC_Os09g08920.1	146	-25.94	146	-25.94	912012	20	610	567
miR1846d-3p	LOC_Os09g08990.1	146	-25.94	146	-25.94	912019	20	1910	860
miR1846d-3p	LOC_Os09g09320.1	151	-22.26	151	-22.26	912051	20	1403	410
miR1846d-3p	LOC_Os09g09320.2	151	-22.26	151	-22.26	912052	20	828	412
miR1846d-3p	LOC_Os09g09360.1	146	-24.13	146	-24.13	912056	20	3939	256
miR1846d-3p	LOC_Os09g09560.1	145	-24.95	145	-24.95	912080	20	1254	646
miR1846d-3p	LOC_Os09g09760.1	156	-25.45	156	-25.45	912099	20	5889	168
miR1846d-3p	LOC_Os09g09790.1	318	-48.13	161	-26.83	912102	20	2166	2080 1761
miR1846d-3p	LOC_Os09g09870.1	145	-20.08	145	-20.08	912110	20	5934	4197
miR1846d-3p	LOC_Os09g09944.1	140	-23.02	140	-23.02	912118	20	828	404
miR1846d-3p	LOC_Os09g10080.1	152	-21.82	152	-21.82	912128	20	2484	1252

miR1846d-3p	LOC_Os09g10201.1	157	-25.31	157	-25.31	912141	20	919	589
miR1846d-3p	LOC_Os09g10300.1	144	-22.71	144	-22.71	912153	20	659	161
miR1846d-3p	LOC_Os09g10420.1	148	-21.44	148	-21.44	912165	20	1188	227
miR1846d-3p	LOC_Os09g10900.1	144	-23.01	144	-23.01	912215	20	3045	595
miR1846d-3p	LOC_Os09g11020.1	147	-21.52	147	-21.52	912231	20	3495	3293
miR1846d-3p	LOC_Os09g11040.1	147	-20.15	147	-20.15	912233	20	5352	1434
miR1846d-3p	LOC_Os09g11270.1	142	-20.63	142	-20.63	912256	20	2458	993
miR1846d-3p	LOC_Os09g11460.1	152	-26.14	152	-26.14	912275	20	1398	895
miR1846d-3p	LOC_Os09g11460.2	152	-26.14	152	-26.14	912276	20	1896	1393
miR1846d-3p	LOC_Os09g11530.1	153	-20.87	153	-20.87	912284	20	828	780
miR1846d-3p	LOC_Os09g11630.1	147	-21.62	147	-21.62	912294	20	5973	2546
miR1846d-3p	LOC_Os09g11720.1	153	-20.87	153	-20.87	912303	20	852	417
miR1846d-3p	LOC_Os09g11940.1	140	-20.35	140	-20.35	912325	20	4578	1868
miR1846d-3p	LOC_Os09g12130.1	305	-50.53	158	-28.91	912341	20	5829	507 2489
miR1846d-3p	LOC_Os09g12350.1	147	-22.09	147	-22.09	912362	20	2001	937
miR1846d-3p	LOC_Os09g12900.1	313	-49.61	157	-25.15	912424	20	276	125 200
miR1846d-3p	LOC_Os09g13760.1	156	-22.71	156	-22.71	912512	20	246	190
miR1846d-3p	LOC_Os09g13850.1	158	-28.91	158	-28.91	912520	20	1398	507
miR1846d-3p	LOC_Os09g13900.1	152	-26.85	152	-26.85	912528	20	2595	1195
miR1846d-3p	LOC_Os09g14410.1	177	-36.6	177	-36.6	912580	20	3784	3623
miR1846d-3p	LOC_Os09g14450.1	160	-27.48	160	-27.48	912583	20	3177	183
miR1846d-3p	LOC_Os09g14580.1	156	-25.77	156	-25.77	912598	20	1509	1149
miR1846d-3p	LOC_Os09g14710.1	141	-20.25	141	-20.25	912617	20	2223	2029
miR1846d-3p	LOC_Os09g14800.1	303	-45.14	156	-23.52	912626	20	6084	507 2546
miR1846d-3p	LOC_Os09g14830.1	147	-21.62	147	-21.62	912629	20	6084	2546
miR1846d-3p	LOC_Os09g14950.1	149	-20.24	149	-20.24	912641	20	483	418
miR1846d-3p	LOC_Os09g14960.1	150	-21.24	150	-21.24	912642	20	2761	2705
miR1846d-3p	LOC_Os09g14960.2	150	-21.24	150	-21.24	912643	20	2687	2631
miR1846d-3p	LOC_Os09g15010.1	156	-23.89	156	-23.89	912648	20	3999	3449
miR1846d-3p	LOC_Os09g15044.1	316	-53.13	159	-30.41	912652	20	1006	711 341
miR1846d-3p	LOC_Os09g15090.1	147	-21.91	147	-21.91	912656	20	2514	1981
miR1846d-3p	LOC_Os09g15250.1	147	-22.71	147	-22.71	912671	20	5211	4952

miR1846d-3p	LOC_Os09g15270.1	159	-35.68	159	-35.68	912673	20	4674	1842
miR1846d-3p	LOC_Os09g15284.1	158	-25.37	158	-25.37	912675	20	886	628
miR1846d-3p	LOC_Os09g15389.1	144	-25.65	144	-25.65	912690	20	2292	1963
miR1846d-3p	LOC_Os09g15389.2	144	-25.65	144	-25.65	912691	20	2428	2109
miR1846d-3p	LOC_Os09g15490.1	151	-25.17	151	-25.17	912704	20	291	51
miR1846d-3p	LOC_Os09g15540.1	169	-35.51	169	-35.51	912709	20	1609	492
miR1846d-3p	LOC_Os09g15980.1	147	-20.06	147	-20.06	912756	20	1482	757
miR1846d-3p	LOC_Os09g15980.2	147	-20.06	147	-20.06	912757	20	1105	757
miR1846d-3p	LOC_Os09g16270.1	162	-28.03	162	-28.03	912785	20	609	329
miR1846d-3p	LOC_Os09g16330.2	142	-21.21	142	-21.21	912791	20	4341	3225
miR1846d-3p	LOC_Os09g16390.1	144	-20.57	144	-20.57	912797	20	513	189
miR1846d-3p	LOC_Os09g16430.1	151	-21.79	151	-21.79	912801	20	3786	2844
miR1846d-3p	LOC_Os09g16510.1	165	-27.98	165	-27.98	912811	20	1774	1058
miR1846d-3p	LOC_Os09g16550.1	161	-27.86	161	-27.86	912815	20	2166	1573
miR1846d-3p	LOC_Os09g16760.1	151	-27.3	151	-27.3	912836	20	2543	155
miR1846d-3p	LOC_Os09g16995.1	153	-26.1	153	-26.1	912860	20	3285	1542
miR1846d-3p	LOC_Os09g17049.1	153	-24.71	153	-24.71	912867	20	2637	285
miR1846d-3p	LOC_Os09g17360.1	151	-26.94	151	-26.94	912897	20	1905	1267
miR1846d-3p	LOC_Os09g17380.1	166	-26.59	166	-26.59	912899	20	2328	659
miR1846d-3p	LOC_Os09g17540.1	153	-25.22	153	-25.22	912915	20	1532	1230
miR1846d-3p	LOC_Os09g19136.1	147	-21.48	147	-21.48	913021	20	1203	39
miR1846d-3p	LOC_Os09g19274.1	157	-24.16	157	-24.16	913034	20	2549	239
miR1846d-3p	LOC_Os09g19440.1	151	-25.37	151	-25.37	913050	20	309	152
miR1846d-3p	LOC_Os09g19530.1	156	-23.52	156	-23.52	913059	20	6054	507
miR1846d-3p	LOC_Os09g19540.1	144	-24.33	144	-24.33	913060	20	3144	1829
miR1846d-3p	LOC_Os09g19600.1	148	-21.6	148	-21.6	913066	20	2436	1207
miR1846d-3p	LOC_Os09g20090.1	143	-22.02	143	-22.02	913122	20	2309	1823
miR1846d-3p	LOC_Os09g20420.1	311	-52.08	160	-27.41	913157	20	1283	1017 671
miR1846d-3p	LOC_Os09g20450.1	141	-21.97	141	-21.97	913160	20	1997	1004
miR1846d-3p	LOC_Os09g20460.1	147	-23.96	147	-23.96	913161	20	1838	1525
miR1846d-3p	LOC_Os09g20480.1	165	-28.47	165	-28.47	913163	20	1964	1356
miR1846d-3p	LOC_Os09g20610.1	145	-23.48	145	-23.48	913178	20	1395	756

miR1846d-3p	LOC_Os09g20720.1	148	-22.75	148	-22.75	913195	20	480	357
miR1846d-3p	LOC_Os09g20870.1	148	-25.46	148	-25.46	913212	20	360	235
miR1846d-3p	LOC_Os09g20930.1	153	-26.7	153	-26.7	913219	20	1757	455
miR1846d-3p	LOC_Os09g20960.1	160	-31.33	160	-31.33	913222	20	276	88
miR1846d-3p	LOC_Os09g20970.1	151	-25.23	151	-25.23	913223	20	2064	253
miR1846d-3p	LOC_Os09g21130.1	151	-24.74	151	-24.74	913239	20	630	46
miR1846d-3p	LOC_Os09g21160.1	156	-28.92	156	-28.92	913242	20	1654	1063
miR1846d-3p	LOC_Os09g21170.1	156	-28.92	156	-28.92	913243	20	621	415
miR1846d-3p	LOC_Os09g21310.1	151	-24.69	151	-24.69	913259	20	1854	1554
miR1846d-3p	LOC_Os09g21500.1	152	-22.52	152	-22.52	913277	20	375	97
miR1846d-3p	LOC_Os09g21600.1	165	-28.85	165	-28.85	913288	20	2499	338
miR1846d-3p	LOC_Os09g21610.1	145	-20.08	145	-20.08	913289	20	6042	4422
miR1846d-3p	LOC_Os09g21802.1	149	-23.18	149	-23.18	913311	20	1906	1637
miR1846d-3p	LOC_Os09g22190.1	157	-25.84	157	-25.84	913349	20	651	345
miR1846d-3p	LOC_Os09g22390.1	142	-23.38	142	-23.38	913367	20	264	198
miR1846d-3p	LOC_Os09g22430.1	304	-50.17	157	-28.76	913371	20	1020	267 715
miR1846d-3p	LOC_Os09g22505.1	160	-26	160	-26	913379	20	1134	317
miR1846d-3p	LOC_Os09g23070.1	147	-24.8	147	-24.8	913387	20	2042	1277
miR1846d-3p	LOC_Os09g23070.2	147	-24.8	147	-24.8	913388	20	2169	1367
miR1846d-3p	LOC_Os09g23100.1	146	-20.01	146	-20.01	913390	20	3963	17
miR1846d-3p	LOC_Os09g23210.1	152	-22.43	152	-22.43	913403	20	519	99
miR1846d-3p	LOC_Os09g23640.1	310	-52.96	156	-28.35	913450	20	2625	1029 458
miR1846d-3p	LOC_Os09g23740.1	147	-21.85	147	-21.85	913462	20	5435	905
miR1846d-3p	LOC_Os09g23790.1	147	-21.61	147	-21.61	913467	20	6093	2555
miR1846d-3p	LOC_Os09g23800.1	172	-35.42	172	-35.42	913468	20	3168	1831
miR1846d-3p	LOC_Os09g24480.1	308	-48.03	160	-24.7	913538	20	777	493 265
miR1846d-3p	LOC_Os09g24760.1	166	-26.59	166	-26.59	913571	20	2613	659
miR1846d-3p	LOC_Os09g24850.1	152	-24.43	152	-24.43	913581	20	4086	3630
miR1846d-3p	LOC_Os09g25110.1	166	-26.29	166	-26.29	913611	20	2913	437
miR1846d-3p	LOC_Os09g25190.1	146	-23.7	146	-23.7	913619	20	1691	1141
miR1846d-3p	LOC_Os09g25390.1	152	-25.62	152	-25.62	913641	20	2774	1046
miR1846d-3p	LOC_Os09g25460.1	153	-23.61	153	-23.61	913649	20	1834	879

miR1846d-3p	LOC_Os09g25550.1	153	-24.48	153	-24.48	913658	20	3421	342
miR1846d-3p	LOC_Os09g25710.1	147	-22.62	147	-22.62	913677	20	435	291
miR1846d-3p	LOC_Os09g25860.1	145	-23	145	-23	913691	20	1233	18
miR1846d-3p	LOC_Os09g25945.1	161	-31.17	161	-31.17	913702	20	1757	1634
miR1846d-3p	LOC_Os09g26110.1	145	-20.4	145	-20.4	913720	20	599	392
miR1846d-3p	LOC_Os09g26120.1	166	-27.37	166	-27.37	913721	20	2691	404
miR1846d-3p	LOC_Os09g26170.1	155	-25.1	155	-25.1	913726	20	1134	921
miR1846d-3p	LOC_Os09g26260.1	175	-39.73	175	-39.73	913735	20	1734	1218
miR1846d-3p	LOC_Os09g26790.1	152	-22.43	152	-22.43	913801	20	240	66
miR1846d-3p	LOC_Os09g26870.1	146	-23.42	146	-23.42	913809	20	2077	426
miR1846d-3p	LOC_Os09g27190.1	167	-32.5	167	-32.5	913847	20	1056	63
miR1846d-3p	LOC_Os09g27260.1	140	-20.4	140	-20.4	913855	20	1394	1207
miR1846d-3p	LOC_Os09g27450.1	149	-25.89	149	-25.89	913875	20	1495	387
miR1846d-3p	LOC_Os09g27480.1	149	-21.66	149	-21.66	913878	20	1253	912
miR1846d-3p	LOC_Os09g27740.1	157	-32.45	157	-32.45	913922	20	612	343
miR1846d-3p	LOC_Os09g27744.1	148	-21.54	148	-21.54	913923	20	957	537
miR1846d-3p	LOC_Os09g27800.1	151	-24.69	151	-24.69	913929	20	2184	1962
miR1846d-3p	LOC_Os09g27850.1	164	-27.03	164	-27.03	913936	20	1606	208
miR1846d-3p	LOC_Os09g27880.1	302	-45.65	156	-22.9	913939	20	1089	145 592
miR1846d-3p	LOC_Os09g27910.1	161	-30.1	161	-30.1	913943	20	1553	1162
miR1846d-3p	LOC_Os09g27970.1	155	-30.5	155	-30.5	913951	20	2683	2467
miR1846d-3p	LOC_Os09g27970.2	155	-30.5	155	-30.5	913952	20	2824	2608
miR1846d-3p	LOC_Os09g27970.3	155	-30.5	155	-30.5	913953	20	3153	2937
miR1846d-3p	LOC_Os09g27970.4	155	-30.5	155	-30.5	913954	20	3167	2951
miR1846d-3p	LOC_Os09g27970.5	155	-30.5	155	-30.5	913955	20	3078	2862
miR1846d-3p	LOC_Os09g28070.1	147	-21.62	147	-21.62	913966	20	3474	128
miR1846d-3p	LOC_Os09g28080.1	172	-34.12	172	-34.12	913967	20	3084	1861
miR1846d-3p	LOC_Os09g28120.1	153	-21.59	153	-21.59	913971	20	1683	194
miR1846d-3p	LOC_Os09g28180.1	291	-40.56	146	-20.55	913977	20	4056	602 1687
miR1846d-3p	LOC_Os09g28190.1	151	-23.7	151	-23.7	913978	20	714	87
miR1846d-3p	LOC_Os09g28370.1	154	-21.35	154	-21.35	913996	20	6942	3475
miR1846d-3p	LOC_Os09g28610.1	165	-28.96	165	-28.96	914024	20	2640	339

miR1846d-3p	LOC_Os09g28720.1	143	-22.98	143	-22.98	914035	20	1445	1018
miR1846d-3p	LOC_Os09g28730.1	313	-51.85	162	-26.33	914036	20	1435	751 1166
miR1846d-3p	LOC_Os09g29060.1	141	-20.58	141	-20.58	914075	20	1502	315
miR1846d-3p	LOC_Os09g29270.1	472	-73.71	162	-28.44	914098	20	1094	312 584 473
miR1846d-3p	LOC_Os09g29300.1	156	-31.4	156	-31.4	914100	20	1002	355
miR1846d-3p	LOC_Os09g29330.1	147	-21.62	147	-21.62	914103	20	5856	2555
miR1846d-3p	LOC_Os09g29500.1	158	-28.91	158	-28.91	914125	20	2139	168
miR1846d-3p	LOC_Os09g29650.1	155	-27.72	155	-27.72	914139	20	237	209
miR1846d-3p	LOC_Os09g29900.1	146	-23.86	146	-23.86	914169	20	786	482
miR1846d-3p	LOC_Os09g30190.1	153	-25.16	153	-25.16	914213	20	3700	2895
miR1846d-3p	LOC_Os09g30230.1	144	-23.49	144	-23.49	914217	20	1754	1536
miR1846d-3p	LOC_Os09g30340.1	161	-28.94	161	-28.94	914229	20	1596	1106
miR1846d-3p	LOC_Os09g30350.1	161	-28.94	161	-28.94	914230	20	3346	1106
miR1846d-3p	LOC_Os09g30414.1	146	-23.15	146	-23.15	914242	20	2438	153
miR1846d-3p	LOC_Os09g30970.1	165	-32.76	165	-32.76	914261	20	651	290
miR1846d-3p	LOC_Os09g31050.1	143	-25.18	143	-25.18	914271	20	1528	1048
miR1846d-3p	LOC_Os09g31310.1	150	-25.21	150	-25.21	914309	20	748	649
miR1846d-3p	LOC_Os09g31498.1	149	-30.69	149	-30.69	914343	20	1182	906
miR1846d-3p	LOC_Os09g31950.1	305	-50.53	158	-28.91	914356	20	5814	507 2546
miR1846d-3p	LOC_Os09g32290.1	152	-25.11	152	-25.11	914399	20	964	489
miR1846d-3p	LOC_Os09g32290.2	152	-25.11	152	-25.11	914400	20	1551	489
miR1846d-3p	LOC_Os09g32300.1	150	-22.49	150	-22.49	914401	20	2763	361
miR1846d-3p	LOC_Os09g32300.2	150	-22.49	150	-22.49	914402	20	2690	361
miR1846d-3p	LOC_Os09g32300.3	150	-22.49	150	-22.49	914403	20	2993	361
miR1846d-3p	LOC_Os09g32410.1	158	-25.71	158	-25.71	914414	20	2862	2508
miR1846d-3p	LOC_Os09g32526.1	157	-27.47	157	-27.47	914429	20	881	723
miR1846d-3p	LOC_Os09g32526.2	157	-27.47	157	-27.47	914430	20	984	826
miR1846d-3p	LOC_Os09g32580.1	146	-23.01	146	-23.01	914438	20	2036	1711
miR1846d-3p	LOC_Os09g32830.1	145	-20.06	145	-20.06	914469	20	1956	985
miR1846d-3p	LOC_Os09g33490.1	150	-21.05	150	-21.05	914508	20	1243	724
miR1846d-3p	LOC_Os09g33530.1	153	-24.83	153	-24.83	914514	20	2152	1667
miR1846d-3p	LOC_Os09g33530.2	153	-24.83	153	-24.83	914515	20	2315	1830

miR1846d-3p	LOC_Os09g33550.1	150	-25.08	150	-25.08	914517	20	1938	786
miR1846d-3p	LOC_Os09g33670.1	153	-22.7	153	-22.7	914532	20	1752	91
miR1846d-3p	LOC_Os09g33920.1	150	-25.68	150	-25.68	914569	20	1627	15
miR1846d-3p	LOC_Os09g33920.2	150	-25.68	150	-25.68	914570	20	1563	15
miR1846d-3p	LOC_Os09g33986.1	148	-24.05	148	-24.05	914582	20	1130	237
miR1846d-3p	LOC_Os09g33986.2	148	-24.05	148	-24.05	914583	20	1083	237
miR1846d-3p	LOC_Os09g34040.1	162	-26.83	162	-26.83	914593	20	1987	1614
miR1846d-3p	LOC_Os09g34320.1	152	-27.84	152	-27.84	914631	20	1781	1362
miR1846d-3p	LOC_Os09g34340.1	916	-152.7	156	-28.73	914633	20	1683	1110 1175 1215 1145
miR1846d-3p	LOC_Os09g34920.1	140	-22.26	140	-22.26	914645	20	1795	899
miR1846d-3p	LOC_Os09g34985.1	140	-20.38	140	-20.38	914652	20	611	557
miR1846d-3p	LOC_Os09g35790.1	148	-23.47	148	-23.47	914688	20	1780	1661
miR1846d-3p	LOC_Os09g35790.2	148	-23.47	148	-23.47	914689	20	1899	1780
miR1846d-3p	LOC_Os09g35790.3	148	-23.47	148	-23.47	914690	20	830	711
miR1846d-3p	LOC_Os09g35810.2	152	-20.79	152	-20.79	914693	20	3544	2920
miR1846d-3p	LOC_Os09g36160.1	159	-29.75	159	-29.75	914734	20	1850	1594
miR1846d-3p	LOC_Os09g36180.1	145	-23.26	145	-23.26	914736	20	3243	1067
miR1846d-3p	LOC_Os09g36360.1	157	-27.71	157	-27.71	914766	20	1594	1070
miR1846d-3p	LOC_Os09g36540.1	153	-25.46	153	-25.46	914786	20	5037	4113
miR1846d-3p	LOC_Os09g36590.1	152	-26.81	152	-26.81	914790	20	2874	1101
miR1846d-3p	LOC_Os09g36920.1	147	-30.23	147	-30.23	914831	20	2073	1798
miR1846d-3p	LOC_Os09g37040.1	156	-27.95	156	-27.95	914847	20	2494	456
miR1846d-3p	LOC_Os09g37990.1	158	-30.14	158	-30.14	914951	20	624	518
miR1846d-3p	LOC_Os09g38070.1	147	-20.08	147	-20.08	914963	20	1570	833
miR1846d-3p	LOC_Os09g38239.1	317	-58.58	166	-33.38	914977	20	1502	307 602
miR1846d-3p	LOC_Os09g38650.1	149	-21.17	149	-21.17	915026	20	1498	1402
miR1846d-3p	LOC_Os09g38700.1	140	-22.18	140	-22.18	915036	20	2775	1996
miR1846d-3p	LOC_Os09g38710.1	140	-22.18	140	-22.18	915037	20	3646	76
miR1846d-3p	LOC_Os09g38710.2	140	-22.18	140	-22.18	915038	20	3612	76
miR1846d-3p	LOC_Os09g38830.1	148	-25.99	148	-25.99	915054	20	1869	626
miR1846d-3p	LOC_Os09g39260.1	302	-47.22	154	-25.34	915099	20	1698	218 459
miR1846d-3p	LOC_Os09g39290.1	147	-21.62	147	-21.62	915102	20	6084	2546

miR1846d-3p	LOC_Os09g39300.1	172	-34.12	172	-34.12	915103	20	2943	1756
miR1846d-3p	LOC_Os09g39420.1	161	-28.89	161	-28.89	915118	20	3697	182
miR1846d-3p	LOC_Os09g39820.1	154	-30.79	154	-30.79	915172	20	4734	2283
miR1846d-3p	LOC_Os09g39890.1	143	-23.95	143	-23.95	915180	20	3075	1415
miR1846d-3p	LOC_Os09g39970.1	143	-20.46	143	-20.46	915190	20	3408	3129
miR1846d-3p	LOC_Os09g40010.1	140	-21.04	140	-21.04	915196	20	2736	1383
miR1846d-3p	LOC_Os09g40050.1	147	-20.79	147	-20.79	915202	20	6414	2858
miR1846d-3p	LOC_Os09g40060.1	172	-34.12	172	-34.12	915203	20	2988	1790
miR1846d-3p	LOC_Os10g01090.1	165	-28.33	165	-28.33	915217	20	576	205
miR1846d-3p	LOC_Os10g01100.1	159	-22.52	159	-22.52	915218	20	2949	312
miR1846d-3p	LOC_Os10g01560.1	148	-26.06	148	-26.06	915261	20	2406	682
miR1846d-3p	LOC_Os10g01570.1	157	-27.06	157	-27.06	915262	20	1600	1023
miR1846d-3p	LOC_Os10g02314.1	152	-22.43	152	-22.43	915342	20	717	66
miR1846d-3p	LOC_Os10g02500.1	290	-41.47	146	-20.77	915359	20	3948	1976 2252
miR1846d-3p	LOC_Os10g02760.1	147	-25.53	147	-25.53	915386	20	2365	2314
miR1846d-3p	LOC_Os10g02760.2	147	-25.53	147	-25.53	915387	20	2362	2311
miR1846d-3p	LOC_Os10g02780.1	148	-25.07	148	-25.07	915389	20	699	255
miR1846d-3p	LOC_Os10g02850.1	166	-26.59	166	-26.59	915396	20	2943	404
miR1846d-3p	LOC_Os10g03420.1	145	-20.41	145	-20.41	915453	20	1152	132
miR1846d-3p	LOC_Os10g03500.1	296	-47.69	152	-23.98	915461	20	495	356 386
miR1846d-3p	LOC_Os10g03780.1	154	-21.7	154	-21.7	915492	20	3148	2870
miR1846d-3p	LOC_Os10g03830.1	154	-21.7	154	-21.7	915495	20	2846	2515
miR1846d-3p	LOC_Os10g03830.2	154	-21.7	154	-21.7	915496	20	2815	2484
miR1846d-3p	LOC_Os10g03950.1	145	-22.59	145	-22.59	915511	20	876	726
miR1846d-3p	LOC_Os10g04050.1	153	-25.98	153	-25.98	915520	20	6620	3296
miR1846d-3p	LOC_Os10g04050.2	153	-25.98	153	-25.98	915521	20	6587	3263
miR1846d-3p	LOC_Os10g04270.1	140	-20.05	140	-20.05	915539	20	432	100
miR1846d-3p	LOC_Os10g04490.1	150	-24.59	150	-24.59	915554	20	1461	1391
miR1846d-3p	LOC_Os10g04674.1	158	-24.86	158	-24.86	915577	20	3397	3354
miR1846d-3p	LOC_Os10g04674.3	158	-24.86	158	-24.86	915579	20	3441	3398
miR1846d-3p	LOC_Os10g04760.1	146	-21.22	146	-21.22	915588	20	273	136
miR1846d-3p	LOC_Os10g05560.1	144	-23.99	144	-23.99	915669	20	483	334

miR1846d-3p	LOC_Os10g06690.1	152	-22.83	152	-22.83	915781	20	503	306
miR1846d-3p	LOC_Os10g06990.1	153	-22.46	153	-22.46	915808	20	2427	1402
miR1846d-3p	LOC_Os10g07004.1	305	-50.58	160	-29.28	915810	20	1992	512 261
miR1846d-3p	LOC_Os10g07574.1	144	-23.56	144	-23.56	915882	20	7014	5895
miR1846d-3p	LOC_Os10g08011.1	159	-27.14	159	-27.14	915901	20	1059	385
miR1846d-3p	LOC_Os10g08260.1	147	-23.73	147	-23.73	915929	20	3180	2963
miR1846d-3p	LOC_Os10g08490.1	151	-27.3	151	-27.3	915947	20	234	164
miR1846d-3p	LOC_Os10g08540.1	153	-26.3	153	-26.3	915952	20	2968	2767
miR1846d-3p	LOC_Os10g08570.1	148	-27.38	148	-27.38	915957	20	2570	156
miR1846d-3p	LOC_Os10g08599.1	153	-21.64	153	-21.64	915960	20	2042	138
miR1846d-3p	LOC_Os10g08650.1	151	-26.66	151	-26.66	915965	20	1638	428
miR1846d-3p	LOC_Os10g08790.1	158	-27.6	158	-27.6	915980	20	465	57
miR1846d-3p	LOC_Os10g08910.1	166	-26.59	166	-26.59	915991	20	2397	599
miR1846d-3p	LOC_Os10g09150.1	152	-26.91	152	-26.91	916014	20	444	406
miR1846d-3p	LOC_Os10g09170.1	302	-42.41	153	-22.14	916016	20	918	152 192
miR1846d-3p	LOC_Os10g09290.1	292	-41.12	147	-20.91	916027	20	366	222 243
miR1846d-3p	LOC_Os10g09620.1	152	-21.9	152	-21.9	916056	20	2121	1548
miR1846d-3p	LOC_Os10g09640.1	153	-33.09	153	-33.09	916058	20	717	620
miR1846d-3p	LOC_Os10g09670.1	143	-21.46	143	-21.46	916061	20	786	184
miR1846d-3p	LOC_Os10g10100.1	153	-22.22	153	-22.22	916105	20	4182	621
miR1846d-3p	LOC_Os10g10200.1	149	-20.83	149	-20.83	916123	20	2049	387
miR1846d-3p	LOC_Os10g10214.1	143	-20.17	143	-20.17	916124	20	3246	2105
miR1846d-3p	LOC_Os10g10620.1	157	-27.98	157	-27.98	916163	20	800	760
miR1846d-3p	LOC_Os10g10820.1	168	-32.42	168	-32.42	916183	20	2580	1267
miR1846d-3p	LOC_Os10g10840.1	148	-21.6	148	-21.6	916185	20	2436	1207
miR1846d-3p	LOC_Os10g10960.1	152	-24.78	152	-24.78	916195	20	360	89
miR1846d-3p	LOC_Os10g11310.1	163	-25.54	163	-25.54	916232	20	2833	2268
miR1846d-3p	LOC_Os10g11310.2	163	-25.54	163	-25.54	916233	20	2891	2268
miR1846d-3p	LOC_Os10g11310.3	163	-25.54	163	-25.54	916234	20	2841	2268
miR1846d-3p	LOC_Os10g11770.1	161	-29.68	161	-29.68	916273	20	375	40
miR1846d-3p	LOC_Os10g12130.1	140	-21.13	140	-21.13	916303	20	3655	1103
miR1846d-3p	LOC_Os10g12220.1	165	-28.85	165	-28.85	916310	20	2499	338

miR1846d-3p	LOC_Os10g12232.1	145	-21.82	145	-21.82	916312	20	2928	1317
miR1846d-3p	LOC_Os10g12280.1	152	-21.82	152	-21.82	916316	20	2538	1306
miR1846d-3p	LOC_Os10g12320.1	162	-28.72	162	-28.72	916319	20	339	26
miR1846d-3p	LOC_Os10g12390.1	151	-22.83	151	-22.83	916324	20	401	115
miR1846d-3p	LOC_Os10g12610.1	293	-45.16	153	-24.43	916340	20	450	278 247
miR1846d-3p	LOC_Os10g12750.1	143	-21.81	143	-21.81	916354	20	1409	1145
miR1846d-3p	LOC_Os10g13390.1	152	-20.54	152	-20.54	916372	20	2403	397
miR1846d-3p	LOC_Os10g13400.1	145	-20.08	145	-20.08	916373	20	5802	4170
miR1846d-3p	LOC_Os10g13810.1	150	-27.11	150	-27.11	916418	20	1775	706
miR1846d-3p	LOC_Os10g13940.1	145	-23.85	145	-23.85	916428	20	2053	28
miR1846d-3p	LOC_Os10g13960.1	160	-23.36	160	-23.36	916430	20	4044	1466
miR1846d-3p	LOC_Os10g13990.1	148	-21.6	148	-21.6	916434	20	2481	1252
miR1846d-3p	LOC_Os10g14295.1	142	-22.55	142	-22.55	916469	20	2754	1970
miR1846d-3p	LOC_Os10g15310.1	149	-22.14	149	-22.14	916514	20	1780	1728
miR1846d-3p	LOC_Os10g15310.2	149	-22.14	149	-22.14	916515	20	1835	1783
miR1846d-3p	LOC_Os10g15310.3	149	-22.14	149	-22.14	916516	20	1902	1850
miR1846d-3p	LOC_Os10g15310.4	149	-22.14	149	-22.14	916517	20	1776	1720
miR1846d-3p	LOC_Os10g15310.5	149	-22.14	149	-22.14	916518	20	2520	2468
miR1846d-3p	LOC_Os10g15180.1	151	-24.69	151	-24.69	916531	20	1935	1827
miR1846d-3p	LOC_Os10g16410.1	151	-23.07	151	-23.07	916537	20	984	395
miR1846d-3p	LOC_Os10g16670.1	172	-34.12	172	-34.12	916563	20	2844	1507
miR1846d-3p	LOC_Os10g16850.1	166	-26.59	166	-26.59	916578	20	2994	659
miR1846d-3p	LOC_Os10g17030.1	144	-21.65	144	-21.65	916595	20	3000	537
miR1846d-3p	LOC_Os10g17230.1	152	-20.54	152	-20.54	916612	20	2298	397
miR1846d-3p	LOC_Os10g17250.1	145	-20.08	145	-20.08	916614	20	5838	4218
miR1846d-3p	LOC_Os10g17610.1	144	-21.04	144	-21.04	916648	20	4104	2046
miR1846d-3p	LOC_Os10g17930.1	150	-25.4	150	-25.4	916679	20	1724	877
miR1846d-3p	LOC_Os10g17940.1	151	-21.8	151	-21.8	916680	20	1825	711
miR1846d-3p	LOC_Os10g18150.1	156	-25.98	156	-25.98	916695	20	2396	1631
miR1846d-3p	LOC_Os10g18190.1	146	-20.01	146	-20.01	916699	20	780	17
miR1846d-3p	LOC_Os10g18260.1	147	-22.17	147	-22.17	916705	20	1248	321
miR1846d-3p	LOC_Os10g18400.1	151	-24.99	151	-24.99	916717	20	2839	886

miR1846d-3p	LOC_Os10g18490.1	158	-30.04	158	-30.04	916726	20	543	240
miR1846d-3p	LOC_Os10g18510.1	158	-24.87	158	-24.87	916728	20	1534	1168
miR1846d-3p	LOC_Os10g19120.1	152	-20.54	152	-20.54	916785	20	2412	397
miR1846d-3p	LOC_Os10g19130.1	145	-20.08	145	-20.08	916786	20	5988	4356
miR1846d-3p	LOC_Os10g19890.1	148	-25.84	148	-25.84	916819	20	1860	1269
miR1846d-3p	LOC_Os10g20000.1	166	-27.37	166	-27.37	916840	20	3039	650
miR1846d-3p	LOC_Os10g20160.1	158	-23.99	158	-23.99	916854	20	2938	2630
miR1846d-3p	LOC_Os10g20660.1	140	-20.04	140	-20.04	916911	20	3890	3254
miR1846d-3p	LOC_Os10g20770.1	152	-27.88	152	-27.88	916921	20	2530	1144
miR1846d-3p	LOC_Os10g20980.1	151	-23.12	151	-23.12	916940	20	2187	1059
miR1846d-3p	LOC_Os10g21060.1	158	-27.67	158	-27.67	916946	20	966	268
miR1846d-3p	LOC_Os10g21640.1	153	-26.16	153	-26.16	917039	20	393	8
miR1846d-3p	LOC_Os10g21720.1	156	-28.87	156	-28.87	917045	20	363	242
miR1846d-3p	LOC_Os10g22000.1	144	-22.93	144	-22.93	917077	20	2869	24
miR1846d-3p	LOC_Os10g22300.1	150	-26.26	150	-26.26	917108	20	2166	16
miR1846d-3p	LOC_Os10g22310.1	149	-21.1	149	-21.1	917109	20	1094	603
miR1846d-3p	LOC_Os10g22310.2	149	-21.1	149	-21.1	917110	20	1042	551
miR1846d-3p	LOC_Os10g22540.1	162	-24.8	162	-24.8	917134	20	3225	659
miR1846d-3p	LOC_Os10g22630.1	153	-24.07	153	-24.07	917145	20	1089	165
miR1846d-3p	LOC_Os10g22630.2	153	-24.07	153	-24.07	917146	20	1030	165
miR1846d-3p	LOC_Os10g22640.1	295	-41.92	148	-21.62	917147	20	6084	507 2546
miR1846d-3p	LOC_Os10g22650.1	172	-35.42	172	-35.42	917148	20	3012	1744
miR1846d-3p	LOC_Os10g23090.1	165	-30.74	165	-30.74	917197	20	1440	1043
miR1846d-3p	LOC_Os10g23090.2	165	-30.74	165	-30.74	917198	20	1400	1003
miR1846d-3p	LOC_Os10g23300.1	314	-46.3	162	-23.99	917221	20	396	76 107
miR1846d-3p	LOC_Os10g23900.1	153	-25.83	153	-25.83	917233	20	1581	878
miR1846d-3p	LOC_Os10g24390.1	156	-28.11	156	-28.11	917284	20	363	186
miR1846d-3p	LOC_Os10g24710.1	154	-22.57	154	-22.57	917315	20	1305	237
miR1846d-3p	LOC_Os10g25100.1	150	-23.24	150	-23.24	917351	20	669	295
miR1846d-3p	LOC_Os10g25140.1	157	-25.65	157	-25.65	917355	20	1745	138
miR1846d-3p	LOC_Os10g25810.1	148	-21.68	148	-21.68	917425	20	1014	405
miR1846d-3p	LOC_Os10g26110.1	312	-56.91	161	-29.41	917454	20	1548	339 121

miR1846d-3p	LOC_Os10g26210.1	153	-22.22	153	-22.22	917467	20	3513	291
miR1846d-3p	LOC_Os10g26370.1	159	-24.37	159	-24.37	917481	20	5832	202
miR1846d-3p	LOC_Os10g26520.1	167	-31.37	167	-31.37	917497	20	2314	698
miR1846d-3p	LOC_Os10g26610.1	148	-20.99	148	-20.99	917505	20	2102	350
miR1846d-3p	LOC_Os10g26610.2	148	-20.99	148	-20.99	917506	20	2138	350
miR1846d-3p	LOC_Os10g26620.1	147	-24.99	147	-24.99	917507	20	2037	617
miR1846d-3p	LOC_Os10g26859.1	156	-22.38	156	-22.38	917534	20	2598	151
miR1846d-3p	LOC_Os10g26990.1	146	-20.7	146	-20.7	917544	20	2201	2058
miR1846d-3p	LOC_Os10g27030.1	142	-20.14	142	-20.14	917548	20	2607	36
miR1846d-3p	LOC_Os10g27110.1	161	-23.8	161	-23.8	917557	20	934	700
miR1846d-3p	LOC_Os10g27150.1	149	-22.27	149	-22.27	917560	20	1431	505
miR1846d-3p	LOC_Os10g27310.1	152	-24.62	152	-24.62	917582	20	2158	1828
miR1846d-3p	LOC_Os10g27330.1	146	-20.97	146	-20.97	917584	20	1814	1244
miR1846d-3p	LOC_Os10g28009.1	155	-24.62	155	-24.62	917603	20	1232	897
miR1846d-3p	LOC_Os10g28040.1	151	-28.91	151	-28.91	917610	20	1941	1537
miR1846d-3p	LOC_Os10g28240.1	157	-31.76	157	-31.76	917629	20	3377	2983
miR1846d-3p	LOC_Os10g28254.1	461	-75.33	157	-28.07	917631	20	902	329 446 224
miR1846d-3p	LOC_Os10g28360.1	144	-24.13	144	-24.13	917647	20	1145	137
miR1846d-3p	LOC_Os10g28450.1	151	-24.99	151	-24.99	917657	20	1944	778
miR1846d-3p	LOC_Os10g28460.1	166	-26.59	166	-26.59	917658	20	2226	659
miR1846d-3p	LOC_Os10g28990.1	146	-21.61	146	-21.61	917713	20	1161	655
miR1846d-3p	LOC_Os10g29400.1	153	-23.35	153	-23.35	917752	20	2334	1782
miR1846d-3p	LOC_Os10g29540.1	167	-28.73	167	-28.73	917769	20	3634	3221
miR1846d-3p	LOC_Os10g29720.1	148	-24.23	148	-24.23	917789	20	930	220
miR1846d-3p	LOC_Os10g30080.1	145	-20.08	145	-20.08	917819	20	1856	1192
miR1846d-3p	LOC_Os10g30080.2	145	-20.08	145	-20.08	917820	20	1864	1200
miR1846d-3p	LOC_Os10g30250.1	166	-26.29	166	-26.29	917838	20	3180	659
miR1846d-3p	LOC_Os10g30590.1	303	-52.11	154	-27.57	917871	20	3675	28 930
miR1846d-3p	LOC_Os10g30590.2	303	-52.11	154	-27.57	917872	20	3160	28 930
miR1846d-3p	LOC_Os10g30790.1	141	-21.71	141	-21.71	917891	20	2444	194
miR1846d-3p	LOC_Os10g30790.2	141	-21.71	141	-21.71	917892	20	2829	194
miR1846d-3p	LOC_Os10g30890.1	154	-26.82	154	-26.82	917904	20	1695	1364

miR1846d-3p	LOC_Os10g31530.1	150	-22.52	150	-22.52	917970	20	871	460
miR1846d-3p	LOC_Os10g31640.1	145	-20.01	145	-20.01	917981	20	870	540
miR1846d-3p	LOC_Os10g31820.1	141	-21.82	141	-21.82	917998	20	3034	1560
miR1846d-3p	LOC_Os10g31820.2	141	-21.82	141	-21.82	917999	20	2672	1518
miR1846d-3p	LOC_Os10g31880.1	150	-23.81	150	-23.81	918007	20	1398	507
miR1846d-3p	LOC_Os10g32030.1	145	-24.81	145	-24.81	918019	20	4722	1969
miR1846d-3p	LOC_Os10g32080.1	145	-23.44	145	-23.44	918024	20	1643	339
miR1846d-3p	LOC_Os10g32110.1	149	-22.42	149	-22.42	918027	20	1597	673
miR1846d-3p	LOC_Os10g32420.1	147	-24.87	147	-24.87	918049	20	426	399
miR1846d-3p	LOC_Os10g32960.1	143	-21.18	143	-21.18	918107	20	1705	1381
miR1846d-3p	LOC_Os10g33040.1	154	-20.76	154	-20.76	918115	20	5076	4442
miR1846d-3p	LOC_Os10g33050.1	154	-20.76	154	-20.76	918116	20	1735	1101
miR1846d-3p	LOC_Os10g33080.1	147	-20.91	147	-20.91	918119	20	4177	1782
miR1846d-3p	LOC_Os10g33380.1	157	-24.05	157	-24.05	918150	20	348	5
miR1846d-3p	LOC_Os10g33630.1	154	-23.27	154	-23.27	918173	20	1639	195
miR1846d-3p	LOC_Os10g33630.2	154	-23.27	154	-23.27	918174	20	1483	110
miR1846d-3p	LOC_Os10g34078.1	145	-21.67	145	-21.67	918238	20	1611	912
miR1846d-3p	LOC_Os10g34380.1	307	-48.02	157	-24.91	918266	20	3503	3458 3175
miR1846d-3p	LOC_Os10g34409.1	154	-23.38	154	-23.38	918270	20	880	265
miR1846d-3p	LOC_Os10g34590.1	146	-20.4	146	-20.4	918287	20	1790	1639
miR1846d-3p	LOC_Os10g34602.1	146	-21.6	146	-21.6	918288	20	1326	437
miR1846d-3p	LOC_Os10g34795.1	474	-78.3	169	-30.47	918312	20	838	684 363 398
miR1846d-3p	LOC_Os10g34820.1	164	-34.3	164	-34.3	918314	20	2528	112
miR1846d-3p	LOC_Os10g34820.2	164	-34.3	164	-34.3	918315	20	2515	108
miR1846d-3p	LOC_Os10g35100.1	146	-20.69	146	-20.69	918346	20	1554	1199
miR1846d-3p	LOC_Os10g35424.1	155	-29.24	155	-29.24	918387	20	528	365
miR1846d-3p	LOC_Os10g35604.1	152	-25.69	152	-25.69	918410	20	1750	939
miR1846d-3p	LOC_Os10g36430.1	147	-21.62	147	-21.62	918501	20	5202	1694
miR1846d-3p	LOC_Os10g36680.1	158	-29.59	158	-29.59	918526	20	1937	1349
miR1846d-3p	LOC_Os10g37170.1	159	-25.62	159	-25.62	918572	20	4020	1248
miR1846d-3p	LOC_Os10g37230.1	153	-24.12	153	-24.12	918577	20	1266	998
miR1846d-3p	LOC_Os10g37540.1	302	-44.09	152	-22.8	918606	20	1672	486 386

miR1846d-3p	LOC_Os10g37760.1	164	-26.69	164	-26.69	918632	20	1468	332
miR1846d-3p	LOC_Os10g37760.2	164	-26.69	164	-26.69	918633	20	1332	332
miR1846d-3p	LOC_Os10g37940.1	150	-22.78	150	-22.78	918658	20	4758	4588
miR1846d-3p	LOC_Os10g38120.1	148	-20.21	148	-20.21	918677	20	1802	1427
miR1846d-3p	LOC_Os10g38150.1	142	-21.96	142	-21.96	918680	20	957	243
miR1846d-3p	LOC_Os10g38189.1	153	-20.28	153	-20.28	918683	20	672	629
miR1846d-3p	LOC_Os10g38234.1	141	-20.87	141	-20.87	918690	20	2205	1629
miR1846d-3p	LOC_Os10g38314.1	153	-20.28	153	-20.28	918696	20	495	197
miR1846d-3p	LOC_Os10g38780.1	144	-24.83	144	-24.83	918736	20	1222	859
miR1846d-3p	LOC_Os10g39044.1	318	-55.47	162	-28.32	918767	20	285	87 117
miR1846d-3p	LOC_Os10g39360.1	156	-20.62	156	-20.62	918805	20	1260	98
miR1846d-3p	LOC_Os10g39470.1	155	-21.75	155	-21.75	918819	20	4041	2441
miR1846d-3p	LOC_Os10g39470.2	155	-21.75	155	-21.75	918820	20	3990	2441
miR1846d-3p	LOC_Os10g39600.1	150	-21.55	150	-21.55	918835	20	1257	1108
miR1846d-3p	LOC_Os10g40080.1	152	-30.01	152	-30.01	918915	20	462	248
miR1846d-3p	LOC_Os10g40090.1	146	-28.18	146	-28.18	918916	20	1180	668
miR1846d-3p	LOC_Os10g40120.1	164	-33.03	164	-33.03	918920	20	1224	1202
miR1846d-3p	LOC_Os10g40250.1	158	-28.5	158	-28.5	918933	20	3738	192
miR1846d-3p	LOC_Os10g40250.2	158	-28.5	158	-28.5	918934	20	3865	192
miR1846d-3p	LOC_Os10g40360.1	153	-23.96	153	-23.96	918941	20	2075	860
miR1846d-3p	LOC_Os10g40460.1	145	-21.87	145	-21.87	918951	20	679	204
miR1846d-3p	LOC_Os10g40470.1	145	-21.87	145	-21.87	918952	20	735	248
miR1846d-3p	LOC_Os10g40660.1	161	-28.13	161	-28.13	918974	20	1692	441
miR1846d-3p	LOC_Os10g41020.1	143	-21.59	143	-21.59	919011	20	2766	867
miR1846d-3p	LOC_Os10g41170.1	151	-25.9	151	-25.9	919028	20	1832	76
miR1846d-3p	LOC_Os10g41370.1	160	-27.24	160	-27.24	919053	20	1984	1844
miR1846d-3p	LOC_Os10g41420.1	147	-20.47	147	-20.47	919061	20	639	592
miR1846d-3p	LOC_Os10g41670.1	150	-23.03	150	-23.03	919085	20	210	61
miR1846d-3p	LOC_Os10g42150.1	168	-32.09	168	-32.09	919150	20	1125	549
miR1846d-3p	LOC_Os10g42320.1	153	-25.11	153	-25.11	919175	20	2498	2381
miR1846d-3p	LOC_Os10g42400.1	160	-28.42	160	-28.42	919179	20	1996	344
miR1846d-3p	LOC_Os10g42770.1	148	-22.65	148	-22.65	919230	20	1191	461

miR1846d-3p	LOC_Os10g42800.1	156	-23.18	156	-23.18	919237	20	1695	256
miR1846d-3p	LOC_Os11g01030.1	306	-56.01	165	-31.71	919276	20	2538	1250 1979
miR1846d-3p	LOC_Os11g01180.1	146	-21.83	146	-21.83	919297	20	1017	994
miR1846d-3p	LOC_Os11g01330.1	149	-21.61	149	-21.61	919312	20	4019	682
miR1846d-3p	LOC_Os11g01800.1	151	-23.97	151	-23.97	919364	20	207	90
miR1846d-3p	LOC_Os11g01860.1	147	-22.67	147	-22.67	919372	20	2438	2032
miR1846d-3p	LOC_Os11g01920.1	305	-50.53	158	-28.91	919387	20	5946	507 2546
miR1846d-3p	LOC_Os11g02120.1	308	-46.76	154	-23.38	919405	20	393	89 196
miR1846d-3p	LOC_Os11g02200.1	148	-22.37	148	-22.37	919416	20	270	34
miR1846d-3p	LOC_Os11g02240.1	148	-24.85	148	-24.85	919420	20	2121	318
miR1846d-3p	LOC_Os11g03090.1	147	-21.62	147	-21.62	919520	20	6093	2555
miR1846d-3p	LOC_Os11g03100.1	172	-34.12	172	-34.12	919521	20	3207	1870
miR1846d-3p	LOC_Os11g03150.1	145	-20.77	145	-20.77	919526	20	624	588
miR1846d-3p	LOC_Os11g03310.1	146	-20.8	146	-20.8	919545	20	5337	4359
miR1846d-3p	LOC_Os11g03410.1	148	-22.96	148	-22.96	919555	20	1919	1814
miR1846d-3p	LOC_Os11g03734.1	156	-27.01	156	-27.01	919594	20	2432	83
miR1846d-3p	LOC_Os11g03940.1	142	-22.66	142	-22.66	919615	20	6084	41
miR1846d-3p	LOC_Os11g03980.1	150	-22	150	-22	919620	20	552	24
miR1846d-3p	LOC_Os11g04104.1	155	-22.47	155	-22.47	919635	20	2299	1965
miR1846d-3p	LOC_Os11g04104.2	155	-22.47	155	-22.47	919636	20	2315	1981
miR1846d-3p	LOC_Os11g04280.1	158	-23.17	158	-23.17	919653	20	657	478
miR1846d-3p	LOC_Os11g04360.1	144	-22.74	144	-22.74	919667	20	1328	1158
miR1846d-3p	LOC_Os11g04590.1	149	-21.18	149	-21.18	919699	20	1383	811
miR1846d-3p	LOC_Os11g04935.1	150	-24.71	150	-24.71	919738	20	615	421
miR1846d-3p	LOC_Os11g05310.1	146	-23.59	146	-23.59	919786	20	931	424
miR1846d-3p	LOC_Os11g05400.1	144	-22.82	144	-22.82	919796	20	2288	295
miR1846d-3p	LOC_Os11g05540.1	146	-21.12	146	-21.12	919812	20	2201	1077
miR1846d-3p	LOC_Os11g05690.2	145	-20.26	145	-20.26	919829	20	2221	57
miR1846d-3p	LOC_Os11g05860.1	143	-22.55	143	-22.55	919847	20	1079	847
miR1846d-3p	LOC_Os11g06200.1	153	-30.59	153	-30.59	919890	20	888	72
miR1846d-3p	LOC_Os11g06230.1	152	-25.35	152	-25.35	919894	20	2187	235
miR1846d-3p	LOC_Os11g06410.1	149	-20.97	149	-20.97	919918	20	2869	88

miR1846d-3p	LOC_Os11g06410.2	149	-20.97	149	-20.97	919919	20	1893	74
miR1846d-3p	LOC_Os11g06410.3	149	-20.97	149	-20.97	919920	20	1850	110
miR1846d-3p	LOC_Os11g06420.1	148	-23.64	148	-23.64	919921	20	2107	1247
miR1846d-3p	LOC_Os11g06420.2	148	-23.64	148	-23.64	919922	20	2103	1243
miR1846d-3p	LOC_Os11g06580.1	156	-26.56	156	-26.56	919937	20	1572	344
miR1846d-3p	LOC_Os11g06840.1	147	-20.9	147	-20.9	919967	20	1594	352
miR1846d-3p	LOC_Os11g07030.1	169	-29.49	169	-29.49	919996	20	2277	1721
miR1846d-3p	LOC_Os11g07060.1	157	-23.33	157	-23.33	919999	20	3785	156
miR1846d-3p	LOC_Os11g07060.3	157	-23.33	157	-23.33	920001	20	3815	156
miR1846d-3p	LOC_Os11g07060.4	157	-23.33	157	-23.33	920002	20	3789	156
miR1846d-3p	LOC_Os11g07060.5	157	-23.33	157	-23.33	920003	20	3703	156
miR1846d-3p	LOC_Os11g07090.1	148	-21.25	148	-21.25	920006	20	1602	508
miR1846d-3p	LOC_Os11g07124.1	167	-32.27	167	-32.27	920011	20	3099	299
miR1846d-3p	LOC_Os11g07630.1	149	-23.43	149	-23.43	920069	20	402	110
miR1846d-3p	LOC_Os11g07730.1	152	-24.39	152	-24.39	920079	20	4815	3712
miR1846d-3p	LOC_Os11g07911.1	173	-36.11	173	-36.11	920099	20	701	220
miR1846d-3p	LOC_Os11g08542.1	159	-25.62	159	-25.62	920172	20	2793	1062
miR1846d-3p	LOC_Os11g08810.1	142	-22.55	142	-22.55	920194	20	3288	1970
miR1846d-3p	LOC_Os11g08970.1	151	-27.45	151	-27.45	920210	20	333	239
miR1846d-3p	LOC_Os11g09240.1	147	-20.79	147	-20.79	920242	20	5709	2546
miR1846d-3p	LOC_Os11g09250.1	172	-34.12	172	-34.12	920243	20	3165	1828
miR1846d-3p	LOC_Os11g09410.1	140	-21.04	140	-21.04	920259	20	3081	1396
miR1846d-3p	LOC_Os11g09640.1	141	-20.37	141	-20.37	920278	20	2287	1600
miR1846d-3p	LOC_Os11g09640.2	141	-20.37	141	-20.37	920279	20	1504	817
miR1846d-3p	LOC_Os11g09710.1	156	-25.29	156	-25.29	920289	20	1256	110
miR1846d-3p	LOC_Os11g09740.1	152	-20.01	152	-20.01	920293	20	564	437
miR1846d-3p	LOC_Os11g09780.1	149	-23	149	-23	920297	20	567	221
miR1846d-3p	LOC_Os11g09850.1	162	-29.79	162	-29.79	920304	20	1514	683
miR1846d-3p	LOC_Os11g09850.2	162	-29.79	162	-29.79	920305	20	1559	683
miR1846d-3p	LOC_Os11g09940.1	149	-20.72	149	-20.72	920315	20	2780	2668
miR1846d-3p	LOC_Os11g10100.1	149	-23.37	149	-23.37	920333	20	2235	352
miR1846d-3p	LOC_Os11g10100.2	149	-23.37	149	-23.37	920334	20	1792	352

miR1846d-3p	LOC_Os11g10340.1	145	-21.13	145	-21.13	920359	20	1998	1869
miR1846d-3p	LOC_Os11g10434.1	152	-21.03	152	-21.03	920368	20	1725	143
miR1846d-3p	LOC_Os11g10720.1	149	-20.33	149	-20.33	920399	20	2304	53
miR1846d-3p	LOC_Os11g10890.1	153	-25	153	-25	920416	20	507	246
miR1846d-3p	LOC_Os11g10980.1	155	-31.86	155	-31.86	920425	20	2465	2387
miR1846d-3p	LOC_Os11g10990.1	155	-31.86	155	-31.86	920426	20	1362	810
miR1846d-3p	LOC_Os11g11090.1	157	-25.07	157	-25.07	920439	20	303	93
miR1846d-3p	LOC_Os11g11350.1	152	-23.44	152	-23.44	920464	20	2532	1935
miR1846d-3p	LOC_Os11g11460.1	153	-23.07	153	-23.07	920478	20	594	282
miR1846d-3p	LOC_Os11g11490.1	159	-29.85	159	-29.85	920480	20	4172	1752
miR1846d-3p	LOC_Os11g11660.1	290	-42.31	145	-22.13	920498	20	1317	96 120
miR1846d-3p	LOC_Os11g12270.1	154	-23.46	154	-23.46	920557	20	1772	560
miR1846d-3p	LOC_Os11g12270.2	154	-23.46	154	-23.46	920558	20	1121	560
miR1846d-3p	LOC_Os11g12450.1	142	-22.55	142	-22.55	920577	20	3303	2003
miR1846d-3p	LOC_Os11g12530.1	159	-23.95	159	-23.95	920585	20	3711	1163
miR1846d-3p	LOC_Os11g12610.1	308	-44.74	154	-24.26	920593	20	288	88 142
miR1846d-3p	LOC_Os11g12690.1	147	-21.62	147	-21.62	920601	20	6084	2546
miR1846d-3p	LOC_Os11g12700.1	172	-35.42	172	-35.42	920602	20	3168	1831
miR1846d-3p	LOC_Os11g12730.1	142	-22.55	142	-22.55	920605	20	5055	3737
miR1846d-3p	LOC_Os11g13540.1	141	-21.46	141	-21.46	920636	20	1431	445
miR1846d-3p	LOC_Os11g14260.1	607	-95.77	157	-26.4	920715	20	1779	1468 1231 1173 1428
miR1846d-3p	LOC_Os11g14270.1	150	-24.48	150	-24.48	920716	20	3153	2683
miR1846d-3p	LOC_Os11g14730.1	149	-20.82	149	-20.82	920772	20	447	380
miR1846d-3p	LOC_Os11g14840.1	153	-30.38	153	-30.38	920782	20	2244	151
miR1846d-3p	LOC_Os11g14850.1	165	-28.24	165	-28.24	920783	20	927	866
miR1846d-3p	LOC_Os11g14940.1	147	-23.41	147	-23.41	920796	20	672	521
miR1846d-3p	LOC_Os11g14960.1	166	-27.37	166	-27.37	920798	20	2997	659
miR1846d-3p	LOC_Os11g14990.1	148	-27.86	148	-27.86	920801	20	836	647
miR1846d-3p	LOC_Os11g15040.1	146	-22.77	146	-22.77	920806	20	1374	725
miR1846d-3p	LOC_Os11g15040.2	146	-22.77	146	-22.77	920807	20	1369	720
miR1846d-3p	LOC_Os11g15040.3	146	-22.77	146	-22.77	920808	20	1481	832
miR1846d-3p	LOC_Os11g15040.4	146	-22.77	146	-22.77	920809	20	1486	837

miR1846d-3p	LOC_Os11g15040.5	146	-22.77	146	-22.77	920810	20	1800	1151
miR1846d-3p	LOC_Os11g15060.1	146	-22.77	146	-22.77	920812	20	1128	634
miR1846d-3p	LOC_Os11g15100.1	152	-21.82	152	-21.82	920816	20	2448	1216
miR1846d-3p	LOC_Os11g15130.1	146	-21.27	146	-21.27	920819	20	864	469
miR1846d-3p	LOC_Os11g15170.1	144	-23.56	144	-23.56	920822	20	5169	4142
miR1846d-3p	LOC_Os11g15180.1	143	-22.16	143	-22.16	920823	20	1098	601
miR1846d-3p	LOC_Os11g15230.1	453	-79.65	156	-28.07	920828	20	1993	734 1428 1406
miR1846d-3p	LOC_Os11g15680.1	156	-25.11	156	-25.11	920872	20	888	77
miR1846d-3p	LOC_Os11g16590.1	157	-22.54	157	-22.54	920933	20	3232	912
miR1846d-3p	LOC_Os11g16810.1	156	-28.42	156	-28.42	920953	20	375	129
miR1846d-3p	LOC_Os11g16820.1	147	-21.62	147	-21.62	920954	20	5370	2075
miR1846d-3p	LOC_Os11g16830.1	172	-34.12	172	-34.12	920955	20	2841	1639
miR1846d-3p	LOC_Os11g17504.1	149	-21.63	149	-21.63	921017	20	1033	289
miR1846d-3p	LOC_Os11g17504.2	149	-21.63	149	-21.63	921018	20	1066	289
miR1846d-3p	LOC_Os11g17799.1	167	-32.35	167	-32.35	921046	20	831	125
miR1846d-3p	LOC_Os11g17799.2	318	-56.25	167	-32.35	921047	20	1460	125 953
miR1846d-3p	LOC_Os11g18450.1	148	-20.48	148	-20.48	921100	20	2643	856
miR1846d-3p	LOC_Os11g18990.1	150	-23.21	150	-23.21	921157	20	1237	207
miR1846d-3p	LOC_Os11g19060.1	151	-29.52	151	-29.52	921164	20	2276	448
miR1846d-3p	LOC_Os11g19200.1	153	-22.46	153	-22.46	921178	20	2517	526
miR1846d-3p	LOC_Os11g19210.1	147	-21.04	147	-21.04	921179	20	2759	1028
miR1846d-3p	LOC_Os11g19510.1	144	-22.93	144	-22.93	921212	20	1920	24
miR1846d-3p	LOC_Os11g19520.1	156	-23.89	156	-23.89	921213	20	4879	4314
miR1846d-3p	LOC_Os11g19850.1	145	-20.67	145	-20.67	921249	20	834	8
miR1846d-3p	LOC_Os11g20150.1	151	-21.79	151	-21.79	921277	20	4521	3330
miR1846d-3p	LOC_Os11g20170.1	158	-25.8	158	-25.8	921279	20	1385	142
miR1846d-3p	LOC_Os11g20179.1	148	-24.19	148	-24.19	921280	20	1089	252
miR1846d-3p	LOC_Os11g20179.2	148	-24.19	148	-24.19	921281	20	1106	228
miR1846d-3p	LOC_Os11g20179.3	148	-24.19	148	-24.19	921282	20	972	225
miR1846d-3p	LOC_Os11g20239.1	302	-48.23	151	-27.3	921287	20	2750	2092 2263
miR1846d-3p	LOC_Os11g20260.1	147	-22.6	147	-22.6	921288	20	1242	1158
miR1846d-3p	LOC_Os11g20290.1	147	-20.79	147	-20.79	921291	20	5745	2207

miR1846d-3p	LOC_Os11g20300.1	172	-34.12	172	-34.12	921292	20	3027	1759
miR1846d-3p	LOC_Os11g20330.1	300	-54.21	151	-28.24	921295	20	864	656 571
miR1846d-3p	LOC_Os11g20330.2	300	-54.21	151	-28.24	921296	20	864	656 571
miR1846d-3p	LOC_Os11g20384.1	141	-21.4	141	-21.4	921301	20	2243	758
miR1846d-3p	LOC_Os11g20460.1	149	-23.91	149	-23.91	921308	20	3966	1009
miR1846d-3p	LOC_Os11g22370.1	146	-20.01	146	-20.01	921402	20	3834	17
miR1846d-3p	LOC_Os11g22650.1	144	-22.11	144	-22.11	921429	20	339	207
miR1846d-3p	LOC_Os11g22750.1	144	-20.02	144	-20.02	921438	20	2142	1769
miR1846d-3p	LOC_Os11g23020.1	149	-22.27	149	-22.27	921464	20	2541	505
miR1846d-3p	LOC_Os11g23100.1	140	-20.77	140	-20.77	921473	20	5196	4823
miR1846d-3p	LOC_Os11g23140.1	148	-20.37	148	-20.37	921477	20	4799	4509
miR1846d-3p	LOC_Os11g23170.1	154	-22.33	154	-22.33	921480	20	1411	263
miR1846d-3p	LOC_Os11g23170.2	154	-22.33	154	-22.33	921481	20	1430	263
miR1846d-3p	LOC_Os11g23170.3	154	-22.33	154	-22.33	921482	20	1503	263
miR1846d-3p	LOC_Os11g23900.1	144	-21.22	144	-21.22	921518	20	762	37
miR1846d-3p	LOC_Os11g24930.1	145	-20.08	145	-20.08	921613	20	5106	3708
miR1846d-3p	LOC_Os11g25040.1	168	-28.65	168	-28.65	921622	20	1232	350
miR1846d-3p	LOC_Os11g25080.1	152	-20.01	152	-20.01	921626	20	408	83
miR1846d-3p	LOC_Os11g25454.1	158	-22.55	158	-22.55	921663	20	1608	858
miR1846d-3p	LOC_Os11g25470.1	286	-41.48	145	-21.42	921664	20	597	163 69
miR1846d-3p	LOC_Os11g26620.1	153	-25.95	153	-25.95	921771	20	567	251
miR1846d-3p	LOC_Os11g27110.1	291	-43.27	148	-22.74	921821	20	1353	908 46
miR1846d-3p	LOC_Os11g27650.1	146	-20.01	146	-20.01	921872	20	3708	17
miR1846d-3p	LOC_Os11g28050.1	167	-31.92	167	-31.92	921911	20	1992	1344
miR1846d-3p	LOC_Os11g28130.1	610	-106.11	155	-28.42	921917	20	1000	159 316 455 618
miR1846d-3p	LOC_Os11g28400.1	142	-20.61	142	-20.61	921949	20	4239	1569
miR1846d-3p	LOC_Os11g28700.1	145	-20.08	145	-20.08	921979	20	5739	4119
miR1846d-3p	LOC_Os11g28720.1	152	-20.54	152	-20.54	921981	20	2499	397
miR1846d-3p	LOC_Os11g28730.1	145	-20.36	145	-20.36	921982	20	5286	3654
miR1846d-3p	LOC_Os11g28770.1	147	-21.62	147	-21.62	921985	20	5820	2282
miR1846d-3p	LOC_Os11g28790.1	153	-23.22	153	-23.22	921987	20	2190	397
miR1846d-3p	LOC_Os11g28830.1	149	-23.84	149	-23.84	921991	20	453	237

miR1846d-3p	LOC_Os11g28850.1	150	-24.27	150	-24.27	921993	20	1017	194
miR1846d-3p	LOC_Os11g28980.1	146	-23.43	146	-23.43	922004	20	2250	775
miR1846d-3p	LOC_Os11g29074.1	146	-20.01	146	-20.01	922011	20	3399	131
miR1846d-3p	LOC_Os11g29090.1	146	-25.99	146	-25.99	922012	20	3153	302
miR1846d-3p	LOC_Os11g29110.1	141	-21.09	141	-21.09	922014	20	3393	3129
miR1846d-3p	LOC_Os11g29230.1	149	-21.08	149	-21.08	922033	20	2785	1210
miR1846d-3p	LOC_Os11g29260.1	152	-22.61	152	-22.61	922036	20	414	298
miR1846d-3p	LOC_Os11g29350.1	142	-20.51	142	-20.51	922043	20	1379	182
miR1846d-3p	LOC_Os11g29360.1	162	-29.46	162	-29.46	922045	20	1484	1377
miR1846d-3p	LOC_Os11g29910.1	158	-27.56	158	-27.56	922104	20	639	345
miR1846d-3p	LOC_Os11g30460.1	142	-22.55	142	-22.55	922157	20	3243	1970
miR1846d-3p	LOC_Os11g30530.1	311	-44.53	158	-24.25	922163	20	5220	168 2098
miR1846d-3p	LOC_Os11g30540.1	143	-23.1	143	-23.1	922164	20	2733	1418
miR1846d-3p	LOC_Os11g30640.1	313	-53.59	157	-28.87	922172	20	477	111 329
miR1846d-3p	LOC_Os11g30740.1	151	-23.12	151	-23.12	922180	20	2094	1563
miR1846d-3p	LOC_Os11g30780.1	151	-27.95	151	-27.95	922185	20	531	233
miR1846d-3p	LOC_Os11g30840.1	158	-22.2	158	-22.2	922190	20	2694	236
miR1846d-3p	LOC_Os11g30910.1	154	-22.54	154	-22.54	922194	20	1756	636
miR1846d-3p	LOC_Os11g30930.1	141	-20.36	141	-20.36	922196	20	1125	381
miR1846d-3p	LOC_Os11g30970.1	144	-22.94	144	-22.94	922200	20	2658	435
miR1846d-3p	LOC_Os11g31390.1	154	-25.75	154	-25.75	922237	20	945	387
miR1846d-3p	LOC_Os11g31480.1	144	-22.66	144	-22.66	922249	20	5421	2379
miR1846d-3p	LOC_Os11g31500.1	152	-23.88	152	-23.88	922251	20	5025	4977
miR1846d-3p	LOC_Os11g31840.1	146	-20.01	146	-20.01	922292	20	4011	17
miR1846d-3p	LOC_Os11g31890.1	308	-49.98	156	-26.47	922297	20	5249	5163 5130
miR1846d-3p	LOC_Os11g31910.1	166	-26.59	166	-26.59	922301	20	3358	810
miR1846d-3p	LOC_Os11g32090.1	149	-20.07	149	-20.07	922318	20	588	252
miR1846d-3p	LOC_Os11g32110.1	147	-23.54	147	-23.54	922320	20	3570	3354
miR1846d-3p	LOC_Os11g32110.2	147	-23.54	147	-23.54	922321	20	3562	3346
miR1846d-3p	LOC_Os11g32110.3	147	-23.54	147	-23.54	922322	20	3567	3351
miR1846d-3p	LOC_Os11g32110.4	147	-23.54	147	-23.54	922323	20	3519	3303
miR1846d-3p	LOC_Os11g32250.1	913	-150.03	160	-29.11	922336	20	764	315 348 567 598 658

miR1846d-3p	LOC_Os11g32620.1	147	-25.9	147	-25.9	922377	20	1224	986
miR1846d-3p	LOC_Os11g32630.1	150	-22.47	150	-22.47	922378	20	765	6
miR1846d-3p	LOC_Os11g32880.1	160	-23.92	160	-23.92	922403	20	3211	1364
miR1846d-3p	LOC_Os11g32880.3	160	-23.92	160	-23.92	922405	20	3228	1364
miR1846d-3p	LOC_Os11g33020.1	149	-22.27	149	-22.27	922423	20	2166	505
miR1846d-3p	LOC_Os11g33030.1	148	-22.15	148	-22.15	922424	20	1618	1213
miR1846d-3p	LOC_Os11g33190.1	157	-21.94	157	-21.94	922441	20	2725	1981
miR1846d-3p	LOC_Os11g33190.2	157	-21.94	157	-21.94	922442	20	2769	2025
miR1846d-3p	LOC_Os11g33220.1	152	-24.34	152	-24.34	922445	20	1755	894
miR1846d-3p	LOC_Os11g33454.1	156	-26.09	156	-26.09	922468	20	1236	1082
miR1846d-3p	LOC_Os11g34410.1	305	-47.07	158	-25.45	922532	20	5220	183 2090
miR1846d-3p	LOC_Os11g34460.1	154	-20.9	154	-20.9	922537	20	2199	1710
miR1846d-3p	LOC_Os11g34460.2	154	-20.9	154	-20.9	922538	20	3837	1710
miR1846d-3p	LOC_Os11g34460.3	154	-20.9	154	-20.9	922539	20	2250	1710
miR1846d-3p	LOC_Os11g34500.1	305	-47.07	158	-25.45	922543	20	5403	507 2207
miR1846d-3p	LOC_Os11g34590.1	166	-26.59	166	-26.59	922551	20	2940	659
miR1846d-3p	LOC_Os11g34810.1	163	-27.07	163	-27.07	922570	20	1590	1436
miR1846d-3p	LOC_Os11g34810.2	163	-27.07	163	-27.07	922571	20	1818	1664
miR1846d-3p	LOC_Os11g35400.1	146	-20.71	146	-20.71	922633	20	2423	1271
miR1846d-3p	LOC_Os11g35400.2	146	-20.71	146	-20.71	922634	20	2354	1222
miR1846d-3p	LOC_Os11g35400.3	146	-20.71	146	-20.71	922635	20	2358	1226
miR1846d-3p	LOC_Os11g35400.4	146	-20.71	146	-20.71	922636	20	2532	1222
miR1846d-3p	LOC_Os11g35690.1	149	-21.28	149	-21.28	922665	20	2370	397
miR1846d-3p	LOC_Os11g35840.1	144	-22.69	144	-22.69	922681	20	4503	3210
miR1846d-3p	LOC_Os11g36000.1	303	-52	157	-30.65	922699	20	2213	371 298
miR1846d-3p	LOC_Os11g36370.1	166	-27.37	166	-27.37	922744	20	2691	302
miR1846d-3p	LOC_Os11g36400.1	150	-21.11	150	-21.11	922747	20	2043	1094
miR1846d-3p	LOC_Os11g36500.1	144	-22.87	144	-22.87	922761	20	957	644
miR1846d-3p	LOC_Os11g36530.1	161	-31.35	161	-31.35	922764	20	873	747
miR1846d-3p	LOC_Os11g36630.1	151	-24.69	151	-24.69	922773	20	2184	1884
miR1846d-3p	LOC_Os11g37040.1	162	-22.84	162	-22.84	922810	20	3758	1528
miR1846d-3p	LOC_Os11g37230.1	148	-25.62	148	-25.62	922828	20	1228	893

miR1846d-3p	LOC_Os11g37230.2	148	-25.62	148	-25.62	922829	20	2667	2299
miR1846d-3p	LOC_Os11g37490.1	150	-23.84	150	-23.84	922854	20	787	404
miR1846d-3p	LOC_Os11g37820.1	295	-41.92	148	-21.62	922891	20	5901	507 2546
miR1846d-3p	LOC_Os11g37830.1	172	-35.42	172	-35.42	922892	20	3093	1756
miR1846d-3p	LOC_Os11g38160.1	309	-52.89	157	-26.47	922927	20	1943	1446 76
miR1846d-3p	LOC_Os11g38350.1	158	-28.91	158	-28.91	922948	20	1398	507
miR1846d-3p	LOC_Os11g38530.1	166	-27.37	166	-27.37	922966	20	2649	470
miR1846d-3p	LOC_Os11g38680.1	156	-27.67	156	-27.67	922981	20	498	310
miR1846d-3p	LOC_Os11g38850.1	160	-31.76	160	-31.76	922999	20	2031	748
miR1846d-3p	LOC_Os11g38890.1	156	-25.15	156	-25.15	923004	20	4410	1552
miR1846d-3p	LOC_Os11g38900.1	144	-20.68	144	-20.68	923005	20	3318	2215
miR1846d-3p	LOC_Os11g39130.1	151	-21.06	151	-21.06	923034	20	1489	270
miR1846d-3p	LOC_Os11g39130.3	151	-21.06	151	-21.06	923035	20	1073	270
miR1846d-3p	LOC_Os11g39130.4	151	-21.06	151	-21.06	923036	20	1575	270
miR1846d-3p	LOC_Os11g39320.1	146	-21.74	146	-21.74	923059	20	3408	2871
miR1846d-3p	LOC_Os11g39870.1	147	-21.62	147	-21.62	923116	20	4980	1667
miR1846d-3p	LOC_Os11g39900.1	172	-34.12	172	-34.12	923119	20	2979	1642
miR1846d-3p	LOC_Os11g40050.1	166	-27.37	166	-27.37	923131	20	3303	671
miR1846d-3p	LOC_Os11g40820.1	147	-21.62	147	-21.62	923225	20	5898	2525
miR1846d-3p	LOC_Os11g40830.1	172	-34.12	172	-34.12	923226	20	3286	1949
miR1846d-3p	LOC_Os11g41090.1	621	-102.01	161	-27.51	923249	20	276	78 181 47 15
miR1846d-3p	LOC_Os11g41570.1	142	-23.28	142	-23.28	923299	20	1425	926
miR1846d-3p	LOC_Os11g41590.1	155	-24.55	155	-24.55	923301	20	1389	589
miR1846d-3p	LOC_Os11g41750.1	157	-23.73	157	-23.73	923318	20	2436	338
miR1846d-3p	LOC_Os11g41760.1	145	-20.08	145	-20.08	923319	20	5985	4365
miR1846d-3p	LOC_Os11g42030.1	145	-22.52	145	-22.52	923347	20	2696	586
miR1846d-3p	LOC_Os11g42040.1	306	-46.51	156	-25.32	923348	20	3871	3650 3452
miR1846d-3p	LOC_Os11g42140.1	148	-27.59	148	-27.59	923359	20	6087	4511
miR1846d-3p	LOC_Os11g42280.1	154	-23.29	154	-23.29	923373	20	1140	984
miR1846d-3p	LOC_Os11g42330.1	152	-21.82	152	-21.82	923378	20	2466	1234
miR1846d-3p	LOC_Os11g42390.1	164	-30.62	164	-30.62	923386	20	1389	511
miR1846d-3p	LOC_Os11g42400.1	304	-50.25	154	-25.99	923387	20	627	261 423

miR1846d-3p	LOC_Os11g42450.1	146	-22.64	146	-22.64	923393	20	1444	988
miR1846d-3p	LOC_Os11g42710.1	144	-22.02	144	-22.02	923420	20	3441	1791
miR1846d-3p	LOC_Os11g42810.1	146	-25.42	146	-25.42	923430	20	988	53
miR1846d-3p	LOC_Os11g42920.1	310	-47.24	157	-25.07	923442	20	519	141 172
miR1846d-3p	LOC_Os11g43140.1	153	-25	153	-25	923464	20	4644	1668
miR1846d-3p	LOC_Os11g43210.1	149	-27.51	149	-27.51	923470	20	1245	1188
miR1846d-3p	LOC_Os11g43290.1	157	-23.86	157	-23.86	923478	20	3369	157
miR1846d-3p	LOC_Os11g43380.1	148	-21.6	148	-21.6	923487	20	2436	1207
miR1846d-3p	LOC_Os11g43480.1	159	-27.33	159	-27.33	923503	20	3147	2657
miR1846d-3p	LOC_Os11g43500.1	152	-27.6	152	-27.6	923505	20	1275	540
miR1846d-3p	LOC_Os11g43610.1	151	-22.86	151	-22.86	923519	20	1091	848
miR1846d-3p	LOC_Os11g43650.1	150	-25.99	150	-25.99	923524	20	645	439
miR1846d-3p	LOC_Os11g43670.1	147	-21.62	147	-21.62	923526	20	6084	2546
miR1846d-3p	LOC_Os11g43680.1	172	-35.42	172	-35.42	923527	20	3210	1873
miR1846d-3p	LOC_Os11g43914.1	148	-23.72	148	-23.72	923552	20	701	523
miR1846d-3p	LOC_Os11g43934.2	144	-21.52	144	-21.52	923555	20	4366	4195
miR1846d-3p	LOC_Os11g44000.1	150	-21.93	150	-21.93	923561	20	1104	559
miR1846d-3p	LOC_Os11g44050.1	152	-20.54	152	-20.54	923565	20	2466	397
miR1846d-3p	LOC_Os11g44070.1	145	-20.08	145	-20.08	923567	20	2472	1059
miR1846d-3p	LOC_Os11g44420.1	159	-28.66	159	-28.66	923601	20	751	460
miR1846d-3p	LOC_Os11g44430.1	159	-28.66	159	-28.66	923602	20	1823	1597
miR1846d-3p	LOC_Os11g44570.1	152	-31.52	152	-31.52	923616	20	1236	264
miR1846d-3p	LOC_Os11g44950.1	144	-21.49	144	-21.49	923657	20	2881	975
miR1846d-3p	LOC_Os11g44950.2	144	-21.49	144	-21.49	923658	20	2839	975
miR1846d-3p	LOC_Os11g45230.1	157	-23.73	157	-23.73	923685	20	2436	338
miR1846d-3p	LOC_Os11g45510.1	150	-25.66	150	-25.66	923714	20	1971	1658
miR1846d-3p	LOC_Os11g45880.1	142	-22.55	142	-22.55	923754	20	2121	1214
miR1846d-3p	LOC_Os11g46220.1	149	-23.18	149	-23.18	923791	20	258	134
miR1846d-3p	LOC_Os11g46980.1	145	-21.32	145	-21.32	923821	20	3519	343
miR1846d-3p	LOC_Os11g47190.1	147	-21.61	147	-21.61	923841	20	6084	2546
miR1846d-3p	LOC_Os11g47350.1	142	-23.91	142	-23.91	923857	20	2451	247
miR1846d-3p	LOC_Os11g47390.1	147	-21.58	147	-21.58	923860	20	2225	1710

miR1846d-3p	LOC_Os11g47420.1	140	-20.15	140	-20.15	923863	20	3207	1522
miR1846d-3p	LOC_Os11g47448.1	152	-26.13	152	-26.13	923881	20	852	623
miR1846d-3p	LOC_Os11g47560.1	143	-23.07	143	-23.07	923899	20	1095	584
miR1846d-3p	LOC_Os11g47600.1	147	-22.34	147	-22.34	923904	20	1293	312
miR1846d-3p	LOC_Os12g01020.1	306	-56.01	165	-31.71	923969	20	2538	1250 1979
miR1846d-3p	LOC_Os12g01360.1	149	-21.61	149	-21.61	924011	20	3908	681
miR1846d-3p	LOC_Os12g01910.1	147	-23.19	147	-23.19	924073	20	2664	2026
miR1846d-3p	LOC_Os12g02150.1	148	-25.14	148	-25.14	924109	20	1002	473
miR1846d-3p	LOC_Os12g02200.1	148	-24.85	148	-24.85	924113	20	2159	280
miR1846d-3p	LOC_Os12g02200.2	148	-24.85	148	-24.85	924114	20	2193	280
miR1846d-3p	LOC_Os12g02460.1	146	-23.14	146	-23.14	924148	20	1671	1510
miR1846d-3p	LOC_Os12g02610.1	151	-27.36	151	-27.36	924165	20	615	254
miR1846d-3p	LOC_Os12g02670.1	146	-20.01	146	-20.01	924177	20	1659	803
miR1846d-3p	LOC_Os12g03100.1	142	-20.13	142	-20.13	924235	20	1934	1847
miR1846d-3p	LOC_Os12g03480.1	156	-27.01	156	-27.01	924276	20	2780	141
miR1846d-3p	LOC_Os12g03720.1	148	-21.71	148	-21.71	924299	20	2473	1830
miR1846d-3p	LOC_Os12g03720.2	148	-21.71	148	-21.71	924300	20	2935	1830
miR1846d-3p	LOC_Os12g03730.1	150	-22.52	150	-22.52	924301	20	2073	1740
miR1846d-3p	LOC_Os12g03816.1	150	-22	150	-22	924312	20	1381	602
miR1846d-3p	LOC_Os12g04040.1	162	-24.8	162	-24.8	924343	20	3111	659
miR1846d-3p	LOC_Os12g04380.1	149	-21.18	149	-21.18	924386	20	1395	823
miR1846d-3p	LOC_Os12g04400.1	308	-49.78	157	-27.3	924388	20	246	147 180
miR1846d-3p	LOC_Os12g04820.1	157	-24.51	157	-24.51	924435	20	2037	161
miR1846d-3p	LOC_Os12g04840.1	153	-27.51	153	-27.51	924437	20	4419	3884
miR1846d-3p	LOC_Os12g04934.1	150	-24.71	150	-24.71	924450	20	784	658
miR1846d-3p	LOC_Os12g04934.2	150	-24.71	150	-24.71	924451	20	873	747
miR1846d-3p	LOC_Os12g04934.3	150	-24.71	150	-24.71	924452	20	659	551
miR1846d-3p	LOC_Os12g05040.3	148	-21.2	148	-21.2	924467	20	2595	2472
miR1846d-3p	LOC_Os12g05040.5	148	-21.2	148	-21.2	924468	20	2821	2698
miR1846d-3p	LOC_Os12g05040.6	148	-21.2	148	-21.2	924469	20	2365	2242
miR1846d-3p	LOC_Os12g05190.1	303	-46.73	153	-24.39	924484	20	612	418 440
miR1846d-3p	LOC_Os12g05550.1	156	-28.84	156	-28.84	924520	20	1579	290

miR1846d-3p	LOC_Os12g05609.1	155	-26.16	155	-26.16	924526	20	1260	600
miR1846d-3p	LOC_Os12g05715.1	295	-41.09	148	-20.63	924541	20	852	291 322
miR1846d-3p	LOC_Os12g06220.1	148	-24.97	148	-24.97	924592	20	1002	397
miR1846d-3p	LOC_Os12g06490.2	147	-21.21	147	-21.21	924622	20	1979	1602
miR1846d-3p	LOC_Os12g06600.1	152	-20.57	152	-20.57	924635	20	915	556
miR1846d-3p	LOC_Os12g06690.1	148	-23.93	148	-23.93	924647	20	2301	36
miR1846d-3p	LOC_Os12g07500.1	150	-22.03	150	-22.03	924739	20	1464	1101
miR1846d-3p	LOC_Os12g07874.1	152	-28.57	152	-28.57	924779	20	2962	1687
miR1846d-3p	LOC_Os12g07900.1	164	-33.03	164	-33.03	924782	20	2007	14
miR1846d-3p	LOC_Os12g07990.1	153	-28.33	153	-28.33	924796	20	2217	284
miR1846d-3p	LOC_Os12g08050.1	153	-28.33	153	-28.33	924803	20	924	284
miR1846d-3p	LOC_Os12g08640.1	158	-26.77	158	-26.77	924867	20	843	84
miR1846d-3p	LOC_Os12g08900.1	147	-24.41	147	-24.41	924896	20	2046	1819
miR1846d-3p	LOC_Os12g09020.1	154	-20.83	154	-20.83	924908	20	693	640
miR1846d-3p	LOC_Os12g09070.1	157	-27.79	157	-27.79	924912	20	681	641
miR1846d-3p	LOC_Os12g09130.1	153	-28.86	153	-28.86	924917	20	816	270
miR1846d-3p	LOC_Os12g09140.1	152	-25.79	152	-25.79	924918	20	375	41
miR1846d-3p	LOC_Os12g09190.1	153	-28.86	153	-28.86	924922	20	2898	784
miR1846d-3p	LOC_Os12g09210.1	149	-22.82	149	-22.82	924924	20	2797	1816
miR1846d-3p	LOC_Os12g09290.1	145	-22.48	145	-22.48	924932	20	2040	148
miR1846d-3p	LOC_Os12g09455.1	162	-30.51	162	-30.51	924951	20	1608	775
miR1846d-3p	LOC_Os12g09630.1	157	-27.76	157	-27.76	924972	20	864	797
miR1846d-3p	LOC_Os12g09800.1	147	-22.16	147	-22.16	924988	20	4239	1446
miR1846d-3p	LOC_Os12g09840.1	147	-21.62	147	-21.62	924991	20	4539	1565
miR1846d-3p	LOC_Os12g09870.1	156	-24.89	156	-24.89	924994	20	2466	1234
miR1846d-3p	LOC_Os12g09954.1	150	-20.26	150	-20.26	925002	20	1611	415
miR1846d-3p	LOC_Os12g09990.1	144	-21.65	144	-21.65	925004	20	3864	1641
miR1846d-3p	LOC_Os12g10680.1	147	-22.67	147	-22.67	925082	20	2112	2061
miR1846d-3p	LOC_Os12g11530.1	155	-27.26	155	-27.26	925167	20	237	59
miR1846d-3p	LOC_Os12g11650.1	148	-22.83	148	-22.83	925178	20	2313	2117
miR1846d-3p	LOC_Os12g11750.1	158	-22.95	158	-22.95	925188	20	2121	470
miR1846d-3p	LOC_Os12g11830.1	452	-73.9	152	-25.27	925196	20	790	249 362 412

miR1846d-3p	LOC_Os12g12150.1	166	-26.29	166	-26.29	925225	20	3132	659
miR1846d-3p	LOC_Os12g12180.1	146	-20.01	146	-20.01	925229	20	4407	278
miR1846d-3p	LOC_Os12g12370.1	148	-23.29	148	-23.29	925250	20	1032	373
miR1846d-3p	LOC_Os12g12470.1	158	-23.84	158	-23.84	925261	20	1541	257
miR1846d-3p	LOC_Os12g12700.1	295	-41.92	148	-21.62	925284	20	5964	507 2546
miR1846d-3p	LOC_Os12g12710.1	172	-35.42	172	-35.42	925285	20	3168	1831
miR1846d-3p	LOC_Os12g12840.1	142	-22.55	142	-22.55	925299	20	3288	1970
miR1846d-3p	LOC_Os12g12950.1	145	-20.06	145	-20.06	925310	20	1121	812
miR1846d-3p	LOC_Os12g13174.1	299	-51.15	155	-27.59	925339	20	3959	3556 3750
miR1846d-3p	LOC_Os12g13180.1	144	-23.56	144	-23.56	925340	20	2532	53
miR1846d-3p	LOC_Os12g13300.1	161	-32.8	161	-32.8	925353	20	2939	1970
miR1846d-3p	LOC_Os12g13480.1	147	-23.94	147	-23.94	925375	20	201	49
miR1846d-3p	LOC_Os12g13670.1	165	-28.18	165	-28.18	925393	20	4400	4235
miR1846d-3p	LOC_Os12g13670.2	165	-28.18	165	-28.18	925394	20	4313	4148
miR1846d-3p	LOC_Os12g13674.1	450	-74.59	152	-28.13	925395	20	1950	299 656 436
miR1846d-3p	LOC_Os12g13680.1	154	-24.21	154	-24.21	925396	20	456	213
miR1846d-3p	LOC_Os12g14240.1	162	-24.8	162	-24.8	925456	20	2631	404
miR1846d-3p	LOC_Os12g14630.1	152	-21.04	152	-21.04	925490	20	1740	786
miR1846d-3p	LOC_Os12g14740.1	156	-25.19	156	-25.19	925499	20	1929	1396
miR1846d-3p	LOC_Os12g14764.1	480	-78.27	166	-31.71	925501	20	795	536 748 589
miR1846d-3p	LOC_Os12g15090.1	148	-27.82	148	-27.82	925529	20	1170	1128
miR1846d-3p	LOC_Os12g15690.1	166	-26.59	166	-26.59	925589	20	1983	659
miR1846d-3p	LOC_Os12g16350.13	157	-25.12	157	-25.12	925659	20	4262	3006
miR1846d-3p	LOC_Os12g16370.1	152	-22.43	152	-22.43	925663	20	282	120
miR1846d-3p	LOC_Os12g16440.1	154	-23.99	154	-23.99	925672	20	1539	507
miR1846d-3p	LOC_Os12g16690.1	151	-23.33	151	-23.33	925700	20	3394	2190
miR1846d-3p	LOC_Os12g16840.1	299	-47.17	150	-24.22	925714	20	4200	2949 4134
miR1846d-3p	LOC_Os12g16920.1	152	-25.43	152	-25.43	925722	20	2388	2337
miR1846d-3p	LOC_Os12g17160.1	147	-28.08	147	-28.08	925747	20	1948	1187
miR1846d-3p	LOC_Os12g17500.1	152	-26.27	152	-26.27	925781	20	414	256
miR1846d-3p	LOC_Os12g17650.1	149	-29.81	149	-29.81	925797	20	591	494
miR1846d-3p	LOC_Os12g17670.1	150	-22.66	150	-22.66	925799	20	450	256

miR1846d-3p	LOC_Os12g18030.1	152	-20.54	152	-20.54	925835	20	2547	397
miR1846d-3p	LOC_Os12g18120.1	153	-26.68	153	-26.68	925846	20	2021	1475
miR1846d-3p	LOC_Os12g18120.2	153	-26.68	153	-26.68	925847	20	2018	1472
miR1846d-3p	LOC_Os12g18120.4	153	-26.68	153	-26.68	925848	20	1651	1105
miR1846d-3p	LOC_Os12g18130.1	147	-22.91	147	-22.91	925849	20	1732	1699
miR1846d-3p	LOC_Os12g18130.2	147	-22.91	147	-22.91	925850	20	668	635
miR1846d-3p	LOC_Os12g18470.1	146	-20.92	146	-20.92	925886	20	1080	412
miR1846d-3p	LOC_Os12g18990.1	158	-25.45	158	-25.45	925942	20	639	168
miR1846d-3p	LOC_Os12g19000.1	147	-21.62	147	-21.62	925943	20	4512	1787
miR1846d-3p	LOC_Os12g19110.1	157	-23.87	157	-23.87	925950	20	303	208
miR1846d-3p	LOC_Os12g19770.1	151	-21.79	151	-21.79	926014	20	4674	3552
miR1846d-3p	LOC_Os12g20000.1	140	-21.83	140	-21.83	926037	20	2199	38
miR1846d-3p	LOC_Os12g20050.1	148	-21.47	148	-21.47	926042	20	837	107
miR1846d-3p	LOC_Os12g20210.1	152	-24.39	152	-24.39	926058	20	4140	3094
miR1846d-3p	LOC_Os12g20290.1	157	-24.4	157	-24.4	926066	20	2295	468
miR1846d-3p	LOC_Os12g20350.1	148	-20.15	148	-20.15	926074	20	2490	1260
miR1846d-3p	LOC_Os12g20390.1	159	-31.53	159	-31.53	926078	20	1793	1481
miR1846d-3p	LOC_Os12g20390.2	159	-31.53	159	-31.53	926079	20	1069	757
miR1846d-3p	LOC_Os12g20390.3	159	-31.53	159	-31.53	926080	20	1572	1260
miR1846d-3p	LOC_Os12g20390.4	159	-31.53	159	-31.53	926081	20	1654	1342
miR1846d-3p	LOC_Os12g20390.5	159	-31.53	159	-31.53	926082	20	1302	990
miR1846d-3p	LOC_Os12g21780.1	150	-22.95	150	-22.95	926121	20	4626	3690
miR1846d-3p	LOC_Os12g21940.1	164	-30.27	164	-30.27	926142	20	2379	2180
miR1846d-3p	LOC_Os12g22145.1	157	-25.31	157	-25.31	926163	20	885	601
miR1846d-3p	LOC_Os12g22600.1	151	-23.92	151	-23.92	926208	20	2069	65
miR1846d-3p	LOC_Os12g22940.1	152	-24.51	152	-24.51	926243	20	1254	433
miR1846d-3p	LOC_Os12g23090.1	152	-24.51	152	-24.51	926254	20	1215	427
miR1846d-3p	LOC_Os12g23220.1	152	-20.54	152	-20.54	926268	20	2502	397
miR1846d-3p	LOC_Os12g23230.1	145	-20.08	145	-20.08	926269	20	6033	4422
miR1846d-3p	LOC_Os12g23520.1	144	-25.97	144	-25.97	926297	20	6172	3134
miR1846d-3p	LOC_Os12g23770.1	318	-54.85	166	-31.79	926322	20	2958	573 4
miR1846d-3p	LOC_Os12g23790.1	153	-22.46	153	-22.46	926324	20	2259	601

miR1846d-3p	LOC_Os12g23920.1	145	-20.08	145	-20.08	926334	20	5955	4344
miR1846d-3p	LOC_Os12g24040.1	142	-20.24	142	-20.24	926347	20	2275	1088
miR1846d-3p	LOC_Os12g24220.1	156	-26.67	156	-26.67	926363	20	4794	1423
miR1846d-3p	LOC_Os12g24550.1	148	-24.19	148	-24.19	926392	20	4545	4455
miR1846d-3p	LOC_Os12g25030.1	159	-24.36	159	-24.36	926437	20	831	3
miR1846d-3p	LOC_Os12g25400.1	142	-21.31	142	-21.31	926473	20	436	94
miR1846d-3p	LOC_Os12g25440.1	146	-25.62	146	-25.62	926476	20	666	315
miR1846d-3p	LOC_Os12g25610.1	148	-24.23	148	-24.23	926495	20	585	539
miR1846d-3p	LOC_Os12g25760.1	458	-71.72	156	-26.09	926514	20	354	182 142 322
miR1846d-3p	LOC_Os12g26240.1	147	-20.79	147	-20.79	926558	20	5952	2537
miR1846d-3p	LOC_Os12g26250.1	172	-34.12	172	-34.12	926559	20	3123	1786
miR1846d-3p	LOC_Os12g26290.1	148	-24.33	148	-24.33	926563	20	2346	1435
miR1846d-3p	LOC_Os12g26290.2	148	-24.33	148	-24.33	926564	20	2412	1435
miR1846d-3p	LOC_Os12g26290.3	148	-24.33	148	-24.33	926565	20	2334	1435
miR1846d-3p	LOC_Os12g26290.4	148	-24.33	148	-24.33	926566	20	2254	1435
miR1846d-3p	LOC_Os12g26290.5	148	-24.33	148	-24.33	926567	20	2833	1922
miR1846d-3p	LOC_Os12g26290.6	148	-24.33	148	-24.33	926568	20	2219	1308
miR1846d-3p	LOC_Os12g26680.1	301	-51.22	153	-26.12	926604	20	342	318 159
miR1846d-3p	LOC_Os12g27030.1	149	-25.46	149	-25.46	926640	20	354	325
miR1846d-3p	LOC_Os12g27335.1	156	-28.28	156	-28.28	926673	20	609	186
miR1846d-3p	LOC_Os12g27710.1	145	-20.08	145	-20.08	926709	20	5871	4239
miR1846d-3p	LOC_Os12g27790.1	151	-24.6	151	-24.6	926717	20	1407	794
miR1846d-3p	LOC_Os12g28015.1	158	-26.39	158	-26.39	926739	20	621	511
miR1846d-3p	LOC_Os12g28200.1	152	-21.82	152	-21.82	926754	20	2463	1234
miR1846d-3p	LOC_Os12g28290.1	297	-45	157	-24.99	926764	20	3951	3717 3866
miR1846d-3p	LOC_Os12g28360.1	147	-21.62	147	-21.62	926770	20	6093	2555
miR1846d-3p	LOC_Os12g28400.1	172	-34.12	172	-34.12	926774	20	3207	1870
miR1846d-3p	LOC_Os12g28810.1	142	-22.55	142	-22.55	926812	20	3355	1970
miR1846d-3p	LOC_Os12g29350.1	151	-20.82	151	-20.82	926863	20	5995	5925
miR1846d-3p	LOC_Os12g29510.1	165	-26.63	165	-26.63	926878	20	516	59
miR1846d-3p	LOC_Os12g30180.1	144	-20.57	144	-20.57	926955	20	1612	217
miR1846d-3p	LOC_Os12g30310.1	146	-20.01	146	-20.01	926966	20	4086	272

miR1846d-3p	LOC_Os12g30350.1	166	-26.29	166	-26.29	926970	20	3123	659
miR1846d-3p	LOC_Os12g31060.1	298	-47.74	155	-27.05	927038	20	318	147 131
miR1846d-3p	LOC_Os12g31070.1	148	-22.31	148	-22.31	927039	20	5475	2235
miR1846d-3p	LOC_Os12g31120.1	149	-21.64	149	-21.64	927044	20	1221	749
miR1846d-3p	LOC_Os12g31510.1	141	-20.36	141	-20.36	927080	20	999	697
miR1846d-3p	LOC_Os12g31680.1	148	-23.65	148	-23.65	927097	20	687	424
miR1846d-3p	LOC_Os12g31780.2	157	-23.95	157	-23.95	927107	20	1006	881
miR1846d-3p	LOC_Os12g32080.1	154	-33.15	154	-33.15	927142	20	741	657
miR1846d-3p	LOC_Os12g32190.1	146	-23.33	146	-23.33	927152	20	2325	784
miR1846d-3p	LOC_Os12g32190.2	146	-23.33	146	-23.33	927153	20	2240	784
miR1846d-3p	LOC_Os12g32536.1	150	-20.84	150	-20.84	927187	20	2200	769
miR1846d-3p	LOC_Os12g32536.2	150	-20.84	150	-20.84	927188	20	2234	769
miR1846d-3p	LOC_Os12g32536.3	150	-20.84	150	-20.84	927189	20	1899	769
miR1846d-3p	LOC_Os12g32536.4	150	-20.84	150	-20.84	927190	20	2095	769
miR1846d-3p	LOC_Os12g32680.1	145	-23.27	145	-23.27	927205	20	3627	3276
miR1846d-3p	LOC_Os12g32850.1	150	-20.34	150	-20.34	927222	20	1935	552
miR1846d-3p	LOC_Os12g33289.1	316	-53.06	158	-26.86	927267	20	2387	1861 2164
miR1846d-3p	LOC_Os12g33415.1	156	-23.03	156	-23.03	927277	20	744	512
miR1846d-3p	LOC_Os12g33890.1	152	-20.54	152	-20.54	927321	20	2496	397
miR1846d-3p	LOC_Os12g33946.1	145	-22.93	145	-22.93	927329	20	1575	1336
miR1846d-3p	LOC_Os12g34300.1	156	-26.35	156	-26.35	927378	20	1954	602
miR1846d-3p	LOC_Os12g34700.1	146	-20.01	146	-20.01	927419	20	3732	17
miR1846d-3p	LOC_Os12g34900.1	147	-27.98	147	-27.98	927446	20	2613	1107
miR1846d-3p	LOC_Os12g34900.3	147	-27.98	147	-27.98	927447	20	1125	696
miR1846d-3p	LOC_Os12g34950.1	171	-36.05	171	-36.05	927451	20	4704	2816
miR1846d-3p	LOC_Os12g35280.1	147	-22.76	147	-22.76	927488	20	4359	2573
miR1846d-3p	LOC_Os12g35350.1	158	-25.58	158	-25.58	927494	20	2599	291
miR1846d-3p	LOC_Os12g35350.2	158	-25.58	158	-25.58	927495	20	2495	291
miR1846d-3p	LOC_Os12g35590.1	453	-64.33	153	-22.06	927525	20	1320	664 526 643
miR1846d-3p	LOC_Os12g36320.1	304	-45.83	158	-25.14	927598	20	690	187 240
miR1846d-3p	LOC_Os12g36360.1	156	-26.16	156	-26.16	927602	20	804	719
miR1846d-3p	LOC_Os12g36540.1	148	-24.97	148	-24.97	927620	20	243	59

miR1846d-3p	LOC_Os12g36670.1	157	-29.81	157	-29.81	927633	20	2972	143
miR1846d-3p	LOC_Os12g36735.1	437	-71.73	152	-25.51	927640	20	567	366 336 305
miR1846d-3p	LOC_Os12g36780.1	147	-21.62	147	-21.62	927648	20	5715	2177
miR1846d-3p	LOC_Os12g36790.1	172	-34.12	172	-34.12	927649	20	3318	1981
miR1846d-3p	LOC_Os12g36850.1	152	-23.12	152	-23.12	927655	20	828	168
miR1846d-3p	LOC_Os12g36890.1	158	-22.37	158	-22.37	927659	20	4184	2357
miR1846d-3p	LOC_Os12g37260.1	161	-27.69	161	-27.69	927698	20	3218	292
miR1846d-3p	LOC_Os12g37340.1	145	-20.08	145	-20.08	927707	20	5976	4356
miR1846d-3p	LOC_Os12g37450.1	166	-26.29	166	-26.29	927722	20	3114	659
miR1846d-3p	LOC_Os12g37760.1	154	-25.62	154	-25.62	927763	20	3368	149
miR1846d-3p	LOC_Os12g37939.1	152	-25.08	152	-25.08	927782	20	1872	1698
miR1846d-3p	LOC_Os12g38040.1	144	-22.67	144	-22.67	927794	20	2239	1503
miR1846d-3p	LOC_Os12g38210.1	152	-26.57	152	-26.57	927808	20	2519	1847
miR1846d-3p	LOC_Os12g38820.1	468	-75.18	157	-25.57	927883	20	279	164 248 23
miR1846d-3p	LOC_Os12g38860.1	150	-20.74	150	-20.74	927889	20	2792	577
miR1846d-3p	LOC_Os12g38930.1	156	-22.54	156	-22.54	927898	20	2789	2276
miR1846d-3p	LOC_Os12g39590.1	145	-25.21	145	-25.21	927978	20	3963	2941
miR1846d-3p	LOC_Os12g39620.1	308	-52.17	155	-30.12	927981	20	2998	583 1816
miR1846d-3p	LOC_Os12g39620.2	153	-22.05	153	-22.05	927982	20	3127	1163
miR1846d-3p	LOC_Os12g39620.3	153	-22.05	153	-22.05	927983	20	2812	848
miR1846d-3p	LOC_Os12g39620.4	153	-22.05	153	-22.05	927984	20	2680	716
miR1846d-3p	LOC_Os12g39620.5	153	-22.05	153	-22.05	927985	20	2768	804
miR1846d-3p	LOC_Os12g39760.1	142	-22.55	142	-22.55	927999	20	3288	1970
miR1846d-3p	LOC_Os12g40010.1	147	-23.64	147	-23.64	928028	20	3783	1796
miR1846d-3p	LOC_Os12g40010.2	147	-23.64	147	-23.64	928029	20	3876	1794
miR1846d-3p	LOC_Os12g40250.1	142	-22.55	142	-22.55	928053	20	2541	1637
miR1846d-3p	LOC_Os12g40540.1	149	-23.95	149	-23.95	928086	20	1763	1398
miR1846d-3p	LOC_Os12g40670.1	166	-26.59	166	-26.59	928101	20	1623	608
miR1846d-3p	LOC_Os12g40810.1	305	-50.53	158	-28.91	928115	20	5727	342 2381
miR1846d-3p	LOC_Os12g41000.1	305	-50.53	158	-28.91	928138	20	5496	168 2051
miR1846d-3p	LOC_Os12g41060.1	149	-23.02	149	-23.02	928144	20	2141	2016
miR1846d-3p	LOC_Os12g41124.1	147	-21.5	147	-21.5	928150	20	2710	253

miR1846d-3p	LOC_Os12g41140.1	141	-21.5	141	-21.5	928152	20	2042	1957
miR1846d-3p	LOC_Os12g41140.3	141	-21.5	141	-21.5	928154	20	2041	1956
miR1846d-3p	LOC_Os12g41250.1	146	-23.69	146	-23.69	928167	20	2736	2254
miR1846d-3p	LOC_Os12g41590.1	153	-22.2	153	-22.2	928203	20	1304	1069
miR1846d-3p	LOC_Os12g41700.1	152	-24.08	152	-24.08	928219	20	1199	1034
miR1846d-3p	LOC_Os12g42044.1	157	-28.14	157	-28.14	928270	20	4172	1121
miR1846d-3p	LOC_Os12g42044.2	157	-28.14	157	-28.14	928271	20	2305	1118
miR1846d-3p	LOC_Os12g42044.3	157	-28.14	157	-28.14	928272	20	2279	1082
miR1846d-3p	LOC_Os12g42044.4	157	-28.14	157	-28.14	928273	20	4172	1121
miR1846d-3p	LOC_Os12g42044.5	157	-28.14	157	-28.14	928274	20	3655	1121
miR1846d-3p	LOC_Os12g42284.1	315	-53.71	164	-31.61	928303	20	807	771 750
miR1846d-3p	LOC_Os12g42370.1	154	-24.06	154	-24.06	928310	20	2380	2231
miR1846d-3p	LOC_Os12g42910.1	157	-24.65	157	-24.65	928379	20	2046	197
miR1846d-3p	LOC_Os12g43350.1	157	-24.66	157	-24.66	928420	20	1354	552
miR1846d-3p	LOC_Os12g43510.1	146	-20.48	146	-20.48	928437	20	817	565
miR1846d-3p	LOC_Os12g43564.1	146	-22.12	146	-22.12	928444	20	834	103
miR1846d-3p	LOC_Os12g43600.1	165	-31.86	165	-31.86	928448	20	864	109
miR1846d-3p	LOC_Os12g43664.1	156	-28.09	156	-28.09	928456	20	2289	1954
miR1846d-3p	LOC_Os12g43770.1	160	-32.38	160	-32.38	928466	20	1389	1348
miR1846d-3p	LOC_Os12g44180.1	148	-25.3	148	-25.3	928517	20	2301	2139
miR1846d-3p	LOC_Os12g44190.1	165	-29.71	165	-29.71	928518	20	2073	837
miR1846d-3p	LOC_Os12g44290.1	147	-21.73	147	-21.73	928530	20	1542	592
miR1846d-3p	ChrUn.fgenesh.mRNA.10	144	-22.94	144	-22.94	928557	20	2400	24
miR1846d-3p	ChrUn.fgenesh.mRNA.13	156	-23.89	156	-23.89	928560	20	645	351
miR1846d-3p	ChrUn.fgenesh.mRNA.80	290	-47.2	148	-25.23	928627	20	5409	125 3042
miR1846d-3p	ChrUn.fgenesh.mRNA.88	147	-22.22	147	-22.22	928635	20	5379	2157
miR1846d-3p	ChrUn.fgenesh.mRNA.89	172	-34.12	172	-34.12	928636	20	3474	2026
miR1846d-3p	ChrSy.fgenesh.mRNA.8	144	-20.35	144	-20.35	928651	20	561	208
miR1846d-3p	ChrSy.fgenesh.mRNA.12	147	-21.62	147	-21.62	928655	20	4860	1469
miR1846d-3p	ChrSy.fgenesh.mRNA.22	160	-26.8	160	-26.8	928665	20	5973	1036
miR1846d-3p	ChrSy.fgenesh.mRNA.24	147	-20.38	147	-20.38	928667	20	585	199
miR1846d-3p	ChrSy.fgenesh.mRNA.33	153	-26.87	153	-26.87	928676	20	1122	146

miR1846d-3p	ChrSy.fgenesh.mRNA.45	148	-23.29	148	-23.29	928688	20	333	122
miR1846d-3p	ChrSy.fgenesh.mRNA.55	147	-21.62	147	-21.62	928698	20	5100	1934
miR1846d-3p	ChrSy.fgenesh.mRNA.56	168	-33.65	168	-33.65	928699	20	1755	1672
miR1846d-3p	ChrSy.fgenesh.mRNA.72	305	-47.07	158	-25.45	928715	20	2583	507 2144
miR1846d-3p	ChrSy.fgenesh.mRNA.74	148	-23.29	148	-23.29	928717	20	1470	480
miR166e-3p	LOC_Os01g01510.1	153	-21.2	153	-21.2	928809	21	2425	1201
miR166e-3p	LOC_Os01g01510.2	153	-21.2	153	-21.2	928810	21	2506	1175
miR166e-3p	LOC_Os01g02910.1	174	-35.73	174	-35.73	928968	21	2195	530
miR166e-3p	LOC_Os01g03160.1	160	-22.8	160	-22.8	929001	21	1900	656
miR166e-3p	LOC_Os01g03160.2	160	-22.8	160	-22.8	929002	21	1804	656
miR166e-3p	LOC_Os01g05320.1	154	-22.34	154	-22.34	929236	21	4128	1912
miR166e-3p	LOC_Os01g05800.1	158	-21.38	158	-21.38	929293	21	2443	467
miR166e-3p	LOC_Os01g05800.2	158	-21.38	158	-21.38	929294	21	2440	467
miR166e-3p	LOC_Os01g06660.1	157	-23.53	157	-23.53	929390	21	2311	2248
miR166e-3p	LOC_Os01g06876.1	160	-23.03	160	-23.03	929410	21	1080	911
miR166e-3p	LOC_Os01g07590.1	150	-22.01	150	-22.01	929497	21	1377	289
miR166e-3p	LOC_Os01g07610.1	160	-20.74	160	-20.74	929499	21	1913	423
miR166e-3p	LOC_Os01g07940.1	156	-20.98	156	-20.98	929541	21	2543	1716
miR166e-3p	LOC_Os01g08140.1	152	-24.58	152	-24.58	929561	21	594	421
miR166e-3p	LOC_Os01g08480.1	154	-22.34	154	-22.34	929600	21	3963	1747
miR166e-3p	LOC_Os01g10230.1	163	-31.08	163	-31.08	929805	21	893	640
miR166e-3p	LOC_Os01g11414.1	154	-28.17	154	-28.17	929929	21	2076	504
miR166e-3p	LOC_Os01g11414.2	154	-28.17	154	-28.17	929930	21	1648	76
miR166e-3p	LOC_Os01g11990.1	161	-20.7	161	-20.7	929988	21	1397	1271
miR166e-3p	LOC_Os01g12740.1	158	-24.74	158	-24.74	930081	21	2005	242
miR166e-3p	LOC_Os01g12860.1	151	-20.2	151	-20.2	930098	21	3786	3069
miR166e-3p	LOC_Os01g13990.1	316	-44.22	161	-23.17	930231	21	891	770 307
miR166e-3p	LOC_Os01g14200.1	144	-20.31	144	-20.31	930256	21	7542	358
miR166e-3p	LOC_Os01g15790.4	160	-22.83	160	-22.83	930431	21	1658	1510
miR166e-3p	LOC_Os01g15910.1	154	-26.52	154	-26.52	930441	21	2262	1563
miR166e-3p	LOC_Os01g18040.1	303	-43.69	152	-23.51	930635	21	5331	3523 344
miR166e-3p	LOC_Os01g19800.1	175	-28.29	175	-28.29	930822	21	915	601

miR166e-3p	LOC_Os01g23310.1	151	-27.59	151	-27.59	931128	21	516	357
miR166e-3p	LOC_Os01g23970.1	154	-25.47	154	-25.47	931198	21	1277	573
miR166e-3p	LOC_Os01g24420.1	151	-22.43	151	-22.43	931244	21	2377	261
miR166e-3p	LOC_Os01g25580.1	154	-20.03	154	-20.03	931371	21	5775	4075
miR166e-3p	LOC_Os01g26240.1	154	-22.34	154	-22.34	931435	21	5112	1747
miR166e-3p	LOC_Os01g27170.1	146	-23.6	146	-23.6	931486	21	3152	779
miR166e-3p	LOC_Os01g27430.1	154	-21.72	154	-21.72	931509	21	1905	1835
miR166e-3p	LOC_Os01g27800.1	158	-22.78	158	-22.78	931545	21	2598	2050
miR166e-3p	LOC_Os01g29040.1	162	-22.09	162	-22.09	931677	21	804	54
miR166e-3p	LOC_Os01g29760.1	161	-22.56	161	-22.56	931744	21	3687	171
miR166e-3p	LOC_Os01g30980.1	156	-20.82	156	-20.82	931766	21	2193	2078
miR166e-3p	LOC_Os01g31460.1	154	-20.61	154	-20.61	931819	21	2277	1347
miR166e-3p	LOC_Os01g31840.1	154	-22.34	154	-22.34	931856	21	3963	1747
miR166e-3p	LOC_Os01g33100.1	154	-22.34	154	-22.34	931994	21	3963	1747
miR166e-3p	LOC_Os01g33460.1	150	-20.47	150	-20.47	932024	21	4548	485
miR166e-3p	LOC_Os01g33540.2	144	-20.58	144	-20.58	932036	21	2412	365
miR166e-3p	LOC_Os01g33910.1	162	-20.24	162	-20.24	932068	21	414	195
miR166e-3p	LOC_Os01g33960.1	150	-21.16	150	-21.16	932073	21	2142	556
miR166e-3p	LOC_Os01g33970.1	171	-23.45	171	-23.45	932074	21	3042	2767
miR166e-3p	LOC_Os01g34000.1	162	-20.24	162	-20.24	932077	21	255	189
miR166e-3p	LOC_Os01g34010.1	161	-24.66	161	-24.66	932078	21	2334	1129
miR166e-3p	LOC_Os01g34080.1	158	-21.49	158	-21.49	932084	21	3541	3261
miR166e-3p	LOC_Os01g36010.1	153	-20.05	153	-20.05	932289	21	2349	178
miR166e-3p	LOC_Os01g36960.1	154	-22.34	154	-22.34	932393	21	3963	1747
miR166e-3p	LOC_Os01g38480.1	163	-23.57	163	-23.57	932561	21	1103	215
miR166e-3p	LOC_Os01g38480.2	163	-23.57	163	-23.57	932562	21	995	215
miR166e-3p	LOC_Os01g38480.3	163	-23.57	163	-23.57	932563	21	1066	215
miR166e-3p	LOC_Os01g39060.1	158	-21.47	158	-21.47	932626	21	2812	2383
miR166e-3p	LOC_Os01g39960.1	150	-22.27	150	-22.27	932724	21	1973	1563
miR166e-3p	LOC_Os01g40660.1	150	-21.49	150	-21.49	932802	21	825	356
miR166e-3p	LOC_Os01g41480.1	153	-20.05	153	-20.05	932889	21	1371	760
miR166e-3p	LOC_Os01g41516.1	154	-22.34	154	-22.34	932893	21	1794	1693

miR166e-3p	LOC_Os01g41890.1	152	-22.71	152	-22.71	932932	21	2964	2779
miR166e-3p	LOC_Os01g41990.1	154	-20.47	154	-20.47	932942	21	1410	1241
miR166e-3p	LOC_Os01g42080.1	157	-22.74	157	-22.74	932953	21	3132	713
miR166e-3p	LOC_Os01g42330.1	150	-20.31	150	-20.31	932979	21	3783	2914
miR166e-3p	LOC_Os01g42540.1	298	-40.72	151	-20.67	933002	21	1290	382 585
miR166e-3p	LOC_Os01g42790.1	162	-20.86	162	-20.86	933034	21	1389	1350
miR166e-3p	LOC_Os01g42980.1	155	-20.35	155	-20.35	933058	21	1335	576
miR166e-3p	LOC_Os01g42980.2	155	-20.35	155	-20.35	933059	21	1440	576
miR166e-3p	LOC_Os01g42980.4	155	-20.35	155	-20.35	933061	21	1006	576
miR166e-3p	LOC_Os01g43740.1	151	-20.49	151	-20.49	933156	21	2009	418
miR166e-3p	LOC_Os01g43750.1	150	-20.49	150	-20.49	933157	21	1797	397
miR166e-3p	LOC_Os01g43844.1	150	-20.49	150	-20.49	933164	21	2060	476
miR166e-3p	LOC_Os01g45840.1	153	-20.53	153	-20.53	933326	21	1597	634
miR166e-3p	LOC_Os01g45860.1	163	-24.97	163	-24.97	933328	21	1678	1014
miR166e-3p	LOC_Os01g46840.1	154	-23.94	154	-23.94	933439	21	2885	474
miR166e-3p	LOC_Os01g46990.1	152	-21.43	152	-21.43	933458	21	204	31
miR166e-3p	LOC_Os01g47530.1	157	-28.11	157	-28.11	933524	21	3301	581
miR166e-3p	LOC_Os01g47530.4	157	-28.11	157	-28.11	933527	21	3224	581
miR166e-3p	LOC_Os01g48330.1	164	-26.6	164	-26.6	933625	21	3973	682
miR166e-3p	LOC_Os01g48670.1	154	-22.34	154	-22.34	933662	21	3963	1747
miR166e-3p	LOC_Os01g49920.1	157	-20.79	157	-20.79	933809	21	4002	1030
miR166e-3p	LOC_Os01g50020.1	162	-24.87	162	-24.87	933820	21	1872	134
miR166e-3p	LOC_Os01g51154.1	145	-22.59	145	-22.59	933945	21	1320	487
miR166e-3p	LOC_Os01g51154.2	145	-22.59	145	-22.59	933946	21	1314	481
miR166e-3p	LOC_Os01g51154.3	145	-22.59	145	-22.59	933947	21	1392	481
miR166e-3p	LOC_Os01g52050.1	162	-24.28	162	-24.28	934051	21	3945	3656
miR166e-3p	LOC_Os01g54160.1	169	-26.03	169	-26.03	934295	21	5346	4660
miR166e-3p	LOC_Os01g54570.1	157	-22.71	157	-22.71	934345	21	975	909
miR166e-3p	LOC_Os01g54620.1	158	-26.03	158	-26.03	934350	21	3479	1486
miR166e-3p	LOC_Os01g55110.1	149	-23.95	149	-23.95	934410	21	1315	217
miR166e-3p	LOC_Os01g55549.1	159	-21.18	159	-21.18	934470	21	2451	2407
miR166e-3p	LOC_Os01g56020.1	146	-25.92	146	-25.92	934529	21	2595	2486

miR166e-3p	LOC_Os01g56310.1	160	-23.54	160	-23.54	934568	21	1394	999
miR166e-3p	LOC_Os01g56550.1	151	-21.02	151	-21.02	934598	21	1296	1228
miR166e-3p	LOC_Os01g56550.2	151	-21.02	151	-21.02	934599	21	1293	1225
miR166e-3p	LOC_Os01g56550.3	151	-21.02	151	-21.02	934600	21	1310	1242
miR166e-3p	LOC_Os01g56630.1	163	-24.33	163	-24.33	934609	21	3467	1358
miR166e-3p	LOC_Os01g56630.2	163	-24.33	163	-24.33	934610	21	3513	1404
miR166e-3p	LOC_Os01g56690.1	154	-20.72	154	-20.72	934622	21	822	746
miR166e-3p	LOC_Os01g59620.1	147	-21.16	147	-21.16	934967	21	2945	200
miR166e-3p	LOC_Os01g60420.1	161	-21.4	161	-21.4	935060	21	1942	839
miR166e-3p	LOC_Os01g60600.1	150	-22.1	150	-22.1	935077	21	1733	1636
miR166e-3p	LOC_Os01g62100.1	150	-20.3	150	-20.3	935271	21	5409	4775
miR166e-3p	LOC_Os01g62480.1	160	-23.43	160	-23.43	935322	21	2076	254
miR166e-3p	LOC_Os01g62630.1	158	-27.47	158	-27.47	935339	21	1675	1553
miR166e-3p	LOC_Os01g62890.1	147	-20.54	147	-20.54	935374	21	1226	1145
miR166e-3p	LOC_Os01g62890.2	147	-20.54	147	-20.54	935375	21	1299	1218
miR166e-3p	LOC_Os01g63420.2	156	-20.82	156	-20.82	935435	21	2463	257
miR166e-3p	LOC_Os01g63420.3	156	-20.82	156	-20.82	935436	21	2543	257
miR166e-3p	LOC_Os01g63930.1	152	-23.74	152	-23.74	935491	21	3781	2551
miR166e-3p	LOC_Os01g63930.2	152	-23.74	152	-23.74	935492	21	2846	1616
miR166e-3p	LOC_Os01g63990.4	155	-23.83	155	-23.83	935501	21	1509	124
miR166e-3p	LOC_Os01g64590.1	154	-20.11	154	-20.11	935580	21	993	382
miR166e-3p	LOC_Os01g65100.1	153	-22.55	153	-22.55	935655	21	2051	1650
miR166e-3p	LOC_Os01g65100.2	153	-22.55	153	-22.55	935656	21	1896	1495
miR166e-3p	LOC_Os01g65100.3	153	-22.55	153	-22.55	935657	21	2715	2314
miR166e-3p	LOC_Os01g65340.1	150	-24.13	150	-24.13	935680	21	417	119
miR166e-3p	LOC_Os01g65620.1	163	-21.94	163	-21.94	935715	21	692	626
miR166e-3p	LOC_Os01g66090.1	169	-26.03	169	-26.03	935774	21	6006	5032
miR166e-3p	LOC_Os01g66990.1	150	-20.39	150	-20.39	935876	21	1113	673
miR166e-3p	LOC_Os01g67970.1	154	-21.17	154	-21.17	935997	21	4251	822
miR166e-3p	LOC_Os01g68350.1	157	-22.03	157	-22.03	936044	21	2940	2819
miR166e-3p	LOC_Os01g68598.1	162	-24.03	162	-24.03	936074	21	858	291
miR166e-3p	LOC_Os01g68598.2	162	-24.03	162	-24.03	936075	21	853	286

miR166e-3p	LOC_Os01g69990.1	157	-23.05	157	-23.05	936192	21	4943	1789
miR166e-3p	LOC_Os01g70740.1	150	-29.35	150	-29.35	936289	21	1692	1392
miR166e-3p	LOC_Os01g72320.1	161	-24.61	161	-24.61	936471	21	3583	505
miR166e-3p	LOC_Os01g73060.1	157	-22.68	157	-22.68	936559	21	1350	867
miR166e-3p	LOC_Os01g73662.1	155	-24.91	155	-24.91	936627	21	1306	855
miR166e-3p	LOC_Os02g01190.1	150	-26.25	150	-26.25	936784	21	1246	268
miR166e-3p	LOC_Os02g01190.2	150	-26.25	150	-26.25	936785	21	1482	497
miR166e-3p	LOC_Os02g01260.1	154	-22.34	154	-22.34	936794	21	3963	1747
miR166e-3p	LOC_Os02g02090.1	150	-20.04	150	-20.04	936893	21	420	264
miR166e-3p	LOC_Os02g03040.1	146	-20.64	146	-20.64	937012	21	1349	1282
miR166e-3p	LOC_Os02g03040.2	146	-20.64	146	-20.64	937013	21	1422	1355
miR166e-3p	LOC_Os02g03040.3	146	-20.64	146	-20.64	937014	21	1859	1792
miR166e-3p	LOC_Os02g03040.4	146	-20.64	146	-20.64	937015	21	1705	1638
miR166e-3p	LOC_Os02g03800.1	163	-23.44	163	-23.44	937115	21	599	457
miR166e-3p	LOC_Os02g05280.1	157	-20.25	157	-20.25	937314	21	315	119
miR166e-3p	LOC_Os02g05660.1	166	-25.54	166	-25.54	937367	21	2578	2239
miR166e-3p	LOC_Os02g05810.1	156	-22.01	156	-22.01	937386	21	3705	856
miR166e-3p	LOC_Os02g05810.2	156	-22.01	156	-22.01	937387	21	3607	850
miR166e-3p	LOC_Os02g06580.1	155	-20.55	155	-20.55	937471	21	3152	1175
miR166e-3p	LOC_Os02g06750.1	157	-22.32	157	-22.32	937494	21	1835	814
miR166e-3p	LOC_Os02g07260.1	163	-23.28	163	-23.28	937569	21	1731	1507
miR166e-3p	LOC_Os02g07260.2	163	-23.28	163	-23.28	937570	21	1841	1617
miR166e-3p	LOC_Os02g07260.3	163	-23.28	163	-23.28	937571	21	1813	1589
miR166e-3p	LOC_Os02g07840.1	143	-21.44	143	-21.44	937644	21	1396	440
miR166e-3p	LOC_Os02g07840.2	143	-21.44	143	-21.44	937645	21	1353	397
miR166e-3p	LOC_Os02g10120.1	153	-23.68	153	-23.68	937863	21	3224	2081
miR166e-3p	LOC_Os02g10140.1	153	-20.78	153	-20.78	937865	21	912	597
miR166e-3p	LOC_Os02g10230.1	145	-25.62	145	-25.62	937876	21	1342	1026
miR166e-3p	LOC_Os02g10260.1	162	-25.11	162	-25.11	937879	21	2519	1859
miR166e-3p	LOC_Os02g10260.2	162	-25.11	162	-25.11	937880	21	1609	949
miR166e-3p	LOC_Os02g10360.1	155	-22.93	155	-22.93	937892	21	1272	868
miR166e-3p	LOC_Os02g10614.1	149	-21.24	149	-21.24	937926	21	501	209

miR166e-3p	LOC_Os02g13000.1	153	-20.49	153	-20.49	938175	21	1299	344
miR166e-3p	LOC_Os02g14390.1	152	-20.86	152	-20.86	938330	21	2184	1303
miR166e-3p	LOC_Os02g14710.1	146	-20.14	146	-20.14	938362	21	489	14
miR166e-3p	LOC_Os02g14929.1	151	-22.72	151	-22.72	938396	21	2014	1989
miR166e-3p	LOC_Os02g16220.1	161	-20.18	161	-20.18	938545	21	2967	2909
miR166e-3p	LOC_Os02g16490.1	146	-22.15	146	-22.15	938573	21	4317	3654
miR166e-3p	LOC_Os02g16760.1	150	-24.16	150	-24.16	938604	21	1173	285
miR166e-3p	LOC_Os02g17720.1	151	-21.9	151	-21.9	938706	21	4497	2654
miR166e-3p	LOC_Os02g17780.1	149	-20.66	149	-20.66	938712	21	2943	956
miR166e-3p	LOC_Os02g17880.1	158	-21.22	158	-21.22	938721	21	891	410
miR166e-3p	LOC_Os02g18064.1	327	-43.66	167	-23.37	938748	21	4783	1030 4568
miR166e-3p	LOC_Os02g18740.1	157	-21.81	157	-21.81	938821	21	2364	667
miR166e-3p	LOC_Os02g19860.1	154	-20.28	154	-20.28	938939	21	4086	3831
miR166e-3p	LOC_Os02g20770.1	150	-24.97	150	-24.97	939030	21	417	389
miR166e-3p	LOC_Os02g21970.1	151	-22.57	151	-22.57	939165	21	1798	822
miR166e-3p	LOC_Os02g23824.1	152	-20.54	152	-20.54	939270	21	1287	646
miR166e-3p	LOC_Os02g24570.1	164	-20.13	164	-20.13	939355	21	1962	435
miR166e-3p	LOC_Os02g25080.1	155	-21.55	155	-21.55	939414	21	1304	565
miR166e-3p	LOC_Os02g25790.1	154	-22.34	154	-22.34	939487	21	4299	1693
miR166e-3p	LOC_Os02g25810.1	152	-23.51	152	-23.51	939489	21	5124	3487
miR166e-3p	LOC_Os02g25880.1	157	-26.02	157	-26.02	939496	21	3606	435
miR166e-3p	LOC_Os02g26780.1	153	-22.1	153	-22.1	939605	21	1680	758
miR166e-3p	LOC_Os02g27150.1	142	-22.39	142	-22.39	939649	21	552	174
miR166e-3p	LOC_Os02g27240.1	150	-22.21	150	-22.21	939658	21	2475	349
miR166e-3p	LOC_Os02g27790.1	159	-22.22	159	-22.22	939723	21	3621	1626
miR166e-3p	LOC_Os02g29080.1	157	-20.74	157	-20.74	939849	21	543	425
miR166e-3p	LOC_Os02g29220.1	166	-28.12	166	-28.12	939867	21	798	350
miR166e-3p	LOC_Os02g29410.1	161	-21.65	161	-21.65	939885	21	2799	1274
miR166e-3p	LOC_Os02g30440.1	153	-21.14	153	-21.14	939999	21	324	273
miR166e-3p	LOC_Os02g30520.1	154	-20.41	154	-20.41	940007	21	2493	2182
miR166e-3p	LOC_Os02g32370.1	157	-26.89	157	-26.89	940164	21	1786	995
miR166e-3p	LOC_Os02g33770.1	155	-20.22	155	-20.22	940326	21	2001	389

miR166e-3p	LOC_Os02g34200.1	157	-20.4	157	-20.4	940363	21	2877	443
miR166e-3p	LOC_Os02g35760.1	313	-48.14	160	-27.74	940537	21	1896	1518 897
miR166e-3p	LOC_Os02g35760.2	313	-48.14	160	-27.74	940538	21	1844	1518 897
miR166e-3p	LOC_Os02g36770.1	162	-24.62	162	-24.62	940662	21	1941	1035
miR166e-3p	LOC_Os02g37020.1	162	-24.88	162	-24.88	940695	21	2334	1941
miR166e-3p	LOC_Os02g37050.1	150	-20.31	150	-20.31	940697	21	3179	2563
miR166e-3p	LOC_Os02g38570.1	154	-23.19	154	-23.19	940883	21	3141	2593
miR166e-3p	LOC_Os02g38670.1	154	-23.19	154	-23.19	940894	21	2985	2473
miR166e-3p	LOC_Os02g39230.1	150	-24.12	150	-24.12	940967	21	1299	810
miR166e-3p	LOC_Os02g39280.1	148	-21.07	148	-21.07	940971	21	1578	647
miR166e-3p	LOC_Os02g39360.1	155	-21.42	155	-21.42	940981	21	1240	718
miR166e-3p	LOC_Os02g40230.1	154	-22.34	154	-22.34	941106	21	3300	1678
miR166e-3p	LOC_Os02g41702.1	148	-20.17	148	-20.17	941222	21	1848	1507
miR166e-3p	LOC_Os02g41740.1	160	-23.51	160	-23.51	941226	21	4383	1541
miR166e-3p	LOC_Os02g42440.1	153	-22.62	153	-22.62	941321	21	1258	1168
miR166e-3p	LOC_Os02g42670.1	154	-20.31	154	-20.31	941348	21	3779	231
miR166e-3p	LOC_Os02g42690.1	154	-20.97	154	-20.97	941349	21	1951	1757
miR166e-3p	LOC_Os02g44270.1	158	-21.57	158	-21.57	941555	21	2998	1490
miR166e-3p	LOC_Os02g44270.2	158	-21.57	158	-21.57	941556	21	2231	1464
miR166e-3p	LOC_Os02g44590.1	157	-21.05	157	-21.05	941597	21	3617	573
miR166e-3p	LOC_Os02g45790.1	153	-20.05	153	-20.05	941747	21	4896	2218
miR166e-3p	LOC_Os02g46010.1	312	-43.08	157	-22.03	941770	21	1908	1787 1324
miR166e-3p	LOC_Os02g46060.1	154	-22.34	154	-22.34	941775	21	3702	2062
miR166e-3p	LOC_Os02g46120.1	152	-20.59	152	-20.59	941782	21	3747	3560
miR166e-3p	LOC_Os02g46120.2	152	-20.59	152	-20.59	941783	21	3606	3419
miR166e-3p	LOC_Os02g47410.1	166	-23.52	166	-23.52	941943	21	3747	956
miR166e-3p	LOC_Os02g47410.2	166	-23.52	166	-23.52	941944	21	3744	956
miR166e-3p	LOC_Os02g47410.3	166	-23.52	166	-23.52	941945	21	3741	956
miR166e-3p	LOC_Os02g47940.1	151	-20.83	151	-20.83	942017	21	1945	1100
miR166e-3p	LOC_Os02g48390.1	150	-22.1	150	-22.1	942076	21	1825	1248
miR166e-3p	LOC_Os02g48710.1	156	-20.54	156	-20.54	942116	21	544	303
miR166e-3p	LOC_Os02g49326.1	159	-21.85	159	-21.85	942192	21	1931	777

miR166e-3p	LOC_Os02g49332.1	158	-24.7	158	-24.7	942193	21	2673	273
miR166e-3p	LOC_Os02g49332.2	158	-24.7	158	-24.7	942194	21	4273	273
miR166e-3p	LOC_Os02g49332.3	158	-24.7	158	-24.7	942195	21	4042	265
miR166e-3p	LOC_Os02g49332.4	158	-24.7	158	-24.7	942196	21	2778	273
miR166e-3p	LOC_Os02g49920.1	148	-24.08	148	-24.08	942273	21	2060	1798
miR166e-3p	LOC_Os02g50490.1	154	-26.7	154	-26.7	942337	21	1981	1533
miR166e-3p	LOC_Os02g50620.1	154	-21.57	154	-21.57	942351	21	1658	927
miR166e-3p	LOC_Os02g51200.1	309	-42.91	158	-22.83	942424	21	5130	2784 2820
miR166e-3p	LOC_Os02g51730.1	155	-21.59	155	-21.59	942490	21	2138	1947
miR166e-3p	LOC_Os02g52360.1	165	-24.9	165	-24.9	942575	21	1746	672
miR166e-3p	LOC_Os02g52744.1	149	-22.77	149	-22.77	942618	21	1127	1084
miR166e-3p	LOC_Os02g52744.2	149	-22.77	149	-22.77	942619	21	1123	1080
miR166e-3p	LOC_Os02g53680.1	152	-23.57	152	-23.57	942740	21	2591	2489
miR166e-3p	LOC_Os02g54150.1	164	-21.58	164	-21.58	942808	21	2665	2555
miR166e-3p	LOC_Os02g54500.1	152	-23.31	152	-23.31	942846	21	3894	2845
miR166e-3p	LOC_Os02g54780.1	142	-21.55	142	-21.55	942875	21	2267	1305
miR166e-3p	LOC_Os02g54810.1	157	-25.29	157	-25.29	942878	21	453	407
miR166e-3p	LOC_Os02g54980.1	156	-23.88	156	-23.88	942898	21	2139	965
miR166e-3p	LOC_Os02g56110.1	158	-20.01	158	-20.01	943042	21	2971	2557
miR166e-3p	LOC_Os02g56110.2	158	-20.01	158	-20.01	943043	21	3211	2797
miR166e-3p	LOC_Os02g56219.1	153	-20.31	153	-20.31	943058	21	1288	212
miR166e-3p	LOC_Os02g56334.1	161	-32.09	161	-32.09	943068	21	2100	999
miR166e-3p	LOC_Os02g56350.1	153	-21.33	153	-21.33	943070	21	315	90
miR166e-3p	LOC_Os02g56354.1	153	-21.33	153	-21.33	943071	21	1878	90
miR166e-3p	LOC_Os02g56820.1	149	-24.22	149	-24.22	943125	21	2065	1389
miR166e-3p	LOC_Os02g57090.1	305	-41.62	154	-21.61	943163	21	3597	1413 1293
miR166e-3p	LOC_Os02g57400.1	150	-20.31	150	-20.31	943210	21	3162	334
miR166e-3p	LOC_Os02g57400.2	150	-20.31	150	-20.31	943211	21	2924	330
miR166e-3p	LOC_Os02g57510.1	155	-20.34	155	-20.34	943233	21	1059	186
miR166e-3p	LOC_Os02g57510.2	155	-20.34	155	-20.34	943234	21	1052	186
miR166e-3p	LOC_Os02g57510.3	155	-20.34	155	-20.34	943235	21	1026	165
miR166e-3p	LOC_Os02g57510.4	155	-20.34	155	-20.34	943236	21	1040	186

miR166e-3p	LOC_Os03g01100.1	165	-21.27	165	-21.27	943425	21	3241	2525
miR166e-3p	LOC_Os03g01200.1	160	-22.19	160	-22.19	943437	21	3239	804
miR166e-3p	LOC_Os03g01442.1	154	-20.92	154	-20.92	943474	21	2647	2430
miR166e-3p	LOC_Os03g01870.1	159	-26.46	159	-26.46	943531	21	1077	306
miR166e-3p	LOC_Os03g01890.1	160	-22.35	160	-22.35	943534	21	3391	1474
miR166e-3p	LOC_Os03g01890.2	160	-22.35	160	-22.35	943535	21	2539	1474
miR166e-3p	LOC_Os03g03210.1	153	-20.05	153	-20.05	943718	21	3162	1177
miR166e-3p	LOC_Os03g03760.1	155	-20.55	155	-20.55	943793	21	1191	744
miR166e-3p	LOC_Os03g06840.1	149	-21.1	149	-21.1	944168	21	765	428
miR166e-3p	LOC_Os03g06920.1	157	-22.23	157	-22.23	944180	21	3594	3449
miR166e-3p	LOC_Os03g07050.1	156	-20.08	156	-20.08	944194	21	3654	2880
miR166e-3p	LOC_Os03g07140.1	152	-21.42	152	-21.42	944211	21	3088	3059
miR166e-3p	LOC_Os03g07150.1	152	-21.42	152	-21.42	944212	21	2170	1093
miR166e-3p	LOC_Os03g07150.2	152	-21.42	152	-21.42	944213	21	1974	1093
miR166e-3p	LOC_Os03g07310.1	150	-20.31	150	-20.31	944232	21	1881	1802
miR166e-3p	LOC_Os03g08290.1	157	-23.67	157	-23.67	944358	21	1496	643
miR166e-3p	LOC_Os03g08380.1	150	-22.46	150	-22.46	944369	21	4449	2991
miR166e-3p	LOC_Os03g08550.1	157	-23.56	157	-23.56	944393	21	2891	2373
miR166e-3p	LOC_Os03g08550.2	157	-23.56	157	-23.56	944394	21	2984	2466
miR166e-3p	LOC_Os03g08560.1	157	-23.56	157	-23.56	944395	21	3643	29
miR166e-3p	LOC_Os03g09020.1	149	-20.49	149	-20.49	944448	21	1083	738
miR166e-3p	LOC_Os03g09120.1	155	-22.55	155	-22.55	944458	21	1388	610
miR166e-3p	LOC_Os03g10280.1	159	-21.02	159	-21.02	944552	21	3141	671
miR166e-3p	LOC_Os03g12840.1	149	-21.48	149	-21.48	944875	21	2070	259
miR166e-3p	LOC_Os03g12840.2	149	-21.48	149	-21.48	944876	21	1780	259
miR166e-3p	LOC_Os03g12840.3	149	-21.48	149	-21.48	944877	21	1877	259
miR166e-3p	LOC_Os03g13720.1	147	-22.35	147	-22.35	944987	21	3002	872
miR166e-3p	LOC_Os03g13910.1	152	-20.03	152	-20.03	945015	21	2826	1716
miR166e-3p	LOC_Os03g14520.1	311	-46.38	161	-25.63	945097	21	1316	906 872
miR166e-3p	LOC_Os03g14720.1	155	-24.31	155	-24.31	945119	21	3496	1949
miR166e-3p	LOC_Os03g14770.1	155	-21.05	155	-21.05	945123	21	2361	1966
miR166e-3p	LOC_Os03g15400.1	147	-25.1	147	-25.1	945197	21	822	535

miR166e-3p	LOC_Os03g15570.1	155	-24.23	155	-24.23	945215	21	2660	2518
miR166e-3p	LOC_Os03g15680.1	148	-21.75	148	-21.75	945228	21	1728	458
miR166e-3p	LOC_Os03g15840.1	157	-20.23	157	-20.23	945252	21	2058	776
miR166e-3p	LOC_Os03g16480.1	153	-20.7	153	-20.7	945355	21	1486	170
miR166e-3p	LOC_Os03g17330.1	162	-27.16	162	-27.16	945463	21	1002	218
miR166e-3p	LOC_Os03g17570.4	154	-24.96	154	-24.96	945491	21	4061	900
miR166e-3p	LOC_Os03g17840.1	162	-21.75	162	-21.75	945520	21	2660	1347
miR166e-3p	LOC_Os03g18720.1	152	-21.94	152	-21.94	945628	21	1304	1149
miR166e-3p	LOC_Os03g18720.2	152	-21.94	152	-21.94	945629	21	1277	1146
miR166e-3p	LOC_Os03g19200.1	158	-23.18	158	-23.18	945680	21	2947	2102
miR166e-3p	LOC_Os03g19452.2	154	-20.76	154	-20.76	945712	21	1321	285
miR166e-3p	LOC_Os03g19490.1	150	-22.02	150	-22.02	945716	21	2626	583
miR166e-3p	LOC_Os03g19590.1	153	-20.2	153	-20.2	945731	21	4286	2223
miR166e-3p	LOC_Os03g20020.1	153	-21.5	153	-21.5	945786	21	2302	312
miR166e-3p	LOC_Os03g20380.1	153	-21.59	153	-21.59	945824	21	1858	1558
miR166e-3p	LOC_Os03g20380.2	153	-21.59	153	-21.59	945825	21	1803	1503
miR166e-3p	LOC_Os03g20380.3	153	-21.59	153	-21.59	945826	21	2163	1863
miR166e-3p	LOC_Os03g20380.4	153	-21.59	153	-21.59	945827	21	2413	2113
miR166e-3p	LOC_Os03g20380.5	153	-21.59	153	-21.59	945828	21	2035	1735
miR166e-3p	LOC_Os03g20380.6	153	-21.59	153	-21.59	945829	21	2269	1969
miR166e-3p	LOC_Os03g20380.7	153	-21.59	153	-21.59	945830	21	2592	1632
miR166e-3p	LOC_Os03g20380.8	153	-21.59	153	-21.59	945831	21	2457	2157
miR166e-3p	LOC_Os03g21120.1	308	-40.23	162	-20.2	945917	21	2219	676 2056
miR166e-3p	LOC_Os03g22190.1	150	-27.83	150	-27.83	946050	21	4940	3944
miR166e-3p	LOC_Os03g23030.1	158	-25.3	158	-25.3	946161	21	8234	5548
miR166e-3p	LOC_Os03g23080.1	147	-20.09	147	-20.09	946166	21	1839	920
miR166e-3p	LOC_Os03g24750.1	145	-20.26	145	-20.26	946275	21	204	119
miR166e-3p	LOC_Os03g25720.1	160	-23.07	160	-23.07	946389	21	1848	1771
miR166e-3p	LOC_Os03g26470.1	152	-21.62	152	-21.62	946458	21	249	161
miR166e-3p	LOC_Os03g29650.1	153	-20.05	153	-20.05	946751	21	4980	2296
miR166e-3p	LOC_Os03g30110.1	162	-21.01	162	-21.01	946796	21	5184	5113
miR166e-3p	LOC_Os03g30570.1	148	-21.05	148	-21.05	946842	21	1686	528

miR166e-3p	LOC_Os03g30570.2	148	-21.05	148	-21.05	946843	21	1596	346
miR166e-3p	LOC_Os03g30810.1	312	-43.9	163	-23.66	946868	21	3411	1740 2909
miR166e-3p	LOC_Os03g31060.1	156	-21.88	156	-21.88	946894	21	1857	155
miR166e-3p	LOC_Os03g32870.1	147	-20.57	147	-20.57	947072	21	1110	409
miR166e-3p	LOC_Os03g32880.1	150	-20.47	150	-20.47	947073	21	474	254
miR166e-3p	LOC_Os03g33319.1	161	-21.72	161	-21.72	947117	21	1275	1113
miR166e-3p	LOC_Os03g33490.1	158	-25.9	158	-25.9	947131	21	357	158
miR166e-3p	LOC_Os03g33710.1	150	-22.93	150	-22.93	947153	21	1227	149
miR166e-3p	LOC_Os03g33900.1	147	-20.57	147	-20.57	947172	21	1428	745
miR166e-3p	LOC_Os03g35340.1	168	-26.42	168	-26.42	947221	21	6746	575
miR166e-3p	LOC_Os03g35390.1	152	-23.51	152	-23.51	947226	21	5727	4153
miR166e-3p	LOC_Os03g35580.1	155	-21.06	155	-21.06	947241	21	1494	3
miR166e-3p	LOC_Os03g36570.1	150	-20.47	150	-20.47	947325	21	6849	542
miR166e-3p	LOC_Os03g36780.1	157	-20.49	157	-20.49	947346	21	2753	1965
miR166e-3p	LOC_Os03g37250.1	144	-22.22	144	-22.22	947387	21	5322	5150
miR166e-3p	LOC_Os03g38284.1	155	-21.05	155	-21.05	947488	21	4548	4246
miR166e-3p	LOC_Os03g39460.1	157	-20.8	157	-20.8	947608	21	363	270
miR166e-3p	LOC_Os03g41000.1	154	-22.34	154	-22.34	947743	21	4194	1747
miR166e-3p	LOC_Os03g41570.1	148	-21.55	148	-21.55	947800	21	2946	123
miR166e-3p	LOC_Os03g41790.1	151	-20.09	151	-20.09	947818	21	1689	958
miR166e-3p	LOC_Os03g43360.2	150	-20.4	150	-20.4	947975	21	2048	1068
miR166e-3p	LOC_Os03g46220.1	153	-20.49	153	-20.49	948284	21	5673	4718
miR166e-3p	LOC_Os03g47010.1	158	-20.42	158	-20.42	948367	21	2584	698
miR166e-3p	LOC_Os03g47550.1	311	-41.38	156	-20.86	948420	21	1357	1109 984
miR166e-3p	LOC_Os03g47550.2	156	-20.86	156	-20.86	948421	21	1254	1004
miR166e-3p	LOC_Os03g47740.2	149	-21.18	149	-21.18	948448	21	2989	372
miR166e-3p	LOC_Os03g47740.3	149	-21.18	149	-21.18	948449	21	2652	372
miR166e-3p	LOC_Os03g47960.1	155	-20.93	155	-20.93	948476	21	1558	1533
miR166e-3p	LOC_Os03g49770.1	306	-47	156	-25.58	948706	21	2862	1268 433
miR166e-3p	LOC_Os03g49770.2	156	-21.42	156	-21.42	948707	21	2369	775
miR166e-3p	LOC_Os03g50364.1	157	-24.45	157	-24.45	948771	21	1371	966
miR166e-3p	LOC_Os03g52239.1	152	-21.75	152	-21.75	948978	21	3575	1130

miR166e-3p	LOC_Os03g52420.1	150	-20.19	150	-20.19	949001	21	743	614
miR166e-3p	LOC_Os03g52540.1	153	-20.05	153	-20.05	949013	21	2859	178
miR166e-3p	LOC_Os03g52880.1	150	-21.42	150	-21.42	949056	21	2781	752
miR166e-3p	LOC_Os03g52900.1	159	-23.98	159	-23.98	949058	21	2772	461
miR166e-3p	LOC_Os03g54804.1	154	-22.34	154	-22.34	949235	21	4383	2167
miR166e-3p	LOC_Os03g55100.1	153	-20.84	153	-20.84	949271	21	2446	660
miR166e-3p	LOC_Os03g55230.1	140	-21.03	140	-21.03	949288	21	1625	745
miR166e-3p	LOC_Os03g55240.1	140	-21.03	140	-21.03	949289	21	2545	1362
miR166e-3p	LOC_Os03g55260.1	140	-21.03	140	-21.03	949291	21	1941	723
miR166e-3p	LOC_Os03g56690.1	158	-20.3	158	-20.3	949469	21	432	242
miR166e-3p	LOC_Os03g56990.1	147	-20.05	147	-20.05	949520	21	3534	1310
miR166e-3p	LOC_Os03g57370.1	153	-20.05	153	-20.05	949572	21	4659	2386
miR166e-3p	LOC_Os03g57430.1	150	-20.39	150	-20.39	949579	21	4080	3695
miR166e-3p	LOC_Os03g59090.1	157	-22.26	157	-22.26	949772	21	855	326
miR166e-3p	LOC_Os03g59090.2	157	-22.26	157	-22.26	949773	21	877	387
miR166e-3p	LOC_Os03g60710.1	159	-24.74	159	-24.74	949947	21	2349	307
miR166e-3p	LOC_Os03g61200.1	150	-22.79	150	-22.79	950010	21	1509	302
miR166e-3p	LOC_Os03g61200.2	150	-22.79	150	-22.79	950011	21	1597	302
miR166e-3p	LOC_Os03g61410.1	149	-20.91	149	-20.91	950037	21	1017	731
miR166e-3p	LOC_Os03g61630.1	157	-20.04	157	-20.04	950061	21	1424	406
miR166e-3p	LOC_Os03g61630.2	157	-20.04	157	-20.04	950062	21	1411	393
miR166e-3p	LOC_Os03g62090.1	164	-21.55	164	-21.55	950127	21	4365	1285
miR166e-3p	LOC_Os03g63690.1	151	-22.45	151	-22.45	950338	21	1382	1358
miR166e-3p	LOC_Os03g63720.1	161	-28.8	161	-28.8	950342	21	1205	1091
miR166e-3p	LOC_Os03g64170.1	158	-20.35	158	-20.35	950395	21	1697	1184
miR166e-3p	LOC_Os04g02730.1	152	-22.59	152	-22.59	950628	21	2506	2449
miR166e-3p	LOC_Os04g03300.1	151	-23.57	151	-23.57	950686	21	3981	2356
miR166e-3p	LOC_Os04g03450.1	152	-21.21	152	-21.21	950701	21	4833	1308
miR166e-3p	LOC_Os04g04500.1	156	-21.47	156	-21.47	950801	21	843	783
miR166e-3p	LOC_Os04g04540.1	145	-21.43	145	-21.43	950805	21	783	283
miR166e-3p	LOC_Os04g05540.1	150	-20.82	150	-20.82	950897	21	543	173
miR166e-3p	LOC_Os04g05920.1	155	-23.02	155	-23.02	950932	21	1917	474

miR166e-3p	LOC_Os04g06320.1	154	-26.58	154	-26.58	950968	21	477	65
miR166e-3p	LOC_Os04g06700.1	147	-20.19	147	-20.19	951006	21	2310	2129
miR166e-3p	LOC_Os04g07110.1	158	-20.72	158	-20.72	951042	21	2340	942
miR166e-3p	LOC_Os04g07780.1	158	-21.62	158	-21.62	951109	21	567	117
miR166e-3p	LOC_Os04g09360.1	148	-22.68	148	-22.68	951237	21	3582	3293
miR166e-3p	LOC_Os04g10780.1	152	-20.37	152	-20.37	951376	21	675	102
miR166e-3p	LOC_Os04g11090.1	152	-23.51	152	-23.51	951402	21	3876	2641
miR166e-3p	LOC_Os04g11620.1	152	-23.51	152	-23.51	951455	21	5544	3670
miR166e-3p	LOC_Os04g11770.1	154	-22.34	154	-22.34	951469	21	3963	1747
miR166e-3p	LOC_Os04g12410.1	150	-20.47	150	-20.47	951535	21	1476	542
miR166e-3p	LOC_Os04g13120.1	306	-40.58	154	-20.41	951594	21	1125	1054 592
miR166e-3p	LOC_Os04g16850.1	153	-20.01	153	-20.01	951945	21	1677	155
miR166e-3p	LOC_Os04g17000.1	150	-20.3	150	-20.3	951958	21	474	408
miR166e-3p	LOC_Os04g17300.1	150	-20.47	150	-20.47	951988	21	1182	542
miR166e-3p	LOC_Os04g18080.1	154	-22.34	154	-22.34	952069	21	3963	1747
miR166e-3p	LOC_Os04g18480.1	152	-23.02	152	-23.02	952103	21	3927	3503
miR166e-3p	LOC_Os04g19060.1	149	-20.04	149	-20.04	952160	21	1281	1148
miR166e-3p	LOC_Os04g19114.1	147	-21.48	147	-21.48	952165	21	3693	18
miR166e-3p	LOC_Os04g19530.1	159	-23.09	159	-23.09	952205	21	795	226
miR166e-3p	LOC_Os04g19900.1	153	-20.05	153	-20.05	952242	21	3771	1495
miR166e-3p	LOC_Os04g19970.1	153	-26.08	153	-26.08	952249	21	543	160
miR166e-3p	LOC_Os04g20950.1	152	-20.03	152	-20.03	952346	21	2697	1668
miR166e-3p	LOC_Os04g21580.1	151	-21.1	151	-21.1	952404	21	5198	4630
miR166e-3p	LOC_Os04g22550.1	155	-20.69	155	-20.69	952500	21	732	308
miR166e-3p	LOC_Os04g23460.1	162	-22.35	162	-22.35	952584	21	336	240
miR166e-3p	LOC_Os04g25360.1	145	-20.89	145	-20.89	952768	21	1995	101
miR166e-3p	LOC_Os04g25360.2	145	-20.89	145	-20.89	952769	21	1518	98
miR166e-3p	LOC_Os04g25820.1	159	-20.04	159	-20.04	952821	21	1077	995
miR166e-3p	LOC_Os04g26010.1	159	-27.57	159	-27.57	952841	21	2634	2092
miR166e-3p	LOC_Os04g26550.1	166	-25.71	166	-25.71	952893	21	1053	751
miR166e-3p	LOC_Os04g26640.1	155	-23.77	155	-23.77	952902	21	441	79
miR166e-3p	LOC_Os04g27020.1	164	-23.4	164	-23.4	952935	21	1536	212

miR166e-3p	LOC_Os04g27670.1	156	-21.27	156	-21.27	952999	21	2147	534
miR166e-3p	LOC_Os04g27720.1	311	-42.89	161	-21.56	953004	21	1518	151 477
miR166e-3p	LOC_Os04g27990.1	156	-23.26	156	-23.26	953026	21	384	299
miR166e-3p	LOC_Os04g28450.1	147	-24	147	-24	953072	21	450	201
miR166e-3p	LOC_Os04g28560.1	152	-23.85	152	-23.85	953084	21	4824	3307
miR166e-3p	LOC_Os04g29650.1	153	-20.05	153	-20.05	953182	21	3198	1303
miR166e-3p	LOC_Os04g31220.1	154	-22.34	154	-22.34	953349	21	3963	1747
miR166e-3p	LOC_Os04g32540.1	151	-25.53	151	-25.53	953475	21	2085	1282
miR166e-3p	LOC_Os04g32540.2	151	-25.53	151	-25.53	953476	21	1811	1008
miR166e-3p	LOC_Os04g34070.1	154	-22.34	154	-22.34	953660	21	3963	1747
miR166e-3p	LOC_Os04g35030.1	177	-29.45	177	-29.45	953758	21	2554	1261
miR166e-3p	LOC_Os04g35080.1	158	-22.02	158	-22.02	953762	21	2625	1327
miR166e-3p	LOC_Os04g35250.1	156	-20.5	156	-20.5	953780	21	1515	951
miR166e-3p	LOC_Os04g35870.1	151	-21.9	151	-21.9	953857	21	4806	2801
miR166e-3p	LOC_Os04g37430.1	146	-20.16	146	-20.16	953924	21	3022	371
miR166e-3p	LOC_Os04g39910.1	155	-20.24	155	-20.24	954218	21	2385	177
miR166e-3p	LOC_Os04g39990.1	153	-20.49	153	-20.49	954225	21	5982	5027
miR166e-3p	LOC_Os04g40920.1	160	-22.66	160	-22.66	954345	21	1643	66
miR166e-3p	LOC_Os04g40920.2	160	-22.66	160	-22.66	954346	21	1664	66
miR166e-3p	LOC_Os04g41810.1	152	-20.46	152	-20.46	954451	21	1876	1725
miR166e-3p	LOC_Os04g41850.1	157	-22.58	157	-22.58	954455	21	4159	977
miR166e-3p	LOC_Os04g42444.1	150	-20.19	150	-20.19	954524	21	708	108
miR166e-3p	LOC_Os04g43760.1	155	-22.92	155	-22.92	954693	21	2553	305
miR166e-3p	LOC_Os04g43760.2	155	-22.92	155	-22.92	954694	21	1452	305
miR166e-3p	LOC_Os04g45140.1	157	-23.9	157	-23.9	954864	21	1979	1571
miR166e-3p	LOC_Os04g45420.1	156	-21.07	156	-21.07	954896	21	7926	5968
miR166e-3p	LOC_Os04g47600.1	154	-22.34	154	-22.34	955160	21	3963	1747
miR166e-3p	LOC_Os04g48290.1	149	-23.81	149	-23.81	955246	21	3940	1690
miR166e-3p	LOC_Os04g48910.1	153	-20.18	153	-20.18	955315	21	2367	82
miR166e-3p	LOC_Os04g49640.1	154	-22.34	154	-22.34	955398	21	3777	1561
miR166e-3p	LOC_Os04g49910.1	151	-21.9	151	-21.9	955428	21	4056	2051
miR166e-3p	LOC_Os04g50192.1	159	-21.02	159	-21.02	955464	21	3621	39

miR166e-3p	LOC_Os04g50930.1	153	-28.79	153	-28.79	955500	21	2047	762
miR166e-3p	LOC_Os04g51000.1	153	-20.76	153	-20.76	955508	21	1493	580
miR166e-3p	LOC_Os04g51120.1	154	-21.34	154	-21.34	955523	21	2391	2236
miR166e-3p	LOC_Os04g51120.3	154	-21.34	154	-21.34	955524	21	2623	2468
miR166e-3p	LOC_Os04g51910.1	158	-25.03	158	-25.03	955637	21	3054	631
miR166e-3p	LOC_Os04g53540.1	157	-21.53	157	-21.53	955841	21	3823	1343
miR166e-3p	LOC_Os04g53540.2	157	-21.53	157	-21.53	955842	21	3885	1343
miR166e-3p	LOC_Os04g53540.3	157	-21.53	157	-21.53	955843	21	3800	1349
miR166e-3p	LOC_Os04g53540.4	157	-21.53	157	-21.53	955844	21	3365	1349
miR166e-3p	LOC_Os04g53720.1	150	-20.17	150	-20.17	955868	21	3864	3802
miR166e-3p	LOC_Os04g54360.1	159	-20.23	159	-20.23	955946	21	2420	1171
miR166e-3p	LOC_Os04g54810.1	150	-23.53	150	-23.53	955999	21	2995	1653
miR166e-3p	LOC_Os04g54930.1	154	-23.17	154	-23.17	956013	21	2544	2443
miR166e-3p	LOC_Os04g56440.1	157	-20.02	157	-20.02	956189	21	2566	1953
miR166e-3p	LOC_Os04g56440.2	157	-20.02	157	-20.02	956190	21	2643	2030
miR166e-3p	LOC_Os04g56450.1	157	-20.02	157	-20.02	956191	21	2162	591
miR166e-3p	LOC_Os04g56450.2	157	-20.02	157	-20.02	956192	21	2015	591
miR166e-3p	LOC_Os04g56990.2	163	-24.19	163	-24.19	956259	21	2765	957
miR166e-3p	LOC_Os04g57210.1	159	-24.8	159	-24.8	956292	21	1970	547
miR166e-3p	LOC_Os04g57210.2	159	-24.8	159	-24.8	956293	21	2137	547
miR166e-3p	LOC_Os04g57210.3	159	-24.8	159	-24.8	956294	21	2393	547
miR166e-3p	LOC_Os04g57610.1	149	-25.08	149	-25.08	956351	21	3361	3250
miR166e-3p	LOC_Os04g57610.3	149	-25.08	149	-25.08	956352	21	3349	3238
miR166e-3p	LOC_Os04g57750.1	151	-25.19	151	-25.19	956372	21	807	762
miR166e-3p	LOC_Os04g58450.1	312	-43.08	157	-22.03	956458	21	5187	5066 4603
miR166e-3p	LOC_Os04g58820.1	157	-25.63	157	-25.63	956511	21	1293	251
miR166e-3p	LOC_Os04g59600.1	158	-20.33	158	-20.33	956603	21	2113	811
miR166e-3p	LOC_Os04g59600.2	158	-20.33	158	-20.33	956604	21	2293	811
miR166e-3p	LOC_Os04g59624.2	149	-22.42	149	-22.42	956610	21	3412	3344
miR166e-3p	LOC_Os04g59624.3	149	-22.42	149	-22.42	956611	21	3481	3413
miR166e-3p	LOC_Os05g01690.1	172	-26.04	172	-26.04	956726	21	4183	4136
miR166e-3p	LOC_Os05g01900.1	160	-21.11	160	-21.11	956750	21	855	12

miR166e-3p	LOC_Os05g02040.1	158	-20.7	158	-20.7	956771	21	3409	1603
miR166e-3p	LOC_Os05g03060.1	163	-23.09	163	-23.09	956895	21	2258	1885
miR166e-3p	LOC_Os05g03070.1	163	-23.09	163	-23.09	956896	21	2074	241
miR166e-3p	LOC_Os05g03280.1	159	-27.57	159	-27.57	956921	21	2607	2065
miR166e-3p	LOC_Os05g03350.1	146	-20.95	146	-20.95	956928	21	1239	1077
miR166e-3p	LOC_Os05g03520.1	173	-30.99	173	-30.99	956951	21	1245	814
miR166e-3p	LOC_Os05g04160.1	156	-21.63	156	-21.63	957020	21	1914	1827
miR166e-3p	LOC_Os05g04584.1	159	-25.12	159	-25.12	957068	21	1935	484
miR166e-3p	LOC_Os05g05470.1	156	-22.14	156	-22.14	957169	21	2092	478
miR166e-3p	LOC_Os05g05630.1	154	-22.34	154	-22.34	957192	21	4230	1747
miR166e-3p	LOC_Os05g08460.1	152	-22.31	152	-22.31	957524	21	1379	406
miR166e-3p	LOC_Os05g08620.1	156	-23.86	156	-23.86	957549	21	657	400
miR166e-3p	LOC_Os05g08880.1	150	-20.47	150	-20.47	957577	21	1332	150
miR166e-3p	LOC_Os05g08890.1	149	-20.45	149	-20.45	957578	21	2433	1725
miR166e-3p	LOC_Os05g09470.1	150	-21.89	150	-21.89	957646	21	504	438
miR166e-3p	LOC_Os05g11040.1	152	-25.36	152	-25.36	957779	21	3840	2270
miR166e-3p	LOC_Os05g12310.1	152	-23.51	152	-23.51	957918	21	1821	175
miR166e-3p	LOC_Os05g13910.1	150	-23.26	150	-23.26	958035	21	2472	2126
miR166e-3p	LOC_Os05g14060.1	149	-24.54	149	-24.54	958053	21	1290	756
miR166e-3p	LOC_Os05g14650.1	152	-23.51	152	-23.51	958112	21	822	571
miR166e-3p	LOC_Os05g17960.1	153	-20.05	153	-20.05	958453	21	5043	2725
miR166e-3p	LOC_Os05g19540.1	154	-22.34	154	-22.34	958623	21	4119	1738
miR166e-3p	LOC_Os05g20360.1	153	-20.05	153	-20.05	958707	21	4542	2341
miR166e-3p	LOC_Os05g20430.1	152	-23.51	152	-23.51	958714	21	4599	2926
miR166e-3p	LOC_Os05g24050.1	152	-20.43	152	-20.43	959004	21	2499	2183
miR166e-3p	LOC_Os05g24180.1	151	-23.38	151	-23.38	959017	21	384	69
miR166e-3p	LOC_Os05g25300.1	156	-21.81	156	-21.81	959138	21	3276	2992
miR166e-3p	LOC_Os05g25440.1	164	-25.65	164	-25.65	959154	21	154	4
miR166e-3p	LOC_Os05g25620.1	154	-23.19	154	-23.19	959173	21	643	459
miR166e-3p	LOC_Os05g26860.1	151	-25.21	151	-25.21	959303	21	630	32
miR166e-3p	LOC_Os05g27490.1	157	-20.25	157	-20.25	959361	21	2430	1904
miR166e-3p	LOC_Os05g28080.1	159	-20.64	159	-20.64	959427	21	1371	217

miR166e-3p	LOC_Os05g29720.1	154	-22.34	154	-22.34	959556	21	4542	2470
miR166e-3p	LOC_Os05g30750.6	153	-22.2	153	-22.2	959681	21	3397	142
miR166e-3p	LOC_Os05g31210.1	151	-21.9	151	-21.9	959736	21	3660	1760
miR166e-3p	LOC_Os05g31570.1	150	-23.31	150	-23.31	959773	21	4693	4312
miR166e-3p	LOC_Os05g31740.1	141	-20.92	141	-20.92	959789	21	1943	1304
miR166e-3p	LOC_Os05g31860.1	151	-21.12	151	-21.12	959803	21	3171	294
miR166e-3p	LOC_Os05g32200.1	156	-20.34	156	-20.34	959838	21	846	588
miR166e-3p	LOC_Os05g32290.1	154	-22.34	154	-22.34	959848	21	3252	709
miR166e-3p	LOC_Os05g33490.1	155	-21.05	155	-21.05	959983	21	5937	5164
miR166e-3p	LOC_Os05g36100.1	152	-22.79	152	-22.79	960288	21	1232	1174
miR166e-3p	LOC_Os05g37070.1	154	-22.34	154	-22.34	960350	21	3963	1747
miR166e-3p	LOC_Os05g37330.1	153	-20.06	153	-20.06	960388	21	819	699
miR166e-3p	LOC_Os05g37820.1	146	-21.15	146	-21.15	960442	21	2087	534
miR166e-3p	LOC_Os05g37970.1	152	-23.17	152	-23.17	960460	21	2258	1605
miR166e-3p	LOC_Os05g38430.1	157	-20.4	157	-20.4	960528	21	2386	792
miR166e-3p	LOC_Os05g39160.1	161	-22.32	161	-22.32	960621	21	1704	1275
miR166e-3p	LOC_Os05g39160.2	161	-22.32	161	-22.32	960622	21	1663	1275
miR166e-3p	LOC_Os05g39180.1	312	-44.26	159	-22.33	960624	21	1728	1701 1353
miR166e-3p	LOC_Os05g39950.1	153	-26.91	153	-26.91	960709	21	1786	639
miR166e-3p	LOC_Os05g40500.1	151	-20.82	151	-20.82	960783	21	2922	1696
miR166e-3p	LOC_Os05g41180.1	158	-20.3	158	-20.3	960862	21	1233	76
miR166e-3p	LOC_Os05g43190.1	142	-25.53	142	-25.53	961034	21	679	400
miR166e-3p	LOC_Os05g43576.1	160	-21.76	160	-21.76	961079	21	1479	1078
miR166e-3p	LOC_Os05g44710.1	165	-24.66	165	-24.66	961221	21	1398	1218
miR166e-3p	LOC_Os05g45980.1	165	-21.05	165	-21.05	961375	21	1224	1052
miR166e-3p	LOC_Os05g46770.1	168	-28.88	168	-28.88	961476	21	3235	2343
miR166e-3p	LOC_Os05g47550.1	154	-23.61	154	-23.61	961517	21	2262	563
miR166e-3p	LOC_Os05g47790.1	154	-20.9	154	-20.9	961539	21	3803	1439
miR166e-3p	LOC_Os05g47870.1	159	-23.06	159	-23.06	961547	21	3077	845
miR166e-3p	LOC_Os05g47870.2	159	-23.06	159	-23.06	961548	21	3074	845
miR166e-3p	LOC_Os05g47980.1	164	-23.9	164	-23.9	961561	21	2140	1468
miR166e-3p	LOC_Os05g50020.1	157	-24.92	157	-24.92	961838	21	1206	17

miR166e-3p	LOC_Os05g50950.1	156	-21.45	156	-21.45	961950	21	3320	3013
miR166e-3p	LOC_Os05g51010.1	154	-22.34	154	-22.34	961959	21	3909	1693
miR166e-3p	LOC_Os05g51170.1	154	-22.34	154	-22.34	961980	21	3963	1747
miR166e-3p	LOC_Os05g51760.1	166	-20.04	166	-20.04	962066	21	2247	1910
miR166e-3p	LOC_Os06g01430.1	154	-23.19	154	-23.19	962142	21	2748	2200
miR166e-3p	LOC_Os06g03540.1	167	-25.37	167	-25.37	962377	21	2614	1102
miR166e-3p	LOC_Os06g03600.1	159	-22.79	159	-22.79	962387	21	3194	373
miR166e-3p	LOC_Os06g03600.2	159	-22.79	159	-22.79	962388	21	3107	373
miR166e-3p	LOC_Os06g03600.3	159	-22.79	159	-22.79	962389	21	3060	373
miR166e-3p	LOC_Os06g04360.1	167	-28.97	167	-28.97	962481	21	2101	2001
miR166e-3p	LOC_Os06g04840.1	146	-21.59	146	-21.59	962540	21	3516	1629
miR166e-3p	LOC_Os06g04840.2	146	-21.59	146	-21.59	962541	21	3505	1629
miR166e-3p	LOC_Os06g05680.1	151	-21.9	151	-21.9	962650	21	4803	2801
miR166e-3p	LOC_Os06g06802.1	151	-21.9	151	-21.9	962798	21	5712	3710
miR166e-3p	LOC_Os06g07630.1	161	-23.36	161	-23.36	962890	21	1573	849
miR166e-3p	LOC_Os06g07923.1	142	-20.47	142	-20.47	962920	21	1573	1024
miR166e-3p	LOC_Os06g08560.1	164	-20.19	164	-20.19	962987	21	4629	475
miR166e-3p	LOC_Os06g08630.1	154	-22.34	154	-22.34	962995	21	3846	1630
miR166e-3p	LOC_Os06g09290.1	147	-24.15	147	-24.15	963079	21	1925	852
miR166e-3p	LOC_Os06g09290.2	147	-24.15	147	-24.15	963080	21	2046	852
miR166e-3p	LOC_Os06g10560.1	149	-27.19	149	-27.19	963227	21	3157	360
miR166e-3p	LOC_Os06g11660.1	152	-20.98	152	-20.98	963349	21	1332	987
miR166e-3p	LOC_Os06g11660.2	152	-20.98	152	-20.98	963350	21	902	720
miR166e-3p	LOC_Os06g12430.1	149	-21.96	149	-21.96	963442	21	495	83
miR166e-3p	LOC_Os06g12710.1	153	-20.18	153	-20.18	963477	21	5355	2788
miR166e-3p	LOC_Os06g13770.1	163	-20.2	163	-20.2	963595	21	831	704
miR166e-3p	LOC_Os06g14110.1	159	-27.57	159	-27.57	963631	21	2751	2209
miR166e-3p	LOC_Os06g15050.1	156	-21.22	156	-21.22	963734	21	1782	1504
miR166e-3p	LOC_Os06g16070.1	150	-21.29	150	-21.29	963836	21	3285	2790
miR166e-3p	LOC_Os06g16170.1	158	-24.13	158	-24.13	963846	21	1485	830
miR166e-3p	LOC_Os06g17110.1	148	-22.87	148	-22.87	963945	21	472	93
miR166e-3p	LOC_Os06g17120.1	148	-22.87	148	-22.87	963946	21	1452	1076

miR166e-3p	LOC_Os06g17830.1	153	-21.54	153	-21.54	964015	21	786	202
miR166e-3p	LOC_Os06g19900.1	160	-25.6	160	-25.6	964173	21	3870	2875
miR166e-3p	LOC_Os06g20710.1	159	-24.92	159	-24.92	964262	21	2613	1425
miR166e-3p	LOC_Os06g20840.1	146	-22.2	146	-22.2	964274	21	1449	1036
miR166e-3p	LOC_Os06g22770.1	153	-20.05	153	-20.05	964487	21	3630	2632
miR166e-3p	LOC_Os06g23114.1	142	-21.21	142	-21.21	964525	21	2486	612
miR166e-3p	LOC_Os06g24100.1	154	-21.26	154	-21.26	964628	21	2442	1055
miR166e-3p	LOC_Os06g24110.1	152	-23.51	152	-23.51	964629	21	4035	3232
miR166e-3p	LOC_Os06g24530.1	161	-20.66	161	-20.66	964668	21	2328	326
miR166e-3p	LOC_Os06g24570.1	145	-22.77	145	-22.77	964672	21	3165	2719
miR166e-3p	LOC_Os06g26100.1	163	-25.54	163	-25.54	964824	21	2772	1857
miR166e-3p	LOC_Os06g28770.1	154	-22.65	154	-22.65	965000	21	1102	908
miR166e-3p	LOC_Os06g29080.1	154	-26.42	154	-26.42	965033	21	3011	2020
miR166e-3p	LOC_Os06g30040.1	161	-20.55	161	-20.55	965133	21	2193	664
miR166e-3p	LOC_Os06g30610.1	171	-24.62	171	-24.62	965190	21	2673	2398
miR166e-3p	LOC_Os06g30620.1	154	-28.96	154	-28.96	965191	21	381	24
miR166e-3p	LOC_Os06g32870.1	153	-20.05	153	-20.05	965414	21	5895	3040
miR166e-3p	LOC_Os06g33290.1	153	-20.05	153	-20.05	965453	21	4380	2752
miR166e-3p	LOC_Os06g34390.1	152	-20.44	152	-20.44	965563	21	1509	1222
miR166e-3p	LOC_Os06g35080.1	153	-21.19	153	-21.19	965633	21	1050	233
miR166e-3p	LOC_Os06g35180.1	170	-28.02	170	-28.02	965644	21	2361	2201
miR166e-3p	LOC_Os06g35310.1	163	-24.79	163	-24.79	965657	21	474	22
miR166e-3p	LOC_Os06g35350.1	149	-20.59	149	-20.59	965660	21	2040	1972
miR166e-3p	LOC_Os06g35550.1	154	-22.34	154	-22.34	965681	21	5926	3425
miR166e-3p	LOC_Os06g35870.1	154	-20.83	154	-20.83	965712	21	1923	1730
miR166e-3p	LOC_Os06g37670.1	156	-20.88	156	-20.88	965905	21	3411	1760
miR166e-3p	LOC_Os06g38050.1	168	-29.48	168	-29.48	965938	21	2151	1783
miR166e-3p	LOC_Os06g38470.1	168	-27.31	168	-27.31	965982	21	2178	573
miR166e-3p	LOC_Os06g38470.3	168	-27.31	168	-27.31	965984	21	2200	573
miR166e-3p	LOC_Os06g39150.1	150	-20.47	150	-20.47	966055	21	831	542
miR166e-3p	LOC_Os06g39220.1	161	-23.73	161	-23.73	966062	21	561	292
miR166e-3p	LOC_Os06g39840.1	156	-20.73	156	-20.73	966137	21	4296	1617

miR166e-3p	LOC_Os06g40330.1	152	-21.82	152	-21.82	966194	21	2410	1516
miR166e-3p	LOC_Os06g41880.1	152	-21.37	152	-21.37	966372	21	3006	237
miR166e-3p	LOC_Os06g44140.1	152	-21.59	152	-21.59	966630	21	2291	1447
miR166e-3p	LOC_Os06g44740.1	145	-20.09	145	-20.09	966697	21	2430	571
miR166e-3p	LOC_Os06g45310.1	145	-22.67	145	-22.67	966769	21	1481	522
miR166e-3p	LOC_Os06g46180.1	156	-20.74	156	-20.74	966874	21	5193	3565
miR166e-3p	LOC_Os06g47190.1	169	-26.03	169	-26.03	967002	21	1209	712
miR166e-3p	LOC_Os06g47260.1	148	-24.71	148	-24.71	967010	21	871	175
miR166e-3p	LOC_Os06g47260.2	148	-24.71	148	-24.71	967011	21	873	175
miR166e-3p	LOC_Os06g47544.1	146	-21.72	146	-21.72	967045	21	581	350
miR166e-3p	LOC_Os06g48990.1	158	-23.39	158	-23.39	967217	21	3409	1587
miR166e-3p	LOC_Os06g48990.2	158	-23.39	158	-23.39	967218	21	3451	1629
miR166e-3p	LOC_Os06g50840.1	155	-23.82	155	-23.82	967456	21	1976	567
miR166e-3p	LOC_Os06g51310.1	157	-24.46	157	-24.46	967522	21	3759	1030
miR166e-3p	LOC_Os06g51310.2	157	-24.46	157	-24.46	967523	21	3747	1018
miR166e-3p	LOC_Os07g01730.1	158	-28.63	158	-28.63	967648	21	1871	1224
miR166e-3p	LOC_Os07g01890.2	160	-20.37	160	-20.37	967672	21	2488	2426
miR166e-3p	LOC_Os07g02510.1	154	-22.34	154	-22.34	967736	21	3963	1747
miR166e-3p	LOC_Os07g03740.1	156	-24.12	156	-24.12	967869	21	1098	236
miR166e-3p	LOC_Os07g04220.1	157	-28.92	157	-28.92	967916	21	2805	513
miR166e-3p	LOC_Os07g05440.1	163	-21.7	163	-21.7	968045	21	5102	4718
miR166e-3p	LOC_Os07g05440.2	163	-21.7	163	-21.7	968046	21	4620	4236
miR166e-3p	LOC_Os07g05600.1	162	-21.13	162	-21.13	968065	21	4341	839
miR166e-3p	LOC_Os07g05740.1	150	-20.1	150	-20.1	968082	21	4163	2237
miR166e-3p	LOC_Os07g05740.2	150	-20.1	150	-20.1	968083	21	4535	2606
miR166e-3p	LOC_Os07g06550.1	159	-21.79	159	-21.79	968163	21	1799	968
miR166e-3p	LOC_Os07g07130.1	162	-21.48	162	-21.48	968235	21	2092	1242
miR166e-3p	LOC_Os07g07530.1	155	-20.75	155	-20.75	968281	21	1992	603
miR166e-3p	LOC_Os07g07690.1	171	-26.88	171	-26.88	968300	21	5108	2811
miR166e-3p	LOC_Os07g07690.2	171	-26.88	171	-26.88	968301	21	4844	2811
miR166e-3p	LOC_Os07g08040.1	146	-24.44	146	-24.44	968335	21	2181	779
miR166e-3p	LOC_Os07g08150.1	147	-20.9	147	-20.9	968344	21	1154	615

miR166e-3p	LOC_Os07g08160.1	150	-25.6	150	-25.6	968345	21	860	466
miR166e-3p	LOC_Os07g11200.1	155	-21.05	155	-21.05	968690	21	1974	1840
miR166e-3p	LOC_Os07g11220.1	156	-21.3	156	-21.3	968692	21	2133	2064
miR166e-3p	LOC_Os07g11400.1	173	-28.97	173	-28.97	968712	21	5247	4336
miR166e-3p	LOC_Os07g11460.1	159	-20.95	159	-20.95	968718	21	2520	575
miR166e-3p	LOC_Os07g11480.1	152	-23.51	152	-23.51	968719	21	3153	2140
miR166e-3p	LOC_Os07g11870.1	163	-22.82	163	-22.82	968755	21	2068	273
miR166e-3p	LOC_Os07g11890.1	163	-22.82	163	-22.82	968756	21	1437	29
miR166e-3p	LOC_Os07g12850.1	152	-28.11	152	-28.11	968860	21	2145	866
miR166e-3p	LOC_Os07g13610.1	154	-22.34	154	-22.34	968936	21	3909	1693
miR166e-3p	LOC_Os07g13900.1	162	-23.2	162	-23.2	968960	21	1350	324
miR166e-3p	LOC_Os07g16120.1	152	-21.21	152	-21.21	969199	21	4737	603
miR166e-3p	LOC_Os07g17260.1	302	-44.32	151	-22.42	969311	21	5431	685 3501
miR166e-3p	LOC_Os07g17260.2	302	-44.32	151	-22.42	969312	21	5288	542 3358
miR166e-3p	LOC_Os07g17270.1	146	-20.44	146	-20.44	969313	21	5502	261
miR166e-3p	LOC_Os07g17630.1	153	-20.05	153	-20.05	969351	21	3405	1171
miR166e-3p	LOC_Os07g18620.1	154	-22.34	154	-22.34	969451	21	3687	1471
miR166e-3p	LOC_Os07g18970.1	151	-21.9	151	-21.9	969480	21	4947	2801
miR166e-3p	LOC_Os07g19070.2	141	-20.3	141	-20.3	969491	21	4176	3027
miR166e-3p	LOC_Os07g22640.1	146	-21.76	146	-21.76	969701	21	2221	1765
miR166e-3p	LOC_Os07g22640.2	146	-21.76	146	-21.76	969702	21	1681	1225
miR166e-3p	LOC_Os07g22640.3	146	-21.76	146	-21.76	969703	21	1319	863
miR166e-3p	LOC_Os07g22640.4	146	-21.76	146	-21.76	969704	21	1995	1539
miR166e-3p	LOC_Os07g23070.1	153	-20.05	153	-20.05	969757	21	3468	2356
miR166e-3p	LOC_Os07g23370.1	160	-20.14	160	-20.14	969784	21	957	197
miR166e-3p	LOC_Os07g25410.1	151	-20.64	151	-20.64	970000	21	1382	1297
miR166e-3p	LOC_Os07g26210.1	170	-31.81	170	-31.81	970081	21	4649	4576
miR166e-3p	LOC_Os07g28370.1	153	-20.33	153	-20.33	970313	21	5385	4420
miR166e-3p	LOC_Os07g30040.1	148	-20.17	148	-20.17	970492	21	3819	3301
miR166e-3p	LOC_Os07g32010.1	155	-27.34	155	-27.34	970697	21	1951	1141
miR166e-3p	LOC_Os07g32020.1	148	-22.03	148	-22.03	970698	21	2402	1149
miR166e-3p	LOC_Os07g32180.1	154	-22.34	154	-22.34	970714	21	3963	1747

miR166e-3p	LOC_Os07g33250.1	148	-24.55	148	-24.55	970833	21	3039	1023
miR166e-3p	LOC_Os07g33450.1	142	-23.7	142	-23.7	970856	21	4422	569
miR166e-3p	LOC_Os07g34980.1	148	-21.55	148	-21.55	971019	21	1977	572
miR166e-3p	LOC_Os07g35290.1	161	-23.6	161	-23.6	971052	21	2402	1096
miR166e-3p	LOC_Os07g36680.1	158	-22.84	158	-22.84	971214	21	675	9
miR166e-3p	LOC_Os07g36690.1	158	-22.99	158	-22.99	971215	21	3005	473
miR166e-3p	LOC_Os07g36700.1	158	-22.99	158	-22.99	971216	21	2583	303
miR166e-3p	LOC_Os07g37350.1	158	-23.41	158	-23.41	971290	21	2368	2293
miR166e-3p	LOC_Os07g37690.1	298	-46.87	150	-24.84	971328	21	1708	780 1157
miR166e-3p	LOC_Os07g37750.1	161	-21.84	161	-21.84	971334	21	5490	908
miR166e-3p	LOC_Os07g38210.1	163	-20.81	163	-20.81	971387	21	321	132
miR166e-3p	LOC_Os07g40910.1	145	-20.29	145	-20.29	971695	21	633	390
miR166e-3p	LOC_Os07g41480.1	154	-22.34	154	-22.34	971754	21	2868	1690
miR166e-3p	LOC_Os07g41630.1	153	-21.96	153	-21.96	971772	21	591	444
miR166e-3p	LOC_Os07g41640.1	164	-22.23	164	-22.23	971773	21	3039	1181
miR166e-3p	LOC_Os07g41640.2	164	-22.23	164	-22.23	971774	21	2924	1066
miR166e-3p	LOC_Os07g41640.3	164	-22.23	164	-22.23	971775	21	2921	1181
miR166e-3p	LOC_Os07g41820.1	142	-20.1	142	-20.1	971804	21	2460	1239
miR166e-3p	LOC_Os07g42640.1	157	-23.24	157	-23.24	971894	21	1482	130
miR166e-3p	LOC_Os07g42640.2	157	-23.24	157	-23.24	971895	21	1362	125
miR166e-3p	LOC_Os07g42714.1	164	-21.09	164	-21.09	971906	21	3539	937
miR166e-3p	LOC_Os07g44340.1	161	-20.23	161	-20.23	972096	21	2199	1751
miR166e-3p	LOC_Os07g44790.1	157	-20.62	157	-20.62	972146	21	1001	776
miR166e-3p	LOC_Os07g45120.1	308	-49.18	154	-24.59	972182	21	3107	38 2843
miR166e-3p	LOC_Os07g45490.1	166	-24.2	166	-24.2	972218	21	2895	225
miR166e-3p	LOC_Os07g45940.1	152	-20.19	152	-20.19	972262	21	5832	172
miR166e-3p	LOC_Os07g46460.1	146	-20.73	146	-20.73	972324	21	5804	3244
miR166e-3p	LOC_Os07g47830.1	151	-20.2	151	-20.2	972528	21	2504	1873
miR166e-3p	LOC_Os07g47900.1	162	-21.45	162	-21.45	972536	21	435	230
miR166e-3p	LOC_Os07g48090.1	317	-43.12	162	-22.36	972556	21	2143	1900 1509
miR166e-3p	LOC_Os07g49000.1	146	-21.06	146	-21.06	972679	21	2582	215
miR166e-3p	LOC_Os07g49310.1	161	-23.65	161	-23.65	972715	21	1690	782

miR166e-3p	LOC_Os07g49310.2	161	-23.65	161	-23.65	972716	21	1695	782
miR166e-3p	LOC_Os08g01120.1	156	-25.48	156	-25.48	972766	21	2176	1026
miR166e-3p	LOC_Os08g01130.1	156	-25.48	156	-25.48	972767	21	2656	116
miR166e-3p	LOC_Os08g02000.1	154	-21.52	154	-21.52	972877	21	4392	3956
miR166e-3p	LOC_Os08g03290.1	151	-22.31	151	-22.31	973017	21	1668	1476
miR166e-3p	LOC_Os08g03290.2	151	-22.31	151	-22.31	973018	21	1660	1468
miR166e-3p	LOC_Os08g03290.3	151	-22.31	151	-22.31	973019	21	1920	1728
miR166e-3p	LOC_Os08g04030.1	146	-20.15	146	-20.15	973110	21	795	109
miR166e-3p	LOC_Os08g04190.1	157	-21.53	157	-21.53	973131	21	2566	646
miR166e-3p	LOC_Os08g07220.1	153	-20.05	153	-20.05	973470	21	2940	1786
miR166e-3p	LOC_Os08g07830.1	154	-25.12	154	-25.12	973531	21	2241	2016
miR166e-3p	LOC_Os08g07830.2	154	-25.12	154	-25.12	973532	21	2263	2095
miR166e-3p	LOC_Os08g08190.1	158	-22.46	158	-22.46	973572	21	1540	1176
miR166e-3p	LOC_Os08g08200.1	153	-21.75	153	-21.75	973573	21	1534	1156
miR166e-3p	LOC_Os08g08200.2	153	-21.75	153	-21.75	973574	21	1590	1212
miR166e-3p	LOC_Os08g08220.1	152	-21.37	152	-21.37	973577	21	3905	1566
miR166e-3p	LOC_Os08g08220.2	152	-21.37	152	-21.37	973578	21	4239	1900
miR166e-3p	LOC_Os08g08220.3	152	-21.37	152	-21.37	973579	21	4030	1566
miR166e-3p	LOC_Os08g08820.1	155	-22.79	155	-22.79	973642	21	3327	3005
miR166e-3p	LOC_Os08g08820.2	155	-22.79	155	-22.79	973643	21	3421	3099
miR166e-3p	LOC_Os08g09340.1	150	-24.32	150	-24.32	973702	21	1913	838
miR166e-3p	LOC_Os08g09340.2	150	-24.32	150	-24.32	973703	21	2000	838
miR166e-3p	LOC_Os08g10970.1	159	-20.53	159	-20.53	973884	21	1368	618
miR166e-3p	LOC_Os08g11150.1	159	-23.67	159	-23.67	973902	21	4680	3735
miR166e-3p	LOC_Os08g11200.1	159	-22.38	159	-22.38	973907	21	5385	4158
miR166e-3p	LOC_Os08g12230.1	154	-21.89	154	-21.89	974014	21	2297	389
miR166e-3p	LOC_Os08g14660.1	149	-20.67	149	-20.67	974273	21	2411	789
miR166e-3p	LOC_Os08g15010.1	151	-21.9	151	-21.9	974313	21	5484	2801
miR166e-3p	LOC_Os08g16090.1	159	-27.57	159	-27.57	974440	21	2751	2209
miR166e-3p	LOC_Os08g16530.1	169	-27.92	169	-27.92	974490	21	2685	908
miR166e-3p	LOC_Os08g18140.1	156	-21.14	156	-21.14	974651	21	2553	142
miR166e-3p	LOC_Os08g18920.1	154	-24.54	154	-24.54	974680	21	3173	3122

miR166e-3p	LOC_Os08g19450.1	153	-20.05	153	-20.05	974743	21	2997	1333
miR166e-3p	LOC_Os08g21330.1	162	-25.42	162	-25.42	974914	21	2153	1322
miR166e-3p	LOC_Os08g21330.3	162	-25.42	162	-25.42	974915	21	2093	1262
miR166e-3p	LOC_Os08g21480.1	148	-22.68	148	-22.68	974931	21	1779	1304
miR166e-3p	LOC_Os08g21920.1	149	-20.75	149	-20.75	974971	21	4683	3941
miR166e-3p	LOC_Os08g22180.1	148	-22.68	148	-22.68	975003	21	4056	3596
miR166e-3p	LOC_Os08g22610.1	155	-22.33	155	-22.33	975038	21	804	42
miR166e-3p	LOC_Os08g22620.1	153	-20.18	153	-20.18	975039	21	3936	1720
miR166e-3p	LOC_Os08g22740.1	161	-21.72	161	-21.72	975049	21	1215	1110
miR166e-3p	LOC_Os08g23250.1	152	-20.76	152	-20.76	975101	21	1557	1134
miR166e-3p	LOC_Os08g24670.1	155	-20.34	155	-20.34	975250	21	786	472
miR166e-3p	LOC_Os08g25510.1	153	-20.57	153	-20.57	975333	21	1293	565
miR166e-3p	LOC_Os08g26660.1	151	-22.79	151	-22.79	975447	21	1599	893
miR166e-3p	LOC_Os08g26850.1	170	-22.17	170	-22.17	975470	21	1827	1081
miR166e-3p	LOC_Os08g27530.1	159	-26.72	159	-26.72	975542	21	2235	151
miR166e-3p	LOC_Os08g27570.1	165	-24.92	165	-24.92	975548	21	2562	1954
miR166e-3p	LOC_Os08g27990.1	159	-22.87	159	-22.87	975594	21	840	29
miR166e-3p	LOC_Os08g28630.1	150	-21.97	150	-21.97	975659	21	4488	105
miR166e-3p	LOC_Os08g28770.1	161	-20.44	161	-20.44	975674	21	2679	2144
miR166e-3p	LOC_Os08g28870.1	150	-20.1	150	-20.1	975685	21	3265	2459
miR166e-3p	LOC_Os08g31090.1	150	-22.92	150	-22.92	975912	21	1264	151
miR166e-3p	LOC_Os08g32370.1	168	-21.7	168	-21.7	976056	21	2133	1979
miR166e-3p	LOC_Os08g33920.1	152	-20.94	152	-20.94	976237	21	757	615
miR166e-3p	LOC_Os08g34280.1	153	-23.99	153	-23.99	976278	21	1495	385
miR166e-3p	LOC_Os08g35210.1	150	-21.41	150	-21.41	976384	21	3945	1247
miR166e-3p	LOC_Os08g35370.1	163	-20.49	163	-20.49	976402	21	2064	1975
miR166e-3p	LOC_Os08g36290.1	154	-22.34	154	-22.34	976500	21	4077	1861
miR166e-3p	LOC_Os08g38220.1	160	-23.76	160	-23.76	976737	21	1297	253
miR166e-3p	LOC_Os08g38800.1	145	-21.2	145	-21.2	976800	21	2448	2299
miR166e-3p	LOC_Os08g39120.1	155	-21.23	155	-21.23	976849	21	2382	2143
miR166e-3p	LOC_Os08g40340.1	154	-25.57	154	-25.57	976993	21	2133	2019
miR166e-3p	LOC_Os08g41410.1	162	-20.9	162	-20.9	977129	21	1521	846

miR166e-3p	LOC_Os08g41940.1	160	-23.36	160	-23.36	977190	21	1708	1190
miR166e-3p	LOC_Os08g43520.1	154	-22.34	154	-22.34	977388	21	3963	1747
miR166e-3p	LOC_Os08g44320.1	152	-22.56	152	-22.56	977476	21	1151	1128
miR166e-3p	LOC_Os08g45030.1	158	-23.41	158	-23.41	977579	21	4618	2786
miR166e-3p	LOC_Os09g01900.1	155	-23.02	155	-23.02	977719	21	2508	987
miR166e-3p	LOC_Os09g02170.1	162	-20.27	162	-20.27	977745	21	1313	2
miR166e-3p	LOC_Os09g05090.1	148	-22.68	148	-22.68	978048	21	756	680
miR166e-3p	LOC_Os09g06150.1	152	-23.51	152	-23.51	978055	21	6033	4144
miR166e-3p	LOC_Os09g06400.1	153	-23.38	153	-23.38	978080	21	1104	303
miR166e-3p	LOC_Os09g07120.1	154	-29.96	154	-29.96	978152	21	3754	3716
miR166e-3p	LOC_Os09g07120.2	154	-29.96	154	-29.96	978153	21	3650	3612
miR166e-3p	LOC_Os09g07220.1	150	-20.47	150	-20.47	978163	21	1689	356
miR166e-3p	LOC_Os09g07470.1	146	-20.96	146	-20.96	978192	21	387	39
miR166e-3p	LOC_Os09g07680.1	153	-20.05	153	-20.05	978220	21	4254	2023
miR166e-3p	LOC_Os09g07870.1	309	-42.2	157	-22.03	978243	21	2412	2366 1900
miR166e-3p	LOC_Os09g08590.1	148	-21.55	148	-21.55	978317	21	2943	123
miR166e-3p	LOC_Os09g09410.1	154	-22.23	154	-22.23	978401	21	567	134
miR166e-3p	LOC_Os09g09600.1	155	-27.9	155	-27.9	978422	21	765	21
miR166e-3p	LOC_Os09g09910.1	154	-22.34	154	-22.34	978452	21	3495	1279
miR166e-3p	LOC_Os09g10390.1	150	-20.13	150	-20.13	978500	21	2562	482
miR166e-3p	LOC_Os09g12150.1	154	-28.34	154	-28.34	978681	21	1386	688
miR166e-3p	LOC_Os09g12560.1	167	-29.15	167	-29.15	978722	21	1970	245
miR166e-3p	LOC_Os09g12560.2	167	-29.15	167	-29.15	978723	21	1866	141
miR166e-3p	LOC_Os09g13040.1	154	-20.24	154	-20.24	978775	21	4149	2741
miR166e-3p	LOC_Os09g13390.1	163	-20.82	163	-20.82	978809	21	243	166
miR166e-3p	LOC_Os09g13770.1	146	-21.1	146	-21.1	978851	21	651	553
miR166e-3p	LOC_Os09g14500.1	151	-21.9	151	-21.9	978926	21	4806	2801
miR166e-3p	LOC_Os09g15180.1	152	-20.37	152	-20.37	979002	21	1299	917
miR166e-3p	LOC_Os09g15680.1	148	-21.78	148	-21.78	979062	21	855	223
miR166e-3p	LOC_Os09g16540.1	161	-23.04	161	-23.04	979152	21	3429	1201
miR166e-3p	LOC_Os09g16920.1	163	-31.55	163	-31.55	979191	21	2140	1187
miR166e-3p	LOC_Os09g17049.1	154	-22.61	154	-22.61	979205	21	2637	2361

miR166e-3p	LOC_Os09g17049.2	154	-22.61	154	-22.61	979206	21	1844	1568
miR166e-3p	LOC_Os09g17180.1	145	-21.2	145	-21.2	979219	21	414	352
miR166e-3p	LOC_Os09g19500.1	154	-21.67	154	-21.67	979394	21	1119	293
miR166e-3p	LOC_Os09g19980.1	160	-26.15	160	-26.15	979447	21	2315	1281
miR166e-3p	LOC_Os09g20810.1	153	-21.16	153	-21.16	979542	21	1089	1017
miR166e-3p	LOC_Os09g21370.1	158	-21.47	158	-21.47	979603	21	2181	323
miR166e-3p	LOC_Os09g21830.1	152	-25.72	152	-25.72	979652	21	408	334
miR166e-3p	LOC_Os09g23120.1	153	-20.05	153	-20.05	979730	21	5328	2782
miR166e-3p	LOC_Os09g24924.1	161	-23.85	161	-23.85	979928	21	2000	78
miR166e-3p	LOC_Os09g25070.1	151	-20.63	151	-20.63	979944	21	1330	444
miR166e-3p	LOC_Os09g25070.2	151	-20.63	151	-20.63	979945	21	1463	444
miR166e-3p	LOC_Os09g26660.1	154	-24.05	154	-24.05	980124	21	3601	1451
miR166e-3p	LOC_Os09g27070.1	152	-23.25	152	-23.25	980170	21	1215	307
miR166e-3p	LOC_Os09g27080.1	155	-22.75	155	-22.75	980171	21	2732	703
miR166e-3p	LOC_Os09g27080.2	155	-22.75	155	-22.75	980172	21	2790	631
miR166e-3p	LOC_Os09g27300.1	151	-22	151	-22	980196	21	6804	6769
miR166e-3p	LOC_Os09g27770.1	153	-20.05	153	-20.05	980264	21	2784	178
miR166e-3p	LOC_Os09g29200.1	172	-28.12	172	-28.12	980430	21	1252	680
miR166e-3p	LOC_Os09g29210.1	172	-28.12	172	-28.12	980431	21	1853	232
miR166e-3p	LOC_Os09g29810.1	151	-21.9	151	-21.9	980493	21	5283	2801
miR166e-3p	LOC_Os09g32780.1	162	-21.29	162	-21.29	980802	21	3841	3658
miR166e-3p	LOC_Os09g33876.1	165	-20.19	165	-20.19	980901	21	2322	439
miR166e-3p	LOC_Os09g36220.1	155	-20.47	155	-20.47	981079	21	2416	2357
miR166e-3p	LOC_Os09g36220.2	155	-20.47	155	-20.47	981080	21	2475	2416
miR166e-3p	LOC_Os09g38030.1	165	-24.31	165	-24.31	981294	21	1758	1368
miR166e-3p	LOC_Os09g38030.2	165	-24.31	165	-24.31	981295	21	1966	1368
miR166e-3p	LOC_Os09g38480.1	164	-28.33	164	-28.33	981339	21	1464	1395
miR166e-3p	LOC_Os09g38800.1	148	-20.02	148	-20.02	981391	21	2351	707
miR166e-3p	LOC_Os09g39290.1	153	-22.06	153	-22.06	981440	21	6084	5129
miR166e-3p	LOC_Os09g39560.1	165	-23.71	165	-23.71	981474	21	1759	950
miR166e-3p	LOC_Os09g39690.1	159	-22.02	159	-22.02	981492	21	2575	2139
miR166e-3p	LOC_Os10g01210.1	149	-31.29	149	-31.29	981565	21	1287	557

miR166e-3p	LOC_Os10g01890.1	147	-20.8	147	-20.8	981632	21	3144	2305
miR166e-3p	LOC_Os10g01950.1	155	-20.22	155	-20.22	981638	21	2889	2223
miR166e-3p	LOC_Os10g02680.1	147	-20.59	147	-20.59	981717	21	270	96
miR166e-3p	LOC_Os10g03830.1	153	-20.95	153	-20.95	981833	21	2846	486
miR166e-3p	LOC_Os10g03830.2	153	-20.95	153	-20.95	981834	21	2815	486
miR166e-3p	LOC_Os10g04074.1	154	-24.59	154	-24.59	981861	21	7816	7442
miR166e-3p	LOC_Os10g04750.1	160	-24.16	160	-24.16	981925	21	393	85
miR166e-3p	LOC_Os10g05290.1	146	-21.03	146	-21.03	981979	21	2042	256
miR166e-3p	LOC_Os10g05290.2	146	-21.03	146	-21.03	981980	21	1549	256
miR166e-3p	LOC_Os10g05690.1	150	-20.83	150	-20.83	982019	21	2129	1772
miR166e-3p	LOC_Os10g05750.1	151	-27.21	151	-27.21	982028	21	1289	739
miR166e-3p	LOC_Os10g06110.1	152	-20.64	152	-20.64	982061	21	2547	1061
miR166e-3p	LOC_Os10g06510.1	145	-20.37	145	-20.37	982103	21	1260	951
miR166e-3p	LOC_Os10g06780.1	145	-20.37	145	-20.37	982128	21	972	877
miR166e-3p	LOC_Os10g08280.1	145	-21.45	145	-21.45	982268	21	1802	934
miR166e-3p	LOC_Os10g08530.1	158	-24.35	158	-24.35	982289	21	912	92
miR166e-3p	LOC_Os10g08660.1	149	-20.59	149	-20.59	982304	21	3771	259
miR166e-3p	LOC_Os10g09900.1	153	-22.36	153	-22.36	982423	21	2637	2338
miR166e-3p	LOC_Os10g10270.1	149	-21.03	149	-21.03	982466	21	786	11
miR166e-3p	LOC_Os10g11200.1	161	-22.31	161	-22.31	982557	21	2184	2069
miR166e-3p	LOC_Os10g11720.1	153	-22.97	153	-22.97	982607	21	630	320
miR166e-3p	LOC_Os10g12290.1	149	-21.17	149	-21.17	982655	21	1165	576
miR166e-3p	LOC_Os10g14180.2	159	-23.38	159	-23.38	982790	21	7259	4074
miR166e-3p	LOC_Os10g14180.3	159	-23.38	159	-23.38	982791	21	5790	2606
miR166e-3p	LOC_Os10g14180.4	159	-23.38	159	-23.38	982792	21	5830	2646
miR166e-3p	LOC_Os10g14180.5	159	-23.38	159	-23.38	982793	21	5827	2643
miR166e-3p	LOC_Os10g14180.6	159	-23.38	159	-23.38	982794	21	5651	2467
miR166e-3p	LOC_Os10g14180.7	159	-23.38	159	-23.38	982795	21	5555	2371
miR166e-3p	LOC_Os10g14180.8	159	-23.38	159	-23.38	982796	21	5552	2368
miR166e-3p	LOC_Os10g14180.9	159	-23.38	159	-23.38	982797	21	7149	3965
miR166e-3p	LOC_Os10g14889.1	146	-20.08	146	-20.08	982817	21	447	186
miR166e-3p	LOC_Os10g16990.1	157	-20.43	157	-20.43	982928	21	3040	2435

miR166e-3p	LOC_Os10g17400.1	166	-32.29	166	-32.29	982967	21	3363	418
miR166e-3p	LOC_Os10g17570.1	151	-21.9	151	-21.9	982982	21	2805	920
miR166e-3p	LOC_Os10g18026.1	163	-24.13	163	-24.13	983025	21	1254	36
miR166e-3p	LOC_Os10g19230.1	150	-20.04	150	-20.04	983132	21	1236	474
miR166e-3p	LOC_Os10g21130.1	151	-20.63	151	-20.63	983290	21	1210	1064
miR166e-3p	LOC_Os10g22010.1	152	-23.51	152	-23.51	983416	21	6108	3997
miR166e-3p	LOC_Os10g22820.1	155	-20.76	155	-20.76	983505	21	381	279
miR166e-3p	LOC_Os10g22930.1	145	-23.2	145	-23.2	983518	21	1947	329
miR166e-3p	LOC_Os10g24940.1	154	-21.52	154	-21.52	983675	21	5451	4997
miR166e-3p	LOC_Os10g25890.1	152	-23.51	152	-23.51	983768	21	3879	3109
miR166e-3p	LOC_Os10g26050.1	153	-20.28	153	-20.28	983783	21	2341	496
miR166e-3p	LOC_Os10g26050.2	153	-20.28	153	-20.28	983784	21	2260	496
miR166e-3p	LOC_Os10g26050.3	153	-20.28	153	-20.28	983785	21	2349	496
miR166e-3p	LOC_Os10g26050.4	153	-20.28	153	-20.28	983786	21	2674	910
miR166e-3p	LOC_Os10g26920.1	153	-20.05	153	-20.05	983876	21	5178	2845
miR166e-3p	LOC_Os10g27080.1	145	-21.2	145	-21.2	983892	21	753	604
miR166e-3p	LOC_Os10g29040.1	153	-23.91	153	-23.91	984055	21	816	589
miR166e-3p	LOC_Os10g29070.1	161	-21.65	161	-21.65	984059	21	2244	515
miR166e-3p	LOC_Os10g29420.1	162	-21.13	162	-21.13	984092	21	5136	1427
miR166e-3p	LOC_Os10g29970.1	154	-22.34	154	-22.34	984147	21	3894	1678
miR166e-3p	LOC_Os10g31610.1	322	-55.33	166	-33.52	984316	21	927	761 779
miR166e-3p	LOC_Os10g32640.1	163	-30.27	163	-30.27	984405	21	2733	950
miR166e-3p	LOC_Os10g32860.1	158	-21.89	158	-21.89	984434	21	558	357
miR166e-3p	LOC_Os10g32860.2	158	-21.89	158	-21.89	984435	21	705	399
miR166e-3p	LOC_Os10g33410.1	149	-22.04	149	-22.04	984490	21	318	180
miR166e-3p	LOC_Os10g33770.1	154	-20.27	154	-20.27	984531	21	2790	1682
miR166e-3p	LOC_Os10g36720.1	154	-22.34	154	-22.34	984872	21	3963	1747
miR166e-3p	LOC_Os10g36780.1	149	-25.29	149	-25.29	984878	21	1978	572
miR166e-3p	LOC_Os10g36780.2	149	-25.29	149	-25.29	984879	21	1952	572
miR166e-3p	LOC_Os10g36780.3	149	-25.29	149	-25.29	984880	21	1958	572
miR166e-3p	LOC_Os10g36780.4	149	-25.29	149	-25.29	984881	21	2078	572
miR166e-3p	LOC_Os10g37170.1	161	-20.71	161	-20.71	984910	21	4020	1016

miR166e-3p	LOC_Os10g37570.1	150	-21.25	150	-21.25	984946	21	1335	123
miR166e-3p	LOC_Os10g37720.1	149	-22.18	149	-22.18	984964	21	2242	296
miR166e-3p	LOC_Os10g38740.1	156	-21.16	156	-21.16	985070	21	1643	468
miR166e-3p	LOC_Os10g40590.1	149	-25.19	149	-25.19	985304	21	2845	387
miR166e-3p	LOC_Os10g41340.1	155	-22.94	155	-22.94	985388	21	1418	804
miR166e-3p	LOC_Os10g42470.1	153	-20.05	153	-20.05	985527	21	3300	379
miR166e-3p	LOC_Os11g01270.1	154	-24.74	154	-24.74	985644	21	2660	2214
miR166e-3p	LOC_Os11g01920.1	153	-20.49	153	-20.49	985725	21	5946	5054
miR166e-3p	LOC_Os11g01970.1	150	-20.88	150	-20.88	985729	21	3777	1
miR166e-3p	LOC_Os11g02054.1	151	-21.7	151	-21.7	985736	21	1350	522
miR166e-3p	LOC_Os11g02540.1	155	-21.64	155	-21.64	985798	21	1132	467
miR166e-3p	LOC_Os11g03010.1	143	-20.09	143	-20.09	985850	21	2388	2315
miR166e-3p	LOC_Os11g03940.1	154	-22.34	154	-22.34	985953	21	6084	3283
miR166e-3p	LOC_Os11g04640.1	154	-22.34	154	-22.34	986042	21	3963	1747
miR166e-3p	LOC_Os11g04700.1	159	-21.51	159	-21.51	986049	21	1347	1004
miR166e-3p	LOC_Os11g07600.1	151	-20.97	151	-20.97	986405	21	2195	1101
miR166e-3p	LOC_Os11g07840.1	157	-21.47	157	-21.47	986428	21	1661	1302
miR166e-3p	LOC_Os11g07870.1	155	-20.44	155	-20.44	986431	21	4467	554
miR166e-3p	LOC_Os11g08020.1	153	-20.15	153	-20.15	986450	21	2024	169
miR166e-3p	LOC_Os11g08340.1	165	-28.08	165	-28.08	986488	21	1842	43
miR166e-3p	LOC_Os11g08450.1	154	-22.34	154	-22.34	986500	21	1584	1483
miR166e-3p	LOC_Os11g09720.1	159	-27.57	159	-27.57	986628	21	2031	1489
miR166e-3p	LOC_Os11g10810.1	296	-48.2	148	-24.1	986746	21	993	597 959
miR166e-3p	LOC_Os11g10820.1	153	-20.05	153	-20.05	986747	21	5790	2962
miR166e-3p	LOC_Os11g11290.1	308	-43.29	155	-22.24	986796	21	4977	4204 4667
miR166e-3p	LOC_Os11g11330.1	159	-22.87	159	-22.87	986800	21	2125	1099
miR166e-3p	LOC_Os11g11880.1	162	-25.7	162	-25.7	986857	21	1623	1346
miR166e-3p	LOC_Os11g11920.1	155	-20.06	155	-20.06	986860	21	3944	576
miR166e-3p	LOC_Os11g12430.1	152	-21.57	152	-21.57	986913	21	1017	605
miR166e-3p	LOC_Os11g14150.1	150	-24.97	150	-24.97	987041	21	1227	1199
miR166e-3p	LOC_Os11g16820.1	153	-20.49	153	-20.49	987292	21	5370	4535
miR166e-3p	LOC_Os11g17200.1	154	-22.34	154	-22.34	987325	21	3909	1693

miR166e-3p	LOC_Os11g17940.1	161	-20.95	161	-20.95	987397	21	609	336
miR166e-3p	LOC_Os11g18810.1	148	-22.68	148	-22.68	987473	21	4776	4295
miR166e-3p	LOC_Os11g19060.1	150	-21.55	150	-21.55	987502	21	2276	1221
miR166e-3p	LOC_Os11g19520.1	152	-23.51	152	-23.51	987551	21	4879	3410
miR166e-3p	LOC_Os11g20480.1	152	-20.43	152	-20.43	987648	21	1407	600
miR166e-3p	LOC_Os11g21920.1	171	-24.62	171	-24.62	987690	21	387	112
miR166e-3p	LOC_Os11g22040.1	149	-20.34	149	-20.34	987703	21	942	217
miR166e-3p	LOC_Os11g22300.1	153	-20.05	153	-20.05	987732	21	4473	2764
miR166e-3p	LOC_Os11g22440.1	154	-22.1	154	-22.1	987747	21	1212	415
miR166e-3p	LOC_Os11g22850.1	163	-30.04	163	-30.04	987786	21	2655	2261
miR166e-3p	LOC_Os11g24260.1	156	-21.72	156	-21.72	987890	21	567	160
miR166e-3p	LOC_Os11g25550.1	156	-20.59	156	-20.59	988010	21	462	393
miR166e-3p	LOC_Os11g27030.1	161	-23.77	161	-23.77	988151	21	510	354
miR166e-3p	LOC_Os11g27430.1	149	-22.21	149	-22.21	988188	21	3048	2390
miR166e-3p	LOC_Os11g27470.1	148	-20.72	148	-20.72	988191	21	588	557
miR166e-3p	LOC_Os11g28250.1	152	-23.51	152	-23.51	988266	21	1788	169
miR166e-3p	LOC_Os11g29200.1	156	-20.7	156	-20.7	988367	21	2934	631
miR166e-3p	LOC_Os11g30020.1	154	-22.34	154	-22.34	988453	21	4740	1666
miR166e-3p	LOC_Os11g30540.1	152	-20.03	152	-20.03	988502	21	2733	1515
miR166e-3p	LOC_Os11g30990.1	152	-23.51	152	-23.51	988540	21	4086	2458
miR166e-3p	LOC_Os11g32190.1	153	-20.94	153	-20.94	988668	21	885	659
miR166e-3p	LOC_Os11g33270.1	153	-21.58	153	-21.58	988790	21	1257	439
miR166e-3p	LOC_Os11g34500.1	153	-20.49	153	-20.49	988881	21	5403	4565
miR166e-3p	LOC_Os11g35756.1	157	-24.4	157	-24.4	989010	21	2802	2229
miR166e-3p	LOC_Os11g37340.1	155	-21.03	155	-21.03	989179	21	1197	264
miR166e-3p	LOC_Os11g39840.1	150	-21.23	150	-21.23	989451	21	492	142
miR166e-3p	LOC_Os11g40315.1	154	-22.35	154	-22.35	989500	21	2838	2693
miR166e-3p	LOC_Os11g40930.1	164	-23.78	164	-23.78	989573	21	618	299
miR166e-3p	LOC_Os11g42100.1	165	-21.36	165	-21.36	989693	21	1863	948
miR166e-3p	LOC_Os11g42930.1	167	-26.09	167	-26.09	989781	21	1595	1554
miR166e-3p	LOC_Os11g43290.1	154	-21.32	154	-21.32	989816	21	3369	2361
miR166e-3p	LOC_Os11g44190.1	151	-21.9	151	-21.9	989916	21	4167	2393

miR166e-3p	LOC_Os11g44770.1	169	-26.03	169	-26.03	989971	21	5943	5032
miR166e-3p	LOC_Os11g45610.1	162	-21.13	162	-21.13	990064	21	4813	1024
miR166e-3p	LOC_Os11g46200.1	150	-22.09	150	-22.09	990127	21	3378	85
miR166e-3p	LOC_Os11g46950.1	158	-20.41	158	-20.41	990156	21	2556	1591
miR166e-3p	LOC_Os11g47446.1	170	-26.32	170	-26.32	990217	21	2100	1843
miR166e-3p	LOC_Os12g01790.1	151	-22.99	151	-22.99	990400	21	3987	1531
miR166e-3p	LOC_Os12g02020.1	151	-22.87	151	-22.87	990430	21	483	411
miR166e-3p	LOC_Os12g02170.1	150	-22.75	150	-22.75	990449	21	1200	948
miR166e-3p	LOC_Os12g02470.1	153	-22.29	153	-22.29	990487	21	1026	434
miR166e-3p	LOC_Os12g04320.1	155	-23.26	155	-23.26	990718	21	1257	530
miR166e-3p	LOC_Os12g04840.1	163	-24.23	163	-24.23	990775	21	4419	3584
miR166e-3p	LOC_Os12g05090.1	162	-21.03	162	-21.03	990812	21	1512	1400
miR166e-3p	LOC_Os12g05380.1	153	-20.12	153	-20.12	990842	21	1541	1101
miR166e-3p	LOC_Os12g05490.1	153	-20.05	153	-20.05	990852	21	4020	2269
miR166e-3p	LOC_Os12g06230.1	142	-22.39	142	-22.39	990931	21	6840	152
miR166e-3p	LOC_Os12g06720.1	311	-44.73	159	-23.3	990988	21	1992	917 1044
miR166e-3p	LOC_Os12g07550.1	159	-21.99	159	-21.99	991082	21	848	734
miR166e-3p	LOC_Os12g07910.1	162	-21.13	162	-21.13	991121	21	4888	839
miR166e-3p	LOC_Os12g10090.1	154	-22.34	154	-22.34	991352	21	1869	1768
miR166e-3p	LOC_Os12g10900.1	151	-21.9	151	-21.9	991446	21	5388	3383
miR166e-3p	LOC_Os12g11900.1	155	-20.44	155	-20.44	991540	21	4265	3492
miR166e-3p	LOC_Os12g12300.1	141	-20.57	141	-20.57	991580	21	1525	418
miR166e-3p	LOC_Os12g13280.1	142	-25.25	142	-25.25	991688	21	408	207
miR166e-3p	LOC_Os12g14260.1	153	-20.05	153	-20.05	991796	21	5424	2596
miR166e-3p	LOC_Os12g14699.1	151	-23.96	151	-23.96	991834	21	2445	1647
miR166e-3p	LOC_Os12g14984.1	311	-45.1	159	-23.67	991857	21	2079	1098 1225
miR166e-3p	LOC_Os12g15420.1	156	-20.85	156	-20.85	991895	21	3404	1291
miR166e-3p	LOC_Os12g15790.1	148	-23.56	148	-23.56	991935	21	639	380
miR166e-3p	LOC_Os12g16670.1	149	-22.44	149	-22.44	992036	21	1625	677
miR166e-3p	LOC_Os12g17070.1	163	-24.76	163	-24.76	992074	21	3599	824
miR166e-3p	LOC_Os12g18840.1	145	-30.84	145	-30.84	992266	21	3681	2816
miR166e-3p	LOC_Os12g19510.1	153	-21.15	153	-21.15	992325	21	603	478

miR166e-3p	LOC_Os12g20000.1	159	-21.42	159	-21.42	992375	21	2199	494
miR166e-3p	LOC_Os12g22110.1	152	-21.71	152	-21.71	992498	21	1719	299
miR166e-3p	LOC_Os12g22670.1	304	-42.43	153	-22.15	992553	21	1167	569 342
miR166e-3p	LOC_Os12g23100.1	151	-20.05	151	-20.05	992593	21	1941	645
miR166e-3p	LOC_Os12g23520.1	152	-20.09	152	-20.09	992635	21	6172	5698
miR166e-3p	LOC_Os12g23730.1	154	-22.34	154	-22.34	992656	21	3963	1747
miR166e-3p	LOC_Os12g25990.1	150	-20.13	150	-20.13	992871	21	3937	542
miR166e-3p	LOC_Os12g27210.1	153	-24.72	153	-24.72	992999	21	2535	217
miR166e-3p	LOC_Os12g27420.1	162	-29.66	162	-29.66	993018	21	810	655
miR166e-3p	LOC_Os12g29520.1	153	-21.85	153	-21.85	993217	21	3622	2091
miR166e-3p	LOC_Os12g29520.2	153	-21.85	153	-21.85	993218	21	3639	2108
miR166e-3p	LOC_Os12g29520.3	153	-21.85	153	-21.85	993219	21	3619	2091
miR166e-3p	LOC_Os12g29520.4	153	-21.85	153	-21.85	993220	21	3619	2091
miR166e-3p	LOC_Os12g29520.5	153	-21.85	153	-21.85	993221	21	3701	2091
miR166e-3p	LOC_Os12g29560.1	171	-22.4	171	-22.4	993225	21	1723	411
miR166e-3p	LOC_Os12g29950.1	158	-22.98	158	-22.98	993265	21	2117	153
miR166e-3p	LOC_Os12g29950.2	158	-22.98	158	-22.98	993266	21	2022	153
miR166e-3p	LOC_Os12g30280.1	155	-22.28	155	-22.28	993302	21	789	432
miR166e-3p	LOC_Os12g31485.1	160	-20.29	160	-20.29	993416	21	410	199
miR166e-3p	LOC_Os12g32499.1	150	-21.53	150	-21.53	993520	21	2070	160
miR166e-3p	LOC_Os12g32499.2	150	-21.53	150	-21.53	993521	21	2080	160
miR166e-3p	LOC_Os12g32499.3	150	-21.53	150	-21.53	993522	21	2102	169
miR166e-3p	LOC_Os12g32730.1	159	-27.57	159	-27.57	993548	21	2610	2086
miR166e-3p	LOC_Os12g34140.1	161	-23.91	161	-23.91	993698	21	1632	289
miR166e-3p	LOC_Os12g35580.1	153	-20.34	153	-20.34	993861	21	1223	793
miR166e-3p	LOC_Os12g35580.2	153	-20.34	153	-20.34	993862	21	1458	793
miR166e-3p	LOC_Os12g36150.1	155	-21.05	155	-21.05	993922	21	2682	2104
miR166e-3p	LOC_Os12g36390.1	166	-23.24	166	-23.24	993943	21	3567	601
miR166e-3p	LOC_Os12g37260.1	158	-21.32	158	-21.32	994036	21	3218	949
miR166e-3p	LOC_Os12g38100.1	163	-26.23	163	-26.23	994135	21	707	281
miR166e-3p	LOC_Os12g38630.1	170	-27.04	170	-27.04	994194	21	2413	431
miR166e-3p	LOC_Os12g38850.1	168	-23.67	168	-23.67	994224	21	2746	973

miR166e-3p	LOC_Os12g38850.2	168	-23.67	168	-23.67	994225	21	2236	468
miR166e-3p	LOC_Os12g38850.3	168	-23.67	168	-23.67	994226	21	2346	578
miR166e-3p	LOC_Os12g39660.1	156	-20.95	156	-20.95	994327	21	3700	1593
miR166e-3p	LOC_Os12g39800.1	154	-22.34	154	-22.34	994340	21	5418	1747
miR166e-3p	LOC_Os12g41260.1	155	-20.89	155	-20.89	994506	21	1692	777
miR166e-3p	LOC_Os12g41280.1	154	-22.34	154	-22.34	994508	21	3909	1693
miR166e-3p	LOC_Os12g42420.1	153	-21.02	153	-21.02	994656	21	2152	1068
miR166e-3p	LOC_Os12g43270.1	153	-20.05	153	-20.05	994750	21	6012	3721
miR166e-3p	LOC_Os12g43664.1	163	-24.65	163	-24.65	994794	21	2289	752
miR166e-3p	LOC_Os12g43680.1	141	-21.22	141	-21.22	994796	21	774	490
miR166e-3p	LOC_Os12g43880.1	145	-21.45	145	-21.45	994815	21	1619	1086
miR166e-3p	ChrUn.fgenesh.mRNA.27	148	-22.68	148	-22.68	994912	21	3765	3629
miR166e-3p	ChrUn.fgenesh.mRNA.63	159	-23.67	159	-23.67	994948	21	1752	519
miR166e-3p	ChrSy.fgenesh.mRNA.7	151	-21.9	151	-21.9	994988	21	2670	758
miR166e-3p	ChrSy.fgenesh.mRNA.21	159	-23.75	159	-23.75	995002	21	2298	300
miR156l-5p	LOC_Os01g01250.1	164	-27.58	164	-27.58	995106	21	2499	1082
miR156l-5p	LOC_Os01g01550.1	161	-21.79	161	-21.79	995152	21	1329	757
miR156l-5p	LOC_Os01g01689.1	162	-21.18	162	-21.18	995166	21	8736	3055
miR156l-5p	LOC_Os01g01689.3	162	-21.18	162	-21.18	995167	21	9026	3055
miR156l-5p	LOC_Os01g01800.1	163	-21.24	163	-21.24	995180	21	3071	972
miR156l-5p	LOC_Os01g01800.2	163	-21.24	163	-21.24	995181	21	3133	1037
miR156l-5p	LOC_Os01g02560.1	149	-20.71	149	-20.71	995271	21	2059	755
miR156l-5p	LOC_Os01g04600.1	162	-25.89	162	-25.89	995489	21	1223	540
miR156l-5p	LOC_Os01g04600.2	162	-25.89	162	-25.89	995490	21	1659	1000
miR156l-5p	LOC_Os01g05980.1	166	-21.73	166	-21.73	995647	21	3129	765
miR156l-5p	LOC_Os01g06460.1	163	-23.37	163	-23.37	995701	21	1231	716
miR156l-5p	LOC_Os01g06700.1	174	-27.3	174	-27.3	995732	21	6213	3218
miR156l-5p	LOC_Os01g07570.1	156	-23.37	156	-23.37	995834	21	1963	1509
miR156l-5p	LOC_Os01g07660.1	147	-21.52	147	-21.52	995842	21	1697	98
miR156l-5p	LOC_Os01g09384.1	159	-21.65	159	-21.65	996047	21	3020	774
miR156l-5p	LOC_Os01g11260.1	164	-24.79	164	-24.79	996254	21	1784	1488
miR156l-5p	LOC_Os01g13270.1	165	-22.18	165	-22.18	996486	21	2066	146

miR156l-5p	LOC_Os01g13270.2	165	-22.18	165	-22.18	996487	21	1968	146
miR156l-5p	LOC_Os01g13550.1	153	-20.35	153	-20.35	996519	21	1862	14
miR156l-5p	LOC_Os01g14440.1	147	-22.79	147	-22.79	996618	21	2114	1120
miR156l-5p	LOC_Os01g18040.1	160	-24.17	160	-24.17	996973	21	5331	3078
miR156l-5p	LOC_Os01g18470.1	152	-23.21	152	-23.21	997015	21	4038	3334
miR156l-5p	LOC_Os01g20190.1	169	-26.67	169	-26.67	997201	21	3183	2715
miR156l-5p	LOC_Os01g22660.1	171	-21.99	171	-21.99	997404	21	1988	659
miR156l-5p	LOC_Os01g22660.2	171	-21.99	171	-21.99	997405	21	3666	2337
miR156l-5p	LOC_Os01g22660.3	171	-21.99	171	-21.99	997406	21	1455	663
miR156l-5p	LOC_Os01g23040.1	156	-24.99	156	-24.99	997443	21	4995	482
miR156l-5p	LOC_Os01g24310.1	161	-21.79	161	-21.79	997571	21	3288	3178
miR156l-5p	LOC_Os01g24620.1	164	-27.58	164	-27.58	997602	21	3144	1727
miR156l-5p	LOC_Os01g27010.1	157	-26.15	157	-26.15	997803	21	1500	478
miR156l-5p	LOC_Os01g27040.1	164	-20.39	164	-20.39	997810	21	3750	3054
miR156l-5p	LOC_Os01g28244.1	164	-21.22	164	-21.22	997925	21	3174	2192
miR156l-5p	LOC_Os01g28420.1	171	-20.92	171	-20.92	997942	21	2019	1903
miR156l-5p	LOC_Os01g28600.1	162	-26.21	162	-26.21	997958	21	2326	403
miR156l-5p	LOC_Os01g28740.1	154	-21.87	154	-21.87	997982	21	3969	3239
miR156l-5p	LOC_Os01g28950.1	156	-24.99	156	-24.99	998002	21	4251	482
miR156l-5p	LOC_Os01g29040.1	157	-27.59	157	-27.59	998015	21	804	24
miR156l-5p	LOC_Os01g29530.1	161	-24.44	161	-24.44	998060	21	3387	338
miR156l-5p	LOC_Os01g29750.1	152	-23.21	152	-23.21	998081	21	4698	3922
miR156l-5p	LOC_Os01g31770.1	157	-20.58	157	-20.58	998186	21	1750	13
miR156l-5p	LOC_Os01g31770.2	157	-20.58	157	-20.58	998187	21	1855	13
miR156l-5p	LOC_Os01g32450.1	150	-22.27	150	-22.27	998261	21	312	165
miR156l-5p	LOC_Os01g32590.1	171	-20.92	171	-20.92	998274	21	675	400
miR156l-5p	LOC_Os01g32730.1	153	-20.25	153	-20.25	998290	21	1457	1395
miR156l-5p	LOC_Os01g32730.2	153	-20.25	153	-20.25	998291	21	1571	1509
miR156l-5p	LOC_Os01g34390.1	156	-20.41	156	-20.41	998448	21	1647	359
miR156l-5p	LOC_Os01g34880.1	153	-20.82	153	-20.82	998510	21	1725	623
miR156l-5p	LOC_Os01g35170.1	165	-24.78	165	-24.78	998540	21	1618	1051
miR156l-5p	LOC_Os01g35170.2	165	-24.78	165	-24.78	998541	21	1615	1048

miR156l-5p	LOC_Os01g35170.3	165	-24.78	165	-24.78	998542	21	1818	1251
miR156l-5p	LOC_Os01g35254.1	165	-20.01	165	-20.01	998550	21	4338	11
miR156l-5p	LOC_Os01g35640.1	155	-20.82	155	-20.82	998592	21	2409	1156
miR156l-5p	LOC_Os01g37250.1	156	-24.27	156	-24.27	998758	21	2220	2040
miR156l-5p	LOC_Os01g37880.1	164	-27.58	164	-27.58	998839	21	3144	1727
miR156l-5p	LOC_Os01g39370.1	156	-24.27	156	-24.27	998995	21	5430	4983
miR156l-5p	LOC_Os01g39810.1	153	-21.38	153	-21.38	999045	21	3109	2613
miR156l-5p	LOC_Os01g39810.3	153	-21.38	153	-21.38	999047	21	4086	3590
miR156l-5p	LOC_Os01g39820.1	153	-21.38	153	-21.38	999049	21	723	336
miR156l-5p	LOC_Os01g40000.1	161	-20.9	161	-20.9	999066	21	2941	1746
miR156l-5p	LOC_Os01g40110.1	175	-23.3	175	-23.3	999076	21	1341	385
miR156l-5p	LOC_Os01g40470.1	174	-28.42	174	-28.42	999117	21	1648	828
miR156l-5p	LOC_Os01g40960.1	153	-20.41	153	-20.41	999172	21	543	51
miR156l-5p	LOC_Os01g40990.2	170	-21.24	170	-21.24	999176	21	7852	5964
miR156l-5p	LOC_Os01g40990.3	170	-21.24	170	-21.24	999177	21	7745	5946
miR156l-5p	LOC_Os01g42140.1	161	-23.15	161	-23.15	999297	21	1380	167
miR156l-5p	LOC_Os01g42140.2	161	-23.15	161	-23.15	999298	21	916	167
miR156l-5p	LOC_Os01g43010.2	162	-22.47	162	-22.47	999401	21	3133	571
miR156l-5p	LOC_Os01g43030.1	161	-21.83	161	-21.83	999403	21	3557	2258
miR156l-5p	LOC_Os01g43030.2	161	-21.83	161	-21.83	999404	21	2664	1376
miR156l-5p	LOC_Os01g45470.1	166	-22.31	166	-22.31	999626	21	458	389
miR156l-5p	LOC_Os01g45580.1	164	-27.58	164	-27.58	999638	21	3144	1727
miR156l-5p	LOC_Os01g45800.1	164	-27.58	164	-27.58	999660	21	3126	1709
miR156l-5p	LOC_Os01g46710.1	169	-22.91	169	-22.91	999761	21	4742	3851
miR156l-5p	LOC_Os01g48270.1	174	-27.84	174	-27.84	999953	21	1706	410
miR156l-5p	LOC_Os01g49065.1	156	-22.62	156	-22.62	1000053	21	3338	1979
miR156l-5p	LOC_Os01g50470.1	166	-20.99	166	-20.99	1000202	21	3727	281
miR156l-5p	LOC_Os01g50710.1	156	-21.8	156	-21.8	1000231	21	435	324
miR156l-5p	LOC_Os01g50800.1	327	-46.61	175	-23.4	1000241	21	2037	546 1309
miR156l-5p	LOC_Os01g52000.1	161	-22.46	161	-22.46	1000385	21	4302	1038
miR156l-5p	LOC_Os01g54080.1	166	-24.7	166	-24.7	1000626	21	3356	2060
miR156l-5p	LOC_Os01g56820.1	159	-22.99	159	-22.99	1000977	21	1191	787

miR156l-5p	LOC_Os01g56820.2	159	-22.99	159	-22.99	1000978	21	1164	760
miR156l-5p	LOC_Os01g57360.1	182	-29.7	182	-29.7	1001039	21	1615	1259
miR156l-5p	LOC_Os01g59260.1	161	-21.79	161	-21.79	1001262	21	3288	3178
miR156l-5p	LOC_Os01g59520.1	166	-20.29	166	-20.29	1001292	21	1423	824
miR156l-5p	LOC_Os01g59520.2	166	-20.29	166	-20.29	1001293	21	1335	824
miR156l-5p	LOC_Os01g59520.3	166	-20.29	166	-20.29	1001294	21	1510	824
miR156l-5p	LOC_Os01g59660.1	158	-25.49	158	-25.49	1001308	21	2215	1491
miR156l-5p	LOC_Os01g59660.2	158	-25.49	158	-25.49	1001309	21	2438	1579
miR156l-5p	LOC_Os01g59660.3	158	-25.49	158	-25.49	1001310	21	2418	1489
miR156l-5p	LOC_Os01g59660.4	158	-25.49	158	-25.49	1001311	21	2270	1411
miR156l-5p	LOC_Os01g59890.1	160	-24.84	160	-24.84	1001336	21	2476	1305
miR156l-5p	LOC_Os01g60430.1	147	-23.95	147	-23.95	1001399	21	2410	1135
miR156l-5p	LOC_Os01g60660.1	175	-21.9	175	-21.9	1001421	21	1541	946
miR156l-5p	LOC_Os01g60660.2	175	-21.9	175	-21.9	1001422	21	1337	742
miR156l-5p	LOC_Os01g63090.1	168	-21.67	168	-21.67	1001732	21	1560	733
miR156l-5p	LOC_Os01g63120.1	161	-24.44	161	-24.44	1001735	21	4308	368
miR156l-5p	LOC_Os01g63760.1	161	-21.79	161	-21.79	1001807	21	2214	2101
miR156l-5p	LOC_Os01g63910.1	157	-25.09	157	-25.09	1001826	21	3306	2468
miR156l-5p	LOC_Os01g63910.2	157	-25.09	157	-25.09	1001827	21	3312	2468
miR156l-5p	LOC_Os01g64560.1	150	-22.81	150	-22.81	1001914	21	3474	1097
miR156l-5p	LOC_Os01g64560.2	150	-22.81	150	-22.81	1001915	21	3471	1094
miR156l-5p	LOC_Os01g64620.1	145	-22.08	145	-22.08	1001921	21	935	124
miR156l-5p	LOC_Os01g65650.1	161	-20.41	161	-20.41	1002057	21	4110	180
miR156l-5p	LOC_Os01g65890.1	161	-24.62	161	-24.62	1002089	21	2496	1215
miR156l-5p	LOC_Os01g65920.1	165	-22.32	165	-22.32	1002093	21	2223	1580
miR156l-5p	LOC_Os01g67490.1	160	-21.64	160	-21.64	1002277	21	1572	959
miR156l-5p	LOC_Os01g67490.2	160	-21.64	160	-21.64	1002278	21	1473	860
miR156l-5p	LOC_Os01g67490.3	160	-21.64	160	-21.64	1002279	21	1614	1001
miR156l-5p	LOC_Os01g67580.1	159	-21.22	159	-21.22	1002292	21	4522	2843
miR156l-5p	LOC_Os01g67590.1	169	-24.53	169	-24.53	1002293	21	2594	1376
miR156l-5p	LOC_Os01g68740.1	145	-21.28	145	-21.28	1002431	21	1294	596
miR156l-5p	LOC_Os01g69830.1	187	-32.65	187	-32.65	1002510	21	1983	1152

miR156l-5p	LOC_Os01g72350.1	146	-20.91	146	-20.91	1002812	21	2418	1143
miR156l-5p	LOC_Os01g72350.2	146	-20.91	146	-20.91	1002813	21	2378	1143
miR156l-5p	LOC_Os01g72880.1	145	-20.32	145	-20.32	1002877	21	2623	818
miR156l-5p	LOC_Os01g74320.1	161	-24.98	161	-24.98	1003049	21	1745	1420
miR156l-5p	LOC_Os02g01640.1	156	-24.99	156	-24.99	1003176	21	4461	482
miR156l-5p	LOC_Os02g01670.1	156	-24.99	156	-24.99	1003178	21	4461	482
miR156l-5p	LOC_Os02g02050.1	152	-20.43	152	-20.43	1003227	21	8431	1645
miR156l-5p	LOC_Os02g02210.1	166	-20.1	166	-20.1	1003243	21	1653	943
miR156l-5p	LOC_Os02g02830.1	152	-22.97	152	-22.97	1003320	21	814	379
miR156l-5p	LOC_Os02g02830.2	152	-22.97	152	-22.97	1003321	21	941	506
miR156l-5p	LOC_Os02g02950.1	152	-23.41	152	-23.41	1003337	21	2366	1756
miR156l-5p	LOC_Os02g04040.1	167	-22.26	167	-22.26	1003488	21	1819	610
miR156l-5p	LOC_Os02g04680.1	187	-32.2	187	-32.2	1003574	21	2517	1964
miR156l-5p	LOC_Os02g04680.2	187	-32.2	187	-32.2	1003575	21	2514	1961
miR156l-5p	LOC_Os02g05220.1	159	-20.66	159	-20.66	1003644	21	4596	2890
miR156l-5p	LOC_Os02g05450.1	141	-20.92	141	-20.92	1003678	21	2750	1384
miR156l-5p	LOC_Os02g06600.1	178	-30.62	178	-30.62	1003815	21	2274	2223
miR156l-5p	LOC_Os02g06940.1	163	-26.56	163	-26.56	1003860	21	918	634
miR156l-5p	LOC_Os02g07260.3	167	-23.38	167	-23.38	1003909	21	1813	1190
miR156l-5p	LOC_Os02g07780.1	187	-31.91	187	-31.91	1003975	21	1668	986
miR156l-5p	LOC_Os02g07780.2	187	-31.91	187	-31.91	1003976	21	2902	2220
miR156l-5p	LOC_Os02g09740.1	151	-20.49	151	-20.49	1004150	21	2904	1563
miR156l-5p	LOC_Os02g09740.2	151	-20.49	151	-20.49	1004151	21	2901	1560
miR156l-5p	LOC_Os02g09960.1	169	-21.57	169	-21.57	1004180	21	2372	942
miR156l-5p	LOC_Os02g10460.1	154	-21.51	154	-21.51	1004243	21	1122	914
miR156l-5p	LOC_Os02g10640.1	152	-20.73	152	-20.73	1004266	21	1732	1657
miR156l-5p	LOC_Os02g11930.1	154	-21.3	154	-21.3	1004372	21	4582	585
miR156l-5p	LOC_Os02g12420.1	166	-21.87	166	-21.87	1004423	21	3234	450
miR156l-5p	LOC_Os02g15500.1	156	-25.25	156	-25.25	1004796	21	1394	953
miR156l-5p	LOC_Os02g18030.1	152	-21.53	152	-21.53	1005082	21	792	482
miR156l-5p	LOC_Os02g18064.1	157	-27.59	157	-27.59	1005086	21	4783	1000
miR156l-5p	LOC_Os02g18810.1	170	-23.48	170	-23.48	1005167	21	3783	2370

miR156l-5p	LOC_Os02g18990.1	157	-22.85	157	-22.85	1005186	21	1182	322
miR156l-5p	LOC_Os02g21150.1	140	-20.08	140	-20.08	1005415	21	2067	832
miR156l-5p	LOC_Os02g22090.1	144	-20.3	144	-20.3	1005517	21	4768	1394
miR156l-5p	LOC_Os02g22520.1	152	-21.24	152	-21.24	1005570	21	4488	482
miR156l-5p	LOC_Os02g23970.1	160	-24.11	160	-24.11	1005631	21	1728	1227
miR156l-5p	LOC_Os02g24090.1	327	-46.89	170	-25.29	1005647	21	4725	4242 3969
miR156l-5p	LOC_Os02g25180.1	148	-25.59	148	-25.59	1005762	21	4233	1308
miR156l-5p	LOC_Os02g27010.1	153	-21.6	153	-21.6	1005971	21	1245	191
miR156l-5p	LOC_Os02g27720.1	160	-20.75	160	-20.75	1006054	21	3957	3068
miR156l-5p	LOC_Os02g27840.1	168	-24.17	168	-24.17	1006066	21	921	165
miR156l-5p	LOC_Os02g29600.1	161	-21.96	161	-21.96	1006242	21	1104	522
miR156l-5p	LOC_Os02g30130.1	159	-22.06	159	-22.06	1006298	21	5964	4578
miR156l-5p	LOC_Os02g34690.1	163	-21.11	163	-21.11	1006753	21	2315	1444
miR156l-5p	LOC_Os02g34860.1	177	-27.71	177	-27.71	1006770	21	1757	5
miR156l-5p	LOC_Os02g36020.1	162	-24.12	162	-24.12	1006904	21	4830	4672
miR156l-5p	LOC_Os02g37109.1	157	-22.56	157	-22.56	1007043	21	1170	1136
miR156l-5p	LOC_Os02g37109.2	157	-22.56	157	-22.56	1007044	21	1105	1071
miR156l-5p	LOC_Os02g37654.1	153	-21.29	153	-21.29	1007102	21	1568	346
miR156l-5p	LOC_Os02g38370.1	164	-27.58	164	-27.58	1007197	21	2970	1406
miR156l-5p	LOC_Os02g39910.1	161	-23.64	161	-23.64	1007399	21	6191	5083
miR156l-5p	LOC_Os02g40360.1	164	-27.58	164	-27.58	1007460	21	3195	1748
miR156l-5p	LOC_Os02g41550.4	158	-24.34	158	-24.34	1007544	21	3428	2662
miR156l-5p	LOC_Os02g42134.2	150	-20.1	150	-20.1	1007611	21	3038	2819
miR156l-5p	LOC_Os02g42750.1	156	-24.99	156	-24.99	1007695	21	5295	482
miR156l-5p	LOC_Os02g45480.2	157	-20.22	157	-20.22	1008046	21	3510	2800
miR156l-5p	LOC_Os02g45480.3	157	-20.22	157	-20.22	1008047	21	3695	2985
miR156l-5p	LOC_Os02g46710.1	159	-21.17	159	-21.17	1008190	21	705	196
miR156l-5p	LOC_Os02g47170.1	159	-20.28	159	-20.28	1008251	21	3591	2046
miR156l-5p	LOC_Os02g47970.1	160	-20.51	160	-20.51	1008358	21	7433	626
miR156l-5p	LOC_Os02g48810.1	148	-20.4	148	-20.4	1008469	21	2505	2403
miR156l-5p	LOC_Os02g49210.1	152	-20.87	152	-20.87	1008513	21	879	70
miR156l-5p	LOC_Os02g49230.1	153	-20.96	153	-20.96	1008515	21	2666	2045

miR156l-5p	LOC_Os02g49230.2	153	-20.96	153	-20.96	1008516	21	2858	2237
miR156l-5p	LOC_Os02g49230.3	153	-20.96	153	-20.96	1008517	21	2750	2129
miR156l-5p	LOC_Os02g49230.4	153	-20.96	153	-20.96	1008518	21	2369	2255
miR156l-5p	LOC_Os02g49230.5	153	-20.96	153	-20.96	1008519	21	3011	2390
miR156l-5p	LOC_Os02g49230.6	153	-20.96	153	-20.96	1008520	21	2455	1834
miR156l-5p	LOC_Os02g49240.1	153	-20.96	153	-20.96	1008521	21	1732	605
miR156l-5p	LOC_Os02g49332.1	158	-22.87	158	-22.87	1008531	21	2673	2220
miR156l-5p	LOC_Os02g49332.2	158	-22.87	158	-22.87	1008532	21	4273	3820
miR156l-5p	LOC_Os02g49332.3	158	-22.87	158	-22.87	1008533	21	4042	3917
miR156l-5p	LOC_Os02g49332.4	158	-22.87	158	-22.87	1008534	21	2778	2325
miR156l-5p	LOC_Os02g50650.1	165	-25.04	165	-25.04	1008692	21	315	193
miR156l-5p	LOC_Os02g51164.1	157	-21.4	157	-21.4	1008758	21	1245	1045
miR156l-5p	LOC_Os02g51240.1	159	-20.66	159	-20.66	1008766	21	4332	3682
miR156l-5p	LOC_Os02g51780.1	158	-22.62	158	-22.62	1008838	21	3901	374
miR156l-5p	LOC_Os02g53070.1	151	-22.25	151	-22.25	1008999	21	2337	656
miR156l-5p	LOC_Os02g53120.1	159	-21.64	159	-21.64	1009006	21	3726	370
miR156l-5p	LOC_Os02g54500.1	170	-21.88	170	-21.88	1009184	21	3894	3249
miR156l-5p	LOC_Os02g55970.1	164	-21.74	164	-21.74	1009362	21	2929	15
miR156l-5p	LOC_Os02g56540.1	165	-21.23	165	-21.23	1009432	21	2970	2785
miR156l-5p	LOC_Os02g58070.1	154	-20.47	154	-20.47	1009654	21	1550	1444
miR156l-5p	LOC_Os03g01720.1	165	-21.81	165	-21.81	1009842	21	2874	2010
miR156l-5p	LOC_Os03g01830.1	162	-23.47	162	-23.47	1009864	21	2399	582
miR156l-5p	LOC_Os03g02120.1	143	-20.57	143	-20.57	1009909	21	2484	900
miR156l-5p	LOC_Os03g02150.1	164	-25.75	164	-25.75	1009912	21	3083	1582
miR156l-5p	LOC_Os03g02150.2	164	-25.75	164	-25.75	1009913	21	3870	1582
miR156l-5p	LOC_Os03g02530.1	165	-22.07	165	-22.07	1009965	21	1370	1295
miR156l-5p	LOC_Os03g03320.1	165	-22.46	165	-22.46	1010067	21	1090	40
miR156l-5p	LOC_Os03g03500.1	166	-20.34	166	-20.34	1010093	21	1621	69
miR156l-5p	LOC_Os03g03700.1	157	-21.31	157	-21.31	1010121	21	2498	2423
miR156l-5p	LOC_Os03g03700.2	157	-21.31	157	-21.31	1010122	21	2647	2572
miR156l-5p	LOC_Os03g03720.1	174	-22.91	174	-22.91	1010124	21	1885	1784
miR156l-5p	LOC_Os03g03720.2	174	-22.91	174	-22.91	1010125	21	1895	1794

miR156l-5p	LOC_Os03g04100.1	151	-21.9	151	-21.9	1010168	21	1950	1500
miR156l-5p	LOC_Os03g04100.2	151	-21.9	151	-21.9	1010169	21	2133	1692
miR156l-5p	LOC_Os03g04480.1	154	-21.26	154	-21.26	1010212	21	1763	693
miR156l-5p	LOC_Os03g05160.1	149	-20.98	149	-20.98	1010294	21	1010	362
miR156l-5p	LOC_Os03g05820.1	158	-20.11	158	-20.11	1010387	21	3107	2302
miR156l-5p	LOC_Os03g05850.1	161	-22.46	161	-22.46	1010390	21	4230	1038
miR156l-5p	LOC_Os03g06860.1	169	-23.02	169	-23.02	1010512	21	2327	485
miR156l-5p	LOC_Os03g07580.1	326	-45.4	170	-24.92	1010608	21	4354	2886 4088
miR156l-5p	LOC_Os03g08350.1	161	-22.46	161	-22.46	1010702	21	1915	1160
miR156l-5p	LOC_Os03g08370.1	161	-22.05	161	-22.05	1010706	21	3109	368
miR156l-5p	LOC_Os03g08770.1	168	-23.83	168	-23.83	1010762	21	1226	958
miR156l-5p	LOC_Os03g08770.2	168	-23.83	168	-23.83	1010763	21	1223	955
miR156l-5p	LOC_Os03g10830.1	155	-22.83	155	-22.83	1010955	21	234	199
miR156l-5p	LOC_Os03g10930.1	157	-22.92	157	-22.92	1010967	21	875	337
miR156l-5p	LOC_Os03g10930.2	157	-22.92	157	-22.92	1010968	21	918	337
miR156l-5p	LOC_Os03g11980.1	166	-24.04	166	-24.04	1011095	21	2434	465
miR156l-5p	LOC_Os03g13460.1	163	-21.6	163	-21.6	1011297	21	3291	1967
miR156l-5p	LOC_Os03g13460.2	163	-21.6	163	-21.6	1011298	21	3715	2391
miR156l-5p	LOC_Os03g15620.1	159	-22.06	159	-22.06	1011558	21	5757	4377
miR156l-5p	LOC_Os03g16030.1	153	-20.93	153	-20.93	1011620	21	980	877
miR156l-5p	LOC_Os03g16690.1	174	-22.39	174	-22.39	1011713	21	2033	2013
miR156l-5p	LOC_Os03g17570.1	166	-21.41	166	-21.41	1011826	21	3610	743
miR156l-5p	LOC_Os03g17570.2	166	-21.41	166	-21.41	1011827	21	3596	743
miR156l-5p	LOC_Os03g17570.3	166	-21.41	166	-21.41	1011828	21	3591	743
miR156l-5p	LOC_Os03g17570.4	166	-21.41	166	-21.41	1011829	21	4061	1194
miR156l-5p	LOC_Os03g17570.5	166	-21.41	166	-21.41	1011830	21	3497	743
miR156l-5p	LOC_Os03g17570.6	166	-21.41	166	-21.41	1011831	21	3691	743
miR156l-5p	LOC_Os03g18060.1	152	-23.21	152	-23.21	1011888	21	5067	4510
miR156l-5p	LOC_Os03g18980.1	170	-21.54	170	-21.54	1011994	21	3045	628
miR156l-5p	LOC_Os03g19920.1	140	-20.47	140	-20.47	1012108	21	2332	631
miR156l-5p	LOC_Os03g20500.1	166	-23.1	166	-23.1	1012185	21	1679	1460
miR156l-5p	LOC_Os03g21100.1	165	-23.57	165	-23.57	1012253	21	2424	1269

miR156l-5p	LOC_Os03g23080.1	150	-22.05	150	-22.05	1012504	21	1839	522
miR156l-5p	LOC_Os03g23110.1	170	-24.22	170	-24.22	1012507	21	4929	1728
miR156l-5p	LOC_Os03g23200.1	156	-24.99	156	-24.99	1012515	21	5175	362
miR156l-5p	LOC_Os03g24910.1	157	-22.65	157	-22.65	1012631	21	1372	375
miR156l-5p	LOC_Os03g27250.1	169	-21.06	169	-21.06	1012876	21	3297	1508
miR156l-5p	LOC_Os03g27670.1	165	-21.22	165	-21.22	1012929	21	1729	1437
miR156l-5p	LOC_Os03g29520.1	154	-21.05	154	-21.05	1013078	21	5769	326
miR156l-5p	LOC_Os03g36290.1	154	-20.93	154	-20.93	1013637	21	3363	102
miR156l-5p	LOC_Os03g36330.1	157	-26.15	157	-26.15	1013641	21	2286	478
miR156l-5p	LOC_Os03g38970.1	167	-22.24	167	-22.24	1013900	21	4644	1492
miR156l-5p	LOC_Os03g38980.1	163	-26.03	163	-26.03	1013901	21	2319	1487
miR156l-5p	LOC_Os03g38980.2	163	-26.03	163	-26.03	1013902	21	2298	1466
miR156l-5p	LOC_Os03g38980.3	163	-26.03	163	-26.03	1013903	21	2273	1421
miR156l-5p	LOC_Os03g39250.1	157	-22.25	157	-22.25	1013927	21	3717	443
miR156l-5p	LOC_Os03g39330.1	153	-22.25	153	-22.25	1013932	21	3603	334
miR156l-5p	LOC_Os03g39432.1	170	-21.17	170	-21.17	1013943	21	2028	267
miR156l-5p	LOC_Os03g39450.1	148	-25.59	148	-25.59	1013945	21	3390	1296
miR156l-5p	LOC_Os03g40160.1	165	-20.34	165	-20.34	1014012	21	3226	2119
miR156l-5p	LOC_Os03g43250.1	161	-24.95	161	-24.95	1014305	21	3273	482
miR156l-5p	LOC_Os03g44130.1	160	-20.39	160	-20.39	1014397	21	1483	931
miR156l-5p	LOC_Os03g44240.1	152	-20.66	152	-20.66	1014410	21	3066	206
miR156l-5p	LOC_Os03g44590.1	166	-22	166	-22	1014452	21	2952	1768
miR156l-5p	LOC_Os03g45440.1	157	-21.91	157	-21.91	1014545	21	480	300
miR156l-5p	LOC_Os03g46080.1	152	-23.21	152	-23.21	1014603	21	3299	2626
miR156l-5p	LOC_Os03g47130.1	152	-23.21	152	-23.21	1014719	21	3867	3358
miR156l-5p	LOC_Os03g47140.1	164	-28.84	164	-28.84	1014720	21	1975	1006
miR156l-5p	LOC_Os03g47610.1	149	-21.35	149	-21.35	1014768	21	2233	2155
miR156l-5p	LOC_Os03g47610.8	149	-21.35	149	-21.35	1014774	21	3682	2137
miR156l-5p	LOC_Os03g47760.1	167	-24.31	167	-24.31	1014790	21	3858	799
miR156l-5p	LOC_Os03g47930.1	166	-20.71	166	-20.71	1014811	21	4005	2032
miR156l-5p	LOC_Os03g48940.1	164	-20.31	164	-20.31	1014929	21	2604	530
miR156l-5p	LOC_Os03g48940.2	164	-20.31	164	-20.31	1014930	21	2668	432

miR156l-5p	LOC_Os03g48940.3	323	-41.11	164	-20.8	1014931	21	1985	747 284
miR156l-5p	LOC_Os03g51140.1	167	-20.69	167	-20.69	1015192	21	4098	518
miR156l-5p	LOC_Os03g52070.1	166	-25.44	166	-25.44	1015298	21	1938	191
miR156l-5p	LOC_Os03g52070.2	166	-25.44	166	-25.44	1015299	21	2970	109
miR156l-5p	LOC_Os03g52070.3	166	-25.44	166	-25.44	1015300	21	1828	191
miR156l-5p	LOC_Os03g52470.1	152	-21.75	152	-21.75	1015344	21	2986	628
miR156l-5p	LOC_Os03g52780.1	163	-21.22	163	-21.22	1015383	21	1886	1137
miR156l-5p	LOC_Os03g53250.1	160	-20.97	160	-20.97	1015435	21	3176	3020
miR156l-5p	LOC_Os03g53640.1	163	-20.5	163	-20.5	1015481	21	980	58
miR156l-5p	LOC_Os03g53860.3	158	-22.09	158	-22.09	1015514	21	2809	294
miR156l-5p	LOC_Os03g53860.5	158	-22.09	158	-22.09	1015516	21	2904	294
miR156l-5p	LOC_Os03g53990.1	171	-24.22	171	-24.22	1015535	21	3957	3887
miR156l-5p	LOC_Os03g54000.1	171	-24.22	171	-24.22	1015536	21	2910	99
miR156l-5p	LOC_Os03g54000.2	171	-24.22	171	-24.22	1015537	21	3003	99
miR156l-5p	LOC_Os03g55090.1	165	-24.66	165	-24.66	1015607	21	2855	2450
miR156l-5p	LOC_Os03g57460.1	161	-21.34	161	-21.34	1015919	21	2062	80
miR156l-5p	LOC_Os03g58160.1	158	-23.77	158	-23.77	1016008	21	2065	281
miR156l-5p	LOC_Os03g58160.2	158	-23.77	158	-23.77	1016009	21	1979	269
miR156l-5p	LOC_Os03g63710.1	145	-20.61	145	-20.61	1016678	21	2249	475
miR156l-5p	LOC_Os03g63710.2	145	-20.61	145	-20.61	1016679	21	2200	475
miR156l-5p	LOC_Os03g64250.5	166	-20.43	166	-20.43	1016747	21	2249	767
miR156l-5p	LOC_Os04g01030.1	156	-24.26	156	-24.26	1016776	21	4293	404
miR156l-5p	LOC_Os04g02140.1	166	-20.17	166	-20.17	1016903	21	1958	952
miR156l-5p	LOC_Os04g02920.1	155	-21.16	155	-21.16	1016991	21	2552	968
miR156l-5p	LOC_Os04g03040.1	161	-21.79	161	-21.79	1017002	21	3288	3178
miR156l-5p	LOC_Os04g03190.1	151	-20.31	151	-20.31	1017013	21	3954	1000
miR156l-5p	LOC_Os04g03490.1	153	-22.6	153	-22.6	1017043	21	4005	541
miR156l-5p	LOC_Os04g05260.1	154	-20.2	154	-20.2	1017210	21	597	494
miR156l-5p	LOC_Os04g05310.1	154	-21.24	154	-21.24	1017215	21	2100	612
miR156l-5p	LOC_Os04g05450.1	168	-23.35	168	-23.35	1017226	21	1305	84
miR156l-5p	LOC_Os04g05610.1	161	-21.79	161	-21.79	1017242	21	3117	3022
miR156l-5p	LOC_Os04g06144.1	156	-23.94	156	-23.94	1017290	21	6192	3215

miR156l-5p	LOC_Os04g06500.1	158	-23.84	158	-23.84	1017329	21	2330	1966
miR156l-5p	LOC_Os04g06820.1	161	-23.51	161	-23.51	1017354	21	1887	1730
miR156l-5p	LOC_Os04g08230.1	160	-24.11	160	-24.11	1017487	21	702	498
miR156l-5p	LOC_Os04g08450.1	156	-20.14	156	-20.14	1017510	21	1049	257
miR156l-5p	LOC_Os04g08450.2	156	-20.14	156	-20.14	1017511	21	1195	257
miR156l-5p	LOC_Os04g08450.3	156	-20.14	156	-20.14	1017512	21	1198	257
miR156l-5p	LOC_Os04g08824.1	158	-26.1	158	-26.1	1017559	21	1593	101
miR156l-5p	LOC_Os04g08828.1	167	-22.48	167	-22.48	1017560	21	3346	103
miR156l-5p	LOC_Os04g09284.1	153	-21.21	153	-21.21	1017566	21	4470	4244
miR156l-5p	LOC_Os04g09480.1	156	-24.99	156	-24.99	1017585	21	4419	482
miR156l-5p	LOC_Os04g10120.1	159	-21.21	159	-21.21	1017646	21	1302	179
miR156l-5p	LOC_Os04g10380.1	162	-21.27	162	-21.27	1017674	21	5131	2536
miR156l-5p	LOC_Os04g11560.1	160	-24.11	160	-24.11	1017787	21	1053	633
miR156l-5p	LOC_Os04g13620.1	161	-21.79	161	-21.79	1017978	21	3237	3127
miR156l-5p	LOC_Os04g13900.1	155	-22.74	155	-22.74	1018007	21	2226	481
miR156l-5p	LOC_Os04g14600.1	152	-22.31	152	-22.31	1018079	21	1404	640
miR156l-5p	LOC_Os04g14890.1	168	-21.29	168	-21.29	1018107	21	4494	621
miR156l-5p	LOC_Os04g16020.1	150	-21.18	150	-21.18	1018166	21	528	269
miR156l-5p	LOC_Os04g16150.1	162	-22.47	162	-22.47	1018179	21	3603	1869
miR156l-5p	LOC_Os04g16220.1	154	-20.14	154	-20.14	1018185	21	864	155
miR156l-5p	LOC_Os04g16400.1	159	-21.69	159	-21.69	1018202	21	3387	2275
miR156l-5p	LOC_Os04g17410.1	156	-24.99	156	-24.99	1018337	21	4368	482
miR156l-5p	LOC_Os04g17440.1	155	-20.76	155	-20.76	1018340	21	6093	4707
miR156l-5p	LOC_Os04g20510.1	164	-25.28	164	-25.28	1018643	21	705	235
miR156l-5p	LOC_Os04g20810.1	156	-21.45	156	-21.45	1018670	21	1359	1162
miR156l-5p	LOC_Os04g20810.2	156	-21.45	156	-21.45	1018671	21	1418	1221
miR156l-5p	LOC_Os04g20810.3	156	-21.45	156	-21.45	1018672	21	1240	1043
miR156l-5p	LOC_Os04g21110.1	166	-23.06	166	-23.06	1018697	21	3403	612
miR156l-5p	LOC_Os04g21700.1	161	-21.79	161	-21.79	1018754	21	1218	919
miR156l-5p	LOC_Os04g22490.1	156	-24.99	156	-24.99	1018834	21	3477	482
miR156l-5p	LOC_Os04g23270.1	166	-20.74	166	-20.74	1018905	21	2016	727
miR156l-5p	LOC_Os04g23470.1	152	-23.21	152	-23.21	1018923	21	4836	4171

miR156l-5p	LOC_Os04g23580.1	163	-20.81	163	-20.81	1018932	21	2110	1287
miR156l-5p	LOC_Os04g24060.1	156	-24.99	156	-24.99	1018972	21	5484	482
miR156l-5p	LOC_Os04g25360.2	166	-23.72	166	-23.72	1019107	21	1518	1131
miR156l-5p	LOC_Os04g25540.1	173	-24.07	173	-24.07	1019123	21	703	279
miR156l-5p	LOC_Os04g25730.1	165	-21.22	165	-21.22	1019141	21	2851	2559
miR156l-5p	LOC_Os04g26570.1	153	-20.01	153	-20.01	1019233	21	2676	952
miR156l-5p	LOC_Os04g26690.1	161	-21.79	161	-21.79	1019245	21	2967	2692
miR156l-5p	LOC_Os04g27670.1	161	-27.78	161	-27.78	1019337	21	2147	746
miR156l-5p	LOC_Os04g27720.1	153	-24.49	153	-24.49	1019342	21	1518	366
miR156l-5p	LOC_Os04g28130.1	159	-20.68	159	-20.68	1019379	21	1398	140
miR156l-5p	LOC_Os04g29980.1	165	-21.22	165	-21.22	1019550	21	934	642
miR156l-5p	LOC_Os04g30230.1	159	-20.03	159	-20.03	1019574	21	909	374
miR156l-5p	LOC_Os04g30850.1	150	-25.84	150	-25.84	1019639	21	555	219
miR156l-5p	LOC_Os04g31860.1	159	-22.06	159	-22.06	1019742	21	5337	4284
miR156l-5p	LOC_Os04g32650.1	163	-21.83	163	-21.83	1019829	21	2496	1532
miR156l-5p	LOC_Os04g32670.1	166	-21.07	166	-21.07	1019831	21	2826	1087
miR156l-5p	LOC_Os04g32770.1	324	-42.64	164	-22.11	1019841	21	4638	972 868
miR156l-5p	LOC_Os04g34440.1	164	-21.15	164	-21.15	1020033	21	2301	1595
miR156l-5p	LOC_Os04g35080.1	164	-20.03	164	-20.03	1020100	21	2625	330
miR156l-5p	LOC_Os04g35880.1	165	-21.57	165	-21.57	1020196	21	1930	384
miR156l-5p	LOC_Os04g37450.1	161	-25.85	161	-25.85	1020264	21	796	310
miR156l-5p	LOC_Os04g37840.1	156	-23.48	156	-23.48	1020307	21	4551	482
miR156l-5p	LOC_Os04g38140.1	159	-22.06	159	-22.06	1020337	21	1389	1041
miR156l-5p	LOC_Os04g38730.1	157	-21.51	157	-21.51	1020408	21	633	457
miR156l-5p	LOC_Os04g38770.1	168	-22.9	168	-22.9	1020412	21	527	146
miR156l-5p	LOC_Os04g39560.1	167	-26.33	167	-26.33	1020516	21	4682	3488
miR156l-5p	LOC_Os04g39560.2	167	-26.33	167	-26.33	1020517	21	4873	3488
miR156l-5p	LOC_Os04g39560.3	167	-26.33	167	-26.33	1020518	21	4591	3489
miR156l-5p	LOC_Os04g40440.1	154	-20.28	154	-20.28	1020617	21	3483	2965
miR156l-5p	LOC_Os04g40450.1	158	-21.63	158	-21.63	1020618	21	3309	2010
miR156l-5p	LOC_Os04g41110.1	154	-25.94	154	-25.94	1020713	21	2786	250
miR156l-5p	LOC_Os04g41110.2	154	-25.94	154	-25.94	1020714	21	2783	250

miR156l-5p	LOC_Os04g42451.1	146	-22.14	146	-22.14	1020864	21	1254	856
miR156l-5p	LOC_Os04g42740.1	158	-21.04	158	-21.04	1020900	21	2382	1356
miR156l-5p	LOC_Os04g42770.1	154	-21.63	154	-21.63	1020903	21	4002	2700
miR156l-5p	LOC_Os04g42770.2	154	-21.63	154	-21.63	1020904	21	3631	2700
miR156l-5p	LOC_Os04g42770.3	154	-21.63	154	-21.63	1020905	21	3492	2700
miR156l-5p	LOC_Os04g42770.4	154	-21.63	154	-21.63	1020906	21	3588	2700
miR156l-5p	LOC_Os04g42770.5	154	-21.63	154	-21.63	1020907	21	3628	2700
miR156l-5p	LOC_Os04g42770.6	154	-21.63	154	-21.63	1020908	21	3645	2700
miR156l-5p	LOC_Os04g45190.3	164	-21.67	164	-21.67	1021207	21	1501	574
miR156l-5p	LOC_Os04g45240.1	152	-20.66	152	-20.66	1021213	21	1385	681
miR156l-5p	LOC_Os04g45420.1	160	-20.83	160	-20.83	1021234	21	7926	5339
miR156l-5p	LOC_Os04g45990.1	158	-24.24	158	-24.24	1021296	21	2714	1474
miR156l-5p	LOC_Os04g46050.1	158	-21.21	158	-21.21	1021303	21	2081	1102
miR156l-5p	LOC_Os04g46050.2	158	-21.21	158	-21.21	1021304	21	2145	1102
miR156l-5p	LOC_Os04g46050.3	158	-21.21	158	-21.21	1021305	21	2136	1102
miR156l-5p	LOC_Os04g46580.1	187	-32.31	187	-32.31	1021369	21	1518	817
miR156l-5p	LOC_Os04g46690.1	162	-22.47	162	-22.47	1021380	21	2700	550
miR156l-5p	LOC_Os04g46830.1	150	-21.69	150	-21.69	1021402	21	1165	627
miR156l-5p	LOC_Os04g46830.2	150	-21.69	150	-21.69	1021403	21	1047	509
miR156l-5p	LOC_Os04g46894.1	161	-20.86	161	-20.86	1021411	21	6662	1224
miR156l-5p	LOC_Os04g46940.1	150	-25.12	150	-25.12	1021417	21	3310	628
miR156l-5p	LOC_Os04g47700.1	164	-20.36	164	-20.36	1021510	21	2619	607
miR156l-5p	LOC_Os04g48094.1	156	-24.99	156	-24.99	1021561	21	9963	5360
miR156l-5p	LOC_Os04g49090.1	161	-21.79	161	-21.79	1021675	21	3288	3178
miR156l-5p	LOC_Os04g50000.1	157	-21.04	157	-21.04	1021777	21	3618	369
miR156l-5p	LOC_Os04g51060.1	159	-20.29	159	-20.29	1021854	21	3669	2018
miR156l-5p	LOC_Os04g51370.1	162	-20.81	162	-20.81	1021893	21	3627	1681
miR156l-5p	LOC_Os04g51370.2	162	-20.81	162	-20.81	1021894	21	3487	1664
miR156l-5p	LOC_Os04g51540.1	159	-22.06	159	-22.06	1021919	21	5928	4542
miR156l-5p	LOC_Os04g51720.1	164	-27.58	164	-27.58	1021940	21	3114	1697
miR156l-5p	LOC_Os04g51740.1	156	-24.99	156	-24.99	1021942	21	5280	482
miR156l-5p	LOC_Os04g52200.1	145	-23.32	145	-23.32	1022010	21	3138	1299

miR156l-5p	LOC_Os04g53300.1	170	-21.72	170	-21.72	1022150	21	2582	2323
miR156l-5p	LOC_Os04g53310.1	170	-21.72	170	-21.72	1022151	21	5084	1061
miR156l-5p	LOC_Os04g53320.1	169	-22.52	169	-22.52	1022152	21	797	2
miR156l-5p	LOC_Os04g54002.1	175	-29.04	175	-29.04	1022244	21	3002	556
miR156l-5p	LOC_Os04g54650.1	156	-24.99	156	-24.99	1022319	21	4881	482
miR156l-5p	LOC_Os04g54759.1	162	-23.39	162	-23.39	1022331	21	1280	941
miR156l-5p	LOC_Os04g54820.2	177	-29.56	177	-29.56	1022339	21	2033	406
miR156l-5p	LOC_Os04g56290.1	153	-20.97	153	-20.97	1022507	21	2214	2143
miR156l-5p	LOC_Os04g56290.2	153	-20.97	153	-20.97	1022508	21	2642	2571
miR156l-5p	LOC_Os04g56290.6	153	-20.97	153	-20.97	1022510	21	3880	3809
miR156l-5p	LOC_Os04g56910.1	156	-20.98	156	-20.98	1022586	21	2006	1203
miR156l-5p	LOC_Os04g57770.1	149	-20.71	149	-20.71	1022712	21	1753	1360
miR156l-5p	LOC_Os04g58550.1	163	-22.85	163	-22.85	1022805	21	1568	962
miR156l-5p	LOC_Os04g59460.1	165	-21.83	165	-21.83	1022919	21	5636	5176
miR156l-5p	LOC_Os04g59540.1	167	-20.96	167	-20.96	1022928	21	4528	3445
miR156l-5p	LOC_Os05g01006.1	153	-20.54	153	-20.54	1022958	21	1839	1150
miR156l-5p	LOC_Os05g01150.1	161	-21.79	161	-21.79	1022984	21	837	532
miR156l-5p	LOC_Os05g01180.1	158	-20.4	158	-20.4	1022987	21	2351	1485
miR156l-5p	LOC_Os05g01630.1	170	-20.6	170	-20.6	1023054	21	2160	641
miR156l-5p	LOC_Os05g01630.2	170	-20.6	170	-20.6	1023055	21	1090	502
miR156l-5p	LOC_Os05g01630.3	170	-20.6	170	-20.6	1023056	21	1256	668
miR156l-5p	LOC_Os05g02564.1	166	-20.75	166	-20.75	1023176	21	2309	980
miR156l-5p	LOC_Os05g03190.1	169	-20.09	169	-20.09	1023249	21	2329	69
miR156l-5p	LOC_Os05g04180.1	156	-22.59	156	-22.59	1023363	21	1385	219
miR156l-5p	LOC_Os05g04430.1	154	-21.24	154	-21.24	1023391	21	2481	918
miR156l-5p	LOC_Os05g05170.1	175	-27.13	175	-27.13	1023470	21	873	571
miR156l-5p	LOC_Os05g05300.1	148	-20.11	148	-20.11	1023486	21	3778	3473
miR156l-5p	LOC_Os05g05310.1	148	-20.11	148	-20.11	1023487	21	3863	2098
miR156l-5p	LOC_Os05g05310.2	148	-20.11	148	-20.11	1023488	21	3859	2098
miR156l-5p	LOC_Os05g05820.1	165	-21.32	165	-21.32	1023552	21	1454	95
miR156l-5p	LOC_Os05g05890.1	156	-24.99	156	-24.99	1023560	21	3429	482
miR156l-5p	LOC_Os05g06280.1	162	-23.99	162	-23.99	1023601	21	3584	3117

miR156l-5p	LOC_Os05g06290.1	157	-21.93	157	-21.93	1023604	21	929	556
miR156l-5p	LOC_Os05g06630.1	155	-22.92	155	-22.92	1023644	21	2462	1551
miR156l-5p	LOC_Os05g07500.1	156	-23.94	156	-23.94	1023749	21	5334	482
miR156l-5p	LOC_Os05g07520.1	156	-23.94	156	-23.94	1023750	21	5298	482
miR156l-5p	LOC_Os05g08320.1	168	-21.65	168	-21.65	1023843	21	1041	470
miR156l-5p	LOC_Os05g09640.1	158	-23.4	158	-23.4	1024006	21	1209	18
miR156l-5p	LOC_Os05g09704.1	157	-23.79	157	-23.79	1024012	21	1089	387
miR156l-5p	LOC_Os05g10470.1	165	-21.22	165	-21.22	1024048	21	991	699
miR156l-5p	LOC_Os05g10730.1	169	-20.22	169	-20.22	1024079	21	6041	4922
miR156l-5p	LOC_Os05g10730.2	169	-20.22	169	-20.22	1024080	21	6094	4975
miR156l-5p	LOC_Os05g10730.3	169	-20.22	169	-20.22	1024081	21	6127	5008
miR156l-5p	LOC_Os05g10740.1	169	-20.22	169	-20.22	1024082	21	1845	507
miR156l-5p	LOC_Os05g10740.2	169	-20.22	169	-20.22	1024083	21	1945	507
miR156l-5p	LOC_Os05g10770.1	147	-21.63	147	-21.63	1024088	21	4353	2437
miR156l-5p	LOC_Os05g11080.1	165	-21.22	165	-21.22	1024122	21	4310	4158
miR156l-5p	LOC_Os05g11110.1	155	-20.04	155	-20.04	1024126	21	3999	170
miR156l-5p	LOC_Os05g11850.1	169	-20.82	169	-20.82	1024201	21	2774	2154
miR156l-5p	LOC_Os05g14580.1	155	-21.67	155	-21.67	1024444	21	552	220
miR156l-5p	LOC_Os05g15040.1	142	-23.49	142	-23.49	1024487	21	4738	2842
miR156l-5p	LOC_Os05g15040.2	142	-23.49	142	-23.49	1024488	21	4631	2828
miR156l-5p	LOC_Os05g15040.3	142	-23.49	142	-23.49	1024489	21	3908	2012
miR156l-5p	LOC_Os05g15040.4	142	-23.49	142	-23.49	1024490	21	4109	2306
miR156l-5p	LOC_Os05g16390.1	153	-23.45	153	-23.45	1024629	21	4785	478
miR156l-5p	LOC_Os05g16590.1	166	-24.94	166	-24.94	1024650	21	1086	981
miR156l-5p	LOC_Os05g17180.1	164	-27.58	164	-27.58	1024712	21	3054	1637
miR156l-5p	LOC_Os05g17460.1	168	-21.67	168	-21.67	1024740	21	2751	766
miR156l-5p	LOC_Os05g19530.1	157	-21.74	157	-21.74	1024960	21	2883	482
miR156l-5p	LOC_Os05g22340.1	154	-20.14	154	-20.14	1025150	21	1983	851
miR156l-5p	LOC_Os05g22410.1	159	-21.03	159	-21.03	1025159	21	2775	125
miR156l-5p	LOC_Os05g23010.1	156	-24.99	156	-24.99	1025223	21	4416	482
miR156l-5p	LOC_Os05g23320.1	158	-21.68	158	-21.68	1025257	21	2616	2221
miR156l-5p	LOC_Os05g23320.2	158	-21.68	158	-21.68	1025258	21	3066	2671

miR156l-5p	LOC_Os05g24010.1	167	-21.01	167	-21.01	1025336	21	3464	1375
miR156l-5p	LOC_Os05g24010.2	167	-21.01	167	-21.01	1025337	21	3444	1365
miR156l-5p	LOC_Os05g24010.3	167	-21.01	167	-21.01	1025338	21	2667	588
miR156l-5p	LOC_Os05g25470.1	152	-23.21	152	-23.21	1025496	21	4704	3976
miR156l-5p	LOC_Os05g25750.1	140	-20.08	140	-20.08	1025522	21	3558	2764
miR156l-5p	LOC_Os05g26820.1	162	-20.11	162	-20.11	1025637	21	2111	1980
miR156l-5p	LOC_Os05g27340.1	161	-21.4	161	-21.4	1025684	21	827	782
miR156l-5p	LOC_Os05g28220.1	167	-21.09	167	-21.09	1025783	21	675	243
miR156l-5p	LOC_Os05g31160.1	157	-21.48	157	-21.48	1026069	21	1523	853
miR156l-5p	LOC_Os05g31160.2	157	-21.48	157	-21.48	1026070	21	1546	869
miR156l-5p	LOC_Os05g32110.1	165	-23.09	165	-23.09	1026164	21	2167	2131
miR156l-5p	LOC_Os05g32110.4	165	-23.09	165	-23.09	1026167	21	2287	2251
miR156l-5p	LOC_Os05g34140.1	154	-21.87	154	-21.87	1026397	21	2355	216
miR156l-5p	LOC_Os05g34760.1	171	-21.34	171	-21.34	1026472	21	3142	2857
miR156l-5p	LOC_Os05g34760.2	171	-21.34	171	-21.34	1026473	21	3677	3392
miR156l-5p	LOC_Os05g35100.1	165	-21.22	165	-21.22	1026512	21	2830	2538
miR156l-5p	LOC_Os05g35730.1	152	-21.68	152	-21.68	1026585	21	2022	1312
miR156l-5p	LOC_Os05g35730.2	152	-21.68	152	-21.68	1026586	21	1530	820
miR156l-5p	LOC_Os05g37230.1	156	-23.48	156	-23.48	1026717	21	4110	482
miR156l-5p	LOC_Os05g39070.1	153	-21.87	153	-21.87	1026945	21	1132	791
miR156l-5p	LOC_Os05g39070.2	153	-21.87	153	-21.87	1026946	21	1646	815
miR156l-5p	LOC_Os05g39070.3	153	-21.87	153	-21.87	1026947	21	1981	815
miR156l-5p	LOC_Os05g39070.4	153	-21.87	153	-21.87	1026948	21	2069	815
miR156l-5p	LOC_Os05g39840.1	159	-22.31	159	-22.31	1027036	21	2074	1111
miR156l-5p	LOC_Os05g40150.1	145	-20.57	145	-20.57	1027070	21	3675	2720
miR156l-5p	LOC_Os05g40320.1	161	-20.12	161	-20.12	1027102	21	3091	2493
miR156l-5p	LOC_Os05g40520.1	140	-20.2	140	-20.2	1027123	21	1212	165
miR156l-5p	LOC_Os05g40770.1	164	-27.57	164	-27.57	1027150	21	3359	1356
miR156l-5p	LOC_Os05g40770.2	164	-27.57	164	-27.57	1027151	21	3031	1356
miR156l-5p	LOC_Os05g41040.1	166	-22	166	-22	1027182	21	2964	1741
miR156l-5p	LOC_Os05g41700.1	147	-22.69	147	-22.69	1027260	21	5205	4790
miR156l-5p	LOC_Os05g44120.1	164	-22.03	164	-22.03	1027481	21	1208	1130

miR156l-5p	LOC_Os05g44340.1	171	-27.54	171	-27.54	1027515	21	3264	3194
miR156l-5p	LOC_Os05g46030.1	156	-22.66	156	-22.66	1027718	21	6999	6832
miR156l-5p	LOC_Os05g47580.1	168	-29.4	168	-29.4	1027857	21	1351	135
miR156l-5p	LOC_Os05g48200.1	163	-23.06	163	-23.06	1027925	21	6926	5207
miR156l-5p	LOC_Os05g48340.1	159	-25.48	159	-25.48	1027943	21	2152	1386
miR156l-5p	LOC_Os05g48340.2	159	-25.48	159	-25.48	1027944	21	2241	1386
miR156l-5p	LOC_Os05g48340.3	159	-25.48	159	-25.48	1027945	21	2387	1386
miR156l-5p	LOC_Os05g48550.1	161	-21.79	161	-21.79	1027976	21	2916	2803
miR156l-5p	LOC_Os05g51290.1	165	-21.22	165	-21.22	1028333	21	2911	2619
miR156l-5p	LOC_Os05g51770.1	157	-22.25	157	-22.25	1028405	21	3762	482
miR156l-5p	LOC_Os05g52070.1	156	-23.48	156	-23.48	1028432	21	5016	479
miR156l-5p	LOC_Os06g02180.1	166	-20.11	166	-20.11	1028571	21	4214	2366
miR156l-5p	LOC_Os06g02630.1	162	-20.97	162	-20.97	1028627	21	1877	1297
miR156l-5p	LOC_Os06g02700.1	162	-22.79	162	-22.79	1028634	21	1011	2
miR156l-5p	LOC_Os06g03930.1	149	-22.62	149	-22.62	1028769	21	1997	1691
miR156l-5p	LOC_Os06g03930.2	149	-22.62	149	-22.62	1028770	21	1646	1340
miR156l-5p	LOC_Os06g04980.1	169	-27.11	169	-27.11	1028895	21	1539	532
miR156l-5p	LOC_Os06g06520.1	153	-20.73	153	-20.73	1029104	21	1484	382
miR156l-5p	LOC_Os06g06810.1	164	-25.1	164	-25.1	1029137	21	2890	2747
miR156l-5p	LOC_Os06g08070.1	152	-23.21	152	-23.21	1029273	21	4011	3469
miR156l-5p	LOC_Os06g08970.1	145	-21.64	145	-21.64	1029382	21	885	290
miR156l-5p	LOC_Os06g11320.1	161	-24.34	161	-24.34	1029648	21	1833	423
miR156l-5p	LOC_Os06g11320.2	161	-24.34	161	-24.34	1029649	21	1776	506
miR156l-5p	LOC_Os06g11960.1	163	-20.79	163	-20.79	1029720	21	453	7
miR156l-5p	LOC_Os06g13580.1	157	-25.01	157	-25.01	1029911	21	1014	522
miR156l-5p	LOC_Os06g13640.1	166	-26.68	166	-26.68	1029917	21	934	285
miR156l-5p	LOC_Os06g13640.2	166	-26.68	166	-26.68	1029918	21	1250	554
miR156l-5p	LOC_Os06g13760.1	153	-21.63	153	-21.63	1029932	21	2159	1904
miR156l-5p	LOC_Os06g13820.1	167	-27.21	167	-27.21	1029938	21	3391	920
miR156l-5p	LOC_Os06g13820.2	167	-27.21	167	-27.21	1029939	21	3313	920
miR156l-5p	LOC_Os06g16310.1	158	-23.52	158	-23.52	1030200	21	3087	1669
miR156l-5p	LOC_Os06g16330.1	165	-20.96	165	-20.96	1030202	21	2580	2148

miR156l-5p	LOC_Os06g17030.1	164	-27.58	164	-27.58	1030275	21	3168	1751
miR156l-5p	LOC_Os06g19470.4	171	-20.37	171	-20.37	1030467	21	2793	2745
miR156l-5p	LOC_Os06g19690.1	173	-23.69	173	-23.69	1030493	21	1171	1063
miR156l-5p	LOC_Os06g20170.1	159	-22.06	159	-22.06	1030541	21	4965	3753
miR156l-5p	LOC_Os06g20390.1	162	-21.89	162	-21.89	1030565	21	2009	1308
miR156l-5p	LOC_Os06g21360.1	157	-21.43	157	-21.43	1030668	21	1845	1747
miR156l-5p	LOC_Os06g21820.1	161	-22.55	161	-22.55	1030719	21	1323	509
miR156l-5p	LOC_Os06g21820.2	161	-22.55	161	-22.55	1030720	21	1320	506
miR156l-5p	LOC_Os06g22090.1	151	-20.84	151	-20.84	1030753	21	3576	2957
miR156l-5p	LOC_Os06g22440.1	151	-21.39	151	-21.39	1030790	21	1464	1359
miR156l-5p	LOC_Os06g22440.2	151	-21.39	151	-21.39	1030791	21	1572	1467
miR156l-5p	LOC_Os06g22460.1	163	-26.52	163	-26.52	1030793	21	2731	164
miR156l-5p	LOC_Os06g22900.1	165	-21.22	165	-21.22	1030841	21	1458	999
miR156l-5p	LOC_Os06g24950.1	156	-23.65	156	-23.65	1031051	21	960	308
miR156l-5p	LOC_Os06g25230.1	149	-23.26	149	-23.26	1031078	21	1134	772
miR156l-5p	LOC_Os06g25540.1	165	-20.64	165	-20.64	1031105	21	1260	276
miR156l-5p	LOC_Os06g25990.1	153	-23.45	153	-23.45	1031151	21	1398	367
miR156l-5p	LOC_Os06g27430.1	167	-20.49	167	-20.49	1031195	21	456	138
miR156l-5p	LOC_Os06g29400.1	169	-22	169	-22	1031407	21	3578	1313
miR156l-5p	LOC_Os06g29420.1	165	-21.22	165	-21.22	1031409	21	2567	2415
miR156l-5p	LOC_Os06g30420.1	157	-22.88	157	-22.88	1031504	21	1929	143
miR156l-5p	LOC_Os06g30700.1	169	-21.48	169	-21.48	1031537	21	5107	248
miR156l-5p	LOC_Os06g31562.1	165	-21.22	165	-21.22	1031629	21	731	579
miR156l-5p	LOC_Os06g31700.1	165	-21.22	165	-21.22	1031641	21	660	579
miR156l-5p	LOC_Os06g32740.1	162	-23.41	162	-23.41	1031738	21	3273	81
miR156l-5p	LOC_Os06g33710.1	158	-20.51	158	-20.51	1031833	21	1665	1420
miR156l-5p	LOC_Os06g33710.2	158	-20.51	158	-20.51	1031834	21	1669	1424
miR156l-5p	LOC_Os06g33710.3	158	-20.51	158	-20.51	1031835	21	1653	1408
miR156l-5p	LOC_Os06g33710.4	158	-20.51	158	-20.51	1031836	21	1659	1414
miR156l-5p	LOC_Os06g34440.1	182	-28.41	182	-28.41	1031906	21	4703	1055
miR156l-5p	LOC_Os06g35390.1	162	-25.91	162	-25.91	1032002	21	1650	1488
miR156l-5p	LOC_Os06g35900.1	158	-23.13	158	-23.13	1032053	21	1721	1601

miR156l-5p	LOC_Os06g36440.1	161	-21.79	161	-21.79	1032114	21	1818	1708
miR156l-5p	LOC_Os06g36590.1	169	-20.72	169	-20.72	1032130	21	2660	15
miR156l-5p	LOC_Os06g36650.1	155	-20.29	155	-20.29	1032135	21	3588	3270
miR156l-5p	LOC_Os06g36770.1	168	-23.22	168	-23.22	1032147	21	2479	650
miR156l-5p	LOC_Os06g36770.2	168	-23.22	168	-23.22	1032148	21	2476	650
miR156l-5p	LOC_Os06g36770.3	168	-23.22	168	-23.22	1032149	21	2548	719
miR156l-5p	LOC_Os06g37630.1	143	-20.57	143	-20.57	1032239	21	2535	1332
miR156l-5p	LOC_Os06g37860.1	165	-21.22	165	-21.22	1032262	21	2164	1872
miR156l-5p	LOC_Os06g38210.1	164	-21.11	164	-21.11	1032293	21	856	839
miR156l-5p	LOC_Os06g38210.2	164	-20.99	164	-20.99	1032294	21	3678	3655
miR156l-5p	LOC_Os06g38900.1	152	-23.21	152	-23.21	1032365	21	2154	1426
miR156l-5p	LOC_Os06g39590.1	171	-20.47	171	-20.47	1032436	21	1239	1165
miR156l-5p	LOC_Os06g39590.2	171	-20.47	171	-20.47	1032437	21	1318	1254
miR156l-5p	LOC_Os06g41690.1	164	-21.3	164	-21.3	1032686	21	7773	6997
miR156l-5p	LOC_Os06g42650.1	153	-20.9	153	-20.9	1032803	21	1065	130
miR156l-5p	LOC_Os06g42810.1	158	-21.55	158	-21.55	1032821	21	1690	215
miR156l-5p	LOC_Os06g43560.1	165	-20.4	165	-20.4	1032896	21	2963	1569
miR156l-5p	LOC_Os06g43690.1	171	-24.03	171	-24.03	1032916	21	3134	2837
miR156l-5p	LOC_Os06g44034.1	173	-28.41	173	-28.41	1032957	21	2020	363
miR156l-5p	LOC_Os06g45000.1	161	-21.53	161	-21.53	1033063	21	1609	1420
miR156l-5p	LOC_Os06g45000.2	161	-21.53	161	-21.53	1033064	21	1578	1389
miR156l-5p	LOC_Os06g45000.3	161	-21.53	161	-21.53	1033065	21	1605	1571
miR156l-5p	LOC_Os06g45030.1	178	-26.22	178	-26.22	1033067	21	3229	3203
miR156l-5p	LOC_Os06g45260.1	164	-27.58	164	-27.58	1033101	21	3132	1715
miR156l-5p	LOC_Os06g46410.1	161	-20.5	161	-20.5	1033236	21	3745	2090
miR156l-5p	LOC_Os06g46410.2	161	-20.5	161	-20.5	1033237	21	3118	2087
miR156l-5p	LOC_Os06g47310.1	161	-20.33	161	-20.33	1033357	21	1476	1403
miR156l-5p	LOC_Os06g47550.1	166	-22.86	166	-22.86	1033384	21	2801	1596
miR156l-5p	LOC_Os06g47550.2	166	-22.86	166	-22.86	1033385	21	3938	2733
miR156l-5p	LOC_Os06g47550.4	166	-22.86	166	-22.86	1033386	21	3110	1905
miR156l-5p	LOC_Os06g48370.1	156	-24.99	156	-24.99	1033483	21	5121	482
miR156l-5p	LOC_Os06g48380.1	156	-24.99	156	-24.99	1033484	21	5121	482

miR156l-5p	LOC_Os06g48390.1	156	-24.99	156	-24.99	1033485	21	2934	482
miR156l-5p	LOC_Os06g49380.1	155	-20.22	155	-20.22	1033613	21	6026	62
miR156l-5p	LOC_Os06g49380.2	155	-20.22	155	-20.22	1033614	21	6029	62
miR156l-5p	LOC_Os06g49380.3	155	-20.22	155	-20.22	1033615	21	5934	62
miR156l-5p	LOC_Os06g49380.4	155	-20.22	155	-20.22	1033616	21	5937	62
miR156l-5p	LOC_Os06g49380.5	155	-20.22	155	-20.22	1033617	21	5799	62
miR156l-5p	LOC_Os06g49380.6	155	-20.22	155	-20.22	1033618	21	5908	62
miR156l-5p	LOC_Os06g49580.1	161	-21.79	161	-21.79	1033645	21	3288	3178
miR156l-5p	LOC_Os06g50210.1	155	-22.03	155	-22.03	1033725	21	1749	560
miR156l-5p	LOC_Os06g50270.1	155	-23.09	155	-23.09	1033732	21	5910	932
miR156l-5p	LOC_Os06g50300.1	183	-28.03	183	-28.03	1033735	21	2857	1746
miR156l-5p	LOC_Os06g50580.1	146	-20.14	146	-20.14	1033769	21	1428	775
miR156l-5p	LOC_Os06g51500.1	162	-21.1	162	-21.1	1033890	21	1724	676
miR156l-5p	LOC_Os07g04080.1	156	-24.99	156	-24.99	1034239	21	2313	482
miR156l-5p	LOC_Os07g04230.1	162	-20.22	162	-20.22	1034255	21	2295	1143
miR156l-5p	LOC_Os07g05430.1	161	-22.46	161	-22.46	1034382	21	4302	1038
miR156l-5p	LOC_Os07g06750.1	159	-25.27	159	-25.27	1034529	21	528	445
miR156l-5p	LOC_Os07g06770.1	165	-25.58	165	-25.58	1034531	21	903	172
miR156l-5p	LOC_Os07g06790.1	170	-28.97	170	-28.97	1034533	21	534	448
miR156l-5p	LOC_Os07g07130.1	157	-27.59	157	-27.59	1034573	21	2092	1212
miR156l-5p	LOC_Os07g07310.1	157	-20.51	157	-20.51	1034594	21	3257	464
miR156l-5p	LOC_Os07g07940.1	169	-24.16	169	-24.16	1034664	21	321	2
miR156l-5p	LOC_Os07g08440.1	161	-20.54	161	-20.54	1034717	21	1196	1051
miR156l-5p	LOC_Os07g08880.1	157	-21.18	157	-21.18	1034763	21	1220	473
miR156l-5p	LOC_Os07g08880.2	157	-21.18	157	-21.18	1034764	21	1216	473
miR156l-5p	LOC_Os07g08880.3	157	-21.18	157	-21.18	1034765	21	1201	473
miR156l-5p	LOC_Os07g08880.4	157	-21.18	157	-21.18	1034766	21	700	473
miR156l-5p	LOC_Os07g08890.1	166	-20.99	166	-20.99	1034767	21	3169	1221
miR156l-5p	LOC_Os07g08900.1	150	-20.08	150	-20.08	1034768	21	3072	1400
miR156l-5p	LOC_Os07g09384.1	162	-22.33	162	-22.33	1034819	21	3349	2398
miR156l-5p	LOC_Os07g10400.1	155	-23.16	155	-23.16	1034928	21	2181	431
miR156l-5p	LOC_Os07g11600.1	164	-27.58	164	-27.58	1035070	21	2955	1538

miR156l-5p	LOC_Os07g11860.1	157	-27.19	157	-27.19	1035092	21	4251	415
miR156l-5p	LOC_Os07g12630.1	152	-20.24	152	-20.24	1035173	21	1526	481
miR156l-5p	LOC_Os07g12730.1	168	-21.16	168	-21.16	1035183	21	1140	425
miR156l-5p	LOC_Os07g13860.1	161	-21.79	161	-21.79	1035294	21	2733	2482
miR156l-5p	LOC_Os07g14920.1	156	-23.01	156	-23.01	1035417	21	2602	70
miR156l-5p	LOC_Os07g15210.1	157	-22.25	157	-22.25	1035447	21	3144	482
miR156l-5p	LOC_Os07g16000.1	156	-23.65	156	-23.65	1035527	21	4893	386
miR156l-5p	LOC_Os07g16010.1	156	-23.65	156	-23.65	1035528	21	627	455
miR156l-5p	LOC_Os07g16900.1	161	-21.79	161	-21.79	1035609	21	2724	2557
miR156l-5p	LOC_Os07g18300.1	171	-21.77	171	-21.77	1035759	21	2687	2120
miR156l-5p	LOC_Os07g18530.1	165	-21.22	165	-21.22	1035780	21	2308	2016
miR156l-5p	LOC_Os07g19190.1	154	-22.22	154	-22.22	1035838	21	1193	1023
miR156l-5p	LOC_Os07g19510.1	163	-20.25	163	-20.25	1035867	21	3390	2718
miR156l-5p	LOC_Os07g20200.1	157	-20.85	157	-20.85	1035888	21	3207	1791
miR156l-5p	LOC_Os07g20250.1	159	-22.06	159	-22.06	1035893	21	1545	159
miR156l-5p	LOC_Os07g20290.1	163	-20.16	163	-20.16	1035897	21	542	119
miR156l-5p	LOC_Os07g22880.1	161	-21.79	161	-21.79	1036071	21	2887	2728
miR156l-5p	LOC_Os07g24020.1	165	-20.74	165	-20.74	1036198	21	2041	1749
miR156l-5p	LOC_Os07g25330.1	175	-23.7	175	-23.7	1036331	21	3960	1647
miR156l-5p	LOC_Os07g27320.1	170	-22.39	170	-22.39	1036543	21	3792	3697
miR156l-5p	LOC_Os07g27670.1	153	-29.05	153	-29.05	1036577	21	791	617
miR156l-5p	LOC_Os07g28090.1	153	-21.25	153	-21.25	1036622	21	2792	1650
miR156l-5p	LOC_Os07g28510.1	156	-24.99	156	-24.99	1036667	21	5128	353
miR156l-5p	LOC_Os07g28760.1	162	-22.47	162	-22.47	1036695	21	2352	670
miR156l-5p	LOC_Os07g29100.1	150	-21.63	150	-21.63	1036728	21	2670	415
miR156l-5p	LOC_Os07g30250.1	161	-21.4	161	-21.4	1036857	21	3206	408
miR156l-5p	LOC_Os07g30580.1	170	-20.8	170	-20.8	1036885	21	5787	2755
miR156l-5p	LOC_Os07g30730.1	156	-24.99	156	-24.99	1036901	21	4566	482
miR156l-5p	LOC_Os07g30910.1	156	-24.99	156	-24.99	1036923	21	5358	482
miR156l-5p	LOC_Os07g31170.1	144	-21.86	144	-21.86	1036949	21	4128	3732
miR156l-5p	LOC_Os07g31690.1	157	-20.02	157	-20.02	1037009	21	2486	1315
miR156l-5p	LOC_Os07g31830.1	157	-22.3	157	-22.3	1037020	21	964	63

miR156l-5p	LOC_Os07g33350.1	157	-24.69	157	-24.69	1037182	21	931	882
miR156l-5p	LOC_Os07g33350.2	157	-24.69	157	-24.69	1037183	21	845	797
miR156l-5p	LOC_Os07g33620.1	151	-20.03	151	-20.03	1037213	21	1841	180
miR156l-5p	LOC_Os07g33730.1	161	-25.49	161	-25.49	1037226	21	4498	450
miR156l-5p	LOC_Os07g33730.2	161	-25.49	161	-25.49	1037227	21	4784	736
miR156l-5p	LOC_Os07g33921.1	160	-21.17	160	-21.17	1037240	21	570	475
miR156l-5p	LOC_Os07g33988.1	160	-21.17	160	-21.17	1037244	21	722	476
miR156l-5p	LOC_Os07g35240.1	161	-21.79	161	-21.79	1037384	21	4551	4102
miR156l-5p	LOC_Os07g36940.1	158	-20.97	158	-20.97	1037581	21	6114	6035
miR156l-5p	LOC_Os07g36940.2	158	-20.97	158	-20.97	1037582	21	6180	6101
miR156l-5p	LOC_Os07g37750.1	166	-22.87	166	-22.87	1037672	21	5490	3186
miR156l-5p	LOC_Os07g41430.1	152	-23.21	152	-23.21	1038088	21	5007	4216
miR156l-5p	LOC_Os07g43380.1	154	-20.14	154	-20.14	1038330	21	1672	207
miR156l-5p	LOC_Os07g43390.1	158	-24.85	158	-24.85	1038331	21	2114	1872
miR156l-5p	LOC_Os07g43780.1	156	-21.66	156	-21.66	1038369	21	1176	533
miR156l-5p	LOC_Os07g44040.1	158	-22.16	158	-22.16	1038398	21	1530	453
miR156l-5p	LOC_Os07g44299.1	148	-20.43	148	-20.43	1038427	21	1824	925
miR156l-5p	LOC_Os07g44700.1	162	-22.29	162	-22.29	1038474	21	1332	243
miR156l-5p	LOC_Os07g45300.1	161	-23.8	161	-23.8	1038535	21	1062	559
miR156l-5p	LOC_Os07g45530.1	161	-23.37	161	-23.37	1038559	21	5832	5355
miR156l-5p	LOC_Os07g45900.1	161	-21.79	161	-21.79	1038596	21	3288	2932
miR156l-5p	LOC_Os07g46020.1	152	-23.21	152	-23.21	1038608	21	3225	2497
miR156l-5p	LOC_Os07g47070.1	150	-20.28	150	-20.28	1038752	21	1050	434
miR156l-5p	LOC_Os07g48030.1	157	-21.42	157	-21.42	1038887	21	1380	94
miR156l-5p	LOC_Os07g48970.1	161	-21.17	161	-21.17	1039014	21	2287	948
miR156l-5p	LOC_Os07g48970.2	161	-21.17	161	-21.17	1039015	21	3104	986
miR156l-5p	LOC_Os07g49230.1	153	-20.17	153	-20.17	1039042	21	3642	1581
miR156l-5p	LOC_Os07g49230.2	153	-20.17	153	-20.17	1039043	21	3581	1460
miR156l-5p	LOC_Os07g49230.3	153	-20.17	153	-20.17	1039044	21	3093	1447
miR156l-5p	LOC_Os07g49260.1	161	-21.99	161	-21.99	1039047	21	3233	2167
miR156l-5p	LOC_Os08g01850.1	165	-20.32	165	-20.32	1039199	21	2341	264
miR156l-5p	LOC_Os08g03430.1	150	-21.66	150	-21.66	1039380	21	1937	392

miR156l-5p	LOC_Os08g03430.2	150	-21.66	150	-21.66	1039381	21	1935	376
miR156l-5p	LOC_Os08g04130.1	162	-20.67	162	-20.67	1039460	21	2056	1825
miR156l-5p	LOC_Os08g04130.2	162	-20.67	162	-20.67	1039461	21	2228	1997
miR156l-5p	LOC_Os08g04130.3	162	-20.67	162	-20.67	1039462	21	1994	1763
miR156l-5p	LOC_Os08g04330.1	161	-21.79	161	-21.79	1039486	21	2064	1951
miR156l-5p	LOC_Os08g04920.1	159	-21.89	159	-21.89	1039551	21	2389	2071
miR156l-5p	LOC_Os08g04920.2	159	-21.89	159	-21.89	1039552	21	2265	1947
miR156l-5p	LOC_Os08g04920.3	159	-21.89	159	-21.89	1039553	21	1856	1538
miR156l-5p	LOC_Os08g04920.4	159	-21.89	159	-21.89	1039554	21	2201	1883
miR156l-5p	LOC_Os08g04920.5	159	-21.89	159	-21.89	1039555	21	2314	1996
miR156l-5p	LOC_Os08g04920.6	159	-21.89	159	-21.89	1039556	21	2174	1856
miR156l-5p	LOC_Os08g04920.7	159	-21.89	159	-21.89	1039557	21	2211	1893
miR156l-5p	LOC_Os08g04920.8	159	-21.89	159	-21.89	1039558	21	2337	2019
miR156l-5p	LOC_Os08g04950.1	156	-24.99	156	-24.99	1039560	21	5187	197
miR156l-5p	LOC_Os08g05400.1	161	-21.79	161	-21.79	1039606	21	1479	1366
miR156l-5p	LOC_Os08g06010.1	159	-24.29	159	-24.29	1039672	21	2316	850
miR156l-5p	LOC_Os08g06690.1	160	-23.99	160	-23.99	1039759	21	1330	182
miR156l-5p	LOC_Os08g06700.1	160	-23.99	160	-23.99	1039760	21	2140	296
miR156l-5p	LOC_Os08g09210.1	158	-25.32	158	-25.32	1040021	21	2320	513
miR156l-5p	LOC_Os08g09210.2	158	-25.32	158	-25.32	1040022	21	2315	508
miR156l-5p	LOC_Os08g10510.1	335	-46.67	169	-26.57	1040172	21	1873	1597 1026
miR156l-5p	LOC_Os08g10510.2	335	-46.67	169	-26.57	1040173	21	1955	1597 1026
miR156l-5p	LOC_Os08g10550.1	177	-26.29	177	-26.29	1040177	21	3404	87
miR156l-5p	LOC_Os08g12170.1	145	-20.86	145	-20.86	1040347	21	561	69
miR156l-5p	LOC_Os08g12430.1	175	-22.96	175	-22.96	1040371	21	2914	2323
miR156l-5p	LOC_Os08g13280.1	154	-22.24	154	-22.24	1040465	21	976	758
miR156l-5p	LOC_Os08g14960.1	160	-23.51	160	-23.51	1040646	21	1473	187
miR156l-5p	LOC_Os08g19850.1	152	-20.76	152	-20.76	1041125	21	2498	765
miR156l-5p	LOC_Os08g23300.1	160	-24.84	160	-24.84	1041444	21	3144	1726
miR156l-5p	LOC_Os08g23910.1	161	-21.79	161	-21.79	1041511	21	2910	2722
miR156l-5p	LOC_Os08g24860.1	164	-27.58	164	-27.58	1041606	21	3165	1793
miR156l-5p	LOC_Os08g25200.1	159	-24.43	159	-24.43	1041640	21	342	196

miR156l-5p	LOC_Os08g26090.1	161	-21.79	161	-21.79	1041727	21	3288	3178
miR156l-5p	LOC_Os08g26360.1	160	-21.89	160	-21.89	1041759	21	284	2
miR156l-5p	LOC_Os08g26800.1	154	-25.34	154	-25.34	1041802	21	672	336
miR156l-5p	LOC_Os08g27000.1	152	-23.21	152	-23.21	1041828	21	4197	3640
miR156l-5p	LOC_Os08g27010.1	156	-21.88	156	-21.88	1041829	21	1340	1119
miR156l-5p	LOC_Os08g28100.1	171	-26.14	171	-26.14	1041943	21	2643	2557
miR156l-5p	LOC_Os08g28460.1	154	-22.06	154	-22.06	1041981	21	2729	2242
miR156l-5p	LOC_Os08g30260.1	159	-21.21	159	-21.21	1042169	21	1329	125
miR156l-5p	LOC_Os08g31219.1	173	-24.07	173	-24.07	1042264	21	944	291
miR156l-5p	LOC_Os08g31228.1	173	-24.07	173	-24.07	1042265	21	1546	559
miR156l-5p	LOC_Os08g31350.1	177	-26.29	177	-26.29	1042276	21	2055	1185
miR156l-5p	LOC_Os08g31400.1	165	-21.22	165	-21.22	1042281	21	2755	2463
miR156l-5p	LOC_Os08g32280.1	165	-21.22	165	-21.22	1042385	21	850	558
miR156l-5p	LOC_Os08g32830.1	161	-21.79	161	-21.79	1042445	21	3288	3178
miR156l-5p	LOC_Os08g34590.1	171	-20.92	171	-20.92	1042648	21	2109	1897
miR156l-5p	LOC_Os08g35570.1	173	-29.9	173	-29.9	1042760	21	677	547
miR156l-5p	LOC_Os08g35650.1	159	-20.93	159	-20.93	1042769	21	1458	805
miR156l-5p	LOC_Os08g36280.1	161	-21.79	161	-21.79	1042837	21	3273	3178
miR156l-5p	LOC_Os08g38410.1	161	-21.9	161	-21.9	1043096	21	1897	1427
miR156l-5p	LOC_Os08g38410.2	161	-21.9	161	-21.9	1043097	21	2012	1427
miR156l-5p	LOC_Os08g38410.3	161	-21.9	161	-21.9	1043098	21	1630	1160
miR156l-5p	LOC_Os08g38890.1	157	-24.51	157	-24.51	1043155	21	1765	520
miR156l-5p	LOC_Os08g38970.1	147	-21.46	147	-21.46	1043168	21	3554	2353
miR156l-5p	LOC_Os08g40350.1	146	-20.82	146	-20.82	1043332	21	2076	438
miR156l-5p	LOC_Os08g40360.1	154	-21.64	154	-21.64	1043333	21	3891	2354
miR156l-5p	LOC_Os08g40900.1	157	-22.91	157	-22.91	1043404	21	4066	1418
miR156l-5p	LOC_Os08g40900.2	157	-22.91	157	-22.91	1043405	21	3870	1418
miR156l-5p	LOC_Os08g41360.1	157	-21.82	157	-21.82	1043461	21	405	253
miR156l-5p	LOC_Os08g41530.1	157	-23.44	157	-23.44	1043486	21	3363	2564
miR156l-5p	LOC_Os08g41750.1	154	-23.6	154	-23.6	1043507	21	1116	46
miR156l-5p	LOC_Os08g41940.1	187	-32.65	187	-32.65	1043528	21	1708	1053
miR156l-5p	LOC_Os08g42930.1	146	-22.83	146	-22.83	1043656	21	3198	1067

miR156l-5p	LOC_Os08g43590.1	151	-20.18	151	-20.18	1043736	21	435	184
miR156l-5p	LOC_Os09g01050.1	152	-20.22	152	-20.22	1043962	21	1167	1128
miR156l-5p	LOC_Os09g01290.2	155	-21.84	155	-21.84	1043988	21	3856	864
miR156l-5p	LOC_Os09g02050.1	167	-21.41	167	-21.41	1044071	21	1671	1114
miR156l-5p	LOC_Os09g02300.1	166	-24.3	166	-24.3	1044098	21	1238	779
miR156l-5p	LOC_Os09g02500.1	165	-21.22	165	-21.22	1044121	21	2800	2508
miR156l-5p	LOC_Os09g03570.1	160	-20.39	160	-20.39	1044226	21	1932	691
miR156l-5p	LOC_Os09g03830.1	156	-24.99	156	-24.99	1044251	21	4344	482
miR156l-5p	LOC_Os09g04010.1	165	-21.22	165	-21.22	1044268	21	2540	2388
miR156l-5p	LOC_Os09g04270.1	148	-25.59	148	-25.59	1044296	21	1734	1473
miR156l-5p	LOC_Os09g04740.1	157	-22.25	157	-22.25	1044348	21	4383	497
miR156l-5p	LOC_Os09g06780.1	169	-26.67	169	-26.67	1044454	21	2115	1875
miR156l-5p	LOC_Os09g08040.1	164	-20.2	164	-20.2	1044598	21	2781	733
miR156l-5p	LOC_Os09g08120.1	160	-25.22	160	-25.22	1044604	21	1352	39
miR156l-5p	LOC_Os09g08420.1	156	-22.78	156	-22.78	1044637	21	2392	426
miR156l-5p	LOC_Os09g08470.1	165	-24.9	165	-24.9	1044643	21	4581	1865
miR156l-5p	LOC_Os09g11540.1	160	-26.79	160	-26.79	1044961	21	441	344
miR156l-5p	LOC_Os09g11900.1	146	-20.82	146	-20.82	1044997	21	1681	462
miR156l-5p	LOC_Os09g11910.1	154	-21.64	154	-21.64	1044998	21	3489	2579
miR156l-5p	LOC_Os09g12180.1	171	-20.92	171	-20.92	1045022	21	804	400
miR156l-5p	LOC_Os09g13320.1	165	-21.22	165	-21.22	1045140	21	1466	1176
miR156l-5p	LOC_Os09g13740.1	165	-20.53	165	-20.53	1045185	21	1728	1092
miR156l-5p	LOC_Os09g13740.2	165	-20.53	165	-20.53	1045186	21	1232	596
miR156l-5p	LOC_Os09g13900.1	171	-20.23	171	-20.23	1045204	21	2595	2479
miR156l-5p	LOC_Os09g15010.1	151	-23.29	151	-23.29	1045324	21	3999	3672
miR156l-5p	LOC_Os09g15470.1	317	-47.68	161	-24.99	1045377	21	4776	774 482
miR156l-5p	LOC_Os09g16430.1	157	-22.25	157	-22.25	1045477	21	3786	482
miR156l-5p	LOC_Os09g17030.1	156	-24.99	156	-24.99	1045541	21	5409	482
miR156l-5p	LOC_Os09g18530.1	164	-20.49	164	-20.49	1045689	21	1395	114
miR156l-5p	LOC_Os09g19830.1	154	-20.19	154	-20.19	1045766	21	3844	3654
miR156l-5p	LOC_Os09g21680.1	148	-21.16	148	-21.16	1045972	21	2415	1758
miR156l-5p	LOC_Os09g23690.1	156	-23.06	156	-23.06	1046132	21	1738	683

miR156l-5p	LOC_Os09g24210.1	158	-21.74	158	-21.74	1046187	21	2550	921
miR156l-5p	LOC_Os09g26160.1	168	-23.13	168	-23.13	1046400	21	3437	1346
miR156l-5p	LOC_Os09g26160.2	168	-23.13	168	-23.13	1046401	21	3000	909
miR156l-5p	LOC_Os09g26400.1	161	-20.37	161	-20.37	1046427	21	1414	487
miR156l-5p	LOC_Os09g27100.1	156	-20.23	156	-20.23	1046512	21	1167	919
miR156l-5p	LOC_Os09g27670.1	158	-21.88	158	-21.88	1046584	21	1029	1002
miR156l-5p	LOC_Os09g28260.1	156	-24.99	156	-24.99	1046661	21	5340	482
miR156l-5p	LOC_Os09g29000.1	157	-20.7	157	-20.7	1046745	21	3579	2629
miR156l-5p	LOC_Os09g30020.1	178	-25.85	178	-25.85	1046863	21	4770	3816
miR156l-5p	LOC_Os09g32944.1	187	-32.65	187	-32.65	1047161	21	1419	1033
miR156l-5p	LOC_Os09g33820.3	172	-24.76	172	-24.76	1047234	21	2361	1383
miR156l-5p	LOC_Os09g33920.1	165	-20.72	165	-20.72	1047245	21	1627	954
miR156l-5p	LOC_Os09g33920.2	165	-20.72	165	-20.72	1047246	21	1563	954
miR156l-5p	LOC_Os09g33980.1	163	-21.7	163	-21.7	1047257	21	4160	2417
miR156l-5p	LOC_Os09g36660.1	145	-20.08	145	-20.08	1047476	21	741	495
miR156l-5p	LOC_Os09g37590.1	149	-20.84	149	-20.84	1047585	21	2880	128
miR156l-5p	LOC_Os09g37760.1	172	-24.59	172	-24.59	1047604	21	2307	1191
miR156l-5p	LOC_Os09g38560.1	153	-20.52	153	-20.52	1047690	21	828	397
miR156l-5p	LOC_Os09g39110.1	153	-23.95	153	-23.95	1047760	21	1401	968
miR156l-5p	LOC_Os09g39410.1	160	-25.82	160	-25.82	1047793	21	2078	2036
miR156l-5p	LOC_Os10g02210.1	145	-21.08	145	-21.08	1048001	21	1771	1628
miR156l-5p	LOC_Os10g02260.2	166	-21.22	166	-21.22	1048011	21	2955	2856
miR156l-5p	LOC_Os10g02350.1	166	-21.31	166	-21.31	1048021	21	2233	1632
miR156l-5p	LOC_Os10g02970.1	165	-24.63	165	-24.63	1048085	21	724	263
miR156l-5p	LOC_Os10g04730.1	145	-20.32	145	-20.32	1048261	21	2210	488
miR156l-5p	LOC_Os10g06430.1	156	-23.65	156	-23.65	1048433	21	4710	455
miR156l-5p	LOC_Os10g08660.1	163	-20.22	163	-20.22	1048642	21	3771	343
miR156l-5p	LOC_Os10g08930.1	163	-24.14	163	-24.14	1048669	21	814	718
miR156l-5p	LOC_Os10g09770.1	154	-22.44	154	-22.44	1048746	21	414	321
miR156l-5p	LOC_Os10g10060.1	157	-21.3	157	-21.3	1048776	21	411	155
miR156l-5p	LOC_Os10g10090.1	160	-20.08	160	-20.08	1048780	21	357	1
miR156l-5p	LOC_Os10g14140.1	165	-21.22	165	-21.22	1049123	21	500	348

miR156l-5p	LOC_Os10g15130.1	156	-23.48	156	-23.48	1049180	21	2961	455
miR156l-5p	LOC_Os10g16720.1	156	-24.99	156	-24.99	1049243	21	5145	482
miR156l-5p	LOC_Os10g16824.1	171	-20.92	171	-20.92	1049252	21	1908	349
miR156l-5p	LOC_Os10g16900.1	155	-25.46	155	-25.46	1049259	21	5322	469
miR156l-5p	LOC_Os10g16950.1	156	-24.99	156	-24.99	1049263	21	4569	482
miR156l-5p	LOC_Os10g17050.1	156	-24.99	156	-24.99	1049273	21	4278	407
miR156l-5p	LOC_Os10g17560.1	156	-24.99	156	-24.99	1049319	21	5334	482
miR156l-5p	LOC_Os10g18740.1	157	-27.59	157	-27.59	1049426	21	2811	24
miR156l-5p	LOC_Os10g18940.1	160	-24.11	160	-24.11	1049445	21	2034	1611
miR156l-5p	LOC_Os10g19050.1	152	-20.34	152	-20.34	1049455	21	834	202
miR156l-5p	LOC_Os10g19830.1	143	-20.57	143	-20.57	1049491	21	1194	219
miR156l-5p	LOC_Os10g20570.1	156	-24.99	156	-24.99	1049578	21	3330	482
miR156l-5p	LOC_Os10g20780.1	160	-24.11	160	-24.11	1049598	21	1053	633
miR156l-5p	LOC_Os10g24620.1	158	-21.41	158	-21.41	1049982	21	5110	336
miR156l-5p	LOC_Os10g25760.1	165	-21.22	165	-21.22	1050094	21	2984	2832
miR156l-5p	LOC_Os10g26070.1	160	-20.18	160	-20.18	1050126	21	1966	570
miR156l-5p	LOC_Os10g26360.1	152	-23.21	152	-23.21	1050156	21	4779	4051
miR156l-5p	LOC_Os10g26380.1	161	-20.15	161	-20.15	1050158	21	432	30
miR156l-5p	LOC_Os10g28000.1	167	-27.56	167	-27.56	1050278	21	1494	1132
miR156l-5p	LOC_Os10g28710.1	142	-20.08	142	-20.08	1050364	21	2322	1486
miR156l-5p	LOC_Os10g29660.2	153	-22.71	153	-22.71	1050458	21	1336	332
miR156l-5p	LOC_Os10g29980.1	156	-24.99	156	-24.99	1050486	21	4095	11
miR156l-5p	LOC_Os10g30090.1	150	-21.47	150	-21.47	1050497	21	2403	823
miR156l-5p	LOC_Os10g32270.1	166	-20.82	166	-20.82	1050715	21	1924	1859
miR156l-5p	LOC_Os10g32649.1	156	-24.99	156	-24.99	1050744	21	3876	401
miR156l-5p	LOC_Os10g33030.1	160	-20.39	160	-20.39	1050790	21	1104	691
miR156l-5p	LOC_Os10g33500.1	149	-20.26	149	-20.26	1050838	21	1977	129
miR156l-5p	LOC_Os10g34310.1	167	-20.49	167	-20.49	1050934	21	2124	105
miR156l-5p	LOC_Os10g34310.2	167	-20.49	167	-20.49	1050935	21	2234	101
miR156l-5p	LOC_Os10g34490.1	161	-23.35	161	-23.35	1050953	21	1014	285
miR156l-5p	LOC_Os10g34740.1	151	-20.48	151	-20.48	1050982	21	1856	1026
miR156l-5p	LOC_Os10g36550.1	145	-20.49	145	-20.49	1051190	21	2075	1783

miR156l-5p	LOC_Os10g36550.2	145	-20.49	145	-20.49	1051191	21	2117	1825
miR156l-5p	LOC_Os10g36550.3	145	-20.49	145	-20.49	1051192	21	2241	1949
miR156l-5p	LOC_Os10g40250.1	144	-22.45	144	-22.45	1051609	21	3738	1759
miR156l-5p	LOC_Os10g40250.2	144	-22.45	144	-22.45	1051610	21	3865	1886
miR156l-5p	LOC_Os10g41320.1	148	-20.44	148	-20.44	1051723	21	237	195
miR156l-5p	LOC_Os10g42080.1	149	-22.52	149	-22.52	1051820	21	819	32
miR156l-5p	LOC_Os10g42730.1	163	-26.61	163	-26.61	1051900	21	1125	40
miR156l-5p	LOC_Os10g42730.2	163	-26.61	163	-26.61	1051901	21	2943	40
miR156l-5p	LOC_Os10g42730.3	163	-26.61	163	-26.61	1051902	21	1125	40
miR156l-5p	LOC_Os11g02270.1	145	-21.86	145	-21.86	1052099	21	1640	992
miR156l-5p	LOC_Os11g02710.1	153	-20.24	153	-20.24	1052156	21	1879	1581
miR156l-5p	LOC_Os11g04950.1	173	-24.84	173	-24.84	1052416	21	2478	601
miR156l-5p	LOC_Os11g05340.1	170	-23.04	170	-23.04	1052464	21	3558	303
miR156l-5p	LOC_Os11g07100.2	159	-20.39	159	-20.39	1052684	21	3541	111
miR156l-5p	LOC_Os11g08460.1	156	-20.4	156	-20.4	1052839	21	2110	487
miR156l-5p	LOC_Os11g08460.2	156	-20.4	156	-20.4	1052840	21	1928	502
miR156l-5p	LOC_Os11g08810.1	161	-21.79	161	-21.79	1052870	21	3288	3178
miR156l-5p	LOC_Os11g09010.1	165	-20.39	165	-20.39	1052890	21	2484	2367
miR156l-5p	LOC_Os11g09010.2	165	-20.39	165	-20.39	1052891	21	2399	2282
miR156l-5p	LOC_Os11g09010.3	165	-20.39	165	-20.39	1052892	21	2307	2190
miR156l-5p	LOC_Os11g10190.1	143	-20.57	143	-20.57	1053019	21	2013	429
miR156l-5p	LOC_Os11g10460.1	165	-20.16	165	-20.16	1053047	21	1892	574
miR156l-5p	LOC_Os11g10910.1	158	-23.93	158	-23.93	1053094	21	1394	1318
miR156l-5p	LOC_Os11g10970.1	153	-22.25	153	-22.25	1053100	21	3300	478
miR156l-5p	LOC_Os11g11070.1	151	-20.76	151	-20.76	1053112	21	2772	1824
miR156l-5p	LOC_Os11g11070.2	151	-20.76	151	-20.76	1053113	21	3376	2725
miR156l-5p	LOC_Os11g12450.1	161	-21.79	161	-21.79	1053253	21	3303	3163
miR156l-5p	LOC_Os11g12730.1	161	-21.79	161	-21.79	1053281	21	5055	4945
miR156l-5p	LOC_Os11g13410.1	165	-23.31	165	-23.31	1053299	21	3291	700
miR156l-5p	LOC_Os11g14960.1	164	-27.58	164	-27.58	1053474	21	2997	1580
miR156l-5p	LOC_Os11g17230.1	157	-26.15	157	-26.15	1053665	21	3858	478
miR156l-5p	LOC_Os11g20590.1	165	-21.22	165	-21.22	1053995	21	2812	2520

miR156l-5p	LOC_Os11g21940.1	156	-23.17	156	-23.17	1054030	21	1062	482
miR156l-5p	LOC_Os11g22220.1	157	-21.74	157	-21.74	1054062	21	3330	482
miR156l-5p	LOC_Os11g22404.1	166	-20.61	166	-20.61	1054082	21	2159	1847
miR156l-5p	LOC_Os11g22570.1	168	-21.67	168	-21.67	1054097	21	4530	616
miR156l-5p	LOC_Os11g24100.1	165	-21.22	165	-21.22	1054215	21	1477	1185
miR156l-5p	LOC_Os11g24450.1	154	-21.45	154	-21.45	1054242	21	1388	326
miR156l-5p	LOC_Os11g24590.1	156	-20.16	156	-20.16	1054260	21	2114	1305
miR156l-5p	LOC_Os11g26520.1	153	-23.45	153	-23.45	1054437	21	3621	478
miR156l-5p	LOC_Os11g26594.1	170	-21.04	170	-21.04	1054444	21	1495	833
miR156l-5p	LOC_Os11g26594.2	170	-21.04	170	-21.04	1054445	21	2603	833
miR156l-5p	LOC_Os11g28370.1	147	-21.06	147	-21.06	1054622	21	663	1
miR156l-5p	LOC_Os11g30360.1	164	-24.53	164	-24.53	1054823	21	562	4
miR156l-5p	LOC_Os11g30460.1	161	-21.79	161	-21.79	1054833	21	3243	3130
miR156l-5p	LOC_Os11g30850.1	156	-23.48	156	-23.48	1054867	21	1173	455
miR156l-5p	LOC_Os11g31470.1	146	-22.27	146	-22.27	1054921	21	3722	1488
miR156l-5p	LOC_Os11g31470.2	146	-22.27	146	-22.27	1054922	21	3753	1522
miR156l-5p	LOC_Os11g31470.3	146	-22.27	146	-22.27	1054923	21	3656	1488
miR156l-5p	LOC_Os11g31470.4	146	-22.27	146	-22.27	1054924	21	3291	1488
miR156l-5p	LOC_Os11g31830.1	152	-23.21	152	-23.21	1054967	21	2400	1843
miR156l-5p	LOC_Os11g33420.1	159	-22.13	159	-22.13	1055140	21	651	180
miR156l-5p	LOC_Os11g34490.1	168	-21.67	168	-21.67	1055218	21	3645	670
miR156l-5p	LOC_Os11g35320.1	149	-22.77	149	-22.77	1055300	21	1779	570
miR156l-5p	LOC_Os11g35550.1	150	-20.87	150	-20.87	1055328	21	1458	539
miR156l-5p	LOC_Os11g35840.1	154	-22.14	154	-22.14	1055357	21	4503	3942
miR156l-5p	LOC_Os11g38030.1	161	-21.07	161	-21.07	1055591	21	2316	915
miR156l-5p	LOC_Os11g38090.1	158	-24.23	158	-24.23	1055596	21	5334	480
miR156l-5p	LOC_Os11g39250.1	152	-23.21	152	-23.21	1055727	21	3042	2218
miR156l-5p	LOC_Os11g42240.1	158	-23.55	158	-23.55	1056045	21	1959	168
miR156l-5p	LOC_Os11g43120.1	161	-21.79	161	-21.79	1056138	21	975	838
miR156l-5p	LOC_Os11g45420.1	155	-24.45	155	-24.45	1056381	21	1977	668
miR156l-5p	LOC_Os11g45680.1	164	-27.58	164	-27.58	1056407	21	3144	1727
miR156l-5p	LOC_Os11g45880.1	161	-21.79	161	-21.79	1056430	21	2121	2008

miR156l-5p	LOC_Os11g45970.1	153	-21	153	-21	1056440	21	3072	213
miR156l-5p	LOC_Os12g01430.1	155	-23.07	155	-23.07	1056694	21	1167	624
miR156l-5p	LOC_Os12g01430.2	155	-23.07	155	-23.07	1056695	21	1488	582
miR156l-5p	LOC_Os12g02230.1	145	-20.79	145	-20.79	1056793	21	1641	989
miR156l-5p	LOC_Os12g02640.1	153	-20.24	153	-20.24	1056852	21	2264	1781
miR156l-5p	LOC_Os12g03150.1	169	-22.17	169	-22.17	1056916	21	1599	1577
miR156l-5p	LOC_Os12g05270.1	144	-21.12	144	-21.12	1057168	21	834	166
miR156l-5p	LOC_Os12g05280.1	144	-21.12	144	-21.12	1057169	21	729	61
miR156l-5p	LOC_Os12g05650.1	158	-23.39	158	-23.39	1057206	21	1459	1413
miR156l-5p	LOC_Os12g06980.1	164	-21.36	164	-21.36	1057359	21	7811	6818
miR156l-5p	LOC_Os12g08060.1	157	-24.39	157	-24.39	1057480	21	3309	2127
miR156l-5p	LOC_Os12g08180.1	163	-20.94	163	-20.94	1057492	21	3607	1112
miR156l-5p	LOC_Os12g08930.1	169	-22.24	169	-22.24	1057574	21	552	85
miR156l-5p	LOC_Os12g11720.1	151	-20.61	151	-20.61	1057861	21	3060	1280
miR156l-5p	LOC_Os12g11730.1	167	-21.78	167	-21.78	1057862	21	2928	1176
miR156l-5p	LOC_Os12g12120.1	151	-20.61	151	-20.61	1057898	21	3297	1287
miR156l-5p	LOC_Os12g12410.1	156	-24.99	156	-24.99	1057932	21	4503	482
miR156l-5p	LOC_Os12g12720.1	161	-21.96	161	-21.96	1057962	21	1045	974
miR156l-5p	LOC_Os12g12840.1	161	-21.79	161	-21.79	1057975	21	3288	3178
miR156l-5p	LOC_Os12g13460.1	161	-21.37	161	-21.37	1058049	21	2064	2042
miR156l-5p	LOC_Os12g14100.1	155	-22.51	155	-22.51	1058116	21	1770	1342
miR156l-5p	LOC_Os12g14170.1	157	-21.7	157	-21.7	1058124	21	2370	1398
miR156l-5p	LOC_Os12g14390.1	154	-20.14	154	-20.14	1058145	21	1152	1100
miR156l-5p	LOC_Os12g14460.1	156	-23.94	156	-23.94	1058152	21	4290	482
miR156l-5p	LOC_Os12g15980.1	146	-20.82	146	-20.82	1058290	21	1599	200
miR156l-5p	LOC_Os12g16000.1	154	-21.64	154	-21.64	1058292	21	3357	2375
miR156l-5p	LOC_Os12g16520.1	159	-20.57	159	-20.57	1058356	21	1728	1485
miR156l-5p	LOC_Os12g16524.1	159	-20.57	159	-20.57	1058357	21	2076	142
miR156l-5p	LOC_Os12g16524.2	159	-20.57	159	-20.57	1058358	21	848	142
miR156l-5p	LOC_Os12g16524.3	159	-20.57	159	-20.57	1058359	21	810	142
miR156l-5p	LOC_Os12g17970.1	156	-24.99	156	-24.99	1058505	21	654	482
miR156l-5p	LOC_Os12g18010.1	147	-21.68	147	-21.68	1058509	21	4176	3714

miR156l-5p	LOC_Os12g18060.1	170	-20.8	170	-20.8	1058514	21	3702	673
miR156l-5p	LOC_Os12g18690.1	150	-20.99	150	-20.99	1058587	21	1761	691
miR156l-5p	LOC_Os12g20450.1	155	-22.74	155	-22.74	1058765	21	3342	46
miR156l-5p	LOC_Os12g22200.1	159	-20.66	159	-20.66	1058845	21	5670	3967
miR156l-5p	LOC_Os12g23130.1	156	-24.99	156	-24.99	1058934	21	3732	107
miR156l-5p	LOC_Os12g24790.1	161	-21.67	161	-21.67	1059090	21	3342	1638
miR156l-5p	LOC_Os12g25390.1	165	-21.22	165	-21.22	1059148	21	1472	1320
miR156l-5p	LOC_Os12g25910.1	156	-24.99	156	-24.99	1059202	21	4708	230
miR156l-5p	LOC_Os12g26310.1	159	-20.7	159	-20.7	1059246	21	5799	229
miR156l-5p	LOC_Os12g26960.1	157	-21.36	157	-21.36	1059308	21	603	493
miR156l-5p	LOC_Os12g27010.1	165	-21.22	165	-21.22	1059313	21	2988	2715
miR156l-5p	LOC_Os12g27254.1	175	-22.22	175	-22.22	1059341	21	1827	648
miR156l-5p	LOC_Os12g27350.1	179	-25.1	179	-25.1	1059351	21	1638	573
miR156l-5p	LOC_Os12g27490.1	156	-24.99	156	-24.99	1059363	21	4836	482
miR156l-5p	LOC_Os12g28470.1	156	-24.99	156	-24.99	1059457	21	5478	482
miR156l-5p	LOC_Os12g28810.1	161	-21.79	161	-21.79	1059488	21	3355	3130
miR156l-5p	LOC_Os12g29660.1	326	-46.36	165	-24.54	1059576	21	2708	1450 1364
miR156l-5p	LOC_Os12g30200.1	161	-24.44	161	-24.44	1059633	21	3339	482
miR156l-5p	LOC_Os12g30350.1	164	-27.58	164	-27.58	1059646	21	3123	1706
miR156l-5p	LOC_Os12g31570.1	156	-24.99	156	-24.99	1059762	21	5328	482
miR156l-5p	LOC_Os12g31748.2	153	-21.31	153	-21.31	1059779	21	1040	547
miR156l-5p	LOC_Os12g32770.1	163	-20.18	163	-20.18	1059890	21	786	9
miR156l-5p	LOC_Os12g32810.1	152	-21.88	152	-21.88	1059893	21	537	126
miR156l-5p	LOC_Os12g34900.1	156	-25.16	156	-25.16	1060122	21	2613	400
miR156l-5p	LOC_Os12g36430.1	145	-20.53	145	-20.53	1060285	21	3661	1671
miR156l-5p	LOC_Os12g36430.2	145	-20.53	145	-20.53	1060286	21	3233	1671
miR156l-5p	LOC_Os12g36760.1	159	-20.57	159	-20.57	1060322	21	4343	889
miR156l-5p	LOC_Os12g37640.2	166	-21.2	166	-21.2	1060425	21	1502	1349
miR156l-5p	LOC_Os12g38030.1	168	-21.67	168	-21.67	1060469	21	5556	733
miR156l-5p	LOC_Os12g38910.1	161	-20.36	161	-20.36	1060571	21	2397	135
miR156l-5p	LOC_Os12g39120.1	163	-21.75	163	-21.75	1060593	21	2007	1329
miR156l-5p	LOC_Os12g39200.1	149	-21.76	149	-21.76	1060601	21	1950	1400

miR156l-5p	LOC_Os12g39200.2	149	-21.76	149	-21.76	1060602	21	1872	1322
miR156l-5p	LOC_Os12g39620.1	165	-28.95	165	-28.95	1060657	21	2998	1462
miR156l-5p	LOC_Os12g39620.2	327	-49.31	165	-28.95	1060658	21	3127	809 2523
miR156l-5p	LOC_Os12g39620.3	327	-49.31	165	-28.95	1060659	21	2812	494 2208
miR156l-5p	LOC_Os12g39620.4	327	-49.31	165	-28.95	1060660	21	2680	362 2076
miR156l-5p	LOC_Os12g39620.5	327	-49.31	165	-28.95	1060661	21	2768	450 2164
miR156l-5p	LOC_Os12g39630.1	162	-20.36	162	-20.36	1060662	21	2684	1068
miR156l-5p	LOC_Os12g39760.1	161	-21.79	161	-21.79	1060675	21	3288	3178
miR156l-5p	LOC_Os12g40250.1	161	-21.79	161	-21.79	1060729	21	2541	2431
miR156l-5p	LOC_Os12g40610.1	165	-21.22	165	-21.22	1060772	21	2865	2601
miR156l-5p	LOC_Os12g42130.1	140	-21.26	140	-21.26	1060962	21	1885	183
miR156l-5p	LOC_Os12g44330.1	165	-20.01	165	-20.01	1061211	21	3663	2941
miR156l-5p	LOC_Os12g44330.2	165	-20.01	165	-20.01	1061212	21	3147	2339
miR156l-5p	ChrUn.fgenesh.mRNA.82	157	-26.15	157	-26.15	1061305	21	1488	280
miR156l-5p	ChrUn.fgenesh.mRNA.85	164	-20.39	164	-20.39	1061308	21	3369	2765
miR156l-5p	ChrSy.fgenesh.mRNA.20	151	-22.56	151	-22.56	1061339	21	339	48
miR156l-5p	ChrSy.fgenesh.mRNA.40	156	-24.99	156	-24.99	1061359	21	1398	482
miR156l-5p	ChrSy.fgenesh.mRNA.48	157	-22.25	157	-22.25	1061367	21	3657	482
miR1428f-5p	LOC_Os01g07720.2	166	-20.8	166	-20.8	1062187	22	2121	409
miR1428f-5p	LOC_Os01g07720.4	166	-20.8	166	-20.8	1062188	22	1665	409
miR1428f-5p	LOC_Os01g07910.1	151	-23.93	151	-23.93	1062213	22	1421	2
miR1428f-5p	LOC_Os01g08190.1	175	-24.32	175	-24.32	1062242	22	3147	2431
miR1428f-5p	LOC_Os01g08190.2	175	-24.32	175	-24.32	1062243	22	3670	2954
miR1428f-5p	LOC_Os01g08190.3	175	-24.32	175	-24.32	1062244	22	3503	2787
miR1428f-5p	LOC_Os01g16180.1	150	-20.53	150	-20.53	1063155	22	5051	3501
miR1428f-5p	LOC_Os01g16180.2	150	-20.53	150	-20.53	1063156	22	4239	2695
miR1428f-5p	LOC_Os01g16180.3	150	-20.53	150	-20.53	1063157	22	4102	2552
miR1428f-5p	LOC_Os01g16180.4	150	-20.53	150	-20.53	1063158	22	4062	2512
miR1428f-5p	LOC_Os01g37470.1	164	-23.72	164	-23.72	1065124	22	2346	1665
miR1428f-5p	LOC_Os01g37470.2	164	-23.72	164	-23.72	1065125	22	2178	1665
miR1428f-5p	LOC_Os01g41280.1	153	-21.04	153	-21.04	1065546	22	1512	1012
miR1428f-5p	LOC_Os01g41950.1	160	-22.73	160	-22.73	1065614	22	3201	37

miR1428f-5p	LOC_Os01g43150.1	171	-24.51	171	-24.51	1065755	22	2473	482
miR1428f-5p	LOC_Os01g43150.2	171	-24.51	171	-24.51	1065756	22	1595	980
miR1428f-5p	LOC_Os01g44040.1	154	-20.72	154	-20.72	1065864	22	2600	2312
miR1428f-5p	LOC_Os01g44040.3	154	-20.72	154	-20.72	1065866	22	5158	4870
miR1428f-5p	LOC_Os01g44960.1	172	-23.83	172	-23.83	1065911	22	1364	557
miR1428f-5p	LOC_Os01g51820.1	154	-20.21	154	-20.21	1066704	22	5964	812
miR1428f-5p	LOC_Os01g54784.1	156	-21.74	156	-21.74	1067043	22	1778	1504
miR1428f-5p	LOC_Os01g56490.1	162	-21.18	162	-21.18	1067263	22	4246	1493
miR1428f-5p	LOC_Os01g56510.1	167	-22.42	167	-22.42	1067265	22	2451	1333
miR1428f-5p	LOC_Os01g56510.2	167	-22.42	167	-22.42	1067266	22	2675	1557
miR1428f-5p	LOC_Os01g56510.3	167	-22.42	167	-22.42	1067267	22	2574	1557
miR1428f-5p	LOC_Os01g56510.4	167	-22.42	167	-22.42	1067268	22	2538	1557
miR1428f-5p	LOC_Os01g56510.5	167	-22.42	167	-22.42	1067269	22	2584	1557
miR1428f-5p	LOC_Os01g57920.1	150	-23.88	150	-23.88	1067444	22	1721	873
miR1428f-5p	LOC_Os01g59490.1	160	-20.27	160	-20.27	1067626	22	1603	1336
miR1428f-5p	LOC_Os01g66920.1	150	-20.87	150	-20.87	1068541	22	2668	2323
miR1428f-5p	LOC_Os01g68680.1	175	-27.3	175	-27.3	1068759	22	3514	1597
miR1428f-5p	LOC_Os02g01180.1	165	-20.44	165	-20.44	1069458	22	3103	431
miR1428f-5p	LOC_Os02g01180.2	165	-20.44	165	-20.44	1069459	22	3149	428
miR1428f-5p	LOC_Os02g01870.1	154	-20.92	154	-20.92	1069542	22	1537	163
miR1428f-5p	LOC_Os02g04380.1	155	-23.71	155	-23.71	1069871	22	2250	1052
miR1428f-5p	LOC_Os02g04490.1	166	-20.87	166	-20.87	1069890	22	5722	673
miR1428f-5p	LOC_Os02g07760.2	150	-21.7	150	-21.7	1070311	22	2318	246
miR1428f-5p	LOC_Os02g18180.1	163	-22.22	163	-22.22	1071439	22	2734	2052
miR1428f-5p	LOC_Os02g25560.1	150	-20.84	150	-20.84	1072137	22	2388	273
miR1428f-5p	LOC_Os02g25930.1	162	-21.23	162	-21.23	1072178	22	3936	3338
miR1428f-5p	LOC_Os02g29300.1	160	-21.33	160	-21.33	1072551	22	2073	946
miR1428f-5p	LOC_Os02g29760.1	162	-22.81	162	-22.81	1072596	22	4224	1787
miR1428f-5p	LOC_Os02g36570.1	162	-21.03	162	-21.03	1073311	22	2858	916
miR1428f-5p	LOC_Os02g36640.1	178	-25.51	178	-25.51	1073319	22	4173	1308
miR1428f-5p	LOC_Os02g38650.1	157	-20.63	157	-20.63	1073568	22	3825	432
miR1428f-5p	LOC_Os02g56270.1	167	-21.14	167	-21.14	1075738	22	3780	1640

miR1428f-5p	LOC_Os02g57620.1	166	-21.4	166	-21.4	1075929	22	2615	547
miR1428f-5p	LOC_Os02g57650.1	172	-25.24	172	-25.24	1075935	22	2575	1208
miR1428f-5p	LOC_Os02g58410.1	162	-20.91	162	-20.91	1076032	22	6975	4739
miR1428f-5p	LOC_Os03g01014.1	157	-20.19	157	-20.19	1076090	22	2010	402
miR1428f-5p	LOC_Os03g02030.1	168	-21.73	168	-21.73	1076233	22	2177	1624
miR1428f-5p	LOC_Os03g02030.2	168	-21.73	168	-21.73	1076234	22	2378	1825
miR1428f-5p	LOC_Os03g07570.1	159	-20.6	159	-20.6	1076943	22	2073	683
miR1428f-5p	LOC_Os03g07570.4	159	-20.6	159	-20.6	1076945	22	2014	677
miR1428f-5p	LOC_Os03g08640.1	143	-21.53	143	-21.53	1077084	22	1446	672
miR1428f-5p	LOC_Os03g08640.2	143	-21.53	143	-21.53	1077085	22	1521	747
miR1428f-5p	LOC_Os03g10080.1	162	-23.49	162	-23.49	1077201	22	1588	148
miR1428f-5p	LOC_Os03g10080.3	162	-23.49	162	-23.49	1077202	22	1276	148
miR1428f-5p	LOC_Os03g10080.4	162	-23.49	162	-23.49	1077203	22	1152	148
miR1428f-5p	LOC_Os03g10080.5	162	-23.49	162	-23.49	1077204	22	1883	148
miR1428f-5p	LOC_Os03g11520.2	143	-21.27	143	-21.27	1077377	22	3426	1589
miR1428f-5p	LOC_Os03g12210.1	160	-21.6	160	-21.6	1077468	22	1972	1834
miR1428f-5p	LOC_Os03g12990.1	157	-21.41	157	-21.41	1077581	22	838	505
miR1428f-5p	LOC_Os03g17480.1	171	-21.43	171	-21.43	1078153	22	1069	1046
miR1428f-5p	LOC_Os03g17700.1	154	-21.28	154	-21.28	1078181	22	1601	1471
miR1428f-5p	LOC_Os03g18540.1	160	-21.52	160	-21.52	1078284	22	2756	386
miR1428f-5p	LOC_Os03g18540.2	160	-21.52	160	-21.52	1078285	22	2896	386
miR1428f-5p	LOC_Os03g18540.4	160	-21.52	160	-21.52	1078286	22	2555	386
miR1428f-5p	LOC_Os03g18760.1	162	-20.13	162	-20.13	1078309	22	603	277
miR1428f-5p	LOC_Os03g20700.1	167	-20.31	167	-20.31	1078546	22	5655	4580
miR1428f-5p	LOC_Os03g20700.2	167	-20.31	167	-20.31	1078547	22	4952	4580
miR1428f-5p	LOC_Os03g23860.1	154	-20.09	154	-20.09	1078866	22	5295	2585
miR1428f-5p	LOC_Os03g25945.1	158	-21.09	158	-21.09	1079085	22	5692	5288
miR1428f-5p	LOC_Os03g26044.1	161	-20.24	161	-20.24	1079098	22	3221	1222
miR1428f-5p	LOC_Os03g26260.1	164	-20.75	164	-20.75	1079116	22	2484	154
miR1428f-5p	LOC_Os03g33680.1	178	-25.71	178	-25.71	1079826	22	3450	1551
miR1428f-5p	LOC_Os03g52430.1	176	-23.22	176	-23.22	1081678	22	1441	967
miR1428f-5p	LOC_Os03g53150.2	151	-20.73	151	-20.73	1081762	22	1475	406

miR1428f-5p	LOC_Os03g53660.1	171	-24.54	171	-24.54	1081824	22	4950	3296
miR1428f-5p	LOC_Os03g53950.1	158	-21.21	158	-21.21	1081867	22	1864	1658
miR1428f-5p	LOC_Os03g60730.1	162	-24.82	162	-24.82	1082625	22	2824	2509
miR1428f-5p	LOC_Os03g60730.2	162	-24.82	162	-24.82	1082626	22	3061	2746
miR1428f-5p	LOC_Os03g62170.1	155	-23.38	155	-23.38	1082813	22	2567	722
miR1428f-5p	LOC_Os03g62170.2	155	-23.38	155	-23.38	1082814	22	2408	697
miR1428f-5p	LOC_Os03g62230.1	148	-25.75	148	-25.75	1082823	22	1325	1087
miR1428f-5p	LOC_Os04g02110.1	162	-21.68	162	-21.68	1083236	22	5222	4254
miR1428f-5p	LOC_Os04g02110.2	162	-21.68	162	-21.68	1083237	22	3912	2944
miR1428f-5p	LOC_Os04g02110.3	162	-21.68	162	-21.68	1083238	22	4947	4254
miR1428f-5p	LOC_Os04g06910.1	161	-22.49	161	-22.49	1083700	22	1917	1210
miR1428f-5p	LOC_Os04g10070.1	166	-24.4	166	-24.4	1083979	22	2577	1751
miR1428f-5p	LOC_Os04g11910.1	168	-20.33	168	-20.33	1084160	22	3351	2884
miR1428f-5p	LOC_Os04g12480.1	141	-20.79	141	-20.79	1084217	22	2449	1297
miR1428f-5p	LOC_Os04g17340.1	166	-24.4	166	-24.4	1084668	22	2628	2237
miR1428f-5p	LOC_Os04g18480.1	162	-22.83	162	-22.83	1084779	22	3927	1121
miR1428f-5p	LOC_Os04g19290.1	166	-23.15	166	-23.15	1084858	22	3618	665
miR1428f-5p	LOC_Os04g19520.1	166	-27.24	166	-27.24	1084880	22	732	194
miR1428f-5p	LOC_Os04g23210.1	165	-22.19	165	-22.19	1085237	22	3024	2133
miR1428f-5p	LOC_Os04g27720.1	170	-20.7	170	-20.7	1085680	22	1518	1258
miR1428f-5p	LOC_Os04g31740.1	174	-22.12	174	-22.12	1086073	22	189	128
miR1428f-5p	LOC_Os04g34984.1	160	-20.15	160	-20.15	1086428	22	2224	1542
miR1428f-5p	LOC_Os04g34984.2	160	-20.15	160	-20.15	1086429	22	2771	2100
miR1428f-5p	LOC_Os04g34984.3	160	-20.15	160	-20.15	1086430	22	2314	1542
miR1428f-5p	LOC_Os04g36660.1	157	-21.34	157	-21.34	1086569	22	2621	1515
miR1428f-5p	LOC_Os04g38090.1	158	-22.26	158	-22.26	1086670	22	3000	1369
miR1428f-5p	LOC_Os04g41410.1	169	-24.98	169	-24.98	1087083	22	1895	964
miR1428f-5p	LOC_Os04g43270.1	164	-20.33	164	-20.33	1087314	22	2391	41
miR1428f-5p	LOC_Os04g43340.1	159	-21.5	159	-21.5	1087322	22	3630	913
miR1428f-5p	LOC_Os04g43870.1	163	-23.65	163	-23.65	1087381	22	2919	2718
miR1428f-5p	LOC_Os04g48010.1	157	-24.87	157	-24.87	1087888	22	2595	911
miR1428f-5p	LOC_Os04g48010.2	157	-24.87	157	-24.87	1087889	22	2826	1178

miR1428f-5p	LOC_Os04g51910.1	171	-20.18	171	-20.18	1088313	22	3054	2078
miR1428f-5p	LOC_Os04g52100.1	150	-24.43	150	-24.43	1088336	22	1436	435
miR1428f-5p	LOC_Os04g53670.1	152	-20.03	152	-20.03	1088538	22	4138	999
miR1428f-5p	LOC_Os04g57739.2	158	-20.57	158	-20.57	1089047	22	1607	1142
miR1428f-5p	LOC_Os05g07030.1	174	-23.4	174	-23.4	1090030	22	2372	598
miR1428f-5p	LOC_Os05g07030.2	174	-23.4	174	-23.4	1090031	22	2311	537
miR1428f-5p	LOC_Os05g10770.1	167	-21.67	167	-21.67	1090426	22	4353	1475
miR1428f-5p	LOC_Os05g19390.1	168	-20.13	168	-20.13	1091277	22	2763	2090
miR1428f-5p	LOC_Os05g21180.1	157	-20.24	157	-20.24	1091471	22	1718	1453
miR1428f-5p	LOC_Os05g21180.2	157	-20.24	157	-20.24	1091472	22	1853	1588
miR1428f-5p	LOC_Os05g21180.3	157	-20.24	157	-20.24	1091473	22	2054	1796
miR1428f-5p	LOC_Os05g21180.4	157	-20.24	157	-20.24	1091474	22	1381	1123
miR1428f-5p	LOC_Os05g22930.1	158	-21.56	158	-21.56	1091554	22	1218	795
miR1428f-5p	LOC_Os05g33360.1	163	-20.95	163	-20.95	1092645	22	1199	1174
miR1428f-5p	LOC_Os05g38000.1	167	-23.84	167	-23.84	1093142	22	2218	1931
miR1428f-5p	LOC_Os05g38000.2	167	-23.84	167	-23.84	1093143	22	1024	737
miR1428f-5p	LOC_Os05g43850.1	165	-24.82	165	-24.82	1093788	22	1777	1382
miR1428f-5p	LOC_Os05g45990.1	158	-20.2	158	-20.2	1094052	22	2102	1985
miR1428f-5p	LOC_Os06g06940.1	157	-22.58	157	-22.58	1095488	22	2079	91
miR1428f-5p	LOC_Os06g06960.1	157	-20.34	157	-20.34	1095490	22	2678	334
miR1428f-5p	LOC_Os06g07860.1	172	-23.17	172	-23.17	1095589	22	1754	1457
miR1428f-5p	LOC_Os06g07960.1	172	-23.17	172	-23.17	1095590	22	1386	1154
miR1428f-5p	LOC_Os06g10770.1	171	-23.06	171	-23.06	1095924	22	1909	892
miR1428f-5p	LOC_Os06g19960.1	164	-20.36	164	-20.36	1096855	22	2091	994
miR1428f-5p	LOC_Os06g29020.3	184	-23.78	184	-23.78	1097703	22	3424	2433
miR1428f-5p	LOC_Os06g29430.1	169	-21.64	169	-21.64	1097748	22	5292	2991
miR1428f-5p	LOC_Os06g36770.1	167	-20.68	167	-20.68	1098485	22	2479	2428
miR1428f-5p	LOC_Os06g36770.2	167	-20.68	167	-20.68	1098486	22	2476	2425
miR1428f-5p	LOC_Os06g36770.3	167	-20.68	167	-20.68	1098487	22	2548	2497
miR1428f-5p	LOC_Os06g38764.1	157	-20.23	157	-20.23	1098690	22	925	643
miR1428f-5p	LOC_Os06g48880.1	170	-25.07	170	-25.07	1099882	22	1498	6
miR1428f-5p	LOC_Os06g50000.1	152	-20.31	152	-20.31	1100040	22	801	407

miR1428f-5p	LOC_Os07g02300.1	161	-22.04	161	-22.04	1100391	22	3409	3386
miR1428f-5p	LOC_Os07g02300.2	161	-22.04	161	-22.04	1100392	22	3487	3464
miR1428f-5p	LOC_Os07g04480.1	156	-21.19	156	-21.19	1100619	22	3346	364
miR1428f-5p	LOC_Os07g06644.1	166	-21.79	166	-21.79	1100853	22	1621	100
miR1428f-5p	LOC_Os07g06644.2	166	-21.79	166	-21.79	1100854	22	2578	100
miR1428f-5p	LOC_Os07g06644.3	166	-21.79	166	-21.79	1100855	22	2572	100
miR1428f-5p	LOC_Os07g14650.2	178	-22.4	178	-22.4	1101729	22	1084	496
miR1428f-5p	LOC_Os07g14960.1	158	-22.5	158	-22.5	1101759	22	1566	1375
miR1428f-5p	LOC_Os07g16590.1	154	-21.85	154	-21.85	1101916	22	5154	1942
miR1428f-5p	LOC_Os07g20544.1	167	-26.42	167	-26.42	1102259	22	2159	108
miR1428f-5p	LOC_Os07g20544.2	167	-26.42	167	-26.42	1102260	22	2153	108
miR1428f-5p	LOC_Os07g22550.1	166	-27.24	166	-27.24	1102368	22	4689	1955
miR1428f-5p	LOC_Os07g23990.1	160	-22.25	160	-22.25	1102532	22	6206	4870
miR1428f-5p	LOC_Os07g23990.2	160	-22.25	160	-22.25	1102533	22	6190	4854
miR1428f-5p	LOC_Os07g24400.1	166	-24.4	166	-24.4	1102573	22	2820	395
miR1428f-5p	LOC_Os07g24640.1	166	-24.4	166	-24.4	1102595	22	2016	1361
miR1428f-5p	LOC_Os07g27420.1	178	-25.71	178	-25.71	1102891	22	4821	2616
miR1428f-5p	LOC_Os07g29540.1	167	-20.33	167	-20.33	1103115	22	1332	1302
miR1428f-5p	LOC_Os07g40280.1	160	-20.6	160	-20.6	1104302	22	567	238
miR1428f-5p	LOC_Os07g44050.1	164	-20.21	164	-20.21	1104737	22	1469	270
miR1428f-5p	LOC_Os07g44050.2	164	-20.21	164	-20.21	1104738	22	1194	270
miR1428f-5p	LOC_Os08g01290.1	165	-20.35	165	-20.35	1105462	22	3383	1992
miR1428f-5p	LOC_Os08g01290.2	165	-20.35	165	-20.35	1105463	22	3355	1965
miR1428f-5p	LOC_Os08g01290.3	165	-20.35	165	-20.35	1105464	22	3118	1728
miR1428f-5p	LOC_Os08g01290.4	165	-20.35	165	-20.35	1105465	22	3105	1715
miR1428f-5p	LOC_Os08g01290.5	165	-20.35	165	-20.35	1105466	22	2877	1487
miR1428f-5p	LOC_Os08g01290.6	165	-20.35	165	-20.35	1105467	22	3258	1992
miR1428f-5p	LOC_Os08g01290.7	165	-20.35	165	-20.35	1105468	22	3288	1898
miR1428f-5p	LOC_Os08g02354.1	163	-20.26	163	-20.26	1105593	22	3391	344
miR1428f-5p	LOC_Os08g03682.1	159	-21.47	159	-21.47	1105748	22	2315	900
miR1428f-5p	LOC_Os08g04060.1	166	-27.24	166	-27.24	1105789	22	2442	641
miR1428f-5p	LOC_Os08g06110.3	152	-20.68	152	-20.68	1106019	22	2967	212

miR1428f-5p	LOC_Os08g06110.4	152	-20.68	152	-20.68	1106020	22	2968	213
miR1428f-5p	LOC_Os08g06110.5	152	-20.68	152	-20.68	1106021	22	2954	199
miR1428f-5p	LOC_Os08g10550.1	165	-21.33	165	-21.33	1106515	22	3404	2922
miR1428f-5p	LOC_Os08g12130.1	150	-20.91	150	-20.91	1106681	22	4047	470
miR1428f-5p	LOC_Os08g13070.1	166	-20.21	166	-20.21	1106783	22	1645	140
miR1428f-5p	LOC_Os08g14530.1	157	-20.63	157	-20.63	1106935	22	5388	1995
miR1428f-5p	LOC_Os08g14860.2	165	-21.54	165	-21.54	1106968	22	1430	1160
miR1428f-5p	LOC_Os08g17910.1	166	-24.8	166	-24.8	1107304	22	4248	2627
miR1428f-5p	LOC_Os08g21879.1	156	-20.44	156	-20.44	1107644	22	3108	1240
miR1428f-5p	LOC_Os08g22180.1	166	-24.4	166	-24.4	1107679	22	4056	1439
miR1428f-5p	LOC_Os08g22250.1	153	-21.19	153	-21.19	1107683	22	678	162
miR1428f-5p	LOC_Os08g23070.1	157	-20.22	157	-20.22	1107756	22	5397	2004
miR1428f-5p	LOC_Os08g24560.1	157	-20.22	157	-20.22	1107915	22	5397	2004
miR1428f-5p	LOC_Os08g25190.1	146	-20.24	146	-20.24	1107977	22	3900	3037
miR1428f-5p	LOC_Os08g32650.1	153	-20.35	153	-20.35	1108766	22	2917	1229
miR1428f-5p	LOC_Os08g33020.1	165	-21.79	165	-21.79	1108805	22	861	837
miR1428f-5p	LOC_Os08g33200.1	158	-21.77	158	-21.77	1108829	22	5684	2069
miR1428f-5p	LOC_Os09g01060.1	157	-20.63	157	-20.63	1110301	22	4839	1998
miR1428f-5p	LOC_Os09g01250.1	157	-20.63	157	-20.63	1110321	22	5433	2040
miR1428f-5p	LOC_Os09g01260.1	157	-20.63	157	-20.63	1110322	22	5433	2040
miR1428f-5p	LOC_Os09g05070.1	162	-22.83	162	-22.83	1110722	22	3018	1217
miR1428f-5p	LOC_Os09g07270.1	159	-20.79	159	-20.79	1110844	22	3726	3285
miR1428f-5p	LOC_Os09g08340.1	163	-26.34	163	-26.34	1110961	22	4083	1426
miR1428f-5p	LOC_Os09g10910.3	167	-24.59	167	-24.59	1111232	22	1408	1212
miR1428f-5p	LOC_Os09g12500.1	168	-20.02	168	-20.02	1111391	22	2271	2213
miR1428f-5p	LOC_Os09g12500.2	168	-20.02	168	-20.02	1111392	22	2264	2206
miR1428f-5p	LOC_Os09g13920.1	153	-21.4	153	-21.4	1111544	22	1268	833
miR1428f-5p	LOC_Os09g16380.1	174	-23.41	174	-23.41	1111810	22	4161	820
miR1428f-5p	LOC_Os09g17630.1	157	-21.87	157	-21.87	1111940	22	3190	225
miR1428f-5p	LOC_Os09g19830.1	168	-25.89	168	-25.89	1112104	22	3844	703
miR1428f-5p	LOC_Os09g21810.1	165	-23.83	165	-23.83	1112326	22	1551	450
miR1428f-5p	LOC_Os09g27660.2	164	-20.36	164	-20.36	1112912	22	3319	3138

miR1428f-5p	LOC_Os09g27660.3	164	-20.36	164	-20.36	1112913	22	3145	2964
miR1428f-5p	LOC_Os09g27660.4	164	-20.36	164	-20.36	1112914	22	1737	1556
miR1428f-5p	LOC_Os09g27660.5	164	-20.36	164	-20.36	1112915	22	2996	2815
miR1428f-5p	LOC_Os09g27660.6	164	-20.36	164	-20.36	1112916	22	3183	3002
miR1428f-5p	LOC_Os09g27660.7	164	-20.36	164	-20.36	1112917	22	3169	2988
miR1428f-5p	LOC_Os09g27660.8	164	-20.36	164	-20.36	1112918	22	3362	3181
miR1428f-5p	LOC_Os09g27660.10	164	-20.36	164	-20.36	1112920	22	3343	3162
miR1428f-5p	LOC_Os09g29080.1	147	-20.78	147	-20.78	1113091	22	971	448
miR1428f-5p	LOC_Os09g30270.1	147	-20.19	147	-20.19	1113235	22	1666	1055
miR1428f-5p	LOC_Os09g30270.2	147	-20.19	147	-20.19	1113236	22	1803	1055
miR1428f-5p	LOC_Os09g34010.1	158	-23.48	158	-23.48	1113604	22	2745	269
miR1428f-5p	LOC_Os09g39820.1	151	-20.32	151	-20.32	1114186	22	4734	656
miR1428f-5p	LOC_Os10g05960.1	160	-21.76	160	-21.76	1114722	22	279	139
miR1428f-5p	LOC_Os10g06760.1	162	-21.76	162	-21.76	1114802	22	3658	870
miR1428f-5p	LOC_Os10g08120.1	154	-21.85	154	-21.85	1114929	22	2592	2092
miR1428f-5p	LOC_Os10g12670.1	158	-20.95	158	-20.95	1115360	22	5481	2088
miR1428f-5p	LOC_Os10g12690.1	157	-20.63	157	-20.63	1115362	22	5415	2022
miR1428f-5p	LOC_Os10g12808.1	157	-20.63	157	-20.63	1115375	22	5523	2130
miR1428f-5p	LOC_Os10g13670.1	154	-21.71	154	-21.71	1115416	22	2473	1250
miR1428f-5p	LOC_Os10g15250.1	166	-27.24	166	-27.24	1115538	22	4992	2228
miR1428f-5p	LOC_Os10g17340.1	157	-20.63	157	-20.63	1115637	22	5412	2019
miR1428f-5p	LOC_Os10g24310.1	166	-27.64	166	-27.64	1116290	22	3051	1178
miR1428f-5p	LOC_Os10g25090.1	164	-20.95	164	-20.95	1116364	22	2780	247
miR1428f-5p	LOC_Os10g30590.2	160	-20.39	160	-20.39	1116886	22	3160	1759
miR1428f-5p	LOC_Os10g33770.1	158	-22.11	158	-22.11	1117207	22	2790	368
miR1428f-5p	LOC_Os10g33874.1	152	-20.28	152	-20.28	1117225	22	3261	1994
miR1428f-5p	LOC_Os10g35880.1	170	-22.01	170	-22.01	1117460	22	3510	2079
miR1428f-5p	LOC_Os10g37090.1	162	-21.98	162	-21.98	1117581	22	2039	2006
miR1428f-5p	LOC_Os10g37660.1	159	-24.5	159	-24.5	1117633	22	2050	2008
miR1428f-5p	LOC_Os11g01220.1	163	-21.46	163	-21.46	1118315	22	2732	116
miR1428f-5p	LOC_Os11g02480.1	156	-22.55	156	-22.55	1118467	22	1074	575
miR1428f-5p	LOC_Os11g02480.2	156	-22.55	156	-22.55	1118468	22	1074	575

miR1428f-5p	LOC_Os11g03010.1	166	-23.15	166	-23.15	1118526	22	2388	638
miR1428f-5p	LOC_Os11g05540.1	160	-21.16	160	-21.16	1118826	22	2201	2112
miR1428f-5p	LOC_Os11g22850.1	152	-20.46	152	-20.46	1120462	22	2655	319
miR1428f-5p	LOC_Os11g26940.1	149	-20.07	149	-20.07	1120818	22	462	137
miR1428f-5p	LOC_Os11g27760.1	150	-20.91	150	-20.91	1120898	22	4218	623
miR1428f-5p	LOC_Os11g32030.1	162	-21.91	162	-21.91	1121326	22	1328	1115
miR1428f-5p	LOC_Os11g33440.1	164	-23.13	164	-23.13	1121484	22	846	526
miR1428f-5p	LOC_Os11g35560.1	154	-23.34	154	-23.34	1121667	22	5289	1837
miR1428f-5p	LOC_Os11g36120.1	159	-23.08	159	-23.08	1121731	22	2268	987
miR1428f-5p	LOC_Os11g39290.1	158	-20.07	158	-20.07	1122070	22	2762	133
miR1428f-5p	LOC_Os11g47030.1	168	-24.55	168	-24.55	1122840	22	3321	2429
miR1428f-5p	LOC_Os11g47240.1	168	-24.55	168	-24.55	1122859	22	3347	2590
miR1428f-5p	LOC_Os12g01220.1	163	-21.46	163	-21.46	1123010	22	3620	718
miR1428f-5p	LOC_Os12g02210.1	159	-21.87	159	-21.87	1123129	22	684	375
miR1428f-5p	LOC_Os12g04570.1	153	-21.43	153	-21.43	1123419	22	711	620
miR1428f-5p	LOC_Os12g05110.1	154	-21.02	154	-21.02	1123489	22	2208	1395
miR1428f-5p	LOC_Os12g09680.1	154	-21.81	154	-21.81	1123992	22	1341	1127
miR1428f-5p	LOC_Os12g12010.1	165	-21.53	165	-21.53	1124224	22	3000	2041
miR1428f-5p	LOC_Os12g22050.1	160	-20.1	160	-20.1	1125167	22	2811	869
miR1428f-5p	LOC_Os12g35110.1	157	-20.63	157	-20.63	1126486	22	5271	2019
miR1428f-5p	LOC_Os12g38270.1	168	-22.54	168	-22.54	1126828	22	689	507
miR1428f-5p	LOC_Os12g38270.2	168	-22.54	168	-22.54	1126829	22	740	558
miR1428f-5p	LOC_Os12g39840.1	169	-22.66	169	-22.66	1127020	22	1966	1534
miR1428f-5p	LOC_Os12g43670.1	156	-21.81	156	-21.81	1127471	22	972	425
miR1428f-5p	ChrUn.fgenesh.mRNA.27	166	-24.4	166	-24.4	1127588	22	3765	1667
miR1428e-5p	LOC_Os01g07720.2	166	-20.8	166	-20.8	1128525	22	2121	409
miR1428e-5p	LOC_Os01g07720.4	166	-20.8	166	-20.8	1128526	22	1665	409
miR1428e-5p	LOC_Os01g07910.1	151	-23.93	151	-23.93	1128551	22	1421	2
miR1428e-5p	LOC_Os01g08190.1	175	-24.32	175	-24.32	1128580	22	3147	2431
miR1428e-5p	LOC_Os01g08190.2	175	-24.32	175	-24.32	1128581	22	3670	2954
miR1428e-5p	LOC_Os01g08190.3	175	-24.32	175	-24.32	1128582	22	3503	2787
miR1428e-5p	LOC_Os01g16180.1	150	-20.53	150	-20.53	1129493	22	5051	3501

miR1428e-5p	LOC_Os01g16180.2	150	-20.53	150	-20.53	1129494	22	4239	2695
miR1428e-5p	LOC_Os01g16180.3	150	-20.53	150	-20.53	1129495	22	4102	2552
miR1428e-5p	LOC_Os01g16180.4	150	-20.53	150	-20.53	1129496	22	4062	2512
miR1428e-5p	LOC_Os01g37470.1	164	-23.72	164	-23.72	1131462	22	2346	1665
miR1428e-5p	LOC_Os01g37470.2	164	-23.72	164	-23.72	1131463	22	2178	1665
miR1428e-5p	LOC_Os01g41280.1	153	-21.04	153	-21.04	1131884	22	1512	1012
miR1428e-5p	LOC_Os01g41950.1	160	-22.73	160	-22.73	1131952	22	3201	37
miR1428e-5p	LOC_Os01g43150.1	171	-24.51	171	-24.51	1132093	22	2473	482
miR1428e-5p	LOC_Os01g43150.2	171	-24.51	171	-24.51	1132094	22	1595	980
miR1428e-5p	LOC_Os01g44040.1	154	-20.72	154	-20.72	1132202	22	2600	2312
miR1428e-5p	LOC_Os01g44040.3	154	-20.72	154	-20.72	1132204	22	5158	4870
miR1428e-5p	LOC_Os01g44960.1	172	-23.83	172	-23.83	1132249	22	1364	557
miR1428e-5p	LOC_Os01g51820.1	154	-20.21	154	-20.21	1133042	22	5964	812
miR1428e-5p	LOC_Os01g54784.1	156	-21.74	156	-21.74	1133381	22	1778	1504
miR1428e-5p	LOC_Os01g56490.1	162	-21.18	162	-21.18	1133601	22	4246	1493
miR1428e-5p	LOC_Os01g56510.1	167	-22.42	167	-22.42	1133603	22	2451	1333
miR1428e-5p	LOC_Os01g56510.2	167	-22.42	167	-22.42	1133604	22	2675	1557
miR1428e-5p	LOC_Os01g56510.3	167	-22.42	167	-22.42	1133605	22	2574	1557
miR1428e-5p	LOC_Os01g56510.4	167	-22.42	167	-22.42	1133606	22	2538	1557
miR1428e-5p	LOC_Os01g56510.5	167	-22.42	167	-22.42	1133607	22	2584	1557
miR1428e-5p	LOC_Os01g57920.1	150	-23.88	150	-23.88	1133782	22	1721	873
miR1428e-5p	LOC_Os01g59490.1	160	-20.27	160	-20.27	1133964	22	1603	1336
miR1428e-5p	LOC_Os01g66920.1	150	-20.87	150	-20.87	1134879	22	2668	2323
miR1428e-5p	LOC_Os01g68680.1	175	-27.3	175	-27.3	1135097	22	3514	1597
miR1428e-5p	LOC_Os02g01180.1	165	-20.44	165	-20.44	1135796	22	3103	431
miR1428e-5p	LOC_Os02g01180.2	165	-20.44	165	-20.44	1135797	22	3149	428
miR1428e-5p	LOC_Os02g01870.1	154	-20.92	154	-20.92	1135880	22	1537	163
miR1428e-5p	LOC_Os02g04380.1	155	-23.71	155	-23.71	1136209	22	2250	1052
miR1428e-5p	LOC_Os02g04490.1	166	-20.87	166	-20.87	1136228	22	5722	673
miR1428e-5p	LOC_Os02g07760.2	150	-21.7	150	-21.7	1136649	22	2318	246
miR1428e-5p	LOC_Os02g18180.1	163	-22.22	163	-22.22	1137777	22	2734	2052
miR1428e-5p	LOC_Os02g25560.1	150	-20.84	150	-20.84	1138475	22	2388	273

miR1428e-5p	LOC_Os02g25930.1	162	-21.23	162	-21.23	1138516	22	3936	3338
miR1428e-5p	LOC_Os02g29300.1	160	-21.33	160	-21.33	1138889	22	2073	946
miR1428e-5p	LOC_Os02g29760.1	162	-22.81	162	-22.81	1138934	22	4224	1787
miR1428e-5p	LOC_Os02g36570.1	162	-21.03	162	-21.03	1139649	22	2858	916
miR1428e-5p	LOC_Os02g36640.1	178	-25.51	178	-25.51	1139657	22	4173	1308
miR1428e-5p	LOC_Os02g38650.1	157	-20.63	157	-20.63	1139906	22	3825	432
miR1428e-5p	LOC_Os02g56270.1	167	-21.14	167	-21.14	1142076	22	3780	1640
miR1428e-5p	LOC_Os02g57620.1	166	-21.4	166	-21.4	1142267	22	2615	547
miR1428e-5p	LOC_Os02g57650.1	172	-25.24	172	-25.24	1142273	22	2575	1208
miR1428e-5p	LOC_Os02g58410.1	162	-20.91	162	-20.91	1142370	22	6975	4739
miR1428e-5p	LOC_Os03g01014.1	157	-20.19	157	-20.19	1142428	22	2010	402
miR1428e-5p	LOC_Os03g02030.1	168	-21.73	168	-21.73	1142571	22	2177	1624
miR1428e-5p	LOC_Os03g02030.2	168	-21.73	168	-21.73	1142572	22	2378	1825
miR1428e-5p	LOC_Os03g07570.1	159	-20.6	159	-20.6	1143281	22	2073	683
miR1428e-5p	LOC_Os03g07570.4	159	-20.6	159	-20.6	1143283	22	2014	677
miR1428e-5p	LOC_Os03g08640.1	143	-21.53	143	-21.53	1143422	22	1446	672
miR1428e-5p	LOC_Os03g08640.2	143	-21.53	143	-21.53	1143423	22	1521	747
miR1428e-5p	LOC_Os03g10080.1	162	-23.49	162	-23.49	1143539	22	1588	148
miR1428e-5p	LOC_Os03g10080.3	162	-23.49	162	-23.49	1143540	22	1276	148
miR1428e-5p	LOC_Os03g10080.4	162	-23.49	162	-23.49	1143541	22	1152	148
miR1428e-5p	LOC_Os03g10080.5	162	-23.49	162	-23.49	1143542	22	1883	148
miR1428e-5p	LOC_Os03g11520.2	143	-21.27	143	-21.27	1143715	22	3426	1589
miR1428e-5p	LOC_Os03g12210.1	160	-21.6	160	-21.6	1143806	22	1972	1834
miR1428e-5p	LOC_Os03g12990.1	157	-21.41	157	-21.41	1143919	22	838	505
miR1428e-5p	LOC_Os03g17480.1	171	-21.43	171	-21.43	1144491	22	1069	1046
miR1428e-5p	LOC_Os03g17700.1	154	-21.28	154	-21.28	1144519	22	1601	1471
miR1428e-5p	LOC_Os03g18540.1	160	-21.52	160	-21.52	1144622	22	2756	386
miR1428e-5p	LOC_Os03g18540.2	160	-21.52	160	-21.52	1144623	22	2896	386
miR1428e-5p	LOC_Os03g18540.4	160	-21.52	160	-21.52	1144624	22	2555	386
miR1428e-5p	LOC_Os03g18760.1	162	-20.13	162	-20.13	1144647	22	603	277
miR1428e-5p	LOC_Os03g20700.1	167	-20.31	167	-20.31	1144884	22	5655	4580
miR1428e-5p	LOC_Os03g20700.2	167	-20.31	167	-20.31	1144885	22	4952	4580

miR1428e-5p	LOC_Os03g23860.1	154	-20.09	154	-20.09	1145204	22	5295	2585
miR1428e-5p	LOC_Os03g25945.1	158	-21.09	158	-21.09	1145423	22	5692	5288
miR1428e-5p	LOC_Os03g26044.1	161	-20.24	161	-20.24	1145436	22	3221	1222
miR1428e-5p	LOC_Os03g26260.1	164	-20.75	164	-20.75	1145454	22	2484	154
miR1428e-5p	LOC_Os03g33680.1	178	-25.71	178	-25.71	1146164	22	3450	1551
miR1428e-5p	LOC_Os03g52430.1	176	-23.22	176	-23.22	1148016	22	1441	967
miR1428e-5p	LOC_Os03g53150.2	151	-20.73	151	-20.73	1148100	22	1475	406
miR1428e-5p	LOC_Os03g53660.1	171	-24.54	171	-24.54	1148162	22	4950	3296
miR1428e-5p	LOC_Os03g53950.1	158	-21.21	158	-21.21	1148205	22	1864	1658
miR1428e-5p	LOC_Os03g60730.1	162	-24.82	162	-24.82	1148963	22	2824	2509
miR1428e-5p	LOC_Os03g60730.2	162	-24.82	162	-24.82	1148964	22	3061	2746
miR1428e-5p	LOC_Os03g62170.1	155	-23.38	155	-23.38	1149151	22	2567	722
miR1428e-5p	LOC_Os03g62170.2	155	-23.38	155	-23.38	1149152	22	2408	697
miR1428e-5p	LOC_Os03g62230.1	148	-25.75	148	-25.75	1149161	22	1325	1087
miR1428e-5p	LOC_Os04g02110.1	162	-21.68	162	-21.68	1149574	22	5222	4254
miR1428e-5p	LOC_Os04g02110.2	162	-21.68	162	-21.68	1149575	22	3912	2944
miR1428e-5p	LOC_Os04g02110.3	162	-21.68	162	-21.68	1149576	22	4947	4254
miR1428e-5p	LOC_Os04g06910.1	161	-22.49	161	-22.49	1150038	22	1917	1210
miR1428e-5p	LOC_Os04g10070.1	166	-24.4	166	-24.4	1150317	22	2577	1751
miR1428e-5p	LOC_Os04g11910.1	168	-20.33	168	-20.33	1150498	22	3351	2884
miR1428e-5p	LOC_Os04g12480.1	141	-20.79	141	-20.79	1150555	22	2449	1297
miR1428e-5p	LOC_Os04g17340.1	166	-24.4	166	-24.4	1151006	22	2628	2237
miR1428e-5p	LOC_Os04g18480.1	162	-22.83	162	-22.83	1151117	22	3927	1121
miR1428e-5p	LOC_Os04g19290.1	166	-23.15	166	-23.15	1151196	22	3618	665
miR1428e-5p	LOC_Os04g19520.1	166	-27.24	166	-27.24	1151218	22	732	194
miR1428e-5p	LOC_Os04g23210.1	165	-22.19	165	-22.19	1151575	22	3024	2133
miR1428e-5p	LOC_Os04g27720.1	170	-20.7	170	-20.7	1152018	22	1518	1258
miR1428e-5p	LOC_Os04g31740.1	174	-22.12	174	-22.12	1152411	22	189	128
miR1428e-5p	LOC_Os04g34984.1	160	-20.15	160	-20.15	1152766	22	2224	1542
miR1428e-5p	LOC_Os04g34984.2	160	-20.15	160	-20.15	1152767	22	2771	2100
miR1428e-5p	LOC_Os04g34984.3	160	-20.15	160	-20.15	1152768	22	2314	1542
miR1428e-5p	LOC_Os04g36660.1	157	-21.34	157	-21.34	1152907	22	2621	1515

miR1428e-5p	LOC_Os04g38090.1	158	-22.26	158	-22.26	1153008	22	3000	1369
miR1428e-5p	LOC_Os04g41410.1	169	-24.98	169	-24.98	1153421	22	1895	964
miR1428e-5p	LOC_Os04g43270.1	164	-20.33	164	-20.33	1153652	22	2391	41
miR1428e-5p	LOC_Os04g43340.1	159	-21.5	159	-21.5	1153660	22	3630	913
miR1428e-5p	LOC_Os04g43870.1	163	-23.65	163	-23.65	1153719	22	2919	2718
miR1428e-5p	LOC_Os04g48010.1	157	-24.87	157	-24.87	1154226	22	2595	911
miR1428e-5p	LOC_Os04g48010.2	157	-24.87	157	-24.87	1154227	22	2826	1178
miR1428e-5p	LOC_Os04g51910.1	171	-20.18	171	-20.18	1154651	22	3054	2078
miR1428e-5p	LOC_Os04g52100.1	150	-24.43	150	-24.43	1154674	22	1436	435
miR1428e-5p	LOC_Os04g53670.1	152	-20.03	152	-20.03	1154876	22	4138	999
miR1428e-5p	LOC_Os04g57739.2	158	-20.57	158	-20.57	1155385	22	1607	1142
miR1428e-5p	LOC_Os05g07030.1	174	-23.4	174	-23.4	1156368	22	2372	598
miR1428e-5p	LOC_Os05g07030.2	174	-23.4	174	-23.4	1156369	22	2311	537
miR1428e-5p	LOC_Os05g10770.1	167	-21.67	167	-21.67	1156764	22	4353	1475
miR1428e-5p	LOC_Os05g19390.1	168	-20.13	168	-20.13	1157615	22	2763	2090
miR1428e-5p	LOC_Os05g21180.1	157	-20.24	157	-20.24	1157809	22	1718	1453
miR1428e-5p	LOC_Os05g21180.2	157	-20.24	157	-20.24	1157810	22	1853	1588
miR1428e-5p	LOC_Os05g21180.3	157	-20.24	157	-20.24	1157811	22	2054	1796
miR1428e-5p	LOC_Os05g21180.4	157	-20.24	157	-20.24	1157812	22	1381	1123
miR1428e-5p	LOC_Os05g22930.1	158	-21.56	158	-21.56	1157892	22	1218	795
miR1428e-5p	LOC_Os05g33360.1	163	-20.95	163	-20.95	1158983	22	1199	1174
miR1428e-5p	LOC_Os05g38000.1	167	-23.84	167	-23.84	1159480	22	2218	1931
miR1428e-5p	LOC_Os05g38000.2	167	-23.84	167	-23.84	1159481	22	1024	737
miR1428e-5p	LOC_Os05g43850.1	165	-24.82	165	-24.82	1160126	22	1777	1382
miR1428e-5p	LOC_Os05g45990.1	158	-20.2	158	-20.2	1160390	22	2102	1985
miR1428e-5p	LOC_Os06g06940.1	157	-22.58	157	-22.58	1161826	22	2079	91
miR1428e-5p	LOC_Os06g06960.1	157	-20.34	157	-20.34	1161828	22	2678	334
miR1428e-5p	LOC_Os06g07860.1	172	-23.17	172	-23.17	1161927	22	1754	1457
miR1428e-5p	LOC_Os06g07960.1	172	-23.17	172	-23.17	1161928	22	1386	1154
miR1428e-5p	LOC_Os06g10770.1	171	-23.06	171	-23.06	1162262	22	1909	892
miR1428e-5p	LOC_Os06g19960.1	164	-20.36	164	-20.36	1163193	22	2091	994
miR1428e-5p	LOC_Os06g29020.3	184	-23.78	184	-23.78	1164041	22	3424	2433

miR1428e-5p	LOC_Os06g29430.1	169	-21.64	169	-21.64	1164086	22	5292	2991
miR1428e-5p	LOC_Os06g36770.1	167	-20.68	167	-20.68	1164823	22	2479	2428
miR1428e-5p	LOC_Os06g36770.2	167	-20.68	167	-20.68	1164824	22	2476	2425
miR1428e-5p	LOC_Os06g36770.3	167	-20.68	167	-20.68	1164825	22	2548	2497
miR1428e-5p	LOC_Os06g38764.1	157	-20.23	157	-20.23	1165028	22	925	643
miR1428e-5p	LOC_Os06g48880.1	170	-25.07	170	-25.07	1166220	22	1498	6
miR1428e-5p	LOC_Os06g50000.1	152	-20.31	152	-20.31	1166378	22	801	407
miR1428e-5p	LOC_Os07g02300.1	161	-22.04	161	-22.04	1166729	22	3409	3386
miR1428e-5p	LOC_Os07g02300.2	161	-22.04	161	-22.04	1166730	22	3487	3464
miR1428e-5p	LOC_Os07g04480.1	156	-21.19	156	-21.19	1166957	22	3346	364
miR1428e-5p	LOC_Os07g06644.1	166	-21.79	166	-21.79	1167191	22	1621	100
miR1428e-5p	LOC_Os07g06644.2	166	-21.79	166	-21.79	1167192	22	2578	100
miR1428e-5p	LOC_Os07g06644.3	166	-21.79	166	-21.79	1167193	22	2572	100
miR1428e-5p	LOC_Os07g14650.2	178	-22.4	178	-22.4	1168067	22	1084	496
miR1428e-5p	LOC_Os07g14960.1	158	-22.5	158	-22.5	1168097	22	1566	1375
miR1428e-5p	LOC_Os07g16590.1	154	-21.85	154	-21.85	1168254	22	5154	1942
miR1428e-5p	LOC_Os07g20544.1	167	-26.42	167	-26.42	1168597	22	2159	108
miR1428e-5p	LOC_Os07g20544.2	167	-26.42	167	-26.42	1168598	22	2153	108
miR1428e-5p	LOC_Os07g22550.1	166	-27.24	166	-27.24	1168706	22	4689	1955
miR1428e-5p	LOC_Os07g23990.1	160	-22.25	160	-22.25	1168870	22	6206	4870
miR1428e-5p	LOC_Os07g23990.2	160	-22.25	160	-22.25	1168871	22	6190	4854
miR1428e-5p	LOC_Os07g24400.1	166	-24.4	166	-24.4	1168911	22	2820	395
miR1428e-5p	LOC_Os07g24640.1	166	-24.4	166	-24.4	1168933	22	2016	1361
miR1428e-5p	LOC_Os07g27420.1	178	-25.71	178	-25.71	1169229	22	4821	2616
miR1428e-5p	LOC_Os07g29540.1	167	-20.33	167	-20.33	1169453	22	1332	1302
miR1428e-5p	LOC_Os07g40280.1	160	-20.6	160	-20.6	1170640	22	567	238
miR1428e-5p	LOC_Os07g44050.1	164	-20.21	164	-20.21	1171075	22	1469	270
miR1428e-5p	LOC_Os07g44050.2	164	-20.21	164	-20.21	1171076	22	1194	270
miR1428e-5p	LOC_Os08g01290.1	165	-20.35	165	-20.35	1171800	22	3383	1992
miR1428e-5p	LOC_Os08g01290.2	165	-20.35	165	-20.35	1171801	22	3355	1965
miR1428e-5p	LOC_Os08g01290.3	165	-20.35	165	-20.35	1171802	22	3118	1728
miR1428e-5p	LOC_Os08g01290.4	165	-20.35	165	-20.35	1171803	22	3105	1715

miR1428e-5p	LOC_Os08g01290.5	165	-20.35	165	-20.35	1171804	22	2877	1487
miR1428e-5p	LOC_Os08g01290.6	165	-20.35	165	-20.35	1171805	22	3258	1992
miR1428e-5p	LOC_Os08g01290.7	165	-20.35	165	-20.35	1171806	22	3288	1898
miR1428e-5p	LOC_Os08g02354.1	163	-20.26	163	-20.26	1171931	22	3391	344
miR1428e-5p	LOC_Os08g03682.1	159	-21.47	159	-21.47	1172086	22	2315	900
miR1428e-5p	LOC_Os08g04060.1	166	-27.24	166	-27.24	1172127	22	2442	641
miR1428e-5p	LOC_Os08g06110.3	152	-20.68	152	-20.68	1172357	22	2967	212
miR1428e-5p	LOC_Os08g06110.4	152	-20.68	152	-20.68	1172358	22	2968	213
miR1428e-5p	LOC_Os08g06110.5	152	-20.68	152	-20.68	1172359	22	2954	199
miR1428e-5p	LOC_Os08g10550.1	165	-21.33	165	-21.33	1172853	22	3404	2922
miR1428e-5p	LOC_Os08g12130.1	150	-20.91	150	-20.91	1173019	22	4047	470
miR1428e-5p	LOC_Os08g13070.1	166	-20.21	166	-20.21	1173121	22	1645	140
miR1428e-5p	LOC_Os08g14530.1	157	-20.63	157	-20.63	1173273	22	5388	1995
miR1428e-5p	LOC_Os08g14860.2	165	-21.54	165	-21.54	1173306	22	1430	1160
miR1428e-5p	LOC_Os08g17910.1	166	-24.8	166	-24.8	1173642	22	4248	2627
miR1428e-5p	LOC_Os08g21879.1	156	-20.44	156	-20.44	1173982	22	3108	1240
miR1428e-5p	LOC_Os08g22180.1	166	-24.4	166	-24.4	1174017	22	4056	1439
miR1428e-5p	LOC_Os08g22250.1	153	-21.19	153	-21.19	1174021	22	678	162
miR1428e-5p	LOC_Os08g23070.1	157	-20.22	157	-20.22	1174094	22	5397	2004
miR1428e-5p	LOC_Os08g24560.1	157	-20.22	157	-20.22	1174253	22	5397	2004
miR1428e-5p	LOC_Os08g25190.1	146	-20.24	146	-20.24	1174315	22	3900	3037
miR1428e-5p	LOC_Os08g32650.1	153	-20.35	153	-20.35	1175104	22	2917	1229
miR1428e-5p	LOC_Os08g33020.1	165	-21.79	165	-21.79	1175143	22	861	837
miR1428e-5p	LOC_Os08g33200.1	158	-21.77	158	-21.77	1175167	22	5684	2069
miR1428e-5p	LOC_Os09g01060.1	157	-20.63	157	-20.63	1176639	22	4839	1998
miR1428e-5p	LOC_Os09g01250.1	157	-20.63	157	-20.63	1176659	22	5433	2040
miR1428e-5p	LOC_Os09g01260.1	157	-20.63	157	-20.63	1176660	22	5433	2040
miR1428e-5p	LOC_Os09g05070.1	162	-22.83	162	-22.83	1177060	22	3018	1217
miR1428e-5p	LOC_Os09g07270.1	159	-20.79	159	-20.79	1177182	22	3726	3285
miR1428e-5p	LOC_Os09g08340.1	163	-26.34	163	-26.34	1177299	22	4083	1426
miR1428e-5p	LOC_Os09g10910.3	167	-24.59	167	-24.59	1177570	22	1408	1212
miR1428e-5p	LOC_Os09g12500.1	168	-20.02	168	-20.02	1177729	22	2271	2213

miR1428e-5p	LOC_Os09g12500.2	168	-20.02	168	-20.02	1177730	22	2264	2206
miR1428e-5p	LOC_Os09g13920.1	153	-21.4	153	-21.4	1177882	22	1268	833
miR1428e-5p	LOC_Os09g16380.1	174	-23.41	174	-23.41	1178148	22	4161	820
miR1428e-5p	LOC_Os09g17630.1	157	-21.87	157	-21.87	1178278	22	3190	225
miR1428e-5p	LOC_Os09g19830.1	168	-25.89	168	-25.89	1178442	22	3844	703
miR1428e-5p	LOC_Os09g21810.1	165	-23.83	165	-23.83	1178664	22	1551	450
miR1428e-5p	LOC_Os09g27660.2	164	-20.36	164	-20.36	1179250	22	3319	3138
miR1428e-5p	LOC_Os09g27660.3	164	-20.36	164	-20.36	1179251	22	3145	2964
miR1428e-5p	LOC_Os09g27660.4	164	-20.36	164	-20.36	1179252	22	1737	1556
miR1428e-5p	LOC_Os09g27660.5	164	-20.36	164	-20.36	1179253	22	2996	2815
miR1428e-5p	LOC_Os09g27660.6	164	-20.36	164	-20.36	1179254	22	3183	3002
miR1428e-5p	LOC_Os09g27660.7	164	-20.36	164	-20.36	1179255	22	3169	2988
miR1428e-5p	LOC_Os09g27660.8	164	-20.36	164	-20.36	1179256	22	3362	3181
miR1428e-5p	LOC_Os09g27660.10	164	-20.36	164	-20.36	1179258	22	3343	3162
miR1428e-5p	LOC_Os09g29080.1	147	-20.78	147	-20.78	1179429	22	971	448
miR1428e-5p	LOC_Os09g30270.1	147	-20.19	147	-20.19	1179573	22	1666	1055
miR1428e-5p	LOC_Os09g30270.2	147	-20.19	147	-20.19	1179574	22	1803	1055
miR1428e-5p	LOC_Os09g34010.1	158	-23.48	158	-23.48	1179942	22	2745	269
miR1428e-5p	LOC_Os09g39820.1	151	-20.32	151	-20.32	1180524	22	4734	656
miR1428e-5p	LOC_Os10g05960.1	160	-21.76	160	-21.76	1181060	22	279	139
miR1428e-5p	LOC_Os10g06760.1	162	-21.76	162	-21.76	1181140	22	3658	870
miR1428e-5p	LOC_Os10g08120.1	154	-21.85	154	-21.85	1181267	22	2592	2092
miR1428e-5p	LOC_Os10g12670.1	158	-20.95	158	-20.95	1181698	22	5481	2088
miR1428e-5p	LOC_Os10g12690.1	157	-20.63	157	-20.63	1181700	22	5415	2022
miR1428e-5p	LOC_Os10g12808.1	157	-20.63	157	-20.63	1181713	22	5523	2130
miR1428e-5p	LOC_Os10g13670.1	154	-21.71	154	-21.71	1181754	22	2473	1250
miR1428e-5p	LOC_Os10g15250.1	166	-27.24	166	-27.24	1181876	22	4992	2228
miR1428e-5p	LOC_Os10g17340.1	157	-20.63	157	-20.63	1181975	22	5412	2019
miR1428e-5p	LOC_Os10g24310.1	166	-27.64	166	-27.64	1182628	22	3051	1178
miR1428e-5p	LOC_Os10g25090.1	164	-20.95	164	-20.95	1182702	22	2780	247
miR1428e-5p	LOC_Os10g30590.2	160	-20.39	160	-20.39	1183224	22	3160	1759
miR1428e-5p	LOC_Os10g33770.1	158	-22.11	158	-22.11	1183545	22	2790	368

miR1428e-5p	LOC_Os10g33874.1	152	-20.28	152	-20.28	1183563	22	3261	1994
miR1428e-5p	LOC_Os10g35880.1	170	-22.01	170	-22.01	1183798	22	3510	2079
miR1428e-5p	LOC_Os10g37090.1	162	-21.98	162	-21.98	1183919	22	2039	2006
miR1428e-5p	LOC_Os10g37660.1	159	-24.5	159	-24.5	1183971	22	2050	2008
miR1428e-5p	LOC_Os11g01220.1	163	-21.46	163	-21.46	1184653	22	2732	116
miR1428e-5p	LOC_Os11g02480.1	156	-22.55	156	-22.55	1184805	22	1074	575
miR1428e-5p	LOC_Os11g02480.2	156	-22.55	156	-22.55	1184806	22	1074	575
miR1428e-5p	LOC_Os11g03010.1	166	-23.15	166	-23.15	1184864	22	2388	638
miR1428e-5p	LOC_Os11g05540.1	160	-21.16	160	-21.16	1185164	22	2201	2112
miR1428e-5p	LOC_Os11g22850.1	152	-20.46	152	-20.46	1186800	22	2655	319
miR1428e-5p	LOC_Os11g26940.1	149	-20.07	149	-20.07	1187156	22	462	137
miR1428e-5p	LOC_Os11g27760.1	150	-20.91	150	-20.91	1187236	22	4218	623
miR1428e-5p	LOC_Os11g32030.1	162	-21.91	162	-21.91	1187664	22	1328	1115
miR1428e-5p	LOC_Os11g33440.1	164	-23.13	164	-23.13	1187822	22	846	526
miR1428e-5p	LOC_Os11g35560.1	154	-23.34	154	-23.34	1188005	22	5289	1837
miR1428e-5p	LOC_Os11g36120.1	159	-23.08	159	-23.08	1188069	22	2268	987
miR1428e-5p	LOC_Os11g39290.1	158	-20.07	158	-20.07	1188408	22	2762	133
miR1428e-5p	LOC_Os11g47030.1	168	-24.55	168	-24.55	1189178	22	3321	2429
miR1428e-5p	LOC_Os11g47240.1	168	-24.55	168	-24.55	1189197	22	3347	2590
miR1428e-5p	LOC_Os12g01220.1	163	-21.46	163	-21.46	1189348	22	3620	718
miR1428e-5p	LOC_Os12g02210.1	159	-21.87	159	-21.87	1189467	22	684	375
miR1428e-5p	LOC_Os12g04570.1	153	-21.43	153	-21.43	1189757	22	711	620
miR1428e-5p	LOC_Os12g05110.1	154	-21.02	154	-21.02	1189827	22	2208	1395
miR1428e-5p	LOC_Os12g09680.1	154	-21.81	154	-21.81	1190330	22	1341	1127
miR1428e-5p	LOC_Os12g12010.1	165	-21.53	165	-21.53	1190562	22	3000	2041
miR1428e-5p	LOC_Os12g22050.1	160	-20.1	160	-20.1	1191505	22	2811	869
miR1428e-5p	LOC_Os12g35110.1	157	-20.63	157	-20.63	1192824	22	5271	2019
miR1428e-5p	LOC_Os12g38270.1	168	-22.54	168	-22.54	1193166	22	689	507
miR1428e-5p	LOC_Os12g38270.2	168	-22.54	168	-22.54	1193167	22	740	558
miR1428e-5p	LOC_Os12g39840.1	169	-22.66	169	-22.66	1193358	22	1966	1534
miR1428e-5p	LOC_Os12g43670.1	156	-21.81	156	-21.81	1193809	22	972	425
miR1428e-5p	ChrUn.fgenesh.mRNA.27	166	-24.4	166	-24.4	1193926	22	3765	1667

miR1428d	LOC_Os01g14690.1	163	-20.08	163	-20.08	1195655	21	1077	620
miR1428d	LOC_Os01g14690.4	163	-20.08	163	-20.08	1195656	21	1035	620
miR1428d	LOC_Os01g39200.1	162	-20.65	162	-20.65	1197993	21	4230	325
miR1428d	LOC_Os01g40499.1	165	-21.58	165	-21.58	1198133	21	2923	2560
miR1428d	LOC_Os02g06830.1	170	-21.96	170	-21.96	1202858	21	3642	3168
miR1428d	LOC_Os02g06830.2	170	-21.96	170	-21.96	1202859	21	3528	3054
miR1428d	LOC_Os02g25720.1	157	-21.43	157	-21.43	1204831	21	2799	417
miR1428d	LOC_Os03g01016.1	178	-20.17	178	-20.17	1208768	21	1692	856
miR1428d	LOC_Os03g01016.2	178	-20.17	178	-20.17	1208769	21	1757	856
miR1428d	LOC_Os04g40370.1	157	-22.28	157	-22.28	1219621	21	2028	686
miR1428d	LOC_Os04g40370.2	157	-22.28	157	-22.28	1219622	21	2025	683
miR1428d	LOC_Os04g40370.3	157	-22.28	157	-22.28	1219623	21	1729	387
miR1428d	LOC_Os05g02010.1	162	-23.51	162	-23.51	1222116	21	1204	892
miR1428d	LOC_Os05g02010.2	162	-23.51	162	-23.51	1222117	21	1209	897
miR1428d	LOC_Os05g02010.3	162	-23.51	162	-23.51	1222118	21	1416	1089
miR1428d	LOC_Os05g02010.4	162	-23.51	162	-23.51	1222119	21	1045	718
miR1428d	LOC_Os06g41100.1	161	-20.21	161	-20.21	1231637	21	1892	1409
miR1428d	LOC_Os07g01060.1	166	-22.63	166	-22.63	1232922	21	2547	563
miR1428d	LOC_Os07g01060.2	166	-22.63	166	-22.63	1232923	21	2162	178
miR1428d	LOC_Os07g01060.3	166	-22.63	166	-22.63	1232924	21	2599	615
miR1428d	LOC_Os07g10080.1	162	-20.65	162	-20.65	1233906	21	5103	556
miR1428d	LOC_Os08g12010.1	162	-20.65	162	-20.65	1239346	21	1585	326
miR1428d	LOC_Os08g12020.1	162	-20.65	162	-20.65	1239347	21	7755	490
miR1428d	LOC_Os09g21390.1	160	-21.67	160	-21.67	1244957	21	2868	1577
miR1428d	LOC_Os09g37840.1	164	-20.67	164	-20.67	1246627	21	7305	4861
miR1428d	LOC_Os10g34884.1	182	-27.76	182	-27.76	1250009	21	3609	1335
miR1428d	LOC_Os11g20460.1	165	-20.19	165	-20.19	1252998	21	3966	2198
miR1428d	LOC_Os11g24990.1	162	-21.03	162	-21.03	1253308	21	5460	3613
miR1428d	LOC_Os11g44710.1	162	-20.65	162	-20.65	1255318	21	5625	1651
miR1428d	LOC_Os12g39210.1	166	-24.83	166	-24.83	1259617	21	1672	905
miR1428c	LOC_Os01g14690.1	163	-20.08	163	-20.08	1261993	21	1077	620
miR1428c	LOC_Os01g14690.4	163	-20.08	163	-20.08	1261994	21	1035	620

miR1428c	LOC_Os01g39200.1	162	-20.65	162	-20.65	1264331	21	4230	325
miR1428c	LOC_Os01g40499.1	165	-21.58	165	-21.58	1264471	21	2923	2560
miR1428c	LOC_Os02g06830.1	170	-21.96	170	-21.96	1269196	21	3642	3168
miR1428c	LOC_Os02g06830.2	170	-21.96	170	-21.96	1269197	21	3528	3054
miR1428c	LOC_Os02g25720.1	157	-21.43	157	-21.43	1271169	21	2799	417
miR1428c	LOC_Os03g01016.1	178	-20.17	178	-20.17	1275106	21	1692	856
miR1428c	LOC_Os03g01016.2	178	-20.17	178	-20.17	1275107	21	1757	856
miR1428c	LOC_Os04g40370.1	157	-22.28	157	-22.28	1285959	21	2028	686
miR1428c	LOC_Os04g40370.2	157	-22.28	157	-22.28	1285960	21	2025	683
miR1428c	LOC_Os04g40370.3	157	-22.28	157	-22.28	1285961	21	1729	387
miR1428c	LOC_Os05g02010.1	162	-23.51	162	-23.51	1288454	21	1204	892
miR1428c	LOC_Os05g02010.2	162	-23.51	162	-23.51	1288455	21	1209	897
miR1428c	LOC_Os05g02010.3	162	-23.51	162	-23.51	1288456	21	1416	1089
miR1428c	LOC_Os05g02010.4	162	-23.51	162	-23.51	1288457	21	1045	718
miR1428c	LOC_Os06g41100.1	161	-20.21	161	-20.21	1297975	21	1892	1409
miR1428c	LOC_Os07g01060.1	166	-22.63	166	-22.63	1299260	21	2547	563
miR1428c	LOC_Os07g01060.2	166	-22.63	166	-22.63	1299261	21	2162	178
miR1428c	LOC_Os07g01060.3	166	-22.63	166	-22.63	1299262	21	2599	615
miR1428c	LOC_Os07g10080.1	162	-20.65	162	-20.65	1300244	21	5103	556
miR1428c	LOC_Os08g12010.1	162	-20.65	162	-20.65	1305684	21	1585	326
miR1428c	LOC_Os08g12020.1	162	-20.65	162	-20.65	1305685	21	7755	490
miR1428c	LOC_Os09g21390.1	160	-21.67	160	-21.67	1311295	21	2868	1577
miR1428c	LOC_Os09g37840.1	164	-20.67	164	-20.67	1312965	21	7305	4861
miR1428c	LOC_Os10g34884.1	182	-27.76	182	-27.76	1316347	21	3609	1335
miR1428c	LOC_Os11g20460.1	165	-20.19	165	-20.19	1319336	21	3966	2198
miR1428c	LOC_Os11g24990.1	162	-21.03	162	-21.03	1319646	21	5460	3613
miR1428c	LOC_Os11g44710.1	162	-20.65	162	-20.65	1321656	21	5625	1651
miR1428c	LOC_Os12g39210.1	166	-24.83	166	-24.83	1325955	21	1672	905
miR1428b	LOC_Os01g14690.1	163	-20.08	163	-20.08	1328331	21	1077	620
miR1428b	LOC_Os01g14690.4	163	-20.08	163	-20.08	1328332	21	1035	620
miR1428b	LOC_Os01g39200.1	162	-20.65	162	-20.65	1330669	21	4230	325
miR1428b	LOC_Os01g40499.1	165	-21.58	165	-21.58	1330809	21	2923	2560

miR1428b	LOC_Os02g06830.1	170	-21.96	170	-21.96	1335534	21	3642	3168
miR1428b	LOC_Os02g06830.2	170	-21.96	170	-21.96	1335535	21	3528	3054
miR1428b	LOC_Os02g25720.1	157	-21.43	157	-21.43	1337507	21	2799	417
miR1428b	LOC_Os03g01016.1	178	-20.17	178	-20.17	1341444	21	1692	856
miR1428b	LOC_Os03g01016.2	178	-20.17	178	-20.17	1341445	21	1757	856
miR1428b	LOC_Os04g40370.1	157	-22.28	157	-22.28	1352297	21	2028	686
miR1428b	LOC_Os04g40370.2	157	-22.28	157	-22.28	1352298	21	2025	683
miR1428b	LOC_Os04g40370.3	157	-22.28	157	-22.28	1352299	21	1729	387
miR1428b	LOC_Os05g02010.1	162	-23.51	162	-23.51	1354792	21	1204	892
miR1428b	LOC_Os05g02010.2	162	-23.51	162	-23.51	1354793	21	1209	897
miR1428b	LOC_Os05g02010.3	162	-23.51	162	-23.51	1354794	21	1416	1089
miR1428b	LOC_Os05g02010.4	162	-23.51	162	-23.51	1354795	21	1045	718
miR1428b	LOC_Os06g41100.1	161	-20.21	161	-20.21	1364313	21	1892	1409
miR1428b	LOC_Os07g01060.1	166	-22.63	166	-22.63	1365598	21	2547	563
miR1428b	LOC_Os07g01060.2	166	-22.63	166	-22.63	1365599	21	2162	178
miR1428b	LOC_Os07g01060.3	166	-22.63	166	-22.63	1365600	21	2599	615
miR1428b	LOC_Os07g10080.1	162	-20.65	162	-20.65	1366582	21	5103	556
miR1428b	LOC_Os08g12010.1	162	-20.65	162	-20.65	1372022	21	1585	326
miR1428b	LOC_Os08g12020.1	162	-20.65	162	-20.65	1372023	21	7755	490
miR1428b	LOC_Os09g21390.1	160	-21.67	160	-21.67	1377633	21	2868	1577
miR1428b	LOC_Os09g37840.1	164	-20.67	164	-20.67	1379303	21	7305	4861
miR1428b	LOC_Os10g34884.1	182	-27.76	182	-27.76	1382685	21	3609	1335
miR1428b	LOC_Os11g20460.1	165	-20.19	165	-20.19	1385674	21	3966	2198
miR1428b	LOC_Os11g24990.1	162	-21.03	162	-21.03	1385984	21	5460	3613
miR1428b	LOC_Os11g44710.1	162	-20.65	162	-20.65	1387994	21	5625	1651
miR1428b	LOC_Os12g39210.1	166	-24.83	166	-24.83	1392293	21	1672	905
miR5799	LOC_Os01g01730.1	142	-20.19	142	-20.19	1393201	24	573	347
miR5799	LOC_Os01g02440.1	143	-20.06	143	-20.06	1393286	24	1686	943
miR5799	LOC_Os01g03570.1	143	-23.08	143	-23.08	1393407	24	2278	323
miR5799	LOC_Os01g03740.1	156	-22.04	156	-22.04	1393423	24	3175	142
miR5799	LOC_Os01g04490.1	150	-20.78	150	-20.78	1393505	24	1734	420
miR5799	LOC_Os01g04710.1	165	-23.15	165	-23.15	1393532	24	1986	1860

miR5799	LOC_Os01g05289.1	151	-21.66	151	-21.66	1393599	24	955	228
miR5799	LOC_Os01g09770.1	147	-21.08	147	-21.08	1394122	24	2078	1114
miR5799	LOC_Os01g12340.1	171	-21.97	171	-21.97	1394393	24	6243	4509
miR5799	LOC_Os01g13730.1	161	-21.99	161	-21.99	1394570	24	1948	435
miR5799	LOC_Os01g15039.1	159	-25.48	159	-25.48	1394713	24	2183	1060
miR5799	LOC_Os01g16310.1	145	-20.25	145	-20.25	1394864	24	3111	339
miR5799	LOC_Os01g16380.1	148	-21.11	148	-21.11	1394874	24	975	542
miR5799	LOC_Os01g16860.1	156	-20.87	156	-20.87	1394921	24	2673	498
miR5799	LOC_Os01g16960.1	147	-22.55	147	-22.55	1394937	24	2445	2149
miR5799	LOC_Os01g18110.1	160	-20.52	160	-20.52	1395008	24	1063	422
miR5799	LOC_Os01g18120.1	160	-22.13	160	-22.13	1395009	24	1467	434
miR5799	LOC_Os01g23760.1	144	-20.08	144	-20.08	1395545	24	1224	114
miR5799	LOC_Os01g27100.1	156	-21.02	156	-21.02	1395844	24	540	62
miR5799	LOC_Os01g38720.1	156	-22.6	156	-22.6	1396956	24	1080	918
miR5799	LOC_Os01g40430.1	146	-21.26	146	-21.26	1397142	24	933	759
miR5799	LOC_Os01g41930.1	145	-20.39	145	-20.39	1397303	24	3887	997
miR5799	LOC_Os01g42700.1	149	-21.45	149	-21.45	1397387	24	2576	2478
miR5799	LOC_Os01g42700.2	149	-21.45	149	-21.45	1397388	24	1875	1781
miR5799	LOC_Os01g42700.3	149	-21.45	149	-21.45	1397389	24	1748	1656
miR5799	LOC_Os01g42700.4	149	-21.45	149	-21.45	1397390	24	1755	1661
miR5799	LOC_Os01g42700.5	149	-21.45	149	-21.45	1397391	24	1837	1743
miR5799	LOC_Os01g43200.1	162	-20.92	162	-20.92	1397453	24	342	267
miR5799	LOC_Os01g43430.1	146	-21.12	146	-21.12	1397481	24	858	686
miR5799	LOC_Os01g46240.1	155	-24.06	155	-24.06	1397737	24	1077	635
miR5799	LOC_Os01g46270.1	157	-21.14	157	-21.14	1397740	24	1047	119
miR5799	LOC_Os01g48446.1	140	-21.18	140	-21.18	1398008	24	1319	385
miR5799	LOC_Os01g50230.1	142	-20.52	142	-20.52	1398207	24	2271	979
miR5799	LOC_Os01g52000.1	174	-24.26	174	-24.26	1398413	24	4302	2497
miR5799	LOC_Os01g56020.1	157	-22.6	157	-22.6	1398895	24	2595	2157
miR5799	LOC_Os01g56750.1	311	-43.38	158	-21.78	1398994	24	1466	1307 282
miR5799	LOC_Os01g57740.1	158	-22.27	158	-22.27	1399116	24	1443	4
miR5799	LOC_Os01g59060.1	151	-22.99	151	-22.99	1399267	24	756	234

miR5799	LOC_Os01g61044.1	162	-21.54	162	-21.54	1399491	24	1917	524
miR5799	LOC_Os01g61044.2	162	-21.54	162	-21.54	1399492	24	1812	524
miR5799	LOC_Os01g62200.1	143	-20.52	143	-20.52	1399646	24	1804	630
miR5799	LOC_Os01g62200.2	143	-20.52	143	-20.52	1399647	24	1574	400
miR5799	LOC_Os01g62520.1	162	-24.47	162	-24.47	1399693	24	1026	132
miR5799	LOC_Os01g62945.1	158	-20.03	158	-20.03	1399745	24	993	175
miR5799	LOC_Os01g63270.1	158	-20.9	158	-20.9	1399782	24	3019	342
miR5799	LOC_Os01g63270.2	158	-20.9	158	-20.9	1399783	24	3287	243
miR5799	LOC_Os01g64520.1	177	-23.77	177	-23.77	1399937	24	1570	95
miR5799	LOC_Os01g64520.2	177	-23.77	177	-23.77	1399938	24	1585	95
miR5799	LOC_Os01g65060.1	141	-20.39	141	-20.39	1400011	24	1743	96
miR5799	LOC_Os01g65330.1	150	-20.36	150	-20.36	1400044	24	2336	210
miR5799	LOC_Os01g65330.2	150	-20.36	150	-20.36	1400045	24	2422	210
miR5799	LOC_Os01g65790.1	141	-21.07	141	-21.07	1400105	24	2023	1014
miR5799	LOC_Os01g66140.1	158	-21.48	158	-21.48	1400147	24	3255	1391
miR5799	LOC_Os01g66530.1	149	-20.01	149	-20.01	1400187	24	904	449
miR5799	LOC_Os01g66590.1	169	-24.84	169	-24.84	1400192	24	1555	1147
miR5799	LOC_Os01g66590.2	169	-24.84	169	-24.84	1400193	24	1702	1294
miR5799	LOC_Os01g66590.3	169	-24.84	169	-24.84	1400194	24	1433	1147
miR5799	LOC_Os01g70190.1	175	-22.48	175	-22.48	1400581	24	2200	272
miR5799	LOC_Os01g71810.1	178	-21.89	178	-21.89	1400783	24	1211	88
miR5799	LOC_Os01g71820.1	174	-20.05	174	-20.05	1400784	24	1230	111
miR5799	LOC_Os01g72930.1	158	-25.59	158	-25.59	1400911	24	2817	424
miR5799	LOC_Os01g73250.1	171	-22.89	171	-22.89	1400949	24	1340	957
miR5799	LOC_Os02g10470.1	176	-21.3	176	-21.3	1402272	24	1227	634
miR5799	LOC_Os02g11070.1	171	-29.02	171	-29.02	1402354	24	2035	832
miR5799	LOC_Os02g11070.2	171	-29.02	171	-29.02	1402355	24	2054	851
miR5799	LOC_Os02g12310.1	144	-21.84	144	-21.84	1402438	24	1130	392
miR5799	LOC_Os02g13210.1	172	-23.19	172	-23.19	1402565	24	1650	1248
miR5799	LOC_Os02g13900.1	173	-23.73	173	-23.73	1402642	24	1456	269
miR5799	LOC_Os02g14760.1	321	-45.66	163	-24.15	1402734	24	1658	1394 456
miR5799	LOC_Os02g14760.2	321	-45.66	163	-24.15	1402735	24	1785	1521 456

miR5799	LOC_Os02g17304.1	176	-26.04	176	-26.04	1403026	24	3188	232
miR5799	LOC_Os02g18000.1	173	-21.65	173	-21.65	1403107	24	4212	1126
miR5799	LOC_Os02g18650.1	141	-23.13	141	-23.13	1403179	24	2015	1092
miR5799	LOC_Os02g20660.1	165	-23.72	165	-23.72	1403386	24	4584	1894
miR5799	LOC_Os02g25680.1	147	-23.19	147	-23.19	1403840	24	995	352
miR5799	LOC_Os02g25680.2	147	-23.19	147	-23.19	1403841	24	1096	339
miR5799	LOC_Os02g26060.1	152	-20.13	152	-20.13	1403878	24	3768	2073
miR5799	LOC_Os02g26190.1	157	-20.35	157	-20.35	1403891	24	6728	4956
miR5799	LOC_Os02g26190.2	157	-20.35	157	-20.35	1403892	24	6844	4956
miR5799	LOC_Os02g26290.1	154	-21.51	154	-21.51	1403902	24	1066	839
miR5799	LOC_Os02g27110.1	160	-20.08	160	-20.08	1404011	24	9893	4734
miR5799	LOC_Os02g32460.1	169	-21.68	169	-21.68	1404540	24	1743	542
miR5799	LOC_Os02g38790.1	155	-22.39	155	-22.39	1405274	24	1416	732
miR5799	LOC_Os02g40310.1	163	-21.47	163	-21.47	1405480	24	2601	1740
miR5799	LOC_Os02g41460.1	158	-20.96	158	-20.96	1405558	24	1212	480
miR5799	LOC_Os02g42310.1	154	-22.48	154	-22.48	1405658	24	1740	336
miR5799	LOC_Os02g42406.1	148	-21.01	148	-21.01	1405683	24	2204	2179
miR5799	LOC_Os02g42780.1	150	-23.42	150	-23.42	1405725	24	2128	1354
miR5799	LOC_Os02g43370.1	171	-22.08	171	-22.08	1405796	24	3207	782
miR5799	LOC_Os02g44680.1	145	-20.74	145	-20.74	1405977	24	1741	1286
miR5799	LOC_Os02g44930.1	165	-20.04	165	-20.04	1406010	24	1101	234
miR5799	LOC_Os02g45000.1	158	-23.08	158	-23.08	1406017	24	1626	678
miR5799	LOC_Os02g45920.1	149	-24.94	149	-24.94	1406127	24	2043	67
miR5799	LOC_Os02g47860.1	161	-23.06	161	-23.06	1406373	24	2753	1275
miR5799	LOC_Os02g47860.2	161	-23.06	161	-23.06	1406374	24	2645	1167
miR5799	LOC_Os02g50330.1	146	-21.11	146	-21.11	1406683	24	3602	1106
miR5799	LOC_Os02g51600.1	158	-22.36	158	-22.36	1406843	24	1899	1107
miR5799	LOC_Os02g51770.1	150	-20.82	150	-20.82	1406861	24	1695	1307
miR5799	LOC_Os02g51770.2	150	-20.82	150	-20.82	1406862	24	1513	1125
miR5799	LOC_Os02g51770.3	150	-20.82	150	-20.82	1406863	24	1803	1415
miR5799	LOC_Os02g51770.4	150	-20.82	150	-20.82	1406864	24	2338	1950
miR5799	LOC_Os02g51770.5	150	-20.82	150	-20.82	1406865	24	1669	1281

miR5799	LOC_Os02g56610.1	162	-24.32	162	-24.32	1407469	24	1587	689
miR5799	LOC_Os02g57810.1	149	-20.61	149	-20.61	1407646	24	1596	257
miR5799	LOC_Os02g58590.1	166	-25.94	166	-25.94	1407747	24	1347	81
miR5799	LOC_Os03g01360.1	172	-21.66	172	-21.66	1407829	24	2430	131
miR5799	LOC_Os03g01360.2	172	-21.66	172	-21.66	1407830	24	2753	131
miR5799	LOC_Os03g01360.3	172	-21.66	172	-21.66	1407831	24	2856	131
miR5799	LOC_Os03g01360.4	172	-21.66	172	-21.66	1407832	24	2852	131
miR5799	LOC_Os03g02920.1	148	-20.06	148	-20.06	1408051	24	1584	837
miR5799	LOC_Os03g05850.1	174	-24.26	174	-24.26	1408418	24	4230	2497
miR5799	LOC_Os03g08710.1	153	-23.41	153	-23.41	1408782	24	727	254
miR5799	LOC_Os03g08710.2	153	-23.41	153	-23.41	1408783	24	858	385
miR5799	LOC_Os03g09230.2	168	-20.31	168	-20.31	1408843	24	1083	55
miR5799	LOC_Os03g10230.1	159	-22.02	159	-22.02	1408912	24	2116	1047
miR5799	LOC_Os03g10950.1	146	-20.02	146	-20.02	1408998	24	1780	295
miR5799	LOC_Os03g11650.1	147	-20.12	147	-20.12	1409085	24	2283	1864
miR5799	LOC_Os03g12000.1	153	-22.49	153	-22.49	1409127	24	2130	2097
miR5799	LOC_Os03g14050.2	186	-23.8	186	-23.8	1409409	24	3093	1836
miR5799	LOC_Os03g16320.1	142	-20.52	142	-20.52	1409704	24	2442	982
miR5799	LOC_Os03g16334.1	146	-20.41	146	-20.41	1409705	24	2245	1380
miR5799	LOC_Os03g19670.1	159	-20.09	159	-20.09	1410109	24	1711	366
miR5799	LOC_Os03g19670.2	159	-20.09	159	-20.09	1410110	24	1801	366
miR5799	LOC_Os03g20680.1	146	-21.61	146	-21.61	1410234	24	1325	817
miR5799	LOC_Os03g21890.1	153	-20.13	153	-20.13	1410376	24	2577	1666
miR5799	LOC_Os03g22050.1	186	-32.28	186	-32.28	1410396	24	3760	692
miR5799	LOC_Os03g22050.2	186	-32.28	186	-32.28	1410397	24	3754	692
miR5799	LOC_Os03g22050.3	186	-32.28	186	-32.28	1410398	24	3723	692
miR5799	LOC_Os03g22050.4	186	-32.28	186	-32.28	1410399	24	2768	692
miR5799	LOC_Os03g24820.1	165	-23.37	165	-23.37	1410648	24	2553	654
miR5799	LOC_Os03g25750.1	162	-20.39	162	-20.39	1410756	24	1812	124
miR5799	LOC_Os03g28110.1	178	-26.01	178	-26.01	1411004	24	3912	2134
miR5799	LOC_Os03g28980.1	151	-20.81	151	-20.81	1411046	24	2622	362
miR5799	LOC_Os03g29180.1	156	-20.44	156	-20.44	1411067	24	1403	1348

miR5799	LOC_Os03g29540.2	172	-23.19	172	-23.19	1411108	24	4133	3093
miR5799	LOC_Os03g33230.1	149	-20.03	149	-20.03	1411474	24	4896	2
miR5799	LOC_Os03g35630.1	163	-22.63	163	-22.63	1411611	24	252	12
miR5799	LOC_Os03g36320.1	144	-21.72	144	-21.72	1411668	24	414	112
miR5799	LOC_Os03g37720.1	158	-20.62	158	-20.62	1411791	24	3801	2571
miR5799	LOC_Os03g38010.1	176	-21.37	176	-21.37	1411829	24	1813	577
miR5799	LOC_Os03g41060.1	160	-20.84	160	-20.84	1412113	24	773	631
miR5799	LOC_Os03g44360.1	155	-22.62	155	-22.62	1412453	24	3900	1128
miR5799	LOC_Os03g45050.1	152	-20.12	152	-20.12	1412539	24	3867	2190
miR5799	LOC_Os03g45290.1	149	-22.07	149	-22.07	1412558	24	1671	1459
miR5799	LOC_Os03g45690.1	162	-22.99	162	-22.99	1412592	24	831	624
miR5799	LOC_Os03g45990.1	185	-27.22	185	-27.22	1412624	24	4865	2013
miR5799	LOC_Os03g46250.1	179	-23.02	179	-23.02	1412653	24	991	941
miR5799	LOC_Os03g46400.1	165	-22.47	165	-22.47	1412670	24	2278	2207
miR5799	LOC_Os03g47034.1	148	-23.17	148	-23.17	1412737	24	574	337
miR5799	LOC_Os03g51080.1	148	-21.33	148	-21.33	1413215	24	2184	239
miR5799	LOC_Os03g51330.1	154	-20.57	154	-20.57	1413242	24	2547	827
miR5799	LOC_Os03g56270.1	149	-20.95	149	-20.95	1413778	24	3741	1323
miR5799	LOC_Os03g56270.2	149	-20.95	149	-20.95	1413779	24	3306	1317
miR5799	LOC_Os03g56270.3	149	-20.95	149	-20.95	1413780	24	3553	1317
miR5799	LOC_Os03g56910.1	165	-21.44	165	-21.44	1413869	24	3408	2958
miR5799	LOC_Os03g60570.1	143	-21.25	143	-21.25	1414297	24	915	244
miR5799	LOC_Os03g61319.1	160	-21.1	160	-21.1	1414392	24	951	88
miR5799	LOC_Os03g63910.1	141	-20.08	141	-20.08	1414734	24	2793	924
miR5799	LOC_Os04g01790.1	155	-20.03	155	-20.03	1414890	24	1710	12
miR5799	LOC_Os04g05280.1	142	-21.25	142	-21.25	1415240	24	441	328
miR5799	LOC_Os04g05310.1	142	-21.25	142	-21.25	1415243	24	2100	328
miR5799	LOC_Os04g06030.1	154	-20.04	154	-20.04	1415309	24	2171	1564
miR5799	LOC_Os04g06290.1	162	-24.47	162	-24.47	1415331	24	2298	621
miR5799	LOC_Os04g09580.1	161	-21.03	161	-21.03	1415623	24	6520	914
miR5799	LOC_Os04g09570.1	161	-21.03	161	-21.03	1415624	24	1151	913
miR5799	LOC_Os04g10270.1	142	-23.72	142	-23.72	1415691	24	2652	807

miR5799	LOC_Os04g16590.1	153	-20.46	153	-20.46	1416249	24	1536	181
miR5799	LOC_Os04g17540.1	171	-20.32	171	-20.32	1416378	24	1263	602
miR5799	LOC_Os04g19760.1	165	-20.97	165	-20.97	1416595	24	2643	1883
miR5799	LOC_Os04g20474.1	318	-50.89	178	-30.28	1416665	24	4313	2416 167
miR5799	LOC_Os04g20474.2	318	-50.89	178	-30.28	1416666	24	4181	2285 167
miR5799	LOC_Os04g20474.3	178	-30.28	178	-30.28	1416667	24	3843	1947
miR5799	LOC_Os04g20474.4	318	-50.89	178	-30.28	1416668	24	4085	2189 167
miR5799	LOC_Os04g20630.1	158	-22.63	158	-22.63	1416682	24	615	27
miR5799	LOC_Os04g25800.1	145	-21.15	145	-21.15	1417185	24	1661	288
miR5799	LOC_Os04g29650.1	160	-21	160	-21	1417548	24	3198	524
miR5799	LOC_Os04g30420.1	157	-23.88	157	-23.88	1417621	24	1426	91
miR5799	LOC_Os04g30470.1	156	-20.31	156	-20.31	1417626	24	1887	177
miR5799	LOC_Os04g30790.1	158	-23.64	158	-23.64	1417659	24	1530	1402
miR5799	LOC_Os04g30790.2	158	-23.64	158	-23.64	1417660	24	1716	1588
miR5799	LOC_Os04g33720.1	182	-28.11	182	-28.11	1417990	24	2288	1952
miR5799	LOC_Os04g33780.1	194	-30.19	194	-30.19	1417994	24	2679	2628
miR5799	LOC_Os04g33780.2	194	-30.19	194	-30.19	1417995	24	1565	1514
miR5799	LOC_Os04g35690.1	152	-20.56	152	-20.56	1418201	24	1443	862
miR5799	LOC_Os04g35700.1	153	-23.24	153	-23.24	1418202	24	2743	457
miR5799	LOC_Os04g37820.1	177	-21.28	177	-21.28	1418333	24	1715	1636
miR5799	LOC_Os04g38230.1	151	-21.82	151	-21.82	1418373	24	1473	301
miR5799	LOC_Os04g40920.1	166	-21.91	166	-21.91	1418711	24	1643	108
miR5799	LOC_Os04g40920.2	166	-21.91	166	-21.91	1418712	24	1664	108
miR5799	LOC_Os04g44354.3	171	-21.85	171	-21.85	1419125	24	1819	1701
miR5799	LOC_Os04g44370.1	165	-21.45	165	-21.45	1419127	24	4962	2997
miR5799	LOC_Os04g45700.1	157	-22.27	157	-22.27	1419289	24	1119	10
miR5799	LOC_Os04g48380.1	176	-23.5	176	-23.5	1419621	24	900	653
miR5799	LOC_Os04g49194.1	145	-20.06	145	-20.06	1419714	24	1366	49
miR5799	LOC_Os04g50840.1	150	-23.33	150	-23.33	1419856	24	2331	871
miR5799	LOC_Os04g51120.1	155	-21.58	155	-21.58	1419889	24	2391	2232
miR5799	LOC_Os04g51120.3	155	-21.58	155	-21.58	1419890	24	2623	2464
miR5799	LOC_Os04g51740.1	154	-22.69	154	-22.69	1419970	24	5280	2697

miR5799	LOC_Os04g55920.1	330	-42.34	165	-21.17	1420490	24	1205	1035 1153
miR5799	LOC_Os04g55920.2	330	-42.34	165	-21.17	1420491	24	1202	1032 1150
miR5799	LOC_Os04g55920.5	330	-42.34	165	-21.17	1420492	24	1036	866 984
miR5799	LOC_Os04g57810.1	157	-20.44	157	-20.44	1420747	24	1654	131
miR5799	LOC_Os04g57810.3	157	-20.44	157	-20.44	1420749	24	1852	128
miR5799	LOC_Os04g58220.1	146	-20.43	146	-20.43	1420797	24	1617	1458
miR5799	LOC_Os04g58730.1	186	-27.77	186	-27.77	1420862	24	3379	3020
miR5799	LOC_Os04g58770.1	166	-24.95	166	-24.95	1420870	24	651	10
miR5799	LOC_Os04g59460.1	159	-20.83	159	-20.83	1420947	24	5636	623
miR5799	LOC_Os05g02620.1	175	-21.23	175	-21.23	1421210	24	782	492
miR5799	LOC_Os05g02790.1	169	-20.16	169	-20.16	1421232	24	3105	1712
miR5799	LOC_Os05g03550.1	170	-21.81	170	-21.81	1421322	24	5167	1234
miR5799	LOC_Os05g03620.1	149	-21.75	149	-21.75	1421330	24	2731	195
miR5799	LOC_Os05g04860.1	156	-20.42	156	-20.42	1421466	24	1464	1084
miR5799	LOC_Os05g04860.2	156	-20.42	156	-20.42	1421467	24	2168	1085
miR5799	LOC_Os05g05080.1	159	-21.12	159	-21.12	1421488	24	515	367
miR5799	LOC_Os05g06290.1	151	-21.95	151	-21.95	1421632	24	929	246
miR5799	LOC_Os05g07560.1	177	-23.77	177	-23.77	1421781	24	4291	3608
miR5799	LOC_Os05g11400.1	158	-21.72	158	-21.72	1422186	24	1329	125
miR5799	LOC_Os05g16970.1	144	-20.07	144	-20.07	1422719	24	2189	1885
miR5799	LOC_Os05g24960.1	162	-20.28	162	-20.28	1423466	24	1902	933
miR5799	LOC_Os05g26820.1	165	-20.37	165	-20.37	1423665	24	2111	1245
miR5799	LOC_Os05g34480.1	156	-24.46	156	-24.46	1424465	24	1281	159
miR5799	LOC_Os05g34480.2	156	-24.46	156	-24.46	1424466	24	848	166
miR5799	LOC_Os05g35860.1	156	-20.27	156	-20.27	1424628	24	3795	1246
miR5799	LOC_Os05g39040.1	166	-21.21	166	-21.21	1424970	24	3537	2377
miR5799	LOC_Os05g39540.1	182	-28.57	182	-28.57	1425033	24	1640	1308
miR5799	LOC_Os05g46550.1	178	-28.81	178	-28.81	1425816	24	1949	295
miR5799	LOC_Os05g46840.1	146	-21.8	146	-21.8	1425851	24	932	53
miR5799	LOC_Os05g47446.2	165	-21.5	165	-21.5	1425866	24	1063	603
miR5799	LOC_Os05g49120.1	175	-22.48	175	-22.48	1426082	24	2351	1734
miR5799	LOC_Os05g49120.2	175	-22.48	175	-22.48	1426083	24	2236	1619

miR5799	LOC_Os06g01210.1	173	-20.88	173	-20.88	1426484	24	863	97
miR5799	LOC_Os06g02460.1	171	-22.17	171	-22.17	1426629	24	1392	610
miR5799	LOC_Os06g02810.1	175	-20.46	175	-20.46	1426673	24	1042	929
miR5799	LOC_Os06g02830.1	159	-22.01	159	-22.01	1426675	24	2741	1369
miR5799	LOC_Os06g03676.1	161	-20.72	161	-20.72	1426764	24	2015	1322
miR5799	LOC_Os06g03820.1	173	-26.66	173	-26.66	1426783	24	1577	1232
miR5799	LOC_Os06g04840.1	185	-27.22	185	-27.22	1426906	24	3516	78
miR5799	LOC_Os06g04840.2	185	-27.22	185	-27.22	1426907	24	3505	78
miR5799	LOC_Os06g05300.1	152	-20.02	152	-20.02	1426965	24	1470	117
miR5799	LOC_Os06g06850.1	155	-22.61	155	-22.61	1427169	24	3344	2757
miR5799	LOC_Os06g07896.1	161	-25.44	161	-25.44	1427294	24	1401	552
miR5799	LOC_Os06g08960.1	141	-21.45	141	-21.45	1427409	24	1263	718
miR5799	LOC_Os06g09220.1	141	-21.45	141	-21.45	1427438	24	1725	892
miR5799	LOC_Os06g09230.2	150	-20.73	150	-20.73	1427439	24	3082	1133
miR5799	LOC_Os06g09717.1	145	-20.65	145	-20.65	1427498	24	624	143
miR5799	LOC_Os06g13190.1	168	-21.86	168	-21.86	1427897	24	789	454
miR5799	LOC_Os06g14460.1	149	-22.51	149	-22.51	1428039	24	1520	1236
miR5799	LOC_Os06g14780.1	172	-23.19	172	-23.19	1428074	24	2375	1176
miR5799	LOC_Os06g17090.1	141	-20.3	141	-20.3	1428309	24	1107	943
miR5799	LOC_Os06g17120.1	141	-20.3	141	-20.3	1428312	24	1452	939
miR5799	LOC_Os06g17880.1	181	-22.59	181	-22.59	1428386	24	3228	2608
miR5799	LOC_Os06g19660.2	147	-21.32	147	-21.32	1428517	24	3989	1840
miR5799	LOC_Os06g20090.1	153	-22.41	153	-22.41	1428561	24	5669	5025
miR5799	LOC_Os06g24390.1	158	-25.51	158	-25.51	1429020	24	243	48
miR5799	LOC_Os06g25670.1	142	-20.77	142	-20.77	1429147	24	2176	629
miR5799	LOC_Os06g29880.1	150	-21.85	150	-21.85	1429486	24	555	355
miR5799	LOC_Os06g30790.1	141	-20.74	141	-20.74	1429577	24	1428	1008
miR5799	LOC_Os06g31780.1	161	-21.79	161	-21.79	1429677	24	555	66
miR5799	LOC_Os06g40784.1	176	-26.31	176	-26.31	1430616	24	488	331
miR5799	LOC_Os06g45940.1	146	-20.45	146	-20.45	1431214	24	2954	1853
miR5799	LOC_Os06g45940.2	146	-20.45	146	-20.45	1431215	24	2885	1784
miR5799	LOC_Os07g02120.1	179	-24.31	179	-24.31	1432063	24	1609	1496

miR5799	LOC_Os07g02960.1	168	-21.08	168	-21.08	1432152	24	3538	3150
miR5799	LOC_Os07g02960.2	168	-21.08	168	-21.08	1432153	24	2741	2353
miR5799	LOC_Os07g02960.3	168	-21.08	168	-21.08	1432154	24	1913	1525
miR5799	LOC_Os07g05430.1	174	-24.26	174	-24.26	1432410	24	4302	2497
miR5799	LOC_Os07g06190.1	147	-21.59	147	-21.59	1432493	24	4374	378
miR5799	LOC_Os07g06300.1	147	-21.59	147	-21.59	1432501	24	4539	378
miR5799	LOC_Os07g07010.1	151	-22.46	151	-22.46	1432587	24	1974	747
miR5799	LOC_Os07g07490.1	146	-20.52	146	-20.52	1432642	24	2523	1894
miR5799	LOC_Os07g07490.2	146	-20.52	146	-20.52	1432643	24	2451	1822
miR5799	LOC_Os07g13690.1	148	-20.43	148	-20.43	1433306	24	1812	508
miR5799	LOC_Os07g26595.1	190	-27.66	190	-27.66	1434490	24	4143	3853
miR5799	LOC_Os07g27970.1	170	-22.23	170	-22.23	1434638	24	1424	118
miR5799	LOC_Os07g28570.1	176	-23.51	176	-23.51	1434700	24	2752	2
miR5799	LOC_Os07g33550.1	176	-23.51	176	-23.51	1435234	24	2846	1659
miR5799	LOC_Os07g35130.1	163	-21.07	163	-21.07	1435400	24	1812	862
miR5799	LOC_Os07g35650.1	155	-22.5	155	-22.5	1435463	24	2049	1936
miR5799	LOC_Os07g36960.1	161	-20.33	161	-20.33	1435612	24	1897	727
miR5799	LOC_Os07g36960.2	161	-20.33	161	-20.33	1435613	24	1712	727
miR5799	LOC_Os07g39290.1	175	-22.97	175	-22.97	1435876	24	1130	998
miR5799	LOC_Os07g39830.1	179	-24.18	179	-24.18	1435942	24	1535	1506
miR5799	LOC_Os07g40180.1	140	-20.13	140	-20.13	1435980	24	450	369
miR5799	LOC_Os07g42410.1	170	-20.63	170	-20.63	1436229	24	4850	737
miR5799	LOC_Os07g47100.2	160	-20.03	160	-20.03	1436785	24	2394	128
miR5799	LOC_Os07g47230.1	145	-21.1	145	-21.1	1436804	24	2022	402
miR5799	LOC_Os07g47760.1	180	-26.04	180	-26.04	1436884	24	644	488
miR5799	LOC_Os07g47920.1	152	-20.67	152	-20.67	1436904	24	2601	570
miR5799	LOC_Os07g49530.1	170	-21.76	170	-21.76	1437114	24	1623	464
miR5799	LOC_Os08g02996.1	157	-21.39	157	-21.39	1437353	24	2578	2318
miR5799	LOC_Os08g02996.2	157	-21.39	157	-21.39	1437354	24	2434	2174
miR5799	LOC_Os08g04170.1	159	-22.08	159	-22.08	1437495	24	3695	2639
miR5799	LOC_Os08g06260.1	163	-23.7	163	-23.7	1437728	24	237	174
miR5799	LOC_Os08g06600.1	176	-21.37	176	-21.37	1437779	24	1750	567

miR5799	LOC_Os08g12740.1	159	-23.24	159	-23.24	1438430	24	3264	3232
miR5799	LOC_Os08g12740.2	159	-23.24	159	-23.24	1438431	24	3063	3017
miR5799	LOC_Os08g13740.1	161	-26.07	161	-26.07	1438543	24	828	369
miR5799	LOC_Os08g17090.1	161	-21.45	161	-21.45	1438915	24	1212	468
miR5799	LOC_Os08g17540.1	157	-20.27	157	-20.27	1438956	24	597	434
miR5799	LOC_Os08g17890.1	158	-20.13	158	-20.13	1438992	24	4483	1164
miR5799	LOC_Os08g20660.1	172	-22.83	172	-22.83	1439230	24	3879	3777
miR5799	LOC_Os08g20660.2	172	-22.83	172	-22.83	1439231	24	3869	3767
miR5799	LOC_Os08g22760.1	152	-20.13	152	-20.13	1439417	24	5106	2898
miR5799	LOC_Os08g28710.1	171	-22.17	171	-22.17	1440034	24	2042	119
miR5799	LOC_Os08g30300.1	162	-24.47	162	-24.47	1440201	24	1440	543
miR5799	LOC_Os08g30900.1	152	-20.83	152	-20.83	1440260	24	4506	1059
miR5799	LOC_Os08g30900.2	152	-20.83	152	-20.83	1440261	24	3928	570
miR5799	LOC_Os08g31250.1	157	-22.82	157	-22.82	1440295	24	1068	371
miR5799	LOC_Os08g32630.1	161	-25	161	-25	1440451	24	2221	103
miR5799	LOC_Os08g32630.2	161	-25	161	-25	1440452	24	2234	103
miR5799	LOC_Os08g32630.3	161	-25	161	-25	1440453	24	2336	103
miR5799	LOC_Os08g32630.4	161	-25	161	-25	1440454	24	2312	103
miR5799	LOC_Os08g32630.5	161	-25	161	-25	1440455	24	2309	103
miR5799	LOC_Os08g37210.1	165	-23.76	165	-23.76	1440966	24	1722	1681
miR5799	LOC_Os08g39980.1	142	-20.55	142	-20.55	1441321	24	2545	1889
miR5799	LOC_Os08g40200.1	158	-21.36	158	-21.36	1441347	24	1667	448
miR5799	LOC_Os08g40490.1	152	-24.72	152	-24.72	1441374	24	1509	882
miR5799	LOC_Os08g43050.1	177	-23.01	177	-23.01	1441697	24	3966	2529
miR5799	LOC_Os08g43740.1	153	-20.21	153	-20.21	1441778	24	1383	503
miR5799	LOC_Os08g44280.1	171	-22.54	171	-22.54	1441837	24	2316	1136
miR5799	LOC_Os08g44280.2	171	-22.54	171	-22.54	1441838	24	1759	579
miR5799	LOC_Os08g44450.1	171	-22.17	171	-22.17	1441863	24	1203	983
miR5799	LOC_Os08g44450.2	171	-22.17	171	-22.17	1441864	24	1203	983
miR5799	LOC_Os08g44450.3	171	-22.17	171	-22.17	1441865	24	1276	1056
miR5799	LOC_Os09g07320.2	177	-23.39	177	-23.39	1442541	24	3042	1135
miR5799	LOC_Os09g07870.1	170	-20.4	170	-20.4	1442609	24	2412	2323

miR5799	LOC_Os09g09810.1	148	-20.24	148	-20.24	1442808	24	2490	2332
miR5799	LOC_Os09g09820.1	148	-20.24	148	-20.24	1442809	24	2964	237
miR5799	LOC_Os09g12400.1	147	-21.53	147	-21.53	1443071	24	765	616
miR5799	LOC_Os09g13180.1	159	-22.4	159	-22.4	1443155	24	4359	3568
miR5799	LOC_Os09g16380.1	150	-22.72	150	-22.72	1443500	24	4161	1248
miR5799	LOC_Os09g17180.1	160	-23.99	160	-23.99	1443585	24	414	88
miR5799	LOC_Os09g18020.1	158	-20.13	158	-20.13	1443672	24	3707	1623
miR5799	LOC_Os09g20240.1	161	-20.22	161	-20.22	1443841	24	1286	1152
miR5799	LOC_Os09g20240.2	161	-20.22	161	-20.22	1443842	24	903	769
miR5799	LOC_Os09g20970.1	150	-23.69	150	-23.69	1443927	24	2064	846
miR5799	LOC_Os09g23350.1	151	-22.89	151	-22.89	1444123	24	4231	1103
miR5799	LOC_Os09g23620.1	175	-21.53	175	-21.53	1444152	24	1404	73
miR5799	LOC_Os09g24720.1	157	-23.47	157	-23.47	1444272	24	633	282
miR5799	LOC_Os09g27100.1	163	-20.35	163	-20.35	1444540	24	1167	1101
miR5799	LOC_Os09g27220.1	164	-23.26	164	-23.26	1444554	24	1219	1042
miR5799	LOC_Os09g28600.1	149	-20.17	149	-20.17	1444727	24	1174	409
miR5799	LOC_Os09g29284.1	150	-20.69	150	-20.69	1444803	24	1711	440
miR5799	LOC_Os09g37540.1	186	-30.55	186	-30.55	1445608	24	1232	1172
miR5799	LOC_Os10g01470.1	148	-20.8	148	-20.8	1445954	24	1450	1211
miR5799	LOC_Os10g01984.1	147	-23.86	147	-23.86	1446007	24	5857	1732
miR5799	LOC_Os10g04600.1	153	-20.62	153	-20.62	1446270	24	1747	788
miR5799	LOC_Os10g04600.2	153	-20.62	153	-20.62	1446271	24	1802	788
miR5799	LOC_Os10g04600.3	153	-20.62	153	-20.62	1446272	24	1858	788
miR5799	LOC_Os10g04674.1	155	-20.67	155	-20.67	1446281	24	3397	1932
miR5799	LOC_Os10g04674.2	155	-20.67	155	-20.67	1446282	24	3381	1932
miR5799	LOC_Os10g04674.3	155	-20.67	155	-20.67	1446283	24	3441	1976
miR5799	LOC_Os10g05730.1	146	-21.8	146	-21.8	1446389	24	2799	1464
miR5799	LOC_Os10g05730.2	146	-21.8	146	-21.8	1446390	24	2429	1090
miR5799	LOC_Os10g06210.1	159	-20.67	159	-20.67	1446439	24	927	256
miR5799	LOC_Os10g07010.1	159	-23.66	159	-23.66	1446515	24	1896	675
miR5799	LOC_Os10g11910.1	159	-20.68	159	-20.68	1446989	24	1389	12
miR5799	LOC_Os10g12740.1	145	-24.2	145	-24.2	1447057	24	4428	138

miR5799	LOC_Os10g17140.1	155	-20.06	155	-20.06	1447310	24	963	716
miR5799	LOC_Os10g19160.1	157	-20.6	157	-20.6	1447493	24	3299	2055
miR5799	LOC_Os10g20820.1	157	-21.63	157	-21.63	1447629	24	366	286
miR5799	LOC_Os10g21170.1	157	-21.98	157	-21.98	1447660	24	393	14
miR5799	LOC_Os10g25040.1	177	-24.82	177	-24.82	1448049	24	3692	781
miR5799	LOC_Os10g25040.2	177	-24.82	177	-24.82	1448050	24	2238	781
miR5799	LOC_Os10g26960.1	164	-24.47	164	-24.47	1448246	24	1629	750
miR5799	LOC_Os10g32160.1	149	-20.32	149	-20.32	1448735	24	1976	1186
miR5799	LOC_Os10g34340.1	162	-22.24	162	-22.24	1448966	24	2103	1407
miR5799	LOC_Os10g34500.1	141	-20.45	141	-20.45	1448982	24	891	783
miR5799	LOC_Os10g35370.1	161	-23.62	161	-23.62	1449085	24	1765	161
miR5799	LOC_Os10g35370.2	161	-23.62	161	-23.62	1449086	24	2951	149
miR5799	LOC_Os10g39936.1	159	-22.4	159	-22.4	1449590	24	687	114
miR5799	LOC_Os11g01470.1	149	-20.71	149	-20.71	1450032	24	5073	304
miR5799	LOC_Os11g02670.1	152	-20.49	152	-20.49	1450180	24	3358	2110
miR5799	LOC_Os11g04560.1	147	-23.55	147	-23.55	1450400	24	1100	550
miR5799	LOC_Os11g05320.1	155	-20.61	155	-20.61	1450491	24	1781	986
miR5799	LOC_Os11g05850.2	160	-20.05	160	-20.05	1450550	24	5438	3858
miR5799	LOC_Os11g06580.1	151	-20.1	151	-20.1	1450641	24	1572	895
miR5799	LOC_Os11g06960.1	166	-22.52	166	-22.52	1450688	24	4715	4346
miR5799	LOC_Os11g08314.1	158	-21.28	158	-21.28	1450852	24	3577	1878
miR5799	LOC_Os11g09260.1	190	-30.49	190	-30.49	1450948	24	1887	834
miR5799	LOC_Os11g14610.1	158	-20.13	158	-20.13	1451464	24	5544	2096
miR5799	LOC_Os11g17970.1	150	-20.64	150	-20.64	1451765	24	2197	1491
miR5799	LOC_Os11g22000.1	155	-20.03	155	-20.03	1452065	24	1923	12
miR5799	LOC_Os11g25510.1	183	-21.93	183	-21.93	1452372	24	1526	1472
miR5799	LOC_Os11g32280.1	171	-21.85	171	-21.85	1453046	24	1209	143
miR5799	LOC_Os11g34020.1	147	-21.42	147	-21.42	1453185	24	1452	562
miR5799	LOC_Os11g34460.2	176	-23.14	176	-23.14	1453242	24	3837	2251
miR5799	LOC_Os11g37540.1	182	-27.45	182	-27.45	1453563	24	4071	3837
miR5799	LOC_Os11g42370.1	145	-20.21	145	-20.21	1454088	24	1584	677
miR5799	LOC_Os11g43390.1	143	-20.71	143	-20.71	1454192	24	2800	412

miR5799	LOC_Os11g45510.1	158	-20.26	158	-20.26	1454418	24	1971	464
miR5799	LOC_Os11g48000.1	169	-23.23	169	-23.23	1454656	24	1647	1062
miR5799	LOC_Os11g48070.1	151	-21.11	151	-21.11	1454664	24	1440	1075
miR5799	LOC_Os12g01680.1	180	-23.45	180	-23.45	1454754	24	834	520
miR5799	LOC_Os12g03050.1	163	-21.48	163	-21.48	1454929	24	1632	1512
miR5799	LOC_Os12g03680.1	163	-24.37	163	-24.37	1454999	24	2499	2156
miR5799	LOC_Os12g04360.1	147	-23.55	147	-23.55	1455088	24	799	549
miR5799	LOC_Os12g04990.4	169	-22.36	169	-22.36	1455164	24	2208	484
miR5799	LOC_Os12g09770.1	170	-24.5	170	-24.5	1455689	24	1359	1010
miR5799	LOC_Os12g09990.1	152	-21.38	152	-21.38	1455708	24	3864	2359
miR5799	LOC_Os12g12400.1	162	-24.47	162	-24.47	1455959	24	1581	672
miR5799	LOC_Os12g16350.13	184	-25.53	184	-25.53	1456363	24	4262	3101
miR5799	LOC_Os12g22110.1	141	-21	141	-21	1456864	24	1719	1589
miR5799	LOC_Os12g23374.1	162	-21.79	162	-21.79	1456988	24	2457	1774
miR5799	LOC_Os12g25980.1	166	-21.48	166	-21.48	1457236	24	4626	1439
miR5799	LOC_Os12g28750.1	149	-20.35	149	-20.35	1457510	24	1050	463
miR5799	LOC_Os12g31040.1	143	-21.36	143	-21.36	1457740	24	3768	264
miR5799	LOC_Os12g33560.1	159	-20.16	159	-20.16	1457995	24	1776	178
miR5799	LOC_Os12g40360.1	155	-26.2	155	-26.2	1458769	24	1941	1328
miR5799	LOC_Os12g41620.1	162	-20.87	162	-20.87	1458911	24	2992	444
miR5799	LOC_Os12g42230.1	156	-21.69	156	-21.69	1459000	24	1697	1369
miR5799	ChrUn.fgenesh.mRNA.90	156	-21.02	156	-21.02	1459341	24	1038	560
miR5337a	LOC_Os01g63820.1	176	-20.47	176	-20.47	1466182	21	3646	1634
miR5337a	LOC_Os01g63820.2	176	-20.47	176	-20.47	1466183	21	3470	1639
miR5337a	LOC_Os02g16140.1	161	-20.26	161	-20.26	1469242	21	1092	717
miR5337a	LOC_Os02g53860.1	191	-29.1	191	-29.1	1473474	21	2841	105
miR5337a	LOC_Os04g36058.1	161	-21.82	161	-21.82	1484581	21	1833	768
miR5337a	LOC_Os04g36058.2	161	-21.82	161	-21.82	1484582	21	1828	763
miR5337a	LOC_Os04g40910.1	175	-23.09	175	-23.09	1485047	21	2810	1872
miR5337a	LOC_Os04g40910.2	175	-23.09	175	-23.09	1485048	21	2711	1773
miR5337a	LOC_Os04g41000.1	166	-20.89	166	-20.89	1485062	21	3679	966
miR5337a	LOC_Os04g57310.1	160	-20.63	160	-20.63	1487013	21	1494	790

miR5337a	LOC_Os04g57310.2	160	-20.63	160	-20.63	1487014	21	1490	786
miR5337a	LOC_Os04g57320.1	160	-20.63	160	-20.63	1487015	21	1685	76
miR5337a	LOC_Os04g57320.2	160	-20.63	160	-20.63	1487016	21	1871	76
miR5337a	LOC_Os05g46740.1	174	-20.2	174	-20.2	1492176	21	3798	427
miR5337a	LOC_Os05g46740.2	174	-20.2	174	-20.2	1492177	21	3801	424
miR5337a	LOC_Os05g50980.1	179	-23.16	179	-23.16	1492657	21	1836	1765
miR5337a	LOC_Os06g03610.1	170	-20.55	170	-20.55	1493094	21	3048	373
miR5337a	LOC_Os06g20770.1	161	-20.91	161	-20.91	1494972	21	1273	566
miR5337a	LOC_Os06g28080.1	167	-21.02	167	-21.02	1495635	21	1728	1084
miR5337a	LOC_Os07g33480.2	166	-20.84	166	-20.84	1501564	21	2307	1173
miR5337a	LOC_Os09g01780.1	161	-20.26	161	-20.26	1508411	21	1947	666
miR5337a	LOC_Os10g05750.1	169	-20.27	169	-20.27	1512732	21	1289	143
miR5337a	LOC_Os10g39620.1	173	-23.05	173	-23.05	1515880	21	2453	867
miR5337a	LOC_Os10g39620.2	173	-23.05	173	-23.05	1515881	21	2536	950
miR5337a	LOC_Os10g39620.3	173	-23.05	173	-23.05	1515882	21	2640	1054
miR5337a	LOC_Os10g39620.4	173	-23.05	173	-23.05	1515883	21	2608	950
miR5337a	LOC_Os10g39620.5	173	-23.05	173	-23.05	1515884	21	2682	1024
miR5337a	LOC_Os10g39620.6	173	-23.05	173	-23.05	1515885	21	2712	1054
miR5337a	LOC_Os10g39620.7	173	-23.05	173	-23.05	1515886	21	2311	653
miR5337a	LOC_Os11g07060.1	173	-21.2	173	-21.2	1517041	21	3785	564
miR5337a	LOC_Os11g07060.2	173	-21.2	173	-21.2	1517042	21	3699	478
miR5337a	LOC_Os11g07060.3	173	-21.2	173	-21.2	1517043	21	3815	594
miR5337a	LOC_Os11g07060.4	173	-21.2	173	-21.2	1517044	21	3789	568
miR5337a	LOC_Os11g07060.5	173	-21.2	173	-21.2	1517045	21	3703	564
miR5337a	LOC_Os11g36830.1	169	-23.29	169	-23.29	1519832	21	2121	598
miR5337a	LOC_Os11g46250.1	165	-22.05	165	-22.05	1520837	21	4950	864
miR529b	LOC_Os01g01710.1	165	-20.92	165	-20.92	1525873	21	1902	1877
miR529b	LOC_Os01g01710.2	165	-20.92	165	-20.92	1525874	21	1801	1776
miR529b	LOC_Os01g02110.1	156	-20.4	156	-20.4	1525924	21	1475	597
miR529b	LOC_Os01g02250.1	151	-23.97	151	-23.97	1525940	21	4035	3541
miR529b	LOC_Os01g02280.1	151	-23.97	151	-23.97	1525943	21	5367	4873
miR529b	LOC_Os01g02560.1	161	-24.07	161	-24.07	1525975	21	2059	36

miR529b	LOC_Os01g02629.1	154	-20.29	154	-20.29	1525981	21	1743	14
miR529b	LOC_Os01g02710.1	158	-23.54	158	-23.54	1525988	21	603	17
miR529b	LOC_Os01g02920.1	339	-48.32	173	-25.65	1526011	21	2288	1724 1826
miR529b	LOC_Os01g03420.1	171	-23.09	171	-23.09	1526070	21	1254	590
miR529b	LOC_Os01g03520.1	168	-24.98	168	-24.98	1526079	21	3960	6
miR529b	LOC_Os01g03914.1	161	-20.73	161	-20.73	1526117	21	2241	1488
miR529b	LOC_Os01g03914.2	161	-20.73	161	-20.73	1526118	21	2233	1488
miR529b	LOC_Os01g03914.3	161	-20.73	161	-20.73	1526119	21	2314	1561
miR529b	LOC_Os01g03950.1	171	-27.51	171	-27.51	1526122	21	3485	245
miR529b	LOC_Os01g04510.1	167	-22.32	167	-22.32	1526183	21	567	479
miR529b	LOC_Os01g05130.1	165	-22.74	165	-22.74	1526262	21	1027	1
miR529b	LOC_Os01g06300.1	156	-21.71	156	-21.71	1526388	21	4458	2754
miR529b	LOC_Os01g06950.1	163	-20.91	163	-20.91	1526459	21	465	54
miR529b	LOC_Os01g07180.1	164	-20.48	164	-20.48	1526488	21	975	94
miR529b	LOC_Os01g07370.1	156	-23.48	156	-23.48	1526515	21	2267	935
miR529b	LOC_Os01g07370.2	156	-23.48	156	-23.48	1526516	21	2288	935
miR529b	LOC_Os01g08500.1	151	-20.15	151	-20.15	1526644	21	1287	948
miR529b	LOC_Os01g08560.1	160	-21.48	160	-21.48	1526650	21	3188	2488
miR529b	LOC_Os01g08560.2	160	-21.48	160	-21.48	1526651	21	3184	2484
miR529b	LOC_Os01g09190.1	156	-23.21	156	-23.21	1526727	21	946	644
miR529b	LOC_Os01g09640.1	159	-22.5	159	-22.5	1526782	21	1769	894
miR529b	LOC_Os01g09670.1	165	-20.86	165	-20.86	1526786	21	1520	56
miR529b	LOC_Os01g09760.1	152	-20.46	152	-20.46	1526797	21	3314	3094
miR529b	LOC_Os01g10150.1	150	-23.22	150	-23.22	1526839	21	2466	1122
miR529b	LOC_Os01g10150.2	150	-23.22	150	-23.22	1526840	21	2462	1118
miR529b	LOC_Os01g10195.1	168	-21.32	168	-21.32	1526844	21	1238	304
miR529b	LOC_Os01g10450.1	166	-26	166	-26	1526875	21	2591	2549
miR529b	LOC_Os01g10450.2	166	-26	166	-26	1526876	21	2579	2531
miR529b	LOC_Os01g10504.1	158	-21.89	158	-21.89	1526881	21	1187	1053
miR529b	LOC_Os01g10504.2	158	-21.89	158	-21.89	1526882	21	1434	1300
miR529b	LOC_Os01g10504.3	158	-21.89	158	-21.89	1526883	21	1188	1054
miR529b	LOC_Os01g11820.1	157	-21.55	157	-21.55	1527012	21	1023	1

miR529b	LOC_Os01g12150.1	156	-20.23	156	-20.23	1527047	21	432	1
miR529b	LOC_Os01g12740.1	166	-21.66	166	-21.66	1527123	21	2005	28
miR529b	LOC_Os01g14010.1	162	-23.48	162	-23.48	1527275	21	2115	1039
miR529b	LOC_Os01g15200.1	161	-20.68	161	-20.68	1527402	21	381	205
miR529b	LOC_Os01g15290.1	159	-21.97	159	-21.97	1527411	21	754	273
miR529b	LOC_Os01g15290.2	159	-21.97	159	-21.97	1527412	21	770	273
miR529b	LOC_Os01g15330.1	164	-20.48	164	-20.48	1527417	21	369	30
miR529b	LOC_Os01g15790.4	162	-20.06	162	-20.06	1527473	21	1658	384
miR529b	LOC_Os01g15900.1	164	-22.31	164	-22.31	1527482	21	2228	1593
miR529b	LOC_Os01g16160.1	149	-21.86	149	-21.86	1527515	21	4917	4229
miR529b	LOC_Os01g16190.1	175	-26.21	175	-26.21	1527525	21	1339	1259
miR529b	LOC_Os01g16414.1	156	-22.08	156	-22.08	1527553	21	2283	1964
miR529b	LOC_Os01g16414.2	156	-22.08	156	-22.08	1527554	21	2044	1685
miR529b	LOC_Os01g16590.1	161	-20.91	161	-20.91	1527573	21	408	261
miR529b	LOC_Os01g16650.1	168	-24.98	168	-24.98	1527580	21	888	14
miR529b	LOC_Os01g16740.1	165	-22.78	165	-22.78	1527587	21	3639	123
miR529b	LOC_Os01g17070.1	177	-28.92	177	-28.92	1527629	21	1873	1762
miR529b	LOC_Os01g17070.2	177	-28.92	177	-28.92	1527630	21	2164	2067
miR529b	LOC_Os01g17120.1	157	-21.69	157	-21.69	1527635	21	3366	387
miR529b	LOC_Os01g17320.1	168	-23.18	168	-23.18	1527651	21	2265	101
miR529b	LOC_Os01g18000.1	153	-20.93	153	-20.93	1527673	21	4224	4139
miR529b	LOC_Os01g18230.1	161	-20.17	161	-20.17	1527696	21	669	23
miR529b	LOC_Os01g18280.1	157	-22.95	157	-22.95	1527701	21	1811	5
miR529b	LOC_Os01g18710.1	161	-23.47	161	-23.47	1527742	21	570	336
miR529b	LOC_Os01g19410.1	168	-24.98	168	-24.98	1527823	21	1650	1262
miR529b	LOC_Os01g19410.2	168	-24.98	168	-24.98	1527824	21	1488	1104
miR529b	LOC_Os01g19410.3	168	-24.98	168	-24.98	1527825	21	1468	1080
miR529b	LOC_Os01g20770.1	160	-20.69	160	-20.69	1527917	21	678	129
miR529b	LOC_Os01g21100.1	156	-21.45	156	-21.45	1527957	21	615	449
miR529b	LOC_Os01g21190.1	156	-21.71	156	-21.71	1527970	21	3489	2502
miR529b	LOC_Os01g22954.1	167	-20.72	167	-20.72	1528139	21	1984	296
miR529b	LOC_Os01g23100.1	156	-20.3	156	-20.3	1528153	21	981	443

miR529b	LOC_Os01g23560.1	169	-23.03	169	-23.03	1528194	21	1233	1
miR529b	LOC_Os01g23730.1	165	-21.46	165	-21.46	1528217	21	804	57
miR529b	LOC_Os01g24130.1	169	-23.84	169	-23.84	1528259	21	1377	950
miR529b	LOC_Os01g24370.1	154	-20.29	154	-20.29	1528281	21	441	14
miR529b	LOC_Os01g24470.1	173	-27.1	173	-27.1	1528291	21	3416	476
miR529b	LOC_Os01g24470.2	173	-27.1	173	-27.1	1528292	21	3378	375
miR529b	LOC_Os01g25210.1	156	-22.58	156	-22.58	1528377	21	585	535
miR529b	LOC_Os01g26130.3	152	-20.69	152	-20.69	1528467	21	1511	379
miR529b	LOC_Os01g26130.5	152	-20.69	152	-20.69	1528469	21	1663	531
miR529b	LOC_Os01g26390.1	160	-23.91	160	-23.91	1528490	21	2602	24
miR529b	LOC_Os01g27040.1	167	-22.66	167	-22.66	1528514	21	3750	1035
miR529b	LOC_Os01g27280.1	166	-23.94	166	-23.94	1528538	21	2799	2650
miR529b	LOC_Os01g29020.1	149	-20.23	149	-20.23	1528717	21	1164	157
miR529b	LOC_Os01g29270.1	161	-22	161	-22	1528744	21	4491	3138
miR529b	LOC_Os01g29409.1	173	-22.24	173	-22.24	1528757	21	2567	2436
miR529b	LOC_Os01g29530.1	161	-20.3	161	-20.3	1528764	21	3387	2833
miR529b	LOC_Os01g29660.1	157	-21.69	157	-21.69	1528776	21	1230	519
miR529b	LOC_Os01g31450.1	170	-23.4	170	-23.4	1528860	21	2831	18
miR529b	LOC_Os01g32220.1	157	-21.69	157	-21.69	1528944	21	4662	531
miR529b	LOC_Os01g32660.1	157	-20.52	157	-20.52	1528984	21	1761	19
miR529b	LOC_Os01g32660.2	157	-20.52	157	-20.52	1528985	21	1758	19
miR529b	LOC_Os01g32660.3	157	-20.52	157	-20.52	1528986	21	1364	19
miR529b	LOC_Os01g32660.4	157	-20.52	157	-20.52	1528987	21	1825	19
miR529b	LOC_Os01g32660.5	157	-20.52	157	-20.52	1528988	21	2272	19
miR529b	LOC_Os01g32830.1	162	-23.43	162	-23.43	1529005	21	2718	2628
miR529b	LOC_Os01g32830.2	162	-23.43	162	-23.43	1529006	21	947	857
miR529b	LOC_Os01g32864.1	149	-20.68	149	-20.68	1529010	21	833	617
miR529b	LOC_Os01g33040.1	154	-20.61	154	-20.61	1529028	21	3824	2236
miR529b	LOC_Os01g33960.1	162	-20.32	162	-20.32	1529115	21	2142	1946
miR529b	LOC_Os01g34420.1	168	-23.18	168	-23.18	1529155	21	803	220
miR529b	LOC_Os01g34830.1	156	-20.31	156	-20.31	1529209	21	1125	10
miR529b	LOC_Os01g35520.1	154	-20.29	154	-20.29	1529278	21	375	14

miR529b	LOC_Os01g35760.1	156	-20.23	156	-20.23	1529307	21	477	369
miR529b	LOC_Os01g36710.1	151	-20.15	151	-20.15	1529402	21	1665	948
miR529b	LOC_Os01g38030.1	159	-20.37	159	-20.37	1529560	21	876	1
miR529b	LOC_Os01g38530.1	158	-22.33	158	-22.33	1529610	21	2832	1238
miR529b	LOC_Os01g38610.1	175	-24.5	175	-24.5	1529620	21	2420	2240
miR529b	LOC_Os01g39050.1	165	-20.26	165	-20.26	1529667	21	5391	570
miR529b	LOC_Os01g39110.1	171	-27.01	171	-27.01	1529673	21	2783	305
miR529b	LOC_Os01g39990.1	163	-20.17	163	-20.17	1529769	21	1658	1124
miR529b	LOC_Os01g40200.1	159	-20.26	159	-20.26	1529789	21	2897	687
miR529b	LOC_Os01g40200.2	159	-20.26	159	-20.26	1529790	21	2988	778
miR529b	LOC_Os01g40310.1	158	-20.41	158	-20.41	1529801	21	885	769
miR529b	LOC_Os01g41950.1	163	-21.76	163	-21.76	1529980	21	3201	304
miR529b	LOC_Os01g41960.1	156	-21.54	156	-21.54	1529981	21	6519	583
miR529b	LOC_Os01g43330.1	166	-23.2	166	-23.2	1530144	21	3403	2515
miR529b	LOC_Os01g43520.1	160	-22.48	160	-22.48	1530169	21	3065	2083
miR529b	LOC_Os01g43520.2	160	-22.48	160	-22.48	1530170	21	3214	2083
miR529b	LOC_Os01g44940.1	169	-25.54	169	-25.54	1530275	21	672	408
miR529b	LOC_Os01g45220.1	161	-25	161	-25	1530305	21	2436	2411
miR529b	LOC_Os01g45439.2	165	-22.56	165	-22.56	1530327	21	1446	1194
miR529b	LOC_Os01g46100.1	169	-23.84	169	-23.84	1530396	21	3162	1973
miR529b	LOC_Os01g46810.1	323	-41	162	-20.95	1530478	21	4832	1842 1721
miR529b	LOC_Os01g47050.1	162	-22.17	162	-22.17	1530505	21	1982	718
miR529b	LOC_Os01g48440.1	170	-24.1	170	-24.1	1530680	21	2322	859
miR529b	LOC_Os01g48440.2	170	-24.1	170	-24.1	1530681	21	2469	1006
miR529b	LOC_Os01g48440.3	170	-24.1	170	-24.1	1530682	21	2750	1287
miR529b	LOC_Os01g48530.1	333	-49.25	168	-26.07	1530691	21	971	25 651
miR529b	LOC_Os01g50100.1	162	-20.82	162	-20.82	1530872	21	4079	2630
miR529b	LOC_Os01g50500.1	157	-21.69	157	-21.69	1530909	21	4575	531
miR529b	LOC_Os01g51280.1	335	-44.99	168	-23.18	1531003	21	2002	24 57
miR529b	LOC_Os01g51280.2	335	-44.99	168	-23.18	1531004	21	2086	24 57
miR529b	LOC_Os01g52230.1	165	-24.89	165	-24.89	1531119	21	1217	1150
miR529b	LOC_Os01g52640.3	165	-24.25	165	-24.25	1531166	21	4050	1461

miR529b	LOC_Os01g52710.1	160	-20.59	160	-20.59	1531173	21	2494	1450
miR529b	LOC_Os01g53960.1	157	-21.69	157	-21.69	1531314	21	4704	531
miR529b	LOC_Os01g54100.1	166	-21.91	166	-21.91	1531332	21	2389	1099
miR529b	LOC_Os01g54100.2	166	-21.91	166	-21.91	1531333	21	2772	1100
miR529b	LOC_Os01g54480.1	154	-20.07	154	-20.07	1531372	21	2284	1107
miR529b	LOC_Os01g54480.2	154	-20.07	154	-20.07	1531373	21	2331	1234
miR529b	LOC_Os01g54480.3	154	-20.07	154	-20.07	1531374	21	2361	1264
miR529b	LOC_Os01g55470.1	169	-23.84	169	-23.84	1531497	21	2799	1808
miR529b	LOC_Os01g55490.1	163	-20.14	163	-20.14	1531499	21	1716	127
miR529b	LOC_Os01g55490.2	163	-20.14	163	-20.14	1531500	21	1713	127
miR529b	LOC_Os01g55490.3	163	-20.14	163	-20.14	1531501	21	1667	127
miR529b	LOC_Os01g55490.4	163	-20.14	163	-20.14	1531502	21	1648	127
miR529b	LOC_Os01g55530.1	160	-20.64	160	-20.64	1531509	21	3675	2277
miR529b	LOC_Os01g55549.1	155	-20.81	155	-20.81	1531512	21	2451	374
miR529b	LOC_Os01g56300.1	171	-28.36	171	-28.36	1531609	21	3646	395
miR529b	LOC_Os01g57050.1	162	-20.68	162	-20.68	1531707	21	1011	550
miR529b	LOC_Os01g57050.2	162	-20.68	162	-20.68	1531708	21	898	550
miR529b	LOC_Os01g57120.1	156	-21.71	156	-21.71	1531715	21	2661	2235
miR529b	LOC_Os01g57550.1	168	-23.18	168	-23.18	1531765	21	1804	147
miR529b	LOC_Os01g57550.2	168	-23.18	168	-23.18	1531766	21	1940	185
miR529b	LOC_Os01g57735.1	160	-20.17	160	-20.17	1531791	21	1601	213
miR529b	LOC_Os01g57854.1	156	-21.65	156	-21.65	1531802	21	1869	71
miR529b	LOC_Os01g58950.1	161	-21.52	161	-21.52	1531932	21	1494	15
miR529b	LOC_Os01g59265.1	169	-24.35	169	-24.35	1531967	21	625	470
miR529b	LOC_Os01g60040.1	153	-20.04	153	-20.04	1532058	21	6605	2163
miR529b	LOC_Os01g60040.2	153	-20.04	153	-20.04	1532059	21	6623	2163
miR529b	LOC_Os01g61590.1	169	-23.87	169	-23.87	1532230	21	2139	57
miR529b	LOC_Os01g61680.1	165	-21.43	165	-21.43	1532243	21	1203	673
miR529b	LOC_Os01g61860.1	165	-24.97	165	-24.97	1532274	21	3308	419
miR529b	LOC_Os01g61860.2	165	-24.97	165	-24.97	1532275	21	3177	419
miR529b	LOC_Os01g61880.1	159	-21.65	159	-21.65	1532277	21	2900	1773
miR529b	LOC_Os01g61880.2	159	-21.65	159	-21.65	1532278	21	2525	1398

miR529b	LOC_Os01g61890.2	159	-21.65	159	-21.65	1532280	21	4288	1819
miR529b	LOC_Os01g61890.3	159	-21.65	159	-21.65	1532281	21	5479	1819
miR529b	LOC_Os01g61910.1	164	-23.85	164	-23.85	1532284	21	3151	2329
miR529b	LOC_Os01g62350.1	159	-20.61	159	-20.61	1532340	21	620	265
miR529b	LOC_Os01g64490.1	166	-21.72	166	-21.72	1532610	21	3544	1897
miR529b	LOC_Os01g64650.1	160	-20.11	160	-20.11	1532630	21	1272	471
miR529b	LOC_Os01g64650.2	160	-20.11	160	-20.11	1532631	21	1400	471
miR529b	LOC_Os01g64810.1	162	-21.73	162	-21.73	1532661	21	2294	52
miR529b	LOC_Os01g65094.1	165	-21.62	165	-21.62	1532696	21	1056	893
miR529b	LOC_Os01g65550.1	161	-20.09	161	-20.09	1532748	21	2219	60
miR529b	LOC_Os01g65550.2	161	-20.09	161	-20.09	1532749	21	2239	60
miR529b	LOC_Os01g65640.1	156	-20.67	156	-20.67	1532760	21	1485	959
miR529b	LOC_Os01g65780.2	168	-23.7	168	-23.7	1532778	21	2665	66
miR529b	LOC_Os01g66490.1	161	-20.91	161	-20.91	1532859	21	2044	233
miR529b	LOC_Os01g66580.1	150	-20.58	150	-20.58	1532867	21	861	681
miR529b	LOC_Os01g67370.1	167	-20.02	167	-20.02	1532966	21	2199	2038
miR529b	LOC_Os01g67370.2	167	-20.02	167	-20.02	1532967	21	2102	1941
miR529b	LOC_Os01g67370.3	167	-20.02	167	-20.02	1532968	21	2318	2157
miR529b	LOC_Os01g67370.4	167	-20.02	167	-20.02	1532969	21	1075	914
miR529b	LOC_Os01g67370.5	167	-20.02	167	-20.02	1532970	21	1339	1178
miR529b	LOC_Os01g67370.6	167	-20.02	167	-20.02	1532971	21	1576	1415
miR529b	LOC_Os01g67810.1	162	-20.02	162	-20.02	1533022	21	2022	42
miR529b	LOC_Os01g67990.1	156	-22.71	156	-22.71	1533041	21	2634	1026
miR529b	LOC_Os01g68010.1	157	-22.07	157	-22.07	1533043	21	1311	1027
miR529b	LOC_Os01g68010.2	157	-22.07	157	-22.07	1533044	21	1318	1034
miR529b	LOC_Os01g68010.3	157	-22.07	157	-22.07	1533045	21	1317	1033
miR529b	LOC_Os01g68010.4	157	-22.07	157	-22.07	1533046	21	855	571
miR529b	LOC_Os01g68010.5	157	-22.07	157	-22.07	1533047	21	1329	571
miR529b	LOC_Os01g68020.1	162	-20.16	162	-20.16	1533048	21	1482	37
miR529b	LOC_Os01g68120.1	166	-22.14	166	-22.14	1533058	21	5199	5100
miR529b	LOC_Os01g68324.3	161	-25.27	161	-25.27	1533083	21	2732	2254
miR529b	LOC_Os01g68340.1	166	-21.94	166	-21.94	1533085	21	1623	123

miR529b	LOC_Os01g68524.1	171	-21.58	171	-21.58	1533108	21	3327	1087	
miR529b	LOC_Os01g68700.1	165	-22.49	165	-22.49	1533127	21	2432	961	
miR529b	LOC_Os01g68700.2	165	-22.49	165	-22.49	1533128	21	2428	957	
miR529b	LOC_Os01g69100.1	168	-21.29	168	-21.29	1533184	21	1272	86	
miR529b	LOC_Os01g69130.1	170	-23.83	170	-23.83	1533190	21	3206	1336	
miR529b	LOC_Os01g69130.2	170	-23.83	170	-23.83	1533191	21	3098	1228	
miR529b	LOC_Os01g69830.1	343	-56.66	186	-35.22	1533214	21	1983	1148	1
miR529b	LOC_Os01g69950.1	165	-21.89	165	-21.89	1533230	21	1968	32	
miR529b	LOC_Os01g70360.1	165	-27.88	165	-27.88	1533283	21	1925	1818	
miR529b	LOC_Os01g70710.1	157	-21.22	157	-21.22	1533326	21	1669	1552	
miR529b	LOC_Os01g70710.2	157	-21.22	157	-21.22	1533327	21	1917	1800	
miR529b	LOC_Os01g70890.1	168	-22.71	168	-22.71	1533349	21	1425	1334	
miR529b	LOC_Os01g70960.1	154	-21.84	154	-21.84	1533361	21	1497	270	
miR529b	LOC_Os01g70960.2	154	-21.84	154	-21.84	1533362	21	2661	277	
miR529b	LOC_Os01g70980.1	168	-22.71	168	-22.71	1533364	21	1041	1001	
miR529b	LOC_Os01g71000.1	157	-20.36	157	-20.36	1533366	21	2574	25	
miR529b	LOC_Os01g71050.1	161	-21.58	161	-21.58	1533371	21	2111	801	
miR529b	LOC_Os01g71310.1	162	-23.75	162	-23.75	1533401	21	2388	869	
miR529b	LOC_Os01g71390.1	168	-23.44	168	-23.44	1533409	21	327	165	
miR529b	LOC_Os01g71624.1	156	-21.58	156	-21.58	1533434	21	828	346	
miR529b	LOC_Os01g71624.2	156	-21.58	156	-21.58	1533435	21	1046	350	
miR529b	LOC_Os01g71624.3	156	-21.58	156	-21.58	1533436	21	756	350	
miR529b	LOC_Os01g71624.4	156	-21.58	156	-21.58	1533437	21	782	350	
miR529b	LOC_Os01g71624.5	156	-21.58	156	-21.58	1533438	21	1829	350	
miR529b	LOC_Os01g71970.1	160	-23.29	160	-23.29	1533474	21	1544	6	
miR529b	LOC_Os01g72210.1	162	-25.65	162	-25.65	1533500	21	2907	2734	
miR529b	LOC_Os01g72210.2	162	-25.65	162	-25.65	1533501	21	3068	2741	
miR529b	LOC_Os01g72220.1	162	-25.65	162	-25.65	1533502	21	2596	281	
miR529b	LOC_Os01g72370.1	158	-20.41	158	-20.41	1533518	21	4096	2055	
miR529b	LOC_Os01g72860.1	156	-20.7	156	-20.7	1533577	21	2808	1937	
miR529b	LOC_Os01g73710.1	155	-20.75	155	-20.75	1533677	21	951	802	
miR529b	LOC_Os01g73790.1	162	-22.58	162	-22.58	1533685	21	1920	1896	

miR529b	LOC_Os01g74510.1	157	-22.38	157	-22.38	1533773	21	3404	2894
miR529b	LOC_Os02g01220.1	157	-23.38	157	-23.38	1533830	21	1369	21
miR529b	LOC_Os02g01580.1	157	-22.3	157	-22.3	1533873	21	2502	2477
miR529b	LOC_Os02g01590.1	160	-22.56	160	-22.56	1533874	21	2763	2080
miR529b	LOC_Os02g01590.2	160	-22.56	160	-22.56	1533875	21	2252	1569
miR529b	LOC_Os02g01870.1	157	-20.08	157	-20.08	1533908	21	1537	639
miR529b	LOC_Os02g01900.1	157	-20.08	157	-20.08	1533911	21	1308	486
miR529b	LOC_Os02g01960.1	152	-21.03	152	-21.03	1533917	21	2190	513
miR529b	LOC_Os02g02290.1	162	-21.81	162	-21.81	1533954	21	7021	2146
miR529b	LOC_Os02g02410.1	153	-22.03	153	-22.03	1533969	21	2476	2382
miR529b	LOC_Os02g02500.1	165	-22.78	165	-22.78	1533980	21	1873	35
miR529b	LOC_Os02g02610.1	156	-21.71	156	-21.71	1533994	21	3378	1674
miR529b	LOC_Os02g03470.1	156	-20.37	156	-20.37	1534113	21	1425	42
miR529b	LOC_Os02g03670.1	157	-22.43	157	-22.43	1534139	21	2051	579
miR529b	LOC_Os02g03670.3	157	-22.43	157	-22.43	1534140	21	2831	579
miR529b	LOC_Os02g03670.5	157	-22.43	157	-22.43	1534141	21	2358	579
miR529b	LOC_Os02g03670.6	157	-22.43	157	-22.43	1534142	21	2259	579
miR529b	LOC_Os02g03670.7	157	-22.43	157	-22.43	1534143	21	2190	579
miR529b	LOC_Os02g04020.1	165	-24.26	165	-24.26	1534188	21	2478	193
miR529b	LOC_Os02g04480.1	161	-20.78	161	-20.78	1534255	21	3120	1127
miR529b	LOC_Os02g04640.1	156	-22.09	156	-22.09	1534273	21	2002	1889
miR529b	LOC_Os02g04680.1	327	-49.96	171	-29.1	1534278	21	2517	1960 984
miR529b	LOC_Os02g04680.2	327	-49.96	171	-29.1	1534279	21	2514	1957 984
miR529b	LOC_Os02g04910.1	156	-21.71	156	-21.71	1534311	21	5157	3033
miR529b	LOC_Os02g04970.1	163	-20.67	163	-20.67	1534319	21	4893	1666
miR529b	LOC_Os02g05570.1	169	-23.84	169	-23.84	1534397	21	3066	1877
miR529b	LOC_Os02g05880.1	156	-20.55	156	-20.55	1534437	21	6900	2699
miR529b	LOC_Os02g06000.1	156	-21.71	156	-21.71	1534449	21	2493	921
miR529b	LOC_Os02g06830.1	168	-22.71	168	-22.71	1534548	21	3642	3602
miR529b	LOC_Os02g06830.2	168	-22.71	168	-22.71	1534549	21	3528	3488
miR529b	LOC_Os02g07170.1	154	-21.04	154	-21.04	1534595	21	1022	865
miR529b	LOC_Os02g07780.1	177	-33.41	177	-33.41	1534679	21	1668	983

miR529b	LOC_Os02g07780.2	177	-33.41	177	-33.41	1534680	21	2902	2217
miR529b	LOC_Os02g07820.1	168	-24.98	168	-24.98	1534684	21	1556	207
miR529b	LOC_Os02g08130.1	160	-20.18	160	-20.18	1534725	21	2798	2503
miR529b	LOC_Os02g08130.2	160	-20.18	160	-20.18	1534726	21	4810	2877
miR529b	LOC_Os02g08130.3	160	-20.18	160	-20.18	1534727	21	4638	2860
miR529b	LOC_Os02g08140.1	160	-20.18	160	-20.18	1534728	21	2131	853
miR529b	LOC_Os02g08440.1	158	-20.15	158	-20.15	1534761	21	1876	155
miR529b	LOC_Os02g08440.2	158	-20.15	158	-20.15	1534762	21	1833	3
miR529b	LOC_Os02g08440.3	158	-20.15	158	-20.15	1534763	21	1987	155
miR529b	LOC_Os02g09060.1	162	-20.77	162	-20.77	1534779	21	1778	1694
miR529b	LOC_Os02g09060.2	162	-20.77	162	-20.77	1534780	21	2162	2078
miR529b	LOC_Os02g10200.1	173	-23.5	173	-23.5	1534914	21	1122	1092
miR529b	LOC_Os02g10200.2	173	-23.5	173	-23.5	1534915	21	1128	1098
miR529b	LOC_Os02g10640.1	168	-22.91	168	-22.91	1534970	21	1732	1638
miR529b	LOC_Os02g10750.1	164	-21.12	164	-21.12	1534983	21	726	63
miR529b	LOC_Os02g10770.2	153	-20.12	153	-20.12	1534985	21	2132	1934
miR529b	LOC_Os02g11990.1	159	-21.3	159	-21.3	1535084	21	897	693
miR529b	LOC_Os02g12660.1	165	-23.03	165	-23.03	1535164	21	2993	1454
miR529b	LOC_Os02g12670.1	174	-25.75	174	-25.75	1535165	21	2975	2870
miR529b	LOC_Os02g13020.1	167	-25.85	167	-25.85	1535219	21	1897	304
miR529b	LOC_Os02g13100.1	155	-21.01	155	-21.01	1535226	21	1919	33
miR529b	LOC_Os02g13520.1	153	-20.09	153	-20.09	1535274	21	1533	660
miR529b	LOC_Os02g13560.2	166	-22.59	166	-22.59	1535280	21	3159	273
miR529b	LOC_Os02g13560.3	166	-22.59	166	-22.59	1535281	21	3025	139
miR529b	LOC_Os02g14130.1	160	-20.17	160	-20.17	1535346	21	2112	2030
miR529b	LOC_Os02g15230.1	161	-21.97	161	-21.97	1535469	21	1863	345
miR529b	LOC_Os02g15230.2	161	-21.97	161	-21.97	1535470	21	3297	345
miR529b	LOC_Os02g15230.3	161	-21.97	161	-21.97	1535471	21	3301	345
miR529b	LOC_Os02g15310.1	161	-20.12	161	-20.12	1535479	21	1269	761
miR529b	LOC_Os02g15310.2	161	-20.12	161	-20.12	1535480	21	2241	761
miR529b	LOC_Os02g15310.3	161	-20.12	161	-20.12	1535481	21	1822	761
miR529b	LOC_Os02g15310.4	161	-20.12	161	-20.12	1535482	21	2071	761

miR529b	LOC_Os02g15670.1	161	-25.21	161	-25.21	1535520	21	387	53
miR529b	LOC_Os02g15820.1	163	-24.09	163	-24.09	1535536	21	3137	2496
miR529b	LOC_Os02g15820.2	163	-24.09	163	-24.09	1535537	21	3161	2614
miR529b	LOC_Os02g17040.1	174	-26.46	174	-26.46	1535675	21	882	802
miR529b	LOC_Os02g17440.1	161	-20.76	161	-20.76	1535717	21	1422	408
miR529b	LOC_Os02g17810.1	166	-21.94	166	-21.94	1535756	21	1992	123
miR529b	LOC_Os02g18290.1	161	-22	161	-22	1535816	21	4581	3405
miR529b	LOC_Os02g18730.1	162	-24.91	162	-24.91	1535862	21	3120	3029
miR529b	LOC_Os02g18850.1	169	-24.94	169	-24.94	1535877	21	2251	187
miR529b	LOC_Os02g19620.1	149	-21.86	149	-21.86	1535956	21	4827	4034
miR529b	LOC_Os02g21430.1	167	-22.49	167	-22.49	1536147	21	3347	61
miR529b	LOC_Os02g22140.1	157	-23.59	157	-23.59	1536232	21	1516	376
miR529b	LOC_Os02g22190.1	149	-20.5	149	-20.5	1536236	21	2052	378
miR529b	LOC_Os02g22520.1	161	-20.28	161	-20.28	1536274	21	4488	528
miR529b	LOC_Os02g22650.1	165	-20.68	165	-20.68	1536286	21	2754	35
miR529b	LOC_Os02g22650.2	165	-20.68	165	-20.68	1536287	21	2751	35
miR529b	LOC_Os02g24090.1	170	-23.3	170	-23.3	1536351	21	4725	4238
miR529b	LOC_Os02g24490.1	153	-20.34	153	-20.34	1536390	21	1281	1251
miR529b	LOC_Os02g24586.1	153	-21.5	153	-21.5	1536400	21	405	141
miR529b	LOC_Os02g25170.1	153	-20.34	153	-20.34	1536465	21	6420	1581
miR529b	LOC_Os02g25230.1	177	-30.99	177	-30.99	1536471	21	3840	594
miR529b	LOC_Os02g25760.1	164	-23.28	164	-23.28	1536526	21	723	141
miR529b	LOC_Os02g26700.1	154	-20.3	154	-20.3	1536632	21	1454	731
miR529b	LOC_Os02g26700.2	154	-20.3	154	-20.3	1536633	21	1558	956
miR529b	LOC_Os02g26700.3	154	-20.3	154	-20.3	1536634	21	1353	731
miR529b	LOC_Os02g26700.4	154	-20.3	154	-20.3	1536635	21	1467	731
miR529b	LOC_Os02g26700.6	154	-20.3	154	-20.3	1536636	21	1419	817
miR529b	LOC_Os02g26700.7	154	-20.3	154	-20.3	1536637	21	1329	727
miR529b	LOC_Os02g27090.1	151	-22.25	151	-22.25	1536685	21	960	753
miR529b	LOC_Os02g28010.1	157	-21.69	157	-21.69	1536788	21	4554	324
miR529b	LOC_Os02g29464.1	169	-22.81	169	-22.81	1536931	21	4549	221
miR529b	LOC_Os02g29550.1	160	-25.46	160	-25.46	1536940	21	2702	392

miR529b	LOC_Os02g30530.1	167	-26.05	167	-26.05	1537050	21	3497	786
miR529b	LOC_Os02g30580.1	157	-21.69	157	-21.69	1537055	21	4542	531
miR529b	LOC_Os02g30590.1	162	-20.95	162	-20.95	1537056	21	504	162
miR529b	LOC_Os02g30955.1	165	-24.08	165	-24.08	1537100	21	762	96
miR529b	LOC_Os02g31150.1	157	-21.15	157	-21.15	1537123	21	2673	1142
miR529b	LOC_Os02g31890.1	151	-20.95	151	-20.95	1537151	21	1479	1349
miR529b	LOC_Os02g33220.1	164	-20.48	164	-20.48	1537306	21	450	115
miR529b	LOC_Os02g33250.1	486	-68.25	162	-22.75	1537309	21	411	11 34 65
miR529b	LOC_Os02g33335.1	168	-26.81	168	-26.81	1537321	21	1078	192
miR529b	LOC_Os02g33450.1	159	-21.56	159	-21.56	1537333	21	1707	392
miR529b	LOC_Os02g33450.2	159	-21.56	159	-21.56	1537334	21	1707	392
miR529b	LOC_Os02g33670.1	165	-25.12	165	-25.12	1537357	21	1442	328
miR529b	LOC_Os02g34650.1	164	-21.32	164	-21.32	1537453	21	1820	1660
miR529b	LOC_Os02g34860.1	165	-23.03	165	-23.03	1537474	21	1757	1
miR529b	LOC_Os02g35140.1	165	-21.81	165	-21.81	1537509	21	3399	34
miR529b	LOC_Os02g36414.1	156	-20.27	156	-20.27	1537658	21	2308	585
miR529b	LOC_Os02g38180.1	161	-24.01	161	-24.01	1537879	21	1515	87
miR529b	LOC_Os02g38280.1	161	-25	161	-25	1537889	21	2415	2390
miR529b	LOC_Os02g38420.1	152	-22.6	152	-22.6	1537906	21	348	205
miR529b	LOC_Os02g38470.1	654	-87.13	174	-24.9	1537911	21	3832	3389 3182 3224 3329
miR529b	LOC_Os02g38508.1	166	-26.23	166	-26.23	1537919	21	1017	676
miR529b	LOC_Os02g38598.1	166	-26.23	166	-26.23	1537929	21	1148	676
miR529b	LOC_Os02g39550.1	175	-28.99	175	-28.99	1538050	21	3098	2021
miR529b	LOC_Os02g39550.2	175	-28.99	175	-28.99	1538051	21	2982	2021
miR529b	LOC_Os02g39550.3	175	-28.99	175	-28.99	1538052	21	3196	2119
miR529b	LOC_Os02g39630.1	170	-23.61	170	-23.61	1538064	21	1041	4
miR529b	LOC_Os02g39630.2	170	-23.61	170	-23.61	1538065	21	932	4
miR529b	LOC_Os02g39630.3	170	-23.61	170	-23.61	1538066	21	1046	4
miR529b	LOC_Os02g39900.1	156	-21.71	156	-21.71	1538102	21	3081	1527
miR529b	LOC_Os02g40040.1	159	-20.17	159	-20.17	1538126	21	788	667
miR529b	LOC_Os02g40180.1	163	-20.12	163	-20.12	1538143	21	2994	72
miR529b	LOC_Os02g40530.1	165	-24.77	165	-24.77	1538186	21	1457	1370

miR529b	LOC_Os02g41720.1	156	-21.71	156	-21.71	1538266	21	4029	3399
miR529b	LOC_Os02g42170.1	159	-20.22	159	-20.22	1538319	21	1034	888
miR529b	LOC_Os02g42320.1	162	-23.41	162	-23.41	1538340	21	1304	1160
miR529b	LOC_Os02g42320.2	162	-23.41	162	-23.41	1538341	21	1195	1051
miR529b	LOC_Os02g42320.3	162	-23.41	162	-23.41	1538342	21	1294	1150
miR529b	LOC_Os02g44102.1	171	-25.94	171	-25.94	1538564	21	1689	575
miR529b	LOC_Os02g44111.1	158	-20.88	158	-20.88	1538570	21	1658	697
miR529b	LOC_Os02g44111.2	158	-20.88	158	-20.88	1538571	21	1294	669
miR529b	LOC_Os02g44550.1	166	-21.35	166	-21.35	1538635	21	3882	1734
miR529b	LOC_Os02g45090.1	165	-22.01	165	-22.01	1538705	21	1648	1601
miR529b	LOC_Os02g45090.2	165	-22.01	165	-22.01	1538706	21	1538	1491
miR529b	LOC_Os02g45170.1	161	-20.3	161	-20.3	1538718	21	2899	27
miR529b	LOC_Os02g45200.1	169	-24.99	169	-24.99	1538720	21	2355	2239
miR529b	LOC_Os02g45410.1	168	-22.24	168	-22.24	1538743	21	273	47
miR529b	LOC_Os02g45480.1	157	-21.55	157	-21.55	1538749	21	2001	74
miR529b	LOC_Os02g45480.2	157	-21.55	157	-21.55	1538750	21	3510	74
miR529b	LOC_Os02g45480.3	157	-21.55	157	-21.55	1538751	21	3695	74
miR529b	LOC_Os02g45520.1	152	-22.44	152	-22.44	1538759	21	2966	1380
miR529b	LOC_Os02g45520.2	152	-22.44	152	-22.44	1538760	21	2487	1030
miR529b	LOC_Os02g46390.1	165	-21.55	165	-21.55	1538859	21	690	46
miR529b	LOC_Os02g46640.1	153	-23.65	153	-23.65	1538887	21	1075	833
miR529b	LOC_Os02g46940.1	163	-23.11	163	-23.11	1538924	21	1259	144
miR529b	LOC_Os02g47010.1	174	-25.91	174	-25.91	1538937	21	1739	589
miR529b	LOC_Os02g47020.1	149	-20.89	149	-20.89	1538938	21	1824	1598
miR529b	LOC_Os02g47040.1	161	-20.16	161	-20.16	1538940	21	567	35
miR529b	LOC_Os02g47180.1	158	-20.36	158	-20.36	1538956	21	2655	198
miR529b	LOC_Os02g47180.2	158	-20.36	158	-20.36	1538957	21	2597	198
miR529b	LOC_Os02g47430.1	165	-24.58	165	-24.58	1538992	21	435	83
miR529b	LOC_Os02g47880.1	316	-43.09	158	-22.35	1539052	21	2777	373 511
miR529b	LOC_Os02g47880.2	316	-43.09	158	-22.35	1539053	21	2707	373 511
miR529b	LOC_Os02g48010.1	166	-23.45	166	-23.45	1539068	21	4018	2371
miR529b	LOC_Os02g48150.1	157	-22.28	157	-22.28	1539085	21	1159	1110

miR529b	LOC_Os02g48800.1	168	-24.08	168	-24.08	1539172	21	805	1
miR529b	LOC_Os02g49480.1	153	-22.29	153	-22.29	1539254	21	1678	762
miR529b	LOC_Os02g50840.1	165	-21.81	165	-21.81	1539422	21	6170	33
miR529b	LOC_Os02g50840.2	165	-21.81	165	-21.81	1539423	21	6076	33
miR529b	LOC_Os02g51390.1	168	-22.91	168	-22.91	1539487	21	3752	452
miR529b	LOC_Os02g51390.2	168	-22.91	168	-22.91	1539488	21	3374	452
miR529b	LOC_Os02g52510.1	152	-21.26	152	-21.26	1539638	21	3465	1977
miR529b	LOC_Os02g53720.1	156	-20.18	156	-20.18	1539792	21	1708	558
miR529b	LOC_Os02g53720.2	156	-20.18	156	-20.18	1539793	21	1608	458
miR529b	LOC_Os02g53750.2	174	-31.89	174	-31.89	1539798	21	3121	1324
miR529b	LOC_Os02g53770.1	161	-20.43	161	-20.43	1539799	21	3611	28
miR529b	LOC_Os02g54110.1	154	-20.29	154	-20.29	1539840	21	4192	1943
miR529b	LOC_Os02g54160.1	153	-20.11	153	-20.11	1539851	21	1703	1613
miR529b	LOC_Os02g54160.2	153	-20.11	153	-20.11	1539852	21	1778	1688
miR529b	LOC_Os02g54820.1	154	-20.77	154	-20.77	1539921	21	3664	2863
miR529b	LOC_Os02g55000.1	146	-21.69	146	-21.69	1539942	21	2017	680
miR529b	LOC_Os02g55130.1	162	-20.03	162	-20.03	1539957	21	2807	250
miR529b	LOC_Os02g55130.2	162	-20.03	162	-20.03	1539958	21	2663	106
miR529b	LOC_Os02g55130.3	162	-20.03	162	-20.03	1539959	21	2908	250
miR529b	LOC_Os02g55130.5	162	-20.03	162	-20.03	1539961	21	2912	250
miR529b	LOC_Os02g55770.4	168	-22.71	168	-22.71	1540037	21	5708	5677
miR529b	LOC_Os02g55770.5	168	-22.71	168	-22.71	1540038	21	2977	2946
miR529b	LOC_Os02g56100.1	165	-23.61	165	-23.61	1540083	21	3008	32
miR529b	LOC_Os02g56530.1	163	-24.54	163	-24.54	1540135	21	2308	1250
miR529b	LOC_Os02g56850.2	167	-24.5	167	-24.5	1540171	21	2176	91
miR529b	LOC_Os02g56850.3	167	-24.5	167	-24.5	1540172	21	2160	172
miR529b	LOC_Os02g57610.1	168	-22.53	168	-22.53	1540293	21	2399	1
miR529b	LOC_Os02g57750.1	161	-20.56	161	-20.56	1540314	21	2985	175
miR529b	LOC_Os02g57750.2	161	-20.56	161	-20.56	1540315	21	3292	125
miR529b	LOC_Os03g01070.1	159	-21.03	159	-21.03	1540464	21	2748	2554
miR529b	LOC_Os03g01230.1	165	-20.37	165	-20.37	1540488	21	797	490
miR529b	LOC_Os03g01230.2	165	-20.37	165	-20.37	1540489	21	733	426

miR529b	LOC_Os03g01350.1	165	-20.29	165	-20.29	1540504	21	3086	90
miR529b	LOC_Os03g01580.1	155	-21.96	155	-21.96	1540530	21	3882	3493
miR529b	LOC_Os03g01580.2	155	-21.96	155	-21.96	1540531	21	3870	3481
miR529b	LOC_Os03g01750.1	170	-21.42	170	-21.42	1540554	21	1651	513
miR529b	LOC_Os03g01750.2	170	-21.42	170	-21.42	1540555	21	1605	467
miR529b	LOC_Os03g01750.4	170	-21.42	170	-21.42	1540556	21	1858	513
miR529b	LOC_Os03g01750.5	170	-21.42	170	-21.42	1540557	21	1752	513
miR529b	LOC_Os03g01750.6	170	-21.42	170	-21.42	1540558	21	2024	513
miR529b	LOC_Os03g01750.7	170	-21.42	170	-21.42	1540559	21	2128	617
miR529b	LOC_Os03g02200.1	160	-20.32	160	-20.32	1540626	21	1400	8
miR529b	LOC_Os03g02200.2	160	-20.32	160	-20.32	1540627	21	1397	8
miR529b	LOC_Os03g02200.3	160	-20.32	160	-20.32	1540628	21	1380	8
miR529b	LOC_Os03g02320.1	166	-25.14	166	-25.14	1540640	21	2930	1
miR529b	LOC_Os03g02320.2	166	-25.14	166	-25.14	1540641	21	3004	1
miR529b	LOC_Os03g02440.1	157	-20.02	157	-20.02	1540656	21	4464	3175
miR529b	LOC_Os03g02440.2	157	-20.02	157	-20.02	1540657	21	4051	2762
miR529b	LOC_Os03g02900.1	156	-23.66	156	-23.66	1540725	21	1959	1771
miR529b	LOC_Os03g03510.1	158	-21.31	158	-21.31	1540798	21	2284	968
miR529b	LOC_Os03g03510.2	158	-21.31	158	-21.31	1540799	21	2395	968
miR529b	LOC_Os03g04020.1	160	-20.83	160	-20.83	1540864	21	1591	1
miR529b	LOC_Os03g04020.2	160	-20.83	160	-20.83	1540865	21	1700	1
miR529b	LOC_Os03g04970.1	162	-24.78	162	-24.78	1540978	21	2269	981
miR529b	LOC_Os03g05730.1	315	-41.35	159	-21.33	1541077	21	2915	2861 2571
miR529b	LOC_Os03g06420.1	156	-20.42	156	-20.42	1541159	21	2345	1906
miR529b	LOC_Os03g06420.2	156	-20.42	156	-20.42	1541160	21	2487	1906
miR529b	LOC_Os03g06420.3	156	-20.42	156	-20.42	1541161	21	2442	2003
miR529b	LOC_Os03g06420.4	156	-20.42	156	-20.42	1541162	21	2601	2162
miR529b	LOC_Os03g06570.1	154	-21.45	154	-21.45	1541177	21	1594	23
miR529b	LOC_Os03g06720.1	155	-21.4	155	-21.4	1541197	21	2983	1284
miR529b	LOC_Os03g07200.1	164	-20.71	164	-20.71	1541262	21	1589	1532
miR529b	LOC_Os03g07200.2	164	-20.71	164	-20.71	1541263	21	1491	1433
miR529b	LOC_Os03g07580.1	161	-21.31	161	-21.31	1541312	21	4354	3829

miR529b	LOC_Os03g07720.1	165	-23.83	165	-23.83	1541326	21	2193	388
miR529b	LOC_Os03g07970.1	156	-21.33	156	-21.33	1541352	21	2148	284
miR529b	LOC_Os03g07980.1	173	-22.16	173	-22.16	1541353	21	748	163
miR529b	LOC_Os03g08280.1	162	-20.37	162	-20.37	1541394	21	1209	523
miR529b	LOC_Os03g08280.2	162	-20.37	162	-20.37	1541395	21	1107	523
miR529b	LOC_Os03g08280.3	162	-20.37	162	-20.37	1541396	21	1194	610
miR529b	LOC_Os03g08530.1	167	-20.26	167	-20.26	1541433	21	2214	991
miR529b	LOC_Os03g08700.1	166	-23.9	166	-23.9	1541457	21	3972	2361
miR529b	LOC_Os03g10800.1	168	-22.71	168	-22.71	1541652	21	3189	342
miR529b	LOC_Os03g10800.2	168	-22.71	168	-22.71	1541653	21	3298	461
miR529b	LOC_Os03g10800.3	168	-22.71	168	-22.71	1541654	21	3518	681
miR529b	LOC_Os03g10800.4	168	-22.71	168	-22.71	1541655	21	3417	342
miR529b	LOC_Os03g11240.1	159	-20.96	159	-20.96	1541706	21	4577	168
miR529b	LOC_Os03g11300.1	150	-20.42	150	-20.42	1541712	21	1400	998
miR529b	LOC_Os03g11300.2	150	-20.42	150	-20.42	1541713	21	1476	998
miR529b	LOC_Os03g11310.1	162	-22.71	162	-22.71	1541714	21	2113	1
miR529b	LOC_Os03g11440.1	313	-44.87	157	-23.81	1541734	21	1982	8 1256
miR529b	LOC_Os03g11490.1	165	-21.34	165	-21.34	1541739	21	3681	28
miR529b	LOC_Os03g12250.1	166	-24.54	166	-24.54	1541842	21	2657	81
miR529b	LOC_Os03g12840.1	154	-24.62	154	-24.62	1541917	21	2070	1116
miR529b	LOC_Os03g12840.2	154	-24.62	154	-24.62	1541918	21	1780	1116
miR529b	LOC_Os03g12840.3	154	-24.62	154	-24.62	1541919	21	1877	1116
miR529b	LOC_Os03g12879.1	316	-42.59	165	-22.01	1541923	21	1193	185 228
miR529b	LOC_Os03g12990.1	154	-22.37	154	-22.37	1541947	21	838	86
miR529b	LOC_Os03g13460.1	168	-22.46	168	-22.46	1542001	21	3291	3018
miR529b	LOC_Os03g13460.2	168	-22.46	168	-22.46	1542002	21	3715	3442
miR529b	LOC_Os03g13680.1	145	-20.29	145	-20.29	1542025	21	2109	1577
miR529b	LOC_Os03g13810.1	159	-21.02	159	-21.02	1542042	21	2361	4
miR529b	LOC_Os03g13810.2	159	-21.02	159	-21.02	1542043	21	2353	2
miR529b	LOC_Os03g13810.3	159	-21.02	159	-21.02	1542044	21	3344	4
miR529b	LOC_Os03g14205.1	161	-20.08	161	-20.08	1542103	21	1266	1
miR529b	LOC_Os03g14860.1	165	-24.72	165	-24.72	1542177	21	1287	506

miR529b	LOC_Os03g14950.1	159	-21.25	159	-21.25	1542187	21	2103	43
miR529b	LOC_Os03g15040.1	162	-23.61	162	-23.61	1542198	21	3441	176
miR529b	LOC_Os03g15040.2	162	-23.61	162	-23.61	1542199	21	3449	176
miR529b	LOC_Os03g15710.1	166	-22.18	166	-22.18	1542274	21	1806	1707
miR529b	LOC_Os03g15890.1	157	-21.94	157	-21.94	1542303	21	1464	602
miR529b	LOC_Os03g15890.2	157	-21.94	157	-21.94	1542304	21	1449	602
miR529b	LOC_Os03g15890.3	157	-21.94	157	-21.94	1542305	21	1827	602
miR529b	LOC_Os03g16390.1	160	-20.74	160	-20.74	1542385	21	1937	656
miR529b	LOC_Os03g16530.1	153	-20.34	153	-20.34	1542400	21	6336	1392
miR529b	LOC_Os03g17270.1	160	-21.27	160	-21.27	1542497	21	2288	370
miR529b	LOC_Os03g17270.2	160	-21.27	160	-21.27	1542498	21	2442	370
miR529b	LOC_Os03g17570.1	163	-20.01	163	-20.01	1542530	21	3610	1582
miR529b	LOC_Os03g17570.2	163	-20.01	163	-20.01	1542531	21	3596	1582
miR529b	LOC_Os03g17570.3	163	-20.01	163	-20.01	1542532	21	3591	1582
miR529b	LOC_Os03g17570.4	163	-20.01	163	-20.01	1542533	21	4061	2033
miR529b	LOC_Os03g17570.5	163	-20.01	163	-20.01	1542534	21	3497	1469
miR529b	LOC_Os03g17570.6	163	-20.01	163	-20.01	1542535	21	3691	1663
miR529b	LOC_Os03g18430.1	308	-42.02	158	-21.22	1542638	21	2821	2024 1358
miR529b	LOC_Os03g18500.1	161	-21.49	161	-21.49	1542645	21	1226	1139
miR529b	LOC_Os03g18500.2	161	-21.49	161	-21.49	1542646	21	1136	1053
miR529b	LOC_Os03g18900.1	168	-24.98	168	-24.98	1542691	21	909	251
miR529b	LOC_Os03g19010.1	161	-21.08	161	-21.08	1542701	21	1211	78
miR529b	LOC_Os03g19150.1	174	-26.46	174	-26.46	1542717	21	2208	631
miR529b	LOC_Os03g19270.1	156	-20.62	156	-20.62	1542730	21	1058	995
miR529b	LOC_Os03g19270.2	156	-20.62	156	-20.62	1542731	21	1018	955
miR529b	LOC_Os03g19270.3	156	-20.62	156	-20.62	1542732	21	1009	946
miR529b	LOC_Os03g19270.4	156	-20.62	156	-20.62	1542733	21	1152	1089
miR529b	LOC_Os03g20470.1	160	-24.14	160	-24.14	1542885	21	1148	196
miR529b	LOC_Os03g20470.2	160	-24.14	160	-24.14	1542886	21	1165	192
miR529b	LOC_Os03g20500.1	157	-20.14	157	-20.14	1542889	21	1679	1291
miR529b	LOC_Os03g21360.1	166	-20.05	166	-20.05	1542985	21	1113	1082
miR529b	LOC_Os03g21680.1	160	-20.16	160	-20.16	1543026	21	4395	74

miR529b	LOC_Os03g21680.2	160	-20.16	160	-20.16	1543027	21	4293	74
miR529b	LOC_Os03g21680.3	160	-20.16	160	-20.16	1543028	21	4460	74
miR529b	LOC_Os03g21680.4	160	-20.16	160	-20.16	1543029	21	4560	74
miR529b	LOC_Os03g21850.1	163	-22.6	163	-22.6	1543048	21	3700	2898
miR529b	LOC_Os03g21850.2	163	-22.6	163	-22.6	1543049	21	3424	2804
miR529b	LOC_Os03g23110.1	167	-21.93	167	-21.93	1543211	21	4929	3693
miR529b	LOC_Os03g24040.1	171	-29.44	171	-29.44	1543249	21	1493	1463
miR529b	LOC_Os03g25050.1	161	-24.34	161	-24.34	1543352	21	671	593
miR529b	LOC_Os03g25170.1	174	-26.46	174	-26.46	1543364	21	684	631
miR529b	LOC_Os03g25330.1	163	-22.35	163	-22.35	1543384	21	1473	16
miR529b	LOC_Os03g25580.1	166	-21.94	166	-21.94	1543417	21	1989	204
miR529b	LOC_Os03g25720.1	157	-21.94	157	-21.94	1543431	21	1848	1128
miR529b	LOC_Os03g26570.1	163	-22.51	163	-22.51	1543510	21	3285	2591
miR529b	LOC_Os03g27390.1	166	-21.43	166	-21.43	1543602	21	2558	949
miR529b	LOC_Os03g27390.2	166	-21.43	166	-21.43	1543603	21	2581	949
miR529b	LOC_Os03g27590.1	162	-20.54	162	-20.54	1543627	21	1999	112
miR529b	LOC_Os03g27590.2	162	-20.54	162	-20.54	1543628	21	2766	112
miR529b	LOC_Os03g27590.3	162	-20.54	162	-20.54	1543629	21	2807	112
miR529b	LOC_Os03g27780.1	167	-23.04	167	-23.04	1543644	21	2522	10
miR529b	LOC_Os03g27780.2	167	-23.04	167	-23.04	1543645	21	2606	10
miR529b	LOC_Os03g29400.1	165	-21.38	165	-21.38	1543772	21	675	214
miR529b	LOC_Os03g29410.1	155	-21.63	155	-21.63	1543773	21	1879	1847
miR529b	LOC_Os03g29790.1	157	-21.69	157	-21.69	1543806	21	4647	531
miR529b	LOC_Os03g29920.1	334	-46.86	169	-23.61	1543815	21	1228	75 33
miR529b	LOC_Os03g30190.1	161	-20.5	161	-20.5	1543847	21	1608	1583
miR529b	LOC_Os03g30810.1	157	-21.06	157	-21.06	1543910	21	3411	1082
miR529b	LOC_Os03g31520.1	153	-20.34	153	-20.34	1543989	21	1536	1431
miR529b	LOC_Os03g32170.1	154	-21	154	-21	1544049	21	1864	727
miR529b	LOC_Os03g32650.1	156	-21.71	156	-21.71	1544094	21	4593	2895
miR529b	LOC_Os03g33530.1	145	-20.29	145	-20.29	1544177	21	6084	5291
miR529b	LOC_Os03g33590.1	161	-21.66	161	-21.66	1544183	21	1839	332
miR529b	LOC_Os03g33590.2	161	-21.66	161	-21.66	1544184	21	1815	308

miR529b	LOC_Os03g33810.1	153	-20.34	153	-20.34	1544205	21	1641	1611
miR529b	LOC_Os03g35710.1	174	-21.84	174	-21.84	1544293	21	1521	1401
miR529b	LOC_Os03g35720.1	161	-24.99	161	-24.99	1544294	21	981	956
miR529b	LOC_Os03g35750.1	159	-20.89	159	-20.89	1544297	21	357	257
miR529b	LOC_Os03g35788.1	149	-21.86	149	-21.86	1544298	21	5724	4931
miR529b	LOC_Os03g35990.1	160	-20.8	160	-20.8	1544317	21	1455	674
miR529b	LOC_Os03g36000.1	161	-21.9	161	-21.9	1544318	21	3555	3040
miR529b	LOC_Os03g36460.1	164	-22.28	164	-22.28	1544355	21	519	8
miR529b	LOC_Os03g36760.1	164	-20.86	164	-20.86	1544385	21	4181	949
miR529b	LOC_Os03g36760.2	164	-20.86	164	-20.86	1544386	21	4090	949
miR529b	LOC_Os03g37270.1	154	-21.03	154	-21.03	1544431	21	1896	1506
miR529b	LOC_Os03g37520.1	154	-20.41	154	-20.41	1544448	21	492	56
miR529b	LOC_Os03g37940.1	154	-23.15	154	-23.15	1544491	21	294	126
miR529b	LOC_Os03g38010.1	169	-23.26	169	-23.26	1544505	21	1813	906
miR529b	LOC_Os03g38270.1	157	-21.1	157	-21.1	1544529	21	2217	153
miR529b	LOC_Os03g39250.1	157	-21.69	157	-21.69	1544631	21	3717	492
miR529b	LOC_Os03g39330.1	161	-21.34	161	-21.34	1544636	21	3603	387
miR529b	LOC_Os03g39362.1	165	-20.64	165	-20.64	1544639	21	315	114
miR529b	LOC_Os03g40160.1	327	-47.72	164	-24.44	1544716	21	3226	1830 3138
miR529b	LOC_Os03g41339.1	156	-20.15	156	-20.15	1544817	21	468	20
miR529b	LOC_Os03g41840.1	145	-20.29	145	-20.29	1544865	21	1305	548
miR529b	LOC_Os03g42070.1	161	-23.63	161	-23.63	1544887	21	2173	404
miR529b	LOC_Os03g42100.1	160	-21.25	160	-21.25	1544890	21	933	200
miR529b	LOC_Os03g42370.1	166	-20.28	166	-20.28	1544918	21	3090	329
miR529b	LOC_Os03g42660.1	156	-20.55	156	-20.55	1544944	21	1926	626
miR529b	LOC_Os03g42770.1	164	-20.92	164	-20.92	1544955	21	2162	459
miR529b	LOC_Os03g43684.1	160	-22.52	160	-22.52	1545051	21	5210	3089
miR529b	LOC_Os03g43720.1	159	-21.25	159	-21.25	1545054	21	2380	1993
miR529b	LOC_Os03g43720.2	159	-21.25	159	-21.25	1545055	21	2405	2018
miR529b	LOC_Os03g43720.3	159	-21.25	159	-21.25	1545056	21	2428	2041
miR529b	LOC_Os03g43720.4	159	-21.25	159	-21.25	1545057	21	2273	1886
miR529b	LOC_Os03g43720.5	159	-21.25	159	-21.25	1545058	21	2337	1960

miR529b	LOC_Os03g43720.6	159	-21.25	159	-21.25	1545059	21	2961	2574
miR529b	LOC_Os03g43990.1	168	-24.98	168	-24.98	1545090	21	2694	2654
miR529b	LOC_Os03g44020.1	152	-20.28	152	-20.28	1545095	21	1663	1462
miR529b	LOC_Os03g44890.1	161	-23.03	161	-23.03	1545196	21	3543	2706
miR529b	LOC_Os03g45300.1	161	-25.45	161	-25.45	1545235	21	4183	3997
miR529b	LOC_Os03g45390.1	166	-23.01	166	-23.01	1545244	21	1815	22
miR529b	LOC_Os03g45750.1	160	-21.44	160	-21.44	1545276	21	1263	10
miR529b	LOC_Os03g46330.1	151	-20.17	151	-20.17	1545340	21	6123	6071
miR529b	LOC_Os03g46340.1	151	-20.17	151	-20.17	1545341	21	3131	1769
miR529b	LOC_Os03g46440.3	151	-20.15	151	-20.15	1545353	21	3770	1082
miR529b	LOC_Os03g46540.1	165	-20.32	165	-20.32	1545364	21	525	19
miR529b	LOC_Os03g47740.1	158	-25.52	158	-25.52	1545489	21	2894	2647
miR529b	LOC_Os03g47740.2	158	-25.52	158	-25.52	1545490	21	2989	2742
miR529b	LOC_Os03g48140.1	151	-21.23	151	-21.23	1545540	21	5166	2406
miR529b	LOC_Os03g48810.1	169	-23.19	169	-23.19	1545617	21	1951	1286
miR529b	LOC_Os03g49710.1	164	-22.83	164	-22.83	1545740	21	882	824
miR529b	LOC_Os03g49800.1	158	-20.42	158	-20.42	1545751	21	2990	2878
miR529b	LOC_Os03g49940.1	161	-24.06	161	-24.06	1545765	21	2040	1144
miR529b	LOC_Os03g50100.1	161	-20.2	161	-20.2	1545784	21	3451	1414
miR529b	LOC_Os03g50970.1	151	-22.27	151	-22.27	1545877	21	2446	630
miR529b	LOC_Os03g51580.1	161	-23.14	161	-23.14	1545947	21	2011	1834
miR529b	LOC_Os03g52390.1	157	-20.26	157	-20.26	1546040	21	503	343
miR529b	LOC_Os03g52794.1	162	-20.53	162	-20.53	1546088	21	11361	7855
miR529b	LOC_Os03g52900.1	165	-21.77	165	-21.77	1546100	21	2772	231
miR529b	LOC_Os03g52950.1	164	-22.71	164	-22.71	1546105	21	3183	982
miR529b	LOC_Os03g53250.1	160	-20.1	160	-20.1	1546139	21	3176	1834
miR529b	LOC_Os03g53980.1	159	-20.13	159	-20.13	1546237	21	693	410
miR529b	LOC_Os03g53980.2	159	-20.13	159	-20.13	1546238	21	613	329
miR529b	LOC_Os03g54114.1	157	-22.31	157	-22.31	1546256	21	492	172
miR529b	LOC_Os03g54840.1	160	-21.55	160	-21.55	1546280	21	474	39
miR529b	LOC_Os03g54850.1	161	-22.53	161	-22.53	1546281	21	1885	60
miR529b	LOC_Os03g55850.1	157	-21.05	157	-21.05	1546406	21	1375	631

miR529b	LOC_Os03g55950.3	159	-22.49	159	-22.49	1546418	21	2437	249
miR529b	LOC_Os03g56310.1	163	-20.66	163	-20.66	1546460	21	2422	1525
miR529b	LOC_Os03g56310.2	163	-20.66	163	-20.66	1546461	21	2415	1525
miR529b	LOC_Os03g56310.3	163	-20.66	163	-20.66	1546462	21	2556	1659
miR529b	LOC_Os03g56310.4	163	-20.66	163	-20.66	1546463	21	1923	1026
miR529b	LOC_Os03g56590.1	161	-26.84	161	-26.84	1546497	21	1853	734
miR529b	LOC_Os03g56730.1	328	-42.38	164	-21.29	1546517	21	1656	1185 1225
miR529b	LOC_Os03g56730.2	328	-42.38	164	-21.29	1546518	21	1701	1185 1225
miR529b	LOC_Os03g57569.1	168	-23.18	168	-23.18	1546635	21	2196	1029
miR529b	LOC_Os03g57569.2	168	-23.18	168	-23.18	1546636	21	1898	731
miR529b	LOC_Os03g57854.1	161	-23.62	161	-23.62	1546668	21	1844	101
miR529b	LOC_Os03g57854.2	161	-23.62	161	-23.62	1546669	21	1864	101
miR529b	LOC_Os03g57940.1	150	-21.96	150	-21.96	1546681	21	2723	1753
miR529b	LOC_Os03g57940.2	150	-21.96	150	-21.96	1546682	21	2715	1745
miR529b	LOC_Os03g57940.3	150	-21.96	150	-21.96	1546683	21	2764	1794
miR529b	LOC_Os03g57940.4	150	-21.96	150	-21.96	1546684	21	2791	1821
miR529b	LOC_Os03g58220.1	161	-20.84	161	-20.84	1546720	21	1242	863
miR529b	LOC_Os03g58372.1	162	-21.54	162	-21.54	1546735	21	2901	497
miR529b	LOC_Os03g58470.1	166	-26.62	166	-26.62	1546744	21	1414	1043
miR529b	LOC_Os03g58870.1	163	-20.45	163	-20.45	1546789	21	987	835
miR529b	LOC_Os03g59170.1	159	-23.66	159	-23.66	1546823	21	1531	314
miR529b	LOC_Os03g59250.1	166	-22.95	166	-22.95	1546832	21	1455	316
miR529b	LOC_Os03g59680.1	159	-20.11	159	-20.11	1546876	21	2266	944
miR529b	LOC_Os03g60150.2	159	-20.38	159	-20.38	1546928	21	2345	790
miR529b	LOC_Os03g60180.1	173	-28.2	173	-28.2	1546930	21	2162	1827
miR529b	LOC_Os03g60200.1	163	-23.62	163	-23.62	1546933	21	3085	2754
miR529b	LOC_Os03g60920.1	168	-24.99	168	-24.99	1547017	21	1431	1
miR529b	LOC_Os03g61010.1	153	-20.56	153	-20.56	1547025	21	2061	1741
miR529b	LOC_Os03g61620.1	168	-24.98	168	-24.98	1547101	21	3188	85
miR529b	LOC_Os03g61620.2	168	-24.98	168	-24.98	1547102	21	3452	85
miR529b	LOC_Os03g61800.1	160	-21.18	160	-21.18	1547124	21	2278	300
miR529b	LOC_Os03g61800.2	160	-21.18	160	-21.18	1547125	21	2290	300

miR529b	LOC_Os03g62090.1	161	-20.17	161	-20.17	1547169	21	4365	222
miR529b	LOC_Os03g62224.1	171	-24.28	171	-24.28	1547188	21	354	84
miR529b	LOC_Os03g62340.1	165	-25.76	165	-25.76	1547200	21	3627	3514
miR529b	LOC_Os03g62379.1	164	-21.78	164	-21.78	1547204	21	657	53
miR529b	LOC_Os03g62650.1	165	-23.73	165	-23.73	1547244	21	3232	478
miR529b	LOC_Os03g62650.2	165	-23.73	165	-23.73	1547245	21	3392	478
miR529b	LOC_Os03g62650.3	165	-23.73	165	-23.73	1547246	21	3209	478
miR529b	LOC_Os03g62730.1	161	-20.56	161	-20.56	1547260	21	3611	83
miR529b	LOC_Os03g62730.2	161	-20.56	161	-20.56	1547261	21	3627	83
miR529b	LOC_Os03g62730.3	161	-20.56	161	-20.56	1547262	21	3695	83
miR529b	LOC_Os03g62830.1	173	-24.33	173	-24.33	1547277	21	2034	1087
miR529b	LOC_Os03g63710.1	161	-20.64	161	-20.64	1547382	21	2249	708
miR529b	LOC_Os03g63710.2	161	-20.64	161	-20.64	1547383	21	2200	914
miR529b	LOC_Os03g64080.1	162	-20.53	162	-20.53	1547430	21	6225	791
miR529b	LOC_Os03g64210.1	156	-20.31	156	-20.31	1547443	21	2439	1054
miR529b	LOC_Os03g64210.2	156	-20.31	156	-20.31	1547444	21	2599	1054
miR529b	LOC_Os03g64210.3	156	-20.31	156	-20.31	1547445	21	2401	1054
miR529b	LOC_Os03g64250.5	168	-23.5	168	-23.5	1547451	21	2249	670
miR529b	LOC_Os04g01030.1	158	-22.08	158	-22.08	1547480	21	4293	2284
miR529b	LOC_Os04g01380.1	162	-26.86	162	-26.86	1547522	21	1614	612
miR529b	LOC_Os04g02420.1	149	-21.35	149	-21.35	1547634	21	2778	460
miR529b	LOC_Os04g02920.1	166	-20.27	166	-20.27	1547695	21	2552	358
miR529b	LOC_Os04g03490.1	157	-21.69	157	-21.69	1547747	21	4005	594
miR529b	LOC_Os04g04170.1	169	-23.84	169	-23.84	1547810	21	1689	548
miR529b	LOC_Os04g04490.1	157	-21.69	157	-21.69	1547842	21	4401	531
miR529b	LOC_Os04g05030.1	161	-20.09	161	-20.09	1547891	21	1622	20
miR529b	LOC_Os04g05140.1	154	-20.29	154	-20.29	1547902	21	1608	14
miR529b	LOC_Os04g05270.1	154	-20.29	154	-20.29	1547915	21	1650	14
miR529b	LOC_Os04g05680.1	157	-22.29	157	-22.29	1547951	21	2418	2393
miR529b	LOC_Os04g05770.1	167	-26.67	167	-26.67	1547959	21	2019	1454
miR529b	LOC_Os04g06130.1	167	-21.93	167	-21.93	1547993	21	4980	3915
miR529b	LOC_Os04g06420.1	154	-20.29	154	-20.29	1548018	21	1686	14

miR529b	LOC_Os04g07690.1	164	-21.57	164	-21.57	1548139	21	513	10
miR529b	LOC_Os04g08034.1	156	-21.26	156	-21.26	1548172	21	2904	712
miR529b	LOC_Os04g08034.2	156	-21.26	156	-21.26	1548173	21	2847	655
miR529b	LOC_Os04g08034.3	156	-21.26	156	-21.26	1548174	21	2708	516
miR529b	LOC_Os04g08034.6	156	-21.26	156	-21.26	1548177	21	1655	264
miR529b	LOC_Os04g08784.1	145	-20.29	145	-20.29	1548254	21	1044	251
miR529b	LOC_Os04g08804.1	169	-23.84	169	-23.84	1548259	21	2901	1973
miR529b	LOC_Os04g09710.1	162	-22.24	162	-22.24	1548310	21	1752	1725
miR529b	LOC_Os04g10380.1	165	-24.08	165	-24.08	1548378	21	5131	1
miR529b	LOC_Os04g10620.1	157	-21.69	157	-21.69	1548396	21	4422	531
miR529b	LOC_Os04g12120.1	168	-24.98	168	-24.98	1548549	21	978	49
miR529b	LOC_Os04g13830.1	154	-20.29	154	-20.29	1548703	21	375	14
miR529b	LOC_Os04g13970.1	161	-20.3	161	-20.3	1548717	21	1686	1198
miR529b	LOC_Os04g14340.1	163	-20.51	163	-20.51	1548755	21	3398	1649
miR529b	LOC_Os04g14890.1	167	-23.13	167	-23.13	1548811	21	4494	3486
miR529b	LOC_Os04g15820.1	172	-25.16	172	-25.16	1548854	21	3199	2435
miR529b	LOC_Os04g16280.1	171	-21.78	171	-21.78	1548895	21	1845	1453
miR529b	LOC_Os04g16450.1	328	-47.34	168	-23.78	1548911	21	1629	1509 1495
miR529b	LOC_Os04g16722.1	153	-21.5	153	-21.5	1548938	21	4203	2186
miR529b	LOC_Os04g16896.1	153	-21.5	153	-21.5	1548939	21	402	138
miR529b	LOC_Os04g18340.1	171	-20.71	171	-20.71	1549132	21	2592	549
miR529b	LOC_Os04g18660.1	310	-42.61	156	-21.59	1549163	21	2622	2379 2139
miR529b	LOC_Os04g18700.1	164	-20.26	164	-20.26	1549167	21	5565	3436
miR529b	LOC_Os04g19820.1	161	-20.17	161	-20.17	1549277	21	3303	24
miR529b	LOC_Os04g20090.1	178	-28.3	178	-28.3	1549301	21	2415	1319
miR529b	LOC_Os04g20180.1	310	-40.4	155	-20.2	1549310	21	2890	2246 2393
miR529b	LOC_Os04g20490.1	154	-21.25	154	-21.25	1549345	21	306	53
miR529b	LOC_Os04g20960.1	154	-22.12	154	-22.12	1549389	21	3648	140
miR529b	LOC_Os04g21100.1	173	-25.41	173	-25.41	1549400	21	2229	1253
miR529b	LOC_Os04g21560.1	157	-21.69	157	-21.69	1549444	21	4578	531
miR529b	LOC_Os04g21890.1	159	-21.04	159	-21.04	1549478	21	2799	1805
miR529b	LOC_Os04g23130.1	165	-22.28	165	-22.28	1549596	21	492	45

miR529b	LOC_Os04g23620.1	160	-20.53	160	-20.53	1549640	21	2130	807
miR529b	LOC_Os04g23890.2	168	-22.71	168	-22.71	1549660	21	3248	3153
miR529b	LOC_Os04g24130.1	174	-24.07	174	-24.07	1549683	21	1302	464
miR529b	LOC_Os04g24670.1	174	-22.74	174	-22.74	1549745	21	417	62
miR529b	LOC_Os04g24820.1	154	-20.28	154	-20.28	1549759	21	1725	1694
miR529b	LOC_Os04g26320.1	162	-21.86	162	-21.86	1549914	21	996	901
miR529b	LOC_Os04g26850.2	165	-22.22	165	-22.22	1549961	21	1493	1305
miR529b	LOC_Os04g27270.1	165	-20.59	165	-20.59	1550003	21	1486	889
miR529b	LOC_Os04g27400.1	168	-21.58	168	-21.58	1550014	21	1524	1379
miR529b	LOC_Os04g28300.1	161	-22.41	161	-22.41	1550101	21	2535	2432
miR529b	LOC_Os04g28620.1	161	-24.19	161	-24.19	1550131	21	1866	1730
miR529b	LOC_Os04g29600.1	156	-21.71	156	-21.71	1550219	21	2436	879
miR529b	LOC_Os04g30300.1	161	-25.68	161	-25.68	1550287	21	2082	1505
miR529b	LOC_Os04g30870.1	321	-44.64	164	-23.28	1550345	21	3486	1806 3397
miR529b	LOC_Os04g30920.1	165	-22.28	165	-22.28	1550352	21	855	728
miR529b	LOC_Os04g32330.1	162	-21.06	162	-21.06	1550498	21	1894	22
miR529b	LOC_Os04g32330.2	162	-21.06	162	-21.06	1550499	21	1866	1
miR529b	LOC_Os04g32550.1	161	-20.33	161	-20.33	1550519	21	854	2
miR529b	LOC_Os04g32670.1	164	-20.85	164	-20.85	1550535	21	2826	1
miR529b	LOC_Os04g32900.1	157	-20.2	157	-20.2	1550564	21	468	426
miR529b	LOC_Os04g33080.1	162	-20.44	162	-20.44	1550588	21	2573	2333
miR529b	LOC_Os04g33080.2	162	-20.44	162	-20.44	1550589	21	2558	2450
miR529b	LOC_Os04g33080.3	162	-20.44	162	-20.44	1550590	21	2619	2466
miR529b	LOC_Os04g33600.1	161	-23.11	161	-23.11	1550652	21	1584	14
miR529b	LOC_Os04g33610.1	159	-21.1	159	-21.1	1550653	21	1252	125
miR529b	LOC_Os04g33810.1	170	-24.03	170	-24.03	1550674	21	1005	17
miR529b	LOC_Os04g35430.1	159	-20.01	159	-20.01	1550843	21	4486	1698
miR529b	LOC_Os04g35510.1	320	-40.46	164	-20.4	1550851	21	1606	915 1225
miR529b	LOC_Os04g35510.2	320	-40.46	164	-20.4	1550852	21	1555	915 1225
miR529b	LOC_Os04g37590.1	165	-23.33	165	-23.33	1550983	21	1242	888
miR529b	LOC_Os04g37840.1	155	-20.37	155	-20.37	1551011	21	4551	4014
miR529b	LOC_Os04g37904.1	158	-20.59	158	-20.59	1551017	21	1768	1197

miR529b	LOC_Os04g37904.2	158	-20.59	158	-20.59	1551018	21	1765	1194
miR529b	LOC_Os04g37980.1	152	-22.16	152	-22.16	1551028	21	1834	626
miR529b	LOC_Os04g38290.1	162	-20.02	162	-20.02	1551056	21	3620	1662
miR529b	LOC_Os04g38380.1	157	-26.74	157	-26.74	1551067	21	762	214
miR529b	LOC_Os04g38410.1	155	-20.01	155	-20.01	1551070	21	1589	1109
miR529b	LOC_Os04g38960.1	158	-21.75	158	-21.75	1551146	21	1839	446
miR529b	LOC_Os04g39260.1	156	-22.48	156	-22.48	1551181	21	2453	1455
miR529b	LOC_Os04g39260.2	156	-22.48	156	-22.48	1551182	21	2570	1453
miR529b	LOC_Os04g39260.3	156	-22.48	156	-22.48	1551183	21	2470	1453
miR529b	LOC_Os04g39410.1	156	-20.84	156	-20.84	1551201	21	3371	313
miR529b	LOC_Os04g40360.1	156	-21.71	156	-21.71	1551310	21	4110	2895
miR529b	LOC_Os04g40850.1	312	-41.4	156	-21	1551375	21	1583	32 1505
miR529b	LOC_Os04g40850.2	156	-21	156	-21	1551376	21	1538	1460
miR529b	LOC_Os04g40970.1	164	-20.09	164	-20.09	1551396	21	3574	3337
miR529b	LOC_Os04g41950.1	171	-30.08	171	-30.08	1551508	21	2796	1815
miR529b	LOC_Os04g42470.1	145	-20.76	145	-20.76	1551570	21	2507	2148
miR529b	LOC_Os04g42470.2	145	-20.76	145	-20.76	1551571	21	2429	2070
miR529b	LOC_Os04g42470.3	145	-20.76	145	-20.76	1551572	21	2426	2067
miR529b	LOC_Os04g42470.4	145	-20.76	145	-20.76	1551573	21	2315	2067
miR529b	LOC_Os04g42470.5	145	-20.76	145	-20.76	1551574	21	2355	1996
miR529b	LOC_Os04g42470.6	145	-20.76	145	-20.76	1551575	21	2499	2140
miR529b	LOC_Os04g42470.7	145	-20.76	145	-20.76	1551576	21	2394	2140
miR529b	LOC_Os04g42470.8	145	-20.76	145	-20.76	1551577	21	1934	1575
miR529b	LOC_Os04g42480.1	173	-29.13	173	-29.13	1551578	21	3297	369
miR529b	LOC_Os04g42620.1	174	-27.24	174	-27.24	1551594	21	1883	205
miR529b	LOC_Os04g42740.1	149	-25.81	149	-25.81	1551604	21	2382	709
miR529b	LOC_Os04g43070.1	146	-22.71	146	-22.71	1551651	21	2476	1703
miR529b	LOC_Os04g43480.1	161	-24.99	161	-24.99	1551706	21	2241	2216
miR529b	LOC_Os04g43500.1	156	-21.71	156	-21.71	1551709	21	4251	3213
miR529b	LOC_Os04g43850.1	156	-21.71	156	-21.71	1551745	21	3315	1968
miR529b	LOC_Os04g44170.1	164	-21.79	164	-21.79	1551778	21	621	1
miR529b	LOC_Os04g44850.1	157	-20.61	157	-20.61	1551865	21	1918	150

miR529b	LOC_Os04g44950.1	159	-20.37	159	-20.37	1551881	21	1318	1262
miR529b	LOC_Os04g45490.1	159	-22.76	159	-22.76	1551945	21	2306	28
miR529b	LOC_Os04g46310.1	161	-20.31	161	-20.31	1552035	21	6429	3539
miR529b	LOC_Os04g46580.1	182	-33.78	182	-33.78	1552073	21	1518	812
miR529b	LOC_Os04g46740.1	169	-20.67	169	-20.67	1552093	21	1542	1499
miR529b	LOC_Os04g46740.2	169	-20.67	169	-20.67	1552094	21	1985	1945
miR529b	LOC_Os04g46990.1	159	-20.21	159	-20.21	1552129	21	1663	999
miR529b	LOC_Os04g48760.1	158	-21.46	158	-21.46	1552339	21	4296	57
miR529b	LOC_Os04g49500.1	159	-20.8	159	-20.8	1552420	21	3369	3229
miR529b	LOC_Os04g50216.1	161	-21.54	161	-21.54	1552513	21	1329	1278
miR529b	LOC_Os04g50920.1	165	-23.61	165	-23.61	1552541	21	1815	24
miR529b	LOC_Os04g51250.1	155	-23.18	155	-23.18	1552584	21	1160	1046
miR529b	LOC_Os04g51809.1	169	-20.93	169	-20.93	1552660	21	2132	942
miR529b	LOC_Os04g51809.2	169	-20.93	169	-20.93	1552661	21	1785	942
miR529b	LOC_Os04g51809.3	169	-20.93	169	-20.93	1552662	21	1658	942
miR529b	LOC_Os04g52060.1	170	-21.78	170	-21.78	1552699	21	2800	1812
miR529b	LOC_Os04g52190.1	162	-25.2	162	-25.2	1552713	21	2824	2495
miR529b	LOC_Os04g52200.1	162	-25.2	162	-25.2	1552714	21	3138	467
miR529b	LOC_Os04g53496.1	322	-43.67	163	-22.2	1552879	21	5424	4314 5082
miR529b	LOC_Os04g53720.1	161	-20.65	161	-20.65	1552910	21	3864	459
miR529b	LOC_Os04g55100.1	156	-22.62	156	-22.62	1553073	21	547	122
miR529b	LOC_Os04g55510.1	162	-22.57	162	-22.57	1553119	21	2891	89
miR529b	LOC_Os04g55560.2	161	-20.7	161	-20.7	1553125	21	2490	207
miR529b	LOC_Os04g55560.3	161	-20.7	161	-20.7	1553126	21	2501	221
miR529b	LOC_Os04g55640.1	158	-21.96	158	-21.96	1553135	21	1419	119
miR529b	LOC_Os04g56440.1	153	-20.35	153	-20.35	1553231	21	2566	1983
miR529b	LOC_Os04g56440.2	153	-20.35	153	-20.35	1553232	21	2643	2060
miR529b	LOC_Os04g56450.1	153	-20.35	153	-20.35	1553233	21	2162	621
miR529b	LOC_Os04g56450.2	153	-20.35	153	-20.35	1553234	21	2015	621
miR529b	LOC_Os04g56630.1	163	-22.43	163	-22.43	1553256	21	3323	752
miR529b	LOC_Os04g56630.2	163	-22.43	163	-22.43	1553257	21	2922	351
miR529b	LOC_Os04g57610.1	171	-21.24	171	-21.24	1553393	21	3361	3095

miR529b	LOC_Os04g57610.3	171	-21.24	171	-21.24	1553394	21	3349	3083
miR529b	LOC_Os04g57830.1	160	-20.56	160	-20.56	1553428	21	2052	13
miR529b	LOC_Os04g58140.1	150	-20.01	150	-20.01	1553459	21	2491	2443
miR529b	LOC_Os04g58140.2	150	-20.01	150	-20.01	1553460	21	3022	2974
miR529b	LOC_Os04g58250.1	152	-20.82	152	-20.82	1553476	21	2608	1447
miR529b	LOC_Os04g58430.1	162	-21.03	162	-21.03	1553498	21	906	189
miR529b	LOC_Os04g58580.1	158	-21.77	158	-21.77	1553517	21	1314	1019
miR529b	LOC_Os04g58580.2	158	-21.77	158	-21.77	1553518	21	1520	1228
miR529b	LOC_Os04g58720.1	157	-21.2	157	-21.2	1553537	21	3860	39
miR529b	LOC_Os04g58820.1	162	-23.68	162	-23.68	1553553	21	1293	277
miR529b	LOC_Os05g01060.1	152	-25.35	152	-25.35	1553676	21	2774	1937
miR529b	LOC_Os05g01060.2	152	-25.35	152	-25.35	1553677	21	2399	1562
miR529b	LOC_Os05g01240.1	160	-21.25	160	-21.25	1553698	21	3944	3902
miR529b	LOC_Os05g01240.5	160	-21.25	160	-21.25	1553702	21	3676	3422
miR529b	LOC_Os05g01256.1	161	-21.64	161	-21.64	1553704	21	1663	1351
miR529b	LOC_Os05g01256.2	161	-21.64	161	-21.64	1553705	21	1684	1388
miR529b	LOC_Os05g01290.1	160	-21.9	160	-21.9	1553711	21	984	64
miR529b	LOC_Os05g01320.1	160	-21.1	160	-21.1	1553716	21	1693	289
miR529b	LOC_Os05g01450.1	158	-21.44	158	-21.44	1553729	21	1273	1192
miR529b	LOC_Os05g01450.2	158	-21.44	158	-21.44	1553730	21	1193	1112
miR529b	LOC_Os05g01520.1	168	-24.98	168	-24.98	1553744	21	2496	2469
miR529b	LOC_Os05g01790.1	162	-21.27	162	-21.27	1553782	21	1314	53
miR529b	LOC_Os05g02790.1	330	-45.92	170	-25.62	1553908	21	3105	1092 1344
miR529b	LOC_Os05g03150.1	161	-23.59	161	-23.59	1553949	21	654	600
miR529b	LOC_Os05g03550.1	164	-22.75	164	-22.75	1553998	21	5167	1082
miR529b	LOC_Os05g04460.1	164	-20.29	164	-20.29	1554098	21	2181	1144
miR529b	LOC_Os05g04520.1	157	-23.99	157	-23.99	1554105	21	1632	53
miR529b	LOC_Os05g04630.1	157	-21.15	157	-21.15	1554114	21	1966	100
miR529b	LOC_Os05g04630.2	157	-21.15	157	-21.15	1554115	21	1961	92
miR529b	LOC_Os05g04630.3	157	-21.15	157	-21.15	1554116	21	1981	50
miR529b	LOC_Os05g04630.4	157	-21.15	157	-21.15	1554117	21	2023	50
miR529b	LOC_Os05g04630.5	157	-21.15	157	-21.15	1554118	21	2020	50

miR529b	LOC_Os05g04630.6	157	-21.15	157	-21.15	1554119	21	2533	72
miR529b	LOC_Os05g05170.1	162	-21.74	162	-21.74	1554174	21	873	565
miR529b	LOC_Os05g05220.1	161	-22.51	161	-22.51	1554181	21	1683	1313
miR529b	LOC_Os05g05300.1	170	-26.33	170	-26.33	1554190	21	3778	3612
miR529b	LOC_Os05g05310.1	170	-26.33	170	-26.33	1554191	21	3863	2237
miR529b	LOC_Os05g05310.2	170	-26.33	170	-26.33	1554192	21	3859	2237
miR529b	LOC_Os05g05420.1	157	-21.69	157	-21.69	1554205	21	4935	531
miR529b	LOC_Os05g05820.1	168	-24.98	168	-24.98	1554256	21	1454	8
miR529b	LOC_Os05g06110.1	168	-24.98	168	-24.98	1554287	21	3260	239
miR529b	LOC_Os05g06710.1	161	-25.59	161	-25.59	1554361	21	1841	1800
miR529b	LOC_Os05g07220.1	169	-22.02	169	-22.02	1554423	21	3840	3723
miR529b	LOC_Os05g07220.2	169	-22.02	169	-22.02	1554424	21	3683	3566
miR529b	LOC_Os05g07280.1	157	-23.38	157	-23.38	1554433	21	2680	2452
miR529b	LOC_Os05g07550.1	161	-25	161	-25	1554456	21	2481	2456
miR529b	LOC_Os05g08110.1	165	-24.08	165	-24.08	1554526	21	940	243
miR529b	LOC_Os05g08370.1	161	-25.23	161	-25.23	1554553	21	4069	1723
miR529b	LOC_Os05g08400.1	154	-20.31	154	-20.31	1554556	21	1788	336
miR529b	LOC_Os05g08970.1	168	-24.98	168	-24.98	1554629	21	2485	43
miR529b	LOC_Os05g09170.1	169	-23.84	169	-23.84	1554653	21	3162	1973
miR529b	LOC_Os05g10840.1	164	-22.08	164	-22.08	1554800	21	2153	21
miR529b	LOC_Os05g10840.2	164	-22.08	164	-22.08	1554801	21	2148	21
miR529b	LOC_Os05g12080.1	162	-22.75	162	-22.75	1554929	21	351	27
miR529b	LOC_Os05g12280.1	170	-23.99	170	-23.99	1554955	21	986	11
miR529b	LOC_Os05g14320.1	161	-21.45	161	-21.45	1555124	21	2950	2067
miR529b	LOC_Os05g14440.1	145	-20.68	145	-20.68	1555136	21	2256	2172
miR529b	LOC_Os05g14930.1	155	-21.04	155	-21.04	1555181	21	2304	2221
miR529b	LOC_Os05g14970.1	155	-21.04	155	-21.04	1555185	21	1784	1441
miR529b	LOC_Os05g16850.1	155	-20.37	155	-20.37	1555385	21	4551	4014
miR529b	LOC_Os05g17130.1	154	-20.29	154	-20.29	1555411	21	1830	14
miR529b	LOC_Os05g17360.1	167	-21.93	167	-21.93	1555434	21	4251	3768
miR529b	LOC_Os05g17420.1	167	-21.93	167	-21.93	1555440	21	3591	3108
miR529b	LOC_Os05g17870.1	161	-24.99	161	-24.99	1555486	21	1248	1223

miR529b	LOC_Os05g18480.1	167	-21.93	167	-21.93	1555547	21	3888	3582
miR529b	LOC_Os05g18560.1	178	-28.3	178	-28.3	1555556	21	2391	1241
miR529b	LOC_Os05g19440.1	153	-20.34	153	-20.34	1555648	21	711	261
miR529b	LOC_Os05g20370.1	161	-20.5	161	-20.5	1555750	21	2280	2255
miR529b	LOC_Os05g22260.1	166	-21.47	166	-21.47	1555846	21	2744	623
miR529b	LOC_Os05g22260.2	166	-21.47	166	-21.47	1555847	21	2848	623
miR529b	LOC_Os05g22730.1	164	-21.23	164	-21.23	1555898	21	731	657
miR529b	LOC_Os05g23840.1	168	-23.18	168	-23.18	1556022	21	2474	52
miR529b	LOC_Os05g23880.1	158	-20.94	158	-20.94	1556026	21	3815	703
miR529b	LOC_Os05g24340.1	162	-23.37	162	-23.37	1556075	21	1236	806
miR529b	LOC_Os05g24680.1	171	-22.79	171	-22.79	1556115	21	5664	4248
miR529b	LOC_Os05g25100.1	167	-23.13	167	-23.13	1556160	21	3372	2571
miR529b	LOC_Os05g26210.1	150	-24.35	150	-24.35	1556280	21	714	616
miR529b	LOC_Os05g27090.1	162	-20.9	162	-20.9	1556365	21	663	558
miR529b	LOC_Os05g28120.1	156	-21.71	156	-21.71	1556474	21	5565	3861
miR529b	LOC_Os05g28980.1	162	-20.93	162	-20.93	1556566	21	1419	243
miR529b	LOC_Os05g28980.2	162	-20.93	162	-20.93	1556567	21	1981	243
miR529b	LOC_Os05g28980.6	162	-20.93	162	-20.93	1556569	21	2119	243
miR529b	LOC_Os05g29090.1	164	-23.33	164	-23.33	1556583	21	1993	647
miR529b	LOC_Os05g29160.1	155	-20.72	155	-20.72	1556590	21	552	398
miR529b	LOC_Os05g31530.1	165	-24.01	165	-24.01	1556811	21	5545	1439
miR529b	LOC_Os05g32110.1	149	-20.22	149	-20.22	1556868	21	2167	329
miR529b	LOC_Os05g32110.2	149	-20.22	149	-20.22	1556869	21	2089	329
miR529b	LOC_Os05g32110.3	149	-20.22	149	-20.22	1556870	21	1846	329
miR529b	LOC_Os05g32110.4	149	-20.22	149	-20.22	1556871	21	2287	329
miR529b	LOC_Os05g32200.1	157	-20.09	157	-20.09	1556880	21	846	26
miR529b	LOC_Os05g32700.1	156	-21.36	156	-21.36	1556938	21	2751	245
miR529b	LOC_Os05g33370.1	156	-21.71	156	-21.71	1557012	21	4437	3078
miR529b	LOC_Os05g33820.1	166	-23.46	166	-23.46	1557068	21	2059	1651
miR529b	LOC_Os05g34610.1	173	-24.66	173	-24.66	1557157	21	930	718
miR529b	LOC_Os05g34830.1	159	-20.81	159	-20.81	1557184	21	1523	24
miR529b	LOC_Os05g34830.2	159	-20.81	159	-20.81	1557185	21	1491	15

miR529b	LOC_Os05g34830.3	159	-20.81	159	-20.81	1557186	21	1592	15
miR529b	LOC_Os05g35200.2	168	-24.98	168	-24.98	1557229	21	2628	2257
miR529b	LOC_Os05g36290.1	161	-20.92	161	-20.92	1557359	21	1883	353
miR529b	LOC_Os05g37230.1	155	-20.37	155	-20.37	1557421	21	4110	3573
miR529b	LOC_Os05g37380.1	154	-20.23	154	-20.23	1557435	21	5979	4558
miR529b	LOC_Os05g37460.1	158	-20.47	158	-20.47	1557444	21	1040	340
miR529b	LOC_Os05g37830.1	160	-21.41	160	-21.41	1557485	21	969	285
miR529b	LOC_Os05g37830.2	160	-21.41	160	-21.41	1557486	21	985	285
miR529b	LOC_Os05g38260.1	148	-21.19	148	-21.19	1557543	21	3978	2006
miR529b	LOC_Os05g38660.1	173	-26.24	173	-26.24	1557602	21	669	494
miR529b	LOC_Os05g38730.1	171	-21.2	171	-21.2	1557611	21	3489	1918
miR529b	LOC_Os05g38730.2	171	-21.2	171	-21.2	1557612	21	3529	2000
miR529b	LOC_Os05g38870.1	163	-21.42	163	-21.42	1557628	21	312	78
miR529b	LOC_Os05g40280.1	160	-22.3	160	-22.3	1557798	21	1852	1797
miR529b	LOC_Os05g40285.1	333	-49.56	168	-24.98	1557799	21	944	345 193
miR529b	LOC_Os05g40410.1	165	-22.78	165	-22.78	1557814	21	1937	51
miR529b	LOC_Os05g40680.1	166	-20.05	166	-20.05	1557843	21	1365	1334
miR529b	LOC_Os05g40720.1	154	-20.21	154	-20.21	1557846	21	2004	1321
miR529b	LOC_Os05g40820.1	149	-20.66	149	-20.66	1557861	21	990	549
miR529b	LOC_Os05g40820.2	149	-20.66	149	-20.66	1557862	21	783	345
miR529b	LOC_Os05g40820.3	149	-20.66	149	-20.66	1557863	21	982	549
miR529b	LOC_Os05g41160.1	158	-21.17	158	-21.17	1557900	21	969	688
miR529b	LOC_Os05g41560.1	156	-21.71	156	-21.71	1557949	21	4062	2358
miR529b	LOC_Os05g41770.1	174	-26.46	174	-26.46	1557971	21	2208	631
miR529b	LOC_Os05g41910.1	162	-22.76	162	-22.76	1557990	21	2034	940
miR529b	LOC_Os05g42300.1	149	-22.81	149	-22.81	1558032	21	3027	1393
miR529b	LOC_Os05g43020.1	161	-25	161	-25	1558060	21	2481	2456
miR529b	LOC_Os05g43040.1	168	-22.91	168	-22.91	1558062	21	1313	38
miR529b	LOC_Os05g43210.1	157	-22.05	157	-22.05	1558077	21	489	455
miR529b	LOC_Os05g43610.1	170	-23.43	170	-23.43	1558126	21	3212	2120
miR529b	LOC_Os05g43910.1	158	-20.82	158	-20.82	1558161	21	2396	83
miR529b	LOC_Os05g44340.1	159	-22.02	159	-22.02	1558219	21	3264	3190

miR529b	LOC_Os05g44810.1	157	-22.95	157	-22.95	1558277	21	1396	1016
miR529b	LOC_Os05g44810.2	157	-22.95	157	-22.95	1558278	21	1353	1016
miR529b	LOC_Os05g44810.3	157	-22.95	157	-22.95	1558279	21	1333	1016
miR529b	LOC_Os05g45550.1	163	-22.18	163	-22.18	1558363	21	3390	2613
miR529b	LOC_Os05g45630.1	157	-21.69	157	-21.69	1558371	21	4575	531
miR529b	LOC_Os05g45850.1	156	-20.44	156	-20.44	1558400	21	5614	233
miR529b	LOC_Os05g46220.1	149	-20.32	149	-20.32	1558444	21	2175	437
miR529b	LOC_Os05g46230.1	149	-20.32	149	-20.32	1558445	21	2753	40
miR529b	LOC_Os05g46510.1	145	-20.34	145	-20.34	1558488	21	2075	442
miR529b	LOC_Os05g46950.1	162	-21.2	162	-21.2	1558537	21	1132	373
miR529b	LOC_Os05g48030.1	156	-20.74	156	-20.74	1558607	21	3047	239
miR529b	LOC_Os05g48170.1	150	-21.79	150	-21.79	1558626	21	3300	3234
miR529b	LOC_Os05g49520.1	170	-26.69	170	-26.69	1558813	21	1824	680
miR529b	LOC_Os05g50310.1	168	-22.71	168	-22.71	1558912	21	1096	998
miR529b	LOC_Os05g50390.1	156	-20.83	156	-20.83	1558921	21	880	20
miR529b	LOC_Os05g51710.1	161	-21.04	161	-21.04	1559101	21	1295	782
miR529b	LOC_Os05g51770.1	157	-21.69	157	-21.69	1559109	21	3762	531
miR529b	LOC_Os05g52070.1	155	-20.37	155	-20.37	1559136	21	5016	3702
miR529b	LOC_Os06g02110.1	151	-21.71	151	-21.71	1559261	21	1705	1317
miR529b	LOC_Os06g02130.1	164	-21.42	164	-21.42	1559265	21	3611	2459
miR529b	LOC_Os06g02130.2	164	-21.42	164	-21.42	1559266	21	3929	2777
miR529b	LOC_Os06g02450.1	164	-20.48	164	-20.48	1559304	21	608	214
miR529b	LOC_Os06g02560.1	160	-20.72	160	-20.72	1559322	21	1703	189
miR529b	LOC_Os06g02560.2	160	-20.72	160	-20.72	1559323	21	1540	101
miR529b	LOC_Os06g02560.3	160	-20.72	160	-20.72	1559324	21	1703	259
miR529b	LOC_Os06g02630.1	156	-21.58	156	-21.58	1559331	21	1877	1631
miR529b	LOC_Os06g02830.1	156	-20.16	156	-20.16	1559351	21	2741	2567
miR529b	LOC_Os06g03000.1	155	-20.44	155	-20.44	1559366	21	1386	542
miR529b	LOC_Os06g03690.1	168	-24.51	168	-24.51	1559443	21	1226	76
miR529b	LOC_Os06g03690.2	168	-24.51	168	-24.51	1559444	21	1431	61
miR529b	LOC_Os06g03710.1	161	-20.2	161	-20.2	1559446	21	3102	419
miR529b	LOC_Os06g03850.1	170	-22.75	170	-22.75	1559463	21	1360	1324

miR529b	LOC_Os06g03970.1	165	-27.36	165	-27.36	1559477	21	3796	16
miR529b	LOC_Os06g04510.1	165	-24.08	165	-24.08	1559543	21	1689	1619
miR529b	LOC_Os06g04510.2	165	-24.08	165	-24.08	1559544	21	2415	2353
miR529b	LOC_Os06g04690.1	159	-21.82	159	-21.82	1559569	21	2006	547
miR529b	LOC_Os06g04710.1	159	-21.82	159	-21.82	1559571	21	2049	635
miR529b	LOC_Os06g05550.1	162	-20.34	162	-20.34	1559672	21	1571	95
miR529b	LOC_Os06g05570.1	157	-22.05	157	-22.05	1559674	21	444	416
miR529b	LOC_Os06g05660.1	166	-21.46	166	-21.46	1559686	21	1590	38
miR529b	LOC_Os06g05660.2	166	-21.46	166	-21.46	1559687	21	1576	36
miR529b	LOC_Os06g05660.3	166	-21.46	166	-21.46	1559688	21	1575	38
miR529b	LOC_Os06g05660.4	166	-21.46	166	-21.46	1559689	21	1549	36
miR529b	LOC_Os06g05660.5	166	-21.46	166	-21.46	1559690	21	1654	36
miR529b	LOC_Os06g05860.1	166	-22.02	166	-22.02	1559717	21	2043	437
miR529b	LOC_Os06g06160.1	164	-23.12	164	-23.12	1559762	21	1612	26
miR529b	LOC_Os06g06160.4	164	-23.12	164	-23.12	1559765	21	1551	13
miR529b	LOC_Os06g07070.1	168	-24.05	168	-24.05	1559870	21	1651	19
miR529b	LOC_Os06g07520.1	157	-21.69	157	-21.69	1559919	21	4575	531
miR529b	LOC_Os06g07760.1	167	-20.33	167	-20.33	1559944	21	749	12
miR529b	LOC_Os06g08060.1	158	-26.3	158	-26.3	1559976	21	1302	864
miR529b	LOC_Os06g08550.1	164	-20.48	164	-20.48	1560028	21	2365	2246
miR529b	LOC_Os06g08660.1	169	-23.06	169	-23.06	1560040	21	3557	1379
miR529b	LOC_Os06g09320.1	158	-20.21	158	-20.21	1560125	21	1869	961
miR529b	LOC_Os06g09320.2	158	-20.21	158	-20.21	1560126	21	2040	1132
miR529b	LOC_Os06g09380.1	165	-23.53	165	-23.53	1560134	21	480	323
miR529b	LOC_Os06g10355.1	178	-27.77	178	-27.77	1560248	21	1534	628
miR529b	LOC_Os06g10880.1	156	-20.23	156	-20.23	1560302	21	1718	131
miR529b	LOC_Os06g10880.2	156	-20.23	156	-20.23	1560303	21	1803	131
miR529b	LOC_Os06g10880.3	156	-20.23	156	-20.23	1560304	21	1400	131
miR529b	LOC_Os06g10990.1	173	-23.61	173	-23.61	1560316	21	1723	1159
miR529b	LOC_Os06g11010.1	161	-21.26	161	-21.26	1560317	21	1419	897
miR529b	LOC_Os06g11320.1	169	-21.69	169	-21.69	1560352	21	1833	705
miR529b	LOC_Os06g11320.2	169	-21.69	169	-21.69	1560353	21	1776	788

miR529b	LOC_Os06g11440.1	156	-20.23	156	-20.23	1560366	21	4515	2152
miR529b	LOC_Os06g11440.2	322	-41.3	166	-21.07	1560367	21	3962	922 1599
miR529b	LOC_Os06g11440.3	156	-20.23	156	-20.23	1560368	21	2702	927
miR529b	LOC_Os06g11830.1	167	-21.97	167	-21.97	1560410	21	2640	2318
miR529b	LOC_Os06g12310.1	157	-23.82	157	-23.82	1560472	21	1544	243
miR529b	LOC_Os06g13540.1	152	-20.96	152	-20.96	1560607	21	4353	2261
miR529b	LOC_Os06g13610.1	487	-70.21	168	-24.51	1560618	21	732	55 27 5
miR529b	LOC_Os06g13630.1	154	-21.82	154	-21.82	1560620	21	2841	231
miR529b	LOC_Os06g13820.1	165	-24.08	165	-24.08	1560642	21	3391	21
miR529b	LOC_Os06g13820.2	165	-24.08	165	-24.08	1560643	21	3313	21
miR529b	LOC_Os06g13940.1	156	-24.16	156	-24.16	1560655	21	807	719
miR529b	LOC_Os06g14600.1	157	-22.3	157	-22.3	1560733	21	2502	2477
miR529b	LOC_Os06g14690.1	157	-22.05	157	-22.05	1560742	21	570	352
miR529b	LOC_Os06g16080.1	169	-23.84	169	-23.84	1560879	21	2706	1715
miR529b	LOC_Os06g16850.1	154	-20.29	154	-20.29	1560959	21	1863	14
miR529b	LOC_Os06g17170.1	154	-20.29	154	-20.29	1560993	21	450	14
miR529b	LOC_Os06g17180.1	154	-20.29	154	-20.29	1560994	21	375	14
miR529b	LOC_Os06g17280.1	164	-20.21	164	-20.21	1561004	21	2602	2519
miR529b	LOC_Os06g17510.1	152	-20.23	152	-20.23	1561027	21	546	71
miR529b	LOC_Os06g17770.1	154	-20.29	154	-20.29	1561052	21	375	14
miR529b	LOC_Os06g20860.1	160	-21.14	160	-21.14	1561318	21	2746	425
miR529b	LOC_Os06g21610.1	156	-21.71	156	-21.71	1561400	21	4341	2829
miR529b	LOC_Os06g23080.1	161	-20.5	161	-20.5	1561564	21	1998	1973
miR529b	LOC_Os06g23160.1	163	-20.13	163	-20.13	1561571	21	1872	1091
miR529b	LOC_Os06g23730.1	149	-22.94	149	-22.94	1561630	21	1995	704
miR529b	LOC_Os06g23890.1	153	-22.37	153	-22.37	1561649	21	2592	1949
miR529b	LOC_Os06g25690.1	153	-20.34	153	-20.34	1561825	21	891	852
miR529b	LOC_Os06g26030.1	175	-23.41	175	-23.41	1561859	21	1557	426
miR529b	LOC_Os06g26320.1	152	-20.75	152	-20.75	1561886	21	718	678
miR529b	LOC_Os06g27430.1	167	-25.04	167	-25.04	1561899	21	456	134
miR529b	LOC_Os06g28850.1	157	-20.97	157	-20.97	1562048	21	1896	379
miR529b	LOC_Os06g28990.1	156	-20.23	156	-20.23	1562064	21	4106	891

miR529b	LOC_Os06g30520.1	156	-24.57	156	-24.57	1562223	21	3678	870
miR529b	LOC_Os06g30800.1	164	-20.21	164	-20.21	1562254	21	441	114
miR529b	LOC_Os06g30894.1	148	-20.3	148	-20.3	1562266	21	4124	936
miR529b	LOC_Os06g30894.2	148	-20.3	148	-20.3	1562267	21	4051	936
miR529b	LOC_Os06g30930.1	158	-24.5	158	-24.5	1562271	21	1374	280
miR529b	LOC_Os06g31650.1	157	-22.29	157	-22.29	1562340	21	648	623
miR529b	LOC_Os06g31660.1	165	-22.01	165	-22.01	1562341	21	843	779
miR529b	LOC_Os06g32270.1	178	-28.3	178	-28.3	1562399	21	2661	1517
miR529b	LOC_Os06g32860.1	162	-23.99	162	-23.99	1562454	21	2534	7
miR529b	LOC_Os06g32880.1	161	-25	161	-25	1562457	21	2436	2411
miR529b	LOC_Os06g33900.1	161	-25	161	-25	1562558	21	2436	2411
miR529b	LOC_Os06g35050.1	154	-20.09	154	-20.09	1562672	21	1695	1242
miR529b	LOC_Os06g35140.1	161	-22.38	161	-22.38	1562681	21	2094	1936
miR529b	LOC_Os06g35920.1	163	-20.9	163	-20.9	1562760	21	693	5
miR529b	LOC_Os06g36500.1	156	-20.87	156	-20.87	1562824	21	3756	78
miR529b	LOC_Os06g36660.1	156	-20.76	156	-20.76	1562840	21	1083	571
miR529b	LOC_Os06g36760.1	158	-20.12	158	-20.12	1562850	21	483	29
miR529b	LOC_Os06g37560.1	170	-24.41	170	-24.41	1562935	21	3071	40
miR529b	LOC_Os06g37810.1	158	-22.84	158	-22.84	1562961	21	657	49
miR529b	LOC_Os06g37890.1	156	-21.71	156	-21.71	1562969	21	3024	2562
miR529b	LOC_Os06g39515.1	168	-23.18	168	-23.18	1563133	21	656	4
miR529b	LOC_Os06g39900.1	166	-22.72	166	-22.72	1563186	21	1683	1642
miR529b	LOC_Os06g39900.2	166	-22.72	166	-22.72	1563187	21	2808	2767
miR529b	LOC_Os06g39900.3	166	-22.72	166	-22.72	1563188	21	2709	2668
miR529b	LOC_Os06g40680.1	152	-21.64	152	-21.64	1563279	21	3486	1571
miR529b	LOC_Os06g41540.1	168	-22.91	168	-22.91	1563373	21	685	34
miR529b	LOC_Os06g41570.1	158	-20.42	158	-20.42	1563376	21	483	154
miR529b	LOC_Os06g42030.1	319	-44.72	170	-23.94	1563434	21	5864	2996 2797
miR529b	LOC_Os06g42400.1	156	-21.71	156	-21.71	1563474	21	4395	3096
miR529b	LOC_Os06g42950.1	154	-20.29	154	-20.29	1563538	21	375	14
miR529b	LOC_Os06g43330.1	166	-21.94	166	-21.94	1563576	21	2106	123
miR529b	LOC_Os06g43560.1	161	-23.39	161	-23.39	1563600	21	2963	2269

miR529b	LOC_Os06g43600.2	155	-21.55	155	-21.55	1563607	21	1657	922
miR529b	LOC_Os06g43620.1	165	-24.08	165	-24.08	1563609	21	1689	49
miR529b	LOC_Os06g43690.1	169	-24.83	169	-24.83	1563620	21	3134	2834
miR529b	LOC_Os06g43770.1	160	-22.13	160	-22.13	1563628	21	963	887
miR529b	LOC_Os06g44030.1	159	-20.31	159	-20.31	1563659	21	2509	1769
miR529b	LOC_Os06g44030.2	159	-20.31	159	-20.31	1563660	21	2473	1769
miR529b	LOC_Os06g44034.1	175	-26.46	175	-26.46	1563661	21	2020	639
miR529b	LOC_Os06g44230.1	162	-20.46	162	-20.46	1563686	21	3746	37
miR529b	LOC_Os06g44880.1	167	-20.8	167	-20.8	1563753	21	2646	1163
miR529b	LOC_Os06g45030.1	156	-23.02	156	-23.02	1563771	21	3229	3162
miR529b	LOC_Os06g45090.1	153	-25.04	153	-25.04	1563779	21	1148	254
miR529b	LOC_Os06g45090.2	153	-25.04	153	-25.04	1563780	21	2200	185
miR529b	LOC_Os06g45100.1	157	-21.6	157	-21.6	1563781	21	1923	193
miR529b	LOC_Os06g45350.1	165	-25.29	165	-25.29	1563817	21	2140	67
miR529b	LOC_Os06g45530.1	157	-22.29	157	-22.29	1563841	21	2412	2387
miR529b	LOC_Os06g45580.1	154	-20.79	154	-20.79	1563846	21	975	93
miR529b	LOC_Os06g45630.1	144	-21.28	144	-21.28	1563851	21	3811	1542
miR529b	LOC_Os06g45860.1	156	-20.19	156	-20.19	1563882	21	3194	2172
miR529b	LOC_Os06g45900.1	165	-20.15	165	-20.15	1563886	21	1647	233
miR529b	LOC_Os06g46140.1	170	-26.96	170	-26.96	1563914	21	1534	507
miR529b	LOC_Os06g46270.1	333	-44.62	168	-23.79	1563924	21	1470	114 25
miR529b	LOC_Os06g46550.1	164	-20.48	164	-20.48	1563960	21	627	46
miR529b	LOC_Os06g46560.1	154	-20.5	154	-20.5	1563961	21	1934	51
miR529b	LOC_Os06g47294.1	155	-21.12	155	-21.12	1564057	21	2283	1030
miR529b	LOC_Os06g47590.1	158	-20.93	158	-20.93	1564096	21	1019	802
miR529b	LOC_Os06g47840.1	165	-20.36	165	-20.36	1564121	21	1684	25
miR529b	LOC_Os06g47900.1	163	-22.72	163	-22.72	1564129	21	1456	987
miR529b	LOC_Os06g47900.3	163	-22.72	163	-22.72	1564131	21	1649	934
miR529b	LOC_Os06g47900.4	163	-22.72	163	-22.72	1564132	21	1539	934
miR529b	LOC_Os06g48030.1	156	-21.57	156	-21.57	1564145	21	2170	1467
miR529b	LOC_Os06g48030.3	156	-21.57	156	-21.57	1564146	21	2644	1941
miR529b	LOC_Os06g48040.1	168	-24.51	168	-24.51	1564147	21	2664	2618

miR529b	LOC_Os06g48590.1	168	-22.71	168	-22.71	1564211	21	1875	152
miR529b	LOC_Os06g49060.1	161	-21.33	161	-21.33	1564274	21	2143	89
miR529b	LOC_Os06g49150.1	156	-20.46	156	-20.46	1564288	21	1543	73
miR529b	LOC_Os06g49310.1	152	-21.12	152	-21.12	1564309	21	3960	2279
miR529b	LOC_Os06g49320.1	152	-21.12	152	-21.12	1564311	21	3711	420
miR529b	LOC_Os06g49510.1	157	-22.52	157	-22.52	1564341	21	2263	1872
miR529b	LOC_Os06g49620.1	169	-26.47	169	-26.47	1564353	21	390	159
miR529b	LOC_Os06g50030.1	168	-23.03	168	-23.03	1564410	21	2327	2093
miR529b	LOC_Os06g50170.1	168	-24.98	168	-24.98	1564425	21	1798	1745
miR529b	LOC_Os06g50210.1	169	-23.27	169	-23.27	1564429	21	1749	389
miR529b	LOC_Os06g50400.1	174	-24.21	174	-24.21	1564452	21	1106	921
miR529b	LOC_Os06g50880.1	169	-23.27	169	-23.27	1564503	21	1800	267
miR529b	LOC_Os06g50930.1	161	-20.91	161	-20.91	1564510	21	1287	914
miR529b	LOC_Os06g50930.2	161	-20.91	161	-20.91	1564511	21	1447	1074
miR529b	LOC_Os06g51310.1	159	-24.07	159	-24.07	1564564	21	3759	70
miR529b	LOC_Os06g51480.2	168	-22.71	168	-22.71	1564592	21	1697	400
miR529b	LOC_Os06g51510.1	164	-23.27	164	-23.27	1564595	21	1372	680
miR529b	LOC_Os06g51510.2	164	-23.27	164	-23.27	1564596	21	1478	680
miR529b	LOC_Os07g01870.1	155	-20.29	155	-20.29	1564710	21	1298	673
miR529b	LOC_Os07g01870.2	155	-20.29	155	-20.29	1564711	21	1282	673
miR529b	LOC_Os07g02470.1	148	-21.14	148	-21.14	1564774	21	1410	592
miR529b	LOC_Os07g03230.2	165	-21.81	165	-21.81	1564865	21	3672	137
miR529b	LOC_Os07g03820.1	156	-20.85	156	-20.85	1564918	21	2255	306
miR529b	LOC_Os07g04090.1	161	-23.5	161	-23.5	1564944	21	1398	649
miR529b	LOC_Os07g04480.1	153	-20.31	153	-20.31	1564985	21	3346	2118
miR529b	LOC_Os07g04530.1	158	-22.24	158	-22.24	1564990	21	5617	4107
miR529b	LOC_Os07g04820.1	169	-25.08	169	-25.08	1565020	21	1611	199
miR529b	LOC_Os07g05140.1	156	-21.71	156	-21.71	1565056	21	3708	2004
miR529b	LOC_Os07g05540.1	145	-20.29	145	-20.29	1565100	21	5325	4532
miR529b	LOC_Os07g05560.1	169	-23.98	169	-23.98	1565102	21	1779	219
miR529b	LOC_Os07g05720.1	156	-20.16	156	-20.16	1565123	21	2109	31
miR529b	LOC_Os07g06130.1	166	-21.51	166	-21.51	1565164	21	4826	4536

miR529b	LOC_Os07g06130.2	166	-21.51	166	-21.51	1565165	21	4831	4541
miR529b	LOC_Os07g06130.3	166	-21.51	166	-21.51	1565166	21	4791	4501
miR529b	LOC_Os07g06166.1	162	-20.27	162	-20.27	1565167	21	522	65
miR529b	LOC_Os07g06250.1	162	-20.27	162	-20.27	1565175	21	522	65
miR529b	LOC_Os07g06300.1	166	-21.51	166	-21.51	1565177	21	4539	4474
miR529b	LOC_Os07g06345.1	162	-20.27	162	-20.27	1565181	21	522	65
miR529b	LOC_Os07g06620.1	165	-24.22	165	-24.22	1565214	21	1380	938
miR529b	LOC_Os07g06620.2	165	-24.22	165	-24.22	1565215	21	1345	903
miR529b	LOC_Os07g06620.4	165	-24.22	165	-24.22	1565217	21	1374	932
miR529b	LOC_Os07g07010.1	167	-22.74	167	-22.74	1565263	21	1974	1560
miR529b	LOC_Os07g07350.1	173	-24.45	173	-24.45	1565301	21	908	825
miR529b	LOC_Os07g07350.3	173	-24.45	173	-24.45	1565303	21	914	829
miR529b	LOC_Os07g07950.1	165	-24.08	165	-24.08	1565369	21	2916	2768
miR529b	LOC_Os07g07950.2	165	-24.08	165	-24.08	1565370	21	2968	2820
miR529b	LOC_Os07g09340.1	170	-28.27	170	-28.27	1565519	21	3434	114
miR529b	LOC_Os07g10030.1	160	-20.09	160	-20.09	1565591	21	1738	1688
miR529b	LOC_Os07g10560.1	157	-21.21	157	-21.21	1565651	21	1110	27
miR529b	LOC_Os07g11010.2	157	-23.14	157	-23.14	1565711	21	2383	7
miR529b	LOC_Os07g11110.1	143	-20.53	143	-20.53	1565722	21	1562	1285
miR529b	LOC_Os07g11190.1	157	-22.72	157	-22.72	1565731	21	2193	715
miR529b	LOC_Os07g11640.1	167	-21.93	167	-21.93	1565778	21	2400	1917
miR529b	LOC_Os07g11680.1	160	-20.98	160	-20.98	1565781	21	5976	3984
miR529b	LOC_Os07g11790.1	165	-23.44	165	-23.44	1565789	21	2016	1909
miR529b	LOC_Os07g12490.1	156	-21.23	156	-21.23	1565859	21	1480	46
miR529b	LOC_Os07g12610.1	156	-20.66	156	-20.66	1565875	21	1996	96
miR529b	LOC_Os07g13700.1	166	-21.94	166	-21.94	1565983	21	2241	204
miR529b	LOC_Os07g14150.5	159	-22.12	159	-22.12	1566031	21	2180	120
miR529b	LOC_Os07g14580.1	168	-23.18	168	-23.18	1566087	21	1671	34
miR529b	LOC_Os07g14920.1	166	-21.13	166	-21.13	1566121	21	2602	1848
miR529b	LOC_Os07g15340.1	167	-21.97	167	-21.97	1566163	21	2694	2318
miR529b	LOC_Os07g16420.1	155	-20.37	155	-20.37	1566265	21	2346	142
miR529b	LOC_Os07g16490.1	157	-22.29	157	-22.29	1566272	21	2331	2306

miR529b	LOC_Os07g16800.1	155	-20.37	155	-20.37	1566303	21	2265	142
miR529b	LOC_Os07g17120.1	169	-21.83	169	-21.83	1566338	21	954	644
miR529b	LOC_Os07g17810.1	167	-23.13	167	-23.13	1566411	21	2151	1170
miR529b	LOC_Os07g18120.1	159	-20.5	159	-20.5	1566443	21	4560	1754
miR529b	LOC_Os07g18154.1	159	-20.5	159	-20.5	1566446	21	2953	1770
miR529b	LOC_Os07g18154.2	159	-20.5	159	-20.5	1566447	21	3082	1914
miR529b	LOC_Os07g19520.1	168	-24.26	168	-24.26	1566572	21	495	30
miR529b	LOC_Os07g20340.1	155	-20.98	155	-20.98	1566606	21	1386	339
miR529b	LOC_Os07g20420.1	169	-24.02	169	-24.02	1566613	21	2401	508
miR529b	LOC_Os07g20430.1	156	-21.71	156	-21.71	1566614	21	5045	3213
miR529b	LOC_Os07g20700.1	163	-21.02	163	-21.02	1566642	21	231	146
miR529b	LOC_Os07g21980.1	149	-20.36	149	-20.36	1566670	21	1605	178
miR529b	LOC_Os07g22430.1	145	-20.29	145	-20.29	1566715	21	5103	4388
miR529b	LOC_Os07g22600.1	158	-20.03	158	-20.03	1566739	21	1558	1499
miR529b	LOC_Os07g22600.2	158	-20.03	158	-20.03	1566740	21	1546	1487
miR529b	LOC_Os07g22860.1	158	-20.32	158	-20.32	1566773	21	2865	1788
miR529b	LOC_Os07g22950.1	163	-24.82	163	-24.82	1566784	21	978	601
miR529b	LOC_Os07g22950.2	163	-24.82	163	-24.82	1566785	21	918	541
miR529b	LOC_Os07g22950.3	163	-24.82	163	-24.82	1566786	21	944	567
miR529b	LOC_Os07g23130.1	157	-20.24	157	-20.24	1566806	21	366	215
miR529b	LOC_Os07g23550.1	168	-23.18	168	-23.18	1566845	21	1220	100
miR529b	LOC_Os07g23550.2	168	-23.18	168	-23.18	1566846	21	1111	100
miR529b	LOC_Os07g23570.1	155	-20.34	155	-20.34	1566848	21	2009	1693
miR529b	LOC_Os07g23770.1	168	-22.71	168	-22.71	1566868	21	630	193
miR529b	LOC_Os07g26210.1	167	-20.52	167	-20.52	1567123	21	4649	4474
miR529b	LOC_Os07g26410.1	162	-24.3	162	-24.3	1567144	21	495	151
miR529b	LOC_Os07g27680.1	162	-20.9	162	-20.9	1567282	21	225	69
miR529b	LOC_Os07g29300.1	156	-20.37	156	-20.37	1567448	21	747	41
miR529b	LOC_Os07g29410.1	160	-21.95	160	-21.95	1567465	21	1449	101
miR529b	LOC_Os07g29410.2	160	-21.95	160	-21.95	1567466	21	1357	101
miR529b	LOC_Os07g29600.2	159	-20.97	159	-20.97	1567489	21	1432	544
miR529b	LOC_Os07g29630.1	166	-24.88	166	-24.88	1567492	21	1006	215

miR529b	LOC_Os07g32430.1	161	-24.76	161	-24.76	1567790	21	3954	67
miR529b	LOC_Os07g32510.1	165	-20.96	165	-20.96	1567796	21	2583	2464
miR529b	LOC_Os07g35310.1	154	-20.51	154	-20.51	1568096	21	2562	1280
miR529b	LOC_Os07g35310.2	154	-20.51	154	-20.51	1568097	21	2663	1381
miR529b	LOC_Os07g35550.1	157	-22.05	157	-22.05	1568127	21	417	338
miR529b	LOC_Os07g35740.1	164	-23.32	164	-23.32	1568153	21	2189	818
miR529b	LOC_Os07g36410.1	168	-23.18	168	-23.18	1568230	21	1623	1007
miR529b	LOC_Os07g36465.1	170	-23.87	170	-23.87	1568235	21	616	18
miR529b	LOC_Os07g37250.1	159	-21.19	159	-21.19	1568322	21	1347	412
miR529b	LOC_Os07g37900.1	161	-21.18	161	-21.18	1568394	21	6156	5660
miR529b	LOC_Os07g38610.1	174	-25.04	174	-25.04	1568475	21	1828	1620
miR529b	LOC_Os07g38760.1	330	-47.7	165	-27.35	1568491	21	3693	966 1599
miR529b	LOC_Os07g39090.1	165	-25.78	165	-25.78	1568530	21	3036	235
miR529b	LOC_Os07g40520.1	152	-21.42	152	-21.42	1568691	21	1914	580
miR529b	LOC_Os07g40680.1	166	-21.94	166	-21.94	1568709	21	936	204
miR529b	LOC_Os07g40940.1	156	-22.53	156	-22.53	1568740	21	1563	135
miR529b	LOC_Os07g40974.1	168	-24.98	168	-24.98	1568742	21	1237	1176
miR529b	LOC_Os07g40974.2	168	-24.98	168	-24.98	1568743	21	1478	1417
miR529b	LOC_Os07g41070.1	158	-21.04	158	-21.04	1568751	21	1676	1595
miR529b	LOC_Os07g42730.1	158	-20.18	158	-20.18	1568949	21	1207	1105
miR529b	LOC_Os07g43070.1	163	-20.13	163	-20.13	1568998	21	1432	1360
miR529b	LOC_Os07g43510.1	171	-22.8	171	-22.8	1569047	21	993	379
miR529b	LOC_Os07g43910.1	174	-25.06	174	-25.06	1569087	21	363	53
miR529b	LOC_Os07g44180.1	165	-21.65	165	-21.65	1569119	21	941	18
miR529b	LOC_Os07g45064.1	157	-20.21	157	-20.21	1569217	21	3757	3726
miR529b	LOC_Os07g45090.1	165	-20.83	165	-20.83	1569221	21	2424	72
miR529b	LOC_Os07g45660.1	157	-22.3	157	-22.3	1569276	21	2481	2456
miR529b	LOC_Os07g45710.1	157	-22.29	157	-22.29	1569281	21	2346	2321
miR529b	LOC_Os07g46920.1	156	-20.37	156	-20.37	1569433	21	1246	1194
miR529b	LOC_Os07g47100.1	158	-21	158	-21	1569460	21	2265	136
miR529b	LOC_Os07g47100.2	158	-21	158	-21	1569461	21	2394	265
miR529b	LOC_Os07g47100.3	158	-21	158	-21	1569462	21	1820	126

miR529b	LOC_Os07g47230.1	160	-20.26	160	-20.26	1569480	21	2022	888
miR529b	LOC_Os07g47800.1	159	-21.27	159	-21.27	1569566	21	2085	681
miR529b	LOC_Os07g48080.1	165	-22.06	165	-22.06	1569597	21	1966	18
miR529b	LOC_Os07g48410.1	162	-23.19	162	-23.19	1569637	21	3468	678
miR529b	LOC_Os07g48410.2	162	-23.19	162	-23.19	1569638	21	3623	678
miR529b	LOC_Os07g49500.1	161	-22	161	-22	1569787	21	4545	3471
miR529b	LOC_Os08g01740.1	165	-26.33	165	-26.33	1569887	21	2297	16
miR529b	LOC_Os08g01740.2	165	-26.33	165	-26.33	1569888	21	2225	16
miR529b	LOC_Os08g01750.3	161	-22.01	161	-22.01	1569891	21	2026	1960
miR529b	LOC_Os08g01910.1	152	-23	152	-23	1569909	21	998	406
miR529b	LOC_Os08g02570.1	144	-21.45	144	-21.45	1569980	21	1179	39
miR529b	LOC_Os08g03360.1	159	-21.76	159	-21.76	1570073	21	1949	885
miR529b	LOC_Os08g04440.1	162	-21.25	162	-21.25	1570200	21	1080	1056
miR529b	LOC_Os08g04920.1	178	-27.88	178	-27.88	1570255	21	2389	2084
miR529b	LOC_Os08g04920.2	178	-27.88	178	-27.88	1570256	21	2265	1960
miR529b	LOC_Os08g04920.3	178	-27.88	178	-27.88	1570257	21	1856	1551
miR529b	LOC_Os08g04920.4	178	-27.88	178	-27.88	1570258	21	2201	1896
miR529b	LOC_Os08g04920.5	178	-27.88	178	-27.88	1570259	21	2314	2009
miR529b	LOC_Os08g04920.6	178	-27.88	178	-27.88	1570260	21	2174	1869
miR529b	LOC_Os08g04920.7	178	-27.88	178	-27.88	1570261	21	2211	1906
miR529b	LOC_Os08g04920.8	178	-27.88	178	-27.88	1570262	21	2337	2032
miR529b	LOC_Os08g05130.1	157	-22.29	157	-22.29	1570284	21	2484	2459
miR529b	LOC_Os08g05440.1	166	-25.57	166	-25.57	1570314	21	4497	1133
miR529b	LOC_Os08g05910.1	166	-25.26	166	-25.26	1570365	21	2356	2254
miR529b	LOC_Os08g06010.1	165	-20.36	165	-20.36	1570376	21	2316	77
miR529b	LOC_Os08g06440.1	179	-26.82	179	-26.82	1570433	21	732	377
miR529b	LOC_Os08g06460.1	162	-20.81	162	-20.81	1570435	21	1692	1468
miR529b	LOC_Os08g06460.2	162	-20.81	162	-20.81	1570436	21	1696	1475
miR529b	LOC_Os08g06470.1	156	-22.3	156	-22.3	1570437	21	2496	1546
miR529b	LOC_Os08g06470.2	156	-22.3	156	-22.3	1570438	21	2902	1950
miR529b	LOC_Os08g06600.1	169	-23.26	169	-23.26	1570455	21	1750	896
miR529b	LOC_Os08g06640.1	168	-23.18	168	-23.18	1570460	21	2001	1

miR529b	LOC_Os08g07300.1	170	-26.12	170	-26.12	1570520	21	1323	3
miR529b	LOC_Os08g07300.2	170	-26.12	170	-26.12	1570521	21	1061	3
miR529b	LOC_Os08g07400.1	153	-20.08	153	-20.08	1570530	21	2582	233
miR529b	LOC_Os08g07460.1	173	-24.66	173	-24.66	1570536	21	1971	499
miR529b	LOC_Os08g08110.1	162	-20.56	162	-20.56	1570604	21	1953	716
miR529b	LOC_Os08g08540.1	154	-20.29	154	-20.29	1570648	21	1758	14
miR529b	LOC_Os08g08780.1	161	-25	161	-25	1570680	21	2436	2411
miR529b	LOC_Os08g08830.1	162	-21.08	162	-21.08	1570686	21	3782	910
miR529b	LOC_Os08g08840.1	173	-26.11	173	-26.11	1570687	21	1819	913
miR529b	LOC_Os08g08840.2	173	-26.11	173	-26.11	1570688	21	1816	910
miR529b	LOC_Os08g11830.1	167	-21.93	167	-21.93	1571018	21	1987	1104
miR529b	LOC_Os08g11850.1	167	-21.93	167	-21.93	1571020	21	4332	3849
miR529b	LOC_Os08g12400.1	149	-20.5	149	-20.5	1571072	21	2466	2441
miR529b	LOC_Os08g12910.1	166	-21.94	166	-21.94	1571132	21	2208	204
miR529b	LOC_Os08g13350.1	164	-22.36	164	-22.36	1571176	21	2261	1948
miR529b	LOC_Os08g13350.2	164	-22.36	164	-22.36	1571177	21	2540	2227
miR529b	LOC_Os08g13760.1	161	-25	161	-25	1571221	21	2481	2456
miR529b	LOC_Os08g14195.1	161	-21.93	161	-21.93	1571263	21	2464	159
miR529b	LOC_Os08g14610.1	165	-21.5	165	-21.5	1571309	21	3447	2524
miR529b	LOC_Os08g14940.1	158	-22.07	158	-22.07	1571348	21	2904	24
miR529b	LOC_Os08g14950.1	162	-23.16	162	-23.16	1571349	21	3169	16
miR529b	LOC_Os08g14990.1	160	-20.85	160	-20.85	1571353	21	3390	3224
miR529b	LOC_Os08g17130.1	160	-21.76	160	-21.76	1571595	21	3447	3421
miR529b	LOC_Os08g17390.1	150	-20.22	150	-20.22	1571617	21	2443	1256
miR529b	LOC_Os08g17500.1	161	-22.02	161	-22.02	1571628	21	1420	84
miR529b	LOC_Os08g19374.1	165	-22.3	165	-22.3	1571775	21	2232	352
miR529b	LOC_Os08g19374.2	165	-22.3	165	-22.3	1571776	21	1725	352
miR529b	LOC_Os08g19374.3	165	-22.3	165	-22.3	1571777	21	1033	352
miR529b	LOC_Os08g19374.4	165	-22.3	165	-22.3	1571778	21	1130	352
miR529b	LOC_Os08g19530.1	153	-20.34	153	-20.34	1571793	21	1464	723
miR529b	LOC_Os08g19610.1	165	-22.01	165	-22.01	1571801	21	1468	421
miR529b	LOC_Os08g19610.2	165	-22.01	165	-22.01	1571802	21	2182	1135

miR529b	LOC_Os08g19610.3	165	-22.01	165	-22.01	1571803	21	1568	521
miR529b	LOC_Os08g19610.4	165	-22.01	165	-22.01	1571804	21	3303	1135
miR529b	LOC_Os08g19694.1	153	-20.85	153	-20.85	1571813	21	3636	1526
miR529b	LOC_Os08g19860.1	150	-20.94	150	-20.94	1571830	21	1854	445
miR529b	LOC_Os08g20580.1	165	-24	165	-24	1571899	21	675	105
miR529b	LOC_Os08g21110.1	159	-23.12	159	-23.12	1571939	21	6117	143
miR529b	LOC_Os08g21541.1	168	-22.44	168	-22.44	1571978	21	4751	476
miR529b	LOC_Os08g22180.1	153	-20.33	153	-20.33	1572045	21	4056	240
miR529b	LOC_Os08g22200.1	152	-20.24	152	-20.24	1572047	21	1088	381
miR529b	LOC_Os08g22284.1	168	-23.18	168	-23.18	1572052	21	2502	2460
miR529b	LOC_Os08g22630.1	161	-25	161	-25	1572082	21	2055	2030
miR529b	LOC_Os08g22760.1	154	-20.71	154	-20.71	1572093	21	5106	3643
miR529b	LOC_Os08g25330.1	157	-21.69	157	-21.69	1572356	21	4575	531
miR529b	LOC_Os08g25440.1	156	-20.02	156	-20.02	1572368	21	1302	1273
miR529b	LOC_Os08g25700.1	153	-20.25	153	-20.25	1572396	21	1747	1081
miR529b	LOC_Os08g26510.1	163	-20.86	163	-20.86	1572478	21	1203	488
miR529b	LOC_Os08g27570.1	154	-21	154	-21	1572590	21	2562	1324
miR529b	LOC_Os08g27790.1	322	-42.01	166	-21.94	1572615	21	1926	204 295
miR529b	LOC_Os08g28080.1	170	-23.08	170	-23.08	1572645	21	2514	1139
miR529b	LOC_Os08g28310.1	169	-23.84	169	-23.84	1572672	21	1848	1535
miR529b	LOC_Os08g28319.1	154	-20.29	154	-20.29	1572673	21	1464	14
miR529b	LOC_Os08g29615.1	161	-20.08	161	-20.08	1572806	21	2608	45
miR529b	LOC_Os08g29700.1	145	-20.29	145	-20.29	1572814	21	1314	695
miR529b	LOC_Os08g30770.1	161	-21.98	161	-21.98	1572923	21	3444	1092
miR529b	LOC_Os08g31630.1	168	-22.91	168	-22.91	1573014	21	2698	62
miR529b	LOC_Os08g31630.2	168	-22.91	168	-22.91	1573015	21	2891	53
miR529b	LOC_Os08g31940.1	322	-46.17	164	-23.37	1573052	21	1747	1247 29
miR529b	LOC_Os08g32490.1	163	-22.03	163	-22.03	1573110	21	360	254
miR529b	LOC_Os08g33082.1	168	-23.18	168	-23.18	1573179	21	3200	80
miR529b	LOC_Os08g34070.1	165	-20.36	165	-20.36	1573296	21	1470	7
miR529b	LOC_Os08g34070.2	165	-20.36	165	-20.36	1573297	21	1389	7
miR529b	LOC_Os08g34320.1	161	-21.02	161	-21.02	1573324	21	2419	1138

miR529b	LOC_Os08g34660.1	165	-22.28	165	-22.28	1573360	21	780	47
miR529b	LOC_Os08g35310.1	162	-21.37	162	-21.37	1573439	21	1041	83
miR529b	LOC_Os08g35990.1	167	-23.67	167	-23.67	1573513	21	381	6
miR529b	LOC_Os08g36110.1	161	-20.09	161	-20.09	1573525	21	1244	74
miR529b	LOC_Os08g37470.1	153	-20.31	153	-20.31	1573678	21	1089	34
miR529b	LOC_Os08g37540.1	157	-22.29	157	-22.29	1573686	21	2466	2441
miR529b	LOC_Os08g37710.1	149	-20.42	149	-20.42	1573709	21	1308	1234
miR529b	LOC_Os08g37780.1	171	-23.02	171	-23.02	1573717	21	2781	1965
miR529b	LOC_Os08g37780.2	171	-23.02	171	-23.02	1573718	21	2776	1965
miR529b	LOC_Os08g37920.1	157	-23.61	157	-23.61	1573737	21	3230	2964
miR529b	LOC_Os08g37920.2	157	-23.61	157	-23.61	1573738	21	3101	2835
miR529b	LOC_Os08g38040.1	156	-21.71	156	-21.71	1573754	21	2964	2526
miR529b	LOC_Os08g38100.1	168	-24.51	168	-24.51	1573762	21	1113	60
miR529b	LOC_Os08g38560.1	168	-24.98	168	-24.98	1573817	21	3625	52
miR529b	LOC_Os08g39310.1	162	-21.7	162	-21.7	1573918	21	1862	1500
miR529b	LOC_Os08g39760.1	145	-20.29	145	-20.29	1573968	21	5856	5063
miR529b	LOC_Os08g39980.1	164	-23.12	164	-23.12	1573997	21	2545	2508
miR529b	LOC_Os08g40290.1	311	-43.61	166	-21.97	1574032	21	3791	2710 2808
miR529b	LOC_Os08g40310.1	166	-20.05	166	-20.05	1574033	21	3132	3101
miR529b	LOC_Os08g40420.1	161	-20.05	161	-20.05	1574042	21	3593	2378
miR529b	LOC_Os08g40420.2	161	-20.05	161	-20.05	1574043	21	3744	2529
miR529b	LOC_Os08g40430.1	161	-20.05	161	-20.05	1574044	21	2237	877
miR529b	LOC_Os08g40480.1	151	-20.15	151	-20.15	1574049	21	1133	948
miR529b	LOC_Os08g40620.1	165	-21.81	165	-21.81	1574071	21	3411	85
miR529b	LOC_Os08g40620.2	165	-21.81	165	-21.81	1574072	21	3483	85
miR529b	LOC_Os08g40890.1	165	-27.42	165	-27.42	1574107	21	3193	1122
miR529b	LOC_Os08g40930.1	157	-21.1	157	-21.1	1574113	21	3772	693
miR529b	LOC_Os08g41620.1	166	-20.01	166	-20.01	1574199	21	2253	704
miR529b	LOC_Os08g41900.1	159	-20.35	159	-20.35	1574228	21	3189	2038
miR529b	LOC_Os08g41940.1	182	-32.61	182	-32.61	1574232	21	1708	1049
miR529b	LOC_Os08g42050.1	165	-21.76	165	-21.76	1574245	21	1106	655
miR529b	LOC_Os08g43010.1	162	-23.85	162	-23.85	1574368	21	3990	2398

miR529b	LOC_Os08g43130.1	153	-20.36	153	-20.36	1574383	21	4634	1362
miR529b	LOC_Os08g43710.1	161	-20.19	161	-20.19	1574452	21	957	883
miR529b	LOC_Os08g44760.1	171	-21.15	171	-21.15	1574578	21	2520	196
miR529b	LOC_Os08g44760.2	171	-21.15	171	-21.15	1574579	21	2505	184
miR529b	LOC_Os09g01100.1	167	-21.93	167	-21.93	1574671	21	4434	3906
miR529b	LOC_Os09g01540.1	161	-25	161	-25	1574718	21	2583	2558
miR529b	LOC_Os09g01830.1	154	-20.29	154	-20.29	1574754	21	1998	14
miR529b	LOC_Os09g01840.1	154	-20.29	154	-20.29	1574755	21	1998	14
miR529b	LOC_Os09g02360.1	178	-26.85	178	-26.85	1574808	21	1835	271
miR529b	LOC_Os09g03160.1	162	-20.9	162	-20.9	1574890	21	2724	554
miR529b	LOC_Os09g03620.1	164	-24.49	164	-24.49	1574938	21	2412	24
miR529b	LOC_Os09g03810.1	170	-23.03	170	-23.03	1574953	21	357	101
miR529b	LOC_Os09g04540.1	161	-20.5	161	-20.5	1575032	21	2067	2042
miR529b	LOC_Os09g04740.1	157	-21.69	157	-21.69	1575052	21	4383	546
miR529b	LOC_Os09g06230.1	168	-22.71	168	-22.71	1575105	21	1722	266
miR529b	LOC_Os09g06499.1	156	-20.49	156	-20.49	1575130	21	2703	673
miR529b	LOC_Os09g06499.2	156	-20.49	156	-20.49	1575131	21	2161	673
miR529b	LOC_Os09g06740.1	162	-21.27	162	-21.27	1575154	21	1258	25
miR529b	LOC_Os09g07970.1	169	-23.84	169	-23.84	1575295	21	2802	1835
miR529b	LOC_Os09g08020.1	145	-20.29	145	-20.29	1575300	21	4923	4346
miR529b	LOC_Os09g08510.1	151	-20.15	151	-20.15	1575351	21	1341	948
miR529b	LOC_Os09g08930.1	169	-23.84	169	-23.84	1575393	21	2934	2006
miR529b	LOC_Os09g09100.1	167	-21.93	167	-21.93	1575410	21	2691	1587
miR529b	LOC_Os09g09290.1	168	-23.18	168	-23.18	1575428	21	7384	4462
miR529b	LOC_Os09g09290.2	168	-23.18	168	-23.18	1575429	21	6473	4455
miR529b	LOC_Os09g10080.1	161	-25	161	-25	1575508	21	2484	2459
miR529b	LOC_Os09g10120.1	164	-23.85	164	-23.85	1575512	21	1626	147
miR529b	LOC_Os09g10140.1	164	-23.85	164	-23.85	1575514	21	1626	147
miR529b	LOC_Os09g10160.1	164	-23.85	164	-23.85	1575516	21	1626	147
miR529b	LOC_Os09g10750.1	153	-21.38	153	-21.38	1575578	21	1370	64
miR529b	LOC_Os09g11390.1	164	-24.01	164	-24.01	1575647	21	2184	1199
miR529b	LOC_Os09g11820.1	156	-21.71	156	-21.71	1575693	21	3594	2349

miR529b	LOC_Os09g11950.1	161	-25	161	-25	1575706	21	2058	2033
miR529b	LOC_Os09g12750.1	157	-20.1	157	-20.1	1575789	21	1420	254
miR529b	LOC_Os09g12750.2	157	-20.1	157	-20.1	1575790	21	2820	254
miR529b	LOC_Os09g12890.1	167	-21.93	167	-21.93	1575803	21	4776	3819
miR529b	LOC_Os09g13050.1	165	-21.55	165	-21.55	1575818	21	540	41
miR529b	LOC_Os09g13890.1	164	-24.87	164	-24.87	1575907	21	1644	12
miR529b	LOC_Os09g14300.1	154	-20.29	154	-20.29	1575949	21	438	72
miR529b	LOC_Os09g14410.1	159	-23.92	159	-23.92	1575960	21	3784	2521
miR529b	LOC_Os09g14930.1	163	-20.91	163	-20.91	1576019	21	1734	30
miR529b	LOC_Os09g15800.1	170	-27.09	170	-27.09	1576116	21	2268	217
miR529b	LOC_Os09g16430.1	157	-21.69	157	-21.69	1576181	21	3786	531
miR529b	LOC_Os09g16850.1	156	-23.58	156	-23.58	1576225	21	1152	52
miR529b	LOC_Os09g16995.1	157	-21.65	157	-21.65	1576240	21	3285	2201
miR529b	LOC_Os09g17600.1	159	-21.27	159	-21.27	1576301	21	2154	20
miR529b	LOC_Os09g17600.2	159	-21.27	159	-21.27	1576302	21	2131	1
miR529b	LOC_Os09g17600.3	159	-21.27	159	-21.27	1576303	21	2315	22
miR529b	LOC_Os09g19600.1	161	-25	161	-25	1576446	21	2436	2411
miR529b	LOC_Os09g19830.1	326	-41.6	170	-20.96	1576470	21	3844	3109 3744
miR529b	LOC_Os09g19840.1	342	-48.8	174	-24.54	1576471	21	435	28 57
miR529b	LOC_Os09g20740.1	153	-20.46	153	-20.46	1576577	21	1317	96
miR529b	LOC_Os09g21120.1	174	-24.54	174	-24.54	1576618	21	2592	29
miR529b	LOC_Os09g21250.1	162	-20.9	162	-20.9	1576634	21	1085	581
miR529b	LOC_Os09g22090.1	162	-20.54	162	-20.54	1576720	21	3525	2922
miR529b	LOC_Os09g23840.1	154	-20.29	154	-20.29	1576852	21	1818	14
miR529b	LOC_Os09g24820.1	174	-25.45	174	-25.45	1576957	21	4291	3585
miR529b	LOC_Os09g24840.1	166	-20.74	166	-20.74	1576959	21	696	86
miR529b	LOC_Os09g24840.2	166	-20.74	166	-20.74	1576960	21	679	86
miR529b	LOC_Os09g26390.1	168	-23.18	168	-23.18	1577128	21	3659	2624
miR529b	LOC_Os09g26390.2	168	-23.18	168	-23.18	1577129	21	4010	2975
miR529b	LOC_Os09g26390.3	168	-23.18	168	-23.18	1577130	21	4062	3027
miR529b	LOC_Os09g26580.1	156	-21.09	156	-21.09	1577154	21	2379	15
miR529b	LOC_Os09g27700.1	158	-20.55	158	-20.55	1577296	21	2717	1341

miR529b	LOC_Os09g27700.2	158	-20.55	158	-20.55	1577297	21	2855	1479
miR529b	LOC_Os09g28370.1	144	-20.23	144	-20.23	1577376	21	6942	2020
miR529b	LOC_Os09g28710.1	172	-24.1	172	-24.1	1577414	21	864	634
miR529b	LOC_Os09g29510.1	164	-24.7	164	-24.7	1577506	21	2271	1096
miR529b	LOC_Os09g29600.1	152	-20.32	152	-20.32	1577514	21	2475	418
miR529b	LOC_Os09g29720.1	169	-23.84	169	-23.84	1577526	21	2775	1847
miR529b	LOC_Os09g30150.1	162	-20.32	162	-20.32	1577582	21	2463	1435
miR529b	LOC_Os09g30150.2	162	-20.32	162	-20.32	1577583	21	2174	1146
miR529b	LOC_Os09g30150.3	162	-20.32	162	-20.32	1577584	21	2313	1285
miR529b	LOC_Os09g30400.1	164	-20.5	164	-20.5	1577615	21	2835	2230
miR529b	LOC_Os09g30411.1	156	-21.65	156	-21.65	1577620	21	1321	512
miR529b	LOC_Os09g30412.1	166	-20.09	166	-20.09	1577621	21	2613	1740
miR529b	LOC_Os09g30418.1	166	-20.09	166	-20.09	1577625	21	2493	1745
miR529b	LOC_Os09g31300.1	165	-20.36	165	-20.36	1577687	21	2112	215
miR529b	LOC_Os09g31300.2	165	-20.36	165	-20.36	1577688	21	2106	215
miR529b	LOC_Os09g31390.1	166	-21.77	166	-21.77	1577696	21	2479	2180
miR529b	LOC_Os09g31390.2	166	-21.77	166	-21.77	1577697	21	2473	2174
miR529b	LOC_Os09g32080.1	159	-21.76	159	-21.76	1577752	21	1850	45
miR529b	LOC_Os09g32080.2	159	-21.76	159	-21.76	1577753	21	1726	45
miR529b	LOC_Os09g32510.1	165	-23.27	165	-23.27	1577803	21	1656	939
miR529b	LOC_Os09g32510.2	165	-23.27	165	-23.27	1577804	21	1741	1024
miR529b	LOC_Os09g32510.3	165	-23.27	165	-23.27	1577805	21	1555	838
miR529b	LOC_Os09g32510.4	165	-23.27	165	-23.27	1577806	21	1577	860
miR529b	LOC_Os09g32510.5	165	-23.27	165	-23.27	1577807	21	1669	860
miR529b	LOC_Os09g32944.1	182	-32.61	182	-32.61	1577865	21	1419	1029
miR529b	LOC_Os09g32964.1	152	-23.18	152	-23.18	1577872	21	1212	154
miR529b	LOC_Os09g33660.1	165	-22.38	165	-22.38	1577911	21	171	43
miR529b	LOC_Os09g33860.1	151	-21.68	151	-21.68	1577941	21	1756	16
miR529b	LOC_Os09g34180.1	168	-24.98	168	-24.98	1577993	21	2888	73
miR529b	LOC_Os09g34860.1	155	-20.07	155	-20.07	1578019	21	1453	1272
miR529b	LOC_Os09g36010.1	168	-24.98	168	-24.98	1578098	21	936	42
miR529b	LOC_Os09g36450.1	164	-23.43	164	-23.43	1578154	21	1459	736

miR529b	LOC_Os09g36450.2	164	-23.43	164	-23.43	1578155	21	1438	715
miR529b	LOC_Os09g37510.1	164	-22.28	164	-22.28	1578281	21	1845	1826
miR529b	LOC_Os09g37910.1	171	-21.22	171	-21.22	1578323	21	956	738
miR529b	LOC_Os09g37930.1	314	-43.15	162	-22.75	1578325	21	3690	3414 3390
miR529b	LOC_Os09g38620.1	158	-20.99	158	-20.99	1578403	21	2678	870
miR529b	LOC_Os09g38970.1	161	-21.84	161	-21.84	1578450	21	2753	2700
miR529b	LOC_Os09g39230.1	158	-20.91	158	-20.91	1578476	21	1682	1431
miR529b	LOC_Os09g39410.1	153	-20.52	153	-20.52	1578497	21	2078	60
miR529b	LOC_Os10g01020.1	145	-20.29	145	-20.29	1578589	21	5976	5390
miR529b	LOC_Os10g01200.1	155	-20.37	155	-20.37	1578606	21	873	405
miR529b	LOC_Os10g01610.1	160	-21.57	160	-21.57	1578646	21	3893	2250
miR529b	LOC_Os10g01750.1	165	-21.47	165	-21.47	1578659	21	4053	3273
miR529b	LOC_Os10g01850.1	160	-20.25	160	-20.25	1578670	21	3147	2370
miR529b	LOC_Os10g01900.1	157	-21.83	157	-21.83	1578675	21	1077	594
miR529b	LOC_Os10g02230.1	156	-20.4	156	-20.4	1578711	21	708	21
miR529b	LOC_Os10g02500.1	161	-21.98	161	-21.98	1578739	21	3948	3839
miR529b	LOC_Os10g02760.1	163	-20.23	163	-20.23	1578766	21	2365	1518
miR529b	LOC_Os10g02760.2	163	-20.23	163	-20.23	1578767	21	2362	1518
miR529b	LOC_Os10g02910.1	159	-21.27	159	-21.27	1578782	21	2821	961
miR529b	LOC_Os10g03330.1	169	-23.84	169	-23.84	1578824	21	3081	1892
miR529b	LOC_Os10g04850.1	165	-23.07	165	-23.07	1578974	21	2187	615
miR529b	LOC_Os10g05130.1	166	-21.48	166	-21.48	1579001	21	1384	102
miR529b	LOC_Os10g05610.1	173	-24.6	173	-24.6	1579054	21	210	58
miR529b	LOC_Os10g05695.1	336	-48.64	168	-24.38	1579062	21	3975	3667 3690
miR529b	LOC_Os10g06520.1	167	-21.93	167	-21.93	1579146	21	4893	3735
miR529b	LOC_Os10g06630.1	174	-22.74	174	-22.74	1579155	21	1015	91
miR529b	LOC_Os10g07510.1	154	-20.53	154	-20.53	1579234	21	1030	830
miR529b	LOC_Os10g09060.1	167	-21.93	167	-21.93	1579385	21	3423	3354
miR529b	LOC_Os10g09970.1	167	-21.93	167	-21.93	1579472	21	3735	2403
miR529b	LOC_Os10g10290.1	153	-20.34	153	-20.34	1579510	21	1509	1275
miR529b	LOC_Os10g10400.1	164	-22.7	164	-22.7	1579523	21	363	38
miR529b	LOC_Os10g10820.1	161	-25	161	-25	1579563	21	2580	2555

miR529b	LOC_Os10g10840.1	161	-25	161	-25	1579565	21	2436	2411
miR529b	LOC_Os10g11220.1	178	-28.3	178	-28.3	1579601	21	2751	1517
miR529b	LOC_Os10g11310.1	165	-22	165	-22	1579612	21	2833	1116
miR529b	LOC_Os10g11310.2	165	-22	165	-22	1579613	21	2891	1116
miR529b	LOC_Os10g11310.3	165	-22	165	-22	1579614	21	2841	1116
miR529b	LOC_Os10g12280.1	157	-22.29	157	-22.29	1579696	21	2538	2513
miR529b	LOC_Os10g12750.1	170	-23.76	170	-23.76	1579734	21	1409	1373
miR529b	LOC_Os10g13340.1	154	-20.29	154	-20.29	1579747	21	1632	14
miR529b	LOC_Os10g13940.1	157	-21.63	157	-21.63	1579808	21	2053	1208
miR529b	LOC_Os10g13990.1	161	-25	161	-25	1579814	21	2481	2456
miR529b	LOC_Os10g14030.1	178	-28.3	178	-28.3	1579818	21	1692	989
miR529b	LOC_Os10g15390.1	158	-20.59	158	-20.59	1579887	21	1045	395
miR529b	LOC_Os10g15390.2	158	-20.59	158	-20.59	1579888	21	1004	364
miR529b	LOC_Os10g16620.1	167	-22.36	167	-22.36	1579937	21	537	5
miR529b	LOC_Os10g17310.1	164	-20.53	164	-20.53	1580000	21	2106	1065
miR529b	LOC_Os10g17410.1	154	-21	154	-21	1580010	21	3336	1381
miR529b	LOC_Os10g19310.1	158	-20.48	158	-20.48	1580181	21	1257	600
miR529b	LOC_Os10g20710.1	161	-20.64	161	-20.64	1580296	21	2550	21
miR529b	LOC_Os10g21460.1	164	-21.13	164	-21.13	1580402	21	1512	114
miR529b	LOC_Os10g21470.1	170	-22.93	170	-22.93	1580403	21	3784	2194
miR529b	LOC_Os10g21470.2	170	-22.93	170	-22.93	1580404	21	1879	289
miR529b	LOC_Os10g22100.1	163	-20.77	163	-20.77	1580468	21	2503	667
miR529b	LOC_Os10g22300.1	163	-23.19	163	-23.19	1580488	21	2166	1074
miR529b	LOC_Os10g22410.1	325	-44.54	164	-23.17	1580502	21	811	384 167
miR529b	LOC_Os10g22950.1	164	-21.49	164	-21.49	1580562	21	3560	3281
miR529b	LOC_Os10g23090.1	164	-20.56	164	-20.56	1580577	21	1440	153
miR529b	LOC_Os10g23100.1	165	-24.26	165	-24.26	1580579	21	1286	1064
miR529b	LOC_Os10g23190.1	156	-21.71	156	-21.71	1580590	21	2772	1026
miR529b	LOC_Os10g23880.1	167	-23.13	167	-23.13	1580610	21	3528	2988
miR529b	LOC_Os10g24230.1	151	-21.1	151	-21.1	1580648	21	3234	2755
miR529b	LOC_Os10g24450.1	164	-20.53	164	-20.53	1580669	21	2798	918
miR529b	LOC_Os10g25200.1	160	-25.47	160	-25.47	1580741	21	4170	1550

miR529b	LOC_Os10g25550.1	146	-22.27	146	-22.27	1580773	21	1485	471
miR529b	LOC_Os10g25690.1	166	-22.69	166	-22.69	1580792	21	405	341
miR529b	LOC_Os10g26510.1	168	-24.51	168	-24.51	1580876	21	1002	76
miR529b	LOC_Os10g26540.1	168	-24.98	168	-24.98	1580879	21	866	5
miR529b	LOC_Os10g26730.1	152	-22.44	152	-22.44	1580901	21	1702	585
miR529b	LOC_Os10g26730.2	152	-22.44	152	-22.44	1580902	21	1610	585
miR529b	LOC_Os10g28060.1	157	-20.83	157	-20.83	1580992	21	1856	1807
miR529b	LOC_Os10g28254.1	316	-42.26	159	-21.55	1581011	21	902	12 23
miR529b	LOC_Os10g28790.1	157	-20.51	157	-20.51	1581075	21	1122	184
miR529b	LOC_Os10g29670.1	166	-21.55	166	-21.55	1581163	21	1249	447
miR529b	LOC_Os10g29670.2	166	-21.55	166	-21.55	1581164	21	1478	676
miR529b	LOC_Os10g30580.1	166	-24.54	166	-24.54	1581249	21	2882	40
miR529b	LOC_Os10g31040.1	161	-22.11	161	-22.11	1581300	21	2525	156
miR529b	LOC_Os10g31040.5	161	-22.11	161	-22.11	1581304	21	2445	154
miR529b	LOC_Os10g31600.1	161	-23.87	161	-23.87	1581357	21	2544	2519
miR529b	LOC_Os10g31830.1	164	-22.08	164	-22.08	1581380	21	1468	938
miR529b	LOC_Os10g33720.1	168	-24.98	168	-24.98	1581566	21	2580	14
miR529b	LOC_Os10g33750.1	155	-20.1	155	-20.1	1581571	21	1309	1077
miR529b	LOC_Os10g33850.1	161	-21.35	161	-21.35	1581587	21	1386	28
miR529b	LOC_Os10g35630.1	158	-22.8	158	-22.8	1581791	21	1625	101
miR529b	LOC_Os10g37510.1	164	-22.28	164	-22.28	1581982	21	708	23
miR529b	LOC_Os10g37520.1	168	-23.18	168	-23.18	1581983	21	1883	29
miR529b	LOC_Os10g37520.2	168	-23.18	168	-23.18	1581984	21	1829	29
miR529b	LOC_Os10g37960.1	168	-25.5	168	-25.5	1582040	21	1692	1234
miR529b	LOC_Os10g38196.1	153	-21.5	153	-21.5	1582064	21	402	138
miR529b	LOC_Os10g38960.1	155	-20.45	155	-20.45	1582133	21	2025	60
miR529b	LOC_Os10g39710.1	174	-25.45	174	-25.45	1582231	21	1865	1542
miR529b	LOC_Os10g39780.1	173	-25.25	173	-25.25	1582240	21	2295	248
miR529b	LOC_Os10g39780.3	173	-25.25	173	-25.25	1582242	21	2284	248
miR529b	LOC_Os10g39780.4	173	-25.25	173	-25.25	1582243	21	1978	248
miR529b	LOC_Os10g40160.1	157	-22.29	157	-22.29	1582304	21	2496	2471
miR529b	LOC_Os10g40280.1	158	-20.25	158	-20.25	1582317	21	1658	1197

miR529b	LOC_Os10g40640.1	155	-20.8	155	-20.8	1582352	21	2374	6
miR529b	LOC_Os10g40730.1	161	-22.23	161	-22.23	1582359	21	1637	309
miR529b	LOC_Os10g40790.1	165	-22.01	165	-22.01	1582364	21	843	36
miR529b	LOC_Os10g41040.1	158	-21.4	158	-21.4	1582395	21	2671	2557
miR529b	LOC_Os10g41040.2	158	-21.4	158	-21.4	1582396	21	2770	2656
miR529b	LOC_Os10g41224.1	165	-21.84	165	-21.84	1582416	21	970	360
miR529b	LOC_Os10g41440.1	166	-25.37	166	-25.37	1582443	21	1823	15
miR529b	LOC_Os10g41480.1	159	-20.6	159	-20.6	1582447	21	1619	34
miR529b	LOC_Os10g41580.1	165	-20.36	165	-20.36	1582457	21	1643	1498
miR529b	LOC_Os10g41960.1	170	-23.73	170	-23.73	1582511	21	2628	349
miR529b	LOC_Os10g42700.1	162	-20.89	162	-20.89	1582594	21	1586	208
miR529b	LOC_Os10g42754.1	165	-22.01	165	-22.01	1582608	21	1473	232
miR529b	LOC_Os11g01170.1	168	-24.98	168	-24.98	1582676	21	1383	102
miR529b	LOC_Os11g01470.1	174	-22.84	174	-22.84	1582708	21	5073	4736
miR529b	LOC_Os11g01480.1	160	-22.17	160	-22.17	1582709	21	1710	378
miR529b	LOC_Os11g01820.1	164	-20.31	164	-20.31	1582746	21	3023	2570
miR529b	LOC_Os11g01836.1	169	-23.56	169	-23.56	1582748	21	2594	874
miR529b	LOC_Os11g02464.1	165	-21.26	165	-21.26	1582831	21	2615	1873
miR529b	LOC_Os11g03420.1	154	-21.28	154	-21.28	1582936	21	744	2
miR529b	LOC_Os11g03700.1	153	-21.69	153	-21.69	1582968	21	1995	1636
miR529b	LOC_Os11g03970.1	155	-20.14	155	-20.14	1582998	21	1940	1476
miR529b	LOC_Os11g03970.2	155	-20.14	155	-20.14	1582999	21	1924	1460
miR529b	LOC_Os11g04220.1	154	-20.16	154	-20.16	1583030	21	5526	936
miR529b	LOC_Os11g04440.2	166	-20.3	166	-20.3	1583061	21	936	168
miR529b	LOC_Os11g04450.1	166	-20.57	166	-20.57	1583063	21	471	60
miR529b	LOC_Os11g05300.1	167	-21.68	167	-21.68	1583165	21	1105	616
miR529b	LOC_Os11g05930.1	153	-20.86	153	-20.86	1583234	21	2358	2216
miR529b	LOC_Os11g05930.2	153	-20.86	153	-20.86	1583235	21	2372	2230
miR529b	LOC_Os11g05930.3	153	-20.86	153	-20.86	1583236	21	2506	2364
miR529b	LOC_Os11g05930.4	153	-20.86	153	-20.86	1583237	21	2490	2355
miR529b	LOC_Os11g05930.5	153	-20.86	153	-20.86	1583238	21	2651	2515
miR529b	LOC_Os11g06200.1	151	-20.44	151	-20.44	1583270	21	888	97

miR529b	LOC_Os11g06420.1	159	-20.11	159	-20.11	1583301	21	2107	241
miR529b	LOC_Os11g06420.2	159	-20.11	159	-20.11	1583302	21	2103	241
miR529b	LOC_Os11g06500.1	168	-21.4	168	-21.4	1583310	21	993	384
miR529b	LOC_Os11g06910.1	161	-20.37	161	-20.37	1583355	21	3414	3241
miR529b	LOC_Os11g06910.2	161	-20.37	161	-20.37	1583356	21	3511	3338
miR529b	LOC_Os11g06910.3	161	-20.37	161	-20.37	1583357	21	3521	3348
miR529b	LOC_Os11g07880.1	164	-21.56	164	-21.56	1583474	21	772	646
miR529b	LOC_Os11g08050.1	161	-22.38	161	-22.38	1583494	21	2130	696
miR529b	LOC_Os11g08210.1	162	-20.28	162	-20.28	1583512	21	1998	409
miR529b	LOC_Os11g08480.1	165	-24	165	-24	1583546	21	651	18
miR529b	LOC_Os11g09900.1	166	-20.56	166	-20.56	1583692	21	1664	701
miR529b	LOC_Os11g10090.1	148	-20.25	148	-20.25	1583712	21	2046	236
miR529b	LOC_Os11g10420.1	157	-20.38	157	-20.38	1583746	21	4891	3011
miR529b	LOC_Os11g10420.2	157	-20.38	157	-20.38	1583747	21	5159	3279
miR529b	LOC_Os11g10970.1	157	-21.69	157	-21.69	1583804	21	3300	531
miR529b	LOC_Os11g12130.1	167	-21.93	167	-21.93	1583923	21	3960	3477
miR529b	LOC_Os11g13780.1	174	-26.46	174	-26.46	1584036	21	1017	937
miR529b	LOC_Os11g14140.1	160	-22.33	160	-22.33	1584082	21	1654	267
miR529b	LOC_Os11g14510.1	156	-21.71	156	-21.71	1584121	21	4059	2220
miR529b	LOC_Os11g14630.1	155	-21.7	155	-21.7	1584142	21	1857	1682
miR529b	LOC_Os11g15100.1	157	-22.29	157	-22.29	1584196	21	2448	2423
miR529b	LOC_Os11g16550.1	158	-23.36	158	-23.36	1584309	21	1573	516
miR529b	LOC_Os11g16580.1	146	-20.79	146	-20.79	1584312	21	1656	596
miR529b	LOC_Os11g17230.1	165	-22.26	165	-22.26	1584369	21	3858	3043
miR529b	LOC_Os11g17530.1	161	-22.38	161	-22.38	1584400	21	2705	2395
miR529b	LOC_Os11g17600.1	161	-23.99	161	-23.99	1584406	21	3207	125
miR529b	LOC_Os11g17600.2	161	-23.99	161	-23.99	1584407	21	3270	125
miR529b	LOC_Os11g17600.3	161	-23.99	161	-23.99	1584408	21	3048	125
miR529b	LOC_Os11g17970.1	153	-21.47	153	-21.47	1584441	21	2197	1839
miR529b	LOC_Os11g18930.1	166	-23.01	166	-23.01	1584528	21	306	1
miR529b	LOC_Os11g19780.1	160	-20.31	160	-20.31	1584621	21	305	204
miR529b	LOC_Os11g19810.1	169	-21.99	169	-21.99	1584624	21	3438	1680

miR529b	LOC_Os11g19810.2	169	-21.99	169	-21.99	1584625	21	3642	1686
miR529b	LOC_Os11g20610.1	316	-41.82	161	-21.52	1584701	21	3786	2977 2526
miR529b	LOC_Os11g21790.1	167	-23.13	167	-23.13	1584720	21	3216	2361
miR529b	LOC_Os11g22020.1	161	-24.99	161	-24.99	1584743	21	2205	2180
miR529b	LOC_Os11g22150.1	149	-20.68	149	-20.68	1584756	21	651	436
miR529b	LOC_Os11g22150.2	149	-20.68	149	-20.68	1584757	21	679	459
miR529b	LOC_Os11g22550.1	164	-23.56	164	-23.56	1584799	21	3138	2681
miR529b	LOC_Os11g23830.1	157	-21.69	157	-21.69	1584888	21	1689	135
miR529b	LOC_Os11g26010.1	162	-20.54	162	-20.54	1585094	21	2798	110
miR529b	LOC_Os11g26520.1	161	-20.3	161	-20.3	1585141	21	3621	2488
miR529b	LOC_Os11g28140.1	169	-23.84	169	-23.84	1585298	21	3162	1973
miR529b	LOC_Os11g28970.1	164	-22.81	164	-22.81	1585383	21	1227	450
miR529b	LOC_Os11g30170.1	156	-21.71	156	-21.71	1585509	21	4053	2763
miR529b	LOC_Os11g30280.1	155	-20.1	155	-20.1	1585520	21	498	132
miR529b	LOC_Os11g30300.1	170	-25.32	170	-25.32	1585522	21	387	1
miR529b	LOC_Os11g31900.1	154	-20.27	154	-20.27	1585678	21	877	269
miR529b	LOC_Os11g31900.2	154	-20.27	154	-20.27	1585679	21	827	331
miR529b	LOC_Os11g31900.3	154	-20.27	154	-20.27	1585680	21	1072	269
miR529b	LOC_Os11g32900.1	162	-20.95	162	-20.95	1585787	21	3416	10
miR529b	LOC_Os11g32900.2	162	-20.95	162	-20.95	1585788	21	3508	8
miR529b	LOC_Os11g33030.1	149	-22.93	149	-22.93	1585804	21	1618	1544
miR529b	LOC_Os11g34750.1	156	-24.29	156	-24.29	1585945	21	2274	1532
miR529b	LOC_Os11g35220.1	161	-20.63	161	-20.63	1585995	21	2284	1722
miR529b	LOC_Os11g35350.1	158	-20.75	158	-20.75	1586007	21	816	382
miR529b	LOC_Os11g35650.1	178	-28.3	178	-28.3	1586041	21	2751	1517
miR529b	LOC_Os11g36430.1	160	-21.25	160	-21.25	1586130	21	1750	1068
miR529b	LOC_Os11g36430.2	160	-21.25	160	-21.25	1586131	21	1627	1068
miR529b	LOC_Os11g36430.3	160	-21.25	160	-21.25	1586132	21	1665	983
miR529b	LOC_Os11g36430.5	160	-21.25	160	-21.25	1586133	21	1833	1151
miR529b	LOC_Os11g36470.1	174	-26.62	174	-26.62	1586137	21	3463	2312
miR529b	LOC_Os11g36510.1	157	-20.12	157	-20.12	1586142	21	2802	1195
miR529b	LOC_Os11g36630.1	166	-21.94	166	-21.94	1586153	21	2184	204

miR529b	LOC_Os11g37090.1	156	-21.1	156	-21.1	1586196	21	3185	3147
miR529b	LOC_Os11g37260.1	168	-22.91	168	-22.91	1586212	21	2897	35
miR529b	LOC_Os11g37520.1	328	-46.71	168	-24.98	1586237	21	3712	15 3626
miR529b	LOC_Os11g37740.1	152	-20.89	152	-20.89	1586260	21	3330	1951
miR529b	LOC_Os11g38520.1	166	-21.86	166	-21.86	1586345	21	4343	2839
miR529b	LOC_Os11g39640.1	156	-20.61	156	-20.61	1586473	21	2007	1866
miR529b	LOC_Os11g40315.1	166	-20.4	166	-20.4	1586542	21	2838	1820
miR529b	LOC_Os11g40400.1	161	-22.93	161	-22.93	1586552	21	891	44
miR529b	LOC_Os11g40490.1	158	-25.74	158	-25.74	1586561	21	1116	1071
miR529b	LOC_Os11g40510.1	165	-20.94	165	-20.94	1586565	21	2088	1036
miR529b	LOC_Os11g40510.2	165	-20.94	165	-20.94	1586566	21	1974	1036
miR529b	LOC_Os11g41390.1	150	-21.82	150	-21.82	1586658	21	1305	1036
miR529b	LOC_Os11g41600.1	161	-20.95	161	-20.95	1586682	21	1112	943
miR529b	LOC_Os11g41600.2	161	-20.95	161	-20.95	1586683	21	1043	874
miR529b	LOC_Os11g42330.1	157	-22.29	157	-22.29	1586758	21	2466	2441
miR529b	LOC_Os11g42340.1	336	-48.17	168	-24.38	1586759	21	423	22 47
miR529b	LOC_Os11g43380.1	161	-25	161	-25	1586867	21	2436	2411
miR529b	LOC_Os11g43390.1	159	-20.19	159	-20.19	1586868	21	2800	1448
miR529b	LOC_Os11g43404.1	165	-25.12	165	-25.12	1586870	21	2264	1386
miR529b	LOC_Os11g43404.2	165	-25.12	165	-25.12	1586871	21	2347	1468
miR529b	LOC_Os11g43404.5	165	-25.12	165	-25.12	1586874	21	1409	530
miR529b	LOC_Os11g44280.1	160	-21.55	160	-21.55	1586967	21	183	18
miR529b	LOC_Os11g45000.1	165	-20.87	165	-20.87	1587043	21	2868	501
miR529b	LOC_Os11g45370.1	164	-22.97	164	-22.97	1587080	21	414	5
miR529b	LOC_Os11g45620.1	166	-23.63	166	-23.63	1587107	21	2838	1580
miR529b	LOC_Os11g45660.1	178	-28.3	178	-28.3	1587109	21	2751	1517
miR529b	LOC_Os11g46120.1	168	-24.98	168	-24.98	1587163	21	2870	2093
miR529b	LOC_Os11g46200.1	161	-20.63	161	-20.63	1587169	21	3378	1672
miR529b	LOC_Os11g46290.1	156	-21.71	156	-21.71	1587179	21	2598	1026
miR529b	LOC_Os11g47430.1	154	-20.23	154	-20.23	1587244	21	5769	4558
miR529b	LOC_Os11g47432.1	166	-25.87	166	-25.87	1587248	21	4251	723
miR529b	LOC_Os11g47438.1	166	-25.87	166	-25.87	1587252	21	4020	723

miR529b	LOC_Os11g47574.1	158	-22.5	158	-22.5	1587281	21	1010	19
miR529b	LOC_Os11g47650.1	162	-20.5	162	-20.5	1587289	21	1830	61
miR529b	LOC_Os11g47650.2	162	-20.5	162	-20.5	1587290	21	1911	61
miR529b	LOC_Os12g01170.1	168	-24.98	168	-24.98	1587367	21	1389	96
miR529b	LOC_Os12g01490.1	158	-22.28	158	-22.28	1587403	21	1803	473
miR529b	LOC_Os12g01820.1	165	-21.92	165	-21.92	1587445	21	2994	2737
miR529b	LOC_Os12g01850.1	169	-23.56	169	-23.56	1587447	21	2535	782
miR529b	LOC_Os12g01955.1	171	-26.04	171	-26.04	1587465	21	1435	694
miR529b	LOC_Os12g02390.1	165	-21.26	165	-21.26	1587517	21	2626	1865
miR529b	LOC_Os12g02730.1	155	-20.23	155	-20.23	1587566	21	779	611
miR529b	LOC_Os12g02840.1	153	-20.33	153	-20.33	1587584	21	2891	2015
miR529b	LOC_Os12g02960.1	165	-20.42	165	-20.42	1587596	21	911	780
miR529b	LOC_Os12g03110.1	154	-21.28	154	-21.28	1587616	21	742	5
miR529b	LOC_Os12g03810.1	155	-20.14	155	-20.14	1587689	21	2183	1721
miR529b	LOC_Os12g03810.2	155	-20.14	155	-20.14	1587690	21	1934	1472
miR529b	LOC_Os12g04030.1	154	-20.16	154	-20.16	1587722	21	3078	1112
miR529b	LOC_Os12g04130.1	165	-22.27	165	-22.27	1587732	21	2105	1495
miR529b	LOC_Os12g05500.1	161	-24.99	161	-24.99	1587895	21	2079	2054
miR529b	LOC_Os12g05810.1	160	-22.87	160	-22.87	1587930	21	1439	123
miR529b	LOC_Os12g05810.2	160	-22.87	160	-22.87	1587931	21	1509	123
miR529b	LOC_Os12g05810.3	160	-22.87	160	-22.87	1587932	21	1156	123
miR529b	LOC_Os12g06040.1	156	-21.71	156	-21.71	1587953	21	4671	2994
miR529b	LOC_Os12g06260.1	165	-20.52	165	-20.52	1587977	21	1397	1205
miR529b	LOC_Os12g06410.1	160	-21.05	160	-21.05	1587993	21	1314	521
miR529b	LOC_Os12g06980.1	145	-20.08	145	-20.08	1588063	21	7811	5349
miR529b	LOC_Os12g07840.1	167	-21.81	167	-21.81	1588155	21	3569	6
miR529b	LOC_Os12g07940.1	161	-21.83	161	-21.83	1588166	21	2873	1861
miR529b	LOC_Os12g09530.1	166	-24.94	166	-24.94	1588340	21	3303	3049
miR529b	LOC_Os12g09870.1	157	-22.29	157	-22.29	1588374	21	2466	2441
miR529b	LOC_Os12g11200.1	163	-21.21	163	-21.21	1588517	21	2034	852
miR529b	LOC_Os12g11680.1	175	-20.79	175	-20.79	1588561	21	3282	3049
miR529b	LOC_Os12g11720.1	175	-20.79	175	-20.79	1588565	21	3060	2960

miR529b	LOC_Os12g11860.1	175	-20.99	175	-20.99	1588578	21	3018	2918
miR529b	LOC_Os12g11930.1	165	-22.15	165	-22.15	1588583	21	3273	996
miR529b	LOC_Os12g12010.1	175	-20.79	175	-20.79	1588590	21	3000	2900
miR529b	LOC_Os12g12120.1	175	-20.79	175	-20.79	1588602	21	3297	2967
miR529b	LOC_Os12g12130.1	179	-23.49	179	-23.49	1588603	21	3329	2994
miR529b	LOC_Os12g12230.1	312	-41.3	161	-21.13	1588614	21	540	140 437
miR529b	LOC_Os12g12664.1	166	-24.87	166	-24.87	1588660	21	1797	449
miR529b	LOC_Os12g12664.2	166	-24.87	166	-24.87	1588661	21	3510	449
miR529b	LOC_Os12g12880.1	169	-25.74	169	-25.74	1588685	21	1198	1022
miR529b	LOC_Os12g13130.1	157	-20.16	157	-20.16	1588709	21	1455	104
miR529b	LOC_Os12g13130.2	157	-20.16	157	-20.16	1588710	21	1462	127
miR529b	LOC_Os12g13130.3	157	-20.16	157	-20.16	1588711	21	1669	127
miR529b	LOC_Os12g13130.4	157	-20.16	157	-20.16	1588712	21	1419	127
miR529b	LOC_Os12g13290.1	157	-21.46	157	-21.46	1588731	21	2419	306
miR529b	LOC_Os12g13400.1	161	-20.44	161	-20.44	1588745	21	1585	210
miR529b	LOC_Os12g14480.1	159	-23.91	159	-23.91	1588858	21	1095	1000
miR529b	LOC_Os12g15620.1	166	-21.94	166	-21.94	1588962	21	1902	204
miR529b	LOC_Os12g15780.1	160	-21.31	160	-21.31	1588976	21	3660	1569
miR529b	LOC_Os12g16350.1	160	-20.16	160	-20.16	1589030	21	2778	546
miR529b	LOC_Os12g16350.13	165	-24.08	165	-24.08	1589039	21	4262	932
miR529b	LOC_Os12g17300.1	164	-20.14	164	-20.14	1589141	21	2370	2345
miR529b	LOC_Os12g17580.1	157	-20.43	157	-20.43	1589169	21	333	289
miR529b	LOC_Os12g18410.1	149	-20.45	149	-20.45	1589260	21	1164	146
miR529b	LOC_Os12g18410.2	149	-20.45	149	-20.45	1589261	21	1009	86
miR529b	LOC_Os12g19290.1	150	-21.26	150	-21.26	1589348	21	2503	175
miR529b	LOC_Os12g19770.1	157	-21.69	157	-21.69	1589394	21	4674	531
miR529b	LOC_Os12g20300.1	168	-21.13	168	-21.13	1589447	21	4695	1724
miR529b	LOC_Os12g20350.1	161	-25	161	-25	1589454	21	2490	2465
miR529b	LOC_Os12g21640.1	155	-20.37	155	-20.37	1589487	21	4551	4014
miR529b	LOC_Os12g23010.1	161	-25	161	-25	1589628	21	1947	1922
miR529b	LOC_Os12g23130.1	158	-23.63	158	-23.63	1589638	21	3732	3157
miR529b	LOC_Os12g23350.1	166	-21.94	166	-21.94	1589662	21	2136	204

miR529b	LOC_Os12g23540.1	153	-20.48	153	-20.48	1589679	21	2328	2303
miR529b	LOC_Os12g24540.1	154	-22.3	154	-22.3	1589771	21	4617	4315
miR529b	LOC_Os12g24770.1	161	-25	161	-25	1589792	21	1521	1496
miR529b	LOC_Os12g25170.1	164	-22.27	164	-22.27	1589831	21	1754	1710
miR529b	LOC_Os12g25690.1	154	-22.32	154	-22.32	1589883	21	1920	32
miR529b	LOC_Os12g26444.1	150	-20.16	150	-20.16	1589962	21	4269	2685
miR529b	LOC_Os12g26890.1	155	-20.72	155	-20.72	1590005	21	435	224
miR529b	LOC_Os12g27490.1	155	-20.37	155	-20.37	1590067	21	4836	3960
miR529b	LOC_Os12g27880.1	167	-21.93	167	-21.93	1590106	21	3846	3363
miR529b	LOC_Os12g28340.1	168	-23.18	168	-23.18	1590148	21	2166	2142
miR529b	LOC_Os12g29060.1	154	-20.29	154	-20.29	1590217	21	1983	14
miR529b	LOC_Os12g29130.1	157	-21.69	157	-21.69	1590224	21	4689	531
miR529b	LOC_Os12g29200.1	161	-25	161	-25	1590231	21	2436	2411
miR529b	LOC_Os12g29380.1	313	-44.17	157	-22.48	1590246	21	4569	531 3998
miR529b	LOC_Os12g29520.1	165	-24.08	165	-24.08	1590259	21	3622	3552
miR529b	LOC_Os12g29520.2	165	-24.08	165	-24.08	1590260	21	3639	3569
miR529b	LOC_Os12g29520.3	165	-24.08	165	-24.08	1590261	21	3619	3549
miR529b	LOC_Os12g29520.4	165	-24.08	165	-24.08	1590262	21	3619	3549
miR529b	LOC_Os12g29520.5	165	-24.08	165	-24.08	1590263	21	3701	3631
miR529b	LOC_Os12g29530.1	174	-24.07	174	-24.07	1590264	21	5167	4765
miR529b	LOC_Os12g29740.1	169	-26.26	169	-26.26	1590289	21	1698	1121
miR529b	LOC_Os12g29820.1	164	-25.78	164	-25.78	1590297	21	1704	173
miR529b	LOC_Os12g30250.1	161	-20.3	161	-20.3	1590341	21	528	40
miR529b	LOC_Os12g30560.1	330	-47.73	168	-24.98	1590373	21	546	94 47
miR529b	LOC_Os12g30590.1	166	-25.36	166	-25.36	1590376	21	3414	40
miR529b	LOC_Os12g31440.1	145	-20.03	145	-20.03	1590453	21	2050	1189
miR529b	LOC_Os12g31860.2	141	-20.65	141	-20.65	1590499	21	3029	2557
miR529b	LOC_Os12g32190.1	155	-21.29	155	-21.29	1590532	21	2325	826
miR529b	LOC_Os12g32190.2	155	-21.29	155	-21.29	1590533	21	2240	826
miR529b	LOC_Os12g33340.1	161	-25	161	-25	1590651	21	2355	2330
miR529b	LOC_Os12g33470.1	174	-25.26	174	-25.26	1590662	21	432	40
miR529b	LOC_Os12g33560.1	161	-24.99	161	-24.99	1590671	21	1776	1751

miR529b	LOC_Os12g34140.1	154	-20.29	154	-20.29	1590740	21	1632	14
miR529b	LOC_Os12g34419.1	161	-21.59	161	-21.59	1590774	21	231	145
miR529b	LOC_Os12g35490.1	157	-20.35	157	-20.35	1590892	21	1906	1089
miR529b	LOC_Os12g35670.1	165	-24.08	165	-24.08	1590916	21	1087	138
miR529b	LOC_Os12g36120.1	166	-21.94	166	-21.94	1590961	21	2007	204
miR529b	LOC_Os12g36180.1	163	-22.18	163	-22.18	1590967	21	3664	1752
miR529b	LOC_Os12g36900.1	165	-22.28	165	-22.28	1591040	21	1561	72
miR529b	LOC_Os12g37720.1	163	-20.14	163	-20.14	1591139	21	4346	1128
miR529b	LOC_Os12g38310.1	164	-20.29	164	-20.29	1591199	21	700	15
miR529b	LOC_Os12g38550.1	167	-21.06	167	-21.06	1591226	21	1410	931
miR529b	LOC_Os12g39630.1	168	-23.18	168	-23.18	1591366	21	2684	2596
miR529b	LOC_Os12g40840.2	166	-27.67	166	-27.67	1591500	21	1554	167
miR529b	LOC_Os12g40840.3	166	-27.67	166	-27.67	1591501	21	1928	167
miR529b	LOC_Os12g41060.1	170	-24.39	170	-24.39	1591524	21	2141	378
miR529b	LOC_Os12g41124.1	328	-45.35	165	-24.08	1591530	21	2710	39 2387
miR529b	LOC_Os12g41680.1	170	-23.69	170	-23.69	1591597	21	1532	115
miR529b	LOC_Os12g41870.1	156	-21.71	156	-21.71	1591622	21	4710	3198
miR529b	LOC_Os12g42220.1	157	-20.48	157	-20.48	1591675	21	1474	1405
miR529b	LOC_Os12g42450.1	159	-21.26	159	-21.26	1591703	21	1044	468
miR529b	LOC_Os12g42500.1	158	-23.18	158	-23.18	1591707	21	741	697
miR529b	LOC_Os12g42900.1	161	-22.2	161	-22.2	1591758	21	1493	332
miR529b	LOC_Os12g43300.1	158	-21	158	-21	1591795	21	1993	1652
miR529b	LOC_Os12g44100.1	160	-20.47	160	-20.47	1591886	21	2301	254
miR529b	ChrUn.fgenesh.mRNA.49	178	-28.3	178	-28.3	1591976	21	2007	1223
miR529b	ChrUn.fgenesh.mRNA.85	167	-22.66	167	-22.66	1592012	21	3369	701
miR529b	ChrSy.fgenesh.mRNA.23	161	-25	161	-25	1592046	21	2427	2402
miR529b	ChrSy.fgenesh.mRNA.48	159	-21.55	159	-21.55	1592071	21	3657	3308
miR5077	LOC_Os01g02060.1	155	-27.64	155	-27.64	1592252	19	2088	561
miR5077	LOC_Os01g02060.2	155	-27.64	155	-27.64	1592253	19	2007	561
miR5077	LOC_Os01g02910.1	156	-24.89	156	-24.89	1592348	19	2195	589
miR5077	LOC_Os01g02980.1	159	-20.82	159	-20.82	1592359	19	1629	313
miR5077	LOC_Os01g03090.1	150	-21.86	150	-21.86	1592374	19	1388	25

miR5077	LOC_Os01g03230.1	161	-27.52	161	-27.52	1592389	19	1554	1081
miR5077	LOC_Os01g03320.1	154	-21.86	154	-21.86	1592398	19	959	219
miR5077	LOC_Os01g03464.1	147	-21.44	147	-21.44	1592412	19	790	158
miR5077	LOC_Os01g03760.1	151	-22.45	151	-22.45	1592440	19	2504	2376
miR5077	LOC_Os01g03760.2	151	-22.45	151	-22.45	1592441	19	2850	2722
miR5077	LOC_Os01g04150.1	156	-25.68	156	-25.68	1592481	19	2829	657
miR5077	LOC_Os01g04220.1	150	-23.46	150	-23.46	1592494	19	1215	570
miR5077	LOC_Os01g05480.3	157	-22.5	157	-22.5	1592634	19	2227	2161
miR5077	LOC_Os01g07930.1	149	-24.68	149	-24.68	1592920	19	1212	990
miR5077	LOC_Os01g08380.1	146	-21.92	146	-21.92	1592969	19	1748	1150
miR5077	LOC_Os01g10090.1	150	-21.86	150	-21.86	1593169	19	1710	913
miR5077	LOC_Os01g10320.1	151	-27.2	151	-27.2	1593200	19	2158	794
miR5077	LOC_Os01g10950.1	156	-23.75	156	-23.75	1593266	19	2424	1879
miR5077	LOC_Os01g11270.1	140	-21.67	140	-21.67	1593297	19	1809	1019
miR5077	LOC_Os01g11700.1	153	-25.26	153	-25.26	1593335	19	1542	1167
miR5077	LOC_Os01g11700.2	153	-25.26	153	-25.26	1593336	19	1625	1250
miR5077	LOC_Os01g12230.1	151	-24.38	151	-24.38	1593394	19	924	739
miR5077	LOC_Os01g13180.1	159	-26.52	159	-26.52	1593520	19	1389	581
miR5077	LOC_Os01g13280.1	149	-21.05	149	-21.05	1593530	19	1721	1448
miR5077	LOC_Os01g14420.1	163	-22.02	163	-22.02	1593658	19	1390	1256
miR5077	LOC_Os01g14720.1	150	-26.12	150	-26.12	1593686	19	1209	1126
miR5077	LOC_Os01g15420.1	148	-27.14	148	-27.14	1593764	19	456	396
miR5077	LOC_Os01g15590.1	157	-25.26	157	-25.26	1593785	19	468	256
miR5077	LOC_Os01g16040.1	158	-29.21	158	-29.21	1593838	19	2029	1720
miR5077	LOC_Os01g18040.1	165	-28.58	165	-28.58	1594015	19	5331	385
miR5077	LOC_Os01g18620.1	156	-25.03	156	-25.03	1594069	19	1869	115
miR5077	LOC_Os01g18670.1	147	-20.54	147	-20.54	1594076	19	3858	3663
miR5077	LOC_Os01g22249.1	147	-22.99	147	-22.99	1594409	19	1393	701
miR5077	LOC_Os01g22280.1	147	-21.31	147	-21.31	1594411	19	1122	634
miR5077	LOC_Os01g23440.1	148	-21.8	148	-21.8	1594521	19	2256	1870
miR5077	LOC_Os01g24550.1	147	-21.94	147	-21.94	1594638	19	1342	757
miR5077	LOC_Os01g25040.1	146	-20.05	146	-20.05	1594695	19	3744	90

miR5077	LOC_Os01g25386.1	148	-20.56	148	-20.56	1594734	19	5280	3057
miR5077	LOC_Os01g29200.1	156	-26.05	156	-26.05	1595075	19	306	68
miR5077	LOC_Os01g29320.1	160	-25.92	160	-25.92	1595087	19	2172	111
miR5077	LOC_Os01g33230.1	146	-21.18	146	-21.18	1595386	19	3167	2833
miR5077	LOC_Os01g33510.1	160	-22.01	160	-22.01	1595409	19	474	387
miR5077	LOC_Os01g35770.1	161	-23.59	161	-23.59	1595646	19	3972	852
miR5077	LOC_Os01g36720.1	145	-21.63	145	-21.63	1595741	19	1572	1039
miR5077	LOC_Os01g38720.1	168	-29.63	168	-29.63	1595970	19	1080	267
miR5077	LOC_Os01g39600.1	141	-22.19	141	-22.19	1596060	19	2905	1888
miR5077	LOC_Os01g39600.2	141	-22.19	141	-22.19	1596061	19	3524	2440
miR5077	LOC_Os01g39690.1	156	-25.23	156	-25.23	1596071	19	1420	1064
miR5077	LOC_Os01g40150.1	163	-28.2	163	-28.2	1596122	19	2991	483
miR5077	LOC_Os01g40480.1	148	-20.56	148	-20.56	1596160	19	3604	1585
miR5077	LOC_Os01g41430.1	163	-29.29	163	-29.29	1596264	19	1964	916
miR5077	LOC_Os01g42780.1	158	-21.08	158	-21.08	1596413	19	1403	1326
miR5077	LOC_Os01g42840.1	151	-21.36	151	-21.36	1596421	19	516	94
miR5077	LOC_Os01g42960.1	158	-22.51	158	-22.51	1596435	19	2853	75
miR5077	LOC_Os01g43350.1	159	-24.11	159	-24.11	1596484	19	2923	774
miR5077	LOC_Os01g45070.1	145	-20.55	145	-20.55	1596627	19	1205	1089
miR5077	LOC_Os01g45070.2	145	-20.55	145	-20.55	1596628	19	887	771
miR5077	LOC_Os01g45110.1	163	-29.29	163	-29.29	1596632	19	1928	927
miR5077	LOC_Os01g45430.1	163	-26.73	163	-26.73	1596663	19	867	191
miR5077	LOC_Os01g47130.1	147	-20.94	147	-20.94	1596849	19	1470	1014
miR5077	LOC_Os01g49680.1	151	-24.08	151	-24.08	1597164	19	2812	277
miR5077	LOC_Os01g50060.1	144	-23.96	144	-23.96	1597206	19	1481	1262
miR5077	LOC_Os01g52480.1	162	-23.99	162	-23.99	1597487	19	1610	787
miR5077	LOC_Os01g52900.1	158	-24.21	158	-24.21	1597535	19	2118	1764
miR5077	LOC_Os01g53090.1	148	-20.56	148	-20.56	1597554	19	1061	615
miR5077	LOC_Os01g53150.1	150	-22.19	150	-22.19	1597559	19	1613	411
miR5077	LOC_Os01g53150.2	150	-22.19	150	-22.19	1597560	19	1549	411
miR5077	LOC_Os01g53480.1	147	-20.49	147	-20.49	1597593	19	2301	1350
miR5077	LOC_Os01g54515.1	152	-22.11	152	-22.11	1597715	19	1965	1324

miR5077	LOC_Os01g55000.1	156	-26.4	156	-26.4	1597769	19	1493	1183
miR5077	LOC_Os01g55610.1	156	-25.6	156	-25.6	1597860	19	2119	1636
miR5077	LOC_Os01g56090.1	144	-20.61	144	-20.61	1597919	19	1005	168
miR5077	LOC_Os01g56640.1	150	-23.81	150	-23.81	1597991	19	2693	2566
miR5077	LOC_Os01g56640.2	150	-23.81	150	-23.81	1597992	19	2898	2771
miR5077	LOC_Os01g56640.3	150	-23.81	150	-23.81	1597993	19	2780	2653
miR5077	LOC_Os01g57170.1	155	-25.78	155	-25.78	1598058	19	660	131
miR5077	LOC_Os01g57510.1	160	-21.5	160	-21.5	1598099	19	2571	314
miR5077	LOC_Os01g58230.1	157	-20.18	157	-20.18	1598188	19	1449	1062
miR5077	LOC_Os01g58240.1	152	-23.62	152	-23.62	1598189	19	2511	630
miR5077	LOC_Os01g58270.1	152	-23.62	152	-23.62	1598190	19	2409	801
miR5077	LOC_Os01g60280.1	150	-20.42	150	-20.42	1598427	19	3072	285
miR5077	LOC_Os01g61010.1	153	-21.62	153	-21.62	1598501	19	2910	2473
miR5077	LOC_Os01g61010.2	153	-21.62	153	-21.62	1598502	19	3248	2811
miR5077	LOC_Os01g61190.1	163	-24.27	163	-24.27	1598520	19	2830	2070
miR5077	LOC_Os01g61200.1	163	-24.27	163	-24.27	1598521	19	1463	703
miR5077	LOC_Os01g61390.1	158	-24.95	158	-24.95	1598541	19	2651	1011
miR5077	LOC_Os01g61460.1	154	-21.82	154	-21.82	1598550	19	2174	926
miR5077	LOC_Os01g62160.1	155	-24.02	155	-24.02	1598656	19	1851	1769
miR5077	LOC_Os01g62244.1	154	-21.2	154	-21.2	1598666	19	1213	1029
miR5077	LOC_Os01g62244.2	154	-21.2	154	-21.2	1598667	19	1128	944
miR5077	LOC_Os01g62870.1	148	-22.87	148	-22.87	1598750	19	1512	543
miR5077	LOC_Os01g63854.1	150	-21.91	150	-21.91	1598863	19	1807	1392
miR5077	LOC_Os01g63970.1	155	-24.33	155	-24.33	1598876	19	1836	1293
miR5077	LOC_Os01g64256.1	148	-20.9	148	-20.9	1598921	19	1998	453
miR5077	LOC_Os01g64810.1	163	-20.99	163	-20.99	1598999	19	2294	2169
miR5077	LOC_Os01g64810.2	163	-20.99	163	-20.99	1599000	19	1971	1846
miR5077	LOC_Os01g64910.1	155	-21.67	155	-21.67	1599010	19	1779	1357
miR5077	LOC_Os01g65260.1	156	-29.02	156	-29.02	1599052	19	1948	1097
miR5077	LOC_Os01g65880.1	150	-22.36	150	-22.36	1599128	19	1303	844
miR5077	LOC_Os01g65880.2	150	-22.36	150	-22.36	1599129	19	1510	1051
miR5077	LOC_Os01g65880.3	150	-22.36	150	-22.36	1599130	19	1736	1277

miR5077	LOC_Os01g66120.1	154	-22.5	154	-22.5	1599158	19	2057	1154
miR5077	LOC_Os01g66120.3	154	-22.5	154	-22.5	1599159	19	2104	1154
miR5077	LOC_Os01g67240.1	151	-22.66	151	-22.66	1599283	19	3423	2479
miR5077	LOC_Os01g67730.1	160	-21.34	160	-21.34	1599353	19	863	419
miR5077	LOC_Os01g71420.1	160	-27.8	160	-27.8	1599750	19	1660	1314
miR5077	LOC_Os01g71690.2	151	-21.45	151	-21.45	1599785	19	2782	653
miR5077	LOC_Os01g71690.3	151	-21.45	151	-21.45	1599786	19	2586	663
miR5077	LOC_Os01g72520.1	147	-20.25	147	-20.25	1599875	19	1955	1383
miR5077	LOC_Os01g73300.1	158	-22.38	158	-22.38	1599969	19	1120	1044
miR5077	LOC_Os01g74020.1	152	-22.3	152	-22.3	1600056	19	1309	820
miR5077	LOC_Os01g74200.1	151	-21.31	151	-21.31	1600076	19	1916	1331
miR5077	LOC_Os01g74260.1	152	-25.26	152	-25.26	1600082	19	5682	2535
miR5077	LOC_Os01g74630.1	147	-20.54	147	-20.54	1600125	19	942	735
miR5077	LOC_Os02g01630.1	155	-28.73	155	-28.73	1600217	19	453	393
miR5077	LOC_Os02g01660.1	155	-28.73	155	-28.73	1600219	19	453	393
miR5077	LOC_Os02g01690.1	155	-28.73	155	-28.73	1600222	19	1665	393
miR5077	LOC_Os02g02820.1	158	-21.34	158	-21.34	1600361	19	2157	1485
miR5077	LOC_Os02g02880.1	151	-20.27	151	-20.27	1600368	19	2131	1907
miR5077	LOC_Os02g02890.1	155	-20.21	155	-20.21	1600369	19	1078	163
miR5077	LOC_Os02g03580.1	147	-21.34	147	-21.34	1600465	19	1729	107
miR5077	LOC_Os02g03580.2	147	-21.34	147	-21.34	1600466	19	1526	107
miR5077	LOC_Os02g03580.3	147	-21.34	147	-21.34	1600467	19	2059	107
miR5077	LOC_Os02g04130.1	145	-20.24	145	-20.24	1600539	19	1079	794
miR5077	LOC_Os02g04540.1	159	-25.59	159	-25.59	1600601	19	2520	2210
miR5077	LOC_Os02g05110.1	150	-23.24	150	-23.24	1600670	19	1728	213
miR5077	LOC_Os02g06090.1	158	-23.42	158	-23.42	1600796	19	3259	3050
miR5077	LOC_Os02g09190.1	155	-21.64	155	-21.64	1601133	19	1561	1229
miR5077	LOC_Os02g09740.1	149	-20.57	149	-20.57	1601192	19	2904	2466
miR5077	LOC_Os02g09740.2	149	-20.57	149	-20.57	1601193	19	2901	2463
miR5077	LOC_Os02g10880.1	163	-26.23	163	-26.23	1601339	19	1437	862
miR5077	LOC_Os02g11665.1	156	-26.05	156	-26.05	1601381	19	4228	1476
miR5077	LOC_Os02g13810.1	158	-20.83	158	-20.83	1601645	19	1308	1033

miR5077	LOC_Os02g13900.1	146	-21.18	146	-21.18	1601656	19	1456	442
miR5077	LOC_Os02g15020.1	148	-22.9	148	-22.9	1601783	19	570	291
miR5077	LOC_Os02g15060.1	154	-23.61	154	-23.61	1601787	19	903	645
miR5077	LOC_Os02g15310.1	148	-21.96	148	-21.96	1601817	19	1269	678
miR5077	LOC_Os02g15310.2	148	-21.96	148	-21.96	1601818	19	2241	678
miR5077	LOC_Os02g15310.3	148	-21.96	148	-21.96	1601819	19	1822	678
miR5077	LOC_Os02g15310.4	148	-21.96	148	-21.96	1601820	19	2071	678
miR5077	LOC_Os02g16040.1	149	-21.61	149	-21.61	1601907	19	1016	43
miR5077	LOC_Os02g16040.2	149	-21.61	149	-21.61	1601908	19	1539	43
miR5077	LOC_Os02g20800.1	152	-20.7	152	-20.7	1602413	19	3732	2505
miR5077	LOC_Os02g22200.1	151	-20.36	151	-20.36	1602575	19	273	174
miR5077	LOC_Os02g22540.1	151	-29.94	151	-29.94	1602614	19	258	72
miR5077	LOC_Os02g25810.1	157	-23.03	157	-23.03	1602869	19	5124	409
miR5077	LOC_Os02g27100.1	148	-21.33	148	-21.33	1603024	19	852	17
miR5077	LOC_Os02g27300.1	168	-30.34	168	-30.34	1603044	19	624	458
miR5077	LOC_Os02g27730.1	147	-20.3	147	-20.3	1603097	19	393	96
miR5077	LOC_Os02g28200.1	163	-26.21	163	-26.21	1603143	19	1607	566
miR5077	LOC_Os02g28240.1	163	-26.21	163	-26.21	1603147	19	1188	375
miR5077	LOC_Os02g31090.1	146	-21.45	146	-21.45	1603456	19	627	105
miR5077	LOC_Os02g32410.1	161	-24.35	161	-24.35	1603548	19	540	318
miR5077	LOC_Os02g32660.1	154	-24.88	154	-24.88	1603585	19	2958	2657
miR5077	LOC_Os02g32660.2	154	-24.88	154	-24.88	1603586	19	2268	1967
miR5077	LOC_Os02g33720.1	153	-24.04	153	-24.04	1603701	19	609	128
miR5077	LOC_Os02g34710.1	164	-33.93	164	-33.93	1603797	19	1354	318
miR5077	LOC_Os02g35600.1	153	-20.51	153	-20.51	1603896	19	1238	734
miR5077	LOC_Os02g36320.1	143	-20.18	143	-20.18	1603979	19	1047	857
miR5077	LOC_Os02g36350.1	153	-23.25	153	-23.25	1603983	19	1701	346
miR5077	LOC_Os02g36350.2	153	-23.25	153	-23.25	1603984	19	1783	345
miR5077	LOC_Os02g38290.1	144	-21.62	144	-21.62	1604228	19	2207	1997
miR5077	LOC_Os02g38290.2	144	-21.62	144	-21.62	1604229	19	2044	1853
miR5077	LOC_Os02g38720.1	154	-20.04	154	-20.04	1604279	19	1146	1014
miR5077	LOC_Os02g38930.1	163	-23.93	163	-23.93	1604305	19	1389	1042

miR5077	LOC_Os02g38940.1	155	-21.64	155	-21.64	1604306	19	1729	1360
miR5077	LOC_Os02g39410.2	156	-23.26	156	-23.26	1604369	19	3697	613
miR5077	LOC_Os02g39410.3	156	-23.26	156	-23.26	1604370	19	3057	1286
miR5077	LOC_Os02g39420.1	155	-24.89	155	-24.89	1604371	19	1173	337
miR5077	LOC_Os02g39590.1	153	-20.38	153	-20.38	1604398	19	1212	22
miR5077	LOC_Os02g40650.1	155	-25.12	155	-25.12	1604536	19	666	385
miR5077	LOC_Os02g41680.1	147	-25.08	147	-25.08	1604599	19	2536	665
miR5077	LOC_Os02g42200.1	148	-20.42	148	-20.42	1604660	19	5793	5718
miR5077	LOC_Os02g42200.3	148	-20.42	148	-20.42	1604661	19	4251	4176
miR5077	LOC_Os02g43410.1	152	-21.23	152	-21.23	1604815	19	2479	610
miR5077	LOC_Os02g43410.2	152	-21.23	152	-21.23	1604816	19	1991	610
miR5077	LOC_Os02g43460.1	155	-21.6	155	-21.6	1604821	19	4320	783
miR5077	LOC_Os02g44490.1	140	-21.12	140	-21.12	1604960	19	2340	2041
miR5077	LOC_Os02g45130.1	147	-21.85	147	-21.85	1605050	19	2664	2500
miR5077	LOC_Os02g45310.1	152	-20.24	152	-20.24	1605072	19	2482	1722
miR5077	LOC_Os02g45930.1	150	-22.06	150	-22.06	1605142	19	1113	605
miR5077	LOC_Os02g46090.1	153	-22.04	153	-22.04	1605159	19	2340	927
miR5077	LOC_Os02g46500.1	165	-26.56	165	-26.56	1605212	19	1490	15
miR5077	LOC_Os02g47200.1	167	-25.36	167	-25.36	1605297	19	1382	361
miR5077	LOC_Os02g48140.1	144	-24.35	144	-24.35	1605421	19	829	7
miR5077	LOC_Os02g48140.2	144	-24.35	144	-24.35	1605422	19	690	7
miR5077	LOC_Os02g48770.1	155	-20.18	155	-20.18	1605503	19	1672	1453
miR5077	LOC_Os02g49520.1	150	-21.91	150	-21.91	1605596	19	4094	3783
miR5077	LOC_Os02g49555.1	153	-22.61	153	-22.61	1605601	19	3162	2422
miR5077	LOC_Os02g50000.1	153	-21.18	153	-21.18	1605665	19	1262	912
miR5077	LOC_Os02g50490.1	149	-23.01	149	-23.01	1605717	19	1981	1189
miR5077	LOC_Os02g51170.1	146	-21	146	-21	1605801	19	525	236
miR5077	LOC_Os02g51710.1	151	-23.27	151	-23.27	1605868	19	1045	298
miR5077	LOC_Os02g51900.1	154	-20.74	154	-20.74	1605897	19	2142	1158
miR5077	LOC_Os02g51910.1	156	-22.25	156	-22.25	1605898	19	2685	1573
miR5077	LOC_Os02g52850.1	295	-42.02	150	-21.86	1606011	19	3127	2014 2590
miR5077	LOC_Os02g52960.1	153	-20.04	153	-20.04	1606028	19	5681	22

miR5077	LOC_Os02g54780.1	156	-21.91	156	-21.91	1606255	19	2267	220
miR5077	LOC_Os02g55900.1	149	-23.43	149	-23.43	1606396	19	828	742
miR5077	LOC_Os02g56910.1	151	-22.45	151	-22.45	1606517	19	1719	1543
miR5077	LOC_Os02g57110.1	155	-21.5	155	-21.5	1606545	19	1563	1142
miR5077	LOC_Os02g57110.2	155	-21.5	155	-21.5	1606546	19	1668	1140
miR5077	LOC_Os03g02700.1	167	-28.66	167	-28.66	1607034	19	2074	1302
miR5077	LOC_Os03g03340.1	150	-26.74	150	-26.74	1607112	19	585	2
miR5077	LOC_Os03g03670.1	153	-25.4	153	-25.4	1607158	19	1434	1057
miR5077	LOC_Os03g03730.1	148	-21.64	148	-21.64	1607170	19	804	339
miR5077	LOC_Os03g04410.1	156	-22.48	156	-22.48	1607244	19	3519	3177
miR5077	LOC_Os03g04550.1	154	-22.26	154	-22.26	1607264	19	1715	787
miR5077	LOC_Os03g04780.1	152	-20.43	152	-20.43	1607293	19	333	165
miR5077	LOC_Os03g07110.1	156	-20.87	156	-20.87	1607582	19	2934	2772
miR5077	LOC_Os03g07110.2	156	-20.87	156	-20.87	1607583	19	2424	2262
miR5077	LOC_Os03g07110.3	156	-20.87	156	-20.87	1607584	19	2374	2212
miR5077	LOC_Os03g07250.1	151	-22.06	151	-22.06	1607607	19	2037	839
miR5077	LOC_Os03g08080.1	148	-21.13	148	-21.13	1607703	19	1133	874
miR5077	LOC_Os03g08090.1	148	-21.13	148	-21.13	1607704	19	1969	359
miR5077	LOC_Os03g08220.1	148	-21.27	148	-21.27	1607722	19	3456	2292
miR5077	LOC_Os03g08310.1	155	-24.89	155	-24.89	1607741	19	1042	642
miR5077	LOC_Os03g08470.1	153	-20.08	153	-20.08	1607761	19	1651	1284
miR5077	LOC_Os03g08470.2	153	-20.08	153	-20.08	1607762	19	1763	1396
miR5077	LOC_Os03g08470.3	153	-20.08	153	-20.08	1607763	19	1676	1309
miR5077	LOC_Os03g08610.1	147	-20.6	147	-20.6	1607783	19	2240	778
miR5077	LOC_Os03g08610.2	147	-20.6	147	-20.6	1607784	19	2244	778
miR5077	LOC_Os03g08770.1	145	-21.76	145	-21.76	1607804	19	1226	51
miR5077	LOC_Os03g08770.2	145	-21.76	145	-21.76	1607805	19	1223	51
miR5077	LOC_Os03g08850.1	149	-20.65	149	-20.65	1607813	19	2299	1418
miR5077	LOC_Os03g09940.1	151	-20.25	151	-20.25	1607889	19	2677	1209
miR5077	LOC_Os03g10100.1	168	-28	168	-28	1607910	19	2256	1572
miR5077	LOC_Os03g11350.1	163	-25.44	163	-25.44	1608058	19	1590	829
miR5077	LOC_Os03g12260.1	156	-22.58	156	-22.58	1608181	19	2021	1603

miR5077	LOC_Os03g12414.1	141	-20.04	141	-20.04	1608205	19	2600	346
miR5077	LOC_Os03g12414.2	141	-20.04	141	-20.04	1608206	19	2675	346
miR5077	LOC_Os03g13080.1	157	-28.03	157	-28.03	1608296	19	1875	363
miR5077	LOC_Os03g15230.1	155	-24.89	155	-24.89	1608559	19	2103	1695
miR5077	LOC_Os03g15660.1	155	-25.6	155	-25.6	1608606	19	1135	396
miR5077	LOC_Os03g15810.1	158	-27.76	158	-27.76	1608629	19	3009	2616
miR5077	LOC_Os03g16120.1	154	-22.59	154	-22.59	1608677	19	1819	1634
miR5077	LOC_Os03g16120.2	154	-22.59	154	-22.59	1608678	19	1810	1625
miR5077	LOC_Os03g16120.3	154	-22.59	154	-22.59	1608679	19	1857	1672
miR5077	LOC_Os03g16120.4	154	-22.59	154	-22.59	1608680	19	1898	1713
miR5077	LOC_Os03g16120.5	154	-22.59	154	-22.59	1608681	19	1939	1754
miR5077	LOC_Os03g16860.1	307	-45.18	156	-25.02	1608778	19	2268	368 1563
miR5077	LOC_Os03g16860.2	307	-45.18	156	-25.02	1608779	19	2168	368 1563
miR5077	LOC_Os03g17370.1	148	-22.93	148	-22.93	1608847	19	2298	1343
miR5077	LOC_Os03g19180.1	155	-23.8	155	-23.8	1609058	19	612	33
miR5077	LOC_Os03g19300.1	164	-26.3	164	-26.3	1609076	19	1891	29
miR5077	LOC_Os03g19870.1	154	-20.4	154	-20.4	1609143	19	1866	212
miR5077	LOC_Os03g19870.2	154	-20.4	154	-20.4	1609144	19	1858	212
miR5077	LOC_Os03g19870.3	154	-20.4	154	-20.4	1609145	19	1938	212
miR5077	LOC_Os03g21480.1	154	-24.67	154	-24.67	1609333	19	1088	117
miR5077	LOC_Os03g21540.1	154	-24.67	154	-24.67	1609344	19	3264	1085
miR5077	LOC_Os03g22940.1	147	-21.82	147	-21.82	1609528	19	852	499
miR5077	LOC_Os03g25280.1	159	-22.33	159	-22.33	1609715	19	1236	365
miR5077	LOC_Os03g25430.1	150	-26.12	150	-26.12	1609737	19	1480	1028
miR5077	LOC_Os03g25840.1	156	-22	156	-22	1609779	19	993	669
miR5077	LOC_Os03g25869.1	156	-22	156	-22	1609781	19	1925	1565
miR5077	LOC_Os03g26430.1	168	-30.72	168	-30.72	1609833	19	1381	631
miR5077	LOC_Os03g29850.1	152	-21.8	152	-21.8	1610147	19	1404	966
miR5077	LOC_Os03g30160.1	160	-29.24	160	-29.24	1610182	19	894	372
miR5077	LOC_Os03g32490.1	151	-27.96	151	-27.96	1610416	19	1230	415
miR5077	LOC_Os03g32730.1	160	-31.35	160	-31.35	1610439	19	1191	521
miR5077	LOC_Os03g33110.1	152	-21.58	152	-21.58	1610478	19	1113	195

miR5077	LOC_Os03g33230.1	160	-31.35	160	-31.35	1610488	19	4896	419
miR5077	LOC_Os03g33580.2	163	-26.53	163	-26.53	1610520	19	1368	240
miR5077	LOC_Os03g33700.1	160	-31.35	160	-31.35	1610532	19	1821	554
miR5077	LOC_Os03g34040.1	150	-23.02	150	-23.02	1610564	19	1413	1030
miR5077	LOC_Os03g34040.2	150	-23.02	150	-23.02	1610565	19	1296	913
miR5077	LOC_Os03g34040.3	150	-23.02	150	-23.02	1610566	19	1540	1157
miR5077	LOC_Os03g34240.1	155	-22.95	155	-22.95	1610583	19	2727	2351
miR5077	LOC_Os03g35390.1	165	-25.18	165	-25.18	1610606	19	5727	553
miR5077	LOC_Os03g35690.1	147	-20.49	147	-20.49	1610629	19	2397	633
miR5077	LOC_Os03g36390.1	151	-29.94	151	-29.94	1610688	19	450	261
miR5077	LOC_Os03g36520.1	156	-26.05	156	-26.05	1610698	19	264	68
miR5077	LOC_Os03g37680.1	155	-24.89	155	-24.89	1610801	19	696	539
miR5077	LOC_Os03g37700.1	157	-21.98	157	-21.98	1610803	19	603	34
miR5077	LOC_Os03g38764.1	150	-20.01	150	-20.01	1610918	19	3690	1555
miR5077	LOC_Os03g40560.1	154	-21.22	154	-21.22	1611091	19	3519	1333
miR5077	LOC_Os03g44740.1	149	-20.64	149	-20.64	1611510	19	1835	350
miR5077	LOC_Os03g45344.1	155	-28.39	155	-28.39	1611579	19	999	851
miR5077	LOC_Os03g46470.1	167	-33.1	167	-33.1	1611695	19	1508	1127
miR5077	LOC_Os03g47810.1	153	-20.61	153	-20.61	1611840	19	1920	1808
miR5077	LOC_Os03g48320.1	160	-23.55	160	-23.55	1611898	19	3540	3481
miR5077	LOC_Os03g49160.1	145	-20.24	145	-20.24	1612000	19	621	387
miR5077	LOC_Os03g49990.1	157	-28.48	157	-28.48	1612109	19	2555	1533
miR5077	LOC_Os03g50920.1	155	-27.1	155	-27.1	1612210	19	819	67
miR5077	LOC_Os03g52010.1	164	-33.03	164	-33.03	1612334	19	2149	1026
miR5077	LOC_Os03g52160.1	141	-20.47	141	-20.47	1612351	19	1742	301
miR5077	LOC_Os03g52510.1	152	-21.58	152	-21.58	1612390	19	1584	195
miR5077	LOC_Os03g52760.1	161	-23.02	161	-23.02	1612423	19	1801	630
miR5077	LOC_Os03g53070.1	158	-20.68	158	-20.68	1612456	19	1331	93
miR5077	LOC_Os03g53410.1	145	-23.22	145	-23.22	1612497	19	1622	1170
miR5077	LOC_Os03g55800.1	153	-24.21	153	-24.21	1612739	19	1999	1740
miR5077	LOC_Os03g56180.1	149	-24.75	149	-24.75	1612783	19	1767	408
miR5077	LOC_Os03g56840.1	155	-24.55	155	-24.55	1612874	19	1748	1437

miR5077	LOC_Os03g56840.2	155	-24.55	155	-24.55	1612875	19	1828	1517
miR5077	LOC_Os03g58130.1	151	-22.57	151	-22.57	1613047	19	1491	35
miR5077	LOC_Os03g58170.1	152	-21.09	152	-21.09	1613052	19	1604	1023
miR5077	LOC_Os03g58170.2	152	-21.09	152	-21.09	1613053	19	1215	634
miR5077	LOC_Os03g58660.1	145	-20.95	145	-20.95	1613106	19	4338	2814
miR5077	LOC_Os03g64170.1	160	-22.32	160	-22.32	1613775	19	1697	1015
miR5077	LOC_Os03g64170.2	160	-22.32	160	-22.32	1613776	19	1305	1015
miR5077	LOC_Os04g01140.1	304	-46.42	153	-23.81	1613829	19	1928	1535 1513
miR5077	LOC_Os04g01140.2	304	-46.42	153	-23.81	1613830	19	1442	1049 1027
miR5077	LOC_Os04g02370.1	151	-22.09	151	-22.09	1613966	19	4635	297
miR5077	LOC_Os04g04750.1	147	-20.82	147	-20.82	1614204	19	1017	108
miR5077	LOC_Os04g04940.1	160	-31.35	160	-31.35	1614221	19	3972	473
miR5077	LOC_Os04g08776.1	151	-29.94	151	-29.94	1614590	19	1263	624
miR5077	LOC_Os04g08828.1	155	-21.82	155	-21.82	1614602	19	3346	1006
miR5077	LOC_Os04g09260.1	154	-26.75	154	-26.75	1614606	19	1774	1261
miR5077	LOC_Os04g09590.1	144	-25.83	144	-25.83	1614639	19	1296	624
miR5077	LOC_Os04g09780.1	156	-25.94	156	-25.94	1614655	19	837	270
miR5077	LOC_Os04g09920.1	155	-21.64	155	-21.64	1614669	19	1818	1431
miR5077	LOC_Os04g11090.1	156	-29.45	156	-29.45	1614782	19	3876	557
miR5077	LOC_Os04g11300.1	162	-27.33	162	-27.33	1614804	19	2487	285
miR5077	LOC_Os04g11620.1	165	-28	165	-28	1614835	19	5544	556
miR5077	LOC_Os04g12140.1	155	-20.22	155	-20.22	1614889	19	3585	82
miR5077	LOC_Os04g14140.1	164	-22.97	164	-22.97	1615071	19	627	291
miR5077	LOC_Os04g14810.1	148	-21.42	148	-21.42	1615142	19	888	218
miR5077	LOC_Os04g18330.1	153	-22.57	153	-22.57	1615469	19	4131	478
miR5077	LOC_Os04g21320.1	142	-20.4	142	-20.4	1615759	19	1199	380
miR5077	LOC_Os04g21320.3	144	-20.4	144	-20.4	1615760	19	1160	341
miR5077	LOC_Os04g22060.1	154	-22.47	154	-22.47	1615833	19	4245	39
miR5077	LOC_Os04g22210.1	148	-20.1	148	-20.1	1615848	19	834	466
miR5077	LOC_Os04g22520.1	150	-23.15	150	-23.15	1615879	19	642	289
miR5077	LOC_Os04g22970.1	160	-31.35	160	-31.35	1615920	19	1797	554
miR5077	LOC_Os04g23220.1	159	-29.37	159	-29.37	1615942	19	3488	2962

miR5077	LOC_Os04g23510.1	153	-21.68	153	-21.68	1615968	19	360	63
miR5077	LOC_Os04g23890.1	161	-23.33	161	-23.33	1615997	19	3330	1717
miR5077	LOC_Os04g23890.2	161	-23.33	161	-23.33	1615998	19	3248	1805
miR5077	LOC_Os04g23890.3	161	-23.33	161	-23.33	1615999	19	3418	1805
miR5077	LOC_Os04g23890.4	161	-23.33	161	-23.33	1616000	19	3285	1811
miR5077	LOC_Os04g24750.1	145	-20.02	145	-20.02	1616090	19	993	475
miR5077	LOC_Os04g24850.1	300	-51.48	156	-27.65	1616100	19	1514	569 1103
miR5077	LOC_Os04g25440.1	156	-27.65	156	-27.65	1616156	19	2092	992
miR5077	LOC_Os04g26520.1	150	-20.42	150	-20.42	1616270	19	264	63
miR5077	LOC_Os04g26570.1	149	-21.07	149	-21.07	1616275	19	2676	2040
miR5077	LOC_Os04g28440.1	156	-22.41	156	-22.41	1616451	19	588	83
miR5077	LOC_Os04g31610.1	161	-27.52	161	-27.52	1616764	19	1524	568
miR5077	LOC_Os04g31890.1	150	-23.15	150	-23.15	1616787	19	648	295
miR5077	LOC_Os04g33370.1	151	-22.45	151	-22.45	1616963	19	1981	1281
miR5077	LOC_Os04g34140.1	147	-24.23	147	-24.23	1617047	19	1773	662
miR5077	LOC_Os04g34600.1	144	-24.47	144	-24.47	1617093	19	1150	474
miR5077	LOC_Os04g35130.1	159	-22.8	159	-22.8	1617146	19	1737	458
miR5077	LOC_Os04g37460.1	158	-25.36	158	-25.36	1617307	19	1925	1744
miR5077	LOC_Os04g37570.1	154	-24.64	154	-24.64	1617319	19	1501	1080
miR5077	LOC_Os04g37790.1	150	-21.85	150	-21.85	1617342	19	2534	728
miR5077	LOC_Os04g37790.2	150	-21.85	150	-21.85	1617343	19	2331	728
miR5077	LOC_Os04g37970.1	156	-24.91	156	-24.91	1617365	19	1563	193
miR5077	LOC_Os04g38060.1	154	-23.68	154	-23.68	1617372	19	1506	309
miR5077	LOC_Os04g38070.1	143	-21.33	143	-21.33	1617373	19	2577	1861
miR5077	LOC_Os04g38860.1	143	-20.61	143	-20.61	1617463	19	1660	918
miR5077	LOC_Os04g38860.2	143	-20.61	143	-20.61	1617464	19	1731	989
miR5077	LOC_Os04g38920.1	150	-24.76	150	-24.76	1617477	19	1416	715
miR5077	LOC_Os04g39030.1	146	-21.1	146	-21.1	1617492	19	1779	341
miR5077	LOC_Os04g39240.1	159	-24.24	159	-24.24	1617517	19	2644	301
miR5077	LOC_Os04g40150.1	158	-20.3	158	-20.3	1617624	19	2661	764
miR5077	LOC_Os04g40150.2	158	-20.3	158	-20.3	1617625	19	2596	764
miR5077	LOC_Os04g40860.1	155	-22.78	155	-22.78	1617715	19	2466	2310

miR5077	LOC_Os04g41470.1	157	-25.24	157	-25.24	1617791	19	2439	241
miR5077	LOC_Os04g41470.2	157	-25.24	157	-25.24	1617792	19	2520	241
miR5077	LOC_Os04g42670.1	158	-30.17	158	-30.17	1617935	19	2621	2132
miR5077	LOC_Os04g42740.1	156	-22.42	156	-22.42	1617942	19	2382	869
miR5077	LOC_Os04g42860.1	157	-20.26	157	-20.26	1617961	19	2004	84
miR5077	LOC_Os04g43080.1	147	-20.31	147	-20.31	1617990	19	438	304
miR5077	LOC_Os04g43210.1	156	-22.72	156	-22.72	1618006	19	2323	1036
miR5077	LOC_Os04g43210.2	156	-22.72	156	-22.72	1618007	19	2487	1036
miR5077	LOC_Os04g43210.3	156	-22.72	156	-22.72	1618008	19	2318	1036
miR5077	LOC_Os04g43340.1	151	-20.01	151	-20.01	1618026	19	3630	3481
miR5077	LOC_Os04g43800.1	147	-25.08	147	-25.08	1618078	19	2654	777
miR5077	LOC_Os04g43910.1	162	-26.73	162	-26.73	1618089	19	2579	375
miR5077	LOC_Os04g44320.1	147	-21.03	147	-21.03	1618134	19	2568	124
miR5077	LOC_Os04g44670.1	152	-22.57	152	-22.57	1618183	19	1939	1603
miR5077	LOC_Os04g44900.1	154	-25.78	154	-25.78	1618209	19	2558	624
miR5077	LOC_Os04g45520.1	157	-25.79	157	-25.79	1618286	19	1187	535
miR5077	LOC_Os04g45950.2	152	-21.78	152	-21.78	1618333	19	2182	2105
miR5077	LOC_Os04g46220.1	157	-22.69	157	-22.69	1618365	19	2768	516
miR5077	LOC_Os04g46660.1	148	-26.07	148	-26.07	1618419	19	1284	615
miR5077	LOC_Os04g46940.1	142	-21.41	142	-21.41	1618459	19	3310	65
miR5077	LOC_Os04g47390.1	156	-22.4	156	-22.4	1618513	19	1104	739
miR5077	LOC_Os04g47420.1	299	-42.08	151	-21.95	1618519	19	1773	1284 789
miR5077	LOC_Os04g48070.1	157	-21.82	157	-21.82	1618599	19	3110	2320
miR5077	LOC_Os04g48070.2	157	-21.82	157	-21.82	1618600	19	3089	2299
miR5077	LOC_Os04g48070.3	157	-21.82	157	-21.82	1618601	19	2246	1559
miR5077	LOC_Os04g48580.1	143	-20.34	143	-20.34	1618657	19	831	719
miR5077	LOC_Os04g49370.1	300	-47.81	150	-25.75	1618744	19	1437	982 1345
miR5077	LOC_Os04g49500.1	156	-23.67	156	-23.67	1618758	19	3369	686
miR5077	LOC_Os04g52800.1	144	-24.05	144	-24.05	1619130	19	1020	966
miR5077	LOC_Os04g54420.1	155	-23.62	155	-23.62	1619332	19	2238	159
miR5077	LOC_Os04g54860.1	153	-23.39	153	-23.39	1619385	19	2545	923
miR5077	LOC_Os04g54870.1	153	-23.39	153	-23.39	1619386	19	3347	37

miR5077	LOC_Os04g54870.2	153	-23.39	153	-23.39	1619387	19	2109	37
miR5077	LOC_Os04g55130.1	158	-26.66	158	-26.66	1619414	19	1500	1348
miR5077	LOC_Os04g55770.1	147	-20.24	147	-20.24	1619488	19	1998	1914
miR5077	LOC_Os04g56290.1	152	-21.64	152	-21.64	1619549	19	2214	404
miR5077	LOC_Os04g56290.2	152	-21.64	152	-21.64	1619550	19	2642	404
miR5077	LOC_Os04g56290.5	152	-21.64	152	-21.64	1619551	19	1569	404
miR5077	LOC_Os04g56290.6	152	-21.64	152	-21.64	1619552	19	3880	404
miR5077	LOC_Os04g56360.1	151	-22	151	-22	1619559	19	1268	933
miR5077	LOC_Os04g56910.1	162	-29.95	162	-29.95	1619628	19	2006	1421
miR5077	LOC_Os04g57100.1	152	-21.58	152	-21.58	1619654	19	2370	651
miR5077	LOC_Os04g57180.1	157	-26.35	157	-26.35	1619665	19	2727	941
miR5077	LOC_Os04g57850.1	153	-23.21	153	-23.21	1619768	19	2333	1470
miR5077	LOC_Os04g58280.1	145	-22.79	145	-22.79	1619816	19	2620	1606
miR5077	LOC_Os04g58280.2	145	-22.79	145	-22.79	1619817	19	2409	1395
miR5077	LOC_Os04g59590.1	146	-21.47	146	-21.47	1619980	19	2574	2007
miR5077	LOC_Os04g59590.2	146	-21.47	146	-21.47	1619981	19	2155	2026
miR5077	LOC_Os04g59590.3	146	-21.47	146	-21.47	1619982	19	2136	2008
miR5077	LOC_Os05g01660.1	159	-22.27	159	-22.27	1620102	19	2553	1935
miR5077	LOC_Os05g02980.1	156	-23.77	156	-23.77	1620262	19	1247	1010
miR5077	LOC_Os05g03110.1	159	-23.43	159	-23.43	1620281	19	1485	1055
miR5077	LOC_Os05g03190.1	156	-22.72	156	-22.72	1620291	19	2329	1618
miR5077	LOC_Os05g04020.1	141	-25.29	141	-25.29	1620385	19	1677	1455
miR5077	LOC_Os05g04760.1	147	-20.15	147	-20.15	1620471	19	339	4
miR5077	LOC_Os05g04930.1	156	-25.03	156	-25.03	1620486	19	1335	25
miR5077	LOC_Os05g06640.1	146	-21.1	146	-21.1	1620687	19	1632	930
miR5077	LOC_Os05g07310.1	145	-20.95	145	-20.95	1620775	19	5265	3354
miR5077	LOC_Os05g08080.1	152	-20.96	152	-20.96	1620858	19	3201	786
miR5077	LOC_Os05g08640.1	154	-26.75	154	-26.75	1620930	19	1908	1312
miR5077	LOC_Os05g09650.1	151	-22.41	151	-22.41	1621049	19	871	151
miR5077	LOC_Os05g10210.1	156	-21.28	156	-21.28	1621063	19	1172	250
miR5077	LOC_Os05g10700.1	154	-20.39	154	-20.39	1621118	19	3114	832
miR5077	LOC_Os05g11780.1	148	-20.6	148	-20.6	1621237	19	1473	203

miR5077	LOC_Os05g13530.1	155	-22.99	155	-22.99	1621375	19	378	284
miR5077	LOC_Os05g13920.1	160	-31.35	160	-31.35	1621416	19	3969	554
miR5077	LOC_Os05g14640.1	165	-28.58	165	-28.58	1621491	19	1686	49
miR5077	LOC_Os05g14820.1	306	-49.84	155	-25.56	1621509	19	1371	841 614
miR5077	LOC_Os05g15820.1	156	-26.05	156	-26.05	1621616	19	2861	2603
miR5077	LOC_Os05g18750.1	154	-20.23	154	-20.23	1621914	19	1021	207
miR5077	LOC_Os05g18890.1	156	-26.05	156	-26.05	1621931	19	378	68
miR5077	LOC_Os05g19080.1	152	-22.15	152	-22.15	1621950	19	1305	194
miR5077	LOC_Os05g23970.1	154	-20.39	154	-20.39	1622373	19	2106	292
miR5077	LOC_Os05g25220.1	159	-22.68	159	-22.68	1622510	19	1767	1296
miR5077	LOC_Os05g25390.1	152	-21.87	152	-21.87	1622528	19	2325	1054
miR5077	LOC_Os05g25580.1	150	-20.86	150	-20.86	1622550	19	1245	402
miR5077	LOC_Os05g25600.1	145	-20.95	145	-20.95	1622551	19	2375	1418
miR5077	LOC_Os05g25620.1	160	-28.05	160	-28.05	1622553	19	643	164
miR5077	LOC_Os05g27880.1	155	-25.03	155	-25.03	1622782	19	2895	455
miR5077	LOC_Os05g27880.2	155	-25.03	155	-25.03	1622783	19	2937	455
miR5077	LOC_Os05g27880.3	155	-25.03	155	-25.03	1622784	19	2830	455
miR5077	LOC_Os05g31120.1	149	-20.38	149	-20.38	1623106	19	1158	510
miR5077	LOC_Os05g31460.1	144	-20.12	144	-20.12	1623140	19	708	668
miR5077	LOC_Os05g31740.1	150	-20.93	150	-20.93	1623169	19	1943	1173
miR5077	LOC_Os05g32070.1	144	-20.04	144	-20.04	1623202	19	1228	477
miR5077	LOC_Os05g32500.1	152	-22.59	152	-22.59	1623252	19	3036	2652
miR5077	LOC_Os05g32500.2	152	-22.59	152	-22.59	1623253	19	2839	2455
miR5077	LOC_Os05g33880.1	155	-24.89	155	-24.89	1623412	19	975	1
miR5077	LOC_Os05g33880.2	155	-24.89	155	-24.89	1623413	19	984	1
miR5077	LOC_Os05g34000.1	155	-22.41	155	-22.41	1623424	19	2754	177
miR5077	LOC_Os05g34150.1	143	-21.43	143	-21.43	1623440	19	1356	848
miR5077	LOC_Os05g34350.1	152	-20.7	152	-20.7	1623466	19	3492	1989
miR5077	LOC_Os05g34800.1	143	-22.31	143	-22.31	1623519	19	2246	1396
miR5077	LOC_Os05g35650.1	141	-21.04	141	-21.04	1623619	19	2038	1724
miR5077	LOC_Os05g35650.2	141	-21.04	141	-21.04	1623620	19	2150	1836
miR5077	LOC_Os05g36340.1	151	-20.8	151	-20.8	1623703	19	857	643

miR5077	LOC_Os05g38320.1	151	-27.65	151	-27.65	1623890	19	1491	998
miR5077	LOC_Os05g38730.1	152	-23.6	152	-23.6	1623949	19	3489	3316
miR5077	LOC_Os05g38730.2	152	-23.6	152	-23.6	1623950	19	3529	3356
miR5077	LOC_Os05g38770.1	148	-27.32	148	-27.32	1623954	19	1994	1318
miR5077	LOC_Os05g39220.1	307	-47.23	161	-25.59	1624008	19	1390	970 1203
miR5077	LOC_Os05g39560.1	150	-21.86	150	-21.86	1624049	19	1809	1008
miR5077	LOC_Os05g40580.1	150	-21.86	150	-21.86	1624171	19	1167	930
miR5077	LOC_Os05g40610.1	148	-20.4	148	-20.4	1624174	19	1101	891
miR5077	LOC_Os05g42060.1	155	-24.89	155	-24.89	1624346	19	1467	495
miR5077	LOC_Os05g42150.1	166	-32.24	166	-32.24	1624354	19	2481	1506
miR5077	LOC_Os05g44100.1	158	-21.64	158	-21.64	1624521	19	3503	65
miR5077	LOC_Os05g44200.1	155	-21.5	155	-21.5	1624533	19	1460	1047
miR5077	LOC_Os05g44330.1	157	-23.52	157	-23.52	1624552	19	2335	247
miR5077	LOC_Os05g44330.2	157	-23.52	157	-23.52	1624553	19	2415	247
miR5077	LOC_Os05g44330.3	157	-23.52	157	-23.52	1624554	19	1586	247
miR5077	LOC_Os05g44330.4	157	-23.52	157	-23.52	1624555	19	1256	247
miR5077	LOC_Os05g44330.5	157	-23.52	157	-23.52	1624556	19	2320	247
miR5077	LOC_Os05g45540.1	152	-24.6	152	-24.6	1624700	19	339	196
miR5077	LOC_Os05g45720.1	153	-21.31	153	-21.31	1624718	19	3413	24
miR5077	LOC_Os05g45720.2	153	-21.31	153	-21.31	1624719	19	3282	19
miR5077	LOC_Os05g45720.3	153	-21.31	153	-21.31	1624720	19	3316	19
miR5077	LOC_Os05g45720.4	153	-21.31	153	-21.31	1624721	19	3329	19
miR5077	LOC_Os05g47940.1	156	-25.03	156	-25.03	1624937	19	1533	855
miR5077	LOC_Os05g48040.1	155	-22.24	155	-22.24	1624946	19	2046	1615
miR5077	LOC_Os05g48040.2	155	-22.24	155	-22.24	1624947	19	2579	2148
miR5077	LOC_Os05g48040.3	155	-22.24	155	-22.24	1624948	19	2962	2611
miR5077	LOC_Os05g48040.4	155	-22.24	155	-22.24	1624949	19	2930	2499
miR5077	LOC_Os05g48330.1	155	-24.04	155	-24.04	1624984	19	1986	876
miR5077	LOC_Os05g48930.1	148	-27.07	148	-27.07	1625072	19	945	51
miR5077	LOC_Os05g49070.1	157	-24.73	157	-24.73	1625091	19	1442	660
miR5077	LOC_Os05g49160.1	163	-25.17	163	-25.17	1625104	19	943	500
miR5077	LOC_Os05g49160.2	163	-25.17	163	-25.17	1625105	19	902	500

miR5077	LOC_Os05g49160.3	163	-25.17	163	-25.17	1625106	19	948	500
miR5077	LOC_Os05g49710.1	154	-21.22	154	-21.22	1625174	19	3705	1786
miR5077	LOC_Os05g49840.1	154	-25.86	154	-25.86	1625191	19	2119	1174
miR5077	LOC_Os05g50000.1	302	-50.26	151	-25.13	1625217	19	1209	477 1083
miR5077	LOC_Os05g50580.1	147	-21.53	147	-21.53	1625280	19	1938	833
miR5077	LOC_Os06g01610.1	164	-30.82	164	-30.82	1625541	19	1359	512
miR5077	LOC_Os06g03940.1	164	-24.92	164	-24.92	1625813	19	1934	1
miR5077	LOC_Os06g04150.1	146	-21.38	146	-21.38	1625837	19	1201	1071
miR5077	LOC_Os06g05424.1	155	-21.25	155	-21.25	1625995	19	832	626
miR5077	LOC_Os06g05430.1	152	-22.56	152	-22.56	1625996	19	590	214
miR5077	LOC_Os06g05520.1	149	-20.2	149	-20.2	1626005	19	1555	1516
miR5077	LOC_Os06g06040.1	151	-21.04	151	-21.04	1626078	19	1140	1052
miR5077	LOC_Os06g06040.2	151	-21.04	151	-21.04	1626079	19	1053	965
miR5077	LOC_Os06g06040.3	151	-21.04	151	-21.04	1626080	19	1212	1124
miR5077	LOC_Os06g06040.4	151	-21.04	151	-21.04	1626081	19	1340	1252
miR5077	LOC_Os06g06040.5	151	-21.04	151	-21.04	1626082	19	1815	1727
miR5077	LOC_Os06g06040.6	151	-21.04	151	-21.04	1626083	19	2116	2028
miR5077	LOC_Os06g06040.7	151	-21.04	151	-21.04	1626084	19	756	668
miR5077	LOC_Os06g06490.1	154	-22.98	154	-22.98	1626142	19	2163	1231
miR5077	LOC_Os06g06490.2	154	-22.98	154	-22.98	1626143	19	2105	1173
miR5077	LOC_Os06g07360.1	147	-21.9	147	-21.9	1626239	19	507	160
miR5077	LOC_Os06g08660.1	148	-22.3	148	-22.3	1626378	19	3557	3180
miR5077	LOC_Os06g08810.1	151	-20.77	151	-20.77	1626396	19	1564	1433
miR5077	LOC_Os06g09210.1	146	-21.59	146	-21.59	1626451	19	1614	844
miR5077	LOC_Os06g10355.1	159	-22.76	159	-22.76	1626586	19	1534	807
miR5077	LOC_Os06g10930.1	151	-25.81	151	-25.81	1626648	19	1921	1055
miR5077	LOC_Os06g10950.1	151	-25.81	151	-25.81	1626650	19	1776	899
miR5077	LOC_Os06g11130.1	308	-50.06	156	-29.36	1626668	19	1561	547 1114
miR5077	LOC_Os06g12190.1	159	-22.3	159	-22.3	1626796	19	1152	564
miR5077	LOC_Os06g13710.1	150	-22.81	150	-22.81	1626969	19	495	305
miR5077	LOC_Os06g14180.1	301	-41.9	151	-20.99	1627018	19	966	559 333
miR5077	LOC_Os06g14260.1	157	-25.68	157	-25.68	1627026	19	2169	474

miR5077	LOC_Os06g15660.1	149	-21.34	149	-21.34	1627178	19	1745	1422
miR5077	LOC_Os06g16070.1	156	-22.05	156	-22.05	1627216	19	3285	2367
miR5077	LOC_Os06g17460.1	155	-21.5	155	-21.5	1627360	19	636	114
miR5077	LOC_Os06g19010.1	150	-24.24	150	-24.24	1627462	19	2896	2519
miR5077	LOC_Os06g19400.1	157	-22.68	157	-22.68	1627499	19	4232	669
miR5077	LOC_Os06g20150.1	163	-26.86	163	-26.86	1627581	19	1263	370
miR5077	LOC_Os06g20670.1	155	-23.95	155	-23.95	1627638	19	1287	697
miR5077	LOC_Os06g21950.1	143	-20.89	143	-20.89	1627774	19	1989	1083
miR5077	LOC_Os06g22290.1	150	-22.98	150	-22.98	1627814	19	2830	530
miR5077	LOC_Os06g22340.1	161	-26.73	161	-26.73	1627819	19	1810	1450
miR5077	LOC_Os06g22440.1	151	-20.15	151	-20.15	1627832	19	1464	728
miR5077	LOC_Os06g22440.2	151	-20.15	151	-20.15	1627833	19	1572	728
miR5077	LOC_Os06g22600.1	143	-22.4	143	-22.4	1627849	19	2265	168
miR5077	LOC_Os06g22770.1	147	-20.49	147	-20.49	1627867	19	3630	717
miR5077	LOC_Os06g23200.1	147	-20.08	147	-20.08	1627913	19	1857	1324
miR5077	LOC_Os06g24110.1	165	-28	165	-28	1628009	19	4035	556
miR5077	LOC_Os06g24650.1	156	-26.05	156	-26.05	1628063	19	372	68
miR5077	LOC_Os06g25360.1	155	-22.95	155	-22.95	1628134	19	1497	1121
miR5077	LOC_Os06g27370.1	156	-24.49	156	-24.49	1628232	19	1646	656
miR5077	LOC_Os06g27560.1	167	-26.12	167	-26.12	1628250	19	2026	1132
miR5077	LOC_Os06g27600.1	152	-21.7	152	-21.7	1628254	19	1326	216
miR5077	LOC_Os06g28310.1	143	-25.67	143	-25.67	1628334	19	5730	5458
miR5077	LOC_Os06g29470.1	155	-21.05	155	-21.05	1628458	19	975	252
miR5077	LOC_Os06g29580.1	155	-21.11	155	-21.11	1628468	19	2493	107
miR5077	LOC_Os06g30960.1	152	-24.6	152	-24.6	1628613	19	864	323
miR5077	LOC_Os06g34240.1	154	-21.22	154	-21.22	1628928	19	2583	112
miR5077	LOC_Os06g34430.1	144	-22.92	144	-22.92	1628947	19	1065	434
miR5077	LOC_Os06g34860.1	150	-22.76	150	-22.76	1628989	19	504	244
miR5077	LOC_Os06g34870.1	299	-47.08	154	-23.94	1628990	19	1074	1051 789
miR5077	LOC_Os06g35650.1	157	-25.79	157	-25.79	1629071	19	1819	1586
miR5077	LOC_Os06g36270.1	154	-20.78	154	-20.78	1629133	19	4377	2292
miR5077	LOC_Os06g36270.2	154	-20.78	154	-20.78	1629134	19	4514	2291

miR5077	LOC_Os06g36270.3	154	-20.78	154	-20.78	1629135	19	4393	2291
miR5077	LOC_Os06g36270.4	154	-20.78	154	-20.78	1629136	19	3762	2292
miR5077	LOC_Os06g36270.5	154	-20.78	154	-20.78	1629137	19	4256	2292
miR5077	LOC_Os06g37080.1	152	-25.45	152	-25.45	1629223	19	2113	1484
miR5077	LOC_Os06g39990.1	155	-22.95	155	-22.95	1629537	19	1791	113
miR5077	LOC_Os06g42030.1	162	-29.85	162	-29.85	1629772	19	5864	2360
miR5077	LOC_Os06g43780.1	158	-23	158	-23	1629967	19	1788	1578
miR5077	LOC_Os06g44270.1	151	-21.41	151	-21.41	1630028	19	1806	115
miR5077	LOC_Os06g45460.1	154	-26.23	154	-26.23	1630169	19	1224	72
miR5077	LOC_Os06g45930.1	147	-20.37	147	-20.37	1630227	19	936	40
miR5077	LOC_Os06g48540.1	151	-29.94	151	-29.94	1630544	19	609	549
miR5077	LOC_Os06g49170.1	150	-23.02	150	-23.02	1630628	19	2223	577
miR5077	LOC_Os06g50679.1	151	-29.83	151	-29.83	1630821	19	4659	1583
miR5077	LOC_Os06g50733.1	150	-22.06	150	-22.06	1630825	19	270	226
miR5077	LOC_Os07g01550.1	143	-25.57	143	-25.57	1631010	19	4010	3679
miR5077	LOC_Os07g01560.1	143	-25.57	143	-25.57	1631011	19	2657	1298
miR5077	LOC_Os07g01560.2	143	-25.57	143	-25.57	1631012	19	2120	1298
miR5077	LOC_Os07g02030.1	153	-22.46	153	-22.46	1631067	19	1215	187
miR5077	LOC_Os07g02490.1	150	-20.62	150	-20.62	1631114	19	5400	2252
miR5077	LOC_Os07g02580.1	155	-22.18	155	-22.18	1631123	19	615	268
miR5077	LOC_Os07g03140.1	156	-21.44	156	-21.44	1631191	19	1762	804
miR5077	LOC_Os07g03810.1	149	-25.38	149	-25.38	1631255	19	2875	1183
miR5077	LOC_Os07g05365.1	160	-21	160	-21	1631417	19	654	103
miR5077	LOC_Os07g05750.1	144	-21.15	144	-21.15	1631464	19	5363	2028
miR5077	LOC_Os07g06810.1	148	-21.32	148	-21.32	1631577	19	852	294
miR5077	LOC_Os07g06960.1	147	-20.15	147	-20.15	1631594	19	1995	943
miR5077	LOC_Os07g08930.1	159	-22.29	159	-22.29	1631813	19	870	282
miR5077	LOC_Os07g10830.1	162	-20.34	162	-20.34	1632025	19	1930	1
miR5077	LOC_Os07g13190.1	153	-20.04	153	-20.04	1632272	19	2829	1925
miR5077	LOC_Os07g14050.1	160	-31.35	160	-31.35	1632355	19	2514	365
miR5077	LOC_Os07g14440.1	153	-23.19	153	-23.19	1632406	19	471	72
miR5077	LOC_Os07g14590.1	147	-20.84	147	-20.84	1632426	19	1602	1498

miR5077	LOC_Os07g16670.1	152	-20.7	152	-20.7	1632628	19	1269	828
miR5077	LOC_Os07g19390.1	158	-22.94	158	-22.94	1632897	19	954	358
miR5077	LOC_Os07g23550.1	148	-22.02	148	-22.02	1633183	19	1220	1141
miR5077	LOC_Os07g23550.2	148	-22.02	148	-22.02	1633184	19	1111	1032
miR5077	LOC_Os07g24920.1	156	-26.05	156	-26.05	1633325	19	396	68
miR5077	LOC_Os07g25010.1	155	-23.99	155	-23.99	1633342	19	2886	2008
miR5077	LOC_Os07g25120.1	156	-26.05	156	-26.05	1633352	19	546	68
miR5077	LOC_Os07g25840.1	163	-27.62	163	-27.62	1633425	19	3042	2579
miR5077	LOC_Os07g25940.1	150	-22.42	150	-22.42	1633435	19	552	339
miR5077	LOC_Os07g25980.1	155	-22.94	155	-22.94	1633439	19	549	339
miR5077	LOC_Os07g27590.1	148	-20.94	148	-20.94	1633613	19	897	448
miR5077	LOC_Os07g28750.1	155	-23.99	155	-23.99	1633736	19	1257	541
miR5077	LOC_Os07g29950.1	151	-22	151	-22	1633863	19	189	93
miR5077	LOC_Os07g30030.1	152	-21.58	152	-21.58	1633871	19	1206	203
miR5077	LOC_Os07g30774.1	150	-23.6	150	-23.6	1633947	19	3259	451
miR5077	LOC_Os07g31090.1	148	-27.14	148	-27.14	1633984	19	477	417
miR5077	LOC_Os07g31160.1	152	-21.58	152	-21.58	1633990	19	2163	528
miR5077	LOC_Os07g31750.1	160	-27.91	160	-27.91	1634056	19	1365	435
miR5077	LOC_Os07g31770.1	160	-30.12	160	-30.12	1634058	19	1726	778
miR5077	LOC_Os07g32010.1	148	-25.7	148	-25.7	1634077	19	1951	1278
miR5077	LOC_Os07g33180.1	140	-20.04	140	-20.04	1634205	19	552	214
miR5077	LOC_Os07g34130.1	156	-26.57	156	-26.57	1634303	19	2103	1265
miR5077	LOC_Os07g35140.1	151	-20.24	151	-20.24	1634415	19	2575	524
miR5077	LOC_Os07g35140.2	151	-20.24	151	-20.24	1634416	19	1999	524
miR5077	LOC_Os07g35330.1	150	-21.85	150	-21.85	1634436	19	2324	314
miR5077	LOC_Os07g35540.1	151	-21.51	151	-21.51	1634464	19	2347	527
miR5077	LOC_Os07g36570.1	150	-23.64	150	-23.64	1634583	19	3238	1461
miR5077	LOC_Os07g36690.1	154	-23.87	154	-23.87	1634595	19	3005	1441
miR5077	LOC_Os07g36700.1	154	-23.87	154	-23.87	1634596	19	2583	1271
miR5077	LOC_Os07g37310.1	151	-20.24	151	-20.24	1634666	19	1386	770
miR5077	LOC_Os07g37320.1	152	-24.01	152	-24.01	1634667	19	1998	1543
miR5077	LOC_Os07g37420.1	168	-30.35	168	-30.35	1634676	19	1091	103

miR5077	LOC_Os07g38130.1	151	-20.8	151	-20.8	1634760	19	1456	289
miR5077	LOC_Os07g38250.1	167	-26.31	167	-26.31	1634771	19	1992	1484
miR5077	LOC_Os07g38810.1	453	-66.23	155	-24.89	1634834	19	2423	1584 717 2222
miR5077	LOC_Os07g38820.1	149	-22.24	149	-22.24	1634835	19	2136	409
miR5077	LOC_Os07g39400.1	315	-41.74	163	-21.32	1634902	19	2250	347 282
miR5077	LOC_Os07g40480.1	146	-20.11	146	-20.11	1635026	19	2547	2087
miR5077	LOC_Os07g43700.1	152	-20.08	152	-20.08	1635401	19	1608	475
miR5077	LOC_Os07g43700.2	152	-20.08	152	-20.08	1635402	19	2109	475
miR5077	LOC_Os07g44170.1	156	-24.5	156	-24.5	1635456	19	2118	281
miR5077	LOC_Os07g44600.1	159	-26.93	159	-26.93	1635501	19	357	163
miR5077	LOC_Os07g45840.1	150	-23.15	150	-23.15	1635632	19	426	73
miR5077	LOC_Os07g45860.1	150	-23.15	150	-23.15	1635634	19	426	73
miR5077	LOC_Os07g46000.1	154	-21.67	154	-21.67	1635648	19	1689	472
miR5077	LOC_Os07g46240.1	153	-22.3	153	-22.3	1635672	19	2351	1847
miR5077	LOC_Os07g46420.1	158	-24.01	158	-24.01	1635697	19	1344	38
miR5077	LOC_Os07g46670.2	159	-26.52	159	-26.52	1635730	19	1785	1706
miR5077	LOC_Os07g46670.3	159	-26.52	159	-26.52	1635731	19	1782	1703
miR5077	LOC_Os07g46860.1	155	-22.61	155	-22.61	1635766	19	864	702
miR5077	LOC_Os07g47150.1	168	-27.86	168	-27.86	1635805	19	1626	935
miR5077	LOC_Os07g47150.2	168	-27.86	168	-27.86	1635806	19	1703	1012
miR5077	LOC_Os07g47370.1	153	-21.96	153	-21.96	1635839	19	2282	1902
miR5077	LOC_Os07g47390.1	144	-23.63	144	-23.63	1635841	19	784	473
miR5077	LOC_Os07g48750.1	150	-23.79	150	-23.79	1636023	19	3212	2557
miR5077	LOC_Os07g49030.1	153	-25.45	153	-25.45	1636062	19	2829	2027
miR5077	LOC_Os07g49030.2	153	-25.45	153	-25.45	1636063	19	2595	2027
miR5077	LOC_Os07g49320.1	144	-20.22	144	-20.22	1636097	19	4116	3642
miR5077	LOC_Os08g01480.1	152	-23.94	152	-23.94	1636195	19	1460	982
miR5077	LOC_Os08g01490.1	157	-25.03	157	-25.03	1636196	19	2232	1598
miR5077	LOC_Os08g01930.1	160	-27.33	160	-27.33	1636249	19	2497	171
miR5077	LOC_Os08g02550.1	304	-43.03	154	-22.74	1636316	19	1794	10 59
miR5077	LOC_Os08g03200.1	151	-23.1	151	-23.1	1636389	19	5565	3362
miR5077	LOC_Os08g03220.1	152	-20.54	152	-20.54	1636391	19	5520	2689

miR5077	LOC_Os08g03380.1	155	-21.5	155	-21.5	1636413	19	1438	1207
miR5077	LOC_Os08g03530.1	150	-24.19	150	-24.19	1636433	19	1149	675
miR5077	LOC_Os08g03880.1	150	-20.22	150	-20.22	1636475	19	4604	167
miR5077	LOC_Os08g04580.1	159	-24.79	159	-24.79	1636557	19	1567	1250
miR5077	LOC_Os08g05360.1	145	-20.95	145	-20.95	1636644	19	5301	3477
miR5077	LOC_Os08g05610.1	161	-30	161	-30	1636671	19	1404	1096
miR5077	LOC_Os08g10570.1	154	-21.82	154	-21.82	1637221	19	2127	1283
miR5077	LOC_Os08g10570.2	154	-21.82	154	-21.82	1637222	19	2120	1276
miR5077	LOC_Os08g10790.1	151	-29.94	151	-29.94	1637246	19	444	192
miR5077	LOC_Os08g12540.1	160	-31.35	160	-31.35	1637425	19	1812	434
miR5077	LOC_Os08g12550.1	160	-31.35	160	-31.35	1637426	19	1509	521
miR5077	LOC_Os08g12800.1	153	-22.45	153	-22.45	1637457	19	2290	1410
miR5077	LOC_Os08g13410.1	150	-20.7	150	-20.7	1637522	19	1905	1299
miR5077	LOC_Os08g14450.1	147	-20.27	147	-20.27	1637630	19	2786	1473
miR5077	LOC_Os08g17520.1	144	-21.12	144	-21.12	1637968	19	669	223
miR5077	LOC_Os08g19440.1	147	-20.49	147	-20.49	1638122	19	1419	633
miR5077	LOC_Os08g19750.1	156	-26.05	156	-26.05	1638156	19	1827	69
miR5077	LOC_Os08g23240.1	157	-25.76	157	-25.76	1638480	19	1719	336
miR5077	LOC_Os08g23250.1	163	-27.65	163	-27.65	1638481	19	1557	1008
miR5077	LOC_Os08g24680.1	157	-24.07	157	-24.07	1638631	19	2880	621
miR5077	LOC_Os08g25380.1	146	-20.11	146	-20.11	1638699	19	3990	3962
miR5077	LOC_Os08g29480.1	157	-24.02	157	-24.02	1639128	19	2415	696
miR5077	LOC_Os08g30020.1	160	-23.43	160	-23.43	1639181	19	2135	586
miR5077	LOC_Os08g30020.2	160	-23.43	160	-23.43	1639182	19	2089	540
miR5077	LOC_Os08g30020.3	160	-23.43	160	-23.43	1639183	19	2039	493
miR5077	LOC_Os08g30020.5	160	-23.43	160	-23.43	1639185	19	1698	493
miR5077	LOC_Os08g30020.6	160	-23.43	160	-23.43	1639186	19	1375	493
miR5077	LOC_Os08g30020.8	160	-23.43	160	-23.43	1639187	19	567	435
miR5077	LOC_Os08g31630.1	158	-22.6	158	-22.6	1639352	19	2698	34
miR5077	LOC_Os08g31630.2	158	-22.6	158	-22.6	1639353	19	2891	25
miR5077	LOC_Os08g32550.1	155	-21.03	155	-21.03	1639454	19	681	381
miR5077	LOC_Os08g33820.1	156	-20.49	156	-20.49	1639604	19	1282	788

miR5077	LOC_Os08g34210.1	159	-20.13	159	-20.13	1639649	19	2023	20
miR5077	LOC_Os08g34210.2	159	-20.13	159	-20.13	1639650	19	2490	20
miR5077	LOC_Os08g34550.1	150	-22.39	150	-22.39	1639685	19	2344	740
miR5077	LOC_Os08g35090.1	160	-27.54	160	-27.54	1639748	19	2809	194
miR5077	LOC_Os08g35510.1	153	-20.91	153	-20.91	1639796	19	1563	120
miR5077	LOC_Os08g36010.1	159	-22.92	159	-22.92	1639853	19	1566	751
miR5077	LOC_Os08g36380.1	145	-20.39	145	-20.39	1639893	19	888	112
miR5077	LOC_Os08g36480.1	147	-21.73	147	-21.73	1639906	19	3236	2560
miR5077	LOC_Os08g36820.1	143	-22.43	143	-22.43	1639940	19	1245	938
miR5077	LOC_Os08g36970.1	150	-20.48	150	-20.48	1639957	19	1004	555
miR5077	LOC_Os08g38092.1	140	-20.21	140	-20.21	1640099	19	1629	1356
miR5077	LOC_Os08g38480.1	147	-22.2	147	-22.2	1640147	19	1915	316
miR5077	LOC_Os08g39030.1	164	-26.96	164	-26.96	1640218	19	1384	23
miR5077	LOC_Os08g39270.1	150	-22.06	150	-22.06	1640249	19	1279	769
miR5077	LOC_Os08g39810.1	144	-21.64	144	-21.64	1640311	19	3127	2971
miR5077	LOC_Os08g40130.1	152	-26.82	152	-26.82	1640351	19	275	121
miR5077	LOC_Os08g40620.1	144	-20.13	144	-20.13	1640409	19	3411	169
miR5077	LOC_Os08g40620.2	144	-20.13	144	-20.13	1640410	19	3483	169
miR5077	LOC_Os08g40870.1	155	-22.56	155	-22.56	1640443	19	1182	943
miR5077	LOC_Os08g41240.1	152	-20.7	152	-20.7	1640490	19	1050	685
miR5077	LOC_Os08g42320.1	151	-20.21	151	-20.21	1640610	19	2599	1724
miR5077	LOC_Os08g42570.1	150	-21.12	150	-21.12	1640650	19	1625	1131
miR5077	LOC_Os08g43180.1	144	-22.45	144	-22.45	1640726	19	3397	2018
miR5077	LOC_Os08g44460.1	160	-24.95	160	-24.95	1640880	19	664	1
miR5077	LOC_Os08g44460.2	158	-22.78	158	-22.78	1640881	19	525	1
miR5077	LOC_Os08g44460.3	160	-24.95	160	-24.95	1640882	19	582	45
miR5077	LOC_Os09g03180.1	156	-26.05	156	-26.05	1641230	19	2883	68
miR5077	LOC_Os09g03240.1	156	-26.05	156	-26.05	1641236	19	414	86
miR5077	LOC_Os09g04820.1	155	-21.63	155	-21.63	1641399	19	1770	391
miR5077	LOC_Os09g04830.1	165	-28	165	-28	1641400	19	3432	523
miR5077	LOC_Os09g06150.1	165	-28	165	-28	1641435	19	6033	553
miR5077	LOC_Os09g07000.1	140	-20.04	140	-20.04	1641519	19	2373	172

miR5077	LOC_Os09g07120.1	147	-21.16	147	-21.16	1641532	19	3754	3373
miR5077	LOC_Os09g07120.2	147	-21.16	147	-21.16	1641533	19	3650	3269
miR5077	LOC_Os09g07540.1	155	-24.89	155	-24.89	1641583	19	2370	2175
miR5077	LOC_Os09g07990.1	144	-22.56	144	-22.56	1641635	19	1587	1565
miR5077	LOC_Os09g10940.1	153	-22.58	153	-22.58	1641940	19	1466	680
miR5077	LOC_Os09g10950.1	153	-21.43	153	-21.43	1641941	19	1665	461
miR5077	LOC_Os09g13150.1	155	-22.95	155	-22.95	1642166	19	4374	2282
miR5077	LOC_Os09g14550.1	163	-22.67	163	-22.67	1642313	19	1069	183
miR5077	LOC_Os09g15950.1	160	-22.35	160	-22.35	1642471	19	2292	303
miR5077	LOC_Os09g16240.1	153	-24.09	153	-24.09	1642499	19	2088	252
miR5077	LOC_Os09g19400.1	159	-21.31	159	-21.31	1642765	19	1101	641
miR5077	LOC_Os09g19880.1	156	-23.58	156	-23.58	1642813	19	2559	512
miR5077	LOC_Os09g19940.1	150	-22.06	150	-22.06	1642820	19	2148	73
miR5077	LOC_Os09g19940.2	150	-22.06	150	-22.06	1642821	19	2416	73
miR5077	LOC_Os09g19970.1	147	-20.99	147	-20.99	1642826	19	1265	551
miR5077	LOC_Os09g20970.1	145	-20.25	145	-20.25	1642941	19	2064	1547
miR5077	LOC_Os09g20980.1	141	-20.09	141	-20.09	1642942	19	1734	1395
miR5077	LOC_Os09g21310.1	148	-21.58	148	-21.58	1642977	19	1854	609
miR5077	LOC_Os09g21840.1	150	-20.22	150	-20.22	1643033	19	3765	167
miR5077	LOC_Os09g22320.1	154	-20.29	154	-20.29	1643078	19	4746	612
miR5077	LOC_Os09g23350.1	148	-20.13	148	-20.13	1643137	19	4231	2574
miR5077	LOC_Os09g24070.1	140	-20.04	140	-20.04	1643214	19	447	3
miR5077	LOC_Os09g24700.1	168	-29.07	168	-29.07	1643282	19	4185	2714
miR5077	LOC_Os09g25000.1	161	-25.65	161	-25.65	1643318	19	1549	757
miR5077	LOC_Os09g25580.1	145	-20.66	145	-20.66	1643379	19	1943	1525
miR5077	LOC_Os09g25650.1	150	-20.54	150	-20.54	1643391	19	3732	3604
miR5077	LOC_Os09g25890.1	151	-21.18	151	-21.18	1643413	19	3052	1368
miR5077	LOC_Os09g25890.2	151	-21.18	151	-21.18	1643414	19	3040	1368
miR5077	LOC_Os09g26300.1	151	-25.4	151	-25.4	1643457	19	1259	643
miR5077	LOC_Os09g26310.1	151	-26.85	151	-26.85	1643458	19	1647	1036
miR5077	LOC_Os09g26340.1	161	-27.33	161	-27.33	1643461	19	1022	864
miR5077	LOC_Os09g26350.1	161	-27.33	161	-27.33	1643462	19	1620	864

miR5077	LOC_Os09g26480.1	288	-43.86	145	-22.37	1643478	19	798	278 380
miR5077	LOC_Os09g27320.1	165	-23.37	165	-23.37	1643578	19	1523	252
miR5077	LOC_Os09g27910.1	152	-22.45	152	-22.45	1643661	19	1553	184
miR5077	LOC_Os09g27960.1	150	-20.28	150	-20.28	1643667	19	1036	737
miR5077	LOC_Os09g27960.2	150	-20.28	150	-20.28	1643668	19	561	262
miR5077	LOC_Os09g29160.1	156	-25.5	156	-25.5	1643802	19	1603	1053
miR5077	LOC_Os09g30160.1	161	-25.8	161	-25.8	1643923	19	965	913
miR5077	LOC_Os09g30462.1	151	-20.7	151	-20.7	1643968	19	904	452
miR5077	LOC_Os09g31380.1	144	-21.64	144	-21.64	1644033	19	1197	1087
miR5077	LOC_Os09g32690.2	160	-21.79	160	-21.79	1644171	19	1916	1671
miR5077	LOC_Os09g32780.1	145	-20.95	145	-20.95	1644182	19	3841	2434
miR5077	LOC_Os09g33980.1	160	-23.94	160	-23.94	1644299	19	4160	51
miR5077	LOC_Os09g36540.1	143	-22.16	143	-22.16	1644504	19	5037	4199
miR5077	LOC_Os09g36600.1	152	-23.32	152	-23.32	1644509	19	2647	1053
miR5077	LOC_Os09g36600.2	152	-23.32	152	-23.32	1644510	19	3728	2134
miR5077	LOC_Os09g37040.1	160	-21.12	160	-21.12	1644565	19	2494	1960
miR5077	LOC_Os09g37300.1	148	-24.72	148	-24.72	1644593	19	2809	669
miR5077	LOC_Os09g37610.1	146	-20.12	146	-20.12	1644629	19	2454	578
miR5077	LOC_Os09g37670.1	145	-20.62	145	-20.62	1644636	19	708	574
miR5077	LOC_Os09g37690.1	154	-23.52	154	-23.52	1644638	19	1584	397
miR5077	LOC_Os10g02100.1	148	-22.88	148	-22.88	1645032	19	1965	442
miR5077	LOC_Os10g02100.2	148	-22.88	148	-22.88	1645033	19	1974	442
miR5077	LOC_Os10g05020.1	157	-23.34	157	-23.34	1645329	19	1856	1450
miR5077	LOC_Os10g07470.1	149	-21.52	149	-21.52	1645570	19	1644	1497
miR5077	LOC_Os10g10700.1	151	-20.96	151	-20.96	1645888	19	1236	627
miR5077	LOC_Os10g11954.1	151	-20.93	151	-20.93	1646007	19	4698	1847
miR5077	LOC_Os10g13610.1	155	-22.95	155	-22.95	1646113	19	5208	1856
miR5077	LOC_Os10g14795.1	156	-29.14	156	-29.14	1646188	19	4161	89
miR5077	LOC_Os10g15340.1	311	-42.42	158	-22.16	1646229	19	2487	2194 99
miR5077	LOC_Os10g16460.1	152	-22.43	152	-22.43	1646260	19	3195	333
miR5077	LOC_Os10g17030.1	154	-21.22	154	-21.22	1646313	19	3000	112
miR5077	LOC_Os10g17610.1	154	-21.22	154	-21.22	1646366	19	4104	1756

miR5077	LOC_Os10g18150.1	152	-22.69	152	-22.69	1646413	19	2396	1264
miR5077	LOC_Os10g18360.1	165	-28	165	-28	1646430	19	3798	556
miR5077	LOC_Os10g18470.1	160	-27.16	160	-27.16	1646442	19	555	108
miR5077	LOC_Os10g19904.1	151	-22.22	151	-22.22	1646542	19	234	86
miR5077	LOC_Os10g20180.1	152	-20.7	152	-20.7	1646574	19	1938	684
miR5077	LOC_Os10g21690.1	152	-22.28	152	-22.28	1646761	19	621	59
miR5077	LOC_Os10g24050.1	154	-21.61	154	-21.61	1646967	19	1239	918
miR5077	LOC_Os10g24110.1	155	-22.95	155	-22.95	1646975	19	2037	1406
miR5077	LOC_Os10g25890.1	160	-31.35	160	-31.35	1647148	19	3879	185
miR5077	LOC_Os10g26030.1	151	-21.89	151	-21.89	1647161	19	3519	1714
miR5077	LOC_Os10g27030.1	158	-25.85	158	-25.85	1647266	19	2607	2085
miR5077	LOC_Os10g27040.1	160	-31.35	160	-31.35	1647267	19	3783	554
miR5077	LOC_Os10g28390.1	156	-22.92	156	-22.92	1647368	19	261	114
miR5077	LOC_Os10g30530.1	156	-20.72	156	-20.72	1647583	19	2560	1370
miR5077	LOC_Os10g32170.1	154	-21.19	154	-21.19	1647750	19	2118	135
miR5077	LOC_Os10g32640.1	155	-20.29	155	-20.29	1647785	19	2733	1677
miR5077	LOC_Os10g33240.1	155	-25.22	155	-25.22	1647849	19	3308	2847
miR5077	LOC_Os10g33370.1	149	-20.87	149	-20.87	1647866	19	3562	2121
miR5077	LOC_Os10g33670.1	158	-24.74	158	-24.74	1647898	19	1600	1136
miR5077	LOC_Os10g33820.1	147	-21.42	147	-21.42	1647920	19	1557	826
miR5077	LOC_Os10g34320.1	149	-20.39	149	-20.39	1647978	19	2242	1773
miR5077	LOC_Os10g34340.1	162	-28.99	162	-28.99	1647980	19	2103	1748
miR5077	LOC_Os10g35260.1	156	-22.54	156	-22.54	1648085	19	1554	1342
miR5077	LOC_Os10g35500.1	152	-22.09	152	-22.09	1648116	19	1611	780
miR5077	LOC_Os10g35500.2	152	-22.09	152	-22.09	1648117	19	2232	780
miR5077	LOC_Os10g35850.1	150	-21.37	150	-21.37	1648162	19	1198	1090
miR5077	LOC_Os10g38040.1	152	-26.51	152	-26.51	1648385	19	1204	444
miR5077	LOC_Os10g38090.1	148	-21.8	148	-21.8	1648391	19	2016	518
miR5077	LOC_Os10g38640.1	150	-21.81	150	-21.81	1648440	19	714	145
miR5077	LOC_Os10g39010.1	158	-22.53	158	-22.53	1648477	19	4606	3760
miR5077	LOC_Os10g39010.2	158	-22.53	158	-22.53	1648478	19	4201	3749
miR5077	LOC_Os10g39010.3	158	-22.53	158	-22.53	1648479	19	4317	3865

miR5077	LOC_Os10g39010.4	158	-22.53	158	-22.53	1648480	19	2875	2509
miR5077	LOC_Os10g39520.1	150	-21.85	150	-21.85	1648543	19	2075	615
miR5077	LOC_Os10g40620.1	152	-22.55	152	-22.55	1648689	19	1825	805
miR5077	LOC_Os11g02680.1	151	-29.94	151	-29.94	1649195	19	465	405
miR5077	LOC_Os11g03420.1	153	-23.96	153	-23.96	1649274	19	744	130
miR5077	LOC_Os11g04480.1	154	-25.68	154	-25.68	1649404	19	1337	854
miR5077	LOC_Os11g05394.1	163	-26.62	163	-26.62	1649512	19	2739	1977
miR5077	LOC_Os11g05394.2	163	-26.62	163	-26.62	1649513	19	2835	1964
miR5077	LOC_Os11g05530.1	160	-27.33	160	-27.33	1649529	19	1079	311
miR5077	LOC_Os11g05820.1	153	-21.35	153	-21.35	1649561	19	3102	881
miR5077	LOC_Os11g06020.1	147	-21.94	147	-21.94	1649587	19	2578	621
miR5077	LOC_Os11g06370.1	147	-20.79	147	-20.79	1649627	19	1518	918
miR5077	LOC_Os11g06690.1	141	-20.6	141	-20.6	1649668	19	2555	46
miR5077	LOC_Os11g07040.1	159	-27.5	159	-27.5	1649715	19	2622	1688
miR5077	LOC_Os11g07190.1	157	-21.69	157	-21.69	1649736	19	801	74
miR5077	LOC_Os11g10970.1	152	-21.83	152	-21.83	1650142	19	3300	3165
miR5077	LOC_Os11g11810.1	162	-23.95	162	-23.95	1650230	19	4244	4140
miR5077	LOC_Os11g13720.1	148	-20.09	148	-20.09	1650368	19	693	294
miR5077	LOC_Os11g13750.1	152	-21.32	152	-21.32	1650371	19	836	414
miR5077	LOC_Os11g14020.1	305	-46.17	155	-24.32	1650408	19	1960	1670 1753
miR5077	LOC_Os11g17650.1	152	-23.6	152	-23.6	1650751	19	564	81
miR5077	LOC_Os11g19520.1	160	-31.35	160	-31.35	1650931	19	4879	660
miR5077	LOC_Os11g22210.1	152	-21.58	152	-21.58	1651103	19	546	276
miR5077	LOC_Os11g24150.1	152	-20.7	152	-20.7	1651262	19	4116	2538
miR5077	LOC_Os11g26240.1	153	-22.57	153	-22.57	1651454	19	594	79
miR5077	LOC_Os11g27730.1	165	-28.19	165	-28.19	1651598	19	1931	1559
miR5077	LOC_Os11g27730.2	165	-28.19	165	-28.19	1651599	19	1855	1483
miR5077	LOC_Os11g28104.1	153	-21.36	153	-21.36	1651634	19	2311	2223
miR5077	LOC_Os11g28240.1	161	-25.2	161	-25.2	1651645	19	2829	556
miR5077	LOC_Os11g28530.1	159	-26.01	159	-26.01	1651679	19	2816	714
miR5077	LOC_Os11g28530.2	159	-26.01	159	-26.01	1651680	19	2967	714
miR5077	LOC_Os11g28530.3	159	-26.01	159	-26.01	1651681	19	2693	717

miR5077	LOC_Os11g29530.1	152	-20.7	152	-20.7	1651781	19	3651	1959
miR5077	LOC_Os11g29620.1	155	-21.42	155	-21.42	1651789	19	585	108
miR5077	LOC_Os11g29880.1	155	-22.95	155	-22.95	1651818	19	4284	2081
miR5077	LOC_Os11g31220.1	145	-20.95	145	-20.95	1651941	19	4158	2538
miR5077	LOC_Os11g34150.1	163	-24.72	163	-24.72	1652211	19	1193	1137
miR5077	LOC_Os11g34150.2	163	-24.72	163	-24.72	1652212	19	2849	2793
miR5077	LOC_Os11g34150.3	163	-24.72	163	-24.72	1652213	19	3077	3021
miR5077	LOC_Os11g36110.1	150	-21.85	150	-21.85	1652434	19	3402	60
miR5077	LOC_Os11g37210.1	159	-26.19	159	-26.19	1652545	19	931	684
miR5077	LOC_Os11g37660.1	150	-24.28	150	-24.28	1652589	19	2978	2356
miR5077	LOC_Os11g38050.1	160	-26.31	160	-26.31	1652635	19	1907	1379
miR5077	LOC_Os11g40430.1	163	-22.08	163	-22.08	1652894	19	1416	312
miR5077	LOC_Os11g42480.1	154	-24.08	154	-24.08	1653113	19	1329	219
miR5077	LOC_Os11g44850.1	155	-24.89	155	-24.89	1653361	19	840	471
miR5077	LOC_Os11g44860.1	155	-20.49	155	-20.49	1653362	19	2007	866
miR5077	LOC_Os11g44950.1	143	-21.23	143	-21.23	1653375	19	2881	2738
miR5077	LOC_Os11g44950.2	143	-21.23	143	-21.23	1653376	19	2839	2692
miR5077	LOC_Os11g45295.1	147	-23.16	147	-23.16	1653413	19	840	228
miR5077	LOC_Os11g47650.1	159	-21.12	159	-21.12	1653627	19	1830	1377
miR5077	LOC_Os11g47650.2	159	-21.12	159	-21.12	1653628	19	1911	1458
miR5077	LOC_Os12g01574.1	155	-21.22	155	-21.22	1653757	19	891	718
miR5077	LOC_Os12g03110.1	153	-23.96	153	-23.96	1653954	19	742	130
miR5077	LOC_Os12g03554.1	143	-20.24	143	-20.24	1654002	19	1584	355
miR5077	LOC_Os12g04240.1	154	-23.96	154	-23.96	1654089	19	1344	862
miR5077	LOC_Os12g04240.2	154	-23.96	154	-23.96	1654090	19	1927	1445
miR5077	LOC_Os12g05640.1	156	-25.39	156	-25.39	1654247	19	2827	2553
miR5077	LOC_Os12g06080.1	151	-25.38	151	-25.38	1654295	19	834	362
miR5077	LOC_Os12g06410.1	150	-22.88	150	-22.88	1654331	19	1314	633
miR5077	LOC_Os12g06810.1	152	-24.35	152	-24.35	1654378	19	2932	2034
miR5077	LOC_Os12g06810.2	152	-24.35	152	-24.35	1654379	19	2440	1525
miR5077	LOC_Os12g06810.4	152	-24.35	152	-24.35	1654381	19	2901	2003
miR5077	LOC_Os12g07280.1	158	-24.51	158	-24.51	1654433	19	2196	1202

miR5077	LOC_Os12g07840.1	155	-21.51	155	-21.51	1654493	19	3569	1071
miR5077	LOC_Os12g08440.1	152	-21.58	152	-21.58	1654565	19	1428	702
miR5077	LOC_Os12g09680.1	155	-21.51	155	-21.51	1654696	19	1341	163
miR5077	LOC_Os12g11250.1	145	-20.06	145	-20.06	1654860	19	1191	324
miR5077	LOC_Os12g12290.1	143	-20.18	143	-20.18	1654959	19	1728	766
miR5077	LOC_Os12g12350.1	151	-29.94	151	-29.94	1654965	19	456	396
miR5077	LOC_Os12g14920.1	152	-25.4	152	-25.4	1655233	19	921	74
miR5077	LOC_Os12g16900.1	150	-24.62	150	-24.62	1655438	19	3042	413
miR5077	LOC_Os12g17450.1	152	-20.73	152	-20.73	1655494	19	3162	1410
miR5077	LOC_Os12g18410.1	152	-24.01	152	-24.01	1655598	19	1164	691
miR5077	LOC_Os12g18410.2	152	-24.01	152	-24.01	1655599	19	1009	631
miR5077	LOC_Os12g18980.1	156	-20.99	156	-20.99	1655659	19	510	116
miR5077	LOC_Os12g23280.1	155	-21.16	155	-21.16	1655991	19	1365	710
miR5077	LOC_Os12g23280.2	155	-21.16	155	-21.16	1655992	19	1323	668
miR5077	LOC_Os12g23280.3	155	-21.16	155	-21.16	1655993	19	1176	521
miR5077	LOC_Os12g23280.4	155	-21.16	155	-21.16	1655994	19	1320	665
miR5077	LOC_Os12g25310.1	150	-21.85	150	-21.85	1656182	19	360	299
miR5077	LOC_Os12g27110.1	157	-23.27	157	-23.27	1656369	19	459	201
miR5077	LOC_Os12g34210.1	145	-20.95	145	-20.95	1657087	19	5544	2373
miR5077	LOC_Os12g35000.1	141	-20.93	141	-20.93	1657177	19	3810	3769
miR5077	LOC_Os12g35364.1	155	-22.55	155	-22.55	1657215	19	486	153
miR5077	LOC_Os12g37070.1	159	-28.83	159	-28.83	1657396	19	423	329
miR5077	LOC_Os12g37419.1	151	-23.66	151	-23.66	1657435	19	2931	2657
miR5077	LOC_Os12g37580.1	144	-20.64	144	-20.64	1657459	19	3516	361
miR5077	LOC_Os12g38150.1	153	-22.27	153	-22.27	1657520	19	1203	1063
miR5077	LOC_Os12g38610.1	298	-45.95	151	-23.62	1657570	19	2596	991 2307
miR5077	LOC_Os12g38620.1	150	-21.06	150	-21.06	1657571	19	2238	2186
miR5077	LOC_Os12g38620.2	150	-21.06	150	-21.06	1657572	19	1854	1802
miR5077	LOC_Os12g38620.3	150	-21.06	150	-21.06	1657573	19	2144	2092
miR5077	LOC_Os12g38850.1	147	-20.37	147	-20.37	1657604	19	2746	1181
miR5077	LOC_Os12g38850.2	147	-20.37	147	-20.37	1657605	19	2236	676
miR5077	LOC_Os12g38850.3	147	-20.37	147	-20.37	1657606	19	2346	786

miR5077	LOC_Os12g39150.1	151	-29.94	151	-29.94	1657638	19	3732	91
miR5077	LOC_Os12g39640.1	155	-26.47	155	-26.47	1657705	19	1540	422
miR5077	LOC_Os12g41140.1	164	-30.59	164	-30.59	1657870	19	2042	1719
miR5077	LOC_Os12g41140.3	164	-30.59	164	-30.59	1657872	19	2041	1718
miR5077	LOC_Os12g44070.1	157	-25.62	157	-25.62	1658221	19	2084	681
miR5077	ChrUn.fgenesh.mRNA.11	160	-31.35	160	-31.35	1658276	19	3456	5
miR5077	ChrUn.fgenesh.mRNA.56	163	-28.2	163	-28.2	1658321	19	624	376
miR399i	LOC_Os01g01080.1	153	-21.18	153	-21.18	1658465	21	1955	63
miR399i	LOC_Os01g01080.2	153	-21.18	153	-21.18	1658466	21	1976	63
miR399i	LOC_Os01g01080.3	153	-21.18	153	-21.18	1658467	21	2042	63
miR399i	LOC_Os01g01100.1	161	-21.95	161	-21.95	1658469	21	4017	3254
miR399i	LOC_Os01g01650.1	155	-26.04	155	-26.04	1658541	21	1313	661
miR399i	LOC_Os01g01680.1	159	-26.57	159	-26.57	1658545	21	1023	111
miR399i	LOC_Os01g01689.1	162	-21.37	162	-21.37	1658546	21	8736	6153
miR399i	LOC_Os01g01689.3	162	-21.37	162	-21.37	1658547	21	9026	6153
miR399i	LOC_Os01g02000.1	168	-27.63	168	-27.63	1658583	21	2767	308
miR399i	LOC_Os01g02230.1	153	-20.56	153	-20.56	1658614	21	2898	1153
miR399i	LOC_Os01g02310.1	149	-23.83	149	-23.83	1658623	21	1884	1548
miR399i	LOC_Os01g02334.1	149	-23.83	149	-23.83	1658625	21	632	239
miR399i	LOC_Os01g02670.1	153	-20.95	153	-20.95	1658660	21	3312	589
miR399i	LOC_Os01g03070.1	153	-22.52	153	-22.52	1658709	21	3512	2165
miR399i	LOC_Os01g03070.2	153	-22.52	153	-22.52	1658710	21	3493	2159
miR399i	LOC_Os01g04950.1	157	-20.34	157	-20.34	1658913	21	2778	305
miR399i	LOC_Os01g04950.4	157	-20.34	157	-20.34	1658916	21	3836	243
miR399i	LOC_Os01g05710.1	154	-20.29	154	-20.29	1659003	21	784	468
miR399i	LOC_Os01g05800.1	149	-24.58	149	-24.58	1659011	21	2443	765
miR399i	LOC_Os01g05800.2	149	-24.58	149	-24.58	1659012	21	2440	765
miR399i	LOC_Os01g06330.1	165	-23.31	165	-23.31	1659067	21	1287	369
miR399i	LOC_Os01g06410.1	151	-21.16	151	-21.16	1659075	21	561	208
miR399i	LOC_Os01g06530.1	147	-25.72	147	-25.72	1659089	21	2805	53
miR399i	LOC_Os01g07640.1	156	-22.12	156	-22.12	1659220	21	1608	321
miR399i	LOC_Os01g08750.1	158	-22.76	158	-22.76	1659355	21	10065	3610

miR399i	LOC_Os01g08750.2	158	-22.76	158	-22.76	1659356	21	9417	2962
miR399i	LOC_Os01g08890.1	151	-20.57	151	-20.57	1659376	21	2771	355
miR399i	LOC_Os01g09550.4	155	-23.39	155	-23.39	1659448	21	5715	2064
miR399i	LOC_Os01g09640.1	163	-24.56	163	-24.56	1659458	21	1769	1371
miR399i	LOC_Os01g12350.1	152	-20.9	152	-20.9	1659746	21	3117	324
miR399i	LOC_Os01g12660.1	146	-23.21	146	-23.21	1659783	21	2967	2403
miR399i	LOC_Os01g12790.1	140	-22.85	140	-22.85	1659804	21	3756	95
miR399i	LOC_Os01g13060.2	165	-23.23	165	-23.23	1659841	21	3190	2349
miR399i	LOC_Os01g13540.1	161	-20.76	161	-20.76	1659898	21	3360	1234
miR399i	LOC_Os01g13730.1	152	-20.39	152	-20.39	1659922	21	1948	1920
miR399i	LOC_Os01g15029.1	157	-21.76	157	-21.76	1660064	21	676	429
miR399i	LOC_Os01g15690.1	154	-23.23	154	-23.23	1660136	21	4635	3326
miR399i	LOC_Os01g15890.1	154	-25.79	154	-25.79	1660157	21	2637	2315
miR399i	LOC_Os01g15900.1	156	-23.57	156	-23.57	1660158	21	2228	1405
miR399i	LOC_Os01g16130.1	149	-21.41	149	-21.41	1660187	21	2027	327
miR399i	LOC_Os01g16220.1	160	-25.98	160	-25.98	1660204	21	1693	482
miR399i	LOC_Os01g16690.1	159	-20.88	159	-20.88	1660259	21	1692	668
miR399i	LOC_Os01g18470.1	149	-20.5	149	-20.5	1660395	21	4038	1682
miR399i	LOC_Os01g21160.3	161	-21.15	161	-21.15	1660641	21	2026	166
miR399i	LOC_Os01g21450.1	147	-26.26	147	-26.26	1660669	21	2470	319
miR399i	LOC_Os01g21450.2	147	-26.26	147	-26.26	1660670	21	3220	447
miR399i	LOC_Os01g21450.3	147	-26.26	147	-26.26	1660671	21	3512	739
miR399i	LOC_Os01g21500.1	155	-24.66	155	-24.66	1660674	21	1164	971
miR399i	LOC_Os01g22640.1	157	-20.71	157	-20.71	1660782	21	1926	1663
miR399i	LOC_Os01g23730.1	168	-25.71	168	-25.71	1660893	21	804	126
miR399i	LOC_Os01g23940.1	154	-20.84	154	-20.84	1660914	21	1353	1225
miR399i	LOC_Os01g24294.1	151	-22.42	151	-22.42	1660950	21	5229	184
miR399i	LOC_Os01g25610.1	153	-20.81	153	-20.81	1661093	21	1242	1
miR399i	LOC_Os01g27040.1	154	-21.32	154	-21.32	1661190	21	3750	3118
miR399i	LOC_Os01g27180.1	150	-23.24	150	-23.24	1661205	21	456	241
miR399i	LOC_Os01g27440.1	163	-25.57	163	-25.57	1661228	21	3384	360
miR399i	LOC_Os01g27640.1	160	-21.61	160	-21.61	1661246	21	1647	1026

miR399i	LOC_Os01g27900.1	149	-20.48	149	-20.48	1661270	21	2379	532
miR399i	LOC_Os01g28020.1	155	-20.23	155	-20.23	1661284	21	1425	1238
miR399i	LOC_Os01g28540.1	159	-25.56	159	-25.56	1661331	21	1315	242
miR399i	LOC_Os01g28540.2	159	-25.56	159	-25.56	1661332	21	1288	242
miR399i	LOC_Os01g28680.8	159	-21.15	159	-21.15	1661351	21	4068	2418
miR399i	LOC_Os01g28680.9	159	-21.15	159	-21.15	1661352	21	3921	2408
miR399i	LOC_Os01g28740.1	149	-20.5	149	-20.5	1661362	21	3969	1478
miR399i	LOC_Os01g29070.1	155	-25.34	155	-25.34	1661398	21	1233	911
miR399i	LOC_Os01g29750.1	149	-20.5	149	-20.5	1661461	21	4698	2480
miR399i	LOC_Os01g31280.1	167	-26.96	167	-26.96	1661515	21	384	32
miR399i	LOC_Os01g31730.1	157	-21.13	157	-21.13	1661562	21	4719	3006
miR399i	LOC_Os01g32350.1	166	-27.79	166	-27.79	1661631	21	1281	835
miR399i	LOC_Os01g32350.2	166	-27.79	166	-27.79	1661632	21	1438	835
miR399i	LOC_Os01g32720.1	143	-23.45	143	-23.45	1661669	21	3548	3486
miR399i	LOC_Os01g32830.1	161	-20.16	161	-20.16	1661681	21	2718	2657
miR399i	LOC_Os01g32830.2	161	-20.16	161	-20.16	1661682	21	947	886
miR399i	LOC_Os01g33260.1	157	-20.35	157	-20.35	1661727	21	7063	3732
miR399i	LOC_Os01g33869.1	166	-20.03	166	-20.03	1661784	21	2391	2347
miR399i	LOC_Os01g34030.1	308	-45.5	155	-24.38	1661798	21	2607	2298 255
miR399i	LOC_Os01g34190.1	159	-28.16	159	-28.16	1661810	21	1531	1184
miR399i	LOC_Os01g34490.1	145	-20.25	145	-20.25	1661840	21	3000	2179
miR399i	LOC_Os01g34630.1	148	-21.32	148	-21.32	1661864	21	2389	412
miR399i	LOC_Os01g34890.1	145	-23.11	145	-23.11	1661891	21	3675	915
miR399i	LOC_Os01g35090.1	314	-42.22	161	-21.35	1661912	21	3960	2572 2623
miR399i	LOC_Os01g35480.1	153	-20.56	153	-20.56	1661951	21	6039	4258
miR399i	LOC_Os01g35740.1	153	-20.56	153	-20.56	1661982	21	6039	4258
miR399i	LOC_Os01g36080.1	145	-20.64	145	-20.64	1662015	21	2886	2621
miR399i	LOC_Os01g36080.2	145	-20.64	145	-20.64	1662016	21	2902	2606
miR399i	LOC_Os01g36080.3	145	-20.64	145	-20.64	1662017	21	2323	2059
miR399i	LOC_Os01g36080.4	150	-20.48	150	-20.48	1662018	21	2944	2680
miR399i	LOC_Os01g36410.1	152	-23.4	152	-23.4	1662049	21	711	192
miR399i	LOC_Os01g36730.1	157	-25.26	157	-25.26	1662080	21	2538	2183

miR399i	LOC_Os01g37250.1	157	-21.13	157	-21.13	1662138	21	2220	776
miR399i	LOC_Os01g37360.1	156	-23.31	156	-23.31	1662157	21	2103	1734
miR399i	LOC_Os01g37560.1	159	-23.16	159	-23.16	1662176	21	2012	1023
miR399i	LOC_Os01g37560.2	159	-23.16	159	-23.16	1662177	21	1836	1023
miR399i	LOC_Os01g37560.3	159	-23.16	159	-23.16	1662178	21	2132	1143
miR399i	LOC_Os01g37570.1	155	-25.34	155	-25.34	1662179	21	1368	911
miR399i	LOC_Os01g37580.1	154	-21.35	154	-21.35	1662180	21	5799	3307
miR399i	LOC_Os01g38260.1	154	-22.63	154	-22.63	1662259	21	2067	1972
miR399i	LOC_Os01g38300.1	157	-23.88	157	-23.88	1662263	21	1131	7
miR399i	LOC_Os01g38410.1	149	-23.81	149	-23.81	1662273	21	1482	835
miR399i	LOC_Os01g38880.1	162	-21.61	162	-21.61	1662323	21	1797	527
miR399i	LOC_Os01g38930.1	159	-20.48	159	-20.48	1662328	21	849	415
miR399i	LOC_Os01g39370.1	161	-21.95	161	-21.95	1662375	21	5430	3722
miR399i	LOC_Os01g39399.1	165	-23.45	165	-23.45	1662380	21	519	1
miR399i	LOC_Os01g40630.1	151	-22.66	151	-22.66	1662513	21	1429	337
miR399i	LOC_Os01g40630.2	151	-22.66	151	-22.66	1662514	21	1856	337
miR399i	LOC_Os01g40630.3	151	-22.66	151	-22.66	1662515	21	1404	337
miR399i	LOC_Os01g41400.1	167	-21.97	167	-21.97	1662599	21	1332	335
miR399i	LOC_Os01g41660.1	179	-33.62	179	-33.62	1662627	21	1641	398
miR399i	LOC_Os01g41950.1	151	-20.04	151	-20.04	1662656	21	3201	1222
miR399i	LOC_Os01g42490.1	151	-23.19	151	-23.19	1662713	21	3092	2997
miR399i	LOC_Os01g42490.2	151	-23.19	151	-23.19	1662714	21	4032	3937
miR399i	LOC_Os01g42490.3	151	-23.19	151	-23.19	1662715	21	3426	3331
miR399i	LOC_Os01g42700.1	163	-28.02	163	-28.02	1662739	21	2576	1552
miR399i	LOC_Os01g42700.2	163	-28.02	163	-28.02	1662740	21	1875	1552
miR399i	LOC_Os01g42700.5	163	-28.02	163	-28.02	1662743	21	1837	1552
miR399i	LOC_Os01g42710.1	152	-22.07	152	-22.07	1662744	21	4918	3090
miR399i	LOC_Os01g43090.1	155	-21.93	155	-21.93	1662791	21	1353	205
miR399i	LOC_Os01g43170.1	148	-20.55	148	-20.55	1662801	21	2132	1379
miR399i	LOC_Os01g43170.2	148	-20.55	148	-20.55	1662802	21	2190	1379
miR399i	LOC_Os01g43170.3	148	-20.55	148	-20.55	1662803	21	2166	1358
miR399i	LOC_Os01g46810.1	157	-20.48	157	-20.48	1663154	21	4832	4246

miR399i	LOC_Os01g47690.1	160	-28.95	160	-28.95	1663266	21	1559	456
miR399i	LOC_Os01g47690.2	160	-28.95	160	-28.95	1663267	21	1385	282
miR399i	LOC_Os01g47820.1	158	-27.56	158	-27.56	1663285	21	2425	559
miR399i	LOC_Os01g48700.1	164	-21.96	164	-21.96	1663385	21	1185	814
miR399i	LOC_Os01g48900.1	145	-21.66	145	-21.66	1663406	21	2691	1309
miR399i	LOC_Os01g49060.1	162	-22.08	162	-22.08	1663430	21	1961	542
miR399i	LOC_Os01g49060.2	162	-22.08	162	-22.08	1663431	21	1955	532
miR399i	LOC_Os01g49060.3	162	-22.08	162	-22.08	1663432	21	2044	532
miR399i	LOC_Os01g49370.1	166	-27.49	166	-27.49	1663464	21	981	936
miR399i	LOC_Os01g50360.1	150	-22.34	150	-22.34	1663572	21	1551	392
miR399i	LOC_Os01g50530.1	141	-23.86	141	-23.86	1663588	21	1236	99
miR399i	LOC_Os01g51890.1	161	-22.93	161	-22.93	1663755	21	2687	2013
miR399i	LOC_Os01g52250.1	154	-21.9	154	-21.9	1663796	21	5093	5056
miR399i	LOC_Os01g52250.2	154	-21.9	154	-21.9	1663797	21	4841	4804
miR399i	LOC_Os01g52250.3	154	-21.9	154	-21.9	1663798	21	4452	4415
miR399i	LOC_Os01g52250.4	154	-21.9	154	-21.9	1663799	21	5084	5047
miR399i	LOC_Os01g52250.5	154	-21.9	154	-21.9	1663800	21	5082	5045
miR399i	LOC_Os01g52250.6	154	-21.9	154	-21.9	1663801	21	5163	5126
miR399i	LOC_Os01g52550.1	152	-22.81	152	-22.81	1663835	21	4255	4128
miR399i	LOC_Os01g53200.1	164	-23.98	164	-23.98	1663903	21	2027	583
miR399i	LOC_Os01g53390.1	144	-24.87	144	-24.87	1663923	21	1949	1580
miR399i	LOC_Os01g53420.1	155	-21.04	155	-21.04	1663924	21	1752	953
miR399i	LOC_Os01g53480.1	161	-21.55	161	-21.55	1663931	21	2301	1199
miR399i	LOC_Os01g53750.1	152	-23.62	152	-23.62	1663961	21	1425	1269
miR399i	LOC_Os01g54080.1	155	-23.65	155	-23.65	1664006	21	3356	492
miR399i	LOC_Os01g54600.1	151	-21.72	151	-21.72	1664066	21	1731	676
miR399i	LOC_Os01g55120.1	158	-22.55	158	-22.55	1664129	21	1767	816
miR399i	LOC_Os01g55360.1	147	-23.35	147	-23.35	1664159	21	1892	725
miR399i	LOC_Os01g55360.2	147	-23.35	147	-23.35	1664160	21	1897	725
miR399i	LOC_Os01g55360.3	147	-23.35	147	-23.35	1664161	21	1806	725
miR399i	LOC_Os01g57280.1	169	-26.68	169	-26.68	1664409	21	4172	1320
miR399i	LOC_Os01g57280.2	169	-26.68	169	-26.68	1664410	21	4423	1571

miR399i	LOC_Os01g57280.3	169	-26.68	169	-26.68	1664411	21	4944	1571
miR399i	LOC_Os01g57810.1	157	-25.26	157	-25.26	1664475	21	2337	2033
miR399i	LOC_Os01g58400.1	155	-21.88	155	-21.88	1664544	21	2032	1116
miR399i	LOC_Os01g58400.2	155	-21.88	155	-21.88	1664545	21	1999	1116
miR399i	LOC_Os01g58470.1	150	-26.44	150	-26.44	1664554	21	1126	436
miR399i	LOC_Os01g58740.1	157	-20.26	157	-20.26	1664584	21	1989	1135
miR399i	LOC_Os01g58740.2	157	-20.26	157	-20.26	1664585	21	2188	1041
miR399i	LOC_Os01g59060.1	152	-20.66	152	-20.66	1664619	21	756	262
miR399i	LOC_Os01g59670.1	151	-21.93	151	-21.93	1664692	21	4538	723
miR399i	LOC_Os01g60520.1	162	-30.93	162	-30.93	1664788	21	852	100
miR399i	LOC_Os01g61710.1	150	-22.8	150	-22.8	1664922	21	1842	1041
miR399i	LOC_Os01g61710.2	150	-22.8	150	-22.8	1664923	21	1954	1153
miR399i	LOC_Os01g61710.3	150	-22.8	150	-22.8	1664924	21	2143	1153
miR399i	LOC_Os01g61710.4	150	-22.8	150	-22.8	1664925	21	1928	1153
miR399i	LOC_Os01g61710.5	150	-22.8	150	-22.8	1664926	21	2224	1151
miR399i	LOC_Os01g61710.6	150	-22.8	150	-22.8	1664927	21	2134	1153
miR399i	LOC_Os01g62100.1	158	-30.13	158	-30.13	1664989	21	5409	5011
miR399i	LOC_Os01g62340.1	157	-25.26	157	-25.26	1665015	21	2397	2210
miR399i	LOC_Os01g62440.1	149	-20.63	149	-20.63	1665033	21	3075	2156
miR399i	LOC_Os01g62440.2	149	-20.63	149	-20.63	1665034	21	2005	1086
miR399i	LOC_Os01g62440.3	149	-20.63	149	-20.63	1665035	21	3154	2235
miR399i	LOC_Os01g63400.1	162	-21.19	162	-21.19	1665149	21	3097	2893
miR399i	LOC_Os01g63400.2	162	-21.19	162	-21.19	1665150	21	3190	2988
miR399i	LOC_Os01g63410.1	162	-21.19	162	-21.19	1665151	21	1378	1176
miR399i	LOC_Os01g63710.1	147	-20.1	147	-20.1	1665180	21	2163	112
miR399i	LOC_Os01g63710.3	147	-20.1	147	-20.1	1665182	21	3745	1579
miR399i	LOC_Os01g63720.1	163	-22.48	163	-22.48	1665183	21	258	218
miR399i	LOC_Os01g64270.1	149	-20.38	149	-20.38	1665262	21	1214	1069
miR399i	LOC_Os01g64280.1	149	-20.38	149	-20.38	1665263	21	2580	1254
miR399i	LOC_Os01g65190.1	167	-25.02	167	-25.02	1665382	21	2156	1185
miR399i	LOC_Os01g65230.1	151	-20.29	151	-20.29	1665388	21	2258	419
miR399i	LOC_Os01g65320.1	302	-40.52	152	-20.45	1665395	21	4563	3809 4538

miR399i	LOC_Os01g65330.1	162	-23.52	162	-23.52	1665396	21	2336	336
miR399i	LOC_Os01g65330.2	162	-23.52	162	-23.52	1665397	21	2422	336
miR399i	LOC_Os01g65460.1	150	-27.58	150	-27.58	1665414	21	3664	1062
miR399i	LOC_Os01g65460.2	150	-27.58	150	-27.58	1665415	21	3787	1062
miR399i	LOC_Os01g66840.1	151	-20.5	151	-20.5	1665572	21	1203	451
miR399i	LOC_Os01g66910.1	169	-33.46	169	-33.46	1665582	21	378	245
miR399i	LOC_Os01g67170.1	157	-28.3	157	-28.3	1665613	21	2118	1129
miR399i	LOC_Os01g68179.1	182	-33.24	182	-33.24	1665742	21	743	658
miR399i	LOC_Os01g68200.1	154	-20.29	154	-20.29	1665743	21	915	827
miR399i	LOC_Os01g68490.1	154	-25.24	154	-25.24	1665780	21	4040	2386
miR399i	LOC_Os01g68750.1	146	-20.44	146	-20.44	1665812	21	3582	852
miR399i	LOC_Os01g70260.1	165	-24.35	165	-24.35	1665941	21	2715	2532
miR399i	LOC_Os01g70260.2	165	-24.35	165	-24.35	1665942	21	2867	2684
miR399i	LOC_Os01g70320.1	163	-22.99	163	-22.99	1665954	21	2213	1185
miR399i	LOC_Os01g70630.1	161	-21.83	161	-21.83	1665993	21	3217	2703
miR399i	LOC_Os01g70710.1	165	-24	165	-24	1666002	21	1669	1036
miR399i	LOC_Os01g70710.2	165	-24	165	-24	1666003	21	1917	1015
miR399i	LOC_Os01g71100.1	146	-22.43	146	-22.43	1666053	21	3575	1054
miR399i	LOC_Os01g71106.2	157	-22.57	157	-22.57	1666054	21	4440	888
miR399i	LOC_Os01g71630.1	143	-21.56	143	-21.56	1666115	21	2405	1113
miR399i	LOC_Os01g71630.2	143	-21.56	143	-21.56	1666116	21	2437	1145
miR399i	LOC_Os01g71630.4	143	-21.56	143	-21.56	1666117	21	2432	1140
miR399i	LOC_Os01g72350.1	145	-23.43	145	-23.43	1666192	21	2418	1553
miR399i	LOC_Os01g72350.2	145	-23.43	145	-23.43	1666193	21	2378	1553
miR399i	LOC_Os01g72870.1	146	-20.27	146	-20.27	1666254	21	1319	952
miR399i	LOC_Os01g72870.2	146	-20.27	146	-20.27	1666255	21	1271	952
miR399i	LOC_Os01g72870.3	146	-20.27	146	-20.27	1666256	21	1217	952
miR399i	LOC_Os01g73000.1	163	-24.82	163	-24.82	1666272	21	2202	999
miR399i	LOC_Os01g73000.2	163	-24.82	163	-24.82	1666273	21	1983	780
miR399i	LOC_Os01g73250.1	156	-21.48	156	-21.48	1666301	21	1340	1186
miR399i	LOC_Os01g73380.1	159	-20.6	159	-20.6	1666313	21	3393	3057
miR399i	LOC_Os01g73410.1	150	-26.17	150	-26.17	1666316	21	3788	1997

miR399i	LOC_Os01g73680.1	144	-22.76	144	-22.76	1666350	21	2524	618
miR399i	LOC_Os01g74460.1	160	-21.4	160	-21.4	1666443	21	2281	2173
miR399i	LOC_Os01g74460.2	160	-21.4	160	-21.4	1666444	21	2364	2256
miR399i	LOC_Os01g74530.2	153	-21.49	153	-21.49	1666452	21	2557	2088
miR399i	LOC_Os02g01220.1	151	-22.2	151	-22.2	1666506	21	1369	1338
miR399i	LOC_Os02g01230.1	151	-22.2	151	-22.2	1666507	21	2378	836
miR399i	LOC_Os02g01230.2	151	-22.2	151	-22.2	1666508	21	2257	715
miR399i	LOC_Os02g01490.1	147	-22.11	147	-22.11	1666539	21	4545	414
miR399i	LOC_Os02g01640.1	156	-24.57	156	-24.57	1666556	21	4461	2881
miR399i	LOC_Os02g01670.1	156	-24.57	156	-24.57	1666558	21	4461	2881
miR399i	LOC_Os02g02700.1	162	-25.5	162	-25.5	1666678	21	2034	2008
miR399i	LOC_Os02g02810.1	152	-22.44	152	-22.44	1666698	21	1170	545
miR399i	LOC_Os02g02970.1	149	-20.98	149	-20.98	1666722	21	1801	314
miR399i	LOC_Os02g03540.1	168	-29.86	168	-29.86	1666797	21	1456	503
miR399i	LOC_Os02g03980.1	164	-25.55	164	-25.55	1666857	21	1185	752
miR399i	LOC_Os02g04230.1	162	-20.14	162	-20.14	1666892	21	2104	1463
miR399i	LOC_Os02g04230.2	162	-20.14	162	-20.14	1666893	21	2004	1463
miR399i	LOC_Os02g04230.3	162	-20.14	162	-20.14	1666894	21	2021	1463
miR399i	LOC_Os02g04590.1	174	-32.7	174	-32.7	1666944	21	429	244
miR399i	LOC_Os02g04630.1	167	-23.63	167	-23.63	1666948	21	1089	304
miR399i	LOC_Os02g04850.1	165	-26.49	165	-26.49	1666981	21	1535	1496
miR399i	LOC_Os02g05510.1	163	-24.96	163	-24.96	1667066	21	1469	1296
miR399i	LOC_Os02g05510.3	163	-24.96	163	-24.96	1667067	21	1397	1224
miR399i	LOC_Os02g06060.1	157	-26.48	157	-26.48	1667131	21	255	184
miR399i	LOC_Os02g06570.1	149	-23.34	149	-23.34	1667187	21	2420	1335
miR399i	LOC_Os02g06570.2	149	-23.34	149	-23.34	1667188	21	2492	1335
miR399i	LOC_Os02g06605.1	159	-21.89	159	-21.89	1667196	21	781	625
miR399i	LOC_Os02g06910.1	151	-20.86	151	-20.86	1667233	21	3929	3757
miR399i	LOC_Os02g07540.1	153	-21.08	153	-21.08	1667320	21	5895	4111
miR399i	LOC_Os02g07654.1	160	-21.58	160	-21.58	1667334	21	795	518
miR399i	LOC_Os02g08130.1	168	-25	168	-25	1667401	21	2798	1365
miR399i	LOC_Os02g08130.2	168	-25	168	-25	1667402	21	4810	1365

miR399i	LOC_Os02g08130.3	168	-25	168	-25	1667403	21	4638	1348
miR399i	LOC_Os02g09960.1	156	-21.21	156	-21.21	1667560	21	2372	928
miR399i	LOC_Os02g10400.1	150	-21.18	150	-21.18	1667616	21	1446	643
miR399i	LOC_Os02g10990.1	150	-22.43	150	-22.43	1667693	21	1963	975
miR399i	LOC_Os02g12660.1	144	-21.53	144	-21.53	1667840	21	2993	2118
miR399i	LOC_Os02g12730.1	155	-21.74	155	-21.74	1667847	21	3169	1489
miR399i	LOC_Os02g12730.2	155	-21.74	155	-21.74	1667848	21	2319	999
miR399i	LOC_Os02g12730.3	155	-21.74	155	-21.74	1667849	21	3257	1577
miR399i	LOC_Os02g13640.1	158	-22.14	158	-22.14	1667966	21	1645	926
miR399i	LOC_Os02g13960.1	143	-21.86	143	-21.86	1668000	21	3311	2398
miR399i	LOC_Os02g14130.1	156	-21.74	156	-21.74	1668022	21	2112	653
miR399i	LOC_Os02g14210.1	153	-24.89	153	-24.89	1668030	21	1979	833
miR399i	LOC_Os02g14340.1	150	-21.11	150	-21.11	1668043	21	576	148
miR399i	LOC_Os02g15310.1	150	-20.28	150	-20.28	1668155	21	1269	920
miR399i	LOC_Os02g15310.2	150	-20.28	150	-20.28	1668156	21	2241	1891
miR399i	LOC_Os02g15310.3	150	-20.28	150	-20.28	1668157	21	1822	1473
miR399i	LOC_Os02g15310.4	150	-20.28	150	-20.28	1668158	21	2071	1721
miR399i	LOC_Os02g15550.1	162	-23.85	162	-23.85	1668182	21	2139	552
miR399i	LOC_Os02g15550.2	162	-23.85	162	-23.85	1668183	21	1699	552
miR399i	LOC_Os02g15550.3	162	-23.85	162	-23.85	1668184	21	1953	552
miR399i	LOC_Os02g16270.1	151	-22.52	151	-22.52	1668268	21	4510	4425
miR399i	LOC_Os02g16270.2	151	-22.52	151	-22.52	1668269	21	4520	4435
miR399i	LOC_Os02g17980.1	166	-25.16	166	-25.16	1668454	21	1725	308
miR399i	LOC_Os02g18080.1	154	-21.05	154	-21.05	1668468	21	4117	3140
miR399i	LOC_Os02g18090.1	158	-20.47	158	-20.47	1668469	21	1128	370
miR399i	LOC_Os02g18140.1	147	-20.81	147	-20.81	1668475	21	2709	1219
miR399i	LOC_Os02g18210.1	314	-41.1	157	-20.97	1668484	21	2970	2578 2631
miR399i	LOC_Os02g18240.1	155	-24.66	155	-24.66	1668487	21	1224	848
miR399i	LOC_Os02g18760.1	151	-24.91	151	-24.91	1668541	21	2733	2371
miR399i	LOC_Os02g19100.1	154	-20.54	154	-20.54	1668577	21	2301	1177
miR399i	LOC_Os02g19170.1	147	-22.43	147	-22.43	1668583	21	2509	771
miR399i	LOC_Os02g19170.2	147	-22.43	147	-22.43	1668584	21	2576	838

miR399i	LOC_Os02g19170.3	147	-22.43	147	-22.43	1668585	21	2524	824
miR399i	LOC_Os02g19320.1	157	-21.4	157	-21.4	1668601	21	426	259
miR399i	LOC_Os02g19550.1	158	-22.47	158	-22.47	1668628	21	2284	1699
miR399i	LOC_Os02g19690.1	150	-25.46	150	-25.46	1668641	21	732	430
miR399i	LOC_Os02g19780.1	319	-48.94	168	-26	1668650	21	537	227 489
miR399i	LOC_Os02g20100.1	318	-46.13	161	-25.26	1668681	21	5415	4036 5045
miR399i	LOC_Os02g20120.1	157	-25.26	157	-25.26	1668683	21	2205	1925
miR399i	LOC_Os02g20450.1	157	-22.71	157	-22.71	1668718	21	462	378
miR399i	LOC_Os02g20460.1	157	-29.89	157	-29.89	1668719	21	2103	1390
miR399i	LOC_Os02g20570.1	153	-20.95	153	-20.95	1668731	21	3369	40
miR399i	LOC_Os02g20640.1	159	-25.65	159	-25.65	1668736	21	1230	33
miR399i	LOC_Os02g21530.1	144	-22.74	144	-22.74	1668835	21	609	114
miR399i	LOC_Os02g21570.1	140	-21.12	140	-21.12	1668839	21	671	161
miR399i	LOC_Os02g22000.1	150	-20.01	150	-20.01	1668886	21	564	138
miR399i	LOC_Os02g22120.1	154	-20.97	154	-20.97	1668906	21	675	216
miR399i	LOC_Os02g22210.1	327	-47.53	164	-26.1	1668914	21	3396	2981 1248
miR399i	LOC_Os02g22260.1	159	-21.12	159	-21.12	1668919	21	1324	986
miR399i	LOC_Os02g22260.2	159	-21.12	159	-21.12	1668920	21	1644	1306
miR399i	LOC_Os02g22320.1	149	-27.19	149	-27.19	1668924	21	837	90
miR399i	LOC_Os02g24090.1	321	-46.66	163	-25.96	1669027	21	4725	2759 52
miR399i	LOC_Os02g25560.1	166	-24.42	166	-24.42	1669179	21	2388	1454
miR399i	LOC_Os02g26060.1	158	-20.06	158	-20.06	1669230	21	3768	3112
miR399i	LOC_Os02g26290.1	154	-24.11	154	-24.11	1669254	21	1066	383
miR399i	LOC_Os02g26370.1	156	-23.63	156	-23.63	1669269	21	1491	768
miR399i	LOC_Os02g27650.1	318	-42.45	161	-21.58	1669426	21	2781	1105 1156
miR399i	LOC_Os02g28780.1	157	-20.91	157	-20.91	1669535	21	3483	2503
miR399i	LOC_Os02g29030.1	146	-22.48	146	-22.48	1669561	21	5010	204
miR399i	LOC_Os02g29040.1	157	-20.1	157	-20.1	1669562	21	1636	439
miR399i	LOC_Os02g29040.2	157	-20.1	157	-20.1	1669563	21	1597	439
miR399i	LOC_Os02g29190.1	158	-21.63	158	-21.63	1669579	21	1395	171
miR399i	LOC_Os02g29210.1	158	-21.63	158	-21.63	1669581	21	1674	664
miR399i	LOC_Os02g29210.2	158	-21.63	158	-21.63	1669582	21	1600	637

miR399i	LOC_Os02g29210.3	158	-21.63	158	-21.63	1669583	21	2226	637
miR399i	LOC_Os02g29210.4	158	-21.63	158	-21.63	1669584	21	1603	637
miR399i	LOC_Os02g29380.1	157	-21.25	157	-21.25	1669600	21	3992	3713
miR399i	LOC_Os02g29480.1	157	-22.57	157	-22.57	1669608	21	2123	1202
miR399i	LOC_Os02g30040.1	157	-20.91	157	-20.91	1669667	21	2751	1150
miR399i	LOC_Os02g30060.1	152	-21.78	152	-21.78	1669669	21	1110	1060
miR399i	LOC_Os02g30290.1	149	-20.5	149	-20.5	1669699	21	4161	1805
miR399i	LOC_Os02g30730.1	155	-22.27	155	-22.27	1669752	21	3090	666
miR399i	LOC_Os02g30730.2	155	-22.27	155	-22.27	1669753	21	3164	740
miR399i	LOC_Os02g31180.1	152	-21.4	152	-21.4	1669802	21	5520	4189
miR399i	LOC_Os02g31926.1	152	-22.13	152	-22.13	1669833	21	1071	464
miR399i	LOC_Os02g31960.1	165	-25.07	165	-25.07	1669836	21	1834	643
miR399i	LOC_Os02g31960.3	165	-25.07	165	-25.07	1669837	21	1739	643
miR399i	LOC_Os02g31960.4	165	-25.07	165	-25.07	1669838	21	1819	643
miR399i	LOC_Os02g32220.1	151	-20.86	151	-20.86	1669865	21	732	706
miR399i	LOC_Os02g32465.1	158	-20.05	158	-20.05	1669893	21	447	369
miR399i	LOC_Os02g32880.1	151	-21.18	151	-21.18	1669944	21	6558	4296
miR399i	LOC_Os02g33280.1	157	-20.97	157	-20.97	1669988	21	3936	2536
miR399i	LOC_Os02g33490.1	156	-21.25	156	-21.25	1670014	21	3299	655
miR399i	LOC_Os02g34490.1	150	-21.96	150	-21.96	1670109	21	3225	2263
miR399i	LOC_Os02g34650.1	157	-22.77	157	-22.77	1670129	21	1820	1592
miR399i	LOC_Os02g34660.1	140	-23.66	140	-23.66	1670130	21	2790	2391
miR399i	LOC_Os02g35190.1	145	-23.3	145	-23.3	1670193	21	2934	379
miR399i	LOC_Os02g35190.2	145	-23.3	145	-23.3	1670194	21	2896	379
miR399i	LOC_Os02g35230.1	148	-21.61	148	-21.61	1670198	21	2360	463
miR399i	LOC_Os02g35290.1	314	-41.1	157	-20.97	1670204	21	4305	3479 3532
miR399i	LOC_Os02g35820.1	147	-21.75	147	-21.75	1670263	21	4812	1111
miR399i	LOC_Os02g35820.2	147	-21.75	147	-21.75	1670264	21	4518	1111
miR399i	LOC_Os02g36340.1	162	-22.19	162	-22.19	1670319	21	2247	389
miR399i	LOC_Os02g36340.2	162	-22.19	162	-22.19	1670320	21	2220	389
miR399i	LOC_Os02g37960.1	155	-22.69	155	-22.69	1670526	21	1836	857
miR399i	LOC_Os02g38640.1	147	-20.09	147	-20.09	1670609	21	873	167

miR399i	LOC_Os02g38790.1	150	-21.18	150	-21.18	1670626	21	1416	1084
miR399i	LOC_Os02g39064.1	153	-20.79	153	-20.79	1670665	21	525	25
miR399i	LOC_Os02g39764.1	158	-21.64	158	-21.64	1670759	21	1338	944
miR399i	LOC_Os02g40450.1	156	-22.54	156	-22.54	1670850	21	3946	945
miR399i	LOC_Os02g41590.1	166	-26.56	166	-26.56	1670928	21	1736	761
miR399i	LOC_Os02g41890.1	161	-22.55	161	-22.55	1670963	21	3612	458
miR399i	LOC_Os02g42040.1	164	-24.01	164	-24.01	1670982	21	4437	3312
miR399i	LOC_Os02g42180.1	153	-27.56	153	-27.56	1670996	21	4578	2829
miR399i	LOC_Os02g42330.1	154	-21.79	154	-21.79	1671020	21	1747	375
miR399i	LOC_Os02g42330.2	154	-21.79	154	-21.79	1671021	21	1498	306
miR399i	LOC_Os02g42950.1	149	-26.74	149	-26.74	1671095	21	1971	915
miR399i	LOC_Os02g42950.2	149	-26.74	149	-26.74	1671096	21	1409	915
miR399i	LOC_Os02g43170.2	151	-21.72	151	-21.72	1671125	21	1610	842
miR399i	LOC_Os02g43860.1	162	-22.93	162	-22.93	1671207	21	2416	1062
miR399i	LOC_Os02g43860.2	162	-22.93	162	-22.93	1671208	21	2677	1062
miR399i	LOC_Os02g44642.1	159	-21.59	159	-21.59	1671325	21	3757	3220
miR399i	LOC_Os02g44720.1	151	-21.54	151	-21.54	1671335	21	851	188
miR399i	LOC_Os02g44870.1	158	-22.31	158	-22.31	1671355	21	1371	1056
miR399i	LOC_Os02g45150.2	157	-20.38	157	-20.38	1671392	21	2026	620
miR399i	LOC_Os02g45390.1	148	-33.67	148	-33.67	1671417	21	1261	1041
miR399i	LOC_Os02g46030.1	303	-45.11	152	-24.11	1671490	21	2083	1068 1715
miR399i	LOC_Os02g46962.1	152	-26.44	152	-26.44	1671604	21	1790	329
miR399i	LOC_Os02g46962.2	152	-26.44	152	-26.44	1671605	21	1702	241
miR399i	LOC_Os02g46962.3	152	-26.44	152	-26.44	1671606	21	1530	69
miR399i	LOC_Os02g47380.1	142	-23.41	142	-23.41	1671657	21	2868	844
miR399i	LOC_Os02g47390.1	158	-23.34	158	-23.34	1671658	21	3305	2608
miR399i	LOC_Os02g47570.1	161	-22.68	161	-22.68	1671686	21	1219	243
miR399i	LOC_Os02g47570.2	161	-22.68	161	-22.68	1671687	21	940	243
miR399i	LOC_Os02g48340.1	151	-21	151	-21	1671782	21	2237	619
miR399i	LOC_Os02g48340.2	151	-21	151	-21	1671783	21	2213	592
miR399i	LOC_Os02g48340.3	151	-21	151	-21	1671784	21	2210	592
miR399i	LOC_Os02g48340.5	151	-21	151	-21	1671785	21	1719	592

miR399i	LOC_Os02g48340.6	151	-21	151	-21	1671786	21	2304	592
miR399i	LOC_Os02g48900.1	157	-20.44	157	-20.44	1671858	21	2024	1794
miR399i	LOC_Os02g48900.2	157	-20.44	157	-20.44	1671859	21	2565	2335
miR399i	LOC_Os02g49050.1	151	-21.12	151	-21.12	1671872	21	2523	1622
miR399i	LOC_Os02g49050.2	151	-21.12	151	-21.12	1671873	21	2501	1615
miR399i	LOC_Os02g49280.1	165	-24.15	165	-24.15	1671905	21	786	263
miR399i	LOC_Os02g49320.1	155	-20.54	155	-20.54	1671909	21	1710	618
miR399i	LOC_Os02g49332.2	159	-21.36	159	-21.36	1671912	21	4273	1427
miR399i	LOC_Os02g49332.3	159	-21.36	159	-21.36	1671913	21	4042	1419
miR399i	LOC_Os02g49670.1	173	-34.32	173	-34.32	1671953	21	4050	3247
miR399i	LOC_Os02g49730.1	161	-21.9	161	-21.9	1671965	21	1452	487
miR399i	LOC_Os02g49800.1	150	-20.77	150	-20.77	1671975	21	2215	1328
miR399i	LOC_Os02g50380.1	160	-21.06	160	-21.06	1672043	21	414	130
miR399i	LOC_Os02g50530.1	164	-21.43	164	-21.43	1672059	21	2460	1904
miR399i	LOC_Os02g50540.1	149	-22.01	149	-22.01	1672060	21	1735	1348
miR399i	LOC_Os02g50570.1	150	-25.11	150	-25.11	1672064	21	3448	2037
miR399i	LOC_Os02g52120.1	155	-24.66	155	-24.66	1672256	21	354	121
miR399i	LOC_Os02g52380.1	150	-24.01	150	-24.01	1672295	21	1623	793
miR399i	LOC_Os02g52744.1	147	-23.22	147	-23.22	1672336	21	1127	815
miR399i	LOC_Os02g52744.2	147	-23.22	147	-23.22	1672337	21	1123	811
miR399i	LOC_Os02g53850.1	161	-22.33	161	-22.33	1672487	21	2187	1379
miR399i	LOC_Os02g54100.1	147	-21.26	147	-21.26	1672515	21	2516	1296
miR399i	LOC_Os02g54810.1	174	-34.8	174	-34.8	1672596	21	453	87
miR399i	LOC_Os02g55010.1	158	-23.19	158	-23.19	1672619	21	3772	3082
miR399i	LOC_Os02g56740.1	157	-22.26	157	-22.26	1672834	21	3142	3004
miR399i	LOC_Os02g56740.2	157	-22.26	157	-22.26	1672835	21	3244	3106
miR399i	LOC_Os02g57340.1	158	-24.14	158	-24.14	1672921	21	3061	1658
miR399i	LOC_Os02g57670.1	150	-20.57	150	-20.57	1672980	21	1087	811
miR399i	LOC_Os02g57880.1	149	-20.93	149	-20.93	1673010	21	1172	1056
miR399i	LOC_Os02g58030.1	154	-24.35	154	-24.35	1673030	21	2311	29
miR399i	LOC_Os02g58480.1	140	-21.49	140	-21.49	1673081	21	3550	3200
miR399i	LOC_Os02g58490.1	140	-21.49	140	-21.49	1673082	21	3647	299

miR399i	LOC_Os02g58710.1	161	-21.95	161	-21.95	1673113	21	4191	3737
miR399i	LOC_Os03g02150.1	154	-24.56	154	-24.56	1673292	21	3083	1740
miR399i	LOC_Os03g02150.2	154	-24.56	154	-24.56	1673293	21	3870	1740
miR399i	LOC_Os03g02630.1	154	-20.95	154	-20.95	1673358	21	672	24
miR399i	LOC_Os03g02890.1	152	-20.33	152	-20.33	1673400	21	2373	1614
miR399i	LOC_Os03g03250.1	153	-20.56	153	-20.56	1673440	21	5862	4192
miR399i	LOC_Os03g03420.1	173	-28.4	173	-28.4	1673459	21	727	420
miR399i	LOC_Os03g03420.2	173	-28.4	173	-28.4	1673460	21	814	420
miR399i	LOC_Os03g03650.1	164	-23.37	164	-23.37	1673490	21	1985	824
miR399i	LOC_Os03g04560.1	155	-23.5	155	-23.5	1673603	21	3431	1385
miR399i	LOC_Os03g05334.1	166	-30.63	166	-30.63	1673698	21	656	356
miR399i	LOC_Os03g05350.1	153	-24.27	153	-24.27	1673700	21	4386	2104
miR399i	LOC_Os03g06180.1	156	-20.88	156	-20.88	1673809	21	2398	590
miR399i	LOC_Os03g06220.1	151	-20.67	151	-20.67	1673813	21	2525	1461
miR399i	LOC_Os03g06420.2	159	-22.39	159	-22.39	1673836	21	2487	2012
miR399i	LOC_Os03g06710.1	159	-21.62	159	-21.62	1673872	21	3133	777
miR399i	LOC_Os03g07130.1	147	-21.99	147	-21.99	1673924	21	2388	1311
miR399i	LOC_Os03g07130.2	147	-21.99	147	-21.99	1673925	21	2181	1104
miR399i	LOC_Os03g07130.3	147	-21.99	147	-21.99	1673926	21	2292	1311
miR399i	LOC_Os03g07130.4	147	-21.99	147	-21.99	1673927	21	2167	1207
miR399i	LOC_Os03g07130.5	147	-21.99	147	-21.99	1673928	21	1528	451
miR399i	LOC_Os03g07420.1	169	-25.72	169	-25.72	1673962	21	1502	46
miR399i	LOC_Os03g07580.1	164	-21.09	164	-21.09	1673988	21	4354	3081
miR399i	LOC_Os03g07620.1	156	-20.18	156	-20.18	1673992	21	396	309
miR399i	LOC_Os03g08120.1	158	-22.13	158	-22.13	1674049	21	1278	50
miR399i	LOC_Os03g08430.1	159	-20.7	159	-20.7	1674093	21	4128	2432
miR399i	LOC_Os03g08430.2	159	-20.7	159	-20.7	1674094	21	4125	2432
miR399i	LOC_Os03g09850.1	157	-20.59	157	-20.59	1674216	21	1781	94
miR399i	LOC_Os03g09970.1	151	-21.29	151	-21.29	1674230	21	2809	1818
miR399i	LOC_Os03g09970.2	151	-21.29	151	-21.29	1674231	21	2621	1630
miR399i	LOC_Os03g09970.3	151	-21.29	151	-21.29	1674232	21	2529	1538
miR399i	LOC_Os03g09970.4	151	-21.29	151	-21.29	1674233	21	2536	1545

miR399i	LOC_Os03g11720.1	147	-20.35	147	-20.35	1674445	21	3129	1916
miR399i	LOC_Os03g11780.1	152	-21.66	152	-21.66	1674452	21	646	392
miR399i	LOC_Os03g11890.1	150	-22.88	150	-22.88	1674464	21	5003	1165
miR399i	LOC_Os03g12180.1	152	-20.15	152	-20.15	1674507	21	2587	2525
miR399i	LOC_Os03g12490.1	157	-20.13	157	-20.13	1674551	21	2292	95
miR399i	LOC_Os03g12680.1	164	-26.18	164	-26.18	1674575	21	1700	804
miR399i	LOC_Os03g12680.2	164	-26.18	164	-26.18	1674576	21	1555	659
miR399i	LOC_Os03g13080.1	155	-22.1	155	-22.1	1674634	21	1875	1020
miR399i	LOC_Os03g13460.1	145	-21.68	145	-21.68	1674677	21	3291	2566
miR399i	LOC_Os03g13460.2	145	-21.68	145	-21.68	1674678	21	3715	2990
miR399i	LOC_Os03g13960.2	155	-20.53	155	-20.53	1674741	21	1370	1046
miR399i	LOC_Os03g15270.1	168	-24.68	168	-24.68	1674901	21	1703	965
miR399i	LOC_Os03g15960.1	158	-23.5	158	-23.5	1674992	21	1035	742
miR399i	LOC_Os03g15960.2	158	-23.5	158	-23.5	1674993	21	993	700
miR399i	LOC_Os03g16540.1	154	-23.34	154	-23.34	1675077	21	1536	1162
miR399i	LOC_Os03g16700.1	153	-23.16	153	-23.16	1675094	21	2980	2616
miR399i	LOC_Os03g16700.2	153	-23.16	153	-23.16	1675095	21	2808	2444
miR399i	LOC_Os03g16700.3	153	-23.16	153	-23.16	1675096	21	2801	2437
miR399i	LOC_Os03g16700.4	153	-23.16	153	-23.16	1675097	21	2967	2603
miR399i	LOC_Os03g17020.1	154	-21.96	154	-21.96	1675145	21	1976	633
miR399i	LOC_Os03g17020.2	154	-21.96	154	-21.96	1675146	21	2171	828
miR399i	LOC_Os03g18060.1	149	-20.5	149	-20.5	1675268	21	5067	2840
miR399i	LOC_Os03g18440.1	149	-20.26	149	-20.26	1675315	21	4114	3563
miR399i	LOC_Os03g18454.1	149	-20.26	149	-20.26	1675316	21	2178	902
miR399i	LOC_Os03g18454.2	149	-20.26	149	-20.26	1675317	21	2081	902
miR399i	LOC_Os03g18470.1	468	-68.5	158	-23.78	1675318	21	1605	482 289 671
miR399i	LOC_Os03g19570.1	162	-23.16	162	-23.16	1675446	21	3258	2395
miR399i	LOC_Os03g20060.1	152	-25.97	152	-25.97	1675507	21	1887	418
miR399i	LOC_Os03g21090.1	159	-22.52	159	-22.52	1675632	21	1949	484
miR399i	LOC_Os03g22490.1	159	-26.63	159	-26.63	1675807	21	1667	973
miR399i	LOC_Os03g22490.2	159	-26.63	159	-26.63	1675808	21	1766	973
miR399i	LOC_Os03g22490.3	159	-26.63	159	-26.63	1675809	21	1731	973

miR399i	LOC_Os03g22490.4	159	-26.63	159	-26.63	1675810	21	1309	973
miR399i	LOC_Os03g22560.1	331	-52.41	166	-26.3	1675816	21	3248	891 973
miR399i	LOC_Os03g24410.1	154	-22.96	154	-22.96	1675961	21	2274	1282
miR399i	LOC_Os03g24960.1	161	-21.81	161	-21.81	1676018	21	1200	187
miR399i	LOC_Os03g25420.1	324	-47.96	162	-25.07	1676074	21	3077	1009 1045
miR399i	LOC_Os03g26044.1	167	-22.62	167	-22.62	1676140	21	3221	2645
miR399i	LOC_Os03g26080.1	162	-24.97	162	-24.97	1676143	21	1899	1052
miR399i	LOC_Os03g26630.1	146	-20.48	146	-20.48	1676190	21	2928	1099
miR399i	LOC_Os03g26630.2	146	-20.48	146	-20.48	1676191	21	3026	1197
miR399i	LOC_Os03g27310.1	151	-23.28	151	-23.28	1676262	21	935	741
miR399i	LOC_Os03g27310.2	151	-23.28	151	-23.28	1676263	21	879	685
miR399i	LOC_Os03g27390.1	149	-20.6	149	-20.6	1676278	21	2558	889
miR399i	LOC_Os03g27390.2	149	-20.6	149	-20.6	1676279	21	2581	889
miR399i	LOC_Os03g27480.1	155	-22.72	155	-22.72	1676291	21	1748	1548
miR399i	LOC_Os03g27480.2	155	-22.72	155	-22.72	1676292	21	1582	1385
miR399i	LOC_Os03g27820.1	159	-24.84	159	-24.84	1676325	21	3028	399
miR399i	LOC_Os03g27960.1	149	-26.59	149	-26.59	1676340	21	2080	559
miR399i	LOC_Os03g27960.2	149	-26.59	149	-26.59	1676341	21	2110	592
miR399i	LOC_Os03g28270.1	146	-20.91	146	-20.91	1676371	21	4432	3301
miR399i	LOC_Os03g29220.1	157	-22.97	157	-22.97	1676427	21	876	804
miR399i	LOC_Os03g29340.1	167	-24.85	167	-24.85	1676440	21	3720	2205
miR399i	LOC_Os03g29340.2	167	-24.85	167	-24.85	1676441	21	3756	2241
miR399i	LOC_Os03g29350.1	167	-24.85	167	-24.85	1676442	21	2931	880
miR399i	LOC_Os03g29350.2	167	-24.85	167	-24.85	1676443	21	2984	880
miR399i	LOC_Os03g29350.4	167	-24.85	167	-24.85	1676444	21	2923	880
miR399i	LOC_Os03g29520.1	147	-21.73	147	-21.73	1676458	21	5769	1648
miR399i	LOC_Os03g30510.1	160	-29.39	160	-29.39	1676553	21	2442	690
miR399i	LOC_Os03g31520.1	165	-21.22	165	-21.22	1676665	21	1536	477
miR399i	LOC_Os03g31750.1	158	-23.46	158	-23.46	1676690	21	3082	2357
miR399i	LOC_Os03g31750.2	158	-23.46	158	-23.46	1676691	21	2743	2357
miR399i	LOC_Os03g31750.3	158	-23.46	158	-23.46	1676692	21	3097	2372
miR399i	LOC_Os03g31986.1	140	-20.83	140	-20.83	1676709	21	3954	2861

miR399i	LOC_Os03g32620.1	149	-21.19	149	-21.19	1676766	21	4010	3637
miR399i	LOC_Os03g33012.1	149	-22.08	149	-22.08	1676804	21	1926	1862
miR399i	LOC_Os03g33770.1	149	-28.65	149	-28.65	1676877	21	2397	298
miR399i	LOC_Os03g34102.1	149	-20.48	149	-20.48	1676908	21	1107	37
miR399i	LOC_Os03g34170.1	152	-23.77	152	-23.77	1676914	21	1167	1006
miR399i	LOC_Os03g34270.1	148	-21.42	148	-21.42	1676924	21	2796	285
miR399i	LOC_Os03g35390.1	155	-23.01	155	-23.01	1676944	21	5727	639
miR399i	LOC_Os03g35540.1	152	-20.4	152	-20.4	1676956	21	4707	3580
miR399i	LOC_Os03g35886.1	155	-25.16	155	-25.16	1676983	21	4339	182
miR399i	LOC_Os03g36560.1	141	-22.89	141	-22.89	1677042	21	1497	1150
miR399i	LOC_Os03g36570.1	143	-21.52	143	-21.52	1677043	21	6849	3642
miR399i	LOC_Os03g37500.1	153	-20.65	153	-20.65	1677123	21	4860	3536
miR399i	LOC_Os03g37720.1	161	-21.12	161	-21.12	1677143	21	3801	1372
miR399i	LOC_Os03g37760.1	163	-25.4	163	-25.4	1677148	21	411	61
miR399i	LOC_Os03g38740.1	160	-21.09	160	-21.09	1677253	21	5009	1403
miR399i	LOC_Os03g38750.1	159	-20.38	159	-20.38	1677255	21	414	144
miR399i	LOC_Os03g39730.1	157	-25.26	157	-25.26	1677355	21	1272	947
miR399i	LOC_Os03g39780.1	146	-21.78	146	-21.78	1677359	21	2388	1337
miR399i	LOC_Os03g39850.1	161	-20.64	161	-20.64	1677363	21	902	219
miR399i	LOC_Os03g40930.1	151	-24.35	151	-24.35	1677456	21	2045	776
miR399i	LOC_Os03g41380.1	165	-23.13	165	-23.13	1677496	21	861	548
miR399i	LOC_Os03g41510.1	146	-22.09	146	-22.09	1677505	21	1670	1328
miR399i	LOC_Os03g41510.2	146	-22.09	146	-22.09	1677506	21	1690	1348
miR399i	LOC_Os03g41510.3	146	-22.09	146	-22.09	1677507	21	1698	1371
miR399i	LOC_Os03g41510.4	146	-22.09	146	-22.09	1677508	21	1641	1299
miR399i	LOC_Os03g41510.5	146	-22.09	146	-22.09	1677509	21	1683	1341
miR399i	LOC_Os03g41510.6	146	-22.09	146	-22.09	1677510	21	1646	1319
miR399i	LOC_Os03g41510.7	146	-22.09	146	-22.09	1677511	21	1757	1430
miR399i	LOC_Os03g41510.8	146	-22.09	146	-22.09	1677512	21	1991	1649
miR399i	LOC_Os03g41710.1	157	-20.24	157	-20.24	1677530	21	1881	107
miR399i	LOC_Os03g41830.1	155	-24.7	155	-24.7	1677540	21	2730	969
miR399i	LOC_Os03g42180.1	152	-21.52	152	-21.52	1677573	21	369	307

miR399i	LOC_Os03g43800.1	152	-20.17	152	-20.17	1677743	21	4253	945
miR399i	LOC_Os03g43800.2	152	-20.17	152	-20.17	1677744	21	4224	945
miR399i	LOC_Os03g44240.1	154	-22.64	154	-22.64	1677790	21	3066	2870
miR399i	LOC_Os03g44530.1	148	-20.87	148	-20.87	1677823	21	2162	1329
miR399i	LOC_Os03g44530.2	148	-20.87	148	-20.87	1677824	21	2123	1290
miR399i	LOC_Os03g45220.1	145	-24.36	145	-24.36	1677904	21	604	199
miR399i	LOC_Os03g45970.1	157	-21.18	157	-21.18	1677972	21	2513	1780
miR399i	LOC_Os03g45970.2	157	-21.18	157	-21.18	1677973	21	2190	1457
miR399i	LOC_Os03g45970.3	157	-21.18	157	-21.18	1677974	21	2358	1625
miR399i	LOC_Os03g45990.1	168	-30.2	168	-30.2	1677976	21	4865	4568
miR399i	LOC_Os03g46340.1	155	-21.23	155	-21.23	1678017	21	3131	2658
miR399i	LOC_Os03g46930.1	147	-23.52	147	-23.52	1678079	21	384	307
miR399i	LOC_Os03g47390.1	163	-23.94	163	-23.94	1678124	21	282	163
miR399i	LOC_Os03g47650.1	159	-20.9	159	-20.9	1678159	21	1824	415
miR399i	LOC_Os03g48450.1	148	-20.17	148	-20.17	1678249	21	3638	275
miR399i	LOC_Os03g48450.3	148	-20.17	148	-20.17	1678251	21	3022	275
miR399i	LOC_Os03g48450.4	148	-20.17	148	-20.17	1678252	21	3112	275
miR399i	LOC_Os03g48970.1	156	-21.44	156	-21.44	1678315	21	1594	497
miR399i	LOC_Os03g48970.2	156	-21.44	156	-21.44	1678316	21	2123	1026
miR399i	LOC_Os03g48970.3	156	-21.44	156	-21.44	1678317	21	2293	1196
miR399i	LOC_Os03g48970.4	156	-21.44	156	-21.44	1678318	21	1586	497
miR399i	LOC_Os03g49340.1	160	-21.42	160	-21.42	1678359	21	1698	994
miR399i	LOC_Os03g49524.1	159	-20.8	159	-20.8	1678387	21	2574	332
miR399i	LOC_Os03g49640.1	152	-20.21	152	-20.21	1678409	21	2537	1644
miR399i	LOC_Os03g49640.2	152	-20.21	152	-20.21	1678410	21	2631	1738
miR399i	LOC_Os03g50670.1	158	-21.12	158	-21.12	1678525	21	4350	1346
miR399i	LOC_Os03g51440.1	155	-21.25	155	-21.25	1678603	21	3315	1375
miR399i	LOC_Os03g51570.1	160	-25.05	160	-25.05	1678622	21	255	197
miR399i	LOC_Os03g51990.1	158	-26.37	158	-26.37	1678670	21	1656	1616
miR399i	LOC_Os03g52700.1	156	-24.76	156	-24.76	1678755	21	3359	2829
miR399i	LOC_Os03g52780.1	156	-21.36	156	-21.36	1678763	21	1886	1238
miR399i	LOC_Os03g52950.1	159	-25.46	159	-25.46	1678781	21	3183	2179

miR399i	LOC_Os03g53280.1	158	-21.12	158	-21.12	1678819	21	11747	864
miR399i	LOC_Os03g53750.1	160	-24.04	160	-24.04	1678878	21	1878	537
miR399i	LOC_Os03g53750.2	160	-24.04	160	-24.04	1678879	21	1861	537
miR399i	LOC_Os03g55820.1	150	-20.07	150	-20.07	1679079	21	1361	1039
miR399i	LOC_Os03g55890.1	150	-21.36	150	-21.36	1679088	21	2451	1760
miR399i	LOC_Os03g56530.1	147	-20.6	147	-20.6	1679167	21	4185	3300
miR399i	LOC_Os03g56572.1	161	-21.95	161	-21.95	1679171	21	3957	2690
miR399i	LOC_Os03g56890.1	155	-21.96	155	-21.96	1679217	21	767	713
miR399i	LOC_Os03g58190.1	166	-25.9	166	-25.9	1679393	21	1542	1466
miR399i	LOC_Os03g58240.1	157	-21.05	157	-21.05	1679398	21	2646	920
miR399i	LOC_Os03g58490.1	153	-22.09	153	-22.09	1679423	21	2903	537
miR399i	LOC_Os03g58490.2	153	-22.09	153	-22.09	1679424	21	2935	527
miR399i	LOC_Os03g58890.1	158	-20.25	158	-20.25	1679467	21	1162	537
miR399i	LOC_Os03g58890.2	158	-20.25	158	-20.25	1679468	21	1168	537
miR399i	LOC_Os03g59680.1	163	-22.43	163	-22.43	1679552	21	2266	949
miR399i	LOC_Os03g59730.1	146	-20.82	146	-20.82	1679557	21	1491	1427
miR399i	LOC_Os03g60460.1	151	-22.5	151	-22.5	1679634	21	2197	1315
miR399i	LOC_Os03g60740.1	155	-21.57	155	-21.57	1679669	21	1114	721
miR399i	LOC_Os03g60750.1	161	-20.64	161	-20.64	1679670	21	1399	1121
miR399i	LOC_Os03g60750.2	161	-20.64	161	-20.64	1679671	21	1488	1210
miR399i	LOC_Os03g60750.3	161	-20.64	161	-20.64	1679672	21	1474	1196
miR399i	LOC_Os03g60920.1	158	-21.87	158	-21.87	1679693	21	1431	940
miR399i	LOC_Os03g61130.1	163	-26.69	163	-26.69	1679714	21	2206	795
miR399i	LOC_Os03g61380.1	153	-24.9	153	-24.9	1679752	21	987	879
miR399i	LOC_Os03g61860.1	143	-21.55	143	-21.55	1679807	21	630	324
miR399i	LOC_Os03g61950.1	155	-20.65	155	-20.65	1679820	21	2690	464
miR399i	LOC_Os03g61950.2	155	-20.65	155	-20.65	1679821	21	3095	464
miR399i	LOC_Os03g62314.1	154	-20.8	154	-20.8	1679874	21	2010	1578
miR399i	LOC_Os03g64210.1	157	-20.51	157	-20.51	1680119	21	2439	523
miR399i	LOC_Os03g64210.2	157	-20.51	157	-20.51	1680120	21	2599	523
miR399i	LOC_Os03g64210.3	157	-20.51	157	-20.51	1680121	21	2401	523
miR399i	LOC_Os04g01240.1	162	-20.91	162	-20.91	1680178	21	1043	346

miR399i	LOC_Os04g01340.1	153	-24.96	153	-24.96	1680195	21	2100	713
miR399i	LOC_Os04g02140.1	171	-29.98	171	-29.98	1680283	21	1958	1497
miR399i	LOC_Os04g02500.1	149	-22.06	149	-22.06	1680317	21	1751	41
miR399i	LOC_Os04g02820.1	156	-22.79	156	-22.79	1680352	21	3228	1578
miR399i	LOC_Os04g02820.2	156	-22.79	156	-22.79	1680353	21	3000	1350
miR399i	LOC_Os04g02820.3	156	-22.79	156	-22.79	1680354	21	2556	1352
miR399i	LOC_Os04g02860.1	153	-20.63	153	-20.63	1680358	21	2928	1131
miR399i	LOC_Os04g03110.1	141	-25.41	141	-25.41	1680386	21	4098	3435
miR399i	LOC_Os04g03230.1	155	-23.06	155	-23.06	1680397	21	525	47
miR399i	LOC_Os04g03300.1	155	-23.06	155	-23.06	1680404	21	3981	3949
miR399i	LOC_Os04g03630.1	162	-20.75	162	-20.75	1680433	21	4899	3592
miR399i	LOC_Os04g03950.1	157	-22.93	157	-22.93	1680465	21	2376	1321
miR399i	LOC_Os04g04020.1	156	-26.5	156	-26.5	1680473	21	3676	468
miR399i	LOC_Os04g05180.1	155	-24.66	155	-24.66	1680582	21	1530	1142
miR399i	LOC_Os04g05210.1	156	-20.19	156	-20.19	1680585	21	2511	2084
miR399i	LOC_Os04g05470.1	149	-23.2	149	-23.2	1680608	21	2076	1435
miR399i	LOC_Os04g06610.1	159	-20.27	159	-20.27	1680716	21	4362	1664
miR399i	LOC_Os04g07020.1	153	-21.08	153	-21.08	1680753	21	5898	4099
miR399i	LOC_Os04g07300.1	153	-21.08	153	-21.08	1680777	21	5997	4201
miR399i	LOC_Os04g07400.1	162	-21.61	162	-21.61	1680787	21	816	262
miR399i	LOC_Os04g07510.1	151	-21.76	151	-21.76	1680797	21	4209	2122
miR399i	LOC_Os04g09284.1	156	-22.81	156	-22.81	1680946	21	4470	3382
miR399i	LOC_Os04g09350.1	167	-22.11	167	-22.11	1680951	21	5391	1297
miR399i	LOC_Os04g09380.1	153	-20.49	153	-20.49	1680957	21	2934	40
miR399i	LOC_Os04g09690.1	165	-25.01	165	-25.01	1680984	21	2172	268
miR399i	LOC_Os04g09720.1	159	-23.94	159	-23.94	1680987	21	1693	277
miR399i	LOC_Os04g09720.2	159	-23.94	159	-23.94	1680988	21	1615	197
miR399i	LOC_Os04g09750.1	150	-23.56	150	-23.56	1680991	21	903	644
miR399i	LOC_Os04g10090.1	149	-20.48	149	-20.48	1681023	21	447	37
miR399i	LOC_Os04g10200.1	153	-20.56	153	-20.56	1681034	21	5976	4192
miR399i	LOC_Os04g10850.1	153	-20.56	153	-20.56	1681099	21	5592	3853
miR399i	LOC_Os04g11090.1	155	-23.01	155	-23.01	1681120	21	3876	645

miR399i	LOC_Os04g11330.1	146	-24.06	146	-24.06	1681145	21	924	229
miR399i	LOC_Os04g11620.1	311	-40.89	157	-20.46	1681173	21	5544	5421 5376
miR399i	LOC_Os04g12130.1	327	-49.04	164	-27.61	1681226	21	3024	2468 1101
miR399i	LOC_Os04g12720.1	163	-23.46	163	-23.46	1681279	21	1732	973
miR399i	LOC_Os04g12950.1	163	-23.46	163	-23.46	1681295	21	1266	661
miR399i	LOC_Os04g12970.1	163	-23.46	163	-23.46	1681297	21	1841	1092
miR399i	LOC_Os04g13010.1	150	-21.77	150	-21.77	1681301	21	846	129
miR399i	LOC_Os04g13030.1	153	-20.56	153	-20.56	1681303	21	6027	4243
miR399i	LOC_Os04g13080.1	166	-27.18	166	-27.18	1681308	21	1815	1422
miR399i	LOC_Os04g13510.1	162	-22.42	162	-22.42	1681347	21	4560	4490
miR399i	LOC_Os04g13560.1	156	-21.19	156	-21.19	1681352	21	1548	796
miR399i	LOC_Os04g13750.1	157	-27.94	157	-27.94	1681371	21	2719	2349
miR399i	LOC_Os04g14000.1	153	-23.45	153	-23.45	1681396	21	1881	118
miR399i	LOC_Os04g14510.1	168	-25.09	168	-25.09	1681449	21	3582	1485
miR399i	LOC_Os04g14510.2	168	-25.09	168	-25.09	1681450	21	3625	1485
miR399i	LOC_Os04g14654.1	159	-20.71	159	-20.71	1681464	21	2328	839
miR399i	LOC_Os04g14654.2	159	-20.71	159	-20.71	1681465	21	2338	839
miR399i	LOC_Os04g14654.3	159	-20.71	159	-20.71	1681466	21	2240	751
miR399i	LOC_Os04g17130.1	151	-20.78	151	-20.78	1681689	21	4533	4029
miR399i	LOC_Os04g17200.1	156	-20.44	156	-20.44	1681696	21	534	185
miR399i	LOC_Os04g17320.1	140	-23.66	140	-23.66	1681708	21	4863	972
miR399i	LOC_Os04g17410.1	156	-24.57	156	-24.57	1681717	21	4368	2788
miR399i	LOC_Os04g17650.1	151	-20.89	151	-20.89	1681739	21	2633	1577
miR399i	LOC_Os04g18570.1	163	-21.83	163	-21.83	1681830	21	3039	425
miR399i	LOC_Os04g18770.1	140	-23.66	140	-23.66	1681850	21	4650	2475
miR399i	LOC_Os04g18884.1	140	-20.45	140	-20.45	1681861	21	1788	1083
miR399i	LOC_Os04g19170.1	160	-22.64	160	-22.64	1681888	21	1083	970
miR399i	LOC_Os04g20810.1	157	-20.43	157	-20.43	1682050	21	1359	1267
miR399i	LOC_Os04g20810.2	157	-20.43	157	-20.43	1682051	21	1418	1326
miR399i	LOC_Os04g20810.3	157	-20.43	157	-20.43	1682052	21	1240	1148
miR399i	LOC_Os04g21100.1	154	-22.84	154	-22.84	1682076	21	2229	1874
miR399i	LOC_Os04g22500.1	157	-22.55	157	-22.55	1682215	21	1809	1545

miR399i	LOC_Os04g22580.1	149	-21.16	149	-21.16	1682221	21	2973	1479
miR399i	LOC_Os04g22960.1	160	-21.87	160	-21.87	1682257	21	1833	78
miR399i	LOC_Os04g23310.1	153	-20.56	153	-20.56	1682289	21	5211	3712
miR399i	LOC_Os04g23799.1	157	-21.58	157	-21.58	1682329	21	3915	2395
miR399i	LOC_Os04g24290.1	157	-24.79	157	-24.79	1682378	21	1611	371
miR399i	LOC_Os04g24590.1	153	-20.56	153	-20.56	1682413	21	5979	4198
miR399i	LOC_Os04g24700.1	173	-25.97	173	-25.97	1682424	21	2280	1255
miR399i	LOC_Os04g24980.1	147	-22.07	147	-22.07	1682450	21	966	547
miR399i	LOC_Os04g25020.1	147	-22.07	147	-22.07	1682456	21	5466	5047
miR399i	LOC_Os04g25030.1	147	-22.07	147	-22.07	1682457	21	5196	4945
miR399i	LOC_Os04g26460.1	155	-20.54	155	-20.54	1682603	21	321	77
miR399i	LOC_Os04g28090.1	151	-20.24	151	-20.24	1682756	21	3576	1567
miR399i	LOC_Os04g28450.1	167	-28.53	167	-28.53	1682790	21	450	242
miR399i	LOC_Os04g28560.1	142	-22.13	142	-22.13	1682802	21	4824	2260
miR399i	LOC_Os04g28880.1	165	-24.86	165	-24.86	1682834	21	4584	1019
miR399i	LOC_Os04g28900.1	153	-23.38	153	-23.38	1682836	21	2442	843
miR399i	LOC_Os04g29500.1	155	-24.66	155	-24.66	1682886	21	1165	848
miR399i	LOC_Os04g30010.1	160	-22.38	160	-22.38	1682933	21	2522	2372
miR399i	LOC_Os04g30210.1	157	-20.21	157	-20.21	1682952	21	2140	1203
miR399i	LOC_Os04g30230.1	157	-20.21	157	-20.21	1682954	21	909	817
miR399i	LOC_Os04g30320.1	157	-20.21	157	-20.21	1682965	21	1849	1102
miR399i	LOC_Os04g30530.1	159	-22.11	159	-22.11	1682984	21	1704	1585
miR399i	LOC_Os04g30610.1	159	-21.61	159	-21.61	1682992	21	1518	1021
miR399i	LOC_Os04g30660.1	159	-22.11	159	-22.11	1682997	21	3231	1276
miR399i	LOC_Os04g30690.1	159	-22.11	159	-22.11	1683000	21	2811	1276
miR399i	LOC_Os04g30860.1	163	-23.26	163	-23.26	1683020	21	2928	826
miR399i	LOC_Os04g30930.1	147	-20.35	147	-20.35	1683029	21	3240	1663
miR399i	LOC_Os04g31030.1	154	-22.01	154	-22.01	1683040	21	1858	138
miR399i	LOC_Os04g32270.1	150	-22.6	150	-22.6	1683167	21	978	355
miR399i	LOC_Os04g32440.1	154	-20.65	154	-20.65	1683185	21	1122	283
miR399i	LOC_Os04g32650.1	159	-22.41	159	-22.41	1683209	21	2496	1549
miR399i	LOC_Os04g32920.1	161	-20.14	161	-20.14	1683242	21	3094	99

miR399i	LOC_Os04g32920.2	161	-20.14	161	-20.14	1683243	21	3174	99
miR399i	LOC_Os04g32920.3	161	-20.14	161	-20.14	1683244	21	3069	101
miR399i	LOC_Os04g32920.5	161	-20.14	161	-20.14	1683246	21	3082	99
miR399i	LOC_Os04g33120.1	150	-27.61	150	-27.61	1683272	21	2805	269
miR399i	LOC_Os04g33160.1	168	-27.15	168	-27.15	1683276	21	399	174
miR399i	LOC_Os04g33360.1	157	-20.39	157	-20.39	1683300	21	1814	133
miR399i	LOC_Os04g33690.1	147	-20.68	147	-20.68	1683339	21	2798	2748
miR399i	LOC_Os04g34080.1	162	-21.91	162	-21.91	1683379	21	2774	1430
miR399i	LOC_Os04g34260.1	153	-20.44	153	-20.44	1683397	21	4551	3810
miR399i	LOC_Os04g34290.1	154	-22.31	154	-22.31	1683400	21	2451	806
miR399i	LOC_Os04g35090.1	298	-41.85	157	-21.18	1683481	21	552	478 502
miR399i	LOC_Os04g35260.1	152	-22.43	152	-22.43	1683499	21	4533	3377
miR399i	LOC_Os04g35800.1	159	-25.77	159	-25.77	1683567	21	2169	987
miR399i	LOC_Os04g35900.1	161	-24.11	161	-24.11	1683578	21	1988	1170
miR399i	LOC_Os04g35920.2	154	-20.05	154	-20.05	1683580	21	2643	1615
miR399i	LOC_Os04g35940.1	150	-20.26	150	-20.26	1683582	21	1638	437
miR399i	LOC_Os04g36720.1	161	-22.11	161	-22.11	1683616	21	2761	394
miR399i	LOC_Os04g36740.1	150	-22.9	150	-22.9	1683618	21	2303	2103
miR399i	LOC_Os04g37970.1	165	-24.67	165	-24.67	1683703	21	1563	100
miR399i	LOC_Os04g38420.1	154	-23.35	154	-23.35	1683747	21	1289	33
miR399i	LOC_Os04g38630.1	313	-44.15	158	-22.65	1683775	21	2789	748 920
miR399i	LOC_Os04g38940.1	165	-23.5	165	-23.5	1683817	21	1212	1081
miR399i	LOC_Os04g38940.2	165	-23.5	165	-23.5	1683818	21	1344	1213
miR399i	LOC_Os04g39190.1	168	-28.42	168	-28.42	1683850	21	3530	2171
miR399i	LOC_Os04g40020.1	154	-25.8	154	-25.8	1683946	21	858	750
miR399i	LOC_Os04g41220.1	153	-23.46	153	-23.46	1684104	21	3158	2703
miR399i	LOC_Os04g41560.2	155	-22.17	155	-22.17	1684140	21	1272	366
miR399i	LOC_Os04g41560.4	155	-22.17	155	-22.17	1684141	21	1367	366
miR399i	LOC_Os04g41690.1	167	-28.81	167	-28.81	1684154	21	3324	3130
miR399i	LOC_Os04g42100.1	150	-27.64	150	-27.64	1684203	21	2247	811
miR399i	LOC_Os04g42610.1	147	-20.7	147	-20.7	1684269	21	2784	759
miR399i	LOC_Os04g43150.1	154	-20.1	154	-20.1	1684334	21	2004	1330

miR399i	LOC_Os04g43150.2	154	-20.1	154	-20.1	1684335	21	2787	1434
miR399i	LOC_Os04g43164.1	152	-21.21	152	-21.21	1684337	21	1378	888
miR399i	LOC_Os04g43164.2	152	-21.21	152	-21.21	1684338	21	1536	1051
miR399i	LOC_Os04g43220.1	162	-20.18	162	-20.18	1684347	21	2911	1887
miR399i	LOC_Os04g43220.2	162	-20.18	162	-20.18	1684348	21	2950	1812
miR399i	LOC_Os04g43220.3	162	-20.18	162	-20.18	1684349	21	2759	1812
miR399i	LOC_Os04g43220.4	162	-20.18	162	-20.18	1684350	21	4011	1873
miR399i	LOC_Os04g43230.1	157	-25.87	157	-25.87	1684351	21	1703	628
miR399i	LOC_Os04g43230.2	157	-25.87	157	-25.87	1684352	21	1778	703
miR399i	LOC_Os04g44900.1	156	-25.14	156	-25.14	1684547	21	2558	1559
miR399i	LOC_Os04g45130.1	161	-22.74	161	-22.74	1684581	21	752	212
miR399i	LOC_Os04g45970.1	153	-25.25	153	-25.25	1684673	21	2102	524
miR399i	LOC_Os04g45970.2	153	-25.25	153	-25.25	1684674	21	1508	525
miR399i	LOC_Os04g45990.1	162	-23.02	162	-23.02	1684676	21	2714	1573
miR399i	LOC_Os04g46710.1	151	-21.94	151	-21.94	1684763	21	696	79
miR399i	LOC_Os04g46860.1	165	-22.68	165	-22.68	1684786	21	2663	264
miR399i	LOC_Os04g49240.1	161	-20.12	161	-20.12	1685070	21	450	52
miR399i	LOC_Os04g49290.1	150	-21.21	150	-21.21	1685075	21	4335	3191
miR399i	LOC_Os04g49530.1	155	-20.13	155	-20.13	1685102	21	2175	2136
miR399i	LOC_Os04g49540.1	155	-20.13	155	-20.13	1685103	21	2011	1101
miR399i	LOC_Os04g50830.1	158	-20.67	158	-20.67	1685207	21	3570	1831
miR399i	LOC_Os04g51120.1	150	-20.36	150	-20.36	1685241	21	2391	1674
miR399i	LOC_Os04g51120.3	150	-20.36	150	-20.36	1685242	21	2623	1906
miR399i	LOC_Os04g51454.1	154	-20.48	154	-20.48	1685290	21	516	151
miR399i	LOC_Os04g51650.1	166	-28.78	166	-28.78	1685314	21	309	225
miR399i	LOC_Os04g51809.1	158	-22.69	158	-22.69	1685336	21	2132	1662
miR399i	LOC_Os04g51809.3	158	-22.69	158	-22.69	1685338	21	1658	1188
miR399i	LOC_Os04g52380.1	155	-21.83	155	-21.83	1685409	21	2155	402
miR399i	LOC_Os04g52380.2	155	-21.83	155	-21.83	1685410	21	3260	402
miR399i	LOC_Os04g52380.3	155	-21.83	155	-21.83	1685411	21	2083	402
miR399i	LOC_Os04g52380.4	155	-21.83	155	-21.83	1685412	21	2048	402
miR399i	LOC_Os04g52390.1	155	-20.48	155	-20.48	1685413	21	3493	3430

miR399i	LOC_Os04g53660.1	150	-20.51	150	-20.51	1685577	21	3132	125
miR399i	LOC_Os04g53660.3	150	-20.51	150	-20.51	1685579	21	3047	107
miR399i	LOC_Os04g53890.1	153	-20.44	153	-20.44	1685607	21	4551	3810
miR399i	LOC_Os04g53900.1	155	-24.66	155	-24.66	1685608	21	1284	812
miR399i	LOC_Os04g53990.1	155	-20.6	155	-20.6	1685620	21	1976	778
miR399i	LOC_Os04g54710.1	152	-20.95	152	-20.95	1685705	21	2139	67
miR399i	LOC_Os04g55140.1	325	-44.27	164	-22.84	1685753	21	3546	2990 1131
miR399i	LOC_Os04g55190.1	150	-21.52	150	-21.52	1685759	21	3245	2953
miR399i	LOC_Os04g55230.1	182	-33.21	182	-33.21	1685763	21	5705	4032
miR399i	LOC_Os04g55330.1	147	-25.24	147	-25.24	1685777	21	588	25
miR399i	LOC_Os04g55960.1	154	-22.45	154	-22.45	1685849	21	2578	1207
miR399i	LOC_Os04g56160.1	157	-20.7	157	-20.7	1685874	21	3639	3501
miR399i	LOC_Os04g56160.2	157	-20.7	157	-20.7	1685875	21	2393	2257
miR399i	LOC_Os04g56160.3	157	-20.7	157	-20.7	1685876	21	3417	3279
miR399i	LOC_Os04g56510.1	154	-20.06	154	-20.06	1685919	21	2865	336
miR399i	LOC_Os04g58250.1	152	-25.82	152	-25.82	1686152	21	2608	1667
miR399i	LOC_Os04g58410.1	169	-24.8	169	-24.8	1686170	21	3345	2619
miR399i	LOC_Os04g58410.2	169	-24.8	169	-24.8	1686171	21	3440	2697
miR399i	LOC_Os04g58470.1	152	-23.14	152	-23.14	1686178	21	2522	2004
miR399i	LOC_Os04g58504.1	152	-23.14	152	-23.14	1686181	21	2463	1973
miR399i	LOC_Os04g58600.2	145	-22.75	145	-22.75	1686196	21	1643	969
miR399i	LOC_Os04g58840.1	163	-26.75	163	-26.75	1686231	21	2098	1957
miR399i	LOC_Os04g58970.1	158	-23.55	158	-23.55	1686244	21	608	519
miR399i	LOC_Os04g58980.1	158	-23.55	158	-23.55	1686245	21	2649	344
miR399i	LOC_Os04g59090.1	162	-24.61	162	-24.61	1686258	21	1947	1592
miR399i	LOC_Os04g59180.1	153	-20.56	153	-20.56	1686268	21	5457	3661
miR399i	LOC_Os04g59320.1	147	-22.4	147	-22.4	1686282	21	2756	1967
miR399i	LOC_Os04g59540.1	167	-26.38	167	-26.38	1686308	21	4528	2231
miR399i	LOC_Os04g59624.2	157	-24.9	157	-24.9	1686328	21	3412	2627
miR399i	LOC_Os04g59624.3	157	-24.9	157	-24.9	1686329	21	3481	2630
miR399i	LOC_Os05g01030.1	159	-23.71	159	-23.71	1686344	21	3570	920
miR399i	LOC_Os05g01350.1	153	-22.33	153	-22.33	1686394	21	3053	2085

miR399i	LOC_Os05g01520.1	156	-20.28	156	-20.28	1686420	21	2496	1622
miR399i	LOC_Os05g02040.1	144	-20.6	144	-20.6	1686489	21	3409	904
miR399i	LOC_Os05g02340.1	157	-21.13	157	-21.13	1686527	21	4074	2678
miR399i	LOC_Os05g02790.1	165	-25.25	165	-25.25	1686584	21	3105	141
miR399i	LOC_Os05g03000.1	141	-20.24	141	-20.24	1686604	21	1818	1008
miR399i	LOC_Os05g03000.2	141	-20.24	141	-20.24	1686605	21	1835	1045
miR399i	LOC_Os05g03310.1	157	-20.65	157	-20.65	1686642	21	1989	385
miR399i	LOC_Os05g03400.1	164	-21.09	164	-21.09	1686654	21	3112	1577
miR399i	LOC_Os05g03420.1	298	-41.66	150	-21.49	1686656	21	912	773 696
miR399i	LOC_Os05g03480.2	156	-20.09	156	-20.09	1686663	21	1654	517
miR399i	LOC_Os05g03480.4	156	-20.09	156	-20.09	1686664	21	1644	517
miR399i	LOC_Os05g03480.5	156	-20.09	156	-20.09	1686665	21	1185	517
miR399i	LOC_Os05g03650.1	153	-24.37	153	-24.37	1686685	21	3084	516
miR399i	LOC_Os05g03810.1	169	-26.08	169	-26.08	1686700	21	819	472
miR399i	LOC_Os05g04420.1	156	-20.19	156	-20.19	1686770	21	5379	2522
miR399i	LOC_Os05g04520.1	157	-22.51	157	-22.51	1686781	21	1632	501
miR399i	LOC_Os05g04980.1	153	-21.08	153	-21.08	1686830	21	5910	4111
miR399i	LOC_Os05g05130.1	163	-29.25	163	-29.25	1686845	21	1350	674
miR399i	LOC_Os05g05310.1	152	-20.37	152	-20.37	1686867	21	3863	2869
miR399i	LOC_Os05g05310.2	152	-20.37	152	-20.37	1686868	21	3859	2869
miR399i	LOC_Os05g05490.1	170	-28.1	170	-28.1	1686890	21	2268	1468
miR399i	LOC_Os05g05620.1	152	-23.16	152	-23.16	1686909	21	700	195
miR399i	LOC_Os05g06280.1	147	-22.03	147	-22.03	1686981	21	3584	285
miR399i	LOC_Os05g06280.2	147	-22.03	147	-22.03	1686982	21	3599	573
miR399i	LOC_Os05g06330.1	160	-22.36	160	-22.36	1686989	21	1404	428
miR399i	LOC_Os05g06910.1	168	-23.65	168	-23.65	1687060	21	1787	1607
miR399i	LOC_Os05g07020.1	143	-20.74	143	-20.74	1687071	21	585	532
miR399i	LOC_Os05g07560.1	157	-21.98	157	-21.98	1687133	21	4291	217
miR399i	LOC_Os05g08310.1	158	-20.06	158	-20.06	1687222	21	4404	3775
miR399i	LOC_Os05g09420.1	157	-23.48	157	-23.48	1687359	21	516	486
miR399i	LOC_Os05g10440.1	164	-27.68	164	-27.68	1687425	21	2346	1204
miR399i	LOC_Os05g10590.1	149	-24.34	149	-24.34	1687441	21	2052	1851

miR399i	LOC_Os05g10590.2	149	-24.34	149	-24.34	1687442	21	1126	843
miR399i	LOC_Os05g10800.1	156	-20.72	156	-20.72	1687471	21	1417	1
miR399i	LOC_Os05g11400.1	167	-28.59	167	-28.59	1687538	21	1329	514
miR399i	LOC_Os05g12570.1	333	-51.88	173	-28.01	1687661	21	3315	1117 669
miR399i	LOC_Os05g12804.1	173	-27.8	173	-27.8	1687688	21	1659	1344
miR399i	LOC_Os05g13830.1	312	-46.17	161	-24.45	1687744	21	762	388 329
miR399i	LOC_Os05g13850.1	161	-24.45	161	-24.45	1687746	21	762	388
miR399i	LOC_Os05g13940.1	616	-84.38	155	-21.79	1687756	21	1900	912 1050 897 1035
miR399i	LOC_Os05g15530.1	155	-25.76	155	-25.76	1687925	21	1328	773
miR399i	LOC_Os05g15920.1	149	-21.11	149	-21.11	1687964	21	1169	248
miR399i	LOC_Os05g15990.1	153	-21.08	153	-21.08	1687971	21	5940	4144
miR399i	LOC_Os05g16410.1	159	-23.38	159	-23.38	1688012	21	1377	970
miR399i	LOC_Os05g16700.1	153	-20.56	153	-20.56	1688044	21	1785	10
miR399i	LOC_Os05g17400.1	161	-25.05	161	-25.05	1688114	21	921	716
miR399i	LOC_Os05g17650.1	155	-22.21	155	-22.21	1688141	21	1902	704
miR399i	LOC_Os05g18294.2	159	-22.76	159	-22.76	1688205	21	1660	870
miR399i	LOC_Os05g18294.3	159	-22.76	159	-22.76	1688206	21	1380	870
miR399i	LOC_Os05g18930.1	161	-21.95	161	-21.95	1688273	21	2118	404
miR399i	LOC_Os05g19010.1	150	-20.1	150	-20.1	1688281	21	937	191
miR399i	LOC_Os05g19050.1	157	-20.82	157	-20.82	1688286	21	4152	3294
miR399i	LOC_Os05g19330.1	153	-20.44	153	-20.44	1688314	21	4488	3747
miR399i	LOC_Os05g19400.1	165	-21.22	165	-21.22	1688320	21	1152	582
miR399i	LOC_Os05g19910.1	157	-21.78	157	-21.78	1688376	21	1602	271
miR399i	LOC_Os05g20000.1	157	-23.7	157	-23.7	1688389	21	3147	481
miR399i	LOC_Os05g20050.1	172	-29.16	172	-29.16	1688394	21	1209	542
miR399i	LOC_Os05g20050.2	172	-29.16	172	-29.16	1688395	21	1119	542
miR399i	LOC_Os05g20210.1	158	-27.35	158	-27.35	1688410	21	759	286
miR399i	LOC_Os05g20270.1	155	-20.23	155	-20.23	1688416	21	2604	2366
miR399i	LOC_Os05g20360.1	161	-23.19	161	-23.19	1688425	21	4542	137
miR399i	LOC_Os05g20640.1	149	-20.48	149	-20.48	1688453	21	447	37
miR399i	LOC_Os05g20730.1	149	-20.48	149	-20.48	1688462	21	1746	37
miR399i	LOC_Os05g21090.1	149	-20.48	149	-20.48	1688504	21	1419	771

miR399i	LOC_Os05g22300.1	146	-24.14	146	-24.14	1688527	21	1395	969
miR399i	LOC_Os05g22500.1	153	-21.13	153	-21.13	1688548	21	1845	37
miR399i	LOC_Os05g22660.1	171	-25.65	171	-25.65	1688564	21	2568	90
miR399i	LOC_Os05g22920.1	164	-25.28	164	-25.28	1688593	21	2405	1171
miR399i	LOC_Os05g22920.2	164	-25.28	164	-25.28	1688594	21	2272	1080
miR399i	LOC_Os05g22920.4	164	-25.28	164	-25.28	1688595	21	2031	1171
miR399i	LOC_Os05g23240.1	168	-26.96	168	-26.96	1688628	21	894	273
miR399i	LOC_Os05g23260.1	145	-20.59	145	-20.59	1688631	21	1181	487
miR399i	LOC_Os05g23290.1	161	-20.87	161	-20.87	1688634	21	2946	2626
miR399i	LOC_Os05g23700.1	154	-23.42	154	-23.42	1688686	21	1393	226
miR399i	LOC_Os05g23740.1	150	-21.04	150	-21.04	1688688	21	2505	782
miR399i	LOC_Os05g23880.1	154	-20.46	154	-20.46	1688702	21	3815	2389
miR399i	LOC_Os05g23970.1	151	-23.65	151	-23.65	1688711	21	2106	455
miR399i	LOC_Os05g23980.1	153	-24.79	153	-24.79	1688712	21	2187	283
miR399i	LOC_Os05g24610.1	153	-24.36	153	-24.36	1688783	21	5970	518
miR399i	LOC_Os05g24790.1	145	-23.03	145	-23.03	1688801	21	1434	1044
miR399i	LOC_Os05g24860.1	310	-42.72	157	-21.37	1688807	21	2574	926 979
miR399i	LOC_Os05g25470.1	149	-20.5	149	-20.5	1688876	21	4704	2180
miR399i	LOC_Os05g25974.1	169	-28.66	169	-28.66	1688929	21	2654	552
miR399i	LOC_Os05g25974.2	169	-28.66	169	-28.66	1688930	21	2590	476
miR399i	LOC_Os05g27160.1	173	-27.57	173	-27.57	1689047	21	291	102
miR399i	LOC_Os05g27400.1	155	-23.01	155	-23.01	1689070	21	3729	642
miR399i	LOC_Os05g27860.1	147	-20.56	147	-20.56	1689118	21	2418	338
miR399i	LOC_Os05g28090.1	161	-24.38	161	-24.38	1689146	21	923	324
miR399i	LOC_Os05g28090.2	161	-24.38	161	-24.38	1689147	21	1479	362
miR399i	LOC_Os05g28090.3	161	-24.38	161	-24.38	1689148	21	1018	324
miR399i	LOC_Os05g28170.1	154	-20.27	154	-20.27	1689154	21	3423	993
miR399i	LOC_Os05g28170.2	154	-20.27	154	-20.27	1689155	21	4616	1990
miR399i	LOC_Os05g28250.1	149	-20.48	149	-20.48	1689165	21	2049	37
miR399i	LOC_Os05g29110.1	166	-26.64	166	-26.64	1689261	21	4722	464
miR399i	LOC_Os05g30070.1	142	-23.41	142	-23.41	1689317	21	4530	2620
miR399i	LOC_Os05g30270.1	153	-20.56	153	-20.56	1689339	21	5205	4192

miR399i	LOC_Os05g30660.1	140	-20.25	140	-20.25	1689384	21	2133	595
miR399i	LOC_Os05g31120.1	153	-24.89	153	-24.89	1689444	21	1158	381
miR399i	LOC_Os05g31440.1	153	-20.56	153	-20.56	1689476	21	5961	4171
miR399i	LOC_Os05g31860.1	151	-20.93	151	-20.93	1689521	21	3171	2530
miR399i	LOC_Os05g32030.1	153	-20.65	153	-20.65	1689536	21	2013	66
miR399i	LOC_Os05g32050.1	157	-20.91	157	-20.91	1689538	21	4278	2530
miR399i	LOC_Os05g33260.2	150	-20.43	150	-20.43	1689676	21	1618	916
miR399i	LOC_Os05g33260.3	150	-20.43	150	-20.43	1689677	21	1555	916
miR399i	LOC_Os05g34650.1	161	-24.38	161	-24.38	1689837	21	2156	1190
miR399i	LOC_Os05g34650.2	161	-24.38	161	-24.38	1689838	21	1658	692
miR399i	LOC_Os05g34720.1	150	-20.01	150	-20.01	1689848	21	1358	839
miR399i	LOC_Os05g34760.2	152	-23.61	152	-23.61	1689853	21	3677	2618
miR399i	LOC_Os05g34870.1	159	-22.72	159	-22.72	1689866	21	1008	50
miR399i	LOC_Os05g35180.1	168	-26.16	168	-26.16	1689903	21	3075	292
miR399i	LOC_Os05g35380.1	171	-26.31	171	-26.31	1689928	21	1145	320
miR399i	LOC_Os05g36090.1	147	-20.39	147	-20.39	1690005	21	1967	1199
miR399i	LOC_Os05g36360.1	153	-21.62	153	-21.62	1690043	21	1623	1165
miR399i	LOC_Os05g36940.1	153	-20.16	153	-20.16	1690054	21	4308	3053
miR399i	LOC_Os05g37500.1	148	-20.68	148	-20.68	1690126	21	6524	2255
miR399i	LOC_Os05g37740.1	153	-24.9	153	-24.9	1690152	21	672	615
miR399i	LOC_Os05g37770.1	158	-22.51	158	-22.51	1690155	21	414	99
miR399i	LOC_Os05g37940.1	160	-22.27	160	-22.27	1690176	21	297	249
miR399i	LOC_Os05g38190.2	163	-23.79	163	-23.79	1690210	21	4395	2674
miR399i	LOC_Os05g38640.1	159	-22.41	159	-22.41	1690276	21	1134	555
miR399i	LOC_Os05g38950.1	161	-22.3	161	-22.3	1690312	21	3127	486
miR399i	LOC_Os05g38950.2	161	-22.3	161	-22.3	1690313	21	3034	393
miR399i	LOC_Os05g39390.1	149	-22.21	149	-22.21	1690367	21	3323	1644
miR399i	LOC_Os05g39410.1	140	-24.77	140	-24.77	1690369	21	2660	1524
miR399i	LOC_Os05g39410.2	140	-24.77	140	-24.77	1690370	21	2908	1775
miR399i	LOC_Os05g39410.3	140	-24.77	140	-24.77	1690371	21	2739	1524
miR399i	LOC_Os05g39670.1	160	-23.82	160	-23.82	1690401	21	1125	784
miR399i	LOC_Os05g40050.1	143	-20.83	143	-20.83	1690439	21	3044	1916

miR399i	LOC_Os05g40050.2	143	-20.83	143	-20.83	1690440	21	3094	1916
miR399i	LOC_Os05g40070.1	156	-24.14	156	-24.14	1690442	21	1174	1067
miR399i	LOC_Os05g40810.1	152	-20.39	152	-20.39	1690536	21	2326	1157
miR399i	LOC_Os05g41510.1	145	-21.53	145	-21.53	1690618	21	6761	4916
miR399i	LOC_Os05g41520.1	145	-21.53	145	-21.53	1690619	21	1891	46
miR399i	LOC_Os05g41830.1	327	-49.04	164	-27.61	1690655	21	3906	2780 1134
miR399i	LOC_Os05g42400.1	157	-20.23	157	-20.23	1690719	21	156	132
miR399i	LOC_Os05g42448.1	166	-29.57	166	-29.57	1690726	21	3933	890
miR399i	LOC_Os05g43220.1	153	-20.95	153	-20.95	1690754	21	3636	40
miR399i	LOC_Os05g43350.1	165	-24.73	165	-24.73	1690769	21	3924	2186
miR399i	LOC_Os05g43390.1	162	-25.99	162	-25.99	1690775	21	2068	1035
miR399i	LOC_Os05g43550.1	155	-20.01	155	-20.01	1690792	21	1299	493
miR399i	LOC_Os05g43700.1	148	-20.32	148	-20.32	1690810	21	435	352
miR399i	LOC_Os05g43800.1	145	-20.03	145	-20.03	1690822	21	2096	1519
miR399i	LOC_Os05g43910.1	153	-24.52	153	-24.52	1690837	21	2396	1977
miR399i	LOC_Os05g44360.1	146	-21.59	146	-21.59	1690897	21	2831	2265
miR399i	LOC_Os05g44360.2	146	-21.59	146	-21.59	1690898	21	2410	2352
miR399i	LOC_Os05g45340.1	146	-21.85	146	-21.85	1691014	21	3239	1864
miR399i	LOC_Os05g45350.2	174	-31.64	174	-31.64	1691016	21	1602	404
miR399i	LOC_Os05g45360.1	165	-24.68	165	-24.68	1691018	21	957	372
miR399i	LOC_Os05g45830.1	162	-24.44	162	-24.44	1691073	21	3058	2339
miR399i	LOC_Os05g45950.1	141	-21.64	141	-21.64	1691090	21	1424	782
miR399i	LOC_Os05g46040.2	162	-22.21	162	-22.21	1691100	21	2459	926
miR399i	LOC_Os05g47520.1	157	-20.78	157	-20.78	1691227	21	831	229
miR399i	LOC_Os05g47560.1	151	-20.55	151	-20.55	1691236	21	2160	1364
miR399i	LOC_Os05g47920.1	327	-49.04	164	-27.61	1691271	21	4734	3443 1422
miR399i	LOC_Os05g48570.1	152	-21.15	152	-21.15	1691358	21	579	479
miR399i	LOC_Os05g49130.1	162	-30.94	162	-30.94	1691436	21	3232	2666
miR399i	LOC_Os05g49130.2	162	-30.94	162	-30.94	1691437	21	2752	2186
miR399i	LOC_Os05g49250.1	157	-21.61	157	-21.61	1691454	21	1360	1176
miR399i	LOC_Os05g49250.2	157	-21.61	157	-21.61	1691455	21	3128	2943
miR399i	LOC_Os05g49470.1	152	-21.14	152	-21.14	1691484	21	1192	1000

miR399i	LOC_Os05g49720.1	171	-25.65	171	-25.65	1691513	21	1299	20
miR399i	LOC_Os05g50080.1	144	-21.19	144	-21.19	1691561	21	1620	190
miR399i	LOC_Os05g50560.1	154	-24.23	154	-24.23	1691616	21	2630	1316
miR399i	LOC_Os05g51220.1	148	-20.18	148	-20.18	1691706	21	1721	184
miR399i	LOC_Os05g51480.1	152	-20.36	152	-20.36	1691738	21	3831	3107
miR399i	LOC_Os05g51480.2	152	-20.36	152	-20.36	1691739	21	3825	3103
miR399i	LOC_Os05g51550.1	162	-21.14	162	-21.14	1691749	21	2700	1259
miR399i	LOC_Os05g51570.1	157	-26.86	157	-26.86	1691753	21	1655	512
miR399i	LOC_Os05g51590.1	159	-23.57	159	-23.57	1691755	21	1439	753
miR399i	LOC_Os06g01320.1	156	-20.39	156	-20.39	1691850	21	7009	4750
miR399i	LOC_Os06g01500.1	152	-21.08	152	-21.08	1691867	21	1877	707
miR399i	LOC_Os06g01500.3	152	-21.08	152	-21.08	1691869	21	1787	707
miR399i	LOC_Os06g01650.1	161	-22.93	161	-22.93	1691887	21	3936	140
miR399i	LOC_Os06g01650.2	161	-22.93	161	-22.93	1691888	21	4037	140
miR399i	LOC_Os06g01750.1	149	-22.28	149	-22.28	1691901	21	840	699
miR399i	LOC_Os06g02180.1	147	-26.67	147	-26.67	1691951	21	4214	2764
miR399i	LOC_Os06g02820.1	161	-24.38	161	-24.38	1692026	21	990	858
miR399i	LOC_Os06g03380.1	146	-22.76	146	-22.76	1692079	21	2575	560
miR399i	LOC_Os06g04010.1	158	-22.84	158	-22.84	1692157	21	1620	801
miR399i	LOC_Os06g04010.2	158	-22.84	158	-22.84	1692158	21	1644	797
miR399i	LOC_Os06g04280.1	165	-23.41	165	-23.41	1692191	21	1911	859
miR399i	LOC_Os06g05240.1	158	-22.59	158	-22.59	1692310	21	2516	1527
miR399i	LOC_Os06g05980.1	146	-21.11	146	-21.11	1692407	21	2211	197
miR399i	LOC_Os06g05980.2	146	-21.11	146	-21.11	1692408	21	2277	255
miR399i	LOC_Os06g05990.1	174	-26.91	174	-26.91	1692409	21	1779	716
miR399i	LOC_Os06g06690.1	164	-27.76	164	-27.76	1692502	21	360	274
miR399i	LOC_Os06g06920.1	146	-20.52	146	-20.52	1692528	21	2433	23
miR399i	LOC_Os06g07070.1	163	-29.83	163	-29.83	1692546	21	1651	971
miR399i	LOC_Os06g08070.1	149	-20.5	149	-20.5	1692653	21	4011	2093
miR399i	LOC_Os06g08380.1	317	-43.44	160	-22.89	1692682	21	6114	5171 2728
miR399i	LOC_Os06g08480.1	163	-23.58	163	-23.58	1692692	21	4808	1320
miR399i	LOC_Os06g08860.1	153	-22.82	153	-22.82	1692747	21	1343	289

miR399i	LOC_Os06g10480.1	148	-20.81	148	-20.81	1692939	21	3205	654
miR399i	LOC_Os06g10620.1	321	-43.28	169	-22.9	1692952	21	2477	287 2349
miR399i	LOC_Os06g10900.1	151	-23.72	151	-23.72	1692983	21	672	337
miR399i	LOC_Os06g11170.1	161	-22.26	161	-22.26	1693011	21	1350	1050
miR399i	LOC_Os06g11170.2	161	-22.26	161	-22.26	1693012	21	1594	1294
miR399i	LOC_Os06g11410.1	151	-20.63	151	-20.63	1693038	21	1580	742
miR399i	LOC_Os06g11410.2	151	-20.63	151	-20.63	1693039	21	1698	852
miR399i	LOC_Os06g11560.1	165	-22.6	165	-22.6	1693056	21	2118	977
miR399i	LOC_Os06g11990.1	163	-22.07	163	-22.07	1693103	21	3815	2163
miR399i	LOC_Os06g12260.1	155	-20.66	155	-20.66	1693141	21	3048	2248
miR399i	LOC_Os06g12260.2	155	-20.66	155	-20.66	1693142	21	3089	2248
miR399i	LOC_Os06g12260.3	155	-20.66	155	-20.66	1693143	21	2094	1294
miR399i	LOC_Os06g12610.1	160	-24.6	160	-24.6	1693181	21	2469	1844
miR399i	LOC_Os06g13860.1	151	-20.07	151	-20.07	1693323	21	2877	2518
miR399i	LOC_Os06g13860.2	151	-20.07	151	-20.07	1693324	21	2768	2409
miR399i	LOC_Os06g13970.1	165	-23.31	165	-23.31	1693334	21	690	549
miR399i	LOC_Os06g14140.1	153	-21.08	153	-21.08	1693352	21	5961	4177
miR399i	LOC_Os06g16980.1	165	-25.79	165	-25.79	1693650	21	4102	725
miR399i	LOC_Os06g16980.2	165	-25.79	165	-25.79	1693651	21	3986	609
miR399i	LOC_Os06g17800.1	157	-25.78	157	-25.78	1693730	21	2514	2342
miR399i	LOC_Os06g18820.2	153	-23.07	153	-23.07	1693781	21	2715	2231
miR399i	LOC_Os06g18970.1	150	-22.28	150	-22.28	1693796	21	1995	357
miR399i	LOC_Os06g19900.1	159	-23.38	159	-23.38	1693891	21	3870	412
miR399i	LOC_Os06g20390.1	162	-30.09	162	-30.09	1693945	21	2009	722
miR399i	LOC_Os06g20420.1	148	-20.9	148	-20.9	1693952	21	441	233
miR399i	LOC_Os06g22030.1	152	-26.52	152	-26.52	1694122	21	2253	1348
miR399i	LOC_Os06g22870.1	163	-25.09	163	-25.09	1694217	21	1414	683
miR399i	LOC_Os06g22870.2	163	-25.09	163	-25.09	1694218	21	1383	683
miR399i	LOC_Os06g23020.1	153	-20.92	153	-20.92	1694234	21	639	244
miR399i	LOC_Os06g23080.1	163	-25.98	163	-25.98	1694240	21	1998	873
miR399i	LOC_Os06g23100.1	294	-46.27	148	-23.72	1694242	21	468	414 440
miR399i	LOC_Os06g23200.1	157	-20.97	157	-20.97	1694251	21	1857	934

miR399i	LOC_Os06g23504.1	157	-21.91	157	-21.91	1694286	21	2023	1105
miR399i	LOC_Os06g24090.1	151	-22.42	151	-22.42	1694345	21	1503	115
miR399i	LOC_Os06g24680.1	151	-20.93	151	-20.93	1694404	21	1902	394
miR399i	LOC_Os06g24730.2	165	-25.96	165	-25.96	1694410	21	3743	2512
miR399i	LOC_Os06g24730.3	165	-25.96	165	-25.96	1694411	21	3081	1850
miR399i	LOC_Os06g25160.1	157	-22.01	157	-22.01	1694451	21	738	171
miR399i	LOC_Os06g25210.1	149	-20.48	149	-20.48	1694456	21	2703	1092
miR399i	LOC_Os06g25230.1	153	-21.31	153	-21.31	1694458	21	1134	876
miR399i	LOC_Os06g25280.1	153	-20.93	153	-20.93	1694463	21	2136	915
miR399i	LOC_Os06g25890.1	159	-20.02	159	-20.02	1694521	21	4704	2015
miR399i	LOC_Os06g26070.1	140	-20.89	140	-20.89	1694539	21	3171	818
miR399i	LOC_Os06g27450.1	149	-20.48	149	-20.48	1694577	21	1125	445
miR399i	LOC_Os06g28080.1	149	-20.48	149	-20.48	1694649	21	1728	306
miR399i	LOC_Os06g29120.1	294	-42.74	147	-21.37	1694754	21	3173	2655 2782
miR399i	LOC_Os06g29120.2	147	-21.37	147	-21.37	1694755	21	2949	2568
miR399i	LOC_Os06g29250.1	154	-26.13	154	-26.13	1694768	21	913	548
miR399i	LOC_Os06g29760.1	153	-20.02	153	-20.02	1694823	21	6015	4219
miR399i	LOC_Os06g30080.1	157	-25.26	157	-25.26	1694855	21	2736	2366
miR399i	LOC_Os06g30770.1	152	-22.06	152	-22.06	1694927	21	489	166
miR399i	LOC_Os06g31750.1	155	-24.66	155	-24.66	1695026	21	1269	797
miR399i	LOC_Os06g32390.1	155	-24.66	155	-24.66	1695087	21	990	518
miR399i	LOC_Os06g33100.1	162	-20.68	162	-20.68	1695153	21	1598	711
miR399i	LOC_Os06g33280.1	153	-20.56	153	-20.56	1695170	21	2868	1153
miR399i	LOC_Os06g35110.1	327	-49.04	164	-27.61	1695354	21	1284	1001 841
miR399i	LOC_Os06g35270.1	157	-25.26	157	-25.26	1695371	21	2232	1877
miR399i	LOC_Os06g35390.1	161	-21.95	161	-21.95	1695382	21	1650	698
miR399i	LOC_Os06g35660.1	156	-20.45	156	-20.45	1695410	21	1587	55
miR399i	LOC_Os06g36500.1	163	-27.61	163	-27.61	1695500	21	3756	1842
miR399i	LOC_Os06g37000.1	148	-20.72	148	-20.72	1695552	21	1543	462
miR399i	LOC_Os06g37000.2	148	-20.72	148	-20.72	1695553	21	1535	454
miR399i	LOC_Os06g37400.1	160	-22.66	160	-22.66	1695593	21	7053	6339
miR399i	LOC_Os06g37540.1	160	-21.32	160	-21.32	1695609	21	237	37

miR399i	LOC_Os06g37850.1	164	-27.68	164	-27.68	1695641	21	1518	601
miR399i	LOC_Os06g38420.1	153	-20.56	153	-20.56	1695695	21	5988	4192
miR399i	LOC_Os06g38480.1	151	-20.86	151	-20.86	1695703	21	2210	1103
miR399i	LOC_Os06g38480.2	151	-20.86	151	-20.86	1695704	21	2126	1019
miR399i	LOC_Os06g38900.1	149	-20.5	149	-20.5	1695745	21	2154	122
miR399i	LOC_Os06g38950.1	158	-20.25	158	-20.25	1695751	21	3289	349
miR399i	LOC_Os06g39980.1	152	-23.41	152	-23.41	1695874	21	1722	78
miR399i	LOC_Os06g40000.1	141	-20.7	141	-20.7	1695876	21	2742	348
miR399i	LOC_Os06g40190.1	161	-24.71	161	-24.71	1695897	21	3157	1580
miR399i	LOC_Os06g40560.1	147	-22.76	147	-22.76	1695936	21	1731	1321
miR399i	LOC_Os06g40650.1	156	-20.07	156	-20.07	1695949	21	2172	716
miR399i	LOC_Os06g40650.2	156	-20.07	156	-20.07	1695950	21	2360	716
miR399i	LOC_Os06g40650.4	156	-20.07	156	-20.07	1695952	21	2613	1248
miR399i	LOC_Os06g40950.1	161	-26.48	161	-26.48	1695987	21	1500	1010
miR399i	LOC_Os06g41010.1	154	-22.31	154	-22.31	1695993	21	1345	222
miR399i	LOC_Os06g41010.3	154	-22.31	154	-22.31	1695994	21	1246	123
miR399i	LOC_Os06g41010.4	154	-22.31	154	-22.31	1695995	21	1428	305
miR399i	LOC_Os06g41510.1	161	-22.7	161	-22.7	1696047	21	2496	148
miR399i	LOC_Os06g41710.1	163	-25.78	163	-25.78	1696068	21	3305	417
miR399i	LOC_Os06g42420.1	157	-20.13	157	-20.13	1696152	21	2730	95
miR399i	LOC_Os06g42640.1	169	-25.72	169	-25.72	1696180	21	2924	566
miR399i	LOC_Os06g42640.2	169	-25.72	169	-25.72	1696181	21	2940	566
miR399i	LOC_Os06g42640.3	169	-25.72	169	-25.72	1696182	21	2929	566
miR399i	LOC_Os06g42860.1	163	-23.17	163	-23.17	1696205	21	1668	216
miR399i	LOC_Os06g43290.1	153	-20.95	153	-20.95	1696249	21	3711	40
miR399i	LOC_Os06g43540.1	153	-20.16	153	-20.16	1696273	21	4220	2657
miR399i	LOC_Os06g43800.1	166	-20.68	166	-20.68	1696307	21	1671	350
miR399i	LOC_Os06g43800.2	166	-20.68	166	-20.68	1696308	21	1836	350
miR399i	LOC_Os06g43820.1	140	-22.64	140	-22.64	1696310	21	765	210
miR399i	LOC_Os06g44200.1	166	-26.45	166	-26.45	1696356	21	2224	662
miR399i	LOC_Os06g44200.2	166	-26.45	166	-26.45	1696357	21	2203	662
miR399i	LOC_Os06g44200.3	166	-26.45	166	-26.45	1696358	21	2282	662

miR399i	LOC_Os06g44770.1	151	-20.67	151	-20.67	1696418	21	516	145
miR399i	LOC_Os06g45020.1	147	-20.53	147	-20.53	1696446	21	2795	190
miR399i	LOC_Os06g45030.1	149	-21.69	149	-21.69	1696447	21	3229	2080
miR399i	LOC_Os06g45800.1	153	-25.14	153	-25.14	1696544	21	4464	3161
miR399i	LOC_Os06g45850.1	149	-20.14	149	-20.14	1696553	21	1279	466
miR399i	LOC_Os06g45850.2	149	-20.14	149	-20.14	1696554	21	1292	479
miR399i	LOC_Os06g45850.3	149	-20.14	149	-20.14	1696555	21	1251	433
miR399i	LOC_Os06g45850.4	149	-20.14	149	-20.14	1696556	21	1250	437
miR399i	LOC_Os06g45850.5	149	-20.14	149	-20.14	1696557	21	1246	433
miR399i	LOC_Os06g46380.1	154	-22.6	154	-22.6	1696613	21	2503	861
miR399i	LOC_Os06g46870.1	167	-23.64	167	-23.64	1696674	21	813	312
miR399i	LOC_Os06g46940.1	155	-20.71	155	-20.71	1696686	21	3151	1617
miR399i	LOC_Os06g46940.2	155	-20.71	155	-20.71	1696687	21	1506	76
miR399i	LOC_Os06g47380.1	153	-20.56	153	-20.56	1696746	21	6033	4258
miR399i	LOC_Os06g48140.1	157	-25.78	157	-25.78	1696835	21	2514	2342
miR399i	LOC_Os06g48470.1	153	-20.56	153	-20.56	1696873	21	6033	4258
miR399i	LOC_Os06g48520.1	155	-21.21	155	-21.21	1696879	21	2235	102
miR399i	LOC_Os06g49060.1	145	-28.94	145	-28.94	1696950	21	2143	663
miR399i	LOC_Os06g49130.1	150	-22.63	150	-22.63	1696961	21	5280	2166
miR399i	LOC_Os06g49130.2	150	-22.63	150	-22.63	1696962	21	4413	1299
miR399i	LOC_Os06g49250.5	148	-20.12	148	-20.12	1696979	21	2215	438
miR399i	LOC_Os06g49540.1	151	-21.38	151	-21.38	1697021	21	4221	1122
miR399i	LOC_Os06g49670.1	142	-23.39	142	-23.39	1697038	21	2060	1495
miR399i	LOC_Os06g49670.2	142	-23.39	142	-23.39	1697039	21	1740	1495
miR399i	LOC_Os06g49980.1	159	-21.84	159	-21.84	1697078	21	1116	1094
miR399i	LOC_Os06g50110.1	157	-22.41	157	-22.41	1697094	21	1586	873
miR399i	LOC_Os06g50270.1	153	-21.08	153	-21.08	1697112	21	5910	4111
miR399i	LOC_Os06g50789.1	149	-25.08	149	-25.08	1697168	21	1485	272
miR399i	LOC_Os06g50930.1	149	-24.6	149	-24.6	1697186	21	1287	470
miR399i	LOC_Os06g50930.2	149	-24.6	149	-24.6	1697187	21	1447	470
miR399i	LOC_Os06g51330.1	162	-21.89	162	-21.89	1697245	21	4277	2711
miR399i	LOC_Os06g51330.2	162	-21.89	162	-21.89	1697246	21	4223	2657

miR399i	LOC_Os06g51430.1	157	-25	157	-25	1697261	21	2037	698
miR399i	LOC_Os06g51460.1	142	-22.04	142	-22.04	1697264	21	3495	1211
miR399i	LOC_Os06g51460.2	142	-22.04	142	-22.04	1697265	21	3483	1169
miR399i	LOC_Os07g01320.1	159	-23.31	159	-23.31	1697323	21	2104	1540
miR399i	LOC_Os07g02600.1	327	-49.04	164	-27.61	1697464	21	2619	2063 828
miR399i	LOC_Os07g02760.1	150	-20.1	150	-20.1	1697481	21	1979	1762
miR399i	LOC_Os07g02760.2	150	-20.1	150	-20.1	1697482	21	1927	1710
miR399i	LOC_Os07g03030.1	161	-21.63	161	-21.63	1697514	21	1895	327
miR399i	LOC_Os07g03310.1	156	-24.31	156	-24.31	1697554	21	3054	156
miR399i	LOC_Os07g03490.1	156	-24.31	156	-24.31	1697563	21	2946	156
miR399i	LOC_Os07g03790.1	140	-20.66	140	-20.66	1697592	21	1971	1122
miR399i	LOC_Os07g03830.1	143	-20.65	143	-20.65	1697595	21	2016	1170
miR399i	LOC_Os07g05500.1	147	-21.7	147	-21.7	1697772	21	966	510
miR399i	LOC_Os07g06274.1	153	-21.08	153	-21.08	1697859	21	5895	4111
miR399i	LOC_Os07g06610.1	157	-21.03	157	-21.03	1697889	21	3023	920
miR399i	LOC_Os07g07020.2	150	-21.47	150	-21.47	1697941	21	1385	1144
miR399i	LOC_Os07g07840.1	153	-21.08	153	-21.08	1698033	21	2799	1000
miR399i	LOC_Os07g07910.1	168	-21.02	168	-21.02	1698041	21	3016	1124
miR399i	LOC_Os07g08140.1	159	-21.32	159	-21.32	1698061	21	1967	257
miR399i	LOC_Os07g08660.1	161	-21.14	161	-21.14	1698118	21	838	691
miR399i	LOC_Os07g09010.1	159	-23.98	159	-23.98	1698159	21	2437	1484
miR399i	LOC_Os07g09090.1	166	-26.65	166	-26.65	1698170	21	1431	1108
miR399i	LOC_Os07g09900.1	142	-21.6	142	-21.6	1698254	21	2082	183
miR399i	LOC_Os07g10150.1	163	-21.07	163	-21.07	1698279	21	3204	1192
miR399i	LOC_Os07g10254.1	151	-21.81	151	-21.81	1698290	21	701	228
miR399i	LOC_Os07g10470.1	149	-20.5	149	-20.5	1698315	21	2130	1784
miR399i	LOC_Os07g10610.2	148	-22.05	148	-22.05	1698334	21	4614	1052
miR399i	LOC_Os07g11240.1	140	-23.66	140	-23.66	1698412	21	5226	1845
miR399i	LOC_Os07g11270.1	157	-22.78	157	-22.78	1698415	21	2682	2327
miR399i	LOC_Os07g12040.1	150	-20.21	150	-20.21	1698485	21	459	130
miR399i	LOC_Os07g12130.1	165	-25.82	165	-25.82	1698494	21	1482	1100
miR399i	LOC_Os07g12700.1	155	-25.12	155	-25.12	1698560	21	282	65

miR399i	LOC_Os07g13130.1	157	-25.76	157	-25.76	1698604	21	2358	1988
miR399i	LOC_Os07g13534.1	156	-20.79	156	-20.79	1698645	21	697	272
miR399i	LOC_Os07g13680.1	156	-22.53	156	-22.53	1698657	21	3288	166
miR399i	LOC_Os07g13980.1	157	-22.64	157	-22.64	1698685	21	2753	429
miR399i	LOC_Os07g13969.1	157	-22.64	157	-22.64	1698686	21	1095	429
miR399i	LOC_Os07g14650.1	161	-23.7	161	-23.7	1698770	21	984	5
miR399i	LOC_Os07g14650.2	161	-23.7	161	-23.7	1698771	21	1084	5
miR399i	LOC_Os07g14990.1	314	-41.1	157	-20.97	1698804	21	3213	1601 1654
miR399i	LOC_Os07g15050.1	141	-20.33	141	-20.33	1698810	21	2043	1295
miR399i	LOC_Os07g15500.1	161	-24.4	161	-24.4	1698856	21	1355	690
miR399i	LOC_Os07g15610.1	160	-21.24	160	-21.24	1698867	21	258	19
miR399i	LOC_Os07g16000.1	163	-22.99	163	-22.99	1698907	21	4893	437
miR399i	LOC_Os07g16030.1	155	-21.71	155	-21.71	1698909	21	3314	1213
miR399i	LOC_Os07g16560.1	150	-21.02	150	-21.02	1698955	21	5169	3767
miR399i	LOC_Os07g17210.1	150	-25.33	150	-25.33	1699023	21	2193	2086
miR399i	LOC_Os07g17360.1	154	-20.86	154	-20.86	1699040	21	2672	231
miR399i	LOC_Os07g17360.2	154	-20.86	154	-20.86	1699041	21	2636	231
miR399i	LOC_Os07g17980.1	160	-21.47	160	-21.47	1699105	21	732	33
miR399i	LOC_Os07g18370.1	157	-25.26	157	-25.26	1699146	21	2499	2168
miR399i	LOC_Os07g18660.1	168	-21.96	168	-21.96	1699172	21	432	356
miR399i	LOC_Os07g18944.1	157	-22.12	157	-22.12	1699196	21	2462	1921
miR399i	LOC_Os07g19390.1	151	-21.35	151	-21.35	1699235	21	954	1
miR399i	LOC_Os07g19470.1	153	-20.25	153	-20.25	1699242	21	1300	891
miR399i	LOC_Os07g19470.2	153	-20.25	153	-20.25	1699243	21	1286	891
miR399i	LOC_Os07g19470.3	153	-20.25	153	-20.25	1699244	21	1399	891
miR399i	LOC_Os07g19580.1	172	-27.86	172	-27.86	1699255	21	543	218
miR399i	LOC_Os07g20270.1	163	-25.33	163	-25.33	1699275	21	1444	598
miR399i	LOC_Os07g20690.1	161	-26.46	161	-26.46	1699317	21	2211	1219
miR399i	LOC_Os07g22390.2	150	-20.69	150	-20.69	1699386	21	1661	928
miR399i	LOC_Os07g22400.1	158	-22.03	158	-22.03	1699387	21	1941	1048
miR399i	LOC_Os07g22400.2	158	-22.03	158	-22.03	1699388	21	1741	848
miR399i	LOC_Os07g22460.1	149	-22.35	149	-22.35	1699394	21	588	344

miR399i	LOC_Os07g23040.1	166	-26.64	166	-26.64	1699472	21	1323	443
miR399i	LOC_Os07g23200.1	150	-21.22	150	-21.22	1699488	21	1365	1028
miR399i	LOC_Os07g23260.1	157	-23.09	157	-23.09	1699493	21	1218	955
miR399i	LOC_Os07g24010.1	159	-22.9	159	-22.9	1699577	21	465	178
miR399i	LOC_Os07g25090.1	153	-20.56	153	-20.56	1699687	21	6018	4222
miR399i	LOC_Os07g25440.1	158	-22.13	158	-22.13	1699722	21	2733	1925
miR399i	LOC_Os07g25440.2	158	-22.13	158	-22.13	1699723	21	2658	1925
miR399i	LOC_Os07g25680.1	159	-21.59	159	-21.59	1699746	21	4485	3894
miR399i	LOC_Os07g26090.1	159	-21.69	159	-21.69	1699787	21	879	483
miR399i	LOC_Os07g26140.1	153	-21.48	153	-21.48	1699792	21	564	13
miR399i	LOC_Os07g26810.1	153	-20.56	153	-20.56	1699865	21	3942	3412
miR399i	LOC_Os07g26840.1	157	-25.76	157	-25.76	1699868	21	2310	1940
miR399i	LOC_Os07g27150.1	144	-20.65	144	-20.65	1699905	21	1077	384
miR399i	LOC_Os07g27150.2	144	-20.65	144	-20.65	1699906	21	1056	363
miR399i	LOC_Os07g27150.3	144	-20.65	144	-20.65	1699907	21	1001	308
miR399i	LOC_Os07g27490.1	159	-22.59	159	-22.59	1699940	21	2259	1292
miR399i	LOC_Os07g27490.2	159	-22.59	159	-22.59	1699941	21	1796	829
miR399i	LOC_Os07g27720.1	142	-22.48	142	-22.48	1699962	21	3018	648
miR399i	LOC_Os07g28080.1	159	-21.38	159	-21.38	1700001	21	393	143
miR399i	LOC_Os07g28810.1	160	-26.43	160	-26.43	1700080	21	966	182
miR399i	LOC_Os07g29150.1	148	-29.41	148	-29.41	1700111	21	639	271
miR399i	LOC_Os07g29770.1	151	-24.2	151	-24.2	1700183	21	3806	1969
miR399i	LOC_Os07g29770.2	151	-24.2	151	-24.2	1700184	21	4270	2433
miR399i	LOC_Os07g29770.3	151	-24.2	151	-24.2	1700185	21	4035	2198
miR399i	LOC_Os07g30160.1	170	-28.55	170	-28.55	1700226	21	4014	3628
miR399i	LOC_Os07g30170.1	170	-28.55	170	-28.55	1700227	21	1700	292
miR399i	LOC_Os07g30420.1	172	-28.89	172	-28.89	1700251	21	264	38
miR399i	LOC_Os07g30640.1	167	-23.54	167	-23.54	1700271	21	702	235
miR399i	LOC_Os07g30730.1	156	-24.57	156	-24.57	1700281	21	4566	2986
miR399i	LOC_Os07g30880.1	168	-27.12	168	-27.12	1700300	21	5415	82
miR399i	LOC_Os07g30910.1	156	-24.57	156	-24.57	1700303	21	5358	2986
miR399i	LOC_Os07g31590.1	155	-20.52	155	-20.52	1700378	21	3315	2217

miR399i	LOC_Os07g31920.1	167	-25.41	167	-25.41	1700407	21	1761	1602
miR399i	LOC_Os07g32360.1	167	-24.33	167	-24.33	1700454	21	366	40
miR399i	LOC_Os07g32390.1	158	-20.8	158	-20.8	1700457	21	2961	1720
miR399i	LOC_Os07g32680.1	149	-25.37	149	-25.37	1700492	21	1424	612
miR399i	LOC_Os07g32700.1	143	-21.87	143	-21.87	1700494	21	891	301
miR399i	LOC_Os07g32710.1	144	-21.84	144	-21.84	1700495	21	1402	312
miR399i	LOC_Os07g33010.1	143	-20.48	143	-20.48	1700523	21	6084	5961
miR399i	LOC_Os07g33420.1	143	-20.99	143	-20.99	1700569	21	1963	1716
miR399i	LOC_Os07g33420.2	143	-20.99	143	-20.99	1700570	21	2047	1723
miR399i	LOC_Os07g33420.3	143	-20.99	143	-20.99	1700571	21	2117	1723
miR399i	LOC_Os07g33420.4	143	-20.99	143	-20.99	1700572	21	2042	1723
miR399i	LOC_Os07g33450.1	163	-21.83	163	-21.83	1700574	21	4422	1311
miR399i	LOC_Os07g33480.1	158	-22.96	158	-22.96	1700577	21	1931	540
miR399i	LOC_Os07g33480.2	158	-22.96	158	-22.96	1700578	21	2307	540
miR399i	LOC_Os07g33480.4	159	-23.56	159	-23.56	1700579	21	2440	1048
miR399i	LOC_Os07g34190.1	154	-26.19	154	-26.19	1700647	21	1197	361
miR399i	LOC_Os07g34710.1	152	-24.24	152	-24.24	1700709	21	1338	529
miR399i	LOC_Os07g34710.2	152	-24.24	152	-24.24	1700710	21	1086	277
miR399i	LOC_Os07g34710.3	152	-24.24	152	-24.24	1700711	21	1068	259
miR399i	LOC_Os07g34930.1	165	-27.34	165	-27.34	1700732	21	609	153
miR399i	LOC_Os07g35300.1	149	-20.93	149	-20.93	1700771	21	2040	1582
miR399i	LOC_Os07g35760.1	159	-22.29	159	-22.29	1700832	21	567	22
miR399i	LOC_Os07g36600.1	157	-20.48	157	-20.48	1700924	21	1816	238
miR399i	LOC_Os07g36600.2	157	-20.48	157	-20.48	1700925	21	1682	238
miR399i	LOC_Os07g36780.1	150	-27.8	150	-27.8	1700942	21	2817	1002
miR399i	LOC_Os07g36860.1	152	-21.27	152	-21.27	1700952	21	1041	180
miR399i	LOC_Os07g37300.1	152	-24.9	152	-24.9	1701003	21	474	309
miR399i	LOC_Os07g37610.1	171	-31.5	171	-31.5	1701037	21	3506	3375
miR399i	LOC_Os07g37790.1	162	-22.8	162	-22.8	1701055	21	1207	406
miR399i	LOC_Os07g38270.1	153	-22.77	153	-22.77	1701111	21	3242	3181
miR399i	LOC_Os07g38360.2	161	-24.14	161	-24.14	1701123	21	5071	4396
miR399i	LOC_Os07g38370.1	164	-20.96	164	-20.96	1701126	21	1027	777

miR399i	LOC_Os07g38620.1	141	-21.44	141	-21.44	1701152	21	1569	839
miR399i	LOC_Os07g38890.1	155	-21.44	155	-21.44	1701182	21	2031	559
miR399i	LOC_Os07g39260.1	153	-22.7	153	-22.7	1701224	21	1478	899
miR399i	LOC_Os07g39390.1	160	-23.38	160	-23.38	1701239	21	2023	1756
miR399i	LOC_Os07g40310.1	157	-25.25	157	-25.25	1701347	21	480	455
miR399i	LOC_Os07g40320.1	159	-20.98	159	-20.98	1701348	21	1119	887
miR399i	LOC_Os07g40620.1	170	-30.19	170	-30.19	1701379	21	1688	1036
miR399i	LOC_Os07g40670.1	157	-23.09	157	-23.09	1701384	21	375	192
miR399i	LOC_Os07g40750.1	155	-25.11	155	-25.11	1701395	21	1827	1387
miR399i	LOC_Os07g40850.1	467	-69.63	157	-23.98	1701406	21	1012	415 619 754
miR399i	LOC_Os07g40860.1	314	-46.94	157	-24.87	1701407	21	1145	547 766
miR399i	LOC_Os07g40860.2	314	-46.94	157	-24.87	1701408	21	1099	501 720
miR399i	LOC_Os07g40890.1	165	-29.14	165	-29.14	1701411	21	699	424
miR399i	LOC_Os07g40950.1	159	-25.14	159	-25.14	1701417	21	984	327
miR399i	LOC_Os07g41400.1	157	-20.93	157	-20.93	1701465	21	460	192
miR399i	LOC_Os07g41430.1	149	-20.5	149	-20.5	1701468	21	5007	2420
miR399i	LOC_Os07g41694.1	153	-23.92	153	-23.92	1701501	21	1878	1087
miR399i	LOC_Os07g42234.1	151	-20.18	151	-20.18	1701559	21	4335	1611
miR399i	LOC_Os07g42370.1	144	-20.04	144	-20.04	1701571	21	1405	479
miR399i	LOC_Os07g42370.2	144	-20.04	144	-20.04	1701572	21	1712	479
miR399i	LOC_Os07g42370.3	144	-20.04	144	-20.04	1701573	21	1056	479
miR399i	LOC_Os07g42680.1	140	-22.49	140	-22.49	1701618	21	3408	1606
miR399i	LOC_Os07g42900.1	155	-23.01	155	-23.01	1701645	21	4108	1721
miR399i	LOC_Os07g43380.1	154	-20.66	154	-20.66	1701710	21	1672	1180
miR399i	LOC_Os07g44540.1	161	-21.95	161	-21.95	1701833	21	2961	1103
miR399i	LOC_Os07g44630.1	155	-22.47	155	-22.47	1701846	21	1180	278
miR399i	LOC_Os07g44630.2	155	-22.47	155	-22.47	1701847	21	1273	278
miR399i	LOC_Os07g44670.1	158	-20.47	158	-20.47	1701851	21	2895	277
miR399i	LOC_Os07g45330.1	159	-20.47	159	-20.47	1701918	21	1663	489
miR399i	LOC_Os07g45650.1	156	-20.19	156	-20.19	1701951	21	5967	2501
miR399i	LOC_Os07g45850.1	157	-20.24	157	-20.24	1701971	21	1116	113
miR399i	LOC_Os07g46270.1	153	-21.08	153	-21.08	1702013	21	2949	1150

miR399i	LOC_Os07g46590.1	157	-20.18	157	-20.18	1702058	21	3775	1188
miR399i	LOC_Os07g47010.1	149	-20.5	149	-20.5	1702121	21	2337	2162
miR399i	LOC_Os07g47201.1	151	-22.74	151	-22.74	1702152	21	1582	624
miR399i	LOC_Os07g47490.1	151	-20.21	151	-20.21	1702191	21	3384	2076
miR399i	LOC_Os07g47490.2	151	-20.21	151	-20.21	1702192	21	5148	2103
miR399i	LOC_Os07g47490.3	151	-20.21	151	-20.21	1702193	21	2509	2076
miR399i	LOC_Os07g47510.1	151	-22.15	151	-22.15	1702199	21	1464	1209
miR399i	LOC_Os07g47510.2	151	-22.15	151	-22.15	1702200	21	993	738
miR399i	LOC_Os07g47580.1	159	-24.01	159	-24.01	1702208	21	1229	791
miR399i	LOC_Os07g47580.2	159	-24.01	159	-24.01	1702209	21	1509	1071
miR399i	LOC_Os07g47580.3	159	-24.01	159	-24.01	1702210	21	1366	791
miR399i	LOC_Os07g47720.1	165	-25.28	165	-25.28	1702232	21	2436	311
miR399i	LOC_Os07g47740.1	153	-21.08	153	-21.08	1702234	21	5895	4111
miR399i	LOC_Os07g47780.1	150	-22.13	150	-22.13	1702238	21	901	517
miR399i	LOC_Os07g47780.2	150	-22.13	150	-22.13	1702239	21	877	493
miR399i	LOC_Os07g47820.1	151	-20.97	151	-20.97	1702244	21	3787	540
miR399i	LOC_Os07g47820.2	151	-20.97	151	-20.97	1702245	21	3765	540
miR399i	LOC_Os07g47920.1	166	-24.99	166	-24.99	1702256	21	2601	1729
miR399i	LOC_Os07g48290.1	153	-21.04	153	-21.04	1702298	21	2436	676
miR399i	LOC_Os07g48290.2	153	-21.04	153	-21.04	1702299	21	2412	676
miR399i	LOC_Os07g48340.1	150	-23.51	150	-23.51	1702305	21	851	620
miR399i	LOC_Os07g48340.2	150	-23.51	150	-23.51	1702306	21	1184	750
miR399i	LOC_Os07g48720.1	154	-20.64	154	-20.64	1702357	21	3146	1908
miR399i	LOC_Os07g48720.2	154	-20.64	154	-20.64	1702358	21	3033	1795
miR399i	LOC_Os07g48720.3	154	-20.64	154	-20.64	1702359	21	3343	1961
miR399i	LOC_Os08g03090.1	152	-20.26	152	-20.26	1702716	21	1899	1504
miR399i	LOC_Os08g03220.1	157	-20.79	157	-20.79	1702729	21	5520	3662
miR399i	LOC_Os08g03360.1	153	-20.07	153	-20.07	1702749	21	1949	778
miR399i	LOC_Os08g04070.1	158	-20.98	158	-20.98	1702832	21	693	599
miR399i	LOC_Os08g04300.1	162	-25.05	162	-25.05	1702863	21	2415	262
miR399i	LOC_Os08g04630.1	163	-24.23	163	-24.23	1702900	21	2636	1067
miR399i	LOC_Os08g04950.1	156	-24.57	156	-24.57	1702940	21	5187	2701

miR399i	LOC_Os08g05120.1	313	-40.35	157	-20.19	1702959	21	5976	569 2510
miR399i	LOC_Os08g05560.1	160	-22.2	160	-22.2	1703003	21	2319	2209
miR399i	LOC_Os08g05590.1	153	-24.7	153	-24.7	1703007	21	1302	675
miR399i	LOC_Os08g05600.1	150	-22.91	150	-22.91	1703008	21	837	205
miR399i	LOC_Os08g05930.1	153	-20.56	153	-20.56	1703044	21	5592	3808
miR399i	LOC_Os08g06360.1	152	-21.14	152	-21.14	1703097	21	1389	1021
miR399i	LOC_Os08g06760.1	160	-21.46	160	-21.46	1703145	21	504	106
miR399i	LOC_Os08g07380.1	151	-20.09	151	-20.09	1703204	21	5334	3108
miR399i	LOC_Os08g08140.1	159	-23.27	159	-23.27	1703285	21	3297	1706
miR399i	LOC_Os08g08910.1	157	-25.05	157	-25.05	1703371	21	162	103
miR399i	LOC_Os08g10060.1	153	-20.56	153	-20.56	1703500	21	6042	4258
miR399i	LOC_Os08g10140.1	157	-22.26	157	-22.26	1703509	21	2436	1166
miR399i	LOC_Os08g11010.1	151	-21.16	151	-21.16	1703606	21	1509	546
miR399i	LOC_Os08g11150.1	332	-52.64	170	-30.08	1703620	21	4680	2754 2063
miR399i	LOC_Os08g11190.1	152	-22.01	152	-22.01	1703624	21	2094	1676
miR399i	LOC_Os08g11500.1	159	-22.4	159	-22.4	1703662	21	1335	125
miR399i	LOC_Os08g11810.1	146	-20.55	146	-20.55	1703692	21	2097	182
miR399i	LOC_Os08g12140.1	157	-21.11	157	-21.11	1703724	21	2220	2024
miR399i	LOC_Os08g12170.1	161	-24.39	161	-24.39	1703727	21	561	515
miR399i	LOC_Os08g12380.1	157	-25.76	157	-25.76	1703746	21	1659	1289
miR399i	LOC_Os08g12960.1	152	-21.89	152	-21.89	1703813	21	921	612
miR399i	LOC_Os08g13520.1	157	-20.97	157	-20.97	1703873	21	1845	865
miR399i	LOC_Os08g13620.1	153	-20.56	153	-20.56	1703883	21	5736	4150
miR399i	LOC_Os08g13640.1	162	-23.41	162	-23.41	1703885	21	1155	159
miR399i	LOC_Os08g14390.1	159	-26.96	159	-26.96	1703960	21	1173	97
miR399i	LOC_Os08g15300.1	160	-22.04	160	-22.04	1704074	21	3561	2566
miR399i	LOC_Os08g15900.1	155	-20.34	155	-20.34	1704138	21	2008	1425
miR399i	LOC_Os08g16010.1	158	-20.84	158	-20.84	1704149	21	1186	972
miR399i	LOC_Os08g16010.2	158	-20.84	158	-20.84	1704150	21	1044	851
miR399i	LOC_Os08g16280.1	151	-20.47	151	-20.47	1704179	21	2736	2028
miR399i	LOC_Os08g16680.1	327	-49.04	164	-27.61	1704223	21	3558	3074 1266
miR399i	LOC_Os08g17020.1	146	-20.26	146	-20.26	1704260	21	825	229

miR399i	LOC_Os08g19540.1	159	-22.7	159	-22.7	1704470	21	3897	1862
miR399i	LOC_Os08g19800.1	166	-25.11	166	-25.11	1704499	21	1104	483
miR399i	LOC_Os08g19980.1	167	-22.88	167	-22.88	1704517	21	3393	2053
miR399i	LOC_Os08g21110.1	142	-23.41	142	-23.41	1704615	21	6117	5728
miR399i	LOC_Os08g21400.1	167	-26.23	167	-26.23	1704641	21	192	65
miR399i	LOC_Os08g21420.1	167	-26.23	167	-26.23	1704643	21	1593	65
miR399i	LOC_Os08g21800.1	142	-23.41	142	-23.41	1704677	21	3618	3229
miR399i	LOC_Os08g21970.1	157	-25.26	157	-25.26	1704694	21	2334	1964
miR399i	LOC_Os08g22006.1	149	-20.48	149	-20.48	1704699	21	2094	1015
miR399i	LOC_Os08g22028.1	157	-25.26	157	-25.26	1704708	21	2334	1964
miR399i	LOC_Os08g22020.1	149	-20.48	149	-20.48	1704712	21	1842	745
miR399i	LOC_Os08g22080.1	149	-20.48	149	-20.48	1704715	21	2196	1015
miR399i	LOC_Os08g22480.1	140	-20.89	140	-20.89	1704745	21	2334	1451
miR399i	LOC_Os08g22630.1	153	-23.38	153	-23.38	1704758	21	2055	882
miR399i	LOC_Os08g22864.1	151	-25.34	151	-25.34	1704778	21	3559	2372
miR399i	LOC_Os08g22864.2	151	-25.34	151	-25.34	1704779	21	3664	2362
miR399i	LOC_Os08g23240.1	153	-24.38	153	-24.38	1704818	21	1719	207
miR399i	LOC_Os08g24270.1	153	-20.56	153	-20.56	1704927	21	5925	4192
miR399i	LOC_Os08g24310.1	155	-21.35	155	-21.35	1704931	21	3277	3213
miR399i	LOC_Os08g24650.1	153	-20.56	153	-20.56	1704966	21	5895	4111
miR399i	LOC_Os08g24680.1	153	-24.9	153	-24.9	1704969	21	2880	492
miR399i	LOC_Os08g25640.1	172	-28.87	172	-28.87	1705066	21	3330	207
miR399i	LOC_Os08g26400.1	149	-20.48	149	-20.48	1705143	21	1764	37
miR399i	LOC_Os08g27000.1	149	-20.5	149	-20.5	1705208	21	4197	2102
miR399i	LOC_Os08g27280.1	153	-21.08	153	-21.08	1705237	21	5892	4111
miR399i	LOC_Os08g27560.1	148	-24.94	148	-24.94	1705265	21	465	81
miR399i	LOC_Os08g28360.1	145	-26.87	145	-26.87	1705352	21	1944	1373
miR399i	LOC_Os08g28600.1	144	-20.64	144	-20.64	1705375	21	2901	345
miR399i	LOC_Os08g29400.1	145	-20.9	145	-20.9	1705458	21	3608	2708
miR399i	LOC_Os08g29400.2	145	-20.9	145	-20.9	1705459	21	3139	2239
miR399i	LOC_Os08g29480.1	153	-24.9	153	-24.9	1705466	21	2415	615
miR399i	LOC_Os08g29930.1	153	-20.56	153	-20.56	1705510	21	5988	4192

miR399i	LOC_Os08g30550.1	158	-20.57	158	-20.57	1705576	21	2712	2563
miR399i	LOC_Os08g31219.1	154	-23.53	154	-23.53	1705644	21	944	808
miR399i	LOC_Os08g31228.1	154	-23.53	154	-23.53	1705645	21	1546	1076
miR399i	LOC_Os08g31370.1	164	-26.13	164	-26.13	1705658	21	2670	1043
miR399i	LOC_Os08g31980.1	169	-26.08	169	-26.08	1705733	21	3289	875
miR399i	LOC_Os08g32300.1	152	-26.16	152	-26.16	1705767	21	2280	2074
miR399i	LOC_Os08g34690.1	162	-26.37	162	-26.37	1706039	21	1695	1363
miR399i	LOC_Os08g35020.1	157	-26.03	157	-26.03	1706078	21	5099	4840
miR399i	LOC_Os08g35080.1	156	-23.39	156	-23.39	1706085	21	285	30
miR399i	LOC_Os08g36490.1	170	-24.65	170	-24.65	1706245	21	2848	2473
miR399i	LOC_Os08g36510.1	158	-21.69	158	-21.69	1706247	21	915	84
miR399i	LOC_Os08g36774.1	161	-20.75	161	-20.75	1706274	21	1953	725
miR399i	LOC_Os08g37750.1	173	-32.29	173	-32.29	1706390	21	1614	1440
miR399i	LOC_Os08g38070.1	154	-20.84	154	-20.84	1706433	21	558	325
miR399i	LOC_Os08g38640.1	153	-20.16	153	-20.16	1706503	21	4116	2861
miR399i	LOC_Os08g38970.1	148	-20.18	148	-20.18	1706548	21	3554	2715
miR399i	LOC_Os08g39950.1	147	-27.82	147	-27.82	1706669	21	2739	1461
miR399i	LOC_Os08g40090.1	168	-23.61	168	-23.61	1706684	21	2804	447
miR399i	LOC_Os08g40530.1	153	-20.33	153	-20.33	1706730	21	2394	1314
miR399i	LOC_Os08g40530.2	153	-20.33	153	-20.33	1706731	21	2804	1724
miR399i	LOC_Os08g40640.1	327	-49.04	164	-27.61	1706751	21	5181	3572 1551
miR399i	LOC_Os08g40650.1	150	-22.81	150	-22.81	1706752	21	2370	568
miR399i	LOC_Os08g41070.1	158	-20.47	158	-20.47	1706808	21	4194	400
miR399i	LOC_Os08g41550.1	164	-27.38	164	-27.38	1706868	21	4852	2091
miR399i	LOC_Os08g41630.1	153	-21.71	153	-21.71	1706876	21	3265	990
miR399i	LOC_Os08g41660.1	151	-22.11	151	-22.11	1706878	21	582	151
miR399i	LOC_Os08g43290.1	155	-20.03	155	-20.03	1707076	21	601	560
miR399i	LOC_Os08g43300.1	157	-24.63	157	-24.63	1707077	21	2181	556
miR399i	LOC_Os08g43400.1	157	-22.09	157	-22.09	1707089	21	3403	1038
miR399i	LOC_Os08g43400.2	157	-22.09	157	-22.09	1707090	21	3381	1038
miR399i	LOC_Os08g43400.3	157	-22.09	157	-22.09	1707091	21	3561	1035
miR399i	LOC_Os08g43400.4	157	-22.09	157	-22.09	1707092	21	3368	1038

miR399i	LOC_Os08g43910.1	167	-28.05	167	-28.05	1707146	21	609	69
miR399i	LOC_Os08g44150.1	171	-25.59	171	-25.59	1707175	21	1611	1226
miR399i	LOC_Os08g44740.1	167	-24.73	167	-24.73	1707252	21	444	305
miR399i	LOC_Os08g44780.1	150	-22.64	150	-22.64	1707259	21	1700	1134
miR399i	LOC_Os08g45000.1	354	-62.08	183	-34.98	1707295	21	1827	292 386
miR399i	LOC_Os08g45260.1	155	-24.66	155	-24.66	1707331	21	1467	941
miR399i	LOC_Os09g01030.1	151	-20.41	151	-20.41	1707340	21	624	313
miR399i	LOC_Os09g01310.1	153	-20.89	153	-20.89	1707370	21	1056	85
miR399i	LOC_Os09g01340.1	154	-22.63	154	-22.63	1707373	21	5541	2059
miR399i	LOC_Os09g01370.1	161	-22.93	161	-22.93	1707376	21	2520	1817
miR399i	LOC_Os09g01560.1	153	-20.56	153	-20.56	1707396	21	5835	4051
miR399i	LOC_Os09g01570.1	153	-20.95	153	-20.95	1707397	21	3246	40
miR399i	LOC_Os09g01850.1	153	-20.56	153	-20.56	1707432	21	2937	1153
miR399i	LOC_Os09g01880.1	154	-23.84	154	-23.84	1707435	21	1023	107
miR399i	LOC_Os09g02040.1	163	-26.69	163	-26.69	1707450	21	1599	781
miR399i	LOC_Os09g02150.1	150	-22.65	150	-22.65	1707461	21	651	61
miR399i	LOC_Os09g02820.1	155	-25.8	155	-25.8	1707533	21	1116	44
miR399i	LOC_Os09g03210.1	155	-23.82	155	-23.82	1707571	21	1280	195
miR399i	LOC_Os09g03500.1	157	-24.92	157	-24.92	1707599	21	1596	205
miR399i	LOC_Os09g03620.1	161	-24.68	161	-24.68	1707614	21	2412	1802
miR399i	LOC_Os09g03630.1	162	-23.71	162	-23.71	1707615	21	1785	1346
miR399i	LOC_Os09g03860.1	158	-21.7	158	-21.7	1707634	21	3492	1988
miR399i	LOC_Os09g03900.1	150	-21.09	150	-21.09	1707638	21	2418	51
miR399i	LOC_Os09g04220.1	162	-22.19	162	-22.19	1707671	21	1365	398
miR399i	LOC_Os09g04680.1	165	-25.47	165	-25.47	1707721	21	1155	66
miR399i	LOC_Os09g04830.1	146	-24.06	146	-24.06	1707738	21	3432	3067
miR399i	LOC_Os09g06150.1	154	-20.43	154	-20.43	1707773	21	6033	5808
miR399i	LOC_Os09g06450.1	153	-21.17	153	-21.17	1707803	21	2130	813
miR399i	LOC_Os09g07070.1	140	-20.83	140	-20.83	1707865	21	4188	1595
miR399i	LOC_Os09g07120.1	153	-21.07	153	-21.07	1707870	21	3754	2636
miR399i	LOC_Os09g07120.2	153	-21.07	153	-21.07	1707871	21	3650	2532
miR399i	LOC_Os09g07230.1	140	-23.66	140	-23.66	1707882	21	3693	642

miR399i	LOC_Os09g07300.1	166	-23.82	166	-23.82	1707890	21	13113	7844
miR399i	LOC_Os09g07590.1	151	-20.3	151	-20.3	1707927	21	834	288
miR399i	LOC_Os09g07610.1	148	-20.83	148	-20.83	1707929	21	3486	3173
miR399i	LOC_Os09g07640.1	157	-25.04	157	-25.04	1707933	21	3891	2481
miR399i	LOC_Os09g07690.1	157	-24.73	157	-24.73	1707939	21	2223	712
miR399i	LOC_Os09g07960.1	161	-21.95	161	-21.95	1707970	21	4074	2237
miR399i	LOC_Os09g08030.1	161	-23.73	161	-23.73	1707977	21	621	40
miR399i	LOC_Os09g08340.1	147	-20.78	147	-20.78	1708003	21	4083	3621
miR399i	LOC_Os09g08440.1	163	-21.35	163	-21.35	1708019	21	1128	43
miR399i	LOC_Os09g09000.1	310	-49.32	155	-24.66	1708076	21	8022	6584 7550
miR399i	LOC_Os09g09020.1	155	-24.66	155	-24.66	1708078	21	1383	911
miR399i	LOC_Os09g09080.1	155	-24.66	155	-24.66	1708084	21	1467	941
miR399i	LOC_Os09g09220.1	144	-21.54	144	-21.54	1708098	21	2529	610
miR399i	LOC_Os09g09470.1	158	-20.94	158	-20.94	1708124	21	3002	1429
miR399i	LOC_Os09g09470.2	158	-20.94	158	-20.94	1708125	21	2874	1316
miR399i	LOC_Os09g10190.1	162	-22.55	162	-22.55	1708195	21	348	119
miR399i	LOC_Os09g10390.1	140	-23.66	140	-23.66	1708218	21	2562	2334
miR399i	LOC_Os09g10540.1	140	-20.45	140	-20.45	1708232	21	4686	3981
[truncated: 642,776 more chars]
